# Combined transcriptome and proteome analyses reveal differences in the longissimus dorsi muscle between Kazakh cattle and Xinjiang brown cattle

**DOI:** 10.5713/ab.20.0751

**Published:** 2021-02-15

**Authors:** XiangMin Yan, Jia Wang, Hongbo Li, Liang Gao, Juan Geng, Zhen Ma, Jianming Liu, Jinshan Zhang, Penggui Xie, Lei Chen

**Affiliations:** 1 Institute of Animal Husbandry, Xinjiang Academy of Animal Husbandry, Urumqi 830057, China; 2 College of Geographic Science, Shanxi Normal University, Linfen 041000, China; 3 Yili Vocational and Technical College, Yili, 835000, China; 4 Xinjiang Animal Husbandry General Station, Urumqi 830057, China; 5 Yili Animal Husbandry General Station, Yili 835000, China; 6 College of Animal Science and Technology, Shihezi University, Shihezi 832000, China

**Keywords:** Kazakh Cattle, Longissimus Muscle, Proteomic, Transcriptome, Xinjiang Brown Cattle

## Abstract

**Objective:**

With the rapid development of proteomics sequencing and RNA sequencing technology, multi-omics analysis has become a current research hotspot. Our previous study indicated that Xinjiang brown cattle have better meat quality than Kazakh cattle. In this study, Xinjiang brown cattle and Kazakh cattle were used as the research objects.

**Methods:**

Proteome sequencing and RNA sequencing technology were used to analyze the proteome and transcriptome of the longissimus dorsi muscle of the two breeds of adult steers (n = 3).

**Results:**

In this project, 22,677 transcripts and 1,874 proteins were identified through quantitative analysis of the transcriptome and proteome. By comparing the identified transcriptome and proteome, we found that 1,737 genes were identified at both the transcriptome and proteome levels. The results of the study revealed 12 differentially expressed genes and proteins: troponin I1, crystallin alpha B, cysteine, and glycine rich protein 3, phosphotriesterase-related, myosin-binding protein H, glutathione s-transferase mu 3, myosin light chain 3, nidogen 2, dihydropyrimidinase like 2, glutamate-oxaloacetic transaminase 1, receptor accessory protein 5, and aspartoacylase. We performed functional enrichment of these differentially expressed genes and proteins. The Kyoto encyclopedia of genes and genomes results showed that these differentially expressed genes and proteins are enriched in the fatty acid degradation and histidine metabolism signaling pathways. We performed parallel reaction monitoring (PRM) verification of the differentially expressed proteins, and the PRM results were consistent with the sequencing results.

**Conclusion:**

Our study provided and identified the differentially expressed genes and proteins. In addition, identifying functional genes and proteins with important breeding value will provide genetic resources and technical support for the breeding and industrialization of new genetically modified beef cattle breeds.

## INTRODUCTION

The cattle industry is an important part of China’s modern agricultural production system. At present, the total number of cattle in China has reached more than 100 million heads, ranking third in the world. With the development of China’s agricultural production, the cattle industry has been pursuing data-based changes in pursuit of quality. China has abundant resources in terms of local breeds, such as Qinchuan cattle (distributed in Shaanxi, and having strong bones and full muscles) [1], Kazakh cattle(distributed in Xinjiang, and exhibiting strong stress resistance and resistance to rough feeding) [2,3], Yanbian yellow cattle (distributed in Jilin, and having strong adaptability and tender meat) [4], and Xinjiang brown cattle (distributed in Xinjiang, and having large body and strong adaptability) [5,6]. These breeds of cattle have very unique growth, meat quality, disease resistance, milk quality and other excellent traits.

As one of the local yellow cattle breeds in China, Kazakh cattle are characterized by stable genetic quality, excellent production performance, good grazing performance, rough feeding resistance, strong adaptability and stress resistance and are deeply loved by farmers and herders. Kazakh cattle are the leading species of the Xinjiang beef industry [2,3,6]. Xinjiang Brown Cattle is an excellent new breed of grass-fed livestock that has independent intellectual property rights and is bred in China. Xinjiang brown cattle breeding and improvement, meat type breeding and other traditional cross-breeding programs have done much work and played an important role in the cattle industry [7–9]. However, the taste and flavor of Xinjiang brown beef severely restrict the development of the Xinjiang brown cattle industry [10]. It is of great significance to screen and explain the functional genes of candidate beef quality traits related to the regulation of intracellular fat deposition and fatty acid metabolism and elucidate the underlying molecular mechanisms.

With the rapid development of proteomics sequencing and RNA sequencing technology, multiomics analysis has become a current research hotspot. Kolder used RNA-Seq to perform transcriptome analyses and performed deep proteomic studies of 19 different tissues from common carp [11]. Bathke performed analyses of the transcriptomic and proteomic variation of *Rhodobacter sphaeroides* during the growth process [12]. Schenk used RNA-seq and the iTRAQ technique at the transcriptome and proteome levels to reveal specific molecular signatures of the circalunar clock phase in the brain [13]. Chen revealed the physiological dynamics of the flavonoid pathway by combined transcriptome and proteome analysis in rice of different colors [14]. Ceciliani revealed metabolic status during the transition period by proteomics and metabolomics in dairy cows [15]. Hou used RNA-seq and the iTRAQ technique to reveal potential regulatory proteins of dominant follicle and subordinate follicle in cattle [16]. Pawłowski showed that lipopolysaccharide restricted nutrient in early lactation holstein cows by transcriptome and proteome [17].

This study aims to address serious problems, such as the lack of functional genes in research on beef cattle in China. Our previous study indicated that Xinjiang brown cattle have better meat quality than Kazakh cattle. In this study, proteomics sequencing and RNA-seq were used to study the backs of the two breeds of cattle. The proteome and transcriptome of the longissimus dorsi muscle were analyzed to screen candidate genes and proteins related to beef quality traits.

## MATERIALS AND METHODS

### Ethics statement

The experiment was executed in strict accordance with the guidelines for the care and use of experimental animals at Jilin University. All of the experiments were approved by the Institutional Animal Care and Use Committee of Jilin University (license number: 201809041).

### Animal and tissue preparation

Kazakh cattle and Xinjiang brown cattle were provided by the Xinjiang Yili Yixin Cattle and Sheep Breeding Cooperative. Three 30-month-old adult Xinjiang brown cattle and three 30-month-old adult Kazakh cattle were selected for slaughter. These adult steers were slaughtered in according to the procedure of the slaughterhouse, and then longissimus dorsi muscle was collected. All of the samples were immediately snap-frozen in liquid nitrogen and stored at −80°C until RNA extraction.

### RNA sample preparation and RNA library construction

Total RNA was extracted from each group (the Kazakh cattle group and the Xinjiang brown cattle group) using TRIzol (Invitrogen, NY, USA). A NanoDrop 2000 spectrophotometer (Thermo, Waltham, MA, USA) was used to evaluate the concentrations and quality of the RNA, and agarose gel electrophoresis was used to evaluate the integrity of the RNA. Use epicentre Ribo-ZeroTM kit to remove sample rRNA, then add Fragmentation Buffer to randomly interrupt rRNA-depleted RNA. Use rRNA-depleted RNA as a template to synthesize the first cDNA strand with six-base random hexamers, and then add buffer, dATP, dUTP, dCTP, dGTP, RNase H, and DNA polymerase I to synthesize the second cDNA strand. Use AMPure XP beads to purify cDNA. The purified double-stranded cDNA is then repaired, added with A and connected to the sequencing adapter, and then used AMPure XP beads for fragment size selection. Finally, the U chain is degraded, and finally a cDNA library is obtained by polymerase chain reaction enrichment. The libraries were sequenced by BioMarker Technologies (Beijing, China) using an Illumina HiSeq X Ten platform.

### Transcriptome analysis

StringTie uses FPKM (fragments per kilobase of transcript per million fragments mapped) as an indicator to measure the level of transcripts or gene expression. edgeR is suitable for differential expression analysis between samples (groups). edgeR is a Bioconductor software package that studies the differential expression of repeated count data. In the process of detecting differentially expressed genes, the fold change (FC) represents the ratio of the expression levels between two samples (groups), and the false discovery rate (FDR) is measured by the p-value obtained by calibration. We finally chose fold change ≥1.5 and p value <0.05 as the screening criteria [3,6].

### Protein preparation, liquid chromatography–tandem mass spectrometry, and tandem mass tags labeling quantification

Before the usage, removing each sample out of storage first, the applicable amount of tissue sample was weighed; then put each sample into a container that it was prepared with precooled liquid nitrogen and ground them into powder. Four volumes of lysis buffer were added into each sample. Then, a high-intensity ultrasonic processor (Scientz, Ningbo, China) was used to sonicate each sample on ice through three sound waves. To remove the cell fragments, the mixture was centrifuged at 12,000×g at 4°C for 25 min. The supernatant was then transferred to a new centrifuge tube and the protein concentration was detected using the BCA kit (Beyotime, Shanghai, China). The concentration of protein is 0.25 μg/μL. The peptides were dissolved in liquid phase A for liquid chromatograph (LC) and separated by an EASY-NLC 1000 ultra-high performance liquid phase system. Using tandem mass spectrometry (MS/MS) under a Q Exactive with an applied electrospray at voltage of 2.1 kV, the peptides were subjected to a nanospray ionization (NSI) source. First, each protein mixture was digested with 5 mM dithiothreitol for 30 min at 56°C and treated with 11 mM iodoacetamide at 25°C in the absence of light for 16 min. The concentrations of urea put into the last protein samples were less than 2 M. Then 1:50 trypsin:protein was used for the samples digested overnight at 37°C, and digested at a mass ratio of 1:100 for 4 hours. Then, the peptides were desalt with vacuum freeze-dried and using Strata X c18 column (Phenomenex, Los Angeles, CA, USA). Use TMT kit (Thermo, USA) to reconstitute the peptides in 0.5 MTEAB according to the manufacturer’s protocol. Thaw the labeling reagent and dissolve it in acetonitrile. Incubate for two hours at room temperature, then concentrate, desalinate and dry under vacuum.

### Proteome analysis

The secondary mass spectrum data were collected using MaxQuant (v1.5.2.8). The database was *Bos taurus*_ensembl_1906 (37538 sequences). The anti-library was added to calculate the FDR caused by random matching, and a common pollution library was added to the database to eliminate contaminated proteins in the identification results. The decoy checkbox in Mascot was selected to search the decoy database automatically and generate a random sequence database. The random sequence database and the real database were tested, and the raw spectra were obtained. Peptides with a significance score of 20 in the 99% confidence interval and peptides greater than “identity” in Mascot probability analysis were regarded as identified, which reduces the probability of false peptides. Every reliable protein identification was associated with at least one unique peptide. Tandem mass tags (TMT)-labeled proteomics analysis was performed twice, and the fold change of differentially expressed proteins (DEPs) was >1.5 (p<0.05) or <0.67 (p<0.05).

### Functional enrichment analysis

First, the bovine protein was re-annotated with the aim of analyzing the potential function of the protein. In brief, bovine proteins were mapped to many public databases, covering the NCBI nonredundant, Swiss-Prot/UniProt, gene ontology (GO), and Kyoto encyclopedia of genes and genomes (KEGG) databases. With all proteins as the background, we used the numbers of DEPs to calculate the p-value and Q-value, respectively, representing the significance of the enriched GO terms/KEGG pathways and the FDR. The p-values were calculated by Fisher’s exact test, and the Q-values were calculated by an R package called “q-value”. The threshold of significance was defined as an FDR of 0.05.

### Proteomic validation by parallel reaction monitoring

To verify the results of the TMT analysis, we used PRM to further analyze the results. The liquid gradient was set to increase from 6% to 25% in 40 minutes. After 40 minutes, the gradient increased from 25% to 35% over 12 minutes and from 35% to 80% over 4 minutes before holding at 80% for at least 4 minutes, with a constant flow of 500 nL/min. Using MS/MS at a 2.0 kV electrospray voltage, the peptides were placed in the NSI source. To detect the complete peptide, the full scan range of the primary MS in the Orbitrap was set to 350 to 1,000 m/z with a resolution of 70,000. The resolution of the secondary MS is 35,000. The peptide were then selected for MS/MS using NCE setting as 27. The AGC of the main MS was set to 3e6, and the maximum injection time was set to 50 ms. The AGC of the secondary MS was set to 1e5, and the maximum injection time was set to 200 MS. The isolation window was set to 1.6 m/z.

### Statistical analysis

All transcription and proteomic samples were planned for two biological replications. The results were expressed as mean±standard error of the mean. The student’s t test was used to analyze the differences between the two groups. When p<0.05, difference was considered statistically significant.

## RESULTS

### Flowchart of sequencing

**Figure 1:**
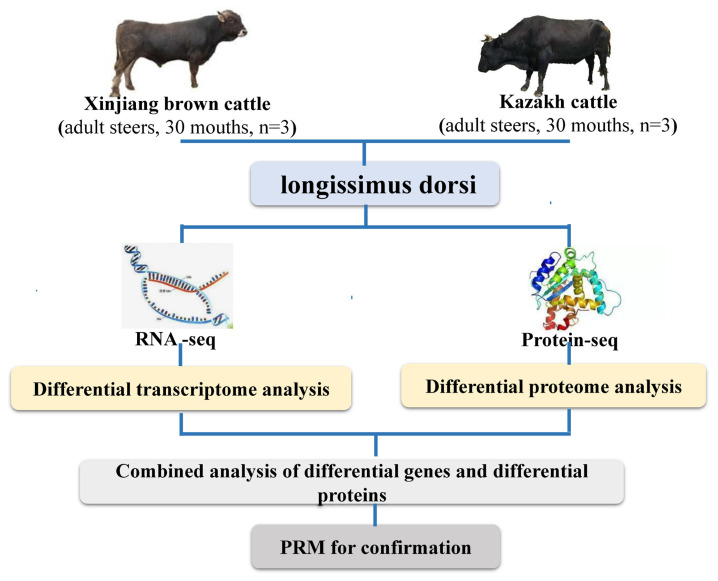
Flowchart of mRNA and protein sequencing. Three adult Xinjiang brown cattle and three adult Kazakh cattle were selected for slaughter to perform RNA-seq and protein-seq, then Screening of differentially expressed proteins and transcripts, finally combined analysis of differential genes and differential proteins.

To promote understanding of the entire experimental process, a flowchart was constructed. Three adult Xinjiang brown cattle and three adult Kazakh cattle were selected for slaughter. These cattle were slaughtered according to the procedure of the slaughterhouse, and then the longissimus dorsi muscle was collected. Later, we extracted the RNA from the sample, and the RNA was used to construct a cDNA library. The libraries were sequenced by BioMarker Technologies (Beijing, China) using an Illumina HiSeq X Ten platform. We performed differential expression analysis of RNA obtained from RNA-seq.Later, the protein was extracted from the sample, digested with trypsin and labeled with TMT. Then, high performance liquid chromatography classification, LC/MS, database searching and MS quality control analysis were performed, and differential expression analysis of the proteins obtained from protein sequencing was performed. Finally, we performed joint analysis of differentially expressed RNA and DEPs (Figure 1).

### Analysis of the differences between the proteome and transcriptome

**Figure 2:**
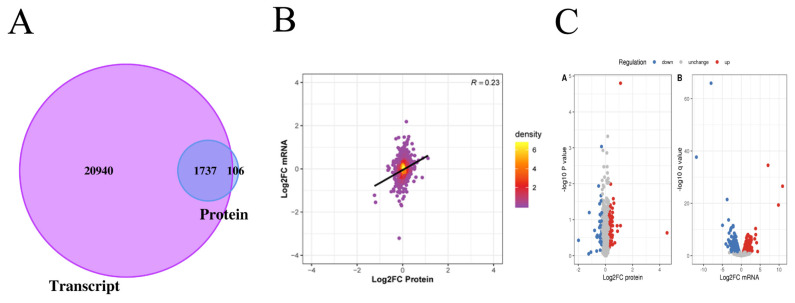
Analysis of the difference between proteome and transcriptome. (A) The Venn diagram shows the identification of transcriptome and proteome. The purple part represents the number of transcripts, and the blue part represents the number of proteins. (B) The PCA plot shows the correlation between mRNA and protein. The abscissa represents the log2FC of protein, and the ordinate represents the log2FC of mRNA. (C) Volcano plots showing differentially expression mRNA and proteins between Xinjiang brown cattle and Kazakhstan cattle. We obtained 929 transcripts and 119 proteins with significant differential expression. FC, fold change.

In this project, 22,677 transcripts and 1,874 proteins were identified through quantitative analysis of the transcriptome and proteome. By comparing the identified transcriptome and proteome, we found that 1,737 genes were identified at both the transcriptome and proteome levels (Figure 2A). The levels of transcriptome and proteome are significantly related (Figure 2B). When Log2 FC>0.585 and p value<0.05, there was a significantly upregulated transcript; when Log2 FC <−0.585 and p value<0.05, there was a significantly downregulated transcript. When the ratio >1.2, there was a significantly upregulated protein; when the ratio <1/1.2, there was a significantly downregulated protein. Through the above screening conditions, we obtained 929 transcripts with significant differential expression, of which 471 were upregulated and 458 were downregulated. There were 119 proteins that were significantly differentially expressed, of which 75 were upregulated and 44 were downregulated (Figure 2C).

### Screening of differentially expressed proteins and transcripts

**Figure 3:**
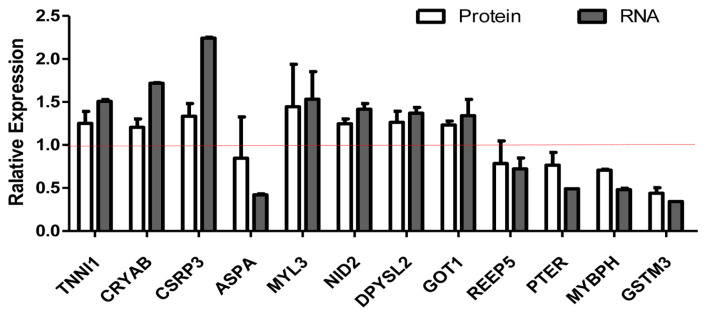
Screening of differentially expressed proteins and transcripts. RNA-seq and protein-seq were used to screen differentially expressed proteins and transcripts. The white column represents the expression of protein, and the gray column represents the expression of mRNA. The RNA and protein expression levels of *TNNI1*, *CRYAB*, *CSRP3*, *NID2*, *DPYSL2*, and *GOT1* were all upregulated, but those of *PTER*, *MYBPH*, and *GSTM3* were all downregulated. The RNA expression of *MYL3* was upregulated, but their protein expression was unchanged. The RNA expression of *ASPA* and *REEP5* were downregulated, but its protein expression. *TNNI1*, troponin I1; *CRYAB*, crystallin alpha B; *CSRP3*, cysteine and glycine rich protein 3; *NID2*, nidogen 2; *DPYSL2*, dihydropyrimidinase like 2; *GOT1*, glutamate-oxaloacetic transaminase 1; *PTER*, phosphotriesterase-related; *MYBPH*, myosin-binding protein H; *GSTM3*, glutathione s-transferase mu 3; *MYL3*, myosin light chain 3; *ASPA*, aspartoacylase; *REEP5*, receptor accessory protein 5.

We selected differentially expressed RNA and protein from 929 transcripts and 119 proteins (Supplementary Table S1 and S2). We compared the relationship between RNA and protein between Xinjiang brown cattle and Kazakh cattle. The expression of RNA and protein was mostly the same, but there were also differences. For example, the RNA and protein expression levels of troponin I1 (*TNNI1*), crystallin alpha B (*CRYAB*), cysteine and glycine rich protein 3 (*CSRP3*), nidogen 2 (*NID2*), dihydropyrimidinase like 2 (*DPYSL2*), and glutamate-oxaloacetic transaminase 1 (*GOT1*) were all upregulated, but those of phosphotriesterase-related (*PTER*), myosin-binding protein H (*MYBPH*), and glutathione s-transferase mu 3 (*GSTM3*) were all downregulated. The RNA expression of myosin light chain 3 (*MYL3*) was upregulated, but their protein expression was unchanged. The RNA expression of aspartoacylase (*ASPA*) and receptor accessory protein 5 (*REEP5*) were downregulated, but its protein expression were unchanged (Figure 3).

### Gene ontology analysis of differentially expressed proteins and transcripts

**Figure 4:**
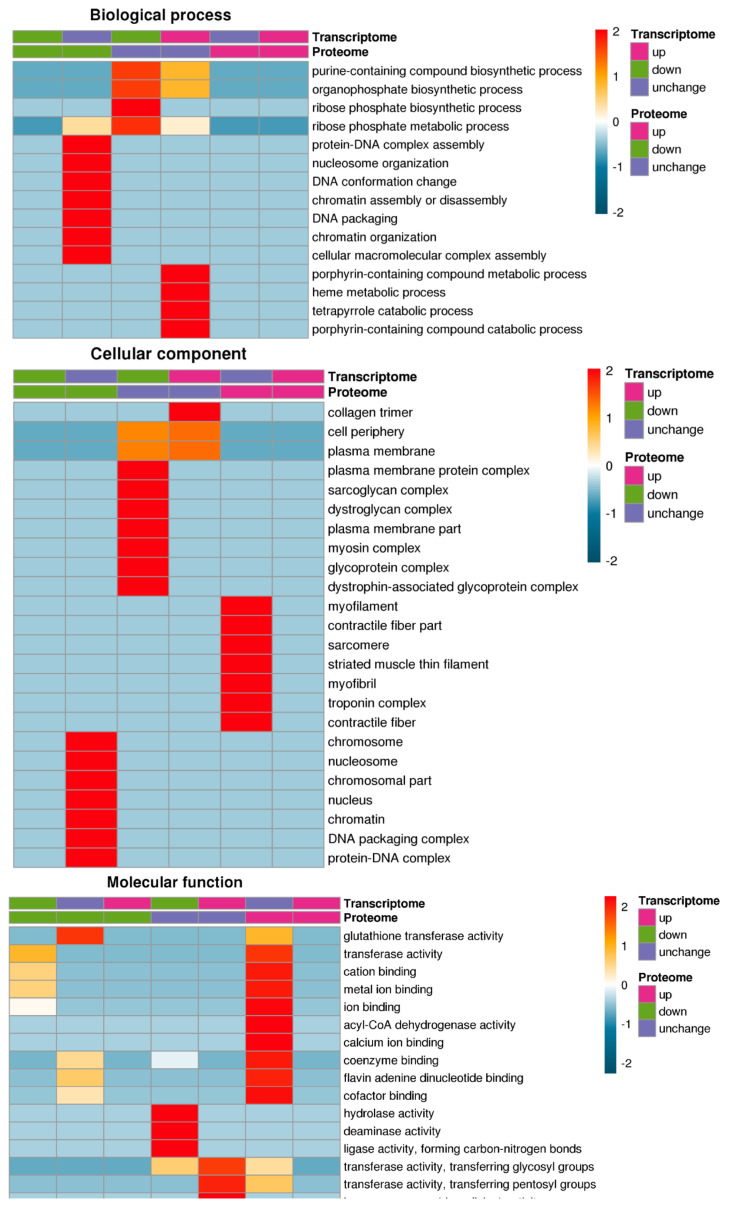
GO enrichment analysis of GO analysis of differentially expressed proteins and transcripts. GO annotations are divided into 3 categories: biological processes, cell composition, and molecular functions. The differentially expressed proteins and transcript were enriched in the collagen trimer, transferase activity transferring pentosyl groups, haem oxygenase activity and flavin adenine dinucleotide binding GO terms. GO, gene ontology.

The GO annotations are divided into 3 categories: biological processes, cell composition, and molecular functions. Fisher’s exact test was used to test the DEPs on the background of the identified proteins, and a GO enrichment test p-value less than 0.05 was considered significant. The DEPs and transcript were enriched in the collagen trimer, transferase activity transferring pentosyl groups, haem oxygenase activity and flavin adenine dinucleotide binding GO terms (Figure 4).

### KEGG analysis of differentially expressed proteins and transcripts

**Figure 5:**
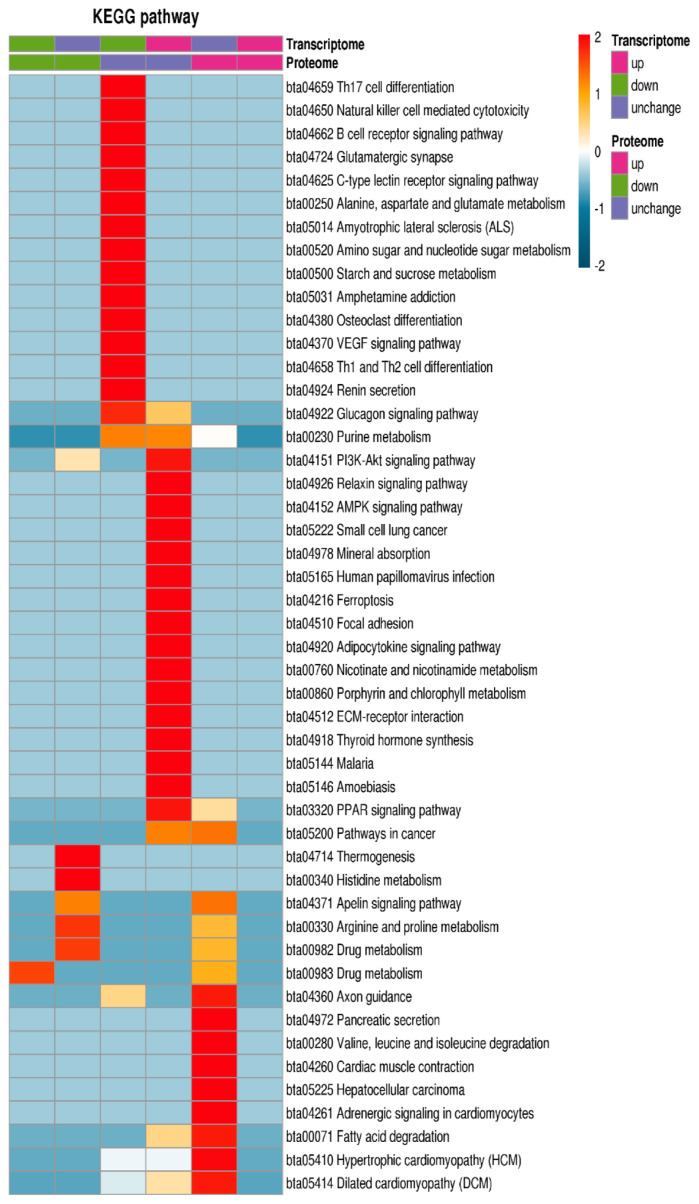
KEGG enrichment analysis of GO analysis of differentially expressed proteins and transcripts. The KEGG database was used for enrichment analysis of pathways. The differentially expressed proteins and transcripts were enriched in adrenergic signaling in PI3K-Akt signaling pathway, AMPK signaling pathway, PPAR signaling pathway, fatty acid degradation and histidine metabolism. KEGG, Kyoto encyclopedia of genes and genomes; GO, gene ontology.

The KEGG database was used for enrichment analysis of pathways. The Fischer exact two-sided test method was used to test whether the DEP was based on the identified protein as the background, and a p-value less than 0.05 in the pathway enrichment test was significant. Finally, these channels were classified according to the KEGG website channel-level classification method. The DEPs and transcripts were enriched in adrenergic signaling in cardiomyocytes, hypertrophic cardiomyopathy, dilated cardiomyopathy, cardiac muscle contraction, PI3K-Akt signaling pathway, AMPK signaling pathway, peroxisome proliferators-activated receptor signaling pathway, fatty acid degradation and histidine metabolism (Figure 5).

### Parallel reaction monitoring for confirmation

**Figure 6:**
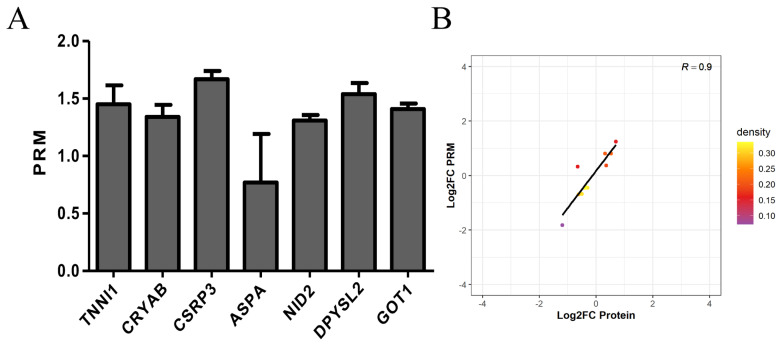
Expression patterns of selected differentially expression proteins using PRM validation. (A) Results of PRM in selected differentially expression proteins. The protein expression of *TNNI1*, *CRYAB*, *CSRP3*, *NID2*, *DPYSL2*, and *GOT1* was upregulated, and the protein expression of *ASPA* was unchanged. (B) Correlation between PRM and TMT results. The PRM results were consistent with the protein sequencing results. PRM, parallel reaction monitoring; *TNNI1*, troponin I1; *CRYAB*, crystallin alpha B; *CSRP3*, cysteine and glycine rich protein 3; *NID2*, nidogen 2; *DPYSL2*, dihydropyrimidinase like 2; *GOT1*, glutamate-oxaloacetic transaminase 1; *ASPA*, aspartoacylase; TMT, tandem mass tags.

In this experiment, we performed PRM quantification of the 20 selected target proteins in 6 samples. Due to the characteristics of protein and the abundance of their expression, we ultimately quantified 7 of them. The protein expression of *TNNI1*, *CRYAB*, *CSRP3*, *ASPA*, *NID2*, *DPYSL2*, and *GOT1* was verified by PRM. The protein expression of *TNNI1*, *CRYAB*, *CSRP3*, *NID2*, *DPYSL2*, and *GOT1* was upregulated, and the protein expression of *ASPA* was unchanged (Figure 6A). The PRM results were consistent with the protein sequencing results (Figure 6B).

## DISCUSSION

The growth and meat quality traits of beef cattle have always been important breeding target traits in beef cattle breeding. With the development of modern molecular breeding techniques such as cell engineering and molecular markers, beef cattle breeding is changing from traditional breeding methods to the direction of combining conventional breeding methods with molecular biology, bioinformatics and computer information technology [18–20]. With the rapid development of second-generation sequencing technology, its application has penetrated the animal husbandry industry [21,22]. Some mature sequencing technologies have been integrated into conventional molecular genetics and breeding research, which has accelerated the process of molecular breeding and significantly improved the breeding level of beef yield and quality [23]. Li [2] studied differentially expressed miRNAs and mRNAs between Kazakh cattle and Xinjiang brown cattle in the longissimus dorsi. We previously studied differentially expressed circular RNAs between Kazakh cattle and Xinjiang brown cattle in the longissimus dorsi [3].

However, at present, most studies are still in the initial stage of screening a large number of candidate genes, and the lack of systematic and in-depth research on gene function has resulted in fewer functional genes with independent intellectual property rights, clear functions, and clear regulatory mechanisms, which hinders the molecular breeding process [24,25]. Fat content and fatty acid content play a vital role in the growth, reproduction and related economic traits of livestock [26,27]. It is also a hotspot in research on the livestock industry in recent years and one of the problems that urgently needs to be solved in current breeding [28]. In-depth study of the functions and regulatory mechanisms of genes involved in lipid metabolism is particularly important. In this study, RNA-seq and TMT markers were used to study the differentially expressed genes and DEPs between Xinjiang brown cattle and Kazakh cattle, and the key candidate genes that determine the meat quality traits of beef cattle muscle growth and fat deposition were systematically screened and investigated.

In our study, the RNA and protein expression levels of *TNNI1*, *CRYAB*, and *CSRP3* were all upregulated, but those of *PTER*, *MYBPH*, and *GSTM3* were all downregulated. *TNNI1* is specifically expressed in slow muscle fibers and plays a key role in muscle development [29,30]. *TNNI1* is expressed in every tissue of the Gaoyou Duck and has a relatively high expression level in muscle tissue, especially in leg muscles [31]. *CRYAB9* was significantly differentially expressed in rectus abdominis muscles compared with four other muscles. *CRYAB* is considered a biomarker of good beef quality traits [32]. The conclusion of this paper is consistent with our result. *CRYAB* protects cardiomyocytes from heat stress by inhibiting F-actin aggregation and reducing caspase-mediated apoptosis [33]. *CSRP3* plays an important role in signal transduction, transcription regulation and the cytoskeleton and can also regulate glucose homeostasis in skeletal muscle. *CSRP3* knockout mice fed a high-fat diet had reduced insulin resistance and glucose tolerance, increased skeletal muscle inflammation, and impaired insulin signaling [34]. We speculated that *TNNI1*, *CRYAB*, and *CSRP3* play important roles in the taste and flavor of Xinjiang brown beef.

The RNA and protein expression levels of *NID2*, *DPYSL2*, and *GOT1* were all upregulated. *NID2* expression is strongly and transiently induced in myogenic differentiation. After treating C2C12 cells with *NID2*-specific siRNA preparation, the expression of the cell cycle inhibitor p21 decreased [35]. *DPYSLs* are a family of proteins that regulate the maturation of the nervous system. *DPYSL2* may play a key role in the influence of inflammation on the neurotransmission of vascular smooth muscle cells [36]. Tag-free shotgun proteome analysis was performed to determine the difference in the expression of *GOT1* between the tender meat group and hard meat group [37].

The RNA expression of *MYL3* was upregulated, but their protein expression was unchanged. The RNA expression of *ASPA* and *REEP5* were downregulated, but its protein expression. Sequence alignment showed that the amino acid sequence similarity between the sheep *MYL3* protein and the mouse, human, rat, cow, and pig proteins exceeded 91%. Previous results showed that *MYL3* mRNA is highly expressed in the heart [38]. Loss of *REEP5* leads to defects in cardiac function and vacuolation of the endoplasmic reticulum [39]. A missense mutation (p. G274R) in the *ASPA* gene caused Canavan disease in a Pakistani family [40]. In our study, the RNA and protein expression levels of *PTER*, *MYBPH*, and *GSTM3* were all downregulated. The beef quality of Kazakh cattle can be improved by reducing the expression of these 3 genes. Compared with that of rats fed a weekly diet, the expression of *PTER* in the adipose tissue of rats fed a high fat diet was upregulated [41]. *MYBPH* inhibits the migration of vascular smooth muscle cells and neointimal hyperplasia in a rat carotid artery balloon injury model [42]. *GSTM3* is an enzyme that is widely present outside the liver and is found in the ciliated airway epithelium and smooth muscle of the lung [43].

Our study showed that the DEPs and transcripts were enriched in fatty acid degradation and histidine metabolism. Fatty acid degradation has a strong relationship with muscle formation [44]. In this study, many DEPs and transcripts were found to be enriched in fatty acid degradation pathway, such as acyl-CoA dehydrogenase short chain (*ACADS*), glutaryl-CoA dehydrogenase (*GCDH*), acyl-CoA dehydrogenase medium chain (*ACADM*), aldehyde dehydrogenase 9 family member A1 (*ALDH9A1*), acyl-CoA synthetase long chain family member 1 (*ACSL1*), and carnitine palmitoyltransferase 1B (*CPT1B*). *ACADS*, *ACADM*, and *ACSL1* are closely related to acyl-CoA [45–47]. It is well known that acyl-CoA regulates fat and muscle metabolism through the tricarboxylic acid cycle [48]. Therefore, we speculate that these genes may also regulate fat and muscle metabolism through acyl-CoA.

## CONCLUSION

In this study, the local breeds in Xinjiang: Xinjiang brown cattle and Kazakh cattle were used as the research objects. Proteomics sequencing and RNA sequencing technology were used to analyze the proteome and transcriptome of the longissimus dorsi muscle of the two breeds of cattle. We obtained 12 differentially expressed genes and proteins, and functionally enriched these differentially expressed genes and proteins. Our research will lay a theoretical foundation for further mining functional genes related to meat quality traits, and provide genetic resources and technical support for the breeding and industrialization of new genetically modified beef cattle breeds.
