# Supplementary material for: Combined transcriptome and proteome analyses reveal differences in the longissimus dorsi muscle between Kazakh cattle and Xinjiang brown cattle
Source: Anim Biosci. 2021 Feb 15;34(9):1439–50. doi: 10.5713/ab.20.0751 (PMC8495333; doi:10.5713/ab.20.0751)
Supplement: Supplementary file 1 [file ab-20-0751-suppl.pdf]

Supplementary Table 1. Protein quantitation

Protein.accession	Transcription.ID	Ratio	P.value	log2FC	log10p
ENSBTAP00000000014.4	ENSBTAG00000000014	NA	NA	NA	NA
ENSBTAP00000000027.5	ENSBTAG00000000025	1.069	0.30814	0.096262	0.511252
ENSBTAP00000000063.4	ENSBTAG00000000057	0.989	0.85386	-0.01596	0.068613
ENSBTAP00000000079.3	ENSBTAG00000037526	NA	NA	NA	NA
ENSBTAP00000000085.6	ENSBTAG00000000077	1.063	0.4781	0.088142	0.320481
ENSBTAP00000000100.1	ENSBTAG00000000091	1.075	0.57142	0.104337	0.243045
ENSBTAP00000000101.5	ENSBTAG00000000092	0.926	0.42518	-0.11092	0.371427
ENSBTAP00000000144.2	ENSBTAG00000000132	1.055	0.2003	0.077243	0.698319
ENSBTAP00000000217.4	ENSBTAG00000000191	1.067	0.59538	0.09356	0.225206
ENSBTAP00000000233.3	ENSBTAG00000000199	1.07	0.054999	0.097611	1.259645
ENSBTAP00000000289.4	ENSBTAG00000000236	0.981	0.73244	-0.02767	0.135228
ENSBTAP00000000315.4	ENSBTAG00000000251	1.036	0.9646	0.051024	0.015653
ENSBTAP00000000341.5	ENSBTAG00000000274	1.067	0.28862	0.09356	0.539674
ENSBTAP00000000359.4	ENSBTAG00000000286	0.892	0.141141	-0.16488	0.850347
ENSBTAP00000000465.6	ENSBTAG00000000359	0.977	0.6479	-0.03357	0.188492
ENSBTAP00000000519.4	ENSBTAG00000000405	NA	NA	NA	NA
ENSBTAP00000000561.5	ENSBTAG00000000440	0.977	0.69332	-0.03357	0.159066
ENSBTAP00000000568.3	ENSBTAG00000023730	0.955	0.37536	-0.06643	0.425552
ENSBTAP00000000581.5	ENSBTAG00000000458	0.994	0.87852	-0.00868	0.056248
ENSBTAP00000000605.5	ENSBTAG00000000478	NA	NA	NA	NA
ENSBTAP00000000630.3	ENSBTAG00000000497	1.036	0.33102	0.051024	0.480146
ENSBTAP00000000643.4	ENSBTAG00000000505	1.074	0.48064	0.102994	0.31818
ENSBTAP00000000662.4	ENSBTAG00000000512	0.963	0.53944	-0.05439	0.268057
ENSBTAP00000000721.2	ENSBTAG00000000550	1.054	0.69834	0.075875	0.155933
ENSBTAP00000000731.3	ENSBTAG00000000555	0.952	0.5575	-0.07097	0.253755
ENSBTAP00000000814.5	ENSBTAG00000000622	NA	NA	NA	NA
ENSBTAP00000000841.4	ENSBTAG00000000639	1.12	0.27042	0.163499	0.567961
ENSBTAP00000000901.5	ENSBTAG00000000678	1.021	0.581	0.029983	0.235824
ENSBTAP00000000927.4	ENSBTAG00000000696	1.086	0.28918	0.119024	0.538832
ENSBTAP00000000935.6	ENSBTAG00000000700	NA	NA	NA	NA
ENSBTAP00000000943.2	ENSBTAG00000000705	1.107	0.42698	0.146655	0.369592
ENSBTAP00000001019.5	ENSBTAG00000000770	1.05	0.199382	0.070389	0.700314
ENSBTAP00000001034.5	ENSBTAG00000000778	1.024	0.85534	0.034216	0.067861
ENSBTAP00000001045.2	ENSBTAG00000000789	0.897	0.40886	-0.15682	0.388425
ENSBTAP00000001076.6	ENSBTAG00000000813	NA	NA	NA	NA
ENSBTAP00000001091.2	ENSBTAG00000000825	NA	NA	NA	NA
ENSBTAP00000001113.4	ENSBTAG00000000837	0.921	0.49468	-0.11873	0.305676
ENSBTAP00000001120.6	ENSBTAG00000011803	0.9	0.53068	-0.152	0.275167
ENSBTAP00000001128.2	ENSBTAG00000006724	1.143	0.29036	0.192825	0.537063
ENSBTAP00000001140.5	ENSBTAG00000000855	1.023	0.76888	0.032806	0.114141
ENSBTAP00000001187.5	ENSBTAG00000000894	0.935	0.38558	-0.09696	0.413886
ENSBTAP00000001208.2	ENSBTAG00000000913	0.791	0.42614	-0.33825	0.370448
ENSBTAP00000001290.4	ENSBTAG00000000974	NA	NA	NA	NA
ENSBTAP00000001303.4	ENSBTAG00000000985	1.028	0.80202	0.03984	0.095815
ENSBTAP00000001330.2	ENSBTAG00000001003	1.135	0.41416	0.182692	0.382832
ENSBTAP00000001373.4	ENSBTAG00000001032	0.954	0.5848	-0.06794	0.232993
ENSBTAP00000001374.5	ENSBTAG00000001036	1.169	0.072101	0.225275	1.142059
ENSBTAP00000001429.6	ENSBTAG00000001078	1.048	0.39264	0.067639	0.406005
ENSBTAP00000001558.6	ENSBTAG00000001173	1.289	0.038923	0.366252	1.409794
ENSBTAP00000001568.6	ENSBTAG00000001182	1.052	0.43384	0.073135	0.36267
ENSBTAP00000001667.6	ENSBTAG00000001265	NA	NA	NA	NA
ENSBTAP00000001698.3	ENSBTAG00000001288	0.805	0.077736	-0.31294	1.109378
ENSBTAP00000001720.2	ENSBTAG00000001303	0.994	0.87264	-0.00868	0.059165
ENSBTAP00000001725.5	ENSBTAG00000001306	0.983	0.73624	-0.02474	0.132981
ENSBTAP00000001791.3	ENSBTAG00000001360	1.003	0.89556	0.004322	0.047905
ENSBTAP00000001839.6	ENSBTAG00000001400	NA	NA	NA	NA

ENSBTAP00000001893.6	ENSBTAG00000001444	1.001	0.93968	0.001442	0.02702
ENSBTAP00000001967.6	ENSBTAG00000001499	1.038	0.15616	0.053806	0.80643
ENSBTAP00000001988.5	ENSBTAG00000001517	0.872	0.44656	-0.1976	0.35012
ENSBTAP00000001993.2	ENSBTAG00000001521	1.097	0.4012	0.133564	0.396639
ENSBTAP00000002033.5	ENSBTAG00000001553	NA	NA	NA	NA
ENSBTAP00000002122.6	ENSBTAG00000001618	0.951	0.28708	-0.07248	0.541997
ENSBTAP00000002168.5	ENSBTAG00000001654	1.049	0.49164	0.069015	0.308353
ENSBTAP00000002256.6	ENSBTAG00000001724	NA	NA	NA	NA
ENSBTAP00000002262.3	ENSBTAG00000001727	0.957	0.3568	-0.06341	0.447575
ENSBTAP00000002316.4	ENSBTAG00000001768	1.279	0.108956	0.355016	0.962749
ENSBTAP00000002326.2	ENSBTAG00000001777	1.02	0.43728	0.028569	0.35924
ENSBTAP00000002364.5	ENSBTAG00000001808	NA	NA	NA	NA
ENSBTAP00000002390.4	ENSBTAG00000001828	1.086	0.55876	0.119024	0.252775
ENSBTAP00000002419.3	ENSBTAG00000001855	1.163	0.129623	0.217851	0.887318
ENSBTAP00000002431.3	ENSBTAG00000001865	0.955	0.42578	-0.06643	0.370815
ENSBTAP00000002506.3	ENSBTAG00000001926	1.049	0.2508	0.069015	0.600672
ENSBTAP00000002508.5	ENSBTAG00000001928	0.958	0.04102	-0.0619	1.387004
ENSBTAP00000002583.6	ENSBTAG00000030301	0.952	0.6863	-0.07097	0.163486
ENSBTAP00000002626.5	ENSBTAG00000002026	0.976	0.49294	-0.03505	0.307206
ENSBTAP00000002648.3	ENSBTAG00000002044	1.051	0.99858	0.071763	0.000617
ENSBTAP00000002650.6	ENSBTAG00000002045	0.995	0.93628	-0.00723	0.028594
ENSBTAP00000002653.5	ENSBTAG00000002048	1.074	0.123919	0.102994	0.906862
ENSBTAP00000002654.3	ENSBTAG00000002049	1.173	0.163258	0.230203	0.787126
ENSBTAP00000002681.4	ENSBTAG00000002075	1.008	0.87014	0.011496	0.060411
ENSBTAP00000002771.6	ENSBTAG00000002137	0.979	0.69808	-0.03062	0.156095
ENSBTAP00000002790.6	ENSBTAG00000002157	1.311	0.01022	0.390668	1.990566
ENSBTAP00000002828.5	ENSBTAG00000002182	1.143	0.105619	0.192825	0.976258
ENSBTAP00000002831.4	ENSBTAG00000002185	NA	NA	NA	NA
ENSBTAP00000002852.5	ENSBTAG00000002203	1.046	0.75034	0.064883	0.124742
ENSBTAP00000002890.3	ENSBTAG00000002238	1.029	0.62416	0.041243	0.204704
ENSBTAP00000002944.5	ENSBTAG00000002278	0.893	0.128622	-0.16327	0.890685
ENSBTAP00000002967.2	ENSBTAG00000002302	1.103	0.114275	0.141433	0.942049
ENSBTAP00000003071.1	ENSBTAG00000002376	0.983	0.8289	-0.02474	0.081498
ENSBTAP00000003073.6	ENSBTAG00000002378	0.973	0.97864	-0.03949	0.009377
ENSBTAP00000003129.5	ENSBTAG00000002412	1.135	0.178858	0.182692	0.747492
ENSBTAP00000003130.4	ENSBTAG00000002413	NA	NA	NA	NA
ENSBTAP00000003145.5	ENSBTAG00000002423	1.045	0.55522	0.063503	0.255535
ENSBTAP00000003165.3	ENSBTAG00000002428	1.075	0.70108	0.104337	0.154232
ENSBTAP00000003192.4	ENSBTAG00000030974	1	0.9761	0	0.010506
ENSBTAP00000003197.5	ENSBTAG00000002463	1.01	0.97654	0.014355	0.01031
ENSBTAP00000003201.3	ENSBTAG00000002468	0.94	0.25132	-0.08927	0.599773
ENSBTAP00000003219.4	ENSBTAG00000002479	0.944	0.4093	-0.08314	0.387958
ENSBTAP00000003234.5	ENSBTAG00000002488	0.862	0.185321	-0.21424	0.732075
ENSBTAP00000003239.2	ENSBTAG00000002493	1.031	0.59002	0.044044	0.229133
ENSBTAP00000003259.3	ENSBTAG00000002507	1.061	0.64562	0.085425	0.190023
ENSBTAP00000003261.4	ENSBTAG00000002510	1.027	0.97774	0.038436	0.009777
ENSBTAP00000003277.3	ENSBTAG00000002526	0.941	0.72952	-0.08773	0.136963
ENSBTAP00000003278.5	ENSBTAG00000002527	0.817	0.124841	-0.29159	0.903643
ENSBTAP00000003330.5	ENSBTAG00000002574	1.064	0.63672	0.089498	0.196052
ENSBTAP00000003343.6	ENSBTAG00000002580	1.026	0.62624	0.037031	0.203259
ENSBTAP00000003377.5	ENSBTAG00000002610	1.034	0.60486	0.048236	0.218345
ENSBTAP00000003429.6	ENSBTAG00000002646	0.978	0.8244	-0.03209	0.083862
ENSBTAP00000003431.2	ENSBTAG00000002648	0.88	0.2273	-0.18442	0.643401
ENSBTAP00000003434.5	ENSBTAG00000032899	0.989	0.8668	-0.01596	0.062081
ENSBTAP00000003437.5	ENSBTAG00000002654	0.96	0.63856	-0.05889	0.194798
ENSBTAP00000003514.6	ENSBTAG00000038842	1.025	0.47176	0.035624	0.326279
ENSBTAP00000003545.4	ENSBTAG00000002734	1.067	0.25284	0.09356	0.597154
ENSBTAP00000003559.4	ENSBTAG00000002745	1.133	0.00954	0.180148	2.020447

ENSBTAP00000003609.5	ENSBTAG00000002784	1.047	0.3943	0.066261	0.404173
ENSBTAP00000003636.4	ENSBTAG00000002808	1.008	0.84972	0.011496	0.070724
ENSBTAP00000003662.3	ENSBTAG00000002829	1.065	0.45218	0.090853	0.344689
ENSBTAP00000003719.3	ENSBTAG00000002866	0.942	0.5518	-0.0862	0.258218
ENSBTAP00000003766.5	ENSBTAG00000002898	0.963	0.45168	-0.05439	0.345169
ENSBTAP00000003800.4	ENSBTAG00000002924	0.95	0.70236	-0.074	0.15344
ENSBTAP00000003869.5	ENSBTAG00000002971	NA	NA	NA	NA
ENSBTAP00000003884.4	ENSBTAG00000002983	1.114	0.32988	0.155749	0.481644
ENSBTAP00000003907.2	ENSBTAG00000003001	1.068	0.32676	0.094912	0.485771
ENSBTAP00000003962.5	ENSBTAG00000005620	1.026	0.52984	0.037031	0.275855
ENSBTAP00000003974.4	ENSBTAG00000003058	1.045	0.53192	0.063503	0.274154
ENSBTAP00000003999.5	ENSBTAG00000003072	1.171	0.199537	0.227741	0.699977
ENSBTAP00000004143.6	ENSBTAG00000003189	0.972	0.6268	-0.04097	0.202871
ENSBTAP00000004145.3	ENSBTAG00000003191	1.094	0.51526	0.129613	0.287974
ENSBTAP00000004190.4	ENSBTAG00000003229	0.986	0.75684	-0.02034	0.120996
ENSBTAP00000004233.3	ENSBTAG00000003268	NA	NA	NA	NA
ENSBTAP00000004244.5	ENSBTAG00000027629	0.989	0.88848	-0.01596	0.051352
ENSBTAP00000004364.4	ENSBTAG00000003362	0.945	0.73774	-0.08161	0.132097
ENSBTAP00000004411.6	ENSBTAG00000003403	1.619	0.43774	0.695103	0.358784
ENSBTAP00000004418.2	ENSBTAG00000003407	NA	NA	NA	NA
ENSBTAP00000004431.5	ENSBTAG00000003415	1.054	0.18786	0.075875	0.726166
ENSBTAP00000004519.5	ENSBTAG00000003476	NA	NA	NA	NA
ENSBTAP00000004524.6	ENSBTAG00000003481	0.886	0.36402	-0.17462	0.438875
ENSBTAP00000004562.2	ENSBTAG00000003505	1.01	0.96512	0.014355	0.015419
ENSBTAP00000004574.4	ENSBTAG00000003519	1.049	0.45052	0.069015	0.346286
ENSBTAP00000004617.5	ENSBTAG00000003550	1.047	0.60742	0.066261	0.216511
ENSBTAP00000004645.5	ENSBTAG00000003570	0.962	0.53058	-0.05589	0.275249
ENSBTAP00000004656.6	ENSBTAG00000003577	1.137	0.173078	0.185232	0.761758
ENSBTAP00000004658.4	ENSBTAG00000003581	1.05	0.64638	0.070389	0.189512
ENSBTAP00000004664.6	ENSBTAG00000003585	NA	NA	NA	NA
ENSBTAP00000004701.3	ENSBTAG00000003609	0.93	0.137765	-0.1047	0.860861
ENSBTAP00000004751.3	ENSBTAG00000003642	1.032	0.50566	0.045443	0.296141
ENSBTAP00000004806.5	ENSBTAG00000003690	0.885	0.25452	-0.17625	0.594278
ENSBTAP00000004813.5	ENSBTAG00000003693	0.992	0.92006	-0.01159	0.036184
ENSBTAP00000004831.6	ENSBTAG00000003708	1.053	0.4507	0.074505	0.346112
ENSBTAP00000004879.4	ENSBTAG00000003746	1.018	0.58992	0.025738	0.229207
ENSBTAP00000004890.3	ENSBTAG00000003757	1.051	0.3602	0.071763	0.443456
ENSBTAP00000004892.4	ENSBTAG00000003758	1.139	0.26438	0.187768	0.577771
ENSBTAP00000004912.6	ENSBTAG00000003773	NA	NA	NA	NA
ENSBTAP00000005064.6	ENSBTAG00000003882	NA	NA	NA	NA
ENSBTAP00000005170.5	ENSBTAG00000003966	0.96	0.100442	-0.05889	0.998085
ENSBTAP00000005174.3	ENSBTAG00000003967	1.151	0.146656	0.202888	0.8337
ENSBTAP00000005236.4	ENSBTAG00000004011	NA	NA	NA	NA
ENSBTAP00000005296.2	ENSBTAG00000004051	1.033	0.49418	0.04684	0.306115
ENSBTAP00000005311.5	ENSBTAG00000004064	NA	NA	NA	NA
ENSBTAP00000005332.6	ENSBTAG00000004081	1.063	0.64552	0.088142	0.19009
ENSBTAP00000005354.5	ENSBTAG00000004098	NA	NA	NA	NA
ENSBTAP00000005375.3	ENSBTAG00000004112	0.885	0.219	-0.17625	0.659556
ENSBTAP00000005496.3	ENSBTAG00000004199	NA	NA	NA	NA
ENSBTAP00000005531.3	ENSBTAG00000004222	NA	NA	NA	NA
ENSBTAP00000005555.5	ENSBTAG00000004242	1.019	0.73004	0.027154	0.136653
ENSBTAP00000005558.3	ENSBTAG00000004243	1.071	0.176502	0.098958	0.75325
ENSBTAP00000005562.3	ENSBTAG00000004248	1.144	0.21734	0.194087	0.66286
ENSBTAP00000005581.3	ENSBTAG00000004258	0.998	0.9315	-0.00289	0.030817
ENSBTAP00000005600.5	ENSBTAG00000004279	1.027	0.48974	0.038436	0.310034
ENSBTAP00000005603.6	ENSBTAG00000004278	1.1	0.44882	0.137504	0.347928
ENSBTAP00000005618.5	ENSBTAG00000021516	1.855	0.20976	0.891419	0.678277
ENSBTAP00000005626.5	ENSBTAG00000021372	NA	NA	NA	NA

ENSBTAP00000005628.5	ENSBTAG00000004295	1.062	0.6643	0.086784	0.177636
ENSBTAP00000005722.2	ENSBTAG00000004367	1.141	0.28842	0.190299	0.539975
ENSBTAP00000005730.4	ENSBTAG00000004371	1.141	0.33718	0.190299	0.472138
ENSBTAP00000005747.4	ENSBTAG00000004379	1.063	0.078841	0.088142	1.103248
ENSBTAP00000005782.4	ENSBTAG00000004409	1.106	0.28886	0.145351	0.539313
ENSBTAP00000005789.3	ENSBTAG00000004416	0.797	0.02142	-0.32735	1.669181
ENSBTAP00000005832.5	ENSBTAG00000004448	0.958	0.82822	-0.0619	0.081854
ENSBTAP00000005874.6	ENSBTAG00000004476	0.867	0.4194	-0.2059	0.377372
ENSBTAP00000005980.6	ENSBTAG00000004552	1.06	0.34004	0.084064	0.46847
ENSBTAP00000005982.5	ENSBTAG00000004553	0.997	0.92908	-0.00433	0.031947
ENSBTAP00000005983.5	ENSBTAG00000004554	NA	NA	NA	NA
ENSBTAP00000006036.4	ENSBTAG00000004598	0.957	0.72278	-0.06341	0.140994
ENSBTAP00000006038.3	ENSBTAG00000004601	1.149	0.142856	0.200379	0.845102
ENSBTAP00000006075.6	ENSBTAG00000004632	1.007	0.9352	0.010064	0.029096
ENSBTAP00000006139.2	ENSBTAG00000004679	0.968	0.2093	-0.04692	0.679231
ENSBTAP00000006190.3	ENSBTAG00000025313	0.988	0.88116	-0.01742	0.054945
ENSBTAP00000006213.5	ENSBTAG00000004732	0.955	0.09264	-0.06643	1.033201
ENSBTAP00000006216.4	ENSBTAG00000004736	1.046	0.44582	0.064883	0.35084
ENSBTAP00000006219.6	ENSBTAG00000004738	1.014	0.86956	0.020058	0.0607
ENSBTAP00000006245.5	ENSBTAG00000003989	1.165	0.113036	0.22033	0.946783
ENSBTAP00000006274.4	ENSBTAG00000004776	1.011	0.86016	0.015783	0.065421
ENSBTAP00000006374.6	ENSBTAG00000004849	0.957	0.113441	-0.06341	0.94523
ENSBTAP00000006383.5	ENSBTAG00000004855	1.093	0.43162	0.128293	0.364898
ENSBTAP00000006476.6	ENSBTAG00000004922	0.82	0.00092	-0.2863	3.036245
ENSBTAP00000006534.2	ENSBTAG00000046332	1.004	0.84008	0.005759	0.075679
ENSBTAP00000006658.4	ENSBTAG00000005048	0.98	0.75604	-0.02915	0.121455
ENSBTAP00000006754.4	ENSBTAG00000005119	1.007	0.85482	0.010064	0.068125
ENSBTAP00000006760.3	ENSBTAG00000005127	0.985	0.82566	-0.0218	0.083199
ENSBTAP00000006806.4	ENSBTAG00000005163	1.135	0.43128	0.182692	0.365241
ENSBTAP00000006821.6	ENSBTAG00000005178	NA	NA	NA	NA
ENSBTAP00000006843.5	ENSBTAG00000005195	NA	NA	NA	NA
ENSBTAP00000006847.5	ENSBTAG00000005191	0.989	0.63192	-0.01596	0.199338
ENSBTAP00000006933.2	ENSBTAG00000023928	NA	NA	NA	NA
ENSBTAP00000006935.5	ENSBTAG00000005272	NA	NA	NA	NA
ENSBTAP00000006947.4	ENSBTAG00000005280	1.098	0.28124	0.134878	0.550923
ENSBTAP00000006992.4	ENSBTAG00000005316	1.014	0.68744	0.020058	0.162765
ENSBTAP00000007039.2	ENSBTAG00000005355	0.963	0.8215	-0.05439	0.085392
ENSBTAP00000007041.4	ENSBTAG00000005353	0.9	0.3096	-0.152	0.509199
ENSBTAP00000007052.4	ENSBTAG00000005364	NA	NA	NA	NA
ENSBTAP00000007093.5	ENSBTAG00000005393	1.079	0.23372	0.109695	0.631304
ENSBTAP00000007111.6	ENSBTAG00000002319	0.915	0.5144	-0.12816	0.288699
ENSBTAP00000007149.3	ENSBTAG00000005431	1.06	0.71212	0.084064	0.147447
ENSBTAP00000007169.6	ENSBTAG00000005448	NA	NA	NA	NA
ENSBTAP00000007189.3	ENSBTAG00000005465	1.047	0.48274	0.066261	0.316287
ENSBTAP00000007216.3	ENSBTAG00000005488	1.109	0.00498	0.149259	2.302762
ENSBTAP00000007309.6	ENSBTAG00000005557	1.079	0.41722	0.109695	0.379635
ENSBTAP00000007359.6	ENSBTAG00000040053	1.074	0.67196	0.102994	0.172657
ENSBTAP00000007398.3	ENSBTAG00000005627	1.028	0.76524	0.03984	0.116202
ENSBTAP00000007403.3	ENSBTAG00000040298	NA	NA	NA	NA
ENSBTAP00000007404.6	ENSBTAG00000005631	1.05	0.51804	0.070389	0.285637
ENSBTAP00000007423.4	ENSBTAG00000039958	0.966	0.3442	-0.0499	0.463189
ENSBTAP00000007478.5	ENSBTAG00000005693	NA	NA	NA	NA
ENSBTAP00000007481.6	ENSBTAG00000005694	1.055	0.24084	0.077243	0.618271
ENSBTAP00000007503.6	ENSBTAG00000005712	NA	NA	NA	NA
ENSBTAP00000007563.3	ENSBTAG00000005754	0.967	0.30668	-0.04841	0.513315
ENSBTAP00000007571.6	ENSBTAG00000005757	1.081	0.39454	0.112367	0.403909
ENSBTAP00000007574.5	ENSBTAG00000005760	0.949	0.52982	-0.07552	0.275872
ENSBTAP00000007589.5	ENSBTAG00000005773	1.067	0.36616	0.09356	0.436329

ENSBTAP00000007642.1	ENSBTAG00000005814	1.033	0.72106	0.04684	0.142029
ENSBTAP00000007684.6	ENSBTAG00000005845	1.001	0.956	0.001442	0.019542
ENSBTAP00000007696.4	ENSBTAG00000005854	0.853	0.014441	-0.22938	1.840391
ENSBTAP00000007705.4	ENSBTAG00000005862	0.86	0.019297	-0.21759	1.714506
ENSBTAP00000007761.2	ENSBTAG00000005907	NA	NA	NA	NA
ENSBTAP00000007840.4	ENSBTAG00000005975	0.925	0.38946	-0.11247	0.409537
ENSBTAP00000007860.5	ENSBTAG00000005989	0.852	0.39082	-0.23107	0.408023
ENSBTAP00000007899.5	ENSBTAG00000006013	NA	NA	NA	NA
ENSBTAP00000007943.5	ENSBTAG00000006045	0.948	0.2434	-0.07704	0.613679
ENSBTAP00000007948.6	ENSBTAG00000006049	0.888	0.052861	-0.17137	1.276865
ENSBTAP00000007950.5	ENSBTAG00000006051	1.104	0.194576	0.14274	0.710911
ENSBTAP00000007970.5	ENSBTAG00000006066	NA	NA	NA	NA
ENSBTAP00000007978.5	ENSBTAG00000006072	0.958	0.09188	-0.0619	1.036779
ENSBTAP00000008019.6	ENSBTAG00000006101	1.05	0.3413	0.070389	0.466864
ENSBTAP00000008061.6	ENSBTAG00000006126	0.965	0.6227	-0.0514	0.205721
ENSBTAP00000008130.3	ENSBTAG00000006187	1.019	0.99514	0.027154	0.002116
ENSBTAP00000008146.5	ENSBTAG00000006202	0.964	0.80768	-0.05289	0.092761
ENSBTAP00000008200.2	ENSBTAG00000006247	0.965	0.78576	-0.0514	0.10471
ENSBTAP00000008293.5	ENSBTAG00000006321	0.878	0.3985	-0.18771	0.399572
ENSBTAP00000008319.3	ENSBTAG00000006342	0.989	0.89404	-0.01596	0.048643
ENSBTAP00000008325.6	ENSBTAG00000006345	0.917	0.5897	-0.12501	0.229369
ENSBTAP00000008327.2	ENSBTAG00000006346	NA	NA	NA	NA
ENSBTAP00000008358.5	ENSBTAG00000006370	1.049	0.5523	0.069015	0.257825
ENSBTAP00000008368.5	ENSBTAG00000006379	NA	NA	NA	NA
ENSBTAP00000008371.6	ENSBTAG00000022158	1.309	0.28894	0.388465	0.539192
ENSBTAP00000008380.6	ENSBTAG00000006387	NA	NA	NA	NA
ENSBTAP00000008382.3	ENSBTAG00000006391	1.117	0.41522	0.159629	0.381722
ENSBTAP00000008410.4	ENSBTAG00000006414	1.062	0.35932	0.086784	0.444519
ENSBTAP00000008434.5	ENSBTAG00000006434	1.051	0.30888	0.071763	0.51021
ENSBTAP00000008473.5	ENSBTAG00000006463	1.142	0.199898	0.191563	0.699192
ENSBTAP00000008517.5	ENSBTAG00000006499	1.025	0.46272	0.035624	0.334682
ENSBTAP00000008518.5	ENSBTAG00000006491	0.96	0.47386	-0.05889	0.32435
ENSBTAP00000008569.6	ENSBTAG00000006523	1.142	0.21076	0.191563	0.676212
ENSBTAP00000008580.5	ENSBTAG00000006533	0.976	0.44322	-0.03505	0.353381
ENSBTAP00000008593.6	ENSBTAG00000006541	0.896	0.141483	-0.15843	0.849296
ENSBTAP00000008601.6	ENSBTAG00000006547	0.978	0.7581	-0.03209	0.120274
ENSBTAP00000008621.2	ENSBTAG00000006564	0.956	0.58894	-0.06492	0.229929
ENSBTAP00000008635.6	ENSBTAG00000006574	1.146	0.49852	0.196607	0.302317
ENSBTAP00000008650.5	ENSBTAG00000006586	1.009	0.68914	0.012926	0.161693
ENSBTAP00000008727.4	ENSBTAG00000006642	1.02	0.83582	0.028569	0.077887
ENSBTAP00000008734.5	ENSBTAG00000006646	NA	NA	NA	NA
ENSBTAP00000008754.3	ENSBTAG00000006665	0.871	0.049477	-0.19926	1.305597
ENSBTAP00000008760.6	ENSBTAG00000006667	0.962	0.43104	-0.05589	0.365482
ENSBTAP00000008809.2	ENSBTAG00000006702	NA	NA	NA	NA
ENSBTAP00000008822.5	ENSBTAG00000006708	1.035	0.91004	0.049631	0.04094
ENSBTAP00000008851.5	ENSBTAG00000006733	0.906	0.03836	-0.14242	1.416121
ENSBTAP00000008894.4	ENSBTAG00000006759	1.08	0.51736	0.111031	0.286207
ENSBTAP00000008930.5	ENSBTAG00000040133	NA	NA	NA	NA
ENSBTAP00000008962.4	ENSBTAG00000006821	NA	NA	NA	NA
ENSBTAP00000008967.6	ENSBTAG00000006823	1.005	0.9517	0.007196	0.0215
ENSBTAP00000008987.5	ENSBTAG00000006838	1.09	0.38312	0.124328	0.416665
ENSBTAP00000008994.3	ENSBTAG00000006852	1.146	0.23146	0.196607	0.635524
ENSBTAP00000009064.6	ENSBTAG00000006898	1.005	0.84848	0.007196	0.071358
ENSBTAP00000009098.5	ENSBTAG00000006927	1.017	0.72704	0.02432	0.138442
ENSBTAP00000009128.4	ENSBTAG00000006950	0.962	0.185538	-0.05589	0.731567
ENSBTAP00000009158.4	ENSBTAG00000006969	1.057	0.6282	0.079975	0.201902
ENSBTAP00000009160.5	ENSBTAG00000006970	1.081	0.46172	0.112367	0.335621
ENSBTAP00000009228.6	ENSBTAG00000006999	0.974	0.35718	-0.03801	0.447113

ENSBTAP00000009261.5	ENSBTAG00000007041	0.924	0.49272	-0.11404	0.3074
ENSBTAP00000009272.6	ENSBTAG00000003948	1.065	0.131405	0.090853	0.881388
ENSBTAP00000009307.4	ENSBTAG00000007080	NA	NA	NA	NA
ENSBTAP00000009327.3	ENSBTAG00000018204	0.863	0.36038	-0.21257	0.443239
ENSBTAP00000009334.5	ENSBTAG00000007097	NA	NA	NA	NA
ENSBTAP00000009346.4	ENSBTAG00000007106	1.198	0.25468	0.260628	0.594005
ENSBTAP00000009440.2	ENSBTAG00000007172	1.104	0.35396	0.14274	0.451046
ENSBTAP00000009461.3	ENSBTAG00000007189	NA	NA	NA	NA
ENSBTAP00000009469.2	ENSBTAG00000007196	1.188	0.25856	0.248535	0.587439
ENSBTAP00000009477.5	ENSBTAG00000007203	0.983	0.70862	-0.02474	0.149587
ENSBTAP00000009531.6	ENSBTAG00000007244	0.986	0.82444	-0.02034	0.083841
ENSBTAP00000009564.6	ENSBTAG00000007273	1.176	0.2118	0.233888	0.674074
ENSBTAP00000009604.3	ENSBTAG00000007300	0.991	0.82158	-0.01304	0.08535
ENSBTAP00000009643.5	ENSBTAG00000007332	1.173	0.055703	0.230203	1.254121
ENSBTAP00000009661.4	ENSBTAG00000011400	0.887	0.175221	-0.17299	0.756414
ENSBTAP00000009662.2	ENSBTAG00000011400	0.925	0.24246	-0.11247	0.61536
ENSBTAP00000009687.4	ENSBTAG00000007367	1.031	0.4688	0.044044	0.329012
ENSBTAP00000009699.3	ENSBTAG00000007375	0.963	0.80404	-0.05439	0.094722
ENSBTAP00000009711.5	ENSBTAG00000007386	NA	NA	NA	NA
ENSBTAP00000009803.2	ENSBTAG00000007454	1.02	0.74174	0.028569	0.129748
ENSBTAP00000009844.4	ENSBTAG00000007484	1.42	0.113016	0.505891	0.94686
ENSBTAP00000009887.5	ENSBTAG00000007513	NA	NA	NA	NA
ENSBTAP00000009897.5	ENSBTAG00000007520	1.031	0.5302	0.044044	0.27556
ENSBTAP00000009921.2	ENSBTAG00000007537	NA	NA	NA	NA
ENSBTAP00000009923.4	ENSBTAG00000007540	1.171	0.2096	0.227741	0.678609
ENSBTAP00000009930.4	ENSBTAG00000007547	0.967	0.6775	-0.04841	0.169091
ENSBTAP00000009998.5	ENSBTAG00000007605	1.064	0.55198	0.089498	0.258077
ENSBTAP00000010108.2	ENSBTAG00000007685	0.983	0.81052	-0.02474	0.091236
ENSBTAP00000010141.5	ENSBTAG00000007712	0.917	0.25328	-0.12501	0.596399
ENSBTAP00000010168.6	ENSBTAG00000007734	1.062	0.183402	0.086784	0.736596
ENSBTAP00000010176.2	ENSBTAG00000007737	NA	NA	NA	NA
ENSBTAP00000010212.5	ENSBTAG00000007766	NA	NA	NA	NA
ENSBTAP00000010231.3	ENSBTAG00000007782	1.063	0.43206	0.088142	0.364456
ENSBTAP00000010276.5	ENSBTAG00000007812	1.043	0.71896	0.060739	0.143295
ENSBTAP00000010299.5	ENSBTAG00000007830	0.9	0.173139	-0.152	0.761605
ENSBTAP00000010309.5	ENSBTAG00000007835	1.172	0.185101	0.228973	0.732591
ENSBTAP00000010351.6	ENSBTAG00000007867	0.979	0.7972	-0.03062	0.098433
ENSBTAP00000010355.5	ENSBTAG00000007871	NA	NA	NA	NA
ENSBTAP00000010366.6	ENSBTAG00000007881	0.957	0.27734	-0.06341	0.556987
ENSBTAP00000010389.3	ENSBTAG00000007900	1.096	0.3833	0.132248	0.416461
ENSBTAP00000010436.4	ENSBTAG00000007939	0.874	0.2036	-0.19429	0.691222
ENSBTAP00000010538.5	ENSBTAG00000008014	1.041	0.40822	0.05797	0.389106
ENSBTAP00000010644.2	ENSBTAG00000008091	1.165	0.27324	0.22033	0.563456
ENSBTAP00000010661.4	ENSBTAG00000008103	0.886	0.2164	-0.17462	0.664743
ENSBTAP00000010674.5	ENSBTAG00000008114	1.06	0.114543	0.084064	0.941031
ENSBTAP00000010692.6	ENSBTAG00000008132	0.914	0.61308	-0.12973	0.212483
ENSBTAP00000010698.5	ENSBTAG00000008137	1	0.95392	0	0.020488
ENSBTAP00000010765.6	ENSBTAG00000008184	0.972	0.77348	-0.04097	0.111551
ENSBTAP00000010780.5	ENSBTAG00000008195	0.909	0.34678	-0.13765	0.459946
ENSBTAP00000010806.5	ENSBTAG00000008218	0.941	0.232	-0.08773	0.634512
ENSBTAP00000010928.4	ENSBTAG00000008303	1.034	0.58252	0.048236	0.234689
ENSBTAP00000010949.4	ENSBTAG00000008314	1.053	0.80174	0.074505	0.095966
ENSBTAP00000010982.4	ENSBTAG00000008340	0.989	0.90418	-0.01596	0.043745
ENSBTAP00000011047.5	ENSBTAG00000008394	1.444	0.49376	0.530071	0.306484
ENSBTAP00000011107.5	ENSBTAG00000008442	1.063	0.25702	0.088142	0.590033
ENSBTAP00000011270.5	ENSBTAG00000008548	1.091	0.098239	0.125651	1.007716
ENSBTAP00000011383.4	ENSBTAG00000008632	NA	NA	NA	NA
ENSBTAP00000011388.2	ENSBTAG00000008635	NA	NA	NA	NA

ENSBTAP00000011403.3	ENSBTAG00000008648	0.985	0.85272	-0.0218	0.069194
ENSBTAP00000011408.5	ENSBTAG00000008652	0.996	0.98254	-0.00578	0.00765
ENSBTAP00000011458.3	ENSBTAG00000008692	1.075	0.48834	0.104337	0.311278
ENSBTAP00000011484.4	ENSBTAG00000008716	0.977	0.68322	-0.03357	0.165439
ENSBTAP00000011485.6	ENSBTAG00000008717	0.992	0.98588	-0.01159	0.006176
ENSBTAP00000011505.1	ENSBTAG00000008731	1.108	0.4333	0.147958	0.363211
ENSBTAP00000011521.4	ENSBTAG00000008743	1.049	0.65358	0.069015	0.184701
ENSBTAP00000011559.6	ENSBTAG00000021039	1.023	0.24084	0.032806	0.618271
ENSBTAP00000011576.2	ENSBTAG00000048094	0.851	0.50984	-0.23277	0.292566
ENSBTAP00000011658.4	ENSBTAG00000008853	NA	NA	NA	NA
ENSBTAP00000011696.4	ENSBTAG00000008884	NA	NA	NA	NA
ENSBTAP00000011729.5	ENSBTAG00000008909	1.074	0.48448	0.102994	0.314724
ENSBTAP00000011801.5	ENSBTAG00000001164	1.057	0.63214	0.079975	0.199187
ENSBTAP00000012108.3	ENSBTAG00000009188	0.991	0.9542	-0.01304	0.020361
ENSBTAP00000012154.4	ENSBTAG00000009223	0.957	0.3447	-0.06341	0.462559
ENSBTAP00000012182.4	ENSBTAG00000009245	1.028	0.4833	0.03984	0.315783
ENSBTAP00000012189.4	ENSBTAG00000009251	1.075	0.48616	0.104337	0.313221
ENSBTAP00000012256.5	ENSBTAG00000009304	1.027	0.48758	0.038436	0.311954
ENSBTAP00000012287.5	ENSBTAG00000009334	1.072	0.57742	0.100305	0.238508
ENSBTAP00000012291.5	ENSBTAG00000009337	1.021	0.76008	0.029983	0.119141
ENSBTAP00000012340.2	ENSBTAG00000009376	NA	NA	NA	NA
ENSBTAP00000012351.6	ENSBTAG00000009387	0.978	0.59458	-0.03209	0.22579
ENSBTAP00000012357.6	ENSBTAG00000009389	NA	NA	NA	NA
ENSBTAP00000012409.6	ENSBTAG00000009430	1.498	0.026101	0.583038	1.583343
ENSBTAP00000012454.5	ENSBTAG00000009462	1.059	0.61418	0.082703	0.211704
ENSBTAP00000012495.3	ENSBTAG00000009495	1.195	0.33786	0.257011	0.471263
ENSBTAP00000012497.3	ENSBTAG00000010125	0.844	0.45754	-0.24469	0.339571
ENSBTAP00000012512.6	ENSBTAG00000009509	1.1	0.38454	0.137504	0.415058
ENSBTAP00000012519.6	ENSBTAG00000009513	1.006	0.89512	0.00863	0.048119
ENSBTAP00000012523.3	ENSBTAG00000009517	1.208	0.115785	0.27262	0.936348
ENSBTAP00000012530.5	ENSBTAG00000009522	NA	NA	NA	NA
ENSBTAP00000012541.3	ENSBTAG00000009533	0.863	0.44288	-0.21257	0.353714
ENSBTAP00000012542.2	ENSBTAG00000009534	1.071	0.58522	0.098958	0.232681
ENSBTAP00000012544.3	ENSBTAG00000009535	0.979	0.58772	-0.03062	0.23083
ENSBTAP00000012551.4	ENSBTAG00000009541	1.135	0.32534	0.182692	0.487663
ENSBTAP00000012592.5	ENSBTAG00000009574	NA	NA	NA	NA
ENSBTAP00000012594.6	ENSBTAG00000009576	0.967	0.56248	-0.04841	0.249893
ENSBTAP00000012599.5	ENSBTAG00000009580	1.035	0.85248	0.049631	0.069316
ENSBTAP00000012655.3	ENSBTAG00000009615	1.045	0.70188	0.063503	0.153737
ENSBTAP00000012735.5	ENSBTAG00000009663	1.063	0.359	0.088142	0.444906
ENSBTAP00000012773.5	ENSBTAG00000009683	0.99	0.73598	-0.0145	0.133134
ENSBTAP00000012777.3	ENSBTAG00000009687	0.988	0.85996	-0.01742	0.065522
ENSBTAP00000012786.5	ENSBTAG00000009696	NA	NA	NA	NA
ENSBTAP00000012797.5	ENSBTAG00000007090	0.983	0.78034	-0.02474	0.107716
ENSBTAP00000012863.4	ENSBTAG00000009755	0.987	0.65448	-0.01888	0.184104
ENSBTAP00000012955.6	ENSBTAG00000009826	0.975	0.5728	-0.03653	0.241997
ENSBTAP00000012969.3	ENSBTAG00000009839	NA	NA	NA	NA
ENSBTAP00000013077.2	ENSBTAG00000033217	1.205	0.195055	0.269033	0.709843
ENSBTAP00000013078.2	ENSBTAG00000009914	1.044	0.76958	0.062122	0.113746
ENSBTAP00000013079.2	ENSBTAG00000009908	NA	NA	NA	NA
ENSBTAP00000013082.3	ENSBTAG00000009902	1.048	0.5282	0.067639	0.277202
ENSBTAP00000013123.2	ENSBTAG00000009949	0.85	0.23572	-0.23447	0.627604
ENSBTAP00000013127.5	ENSBTAG00000009951	0.936	0.30586	-0.09542	0.514477
ENSBTAP00000013135.5	ENSBTAG00000009960	0.995	0.94762	-0.00723	0.023366
ENSBTAP00000013139.4	ENSBTAG00000009962	NA	NA	NA	NA
ENSBTAP00000013144.3	ENSBTAG00000009965	1.12	0.26858	0.163499	0.570926
ENSBTAP00000013207.5	ENSBTAG00000010013	1.443	0.085961	0.529071	1.065699
ENSBTAP00000013231.4	ENSBTAG00000040295	NA	NA	NA	NA

ENSBTAP00000013288.6	ENSBTAG00000010073	0.907	0.4173	-0.14083	0.379552
ENSBTAP00000013316.5	ENSBTAG00000003345	0.982	0.6219	-0.02621	0.206279
ENSBTAP00000013367.5	ENSBTAG00000010132	1.198	0.009179	0.260628	2.037186
ENSBTAP00000013373.6	ENSBTAG00000010136	1.046	0.49178	0.064883	0.308229
ENSBTAP00000013402.5	ENSBTAG00000010156	0.914	0.43356	-0.12973	0.362951
ENSBTAP00000013424.5	ENSBTAG00000010167	0.991	0.95334	-0.01304	0.020752
ENSBTAP00000013451.3	ENSBTAG00000010193	1.094	0.57562	0.129613	0.239864
ENSBTAP00000013568.2	ENSBTAG00000010270	1.122	0.193462	0.166073	0.713404
ENSBTAP00000013587.4	ENSBTAG00000010284	1.132	0.20438	0.178874	0.689562
ENSBTAP00000013600.4	ENSBTAG00000010297	0.988	0.91662	-0.01742	0.037811
ENSBTAP00000013623.6	ENSBTAG00000010312	0.959	0.7643	-0.0604	0.116736
ENSBTAP00000013636.5	ENSBTAG00000010322	NA	NA	NA	NA
ENSBTAP00000013650.3	ENSBTAG00000010336	1.02	0.77474	0.028569	0.110844
ENSBTAP00000013663.4	ENSBTAG00000010347	NA	NA	NA	NA
ENSBTAP00000013671.6	ENSBTAG00000010356	1.006	0.7854	0.00863	0.104909
ENSBTAP00000013713.5	ENSBTAG00000010389	1.009	0.87492	0.012926	0.058032
ENSBTAP00000013734.4	ENSBTAG00000012739	1.054	0.70398	0.075875	0.15244
ENSBTAP00000013737.6	ENSBTAG00000010402	1.074	0.190696	0.102994	0.719658
ENSBTAP00000013796.2	ENSBTAG00000010451	1.069	0.089116	0.096262	1.050044
ENSBTAP00000013841.6	ENSBTAG00000010478	NA	NA	NA	NA
ENSBTAP00000013845.2	ENSBTAG00000010481	1.068	0.15246	0.094912	0.816844
ENSBTAP00000013873.5	ENSBTAG00000010503	1.067	0.55526	0.09356	0.255504
ENSBTAP00000013889.4	ENSBTAG00000010508	1.091	0.38894	0.125651	0.410117
ENSBTAP00000013895.6	ENSBTAG00000010504	NA	NA	NA	NA
ENSBTAP00000013932.5	ENSBTAG00000045785	0.972	0.51004	-0.04097	0.292396
ENSBTAP00000013956.3	ENSBTAG00000010555	NA	NA	NA	NA
ENSBTAP00000013988.6	ENSBTAG00000010579	0.885	0.70052	-0.17625	0.154579
ENSBTAP00000013990.5	ENSBTAG00000010587	0.826	0.2252	-0.27579	0.647432
ENSBTAP00000014027.5	ENSBTAG00000010612	1.017	0.80192	0.02432	0.095869
ENSBTAP00000014053.4	ENSBTAG00000010624	0.984	0.79458	-0.02327	0.099862
ENSBTAP00000014176.5	ENSBTAG00000010709	1.154	0.12916	0.206643	0.888872
ENSBTAP00000014213.4	ENSBTAG00000010735	1.137	0.41	0.185232	0.387216
ENSBTAP00000014218.3	ENSBTAG00000010740	1.003	0.9765	0.004322	0.010328
ENSBTAP00000014219.5	ENSBTAG00000010741	0.97	0.63184	-0.04394	0.199393
ENSBTAP00000014281.5	ENSBTAG00000010790	1.075	0.23144	0.104337	0.635562
ENSBTAP00000014306.5	ENSBTAG00000010799	1.131	0.073083	0.177599	1.136184
ENSBTAP00000014308.4	ENSBTAG00000010801	0.922	0.52864	-0.11716	0.27684
ENSBTAP00000014377.3	ENSBTAG00000010835	NA	NA	NA	NA
ENSBTAP00000014390.3	ENSBTAG00000010843	0.941	0.32472	-0.08773	0.488491
ENSBTAP00000014452.6	ENSBTAG00000010880	0.913	0.2696	-0.13131	0.56928
ENSBTAP00000014486.6	ENSBTAG00000010907	0.987	0.76158	-0.01888	0.118284
ENSBTAP00000014505.6	ENSBTAG00000010919	0.993	0.94766	-0.01013	0.023347
ENSBTAP00000014563.5	ENSBTAG00000010968	1.027	0.63774	0.038436	0.195356
ENSBTAP00000014585.3	ENSBTAG00000010991	1.072	0.6209	0.100305	0.206978
ENSBTAP00000014642.3	ENSBTAG00000011022	1.13	0.190261	0.176323	0.72065
ENSBTAP00000014645.4	ENSBTAG00000011024	1.072	0.5576	0.100305	0.253677
ENSBTAP00000014649.3	ENSBTAG00000011027	NA	NA	NA	NA
ENSBTAP00000014665.3	ENSBTAG00000011045	1.159	0.33768	0.212881	0.471495
ENSBTAP00000014699.5	ENSBTAG00000011070	1.009	0.82306	0.012926	0.084569
ENSBTAP00000014702.5	ENSBTAG00000011072	1.025	0.70496	0.035624	0.151836
ENSBTAP00000014744.2	ENSBTAG00000011104	0.95	0.27448	-0.074	0.561489
ENSBTAP00000014754.5	ENSBTAG00000011111	0.918	0.142076	-0.12343	0.847479
ENSBTAP00000014796.6	ENSBTAG00000011140	1.099	0.33998	0.136191	0.468547
ENSBTAP00000014801.2	ENSBTAG00000011145	1.163	0.168577	0.217851	0.773202
ENSBTAP00000014805.5	ENSBTAG00000011150	1.02	0.84822	0.028569	0.071491
ENSBTAP00000014850.2	ENSBTAG00000011182	1.145	0.189895	0.195348	0.721486
ENSBTAP00000014878.4	ENSBTAG00000011202	1.066	0.29404	0.092207	0.531594
ENSBTAP00000014883.5	ENSBTAG00000011207	NA	NA	NA	NA

ENSBTAP00000014888.3	ENSBTAG00000011212	0.975	0.5603	-0.03653	0.251579
ENSBTAP00000014910.6	ENSBTAG00000011226	0.966	0.103685	-0.0499	0.984284
ENSBTAP00000015071.6	ENSBTAG00000011333	1.17	0.22914	0.226509	0.639899
ENSBTAP00000015136.4	ENSBTAG00000011388	1.134	0.23776	0.181421	0.623861
ENSBTAP00000015166.6	ENSBTAG00000011412	1.018	0.84756	0.025738	0.07183
ENSBTAP00000015172.4	ENSBTAG00000011419	1.11	0.32468	0.15056	0.488544
ENSBTAP00000015186.5	ENSBTAG00000011424	0.998	0.9153	-0.00289	0.038437
ENSBTAP00000015199.5	ENSBTAG00000011435	1.015	0.71586	0.02148	0.145172
ENSBTAP00000015235.4	ENSBTAG00000011465	0.707	0.011545	-0.50022	1.937625
ENSBTAP00000015238.4	ENSBTAG00000011466	1.018	0.7401	0.025738	0.13071
ENSBTAP00000015282.5	ENSBTAG00000006531	0.919	0.48994	-0.12186	0.309857
ENSBTAP00000015285.5	ENSBTAG00000011500	0.997	0.79356	-0.00433	0.10042
ENSBTAP00000015290.5	ENSBTAG00000011505	1.064	0.5764	0.089498	0.239276
ENSBTAP00000015348.6	ENSBTAG00000011548	0.926	0.31854	-0.11092	0.496836
ENSBTAP00000015354.4	ENSBTAG00000011553	0.999	0.94712	-0.00144	0.023595
ENSBTAP00000015358.3	ENSBTAG00000011559	0.867	0.33976	-0.2059	0.468828
ENSBTAP00000015362.6	ENSBTAG00000011562	1.063	0.33386	0.088142	0.476436
ENSBTAP00000015548.2	ENSBTAG00000011709	1.191	0.174905	0.252173	0.757198
ENSBTAP00000015575.5	ENSBTAG00000011726	1.162	0.077202	0.21661	1.112371
ENSBTAP00000015579.2	ENSBTAG00000011730	1.244	0.04366	0.314986	1.359916
ENSBTAP00000015581.5	ENSBTAG00000006982	1.065	0.47168	0.090853	0.326353
ENSBTAP00000015584.3	ENSBTAG00000011734	1.351	0.42166	0.434028	0.375038
ENSBTAP00000015606.5	ENSBTAG00000011752	0.976	0.70534	-0.03505	0.151601
ENSBTAP00000015631.5	ENSBTAG00000011772	NA	NA	NA	NA
ENSBTAP00000015668.5	ENSBTAG00000011802	0.994	0.94922	-0.00868	0.022633
ENSBTAP00000015694.3	ENSBTAG00000011824	0.994	0.89296	-0.00868	0.049168
ENSBTAP00000015716.4	ENSBTAG00000011843	1.077	0.177496	0.107018	0.750811
ENSBTAP00000015719.5	ENSBTAG00000011837	1.102	0.159083	0.140124	0.798376
ENSBTAP00000015873.4	ENSBTAG00000011960	1.233	0.045455	0.302173	1.342418
ENSBTAP00000015875.5	ENSBTAG00000011963	1.039	0.106318	0.055196	0.973393
ENSBTAP00000015881.5	ENSBTAG00000011966	1.034	0.45282	0.048236	0.344074
ENSBTAP00000015889.5	ENSBTAG00000011975	1.003	0.92928	0.004322	0.031853
ENSBTAP00000015907.4	ENSBTAG00000011991	0.992	0.75786	-0.01159	0.120411
ENSBTAP00000015924.4	ENSBTAG00000012003	0.932	0.52122	-0.1016	0.282979
ENSBTAP00000015941.6	ENSBTAG00000012015	0.253	0.37414	-1.98279	0.426966
ENSBTAP00000015996.1	ENSBTAG00000012062	1.187	0.092618	0.24732	1.033305
ENSBTAP00000016093.3	ENSBTAG00000012125	0.758	0.28968	-0.39973	0.538081
ENSBTAP00000016111.6	ENSBTAG00000012146	0.997	0.9641	-0.00433	0.015878
ENSBTAP00000016130.5	ENSBTAG00000012159	0.966	0.40806	-0.0499	0.389276
ENSBTAP00000016153.3	ENSBTAG00000012177	1.067	0.166583	0.09356	0.778369
ENSBTAP00000016224.5	ENSBTAG00000012232	1.097	0.38222	0.133564	0.417687
ENSBTAP00000016263.3	ENSBTAG00000012259	NA	NA	NA	NA
ENSBTAP00000016300.6	ENSBTAG00000012289	0.978	0.62136	-0.03209	0.206657
ENSBTAP00000016315.6	ENSBTAG00000031261	NA	NA	NA	NA
ENSBTAP00000016346.6	ENSBTAG00000012317	1.099	0.156182	0.136191	0.806369
ENSBTAP00000016359.2	ENSBTAG00000012330	NA	NA	NA	NA
ENSBTAP00000016432.6	ENSBTAG00000012380	NA	NA	NA	NA
ENSBTAP00000016503.6	ENSBTAG00000012434	0.925	0.163861	-0.11247	0.785524
ENSBTAP00000016505.3	ENSBTAG00000030255	1.035	0.46872	0.049631	0.329087
ENSBTAP00000016564.3	ENSBTAG00000012484	NA	NA	NA	NA
ENSBTAP00000016570.3	ENSBTAG00000012490	1.113	0.37102	0.154454	0.430603
ENSBTAP00000016601.5	ENSBTAG00000040569	1.006	0.95734	0.00863	0.018934
ENSBTAP00000016661.6	ENSBTAG00000012550	1.221	0.3312	0.288063	0.47991
ENSBTAP00000016712.3	ENSBTAG00000012589	1.033	0.85212	0.04684	0.069499
ENSBTAP00000016718.4	ENSBTAG00000012595	0.994	0.96114	-0.00868	0.017213
ENSBTAP00000016768.3	ENSBTAG00000012634	1.047	0.37084	0.066261	0.430813
ENSBTAP00000016780.2	ENSBTAG00000012649	1.008	0.8869	0.011496	0.052125
ENSBTAP00000016865.6	ENSBTAG00000001842	0.439	0.063299	-1.18771	1.198603

ENSBTAP00000016866.6	ENSBTAG00000012692	0.821	0.20576	-0.28455	0.686639
ENSBTAP00000016884.4	ENSBTAG00000012703	0.831	0.195957	-0.26708	0.707839
ENSBTAP00000016907.6	ENSBTAG00000012720	1.132	0.65916	0.178874	0.181009
ENSBTAP00000016918.2	ENSBTAG00000012726	0.972	0.69616	-0.04097	0.157291
ENSBTAP00000016937.4	ENSBTAG00000012744	1.068	0.32074	0.094912	0.493847
ENSBTAP00000016957.3	ENSBTAG00000012760	1.045	0.69144	0.063503	0.160246
ENSBTAP00000017009.4	ENSBTAG00000012804	0.785	0.26276	-0.34924	0.580441
ENSBTAP00000017030.4	ENSBTAG00000012818	1.019	0.92774	0.027154	0.032574
ENSBTAP00000017122.4	ENSBTAG00000012885	1.127	0.55754	0.172488	0.253724
ENSBTAP00000017145.6	ENSBTAG00000012897	NA	NA	NA	NA
ENSBTAP00000017150.2	ENSBTAG00000012902	1.009	0.81304	0.012926	0.089888
ENSBTAP00000017182.5	ENSBTAG00000012931	1.818	0.1475	0.862352	0.831208
ENSBTAP00000017188.6	ENSBTAG00000012937	0.831	0.4048	-0.26708	0.392759
ENSBTAP00000017211.5	ENSBTAG00000012957	1.017	0.77202	0.02432	0.112371
ENSBTAP00000017232.3	ENSBTAG00000012962	0.975	0.79338	-0.03653	0.100519
ENSBTAP00000017239.5	ENSBTAG00000012966	0.847	0.02822	-0.23957	1.549443
ENSBTAP00000017251.1	ENSBTAG00000012975	1.071	0.48224	0.098958	0.316737
ENSBTAP00000017298.5	ENSBTAG00000013011	0.963	0.41284	-0.05439	0.384218
ENSBTAP00000017301.5	ENSBTAG00000013013	1.029	0.55212	0.041243	0.257967
ENSBTAP00000017416.5	ENSBTAG00000013099	0.962	0.51368	-0.05589	0.289307
ENSBTAP00000017420.3	ENSBTAG00000013103	1.067	0.8898	0.09356	0.050708
ENSBTAP00000017425.5	ENSBTAG00000013109	0.973	0.60232	-0.03949	0.220173
ENSBTAP00000017435.6	ENSBTAG00000038540	0.944	0.9298	-0.08314	0.03161
ENSBTAP00000017497.2	ENSBTAG00000013162	1.03	0.54904	0.042644	0.260396
ENSBTAP00000017500.4	ENSBTAG00000025441	1.019	0.87352	0.027154	0.058727
ENSBTAP00000017503.3	ENSBTAG00000003418	1.026	0.884	0.037031	0.053548
ENSBTAP00000017505.5	ENSBTAG00000013155	1.072	0.7112	0.100305	0.148008
ENSBTAP00000017580.2	ENSBTAG00000013208	1.139	0.3295	0.187768	0.482145
ENSBTAP00000017629.5	ENSBTAG00000013254	1.076	0.64862	0.105678	0.18801
ENSBTAP00000017696.5	ENSBTAG00000013308	1.006	0.7586	0.00863	0.119987
ENSBTAP00000017710.6	ENSBTAG00000013315	1.068	0.47736	0.094912	0.321154
ENSBTAP00000017770.3	ENSBTAG00000013358	0.851	0.30724	-0.23277	0.512522
ENSBTAP00000017775.5	ENSBTAG00000013363	1.057	0.5238	0.079975	0.280835
ENSBTAP00000017812.5	ENSBTAG00000013387	1.085	0.25318	0.117695	0.596571
ENSBTAP00000017816.2	ENSBTAG00000013390	0.967	0.41408	-0.04841	0.382916
ENSBTAP00000017835.5	ENSBTAG00000013406	NA	NA	NA	NA
ENSBTAP00000017839.5	ENSBTAG00000013411	NA	NA	NA	NA
ENSBTAP00000017860.3	ENSBTAG00000013423	1.129	0.47394	0.175045	0.324277
ENSBTAP00000017905.3	ENSBTAG00000013461	NA	NA	NA	NA
ENSBTAP00000017988.6	ENSBTAG00000013527	1.052	0.03254	0.073135	1.487582
ENSBTAP00000017995.2	ENSBTAG00000013533	NA	NA	NA	NA
ENSBTAP00000018092.5	ENSBTAG00000013607	1.018	0.80628	0.025738	0.093514
ENSBTAP00000018099.4	ENSBTAG00000013614	0.915	0.5095	-0.12816	0.292856
ENSBTAP00000018109.5	ENSBTAG00000013623	1.201	0.108898	0.264236	0.96298
ENSBTAP00000018110.6	ENSBTAG00000013624	0.975	0.68908	-0.03653	0.16173
ENSBTAP00000018114.5	ENSBTAG00000013627	1.011	0.78748	0.015783	0.10376
ENSBTAP00000018131.4	ENSBTAG00000013641	1.067	0.008184	0.09356	2.087029
ENSBTAP00000018167.5	ENSBTAG00000013669	0.992	0.97126	-0.01159	0.012664
ENSBTAP00000018226.4	ENSBTAG00000013721	1.117	0.062319	0.159629	1.20538
ENSBTAP00000018283.3	ENSBTAG00000036028	1.12	0.6671	0.163499	0.175809
ENSBTAP00000018325.4	ENSBTAG00000013790	1.216	0.160242	0.282143	0.795224
ENSBTAP00000018327.4	ENSBTAG00000013782	1.007	0.98602	0.010064	0.006114
ENSBTAP00000018391.6	ENSBTAG00000047424	0.902	0.124883	-0.1488	0.903497
ENSBTAP00000018414.5	ENSBTAG00000013866	1.032	0.59654	0.045443	0.22436
ENSBTAP00000018429.2	ENSBTAG00000013882	1.067	0.39376	0.09356	0.404768
ENSBTAP00000018473.7	ENSBTAG00000013912	1.544	0.034776	0.626673	1.45872
ENSBTAP00000018479.6	ENSBTAG00000030190	1.021	0.79284	0.029983	0.100814
ENSBTAP00000018491.1	ENSBTAG00000013924	0.961	0.53444	-0.05739	0.272101

ENSBTAP00000018492.2	ENSBTAG00000013921	0.964	0.70994	-0.05289	0.148778
ENSBTAP00000018501.4	ENSBTAG00000013929	1.016	0.81922	0.0229	0.086599
ENSBTAP00000018514.3	ENSBTAG00000013935	NA	NA	NA	NA
ENSBTAP00000018555.4	ENSBTAG00000013956	1.089	0.106155	0.123004	0.97406
ENSBTAP00000018566.4	ENSBTAG00000013953	1.118	0.127221	0.16092	0.895441
ENSBTAP00000018595.2	ENSBTAG00000013992	1.028	0.042778	0.03984	1.36878
ENSBTAP00000018626.3	ENSBTAG00000014024	0.965	0.052764	-0.0514	1.277662
ENSBTAP00000018658.4	ENSBTAG00000014042	1.378	0.144181	0.462576	0.841092
ENSBTAP00000018720.6	ENSBTAG00000014079	1.031	0.99482	0.044044	0.002255
ENSBTAP00000018741.4	ENSBTAG00000014093	1.094	0.64896	0.129613	0.187782
ENSBTAP00000018753.4	ENSBTAG00000014102	0.985	0.75004	-0.0218	0.124916
ENSBTAP00000018777.5	ENSBTAG00000014129	1.123	0.055284	0.167358	1.257401
ENSBTAP00000018840.5	ENSBTAG00000014175	1.003	0.86326	0.004322	0.063858
ENSBTAP00000018845.5	ENSBTAG00000014177	NA	NA	NA	NA
ENSBTAP00000018886.5	ENSBTAG00000014205	1.048	0.5577	0.067639	0.253599
ENSBTAP00000018888.3	ENSBTAG00000014208	0.966	0.73102	-0.0499	0.136071
ENSBTAP00000018917.2	ENSBTAG00000014233	1.041	0.56096	0.05797	0.251068
ENSBTAP00000018947.3	ENSBTAG00000014261	1.076	0.11992	0.105678	0.921108
ENSBTAP00000019001.5	ENSBTAG00000014302	1.046	0.5824	0.064883	0.234779
ENSBTAP00000019039.4	ENSBTAG00000014316	0.995	0.9688	-0.00723	0.013766
ENSBTAP00000019064.5	ENSBTAG00000014337	0.977	0.28074	-0.03357	0.551696
ENSBTAP00000019176.6	ENSBTAG00000014417	1.039	0.56666	0.055196	0.246677
ENSBTAP00000019184.5	ENSBTAG00000014423	0.989	0.52296	-0.01596	0.281532
ENSBTAP00000019203.2	ENSBTAG00000014440	0.973	0.48212	-0.03949	0.316845
ENSBTAP00000019278.4	ENSBTAG00000014501	0.95	0.48736	-0.074	0.31215
ENSBTAP00000019336.2	ENSBTAG00000014547	0.948	0.47096	-0.07704	0.327016
ENSBTAP00000019338.5	ENSBTAG00000014548	1.079	0.76992	0.109695	0.113554
ENSBTAP00000019385.6	ENSBTAG00000014567	0.892	0.41544	-0.16488	0.381492
ENSBTAP00000019475.3	ENSBTAG00000014626	1.023	0.67142	0.032806	0.173006
ENSBTAP00000019505.4	ENSBTAG00000014649	1.172	0.32608	0.228973	0.486676
ENSBTAP00000019529.5	ENSBTAG00000014669	1.058	0.5245	0.08134	0.280255
ENSBTAP00000019556.3	ENSBTAG00000014695	1.144	0.37668	0.194087	0.424027
ENSBTAP00000019566.5	ENSBTAG00000014700	0.969	0.5351	-0.04543	0.271565
ENSBTAP00000019575.5	ENSBTAG00000008967	NA	NA	NA	NA
ENSBTAP00000019585.6	ENSBTAG00000014719	1.108	0.43056	0.147958	0.365966
ENSBTAP00000019596.4	ENSBTAG00000014724	1.001	0.9801	0.001442	0.00873
ENSBTAP00000019702.3	ENSBTAG00000014805	NA	NA	NA	NA
ENSBTAP00000019803.3	ENSBTAG00000014872	1.002	0.96622	0.002883	0.014924
ENSBTAP00000019808.5	ENSBTAG00000014878	1.164	0.20962	0.219091	0.678567
ENSBTAP00000019817.6	ENSBTAG00000014885	NA	NA	NA	NA
ENSBTAP00000019854.5	ENSBTAG00000014912	NA	NA	NA	NA
ENSBTAP00000019861.4	ENSBTAG00000014918	NA	NA	NA	NA
ENSBTAP00000019872.4	ENSBTAG00000014927	1.053	0.24412	0.074505	0.612397
ENSBTAP00000019913.5	ENSBTAG00000014956	1.015	0.7694	0.02148	0.113848
ENSBTAP00000019921.6	ENSBTAG00000014964	1.061	0.195045	0.085425	0.709865
ENSBTAP00000019924.2	ENSBTAG00000014967	1.192	0.064079	0.253384	1.193284
ENSBTAP00000019973.3	ENSBTAG00000015004	1.041	0.26132	0.05797	0.582827
ENSBTAP00000019994.6	ENSBTAG00000015018	0.982	0.80478	-0.02621	0.094323
ENSBTAP00000020018.1	ENSBTAG00000015041	1.035	0.80498	0.049631	0.094215
ENSBTAP00000020020.3	ENSBTAG00000015038	1.125	0.37154	0.169925	0.429994
ENSBTAP00000020036.3	ENSBTAG00000015053	0.987	0.71608	-0.01888	0.145038
ENSBTAP00000020080.5	ENSBTAG00000015089	0.942	0.5226	-0.0862	0.281831
ENSBTAP00000020092.6	ENSBTAG00000015099	NA	NA	NA	NA
ENSBTAP00000020102.2	ENSBTAG00000015107	1.084	0.30088	0.116365	0.521607
ENSBTAP00000020108.2	ENSBTAG00000015112	1.042	0.43592	0.059355	0.360593
ENSBTAP00000020111.2	ENSBTAG00000015114	1.012	0.8178	0.017209	0.087353
ENSBTAP00000020148.5	ENSBTAG00000015145	0.98	0.81514	-0.02915	0.088768
ENSBTAP00000020150.3	ENSBTAG00000015147	1.017	0.8693	0.02432	0.06083

ENSBTAP00000020160.4	ENSBTAG00000015156	1.029	0.839	0.041243	0.076238
ENSBTAP00000020184.5	ENSBTAG00000015169	NA	NA	NA	NA
ENSBTAP00000020196.6	ENSBTAG00000015178	1.159	0.122881	0.212881	0.910515
ENSBTAP00000020200.5	ENSBTAG00000015181	1.015	0.81616	0.02148	0.088225
ENSBTAP00000020226.3	ENSBTAG00000015200	1.091	0.44288	0.125651	0.353714
ENSBTAP00000020228.3	ENSBTAG00000015202	1.026	0.76098	0.037031	0.118627
ENSBTAP00000020229.2	ENSBTAG00000015204	0.965	0.50424	-0.0514	0.297363
ENSBTAP00000020243.2	ENSBTAG00000015214	1.01	0.86436	0.014355	0.063305
ENSBTAP00000020277.6	ENSBTAG00000015240	NA	NA	NA	NA
ENSBTAP00000020326.6	ENSBTAG00000015285	0.896	0.603	-0.15843	0.219683
ENSBTAP00000020388.4	ENSBTAG00000015336	1.127	0.188699	0.172488	0.72423
ENSBTAP00000020406.5	ENSBTAG00000015350	1.122	0.58416	0.166073	0.233468
ENSBTAP00000020561.5	ENSBTAG00000015470	0.925	0.012221	-0.11247	1.912879
ENSBTAP00000020608.4	ENSBTAG00000015509	1.137	0.112101	0.185232	0.950391
ENSBTAP00000020644.5	ENSBTAG00000015536	1.037	0.40452	0.052416	0.39306
ENSBTAP00000020672.5	ENSBTAG00000015556	NA	NA	NA	NA
ENSBTAP00000020681.6	ENSBTAG00000015567	1.044	0.67568	0.062122	0.170259
ENSBTAP00000020701.5	ENSBTAG00000015582	NA	NA	NA	NA
ENSBTAP00000020709.5	ENSBTAG00000015591	1.019	0.66702	0.027154	0.175861
ENSBTAP00000020719.5	ENSBTAG00000015598	0.979	0.60216	-0.03062	0.220288
ENSBTAP00000020809.2	ENSBTAG00000015663	0.973	0.78916	-0.03949	0.102835
ENSBTAP00000020818.4	ENSBTAG00000015677	0.967	0.56496	-0.04841	0.247982
ENSBTAP00000020825.5	ENSBTAG00000015683	1.045	0.66242	0.063503	0.178867
ENSBTAP00000020886.5	ENSBTAG00000015732	0.951	0.2253	-0.07248	0.647239
ENSBTAP00000020904.5	ENSBTAG00000015743	0.9	0.52616	-0.152	0.278882
ENSBTAP00000020908.2	ENSBTAG00000007586	NA	NA	NA	NA
ENSBTAP00000020929.4	ENSBTAG00000015761	1.2	0.143925	0.263034	0.841864
ENSBTAP00000020933.6	ENSBTAG00000015764	1.11	0.112065	0.15056	0.95053
ENSBTAP00000020950.3	ENSBTAG00000015780	NA	NA	NA	NA
ENSBTAP00000020951.5	ENSBTAG00000015778	0.88	0.34942	-0.18442	0.456652
ENSBTAP00000020974.6	ENSBTAG00000015794	0.921	0.72214	-0.11873	0.141379
ENSBTAP00000021033.2	ENSBTAG00000015831	0.927	0.26914	-0.10936	0.570022
ENSBTAP00000021068.5	ENSBTAG00000015848	0.929	0.4788	-0.10625	0.319846
ENSBTAP00000021123.3	ENSBTAG00000015892	1.09	0.186662	0.124328	0.728944
ENSBTAP00000021129.6	ENSBTAG00000015896	NA	NA	NA	NA
ENSBTAP00000021156.6	ENSBTAG00000015910	1.067	0.46688	0.09356	0.330795
ENSBTAP00000021166.5	ENSBTAG00000015917	0.988	0.7316	-0.01742	0.135726
ENSBTAP00000021189.6	ENSBTAG00000015926	1.078	0.191103	0.108357	0.718732
ENSBTAP00000021256.5	ENSBTAG00000015978	0.979	0.88592	-0.03062	0.052605
ENSBTAP00000021267.3	ENSBTAG00000026283	0.888	0.035143	-0.17137	1.454161
ENSBTAP00000021268.3	ENSBTAG00000015989	1.057	0.2702	0.079975	0.568315
ENSBTAP00000021305.4	ENSBTAG00000016005	NA	NA	NA	NA
ENSBTAP00000021357.4	ENSBTAG00000016048	1.062	0.46294	0.086784	0.334475
ENSBTAP00000021369.5	ENSBTAG00000016057	1.009	0.85976	0.012926	0.065623
ENSBTAP00000021425.4	ENSBTAG00000016093	NA	NA	NA	NA
ENSBTAP00000021434.5	ENSBTAG00000016103	1.082	0.49088	0.1137	0.309025
ENSBTAP00000021517.5	ENSBTAG00000016161	1.009	0.95268	0.012926	0.021053
ENSBTAP00000021569.4	ENSBTAG00000016208	1.139	0.34624	0.187768	0.460623
ENSBTAP00000021584.6	ENSBTAG00000016218	0.92	0.60744	-0.12029	0.216497
ENSBTAP00000021587.3	ENSBTAG00000016224	0.976	0.65204	-0.03505	0.185726
ENSBTAP00000021637.4	ENSBTAG00000016265	1.12	0.34052	0.163499	0.467857
ENSBTAP00000021671.5	ENSBTAG00000016290	1.011	0.88926	0.015783	0.050971
ENSBTAP00000021683.5	ENSBTAG00000016296	1.047	0.58854	0.066261	0.230224
ENSBTAP00000021691.3	ENSBTAG00000016302	NA	NA	NA	NA
ENSBTAP00000021704.4	ENSBTAG00000016315	NA	NA	NA	NA
ENSBTAP00000021719.5	ENSBTAG00000016328	0.988	0.84314	-0.01742	0.0741
ENSBTAP00000021731.5	ENSBTAG00000016337	0.974	0.7825	-0.03801	0.106516
ENSBTAP00000021773.6	ENSBTAG00000016368	1.086	0.63466	0.119024	0.197459

ENSBTAP00000021808.3	ENSBTAG00000016401	NA	NA	NA	NA
ENSBTAP00000021895.2	ENSBTAG00000016471	1.062	0.36432	0.086784	0.438517
ENSBTAP00000021896.5	ENSBTAG00000037673	0.937	0.6082	-0.09388	0.215954
ENSBTAP00000021909.3	ENSBTAG00000016481	1.065	0.7051	0.090853	0.151749
ENSBTAP00000022054.4	ENSBTAG00000016578	NA	NA	NA	NA
ENSBTAP00000022082.3	ENSBTAG00000016599	1.13	0.36266	0.176323	0.4405
ENSBTAP00000022098.5	ENSBTAG00000016612	NA	NA	NA	NA
ENSBTAP00000022108.4	ENSBTAG00000016618	0.967	0.66062	-0.04841	0.180048
ENSBTAP00000022146.3	ENSBTAG00000016650	NA	NA	NA	NA
ENSBTAP00000022180.3	ENSBTAG00000016680	1.068	0.62944	0.094912	0.201046
ENSBTAP00000022210.6	ENSBTAG00000016708	1.029	0.61894	0.041243	0.208351
ENSBTAP00000022212.2	ENSBTAG00000016710	0.991	0.85178	-0.01304	0.069673
ENSBTAP00000022232.6	ENSBTAG00000016724	1.099	0.009598	0.136191	2.017828
ENSBTAP00000022292.4	ENSBTAG00000016762	0.997	0.95322	-0.00433	0.020807
ENSBTAP00000022299.5	ENSBTAG00000016764	1.023	0.61162	0.032806	0.213518
ENSBTAP00000022314.2	ENSBTAG00000016783	0.999	0.99014	-0.00144	0.004303
ENSBTAP00000022364.4	ENSBTAG00000016810	0.984	0.7993	-0.02327	0.09729
ENSBTAP00000022375.4	ENSBTAG00000016819	1.037	0.85566	0.052416	0.067699
ENSBTAP00000022378.4	ENSBTAG00000016822	0.967	0.47664	-0.04841	0.32181
ENSBTAP00000022382.4	ENSBTAG00000010723	1.118	0.104241	0.16092	0.981961
ENSBTAP00000022386.5	ENSBTAG00000016828	NA	NA	NA	NA
ENSBTAP00000022411.2	ENSBTAG00000016846	1.095	0.095262	0.130931	1.02108
ENSBTAP00000022474.6	ENSBTAG00000025324	NA	NA	NA	NA
ENSBTAP00000022534.5	ENSBTAG00000016943	0.906	0.042381	-0.14242	1.372829
ENSBTAP00000022548.4	ENSBTAG00000016952	1.006	0.85376	0.00863	0.068664
ENSBTAP00000022552.4	ENSBTAG00000016951	1.039	0.60274	0.055196	0.21987
ENSBTAP00000022579.2	ENSBTAG00000016977	1.035	0.47302	0.049631	0.32512
ENSBTAP00000022592.2	ENSBTAG00000016988	1.039	0.66366	0.055196	0.178054
ENSBTAP00000022601.5	ENSBTAG00000016995	1.021	0.79416	0.029983	0.100092
ENSBTAP00000022642.4	ENSBTAG00000017028	1.034	0.190417	0.048236	0.720294
ENSBTAP00000022679.4	ENSBTAG00000017055	0.923	0.003365	-0.1156	2.473067
ENSBTAP00000022744.5	ENSBTAG00000017122	1.092	0.046481	0.126973	1.332725
ENSBTAP00000022761.4	ENSBTAG00000017125	1.023	0.36356	0.032806	0.439424
ENSBTAP00000022789.3	ENSBTAG00000017147	1.118	0.45674	0.16092	0.340331
ENSBTAP00000022832.3	ENSBTAG00000017181	1.029	0.72944	0.041243	0.13701
ENSBTAP00000022854.3	ENSBTAG00000017196	0.995	0.92678	-0.00723	0.033023
ENSBTAP00000022932.5	ENSBTAG00000017253	1.05	0.38128	0.070389	0.418756
ENSBTAP00000022946.3	ENSBTAG00000017265	0.957	0.006555	-0.06341	2.183427
ENSBTAP00000022949.3	ENSBTAG00000017267	1.144	0.24566	0.194087	0.609666
ENSBTAP00000022970.3	ENSBTAG00000017279	0.99	0.86664	-0.0145	0.062161
ENSBTAP00000022993.2	ENSBTAG00000017298	1.088	0.098196	0.121679	1.007906
ENSBTAP00000023026.5	ENSBTAG00000011893	1.024	0.60272	0.034216	0.219884
ENSBTAP00000023082.6	ENSBTAG00000030499	NA	NA	NA	NA
ENSBTAP00000023098.6	ENSBTAG00000017373	1.046	0.53108	0.064883	0.27484
ENSBTAP00000023111.6	ENSBTAG00000017380	1.028	0.62906	0.03984	0.201308
ENSBTAP00000023150.6	ENSBTAG00000017416	1.008	0.806	0.011496	0.093665
ENSBTAP00000023164.4	ENSBTAG00000017424	1.006	0.91428	0.00863	0.038921
ENSBTAP00000023165.6	ENSBTAG00000017426	1.009	0.54914	0.012926	0.260317
ENSBTAP00000023183.5	ENSBTAG00000017441	0.923	0.55066	-0.1156	0.259116
ENSBTAP00000023199.6	ENSBTAG00000017457	1.432	0.047739	0.518031	1.321127
ENSBTAP00000023209.5	ENSBTAG00000017461	0.971	0.691	-0.04246	0.160522
ENSBTAP00000023273.5	ENSBTAG00000017509	0.932	0.072861	-0.1016	1.137505
ENSBTAP00000023306.3	ENSBTAG00000017530	NA	NA	NA	NA
ENSBTAP00000023334.4	ENSBTAG00000017554	NA	NA	NA	NA
ENSBTAP00000023373.4	ENSBTAG00000017574	1.042	0.15134	0.059355	0.820046
ENSBTAP00000023406.3	ENSBTAG00000017605	1.166	0.21314	0.221568	0.671335
ENSBTAP00000023455.3	ENSBTAG00000017633	NA	NA	NA	NA
ENSBTAP00000023487.5	ENSBTAG00000017662	1.045	0.32776	0.063503	0.484444

ENSBTAP00000023493.4	ENSBTAG00000017665	NA	NA	NA	NA
ENSBTAP00000023581.4	ENSBTAG00000017733	1.278	0.46086	0.353888	0.336431
ENSBTAP00000023596.5	ENSBTAG00000017747	1.023	0.49372	0.032806	0.306519
ENSBTAP00000023622.5	ENSBTAG00000017765	NA	NA	NA	NA
ENSBTAP00000023642.1	ENSBTAG00000017779	1.063	0.24078	0.088142	0.61838
ENSBTAP00000023671.5	ENSBTAG00000017803	1.007	0.94122	0.010064	0.026309
ENSBTAP00000023709.6	ENSBTAG00000017834	0.966	0.82992	-0.0499	0.080964
ENSBTAP00000023751.3	ENSBTAG00000017869	1.061	0.70112	0.085425	0.154208
ENSBTAP00000023778.5	ENSBTAG00000017889	1.034	0.6918	0.048236	0.160019
ENSBTAP00000023908.5	ENSBTAG00000017967	1.074	0.032782	0.102994	1.484365
ENSBTAP00000023996.5	ENSBTAG00000018026	1.104	0.144961	0.14274	0.838749
ENSBTAP00000024045.6	ENSBTAG00000018061	0.933	0.5338	-0.10005	0.272621
ENSBTAP00000024051.3	ENSBTAG00000018065	1.023	0.68034	0.032806	0.167274
ENSBTAP00000024086.5	ENSBTAG00000022570	1.223	0.58414	0.290424	0.233483
ENSBTAP00000024092.2	ENSBTAG00000018101	0.988	0.83352	-0.01742	0.079084
ENSBTAP00000024094.3	ENSBTAG00000018103	1.075	0.001383	0.104337	2.859143
ENSBTAP00000024107.3	ENSBTAG00000018115	0.891	0.110637	-0.1665	0.9561
ENSBTAP00000024122.2	ENSBTAG00000018123	0.974	0.76786	-0.03801	0.114718
ENSBTAP00000024128.3	ENSBTAG00000018127	1.029	0.76714	0.041243	0.115125
ENSBTAP00000024159.4	ENSBTAG00000018153	NA	NA	NA	NA
ENSBTAP00000024209.3	ENSBTAG00000018188	1.085	0.53726	0.117695	0.269815
ENSBTAP00000024225.5	ENSBTAG00000054131	1.041	0.54272	0.05797	0.265424
ENSBTAP00000024261.5	ENSBTAG00000018228	1.453	0.147282	0.539035	0.83185
ENSBTAP00000024307.5	ENSBTAG00000018261	1.111	0.125703	0.151859	0.900654
ENSBTAP00000024312.4	ENSBTAG00000018267	1.013	0.87748	0.018634	0.056763
ENSBTAP00000024335.2	ENSBTAG00000018283	1.02	0.81244	0.028569	0.090209
ENSBTAP00000024375.5	ENSBTAG00000018318	NA	NA	NA	NA
ENSBTAP00000024376.2	ENSBTAG00000018320	1.011	0.4335	0.015783	0.363011
ENSBTAP00000024422.5	ENSBTAG00000018358	0.978	0.66072	-0.03209	0.179983
ENSBTAP00000024427.2	ENSBTAG00000018362	0.915	0.2402	-0.12816	0.619427
ENSBTAP00000024444.2	ENSBTAG00000018369	1.222	0.180898	0.289244	0.742566
ENSBTAP00000024516.5	ENSBTAG00000018426	0.955	0.30196	-0.06643	0.520051
ENSBTAP00000024531.5	ENSBTAG00000018438	0.997	0.93888	-0.00433	0.02739
ENSBTAP00000024551.6	ENSBTAG00000018447	1.047	0.62132	0.066261	0.206685
ENSBTAP00000024553.6	ENSBTAG00000018449	1.05	0.67756	0.070389	0.169052
ENSBTAP00000024572.3	ENSBTAG00000018463	1.038	0.733	0.053806	0.134896
ENSBTAP00000024600.5	ENSBTAG00000018483	1.044	0.61936	0.062122	0.208057
ENSBTAP00000024641.6	ENSBTAG00000018513	1.001	0.97348	0.001442	0.011673
ENSBTAP00000024663.3	ENSBTAG00000018530	1.078	0.155463	0.108357	0.808373
ENSBTAP00000024749.6	ENSBTAG00000018598	1.058	0.37084	0.08134	0.430813
ENSBTAP00000024782.5	ENSBTAG00000037377	1.158	0.042162	0.211635	1.375079
ENSBTAP00000024823.3	ENSBTAG00000018655	NA	NA	NA	NA
ENSBTAP00000024898.5	ENSBTAG00000018706	NA	NA	NA	NA
ENSBTAP00000024904.5	ENSBTAG00000018707	NA	NA	NA	NA
ENSBTAP00000024956.6	ENSBTAG00000018742	1.086	0.32014	0.119024	0.49466
ENSBTAP00000024965.5	ENSBTAG00000018747	0.91	0.40674	-0.13606	0.390683
ENSBTAP00000025020.4	ENSBTAG00000018796	1.031	0.2317	0.044044	0.635074
ENSBTAP00000025031.3	ENSBTAG00000018803	0.885	0.2358	-0.17625	0.627456
ENSBTAP00000025094.3	ENSBTAG00000018847	0.967	0.55988	-0.04841	0.251905
ENSBTAP00000025174.3	ENSBTAG00000018918	1.065	0.5597	0.090853	0.252045
ENSBTAP00000025179.3	ENSBTAG00000031837	1.025	0.4704	0.035624	0.327533
ENSBTAP00000025191.6	ENSBTAG00000018928	1.034	0.194497	0.048236	0.711087
ENSBTAP00000025254.5	ENSBTAG00000018972	NA	NA	NA	NA
ENSBTAP00000025255.3	ENSBTAG00000018973	0.983	0.4977	-0.02474	0.303032
ENSBTAP00000025308.6	ENSBTAG00000019011	1.014	0.90068	0.020058	0.045429
ENSBTAP00000025318.2	ENSBTAG00000019023	NA	NA	NA	NA
ENSBTAP00000025322.3	ENSBTAG00000019025	0.64	0.7425	-0.64386	0.129304
ENSBTAP00000025371.4	ENSBTAG00000019052	0.987	0.77958	-0.01888	0.108139

ENSBTAP00000025422.3	ENSBTAG00000019096	1.084	0.37172	0.116365	0.429784
ENSBTAP00000025437.3	ENSBTAG00000019106	1	0.9518	0	0.021454
ENSBTAP00000025473.6	ENSBTAG00000019136	1.006	0.70724	0.00863	0.150433
ENSBTAP00000025484.2	ENSBTAG00000019147	NA	NA	NA	NA
ENSBTAP00000025501.6	ENSBTAG00000019160	0.974	0.7789	-0.03801	0.108518
ENSBTAP00000025522.4	ENSBTAG00000019177	0.94	0.20364	-0.08927	0.691137
ENSBTAP00000025561.3	ENSBTAG00000019203	0.895	0.5268	-0.16004	0.278354
ENSBTAP00000025580.6	ENSBTAG00000019214	1.009	0.86926	0.012926	0.06085
ENSBTAP00000025586.1	ENSBTAG00000019216	1.138	0.3043	0.186501	0.516698
ENSBTAP00000025662.5	ENSBTAG00000019274	0.911	0.46544	-0.13448	0.332136
ENSBTAP00000025663.6	ENSBTAG00000019269	1.007	0.9079	0.010064	0.041962
ENSBTAP00000025691.6	ENSBTAG00000019295	1.086	0.32472	0.119024	0.488491
ENSBTAP00000025697.5	ENSBTAG00000019299	0.999	0.96594	-0.00144	0.01505
ENSBTAP00000025728.6	ENSBTAG00000019317	1.078	0.4172	0.108357	0.379656
ENSBTAP00000025807.5	ENSBTAG00000019370	0.978	0.7011	-0.03209	0.15422
ENSBTAP00000025963.4	ENSBTAG00000019494	0.994	0.86046	-0.00868	0.065269
ENSBTAP00000025978.3	ENSBTAG00000019501	NA	NA	NA	NA
ENSBTAP00000025996.5	ENSBTAG00000019512	1.067	0.58992	0.09356	0.229207
ENSBTAP00000026002.3	ENSBTAG00000019521	1.112	0.121861	0.153157	0.914135
ENSBTAP00000026036.5	ENSBTAG00000019543	0.979	0.70758	-0.03062	0.150224
ENSBTAP00000026056.2	ENSBTAG00000019554	1.027	0.62534	0.038436	0.203884
ENSBTAP00000026080.4	ENSBTAG00000019574	1.003	0.95116	0.004322	0.021746
ENSBTAP00000026118.5	ENSBTAG00000019603	1.111	0.55178	0.151859	0.258234
ENSBTAP00000026127.4	ENSBTAG00000019612	NA	NA	NA	NA
ENSBTAP00000026183.3	ENSBTAG00000019648	1.028	0.64176	0.03984	0.192627
ENSBTAP00000026197.4	ENSBTAG00000019658	0.976	0.87718	-0.03505	0.056911
ENSBTAP00000026261.5	ENSBTAG00000019703	1.012	0.73282	0.017209	0.135003
ENSBTAP00000026292.5	ENSBTAG00000019729	1.183	0.036781	0.24245	1.434376
ENSBTAP00000026323.4	ENSBTAG00000019754	1.119	0.4554	0.16221	0.341607
ENSBTAP00000026356.5	ENSBTAG00000019780	0.981	0.43574	-0.02767	0.360773
ENSBTAP00000026358.3	ENSBTAG00000019782	0.946	0.58026	-0.08009	0.236377
ENSBTAP00000026361.4	ENSBTAG00000019784	NA	NA	NA	NA
ENSBTAP00000026393.4	ENSBTAG00000019810	1.051	0.3654	0.071763	0.437231
ENSBTAP00000026407.4	ENSBTAG00000019822	0.951	0.6619	-0.07248	0.179208
ENSBTAP00000026518.4	ENSBTAG00000019906	NA	NA	NA	NA
ENSBTAP00000026525.4	ENSBTAG00000019911	1.13	0.29682	0.176323	0.527507
ENSBTAP00000026548.5	ENSBTAG00000019927	1.065	0.59098	0.090853	0.228427
ENSBTAP00000026568.4	ENSBTAG00000019944	NA	NA	NA	NA
ENSBTAP00000026582.4	ENSBTAG00000019956	0.99	0.90306	-0.0145	0.044283
ENSBTAP00000026607.6	ENSBTAG00000021841	NA	NA	NA	NA
ENSBTAP00000026635.4	ENSBTAG00000019998	0.976	0.71624	-0.03505	0.144941
ENSBTAP00000026725.6	ENSBTAG00000020056	1.39	0.49624	0.475085	0.304308
ENSBTAP00000026756.6	ENSBTAG00000020080	0.951	0.51464	-0.07248	0.288496
ENSBTAP00000026769.6	ENSBTAG00000020093	0.977	0.27356	-0.03357	0.562947
ENSBTAP00000026786.3	ENSBTAG00000020109	NA	NA	NA	NA
ENSBTAP00000026802.2	ENSBTAG00000020123	NA	NA	NA	NA
ENSBTAP00000026826.4	ENSBTAG00000020139	0.829	0.35214	-0.27056	0.453285
ENSBTAP00000026834.4	ENSBTAG00000022242	1.073	0.20426	0.10165	0.689817
ENSBTAP00000026841.5	ENSBTAG00000020152	1.001	0.99008	0.001442	0.00433
ENSBTAP00000026869.5	ENSBTAG00000020174	1.037	0.34252	0.052416	0.465314
ENSBTAP00000026871.5	ENSBTAG00000020175	0.968	0.2898	-0.04692	0.537902
ENSBTAP00000026887.6	ENSBTAG00000027321	1.066	0.4627	0.092207	0.334701
ENSBTAP00000026992.6	ENSBTAG00000021132	1.016	0.64112	0.0229	0.193061
ENSBTAP00000027067.5	ENSBTAG00000020309	0.983	0.7993	-0.02474	0.09729
ENSBTAP00000027078.6	ENSBTAG00000020316	0.98	0.74784	-0.02915	0.126191
ENSBTAP00000027093.3	ENSBTAG00000020331	0.745	0.111943	-0.42469	0.951003
ENSBTAP00000027095.6	ENSBTAG00000020332	1.073	0.175477	0.10165	0.75578
ENSBTAP00000027181.4	ENSBTAG00000020393	1.022	0.43672	0.031395	0.359797

ENSBTAP00000027191.2	ENSBTAG00000020405	1.055	0.3913	0.077243	0.40749
ENSBTAP00000027202.4	ENSBTAG00000020410	NA	NA	NA	NA
ENSBTAP00000027246.5	ENSBTAG00000020446	NA	NA	NA	NA
ENSBTAP00000027250.6	ENSBTAG00000020447	1.086	0.58516	0.119024	0.232725
ENSBTAP00000027339.3	ENSBTAG00000020518	0.965	0.69234	-0.0514	0.159681
ENSBTAP00000027348.2	ENSBTAG00000020527	1.087	0.31804	0.120352	0.497518
ENSBTAP00000027352.6	ENSBTAG00000020530	0.939	0.137204	-0.0908	0.862633
ENSBTAP00000027409.6	ENSBTAG00000020569	0.918	0.2016	-0.12343	0.695509
ENSBTAP00000027507.3	ENSBTAG00000020641	0.998	0.9985	-0.00289	0.000652
ENSBTAP00000027511.4	ENSBTAG00000020645	1.25	0.123285	0.321928	0.90909
ENSBTAP00000027666.5	ENSBTAG00000020760	1.195	0.091601	0.257011	1.0381
ENSBTAP00000027713.3	ENSBTAG00000020795	1.019	0.7767	0.027154	0.109747
ENSBTAP00000027808.4	ENSBTAG00000020873	1.092	0.31272	0.126973	0.504844
ENSBTAP00000027927.4	ENSBTAG00000020968	1.106	0.55728	0.145351	0.253927
ENSBTAP00000027941.5	ENSBTAG00000020980	0.921	0.41278	-0.11873	0.384281
ENSBTAP00000027964.5	ENSBTAG00000020998	0.897	0.83788	-0.15682	0.076818
ENSBTAP00000028093.4	ENSBTAG00000021093	NA	NA	NA	NA
ENSBTAP00000028111.3	ENSBTAG00000021102	0.895	0.36604	-0.16004	0.436471
ENSBTAP00000028141.4	ENSBTAG00000021120	0.999	0.9631	-0.00144	0.016329
ENSBTAP00000028151.2	ENSBTAG00000021127	1.124	0.26928	0.168642	0.569796
ENSBTAP00000028159.6	ENSBTAG00000033008	1.016	0.83206	0.0229	0.079845
ENSBTAP00000028239.5	ENSBTAG00000021191	1.09	0.46782	0.124328	0.329921
ENSBTAP00000028263.4	ENSBTAG00000021211	0.992	0.96798	-0.01159	0.014134
ENSBTAP00000028280.4	ENSBTAG00000021226	0.92	0.140898	-0.12029	0.851095
ENSBTAP00000028291.3	ENSBTAG00000021232	1.148	0.083237	0.199123	1.079684
ENSBTAP00000028299.2	ENSBTAG00000021242	1.177	0.037199	0.235114	1.429469
ENSBTAP00000028330.5	ENSBTAG00000021262	NA	NA	NA	NA
ENSBTAP00000028333.4	ENSBTAG00000030836	1.409	0.20164	0.494672	0.695423
ENSBTAP00000028364.2	ENSBTAG00000021288	NA	NA	NA	NA
ENSBTAP00000028490.5	ENSBTAG00000021370	0.981	0.72152	-0.02767	0.141752
ENSBTAP00000028494.4	ENSBTAG00000021374	0.889	0.063485	-0.16974	1.197329
ENSBTAP00000028498.3	ENSBTAG00000021377	NA	NA	NA	NA
ENSBTAP00000028520.3	ENSBTAG00000021395	1.009	0.9591	0.012926	0.018136
ENSBTAP00000028551.5	ENSBTAG00000021416	0.963	0.7307	-0.05439	0.136261
ENSBTAP00000028602.5	ENSBTAG00000021455	0.983	0.60556	-0.02474	0.217843
ENSBTAP00000028608.4	ENSBTAG00000021461	1.014	0.6389	0.020058	0.194567
ENSBTAP00000028634.3	ENSBTAG00000021481	1.045	0.89062	0.063503	0.050308
ENSBTAP00000028640.4	ENSBTAG00000004288	1.225	0.54328	0.292782	0.264976
ENSBTAP00000028645.4	ENSBTAG00000021491	1.013	0.93918	0.018634	0.027251
ENSBTAP00000028661.1	ENSBTAG00000021506	1.146	0.54264	0.196607	0.265488
ENSBTAP00000028662.4	ENSBTAG00000021508	0.911	0.4305	-0.13448	0.366027
ENSBTAP00000028740.5	ENSBTAG00000021569	1.004	0.77922	0.005759	0.10834
ENSBTAP00000028757.6	ENSBTAG00000021580	NA	NA	NA	NA
ENSBTAP00000028809.4	ENSBTAG00000021620	1.023	0.4793	0.032806	0.319393
ENSBTAP00000028825.5	ENSBTAG00000021632	1.035	0.48522	0.049631	0.314061
ENSBTAP00000028853.4	ENSBTAG00000021651	1.075	0.4631	0.104337	0.334325
ENSBTAP00000028899.3	ENSBTAG00000021685	1.018	0.76132	0.025738	0.118433
ENSBTAP00000028902.4	ENSBTAG00000021688	1.092	0.27576	0.126973	0.559469
ENSBTAP00000028950.5	ENSBTAG00000021715	1.011	0.58582	0.015783	0.232236
ENSBTAP00000028958.4	ENSBTAG00000021724	1.071	0.3118	0.098958	0.506124
ENSBTAP00000028983.6	ENSBTAG00000021744	0.975	0.52044	-0.03653	0.283629
ENSBTAP00000028994.4	ENSBTAG00000021752	0.957	0.63506	-0.06341	0.197185
ENSBTAP00000029028.3	ENSBTAG00000021779	1.168	0.34362	0.22404	0.463922
ENSBTAP00000029044.5	ENSBTAG00000021790	1.069	0.39912	0.096262	0.398897
ENSBTAP00000029075.5	ENSBTAG00000021813	0.972	0.72136	-0.04097	0.141848
ENSBTAP00000029129.5	ENSBTAG00000021849	1.011	0.75756	0.015783	0.120583
ENSBTAP00000029203.5	ENSBTAG00000021902	1.047	0.8468	0.066261	0.072219
ENSBTAP00000029256.2	ENSBTAG00000021944	0.971	0.30436	-0.04246	0.516612

ENSBTAP00000029257.6	ENSBTAG00000021945	1.247	0.057118	0.318461	1.243227
ENSBTAP00000029285.6	ENSBTAG00000025450	1.009	0.91754	0.012926	0.037375
ENSBTAP00000029301.3	ENSBTAG00000021976	1.049	0.6641	0.069015	0.177767
ENSBTAP00000029307.6	ENSBTAG00000021978	NA	NA	NA	NA
ENSBTAP00000029363.3	ENSBTAG00000022013	1.143	0.32452	0.192825	0.488759
ENSBTAP00000029458.2	ENSBTAG00000022027	1.239	0.199217	0.309176	0.700674
ENSBTAP00000029468.4	ENSBTAG00000022032	0.979	0.99272	-0.03062	0.003173
ENSBTAP00000029514.5	ENSBTAG00000022044	1.018	0.79404	0.025738	0.100158
ENSBTAP00000029667.3	ENSBTAG00000039630	1.017	0.8057	0.02432	0.093827
ENSBTAP00000029809.4	ENSBTAG00000022114	NA	NA	NA	NA
ENSBTAP00000029840.3	ENSBTAG00000007234	1.13	0.141117	0.176323	0.850421
ENSBTAP00000029885.5	ENSBTAG00000022158	0.664	0.2723	-0.59074	0.564952
ENSBTAP00000029915.2	ENSBTAG00000002820	0.971	0.34602	-0.04246	0.460899
ENSBTAP00000030018.4	ENSBTAG00000022244	0.918	0.34496	-0.12343	0.462231
ENSBTAP00000030114.1	ENSBTAG00000022292	1.042	0.66434	0.059355	0.17761
ENSBTAP00000030177.1	ENSBTAG00000022314	1.044	0.28482	0.062122	0.54543
ENSBTAP00000030179.5	ENSBTAG00000011190	1.074	0.35938	0.102994	0.444446
ENSBTAP00000030212.3	ENSBTAG00000001059	1.059	0.69074	0.082703	0.160685
ENSBTAP00000030260.3	ENSBTAG00000016282	1.024	0.75566	0.034216	0.121674
ENSBTAP00000030304.1	ENSBTAG00000011869	1.334	0.148256	0.415759	0.828988
ENSBTAP00000030466.2	ENSBTAG00000015136	1.023	0.71798	0.032806	0.143888
ENSBTAP00000030786.2	ENSBTAG00000010832	NA	NA	NA	NA
ENSBTAP00000030791.4	ENSBTAG00000022714	0.928	0.43786	-0.1078	0.358665
ENSBTAP00000031126.5	ENSBTAG00000002069	NA	NA	NA	NA
ENSBTAP00000031700.2	ENSBTAG00000023343	0.915	0.58222	-0.12816	0.234913
ENSBTAP00000031784.5	ENSBTAG00000023384	NA	NA	NA	NA
ENSBTAP00000031831.2	ENSBTAG00000039522	0.949	0.67942	-0.07552	0.167862
ENSBTAP00000031937.3	ENSBTAG00000016313	1.058	0.54004	0.08134	0.267574
ENSBTAP00000031949.4	ENSBTAG00000023472	0.954	0.8042	-0.06794	0.094636
ENSBTAP00000031993.4	ENSBTAG00000001055	1.033	0.40814	0.04684	0.389191
ENSBTAP00000032188.4	ENSBTAG00000001781	NA	NA	NA	NA
ENSBTAP00000032251.2	ENSBTAG00000020632	1.168	0.125121	0.22404	0.90267
ENSBTAP00000032258.3	ENSBTAG00000023628	1.161	0.26314	0.215368	0.579813
ENSBTAP00000032364.5	ENSBTAG00000000201	0.879	0.44142	-0.18606	0.355148
ENSBTAP00000032384.3	ENSBTAG00000018854	0.918	0.0457	-0.12343	1.340084
ENSBTAP00000032594.3	ENSBTAG00000023806	0.912	0.160565	-0.13289	0.794349
ENSBTAP00000032777.2	ENSBTAG00000001140	1.061	0.3592	0.085425	0.444664
ENSBTAP00000032779.2	ENSBTAG00000002108	NA	NA	NA	NA
ENSBTAP00000032864.2	ENSBTAG00000012697	1.083	0.11858	0.115033	0.925989
ENSBTAP00000033015.3	ENSBTAG00000015473	1.265	0.176518	0.339137	0.753211
ENSBTAP00000033077.5	ENSBTAG00000013290	0.973	0.40638	-0.03949	0.391068
ENSBTAP00000033383.4	ENSBTAG00000024240	1.207	0.174575	0.271426	0.758018
ENSBTAP00000033610.2	ENSBTAG00000016709	1.049	0.6138	0.069015	0.211973
ENSBTAP00000033696.4	ENSBTAG00000001908	1.041	0.58372	0.05797	0.233795
ENSBTAP00000033736.3	ENSBTAG00000039851	1.029	0.63504	0.041243	0.197199
ENSBTAP00000033771.4	ENSBTAG00000013472	1.067	0.74974	0.09356	0.125089
ENSBTAP00000033800.4	ENSBTAG00000005166	NA	NA	NA	NA
ENSBTAP00000033805.4	ENSBTAG00000002280	1.026	0.72038	0.037031	0.142438
ENSBTAP00000033825.2	ENSBTAG00000014827	0.994	0.91084	-0.00868	0.040558
ENSBTAP00000033996.2	ENSBTAG00000017852	1.37	0.39244	0.454176	0.406227
ENSBTAP00000034331.3	ENSBTAG00000024723	1.117	0.21126	0.159629	0.675183
ENSBTAP00000034926.1	ENSBTAG00000011847	0.963	0.8589	-0.05439	0.066057
ENSBTAP00000035055.5	ENSBTAG00000025136	1.098	0.51956	0.134878	0.284364
ENSBTAP00000035133.2	ENSBTAG00000011415	0.978	0.70914	-0.03209	0.149268
ENSBTAP00000035177.2	ENSBTAG00000020928	1.01	0.99316	0.014355	0.002981
ENSBTAP00000035184.4	ENSBTAG00000025191	1.024	0.63964	0.034216	0.194064
ENSBTAP00000035243.3	ENSBTAG00000020389	0.968	0.06216	-0.04692	1.206489
ENSBTAP00000035421.5	ENSBTAG00000025337	0.931	0.1484	-0.10315	0.828566

ENSBTAP00000035425.1	ENSBTAG00000020477	1.012	0.80896	0.017209	0.092073
ENSBTAP00000035516.3	ENSBTAG00000000341	NA	NA	NA	NA
ENSBTAP00000035570.4	ENSBTAG00000015796	NA	NA	NA	NA
ENSBTAP00000035635.4	ENSBTAG00000025442	NA	NA	NA	NA
ENSBTAP00000035657.4	ENSBTAG00000019685	1.183	0.067896	0.24245	1.168156
ENSBTAP00000035716.4	ENSBTAG00000025496	0.857	0.169657	-0.22263	0.770428
ENSBTAP00000035767.5	ENSBTAG00000025526	1.057	0.5804	0.079975	0.236273
ENSBTAP00000035907.2	ENSBTAG00000005974	1.031	0.80924	0.044044	0.091923
ENSBTAP00000036036.5	ENSBTAG00000011699	1.034	0.59952	0.048236	0.222196
ENSBTAP00000036460.4	ENSBTAG00000012866	0.915	0.67754	-0.12816	0.169065
ENSBTAP00000036487.5	ENSBTAG00000025868	1.027	0.57956	0.038436	0.236902
ENSBTAP00000036650.4	ENSBTAG00000012442	1.159	0.038481	0.212881	1.414754
ENSBTAP00000036700.4	ENSBTAG00000007068	0.985	0.6578	-0.0218	0.181906
ENSBTAP00000036739.5	ENSBTAG00000026199	1.089	0.026156	0.123004	1.582429
ENSBTAP00000037041.2	ENSBTAG00000032432	0.951	0.47786	-0.07248	0.320699
ENSBTAP00000037071.4	ENSBTAG00000026250	1.027	0.78284	0.038436	0.106327
ENSBTAP00000037091.3	ENSBTAG00000026266	1.079	0.33676	0.109695	0.472679
ENSBTAP00000037200.3	ENSBTAG00000026327	0.924	0.61166	-0.11404	0.21349
ENSBTAP00000037239.5	ENSBTAG00000001503	0.957	0.57952	-0.06341	0.236932
ENSBTAP00000037526.3	ENSBTAG00000014238	1.015	0.78504	0.02148	0.105108
ENSBTAP00000037577.4	ENSBTAG00000014731	0.919	0.188603	-0.12186	0.724451
ENSBTAP00000037804.2	ENSBTAG00000001988	1.039	0.27314	0.055196	0.563615
ENSBTAP00000038059.2	ENSBTAG00000031500	1.016	0.7675	0.0229	0.114922
ENSBTAP00000038183.3	ENSBTAG00000030973	0.974	0.77924	-0.03801	0.108329
ENSBTAP00000038273.1	ENSBTAG00000026886	0.759	0.30948	-0.39783	0.509367
ENSBTAP00000038290.4	ENSBTAG00000015396	0.95	0.083879	-0.074	1.076347
ENSBTAP00000038686.5	ENSBTAG00000014069	2.166	1.59E-05	1.115033	4.798985
ENSBTAP00000039041.5	ENSBTAG00000003512	NA	NA	NA	NA
ENSBTAP00000039119.4	ENSBTAG00000027397	0.961	0.75642	-0.05739	0.121237
ENSBTAP00000039209.3	ENSBTAG00000027446	0.978	0.68512	-0.03209	0.164233
ENSBTAP00000039548.4	ENSBTAG00000027612	NA	NA	NA	NA
ENSBTAP00000039580.5	ENSBTAG00000027630	NA	NA	NA	NA
ENSBTAP00000039862.5	ENSBTAG00000016648	1.068	0.192065	0.094912	0.716552
ENSBTAP00000039979.4	ENSBTAG00000027879	1.245	0.130399	0.316146	0.884726
ENSBTAP00000040182.3	ENSBTAG00000030164	NA	NA	NA	NA
ENSBTAP00000040198.2	ENSBTAG00000030172	1.005	0.91696	0.007196	0.03765
ENSBTAP00000040249.3	ENSBTAG00000019105	0.992	0.69464	-0.01159	0.15824
ENSBTAP00000040280.4	ENSBTAG00000030209	1.029	0.191382	0.041243	0.718099
ENSBTAP00000040330.4	ENSBTAG00000006085	1.045	0.35944	0.063503	0.444374
ENSBTAP00000040410.1	ENSBTAG00000030302	0.904	0.42628	-0.14561	0.370305
ENSBTAP00000040442.4	ENSBTAG00000030333	1.126	0.43862	0.171207	0.357912
ENSBTAP00000040631.2	ENSBTAG00000008135	NA	NA	NA	NA
ENSBTAP00000040682.3	ENSBTAG00000013337	0.948	0.29708	-0.07704	0.527127
ENSBTAP00000040732.2	ENSBTAG00000005296	0.916	0.50994	-0.12658	0.292481
ENSBTAP00000040759.3	ENSBTAG00000008881	0.955	0.41816	-0.06643	0.378658
ENSBTAP00000040778.3	ENSBTAG00000002036	0.933	0.138078	-0.10005	0.859876
ENSBTAP00000040793.2	ENSBTAG00000030587	1.074	0.46316	0.102994	0.334269
ENSBTAP00000040860.2	ENSBTAG00000009479	1.018	0.82928	0.025738	0.081299
ENSBTAP00000040861.3	ENSBTAG00000003956	0.993	0.88292	-0.01013	0.054079
ENSBTAP00000040892.2	ENSBTAG00000030648	1.166	0.039824	0.221568	1.399855
ENSBTAP00000040941.2	ENSBTAG00000030674	1.268	0.46726	0.342555	0.330441
ENSBTAP00000040954.1	ENSBTAG00000030683	NA	NA	NA	NA
ENSBTAP00000040986.3	ENSBTAG00000019269	0.924	0.4691	-0.11404	0.328735
ENSBTAP00000041066.4	ENSBTAG00000047186	0.92	0.45648	-0.12029	0.340578
ENSBTAP00000041096.3	ENSBTAG00000030801	1.152	0.30674	0.204141	0.51323
ENSBTAP00000041206.2	ENSBTAG00000011392	1.005	0.93392	0.007196	0.02969
ENSBTAP00000041279.1	ENSBTAG00000013253	1.067	0.40228	0.09356	0.395472
ENSBTAP00000041378.4	ENSBTAG00000027524	1.09	0.60332	0.124328	0.219452

ENSBTAP00000041398.2	ENSBTAG00000030979	NA	NA	NA	NA
ENSBTAP00000041551.4	ENSBTAG00000011953	0.995	0.83914	-0.00723	0.076166
ENSBTAP00000041575.4	ENSBTAG00000031134	1.083	0.23946	0.115033	0.620767
ENSBTAP00000041599.4	ENSBTAG00000015273	1.003	0.81584	0.004322	0.088395
ENSBTAP00000041643.4	ENSBTAG00000031171	0.738	0.165741	-0.43831	0.78057
ENSBTAP00000041673.3	ENSBTAG00000009543	0.966	0.44382	-0.0499	0.352793
ENSBTAP00000041719.1	ENSBTAG00000031217	0.873	0.79152	-0.19595	0.101538
ENSBTAP00000041797.4	ENSBTAG00000006712	0.941	0.171742	-0.08773	0.765123
ENSBTAP00000041803.3	ENSBTAG00000017616	0.892	0.062476	-0.16488	1.204287
ENSBTAP00000041860.3	ENSBTAG00000002953	1.021	0.62028	0.029983	0.207412
ENSBTAP00000041898.3	ENSBTAG00000011969	1.043	0.78366	0.060739	0.105872
ENSBTAP00000041967.4	ENSBTAG00000003908	1.021	0.72414	0.029983	0.140177
ENSBTAP00000042008.3	ENSBTAG00000002783	1.113	0.30444	0.154454	0.516498
ENSBTAP00000042130.3	ENSBTAG00000016598	1.025	0.60292	0.035624	0.21974
ENSBTAP00000042206.3	ENSBTAG00000016720	0.992	0.95698	-0.01159	0.019097
ENSBTAP00000042249.3	ENSBTAG00000031598	1.16	0.30068	0.214125	0.521895
ENSBTAP00000042255.4	ENSBTAG00000005373	0.935	0.41158	-0.09696	0.385546
ENSBTAP00000042274.3	ENSBTAG00000004531	1.134	0.2835	0.181421	0.547447
ENSBTAP00000042386.3	ENSBTAG00000017710	1.064	0.5289	0.089498	0.276626
ENSBTAP00000042411.2	ENSBTAG00000031723	NA	NA	NA	NA
ENSBTAP00000042488.3	ENSBTAG00000017765	0.783	0.29658	-0.35292	0.527858
ENSBTAP00000042493.1	ENSBTAG00000031793	NA	NA	NA	NA
ENSBTAP00000042500.2	ENSBTAG00000031797	1.033	0.64582	0.04684	0.189889
ENSBTAP00000042551.2	ENSBTAG00000014295	0.95	0.6058	-0.074	0.217671
ENSBTAP00000042771.4	ENSBTAG00000011588	1.01	0.83728	0.014355	0.077129
ENSBTAP00000042789.2	ENSBTAG00000010196	NA	NA	NA	NA
ENSBTAP00000042833.3	ENSBTAG00000018813	1.057	0.63484	0.079975	0.197336
ENSBTAP00000042926.1	ENSBTAG00000008837	1.108	0.061244	0.147958	1.212936
ENSBTAP00000043175.1	ENSBTAG00000032304	1.112	0.075196	0.153157	1.123805
ENSBTAP00000043333.2	ENSBTAG00000016211	1.239	0.103602	0.309176	0.984632
ENSBTAP00000043481.1	ENSBTAG00000032531	1.323	0.079523	0.403813	1.099507
ENSBTAP00000043544.4	ENSBTAG00000023891	0.93	0.67344	-0.1047	0.171701
ENSBTAP00000043640.1	ENSBTAG00000018071	0.94	0.20332	-0.08927	0.69182
ENSBTAP00000043669.4	ENSBTAG00000002608	1.084	0.35326	0.116365	0.451906
ENSBTAP00000043726.4	ENSBTAG00000032719	1.393	0.092303	0.478195	1.034784
ENSBTAP00000043732.2	ENSBTAG00000011480	0.933	0.68296	-0.10005	0.165605
ENSBTAP00000043804.2	ENSBTAG00000018499	NA	NA	NA	NA
ENSBTAP00000043833.1	ENSBTAG00000032774	0.972	0.78468	-0.04097	0.105307
ENSBTAP00000043944.4	ENSBTAG00000032872	NA	NA	NA	NA
ENSBTAP00000044053.2	ENSBTAG00000032961	0.946	0.54644	-0.08009	0.262458
ENSBTAP00000044075.1	ENSBTAG00000005078	0.907	0.75402	-0.14083	0.122617
ENSBTAP00000044088.3	ENSBTAG00000015943	1.092	0.45598	0.126973	0.341054
ENSBTAP00000044120.3	ENSBTAG00000033015	1.15	0.7219	0.201634	0.141523
ENSBTAP00000044243.4	ENSBTAG00000014068	0.99	0.85882	-0.0145	0.066098
ENSBTAP00000044292.3	ENSBTAG00000018937	NA	NA	NA	NA
ENSBTAP00000044376.3	ENSBTAG00000033190	1.032	0.52332	0.045443	0.281233
ENSBTAP00000044462.3	ENSBTAG00000005250	1.026	0.9055	0.037031	0.043112
ENSBTAP00000044536.4	ENSBTAG00000012044	0.861	0.53298	-0.21591	0.273289
ENSBTAP00000044619.3	ENSBTAG00000001601	0.904	0.33774	-0.14561	0.471418
ENSBTAP00000044670.3	ENSBTAG00000003728	1.032	0.85144	0.045443	0.069846
ENSBTAP00000044733.3	ENSBTAG00000012778	1.068	0.31568	0.094912	0.500753
ENSBTAP00000044734.4	ENSBTAG00000033422	0.962	0.82528	-0.05589	0.083399
ENSBTAP00000044772.1	ENSBTAG00000033446	0.913	0.35076	-0.13131	0.45499
ENSBTAP00000044932.3	ENSBTAG00000020693	NA	NA	NA	NA
ENSBTAP00000045010.3	ENSBTAG00000033680	0.973	0.48786	-0.03949	0.311705
ENSBTAP00000045066.3	ENSBTAG00000033766	1.177	0.093697	0.235114	1.028274
ENSBTAP00000045141.2	ENSBTAG00000033835	0.653	0.30542	-0.61485	0.515103
ENSBTAP00000045293.3	ENSBTAG00000033983	1.047	0.35498	0.066261	0.449796

ENSBTAP00000045408.3	ENSBTAG00000014262	1.036	0.37176	0.051024	0.429737
ENSBTAP00000045674.2	ENSBTAG00000034360	1.117	0.62824	0.159629	0.201874
ENSBTAP00000045691.3	ENSBTAG00000013004	NA	NA	NA	NA
ENSBTAP00000045734.3	ENSBTAG00000019854	0.94	0.29208	-0.08927	0.534498
ENSBTAP00000045753.1	ENSBTAG00000012252	NA	NA	NA	NA
ENSBTAP00000046086.3	ENSBTAG00000025320	NA	NA	NA	NA
ENSBTAP00000046095.2	ENSBTAG00000010551	1.098	0.069938	0.134878	1.155287
ENSBTAP00000046146.4	ENSBTAG00000004861	0.954	0.103344	-0.06794	0.985715
ENSBTAP00000046157.3	ENSBTAG00000034776	NA	NA	NA	NA
ENSBTAP00000046293.2	ENSBTAG00000007399	1.06	0.4125	0.084064	0.384576
ENSBTAP00000046356.1	ENSBTAG00000034992	0.995	0.87464	-0.00723	0.058171
ENSBTAP00000046374.1	ENSBTAG00000014766	0.87	0.45108	-0.20091	0.345746
ENSBTAP00000046432.1	ENSBTAG00000006745	0.895	0.65276	-0.16004	0.185246
ENSBTAP00000046832.2	ENSBTAG00000000223	1.036	0.40408	0.051024	0.393533
ENSBTAP00000046882.1	ENSBTAG00000020218	0.963	0.48372	-0.05439	0.315406
ENSBTAP00000047095.3	ENSBTAG00000006242	1.115	0.3705	0.157044	0.431212
ENSBTAP00000047181.1	ENSBTAG00000035998	NA	NA	NA	NA
ENSBTAP00000047262.4	ENSBTAG00000036101	1.027	0.82486	0.038436	0.08362
ENSBTAP00000047452.3	ENSBTAG00000019265	NA	NA	NA	NA
ENSBTAP00000047554.1	ENSBTAG00000016347	NA	NA	NA	NA
ENSBTAP00000047563.3	ENSBTAG00000011758	NA	NA	NA	NA
ENSBTAP00000047729.2	ENSBTAG00000008683	0.838	0.13818	-0.25498	0.859555
ENSBTAP00000047792.3	ENSBTAG00000017248	0.936	0.051701	-0.09542	1.286501
ENSBTAP00000047848.1	ENSBTAG00000016005	0.894	0.122701	-0.16165	0.911152
ENSBTAP00000047882.3	ENSBTAG00000003358	1.101	0.40032	0.138814	0.397593
ENSBTAP00000047901.1	ENSBTAG00000004284	0.864	0.1726	-0.2109	0.762959
ENSBTAP00000047927.2	ENSBTAG00000005957	0.938	0.42676	-0.09234	0.369816
ENSBTAP00000048125.2	ENSBTAG00000039121	0.98	0.72708	-0.02915	0.138418
ENSBTAP00000048183.2	ENSBTAG00000017389	1.025	0.47036	0.035624	0.32757
ENSBTAP00000048297.2	ENSBTAG00000009646	1.063	0.3847	0.088142	0.414878
ENSBTAP00000048368.3	ENSBTAG00000007606	1.034	0.48764	0.048236	0.311901
ENSBTAP00000048415.3	ENSBTAG00000038849	0.951	0.33458	-0.07248	0.4755
ENSBTAP00000048641.2	ENSBTAG00000015786	1.022	0.78548	0.031395	0.104865
ENSBTAP00000048703.2	ENSBTAG00000026586	0.801	0.03006	-0.32013	1.522011
ENSBTAP00000048807.2	ENSBTAG00000012890	1.093	0.37838	0.128293	0.422072
ENSBTAP00000048918.3	ENSBTAG00000039943	1.124	0.196124	0.168642	0.707469
ENSBTAP00000048950.2	ENSBTAG00000009470	1.137	0.27888	0.185232	0.554583
ENSBTAP00000049065.3	ENSBTAG00000020940	0.978	0.6256	-0.03209	0.203703
ENSBTAP00000049070.3	ENSBTAG00000011625	1.008	0.87162	0.011496	0.059673
ENSBTAP00000049115.1	ENSBTAG00000001635	1.072	0.21764	0.100305	0.662261
ENSBTAP00000049139.3	ENSBTAG00000023462	0.795	0.40498	-0.33097	0.392566
ENSBTAP00000049205.3	ENSBTAG00000010954	1.245	0.103659	0.316146	0.984393
ENSBTAP00000049248.3	ENSBTAG00000027444	1.023	0.66704	0.032806	0.175848
ENSBTAP00000049251.1	ENSBTAG00000013952	0.969	0.415	-0.04543	0.381952
ENSBTAP00000049262.3	ENSBTAG00000013113	0.987	0.8333	-0.01888	0.079199
ENSBTAP00000049383.2	ENSBTAG00000008985	0.987	0.47496	-0.01888	0.323343
ENSBTAP00000049550.2	ENSBTAG00000040006	1.222	0.074741	0.289244	1.126441
ENSBTAP00000049613.2	ENSBTAG00000016079	1.176	0.114303	0.233888	0.941942
ENSBTAP00000049614.2	ENSBTAG00000020308	1.006	0.94314	0.00863	0.025424
ENSBTAP00000049636.2	ENSBTAG00000009707	0.995	0.87562	-0.00723	0.057684
ENSBTAP00000049659.2	ENSBTAG00000040333	1.101	0.122261	0.138814	0.912712
ENSBTAP00000049804.2	ENSBTAG00000008001	NA	NA	NA	NA
ENSBTAP00000049826.2	ENSBTAG00000047787	NA	NA	NA	NA
ENSBTAP00000049910.2	ENSBTAG00000040116	NA	NA	NA	NA
ENSBTAP00000050158.3	ENSBTAG00000038051	1.131	0.2546	0.177599	0.594142
ENSBTAP00000050170.2	ENSBTAG00000039161	NA	NA	NA	NA
ENSBTAP00000050222.2	ENSBTAG00000022902	0.838	0.36776	-0.25498	0.434436
ENSBTAP00000050302.2	ENSBTAG00000022009	NA	NA	NA	NA

ENSBTAP00000050547.1	ENSBTAG00000016775	1.444	0.062725	0.530071	1.202559
ENSBTAP00000050842.2	ENSBTAG00000039321	NA	NA	NA	NA
ENSBTAP00000050939.3	ENSBTAG00000012341	0.938	0.72922	-0.09234	0.137141
ENSBTAP00000050973.1	ENSBTAG00000006802	1.196	0.118917	0.258217	0.924756
ENSBTAP00000051010.3	ENSBTAG00000030705	NA	NA	NA	NA
ENSBTAP00000051012.2	ENSBTAG00000038379	0.898	0.35776	-0.15521	0.446408
ENSBTAP00000051069.2	ENSBTAG00000027075	NA	NA	NA	NA
ENSBTAP00000051115.2	ENSBTAG00000006756	1.054	0.31016	0.075875	0.508414
ENSBTAP00000051130.3	ENSBTAG00000022635	1.109	0.20478	0.149259	0.688712
ENSBTAP00000051138.3	ENSBTAG00000033008	1.007	0.93302	0.010064	0.030109
ENSBTAP00000051168.3	ENSBTAG00000039035	1.052	0.21114	0.073135	0.675429
ENSBTAP00000051340.3	ENSBTAG00000030520	1.048	0.41014	0.067639	0.387068
ENSBTAP00000051358.1	ENSBTAG00000020796	NA	NA	NA	NA
ENSBTAP00000051545.2	ENSBTAG00000010658	0.965	0.7092	-0.0514	0.149231
ENSBTAP00000051735.1	ENSBTAG00000019915	NA	NA	NA	NA
ENSBTAP00000051744.2	ENSBTAG00000019419	1.053	0.61642	0.074505	0.210123
ENSBTAP00000051774.1	ENSBTAG00000038186	0.979	0.60664	-0.03062	0.217069
ENSBTAP00000051891.1	ENSBTAG00000039582	1.071	0.74482	0.098958	0.127949
ENSBTAP00000051956.1	ENSBTAG00000040028	1.062	0.087198	0.086784	1.059493
ENSBTAP00000052045.3	ENSBTAG00000002082	1.062	0.48192	0.086784	0.317025
ENSBTAP00000052105.2	ENSBTAG00000040308	0.936	0.169535	-0.09542	0.770741
ENSBTAP00000052156.2	ENSBTAG00000040055	1.026	0.50192	0.037031	0.299365
ENSBTAP00000052192.2	ENSBTAG00000003546	1.069	0.051241	0.096262	1.290382
ENSBTAP00000052226.1	ENSBTAG00000025617	0.927	0.156683	-0.10936	0.804978
ENSBTAP00000052296.2	ENSBTAG00000021767	0.954	0.32738	-0.06794	0.484948
ENSBTAP00000052356.3	ENSBTAG00000013127	0.9	0.25206	-0.152	0.598496
ENSBTAP00000052422.3	ENSBTAG00000007662	1.016	0.7187	0.0229	0.143452
ENSBTAP00000052479.3	ENSBTAG00000054812	1.035	0.63906	0.049631	0.194458
ENSBTAP00000052639.2	ENSBTAG00000014601	1.01	0.83616	0.014355	0.077711
ENSBTAP00000052689.3	ENSBTAG00000002882	NA	NA	NA	NA
ENSBTAP00000052906.2	ENSBTAG00000004871	1.056	0.62102	0.07861	0.206894
ENSBTAP00000052922.2	ENSBTAG00000006563	1.048	0.60848	0.067639	0.215754
ENSBTAP00000053143.1	ENSBTAG00000043564	1.142	0.83826	0.191563	0.076621
ENSBTAP00000053146.3	ENSBTAG00000043553	0.902	0.58008	-0.1488	0.236512
ENSBTAP00000053151.1	ENSBTAG00000043556	1.138	0.166697	0.186501	0.778072
ENSBTAP00000053152.1	ENSBTAG00000043563	1.05	0.69492	0.070389	0.158065
ENSBTAP00000053158.3	ENSBTAG00000043581	NA	NA	NA	NA
ENSBTAP00000053160.1	ENSBTAG00000043571	1.075	0.85778	0.104337	0.066624
ENSBTAP00000053169.3	ENSBTAG00000000793	1.004	0.9765	0.005759	0.010328
ENSBTAP00000053179.3	ENSBTAG00000010229	1.019	0.515	0.027154	0.288193
ENSBTAP00000053182.3	ENSBTAG00000002528	1.001	0.95214	0.001442	0.021299
ENSBTAP00000053200.2	ENSBTAG00000019625	0.938	0.59554	-0.09234	0.225089
ENSBTAP00000053217.3	ENSBTAG00000009703	1.224	0.20956	0.291604	0.678692
ENSBTAP00000053221.2	ENSBTAG00000005946	0.942	0.2522	-0.0862	0.598255
ENSBTAP00000053274.3	ENSBTAG00000012307	1.018	0.52324	0.025738	0.281299
ENSBTAP00000053303.3	ENSBTAG00000020296	0.986	0.84248	-0.02034	0.07444
ENSBTAP00000053339.3	ENSBTAG00000013658	0.888	0.054141	-0.17137	1.266474
ENSBTAP00000053356.3	ENSBTAG00000044167	1.098	0.48658	0.134878	0.312846
ENSBTAP00000053370.2	ENSBTAG00000044046	1.009	0.75636	0.012926	0.121271
ENSBTAP00000053400.3	ENSBTAG00000044040	1.074	0.054223	0.102994	1.265816
ENSBTAP00000053408.3	ENSBTAG00000037794	1.118	0.186364	0.16092	0.729638
ENSBTAP00000053447.3	ENSBTAG00000012582	1.105	0.22256	0.144046	0.652553
ENSBTAP00000053490.3	ENSBTAG00000044141	1.001	0.98652	0.001442	0.005894
ENSBTAP00000053491.3	ENSBTAG00000019585	1.005	0.82608	0.007196	0.082978
ENSBTAP00000053520.3	ENSBTAG00000006907	NA	NA	NA	NA
ENSBTAP00000053550.3	ENSBTAG00000010243	NA	NA	NA	NA
ENSBTAP00000053553.3	ENSBTAG00000039684	1.051	0.5045	0.071763	0.297139
ENSBTAP00000053607.3	ENSBTAG00000005108	1.259	0.45176	0.332278	0.345092

ENSBTAP00000053628.3	ENSBTAG00000012882	1	0.99776	0	0.000974
ENSBTAP00000053652.3	ENSBTAG00000016167	0.967	0.51144	-0.04841	0.291205
ENSBTAP00000053653.3	ENSBTAG00000009702	0.908	0.60066	-0.13924	0.221371
ENSBTAP00000053899.2	ENSBTAG00000035907	0.991	0.69572	-0.01304	0.157566
ENSBTAP00000053967.1	ENSBTAG00000002688	1.073	0.120883	0.10165	0.917635
ENSBTAP00000053982.2	ENSBTAG00000034170	1.033	0.72118	0.04684	0.141956
ENSBTAP00000053997.2	ENSBTAG00000046498	NA	NA	NA	NA
ENSBTAP00000054012.2	ENSBTAG00000047206	0.754	0.41194	-0.40736	0.385166
ENSBTAP00000054121.2	ENSBTAG00000003100	0.946	0.54334	-0.08009	0.264928
ENSBTAP00000054169.2	ENSBTAG00000017839	1.006	0.78056	0.00863	0.107594
ENSBTAP00000054196.2	ENSBTAG00000046786	1.066	0.32516	0.092207	0.487903
ENSBTAP00000054206.1	ENSBTAG00000047845	1.049	0.68276	0.069015	0.165732
ENSBTAP00000054343.1	ENSBTAG00000046333	1.01	0.89998	0.014355	0.045767
ENSBTAP00000054446.1	ENSBTAG00000046512	1.204	0.079504	0.267835	1.099611
ENSBTAP00000054484.2	ENSBTAG00000024929	0.946	0.6665	-0.08009	0.1762
ENSBTAP00000054495.2	ENSBTAG00000046019	1.053	0.69426	0.074505	0.158478
ENSBTAP00000054509.2	ENSBTAG00000045877	NA	NA	NA	NA
ENSBTAP00000054517.1	ENSBTAG00000048107	1.024	0.7164	0.034216	0.144844
ENSBTAP00000054589.2	ENSBTAG00000055225	1.028	0.93	0.03984	0.031517
ENSBTAP00000054670.2	ENSBTAG00000046485	1.011	0.809	0.015783	0.092051
ENSBTAP00000054671.2	ENSBTAG00000047231	1.252	0.139637	0.324235	0.854999
ENSBTAP00000054675.1	ENSBTAG00000000605	1.095	0.39268	0.130931	0.405961
ENSBTAP00000054688.2	ENSBTAG00000032427	0.955	0.42916	-0.06643	0.367381
ENSBTAP00000054828.2	ENSBTAG00000006419	1.24	0.2616	0.31034	0.582362
ENSBTAP00000054852.2	ENSBTAG00000045604	1.051	0.95052	0.071763	0.022039
ENSBTAP00000054891.2	ENSBTAG00000018513	1.031	0.72828	0.044044	0.137702
ENSBTAP00000054939.2	ENSBTAG00000047856	1.069	0.049776	0.096262	1.30298
ENSBTAP00000054964.1	ENSBTAG00000034433	1.039	0.35622	0.055196	0.448282
ENSBTAP00000054985.2	ENSBTAG00000047998	0.976	0.80034	-0.03505	0.096725
ENSBTAP00000054994.2	ENSBTAG00000017512	0.891	0.22058	-0.1665	0.656434
ENSBTAP00000054999.2	ENSBTAG00000045717	0.924	0.30238	-0.11404	0.519447
ENSBTAP00000055029.2	ENSBTAG00000014448	1.006	0.91876	0.00863	0.036798
ENSBTAP00000055099.2	ENSBTAG00000046177	1.048	0.2063	0.067639	0.685501
ENSBTAP00000055147.2	ENSBTAG00000045957	1.142	0.152822	0.191563	0.815814
ENSBTAP00000055150.2	ENSBTAG00000010204	0.994	0.89692	-0.00868	0.047246
ENSBTAP00000055155.1	ENSBTAG00000045954	NA	NA	NA	NA
ENSBTAP00000055171.2	ENSBTAG00000045548	0.999	0.95368	-0.00144	0.020597
ENSBTAP00000055177.2	ENSBTAG00000046176	0.983	0.78342	-0.02474	0.106005
ENSBTAP00000055254.2	ENSBTAG00000047694	1.02	0.8793	0.028569	0.055863
ENSBTAP00000055316.2	ENSBTAG00000046838	1.027	0.2415	0.038436	0.617083
ENSBTAP00000055370.1	ENSBTAG00000047418	1.15	0.28564	0.201634	0.544181
ENSBTAP00000055431.1	ENSBTAG00000012586	1.1	0.2507	0.137504	0.600846
ENSBTAP00000055449.1	ENSBTAG00000051631	0.956	0.69144	-0.06492	0.160246
ENSBTAP00000055456.1	ENSBTAG00000045678	1.11	0.195144	0.15056	0.709645
ENSBTAP00000055469.1	ENSBTAG00000048135	0.767	0.70348	-0.3827	0.152748
ENSBTAP00000055705.1	ENSBTAG00000010083	1.112	0.4428	0.153157	0.353792
ENSBTAP00000055780.1	ENSBTAG00000045757	1.24	0.2592	0.31034	0.586365
ENSBTAP00000055847.1	ENSBTAG00000047040	0.575	0.198761	-0.79837	0.701669
ENSBTAP00000055884.1	ENSBTAG00000018167	0.902	0.187483	-0.1488	0.727038
ENSBTAP00000055956.2	ENSBTAG00000009035	NA	NA	NA	NA
ENSBTAP00000055977.2	ENSBTAG00000047330	1.179	0.164003	0.237564	0.785148
ENSBTAP00000056220.2	ENSBTAG00000046385	1.083	0.30834	0.115033	0.51097
ENSBTAP00000056372.1	ENSBTAG00000046712	0.989	0.80896	-0.01596	0.092073
ENSBTAP00000056488.2	ENSBTAG00000032292	1.129	0.189255	0.175045	0.722953
ENSBTAP00000056535.2	ENSBTAG00000002392	0.98	0.71694	-0.02915	0.144517
ENSBTAP00000056536.1	ENSBTAG00000045980	NA	NA	NA	NA
ENSBTAP00000056662.1	ENSBTAG00000010428	0.958	0.39944	-0.0619	0.398548
ENSBTAP00000056719.1	ENSBTAG00000007240	1.028	0.68074	0.03984	0.167019

ENSBTAP00000056758.1	ENSBTAG00000018201	1.168	0.033659	0.22404	1.472899
ENSBTAP00000056778.1	ENSBTAG00000004833	NA	NA	NA	NA
ENSBTAP00000056810.1	ENSBTAG00000055296	NA	NA	NA	NA
ENSBTAP00000056819.1	ENSBTAG00000003887	1.081	0.36224	0.112367	0.441004
ENSBTAP00000056879.1	ENSBTAG00000008744	NA	NA	NA	NA
ENSBTAP00000056903.1	ENSBTAG00000021061	0.99	0.91396	-0.0145	0.039073
ENSBTAP00000056905.1	ENSBTAG00000014151	0.849	0.008042	-0.23616	2.094652
ENSBTAP00000056916.1	ENSBTAG00000015314	1.089	0.36936	0.123004	0.43255
ENSBTAP00000056945.1	ENSBTAG00000051336	1.168	0.113715	0.22404	0.944182
ENSBTAP00000057058.1	ENSBTAG00000010677	1.054	0.62254	0.075875	0.205833
ENSBTAP00000057102.1	ENSBTAG00000015731	0.828	0.149895	-0.2723	0.824213
ENSBTAP00000057147.1	ENSBTAG00000008024	1.02	0.89908	0.028569	0.046202
ENSBTAP00000057222.1	ENSBTAG00000005373	1.196	0.184382	0.258217	0.734281
ENSBTAP00000057236.1	ENSBTAG00000004672	0.94	0.23368	-0.08927	0.631378
ENSBTAP00000057245.1	ENSBTAG00000044160	1.095	0.00334	0.130931	2.476202
ENSBTAP00000057247.1	ENSBTAG00000010576	1.065	0.20062	0.090853	0.697626
ENSBTAP00000057259.1	ENSBTAG00000012357	0.944	0.64578	-0.08314	0.189915
ENSBTAP00000057277.1	ENSBTAG00000011772	1.184	0.24374	0.243669	0.613073
ENSBTAP00000057299.1	ENSBTAG00000001120	0.945	0.369	-0.08161	0.432974
ENSBTAP00000057302.1	ENSBTAG00000000874	0.91	0.29576	-0.13606	0.529061
ENSBTAP00000057314.1	ENSBTAG00000014648	1.02	0.74866	0.028569	0.125715
ENSBTAP00000057366.1	ENSBTAG00000050560	1.116	0.09774	0.158337	1.009928
ENSBTAP00000057372.1	ENSBTAG00000004887	1.011	0.84632	0.015783	0.072465
ENSBTAP00000057459.1	ENSBTAG00000050873	1.014	0.87742	0.020058	0.056792
ENSBTAP00000057467.1	ENSBTAG00000001933	1.03	0.21248	0.042644	0.672682
ENSBTAP00000057509.1	ENSBTAG00000012279	1.094	0.29284	0.129613	0.53337
ENSBTAP00000057536.1	ENSBTAG00000003876	0.976	0.76578	-0.03505	0.115896
ENSBTAP00000057600.1	ENSBTAG00000002767	1.044	0.28046	0.062122	0.552129
ENSBTAP00000057643.1	ENSBTAG00000010701	1.004	0.75966	0.005759	0.119381
ENSBTAP00000057668.1	ENSBTAG00000014388	1.144	0.109441	0.194087	0.96082
ENSBTAP00000057679.1	ENSBTAG00000025853	1.02	0.77794	0.028569	0.109054
ENSBTAP00000057708.1	ENSBTAG00000006907	NA	NA	NA	NA
ENSBTAP00000057848.1	ENSBTAG00000015609	1.416	0.30898	0.501821	0.51007
ENSBTAP00000057873.1	ENSBTAG00000001942	1.034	0.50538	0.048236	0.296382
ENSBTAP00000057919.1	ENSBTAG00000010232	1.065	0.67884	0.090853	0.168233
ENSBTAP00000057926.1	ENSBTAG00000049723	1.001	0.98244	0.001442	0.007694
ENSBTAP00000057947.1	ENSBTAG00000003747	1.043	0.37744	0.060739	0.423152
ENSBTAP00000057956.1	ENSBTAG00000014319	1.074	0.197021	0.102994	0.705487
ENSBTAP00000058051.1	ENSBTAG00000002377	0.957	0.5488	-0.06341	0.260586
ENSBTAP00000058088.1	ENSBTAG00000006122	NA	NA	NA	NA
ENSBTAP00000058093.1	ENSBTAG00000050219	NA	NA	NA	NA
ENSBTAP00000058102.1	ENSBTAG00000009067	1.014	0.74022	0.020058	0.130639
ENSBTAP00000058112.1	ENSBTAG00000032477	NA	NA	NA	NA
ENSBTAP00000058144.1	ENSBTAG00000018497	1.077	0.65526	0.107018	0.183586
ENSBTAP00000058148.1	ENSBTAG00000048057	NA	NA	NA	NA
ENSBTAP00000058153.1	ENSBTAG00000009778	1.005	0.90536	0.007196	0.043179
ENSBTAP00000058160.1	ENSBTAG00000018469	1.012	0.83606	0.017209	0.077763
ENSBTAP00000058193.1	ENSBTAG00000047249	1.009	0.65604	0.012926	0.18307
ENSBTAP00000058286.1	ENSBTAG00000021746	1.109	0.24452	0.149259	0.611686
ENSBTAP00000058351.1	ENSBTAG00000015403	1.141	0.27584	0.190299	0.559343
ENSBTAP00000058362.1	ENSBTAG00000014806	0.879	0.2572	-0.18606	0.589729
ENSBTAP00000058366.1	ENSBTAG00000007268	1.017	0.83996	0.02432	0.075741
ENSBTAP00000058394.1	ENSBTAG00000010082	1.026	0.20688	0.037031	0.684281
ENSBTAP00000058396.1	ENSBTAG00000020043	0.99	0.76672	-0.0145	0.115363
ENSBTAP00000058462.1	ENSBTAG00000010971	1.227	0.137222	0.295135	0.862576
ENSBTAP00000058471.1	ENSBTAG00000012128	0.935	0.29872	-0.09696	0.524736
ENSBTAP00000058501.1	ENSBTAG00000021979	1.007	0.80802	0.010064	0.092578
ENSBTAP00000058549.1	ENSBTAG00000004271	1.021	0.71782	0.029983	0.143984

ENSBTAP00000058561.1	ENSBTAG00000010619	0.954	0.26584	-0.06794	0.57538
ENSBTAP00000058569.1	ENSBTAG00000020783	0.884	0.50724	-0.17788	0.294787
ENSBTAP00000058576.1	ENSBTAG00000051767	0.936	0.61546	-0.09542	0.2108
ENSBTAP00000058608.1	ENSBTAG00000051486	NA	NA	NA	NA
ENSBTAP00000058610.1	ENSBTAG00000025644	0.971	0.56924	-0.04246	0.244705
ENSBTAP00000058673.1	ENSBTAG00000013491	1.081	0.098481	0.112367	1.006648
ENSBTAP00000058705.1	ENSBTAG00000019663	1.283	0.194218	0.359521	0.711711
ENSBTAP00000058720.1	ENSBTAG00000053755	NA	NA	NA	NA
ENSBTAP00000058727.1	ENSBTAG00000021879	0.935	0.26114	-0.09696	0.583127
ENSBTAP00000058744.1	ENSBTAG00000001795	1.115	0.20722	0.157044	0.683568
ENSBTAP00000058780.1	ENSBTAG00000006071	NA	NA	NA	NA
ENSBTAP00000058812.1	ENSBTAG00000050723	1.006	0.92298	0.00863	0.034808
ENSBTAP00000058814.1	ENSBTAG00000006693	NA	NA	NA	NA
ENSBTAP00000058825.1	ENSBTAG00000015625	NA	NA	NA	NA
ENSBTAP00000058833.1	ENSBTAG00000048924	0.948	0.51386	-0.07704	0.289155
ENSBTAP00000058983.1	ENSBTAG00000046033	1.067	0.27512	0.09356	0.560478
ENSBTAP00000058989.1	ENSBTAG00000005373	1.09	0.3726	0.124328	0.428757
ENSBTAP00000059026.1	ENSBTAG00000003530	NA	NA	NA	NA
ENSBTAP00000059066.1	ENSBTAG00000048800	NA	NA	NA	NA
ENSBTAP00000059096.1	ENSBTAG00000010786	0.932	0.51364	-0.1016	0.289341
ENSBTAP00000059114.1	ENSBTAG00000003162	0.956	0.21402	-0.06492	0.669546
ENSBTAP00000059143.1	ENSBTAG00000018278	1.09	0.44888	0.124328	0.34787
ENSBTAP00000059149.1	ENSBTAG00000048654	NA	NA	NA	NA
ENSBTAP00000059175.1	ENSBTAG00000012072	1.052	0.8169	0.073135	0.087831
ENSBTAP00000059218.1	ENSBTAG00000010611	1.037	0.3384	0.052416	0.47057
ENSBTAP00000059246.1	ENSBTAG00000018317	1.066	0.052815	0.092207	1.277243
ENSBTAP00000059250.1	ENSBTAG00000005969	1.008	0.8845	0.011496	0.053302
ENSBTAP00000059334.1	ENSBTAG00000011580	1.032	0.61906	0.045443	0.208267
ENSBTAP00000059347.1	ENSBTAG00000016420	1.009	0.95746	0.012926	0.018879
ENSBTAP00000059369.1	ENSBTAG00000011950	1.013	0.61128	0.018634	0.21376
ENSBTAP00000059410.1	ENSBTAG00000052033	1.004	0.84218	0.005759	0.074595
ENSBTAP00000059451.1	ENSBTAG00000011414	1.189	0.2502	0.249749	0.601713
ENSBTAP00000059478.1	ENSBTAG00000046869	0.985	0.90328	-0.0218	0.044178
ENSBTAP00000059541.1	ENSBTAG00000009841	0.817	0.160219	-0.29159	0.795286
ENSBTAP00000059699.1	ENSBTAG00000005681	0.976	0.8225	-0.03505	0.084864
ENSBTAP00000059713.1	ENSBTAG00000039117	NA	NA	NA	NA
ENSBTAP00000059727.1	ENSBTAG00000006393	NA	NA	NA	NA
ENSBTAP00000059747.1	ENSBTAG00000001398	1.205	0.40658	0.269033	0.390854
ENSBTAP00000059787.1	ENSBTAG00000013123	1.097	0.28184	0.133564	0.549997
ENSBTAP00000059803.1	ENSBTAG00000016363	1.042	0.25536	0.059355	0.592847
ENSBTAP00000059820.1	ENSBTAG00000012632	0.949	0.30038	-0.07552	0.522329
ENSBTAP00000059844.1	ENSBTAG00000012338	1.095	0.59504	0.130931	0.225454
ENSBTAP00000059895.1	ENSBTAG00000005791	NA	NA	NA	NA
ENSBTAP00000059946.1	ENSBTAG00000039555	1.115	0.2426	0.157044	0.615109
ENSBTAP00000059956.1	ENSBTAG00000050515	2.162	0.146636	1.112367	0.833759
ENSBTAP00000060013.1	ENSBTAG00000007335	1.149	0.38956	0.200379	0.409426
ENSBTAP00000060079.1	ENSBTAG00000017664	0.983	0.89572	-0.02474	0.047828
ENSBTAP00000060095.1	ENSBTAG00000016336	0.872	0.29724	-0.1976	0.526893
ENSBTAP00000060130.1	ENSBTAG00000003484	1.043	0.39656	0.060739	0.401691
ENSBTAP00000060196.1	ENSBTAG00000014463	0.994	0.86122	-0.00868	0.064886
ENSBTAP00000060226.1	ENSBTAG00000048244	0.947	0.40272	-0.07856	0.394997
ENSBTAP00000060242.1	ENSBTAG00000020969	1.079	0.169976	0.109695	0.769612
ENSBTAP00000060332.1	ENSBTAG00000015551	NA	NA	NA	NA
ENSBTAP00000060352.1	ENSBTAG00000009449	0.746	0.036058	-0.42275	1.442998
ENSBTAP00000060375.1	ENSBTAG00000011834	NA	NA	NA	NA
ENSBTAP00000060423.1	ENSBTAG00000018399	0.884	0.39328	-0.17788	0.405298
ENSBTAP00000060466.1	ENSBTAG00000007474	1.147	0.108182	0.197865	0.965845
ENSBTAP00000060497.1	ENSBTAG00000013930	1.091	0.4752	0.125651	0.323124

ENSBTAP00000060538.1	ENSBTAG00000027020	1.014	0.88834	0.020058	0.051421
ENSBTAP00000060553.1	ENSBTAG00000011215	1.085	0.27608	0.117695	0.558965
ENSBTAP00000060578.1	ENSBTAG00000013411	0.924	0.166943	-0.11404	0.777432
ENSBTAP00000060682.1	ENSBTAG00000007109	0.992	0.85114	-0.01159	0.069999
ENSBTAP00000060698.1	ENSBTAG00000003307	1.285	0.04264	0.361768	1.370183
ENSBTAP00000060747.1	ENSBTAG00000053103	0.984	0.93426	-0.02327	0.029532
ENSBTAP00000060756.1	ENSBTAG00000006995	0.915	0.4677	-0.12816	0.330033
ENSBTAP00000060808.1	ENSBTAG00000020087	1.03	0.5837	0.042644	0.23381
ENSBTAP00000060872.1	ENSBTAG00000001932	1.106	0.52852	0.145351	0.276939
ENSBTAP00000060889.1	ENSBTAG00000008842	0.977	0.44626	-0.03357	0.350412
ENSBTAP00000060915.1	ENSBTAG00000002985	1.074	0.52108	0.102994	0.283096
ENSBTAP00000060921.1	ENSBTAG00000031295	1.119	0.065859	0.16221	1.181385
ENSBTAP00000060938.1	ENSBTAG00000054647	NA	NA	NA	NA
ENSBTAP00000060946.1	ENSBTAG00000018282	1.064	0.123081	0.089498	0.909809
ENSBTAP00000060958.1	ENSBTAG00000016105	1.049	0.2966	0.069015	0.527829
ENSBTAP00000061027.1	ENSBTAG00000019156	1.036	0.81292	0.051024	0.089952
ENSBTAP00000061047.1	ENSBTAG00000039374	0.894	0.011076	-0.16165	1.955637
ENSBTAP00000061121.1	ENSBTAG00000018669	0.99	0.8433	-0.0145	0.074018
ENSBTAP00000061122.1	ENSBTAG00000054195	0.814	0.2529	-0.2969	0.597051
ENSBTAP00000061148.1	ENSBTAG00000004438	1.026	0.7562	0.037031	0.121363
ENSBTAP00000061170.1	ENSBTAG00000015839	1.003	0.88304	0.004322	0.05402
ENSBTAP00000061184.1	ENSBTAG00000004709	NA	NA	NA	NA
ENSBTAP00000061185.1	ENSBTAG00000010890	0.888	0.27868	-0.17137	0.554894
ENSBTAP00000061187.1	ENSBTAG00000015437	NA	NA	NA	NA
ENSBTAP00000061210.1	ENSBTAG00000044003	0.988	0.6612	-0.01742	0.179667
ENSBTAP00000061238.1	ENSBTAG00000014032	1.036	0.25116	0.051024	0.60005
ENSBTAP00000061245.1	ENSBTAG00000014809	1.094	0.120676	0.129613	0.918379
ENSBTAP00000061249.1	ENSBTAG00000051210	0.971	0.75682	-0.04246	0.121007
ENSBTAP00000061269.1	ENSBTAG00000016516	0.962	0.44772	-0.05589	0.348994
ENSBTAP00000061306.1	ENSBTAG00000006253	1.038	0.40296	0.053806	0.394738
ENSBTAP00000061316.1	ENSBTAG00000012653	1.061	0.178656	0.085425	0.747982
ENSBTAP00000061325.1	ENSBTAG00000015692	1.082	0.47626	0.1137	0.322156
ENSBTAP00000061339.1	ENSBTAG00000020223	0.96	0.41554	-0.05889	0.381387
ENSBTAP00000061356.1	ENSBTAG00000012667	0.99	0.99074	-0.0145	0.00404
ENSBTAP00000061432.1	ENSBTAG00000017713	1.035	0.77108	0.049631	0.112901
ENSBTAP00000061449.1	ENSBTAG00000005762	0.902	0.31136	-0.1488	0.506737
ENSBTAP00000061542.1	ENSBTAG00000000207	NA	NA	NA	NA
ENSBTAP00000061615.1	ENSBTAG00000006165	1.058	0.025301	0.08134	1.596862
ENSBTAP00000061667.1	ENSBTAG00000003809	0.936	0.51776	-0.09542	0.285872
ENSBTAP00000061676.1	ENSBTAG00000022004	NA	NA	NA	NA
ENSBTAP00000061717.1	ENSBTAG00000005664	0.977	0.45774	-0.03357	0.339381
ENSBTAP00000061723.1	ENSBTAG00000055118	23.267	0.23266	4.540213	0.633278
ENSBTAP00000061773.1	ENSBTAG00000008935	0.979	0.6426	-0.03062	0.192059
ENSBTAP00000061793.1	ENSBTAG00000005464	NA	NA	NA	NA
ENSBTAP00000061829.1	ENSBTAG00000055045	1.227	0.131417	0.295135	0.881348
ENSBTAP00000061830.1	ENSBTAG00000054218	NA	NA	NA	NA
ENSBTAP00000061832.1	ENSBTAG00000017799	NA	NA	NA	NA
ENSBTAP00000061900.1	ENSBTAG00000003830	1.016	0.79506	0.0229	0.0996
ENSBTAP00000061941.1	ENSBTAG00000019284	0.972	0.50974	-0.04097	0.292651
ENSBTAP00000061946.1	ENSBTAG00000011922	0.974	0.37204	-0.03801	0.42941
ENSBTAP00000061950.1	ENSBTAG00000000792	0.916	0.42822	-0.12658	0.368333
ENSBTAP00000061953.1	ENSBTAG00000008293	NA	NA	NA	NA
ENSBTAP00000061957.1	ENSBTAG00000014840	0.859	0.177915	-0.21927	0.749787
ENSBTAP00000061981.1	ENSBTAG00000007807	1.09	0.72878	0.124328	0.137404
ENSBTAP00000062056.1	ENSBTAG00000013744	0.969	0.20104	-0.04543	0.696718
ENSBTAP00000062075.1	ENSBTAG00000016975	1.044	0.57722	0.062122	0.238659
ENSBTAP00000062110.1	ENSBTAG00000007360	1.087	0.3902	0.120352	0.408713
ENSBTAP00000062118.1	ENSBTAG00000010661	1.213	0.50048	0.27858	0.300613

ENSBTAP00000062132.1	ENSBTAG00000050974	NA	NA	NA	NA
ENSBTAP00000062154.1	ENSBTAG00000050904	NA	NA	NA	NA
ENSBTAP00000062172.1	ENSBTAG00000003279	1.05	0.72734	0.070389	0.138263
ENSBTAP00000062239.1	ENSBTAG00000002871	0.921	0.137024	-0.11873	0.863203
ENSBTAP00000062291.1	ENSBTAG00000005373	1.167	0.029877	0.222805	1.524663
ENSBTAP00000062336.1	ENSBTAG00000001851	0.759	0.07166	-0.39783	1.144723
ENSBTAP00000062338.1	ENSBTAG00000011263	1.046	0.6873	0.064883	0.162854
ENSBTAP00000062470.1	ENSBTAG00000017183	1.436	0.27164	0.522056	0.566006
ENSBTAP00000062473.1	ENSBTAG00000005207	NA	NA	NA	NA
ENSBTAP00000062506.1	ENSBTAG00000052821	0.984	0.82496	-0.02327	0.083567
ENSBTAP00000062514.1	ENSBTAG00000009554	NA	NA	NA	NA
ENSBTAP00000062524.1	ENSBTAG00000013108	NA	NA	NA	NA
ENSBTAP00000062556.1	ENSBTAG00000006765	NA	NA	NA	NA
ENSBTAP00000062563.1	ENSBTAG00000002847	1.233	0.068683	0.302173	1.163151
ENSBTAP00000062596.1	ENSBTAG00000019327	1.033	0.30798	0.04684	0.511477
ENSBTAP00000062685.1	ENSBTAG00000049190	0.946	0.51856	-0.08009	0.285201
ENSBTAP00000062687.1	ENSBTAG00000011198	NA	NA	NA	NA
ENSBTAP00000062703.1	ENSBTAG00000052443	0.932	0.3117	-0.1016	0.506263
ENSBTAP00000062815.1	ENSBTAG00000019011	0.971	0.71926	-0.04246	0.143114
ENSBTAP00000062820.1	ENSBTAG00000002880	1	0.9633	0	0.016238
ENSBTAP00000062837.1	ENSBTAG00000017143	1.019	0.82634	0.027154	0.082841
ENSBTAP00000062873.1	ENSBTAG00000016956	1.097	0.119559	0.133564	0.922418
ENSBTAP00000062926.1	ENSBTAG00000020547	0.94	0.46938	-0.08927	0.328475
ENSBTAP00000062993.1	ENSBTAG00000000902	1.027	0.69228	0.038436	0.159718
ENSBTAP00000063000.1	ENSBTAG00000010693	1.125	0.000479	0.169925	3.31922
ENSBTAP00000063027.1	ENSBTAG00000051801	1.067	0.61906	0.09356	0.208267
ENSBTAP00000063043.1	ENSBTAG00000023039	1.103	0.31454	0.141433	0.502324
ENSBTAP00000063060.1	ENSBTAG00000016174	0.975	0.69176	-0.03653	0.160045
ENSBTAP00000063097.1	ENSBTAG00000007133	1.004	0.82456	0.005759	0.083778
ENSBTAP00000063101.1	ENSBTAG00000005851	1.009	0.86716	0.012926	0.061901
ENSBTAP00000063112.1	ENSBTAG00000016133	NA	NA	NA	NA
ENSBTAP00000063127.1	ENSBTAG00000008449	0.918	0.48436	-0.12343	0.314832
ENSBTAP00000063200.1	ENSBTAG00000012849	1.087	0.3655	0.120352	0.437113
ENSBTAP00000063221.1	ENSBTAG00000024450	NA	NA	NA	NA
ENSBTAP00000063261.1	ENSBTAG00000006075	1.083	0.5696	0.115033	0.24443
ENSBTAP00000063263.1	ENSBTAG00000000111	0.934	0.42964	-0.09851	0.366895
ENSBTAP00000063274.1	ENSBTAG00000018041	NA	NA	NA	NA
ENSBTAP00000063275.1	ENSBTAG00000020757	0.993	0.91332	-0.01013	0.039377
ENSBTAP00000063281.1	ENSBTAG00000006618	0.92	0.129018	-0.12029	0.88935
ENSBTAP00000063287.1	ENSBTAG00000009861	1.042	0.75588	0.059355	0.121547
ENSBTAP00000063290.1	ENSBTAG00000049452	NA	NA	NA	NA
ENSBTAP00000063310.1	ENSBTAG00000014581	1.001	0.9595	0.001442	0.017955
ENSBTAP00000063391.1	ENSBTAG00000010230	1.009	0.82412	0.012926	0.08401
ENSBTAP00000063536.1	ENSBTAG00000051082	1.061	0.3874	0.085425	0.41184
ENSBTAP00000063550.1	ENSBTAG00000016007	0.99	0.9694	-0.0145	0.013497
ENSBTAP00000063565.1	ENSBTAG00000007523	1.034	0.43494	0.048236	0.361571
ENSBTAP00000063578.1	ENSBTAG00000048761	1.057	0.45892	0.079975	0.338263
ENSBTAP00000063609.1	ENSBTAG00000019271	1.013	0.822	0.018634	0.085128
ENSBTAP00000063617.1	ENSBTAG00000000310	0.849	0.086456	-0.23616	1.063205
ENSBTAP00000063618.1	ENSBTAG00000015000	1.014	0.71328	0.020058	0.14674
ENSBTAP00000063657.1	ENSBTAG00000025210	1.043	0.55874	0.060739	0.25279
ENSBTAP00000063669.1	ENSBTAG00000008185	0.974	0.50242	-0.03801	0.298933
ENSBTAP00000063744.1	ENSBTAG00000019525	1.025	0.45956	0.035624	0.337658
ENSBTAP00000063748.1	ENSBTAG00000009414	1.011	0.69988	0.015783	0.154976
ENSBTAP00000063749.1	ENSBTAG00000044017	1.109	0.189602	0.149259	0.722157
ENSBTAP00000063760.1	ENSBTAG00000015611	1.126	0.31558	0.171207	0.500891
ENSBTAP00000063785.1	ENSBTAG00000014426	1.002	0.99342	0.002883	0.002867
ENSBTAP00000063798.1	ENSBTAG00000018707	0.97	0.5148	-0.04394	0.288361

ENSBTAP00000063812.1	ENSBTAG00000032829	1.08	0.3235	0.111031	0.490126
ENSBTAP00000063820.1	ENSBTAG00000014059	NA	NA	NA	NA
ENSBTAP00000063846.1	ENSBTAG00000006661	NA	NA	NA	NA
ENSBTAP00000063847.1	ENSBTAG00000030335	1.025	0.77582	0.035624	0.110239
ENSBTAP00000063850.1	ENSBTAG00000014930	0.972	0.76958	-0.04097	0.113746
ENSBTAP00000063874.1	ENSBTAG00000007784	NA	NA	NA	NA
ENSBTAP00000063896.1	ENSBTAG00000006907	1.025	0.75768	0.035624	0.120514
ENSBTAP00000063903.1	ENSBTAG00000014226	NA	NA	NA	NA
ENSBTAP00000063932.1	ENSBTAG00000019851	1.073	0.145398	0.10165	0.837442
ENSBTAP00000063933.1	ENSBTAG00000002408	0.946	0.151216	-0.08009	0.820402
ENSBTAP00000063991.1	ENSBTAG00000008736	1.003	0.96072	0.004322	0.017403
ENSBTAP00000064065.1	ENSBTAG00000011571	NA	NA	NA	NA
ENSBTAP00000064080.1	ENSBTAG00000015571	NA	NA	NA	NA
ENSBTAP00000064112.1	ENSBTAG00000009696	0.842	0.187159	-0.24811	0.727789
ENSBTAP00000064123.1	ENSBTAG00000001721	1.044	0.63614	0.062122	0.196447
ENSBTAP00000064153.1	ENSBTAG00000009106	1.033	0.27592	0.04684	0.559217
ENSBTAP00000064221.1	ENSBTAG00000006253	0.905	0.08756	-0.14401	1.057694
ENSBTAP00000064227.1	ENSBTAG00000006883	1.03	0.61092	0.042644	0.214016
ENSBTAP00000064251.1	ENSBTAG00000007865	0.893	0.155902	-0.16327	0.807148
ENSBTAP00000064340.1	ENSBTAG00000000679	1.024	0.6091	0.034216	0.215311
ENSBTAP00000064357.1	ENSBTAG00000021514	1.011	0.81558	0.015783	0.088533
ENSBTAP00000064368.1	ENSBTAG00000000256	0.642	0.2975	-0.63935	0.526513
ENSBTAP00000064437.1	ENSBTAG00000013606	NA	NA	NA	NA
ENSBTAP00000064454.1	ENSBTAG00000052518	1.038	0.50264	0.053806	0.298743
ENSBTAP00000064487.1	ENSBTAG00000011395	0.796	0.4411	-0.32916	0.355463
ENSBTAP00000064496.1	ENSBTAG00000003098	1.042	0.059905	0.059355	1.222537
ENSBTAP00000064670.1	ENSBTAG00000015805	1.17	0.65798	0.226509	0.181787
ENSBTAP00000064676.1	ENSBTAG00000048553	0.98	0.41956	-0.02915	0.377206
ENSBTAP00000064689.1	ENSBTAG00000047491	0.926	0.038283	-0.11092	1.416994
ENSBTAP00000064693.1	ENSBTAG00000055082	1.131	0.20516	0.177599	0.687907
ENSBTAP00000064698.1	ENSBTAG00000053489	1.043	0.7891	0.060739	0.102868
ENSBTAP00000064770.1	ENSBTAG00000016676	0.766	0.148458	-0.38458	0.828396
ENSBTAP00000064800.1	ENSBTAG00000009770	1.072	0.23442	0.100305	0.630005
ENSBTAP00000064923.1	ENSBTAG00000007635	0.943	0.19956	-0.08467	0.699927
ENSBTAP00000064952.1	ENSBTAG00000033186	0.973	0.90436	-0.03949	0.043659
ENSBTAP00000064995.1	ENSBTAG00000009641	1.04	0.69694	0.056584	0.156805
ENSBTAP00000065000.1	ENSBTAG00000005333	1.029	0.78592	0.041243	0.104622
ENSBTAP00000065017.1	ENSBTAG00000001700	1.014	0.76126	0.020058	0.118467
ENSBTAP00000065079.1	ENSBTAG00000005960	0.979	0.84082	-0.03062	0.075297
ENSBTAP00000065107.1	ENSBTAG00000046534	0.997	0.92002	-0.00433	0.036203
ENSBTAP00000065109.1	ENSBTAG00000021491	1.176	0.3188	0.233888	0.496482
ENSBTAP00000065138.1	ENSBTAG00000013653	NA	NA	NA	NA
ENSBTAP00000065168.1	ENSBTAG00000027525	1.029	0.44904	0.041243	0.347715
ENSBTAP00000065170.1	ENSBTAG00000016278	0.979	0.32744	-0.03062	0.484868
ENSBTAP00000065210.1	ENSBTAG00000001113	NA	NA	NA	NA
ENSBTAP00000065213.1	ENSBTAG00000024487	0.967	0.5137	-0.04841	0.28929
ENSBTAP00000065243.1	ENSBTAG00000016281	1.014	0.77214	0.020058	0.112304
ENSBTAP00000065269.1	ENSBTAG00000053210	1.05	0.43358	0.070389	0.362931
ENSBTAP00000065270.1	ENSBTAG00000015414	0.941	0.118602	-0.08773	0.925908
ENSBTAP00000065272.1	ENSBTAG00000001083	1.01	0.84876	0.014355	0.071215
ENSBTAP00000065281.1	ENSBTAG00000008817	1.032	0.66788	0.045443	0.175302
ENSBTAP00000065306.1	ENSBTAG00000002964	0.983	0.76332	-0.02474	0.117293
ENSBTAP00000065312.1	ENSBTAG00000014885	NA	NA	NA	NA
ENSBTAP00000065351.1	ENSBTAG00000000242	0.977	0.54136	-0.03357	0.266514
ENSBTAP00000065358.1	ENSBTAG00000004910	1.033	0.75704	0.04684	0.120881
ENSBTAP00000065366.1	ENSBTAG00000020329	1.041	0.72546	0.05797	0.139387
ENSBTAP00000065386.1	ENSBTAG00000017120	1.089	0.2795	0.123004	0.553618
ENSBTAP00000065456.1	ENSBTAG00000011623	NA	NA	NA	NA

ENSBTAP00000065459.1	ENSBTAG00000018905	NA	NA	NA	NA
ENSBTAP00000065495.1	ENSBTAG00000004072	0.979	0.30936	-0.03062	0.509536
ENSBTAP00000065522.1	ENSBTAG00000003667	1.002	0.93026	0.002883	0.031396
ENSBTAP00000065591.1	ENSBTAG00000009761	NA	NA	NA	NA
ENSBTAP00000065593.1	ENSBTAG00000008575	1.011	0.92854	0.015783	0.032199
ENSBTAP00000065595.1	ENSBTAG00000010015	NA	NA	NA	NA
ENSBTAP00000065612.1	ENSBTAG00000040338	1.091	0.08776	0.125651	1.056703
ENSBTAP00000065670.1	ENSBTAG00000016415	1.081	0.24234	0.112367	0.615575
ENSBTAP00000065724.1	ENSBTAG00000021218	0.933	0.37536	-0.10005	0.425552
ENSBTAP00000065838.1	ENSBTAG00000053114	1.383	0.079724	0.467801	1.098411
ENSBTAP00000065840.1	ENSBTAG00000021151	NA	NA	NA	NA
ENSBTAP00000065948.1	ENSBTAG00000053974	1.019	0.43602	0.027154	0.360494
ENSBTAP00000065986.1	ENSBTAG00000004806	0.933	0.48316	-0.10005	0.315909
ENSBTAP00000066071.1	ENSBTAG00000010206	1.059	0.28298	0.082703	0.548244
ENSBTAP00000066083.1	ENSBTAG00000051871	0.951	0.53728	-0.07248	0.269799
ENSBTAP00000066121.1	ENSBTAG00000038794	0.897	0.70872	-0.15682	0.149525
ENSBTAP00000066127.1	ENSBTAG00000001609	1.048	0.4058	0.067639	0.391688
ENSBTAP00000066128.1	ENSBTAG00000002853	1.029	0.71436	0.041243	0.146083
ENSBTAP00000066190.1	ENSBTAG00000017831	1.058	0.70778	0.08134	0.150102
ENSBTAP00000066203.1	ENSBTAG00000010777	1.103	0.27472	0.141433	0.56111
ENSBTAP00000066226.1	ENSBTAG00000016679	1.074	0.67506	0.102994	0.170658
ENSBTAP00000066231.1	ENSBTAG00000008621	0.996	0.86436	-0.00578	0.063305
ENSBTAP00000066281.1	ENSBTAG00000013530	NA	NA	NA	NA
ENSBTAP00000066303.1	ENSBTAG00000050870	0.921	0.5758	-0.11873	0.239728
ENSBTAP00000066319.1	ENSBTAG00000015958	1.028	0.69532	0.03984	0.157815
ENSBTAP00000066336.1	ENSBTAG00000006907	1.119	0.48344	0.16221	0.315657
ENSBTAP00000066341.1	ENSBTAG00000017866	1.046	0.61464	0.064883	0.211379
ENSBTAP00000066347.1	ENSBTAG00000016244	0.477	0.79658	-1.06794	0.098771
ENSBTAP00000066349.1	ENSBTAG00000008921	0.867	0.050961	-0.2059	1.292762
ENSBTAP00000066420.1	ENSBTAG00000011250	1.133	0.36646	0.180148	0.435973
ENSBTAP00000066432.1	ENSBTAG00000014138	0.986	0.94934	-0.02034	0.022578
ENSBTAP00000066442.1	ENSBTAG00000020649	0.425	0.89106	-1.23447	0.050093
ENSBTAP00000066467.1	ENSBTAG00000013369	0.938	0.65904	-0.09234	0.181088
ENSBTAP00000066475.1	ENSBTAG00000020498	1.037	0.5142	0.052416	0.288868
ENSBTAP00000066476.1	ENSBTAG00000016779	1.018	0.106238	0.025738	0.97372
ENSBTAP00000066521.1	ENSBTAG00000021992	0.982	0.55458	-0.02621	0.256036
ENSBTAP00000066541.1	ENSBTAG00000016566	0.996	0.9149	-0.00578	0.038626
ENSBTAP00000066552.1	ENSBTAG00000051755	1.006	0.92222	0.00863	0.035165
ENSBTAP00000066597.1	ENSBTAG00000003401	0.944	0.26366	-0.08314	0.578956
ENSBTAP00000066604.1	ENSBTAG00000004344	1.075	0.5492	0.104337	0.260269
ENSBTAP00000066612.1	ENSBTAG00000006999	NA	NA	NA	NA
ENSBTAP00000066638.1	ENSBTAG00000016311	1.015	0.7436	0.02148	0.128661
ENSBTAP00000066667.1	ENSBTAG00000020456	0.946	0.062842	-0.08009	1.20175
ENSBTAP00000066719.1	ENSBTAG00000010244	1.09	0.50212	0.124328	0.299192
ENSBTAP00000066722.1	ENSBTAG00000002997	0.926	0.029743	-0.11092	1.526615
ENSBTAP00000066752.1	ENSBTAG00000051352	1.039	0.55654	0.055196	0.254504
ENSBTAP00000066803.1	ENSBTAG00000004771	0.91	0.1476	-0.13606	0.830914
ENSBTAP00000066855.1	ENSBTAG00000008609	1.063	0.83584	0.088142	0.077877
ENSBTAP00000066883.1	ENSBTAG00000002038	0.872	0.44168	-0.1976	0.354892
ENSBTAP00000066912.1	ENSBTAG00000019386	NA	NA	NA	NA
ENSBTAP00000067034.1	ENSBTAG00000055033	1.004	0.89328	0.005759	0.049012
ENSBTAP00000067036.1	ENSBTAG00000008868	1.005	0.88042	0.007196	0.05531
ENSBTAP00000067117.1	ENSBTAG00000003061	1.03	0.89982	0.042644	0.045844
ENSBTAP00000067188.1	ENSBTAG00000014130	1.162	0.21926	0.21661	0.659041
ENSBTAP00000067222.1	ENSBTAG00000004554	1.031	0.34162	0.044044	0.466457
ENSBTAP00000067245.1	ENSBTAG00000014643	1.09	0.29744	0.124328	0.526601
ENSBTAP00000067251.1	ENSBTAG00000015221	0.96	0.7503	-0.05889	0.124765
ENSBTAP00000067259.1	ENSBTAG00000003629	0.846	0.48046	-0.24127	0.318343

ENSBTAP00000067275.1	ENSBTAG00000013699	0.967	0.54346	-0.04841	0.264832
ENSBTAP00000067311.1	ENSBTAG00000020905	1.061	0.104322	0.085425	0.981624
ENSBTAP00000067361.1	ENSBTAG00000011583	1.143	0.2301	0.192825	0.638083
ENSBTAP00000067366.1	ENSBTAG00000018108	1.007	0.96116	0.010064	0.017204
ENSBTAP00000067372.1	ENSBTAG00000008417	0.997	0.92188	-0.00433	0.035326
ENSBTAP00000067377.1	ENSBTAG00000000799	1.073	0.30574	0.10165	0.514648
ENSBTAP00000067387.1	ENSBTAG00000009603	1.123	0.26372	0.167358	0.578857
ENSBTAP00000067437.1	ENSBTAG00000002042	NA	NA	NA	NA
ENSBTAP00000067442.1	ENSBTAG00000051287	1.078	0.54252	0.108357	0.265584
ENSBTAP00000067483.1	ENSBTAG00000006543	1.059	0.031863	0.082703	1.496713
ENSBTAP00000067484.1	ENSBTAG00000014490	1.036	0.20434	0.051024	0.689647
ENSBTAP00000067531.1	ENSBTAG00000015090	NA	NA	NA	NA
ENSBTAP00000067544.1	ENSBTAG00000005403	0.911	0.52146	-0.13448	0.282779
ENSBTAP00000067547.1	ENSBTAG00000012797	1.027	0.5836	0.038436	0.233885
ENSBTAP00000067548.1	ENSBTAG00000052525	NA	NA	NA	NA
ENSBTAP00000067566.1	ENSBTAG00000003178	0.997	0.96182	-0.00433	0.016906
ENSBTAP00000067579.1	ENSBTAG00000021067	0.944	0.47822	-0.08314	0.320372
ENSBTAP00000067614.1	ENSBTAG00000052481	0.895	0.46878	-0.16004	0.329031
ENSBTAP00000067615.1	ENSBTAG00000012065	NA	NA	NA	NA
ENSBTAP00000067618.1	ENSBTAG00000000330	0.984	0.8241	-0.02327	0.08402
ENSBTAP00000067657.1	ENSBTAG00000017697	0.996	0.88274	-0.00578	0.054167
ENSBTAP00000067666.1	ENSBTAG00000022927	1.033	0.44446	0.04684	0.352167
ENSBTAP00000067698.1	ENSBTAG00000014178	1.314	0.112216	0.393965	0.949945
ENSBTAP00000067710.1	ENSBTAG00000015116	1.044	0.56226	0.062122	0.250063
ENSBTAP00000067722.1	ENSBTAG00000052382	1.033	0.70722	0.04684	0.150445
ENSBTAP00000067735.1	ENSBTAG00000020605	0.875	0.3981	-0.19265	0.400008
ENSBTAP00000067749.1	ENSBTAG00000039793	1.026	0.136155	0.037031	0.865966
ENSBTAP00000067758.1	ENSBTAG00000053003	NA	NA	NA	NA
ENSBTAP00000067764.1	ENSBTAG00000018255	1.013	0.9033	0.018634	0.044168
ENSBTAP00000067836.1	ENSBTAG00000008077	0.88	0.001179	-0.18442	2.928446
ENSBTAP00000067851.1	ENSBTAG00000001332	1.042	0.70692	0.059355	0.15063
ENSBTAP00000067864.1	ENSBTAG00000012012	1.013	0.8055	0.018634	0.093934
ENSBTAP00000067867.1	ENSBTAG00000006672	0.929	0.26724	-0.10625	0.573099
ENSBTAP00000067874.1	ENSBTAG00000010559	1.01	0.84822	0.014355	0.071491
ENSBTAP00000067878.1	ENSBTAG00000009496	1.208	0.42308	0.27262	0.373578
ENSBTAP00000067900.1	ENSBTAG00000018229	NA	NA	NA	NA
ENSBTAP00000067919.1	ENSBTAG00000021131	0.987	0.71218	-0.01888	0.14741
ENSBTAP00000067948.1	ENSBTAG00000046177	1.029	0.61058	0.041243	0.214257
ENSBTAP00000068000.1	ENSBTAG00000053107	1.131	0.27678	0.177599	0.557865
ENSBTAP00000068009.1	ENSBTAG00000050712	0.977	0.78784	-0.03357	0.103562
ENSBTAP00000068097.1	ENSBTAG00000010577	0.875	0.21246	-0.19265	0.672723
ENSBTAP00000068142.1	ENSBTAG00000005534	0.895	0.26168	-0.16004	0.582229
ENSBTAP00000068153.1	ENSBTAG00000006305	0.96	0.62098	-0.05889	0.206922
ENSBTAP00000068212.1	ENSBTAG00000009906	1.022	0.4666	0.031395	0.331055
ENSBTAP00000068259.1	ENSBTAG00000013235	NA	NA	NA	NA
ENSBTAP00000068277.1	ENSBTAG00000007622	1.042	0.41256	0.059355	0.384513
ENSBTAP00000068283.1	ENSBTAG00000010937	1.17	0.186376	0.226509	0.72961
ENSBTAP00000068343.1	ENSBTAG00000013774	1.099	0.44782	0.136191	0.348897
ENSBTAP00000068372.1	ENSBTAG00000026684	NA	NA	NA	NA
ENSBTAP00000068468.1	ENSBTAG00000002863	1.08	0.48204	0.111031	0.316917
ENSBTAP00000068472.1	ENSBTAG00000003644	NA	NA	NA	NA
ENSBTAP00000068478.1	ENSBTAG00000018897	0.966	0.50664	-0.0499	0.295301
ENSBTAP00000068482.1	ENSBTAG00000018656	0.821	0.48946	-0.28455	0.310283
ENSBTAP00000068498.1	ENSBTAG00000011444	1.256	0.26564	0.328836	0.575707
ENSBTAP00000068503.1	ENSBTAG00000005714	1.137	0.26436	0.185232	0.577804
ENSBTAP00000068509.1	ENSBTAG00000015875	NA	NA	NA	NA
ENSBTAP00000068516.1	ENSBTAG00000021164	1.024	0.8061	0.034216	0.093611
ENSBTAP00000068526.1	ENSBTAG00000001105	0.98	0.82866	-0.02915	0.081624

ENSBTAP00000068545.1	ENSBTAG00000006441	1.102	0.45624	0.140124	0.340807
ENSBTAP00000068572.1	ENSBTAG00000013169	NA	NA	NA	NA
ENSBTAP00000068634.1	ENSBTAG00000021227	1.046	0.69896	0.064883	0.155548
ENSBTAP00000068653.1	ENSBTAG00000000586	0.936	0.62412	-0.09542	0.204732
ENSBTAP00000068672.1	ENSBTAG00000019725	0.998	0.97156	-0.00289	0.01253
ENSBTAP00000068679.1	ENSBTAG00000051298	1.065	0.225	0.090853	0.647817
ENSBTAP00000068694.1	ENSBTAG00000018425	1.027	0.66936	0.038436	0.17434
ENSBTAP00000068698.1	ENSBTAG00000052348	0.879	0.85298	-0.18606	0.069061
ENSBTAP00000068739.1	ENSBTAG00000000607	0.961	0.41814	-0.05739	0.378678
ENSBTAP00000068756.1	ENSBTAG00000050722	1.02	0.32894	0.028569	0.482883
ENSBTAP00000068817.1	ENSBTAG00000016457	0.996	0.89064	-0.00578	0.050298
ENSBTAP00000068855.1	ENSBTAG00000006320	1.241	0.39994	0.311503	0.398005
ENSBTAP00000068884.1	ENSBTAG00000005660	0.991	0.86992	-0.01304	0.060521
ENSBTAP00000068885.1	ENSBTAG00000026320	NA	NA	NA	NA
ENSBTAP00000068917.1	ENSBTAG00000000434	1.206	0.097262	0.27023	1.012057
ENSBTAP00000068950.1	ENSBTAG00000002068	0.98	0.7615	-0.02915	0.11833
ENSBTAP00000068970.1	ENSBTAG00000016481	NA	NA	NA	NA
ENSBTAP00000068972.1	ENSBTAG00000051745	0.958	0.53408	-0.0619	0.272394
ENSBTAP00000069012.1	ENSBTAG00000048504	1	0.9889	0	0.004848
ENSBTAP00000069035.1	ENSBTAG00000013492	0.818	0.155778	-0.28983	0.807494
ENSBTAP00000069089.1	ENSBTAG00000014685	1.248	0.109723	0.319618	0.959702
ENSBTAP00000069104.1	ENSBTAG00000002018	0.953	0.24576	-0.06945	0.609489
ENSBTAP00000069118.1	ENSBTAG00000050667	0.83	0.37454	-0.26882	0.426502
ENSBTAP00000069213.1	ENSBTAG00000022160	1.051	0.68782	0.071763	0.162525
ENSBTAP00000069216.1	ENSBTAG00000033453	1.033	0.77694	0.04684	0.109613
ENSBTAP00000069247.1	ENSBTAG00000003307	1.122	0.42628	0.166073	0.370305
ENSBTAP00000069272.1	ENSBTAG00000007442	NA	NA	NA	NA
ENSBTAP00000069330.1	ENSBTAG00000020701	0.92	0.49168	-0.12029	0.308317
ENSBTAP00000069334.1	ENSBTAG00000013084	0.96	0.52576	-0.05889	0.279212
ENSBTAP00000069350.1	ENSBTAG00000052280	1.064	0.67804	0.089498	0.168745
ENSBTAP00000069356.1	ENSBTAG00000004564	0.965	0.34762	-0.0514	0.458895
ENSBTAP00000069367.1	ENSBTAG00000006270	1.04	0.66404	0.056584	0.177806
ENSBTAP00000069373.1	ENSBTAG00000006429	0.975	0.75116	-0.03653	0.124268
ENSBTAP00000069443.1	ENSBTAG00000023218	0.919	0.20728	-0.12186	0.683443
ENSBTAP00000069472.1	ENSBTAG00000004604	0.955	0.56536	-0.06643	0.247675
ENSBTAP00000069497.1	ENSBTAG00000003067	0.95	0.140985	-0.074	0.850827
ENSBTAP00000069509.1	ENSBTAG00000016924	1.019	0.83978	0.027154	0.075834
ENSBTAP00000069536.1	ENSBTAG00000006907	NA	NA	NA	NA
ENSBTAP00000069581.1	ENSBTAG00000000071	NA	NA	NA	NA
ENSBTAP00000069583.1	ENSBTAG00000050353	0.948	0.51424	-0.07704	0.288834
ENSBTAP00000069589.1	ENSBTAG00000014161	NA	NA	NA	NA
ENSBTAP00000069641.1	ENSBTAG00000011257	NA	NA	NA	NA
ENSBTAP00000069680.1	ENSBTAG00000000404	0.958	0.65058	-0.0619	0.186699
ENSBTAP00000069684.1	ENSBTAG00000010181	1.091	0.4596	0.125651	0.33762
ENSBTAP00000069755.1	ENSBTAG00000012873	NA	NA	NA	NA
ENSBTAP00000069852.1	ENSBTAG00000011125	0.943	0.49664	-0.08467	0.303958
ENSBTAP00000069856.1	ENSBTAG00000016806	0.993	0.46182	-0.01013	0.335527
ENSBTAP00000069890.1	ENSBTAG00000013468	1.028	0.45004	0.03984	0.346749
ENSBTAP00000069944.1	ENSBTAG00000020801	0.965	0.51214	-0.0514	0.290611
ENSBTAP00000069975.1	ENSBTAG00000054580	0.928	0.36482	-0.1078	0.437921
ENSBTAP00000069976.1	ENSBTAG00000015066	1.05	0.61442	0.070389	0.211535
ENSBTAP00000070004.1	ENSBTAG00000021378	1.128	0.39454	0.173767	0.403909
ENSBTAP00000070012.1	ENSBTAG00000015073	1.002	0.9417	0.002883	0.026087
ENSBTAP00000070019.1	ENSBTAG00000049766	0.956	0.56012	-0.06492	0.251719
ENSBTAP00000070033.1	ENSBTAG00000051517	NA	NA	NA	NA
ENSBTAP00000070041.1	ENSBTAG00000050426	0.851	0.084941	-0.23277	1.070883
ENSBTAP00000070078.1	ENSBTAG00000017379	NA	NA	NA	NA
ENSBTAP00000070102.1	ENSBTAG00000007236	1.021	0.75424	0.029983	0.12249

ENSBTAP00000070122.1	ENSBTAG00000014041	1.004	0.98332	0.005759	0.007305
ENSBTAP00000070140.1	ENSBTAG00000021776	1.059	0.6836	0.082703	0.165198
ENSBTAP00000070170.1	ENSBTAG00000038107	NA	NA	NA	NA
ENSBTAP00000070178.1	ENSBTAG00000009667	NA	NA	NA	NA
ENSBTAP00000070302.1	ENSBTAG00000000948	1.049	0.42962	0.069015	0.366916
ENSBTAP00000070314.1	ENSBTAG00000005483	0.875	0.051896	-0.19265	1.284866
ENSBTAP00000070354.1	ENSBTAG00000004230	1.066	0.134259	0.092207	0.872057
ENSBTAP00000070358.1	ENSBTAG00000046155	NA	NA	NA	NA
ENSBTAP00000070382.1	ENSBTAG00000019514	1.138	0.21224	0.186501	0.673173
ENSBTAP00000070446.1	ENSBTAG00000001081	0.928	0.38054	-0.1078	0.4196
ENSBTAP00000070448.1	ENSBTAG00000013060	1.043	0.87098	0.060739	0.059992
ENSBTAP00000070458.1	ENSBTAG00000049516	0.985	0.8146	-0.0218	0.089056
ENSBTAP00000070472.1	ENSBTAG00000005211	0.835	0.39148	-0.26015	0.40729
ENSBTAP00000070573.1	ENSBTAG00000005726	1.007	0.82574	0.010064	0.083157
ENSBTAP00000070639.1	ENSBTAG00000052709	1.051	0.152376	0.071763	0.817083
ENSBTAP00000070677.1	ENSBTAG00000031941	1.073	0.76334	0.10165	0.117282
ENSBTAP00000070700.1	ENSBTAG00000015853	1.028	0.94092	0.03984	0.026447
ENSBTAP00000070732.1	ENSBTAG00000045523	1.017	0.67616	0.02432	0.169951
ENSBTAP00000070814.1	ENSBTAG00000012818	1.131	0.099262	0.177599	1.003217
ENSBTAP00000070878.1	ENSBTAG00000019852	1.066	0.35802	0.092207	0.446093
ENSBTAP00000070883.1	ENSBTAG00000005666	1.096	0.061283	0.132248	1.21266
ENSBTAP00000070935.1	ENSBTAG00000007075	1.108	0.39966	0.147958	0.398309
ENSBTAP00000070967.1	ENSBTAG00000050155	1.112	0.189463	0.153157	0.722476
ENSBTAP00000071019.1	ENSBTAG00000012927	0.916	0.170258	-0.12658	0.768892
ENSBTAP00000071036.1	ENSBTAG00000005027	1.02	0.69474	0.028569	0.158178
ENSBTAP00000071115.1	ENSBTAG00000012471	NA	NA	NA	NA
ENSBTAP00000071202.1	ENSBTAG00000007562	NA	NA	NA	NA
ENSBTAP00000071203.1	ENSBTAG00000010265	1.037	0.49826	0.052416	0.302544
ENSBTAP00000071239.1	ENSBTAG00000010492	1.036	0.5237	0.051024	0.280917
ENSBTAP00000071274.1	ENSBTAG00000051744	1.151	0.007136	0.202888	2.146563
ENSBTAP00000071321.1	ENSBTAG00000007107	NA	NA	NA	NA
ENSBTAP00000071336.1	ENSBTAG00000006487	0.955	0.5579	-0.06643	0.253444
ENSBTAP00000071415.1	ENSBTAG00000005158	0.939	0.063738	-0.0908	1.195602
ENSBTAP00000071420.1	ENSBTAG00000018382	0.923	0.44726	-0.1156	0.34944
ENSBTAP00000071428.1	ENSBTAG00000004240	1.008	0.95296	0.011496	0.020925
ENSBTAP00000071435.1	ENSBTAG00000015690	0.972	0.42176	-0.04097	0.374935
ENSBTAP00000071451.1	ENSBTAG00000010153	1.053	0.66292	0.074505	0.178539
ENSBTAP00000071483.1	ENSBTAG00000015980	0.967	0.92354	-0.04841	0.034544
ENSBTAP00000071492.1	ENSBTAG00000008025	NA	NA	NA	NA
ENSBTAP00000071528.1	ENSBTAG00000014204	NA	NA	NA	NA
ENSBTAP00000071535.1	ENSBTAG00000013937	NA	NA	NA	NA
ENSBTAP00000071585.1	ENSBTAG00000008300	1.021	0.88556	0.029983	0.052782
ENSBTAP00000071596.1	ENSBTAG00000007787	1.093	0.45884	0.128293	0.338339
ENSBTAP00000071617.1	ENSBTAG00000038920	0.964	0.49074	-0.05289	0.309149
ENSBTAP00000071650.1	ENSBTAG00000016399	1.281	0.28908	0.35727	0.538982
ENSBTAP00000071655.1	ENSBTAG00000052473	NA	NA	NA	NA
ENSBTAP00000071723.1	ENSBTAG00000053626	1.009	0.69126	0.012926	0.160359
ENSBTAP00000071742.1	ENSBTAG00000037651	1.27	0.25512	0.344828	0.593255
ENSBTAP00000071789.1	ENSBTAG00000054773	1.183	0.0503	0.24245	1.298432
ENSBTAP00000071794.1	ENSBTAG00000021880	1.203	0.191099	0.266637	0.718742
ENSBTAP00000071809.1	ENSBTAG00000013038	0.946	0.24622	-0.08009	0.608677
ENSBTAP00000071836.1	ENSBTAG00000007330	0.952	0.44858	-0.07097	0.34816
ENSBTAP00000071848.1	ENSBTAG00000008570	1.046	0.121317	0.064883	0.916078
ENSBTAP00000071957.1	ENSBTAG00000006227	1.101	0.51782	0.138814	0.285821
ENSBTAP00000071966.1	ENSBTAG00000017082	NA	NA	NA	NA
ENSBTAP00000071969.1	ENSBTAG00000020927	NA	NA	NA	NA
ENSBTAP00000071983.1	ENSBTAG00000048756	1.167	0.185523	0.222805	0.731602
ENSBTAP00000071995.1	ENSBTAG00000006690	1.127	0.151322	0.172488	0.820098

ENSBTAP00000072040.1	ENSBTAG00000021216	NA	NA	NA	NA
ENSBTAP00000072114.1	ENSBTAG00000001579	1.066	0.142339	0.092207	0.846676
ENSBTAP00000072117.1	ENSBTAG00000054520	1.152	0.177523	0.204141	0.750745
ENSBTAP00000072156.1	ENSBTAG00000009757	0.989	0.93646	-0.01596	0.028511
ENSBTAP00000072286.1	ENSBTAG00000051467	NA	NA	NA	NA
ENSBTAP00000072316.1	ENSBTAG00000008248	1.021	0.60908	0.029983	0.215326
ENSBTAP00000072318.1	ENSBTAG00000002896	NA	NA	NA	NA
ENSBTAP00000072322.1	ENSBTAG00000001027	0.961	0.83114	-0.05739	0.080326
ENSBTAP00000072324.1	ENSBTAG00000006999	0.921	0.15444	-0.11873	0.81124
ENSBTAP00000072381.1	ENSBTAG00000052413	0.946	0.100676	-0.08009	0.997074
ENSBTAP00000072385.1	ENSBTAG00000015205	1.007	0.8318	0.010064	0.079981
ENSBTAP00000072391.1	ENSBTAG00000000421	0.974	0.81812	-0.03801	0.087183
ENSBTAP00000072406.1	ENSBTAG00000021174	NA	NA	NA	NA
ENSBTAP00000072408.1	ENSBTAG00000001776	NA	NA	NA	NA
ENSBTAP00000072419.1	ENSBTAG00000021769	1.055	0.143403	0.077243	0.843442
ENSBTAP00000072451.1	ENSBTAG00000015950	NA	NA	NA	NA
ENSBTAP00000072457.1	ENSBTAG00000004452	0.765	0.323	-0.38647	0.490797
ENSBTAP00000072488.1	ENSBTAG00000020108	1.05	0.62792	0.070389	0.202096
ENSBTAP00000072569.1	ENSBTAG00000004077	0.978	0.52736	-0.03209	0.277893
ENSBTAP00000072687.1	ENSBTAG00000010957	0.87	0.20154	-0.20091	0.695639
ENSBTAP00000072715.1	ENSBTAG00000050621	1.087	0.37242	0.120352	0.428967
ENSBTAP00000072774.1	ENSBTAG00000005349	1.004	0.9769	0.005759	0.01015
ENSBTAP00000072815.1	ENSBTAG00000011885	1.148	0.32586	0.199123	0.486969
ENSBTAP00000072841.1	ENSBTAG00000009915	1.087	0.32948	0.120352	0.482171
ENSBTAP00000072854.1	ENSBTAG00000004850	1.071	0.32952	0.098958	0.482118
ENSBTAP00000072868.1	ENSBTAG00000012881	0.994	0.63542	-0.00868	0.196939
ENSBTAP00000072879.1	ENSBTAG00000010480	NA	NA	NA	NA
ENSBTAP00000072885.1	ENSBTAG00000015327	1.026	0.65528	0.037031	0.183573
ENSBTAP00000072895.1	ENSBTAG00000054952	1.029	0.48006	0.041243	0.318704
ENSBTAP00000072901.1	ENSBTAG00000007952	NA	NA	NA	NA
ENSBTAP00000072932.1	ENSBTAG00000046468	0.982	0.83192	-0.02621	0.079918
ENSBTAP00000072945.1	ENSBTAG00000012113	NA	NA	NA	NA
ENSBTAP00000072974.1	ENSBTAG00000006029	1.1	0.3744	0.137504	0.426664
ENSBTAP00000072998.1	ENSBTAG00000015789	0.965	0.74224	-0.0514	0.129456
ENSBTAP00000073054.1	ENSBTAG00000030669	NA	NA	NA	NA
ENSBTAP00000073075.1	ENSBTAG00000007979	0.944	0.27462	-0.08314	0.561268
ENSBTAP00000073213.1	ENSBTAG00000015988	1.15	0.28528	0.201634	0.544729
ENSBTAP00000073258.1	ENSBTAG00000016523	0.936	0.14856	-0.09542	0.828098
ENSBTAP00000073268.1	ENSBTAG00000002219	NA	NA	NA	NA
ENSBTAP00000073334.1	ENSBTAG00000002117	1.061	0.60194	0.085425	0.220447
ENSBTAP00000073361.1	ENSBTAG00000014387	0.882	0.160118	-0.18115	0.79556
ENSBTAP00000073363.1	ENSBTAG00000002487	NA	NA	NA	NA
ENSBTAP00000073384.1	ENSBTAG00000008013	1.242	0.256	0.312665	0.59176
ENSBTAP00000073429.1	ENSBTAG00000002485	NA	NA	NA	NA
ENSBTAP00000073470.1	ENSBTAG00000013651	NA	NA	NA	NA
ENSBTAP00000073524.1	ENSBTAG00000008202	1.018	0.80202	0.025738	0.095815
ENSBTAP00000073589.1	ENSBTAG00000000843	0.927	0.29206	-0.10936	0.534528
ENSBTAP00000073602.1	ENSBTAG00000015986	NA	NA	NA	NA
ENSBTAP00000073676.1	ENSBTAG00000005426	0.991	0.82106	-0.01304	0.085625
ENSBTAP00000073706.1	ENSBTAG00000019516	1.02	0.65812	0.028569	0.181695
ENSBTAP00000073749.1	ENSBTAG00000013414	0.93	0.48356	-0.1047	0.31555
ENSBTAP00000073837.1	ENSBTAG00000044126	1.126	0.30234	0.171207	0.519504
ENSBTAP00000073891.1	ENSBTAG00000050514	0.989	0.80068	-0.01596	0.096541
ENSBTAP00000073907.1	ENSBTAG00000046725	0.894	0.25344	-0.16165	0.596125
ENSBTAP00000074005.1	ENSBTAG00000017765	NA	NA	NA	NA
ENSBTAP00000074045.1	ENSBTAG00000017368	1.3	0.22674	0.378512	0.644472
ENSBTAP00000074076.1	ENSBTAG00000034373	0.95	0.21462	-0.074	0.66833
ENSBTAP00000074112.1	ENSBTAG00000014598	1.03	0.57248	0.042644	0.24224

ENSBTAP00000074122.1	ENSBTAG00000018373	1.264	0.12796	0.337996	0.892926
ENSBTAP00000074135.1	ENSBTAG00000014904	1.015	0.87562	0.02148	0.057684
ENSBTAP00000074167.1	ENSBTAG00000003422	NA	NA	NA	NA
ENSBTAP00000074266.1	ENSBTAG00000002475	0.993	0.93786	-0.01013	0.027862
ENSBTAP00000074281.1	ENSBTAG00000001513	NA	NA	NA	NA
ENSBTAP00000074298.1	ENSBTAG00000011274	0.817	0.108658	-0.29159	0.963938
ENSBTAP00000074299.1	ENSBTAG00000052700	1.056	0.33786	0.07861	0.471263
ENSBTAP00000074345.1	ENSBTAG00000050071	1.118	0.31514	0.16092	0.501496
ENSBTAP00000074366.1	ENSBTAG00000034867	0.83	0.06402	-0.26882	1.193684
ENSBTAP00000074368.1	ENSBTAG00000054199	NA	NA	NA	NA
ENSBTAP00000074375.1	ENSBTAG00000004992	1.023	0.73512	0.032806	0.133642
ENSBTAP00000074391.1	ENSBTAG00000017183	1.014	0.70526	0.020058	0.151651
ENSBTAP00000074423.1	ENSBTAG00000054051	0.988	0.57346	-0.01742	0.241497
ENSBTAP00000074427.1	ENSBTAG00000019794	NA	NA	NA	NA
ENSBTAP00000074431.1	ENSBTAG00000017715	NA	NA	NA	NA
ENSBTAP00000074458.1	ENSBTAG00000012756	1.037	0.6881	0.052416	0.162348
ENSBTAP00000074486.1	ENSBTAG00000012447	1.022	0.32122	0.031395	0.493197
ENSBTAP00000074521.1	ENSBTAG00000020747	0.983	0.8592	-0.02474	0.065906
ENSBTAP00000074529.1	ENSBTAG00000048478	0.807	0.40924	-0.30936	0.388022
ENSBTAP00000074598.1	ENSBTAG00000047637	1.08	0.127761	0.111031	0.893602

Supplementary Table 2. Transcript quantitation

Transcription.ID	log2FC	Pvalue	log10p
ENSBTAG00000000005	-0.108218	0.790068	0.1023355
ENSBTAG00000000009	NA	NA	NA
ENSBTAG00000000010	0.1684901	0.5160809	0.2872822
ENSBTAG00000000011	NA	NA	NA
ENSBTAG00000000012	0.1273983	0.6548675	0.1838466
ENSBTAG00000000013	0.3961374	0.1379048	0.8604205
ENSBTAG00000000014	0.2217257	0.3727386	0.4285957
ENSBTAG00000000015	NA	NA	NA
ENSBTAG00000000019	-0.488303	0.0932712	1.0302526
ENSBTAG00000000020	NA	NA	NA
ENSBTAG00000000021	-0.149016	0.6169583	0.2097442
ENSBTAG00000000022	-0.170874	0.6195086	0.2079527
ENSBTAG00000000023	0.1027384	0.7538671	0.1227052
ENSBTAG00000000024	-0.299663	0.2281911	0.6417014
ENSBTAG00000000025	0.2403384	0.4584923	0.3386679
ENSBTAG00000000026	0.4328263	0.2544212	0.5944468
ENSBTAG00000000029	NA	NA	NA
ENSBTAG00000000030	NA	NA	NA
ENSBTAG00000000031	NA	NA	NA
ENSBTAG00000000032	NA	NA	NA
ENSBTAG00000000033	0.1507588	0.7441186	0.1283579
ENSBTAG00000000037	-0.046671	0.8829285	0.0540745
ENSBTAG00000000039	0.1980286	0.4785658	0.3200583
ENSBTAG00000000040	NA	NA	NA
ENSBTAG00000000042	-0.451697	0.339349	0.4693534
ENSBTAG00000000044	-0.140506	0.6174509	0.2093976
ENSBTAG00000000046	0.1292659	0.7719678	0.1124008
ENSBTAG00000000049	0.2859895	0.4364179	0.3600974
ENSBTAG00000000050	-0.249816	0.4320809	0.364435
ENSBTAG00000000052	0.2408583	0.5544192	0.2561617
ENSBTAG00000000053	0.0007554	0.9979386	0.0008962
ENSBTAG00000000054	0.5252523	0.143901	0.8419362
ENSBTAG00000000056	0.4380466	0.2924032	0.5340179
ENSBTAG00000000057	-0.068074	0.8695118	0.0607245
ENSBTAG00000000061	0.0593324	0.8356849	0.0779574
ENSBTAG00000000062	-0.07781	0.8734204	0.0587767
ENSBTAG00000000064	-0.002475	0.9965389	0.0015057
ENSBTAG00000000065	-0.358723	0.1688381	0.7725295
ENSBTAG00000000066	NA	NA	NA
ENSBTAG00000000067	NA	NA	NA
ENSBTAG00000000070	NA	NA	NA
ENSBTAG00000000071	-1.572034	0.0096795	2.0141476
ENSBTAG00000000072	-0.028917	0.9065852	0.0425914
ENSBTAG00000000073	0.2939169	0.3475141	0.4590276
ENSBTAG00000000074	0.2860861	0.5850671	0.2327943
ENSBTAG00000000076	0.4199293	0.292667	0.5336262
ENSBTAG00000000077	0.1585817	0.5357205	0.2710617
ENSBTAG00000000078	0.3644061	0.3285035	0.48346
ENSBTAG00000000079	-0.00488	1	0
ENSBTAG00000000080	0.5921797	0.0494566	1.3057761
ENSBTAG00000000081	-0.08026	0.7675043	0.1149192
ENSBTAG00000000082	NA	NA	NA
ENSBTAG00000000084	0.1612991	0.5679377	0.2456993
ENSBTAG00000000085	NA	NA	NA
ENSBTAG00000000087	-0.071667	0.7773781	0.1093677
ENSBTAG00000000088	0.1203102	0.7100914	0.1486857

ENSBTAG00000000090	0.1853966	0.5917351	0.2278726
ENSBTAG00000000091	-0.075878	0.7756079	0.1103577
ENSBTAG00000000092	0.7133082	0.030052	1.5221259
ENSBTAG00000000094	-0.319062	0.2151924	0.6671732
ENSBTAG00000000095	NA	NA	NA
ENSBTAG00000000097	0.0218794	0.9436018	0.0252112
ENSBTAG00000000098	0.353811	0.2191608	0.659237
ENSBTAG00000000099	0.5946636	0.0420871	1.3758506
ENSBTAG00000000102	NA	NA	NA
ENSBTAG00000000103	-0.064035	0.8279155	0.082014
ENSBTAG00000000105	-0.216292	0.3932691	0.4053102
ENSBTAG00000000106	1.6307213	0.0016812	2.774368
ENSBTAG00000000108	0.0622531	0.8310499	0.0803729
ENSBTAG00000000109	NA	NA	NA
ENSBTAG00000000111	0.4413805	0.2358837	0.6273021
ENSBTAG00000000113	0.0508306	0.8526576	0.0692253
ENSBTAG00000000115	0.0590962	0.826913	0.0825402
ENSBTAG00000000120	NA	NA	NA
ENSBTAG00000000123	NA	NA	NA
ENSBTAG00000000124	NA	NA	NA
ENSBTAG00000000125	0.4861493	0.0723339	1.1406583
ENSBTAG00000000128	NA	NA	NA
ENSBTAG00000000130	NA	NA	NA
ENSBTAG00000000131	0.1807855	0.5658615	0.2472899
ENSBTAG00000000132	0.036707	0.8880481	0.0515635
ENSBTAG00000000133	NA	NA	NA
ENSBTAG00000000134	0.4221985	0.2065275	0.6850221
ENSBTAG00000000137	0.1490342	0.5799387	0.2366179
ENSBTAG00000000138	NA	NA	NA
ENSBTAG00000000139	-0.480782	0.1178804	0.9285583
ENSBTAG00000000140	0.1188598	0.7793624	0.1082605
ENSBTAG00000000141	-0.09831	0.6926801	0.1594673
ENSBTAG00000000144	NA	NA	NA
ENSBTAG00000000146	-0.086829	0.7658611	0.11585
ENSBTAG00000000147	-0.434116	0.1252087	0.9023655
ENSBTAG00000000149	0.0982563	0.7045955	0.1520601
ENSBTAG00000000152	-0.091658	0.7181412	0.1437902
ENSBTAG00000000153	NA	NA	NA
ENSBTAG00000000154	-0.158123	0.60587	0.2176206
ENSBTAG00000000156	-0.47763	0.0596694	1.2242486
ENSBTAG00000000157	-1.082151	0.0230748	1.6368615
ENSBTAG00000000160	0.0337507	0.9478678	0.0232522
ENSBTAG00000000161	NA	NA	NA
ENSBTAG00000000162	-0.434193	0.1035186	0.9849817
ENSBTAG00000000163	-0.413919	0.2752008	0.5603504
ENSBTAG00000000164	NA	NA	NA
ENSBTAG00000000169	NA	NA	NA
ENSBTAG00000000170	NA	NA	NA
ENSBTAG00000000172	-0.186214	0.4515133	0.3453295
ENSBTAG00000000175	0.0769065	0.7780517	0.1089916
ENSBTAG00000000176	-0.006462	0.9801101	0.0087252
ENSBTAG00000000177	NA	NA	NA
ENSBTAG00000000179	NA	NA	NA
ENSBTAG00000000181	NA	NA	NA
ENSBTAG00000000182	0.1443303	0.6634862	0.1781681
ENSBTAG00000000183	NA	NA	NA
ENSBTAG00000000184	0.4529747	0.1463998	0.8344597
ENSBTAG00000000185	NA	NA	NA

ENSBTAG00000000186	0.0934375	0.7877317	0.1036217
ENSBTAG00000000188	-0.649363	0.0973117	1.0118348
ENSBTAG00000000189	NA	NA	NA
ENSBTAG00000000191	0.2940505	0.2430064	0.6143823
ENSBTAG00000000195	0.0253027	0.9413629	0.0262429
ENSBTAG00000000197	0.4189157	0.2411814	0.6176563
ENSBTAG00000000198	NA	NA	NA
ENSBTAG00000000199	-0.664487	0.0085055	2.0703007
ENSBTAG00000000201	0.118503	0.6354722	0.1969034
ENSBTAG00000000202	0.0255609	0.9528168	0.0209906
ENSBTAG00000000203	-0.229196	0.3765778	0.4241452
ENSBTAG00000000204	0.3687466	0.4170381	0.3798243
ENSBTAG00000000205	0.2809768	0.3689155	0.4330731
ENSBTAG00000000207	0.2302951	0.5310026	0.2749034
ENSBTAG00000000210	0.1656981	0.539454	0.2680456
ENSBTAG00000000211	NA	NA	NA
ENSBTAG00000000212	0.1510055	0.5895897	0.2294501
ENSBTAG00000000213	0.0770099	0.8168068	0.0878807
ENSBTAG00000000215	0.0804191	0.7438728	0.1285013
ENSBTAG00000000218	0.0776284	0.8210833	0.0856128
ENSBTAG00000000219	NA	NA	NA
ENSBTAG00000000220	NA	NA	NA
ENSBTAG00000000221	-0.092572	0.792739	0.1008698
ENSBTAG00000000222	-0.085872	0.7403269	0.1305765
ENSBTAG00000000223	-0.198312	0.4578341	0.3392919
ENSBTAG00000000224	-0.442199	0.264212	0.5780475
ENSBTAG00000000225	-0.247782	0.3972429	0.4009439
ENSBTAG00000000229	NA	NA	NA
ENSBTAG00000000231	-0.33322	0.1963351	0.707002
ENSBTAG00000000232	NA	NA	NA
ENSBTAG00000000233	NA	NA	NA
ENSBTAG00000000236	-0.164908	0.6183059	0.2087966
ENSBTAG00000000240	-0.527055	0.1803443	0.7438976
ENSBTAG00000000241	-0.087356	0.7234697	0.1405797
ENSBTAG00000000243	0.4236541	0.3625469	0.4406358
ENSBTAG00000000244	NA	NA	NA
ENSBTAG00000000245	-0.362892	0.4292968	0.3672424
ENSBTAG00000000246	0.4454495	0.192944	0.7145688
ENSBTAG00000000250	0.6179827	0.0239021	1.6215636
ENSBTAG00000000251	0.3906807	0.1307275	0.8836329
ENSBTAG00000000252	-0.025896	0.9280894	0.0324102
ENSBTAG00000000253	0.1452017	0.76992	0.1135544
ENSBTAG00000000256	-0.061025	0.8628452	0.0640671
ENSBTAG00000000257	NA	NA	NA
ENSBTAG00000000258	0.7479838	0.2856915	0.5441027
ENSBTAG00000000260	-0.348535	0.2728792	0.5640296
ENSBTAG00000000261	NA	NA	NA
ENSBTAG00000000264	NA	NA	NA
ENSBTAG00000000265	NA	NA	NA
ENSBTAG00000000266	-0.139641	0.5883364	0.2303743
ENSBTAG00000000267	0.1512952	0.6234914	0.2051695
ENSBTAG00000000269	-0.480256	0.1180477	0.9279424
ENSBTAG00000000271	-0.216979	0.5707507	0.2435536
ENSBTAG00000000273	NA	NA	NA
ENSBTAG00000000274	0.0425563	0.8665123	0.0622253
ENSBTAG00000000275	0.2396954	0.4512182	0.3456134
ENSBTAG00000000277	NA	NA	NA
ENSBTAG00000000278	NA	NA	NA

ENSBTAG00000000279	NA	NA	NA
ENSBTAG00000000280	NA	NA	NA
ENSBTAG00000000281	NA	NA	NA
ENSBTAG00000000283	0.207873	0.5749615	0.2403612
ENSBTAG00000000284	NA	NA	NA
ENSBTAG00000000285	-0.022693	0.9374319	0.0280603
ENSBTAG00000000286	-0.100109	0.7240517	0.1402304
ENSBTAG00000000287	0.296686	0.2996748	0.5233498
ENSBTAG00000000288	-0.141188	0.5668001	0.2465701
ENSBTAG00000000289	-0.063498	0.8120654	0.090409
ENSBTAG00000000290	-0.026997	0.9112967	0.0403402
ENSBTAG00000000291	-0.196644	0.4775361	0.3209938
ENSBTAG00000000292	0.3158557	0.3989359	0.3990969
ENSBTAG00000000295	0.0818352	0.7810958	0.1072957
ENSBTAG00000000296	NA	NA	NA
ENSBTAG00000000297	-0.06974	0.8238404	0.0841569
ENSBTAG00000000300	0.2831123	0.6371734	0.1957424
ENSBTAG00000000301	NA	NA	NA
ENSBTAG00000000305	-0.065618	0.8081099	0.0925296
ENSBTAG00000000306	-0.250869	0.5542881	0.2562645
ENSBTAG00000000308	-0.096346	0.6964577	0.1571053
ENSBTAG00000000309	NA	NA	NA
ENSBTAG00000000310	0.449645	0.1040192	0.9828865
ENSBTAG00000000312	0.122675	0.7461675	0.1271637
ENSBTAG00000000313	-0.079586	0.8000507	0.0968825
ENSBTAG00000000315	-0.2246	0.4164941	0.3803911
ENSBTAG00000000317	NA	NA	NA
ENSBTAG00000000320	-0.058417	0.8373471	0.0770945
ENSBTAG00000000321	0.3311314	0.3659191	0.4366149
ENSBTAG00000000322	-0.240216	0.357448	0.4467872
ENSBTAG00000000326	NA	NA	NA
ENSBTAG00000000328	0.540922	0.0725821	1.1391707
ENSBTAG00000000329	0.0651633	0.8649723	0.0629978
ENSBTAG00000000330	0.2157632	0.4875702	0.3119629
ENSBTAG00000000332	-0.762177	0.0441225	1.3553399
ENSBTAG00000000333	0.1475647	0.7812365	0.1072175
ENSBTAG00000000336	NA	NA	NA
ENSBTAG00000000340	0.1217203	0.6729635	0.1720085
ENSBTAG00000000341	-0.050089	0.8845197	0.0532925
ENSBTAG00000000342	0.5333384	0.1266823	0.8972841
ENSBTAG00000000343	-0.148039	0.6528506	0.1851862
ENSBTAG00000000345	NA	NA	NA
ENSBTAG00000000347	-0.116932	0.8056474	0.093855
ENSBTAG00000000355	0.6759337	0.0795142	1.0995554
ENSBTAG00000000356	0.3820092	0.341977	0.4660032
ENSBTAG00000000357	1.1722303	0.0141168	1.8502628
ENSBTAG00000000359	-0.260177	0.344096	0.4633204
ENSBTAG00000000362	-0.115055	0.6636362	0.1780699
ENSBTAG00000000363	-0.258609	0.2932742	0.5327262
ENSBTAG00000000365	-0.284368	0.3389655	0.4698445
ENSBTAG00000000369	0.3546459	0.1771917	0.7515566
ENSBTAG00000000371	-0.11195	0.6543368	0.1841986
ENSBTAG00000000372	0.4384939	0.2792819	0.5539572
ENSBTAG00000000374	NA	NA	NA
ENSBTAG00000000375	0.1290308	0.6185062	0.2086559
ENSBTAG00000000377	0.0664587	0.8902858	0.0504705
ENSBTAG00000000379	0.0040985	0.9914836	0.0037145
ENSBTAG00000000380	-0.740218	0.0969301	1.0135411

ENSBTAG00000000381	NA	NA	NA
ENSBTAG00000000382	0.0267787	0.934731	0.0293133
ENSBTAG00000000385	-0.648156	0.0127913	1.893086
ENSBTAG00000000387	-0.264182	0.5972371	0.2238532
ENSBTAG00000000389	-0.053817	0.8807366	0.055154
ENSBTAG00000000390	0.2610993	0.4511184	0.3457095
ENSBTAG00000000392	-0.477444	0.3876176	0.4115965
ENSBTAG00000000393	1.3545804	0.007508	2.1244748
ENSBTAG00000000394	-0.109151	0.7649603	0.1163611
ENSBTAG00000000395	-0.204252	0.5127389	0.2901037
ENSBTAG00000000396	0.3944812	0.3409439	0.4673171
ENSBTAG00000000399	NA	NA	NA
ENSBTAG00000000400	NA	NA	NA
ENSBTAG00000000401	NA	NA	NA
ENSBTAG00000000404	-0.160393	0.5658772	0.2472778
ENSBTAG00000000405	0.1719106	0.6147592	0.211295
ENSBTAG00000000406	0.1687935	0.6979238	0.156192
ENSBTAG00000000409	0.0143719	0.9590911	0.0181401
ENSBTAG00000000411	0.1846584	0.5309694	0.2749305
ENSBTAG00000000413	NA	NA	NA
ENSBTAG00000000415	-0.079545	0.76538	0.1161229
ENSBTAG00000000417	-0.051591	0.8443414	0.0734819
ENSBTAG00000000418	NA	NA	NA
ENSBTAG00000000419	NA	NA	NA
ENSBTAG00000000421	-0.297959	0.255871	0.591979
ENSBTAG00000000422	-0.522542	0.2057196	0.6867244
ENSBTAG00000000423	-0.145098	0.6758683	0.1701379
ENSBTAG00000000425	-0.341591	0.2639714	0.5784431
ENSBTAG00000000428	0.2350313	0.4905743	0.3092952
ENSBTAG00000000429	0.2341766	0.380117	0.4200827
ENSBTAG00000000431	-0.182838	0.6821171	0.166141
ENSBTAG00000000432	0.0601843	0.9106725	0.0406378
ENSBTAG00000000434	0.7810766	0.0060003	2.2218247
ENSBTAG00000000435	-0.444121	0.1765263	0.7531905
ENSBTAG00000000436	-0.610228	0.1839783	0.7352334
ENSBTAG00000000437	NA	NA	NA
ENSBTAG00000000439	0.1097148	0.6955828	0.1576512
ENSBTAG00000000440	-0.300402	0.2545258	0.5942682
ENSBTAG00000000441	0.2267364	0.5719164	0.2426674
ENSBTAG00000000442	-0.139566	0.7972435	0.098409
ENSBTAG00000000445	NA	NA	NA
ENSBTAG00000000446	-0.116328	0.7372992	0.1323562
ENSBTAG00000000447	-0.148183	0.6651616	0.1770728
ENSBTAG00000000448	-0.823726	0.0834701	1.0784693
ENSBTAG00000000451	-0.169258	0.5049484	0.296753
ENSBTAG00000000454	-0.59578	0.2258202	0.6462372
ENSBTAG00000000455	-0.163712	0.5847576	0.2330241
ENSBTAG00000000456	NA	NA	NA
ENSBTAG00000000457	NA	NA	NA
ENSBTAG00000000458	0.2322948	0.346652	0.4601063
ENSBTAG00000000459	0.5236978	0.1347164	0.8705795
ENSBTAG00000000460	-0.198781	0.4658005	0.3318001
ENSBTAG00000000462	0.1584182	0.7044475	0.1521514
ENSBTAG00000000469	0.0341422	0.9033271	0.044155
ENSBTAG00000000470	NA	NA	NA
ENSBTAG00000000472	0.1681455	0.5620351	0.2502366
ENSBTAG00000000473	0.6749389	0.076689	1.1152671
ENSBTAG00000000475	-0.082395	0.7502278	0.1248068

ENSBTAG00000000476	NA	NA	NA
ENSBTAG00000000477	1.3734475	0.0254789	1.5938185
ENSBTAG00000000478	0.0185186	0.9631698	0.0162971
ENSBTAG00000000480	0.0367046	0.8903532	0.0504377
ENSBTAG00000000483	0.0993104	0.7902979	0.1022092
ENSBTAG00000000484	-0.818302	0.0235202	1.6285584
ENSBTAG00000000489	NA	NA	NA
ENSBTAG00000000490	0.0553141	0.917902	0.0372037
ENSBTAG00000000492	-0.186399	0.4640532	0.3334322
ENSBTAG00000000494	0.0265528	0.9261682	0.0333102
ENSBTAG00000000495	0.2857403	0.2720393	0.5653684
ENSBTAG00000000496	NA	NA	NA
ENSBTAG00000000497	-0.183039	0.5896646	0.229395
ENSBTAG00000000500	0.3164624	0.5073479	0.2946941
ENSBTAG00000000501	0.2181354	0.6096139	0.2149451
ENSBTAG00000000502	NA	NA	NA
ENSBTAG00000000504	0.1257789	0.6407455	0.1933144
ENSBTAG00000000505	-0.166997	0.5354175	0.2713074
ENSBTAG00000000507	0.1743523	0.5697474	0.2443177
ENSBTAG00000000510	0.7035964	0.3430556	0.4646355
ENSBTAG00000000511	-0.255533	0.3563922	0.4480718
ENSBTAG00000000512	-0.450417	0.1005801	0.9974879
ENSBTAG00000000516	NA	NA	NA
ENSBTAG00000000517	NA	NA	NA
ENSBTAG00000000520	NA	NA	NA
ENSBTAG00000000521	0.2594809	0.3562738	0.4482162
ENSBTAG00000000522	NA	NA	NA
ENSBTAG00000000524	0.4046711	0.3480187	0.4583974
ENSBTAG00000000526	-0.254017	0.3173576	0.4984511
ENSBTAG00000000527	-0.677449	0.0807158	1.0930413
ENSBTAG00000000528	0.1045974	0.7902343	0.1022441
ENSBTAG00000000529	-1.273874	0.0001205	3.9190742
ENSBTAG00000000531	NA	NA	NA
ENSBTAG00000000532	0.461352	0.2418322	0.6164859
ENSBTAG00000000533	NA	NA	NA
ENSBTAG00000000535	NA	NA	NA
ENSBTAG00000000536	0.1659826	0.6070319	0.2167885
ENSBTAG00000000539	-0.228563	0.369942	0.4318663
ENSBTAG00000000540	NA	NA	NA
ENSBTAG00000000542	-0.115154	0.682476	0.1659126
ENSBTAG00000000545	-0.203146	0.4414247	0.3551434
ENSBTAG00000000546	-0.73531	0.011571	1.9366278
ENSBTAG00000000548	-0.166519	0.5633255	0.2492406
ENSBTAG00000000550	0.2244497	0.3843034	0.4153258
ENSBTAG00000000551	-0.082692	0.7401434	0.1306841
ENSBTAG00000000552	NA	NA	NA
ENSBTAG00000000555	0.2163623	0.4142702	0.3827163
ENSBTAG00000000559	-0.43512	0.3681802	0.4339395
ENSBTAG00000000560	NA	NA	NA
ENSBTAG00000000561	0.3257586	0.4446622	0.3519698
ENSBTAG00000000562	-0.140732	0.6082068	0.2159488
ENSBTAG00000000563	0.6226637	0.1292473	0.8885786
ENSBTAG00000000564	NA	NA	NA
ENSBTAG00000000565	0.4595165	0.3939292	0.4045818
ENSBTAG00000000566	0.435321	0.2866527	0.542644
ENSBTAG00000000568	NA	NA	NA
ENSBTAG00000000569	0.4048294	0.1989378	0.7012827
ENSBTAG00000000570	-0.785947	0.0075147	2.1240866

ENSBTAG00000000571	0.5166415	0.1984926	0.7022556
ENSBTAG00000000573	NA	NA	NA
ENSBTAG00000000575	0.1521734	0.7610401	0.1185924
ENSBTAG00000000576	NA	NA	NA
ENSBTAG00000000578	-0.04404	0.8730656	0.0589531
ENSBTAG00000000579	-0.200909	0.4600064	0.3372362
ENSBTAG00000000580	NA	NA	NA
ENSBTAG00000000581	0.3747211	0.1984132	0.7024295
ENSBTAG00000000582	NA	NA	NA
ENSBTAG00000000584	NA	NA	NA
ENSBTAG00000000585	NA	NA	NA
ENSBTAG00000000586	0.2322233	0.4093173	0.3879399
ENSBTAG00000000588	0.3382249	0.4697255	0.3281558
ENSBTAG00000000589	NA	NA	NA
ENSBTAG00000000590	NA	NA	NA
ENSBTAG00000000593	0.5191808	0.2926902	0.5335919
ENSBTAG00000000595	NA	NA	NA
ENSBTAG00000000598	0.1109745	0.6620007	0.1791416
ENSBTAG00000000599	-0.074354	0.7656469	0.1159715
ENSBTAG00000000601	0.8794048	0.164679	0.7833617
ENSBTAG00000000602	0.2042764	0.4321973	0.364318
ENSBTAG00000000603	0.5423408	0.1050597	0.978564
ENSBTAG00000000604	-0.118533	0.8529968	0.0690526
ENSBTAG00000000605	0.0608675	0.8287507	0.0815761
ENSBTAG00000000606	NA	NA	NA
ENSBTAG00000000607	-0.063333	0.8055841	0.0938891
ENSBTAG00000000608	0.1573408	0.5614627	0.2506791
ENSBTAG00000000613	0.0556441	0.8485039	0.0713462
ENSBTAG00000000616	0.2815369	0.5015729	0.2996659
ENSBTAG00000000619	NA	NA	NA
ENSBTAG00000000620	NA	NA	NA
ENSBTAG00000000621	0.1144877	0.8195664	0.0864159
ENSBTAG00000000622	0.0911688	0.7266017	0.1387036
ENSBTAG00000000623	NA	NA	NA
ENSBTAG00000000625	-0.955872	0.0465366	1.3322053
ENSBTAG00000000626	NA	NA	NA
ENSBTAG00000000628	NA	NA	NA
ENSBTAG00000000629	-0.333821	0.4418492	0.3547259
ENSBTAG00000000630	-0.028543	0.9076944	0.0420604
ENSBTAG00000000632	-0.727848	0.0544261	1.2641925
ENSBTAG00000000634	NA	NA	NA
ENSBTAG00000000638	NA	NA	NA
ENSBTAG00000000639	2.1874185	0.0028704	2.5420502
ENSBTAG00000000640	0.157068	0.6036223	0.2192347
ENSBTAG00000000641	-0.385904	0.445784	0.3508755
ENSBTAG00000000642	0.340223	0.2047141	0.6888522
ENSBTAG00000000644	NA	NA	NA
ENSBTAG00000000646	0.0092762	0.9773292	0.0099591
ENSBTAG00000000647	0.241674	0.3429385	0.4647838
ENSBTAG00000000648	0.7556764	0.0151797	1.8187372
ENSBTAG00000000650	0.0557676	0.8478214	0.0716956
ENSBTAG00000000653	0.222619	0.5131564	0.2897502
ENSBTAG00000000654	NA	NA	NA
ENSBTAG00000000655	0.1930202	0.5652806	0.2477359
ENSBTAG00000000656	-0.161024	0.6581375	0.1816833
ENSBTAG00000000658	0.4857928	0.2584654	0.5875975
ENSBTAG00000000660	NA	NA	NA
ENSBTAG00000000662	0.0874001	0.7648376	0.1164308

ENSBTAG00000000664	NA	NA	NA
ENSBTAG00000000666	-0.124322	0.6671756	0.1757599
ENSBTAG00000000667	0.3853539	0.4152801	0.3816589
ENSBTAG00000000668	-0.137959	0.6555027	0.1834255
ENSBTAG00000000670	0.0041405	0.9940335	0.002599
ENSBTAG00000000671	-0.222068	0.5995872	0.2221476
ENSBTAG00000000672	0.3963859	0.3280672	0.4840372
ENSBTAG00000000675	0.4309016	0.1697869	0.7700958
ENSBTAG00000000676	0.3528426	0.19436	0.7113931
ENSBTAG00000000678	-0.253942	0.3101629	0.5084101
ENSBTAG00000000679	-0.042103	0.9150678	0.0385467
ENSBTAG00000000682	-0.190307	0.4715529	0.3264696
ENSBTAG00000000683	0.2580538	0.328716	0.4831792
ENSBTAG00000000684	-0.190099	0.4430779	0.3535199
ENSBTAG00000000686	NA	NA	NA
ENSBTAG00000000687	-0.020639	0.9505247	0.0220366
ENSBTAG00000000693	-0.093725	0.7091074	0.149288
ENSBTAG00000000694	-0.306486	0.2603837	0.5843862
ENSBTAG00000000695	-0.193696	0.4589	0.3382819
ENSBTAG00000000696	-0.146875	0.5649073	0.2480228
ENSBTAG00000000697	0.1076139	0.6975229	0.1564416
ENSBTAG00000000698	0.06962	0.8198279	0.0862773
ENSBTAG00000000699	-1.205349	0.1773008	0.7512892
ENSBTAG00000000700	0.5086952	0.1783621	0.7486975
ENSBTAG00000000703	NA	NA	NA
ENSBTAG00000000704	0.0951097	0.8046131	0.0944129
ENSBTAG00000000705	0.3697025	0.1738411	0.7598475
ENSBTAG00000000706	-0.059602	0.8296312	0.0811149
ENSBTAG00000000707	NA	NA	NA
ENSBTAG00000000710	0.2195518	0.6030392	0.2196544
ENSBTAG00000000711	0.057771	0.8557134	0.0676717
ENSBTAG00000000712	-0.108644	0.7241909	0.140147
ENSBTAG00000000713	-0.10493	0.7772152	0.1094587
ENSBTAG00000000715	NA	NA	NA
ENSBTAG00000000717	NA	NA	NA
ENSBTAG00000000718	NA	NA	NA
ENSBTAG00000000719	NA	NA	NA
ENSBTAG00000000720	NA	NA	NA
ENSBTAG00000000721	0.1227372	0.6974842	0.1564656
ENSBTAG00000000725	0.0980103	0.8169702	0.0877938
ENSBTAG00000000726	NA	NA	NA
ENSBTAG00000000727	0.0624617	0.8580956	0.0664643
ENSBTAG00000000728	NA	NA	NA
ENSBTAG00000000730	NA	NA	NA
ENSBTAG00000000731	-0.248368	0.4196349	0.3771284
ENSBTAG00000000735	NA	NA	NA
ENSBTAG00000000736	-0.123427	0.8035071	0.0950103
ENSBTAG00000000737	-0.057505	0.8830362	0.0540215
ENSBTAG00000000738	-0.263181	0.5435491	0.2647612
ENSBTAG00000000742	0.1713084	0.5518493	0.2581795
ENSBTAG00000000743	NA	NA	NA
ENSBTAG00000000744	-0.136782	0.6007263	0.2213234
ENSBTAG00000000745	0.6691778	0.0302345	1.5194973
ENSBTAG00000000746	-0.47367	0.0625221	1.2039662
ENSBTAG00000000748	-0.22121	0.6312961	0.1997669
ENSBTAG00000000749	NA	NA	NA
ENSBTAG00000000750	0.390887	0.1912197	0.7184674
ENSBTAG00000000751	NA	NA	NA

ENSBTAG00000000752	NA	NA	NA
ENSBTAG00000000753	-0.163782	0.6676394	0.175458
ENSBTAG00000000754	-0.070312	0.7778445	0.1091072
ENSBTAG00000000755	NA	NA	NA
ENSBTAG00000000758	0.0502481	0.8942204	0.0485554
ENSBTAG00000000759	0.2452346	0.3439842	0.4634615
ENSBTAG00000000764	0.0997777	0.8168664	0.087849
ENSBTAG00000000765	NA	NA	NA
ENSBTAG00000000767	0.1683993	0.6941963	0.1585177
ENSBTAG00000000770	-0.26848	0.2968964	0.527395
ENSBTAG00000000771	0.0848276	0.7500012	0.1249381
ENSBTAG00000000773	-0.017259	0.9589327	0.0182119
ENSBTAG00000000778	0.0112115	0.9647083	0.015604
ENSBTAG00000000781	0.6194157	0.0610386	1.2143955
ENSBTAG00000000782	0.7529128	0.004901	2.3097139
ENSBTAG00000000784	0.1849676	0.5393878	0.2680989
ENSBTAG00000000785	0.6385066	0.1880403	0.7257492
ENSBTAG00000000786	0.1717781	0.6336527	0.1981487
ENSBTAG00000000789	-0.072054	0.8003002	0.0967471
ENSBTAG00000000791	NA	NA	NA
ENSBTAG00000000792	NA	NA	NA
ENSBTAG00000000793	NA	NA	NA
ENSBTAG00000000795	NA	NA	NA
ENSBTAG00000000797	-0.053948	0.8294699	0.0811994
ENSBTAG00000000799	0.1926377	0.6341039	0.1978396
ENSBTAG00000000801	-0.051705	0.8374472	0.0770425
ENSBTAG00000000802	-0.116537	0.7866385	0.1042248
ENSBTAG00000000803	-0.101269	0.7540082	0.1226239
ENSBTAG00000000804	0.0748767	0.831292	0.0802464
ENSBTAG00000000805	NA	NA	NA
ENSBTAG00000000806	-0.174735	0.6145884	0.2114157
ENSBTAG00000000807	0.1562328	0.7290029	0.1372707
ENSBTAG00000000808	-0.166444	0.5115869	0.2910806
ENSBTAG00000000809	NA	NA	NA
ENSBTAG00000000810	-0.002407	0.9927998	0.0031383
ENSBTAG00000000811	0.3103852	0.3747733	0.4262314
ENSBTAG00000000812	NA	NA	NA
ENSBTAG00000000813	-0.112016	0.6939394	0.1586784
ENSBTAG00000000815	-0.41824	0.2898667	0.5378017
ENSBTAG00000000816	0.3496784	0.4603298	0.336931
ENSBTAG00000000817	0.4096702	0.3314802	0.4795423
ENSBTAG00000000818	NA	NA	NA
ENSBTAG00000000819	-0.023007	0.957548	0.0188394
ENSBTAG00000000820	0.6836149	0.0262794	1.5803838
ENSBTAG00000000821	NA	NA	NA
ENSBTAG00000000824	0.0798609	0.8262352	0.0828963
ENSBTAG00000000825	-0.287289	0.297207	0.526941
ENSBTAG00000000827	-0.201906	0.4744741	0.3237875
ENSBTAG00000000828	0.4155361	0.1968648	0.7058319
ENSBTAG00000000829	NA	NA	NA
ENSBTAG00000000830	NA	NA	NA
ENSBTAG00000000831	0.3491396	0.3139368	0.5031578
ENSBTAG00000000833	-0.006303	0.9857505	0.006233
ENSBTAG00000000835	-0.525148	0.33223	0.4785612
ENSBTAG00000000836	-1.205526	0.0209558	1.6786967
ENSBTAG00000000837	0.0261475	0.9244215	0.03413
ENSBTAG00000000838	-0.297581	0.2448003	0.6111881
ENSBTAG00000000841	0.0051249	1	0

ENSBTAG00000000842	NA	NA	NA
ENSBTAG00000000843	-0.182426	0.5643235	0.2484718
ENSBTAG00000000848	0.1514939	0.5495696	0.2599773
ENSBTAG00000000851	0.0273343	0.9401196	0.0268169
ENSBTAG00000000854	NA	NA	NA
ENSBTAG00000000855	-0.091629	0.7569157	0.1209525
ENSBTAG00000000856	0.0812443	0.8760004	0.0574957
ENSBTAG00000000857	0.2819574	0.3904678	0.4084147
ENSBTAG00000000859	0.5092995	0.1858057	0.730941
ENSBTAG00000000868	NA	NA	NA
ENSBTAG00000000869	-0.021605	0.9545216	0.0202142
ENSBTAG00000000871	0.1194585	0.676947	0.1694453
ENSBTAG00000000873	NA	NA	NA
ENSBTAG00000000874	-0.11089	0.6552362	0.1836021
ENSBTAG00000000875	0.7001468	0.0747218	1.1265528
ENSBTAG00000000877	-0.060531	0.8406594	0.0753799
ENSBTAG00000000878	0.2031251	0.4175719	0.3792688
ENSBTAG00000000879	-0.038488	0.9102873	0.0408215
ENSBTAG00000000880	-0.233355	0.3675191	0.4347201
ENSBTAG00000000885	NA	NA	NA
ENSBTAG00000000888	NA	NA	NA
ENSBTAG00000000892	NA	NA	NA
ENSBTAG00000000894	-0.425453	0.0979395	1.0090421
ENSBTAG00000000895	-0.112148	0.7587128	0.1199226
ENSBTAG00000000897	-0.315926	0.3982808	0.3998106
ENSBTAG00000000898	1.1490582	0.0065421	2.1842812
ENSBTAG00000000899	0.4315152	0.3145151	0.5023585
ENSBTAG00000000900	-0.169236	0.6347457	0.1974002
ENSBTAG00000000902	-0.244346	0.3543139	0.4506118
ENSBTAG00000000905	NA	NA	NA
ENSBTAG00000000908	NA	NA	NA
ENSBTAG00000000910	NA	NA	NA
ENSBTAG00000000911	-0.183709	0.529136	0.2764327
ENSBTAG00000000913	-0.010028	0.9698096	0.0133135
ENSBTAG00000000916	NA	NA	NA
ENSBTAG00000000917	0.2332802	0.6031219	0.2195949
ENSBTAG00000000918	0.0859126	0.788822	0.103021
ENSBTAG00000000919	0.485191	0.2946966	0.5306248
ENSBTAG00000000920	NA	NA	NA
ENSBTAG00000000925	-0.365213	0.3110246	0.5072053
ENSBTAG00000000926	NA	NA	NA
ENSBTAG00000000928	0.2358718	0.4271508	0.3694187
ENSBTAG00000000930	NA	NA	NA
ENSBTAG00000000933	NA	NA	NA
ENSBTAG00000000936	NA	NA	NA
ENSBTAG00000000937	0.1507116	0.5665185	0.2467859
ENSBTAG00000000939	0.399428	0.1561202	0.8065409
ENSBTAG00000000940	0.0174042	0.9501312	0.0222164
ENSBTAG00000000941	-0.292415	0.2442625	0.6121432
ENSBTAG00000000942	0.0391806	0.9180294	0.0371434
ENSBTAG00000000943	0.1247148	0.7023155	0.1534678
ENSBTAG00000000944	NA	NA	NA
ENSBTAG00000000945	-0.152043	0.7067101	0.1507587
ENSBTAG00000000946	0.264136	0.5209281	0.2832222
ENSBTAG00000000948	0.2200756	0.4013735	0.3964513
ENSBTAG00000000949	-0.217803	0.5709659	0.2433898
ENSBTAG00000000950	-0.051107	0.8436828	0.0738208
ENSBTAG00000000951	NA	NA	NA

ENSBTAG00000000954	NA	NA	NA
ENSBTAG00000000955	-0.302768	0.2894162	0.5384771
ENSBTAG00000000957	-0.014341	0.9623115	0.0166843
ENSBTAG00000000958	NA	NA	NA
ENSBTAG00000000959	NA	NA	NA
ENSBTAG00000000960	NA	NA	NA
ENSBTAG00000000961	0.374944	0.3316765	0.4792853
ENSBTAG00000000962	NA	NA	NA
ENSBTAG00000000964	NA	NA	NA
ENSBTAG00000000965	NA	NA	NA
ENSBTAG00000000966	NA	NA	NA
ENSBTAG00000000967	0.2356692	0.5073426	0.2946987
ENSBTAG00000000973	NA	NA	NA
ENSBTAG00000000974	-0.136488	0.5790509	0.2372833
ENSBTAG00000000977	0.2302781	0.5122433	0.2905237
ENSBTAG00000000978	0.0838215	0.8021295	0.0957555
ENSBTAG00000000979	-0.362692	0.1460727	0.835431
ENSBTAG00000000981	-0.976564	0.0151678	1.8190781
ENSBTAG00000000983	NA	NA	NA
ENSBTAG00000000985	-0.286654	0.274053	0.5621654
ENSBTAG00000000986	NA	NA	NA
ENSBTAG00000000987	NA	NA	NA
ENSBTAG00000000988	-0.290709	0.5452066	0.2634389
ENSBTAG00000000990	-0.043571	0.8650083	0.0629797
ENSBTAG00000000991	-0.047434	0.8770201	0.0569905
ENSBTAG00000000993	0.0302292	0.9137439	0.0391755
ENSBTAG00000000995	0.0558602	0.9102837	0.0408232
ENSBTAG00000000998	-0.029239	0.9310546	0.0310249
ENSBTAG00000001000	NA	NA	NA
ENSBTAG00000001002	NA	NA	NA
ENSBTAG00000001003	0.173443	0.5250144	0.2798288
ENSBTAG00000001004	0.3430477	0.3115887	0.5064184
ENSBTAG00000001005	0.0183977	0.9680457	0.0141042
ENSBTAG00000001006	NA	NA	NA
ENSBTAG00000001007	-0.450789	0.2682611	0.5714423
ENSBTAG00000001008	NA	NA	NA
ENSBTAG00000001009	0.3249992	0.4066498	0.3907794
ENSBTAG00000001010	NA	NA	NA
ENSBTAG00000001013	0.2695475	0.3349647	0.4750009
ENSBTAG00000001014	-0.088527	0.8412348	0.0750828
ENSBTAG00000001015	0.3351116	0.2276808	0.6426735
ENSBTAG00000001016	-0.10162	0.6867414	0.1632068
ENSBTAG00000001017	-0.278291	0.2662271	0.5747478
ENSBTAG00000001019	NA	NA	NA
ENSBTAG00000001020	NA	NA	NA
ENSBTAG00000001021	-0.621882	0.1339471	0.8730667
ENSBTAG00000001022	0.2358911	0.4963143	0.3042432
ENSBTAG00000001023	0.1777072	0.5484971	0.2608257
ENSBTAG00000001024	-0.014764	0.9617239	0.0169496
ENSBTAG00000001026	NA	NA	NA
ENSBTAG00000001027	-0.162272	0.5706993	0.2435927
ENSBTAG00000001028	NA	NA	NA
ENSBTAG00000001029	0.023415	0.9718509	0.0124004
ENSBTAG00000001030	-0.198508	0.4313495	0.3651707
ENSBTAG00000001032	-0.355471	0.1642757	0.7844266
ENSBTAG00000001034	-0.588924	0.2675177	0.5726475
ENSBTAG00000001035	0.0325895	0.9025648	0.0445216
ENSBTAG00000001036	-0.234509	0.3446803	0.4625836

ENSBTAG00000001037	0.7459363	0.0449859	1.3469232
ENSBTAG00000001038	-0.004277	0.9871021	0.0056379
ENSBTAG00000001041	NA	NA	NA
ENSBTAG00000001042	-0.012399	0.9759301	0.0105813
ENSBTAG00000001043	NA	NA	NA
ENSBTAG00000001044	-0.14585	0.6206005	0.2071879
ENSBTAG00000001049	0.1244929	0.7676638	0.114829
ENSBTAG00000001050	-0.088921	0.8383181	0.0765912
ENSBTAG00000001051	NA	NA	NA
ENSBTAG00000001052	1.9450172	0.0004134	3.3835865
ENSBTAG00000001055	0.0265863	0.9236633	0.0344863
ENSBTAG00000001057	0.4050757	0.2265569	0.6448227
ENSBTAG00000001058	-0.069551	0.7948394	0.0997206
ENSBTAG00000001059	0.1100163	0.6731539	0.1718856
ENSBTAG00000001060	0.0782016	0.8095559	0.0917531
ENSBTAG00000001061	0.1133726	0.6875776	0.1626783
ENSBTAG00000001063	NA	NA	NA
ENSBTAG00000001066	NA	NA	NA
ENSBTAG00000001067	NA	NA	NA
ENSBTAG00000001068	NA	NA	NA
ENSBTAG00000001069	0.3915967	0.4303296	0.3661988
ENSBTAG00000001071	-0.119648	0.7593986	0.1195302
ENSBTAG00000001074	0.0453548	0.9244296	0.0341262
ENSBTAG00000001075	NA	NA	NA
ENSBTAG00000001077	0.40771	0.2519877	0.5986207
ENSBTAG00000001078	0.7224215	0.0443645	1.3529649
ENSBTAG00000001080	NA	NA	NA
ENSBTAG00000001081	-0.74714	0.018185	1.7402864
ENSBTAG00000001082	NA	NA	NA
ENSBTAG00000001083	-0.430276	0.109	0.9625734
ENSBTAG00000001085	NA	NA	NA
ENSBTAG00000001086	-0.065148	0.9008598	0.0453428
ENSBTAG00000001088	NA	NA	NA
ENSBTAG00000001090	NA	NA	NA
ENSBTAG00000001092	-0.416985	0.2361549	0.626803
ENSBTAG00000001093	-0.316381	0.2248616	0.6480846
ENSBTAG00000001094	NA	NA	NA
ENSBTAG00000001096	-0.670626	0.128222	0.8920376
ENSBTAG00000001097	0.0884555	0.83549	0.0780587
ENSBTAG00000001098	-0.002878	0.9937843	0.0027079
ENSBTAG00000001099	NA	NA	NA
ENSBTAG00000001100	NA	NA	NA
ENSBTAG00000001101	0.5701223	0.3407807	0.467525
ENSBTAG00000001102	NA	NA	NA
ENSBTAG00000001104	NA	NA	NA
ENSBTAG00000001105	-0.108615	0.6851006	0.1642457
ENSBTAG00000001107	-0.212222	0.4165175	0.3803667
ENSBTAG00000001108	-0.37099	0.2341638	0.6304802
ENSBTAG00000001109	-0.376115	0.153988	0.8125132
ENSBTAG00000001110	0.0185616	0.9586196	0.0183537
ENSBTAG00000001112	-0.082023	0.874551	0.0582149
ENSBTAG00000001113	-0.120193	0.6700048	0.1739221
ENSBTAG00000001114	-0.039838	0.8893828	0.0509113
ENSBTAG00000001116	0.0391948	0.9005399	0.045497
ENSBTAG00000001117	-0.05165	0.8775374	0.0567343
ENSBTAG00000001119	NA	NA	NA
ENSBTAG00000001120	0.0799213	0.7524246	0.123537
ENSBTAG00000001123	-0.077446	0.8447185	0.073288

ENSBTAG00000001124	-0.237978	0.5553444	0.2554376
ENSBTAG00000001126	0.1720955	0.5475435	0.2615814
ENSBTAG00000001128	NA	NA	NA
ENSBTAG00000001131	0.0432681	0.934799	0.0292818
ENSBTAG00000001132	-0.118563	0.6871598	0.1629422
ENSBTAG00000001133	0.3555252	0.1908579	0.7192899
ENSBTAG00000001134	0.4053108	0.1478257	0.83025
ENSBTAG00000001136	NA	NA	NA
ENSBTAG00000001137	0.2410761	0.3512391	0.4543972
ENSBTAG00000001138	0.198268	0.5747871	0.240493
ENSBTAG00000001139	NA	NA	NA
ENSBTAG00000001140	0.0877239	0.7821518	0.106709
ENSBTAG00000001141	0.1242852	0.6695972	0.1741863
ENSBTAG00000001142	-0.092099	0.7708857	0.11301
ENSBTAG00000001143	0.0276034	0.9655878	0.0152082
ENSBTAG00000001144	0.0539306	0.8375701	0.0769788
ENSBTAG00000001146	-1.050583	0.0026786	2.5720988
ENSBTAG00000001150	NA	NA	NA
ENSBTAG00000001151	-0.39438	0.346743	0.4599923
ENSBTAG00000001152	NA	NA	NA
ENSBTAG00000001153	-0.067873	0.8564823	0.0672816
ENSBTAG00000001154	-0.443751	0.2588311	0.5869836
ENSBTAG00000001156	1.6492025	2.43E-05	4.614864
ENSBTAG00000001160	0.3857588	0.3027302	0.5189443
ENSBTAG00000001161	0.2411758	0.5580846	0.2532999
ENSBTAG00000001163	NA	NA	NA
ENSBTAG00000001164	0.0085514	0.9806998	0.0084639
ENSBTAG00000001165	0.1390011	0.6515639	0.186043
ENSBTAG00000001166	NA	NA	NA
ENSBTAG00000001168	0.1102556	0.7143834	0.1460686
ENSBTAG00000001171	-0.031373	0.9106099	0.0406676
ENSBTAG00000001173	-0.236144	0.4565009	0.3405584
ENSBTAG00000001174	-0.052934	0.8492334	0.0709729
ENSBTAG00000001176	-0.599628	0.4359661	0.3605473
ENSBTAG00000001178	0.1007188	0.7416661	0.1297916
ENSBTAG00000001179	0.7017712	0.0103824	1.9837027
ENSBTAG00000001181	-0.138456	0.5950015	0.2254819
ENSBTAG00000001182	-0.416214	0.0983075	1.0074133
ENSBTAG00000001183	0.0009606	0.9978155	0.0009497
ENSBTAG00000001185	-0.128528	0.6149661	0.2111488
ENSBTAG00000001186	NA	NA	NA
ENSBTAG00000001187	NA	NA	NA
ENSBTAG00000001188	NA	NA	NA
ENSBTAG00000001189	0.2604595	0.2970225	0.5272107
ENSBTAG00000001191	NA	NA	NA
ENSBTAG00000001192	-0.016758	0.9521318	0.0213029
ENSBTAG00000001193	0.1316569	0.7518321	0.1238792
ENSBTAG00000001195	NA	NA	NA
ENSBTAG00000001197	NA	NA	NA
ENSBTAG00000001198	-0.327871	0.4851607	0.3141144
ENSBTAG00000001199	-0.091198	0.7588642	0.1198359
ENSBTAG00000001204	0.0843585	0.8069037	0.0931783
ENSBTAG00000001206	NA	NA	NA
ENSBTAG00000001207	-0.018528	0.975033	0.0109807
ENSBTAG00000001209	0.0131997	0.9600828	0.0176913
ENSBTAG00000001212	0.1093456	0.6661063	0.1764565
ENSBTAG00000001219	NA	NA	NA
ENSBTAG00000001223	-0.02315	0.9413364	0.0262552

ENSBTAG00000001224	0.1418985	0.6807094	0.1670383
ENSBTAG00000001225	0.3338353	0.3514471	0.4541401
ENSBTAG00000001228	NA	NA	NA
ENSBTAG00000001229	NA	NA	NA
ENSBTAG00000001231	NA	NA	NA
ENSBTAG00000001233	0.6307391	0.1218975	0.9140052
ENSBTAG00000001235	0.5213095	0.2588952	0.586876
ENSBTAG00000001242	NA	NA	NA
ENSBTAG00000001243	0.1609505	0.6787966	0.1682603
ENSBTAG00000001244	-0.467413	0.1639483	0.7852932
ENSBTAG00000001246	-0.020704	0.9628222	0.0164539
ENSBTAG00000001249	NA	NA	NA
ENSBTAG00000001250	NA	NA	NA
ENSBTAG00000001252	0.0122009	0.9801712	0.0086981
ENSBTAG00000001254	-0.13081	0.5959173	0.224814
ENSBTAG00000001255	NA	NA	NA
ENSBTAG00000001257	-0.531009	0.0395902	1.4024127
ENSBTAG00000001258	-0.243567	0.4279219	0.3686355
ENSBTAG00000001262	0.0642443	0.8156711	0.0884849
ENSBTAG00000001265	NA	NA	NA
ENSBTAG00000001269	-0.452088	0.2509212	0.6004627
ENSBTAG00000001271	NA	NA	NA
ENSBTAG00000001273	NA	NA	NA
ENSBTAG00000001274	-0.668357	0.0224118	1.6495224
ENSBTAG00000001279	NA	NA	NA
ENSBTAG00000001280	-0.243648	0.4289392	0.3676042
ENSBTAG00000001282	-0.225029	0.4040916	0.3935202
ENSBTAG00000001283	-0.230808	0.3527227	0.4525667
ENSBTAG00000001286	NA	NA	NA
ENSBTAG00000001287	NA	NA	NA
ENSBTAG00000001288	-0.149922	0.6603289	0.1802397
ENSBTAG00000001289	0.1725607	0.53653	0.270406
ENSBTAG00000001290	0.3019435	0.4735182	0.3246634
ENSBTAG00000001292	NA	NA	NA
ENSBTAG00000001293	0.1396595	0.7599367	0.1192226
ENSBTAG00000001294	0.7821321	0.0101224	1.9947162
ENSBTAG00000001295	-0.004816	0.989955	0.0043846
ENSBTAG00000001296	0.1625066	0.5466659	0.262278
ENSBTAG00000001298	0.1313023	0.7065563	0.1508533
ENSBTAG00000001299	0.4284868	0.2407558	0.6184233
ENSBTAG00000001301	0.2764594	0.5521926	0.2579094
ENSBTAG00000001302	-0.261121	0.3661203	0.4363762
ENSBTAG00000001303	0.9627801	0.0024762	2.6062081
ENSBTAG00000001305	-0.619676	0.1241544	0.906038
ENSBTAG00000001306	-0.357989	0.2080689	0.6817927
ENSBTAG00000001308	NA	NA	NA
ENSBTAG00000001310	NA	NA	NA
ENSBTAG00000001311	-0.071895	0.7872919	0.1038642
ENSBTAG00000001314	0.0781775	0.7912054	0.1017108
ENSBTAG00000001315	NA	NA	NA
ENSBTAG00000001320	0.2693309	0.429744	0.3667902
ENSBTAG00000001321	-1.142085	0.0314345	1.5025929
ENSBTAG00000001322	NA	NA	NA
ENSBTAG00000001323	-0.399911	0.1211593	0.9166434
ENSBTAG00000001324	NA	NA	NA
ENSBTAG00000001325	NA	NA	NA
ENSBTAG00000001326	NA	NA	NA
ENSBTAG00000001328	-0.155507	0.7043088	0.1522369

ENSBTAG00000001329	0.5350671	0.0968932	1.0137065
ENSBTAG00000001331	NA	NA	NA
ENSBTAG00000001332	-0.154044	0.5632352	0.2493102
ENSBTAG00000001333	0.0846677	0.8495147	0.0708291
ENSBTAG00000001335	-0.228549	0.4338066	0.3627039
ENSBTAG00000001336	NA	NA	NA
ENSBTAG00000001338	NA	NA	NA
ENSBTAG00000001341	NA	NA	NA
ENSBTAG00000001342	0.0122394	0.9648446	0.0155426
ENSBTAG00000001343	NA	NA	NA
ENSBTAG00000001344	-0.076135	0.8742388	0.0583699
ENSBTAG00000001346	NA	NA	NA
ENSBTAG00000001348	-0.37638	0.158594	0.7997131
ENSBTAG00000001349	-0.158999	0.5625424	0.2498447
ENSBTAG00000001352	0.0966176	0.7464663	0.1269898
ENSBTAG00000001353	-0.122959	0.6240192	0.204802
ENSBTAG00000001354	0.46421	0.2897686	0.5379486
ENSBTAG00000001356	-0.1802	0.4689644	0.3288601
ENSBTAG00000001359	NA	NA	NA
ENSBTAG00000001360	0.0052751	0.9836808	0.0071458
ENSBTAG00000001361	-0.36332	0.1644298	0.7840194
ENSBTAG00000001364	-0.551493	0.1864195	0.7295087
ENSBTAG00000001365	1.2984966	0.0003083	3.5109758
ENSBTAG00000001367	NA	NA	NA
ENSBTAG00000001368	0.233596	0.509215	0.2930988
ENSBTAG00000001374	NA	NA	NA
ENSBTAG00000001376	NA	NA	NA
ENSBTAG00000001382	NA	NA	NA
ENSBTAG00000001385	1.0874676	0.0140343	1.8528099
ENSBTAG00000001387	0.1303294	0.6285655	0.2016494
ENSBTAG00000001388	NA	NA	NA
ENSBTAG00000001390	0.5823988	0.0917463	1.0374116
ENSBTAG00000001391	0.2006374	0.4635694	0.3338853
ENSBTAG00000001392	NA	NA	NA
ENSBTAG00000001393	-0.449466	0.0751008	1.1243554
ENSBTAG00000001394	0.1765917	0.470346	0.3275825
ENSBTAG00000001395	0.415785	0.3370474	0.472309
ENSBTAG00000001396	NA	NA	NA
ENSBTAG00000001398	-0.198472	0.5171377	0.2863938
ENSBTAG00000001400	0.1299116	0.6487076	0.187951
ENSBTAG00000001403	-0.165064	0.6018168	0.2205357
ENSBTAG00000001405	0.6812209	0.0453486	1.3434363
ENSBTAG00000001406	NA	NA	NA
ENSBTAG00000001407	-0.040043	0.8773195	0.0568422
ENSBTAG00000001408	0.0576848	0.841398	0.0749985
ENSBTAG00000001410	-0.543568	0.2245697	0.6486488
ENSBTAG00000001412	0.106347	0.7609033	0.1186705
ENSBTAG00000001414	0.5626508	0.1685915	0.7731643
ENSBTAG00000001415	1.2111917	0.0063718	2.1957405
ENSBTAG00000001417	0.1586589	0.7544926	0.122345
ENSBTAG00000001419	0.1597802	0.5402916	0.2673718
ENSBTAG00000001420	0.3664482	0.3121476	0.5056399
ENSBTAG00000001422	-0.164843	0.5331063	0.2731862
ENSBTAG00000001423	NA	NA	NA
ENSBTAG00000001424	-0.187097	0.4791612	0.3195184
ENSBTAG00000001425	-0.304167	0.2259059	0.6460724
ENSBTAG00000001429	0.706925	0.0248544	1.604596
ENSBTAG00000001432	NA	NA	NA

ENSBTAG00000001435	0.1835446	0.5809112	0.2358903
ENSBTAG00000001439	NA	NA	NA
ENSBTAG00000001440	0.3449937	0.4334121	0.363099
ENSBTAG00000001441	-0.204618	0.5945349	0.2258226
ENSBTAG00000001443	NA	NA	NA
ENSBTAG00000001444	-0.04538	0.8730016	0.058985
ENSBTAG00000001446	NA	NA	NA
ENSBTAG00000001447	1.1403217	0.2485713	0.6045491
ENSBTAG00000001449	NA	NA	NA
ENSBTAG00000001450	0.0301308	0.9103741	0.0407801
ENSBTAG00000001455	0.7253702	0.0790405	1.1021501
ENSBTAG00000001457	0.6586347	0.1384381	0.8587443
ENSBTAG00000001460	0.1311822	0.5921161	0.2275931
ENSBTAG00000001462	-0.008173	0.9925146	0.0032631
ENSBTAG00000001463	-0.158024	0.5307602	0.2751017
ENSBTAG00000001464	NA	NA	NA
ENSBTAG00000001465	NA	NA	NA
ENSBTAG00000001468	0.0065638	0.9799587	0.0087922
ENSBTAG00000001470	0.2468137	0.4739718	0.3242475
ENSBTAG00000001471	0.1250219	0.7878003	0.1035839
ENSBTAG00000001473	-0.352342	0.2256761	0.6465145
ENSBTAG00000001474	NA	NA	NA
ENSBTAG00000001475	0.1332549	0.7231537	0.1407694
ENSBTAG00000001476	NA	NA	NA
ENSBTAG00000001478	-0.255123	0.3010876	0.5213071
ENSBTAG00000001481	NA	NA	NA
ENSBTAG00000001483	0.5148407	0.1308345	0.8832778
ENSBTAG00000001484	-0.373568	0.2878041	0.5409031
ENSBTAG00000001485	-0.209594	0.4033125	0.3943583
ENSBTAG00000001486	-0.168733	0.5010092	0.3001543
ENSBTAG00000001489	-0.457986	0.0833479	1.0791056
ENSBTAG00000001492	-0.246329	0.6415354	0.1927794
ENSBTAG00000001497	-0.058419	0.8590748	0.065969
ENSBTAG00000001498	0.1035494	0.7400263	0.1307528
ENSBTAG00000001499	-0.242048	0.3563594	0.4481118
ENSBTAG00000001500	NA	NA	NA
ENSBTAG00000001503	0.1375082	0.661208	0.1796619
ENSBTAG00000001504	0.147579	0.6396871	0.1940324
ENSBTAG00000001505	-0.316538	0.4145317	0.3824422
ENSBTAG00000001506	0.1731262	0.5356666	0.2711054
ENSBTAG00000001507	-0.349626	0.2432586	0.6139319
ENSBTAG00000001508	0.1303278	0.7233405	0.1406572
ENSBTAG00000001509	0.4661145	0.312855	0.5046568
ENSBTAG00000001510	-0.347315	0.2075656	0.6828446
ENSBTAG00000001511	0.2831514	0.3230171	0.4907744
ENSBTAG00000001512	0.0061711	0.9869123	0.0057215
ENSBTAG00000001513	0.4881418	0.1125483	0.9486611
ENSBTAG00000001514	-0.566159	0.0249952	1.6021429
ENSBTAG00000001516	NA	NA	NA
ENSBTAG00000001517	NA	NA	NA
ENSBTAG00000001518	0.4846356	0.2106041	0.6765332
ENSBTAG00000001519	-0.424124	0.3399777	0.4685496
ENSBTAG00000001520	-0.125514	0.6110915	0.2138938
ENSBTAG00000001521	-0.142595	0.6274213	0.2024408
ENSBTAG00000001522	-0.043181	0.8927281	0.0492808
ENSBTAG00000001523	-0.185379	0.6539075	0.1844837
ENSBTAG00000001528	-0.344785	0.2207188	0.6561607
ENSBTAG00000001529	-0.035751	0.8906539	0.050291

ENSBTAG00000001530	NA	NA	NA
ENSBTAG00000001533	-0.004687	0.9878969	0.0052884
ENSBTAG00000001537	0.1601659	0.6833351	0.1653662
ENSBTAG00000001538	NA	NA	NA
ENSBTAG00000001539	-0.380248	0.1913191	0.7182417
ENSBTAG00000001543	NA	NA	NA
ENSBTAG00000001545	NA	NA	NA
ENSBTAG00000001546	0.1620509	0.5947286	0.2256812
ENSBTAG00000001548	0.1333333	0.666622	0.1761204
ENSBTAG00000001551	0.2714658	0.4014367	0.3963829
ENSBTAG00000001552	0.147892	0.5654944	0.2475717
ENSBTAG00000001553	0.0822891	0.7536198	0.1228477
ENSBTAG00000001557	-0.603514	0.2197933	0.6579856
ENSBTAG00000001558	NA	NA	NA
ENSBTAG00000001562	NA	NA	NA
ENSBTAG00000001564	-0.135885	0.6101805	0.2145417
ENSBTAG00000001565	0.3510019	0.2200439	0.6574907
ENSBTAG00000001567	-0.166496	0.6516956	0.1859552
ENSBTAG00000001568	0.6987821	0.0712165	1.1474193
ENSBTAG00000001571	NA	NA	NA
ENSBTAG00000001572	NA	NA	NA
ENSBTAG00000001573	-0.780162	0.0035564	2.448987
ENSBTAG00000001574	0.3584075	0.2474518	0.6065094
ENSBTAG00000001575	-0.219676	0.3814507	0.4185616
ENSBTAG00000001576	0.0697468	0.8073361	0.0929456
ENSBTAG00000001578	0.3221508	0.3711668	0.4304309
ENSBTAG00000001579	-0.016232	0.9604536	0.0175236
ENSBTAG00000001580	NA	NA	NA
ENSBTAG00000001582	NA	NA	NA
ENSBTAG00000001585	0.2346974	0.46392	0.3335569
ENSBTAG00000001586	0.3156858	0.2673268	0.5729575
ENSBTAG00000001589	-0.013549	0.9566582	0.0192432
ENSBTAG00000001592	-0.151396	0.712321	0.1473242
ENSBTAG00000001593	-0.048443	0.8714264	0.0597693
ENSBTAG00000001594	NA	NA	NA
ENSBTAG00000001595	NA	NA	NA
ENSBTAG00000001597	0.0161892	0.9767129	0.0102331
ENSBTAG00000001598	NA	NA	NA
ENSBTAG00000001599	NA	NA	NA
ENSBTAG00000001600	-0.012236	0.9751742	0.0109178
ENSBTAG00000001601	-0.52534	0.0899995	1.0457598
ENSBTAG00000001602	0.0142009	0.9630969	0.01633
ENSBTAG00000001603	-0.377177	0.364814	0.4379285
ENSBTAG00000001604	-0.143265	0.6978552	0.1562347
ENSBTAG00000001605	0.29172	0.5927237	0.2271477
ENSBTAG00000001606	NA	NA	NA
ENSBTAG00000001607	0.0495805	0.8602001	0.0654005
ENSBTAG00000001609	-0.13251	0.6243121	0.2045983
ENSBTAG00000001610	NA	NA	NA
ENSBTAG00000001612	0.031063	0.9148501	0.0386501
ENSBTAG00000001614	0.4136488	0.3178273	0.4978088
ENSBTAG00000001615	NA	NA	NA
ENSBTAG00000001616	0.1702012	0.5737385	0.241286
ENSBTAG00000001617	0.096031	0.7463454	0.1270601
ENSBTAG00000001618	-0.664479	0.0638374	1.194925
ENSBTAG00000001619	-0.145664	0.6564556	0.1827947
ENSBTAG00000001621	NA	NA	NA
ENSBTAG00000001626	-0.33865	0.4462256	0.3504455

ENSBTAG00000001627	NA	NA	NA
ENSBTAG00000001628	NA	NA	NA
ENSBTAG00000001629	0.0968153	0.7253584	0.1394474
ENSBTAG00000001631	NA	NA	NA
ENSBTAG00000001632	0.1700602	0.5070762	0.2949268
ENSBTAG00000001634	-0.076974	0.8080947	0.0925377
ENSBTAG00000001635	0.1615981	0.5903109	0.2289192
ENSBTAG00000001637	-0.273097	0.4091279	0.3881409
ENSBTAG00000001638	NA	NA	NA
ENSBTAG00000001639	NA	NA	NA
ENSBTAG00000001640	-0.310727	0.4732092	0.3249468
ENSBTAG00000001644	0.0641292	0.803323	0.0951098
ENSBTAG00000001645	0.2421868	0.4554406	0.3415683
ENSBTAG00000001647	-0.264857	0.5119271	0.2907919
ENSBTAG00000001648	0.0713756	0.782681	0.1064152
ENSBTAG00000001649	-2.85214	2.14E-10	9.6697506
ENSBTAG00000001651	-0.0437	0.8680846	0.061438
ENSBTAG00000001652	0.1258427	0.7527137	0.1233702
ENSBTAG00000001654	-0.060275	0.866921	0.0620205
ENSBTAG00000001656	0.3757125	0.289003	0.5390977
ENSBTAG00000001657	-0.251625	0.3779086	0.4226132
ENSBTAG00000001658	NA	NA	NA
ENSBTAG00000001659	0.0165345	0.9482708	0.0230676
ENSBTAG00000001660	0.1507579	0.5967033	0.2242416
ENSBTAG00000001662	0.6850006	0.1494421	0.8255269
ENSBTAG00000001663	-0.244921	0.5273913	0.277867
ENSBTAG00000001665	0.2899334	0.3751681	0.4257742
ENSBTAG00000001666	0.1525937	0.6789958	0.1681329
ENSBTAG00000001671	-0.439625	0.0967755	1.0142347
ENSBTAG00000001673	0.0121242	0.9637805	0.0160219
ENSBTAG00000001675	NA	NA	NA
ENSBTAG00000001683	NA	NA	NA
ENSBTAG00000001686	NA	NA	NA
ENSBTAG00000001687	-1.051079	0.0261486	1.5825519
ENSBTAG00000001692	0.2486274	0.4275384	0.3690248
ENSBTAG00000001693	0.1267286	0.6753523	0.1704696
ENSBTAG00000001694	NA	NA	NA
ENSBTAG00000001696	NA	NA	NA
ENSBTAG00000001697	-0.008209	0.9749446	0.0110201
ENSBTAG00000001698	0.4531599	0.1411329	0.8503719
ENSBTAG00000001700	-0.162482	0.5277548	0.2775678
ENSBTAG00000001702	-0.102148	0.7718864	0.1124466
ENSBTAG00000001703	NA	NA	NA
ENSBTAG00000001704	NA	NA	NA
ENSBTAG00000001706	-0.74108	0.0051712	2.286405
ENSBTAG00000001707	-0.234465	0.4489861	0.3477671
ENSBTAG00000001708	-0.008102	0.978483	0.0094467
ENSBTAG00000001710	NA	NA	NA
ENSBTAG00000001711	-0.183472	0.5675258	0.2460144
ENSBTAG00000001712	0.2495318	0.6110682	0.2139103
ENSBTAG00000001714	0.8298731	0.078444	1.1054403
ENSBTAG00000001717	NA	NA	NA
ENSBTAG00000001721	-0.234351	0.4056542	0.391844
ENSBTAG00000001724	NA	NA	NA
ENSBTAG00000001725	NA	NA	NA
ENSBTAG00000001727	-0.45962	0.0945269	1.0244444
ENSBTAG00000001728	NA	NA	NA
ENSBTAG00000001729	0.2063632	0.4754991	0.3228503

ENSBTAG00000001731	-0.089167	0.7464794	0.1269821
ENSBTAG00000001735	NA	NA	NA
ENSBTAG00000001736	0.0043883	0.9899465	0.0043883
ENSBTAG00000001737	-0.12565	0.6170606	0.2096722
ENSBTAG00000001739	NA	NA	NA
ENSBTAG00000001740	0.153832	0.7659389	0.1158059
ENSBTAG00000001741	0.0993731	0.7475649	0.1263511
ENSBTAG00000001744	0.3738652	0.286116	0.5434578
ENSBTAG00000001745	-0.382053	0.2234321	0.6508544
ENSBTAG00000001747	-0.355726	0.2021787	0.6942646
ENSBTAG00000001748	0.137573	0.6948715	0.1580955
ENSBTAG00000001749	-0.061696	0.8218756	0.0851939
ENSBTAG00000001751	NA	NA	NA
ENSBTAG00000001752	-0.063771	0.867356	0.0618026
ENSBTAG00000001753	NA	NA	NA
ENSBTAG00000001754	-0.126198	0.624862	0.2042159
ENSBTAG00000001762	0.2627742	0.4877241	0.3118258
ENSBTAG00000001763	0.4585196	0.2502573	0.6016132
ENSBTAG00000001767	-0.24207	0.470127	0.3277848
ENSBTAG00000001768	-0.103074	0.7769783	0.1095911
ENSBTAG00000001770	0.093303	0.7646975	0.1165103
ENSBTAG00000001771	-0.148369	0.5728679	0.2419455
ENSBTAG00000001773	0.160605	0.6320797	0.1992281
ENSBTAG00000001774	0.3969007	0.4722197	0.3258559
ENSBTAG00000001775	NA	NA	NA
ENSBTAG00000001776	-0.09339	0.7130921	0.1468544
ENSBTAG00000001777	0.1927308	0.4560807	0.3409583
ENSBTAG00000001778	0.5083567	0.180522	0.7434699
ENSBTAG00000001780	0.2930744	0.4083973	0.3889172
ENSBTAG00000001781	0.0124874	0.9629981	0.0163746
ENSBTAG00000001782	0.096444	0.7766513	0.109774
ENSBTAG00000001783	-0.022448	0.9684686	0.0139145
ENSBTAG00000001784	NA	NA	NA
ENSBTAG00000001785	NA	NA	NA
ENSBTAG00000001786	-0.269222	0.5262333	0.2788216
ENSBTAG00000001788	0.2936711	0.3943768	0.4040886
ENSBTAG00000001790	-0.223466	0.4107359	0.3864373
ENSBTAG00000001792	0.1293414	0.6386618	0.1947291
ENSBTAG00000001793	NA	NA	NA
ENSBTAG00000001794	0.0948555	0.7177747	0.1440119
ENSBTAG00000001795	0.0613793	0.8072856	0.0929728
ENSBTAG00000001796	0.3494541	0.3212391	0.4931716
ENSBTAG00000001801	-0.668803	0.1341776	0.8723201
ENSBTAG00000001803	0.3593209	0.1918893	0.7169492
ENSBTAG00000001804	NA	NA	NA
ENSBTAG00000001805	0.1637075	0.5286094	0.2768652
ENSBTAG00000001806	NA	NA	NA
ENSBTAG00000001807	0.4118453	0.1265757	0.8976498
ENSBTAG00000001808	-0.155801	0.6027331	0.219875
ENSBTAG00000001810	0.0460389	0.8538597	0.0686135
ENSBTAG00000001812	NA	NA	NA
ENSBTAG00000001814	0.0174271	0.9584201	0.0184441
ENSBTAG00000001815	NA	NA	NA
ENSBTAG00000001816	0.0748701	0.8722613	0.0593534
ENSBTAG00000001817	-0.162987	0.5467101	0.2622429
ENSBTAG00000001818	NA	NA	NA
ENSBTAG00000001821	-0.109308	0.6660105	0.1765189
ENSBTAG00000001822	-0.016186	0.9736801	0.0115837

ENSBTAG00000001823	0.7987371	0.1764686	0.7533326
ENSBTAG00000001824	NA	NA	NA
ENSBTAG00000001825	NA	NA	NA
ENSBTAG00000001826	0.2122685	0.4582135	0.3389321
ENSBTAG00000001827	0.0473321	0.8773271	0.0568385
ENSBTAG00000001828	0.0007805	1	0
ENSBTAG00000001829	NA	NA	NA
ENSBTAG00000001830	NA	NA	NA
ENSBTAG00000001832	0.2284288	0.4299778	0.366554
ENSBTAG00000001834	0.7667985	0.0217604	1.662333
ENSBTAG00000001835	0.1963959	0.5070145	0.2949796
ENSBTAG00000001836	0.0727568	0.9258655	0.0334521
ENSBTAG00000001838	NA	NA	NA
ENSBTAG00000001839	0.1845935	0.601459	0.2207939
ENSBTAG00000001840	0.3731708	0.30399	0.5171407
ENSBTAG00000001842	-1.548332	0.0005751	3.2402196
ENSBTAG00000001843	-0.271165	0.3273214	0.4850257
ENSBTAG00000001844	0.5887538	0.09118	1.0401006
ENSBTAG00000001847	0.0628258	0.8444102	0.0734465
ENSBTAG00000001848	0.0758677	0.8258008	0.0831247
ENSBTAG00000001851	-0.940868	0.0116353	1.9342221
ENSBTAG00000001852	0.5896079	0.1099614	0.9587597
ENSBTAG00000001854	0.0482825	0.8774633	0.0567711
ENSBTAG00000001855	0.0563967	0.8183353	0.0870687
ENSBTAG00000001856	0.1740923	0.492326	0.3077472
ENSBTAG00000001857	0.1089741	0.8365323	0.0775173
ENSBTAG00000001861	-0.054756	0.8458932	0.0726845
ENSBTAG00000001862	NA	NA	NA
ENSBTAG00000001864	-0.100269	0.8043854	0.0945358
ENSBTAG00000001865	-0.852594	0.0021029	2.6771744
ENSBTAG00000001867	-0.128809	0.6253634	0.2038675
ENSBTAG00000001868	-0.114612	0.6912347	0.1603744
ENSBTAG00000001872	-0.230716	0.5263233	0.2787474
ENSBTAG00000001874	1.1285999	0.0571892	1.2426856
ENSBTAG00000001877	-0.436032	0.3955991	0.4027447
ENSBTAG00000001879	-1.144637	0.0021954	2.6584935
ENSBTAG00000001881	NA	NA	NA
ENSBTAG00000001882	NA	NA	NA
ENSBTAG00000001885	-0.325542	0.3650706	0.4376231
ENSBTAG00000001886	NA	NA	NA
ENSBTAG00000001887	-0.359844	0.3057298	0.5146622
ENSBTAG00000001888	-0.302036	0.3695352	0.4323442
ENSBTAG00000001889	NA	NA	NA
ENSBTAG00000001890	0.6176829	0.0714398	1.1460598
ENSBTAG00000001892	-0.276051	0.3470266	0.4596372
ENSBTAG00000001893	NA	NA	NA
ENSBTAG00000001894	-0.139136	0.5821506	0.2349647
ENSBTAG00000001895	-0.191226	0.5206738	0.2834343
ENSBTAG00000001898	-0.56094	0.1510986	0.8207394
ENSBTAG00000001902	-0.140823	0.5793014	0.2370954
ENSBTAG00000001904	0.259489	0.4213101	0.3753981
ENSBTAG00000001906	NA	NA	NA
ENSBTAG00000001908	-0.337301	0.2414473	0.6171777
ENSBTAG00000001911	NA	NA	NA
ENSBTAG00000001915	NA	NA	NA
ENSBTAG00000001917	NA	NA	NA
ENSBTAG00000001918	2.1738543	8.69E-05	4.0610132
ENSBTAG00000001919	0.1335043	0.7105992	0.1483753

ENSBTAG00000001920	1.1470739	0.0322481	1.491496
ENSBTAG00000001922	-0.206183	0.5976318	0.2235663
ENSBTAG00000001926	0.3062995	0.2449533	0.6109167
ENSBTAG00000001927	NA	NA	NA
ENSBTAG00000001928	-0.171463	0.4897728	0.3100053
ENSBTAG00000001931	-0.279562	0.3431346	0.4645355
ENSBTAG00000001932	0.2303435	0.3584325	0.4455926
ENSBTAG00000001933	-0.310835	0.2168409	0.6638587
ENSBTAG00000001936	-0.126066	0.8297818	0.0810361
ENSBTAG00000001937	0.0314567	0.9027685	0.0444236
ENSBTAG00000001938	NA	NA	NA
ENSBTAG00000001939	0.0947058	0.7160068	0.1450828
ENSBTAG00000001941	-0.093546	0.7144478	0.1460295
ENSBTAG00000001942	-0.203301	0.4447217	0.3519117
ENSBTAG00000001945	-1.375877	0.1334881	0.8745573
ENSBTAG00000001947	NA	NA	NA
ENSBTAG00000001948	-0.222233	0.3715406	0.4299937
ENSBTAG00000001949	0.0643952	0.8564772	0.0672842
ENSBTAG00000001950	0.173625	0.6223766	0.2059467
ENSBTAG00000001952	NA	NA	NA
ENSBTAG00000001956	-0.275992	0.2605357	0.5841327
ENSBTAG00000001957	0.2956541	0.323216	0.4905071
ENSBTAG00000001961	-0.248049	0.3520079	0.4534476
ENSBTAG00000001962	0.1119543	0.6868369	0.1631464
ENSBTAG00000001966	-0.270687	0.4233236	0.3733275
ENSBTAG00000001968	0.0315359	0.9076987	0.0420583
ENSBTAG00000001969	NA	NA	NA
ENSBTAG00000001975	-0.286127	0.5504773	0.2592606
ENSBTAG00000001977	0.2313377	0.3772794	0.4233369
ENSBTAG00000001979	NA	NA	NA
ENSBTAG00000001981	NA	NA	NA
ENSBTAG00000001983	NA	NA	NA
ENSBTAG00000001985	NA	NA	NA
ENSBTAG00000001987	-0.070657	0.7924622	0.1010215
ENSBTAG00000001988	0.1240265	0.6206138	0.2071786
ENSBTAG00000001992	-0.856717	0.013587	1.866876
ENSBTAG00000001996	0.077503	0.7914345	0.101585
ENSBTAG00000001997	NA	NA	NA
ENSBTAG00000001998	NA	NA	NA
ENSBTAG00000001999	0.6892795	0.060803	1.2160748
ENSBTAG00000002000	-0.243055	0.3473843	0.4591898
ENSBTAG00000002001	NA	NA	NA
ENSBTAG00000002002	-0.084986	0.8068912	0.093185
ENSBTAG00000002004	1.1869369	0.327382	0.4849452
ENSBTAG00000002006	0.3485867	0.2084865	0.6809222
ENSBTAG00000002010	0.3819495	0.223497	0.6507284
ENSBTAG00000002011	NA	NA	NA
ENSBTAG00000002012	-0.019517	0.9427693	0.0255946
ENSBTAG00000002014	-0.457898	0.0763209	1.1173567
ENSBTAG00000002015	0.0664757	0.8814283	0.054813
ENSBTAG00000002018	-0.272851	0.3016523	0.5204933
ENSBTAG00000002019	NA	NA	NA
ENSBTAG00000002020	0.0629721	0.8238234	0.0841659
ENSBTAG00000002021	0.1064253	0.7737614	0.1113929
ENSBTAG00000002023	0.5534016	0.1188152	0.9251278
ENSBTAG00000002024	0.132869	0.712445	0.1472486
ENSBTAG00000002025	NA	NA	NA
ENSBTAG00000002026	-0.013155	0.9586075	0.0183592

ENSBTAG00000002027	NA	NA	NA
ENSBTAG00000002028	-0.307479	0.3294905	0.482157
ENSBTAG00000002029	NA	NA	NA
ENSBTAG00000002030	0.1477813	0.8193554	0.0865277
ENSBTAG00000002033	-0.461308	0.1825033	0.7387292
ENSBTAG00000002034	NA	NA	NA
ENSBTAG00000002035	NA	NA	NA
ENSBTAG00000002036	-0.246717	0.3600568	0.443629
ENSBTAG00000002037	0.298163	0.3223421	0.491683
ENSBTAG00000002038	0.0847699	0.7430992	0.1289532
ENSBTAG00000002039	0.3140275	0.3894302	0.4095703
ENSBTAG00000002041	NA	NA	NA
ENSBTAG00000002042	0.0014435	0.9981038	0.0008243
ENSBTAG00000002044	-0.031994	0.9059183	0.0429109
ENSBTAG00000002045	0.2037288	0.4047447	0.3928188
ENSBTAG00000002046	0.1389764	0.5878703	0.2307185
ENSBTAG00000002048	-0.083486	0.7386826	0.1315422
ENSBTAG00000002049	0.3247512	0.2008603	0.697106
ENSBTAG00000002050	0.9052294	0.0435273	1.3612384
ENSBTAG00000002051	0.1638707	0.5915123	0.2280362
ENSBTAG00000002052	0.5627214	0.0352379	1.4529894
ENSBTAG00000002055	0.1198909	0.720398	0.1424275
ENSBTAG00000002056	0.4943878	0.2049511	0.6883498
ENSBTAG00000002058	0.0460036	0.8786467	0.0561857
ENSBTAG00000002059	0.2303032	0.5641208	0.2486278
ENSBTAG00000002060	0.0729059	0.7799922	0.1079098
ENSBTAG00000002062	NA	NA	NA
ENSBTAG00000002065	NA	NA	NA
ENSBTAG00000002068	-0.394444	0.1529747	0.8153803
ENSBTAG00000002069	1.2057668	0.0542342	1.2657265
ENSBTAG00000002070	NA	NA	NA
ENSBTAG00000002072	0.2126735	0.5468928	0.2620978
ENSBTAG00000002073	0.4350529	0.1440151	0.8415919
ENSBTAG00000002075	-0.13616	0.6411154	0.1930638
ENSBTAG00000002076	-0.247084	0.4307095	0.3658156
ENSBTAG00000002078	0.1876575	0.4713038	0.3266991
ENSBTAG00000002080	-0.408308	0.1831089	0.7372904
ENSBTAG00000002081	-0.802468	0.0331121	1.4800132
ENSBTAG00000002082	0.0181536	0.9446403	0.0247335
ENSBTAG00000002083	0.022224	0.947958	0.0232109
ENSBTAG00000002084	NA	NA	NA
ENSBTAG00000002086	0.0720538	0.7793277	0.1082799
ENSBTAG00000002087	NA	NA	NA
ENSBTAG00000002089	NA	NA	NA
ENSBTAG00000002090	0.1864542	0.5999186	0.2219076
ENSBTAG00000002092	0.0070582	0.9832524	0.007335
ENSBTAG00000002094	-0.091543	0.7411541	0.1300915
ENSBTAG00000002095	0.2037046	0.6439275	0.191163
ENSBTAG00000002096	-0.135615	0.758473	0.1200599
ENSBTAG00000002097	NA	NA	NA
ENSBTAG00000002098	-0.189657	0.4801505	0.3186226
ENSBTAG00000002099	NA	NA	NA
ENSBTAG00000002100	NA	NA	NA
ENSBTAG00000002101	-0.238332	0.3363085	0.4732621
ENSBTAG00000002103	-0.016559	0.9682617	0.0140072
ENSBTAG00000002104	-0.071068	0.9076396	0.0420866
ENSBTAG00000002105	0.0192136	0.9450214	0.0245584
ENSBTAG00000002107	0.401196	0.3378107	0.4713266

ENSBTAG00000002108	0.0071444	0.9783263	0.0095163
ENSBTAG00000002110	NA	NA	NA
ENSBTAG00000002112	0.1844633	0.5613229	0.2507873
ENSBTAG00000002113	-0.440244	0.2531744	0.5965802
ENSBTAG00000002115	NA	NA	NA
ENSBTAG00000002116	0.0842684	0.8491229	0.0710295
ENSBTAG00000002117	NA	NA	NA
ENSBTAG00000002121	0.3116778	0.4660303	0.3315858
ENSBTAG00000002122	0.7464349	0.0270367	1.5680469
ENSBTAG00000002123	0.5123918	0.487144	0.3123427
ENSBTAG00000002125	0.4162626	0.1126231	0.9483724
ENSBTAG00000002126	0.1338021	0.615553	0.2107346
ENSBTAG00000002127	0.414364	0.1440448	0.8415025
ENSBTAG00000002128	0.0338094	0.9369694	0.0282746
ENSBTAG00000002129	0.2379802	0.6439508	0.1911473
ENSBTAG00000002130	0.3963882	0.2293087	0.6395795
ENSBTAG00000002134	NA	NA	NA
ENSBTAG00000002135	-0.456334	0.4365313	0.3599846
ENSBTAG00000002136	NA	NA	NA
ENSBTAG00000002137	-0.180551	0.5356839	0.2710914
ENSBTAG00000002138	NA	NA	NA
ENSBTAG00000002143	-0.578739	0.2407551	0.6184244
ENSBTAG00000002144	-0.130391	0.6834878	0.1652692
ENSBTAG00000002145	NA	NA	NA
ENSBTAG00000002147	-1.053356	0.0028189	2.5499148
ENSBTAG00000002148	-0.16397	0.6020538	0.2203647
ENSBTAG00000002151	-0.435628	0.0874592	1.0581946
ENSBTAG00000002154	NA	NA	NA
ENSBTAG00000002157	0.6516691	0.0504807	1.2968747
ENSBTAG00000002158	NA	NA	NA
ENSBTAG00000002163	NA	NA	NA
ENSBTAG00000002164	NA	NA	NA
ENSBTAG00000002166	NA	NA	NA
ENSBTAG00000002170	0.016119	0.9650797	0.0154368
ENSBTAG00000002171	0.4366645	0.1462909	0.8347827
ENSBTAG00000002174	0.9897818	0.0044868	2.3480599
ENSBTAG00000002175	-0.283239	0.2469216	0.6074409
ENSBTAG00000002176	0.0546578	0.8341073	0.0787781
ENSBTAG00000002177	0.4969458	0.069468	1.1582151
ENSBTAG00000002178	0.1463441	0.6570077	0.1824295
ENSBTAG00000002179	-0.207879	0.5196743	0.2842688
ENSBTAG00000002181	-0.389044	0.4365356	0.3599804
ENSBTAG00000002182	0.0511498	0.841631	0.0748783
ENSBTAG00000002184	-0.230271	0.5015775	0.299662
ENSBTAG00000002185	-0.400524	0.1207	0.9182928
ENSBTAG00000002186	-0.161066	0.7005591	0.1545552
ENSBTAG00000002187	0.0215536	0.9321975	0.0304921
ENSBTAG00000002188	NA	NA	NA
ENSBTAG00000002189	0.338275	0.4281218	0.3684327
ENSBTAG00000002190	-0.03637	0.9174913	0.037398
ENSBTAG00000002191	0.3409212	0.2221389	0.6533754
ENSBTAG00000002192	NA	NA	NA
ENSBTAG00000002194	0.3905537	0.2491634	0.6035157
ENSBTAG00000002196	-0.031722	0.9030391	0.0442934
ENSBTAG00000002201	0.1195561	0.7134961	0.1466084
ENSBTAG00000002202	-0.560624	0.128277	0.8918513
ENSBTAG00000002203	-0.063591	0.8279999	0.0819697
ENSBTAG00000002206	NA	NA	NA

ENSBTAG00000002209	0.5493374	0.2281965	0.641691
ENSBTAG00000002210	-0.118223	0.6398733	0.193906
ENSBTAG00000002211	0.4526746	0.188834	0.7239198
ENSBTAG00000002214	NA	NA	NA
ENSBTAG00000002215	-0.363863	0.2611615	0.5830909
ENSBTAG00000002216	-0.403258	0.3879474	0.4112271
ENSBTAG00000002219	0.9805199	0.0055321	2.2571112
ENSBTAG00000002220	0.0754769	0.7670588	0.1151713
ENSBTAG00000002223	0.1266892	0.6957294	0.1575597
ENSBTAG00000002224	NA	NA	NA
ENSBTAG00000002226	0.4813321	0.0936298	1.028586
ENSBTAG00000002227	0.1001329	0.7795753	0.108142
ENSBTAG00000002231	NA	NA	NA
ENSBTAG00000002232	-0.17566	0.5962703	0.2245568
ENSBTAG00000002233	-0.061108	0.9122736	0.0398749
ENSBTAG00000002236	-0.035789	0.8891596	0.0510203
ENSBTAG00000002238	0.0630643	0.8043215	0.0945703
ENSBTAG00000002240	NA	NA	NA
ENSBTAG00000002242	0.0561644	0.8313335	0.0802247
ENSBTAG00000002243	0.0833392	0.8426974	0.0743283
ENSBTAG00000002248	NA	NA	NA
ENSBTAG00000002249	NA	NA	NA
ENSBTAG00000002251	NA	NA	NA
ENSBTAG00000002253	NA	NA	NA
ENSBTAG00000002255	NA	NA	NA
ENSBTAG00000002256	-0.092988	0.7933085	0.1005579
ENSBTAG00000002258	0.3648355	0.4581699	0.3389735
ENSBTAG00000002259	NA	NA	NA
ENSBTAG00000002260	0.1963263	0.5316886	0.2743427
ENSBTAG00000002261	0.3305516	0.369639	0.4322222
ENSBTAG00000002266	NA	NA	NA
ENSBTAG00000002267	-0.169448	0.4947539	0.3056108
ENSBTAG00000002271	0.2072307	0.4085384	0.3887671
ENSBTAG00000002272	0.5311978	0.1506882	0.8219209
ENSBTAG00000002273	NA	NA	NA
ENSBTAG00000002275	-0.174083	0.5652409	0.2477664
ENSBTAG00000002277	0.0418865	0.9172478	0.0375133
ENSBTAG00000002278	0.2020759	0.4547885	0.3421905
ENSBTAG00000002279	-0.154875	0.5349636	0.2716757
ENSBTAG00000002280	-0.428444	0.100958	0.9958591
ENSBTAG00000002281	0.1821457	0.7201985	0.1425478
ENSBTAG00000002282	0.0950492	0.7059658	0.1512163
ENSBTAG00000002283	NA	NA	NA
ENSBTAG00000002284	NA	NA	NA
ENSBTAG00000002286	0.244085	0.3668755	0.4354813
ENSBTAG00000002287	-0.017351	0.9467614	0.0237595
ENSBTAG00000002288	NA	NA	NA
ENSBTAG00000002289	NA	NA	NA
ENSBTAG00000002290	-0.877652	0.5573625	0.2538623
ENSBTAG00000002291	0.2456867	0.3850692	0.4144613
ENSBTAG00000002292	-0.326067	0.298843	0.5245569
ENSBTAG00000002293	-0.349097	0.1607736	0.7937854
ENSBTAG00000002294	-0.328275	0.7435353	0.1286984
ENSBTAG00000002295	0.0728835	0.7642978	0.1167374
ENSBTAG00000002296	0.2489037	0.5979724	0.2233189
ENSBTAG00000002297	NA	NA	NA
ENSBTAG00000002298	NA	NA	NA
ENSBTAG00000002299	-0.166121	0.5199068	0.2840745

ENSBTAG00000002302	0.8012705	0.0645485	1.1901138
ENSBTAG00000002303	0.3410772	0.1740198	0.7594013
ENSBTAG00000002305	-0.174	0.7052288	0.15167
ENSBTAG00000002306	-0.095208	0.7133491	0.1466979
ENSBTAG00000002309	NA	NA	NA
ENSBTAG00000002311	NA	NA	NA
ENSBTAG00000002313	0.9980075	0.0004443	3.3523403
ENSBTAG00000002315	0.5350709	0.0612219	1.2130935
ENSBTAG00000002316	-0.186679	0.6883236	0.1622073
ENSBTAG00000002317	-0.02749	0.9495901	0.0224638
ENSBTAG00000002319	0.1209812	0.6891264	0.1617011
ENSBTAG00000002321	0.0142307	0.9707195	0.0129062
ENSBTAG00000002323	-0.083212	0.7786471	0.1086594
ENSBTAG00000002326	0.1765399	0.5890772	0.2298278
ENSBTAG00000002327	-0.035573	0.9130867	0.039488
ENSBTAG00000002328	-0.180252	0.6053159	0.2180179
ENSBTAG00000002329	0.1506071	0.6681876	0.1751016
ENSBTAG00000002331	NA	NA	NA
ENSBTAG00000002332	-0.289696	0.3759175	0.4249074
ENSBTAG00000002333	-0.17466	0.7188297	0.143374
ENSBTAG00000002335	NA	NA	NA
ENSBTAG00000002336	-0.417056	0.1719743	0.7645364
ENSBTAG00000002340	1.6916079	0.0068403	2.1649261
ENSBTAG00000002341	0.2124064	0.4538789	0.34306
ENSBTAG00000002344	0.3223795	0.442646	0.3539435
ENSBTAG00000002345	0.048044	0.9135484	0.0392684
ENSBTAG00000002346	-0.658959	0.0863123	1.0639271
ENSBTAG00000002347	NA	NA	NA
ENSBTAG00000002348	-2.14171	9.90E-12	11.004537
ENSBTAG00000002350	0.077954	0.8761201	0.0574364
ENSBTAG00000002352	-0.290712	0.345871	0.4610859
ENSBTAG00000002355	NA	NA	NA
ENSBTAG00000002356	NA	NA	NA
ENSBTAG00000002357	0.4935425	0.1870815	0.7279691
ENSBTAG00000002361	0.0396806	0.9621679	0.0167491
ENSBTAG00000002362	-0.21042	0.6883112	0.1622152
ENSBTAG00000002363	NA	NA	NA
ENSBTAG00000002367	0.0039757	0.9896084	0.0045366
ENSBTAG00000002368	NA	NA	NA
ENSBTAG00000002369	NA	NA	NA
ENSBTAG00000002370	0.210434	0.5112601	0.2913581
ENSBTAG00000002374	0.7283403	0.1119057	0.9511477
ENSBTAG00000002376	-0.009908	0.9786844	0.0093573
ENSBTAG00000002377	0.2379895	0.3421096	0.4658348
ENSBTAG00000002378	0.0284358	0.9104896	0.040725
ENSBTAG00000002381	0.2139433	0.5006618	0.3004555
ENSBTAG00000002382	0.2958343	0.3835744	0.4161504
ENSBTAG00000002385	1.8762684	0.0007169	3.1445499
ENSBTAG00000002389	-0.149772	0.6284973	0.2016966
ENSBTAG00000002390	NA	NA	NA
ENSBTAG00000002391	0.3314188	0.4448098	0.3518257
ENSBTAG00000002392	-0.625336	0.0131622	1.8806724
ENSBTAG00000002393	0.3640903	0.1582766	0.8005832
ENSBTAG00000002394	0.2522504	0.5198897	0.2840888
ENSBTAG00000002395	-0.546523	0.0505437	1.2963326
ENSBTAG00000002398	-0.223971	0.5346195	0.2719552
ENSBTAG00000002402	0.3395062	0.1995562	0.6999347
ENSBTAG00000002404	0.0886325	0.7438881	0.1284924

ENSBTAG00000002408	NA	NA	NA
ENSBTAG00000002411	0.4157296	0.10842	0.9648904
ENSBTAG00000002412	0.6254281	0.0377699	1.4228542
ENSBTAG00000002413	-0.315417	0.2450621	0.6107238
ENSBTAG00000002414	NA	NA	NA
ENSBTAG00000002415	-0.324735	0.2019533	0.694749
ENSBTAG00000002416	-1.337996	0.1080165	0.9665101
ENSBTAG00000002417	0.4738195	0.0776721	1.109735
ENSBTAG00000002418	NA	NA	NA
ENSBTAG00000002419	0.6063559	0.1027226	0.9883341
ENSBTAG00000002422	0.2421024	0.6001509	0.2217396
ENSBTAG00000002423	0.0760273	0.7582762	0.1201725
ENSBTAG00000002425	-1.094784	0.0558726	1.252801
ENSBTAG00000002428	-0.017286	0.9503889	0.0220986
ENSBTAG00000002430	NA	NA	NA
ENSBTAG00000002431	0.1226915	0.6783912	0.1685198
ENSBTAG00000002432	NA	NA	NA
ENSBTAG00000002434	0.1279303	0.7047118	0.1519885
ENSBTAG00000002435	-0.040773	0.926448	0.033179
ENSBTAG00000002440	0.3421702	0.4119241	0.3851828
ENSBTAG00000002441	NA	NA	NA
ENSBTAG00000002444	-0.859803	0.0942022	1.0259392
ENSBTAG00000002445	-0.138984	0.7069054	0.1506387
ENSBTAG00000002447	NA	NA	NA
ENSBTAG00000002448	NA	NA	NA
ENSBTAG00000002450	NA	NA	NA
ENSBTAG00000002451	NA	NA	NA
ENSBTAG00000002452	0.2122307	0.4858632	0.313486
ENSBTAG00000002454	0.3805843	0.2641002	0.5782313
ENSBTAG00000002455	-0.283595	0.4054766	0.3920342
ENSBTAG00000002457	0.2435068	0.4043652	0.3932263
ENSBTAG00000002458	-0.049108	0.8432132	0.0740626
ENSBTAG00000002460	0.3136396	0.4371041	0.3594151
ENSBTAG00000002462	-0.069801	0.7922451	0.1011404
ENSBTAG00000002463	-0.188134	0.4737988	0.3244061
ENSBTAG00000002464	NA	NA	NA
ENSBTAG00000002467	-0.07781	0.8070041	0.0931243
ENSBTAG00000002468	0.2616425	0.306884	0.5130258
ENSBTAG00000002469	0.1346344	0.6621739	0.1790279
ENSBTAG00000002470	NA	NA	NA
ENSBTAG00000002471	0.9600883	0.0532646	1.2735612
ENSBTAG00000002472	-0.143619	0.744441	0.1281697
ENSBTAG00000002473	1.326333	0.0015532	2.8087653
ENSBTAG00000002474	0.3041193	0.3715851	0.4299417
ENSBTAG00000002475	0.4647587	0.1251179	0.9026804
ENSBTAG00000002476	0.2417252	0.3983499	0.3997352
ENSBTAG00000002477	-0.198486	0.5630708	0.249437
ENSBTAG00000002478	0.5010179	0.3324664	0.4782523
ENSBTAG00000002479	0.017688	0.9469065	0.0236929
ENSBTAG00000002480	-0.046474	0.9044485	0.0436161
ENSBTAG00000002481	-0.137738	0.6611946	0.1796707
ENSBTAG00000002483	NA	NA	NA
ENSBTAG00000002484	0.0624798	0.8219266	0.0851669
ENSBTAG00000002485	-0.284456	0.2760555	0.5590036
ENSBTAG00000002487	-0.204709	0.419338	0.3774358
ENSBTAG00000002488	NA	NA	NA
ENSBTAG00000002489	NA	NA	NA
ENSBTAG00000002490	-0.355726	0.1619073	0.7907337

ENSBTAG00000002492	NA	NA	NA
ENSBTAG00000002493	-0.304129	0.2171626	0.663215
ENSBTAG00000002495	0.4776313	0.0751938	1.1238178
ENSBTAG00000002497	NA	NA	NA
ENSBTAG00000002500	-0.057419	0.8691367	0.0609119
ENSBTAG00000002501	0.0406796	0.8731244	0.0589239
ENSBTAG00000002503	0.337583	0.3086969	0.5104677
ENSBTAG00000002504	0.0402083	0.8736776	0.0586488
ENSBTAG00000002507	-0.059649	0.8271531	0.0824141
ENSBTAG00000002508	NA	NA	NA
ENSBTAG00000002510	0.0717919	0.7972356	0.0984133
ENSBTAG00000002512	0.012094	0.9807835	0.0084269
ENSBTAG00000002515	NA	NA	NA
ENSBTAG00000002516	0.4896306	0.2059886	0.6861567
ENSBTAG00000002520	-0.098026	0.7008227	0.1543918
ENSBTAG00000002522	NA	NA	NA
ENSBTAG00000002524	0.2816629	0.4956294	0.3048429
ENSBTAG00000002525	0.045433	0.8840719	0.0535124
ENSBTAG00000002526	-0.171872	0.5491093	0.2603412
ENSBTAG00000002527	1.3910491	0.0037542	2.4254784
ENSBTAG00000002528	-0.011635	0.9627253	0.0164976
ENSBTAG00000002531	-0.139795	0.583171	0.234204
ENSBTAG00000002534	-0.45607	0.1447815	0.839287
ENSBTAG00000002539	-0.193614	0.5386151	0.2687215
ENSBTAG00000002542	-0.22802	0.4120381	0.3850626
ENSBTAG00000002549	0.1034707	0.6934524	0.1589833
ENSBTAG00000002551	0.1389183	0.6771369	0.1693235
ENSBTAG00000002554	0.1085219	0.6892864	0.1616003
ENSBTAG00000002555	NA	NA	NA
ENSBTAG00000002557	-0.219796	0.4196511	0.3771117
ENSBTAG00000002561	NA	NA	NA
ENSBTAG00000002562	0.2070907	0.5634344	0.2491567
ENSBTAG00000002563	0.5193377	0.0401569	1.3962403
ENSBTAG00000002564	NA	NA	NA
ENSBTAG00000002566	-0.499513	0.1230733	0.9098362
ENSBTAG00000002568	0.0761031	0.7944174	0.0999513
ENSBTAG00000002570	-0.131374	0.7094752	0.1490628
ENSBTAG00000002571	0.1541918	0.7053041	0.1516236
ENSBTAG00000002573	-0.074828	0.7725754	0.1120591
ENSBTAG00000002574	0.0445373	0.8784695	0.0562733
ENSBTAG00000002575	0.1051743	0.7109995	0.1481307
ENSBTAG00000002576	NA	NA	NA
ENSBTAG00000002578	-0.199913	0.4195573	0.3772087
ENSBTAG00000002579	-0.056907	0.8295998	0.0811313
ENSBTAG00000002580	0.2301968	0.3779132	0.422608
ENSBTAG00000002581	NA	NA	NA
ENSBTAG00000002582	NA	NA	NA
ENSBTAG00000002583	NA	NA	NA
ENSBTAG00000002585	0.2109144	0.6799342	0.1675331
ENSBTAG00000002586	0.0102973	0.9745132	0.0112123
ENSBTAG00000002590	0.1049828	0.7095199	0.1490354
ENSBTAG00000002591	-0.01462	0.9736349	0.0116039
ENSBTAG00000002593	NA	NA	NA
ENSBTAG00000002594	0.2137873	0.50806	0.294085
ENSBTAG00000002595	NA	NA	NA
ENSBTAG00000002596	NA	NA	NA
ENSBTAG00000002599	1.0601877	0.0236582	1.6260189
ENSBTAG00000002600	0.2327331	0.4670762	0.3306123

ENSBTAG00000002603	-0.086927	0.7427936	0.1291319
ENSBTAG00000002605	-0.705337	0.064201	1.192458
ENSBTAG00000002606	-0.531749	0.062231	1.2059933
ENSBTAG00000002608	0.1204563	0.6316574	0.1995184
ENSBTAG00000002610	-0.02988	0.9075062	0.0421504
ENSBTAG00000002612	NA	NA	NA
ENSBTAG00000002613	NA	NA	NA
ENSBTAG00000002614	-0.075461	0.7950576	0.0996014
ENSBTAG00000002615	0.7593383	0.0067658	2.1696791
ENSBTAG00000002620	NA	NA	NA
ENSBTAG00000002623	-0.002113	1	0
ENSBTAG00000002624	-0.603784	0.0273176	1.5635568
ENSBTAG00000002625	0.1523374	0.5465434	0.2623754
ENSBTAG00000002626	-0.303948	0.4010503	0.3968012
ENSBTAG00000002627	-0.047107	0.8529161	0.0690937
ENSBTAG00000002628	0.1070094	0.6651866	0.1770565
ENSBTAG00000002629	NA	NA	NA
ENSBTAG00000002630	-0.463032	0.1696259	0.7705077
ENSBTAG00000002633	0.5787493	0.0590681	1.228647
ENSBTAG00000002634	0.6972948	0.0249383	1.6031324
ENSBTAG00000002639	-0.011871	0.9632553	0.0162586
ENSBTAG00000002640	0.6684537	0.0234711	1.6294667
ENSBTAG00000002641	0.7747536	0.1028844	0.9876506
ENSBTAG00000002642	NA	NA	NA
ENSBTAG00000002643	0.5149355	0.1300647	0.8858407
ENSBTAG00000002644	1.0625902	0.0007317	3.1356812
ENSBTAG00000002645	-0.053739	0.874807	0.0580877
ENSBTAG00000002646	-0.02029	0.9346802	0.029337
ENSBTAG00000002647	NA	NA	NA
ENSBTAG00000002648	0.1103026	0.6689571	0.1746017
ENSBTAG00000002650	-0.090346	0.7513643	0.1241494
ENSBTAG00000002651	NA	NA	NA
ENSBTAG00000002654	-0.304796	0.2507353	0.6007846
ENSBTAG00000002655	-0.168919	0.7522592	0.1236325
ENSBTAG00000002657	0.0988991	0.7117246	0.147688
ENSBTAG00000002658	-0.330227	0.406801	0.390618
ENSBTAG00000002660	NA	NA	NA
ENSBTAG00000002661	NA	NA	NA
ENSBTAG00000002663	-0.312279	0.3054571	0.5150498
ENSBTAG00000002664	NA	NA	NA
ENSBTAG00000002668	NA	NA	NA
ENSBTAG00000002669	0.2665068	0.5756523	0.2398397
ENSBTAG00000002670	1.4095957	0.0020335	2.6917488
ENSBTAG00000002673	-0.044781	0.9271294	0.0328596
ENSBTAG00000002674	NA	NA	NA
ENSBTAG00000002676	-0.269484	0.3340627	0.476172
ENSBTAG00000002678	0.1917586	0.630958	0.1999996
ENSBTAG00000002680	NA	NA	NA
ENSBTAG00000002682	0.0702859	0.883516	0.0537856
ENSBTAG00000002683	0.3389448	0.2712533	0.566625
ENSBTAG00000002685	-0.234145	0.4288831	0.3676611
ENSBTAG00000002687	NA	NA	NA
ENSBTAG00000002688	0.3982291	0.1877968	0.7263119
ENSBTAG00000002689	-0.062614	0.8526043	0.0692525
ENSBTAG00000002690	-0.098194	0.6955587	0.1576662
ENSBTAG00000002691	NA	NA	NA
ENSBTAG00000002693	-0.026893	0.9193015	0.036542
ENSBTAG00000002695	NA	NA	NA

ENSBTAG00000002697	0.3085362	0.3346122	0.4754582
ENSBTAG00000002698	-0.056378	0.8622697	0.0643569
ENSBTAG00000002699	0.3099886	0.3623537	0.4408673
ENSBTAG00000002701	NA	NA	NA
ENSBTAG00000002702	NA	NA	NA
ENSBTAG00000002703	-0.145025	0.5918	0.2278251
ENSBTAG00000002704	0.3201502	0.3304559	0.4808865
ENSBTAG00000002705	NA	NA	NA
ENSBTAG00000002706	-0.176352	0.6026073	0.2199656
ENSBTAG00000002707	0.102784	0.7264297	0.1388064
ENSBTAG00000002708	0.1637356	0.5705904	0.2436756
ENSBTAG00000002709	-0.048742	0.8743009	0.0583391
ENSBTAG00000002710	NA	NA	NA
ENSBTAG00000002712	-0.118981	0.7546665	0.1222449
ENSBTAG00000002714	0.2514333	0.5299162	0.2757928
ENSBTAG00000002715	0.109631	0.7724265	0.1121428
ENSBTAG00000002716	-0.187353	0.5978778	0.2233875
ENSBTAG00000002717	NA	NA	NA
ENSBTAG00000002718	0.2588896	0.4316084	0.3649102
ENSBTAG00000002719	NA	NA	NA
ENSBTAG00000002720	0.0572457	0.8393189	0.076073
ENSBTAG00000002721	-0.194768	0.4664388	0.3312053
ENSBTAG00000002724	NA	NA	NA
ENSBTAG00000002725	0.2082611	0.4375472	0.3589751
ENSBTAG00000002726	-0.119153	0.6376274	0.195433
ENSBTAG00000002727	0.0127951	0.9726829	0.0120287
ENSBTAG00000002728	-0.183513	0.602387	0.2201244
ENSBTAG00000002730	0.0120492	0.9671511	0.0145057
ENSBTAG00000002733	NA	NA	NA
ENSBTAG00000002734	-0.028867	0.9096283	0.0411361
ENSBTAG00000002735	-0.12842	0.6598364	0.1805637
ENSBTAG00000002736	0.3108064	0.3602217	0.4434301
ENSBTAG00000002737	NA	NA	NA
ENSBTAG00000002738	0.37662	0.3604186	0.4431928
ENSBTAG00000002739	-0.198908	0.5170295	0.2864847
ENSBTAG00000002742	-0.114724	0.7064644	0.1509097
ENSBTAG00000002743	-0.089966	0.7533112	0.1230256
ENSBTAG00000002744	0.0568241	0.9071177	0.0423364
ENSBTAG00000002745	0.0473128	0.8489482	0.0711188
ENSBTAG00000002746	-0.002553	0.9932582	0.0029378
ENSBTAG00000002747	0.1304165	0.6433079	0.1915811
ENSBTAG00000002748	0.2293963	0.5648295	0.2480826
ENSBTAG00000002749	-0.282896	0.4861155	0.3132605
ENSBTAG00000002750	-0.075527	0.7736157	0.1114747
ENSBTAG00000002751	NA	NA	NA
ENSBTAG00000002755	-0.579018	0.0337215	1.4720933
ENSBTAG00000002756	NA	NA	NA
ENSBTAG00000002758	0.1241226	0.699078	0.1554744
ENSBTAG00000002763	0.1895135	0.560643	0.2513136
ENSBTAG00000002765	NA	NA	NA
ENSBTAG00000002767	0.5019933	0.2196452	0.6582784
ENSBTAG00000002768	0.2044994	0.5640819	0.2486579
ENSBTAG00000002769	-0.127568	0.5994634	0.2222373
ENSBTAG00000002770	0.4620678	0.1020835	0.9910445
ENSBTAG00000002772	NA	NA	NA
ENSBTAG00000002773	NA	NA	NA
ENSBTAG00000002774	0.3875853	0.3365069	0.473006
ENSBTAG00000002778	0.0460947	0.855993	0.0675298

ENSBTAG00000002781	0.7634856	0.1490474	0.8266755
ENSBTAG00000002782	0.1309636	0.6455616	0.1900623
ENSBTAG00000002783	-0.570525	0.0289659	1.5381131
ENSBTAG00000002784	-0.194587	0.4365115	0.3600043
ENSBTAG00000002786	NA	NA	NA
ENSBTAG00000002788	0.0605704	0.8890644	0.0510668
ENSBTAG00000002791	-0.051006	0.8451535	0.0730644
ENSBTAG00000002792	0.9134154	0.0132271	1.8785348
ENSBTAG00000002795	-0.024314	0.9341352	0.0295903
ENSBTAG00000002796	-0.381874	0.3161285	0.5001363
ENSBTAG00000002798	0.3126376	0.2787318	0.5548134
ENSBTAG00000002799	NA	NA	NA
ENSBTAG00000002803	NA	NA	NA
ENSBTAG00000002804	0.4244331	0.1108408	0.9553004
ENSBTAG00000002808	-0.144158	0.5866572	0.2316156
ENSBTAG00000002809	0.0378705	0.9236177	0.0345077
ENSBTAG00000002810	0.2869854	0.4660068	0.3316077
ENSBTAG00000002813	-0.029273	0.9271361	0.0328565
ENSBTAG00000002816	0.1727901	0.7240373	0.1402391
ENSBTAG00000002817	NA	NA	NA
ENSBTAG00000002820	-0.109532	0.6589583	0.181142
ENSBTAG00000002821	-0.457667	0.3963233	0.4019504
ENSBTAG00000002822	NA	NA	NA
ENSBTAG00000002823	0.1441392	0.5666726	0.2466678
ENSBTAG00000002824	NA	NA	NA
ENSBTAG00000002826	NA	NA	NA
ENSBTAG00000002827	-0.100233	0.8233163	0.0844333
ENSBTAG00000002828	0.1001035	0.7375934	0.132183
ENSBTAG00000002829	-0.09502	0.7048958	0.1518751
ENSBTAG00000002830	0.2924174	0.2708663	0.5672449
ENSBTAG00000002833	0.3909987	0.2363463	0.6264512
ENSBTAG00000002834	0.135791	0.6297778	0.2008126
ENSBTAG00000002835	-0.051619	0.8664895	0.0622367
ENSBTAG00000002836	0.0558913	0.88089	0.0550783
ENSBTAG00000002837	0.2425605	0.6389944	0.194503
ENSBTAG00000002842	0.8038881	0.099186	1.0035498
ENSBTAG00000002843	-0.426827	0.2360836	0.6269343
ENSBTAG00000002844	NA	NA	NA
ENSBTAG00000002845	NA	NA	NA
ENSBTAG00000002846	-0.55233	0.2414521	0.617169
ENSBTAG00000002847	-0.131967	0.6187119	0.2085115
ENSBTAG00000002848	-0.184841	0.6214655	0.206583
ENSBTAG00000002849	NA	NA	NA
ENSBTAG00000002850	NA	NA	NA
ENSBTAG00000002853	-0.056405	0.8251522	0.0834659
ENSBTAG00000002854	0.3215693	0.3002598	0.5225028
ENSBTAG00000002855	NA	NA	NA
ENSBTAG00000002858	0.0095265	0.9918908	0.0035361
ENSBTAG00000002859	NA	NA	NA
ENSBTAG00000002863	0.0020493	0.9940116	0.0026085
ENSBTAG00000002865	NA	NA	NA
ENSBTAG00000002866	-0.35958	0.232784	0.6330469
ENSBTAG00000002869	-0.588081	0.0922636	1.0349694
ENSBTAG00000002871	0.1527062	0.5833396	0.2340785
ENSBTAG00000002874	-0.11732	0.6912206	0.1603833
ENSBTAG00000002878	-0.265071	0.5525669	0.2576151
ENSBTAG00000002879	0.2204141	0.5537061	0.2567207
ENSBTAG00000002880	0.1135392	0.6560893	0.1830371

ENSBTAG00000002881	0.1574541	0.6503352	0.1868627
ENSBTAG00000002882	-0.103466	0.8404103	0.0755086
ENSBTAG00000002883	0.6799381	0.0218133	1.6612786
ENSBTAG00000002885	-0.216637	0.6534057	0.1848171
ENSBTAG00000002887	NA	NA	NA
ENSBTAG00000002888	0.077824	0.8041249	0.0946765
ENSBTAG00000002890	0.2709696	0.3189381	0.4962937
ENSBTAG00000002894	NA	NA	NA
ENSBTAG00000002895	NA	NA	NA
ENSBTAG00000002896	0.0886669	0.7200077	0.1426628
ENSBTAG00000002898	-0.024599	0.9233849	0.0346172
ENSBTAG00000002902	-0.174565	0.5762492	0.2393897
ENSBTAG00000002904	0.2341845	0.4879555	0.3116197
ENSBTAG00000002907	0.1462629	0.6161772	0.2102944
ENSBTAG00000002908	NA	NA	NA
ENSBTAG00000002910	0.0757881	0.8236854	0.0842387
ENSBTAG00000002912	NA	NA	NA
ENSBTAG00000002914	-0.10417	0.7605677	0.1188621
ENSBTAG00000002915	NA	NA	NA
ENSBTAG00000002917	0.562456	0.0791007	1.1018194
ENSBTAG00000002918	0.0295702	0.9636534	0.0160791
ENSBTAG00000002919	NA	NA	NA
ENSBTAG00000002920	0.0324883	0.9049845	0.0433589
ENSBTAG00000002921	0.0188037	0.953532	0.0206647
ENSBTAG00000002922	0.3717029	0.2391731	0.6212877
ENSBTAG00000002923	0.4246125	0.412634	0.384435
ENSBTAG00000002924	-0.636626	0.0167086	1.7770612
ENSBTAG00000002929	NA	NA	NA
ENSBTAG00000002931	-0.186318	0.4880541	0.311532
ENSBTAG00000002935	0.2647869	0.3809438	0.4191391
ENSBTAG00000002936	NA	NA	NA
ENSBTAG00000002937	NA	NA	NA
ENSBTAG00000002938	0.9646195	0.0066557	2.1768072
ENSBTAG00000002939	0.3899945	0.1777559	0.7501759
ENSBTAG00000002940	NA	NA	NA
ENSBTAG00000002941	0.2994559	0.4584669	0.338692
ENSBTAG00000002942	NA	NA	NA
ENSBTAG00000002943	NA	NA	NA
ENSBTAG00000002944	-0.049825	0.8697264	0.0606173
ENSBTAG00000002947	-0.405084	0.3741585	0.4269444
ENSBTAG00000002948	0.1719543	0.5031884	0.2982694
ENSBTAG00000002949	-0.393493	0.1798836	0.7450084
ENSBTAG00000002950	NA	NA	NA
ENSBTAG00000002951	-0.298758	0.4559215	0.34111
ENSBTAG00000002952	NA	NA	NA
ENSBTAG00000002953	-0.03281	0.9006013	0.0454674
ENSBTAG00000002955	-0.340291	0.5076131	0.2944672
ENSBTAG00000002956	-0.045491	0.9011651	0.0451956
ENSBTAG00000002959	-0.273474	0.4552084	0.3417897
ENSBTAG00000002960	0.6856581	0.0103029	1.9870402
ENSBTAG00000002962	0.2505148	0.4777596	0.3207906
ENSBTAG00000002963	NA	NA	NA
ENSBTAG00000002964	-0.445918	0.0872276	1.059346
ENSBTAG00000002966	-0.132572	0.6010473	0.2210914
ENSBTAG00000002970	-0.651019	0.0218944	1.6596678
ENSBTAG00000002971	-0.14406	0.5864861	0.2317423
ENSBTAG00000002972	-0.333312	0.5299554	0.2757607
ENSBTAG00000002973	0.1541671	0.5603013	0.2515783

ENSBTAG00000002977	0.192072	0.5017233	0.2995357
ENSBTAG00000002978	0.7058727	0.1501818	0.8233827
ENSBTAG00000002979	-0.245535	0.5608601	0.2511455
ENSBTAG00000002980	-0.062538	0.8118548	0.0905217
ENSBTAG00000002981	NA	NA	NA
ENSBTAG00000002982	NA	NA	NA
ENSBTAG00000002983	0.0043109	0.9906865	0.0040637
ENSBTAG00000002985	-0.201563	0.6808323	0.1669598
ENSBTAG00000002988	0.0580148	0.8198569	0.0862619
ENSBTAG00000002993	-0.028952	0.9341741	0.0295722
ENSBTAG00000002995	0.4575278	0.1420329	0.8476111
ENSBTAG00000002996	0.1152528	0.6764025	0.1697948
ENSBTAG00000002997	-0.530568	0.2759233	0.5592117
ENSBTAG00000002998	NA	NA	NA
ENSBTAG00000002999	-0.1018	0.6989391	0.1555607
ENSBTAG00000003000	NA	NA	NA
ENSBTAG00000003001	0.1520634	0.57753	0.2384254
ENSBTAG00000003002	0.2741478	0.3458115	0.4611606
ENSBTAG00000003012	0.1791752	0.6869407	0.1630808
ENSBTAG00000003014	-0.184071	0.6984911	0.1558391
ENSBTAG00000003015	-0.676806	0.0509252	1.293067
ENSBTAG00000003016	NA	NA	NA
ENSBTAG00000003017	0.3594021	0.4168234	0.380048
ENSBTAG00000003018	-0.018926	0.9696053	0.013405
ENSBTAG00000003019	NA	NA	NA
ENSBTAG00000003020	NA	NA	NA
ENSBTAG00000003021	0.1848822	0.5078328	0.2942793
ENSBTAG00000003025	-0.297768	0.345871	0.4610858
ENSBTAG00000003027	NA	NA	NA
ENSBTAG00000003033	-0.211107	0.7084958	0.1496627
ENSBTAG00000003034	0.2597267	0.3791252	0.4212173
ENSBTAG00000003035	-0.200559	0.5317966	0.2742544
ENSBTAG00000003036	NA	NA	NA
ENSBTAG00000003037	0.8439811	0.0470839	1.3271276
ENSBTAG00000003038	0.5382335	0.094043	1.0266736
ENSBTAG00000003039	0.4201303	0.2007262	0.6973959
ENSBTAG00000003040	-0.094813	0.7018801	0.1537371
ENSBTAG00000003043	0.5042174	0.2312169	0.6359803
ENSBTAG00000003044	-0.250902	0.3351898	0.4747092
ENSBTAG00000003045	0.4187249	0.3635034	0.4394915
ENSBTAG00000003047	NA	NA	NA
ENSBTAG00000003049	0.0423273	0.921177	0.0356569
ENSBTAG00000003051	-0.088568	0.7428874	0.129077
ENSBTAG00000003052	0.2130102	0.4906606	0.3092188
ENSBTAG00000003054	NA	NA	NA
ENSBTAG00000003056	NA	NA	NA
ENSBTAG00000003058	0.2026939	0.4250832	0.3715261
ENSBTAG00000003059	0.1165582	0.67744	0.1691291
ENSBTAG00000003060	NA	NA	NA
ENSBTAG00000003061	0.1741789	0.571823	0.2427384
ENSBTAG00000003062	0.1253709	0.6353174	0.1970092
ENSBTAG00000003063	0.1371771	0.6206053	0.2071845
ENSBTAG00000003064	-0.153921	0.5836235	0.2338673
ENSBTAG00000003066	-0.16813	0.4931309	0.3070378
ENSBTAG00000003067	-0.088328	0.7246083	0.1398967
ENSBTAG00000003068	-0.554121	0.0777748	1.109161
ENSBTAG00000003069	0.4595386	0.2282328	0.6416219
ENSBTAG00000003072	0.4894252	0.0567226	1.2462439

ENSBTAG00000003073	0.8356384	0.0656712	1.1826254
ENSBTAG00000003074	-0.640516	0.132573	0.8775449
ENSBTAG00000003075	-0.141565	0.6197735	0.207767
ENSBTAG00000003076	NA	NA	NA
ENSBTAG00000003077	0.2626531	0.4046761	0.3928925
ENSBTAG00000003078	NA	NA	NA
ENSBTAG00000003079	NA	NA	NA
ENSBTAG00000003081	-0.260668	0.3067016	0.5132839
ENSBTAG00000003083	-0.284684	0.5627445	0.2496887
ENSBTAG00000003084	-0.077411	0.7564508	0.1212193
ENSBTAG00000003086	NA	NA	NA
ENSBTAG00000003087	-0.341269	0.1789551	0.7472559
ENSBTAG00000003088	NA	NA	NA
ENSBTAG00000003089	0.0542635	0.9279528	0.0324741
ENSBTAG00000003090	NA	NA	NA
ENSBTAG00000003092	-0.057622	0.8484994	0.0713485
ENSBTAG00000003093	NA	NA	NA
ENSBTAG00000003094	0.1909958	0.4718798	0.3261686
ENSBTAG00000003097	0.0093181	0.9725459	0.0120899
ENSBTAG00000003098	-0.522925	0.0392014	1.4066989
ENSBTAG00000003100	-0.039078	0.8800642	0.0554856
ENSBTAG00000003101	NA	NA	NA
ENSBTAG00000003102	0.4073857	0.3507987	0.4549421
ENSBTAG00000003107	NA	NA	NA
ENSBTAG00000003109	0.3571426	0.1516733	0.8190908
ENSBTAG00000003110	NA	NA	NA
ENSBTAG00000003111	-0.021465	0.9337944	0.0297487
ENSBTAG00000003112	0.1031316	0.677079	0.1693606
ENSBTAG00000003113	0.2827686	0.5731962	0.2416967
ENSBTAG00000003114	0.0896193	0.7542031	0.1225117
ENSBTAG00000003115	-0.229081	0.4608906	0.3364021
ENSBTAG00000003116	-0.077711	0.7796796	0.1080838
ENSBTAG00000003120	-0.593255	0.1029829	0.987235
ENSBTAG00000003121	NA	NA	NA
ENSBTAG00000003124	0.1194837	0.6846916	0.164505
ENSBTAG00000003126	0.3066297	0.3467686	0.4599602
ENSBTAG00000003128	0.3330594	0.6194978	0.2079603
ENSBTAG00000003130	NA	NA	NA
ENSBTAG00000003131	NA	NA	NA
ENSBTAG00000003132	NA	NA	NA
ENSBTAG00000003137	-1.772378	0.0003445	3.4628461
ENSBTAG00000003140	NA	NA	NA
ENSBTAG00000003143	0.1081378	0.7967763	0.0986636
ENSBTAG00000003144	0.1308778	0.7461244	0.1271888
ENSBTAG00000003147	0.1500241	0.5440001	0.2644011
ENSBTAG00000003148	0.1237212	0.6301797	0.2005356
ENSBTAG00000003151	-0.163127	0.6482034	0.1882887
ENSBTAG00000003152	0.6720511	0.3735867	0.4276086
ENSBTAG00000003155	0.4965089	0.2982063	0.5254832
ENSBTAG00000003160	-0.135413	0.7052648	0.1516478
ENSBTAG00000003161	-0.214393	0.4847464	0.3144854
ENSBTAG00000003162	-0.435141	0.0805528	1.0939196
ENSBTAG00000003164	NA	NA	NA
ENSBTAG00000003165	-0.498526	0.190725	0.7195923
ENSBTAG00000003166	0.037285	0.9037684	0.0439428
ENSBTAG00000003168	-0.112916	0.6475418	0.1887322
ENSBTAG00000003169	NA	NA	NA
ENSBTAG00000003171	NA	NA	NA

ENSBTAG00000003172	0.9150918	0.068631	1.16348
ENSBTAG00000003174	0.7170223	0.0568545	1.2452353
ENSBTAG00000003176	0.314564	0.2528244	0.597181
ENSBTAG00000003177	-0.403253	0.3297042	0.4818755
ENSBTAG00000003178	0.1531167	0.539483	0.2680223
ENSBTAG00000003180	0.0426697	0.8859824	0.0525749
ENSBTAG00000003183	-0.40553	0.1964811	0.7066793
ENSBTAG00000003184	NA	NA	NA
ENSBTAG00000003185	-0.156623	0.6950178	0.1580041
ENSBTAG00000003186	0.1511388	0.6436525	0.1913485
ENSBTAG00000003189	-0.274089	0.2894919	0.5383636
ENSBTAG00000003191	0.0070693	0.9897597	0.0044702
ENSBTAG00000003192	-0.406749	0.2762754	0.5586579
ENSBTAG00000003193	0.7692265	0.119547	0.9224612
ENSBTAG00000003196	0.8499779	0.0825523	1.0832706
ENSBTAG00000003199	NA	NA	NA
ENSBTAG00000003200	-0.752747	0.0143395	1.8434664
ENSBTAG00000003201	-0.128695	0.6119457	0.2132871
ENSBTAG00000003202	NA	NA	NA
ENSBTAG00000003205	0.0747387	0.7777125	0.1091809
ENSBTAG00000003207	NA	NA	NA
ENSBTAG00000003208	0.2005249	0.4641593	0.3333329
ENSBTAG00000003209	0.0277609	0.9134119	0.0393334
ENSBTAG00000003212	0.3052705	0.5242243	0.2804829
ENSBTAG00000003215	0.2771331	0.3969298	0.4012862
ENSBTAG00000003217	-0.058674	0.9190541	0.0366589
ENSBTAG00000003218	0.1779275	0.570735	0.2435655
ENSBTAG00000003219	0.3549312	0.4386178	0.3579138
ENSBTAG00000003220	NA	NA	NA
ENSBTAG00000003221	-0.501278	0.0864083	1.0634447
ENSBTAG00000003222	0.0174018	0.9721397	0.0122713
ENSBTAG00000003225	0.090248	0.7638864	0.1169712
ENSBTAG00000003228	-0.155629	0.5734656	0.2414927
ENSBTAG00000003229	-0.00054	0.9983846	0.0007021
ENSBTAG00000003231	NA	NA	NA
ENSBTAG00000003232	0.2032873	0.6515994	0.1860193
ENSBTAG00000003235	0.1714743	0.5997484	0.2220309
ENSBTAG00000003237	0.1173776	0.7042376	0.1522808
ENSBTAG00000003238	0.7980378	0.0031967	2.4952928
ENSBTAG00000003239	0.0136258	0.9584069	0.0184501
ENSBTAG00000003240	NA	NA	NA
ENSBTAG00000003242	0.3363407	0.3365572	0.4729411
ENSBTAG00000003245	-0.105454	0.7759063	0.1101907
ENSBTAG00000003250	NA	NA	NA
ENSBTAG00000003253	-1.77355	0.0030255	2.5191974
ENSBTAG00000003255	NA	NA	NA
ENSBTAG00000003256	-0.553473	0.2140138	0.6695582
ENSBTAG00000003257	0.1838017	0.6421569	0.1923588
ENSBTAG00000003259	-0.120876	0.6423238	0.192246
ENSBTAG00000003261	NA	NA	NA
ENSBTAG00000003263	-0.162831	0.6013174	0.2208963
ENSBTAG00000003264	0.036729	0.8919508	0.0496591
ENSBTAG00000003265	0.2030111	0.4132428	0.3837947
ENSBTAG00000003267	NA	NA	NA
ENSBTAG00000003268	0.291675	0.2953327	0.5296885
ENSBTAG00000003275	-0.051942	0.8402381	0.0755976
ENSBTAG00000003276	-0.160983	0.5703575	0.2438528
ENSBTAG00000003278	0.520423	0.2773143	0.5570277

ENSBTAG00000003279	-0.116276	0.6887436	0.1619424
ENSBTAG00000003280	0.4439024	0.4264811	0.3701002
ENSBTAG00000003282	NA	NA	NA
ENSBTAG00000003288	-0.139046	0.6768439	0.1695115
ENSBTAG00000003289	NA	NA	NA
ENSBTAG00000003290	-0.422293	0.404159	0.3934477
ENSBTAG00000003291	-0.259951	0.5129057	0.2899625
ENSBTAG00000003294	0.1014905	0.7295173	0.1369644
ENSBTAG00000003295	-0.045462	0.8910907	0.0500781
ENSBTAG00000003296	0.1145319	0.6752192	0.1705552
ENSBTAG00000003297	NA	NA	NA
ENSBTAG00000003298	-0.153193	0.6279005	0.2021092
ENSBTAG00000003299	NA	NA	NA
ENSBTAG00000003300	0.8870893	0.0032334	2.4903426
ENSBTAG00000003301	NA	NA	NA
ENSBTAG00000003303	-0.025073	0.9205468	0.0359541
ENSBTAG00000003304	NA	NA	NA
ENSBTAG00000003305	NA	NA	NA
ENSBTAG00000003307	0.0743608	0.7694518	0.1138186
ENSBTAG00000003308	NA	NA	NA
ENSBTAG00000003312	0.1432724	0.7092155	0.1492218
ENSBTAG00000003313	NA	NA	NA
ENSBTAG00000003314	NA	NA	NA
ENSBTAG00000003315	0.0020152	0.9958723	0.0017963
ENSBTAG00000003316	-0.345245	0.2088834	0.6800961
ENSBTAG00000003319	NA	NA	NA
ENSBTAG00000003321	NA	NA	NA
ENSBTAG00000003322	-0.667208	0.0085766	2.0666855
ENSBTAG00000003323	1.0595405	0.0301046	1.521367
ENSBTAG00000003325	-0.349175	0.2151495	0.6672596
ENSBTAG00000003326	0.2937022	0.5238379	0.2808031
ENSBTAG00000003327	NA	NA	NA
ENSBTAG00000003328	-0.437143	0.301099	0.5212907
ENSBTAG00000003329	-0.123567	0.7745505	0.1109503
ENSBTAG00000003330	0.3393014	0.1912046	0.7185016
ENSBTAG00000003332	0.3580744	0.336194	0.47341
ENSBTAG00000003334	0.5264386	0.1577971	0.8019009
ENSBTAG00000003336	NA	NA	NA
ENSBTAG00000003338	-0.672135	0.026987	1.5688455
ENSBTAG00000003339	0.1869661	0.4563024	0.3407473
ENSBTAG00000003340	-0.306636	0.3210391	0.4934421
ENSBTAG00000003341	NA	NA	NA
ENSBTAG00000003342	0.2752205	0.3005798	0.5220403
ENSBTAG00000003345	0.5603448	0.0830369	1.0807289
ENSBTAG00000003349	NA	NA	NA
ENSBTAG00000003352	0.0630647	0.9562638	0.0194223
ENSBTAG00000003353	0.4131144	0.3898283	0.4091266
ENSBTAG00000003354	0.1605534	0.5216057	0.2826577
ENSBTAG00000003357	NA	NA	NA
ENSBTAG00000003358	0.0739431	0.7897098	0.1025325
ENSBTAG00000003359	0.3508895	0.394983	0.4034216
ENSBTAG00000003361	0.2048925	0.5128689	0.2899936
ENSBTAG00000003362	-0.019051	0.9405298	0.0266274
ENSBTAG00000003365	0.1126136	0.7344222	0.1340542
ENSBTAG00000003366	0.4801708	0.1091216	0.9620892
ENSBTAG00000003369	NA	NA	NA
ENSBTAG00000003370	NA	NA	NA
ENSBTAG00000003371	0.1279849	0.7317975	0.135609

ENSBTAG00000003372	NA	NA	NA
ENSBTAG00000003375	0.1133435	0.7716525	0.1125782
ENSBTAG00000003376	1.1995452	2.07E-05	4.6844894
ENSBTAG00000003378	-0.083238	0.7431343	0.1289327
ENSBTAG00000003381	-0.052041	0.8893363	0.050934
ENSBTAG00000003383	NA	NA	NA
ENSBTAG00000003384	0.422639	0.1933288	0.7137034
ENSBTAG00000003386	0.2617512	0.334571	0.4755117
ENSBTAG00000003387	NA	NA	NA
ENSBTAG00000003388	0.0974483	0.8384133	0.0765418
ENSBTAG00000003390	0.5500677	0.1052646	0.9777175
ENSBTAG00000003393	NA	NA	NA
ENSBTAG00000003394	-0.188837	0.6002461	0.2216706
ENSBTAG00000003395	-0.324797	0.2164263	0.66469
ENSBTAG00000003396	0.052536	0.9143279	0.038898
ENSBTAG00000003397	-0.058149	0.8343775	0.0786374
ENSBTAG00000003398	NA	NA	NA
ENSBTAG00000003399	-0.235869	0.3881448	0.4110062
ENSBTAG00000003401	-0.149606	0.5561821	0.254783
ENSBTAG00000003403	0.3975319	0.3143346	0.5026078
ENSBTAG00000003404	NA	NA	NA
ENSBTAG00000003405	NA	NA	NA
ENSBTAG00000003407	0.0614733	0.8256334	0.0832127
ENSBTAG00000003408	NA	NA	NA
ENSBTAG00000003410	-0.112172	0.6544298	0.184137
ENSBTAG00000003414	NA	NA	NA
ENSBTAG00000003415	-0.051019	0.8393751	0.0760439
ENSBTAG00000003417	0.6855595	0.0697261	1.1566049
ENSBTAG00000003418	0.0369463	0.8870984	0.0520282
ENSBTAG00000003419	0.311444	0.2351377	0.6286777
ENSBTAG00000003421	0.2644302	0.3670743	0.435246
ENSBTAG00000003422	-0.136839	0.5809202	0.2358835
ENSBTAG00000003423	-0.089107	0.7166499	0.144693
ENSBTAG00000003424	-0.052019	0.8773166	0.0568436
ENSBTAG00000003425	-0.048535	0.9116541	0.0401699
ENSBTAG00000003432	NA	NA	NA
ENSBTAG00000003434	NA	NA	NA
ENSBTAG00000003436	0.8361527	0.0926645	1.0330868
ENSBTAG00000003438	1.0339022	0.0107426	1.968889
ENSBTAG00000003439	-0.038552	0.8913874	0.0499335
ENSBTAG00000003440	-1.727145	0.0034048	2.4679058
ENSBTAG00000003443	0.071707	0.8254608	0.0833035
ENSBTAG00000003444	-0.343393	0.3204497	0.4942401
ENSBTAG00000003446	0.2008288	0.5447051	0.2638386
ENSBTAG00000003447	0.2001542	0.5764648	0.2392272
ENSBTAG00000003449	NA	NA	NA
ENSBTAG00000003450	0.1281979	0.6653327	0.1769612
ENSBTAG00000003454	NA	NA	NA
ENSBTAG00000003455	0.1879453	0.6465848	0.1893745
ENSBTAG00000003456	0.1028897	0.699432	0.1552545
ENSBTAG00000003457	-0.363501	0.4188491	0.3779424
ENSBTAG00000003458	NA	NA	NA
ENSBTAG00000003460	0.1258863	0.635305	0.1970177
ENSBTAG00000003462	0.5529529	0.1872791	0.7275107
ENSBTAG00000003469	-0.309034	0.2273533	0.6432988
ENSBTAG00000003470	0.7010893	0.165546	0.7810813
ENSBTAG00000003472	NA	NA	NA
ENSBTAG00000003474	NA	NA	NA

ENSBTAG00000003476	-0.121227	0.6285714	0.2016454
ENSBTAG00000003479	NA	NA	NA
ENSBTAG00000003481	1.4483007	0.0575442	1.2399987
ENSBTAG00000003483	NA	NA	NA
ENSBTAG00000003484	0.01979	0.9383433	0.0276382
ENSBTAG00000003485	-0.083649	0.7945391	0.0998847
ENSBTAG00000003489	-0.47417	0.370664	0.4310196
ENSBTAG00000003490	-0.020342	0.9510264	0.0218074
ENSBTAG00000003491	0.2317852	0.5897131	0.2293592
ENSBTAG00000003494	0.2877334	0.3579699	0.4461535
ENSBTAG00000003495	-0.018113	0.9423196	0.0258018
ENSBTAG00000003496	-0.223043	0.5216681	0.2826057
ENSBTAG00000003498	NA	NA	NA
ENSBTAG00000003499	0.2068051	0.4684259	0.3293591
ENSBTAG00000003500	0.6603867	0.0582656	1.2345881
ENSBTAG00000003501	0.1357964	0.6205329	0.2072352
ENSBTAG00000003502	NA	NA	NA
ENSBTAG00000003503	-0.021583	0.9454529	0.0243601
ENSBTAG00000003504	-0.067194	0.8632851	0.0638458
ENSBTAG00000003505	0.1511009	0.5546136	0.2560095
ENSBTAG00000003506	NA	NA	NA
ENSBTAG00000003508	NA	NA	NA
ENSBTAG00000003509	0.0871676	0.8234501	0.0843627
ENSBTAG00000003510	-0.201748	0.4869283	0.312535
ENSBTAG00000003511	NA	NA	NA
ENSBTAG00000003512	0.2095044	0.4356763	0.3608361
ENSBTAG00000003514	0.3555793	0.3153969	0.5011426
ENSBTAG00000003515	NA	NA	NA
ENSBTAG00000003516	-0.176167	0.4949346	0.3054522
ENSBTAG00000003519	-0.199103	0.4203909	0.3763467
ENSBTAG00000003521	-1.304764	0.01831	1.7373124
ENSBTAG00000003523	NA	NA	NA
ENSBTAG00000003525	-0.039832	0.9379038	0.0278417
ENSBTAG00000003526	-0.189058	0.5244537	0.2802929
ENSBTAG00000003527	-0.043817	0.9194898	0.0364531
ENSBTAG00000003528	NA	NA	NA
ENSBTAG00000003530	0.215289	0.4138694	0.3831367
ENSBTAG00000003531	NA	NA	NA
ENSBTAG00000003532	-0.191146	0.5433074	0.2649544
ENSBTAG00000003535	0.0491854	0.8862861	0.0524261
ENSBTAG00000003536	-0.181649	0.4634661	0.333982
ENSBTAG00000003539	NA	NA	NA
ENSBTAG00000003540	NA	NA	NA
ENSBTAG00000003541	-0.150335	0.7138532	0.1463911
ENSBTAG00000003543	0.2761139	0.290632	0.5366566
ENSBTAG00000003544	NA	NA	NA
ENSBTAG00000003545	-0.354302	0.2184473	0.6606533
ENSBTAG00000003546	0.175222	0.5482038	0.2610579
ENSBTAG00000003547	-0.039874	0.9011562	0.0451999
ENSBTAG00000003548	-0.358871	0.1917702	0.7172189
ENSBTAG00000003549	NA	NA	NA
ENSBTAG00000003550	-0.342624	0.2194674	0.6586299
ENSBTAG00000003552	0.0069378	0.9795899	0.0089557
ENSBTAG00000003553	0.4539419	0.0665276	1.1769979
ENSBTAG00000003555	-0.136387	0.6382134	0.1950341
ENSBTAG00000003556	0.2838803	0.4265937	0.3699856
ENSBTAG00000003557	-0.218229	0.3912693	0.4075242
ENSBTAG00000003559	0.2639922	0.3959558	0.4023533

ENSBTAG00000003560	NA	NA	NA
ENSBTAG00000003561	0.0183055	0.9630744	0.0163401
ENSBTAG00000003564	NA	NA	NA
ENSBTAG00000003565	-0.120399	0.7393382	0.1311569
ENSBTAG00000003566	0.5412256	0.1370898	0.8629949
ENSBTAG00000003567	0.0022107	1	0
ENSBTAG00000003568	NA	NA	NA
ENSBTAG00000003569	0.1696662	0.6649524	0.1772095
ENSBTAG00000003570	-0.20887	0.3915735	0.4071867
ENSBTAG00000003572	NA	NA	NA
ENSBTAG00000003574	NA	NA	NA
ENSBTAG00000003575	NA	NA	NA
ENSBTAG00000003576	0.1142516	0.7490488	0.1254899
ENSBTAG00000003577	-0.116828	0.6385866	0.1947802
ENSBTAG00000003578	0.0266181	0.9365485	0.0284697
ENSBTAG00000003579	-0.024544	0.9292071	0.0318875
ENSBTAG00000003580	NA	NA	NA
ENSBTAG00000003581	0.2591387	0.3153337	0.5012296
ENSBTAG00000003585	0.4466707	0.087437	1.0583048
ENSBTAG00000003586	0.4147509	0.1015423	0.9933532
ENSBTAG00000003587	-0.20203	0.5469241	0.2620729
ENSBTAG00000003588	0.2367016	0.4262879	0.370297
ENSBTAG00000003589	-0.180184	0.4969531	0.3036846
ENSBTAG00000003592	NA	NA	NA
ENSBTAG00000003593	NA	NA	NA
ENSBTAG00000003594	NA	NA	NA
ENSBTAG00000003598	0.2651576	0.4773078	0.3212014
ENSBTAG00000003600	NA	NA	NA
ENSBTAG00000003601	NA	NA	NA
ENSBTAG00000003602	0.1223105	0.717861	0.1439596
ENSBTAG00000003604	0.1593484	0.6954358	0.1577429
ENSBTAG00000003606	NA	NA	NA
ENSBTAG00000003607	0.3352378	0.4566521	0.3404145
ENSBTAG00000003608	-0.108794	0.8201472	0.0861082
ENSBTAG00000003609	0.1232722	0.6713897	0.1730253
ENSBTAG00000003610	0.2688978	0.4616849	0.3356544
ENSBTAG00000003614	-0.166976	0.6061228	0.2174394
ENSBTAG00000003616	NA	NA	NA
ENSBTAG00000003617	-0.370311	0.1635488	0.7863525
ENSBTAG00000003619	0.163649	0.6173998	0.2094335
ENSBTAG00000003622	-0.031554	0.9637749	0.0160244
ENSBTAG00000003624	NA	NA	NA
ENSBTAG00000003625	NA	NA	NA
ENSBTAG00000003626	NA	NA	NA
ENSBTAG00000003629	-1.25273	0.012437	1.9052859
ENSBTAG00000003632	NA	NA	NA
ENSBTAG00000003634	-0.284612	0.2589803	0.5867332
ENSBTAG00000003635	-0.147937	0.6647426	0.1773465
ENSBTAG00000003636	-0.207917	0.5607582	0.2512244
ENSBTAG00000003638	NA	NA	NA
ENSBTAG00000003639	0.3567158	0.2489401	0.6039051
ENSBTAG00000003642	-0.193359	0.4365842	0.3599319
ENSBTAG00000003644	0.1643747	0.6145206	0.2114635
ENSBTAG00000003645	NA	NA	NA
ENSBTAG00000003649	-0.135152	0.6657747	0.1766727
ENSBTAG00000003650	-2.342075	3.15E-05	4.5019225
ENSBTAG00000003651	NA	NA	NA
ENSBTAG00000003652	-0.022246	0.9386683	0.0274879

ENSBTAG00000003653	-0.24365	0.4819273	0.3170184
ENSBTAG00000003658	0.3800203	0.3169828	0.4989643
ENSBTAG00000003661	NA	NA	NA
ENSBTAG00000003665	0.3030434	0.4611821	0.3361275
ENSBTAG00000003667	-0.565367	0.0385565	1.4139029
ENSBTAG00000003668	NA	NA	NA
ENSBTAG00000003669	0.114819	0.7173171	0.1442888
ENSBTAG00000003671	NA	NA	NA
ENSBTAG00000003675	NA	NA	NA
ENSBTAG00000003676	NA	NA	NA
ENSBTAG00000003679	0.1273907	0.6893054	0.1615883
ENSBTAG00000003682	NA	NA	NA
ENSBTAG00000003684	NA	NA	NA
ENSBTAG00000003687	0.2408729	0.4722073	0.3258673
ENSBTAG00000003690	-0.03805	0.8811856	0.0549326
ENSBTAG00000003691	NA	NA	NA
ENSBTAG00000003692	0.4108134	0.406097	0.3913702
ENSBTAG00000003693	-0.113447	0.6661052	0.1764572
ENSBTAG00000003695	0.3296584	0.2938716	0.5318424
ENSBTAG00000003696	NA	NA	NA
ENSBTAG00000003697	-0.417445	0.1356823	0.8674768
ENSBTAG00000003699	0.485045	0.304822	0.5159537
ENSBTAG00000003700	NA	NA	NA
ENSBTAG00000003701	-0.193162	0.5663049	0.2469497
ENSBTAG00000003702	0.1075368	0.6760793	0.1700024
ENSBTAG00000003705	NA	NA	NA
ENSBTAG00000003707	-0.11201	0.8150249	0.0888291
ENSBTAG00000003708	-0.245602	0.3427458	0.4650278
ENSBTAG00000003709	-0.154903	0.7022729	0.1534941
ENSBTAG00000003710	-0.123873	0.7211601	0.1419683
ENSBTAG00000003711	0.3876407	0.1382333	0.8593873
ENSBTAG00000003712	NA	NA	NA
ENSBTAG00000003713	0.3572525	0.2128515	0.6719234
ENSBTAG00000003715	NA	NA	NA
ENSBTAG00000003718	-0.756672	0.0171906	1.7647091
ENSBTAG00000003719	-0.434257	0.1689035	0.7723613
ENSBTAG00000003721	-0.254371	0.6424354	0.1921705
ENSBTAG00000003726	0.5019761	0.1185122	0.926237
ENSBTAG00000003727	-0.27253	0.5175263	0.2860675
ENSBTAG00000003728	-0.195976	0.4565827	0.3404805
ENSBTAG00000003733	NA	NA	NA
ENSBTAG00000003740	-0.148678	0.5530717	0.2572186
ENSBTAG00000003741	0.2759237	0.4231848	0.37347
ENSBTAG00000003743	NA	NA	NA
ENSBTAG00000003745	-0.180037	0.5087237	0.293518
ENSBTAG00000003746	-0.292346	0.237095	0.6250776
ENSBTAG00000003747	-1.280494	0.0224521	1.6487435
ENSBTAG00000003748	-0.126859	0.6176198	0.2092788
ENSBTAG00000003749	0.5311922	0.2094783	0.6788609
ENSBTAG00000003751	NA	NA	NA
ENSBTAG00000003752	-0.246312	0.4482308	0.3484983
ENSBTAG00000003754	0.8341944	0.0018908	2.7233565
ENSBTAG00000003757	-0.195241	0.4306164	0.3659094
ENSBTAG00000003758	0.021897	0.9531802	0.020825
ENSBTAG00000003759	0.3670502	0.3069721	0.5129011
ENSBTAG00000003762	0.2009041	0.621596	0.2064918
ENSBTAG00000003763	-0.634415	0.2278922	0.6422705
ENSBTAG00000003764	0.0975861	0.7153363	0.1454898

ENSBTAG00000003766	-0.000619	1	0
ENSBTAG00000003770	-1.006181	0.0023756	2.6242225
ENSBTAG00000003771	NA	NA	NA
ENSBTAG00000003773	0.1194773	0.6884555	0.1621242
ENSBTAG00000003774	NA	NA	NA
ENSBTAG00000003775	NA	NA	NA
ENSBTAG00000003776	NA	NA	NA
ENSBTAG00000003777	0.5642327	0.067108	1.173226
ENSBTAG00000003779	0.5017236	0.29046	0.5369136
ENSBTAG00000003781	NA	NA	NA
ENSBTAG00000003784	-0.476695	0.0780133	1.1078313
ENSBTAG00000003786	NA	NA	NA
ENSBTAG00000003788	-0.293163	0.3866226	0.4127128
ENSBTAG00000003789	0.1243306	0.6222392	0.2060426
ENSBTAG00000003791	NA	NA	NA
ENSBTAG00000003793	-0.169772	0.5083757	0.2938152
ENSBTAG00000003794	NA	NA	NA
ENSBTAG00000003797	NA	NA	NA
ENSBTAG00000003798	-0.461206	0.1094042	0.960966
ENSBTAG00000003799	0.0720438	0.776627	0.1097875
ENSBTAG00000003800	-0.029625	0.9587269	0.0183051
ENSBTAG00000003801	0.0382715	0.8999534	0.04578
ENSBTAG00000003802	NA	NA	NA
ENSBTAG00000003804	-0.025422	0.9400131	0.0268661
ENSBTAG00000003806	0.6895189	0.1116853	0.952004
ENSBTAG00000003807	0.1725427	0.5261083	0.2789248
ENSBTAG00000003808	-1.687386	8.45E-09	8.0731685
ENSBTAG00000003809	0.1244075	0.6530126	0.1850784
ENSBTAG00000003810	0.16937	0.663609	0.1780878
ENSBTAG00000003813	-0.187853	0.4938779	0.3063804
ENSBTAG00000003815	0.0985502	0.7703384	0.1133184
ENSBTAG00000003817	0.2608569	0.3919748	0.4067418
ENSBTAG00000003819	-0.151576	0.7494393	0.1252635
ENSBTAG00000003820	0.6697007	0.0297236	1.5268984
ENSBTAG00000003822	0.2019731	0.5536555	0.2567604
ENSBTAG00000003825	-0.327652	0.3197205	0.4952295
ENSBTAG00000003826	-0.304791	0.2285296	0.6410576
ENSBTAG00000003827	NA	NA	NA
ENSBTAG00000003829	0.2332818	0.6231688	0.2053943
ENSBTAG00000003830	0.0034339	0.9893272	0.0046601
ENSBTAG00000003832	0.4305738	0.3097655	0.508967
ENSBTAG00000003833	NA	NA	NA
ENSBTAG00000003835	0.254166	0.4998727	0.3011406
ENSBTAG00000003836	0.040507	0.8964877	0.0474557
ENSBTAG00000003837	NA	NA	NA
ENSBTAG00000003840	0.3706904	0.1663715	0.778921
ENSBTAG00000003842	0.0105291	0.9669779	0.0145835
ENSBTAG00000003843	0.1702022	0.5856968	0.2323272
ENSBTAG00000003845	-0.103928	0.7630023	0.1174742
ENSBTAG00000003846	0.0891167	0.7365786	0.1327809
ENSBTAG00000003848	0.0433198	0.8657418	0.0626116
ENSBTAG00000003849	-0.092407	0.7560544	0.1214469
ENSBTAG00000003851	-0.42443	0.344479	0.4628372
ENSBTAG00000003855	-0.255862	0.4010898	0.3967584
ENSBTAG00000003857	0.6686835	0.0177318	1.751247
ENSBTAG00000003861	0.0823508	0.8307612	0.0805238
ENSBTAG00000003863	0.1814694	0.6752398	0.170542
ENSBTAG00000003864	NA	NA	NA

ENSBTAG00000003865	NA	NA	NA
ENSBTAG00000003866	0.0085115	0.9757403	0.0106657
ENSBTAG00000003871	NA	NA	NA
ENSBTAG00000003872	0.1708235	0.5558737	0.2550239
ENSBTAG00000003876	-0.180893	0.4817494	0.3171789
ENSBTAG00000003877	-0.012748	0.9595688	0.0179239
ENSBTAG00000003878	0.0766482	0.7944299	0.0999444
ENSBTAG00000003880	0.0315895	0.9294522	0.0317729
ENSBTAG00000003882	-0.170394	0.5930275	0.2269251
ENSBTAG00000003884	-0.299671	0.4337085	0.3628021
ENSBTAG00000003885	0.0657022	0.8579484	0.0665388
ENSBTAG00000003887	-0.282776	0.2991303	0.5241396
ENSBTAG00000003889	-0.626751	0.0469931	1.3279662
ENSBTAG00000003891	NA	NA	NA
ENSBTAG00000003892	0.1760373	0.5706569	0.2436249
ENSBTAG00000003893	NA	NA	NA
ENSBTAG00000003894	0.2956863	0.4880817	0.3115075
ENSBTAG00000003895	-0.097431	0.8335615	0.0790623
ENSBTAG00000003897	-0.406411	0.1477152	0.8305749
ENSBTAG00000003898	NA	NA	NA
ENSBTAG00000003901	NA	NA	NA
ENSBTAG00000003902	0.2620528	0.4014227	0.396398
ENSBTAG00000003904	-0.052312	0.8555143	0.0677727
ENSBTAG00000003906	0.2405801	0.4613305	0.3359878
ENSBTAG00000003907	0.257126	0.444065	0.3525535
ENSBTAG00000003908	-0.450015	0.076521	1.1162195
ENSBTAG00000003914	-0.047888	0.8661675	0.0623981
ENSBTAG00000003915	0.4498964	0.2163671	0.6648088
ENSBTAG00000003916	0.2393866	0.3541068	0.4508657
ENSBTAG00000003919	-0.390496	0.2564266	0.5910369
ENSBTAG00000003920	NA	NA	NA
ENSBTAG00000003921	NA	NA	NA
ENSBTAG00000003922	-0.004176	0.9904075	0.0041861
ENSBTAG00000003923	NA	NA	NA
ENSBTAG00000003925	-0.178916	0.4790073	0.3196579
ENSBTAG00000003927	-0.089351	0.7172435	0.1443334
ENSBTAG00000003928	0.1066604	0.718831	0.1433732
ENSBTAG00000003929	-0.035322	0.8883854	0.0513986
ENSBTAG00000003934	-0.513333	0.2081368	0.6816512
ENSBTAG00000003935	0.1188267	0.6683522	0.1749946
ENSBTAG00000003936	-0.101964	0.6789946	0.1681337
ENSBTAG00000003937	NA	NA	NA
ENSBTAG00000003938	0.2858235	0.5666937	0.2466517
ENSBTAG00000003941	-0.46757	0.1110024	0.9546677
ENSBTAG00000003942	0.4897924	0.1894696	0.7224604
ENSBTAG00000003943	0.4297275	0.2183142	0.6609181
ENSBTAG00000003944	NA	NA	NA
ENSBTAG00000003946	0.0788595	0.8833199	0.053882
ENSBTAG00000003947	0.1571563	0.6343561	0.1976669
ENSBTAG00000003948	0.1144501	0.6661994	0.1763958
ENSBTAG00000003949	-0.081483	0.7514921	0.1240756
ENSBTAG00000003950	NA	NA	NA
ENSBTAG00000003952	-0.090676	0.8472875	0.0719692
ENSBTAG00000003953	0.1118697	0.7175355	0.1441566
ENSBTAG00000003954	-0.168176	0.5092993	0.2930269
ENSBTAG00000003955	NA	NA	NA
ENSBTAG00000003956	-0.26851	0.3460512	0.4608597
ENSBTAG00000003958	-0.180743	0.7225318	0.141143

ENSBTAG00000003959	0.2118926	0.5810353	0.2357975
ENSBTAG00000003960	-0.465415	0.1660492	0.7797632
ENSBTAG00000003961	NA	NA	NA
ENSBTAG00000003962	NA	NA	NA
ENSBTAG00000003963	NA	NA	NA
ENSBTAG00000003965	0.3343631	0.4386936	0.3578387
ENSBTAG00000003966	-0.200195	0.4303609	0.3661672
ENSBTAG00000003967	0.0838871	0.844238	0.0735351
ENSBTAG00000003968	0.6903759	0.0169704	1.7703089
ENSBTAG00000003970	0.2668772	0.5337177	0.2726884
ENSBTAG00000003971	NA	NA	NA
ENSBTAG00000003975	0.0358883	0.9109374	0.0405115
ENSBTAG00000003981	0.2041061	0.535551	0.2711992
ENSBTAG00000003983	0.3343715	0.1938739	0.7124806
ENSBTAG00000003985	0.0780945	0.8078495	0.0926695
ENSBTAG00000003986	-0.451521	0.1943468	0.7114227
ENSBTAG00000003989	-0.297351	0.3190182	0.4961846
ENSBTAG00000003990	-0.127791	0.8294178	0.0812267
ENSBTAG00000003994	-0.371244	0.2687967	0.5705761
ENSBTAG00000003997	0.0704685	0.7962631	0.0989434
ENSBTAG00000004000	-0.006864	0.9795223	0.0089857
ENSBTAG00000004003	NA	NA	NA
ENSBTAG00000004004	NA	NA	NA
ENSBTAG00000004005	0.0666368	0.7852375	0.104999
ENSBTAG00000004007	NA	NA	NA
ENSBTAG00000004008	-0.351983	0.173526	0.7606354
ENSBTAG00000004009	0.4045306	0.3619525	0.4413485
ENSBTAG00000004010	0.0561065	0.9126407	0.0397002
ENSBTAG00000004011	-0.244744	0.4731793	0.3249742
ENSBTAG00000004013	0.2096609	0.6712315	0.1731277
ENSBTAG00000004014	0.181005	0.5310342	0.2748775
ENSBTAG00000004015	0.1291702	0.6347261	0.1974137
ENSBTAG00000004017	0.3217935	0.4918673	0.308152
ENSBTAG00000004018	0.4507808	0.2977398	0.5261631
ENSBTAG00000004019	NA	NA	NA
ENSBTAG00000004021	0.134056	0.7403872	0.1305411
ENSBTAG00000004022	NA	NA	NA
ENSBTAG00000004023	NA	NA	NA
ENSBTAG00000004024	-0.188913	0.4598648	0.3373698
ENSBTAG00000004028	-0.186126	0.550036	0.2596089
ENSBTAG00000004029	-0.130676	0.6108458	0.2140684
ENSBTAG00000004034	-0.333121	0.2234066	0.6509039
ENSBTAG00000004035	0.3511856	0.370573	0.4311263
ENSBTAG00000004036	-0.151542	0.6777069	0.1689581
ENSBTAG00000004037	0.4728943	0.7009391	0.1543197
ENSBTAG00000004038	-0.360308	0.1890234	0.7234843
ENSBTAG00000004039	0.1133252	0.7558683	0.1215539
ENSBTAG00000004041	-0.63644	0.0780817	1.1074509
ENSBTAG00000004043	NA	NA	NA
ENSBTAG00000004046	0.3092147	0.5331101	0.2731831
ENSBTAG00000004048	-1.088166	0.0167193	1.7767809
ENSBTAG00000004051	-6.37E-05	0.9999348	2.83E-05
ENSBTAG00000004052	NA	NA	NA
ENSBTAG00000004054	-0.339423	0.1961836	0.7073373
ENSBTAG00000004059	0.1171827	0.6893951	0.1615318
ENSBTAG00000004061	NA	NA	NA
ENSBTAG00000004063	0.6693199	0.102607	0.9888231
ENSBTAG00000004064	0.4214539	0.1636478	0.7860898

ENSBTAG00000004066	-0.082554	0.8308306	0.0804875
ENSBTAG00000004072	-0.550801	0.0382216	1.4176911
ENSBTAG00000004073	0.00327	0.9961687	0.0016671
ENSBTAG00000004075	-0.292801	0.3406351	0.4677106
ENSBTAG00000004077	-0.04257	0.8660692	0.0624474
ENSBTAG00000004078	NA	NA	NA
ENSBTAG00000004079	-0.047418	0.8747793	0.0581015
ENSBTAG00000004080	-0.223953	0.4132284	0.3838098
ENSBTAG00000004081	0.480407	0.4599868	0.3372546
ENSBTAG00000004085	NA	NA	NA
ENSBTAG00000004086	NA	NA	NA
ENSBTAG00000004088	NA	NA	NA
ENSBTAG00000004091	0.2602925	0.4179207	0.3789062
ENSBTAG00000004092	NA	NA	NA
ENSBTAG00000004093	-0.544957	0.2145586	0.6684541
ENSBTAG00000004094	0.261492	0.3148603	0.501882
ENSBTAG00000004095	NA	NA	NA
ENSBTAG00000004097	NA	NA	NA
ENSBTAG00000004098	0.3842405	0.3269413	0.4855302
ENSBTAG00000004099	NA	NA	NA
ENSBTAG00000004100	0.2722868	0.3667153	0.435671
ENSBTAG00000004104	NA	NA	NA
ENSBTAG00000004106	0.0017767	0.9972218	0.0012082
ENSBTAG00000004108	-0.048361	0.8971864	0.0471173
ENSBTAG00000004110	NA	NA	NA
ENSBTAG00000004111	NA	NA	NA
ENSBTAG00000004112	-0.518878	0.0714541	1.1459727
ENSBTAG00000004114	0.0126199	0.9719864	0.0123398
ENSBTAG00000004115	0.4207277	0.1304763	0.8844682
ENSBTAG00000004117	0.0018426	0.9958664	0.0017989
ENSBTAG00000004118	-0.061468	0.8107053	0.091137
ENSBTAG00000004120	0.1756306	0.6349901	0.197233
ENSBTAG00000004124	0.1212101	0.6464093	0.1894924
ENSBTAG00000004126	NA	NA	NA
ENSBTAG00000004129	NA	NA	NA
ENSBTAG00000004130	NA	NA	NA
ENSBTAG00000004131	-0.728779	0.0385566	1.4139013
ENSBTAG00000004135	0.1372046	0.6467316	0.1892759
ENSBTAG00000004136	NA	NA	NA
ENSBTAG00000004138	0.9425288	0.0642197	1.1923315
ENSBTAG00000004139	-0.295886	0.4172227	0.3796321
ENSBTAG00000004140	NA	NA	NA
ENSBTAG00000004145	NA	NA	NA
ENSBTAG00000004147	-0.004037	0.9885599	0.004997
ENSBTAG00000004148	NA	NA	NA
ENSBTAG00000004150	NA	NA	NA
ENSBTAG00000004151	NA	NA	NA
ENSBTAG00000004153	NA	NA	NA
ENSBTAG00000004154	-0.853005	0.0259674	1.5855719
ENSBTAG00000004155	0.2078825	0.5455748	0.2631457
ENSBTAG00000004159	-0.559252	0.1526205	0.8163872
ENSBTAG00000004161	0.0339696	0.8904923	0.0503698
ENSBTAG00000004165	0.1859647	0.5221293	0.282222
ENSBTAG00000004168	0.272475	0.4032962	0.3943758
ENSBTAG00000004171	0.2595502	0.3481936	0.4581792
ENSBTAG00000004172	0.2083764	0.5945522	0.22581
ENSBTAG00000004173	0.0270583	0.9377911	0.0278939
ENSBTAG00000004175	-0.568436	0.2653044	0.5762556

ENSBTAG00000004177	-0.320314	0.2346136	0.6296468
ENSBTAG00000004178	0.4726739	0.1186586	0.9257009
ENSBTAG00000004179	0.2796687	0.3978249	0.4003081
ENSBTAG00000004184	0.1635485	0.7321722	0.1353868
ENSBTAG00000004187	NA	NA	NA
ENSBTAG00000004188	0.0729917	0.8134508	0.0896687
ENSBTAG00000004189	-0.132881	0.6231069	0.2054375
ENSBTAG00000004190	0.3609987	0.1993602	0.7003615
ENSBTAG00000004191	-0.196872	0.6457878	0.1899102
ENSBTAG00000004192	NA	NA	NA
ENSBTAG00000004193	0.1818004	0.4829046	0.3161387
ENSBTAG00000004194	0.2771561	0.4882498	0.3113579
ENSBTAG00000004196	-0.067333	0.8814654	0.0547947
ENSBTAG00000004197	NA	NA	NA
ENSBTAG00000004199	0.0270686	0.9200221	0.0362017
ENSBTAG00000004200	0.1505618	0.6514561	0.1861149
ENSBTAG00000004203	-0.011893	0.9717545	0.0124435
ENSBTAG00000004204	0.3292209	0.2229002	0.6518896
ENSBTAG00000004206	NA	NA	NA
ENSBTAG00000004207	0.363941	0.1748897	0.7572359
ENSBTAG00000004208	NA	NA	NA
ENSBTAG00000004211	0.2616211	0.4026119	0.3951134
ENSBTAG00000004212	NA	NA	NA
ENSBTAG00000004215	0.1006149	0.7118335	0.1476216
ENSBTAG00000004216	-0.822679	0.0060782	2.2162284
ENSBTAG00000004221	NA	NA	NA
ENSBTAG00000004222	-0.409123	0.1926294	0.7152773
ENSBTAG00000004223	NA	NA	NA
ENSBTAG00000004224	NA	NA	NA
ENSBTAG00000004225	-0.213212	0.3888825	0.4101816
ENSBTAG00000004226	NA	NA	NA
ENSBTAG00000004227	0.2417323	0.4653329	0.3322362
ENSBTAG00000004230	-0.206979	0.4042579	0.3933415
ENSBTAG00000004231	NA	NA	NA
ENSBTAG00000004232	NA	NA	NA
ENSBTAG00000004237	-0.330568	0.2580111	0.5883616
ENSBTAG00000004238	0.3401734	0.190816	0.7193853
ENSBTAG00000004240	-0.366052	0.1609689	0.7932582
ENSBTAG00000004242	0.6084313	0.0207131	1.6837554
ENSBTAG00000004243	0.0176641	0.946936	0.0236794
ENSBTAG00000004246	0.1141643	0.7218599	0.1415471
ENSBTAG00000004247	NA	NA	NA
ENSBTAG00000004248	1.2680116	3.01E-06	5.5208395
ENSBTAG00000004249	-0.800413	0.0179261	1.7465145
ENSBTAG00000004256	-0.063956	0.829771	0.0810417
ENSBTAG00000004257	0.0506396	0.908476	0.0416866
ENSBTAG00000004258	0.19187	0.4448799	0.3517572
ENSBTAG00000004259	0.1513541	0.7443289	0.1282351
ENSBTAG00000004261	-0.620613	0.0641315	1.1929284
ENSBTAG00000004262	NA	NA	NA
ENSBTAG00000004263	NA	NA	NA
ENSBTAG00000004266	-0.846648	0.0839641	1.0759063
ENSBTAG00000004267	NA	NA	NA
ENSBTAG00000004268	-0.21571	0.4086707	0.3886264
ENSBTAG00000004269	-0.216619	0.5936676	0.2264566
ENSBTAG00000004270	NA	NA	NA
ENSBTAG00000004271	-0.05319	0.8496126	0.0707791
ENSBTAG00000004272	NA	NA	NA

ENSBTAG00000004273	NA	NA	NA
ENSBTAG00000004275	0.6015947	0.130747	0.8835683
ENSBTAG00000004277	-0.004511	0.9924514	0.0032907
ENSBTAG00000004278	0.1337993	0.6559119	0.1831545
ENSBTAG00000004279	-0.02067	0.9391903	0.0272464
ENSBTAG00000004280	0.9943475	0.0326069	1.4866906
ENSBTAG00000004281	-0.132708	0.6828326	0.1656857
ENSBTAG00000004282	0.1484126	0.5961295	0.2246594
ENSBTAG00000004283	-0.599721	0.025138	1.5996701
ENSBTAG00000004284	-0.149265	0.5637787	0.2488913
ENSBTAG00000004286	0.0187886	0.9678688	0.0141835
ENSBTAG00000004287	0.0894225	0.8238081	0.0841739
ENSBTAG00000004288	0.0721869	0.8042145	0.0946281
ENSBTAG00000004290	0.5113556	0.1102413	0.9576557
ENSBTAG00000004291	0.0391685	0.9151753	0.0384957
ENSBTAG00000004292	NA	NA	NA
ENSBTAG00000004294	-0.006488	0.9808988	0.0083758
ENSBTAG00000004295	0.1779367	0.4924896	0.307603
ENSBTAG00000004297	NA	NA	NA
ENSBTAG00000004300	NA	NA	NA
ENSBTAG00000004302	-0.243899	0.4496007	0.347173
ENSBTAG00000004303	NA	NA	NA
ENSBTAG00000004305	-2.828762	2.91E-05	4.536113
ENSBTAG00000004307	-0.110469	0.6710785	0.1732267
ENSBTAG00000004310	-0.045302	0.8683266	0.0613169
ENSBTAG00000004315	0.244098	0.3413611	0.466786
ENSBTAG00000004316	0.1142336	0.6696172	0.1741734
ENSBTAG00000004318	0.2771579	0.4841138	0.3150525
ENSBTAG00000004321	NA	NA	NA
ENSBTAG00000004322	-0.738217	0.0938132	1.027736
ENSBTAG00000004324	NA	NA	NA
ENSBTAG00000004327	-0.82389	0.0337824	1.471309
ENSBTAG00000004328	-0.182548	0.5868759	0.2314537
ENSBTAG00000004329	0.3834925	0.275968	0.5591412
ENSBTAG00000004330	NA	NA	NA
ENSBTAG00000004331	0.3976768	0.2791609	0.5541454
ENSBTAG00000004333	-0.12404	0.617386	0.2094432
ENSBTAG00000004334	0.349993	0.2053825	0.6874366
ENSBTAG00000004337	-0.049655	0.9180545	0.0371315
ENSBTAG00000004339	0.9938082	0.0078472	2.1052834
ENSBTAG00000004342	NA	NA	NA
ENSBTAG00000004343	0.0385616	0.8845595	0.0532729
ENSBTAG00000004344	0.8877951	0.0008421	3.0746152
ENSBTAG00000004347	0.690125	0.011773	1.9291118
ENSBTAG00000004348	0.0169051	0.9466018	0.0238327
ENSBTAG00000004349	0.1017554	0.7017895	0.1537931
ENSBTAG00000004353	0.3329427	0.3837278	0.4159767
ENSBTAG00000004354	NA	NA	NA
ENSBTAG00000004355	NA	NA	NA
ENSBTAG00000004356	0.353388	0.3188076	0.4964713
ENSBTAG00000004358	-0.068236	0.8469414	0.0721466
ENSBTAG00000004361	-0.184962	0.6838369	0.1650475
ENSBTAG00000004362	-0.194602	0.7438666	0.1285049
ENSBTAG00000004364	-0.413018	0.3780948	0.4223993
ENSBTAG00000004367	0.0877385	0.7268469	0.1385571
ENSBTAG00000004368	-0.208599	0.6374506	0.1955534
ENSBTAG00000004371	0.1099392	0.6946852	0.1582119
ENSBTAG00000004374	0.0472566	0.8819585	0.0545519

ENSBTAG00000004375	NA	NA	NA
ENSBTAG00000004376	0.3399415	0.2784549	0.5552451
ENSBTAG00000004377	0.9512723	0.0592822	1.2270758
ENSBTAG00000004378	NA	NA	NA
ENSBTAG00000004379	0.4254054	0.1879277	0.7260093
ENSBTAG00000004380	-0.172034	0.5536538	0.2567617
ENSBTAG00000004381	NA	NA	NA
ENSBTAG00000004383	0.1999359	0.4667522	0.3309136
ENSBTAG00000004384	NA	NA	NA
ENSBTAG00000004386	NA	NA	NA
ENSBTAG00000004387	0.4630199	0.1147115	0.9403932
ENSBTAG00000004388	NA	NA	NA
ENSBTAG00000004392	0.4072322	0.3674343	0.4348203
ENSBTAG00000004394	NA	NA	NA
ENSBTAG00000004398	-0.717326	0.0418955	1.3778324
ENSBTAG00000004399	NA	NA	NA
ENSBTAG00000004400	0.0233397	0.927584	0.0326468
ENSBTAG00000004401	0.1961834	0.6450892	0.1903802
ENSBTAG00000004402	-0.599198	0.0479631	1.3190931
ENSBTAG00000004403	-0.082806	0.8136826	0.0895449
ENSBTAG00000004405	-0.956412	0.0293587	1.532263
ENSBTAG00000004406	-0.02379	0.9273624	0.0327505
ENSBTAG00000004407	NA	NA	NA
ENSBTAG00000004409	-0.152213	0.5442437	0.2642066
ENSBTAG00000004411	0.3398816	0.5150729	0.2881313
ENSBTAG00000004413	-0.570523	0.0610904	1.2140268
ENSBTAG00000004416	0.0456209	0.8661509	0.0624065
ENSBTAG00000004420	0.1470671	0.6991435	0.1554337
ENSBTAG00000004421	0.4374512	0.328076	0.4840256
ENSBTAG00000004422	-0.205109	0.6314202	0.1996815
ENSBTAG00000004423	-0.122007	0.7324882	0.1351994
ENSBTAG00000004425	0.1563512	0.6908969	0.1605868
ENSBTAG00000004426	0.031869	0.9150293	0.038565
ENSBTAG00000004427	-0.042595	0.8648438	0.0630623
ENSBTAG00000004428	0.0378015	0.9007735	0.0453844
ENSBTAG00000004429	NA	NA	NA
ENSBTAG00000004430	0.341382	0.3369585	0.4724235
ENSBTAG00000004432	0.3529743	0.2739571	0.5623175
ENSBTAG00000004436	-0.573928	0.0800307	1.0967436
ENSBTAG00000004438	0.1045369	0.7138294	0.1464055
ENSBTAG00000004440	NA	NA	NA
ENSBTAG00000004442	0.0574314	0.9030159	0.0443046
ENSBTAG00000004443	NA	NA	NA
ENSBTAG00000004448	0.0645104	0.7955224	0.0993476
ENSBTAG00000004449	-0.05707	0.8594743	0.0657671
ENSBTAG00000004452	0.1829925	0.4937098	0.3065282
ENSBTAG00000004456	NA	NA	NA
ENSBTAG00000004457	-0.660316	0.0203511	1.6914119
ENSBTAG00000004458	NA	NA	NA
ENSBTAG00000004459	0.5200727	0.269177	0.5699621
ENSBTAG00000004460	0.1333185	0.7124866	0.1472233
ENSBTAG00000004461	0.1346035	0.7001174	0.1548291
ENSBTAG00000004462	-0.482906	0.2996837	0.5233369
ENSBTAG00000004463	0.124231	0.7538058	0.1227405
ENSBTAG00000004464	NA	NA	NA
ENSBTAG00000004468	0.2583239	0.6310723	0.1999209
ENSBTAG00000004471	0.0797979	0.7782791	0.1088646
ENSBTAG00000004472	-0.307542	0.3288303	0.4830282

ENSBTAG00000004475	NA	NA	NA
ENSBTAG00000004476	-0.643903	0.0820079	1.0861445
ENSBTAG00000004484	-0.00733	0.9810965	0.0082883
ENSBTAG00000004488	NA	NA	NA
ENSBTAG00000004489	-0.198361	0.4436486	0.3529609
ENSBTAG00000004490	NA	NA	NA
ENSBTAG00000004492	-0.242243	0.4130825	0.3839632
ENSBTAG00000004494	NA	NA	NA
ENSBTAG00000004495	0.0422383	0.8704867	0.0602379
ENSBTAG00000004496	0.1026606	0.7145579	0.1459626
ENSBTAG00000004497	0.4357275	0.1280859	0.8924988
ENSBTAG00000004498	NA	NA	NA
ENSBTAG00000004499	-0.664689	0.0139773	1.8545762
ENSBTAG00000004502	0.3255356	0.3380063	0.4710752
ENSBTAG00000004505	0.5492834	0.2390513	0.6215089
ENSBTAG00000004507	NA	NA	NA
ENSBTAG00000004509	0.4079026	0.2823447	0.5492204
ENSBTAG00000004510	NA	NA	NA
ENSBTAG00000004511	-0.118126	0.7637942	0.1170237
ENSBTAG00000004512	-0.064221	0.8229967	0.0846019
ENSBTAG00000004514	-0.002106	0.9940443	0.0025942
ENSBTAG00000004515	NA	NA	NA
ENSBTAG00000004517	0.0488536	0.8612723	0.0648595
ENSBTAG00000004519	NA	NA	NA
ENSBTAG00000004521	0.0886676	0.779344	0.1082708
ENSBTAG00000004524	0.7136242	0.0514388	1.2887089
ENSBTAG00000004526	1.019502	0.028375	1.5470648
ENSBTAG00000004531	-0.236911	0.3520063	0.4534496
ENSBTAG00000004532	-0.211423	0.4513206	0.3455148
ENSBTAG00000004538	0.0220254	0.9672761	0.0144495
ENSBTAG00000004540	-0.065375	0.7938534	0.1002597
ENSBTAG00000004541	NA	NA	NA
ENSBTAG00000004542	-0.521698	0.0372923	1.4283806
ENSBTAG00000004544	-0.036119	0.8960084	0.0476879
ENSBTAG00000004547	NA	NA	NA
ENSBTAG00000004549	-0.357239	0.2013376	0.6960751
ENSBTAG00000004551	0.1777922	0.6090645	0.2153367
ENSBTAG00000004552	0.0633498	0.8150381	0.0888221
ENSBTAG00000004553	-0.379934	0.1461657	0.8351544
ENSBTAG00000004554	-0.211349	0.4181938	0.3786224
ENSBTAG00000004555	NA	NA	NA
ENSBTAG00000004556	0.6658059	0.031986	1.4950406
ENSBTAG00000004557	NA	NA	NA
ENSBTAG00000004558	-0.160969	0.7281728	0.1377656
ENSBTAG00000004561	NA	NA	NA
ENSBTAG00000004562	NA	NA	NA
ENSBTAG00000004564	-0.216642	0.4174901	0.3793538
ENSBTAG00000004567	-0.246822	0.4239984	0.3726357
ENSBTAG00000004568	-0.221991	0.6367643	0.1960213
ENSBTAG00000004570	0.4203479	0.2203584	0.6568703
ENSBTAG00000004571	0.2354218	0.4065425	0.3908941
ENSBTAG00000004572	NA	NA	NA
ENSBTAG00000004574	-0.265591	0.6414299	0.1928508
ENSBTAG00000004575	-0.195324	0.4692063	0.3286361
ENSBTAG00000004580	NA	NA	NA
ENSBTAG00000004581	NA	NA	NA
ENSBTAG00000004582	NA	NA	NA
ENSBTAG00000004583	NA	NA	NA

ENSBTAG00000004585	-0.321239	0.3905146	0.4083627
ENSBTAG00000004586	-0.064638	0.8091464	0.0919729
ENSBTAG00000004587	0.4357572	0.2330843	0.632487
ENSBTAG00000004588	NA	NA	NA
ENSBTAG00000004590	0.0800248	0.7829358	0.1062739
ENSBTAG00000004591	NA	NA	NA
ENSBTAG00000004592	-0.031098	0.9184989	0.0369214
ENSBTAG00000004593	-0.116775	0.6440776	0.1910618
ENSBTAG00000004597	-0.262396	0.5768859	0.2389101
ENSBTAG00000004598	-0.01512	0.9540592	0.0204247
ENSBTAG00000004599	0.3378401	0.4454576	0.3511936
ENSBTAG00000004600	0.4933963	0.0983201	1.0073576
ENSBTAG00000004601	-0.063019	0.8426049	0.074376
ENSBTAG00000004602	NA	NA	NA
ENSBTAG00000004603	0.3009112	0.2563353	0.5911915
ENSBTAG00000004604	-0.209681	0.4389669	0.3575683
ENSBTAG00000004607	NA	NA	NA
ENSBTAG00000004612	NA	NA	NA
ENSBTAG00000004613	-0.164702	0.6828475	0.1656763
ENSBTAG00000004617	NA	NA	NA
ENSBTAG00000004620	0.7323193	0.0396115	1.4021783
ENSBTAG00000004622	0.686571	0.1265162	0.8978537
ENSBTAG00000004624	NA	NA	NA
ENSBTAG00000004625	0.3932292	0.2902497	0.5372282
ENSBTAG00000004629	NA	NA	NA
ENSBTAG00000004630	NA	NA	NA
ENSBTAG00000004631	0.1616529	0.539633	0.2679015
ENSBTAG00000004632	0.1885431	0.4957143	0.3047685
ENSBTAG00000004633	NA	NA	NA
ENSBTAG00000004635	-0.350315	0.2808307	0.5515554
ENSBTAG00000004636	-0.287785	0.3027356	0.5189364
ENSBTAG00000004639	-0.18007	0.7104443	0.1484699
ENSBTAG00000004641	NA	NA	NA
ENSBTAG00000004643	-0.154057	0.5546542	0.2559777
ENSBTAG00000004645	0.319747	0.3037387	0.5174998
ENSBTAG00000004647	NA	NA	NA
ENSBTAG00000004650	-0.361827	0.2499089	0.6022182
ENSBTAG00000004651	0.169019	0.7181538	0.1437825
ENSBTAG00000004652	NA	NA	NA
ENSBTAG00000004653	-0.025443	0.9225006	0.0350333
ENSBTAG00000004654	-0.08452	0.7499076	0.1249922
ENSBTAG00000004657	NA	NA	NA
ENSBTAG00000004659	-1.930048	4.52E-06	5.3450096
ENSBTAG00000004660	-0.007851	0.9862838	0.0059981
ENSBTAG00000004662	0.2677562	0.5587449	0.2527864
ENSBTAG00000004663	-0.099649	0.7089054	0.1494117
ENSBTAG00000004664	-0.112745	0.7121578	0.1474238
ENSBTAG00000004672	-0.266701	0.2882492	0.5402319
ENSBTAG00000004674	NA	NA	NA
ENSBTAG00000004675	-0.081757	0.8536951	0.0686972
ENSBTAG00000004676	-0.434231	0.4134502	0.3835767
ENSBTAG00000004679	-0.001051	0.9973887	0.0011355
ENSBTAG00000004680	NA	NA	NA
ENSBTAG00000004685	NA	NA	NA
ENSBTAG00000004688	-0.247836	0.5724214	0.2422841
ENSBTAG00000004690	NA	NA	NA
ENSBTAG00000004693	NA	NA	NA
ENSBTAG00000004694	NA	NA	NA

ENSBTAG00000004695	0.0872076	0.7458885	0.1273261
ENSBTAG00000004705	NA	NA	NA
ENSBTAG00000004706	-0.215541	0.6385916	0.1947768
ENSBTAG00000004709	-0.371967	0.1782371	0.749002
ENSBTAG00000004712	NA	NA	NA
ENSBTAG00000004714	NA	NA	NA
ENSBTAG00000004715	-0.172735	0.4836415	0.3154764
ENSBTAG00000004716	NA	NA	NA
ENSBTAG00000004718	-0.285297	0.4487134	0.3480309
ENSBTAG00000004720	-0.095663	0.7793441	0.1082707
ENSBTAG00000004722	0.0412586	0.9124841	0.0397747
ENSBTAG00000004723	-0.354246	0.1803283	0.7439362
ENSBTAG00000004727	0.1368085	0.8132016	0.0898018
ENSBTAG00000004729	NA	NA	NA
ENSBTAG00000004732	-0.439522	0.0966101	1.0149773
ENSBTAG00000004735	-0.284991	0.4420787	0.3545004
ENSBTAG00000004736	-0.07032	0.8030849	0.0952386
ENSBTAG00000004738	0.2280835	0.3791423	0.4211978
ENSBTAG00000004739	NA	NA	NA
ENSBTAG00000004741	NA	NA	NA
ENSBTAG00000004742	NA	NA	NA
ENSBTAG00000004745	-0.317865	0.247271	0.6068269
ENSBTAG00000004746	-0.131553	0.6420546	0.192428
ENSBTAG00000004747	0.1778411	0.5397147	0.2678358
ENSBTAG00000004749	-0.28259	0.3251847	0.4878698
ENSBTAG00000004750	-0.443357	0.3914199	0.4073571
ENSBTAG00000004751	NA	NA	NA
ENSBTAG00000004753	-0.072444	0.8454194	0.0729278
ENSBTAG00000004754	NA	NA	NA
ENSBTAG00000004755	0.8551746	0.0415571	1.3813551
ENSBTAG00000004757	0.2581714	0.3369184	0.4724752
ENSBTAG00000004761	NA	NA	NA
ENSBTAG00000004766	NA	NA	NA
ENSBTAG00000004767	-0.074634	0.8813046	0.0548739
ENSBTAG00000004768	0.8509008	0.0440163	1.3563861
ENSBTAG00000004769	-0.144557	0.8066928	0.0932918
ENSBTAG00000004770	0.1245282	0.662515	0.1788043
ENSBTAG00000004772	-0.443198	0.3629823	0.4401145
ENSBTAG00000004775	NA	NA	NA
ENSBTAG00000004776	-0.119026	0.7172778	0.1443126
ENSBTAG00000004777	-0.374464	0.1475377	0.8310969
ENSBTAG00000004780	NA	NA	NA
ENSBTAG00000004781	-0.124001	0.6612408	0.1796404
ENSBTAG00000004782	0.1457087	0.7833279	0.1060564
ENSBTAG00000004783	0.4093567	0.2260767	0.6457442
ENSBTAG00000004786	-0.067618	0.822675	0.0847717
ENSBTAG00000004787	0.0774172	0.8286635	0.0816218
ENSBTAG00000004788	0.3789453	0.302496	0.5192803
ENSBTAG00000004789	NA	NA	NA
ENSBTAG00000004790	-0.394613	0.2036398	0.6911373
ENSBTAG00000004791	0.0699502	0.840723	0.0753471
ENSBTAG00000004792	-0.385585	0.4608574	0.3364334
ENSBTAG00000004793	NA	NA	NA
ENSBTAG00000004795	NA	NA	NA
ENSBTAG00000004796	NA	NA	NA
ENSBTAG00000004797	0.1104261	0.7759837	0.1101474
ENSBTAG00000004799	0.0031667	0.9944558	0.0024145
ENSBTAG00000004801	-0.168705	0.5719384	0.2426508

ENSBTAG00000004802	-0.11342	0.8338667	0.0789034
ENSBTAG00000004803	-0.177517	0.4931088	0.3070573
ENSBTAG00000004804	-0.064634	0.7983671	0.0977974
ENSBTAG00000004805	-0.381548	0.1782324	0.7490134
ENSBTAG00000004806	-0.390613	0.1238446	0.9071231
ENSBTAG00000004813	NA	NA	NA
ENSBTAG00000004814	-0.283179	0.3092603	0.5096759
ENSBTAG00000004815	-0.061134	0.8526294	0.0692397
ENSBTAG00000004820	-0.02288	0.9276685	0.0326072
ENSBTAG00000004822	NA	NA	NA
ENSBTAG00000004823	-0.075552	0.77416	0.1111693
ENSBTAG00000004824	-0.381366	0.1383142	0.8591334
ENSBTAG00000004826	0.1198852	0.8097911	0.091627
ENSBTAG00000004829	0.1850683	0.4587656	0.3384091
ENSBTAG00000004830	NA	NA	NA
ENSBTAG00000004832	-0.5469	0.035297	1.4522618
ENSBTAG00000004833	0.0551681	0.8560729	0.0674892
ENSBTAG00000004834	-0.175407	0.5564545	0.2545703
ENSBTAG00000004835	0.4454799	0.2232902	0.6511304
ENSBTAG00000004836	-1.044056	0.0166251	1.7792367
ENSBTAG00000004838	NA	NA	NA
ENSBTAG00000004840	0.1084714	0.6793869	0.1678828
ENSBTAG00000004842	-0.373234	0.2851609	0.54491
ENSBTAG00000004844	-0.105294	0.7096707	0.1489431
ENSBTAG00000004848	0.3995511	0.3816588	0.4183247
ENSBTAG00000004849	-0.064195	0.8543427	0.0683679
ENSBTAG00000004850	-0.202555	0.4396479	0.356895
ENSBTAG00000004851	0.155643	0.700325	0.1547003
ENSBTAG00000004852	-0.012955	0.9698968	0.0132745
ENSBTAG00000004855	-0.073756	0.7727596	0.1119556
ENSBTAG00000004856	-0.518533	0.0419949	1.3768032
ENSBTAG00000004860	0.0192011	0.9693322	0.0135274
ENSBTAG00000004861	0.3502094	0.2142312	0.6691173
ENSBTAG00000004862	-0.845219	0.0298424	1.5251662
ENSBTAG00000004863	0.6005814	0.1231472	0.9095756
ENSBTAG00000004864	0.3519431	0.3411539	0.4670497
ENSBTAG00000004869	0.5033294	0.2077056	0.6825519
ENSBTAG00000004870	0.1411049	0.6367224	0.1960499
ENSBTAG00000004871	-0.206693	0.421062	0.3756539
ENSBTAG00000004872	0.0251576	0.9302901	0.0313816
ENSBTAG00000004873	0.1137043	0.7087102	0.1495313
ENSBTAG00000004874	-0.062819	0.8165368	0.0880243
ENSBTAG00000004875	0.1773031	0.5928327	0.2270679
ENSBTAG00000004876	0.221647	0.4939377	0.3063278
ENSBTAG00000004877	NA	NA	NA
ENSBTAG00000004878	NA	NA	NA
ENSBTAG00000004879	-0.086918	0.7426301	0.1292275
ENSBTAG00000004881	-0.10915	0.8158289	0.0884009
ENSBTAG00000004884	NA	NA	NA
ENSBTAG00000004885	-0.221996	0.5557517	0.2551192
ENSBTAG00000004886	NA	NA	NA
ENSBTAG00000004887	-0.066601	0.7979803	0.0980078
ENSBTAG00000004888	NA	NA	NA
ENSBTAG00000004891	0.1391291	0.6824362	0.165938
ENSBTAG00000004893	0.7461486	0.032497	1.488157
ENSBTAG00000004894	NA	NA	NA
ENSBTAG00000004895	-0.026334	0.9459172	0.0241469
ENSBTAG00000004896	0.1637352	0.5711022	0.2432861

ENSBTAG00000004899	0.3386159	0.269234	0.5698701
ENSBTAG00000004901	-0.31704	0.1878276	0.7262407
ENSBTAG00000004902	-0.022065	0.9370263	0.0282482
ENSBTAG00000004905	NA	NA	NA
ENSBTAG00000004906	0.9714875	0.0524486	1.280266
ENSBTAG00000004907	0.1867382	0.570675	0.2436112
ENSBTAG00000004908	-0.6885	0.1443697	0.840524
ENSBTAG00000004910	-0.222237	0.3998824	0.3980677
ENSBTAG00000004912	0.2308653	0.4685357	0.3292573
ENSBTAG00000004913	0.4485663	0.1876797	0.7265827
ENSBTAG00000004915	-0.067063	0.7996522	0.0970989
ENSBTAG00000004916	0.5109409	0.1106477	0.9560577
ENSBTAG00000004917	NA	NA	NA
ENSBTAG00000004920	0.4866174	0.1833457	0.7367293
ENSBTAG00000004921	NA	NA	NA
ENSBTAG00000004922	-0.670377	0.0134256	1.8720663
ENSBTAG00000004924	NA	NA	NA
ENSBTAG00000004925	1.4754968	0.0023314	2.632389
ENSBTAG00000004926	0.8180966	0.1125853	0.9485184
ENSBTAG00000004929	-0.328306	0.3504602	0.4553613
ENSBTAG00000004930	0.4439164	0.2239495	0.6498499
ENSBTAG00000004931	-0.574853	0.2016397	0.695424
ENSBTAG00000004933	-0.145177	0.5555637	0.2552662
ENSBTAG00000004934	-0.077848	0.7576849	0.1205114
ENSBTAG00000004936	NA	NA	NA
ENSBTAG00000004937	0.3811409	0.1769156	0.7522339
ENSBTAG00000004939	-0.405641	0.1629673	0.7878995
ENSBTAG00000004940	NA	NA	NA
ENSBTAG00000004942	0.0193014	0.9393696	0.0271635
ENSBTAG00000004943	NA	NA	NA
ENSBTAG00000004945	0.3330638	0.4502614	0.3465353
ENSBTAG00000004948	NA	NA	NA
ENSBTAG00000004949	NA	NA	NA
ENSBTAG00000004950	0.3374265	0.3799885	0.4202295
ENSBTAG00000004951	NA	NA	NA
ENSBTAG00000004952	NA	NA	NA
ENSBTAG00000004953	0.1811393	0.5083364	0.2938488
ENSBTAG00000004954	NA	NA	NA
ENSBTAG00000004956	NA	NA	NA
ENSBTAG00000004958	NA	NA	NA
ENSBTAG00000004961	0.0726212	0.7783896	0.108803
ENSBTAG00000004962	0.2751439	0.5455244	0.2631858
ENSBTAG00000004964	-0.148747	0.5516857	0.2583082
ENSBTAG00000004965	0.1726369	0.5501972	0.2594816
ENSBTAG00000004966	-0.574045	0.0371143	1.4304593
ENSBTAG00000004969	0.4099048	0.293602	0.532241
ENSBTAG00000004970	0.3859497	0.3945178	0.4039334
ENSBTAG00000004971	0.8160454	0.0447682	1.3490304
ENSBTAG00000004974	-1.120992	0.0021073	2.6762802
ENSBTAG00000004975	NA	NA	NA
ENSBTAG00000004976	-0.233634	0.520418	0.2836477
ENSBTAG00000004977	0.5801642	0.1268636	0.8966629
ENSBTAG00000004979	-0.138541	0.6034915	0.2193288
ENSBTAG00000004982	NA	NA	NA
ENSBTAG00000004987	-0.392096	0.4854951	0.3138151
ENSBTAG00000004989	0.6145726	0.1945809	0.7108999
ENSBTAG00000004990	-0.737786	0.0055542	2.2553783
ENSBTAG00000004991	-0.287183	0.2710887	0.5668886

ENSBTAG00000004992	-0.216821	0.3863768	0.412989
ENSBTAG00000004993	-0.063469	0.8930794	0.0491099
ENSBTAG00000004995	0.0426544	0.8686116	0.0611744
ENSBTAG00000004996	-0.231986	0.4723823	0.3257064
ENSBTAG00000004997	0.2057557	0.4582084	0.3389369
ENSBTAG00000004999	-0.067462	0.8027615	0.0954135
ENSBTAG00000005002	-0.131876	0.7906837	0.1019972
ENSBTAG00000005003	NA	NA	NA
ENSBTAG00000005008	-0.116565	0.6785836	0.1683966
ENSBTAG00000005009	0.459906	0.2055278	0.6871294
ENSBTAG00000005010	-2.360627	0.0026633	2.5745796
ENSBTAG00000005012	-0.33558	0.4411122	0.355451
ENSBTAG00000005014	0.0068644	0.9853836	0.0063947
ENSBTAG00000005015	0.3799459	0.3675282	0.4347093
ENSBTAG00000005016	0.016378	0.9495684	0.0224738
ENSBTAG00000005017	0.129684	0.738878	0.1314273
ENSBTAG00000005018	NA	NA	NA
ENSBTAG00000005019	NA	NA	NA
ENSBTAG00000005021	1.4039716	0.0001854	3.731909
ENSBTAG00000005025	0.0527753	0.8454957	0.0728886
ENSBTAG00000005026	0.1627724	0.5736058	0.2413865
ENSBTAG00000005027	-0.075765	0.7626605	0.1176687
ENSBTAG00000005028	0.0180793	0.9656678	0.0151723
ENSBTAG00000005029	-0.043095	0.9179384	0.0371864
ENSBTAG00000005030	0.5785926	0.1039165	0.9833154
ENSBTAG00000005031	NA	NA	NA
ENSBTAG00000005033	NA	NA	NA
ENSBTAG00000005034	-0.254827	0.4819712	0.3169789
ENSBTAG00000005038	NA	NA	NA
ENSBTAG00000005039	0.0828223	0.74383	0.1285263
ENSBTAG00000005040	-0.116463	0.6514009	0.1861517
ENSBTAG00000005041	NA	NA	NA
ENSBTAG00000005042	NA	NA	NA
ENSBTAG00000005043	0.1372424	0.7458329	0.1273585
ENSBTAG00000005048	-0.606618	0.0264485	1.5775993
ENSBTAG00000005049	NA	NA	NA
ENSBTAG00000005052	NA	NA	NA
ENSBTAG00000005055	NA	NA	NA
ENSBTAG00000005057	-0.27102	0.2708151	0.5673271
ENSBTAG00000005058	0.4229719	0.2120459	0.6735701
ENSBTAG00000005062	-0.76394	0.1469272	0.8328979
ENSBTAG00000005063	-0.239721	0.6212626	0.2067248
ENSBTAG00000005064	0.6560462	0.0759709	1.1193528
ENSBTAG00000005066	-0.046097	0.8988863	0.0462953
ENSBTAG00000005067	0.0167651	0.9485034	0.0229611
ENSBTAG00000005068	0.0143559	0.9699117	0.0132678
ENSBTAG00000005069	0.619178	0.0818915	1.0867613
ENSBTAG00000005071	-0.490547	0.0650929	1.1864665
ENSBTAG00000005072	-0.856168	0.2014404	0.6958535
ENSBTAG00000005073	0.5054795	0.2485852	0.6045248
ENSBTAG00000005075	-0.199931	0.4968318	0.3037906
ENSBTAG00000005076	0.285508	0.4818486	0.3170894
ENSBTAG00000005077	0.1480611	0.6008552	0.2212302
ENSBTAG00000005078	-0.24659	0.6592363	0.1809589
ENSBTAG00000005082	-0.258845	0.3172377	0.4986152
ENSBTAG00000005083	-0.308181	0.284901	0.5453061
ENSBTAG00000005085	0.7967468	0.0096446	2.0157173
ENSBTAG00000005086	0.2171524	0.4330673	0.3634446

ENSBTAG00000005089	0.4766667	0.2645253	0.5775327
ENSBTAG00000005090	0.2438096	0.344434	0.462894
ENSBTAG00000005091	NA	NA	NA
ENSBTAG00000005092	NA	NA	NA
ENSBTAG00000005093	0.2041867	0.615381	0.2108559
ENSBTAG00000005094	NA	NA	NA
ENSBTAG00000005096	0.3681087	0.2754492	0.5599585
ENSBTAG00000005100	0.099816	0.6880866	0.1623569
ENSBTAG00000005102	-0.722029	0.00742	2.1295981
ENSBTAG00000005104	NA	NA	NA
ENSBTAG00000005105	0.4338752	0.1994522	0.7001612
ENSBTAG00000005106	NA	NA	NA
ENSBTAG00000005107	-1.139037	0.0012012	2.9203926
ENSBTAG00000005108	-0.174718	0.7254614	0.1393857
ENSBTAG00000005110	-0.060626	0.8828349	0.0541205
ENSBTAG00000005111	-0.356464	0.3601772	0.4434838
ENSBTAG00000005115	0.0058568	0.9869959	0.0056847
ENSBTAG00000005116	0.564814	0.0937163	1.028185
ENSBTAG00000005119	-0.01207	0.9613468	0.0171199
ENSBTAG00000005120	NA	NA	NA
ENSBTAG00000005122	NA	NA	NA
ENSBTAG00000005124	-0.227329	0.5715643	0.2429349
ENSBTAG00000005127	-0.020924	0.9355205	0.0289467
ENSBTAG00000005128	NA	NA	NA
ENSBTAG00000005129	NA	NA	NA
ENSBTAG00000005133	NA	NA	NA
ENSBTAG00000005136	-0.031632	0.9097801	0.0410636
ENSBTAG00000005137	0.6608854	0.0211188	1.6753315
ENSBTAG00000005138	0.3621365	0.2952414	0.5298227
ENSBTAG00000005140	-0.230296	0.4803921	0.3184042
ENSBTAG00000005141	-0.052233	0.8948209	0.0482639
ENSBTAG00000005142	0.0576847	0.827902	0.0820211
ENSBTAG00000005143	NA	NA	NA
ENSBTAG00000005144	0.9692832	0.001053	2.9775663
ENSBTAG00000005145	0.1268881	0.6978053	0.1562657
ENSBTAG00000005146	0.4544478	0.2526165	0.5975382
ENSBTAG00000005147	-0.065845	0.8105322	0.0912298
ENSBTAG00000005148	NA	NA	NA
ENSBTAG00000005149	NA	NA	NA
ENSBTAG00000005150	NA	NA	NA
ENSBTAG00000005151	0.4647066	0.1743203	0.7586519
ENSBTAG00000005152	0.5680066	0.1115974	0.9523461
ENSBTAG00000005154	0.2651919	0.4087086	0.3885863
ENSBTAG00000005155	-0.101459	0.7426237	0.1292312
ENSBTAG00000005156	-0.259712	0.565306	0.2477164
ENSBTAG00000005158	-0.080779	0.7615986	0.1182738
ENSBTAG00000005160	-0.026043	0.9278631	0.0325161
ENSBTAG00000005161	0.4179446	0.1969135	0.7057245
ENSBTAG00000005162	NA	NA	NA
ENSBTAG00000005163	0.0925793	0.7705338	0.1132083
ENSBTAG00000005164	NA	NA	NA
ENSBTAG00000005165	0.9751218	0.022304	1.6516177
ENSBTAG00000005166	0.1776857	0.4798051	0.3189352
ENSBTAG00000005168	NA	NA	NA
ENSBTAG00000005169	NA	NA	NA
ENSBTAG00000005170	NA	NA	NA
ENSBTAG00000005174	0.4188889	0.3759859	0.4248284
ENSBTAG00000005176	NA	NA	NA

ENSBTAG00000005178	0.0204756	0.9574734	0.0188733
ENSBTAG00000005180	-0.934707	0.0014604	2.8355258
ENSBTAG00000005181	0.3533552	0.1998669	0.6992591
ENSBTAG00000005182	0.857619	0.136071	0.8662345
ENSBTAG00000005183	-0.068753	0.8382324	0.0766356
ENSBTAG00000005184	NA	NA	NA
ENSBTAG00000005186	-0.119666	0.6330726	0.1985465
ENSBTAG00000005189	NA	NA	NA
ENSBTAG00000005190	0.2118135	0.4328665	0.363646
ENSBTAG00000005191	0.1259401	0.6200646	0.2075631
ENSBTAG00000005193	NA	NA	NA
ENSBTAG00000005195	0.1908064	0.6426277	0.1920406
ENSBTAG00000005196	0.0430342	0.9088849	0.0414911
ENSBTAG00000005197	0.1153008	0.649324	0.1875386
ENSBTAG00000005198	0.8415362	0.0203402	1.6916447
ENSBTAG00000005202	NA	NA	NA
ENSBTAG00000005203	0.042875	0.9333408	0.0299597
ENSBTAG00000005205	-0.014272	0.9722401	0.0122265
ENSBTAG00000005206	0.3664268	0.4460357	0.3506304
ENSBTAG00000005207	0.2003606	0.5525289	0.257645
ENSBTAG00000005208	0.4333133	0.1628321	0.7882599
ENSBTAG00000005209	-0.16871	0.539145	0.2682944
ENSBTAG00000005210	0.0621056	0.8481185	0.0715435
ENSBTAG00000005211	-0.04655	0.8523989	0.0693571
ENSBTAG00000005212	0.0313244	0.9240727	0.0342938
ENSBTAG00000005213	-0.368272	0.2469923	0.6073165
ENSBTAG00000005214	NA	NA	NA
ENSBTAG00000005215	0.1585121	0.7189469	0.1433032
ENSBTAG00000005217	NA	NA	NA
ENSBTAG00000005218	0.2307435	0.6444759	0.1907933
ENSBTAG00000005219	NA	NA	NA
ENSBTAG00000005221	-0.08771	0.726412	0.138817
ENSBTAG00000005222	NA	NA	NA
ENSBTAG00000005225	-0.492597	0.0460052	1.3371927
ENSBTAG00000005227	0.1606019	0.5427951	0.2653641
ENSBTAG00000005230	0.6871127	0.185254	0.7322325
ENSBTAG00000005231	NA	NA	NA
ENSBTAG00000005234	NA	NA	NA
ENSBTAG00000005235	NA	NA	NA
ENSBTAG00000005236	NA	NA	NA
ENSBTAG00000005238	NA	NA	NA
ENSBTAG00000005239	-0.215688	0.6153902	0.2108494
ENSBTAG00000005240	0.282814	0.4235767	0.373068
ENSBTAG00000005243	NA	NA	NA
ENSBTAG00000005244	0.8533149	0.0311001	1.5072387
ENSBTAG00000005246	0.2478185	0.3261787	0.4865444
ENSBTAG00000005247	-0.257606	0.4545263	0.342441
ENSBTAG00000005248	NA	NA	NA
ENSBTAG00000005249	-0.193051	0.4606723	0.3366079
ENSBTAG00000005250	0.686317	0.1104344	0.9568955
ENSBTAG00000005251	NA	NA	NA
ENSBTAG00000005252	NA	NA	NA
ENSBTAG00000005257	0.0008547	0.9982781	0.0007485
ENSBTAG00000005259	1.5105396	4.68E-08	7.3299732
ENSBTAG00000005260	NA	NA	NA
ENSBTAG00000005261	NA	NA	NA
ENSBTAG00000005263	0.0525184	0.8685364	0.061212
ENSBTAG00000005265	-0.644762	0.1777219	0.7502589

ENSBTAG00000005269	NA	NA	NA
ENSBTAG00000005272	-0.057989	0.8747636	0.0581093
ENSBTAG00000005273	0.6400839	0.1124328	0.9491068
ENSBTAG00000005275	-0.486986	0.172123	0.7641611
ENSBTAG00000005278	-0.568791	0.1523365	0.8171961
ENSBTAG00000005280	0.4301451	0.1307986	0.8833968
ENSBTAG00000005284	NA	NA	NA
ENSBTAG00000005285	0.1795018	0.5029837	0.2984461
ENSBTAG00000005286	NA	NA	NA
ENSBTAG00000005287	NA	NA	NA
ENSBTAG00000005288	NA	NA	NA
ENSBTAG00000005289	-0.038201	0.9377193	0.0279271
ENSBTAG00000005290	NA	NA	NA
ENSBTAG00000005293	NA	NA	NA
ENSBTAG00000005294	NA	NA	NA
ENSBTAG00000005295	-0.036273	0.9125992	0.0397199
ENSBTAG00000005296	-0.107706	0.6767687	0.1695597
ENSBTAG00000005299	-0.425499	0.2184095	0.6607284
ENSBTAG00000005300	-0.415622	0.3738673	0.4272825
ENSBTAG00000005301	NA	NA	NA
ENSBTAG00000005304	0.2034508	0.5836397	0.2338551
ENSBTAG00000005305	NA	NA	NA
ENSBTAG00000005308	NA	NA	NA
ENSBTAG00000005309	0.2791648	0.4807646	0.3180675
ENSBTAG00000005310	0.2836246	0.5227229	0.2817285
ENSBTAG00000005311	0.2939496	0.4818605	0.3170786
ENSBTAG00000005312	0.1426614	0.7441158	0.1283595
ENSBTAG00000005313	NA	NA	NA
ENSBTAG00000005314	-0.080481	0.7519294	0.123823
ENSBTAG00000005315	-0.014701	0.953266	0.0207859
ENSBTAG00000005316	0.0665911	0.7861846	0.1044754
ENSBTAG00000005318	-0.354638	0.1930174	0.7144035
ENSBTAG00000005321	NA	NA	NA
ENSBTAG00000005326	-0.037315	0.8889306	0.0511322
ENSBTAG00000005327	NA	NA	NA
ENSBTAG00000005328	0.296555	0.6207592	0.2070768
ENSBTAG00000005333	-0.109193	0.6655828	0.1767979
ENSBTAG00000005337	NA	NA	NA
ENSBTAG00000005338	-0.09509	0.7128732	0.1469877
ENSBTAG00000005339	0.6431122	0.0430333	1.3661958
ENSBTAG00000005340	-0.026105	0.9555138	0.019763
ENSBTAG00000005344	-0.191896	0.5966059	0.2243124
ENSBTAG00000005345	0.0396972	0.8800648	0.0554853
ENSBTAG00000005347	0.0293501	0.9296659	0.0316731
ENSBTAG00000005349	0.0164367	0.9492396	0.0226241
ENSBTAG00000005351	0.3736895	0.3238275	0.4896863
ENSBTAG00000005353	0.111978	0.6777894	0.1689052
ENSBTAG00000005354	-0.526344	0.0408047	1.3892898
ENSBTAG00000005355	0.2669772	0.379276	0.4210447
ENSBTAG00000005356	0.1653361	0.5871191	0.2312738
ENSBTAG00000005357	0.2484571	0.3569336	0.4474126
ENSBTAG00000005359	-0.270884	0.3920004	0.4067135
ENSBTAG00000005362	0.1251901	0.6807122	0.1670365
ENSBTAG00000005363	NA	NA	NA
ENSBTAG00000005364	0.2242131	0.4340987	0.3624116
ENSBTAG00000005365	NA	NA	NA
ENSBTAG00000005367	0.1293645	0.6880295	0.162393
ENSBTAG00000005370	-0.254621	0.478732	0.3199075

ENSBTAG00000005371	0.2597021	0.5033569	0.298124
ENSBTAG00000005372	NA	NA	NA
ENSBTAG00000005373	-0.424007	0.1050974	0.9784082
ENSBTAG00000005376	0.3399699	0.2394479	0.6207889
ENSBTAG00000005377	NA	NA	NA
ENSBTAG00000005378	NA	NA	NA
ENSBTAG00000005379	0.1844861	0.6054525	0.2179199
ENSBTAG00000005380	-0.318249	0.2318154	0.6348578
ENSBTAG00000005382	0.5515362	0.2092591	0.6793156
ENSBTAG00000005384	0.1295118	0.5970281	0.2240053
ENSBTAG00000005385	-0.090623	0.7907416	0.1019654
ENSBTAG00000005386	NA	NA	NA
ENSBTAG00000005390	0.2822136	0.5549825	0.2557207
ENSBTAG00000005392	-0.211687	0.4743761	0.3238772
ENSBTAG00000005393	-0.503348	0.071605	1.1450568
ENSBTAG00000005394	-0.176676	0.6029014	0.2197537
ENSBTAG00000005395	NA	NA	NA
ENSBTAG00000005396	NA	NA	NA
ENSBTAG00000005397	0.0792699	0.7510808	0.1243133
ENSBTAG00000005400	-0.033965	0.9231797	0.0347138
ENSBTAG00000005403	0.1399711	0.5971401	0.2239238
ENSBTAG00000005404	NA	NA	NA
ENSBTAG00000005408	-0.36474	0.2753985	0.5600384
ENSBTAG00000005410	NA	NA	NA
ENSBTAG00000005412	0.0208983	0.9455808	0.0243014
ENSBTAG00000005413	0.0832277	0.7947155	0.0997883
ENSBTAG00000005414	0.3481294	0.2634568	0.5792906
ENSBTAG00000005416	0.1758102	0.6642194	0.1776884
ENSBTAG00000005419	-0.198268	0.6846849	0.1645093
ENSBTAG00000005424	NA	NA	NA
ENSBTAG00000005425	NA	NA	NA
ENSBTAG00000005426	0.0478279	0.8485987	0.0712976
ENSBTAG00000005427	0.3736832	0.2353594	0.6282684
ENSBTAG00000005430	NA	NA	NA
ENSBTAG00000005431	-0.022163	0.9378816	0.027852
ENSBTAG00000005432	0.2448928	0.5284276	0.2770145
ENSBTAG00000005433	NA	NA	NA
ENSBTAG00000005434	0.6596067	0.1716237	0.7654227
ENSBTAG00000005436	0.0865137	0.7963022	0.0989221
ENSBTAG00000005439	-0.023992	0.9387166	0.0274655
ENSBTAG00000005441	-0.203643	0.4258841	0.3707085
ENSBTAG00000005442	NA	NA	NA
ENSBTAG00000005443	-0.277248	0.252261	0.5981499
ENSBTAG00000005444	0.1788301	0.4728137	0.32531
ENSBTAG00000005445	0.14016	0.7684762	0.1143696
ENSBTAG00000005446	0.2117656	0.4437793	0.352833
ENSBTAG00000005448	-0.414113	0.2951591	0.5299438
ENSBTAG00000005450	NA	NA	NA
ENSBTAG00000005453	0.2508923	0.4022806	0.3954709
ENSBTAG00000005454	0.5216263	0.193381	0.7135862
ENSBTAG00000005455	-0.067824	0.7872762	0.1038729
ENSBTAG00000005456	NA	NA	NA
ENSBTAG00000005458	0.1850145	0.5349168	0.2717138
ENSBTAG00000005461	NA	NA	NA
ENSBTAG00000005462	-0.02978	0.9067539	0.0425106
ENSBTAG00000005464	0.7036226	0.0170129	1.7692215
ENSBTAG00000005465	0.1244468	0.6309377	0.2000135
ENSBTAG00000005466	NA	NA	NA

ENSBTAG00000005469	0.1125374	0.6697376	0.1740953
ENSBTAG00000005470	0.8076159	0.0426411	1.3701714
ENSBTAG00000005474	0.02651	0.9468943	0.0236985
ENSBTAG00000005475	NA	NA	NA
ENSBTAG00000005476	-0.140017	0.6947812	0.1581519
ENSBTAG00000005477	0.1952961	0.5104808	0.2920206
ENSBTAG00000005478	0.0859773	0.8047405	0.0943441
ENSBTAG00000005479	NA	NA	NA
ENSBTAG00000005481	0.1619216	0.5183529	0.2853745
ENSBTAG00000005482	0.1001239	0.766376	0.1155581
ENSBTAG00000005483	-0.020486	0.9394962	0.027105
ENSBTAG00000005488	-0.012308	0.9606492	0.0174352
ENSBTAG00000005489	0.0697417	0.7735882	0.1114902
ENSBTAG00000005490	-0.008385	0.9856037	0.0062977
ENSBTAG00000005492	0.030254	0.9058409	0.0429481
ENSBTAG00000005493	0.580951	0.0775844	1.1102256
ENSBTAG00000005495	NA	NA	NA
ENSBTAG00000005496	0.0172292	0.9681706	0.0140481
ENSBTAG00000005497	-0.291823	0.2726491	0.5643959
ENSBTAG00000005498	-1.425153	0.0090407	2.0437956
ENSBTAG00000005499	NA	NA	NA
ENSBTAG00000005501	-0.195467	0.6108006	0.2141006
ENSBTAG00000005503	-0.595415	0.1120372	0.9506379
ENSBTAG00000005504	-0.475955	0.1771013	0.7517782
ENSBTAG00000005514	-0.104954	0.6777399	0.1689369
ENSBTAG00000005517	-0.01745	0.9470758	0.0236153
ENSBTAG00000005519	0.0188325	0.9552548	0.0198808
ENSBTAG00000005522	NA	NA	NA
ENSBTAG00000005523	0.278286	0.4903255	0.3095156
ENSBTAG00000005524	0.0033863	0.9897626	0.004469
ENSBTAG00000005525	NA	NA	NA
ENSBTAG00000005526	-0.271049	0.3066769	0.5133189
ENSBTAG00000005527	NA	NA	NA
ENSBTAG00000005530	NA	NA	NA
ENSBTAG00000005532	0.5248856	0.0911681	1.0401573
ENSBTAG00000005533	0.1095712	0.7325894	0.1351394
ENSBTAG00000005534	-0.438371	0.096721	1.0144794
ENSBTAG00000005536	-0.372596	0.1788692	0.7474645
ENSBTAG00000005540	NA	NA	NA
ENSBTAG00000005542	-0.052026	0.8387774	0.0763533
ENSBTAG00000005546	NA	NA	NA
ENSBTAG00000005547	0.5496572	0.4557544	0.3412691
ENSBTAG00000005550	-0.259852	0.3263163	0.4863612
ENSBTAG00000005556	0.1964548	0.6596942	0.1806573
ENSBTAG00000005557	0.4479759	0.0742488	1.1293106
ENSBTAG00000005560	NA	NA	NA
ENSBTAG00000005562	NA	NA	NA
ENSBTAG00000005564	0.0259373	0.9374568	0.0280487
ENSBTAG00000005565	0.1357562	0.751186	0.1242525
ENSBTAG00000005567	NA	NA	NA
ENSBTAG00000005571	-0.179522	0.4987422	0.3021239
ENSBTAG00000005572	0.0462108	0.8812176	0.0549169
ENSBTAG00000005573	0.1451497	0.686032	0.1636557
ENSBTAG00000005574	0.4913308	0.0857831	1.0665985
ENSBTAG00000005576	NA	NA	NA
ENSBTAG00000005577	-0.036998	0.889327	0.0509385
ENSBTAG00000005578	0.3584389	0.2701012	0.5684734
ENSBTAG00000005580	0.4945708	0.1375403	0.86157

ENSBTAG00000005581	NA	NA	NA
ENSBTAG00000005583	-0.448819	0.2961784	0.5284467
ENSBTAG00000005585	NA	NA	NA
ENSBTAG00000005586	-0.290899	0.6482071	0.1882863
ENSBTAG00000005587	0.6699082	0.0514635	1.2885005
ENSBTAG00000005588	0.3016929	0.5389956	0.2684148
ENSBTAG00000005589	0.3186559	0.3547245	0.4501089
ENSBTAG00000005592	0.0080978	0.9789402	0.0092438
ENSBTAG00000005595	-0.188773	0.4682596	0.3295134
ENSBTAG00000005596	NA	NA	NA
ENSBTAG00000005603	NA	NA	NA
ENSBTAG00000005604	-0.485115	0.1587318	0.7993362
ENSBTAG00000005606	-0.097542	0.7631819	0.117372
ENSBTAG00000005607	NA	NA	NA
ENSBTAG00000005609	NA	NA	NA
ENSBTAG00000005614	-0.04128	0.8993136	0.0460888
ENSBTAG00000005615	0.051861	0.8836693	0.0537102
ENSBTAG00000005617	-0.766067	0.1151303	0.9388102
ENSBTAG00000005620	0.063463	0.8051926	0.0941002
ENSBTAG00000005622	-0.219908	0.5269403	0.2782386
ENSBTAG00000005623	NA	NA	NA
ENSBTAG00000005627	-0.164167	0.5009475	0.3002078
ENSBTAG00000005628	-0.301966	0.4862456	0.3131443
ENSBTAG00000005629	NA	NA	NA
ENSBTAG00000005630	0.0533449	0.8864543	0.0523437
ENSBTAG00000005631	0.1494019	0.6095801	0.2149692
ENSBTAG00000005633	0.5237778	0.2416176	0.6168714
ENSBTAG00000005634	-0.148068	0.6785589	0.1684125
ENSBTAG00000005635	0.2415892	0.4918334	0.308182
ENSBTAG00000005638	NA	NA	NA
ENSBTAG00000005644	NA	NA	NA
ENSBTAG00000005647	NA	NA	NA
ENSBTAG00000005650	0.0517708	0.8704153	0.0602735
ENSBTAG00000005652	NA	NA	NA
ENSBTAG00000005653	NA	NA	NA
ENSBTAG00000005654	0.0332268	0.8960834	0.0476515
ENSBTAG00000005657	NA	NA	NA
ENSBTAG00000005658	0.5014617	0.2810825	0.5511662
ENSBTAG00000005660	-0.031691	0.8994758	0.0460105
ENSBTAG00000005661	-0.169963	0.4874285	0.3120891
ENSBTAG00000005663	-0.165484	0.5873082	0.2311339
ENSBTAG00000005664	-0.08177	0.7462529	0.127114
ENSBTAG00000005665	-0.197916	0.4234394	0.3732088
ENSBTAG00000005666	0.0143078	0.9536939	0.020591
ENSBTAG00000005667	NA	NA	NA
ENSBTAG00000005668	0.6170353	0.1572421	0.8034313
ENSBTAG00000005670	NA	NA	NA
ENSBTAG00000005672	NA	NA	NA
ENSBTAG00000005674	0.2111338	0.583726	0.2337909
ENSBTAG00000005675	NA	NA	NA
ENSBTAG00000005676	0.0062669	0.9870009	0.0056824
ENSBTAG00000005678	-0.311855	0.4043026	0.3932935
ENSBTAG00000005679	NA	NA	NA
ENSBTAG00000005681	-0.044582	0.9122199	0.0399005
ENSBTAG00000005682	-0.141583	0.569742	0.2443218
ENSBTAG00000005683	0.1581755	0.6238538	0.2049172
ENSBTAG00000005685	NA	NA	NA
ENSBTAG00000005688	0.2768452	0.3635562	0.4394284

ENSBTAG00000005691	-0.084537	0.8068995	0.0931806
ENSBTAG00000005693	-0.193166	0.4484168	0.3483181
ENSBTAG00000005694	0.2184911	0.3858478	0.413584
ENSBTAG00000005695	0.1200482	0.7063575	0.1509755
ENSBTAG00000005696	NA	NA	NA
ENSBTAG00000005697	NA	NA	NA
ENSBTAG00000005699	NA	NA	NA
ENSBTAG00000005700	0.300976	0.5747749	0.2405022
ENSBTAG00000005702	NA	NA	NA
ENSBTAG00000005704	NA	NA	NA
ENSBTAG00000005708	0.0338332	0.9465617	0.0238511
ENSBTAG00000005709	1.1249161	0.0056408	2.2486595
ENSBTAG00000005710	-0.203439	0.5761978	0.2394284
ENSBTAG00000005711	NA	NA	NA
ENSBTAG00000005712	0.2809857	0.3570033	0.4473278
ENSBTAG00000005714	1.1886082	0.0515582	1.2877025
ENSBTAG00000005715	0.8505323	0.0056518	2.2478121
ENSBTAG00000005716	NA	NA	NA
ENSBTAG00000005717	NA	NA	NA
ENSBTAG00000005718	0.5459883	0.0849647	1.0707615
ENSBTAG00000005723	-0.047253	0.9245502	0.0340695
ENSBTAG00000005724	-0.224541	0.5188999	0.2849164
ENSBTAG00000005725	NA	NA	NA
ENSBTAG00000005726	-0.338967	0.2031964	0.692084
ENSBTAG00000005728	NA	NA	NA
ENSBTAG00000005729	-0.386556	0.2099478	0.6778887
ENSBTAG00000005730	0.3404918	0.2346837	0.6295171
ENSBTAG00000005732	-0.108765	0.7235417	0.1405364
ENSBTAG00000005733	0.5814737	0.0564537	1.2483078
ENSBTAG00000005734	NA	NA	NA
ENSBTAG00000005735	-0.17091	0.511121	0.2914763
ENSBTAG00000005738	-0.35052	0.3466868	0.4600627
ENSBTAG00000005742	0.3666838	0.3628846	0.4402315
ENSBTAG00000005743	-0.061845	0.8434593	0.0739359
ENSBTAG00000005744	-0.2562	0.361359	0.4420611
ENSBTAG00000005745	NA	NA	NA
ENSBTAG00000005748	-0.283479	0.4006081	0.3972803
ENSBTAG00000005750	0.0083917	0.9861651	0.0060504
ENSBTAG00000005751	0.2993979	0.3257522	0.4871126
ENSBTAG00000005753	-0.442141	0.0850469	1.0703415
ENSBTAG00000005754	-1.70093	0.0001895	3.7224068
ENSBTAG00000005756	0.6603954	0.1530802	0.8150811
ENSBTAG00000005757	0.2946712	0.3069647	0.5129116
ENSBTAG00000005760	-0.492632	0.0748309	1.1259193
ENSBTAG00000005761	-0.393306	0.2680032	0.5718601
ENSBTAG00000005762	0.1168254	0.7046125	0.1520497
ENSBTAG00000005763	0.0963762	0.8299564	0.0809447
ENSBTAG00000005765	0.034681	0.9156858	0.0382535
ENSBTAG00000005766	0.1165977	0.6709683	0.173298
ENSBTAG00000005769	0.4490129	0.2429345	0.6145108
ENSBTAG00000005772	NA	NA	NA
ENSBTAG00000005773	0.1674854	0.4927992	0.30733
ENSBTAG00000005776	NA	NA	NA
ENSBTAG00000005780	0.1099441	0.769721	0.1136666
ENSBTAG00000005784	-0.345645	0.4631699	0.3342597
ENSBTAG00000005785	0.0411721	0.898349	0.0465549
ENSBTAG00000005786	0.0274135	0.9337996	0.0297463
ENSBTAG00000005787	0.1397066	0.6993373	0.1553133

ENSBTAG00000005788	0.5169438	0.164889	0.7828083
ENSBTAG00000005791	0.2000772	0.6202087	0.2074622
ENSBTAG00000005792	-0.195545	0.4595435	0.3376734
ENSBTAG00000005793	0.0646991	0.8051635	0.0941159
ENSBTAG00000005795	-0.098719	0.7420814	0.1295485
ENSBTAG00000005796	-0.024774	0.9197302	0.0363395
ENSBTAG00000005799	0.1996029	0.557121	0.2540505
ENSBTAG00000005800	NA	NA	NA
ENSBTAG00000005803	-0.08437	0.8257867	0.0831321
ENSBTAG00000005805	NA	NA	NA
ENSBTAG00000005807	-0.35528	0.1599896	0.7959083
ENSBTAG00000005808	-0.099496	0.7082898	0.149789
ENSBTAG00000005809	0.0506746	0.8627193	0.0641305
ENSBTAG00000005810	NA	NA	NA
ENSBTAG00000005812	NA	NA	NA
ENSBTAG00000005814	-0.292306	0.2653754	0.5761394
ENSBTAG00000005815	0.2325317	0.3978095	0.4003249
ENSBTAG00000005816	-0.332003	0.4749222	0.3233775
ENSBTAG00000005819	0.3668548	0.4225241	0.3741485
ENSBTAG00000005824	-0.120295	0.6459782	0.1897822
ENSBTAG00000005825	NA	NA	NA
ENSBTAG00000005826	0.1943451	0.4346405	0.3618698
ENSBTAG00000005827	-0.024611	0.9386096	0.027515
ENSBTAG00000005828	0.324817	0.4606883	0.3365928
ENSBTAG00000005830	-0.223982	0.4249277	0.371685
ENSBTAG00000005832	0.0897119	0.7379863	0.1319517
ENSBTAG00000005833	-1.176208	0.0016809	2.7744674
ENSBTAG00000005835	0.3023089	0.3331742	0.4773286
ENSBTAG00000005838	0.4300898	0.2008294	0.6971726
ENSBTAG00000005841	NA	NA	NA
ENSBTAG00000005842	0.5140725	0.188694	0.7242418
ENSBTAG00000005843	-0.182209	0.4590099	0.338178
ENSBTAG00000005844	0.543322	0.0566629	1.2467014
ENSBTAG00000005845	-0.046189	0.8838715	0.0536109
ENSBTAG00000005846	0.4048865	0.4203779	0.3763602
ENSBTAG00000005847	-0.62844	0.0414047	1.3829507
ENSBTAG00000005848	NA	NA	NA
ENSBTAG00000005850	NA	NA	NA
ENSBTAG00000005851	-0.181149	0.480555	0.3182569
ENSBTAG00000005852	NA	NA	NA
ENSBTAG00000005854	-0.125407	0.6125772	0.2128391
ENSBTAG00000005857	-0.117354	0.7740313	0.1112415
ENSBTAG00000005861	-0.071218	0.7841975	0.1055745
ENSBTAG00000005862	-0.253581	0.4186099	0.3781905
ENSBTAG00000005863	0.1832131	0.654947	0.1837939
ENSBTAG00000005865	0.1156895	0.6550976	0.183694
ENSBTAG00000005866	0.3262417	0.3821657	0.4177483
ENSBTAG00000005868	0.3935803	0.2973576	0.5267209
ENSBTAG00000005869	-0.203177	0.4334626	0.3630484
ENSBTAG00000005870	-0.310218	0.4583276	0.338824
ENSBTAG00000005871	0.0751791	0.8180573	0.0872163
ENSBTAG00000005882	0.2179001	0.492843	0.3072914
ENSBTAG00000005884	NA	NA	NA
ENSBTAG00000005885	NA	NA	NA
ENSBTAG00000005888	NA	NA	NA
ENSBTAG00000005891	NA	NA	NA
ENSBTAG00000005892	0.1916386	0.6447653	0.1905984
ENSBTAG00000005893	0.4142889	0.2263968	0.6451298

ENSBTAG00000005897	-0.003108	0.9929886	0.0030557
ENSBTAG00000005898	0.0885219	0.7909474	0.1018524
ENSBTAG00000005903	0.0328653	0.9144734	0.0388289
ENSBTAG00000005904	0.1415826	0.5666768	0.2466645
ENSBTAG00000005905	-0.059056	0.8241861	0.0839747
ENSBTAG00000005907	-0.1467	0.5789664	0.2373466
ENSBTAG00000005908	-0.730286	0.1010731	0.9953643
ENSBTAG00000005909	-0.212672	0.4839003	0.3152441
ENSBTAG00000005910	0.4899026	0.1949485	0.7100802
ENSBTAG00000005912	0.0775415	0.7655119	0.1160481
ENSBTAG00000005913	0.2119226	0.5238961	0.2807548
ENSBTAG00000005914	NA	NA	NA
ENSBTAG00000005915	0.6285537	0.0705252	1.1516555
ENSBTAG00000005916	0.0014638	0.9989146	0.0004716
ENSBTAG00000005917	0.3252997	0.4937246	0.3065152
ENSBTAG00000005922	NA	NA	NA
ENSBTAG00000005923	0.8977368	0.0548031	1.2611946
ENSBTAG00000005928	-0.502658	0.2771758	0.5572447
ENSBTAG00000005929	-1.213745	0.0050173	2.2995311
ENSBTAG00000005932	-0.39164	0.2552852	0.5929743
ENSBTAG00000005933	-0.282354	0.5525583	0.2576219
ENSBTAG00000005934	-0.155375	0.6939131	0.1586949
ENSBTAG00000005937	NA	NA	NA
ENSBTAG00000005940	0.2812109	0.3691437	0.4328045
ENSBTAG00000005945	0.6335822	0.1911686	0.7185835
ENSBTAG00000005946	-0.130997	0.6102136	0.2145181
ENSBTAG00000005947	0.2402791	0.4817291	0.3171971
ENSBTAG00000005951	NA	NA	NA
ENSBTAG00000005952	NA	NA	NA
ENSBTAG00000005954	NA	NA	NA
ENSBTAG00000005955	NA	NA	NA
ENSBTAG00000005957	-0.277865	0.2723501	0.5648725
ENSBTAG00000005958	0.4143811	0.3564651	0.447983
ENSBTAG00000005960	-0.497842	0.083787	1.0768231
ENSBTAG00000005961	-0.097715	0.7235585	0.1405263
ENSBTAG00000005964	0.3994179	0.1539729	0.8125556
ENSBTAG00000005965	NA	NA	NA
ENSBTAG00000005967	0.0640633	0.8050424	0.0941813
ENSBTAG00000005968	NA	NA	NA
ENSBTAG00000005969	-0.244089	0.3326783	0.4779755
ENSBTAG00000005970	-0.143266	0.5616285	0.2505509
ENSBTAG00000005971	NA	NA	NA
ENSBTAG00000005973	0.5630486	0.148815	0.8273534
ENSBTAG00000005974	-0.05196	0.8464092	0.0724196
ENSBTAG00000005975	-0.406852	0.2156659	0.6662186
ENSBTAG00000005976	-0.281917	0.3611364	0.4423287
ENSBTAG00000005978	NA	NA	NA
ENSBTAG00000005979	NA	NA	NA
ENSBTAG00000005980	0.2087473	0.4791974	0.3194855
ENSBTAG00000005984	-0.206199	0.4268364	0.3697385
ENSBTAG00000005985	NA	NA	NA
ENSBTAG00000005986	0.3128021	0.5187395	0.2850507
ENSBTAG00000005987	-0.280736	0.3215663	0.4927294
ENSBTAG00000005989	-0.024592	0.923456	0.0345838
ENSBTAG00000005990	0.2843277	0.2893837	0.5385259
ENSBTAG00000005994	NA	NA	NA
ENSBTAG00000005997	-0.477473	0.3366223	0.4728572
ENSBTAG00000005998	-0.042885	0.8972476	0.0470877

ENSBTAG00000005999	NA	NA	NA
ENSBTAG00000006001	NA	NA	NA
ENSBTAG00000006002	0.4121004	0.1243777	0.9052575
ENSBTAG00000006003	0.2522742	0.5270287	0.2781657
ENSBTAG00000006004	0.4699456	0.152783	0.8159249
ENSBTAG00000006005	-0.023941	0.961139	0.0172138
ENSBTAG00000006007	0.5996337	0.1161741	0.9348908
ENSBTAG00000006008	0.0129759	0.966556	0.014773
ENSBTAG00000006010	NA	NA	NA
ENSBTAG00000006013	-0.353317	0.3673149	0.4349614
ENSBTAG00000006014	0.0726129	0.7819942	0.1067965
ENSBTAG00000006015	-0.258656	0.4585045	0.3386564
ENSBTAG00000006016	0.470456	0.0997143	1.0012427
ENSBTAG00000006017	0.4131895	0.187303	0.7274554
ENSBTAG00000006019	NA	NA	NA
ENSBTAG00000006021	0.023023	0.9521076	0.0213139
ENSBTAG00000006022	-1.850413	0.0003878	3.4114269
ENSBTAG00000006024	-0.642908	0.0691701	1.1600813
ENSBTAG00000006025	-0.476977	0.3073719	0.5123358
ENSBTAG00000006026	-0.121061	0.7995702	0.0971434
ENSBTAG00000006027	-0.283575	0.2876727	0.5411014
ENSBTAG00000006029	0.0474687	0.8554942	0.0677829
ENSBTAG00000006030	0.4965335	0.1150477	0.9391219
ENSBTAG00000006031	-0.722946	0.137011	0.8632447
ENSBTAG00000006033	-0.120647	0.6580253	0.1817574
ENSBTAG00000006034	-0.058605	0.8432	0.0740694
ENSBTAG00000006035	-0.075376	0.874211	0.0583837
ENSBTAG00000006036	NA	NA	NA
ENSBTAG00000006037	0.077184	0.884144	0.053477
ENSBTAG00000006039	0.6504859	0.029388	1.5318295
ENSBTAG00000006040	0.287385	0.2961118	0.5285443
ENSBTAG00000006042	NA	NA	NA
ENSBTAG00000006043	-0.562592	0.2263004	0.6453148
ENSBTAG00000006044	-0.05106	0.8807928	0.0551263
ENSBTAG00000006045	0.3775712	0.1369904	0.86331
ENSBTAG00000006048	0.3188622	0.4673482	0.3303594
ENSBTAG00000006049	-0.12303	0.6343511	0.1976703
ENSBTAG00000006050	-0.059628	0.8461443	0.0725556
ENSBTAG00000006051	0.3567367	0.15213	0.8177852
ENSBTAG00000006052	-0.09469	0.7142472	0.1461515
ENSBTAG00000006054	-0.213867	0.5205275	0.2835563
ENSBTAG00000006056	-0.179905	0.5192878	0.2845919
ENSBTAG00000006059	-0.135337	0.6129877	0.2125482
ENSBTAG00000006060	NA	NA	NA
ENSBTAG00000006063	-0.240159	0.394119	0.4043726
ENSBTAG00000006064	NA	NA	NA
ENSBTAG00000006065	-0.101265	0.710137	0.1486579
ENSBTAG00000006066	-0.006471	0.9801306	0.008716
ENSBTAG00000006067	NA	NA	NA
ENSBTAG00000006068	0.2137222	0.4968599	0.3037661
ENSBTAG00000006069	NA	NA	NA
ENSBTAG00000006070	0.1013324	0.6976087	0.1563881
ENSBTAG00000006071	0.0577244	0.8135468	0.0896175
ENSBTAG00000006072	0.3405079	0.2780718	0.555843
ENSBTAG00000006073	NA	NA	NA
ENSBTAG00000006075	0.0095383	0.9701352	0.0131677
ENSBTAG00000006078	0.0311299	0.9537593	0.0205612
ENSBTAG00000006080	NA	NA	NA

ENSBTAG00000006081	0.1803851	0.5142422	0.2888323
ENSBTAG00000006082	-0.120784	0.7797155	0.1080638
ENSBTAG00000006083	NA	NA	NA
ENSBTAG00000006084	0.0349503	0.910371	0.0407816
ENSBTAG00000006085	0.2020319	0.432784	0.3637288
ENSBTAG00000006086	0.7282311	0.1316292	0.8806477
ENSBTAG00000006087	0.1551204	0.6169282	0.2097654
ENSBTAG00000006088	0.5032543	0.2603404	0.5844585
ENSBTAG00000006089	0.2763604	0.366672	0.4357222
ENSBTAG00000006095	NA	NA	NA
ENSBTAG00000006099	0.3595619	0.1555012	0.8082663
ENSBTAG00000006101	-0.065658	0.8027165	0.0954378
ENSBTAG00000006103	-0.088913	0.7502411	0.1247992
ENSBTAG00000006104	0.184965	0.4634939	0.333956
ENSBTAG00000006107	-0.119669	0.6796502	0.1677146
ENSBTAG00000006108	NA	NA	NA
ENSBTAG00000006110	NA	NA	NA
ENSBTAG00000006111	0.2523749	0.3804582	0.419693
ENSBTAG00000006112	-0.165963	0.5321465	0.2739688
ENSBTAG00000006114	-0.036057	0.9012895	0.0451357
ENSBTAG00000006116	0.0086121	0.9815851	0.008072
ENSBTAG00000006117	-0.043306	0.8858666	0.0526317
ENSBTAG00000006118	-0.179505	0.5177151	0.2859091
ENSBTAG00000006121	NA	NA	NA
ENSBTAG00000006122	-0.063182	0.8063768	0.093462
ENSBTAG00000006124	0.2583176	0.3236184	0.4899667
ENSBTAG00000006125	0.0499284	0.9046742	0.0435078
ENSBTAG00000006126	0.2245707	0.4293329	0.3672058
ENSBTAG00000006129	0.3350462	0.4007722	0.3971025
ENSBTAG00000006130	0.5105739	0.0647487	1.1887692
ENSBTAG00000006132	0.529105	0.0939448	1.0271273
ENSBTAG00000006134	0.2448223	0.369597	0.4322715
ENSBTAG00000006135	-0.134082	0.6402335	0.1936616
ENSBTAG00000006136	NA	NA	NA
ENSBTAG00000006138	-0.627888	0.014411	1.841305
ENSBTAG00000006139	0.5384471	0.1981611	0.7029816
ENSBTAG00000006140	-0.216322	0.5500705	0.2595816
ENSBTAG00000006141	NA	NA	NA
ENSBTAG00000006142	-0.055401	0.8271439	0.0824189
ENSBTAG00000006143	NA	NA	NA
ENSBTAG00000006150	-0.006029	0.9846263	0.0067286
ENSBTAG00000006155	1.1650574	0.00622	2.2062095
ENSBTAG00000006156	NA	NA	NA
ENSBTAG00000006157	0.2052876	0.4833541	0.3157346
ENSBTAG00000006158	0.1922391	0.6213689	0.2066505
ENSBTAG00000006159	NA	NA	NA
ENSBTAG00000006160	0.0197377	0.9541089	0.020402
ENSBTAG00000006161	-0.011425	0.9672645	0.0144547
ENSBTAG00000006162	0.1577484	0.6648731	0.1772613
ENSBTAG00000006165	-0.304676	0.2943566	0.5311263
ENSBTAG00000006166	-0.178948	0.5268646	0.278301
ENSBTAG00000006167	0.0861428	0.8148668	0.0889134
ENSBTAG00000006168	-0.003979	0.9888742	0.0048589
ENSBTAG00000006170	-0.035825	0.8919071	0.0496804
ENSBTAG00000006173	NA	NA	NA
ENSBTAG00000006175	-0.051998	0.8367619	0.0773981
ENSBTAG00000006176	NA	NA	NA
ENSBTAG00000006177	-0.0836	0.7481159	0.1260311

ENSBTAG00000006185	NA	NA	NA
ENSBTAG00000006186	0.0966008	0.7489686	0.1255364
ENSBTAG00000006187	0.0545211	0.8925412	0.0493717
ENSBTAG00000006188	NA	NA	NA
ENSBTAG00000006189	0.1399415	0.593458	0.22661
ENSBTAG00000006193	-0.59706	0.082944	1.0812152
ENSBTAG00000006194	NA	NA	NA
ENSBTAG00000006195	0.1718698	0.5819266	0.2351318
ENSBTAG00000006197	0.2434253	0.3630375	0.4400485
ENSBTAG00000006198	NA	NA	NA
ENSBTAG00000006199	-0.086173	0.728424	0.1376158
ENSBTAG00000006200	0.1606901	0.6339049	0.1979759
ENSBTAG00000006201	-0.001197	0.9976442	0.0010243
ENSBTAG00000006202	-0.400048	0.1055051	0.9767265
ENSBTAG00000006204	-0.045529	0.8896624	0.0507748
ENSBTAG00000006205	NA	NA	NA
ENSBTAG00000006208	-0.25817	0.5076326	0.2944505
ENSBTAG00000006209	-0.035698	0.9075615	0.042124
ENSBTAG00000006212	0.174621	0.7517757	0.1239117
ENSBTAG00000006213	NA	NA	NA
ENSBTAG00000006214	-0.04598	0.8866075	0.0522686
ENSBTAG00000006221	NA	NA	NA
ENSBTAG00000006222	-0.379033	0.1663596	0.7789521
ENSBTAG00000006223	0.4086573	0.3846054	0.4149847
ENSBTAG00000006225	-0.013982	0.9587512	0.0182941
ENSBTAG00000006227	-0.152263	0.5996884	0.2220743
ENSBTAG00000006231	NA	NA	NA
ENSBTAG00000006232	NA	NA	NA
ENSBTAG00000006233	-0.223359	0.5845721	0.2331619
ENSBTAG00000006234	0.4358435	0.2512467	0.5998997
ENSBTAG00000006235	0.2798754	0.3386411	0.4702604
ENSBTAG00000006239	NA	NA	NA
ENSBTAG00000006240	0.300778	0.3909542	0.4078742
ENSBTAG00000006241	-0.059927	0.8340701	0.0787974
ENSBTAG00000006242	-0.372666	0.1361144	0.8660959
ENSBTAG00000006243	NA	NA	NA
ENSBTAG00000006244	0.024267	0.9285676	0.0321865
ENSBTAG00000006245	-0.007656	0.9811922	0.0082459
ENSBTAG00000006246	-0.303851	0.2288577	0.6404345
ENSBTAG00000006247	-0.020113	0.9360443	0.0287036
ENSBTAG00000006250	0.2735213	0.2896538	0.5381208
ENSBTAG00000006252	-0.216303	0.6283601	0.2017914
ENSBTAG00000006253	0.5985959	0.0520446	1.2836241
ENSBTAG00000006255	0.0177551	0.9535151	0.0206724
ENSBTAG00000006256	NA	NA	NA
ENSBTAG00000006259	0.1661421	0.5018641	0.2994139
ENSBTAG00000006260	0.2253337	0.6307993	0.2001088
ENSBTAG00000006262	-0.070346	0.8252069	0.0834372
ENSBTAG00000006264	NA	NA	NA
ENSBTAG00000006268	0.6185891	0.06269	1.2028018
ENSBTAG00000006270	-0.119838	0.6387383	0.194677
ENSBTAG00000006272	0.189278	0.6239662	0.2048389
ENSBTAG00000006276	0.14524	0.7351736	0.1336101
ENSBTAG00000006277	0.4013079	0.2402401	0.6193545
ENSBTAG00000006278	NA	NA	NA
ENSBTAG00000006279	NA	NA	NA
ENSBTAG00000006280	NA	NA	NA
ENSBTAG00000006282	NA	NA	NA

ENSBTAG00000006287	0.3930895	0.1783286	0.7487789
ENSBTAG00000006288	0.831426	0.0217809	1.6619252
ENSBTAG00000006291	0.0103811	0.9670521	0.0145501
ENSBTAG00000006295	NA	NA	NA
ENSBTAG00000006296	-0.228617	0.3779333	0.4225848
ENSBTAG00000006305	-0.332888	0.2172143	0.6631116
ENSBTAG00000006307	0.0971644	0.7865005	0.104301
ENSBTAG00000006320	-0.128998	0.6281562	0.2019323
ENSBTAG00000006321	-0.15311	0.5516113	0.2583668
ENSBTAG00000006322	-0.024149	0.9235834	0.0345239
ENSBTAG00000006323	-0.009024	0.9714708	0.0125702
ENSBTAG00000006324	-0.274327	0.3739574	0.4271779
ENSBTAG00000006325	0.2546221	0.3206132	0.4940186
ENSBTAG00000006326	0.7792208	0.0605476	1.2179033
ENSBTAG00000006328	0.038231	0.8808432	0.0551014
ENSBTAG00000006330	-0.227705	0.4059961	0.3914781
ENSBTAG00000006335	0.2638993	0.4249404	0.371672
ENSBTAG00000006338	NA	NA	NA
ENSBTAG00000006341	NA	NA	NA
ENSBTAG00000006342	-0.254005	0.3186623	0.4966693
ENSBTAG00000006343	NA	NA	NA
ENSBTAG00000006345	-1.110402	0.0095174	2.0214812
ENSBTAG00000006346	0.0334486	0.9133374	0.0393688
ENSBTAG00000006347	-0.064187	0.8481696	0.0715173
ENSBTAG00000006349	0.054104	0.8921557	0.0495594
ENSBTAG00000006350	NA	NA	NA
ENSBTAG00000006352	NA	NA	NA
ENSBTAG00000006353	0.1365163	0.6984798	0.1558461
ENSBTAG00000006354	NA	NA	NA
ENSBTAG00000006355	NA	NA	NA
ENSBTAG00000006357	NA	NA	NA
ENSBTAG00000006359	NA	NA	NA
ENSBTAG00000006360	NA	NA	NA
ENSBTAG00000006364	1.0416553	0.0193068	1.7142897
ENSBTAG00000006366	0.199475	0.6660075	0.1765209
ENSBTAG00000006367	0.4977802	0.1126839	0.9481383
ENSBTAG00000006368	0.1699368	0.7442373	0.1282886
ENSBTAG00000006369	1.5629166	0.089696	1.0472267
ENSBTAG00000006370	0.1291999	0.6113025	0.2137438
ENSBTAG00000006371	0.0990771	0.7805372	0.1076064
ENSBTAG00000006372	0.6050422	0.2032203	0.6920329
ENSBTAG00000006373	NA	NA	NA
ENSBTAG00000006374	-0.028134	0.9174168	0.0374333
ENSBTAG00000006377	-0.15197	0.7658863	0.1158357
ENSBTAG00000006378	-0.140104	0.6684557	0.1749274
ENSBTAG00000006379	0.0315693	0.9277441	0.0325718
ENSBTAG00000006383	1.9173349	0.0031459	2.5022609
ENSBTAG00000006385	-0.003562	1	0
ENSBTAG00000006386	NA	NA	NA
ENSBTAG00000006387	NA	NA	NA
ENSBTAG00000006388	0.115351	0.7213455	0.1418567
ENSBTAG00000006391	0.0057339	0.9827479	0.0075579
ENSBTAG00000006392	0.1251854	0.763589	0.1171404
ENSBTAG00000006393	-0.066713	0.835655	0.077973
ENSBTAG00000006395	0.0570642	0.8226791	0.0847695
ENSBTAG00000006396	-0.493602	0.0689861	1.1612385
ENSBTAG00000006398	NA	NA	NA
ENSBTAG00000006403	0.5455574	0.2372445	0.6248039

ENSBTAG00000006404	0.0422185	0.9421829	0.0258648
ENSBTAG00000006405	0.2030724	0.5663254	0.246934
ENSBTAG00000006409	NA	NA	NA
ENSBTAG00000006410	0.0440077	0.8566268	0.0672084
ENSBTAG00000006411	-0.204181	0.4303813	0.3661466
ENSBTAG00000006413	-0.221438	0.4506671	0.3461442
ENSBTAG00000006414	0.1764029	0.4957124	0.3047702
ENSBTAG00000006416	0.3239133	0.306195	0.5140019
ENSBTAG00000006417	0.0951826	0.7374195	0.1322854
ENSBTAG00000006419	-0.098646	0.706155	0.1511
ENSBTAG00000006420	0.3401267	0.3021094	0.5198357
ENSBTAG00000006422	0.2101391	0.4473688	0.3493344
ENSBTAG00000006423	0.3915631	0.2365627	0.6260537
ENSBTAG00000006424	NA	NA	NA
ENSBTAG00000006428	0.1386791	0.6808416	0.1669539
ENSBTAG00000006429	0.2821547	0.3297477	0.4818182
ENSBTAG00000006432	0.0822221	0.8863579	0.0523909
ENSBTAG00000006434	-0.134954	0.5995398	0.222182
ENSBTAG00000006438	NA	NA	NA
ENSBTAG00000006439	-0.125785	0.645477	0.1901192
ENSBTAG00000006440	-1.212469	0.0013558	2.8677973
ENSBTAG00000006441	0.0356504	0.8983317	0.0465633
ENSBTAG00000006443	NA	NA	NA
ENSBTAG00000006446	NA	NA	NA
ENSBTAG00000006447	NA	NA	NA
ENSBTAG00000006448	0.4576803	0.2949749	0.5302149
ENSBTAG00000006449	0.1020097	0.8048452	0.0942877
ENSBTAG00000006452	0.0984741	0.82275	0.0847321
ENSBTAG00000006453	-0.274669	0.583452	0.2339949
ENSBTAG00000006457	NA	NA	NA
ENSBTAG00000006463	-0.174985	0.6275189	0.2023732
ENSBTAG00000006464	0.0669373	0.8063234	0.0934907
ENSBTAG00000006466	0.3015895	0.5044002	0.2972248
ENSBTAG00000006470	0.0799848	0.7494219	0.1252736
ENSBTAG00000006471	0.9785467	5.77E-05	4.2391103
ENSBTAG00000006472	NA	NA	NA
ENSBTAG00000006474	NA	NA	NA
ENSBTAG00000006478	0.6818321	0.1366626	0.8643504
ENSBTAG00000006479	0.0718983	0.7902063	0.1022595
ENSBTAG00000006481	0.472114	0.1169155	0.9321279
ENSBTAG00000006482	0.1339958	0.5876753	0.2308625
ENSBTAG00000006486	-0.23932	0.5202978	0.283748
ENSBTAG00000006487	0.1151323	0.6758958	0.1701203
ENSBTAG00000006489	0.0293989	0.9296935	0.0316602
ENSBTAG00000006490	NA	NA	NA
ENSBTAG00000006491	-0.60622	0.0173917	1.7596586
ENSBTAG00000006492	0.1766731	0.5460948	0.2627319
ENSBTAG00000006493	0.4432106	0.2201612	0.6572591
ENSBTAG00000006494	NA	NA	NA
ENSBTAG00000006495	0.0900077	0.7416135	0.1298224
ENSBTAG00000006499	-0.410485	0.0992933	1.00308
ENSBTAG00000006500	0.1700618	0.6260247	0.2034085
ENSBTAG00000006501	0.1240583	0.6410832	0.1930856
ENSBTAG00000006504	0.1680098	0.7055039	0.1515006
ENSBTAG00000006505	NA	NA	NA
ENSBTAG00000006506	0.214783	0.450624	0.3461857
ENSBTAG00000006507	-0.035732	0.9593332	0.0180305
ENSBTAG00000006510	0.2276445	0.5340309	0.2724336

ENSBTAG00000006511	-0.061636	0.8369797	0.0772851
ENSBTAG00000006515	NA	NA	NA
ENSBTAG00000006517	0.2820067	0.3389473	0.4698679
ENSBTAG00000006519	NA	NA	NA
ENSBTAG00000006520	NA	NA	NA
ENSBTAG00000006523	-0.124911	0.6264759	0.2030956
ENSBTAG00000006525	-0.048377	0.9100863	0.0409174
ENSBTAG00000006526	0.141603	0.6436339	0.1913611
ENSBTAG00000006528	0.0530661	0.8720582	0.0594545
ENSBTAG00000006529	0.4628225	0.2522677	0.5981383
ENSBTAG00000006531	-0.111001	0.6891032	0.1617157
ENSBTAG00000006532	-0.091409	0.768519	0.1143454
ENSBTAG00000006533	-0.027847	0.9120209	0.0399952
ENSBTAG00000006534	0.0862427	0.8172736	0.0876326
ENSBTAG00000006535	NA	NA	NA
ENSBTAG00000006536	NA	NA	NA
ENSBTAG00000006538	NA	NA	NA
ENSBTAG00000006539	NA	NA	NA
ENSBTAG00000006541	0.0107846	0.9669836	0.0145809
ENSBTAG00000006542	0.2701087	0.3977928	0.400343
ENSBTAG00000006543	-0.028398	0.9091843	0.0413481
ENSBTAG00000006546	NA	NA	NA
ENSBTAG00000006547	-0.110583	0.7028159	0.1531584
ENSBTAG00000006548	0.0104282	0.9765541	0.0103037
ENSBTAG00000006549	NA	NA	NA
ENSBTAG00000006550	-0.164634	0.5140296	0.2890119
ENSBTAG00000006551	NA	NA	NA
ENSBTAG00000006552	NA	NA	NA
ENSBTAG00000006555	-0.126828	0.6121746	0.2131247
ENSBTAG00000006556	-0.094679	0.6990888	0.1554676
ENSBTAG00000006560	NA	NA	NA
ENSBTAG00000006561	-0.161429	0.5930212	0.2269298
ENSBTAG00000006563	1.469314	0.0005426	3.2654858
ENSBTAG00000006564	-0.144144	0.5706991	0.2435928
ENSBTAG00000006566	NA	NA	NA
ENSBTAG00000006567	0.076568	0.8905556	0.050339
ENSBTAG00000006568	-0.073498	0.8675586	0.0617012
ENSBTAG00000006569	NA	NA	NA
ENSBTAG00000006570	-0.254946	0.4430447	0.3535524
ENSBTAG00000006572	NA	NA	NA
ENSBTAG00000006573	-0.036468	0.9156291	0.0382804
ENSBTAG00000006574	-0.382148	0.3155914	0.5008749
ENSBTAG00000006578	NA	NA	NA
ENSBTAG00000006579	-0.631127	0.0676646	1.1696384
ENSBTAG00000006581	-0.257888	0.4252918	0.371313
ENSBTAG00000006582	NA	NA	NA
ENSBTAG00000006586	-0.074602	0.7706522	0.1131416
ENSBTAG00000006587	-0.241862	0.446806	0.349881
ENSBTAG00000006588	0.5088538	0.1891508	0.7231918
ENSBTAG00000006589	NA	NA	NA
ENSBTAG00000006590	0.1448351	0.7024674	0.1533738
ENSBTAG00000006591	NA	NA	NA
ENSBTAG00000006592	0.0893376	0.8653084	0.0628291
ENSBTAG00000006593	0.2924961	0.3729293	0.4283735
ENSBTAG00000006595	NA	NA	NA
ENSBTAG00000006601	0.0594184	0.8732317	0.0588705
ENSBTAG00000006605	NA	NA	NA
ENSBTAG00000006606	0.0126193	0.980937	0.0083589

ENSBTAG00000006607	-0.31055	0.2421033	0.6159993
ENSBTAG00000006608	0.3018504	0.5127764	0.2900719
ENSBTAG00000006609	0.2118862	0.6149363	0.2111699
ENSBTAG00000006610	-0.057549	0.9032014	0.0442154
ENSBTAG00000006611	-0.136872	0.613126	0.2124503
ENSBTAG00000006612	0.3076286	0.3295855	0.4820319
ENSBTAG00000006613	0.0292367	0.9234622	0.0345809
ENSBTAG00000006614	-0.009455	0.9714139	0.0125957
ENSBTAG00000006615	-0.030143	0.9345809	0.0293831
ENSBTAG00000006616	0.0966172	0.7801136	0.1078422
ENSBTAG00000006618	-0.632683	0.0097449	2.0112207
ENSBTAG00000006619	NA	NA	NA
ENSBTAG00000006620	NA	NA	NA
ENSBTAG00000006625	0.0957749	0.7412877	0.1300132
ENSBTAG00000006626	NA	NA	NA
ENSBTAG00000006630	NA	NA	NA
ENSBTAG00000006631	NA	NA	NA
ENSBTAG00000006633	0.4823342	0.1643752	0.7841637
ENSBTAG00000006634	NA	NA	NA
ENSBTAG00000006635	NA	NA	NA
ENSBTAG00000006638	0.1064866	0.7724698	0.1121185
ENSBTAG00000006639	0.3456633	0.2889498	0.5391776
ENSBTAG00000006640	-0.233467	0.4195094	0.3772584
ENSBTAG00000006642	0.1331319	0.6729013	0.1720486
ENSBTAG00000006643	NA	NA	NA
ENSBTAG00000006644	0.0740656	0.7799421	0.1079376
ENSBTAG00000006645	2.2751059	0.0121691	1.9147431
ENSBTAG00000006646	-0.144778	0.5642707	0.2485125
ENSBTAG00000006647	-0.335109	0.2854731	0.5444348
ENSBTAG00000006649	NA	NA	NA
ENSBTAG00000006650	0.715019	0.1112646	0.9536429
ENSBTAG00000006651	NA	NA	NA
ENSBTAG00000006654	0.3121695	0.2432059	0.614026
ENSBTAG00000006656	0.2053514	0.5590595	0.2525419
ENSBTAG00000006657	NA	NA	NA
ENSBTAG00000006659	NA	NA	NA
ENSBTAG00000006661	-0.155318	0.5622623	0.2500611
ENSBTAG00000006662	0.8299109	0.0554656	1.2559762
ENSBTAG00000006663	0.0381556	0.9104342	0.0407514
ENSBTAG00000006665	0.0198014	0.9451981	0.0244772
ENSBTAG00000006666	0.3089712	0.2255593	0.6467393
ENSBTAG00000006667	-0.1452	0.5609753	0.2510563
ENSBTAG00000006670	0.2326772	0.4381196	0.3584073
ENSBTAG00000006671	0.0032304	0.9923511	0.0033346
ENSBTAG00000006672	0.1703617	0.5639012	0.248797
ENSBTAG00000006673	NA	NA	NA
ENSBTAG00000006675	-0.10353	0.7802961	0.1077405
ENSBTAG00000006676	-0.232225	0.5024948	0.2988684
ENSBTAG00000006678	-0.079017	0.8213682	0.0854621
ENSBTAG00000006679	-0.696649	0.1082875	0.9654217
ENSBTAG00000006680	0.0322992	0.9105184	0.0407113
ENSBTAG00000006683	NA	NA	NA
ENSBTAG00000006685	NA	NA	NA
ENSBTAG00000006686	-0.204524	0.5672461	0.2462285
ENSBTAG00000006688	0.1375906	0.76944	0.1138253
ENSBTAG00000006689	NA	NA	NA
ENSBTAG00000006690	-0.00126	0.9961963	0.0016551
ENSBTAG00000006691	NA	NA	NA

ENSBTAG00000006692	NA	NA	NA
ENSBTAG00000006693	-0.025618	0.9245917	0.03405
ENSBTAG00000006694	-0.330099	0.4084598	0.3888507
ENSBTAG00000006695	0.2953378	0.3205453	0.4941106
ENSBTAG00000006697	0.2488669	0.3872508	0.4120076
ENSBTAG00000006702	-0.037171	0.8827772	0.0541489
ENSBTAG00000006703	0.7840288	0.028412	1.5464979
ENSBTAG00000006704	NA	NA	NA
ENSBTAG00000006707	0.5014685	0.1328488	0.8766423
ENSBTAG00000006708	0.358127	0.2448661	0.6110713
ENSBTAG00000006712	-0.178334	0.4804596	0.3183432
ENSBTAG00000006713	-0.020086	0.9597503	0.0178417
ENSBTAG00000006714	0.2290118	0.4120692	0.3850298
ENSBTAG00000006715	0.250093	0.4529775	0.3439234
ENSBTAG00000006716	-0.219847	0.547367	0.2617214
ENSBTAG00000006718	-0.202651	0.4865174	0.3129016
ENSBTAG00000006719	0.6265837	0.0670384	1.1736762
ENSBTAG00000006721	-0.167347	0.5620061	0.250259
ENSBTAG00000006722	NA	NA	NA
ENSBTAG00000006724	-0.398113	0.1043145	0.9816555
ENSBTAG00000006726	NA	NA	NA
ENSBTAG00000006729	0.0020304	0.9952571	0.0020647
ENSBTAG00000006730	NA	NA	NA
ENSBTAG00000006731	NA	NA	NA
ENSBTAG00000006732	NA	NA	NA
ENSBTAG00000006733	-0.501784	0.0882819	1.0541286
ENSBTAG00000006734	0.4781343	0.2604493	0.5842769
ENSBTAG00000006735	-0.294555	0.5083894	0.2938035
ENSBTAG00000006738	NA	NA	NA
ENSBTAG00000006740	-0.077066	0.7714406	0.1126975
ENSBTAG00000006742	NA	NA	NA
ENSBTAG00000006743	-0.444015	0.1918313	0.7170805
ENSBTAG00000006744	-0.141092	0.6079874	0.2161054
ENSBTAG00000006745	-0.854279	0.2308546	0.6366615
ENSBTAG00000006747	0.3288292	0.3471588	0.4594718
ENSBTAG00000006748	0.0516244	0.8370979	0.0772237
ENSBTAG00000006751	0.1564618	0.5476243	0.2615173
ENSBTAG00000006752	0.5535698	0.0621571	1.2065091
ENSBTAG00000006754	0.2856065	0.4636761	0.3337853
ENSBTAG00000006755	-0.090911	0.7275706	0.1381249
ENSBTAG00000006756	-0.298684	0.2369601	0.6253247
ENSBTAG00000006757	-0.21778	0.6308638	0.2000644
ENSBTAG00000006759	-0.26337	0.3506503	0.4551257
ENSBTAG00000006762	-1.76857	1.54E-06	5.8131905
ENSBTAG00000006765	-0.19165	0.4574227	0.3396823
ENSBTAG00000006767	-0.171789	0.5116302	0.2910439
ENSBTAG00000006768	-0.30304	0.3384052	0.4705629
ENSBTAG00000006770	-0.551692	0.1988835	0.7014013
ENSBTAG00000006771	-0.051399	0.8429949	0.074175
ENSBTAG00000006775	0.0721768	0.813198	0.0898037
ENSBTAG00000006776	0.1189067	0.7343608	0.1340905
ENSBTAG00000006777	NA	NA	NA
ENSBTAG00000006779	-0.25875	0.4805631	0.3182496
ENSBTAG00000006780	NA	NA	NA
ENSBTAG00000006784	0.386869	0.4332167	0.3632948
ENSBTAG00000006785	-0.560059	0.0807265	1.0929837
ENSBTAG00000006786	-0.025573	0.9256017	0.0335759
ENSBTAG00000006789	-0.234582	0.4290481	0.367494

ENSBTAG00000006790	-0.092868	0.871679	0.0596434
ENSBTAG00000006792	-0.088107	0.8063249	0.0934899
ENSBTAG00000006795	-0.331716	0.2164862	0.6645697
ENSBTAG00000006797	-1.112624	0.0159052	1.7984606
ENSBTAG00000006800	1.7654861	0.0006043	3.2187244
ENSBTAG00000006801	0.3550299	0.5398574	0.267721
ENSBTAG00000006804	NA	NA	NA
ENSBTAG00000006805	0.4469223	0.2395959	0.6205207
ENSBTAG00000006806	NA	NA	NA
ENSBTAG00000006811	-0.498067	0.2014088	0.6959215
ENSBTAG00000006812	1.0213708	0.0533952	1.2724978
ENSBTAG00000006816	0.385598	0.1791252	0.7468432
ENSBTAG00000006817	-0.093779	0.7678713	0.1147116
ENSBTAG00000006818	0.0484747	0.8916777	0.0497921
ENSBTAG00000006819	-0.44617	0.0980512	1.008547
ENSBTAG00000006820	NA	NA	NA
ENSBTAG00000006821	0.0707966	0.8137425	0.089513
ENSBTAG00000006823	-0.310239	0.2533296	0.596314
ENSBTAG00000006824	-0.178083	0.5301296	0.275618
ENSBTAG00000006828	0.0871514	0.8025418	0.0955323
ENSBTAG00000006831	-0.1786	0.6703223	0.1737163
ENSBTAG00000006832	-0.24544	0.6156085	0.2106954
ENSBTAG00000006833	0.1887863	0.4740518	0.3241742
ENSBTAG00000006835	0.2099248	0.5303495	0.2754378
ENSBTAG00000006836	-0.246438	0.4285805	0.3679676
ENSBTAG00000006837	-0.357849	0.1606896	0.7940122
ENSBTAG00000006838	0.0874917	0.7452675	0.1276878
ENSBTAG00000006839	-0.074943	0.812378	0.0902419
ENSBTAG00000006843	0.1149064	0.6620058	0.1791382
ENSBTAG00000006844	NA	NA	NA
ENSBTAG00000006846	0.3816521	0.2615757	0.5824026
ENSBTAG00000006848	0.5232768	0.2221275	0.6533977
ENSBTAG00000006851	-0.419785	0.3947783	0.4036468
ENSBTAG00000006852	0.0515332	0.8501924	0.0704828
ENSBTAG00000006853	NA	NA	NA
ENSBTAG00000006855	NA	NA	NA
ENSBTAG00000006859	NA	NA	NA
ENSBTAG00000006860	NA	NA	NA
ENSBTAG00000006861	NA	NA	NA
ENSBTAG00000006862	NA	NA	NA
ENSBTAG00000006864	0.1356741	0.7037391	0.1525883
ENSBTAG00000006868	NA	NA	NA
ENSBTAG00000006869	-0.264454	0.3061524	0.5140624
ENSBTAG00000006870	NA	NA	NA
ENSBTAG00000006876	0.1267351	0.7270488	0.1384364
ENSBTAG00000006877	-0.049662	0.9125431	0.0397466
ENSBTAG00000006878	-0.068552	0.7865186	0.104291
ENSBTAG00000006879	NA	NA	NA
ENSBTAG00000006881	-0.219444	0.4054281	0.3920862
ENSBTAG00000006882	NA	NA	NA
ENSBTAG00000006883	-0.014486	0.9545391	0.0202063
ENSBTAG00000006884	0.2051916	0.4633117	0.3341267
ENSBTAG00000006886	-0.005861	0.9947753	0.002275
ENSBTAG00000006893	0.2605656	0.3568133	0.4475589
ENSBTAG00000006894	NA	NA	NA
ENSBTAG00000006896	0.5512788	0.0463595	1.3338613
ENSBTAG00000006898	0.0320773	0.897368	0.0470294
ENSBTAG00000006899	NA	NA	NA

ENSBTAG00000006901	0.7876398	0.0571825	1.242737
ENSBTAG00000006903	NA	NA	NA
ENSBTAG00000006904	0.1873977	0.5838291	0.2337143
ENSBTAG00000006905	0.4582684	0.2276529	0.6427269
ENSBTAG00000006907	-0.342483	0.2265996	0.6447408
ENSBTAG00000006909	0.1069818	0.7152594	0.1455364
ENSBTAG00000006910	0.1884328	0.5315909	0.2744225
ENSBTAG00000006911	-0.018243	0.9427068	0.0256234
ENSBTAG00000006912	NA	NA	NA
ENSBTAG00000006914	NA	NA	NA
ENSBTAG00000006916	0.005793	0.98569	0.0062597
ENSBTAG00000006918	-0.161967	0.5500883	0.2595676
ENSBTAG00000006919	-0.017535	0.9439133	0.0250679
ENSBTAG00000006920	0.2592657	0.305383	0.5151552
ENSBTAG00000006921	0.6634463	0.0164778	1.7831012
ENSBTAG00000006925	0.4504231	0.2643606	0.5778033
ENSBTAG00000006927	-0.204458	0.4190435	0.3777409
ENSBTAG00000006928	-0.307605	0.2281101	0.6418556
ENSBTAG00000006933	0.1347903	0.6864938	0.1633634
ENSBTAG00000006934	NA	NA	NA
ENSBTAG00000006936	0.2862125	0.2861103	0.5434666
ENSBTAG00000006937	0.1844013	0.6035101	0.2193155
ENSBTAG00000006938	0.0468727	0.8937456	0.0487861
ENSBTAG00000006939	-0.221279	0.6799072	0.1675504
ENSBTAG00000006940	0.0041547	0.9910579	0.003901
ENSBTAG00000006941	NA	NA	NA
ENSBTAG00000006945	0.3765561	0.1964984	0.706641
ENSBTAG00000006947	NA	NA	NA
ENSBTAG00000006948	NA	NA	NA
ENSBTAG00000006949	NA	NA	NA
ENSBTAG00000006950	-0.391611	0.1157371	0.9365275
ENSBTAG00000006951	0.3677969	0.2228006	0.6520837
ENSBTAG00000006954	0.082561	0.7975755	0.0982282
ENSBTAG00000006955	-0.275128	0.3042105	0.5168258
ENSBTAG00000006957	0.01394	0.9805252	0.0085413
ENSBTAG00000006958	0.3802077	0.4239779	0.3726567
ENSBTAG00000006960	0.0731045	0.803354	0.0950931
ENSBTAG00000006961	NA	NA	NA
ENSBTAG00000006962	-0.151157	0.545762	0.2629967
ENSBTAG00000006963	0.4004915	0.1743783	0.7585075
ENSBTAG00000006966	NA	NA	NA
ENSBTAG00000006969	-0.112936	0.6689261	0.1746219
ENSBTAG00000006970	-0.147115	0.5892726	0.2296838
ENSBTAG00000006971	0.0589316	0.8283242	0.0817997
ENSBTAG00000006972	NA	NA	NA
ENSBTAG00000006973	NA	NA	NA
ENSBTAG00000006974	NA	NA	NA
ENSBTAG00000006975	NA	NA	NA
ENSBTAG00000006977	-0.088882	0.87903	0.0559963
ENSBTAG00000006978	0.0330077	0.8957139	0.0478307
ENSBTAG00000006982	-0.305823	0.3463923	0.4604318
ENSBTAG00000006984	0.6880356	0.1156363	0.9369057
ENSBTAG00000006985	-0.130239	0.6302282	0.2005022
ENSBTAG00000006986	NA	NA	NA
ENSBTAG00000006987	NA	NA	NA
ENSBTAG00000006988	NA	NA	NA
ENSBTAG00000006989	NA	NA	NA
ENSBTAG00000006990	NA	NA	NA

ENSBTAG00000006991	NA	NA	NA
ENSBTAG00000006995	0.5224046	0.0497202	1.3034675
ENSBTAG00000006998	NA	NA	NA
ENSBTAG00000006999	0.1166377	0.6764718	0.1697503
ENSBTAG00000007000	0.0635322	0.8619868	0.0644994
ENSBTAG00000007001	NA	NA	NA
ENSBTAG00000007002	0.4024945	0.3313801	0.4796736
ENSBTAG00000007003	0.3169679	0.3690186	0.4329518
ENSBTAG00000007007	0.0626566	0.8364587	0.0775555
ENSBTAG00000007008	NA	NA	NA
ENSBTAG00000007012	0.444522	0.207247	0.6835119
ENSBTAG00000007013	-0.147644	0.7204378	0.1424035
ENSBTAG00000007014	-0.210657	0.5443043	0.2641582
ENSBTAG00000007015	-0.199563	0.4721127	0.3259543
ENSBTAG00000007016	0.213735	0.5104224	0.2920703
ENSBTAG00000007019	NA	NA	NA
ENSBTAG00000007020	0.1151733	0.8008312	0.096459
ENSBTAG00000007031	0.3152698	0.3044414	0.5164963
ENSBTAG00000007036	NA	NA	NA
ENSBTAG00000007039	-0.095381	0.7555561	0.1217333
ENSBTAG00000007041	NA	NA	NA
ENSBTAG00000007043	NA	NA	NA
ENSBTAG00000007047	NA	NA	NA
ENSBTAG00000007049	NA	NA	NA
ENSBTAG00000007052	NA	NA	NA
ENSBTAG00000007053	NA	NA	NA
ENSBTAG00000007058	0.464702	0.2370932	0.6250808
ENSBTAG00000007061	0.1413273	0.6548412	0.183864
ENSBTAG00000007062	-0.971036	0.0033268	2.4779764
ENSBTAG00000007065	-0.393429	0.3826001	0.4172549
ENSBTAG00000007066	0.3068397	0.3787096	0.4216937
ENSBTAG00000007067	-0.455949	0.0820809	1.0857579
ENSBTAG00000007068	-0.466806	0.0669255	1.1744081
ENSBTAG00000007070	NA	NA	NA
ENSBTAG00000007071	-0.348011	0.1690869	0.7718902
ENSBTAG00000007074	0.2154755	0.4981361	0.302652
ENSBTAG00000007075	NA	NA	NA
ENSBTAG00000007077	NA	NA	NA
ENSBTAG00000007079	0.1362062	0.6978909	0.1562125
ENSBTAG00000007080	0.1487689	0.5557006	0.2551591
ENSBTAG00000007084	0.9597639	0.0121805	1.9143361
ENSBTAG00000007089	0.3238397	0.3587158	0.4452495
ENSBTAG00000007090	-0.074405	0.831277	0.0802542
ENSBTAG00000007093	-0.667844	0.1696104	0.7705476
ENSBTAG00000007094	NA	NA	NA
ENSBTAG00000007096	NA	NA	NA
ENSBTAG00000007097	0.1502145	0.7396867	0.1309522
ENSBTAG00000007099	0.1972773	0.6655074	0.1768471
ENSBTAG00000007100	-0.164628	0.5347534	0.2718464
ENSBTAG00000007101	-0.29352	0.4650686	0.332483
ENSBTAG00000007102	NA	NA	NA
ENSBTAG00000007103	-0.144261	0.7793524	0.1082661
ENSBTAG00000007104	NA	NA	NA
ENSBTAG00000007105	0.5050843	0.1983131	0.7026486
ENSBTAG00000007106	0.1225858	0.6156166	0.2106897
ENSBTAG00000007107	0.4951057	0.1049573	0.9789873
ENSBTAG00000007108	NA	NA	NA
ENSBTAG00000007109	-0.09474	0.7229301	0.1409037

ENSBTAG00000007110	0.0865131	0.7636982	0.1170782
ENSBTAG00000007111	-0.190887	0.5308265	0.2750474
ENSBTAG00000007112	0.1358702	0.7163876	0.1448519
ENSBTAG00000007113	0.1549803	0.5530277	0.2572531
ENSBTAG00000007114	0.0877928	0.8117893	0.0905567
ENSBTAG00000007115	0.2560498	0.5288553	0.2766632
ENSBTAG00000007116	-0.404939	0.2594208	0.5859951
ENSBTAG00000007117	0.277519	0.4201112	0.3766357
ENSBTAG00000007118	0.0935091	0.8263628	0.0828292
ENSBTAG00000007120	0.1649087	0.507366	0.2946786
ENSBTAG00000007121	-0.213209	0.5754554	0.2399883
ENSBTAG00000007122	0.2601875	0.4321736	0.3643418
ENSBTAG00000007123	0.135118	0.5893382	0.2296354
ENSBTAG00000007125	NA	NA	NA
ENSBTAG00000007128	0.2116056	0.5021113	0.2992
ENSBTAG00000007129	0.5696995	0.1699179	0.7697609
ENSBTAG00000007130	-0.138883	0.6313521	0.1997284
ENSBTAG00000007131	-1.116089	1.81E-05	4.7422111
ENSBTAG00000007133	-0.152046	0.533744	0.272667
ENSBTAG00000007136	-0.122198	0.709174	0.1492472
ENSBTAG00000007137	0.0884775	0.8156981	0.0884706
ENSBTAG00000007139	-0.24607	0.3335424	0.4768489
ENSBTAG00000007141	0.1961919	0.6310493	0.1999367
ENSBTAG00000007142	0.0993752	0.8229599	0.0846213
ENSBTAG00000007146	NA	NA	NA
ENSBTAG00000007147	-0.707016	0.0082634	2.0828439
ENSBTAG00000007148	NA	NA	NA
ENSBTAG00000007149	0.6771639	0.1254481	0.9015358
ENSBTAG00000007152	0.1072474	0.6712355	0.1731251
ENSBTAG00000007153	0.0148347	0.9676075	0.0143008
ENSBTAG00000007156	0.0320528	0.9533813	0.0207334
ENSBTAG00000007158	0.0361262	0.8890868	0.0510558
ENSBTAG00000007159	-0.361371	0.2141366	0.6693091
ENSBTAG00000007160	0.2983596	0.3832371	0.4165324
ENSBTAG00000007163	-0.142007	0.6457846	0.1899123
ENSBTAG00000007164	-1.544108	0.0115569	1.937159
ENSBTAG00000007166	NA	NA	NA
ENSBTAG00000007167	-0.160829	0.5644106	0.2484048
ENSBTAG00000007169	NA	NA	NA
ENSBTAG00000007170	1.4985502	7.75E-05	4.1109477
ENSBTAG00000007172	-0.050438	0.844491	0.073405
ENSBTAG00000007173	0.3899299	0.1213442	0.9159808
ENSBTAG00000007175	NA	NA	NA
ENSBTAG00000007176	0.216873	0.6240596	0.204774
ENSBTAG00000007177	0.2693198	0.5905349	0.2287545
ENSBTAG00000007181	-0.134919	0.7293756	0.1370487
ENSBTAG00000007184	0.5073796	0.1624438	0.789297
ENSBTAG00000007186	NA	NA	NA
ENSBTAG00000007187	0.6967168	0.0720062	1.1426303
ENSBTAG00000007189	-0.145144	0.5597931	0.2519724
ENSBTAG00000007190	-0.282852	0.4040558	0.3935587
ENSBTAG00000007191	NA	NA	NA
ENSBTAG00000007192	-0.189582	0.5857442	0.232292
ENSBTAG00000007193	-0.050492	0.919526	0.036436
ENSBTAG00000007195	NA	NA	NA
ENSBTAG00000007196	0.4853722	0.0587532	1.2309686
ENSBTAG00000007200	NA	NA	NA
ENSBTAG00000007201	0.2956513	0.3284161	0.4835756

ENSBTAG00000007202	0.1504976	0.6057882	0.2176792
ENSBTAG00000007203	0.0929687	0.7059961	0.1511977
ENSBTAG00000007204	NA	NA	NA
ENSBTAG00000007206	0.0026949	0.9921945	0.0034032
ENSBTAG00000007208	0.1302078	0.6402336	0.1936615
ENSBTAG00000007210	-0.56107	0.0211563	1.6745606
ENSBTAG00000007211	-0.024568	0.9343535	0.0294888
ENSBTAG00000007213	0.1086832	0.7245534	0.1399296
ENSBTAG00000007214	-1.370195	0.0002392	3.621286
ENSBTAG00000007215	0.1261399	0.6789917	0.1681355
ENSBTAG00000007216	NA	NA	NA
ENSBTAG00000007217	0.3941644	0.1522359	0.8174828
ENSBTAG00000007220	-0.003649	0.9942876	0.002488
ENSBTAG00000007223	NA	NA	NA
ENSBTAG00000007228	-0.130941	0.651495	0.1860889
ENSBTAG00000007230	NA	NA	NA
ENSBTAG00000007231	NA	NA	NA
ENSBTAG00000007233	0.2087772	0.6435573	0.1914128
ENSBTAG00000007234	-0.152091	0.5377196	0.2694441
ENSBTAG00000007235	NA	NA	NA
ENSBTAG00000007236	-0.285681	0.2518503	0.5988576
ENSBTAG00000007237	NA	NA	NA
ENSBTAG00000007238	NA	NA	NA
ENSBTAG00000007239	-0.61391	0.1421114	0.8473711
ENSBTAG00000007240	0.2913115	0.3842407	0.4153967
ENSBTAG00000007241	NA	NA	NA
ENSBTAG00000007244	0.4107033	0.1096187	0.9601153
ENSBTAG00000007245	0.3288158	0.4738979	0.3243152
ENSBTAG00000007246	-0.13125	0.6921835	0.1597787
ENSBTAG00000007247	NA	NA	NA
ENSBTAG00000007253	NA	NA	NA
ENSBTAG00000007256	-0.614323	0.0305381	1.5151584
ENSBTAG00000007258	NA	NA	NA
ENSBTAG00000007259	NA	NA	NA
ENSBTAG00000007262	NA	NA	NA
ENSBTAG00000007266	-0.10405	0.7693757	0.1138615
ENSBTAG00000007268	0.4013246	0.2635779	0.579091
ENSBTAG00000007269	0.2040412	0.5166549	0.2867995
ENSBTAG00000007270	0.3272659	0.3630643	0.4400164
ENSBTAG00000007271	0.4525167	0.2962788	0.5282995
ENSBTAG00000007272	0.0816101	0.7664404	0.1155216
ENSBTAG00000007273	NA	NA	NA
ENSBTAG00000007275	NA	NA	NA
ENSBTAG00000007280	NA	NA	NA
ENSBTAG00000007281	-0.154976	0.5670046	0.2464135
ENSBTAG00000007284	NA	NA	NA
ENSBTAG00000007288	0.3532387	0.2288652	0.6404203
ENSBTAG00000007296	NA	NA	NA
ENSBTAG00000007298	NA	NA	NA
ENSBTAG00000007299	0.3439556	0.2629116	0.5801902
ENSBTAG00000007300	0.3776048	0.1771063	0.7517661
ENSBTAG00000007302	NA	NA	NA
ENSBTAG00000007303	-0.084085	0.7377586	0.1320857
ENSBTAG00000007304	0.1427281	0.6488901	0.1878288
ENSBTAG00000007305	-0.15584	0.5331528	0.2731483
ENSBTAG00000007306	NA	NA	NA
ENSBTAG00000007307	NA	NA	NA
ENSBTAG00000007308	0.2638935	0.3534964	0.451615

ENSBTAG00000007309	NA	NA	NA
ENSBTAG00000007312	NA	NA	NA
ENSBTAG00000007315	0.1188479	0.6612809	0.179614
ENSBTAG00000007316	NA	NA	NA
ENSBTAG00000007318	0.5263341	0.1305004	0.8843882
ENSBTAG00000007319	0.2469057	0.4172568	0.3795966
ENSBTAG00000007320	0.3216653	0.214579	0.6684129
ENSBTAG00000007321	-0.307838	0.3064684	0.5136142
ENSBTAG00000007323	-0.024557	0.9215517	0.0354803
ENSBTAG00000007324	NA	NA	NA
ENSBTAG00000007325	NA	NA	NA
ENSBTAG00000007329	0.28768	0.3514156	0.4541789
ENSBTAG00000007330	-0.31325	0.2312006	0.6360111
ENSBTAG00000007331	0.4949646	0.149551	0.8252108
ENSBTAG00000007332	0.0460078	0.864882	0.0630432
ENSBTAG00000007333	NA	NA	NA
ENSBTAG00000007334	NA	NA	NA
ENSBTAG00000007335	0.2721946	0.4571877	0.3399055
ENSBTAG00000007336	NA	NA	NA
ENSBTAG00000007338	-0.124452	0.8063695	0.0934659
ENSBTAG00000007343	-0.793604	0.0044914	2.3476186
ENSBTAG00000007346	-0.103984	0.7125815	0.1471655
ENSBTAG00000007348	NA	NA	NA
ENSBTAG00000007349	NA	NA	NA
ENSBTAG00000007350	NA	NA	NA
ENSBTAG00000007352	-0.355283	0.2990537	0.5242508
ENSBTAG00000007356	-0.700277	0.0298123	1.5256045
ENSBTAG00000007357	-0.085515	0.8006347	0.0965656
ENSBTAG00000007360	0.4724531	0.0742366	1.1293819
ENSBTAG00000007361	-0.270515	0.312295	0.505435
ENSBTAG00000007362	-0.107318	0.7290715	0.1372299
ENSBTAG00000007363	-0.288669	0.3038902	0.5172832
ENSBTAG00000007364	2.5441776	7.56E-06	5.1213174
ENSBTAG00000007365	0.2780827	0.5542328	0.2563078
ENSBTAG00000007366	-0.051096	0.9105679	0.0406877
ENSBTAG00000007367	0.1116282	0.6745011	0.1710173
ENSBTAG00000007368	0.3266638	0.3444914	0.4628216
ENSBTAG00000007369	-0.180806	0.7300532	0.1366455
ENSBTAG00000007370	0.1739836	0.5801069	0.236492
ENSBTAG00000007371	-0.321428	0.255615	0.5924136
ENSBTAG00000007372	NA	NA	NA
ENSBTAG00000007374	-0.2742	0.4596054	0.3376149
ENSBTAG00000007375	0.3594245	0.2322793	0.6339895
ENSBTAG00000007378	-0.442897	0.1083994	0.9649731
ENSBTAG00000007379	NA	NA	NA
ENSBTAG00000007382	-0.107482	0.6938879	0.1587107
ENSBTAG00000007383	0.3930953	0.2993915	0.5237606
ENSBTAG00000007384	0.360607	0.1864313	0.7294812
ENSBTAG00000007385	-0.046641	0.8558535	0.0676006
ENSBTAG00000007386	-0.141008	0.6280095	0.2020338
ENSBTAG00000007387	-0.292082	0.2632594	0.5796162
ENSBTAG00000007388	NA	NA	NA
ENSBTAG00000007389	0.3136769	0.3162605	0.4999551
ENSBTAG00000007390	-0.363824	0.1777279	0.7502445
ENSBTAG00000007393	0.1973309	0.6810653	0.1668113
ENSBTAG00000007394	0.014194	0.963506	0.0161456
ENSBTAG00000007395	0.1195896	0.6535003	0.1847542
ENSBTAG00000007397	-0.025073	0.9424362	0.0257481

ENSBTAG00000007398	0.0425916	0.8960318	0.0476766
ENSBTAG00000007399	-0.011342	0.9649536	0.0154936
ENSBTAG00000007402	-0.312481	0.4932041	0.3069733
ENSBTAG00000007409	NA	NA	NA
ENSBTAG00000007411	NA	NA	NA
ENSBTAG00000007413	NA	NA	NA
ENSBTAG00000007414	-0.215844	0.4608632	0.336428
ENSBTAG00000007415	1.7035578	1.20E-08	7.9201895
ENSBTAG00000007417	-0.102637	0.7976836	0.0981693
ENSBTAG00000007421	0.2812915	0.2871805	0.541845
ENSBTAG00000007422	-0.030488	0.9104636	0.0407374
ENSBTAG00000007423	NA	NA	NA
ENSBTAG00000007424	NA	NA	NA
ENSBTAG00000007427	0.374351	0.3367783	0.4726559
ENSBTAG00000007428	-1.625959	0.0013436	2.87173
ENSBTAG00000007429	-0.004537	1	0
ENSBTAG00000007430	0.235859	0.4821856	0.3167858
ENSBTAG00000007431	NA	NA	NA
ENSBTAG00000007433	-0.163527	0.5114774	0.2911736
ENSBTAG00000007434	0.5822264	0.0570953	1.2433998
ENSBTAG00000007435	0.4119494	0.1669028	0.7775365
ENSBTAG00000007436	NA	NA	NA
ENSBTAG00000007438	-0.226436	0.3809513	0.4191305
ENSBTAG00000007439	-0.311923	0.5651272	0.2478538
ENSBTAG00000007440	NA	NA	NA
ENSBTAG00000007441	-0.009714	0.9703301	0.0130805
ENSBTAG00000007442	-0.539565	0.0386522	1.4128256
ENSBTAG00000007444	-1.011778	0.0052329	2.2812557
ENSBTAG00000007445	NA	NA	NA
ENSBTAG00000007446	NA	NA	NA
ENSBTAG00000007447	0.5425545	0.0828996	1.0814477
ENSBTAG00000007449	-0.130436	0.8321594	0.0797935
ENSBTAG00000007450	0.0050673	1	0
ENSBTAG00000007453	0.1777517	0.5822291	0.2349061
ENSBTAG00000007454	0.3218438	0.2184746	0.6605991
ENSBTAG00000007455	-0.121637	0.6343457	0.197674
ENSBTAG00000007456	0.0726834	0.8450387	0.0731234
ENSBTAG00000007460	-0.042205	0.8767942	0.0571023
ENSBTAG00000007461	0.0414329	0.9181351	0.0370934
ENSBTAG00000007462	0.4184839	0.2893283	0.538609
ENSBTAG00000007467	0.5974086	0.0252533	1.5976821
ENSBTAG00000007470	NA	NA	NA
ENSBTAG00000007473	NA	NA	NA
ENSBTAG00000007474	0.0508448	0.8469108	0.0721623
ENSBTAG00000007476	-0.091812	0.7630752	0.1174326
ENSBTAG00000007477	NA	NA	NA
ENSBTAG00000007479	-0.102389	0.7101889	0.1486261
ENSBTAG00000007480	-0.158406	0.6063756	0.2172583
ENSBTAG00000007483	0.3767924	0.2290242	0.6401187
ENSBTAG00000007484	0.1631458	0.5423459	0.2657236
ENSBTAG00000007485	NA	NA	NA
ENSBTAG00000007488	-0.138449	0.6615265	0.1794528
ENSBTAG00000007489	-0.321177	0.1992453	0.7006118
ENSBTAG00000007490	0.2592584	0.503858	0.2976918
ENSBTAG00000007492	-0.153714	0.7245798	0.1399138
ENSBTAG00000007493	NA	NA	NA
ENSBTAG00000007494	0.0168515	0.9487028	0.0228698
ENSBTAG00000007497	-0.013613	0.959906	0.0177713

ENSBTAG00000007498	0.044857	0.877282	0.0568608
ENSBTAG00000007499	0.0141831	0.9561813	0.0194598
ENSBTAG00000007501	NA	NA	NA
ENSBTAG00000007502	NA	NA	NA
ENSBTAG00000007506	NA	NA	NA
ENSBTAG00000007507	0.8522481	0.0553742	1.2566923
ENSBTAG00000007508	0.3956926	0.1715114	0.765707
ENSBTAG00000007509	NA	NA	NA
ENSBTAG00000007510	-0.759518	0.0073635	2.1329166
ENSBTAG00000007512	-0.279501	0.5223504	0.2820381
ENSBTAG00000007513	-0.00957	0.9715284	0.0125445
ENSBTAG00000007514	NA	NA	NA
ENSBTAG00000007515	0.0908236	0.7235872	0.1405091
ENSBTAG00000007519	0.3017702	0.3193996	0.4956657
ENSBTAG00000007520	-0.922607	0.0152696	1.816172
ENSBTAG00000007522	-0.288325	0.4702857	0.3276383
ENSBTAG00000007523	-0.046057	0.88063	0.0552065
ENSBTAG00000007530	NA	NA	NA
ENSBTAG00000007531	NA	NA	NA
ENSBTAG00000007532	NA	NA	NA
ENSBTAG00000007534	-0.001143	1	0
ENSBTAG00000007537	-0.165867	0.4974112	0.3032844
ENSBTAG00000007540	0.005308	0.9835277	0.0072134
ENSBTAG00000007543	-0.201882	0.5726075	0.2421429
ENSBTAG00000007545	-0.109097	0.7565653	0.1211536
ENSBTAG00000007546	0.1434168	0.6908386	0.1606234
ENSBTAG00000007547	-0.454246	0.0993335	1.0029043
ENSBTAG00000007550	NA	NA	NA
ENSBTAG00000007553	-0.027621	0.9475885	0.0233802
ENSBTAG00000007554	0.1942453	0.7028363	0.1531458
ENSBTAG00000007558	NA	NA	NA
ENSBTAG00000007559	-0.284963	0.2816906	0.5502276
ENSBTAG00000007562	0.1037685	0.7543765	0.1224118
ENSBTAG00000007564	-0.346126	0.343342	0.4642731
ENSBTAG00000007566	0.0533585	0.8375198	0.0770049
ENSBTAG00000007567	-0.032954	0.9003939	0.0455675
ENSBTAG00000007569	NA	NA	NA
ENSBTAG00000007570	0.2081156	0.4235676	0.3730772
ENSBTAG00000007572	-0.188062	0.4671667	0.3305281
ENSBTAG00000007577	0.0208679	0.9423412	0.0257918
ENSBTAG00000007578	0.3654174	0.4310218	0.3655008
ENSBTAG00000007580	NA	NA	NA
ENSBTAG00000007581	0.1554679	0.7662222	0.1156453
ENSBTAG00000007584	0.1731867	0.7276807	0.1380592
ENSBTAG00000007586	-0.005054	0.9839864	0.0070109
ENSBTAG00000007588	0.0597435	0.8673958	0.0617827
ENSBTAG00000007589	NA	NA	NA
ENSBTAG00000007591	-0.05526	0.8263996	0.0828099
ENSBTAG00000007592	0.0547251	0.8850279	0.0530431
ENSBTAG00000007593	0.0087529	0.9739515	0.0114627
ENSBTAG00000007594	-0.101956	0.759125	0.1196867
ENSBTAG00000007595	-0.125105	0.6168584	0.2098145
ENSBTAG00000007596	-0.291192	0.3926419	0.4060033
ENSBTAG00000007599	0.0643796	0.8462116	0.072521
ENSBTAG00000007602	0.2513292	0.561269	0.2508289
ENSBTAG00000007605	0.0771985	0.7546722	0.1222417
ENSBTAG00000007606	-0.024354	0.9217135	0.0354041
ENSBTAG00000007608	-0.448804	0.1810375	0.7422314

ENSBTAG00000007609	0.1592159	0.6489683	0.1877765
ENSBTAG00000007611	0.5114984	0.2715006	0.5662291
ENSBTAG00000007614	-0.479461	0.0580717	1.2360358
ENSBTAG00000007615	0.4998922	0.2759943	0.5590999
ENSBTAG00000007616	NA	NA	NA
ENSBTAG00000007617	0.2891195	0.4309502	0.3655729
ENSBTAG00000007619	NA	NA	NA
ENSBTAG00000007620	-0.029317	0.9092237	0.0413293
ENSBTAG00000007622	0.5021093	0.0510867	1.2916923
ENSBTAG00000007623	-0.219038	0.5757214	0.2397876
ENSBTAG00000007624	-0.642819	0.1125448	0.9486744
ENSBTAG00000007626	0.0220764	0.9676574	0.0142784
ENSBTAG00000007629	0.4766444	0.1922683	0.7160924
ENSBTAG00000007630	-0.176991	0.578769	0.2374947
ENSBTAG00000007632	-0.360804	0.285757	0.5440031
ENSBTAG00000007633	NA	NA	NA
ENSBTAG00000007634	0.6336535	0.1025844	0.9889187
ENSBTAG00000007635	-0.722022	0.0051568	2.2876216
ENSBTAG00000007636	-0.077133	0.8025602	0.0955224
ENSBTAG00000007638	NA	NA	NA
ENSBTAG00000007639	-0.022544	0.9352462	0.029074
ENSBTAG00000007642	NA	NA	NA
ENSBTAG00000007643	0.1124813	0.7123734	0.1472923
ENSBTAG00000007644	0.2448648	0.3846462	0.4149385
ENSBTAG00000007646	0.5107863	0.2936742	0.5321341
ENSBTAG00000007648	0.1719287	0.5168554	0.2866309
ENSBTAG00000007649	0.0539902	0.8776222	0.0566924
ENSBTAG00000007650	NA	NA	NA
ENSBTAG00000007651	-0.596158	0.2725786	0.5645083
ENSBTAG00000007652	0.8216764	0.0180272	1.7440724
ENSBTAG00000007654	0.1515779	0.6885606	0.1620579
ENSBTAG00000007657	0.4900524	0.2576799	0.5889195
ENSBTAG00000007658	-0.078519	0.7960665	0.0990507
ENSBTAG00000007659	NA	NA	NA
ENSBTAG00000007660	NA	NA	NA
ENSBTAG00000007661	-0.331402	0.44061	0.3559456
ENSBTAG00000007662	0.2019059	0.4298902	0.3666425
ENSBTAG00000007665	0.6265793	0.0991199	1.0038391
ENSBTAG00000007666	0.5461383	0.423335	0.3733158
ENSBTAG00000007668	NA	NA	NA
ENSBTAG00000007675	0.2252589	0.5913004	0.2281919
ENSBTAG00000007678	NA	NA	NA
ENSBTAG00000007680	-0.098859	0.832551	0.0795891
ENSBTAG00000007681	-0.264841	0.4109201	0.3862426
ENSBTAG00000007683	-0.04793	0.8743931	0.0582933
ENSBTAG00000007684	-0.606927	0.01282	1.8921129
ENSBTAG00000007685	0.0455024	0.8605196	0.0652392
ENSBTAG00000007686	NA	NA	NA
ENSBTAG00000007687	NA	NA	NA
ENSBTAG00000007689	0.043481	0.8924036	0.0494387
ENSBTAG00000007693	NA	NA	NA
ENSBTAG00000007694	1.1202904	0.0782648	1.1064335
ENSBTAG00000007696	-0.106795	0.6726854	0.172188
ENSBTAG00000007698	NA	NA	NA
ENSBTAG00000007700	0.0943485	0.7138567	0.146389
ENSBTAG00000007702	0.5130094	0.1526417	0.8163269
ENSBTAG00000007703	0.3111058	0.387721	0.4114807
ENSBTAG00000007704	NA	NA	NA

ENSBTAG00000007705	-0.203114	0.4822238	0.3167514
ENSBTAG00000007708	NA	NA	NA
ENSBTAG00000007709	-0.056192	0.8250594	0.0835148
ENSBTAG00000007712	0.0677435	0.8134253	0.0896823
ENSBTAG00000007714	0.364897	0.3285987	0.4833341
ENSBTAG00000007718	1.0002494	0.0174671	1.7577801
ENSBTAG00000007719	0.1659333	0.7076813	0.1501623
ENSBTAG00000007720	NA	NA	NA
ENSBTAG00000007721	-0.354109	0.1667856	0.7778415
ENSBTAG00000007722	NA	NA	NA
ENSBTAG00000007723	0.0882227	0.8061511	0.0935836
ENSBTAG00000007724	NA	NA	NA
ENSBTAG00000007725	0.1098228	0.6669014	0.1759384
ENSBTAG00000007728	0.0815403	0.7940148	0.1001714
ENSBTAG00000007730	-0.138475	0.5861071	0.232023
ENSBTAG00000007731	-0.65573	0.0312711	1.5048565
ENSBTAG00000007732	-0.112954	0.704902	0.1518712
ENSBTAG00000007734	-0.221909	0.3774808	0.4231051
ENSBTAG00000007737	0.2347557	0.3882724	0.4108634
ENSBTAG00000007739	-0.262336	0.3721629	0.4292669
ENSBTAG00000007740	NA	NA	NA
ENSBTAG00000007741	NA	NA	NA
ENSBTAG00000007743	-0.578244	0.0598313	1.2230714
ENSBTAG00000007746	0.6870006	0.0600141	1.2217466
ENSBTAG00000007749	0.3058714	0.544488	0.2640117
ENSBTAG00000007753	NA	NA	NA
ENSBTAG00000007754	0.1907671	0.4765485	0.3218929
ENSBTAG00000007755	NA	NA	NA
ENSBTAG00000007756	0.3761614	0.2313683	0.6356962
ENSBTAG00000007757	0.5365096	0.1081109	0.9661307
ENSBTAG00000007758	-0.35352	0.470081	0.3278273
ENSBTAG00000007759	0.3063962	0.3950105	0.4033914
ENSBTAG00000007761	-0.187766	0.5867417	0.2315531
ENSBTAG00000007762	NA	NA	NA
ENSBTAG00000007763	0.2967632	0.3603136	0.4433193
ENSBTAG00000007765	NA	NA	NA
ENSBTAG00000007766	-0.299483	0.361326	0.4421008
ENSBTAG00000007767	-0.202283	0.469407	0.3284504
ENSBTAG00000007772	NA	NA	NA
ENSBTAG00000007773	0.402427	0.2335961	0.6315345
ENSBTAG00000007776	0.3638776	0.2636083	0.579041
ENSBTAG00000007777	0.0056064	0.9943362	0.0024667
ENSBTAG00000007778	1.1238242	0.0151413	1.8198374
ENSBTAG00000007779	0.0548987	0.8416639	0.0748613
ENSBTAG00000007780	-0.322137	0.4957121	0.3047705
ENSBTAG00000007782	0.3592374	0.1727986	0.7624598
ENSBTAG00000007783	NA	NA	NA
ENSBTAG00000007784	-0.157979	0.5517692	0.2582426
ENSBTAG00000007785	NA	NA	NA
ENSBTAG00000007786	0.8902463	0.0352877	1.4523768
ENSBTAG00000007787	0.0276704	0.9173511	0.0374644
ENSBTAG00000007788	0.2356868	0.4496467	0.3471285
ENSBTAG00000007791	0.2556437	0.5579829	0.2533791
ENSBTAG00000007793	NA	NA	NA
ENSBTAG00000007794	NA	NA	NA
ENSBTAG00000007796	-0.125346	0.795996	0.0990891
ENSBTAG00000007797	-0.141967	0.5728039	0.241994
ENSBTAG00000007798	NA	NA	NA

ENSBTAG00000007799	NA	NA	NA
ENSBTAG00000007802	-0.028867	0.9086737	0.0415921
ENSBTAG00000007804	0.4614085	0.2407352	0.6184603
ENSBTAG00000007806	-0.085681	0.727257	0.1383121
ENSBTAG00000007808	0.3450635	0.3773049	0.4233075
ENSBTAG00000007809	-0.029931	0.9719589	0.0123521
ENSBTAG00000007812	0.0761464	0.7736961	0.1114296
ENSBTAG00000007813	-0.335514	0.3966264	0.4016184
ENSBTAG00000007814	0.5780882	0.0620685	1.2071286
ENSBTAG00000007817	-0.042818	0.8715732	0.0596961
ENSBTAG00000007818	-0.128835	0.6395951	0.1940949
ENSBTAG00000007823	NA	NA	NA
ENSBTAG00000007825	0.3439988	0.2601686	0.5847452
ENSBTAG00000007826	NA	NA	NA
ENSBTAG00000007827	NA	NA	NA
ENSBTAG00000007828	0.2443111	0.6263742	0.2031661
ENSBTAG00000007829	-0.45461	0.2671	0.5733261
ENSBTAG00000007830	-0.234015	0.4692673	0.3285797
ENSBTAG00000007833	-0.213561	0.4459904	0.3506745
ENSBTAG00000007834	NA	NA	NA
ENSBTAG00000007835	-0.147621	0.5668549	0.2465281
ENSBTAG00000007836	-0.214502	0.4197605	0.3769984
ENSBTAG00000007837	0.8720051	0.0184255	1.7345796
ENSBTAG00000007838	NA	NA	NA
ENSBTAG00000007840	-0.221243	0.5100126	0.2924191
ENSBTAG00000007841	0.0121746	0.9794097	0.0090356
ENSBTAG00000007842	-0.19896	0.4352635	0.3612478
ENSBTAG00000007843	NA	NA	NA
ENSBTAG00000007844	-0.333219	0.2438188	0.6129328
ENSBTAG00000007846	NA	NA	NA
ENSBTAG00000007847	-0.041368	0.872801	0.0590848
ENSBTAG00000007850	-0.369598	0.2663183	0.574599
ENSBTAG00000007855	0.4152198	0.3744399	0.4266179
ENSBTAG00000007860	NA	NA	NA
ENSBTAG00000007863	-0.236726	0.3463443	0.460492
ENSBTAG00000007865	-0.744866	0.0481612	1.3173031
ENSBTAG00000007866	NA	NA	NA
ENSBTAG00000007867	-0.188961	0.4607642	0.3365213
ENSBTAG00000007870	0.1074857	0.7460161	0.1272518
ENSBTAG00000007871	0.0297546	0.9124471	0.0397923
ENSBTAG00000007872	-0.218551	0.4810327	0.3178254
ENSBTAG00000007873	NA	NA	NA
ENSBTAG00000007875	-0.192093	0.4679235	0.3298252
ENSBTAG00000007876	-0.217314	0.3993927	0.3985999
ENSBTAG00000007878	-0.037703	0.9318231	0.0306665
ENSBTAG00000007879	NA	NA	NA
ENSBTAG00000007880	0.2349808	0.4922533	0.3078113
ENSBTAG00000007881	NA	NA	NA
ENSBTAG00000007882	NA	NA	NA
ENSBTAG00000007884	-0.020554	0.9404717	0.0266543
ENSBTAG00000007887	NA	NA	NA
ENSBTAG00000007888	NA	NA	NA
ENSBTAG00000007890	0.1469904	0.8030648	0.0952494
ENSBTAG00000007893	0.2901598	0.3734389	0.4277805
ENSBTAG00000007895	-0.067794	0.8148822	0.0889052
ENSBTAG00000007896	0.5555596	0.2442484	0.6121683
ENSBTAG00000007897	0.4216723	0.1322922	0.8784659
ENSBTAG00000007898	0.5731825	0.099932	1.0002954

ENSBTAG00000007900	0.1628234	0.5207153	0.2833996
ENSBTAG00000007901	NA	NA	NA
ENSBTAG00000007904	NA	NA	NA
ENSBTAG00000007906	NA	NA	NA
ENSBTAG00000007909	-0.047496	0.8685649	0.0611977
ENSBTAG00000007910	-0.13866	0.5965917	0.2243228
ENSBTAG00000007913	-0.018623	0.9428563	0.0255545
ENSBTAG00000007916	NA	NA	NA
ENSBTAG00000007917	NA	NA	NA
ENSBTAG00000007920	-0.132849	0.6065539	0.2171306
ENSBTAG00000007921	NA	NA	NA
ENSBTAG00000007922	NA	NA	NA
ENSBTAG00000007923	0.2855024	0.3078009	0.5117301
ENSBTAG00000007927	NA	NA	NA
ENSBTAG00000007930	-0.098568	0.7570807	0.1208578
ENSBTAG00000007931	0.1223573	0.7532419	0.1230655
ENSBTAG00000007932	NA	NA	NA
ENSBTAG00000007933	0.3431757	0.2658587	0.5753491
ENSBTAG00000007934	0.2282663	0.4328302	0.3636825
ENSBTAG00000007935	-0.061337	0.8053257	0.0940284
ENSBTAG00000007937	0.1586015	0.7167526	0.1446307
ENSBTAG00000007939	0.1028285	0.6902813	0.1609739
ENSBTAG00000007942	-0.587839	0.0341687	1.466372
ENSBTAG00000007943	-0.56418	0.029571	1.5291341
ENSBTAG00000007944	NA	NA	NA
ENSBTAG00000007946	NA	NA	NA
ENSBTAG00000007948	NA	NA	NA
ENSBTAG00000007952	0.0063639	0.9800444	0.0087542
ENSBTAG00000007953	-0.202966	0.4334919	0.363019
ENSBTAG00000007954	0.5911298	0.0774555	1.1109477
ENSBTAG00000007956	NA	NA	NA
ENSBTAG00000007958	NA	NA	NA
ENSBTAG00000007960	0.1552133	0.5776424	0.238341
ENSBTAG00000007961	-0.036996	0.9032328	0.0442003
ENSBTAG00000007962	-0.302711	0.2531522	0.5966183
ENSBTAG00000007963	-0.15851	0.5331166	0.2731778
ENSBTAG00000007964	-0.538871	0.2142339	0.6691117
ENSBTAG00000007966	0.2227938	0.4489299	0.3478214
ENSBTAG00000007968	0.0070088	0.9836426	0.0071627
ENSBTAG00000007969	-0.084022	0.88893	0.0511325
ENSBTAG00000007970	-2.724182	0.004724	2.3256895
ENSBTAG00000007974	-0.043162	0.8679674	0.0614966
ENSBTAG00000007975	NA	NA	NA
ENSBTAG00000007976	-0.237259	0.4455789	0.3510754
ENSBTAG00000007979	-0.064158	0.8037109	0.0949001
ENSBTAG00000007981	-0.203125	0.5637778	0.248892
ENSBTAG00000007986	-0.098883	0.7606592	0.1188099
ENSBTAG00000007988	-0.279481	0.4053885	0.3921286
ENSBTAG00000007990	NA	NA	NA
ENSBTAG00000007992	NA	NA	NA
ENSBTAG00000007993	-0.055364	0.8395529	0.0759519
ENSBTAG00000007994	NA	NA	NA
ENSBTAG00000007996	NA	NA	NA
ENSBTAG00000007998	0.1399156	0.7444819	0.1281459
ENSBTAG00000008001	-0.216205	0.3911094	0.4077018
ENSBTAG00000008003	0.0644149	0.8130568	0.0898791
ENSBTAG00000008004	NA	NA	NA
ENSBTAG00000008005	NA	NA	NA

ENSBTAG00000008006	0.6103925	0.0952986	1.0209134
ENSBTAG00000008008	NA	NA	NA
ENSBTAG00000008009	NA	NA	NA
ENSBTAG00000008010	0.1125921	0.6813003	0.1666615
ENSBTAG00000008013	-0.115358	0.7985652	0.0976896
ENSBTAG00000008014	0.0825476	0.7483732	0.1258818
ENSBTAG00000008016	-0.161019	0.6493604	0.1875142
ENSBTAG00000008017	NA	NA	NA
ENSBTAG00000008021	NA	NA	NA
ENSBTAG00000008022	-0.049436	0.8494332	0.0708708
ENSBTAG00000008023	NA	NA	NA
ENSBTAG00000008024	-0.045471	0.8884426	0.0513706
ENSBTAG00000008025	-0.148198	0.5610368	0.2510086
ENSBTAG00000008027	NA	NA	NA
ENSBTAG00000008028	-1.01519	0.0116448	1.9338698
ENSBTAG00000008032	0.0963512	0.7110758	0.1480841
ENSBTAG00000008033	-0.021132	0.9349729	0.029201
ENSBTAG00000008034	0.4768603	0.0914265	1.0389278
ENSBTAG00000008036	NA	NA	NA
ENSBTAG00000008039	NA	NA	NA
ENSBTAG00000008040	-0.479343	0.0704225	1.1522888
ENSBTAG00000008047	NA	NA	NA
ENSBTAG00000008048	-0.459823	0.0793584	1.1004069
ENSBTAG00000008053	-0.158093	0.5747622	0.2405118
ENSBTAG00000008054	-0.302404	0.2274307	0.643151
ENSBTAG00000008056	NA	NA	NA
ENSBTAG00000008057	NA	NA	NA
ENSBTAG00000008059	NA	NA	NA
ENSBTAG00000008060	0.054192	0.8819288	0.0545665
ENSBTAG00000008061	0.4332406	0.1093722	0.9610932
ENSBTAG00000008062	0.3380948	0.3928337	0.4057913
ENSBTAG00000008063	0.6602408	0.1221645	0.9130551
ENSBTAG00000008064	NA	NA	NA
ENSBTAG00000008065	0.2009552	0.6062511	0.2173475
ENSBTAG00000008066	NA	NA	NA
ENSBTAG00000008068	-0.295741	0.2528515	0.5971345
ENSBTAG00000008072	0.0845857	0.7554309	0.1218053
ENSBTAG00000008074	NA	NA	NA
ENSBTAG00000008075	0.1238605	0.6576877	0.1819803
ENSBTAG00000008076	-0.112157	0.7469777	0.1266923
ENSBTAG00000008077	-0.434164	0.0992957	1.0030695
ENSBTAG00000008078	NA	NA	NA
ENSBTAG00000008079	0.2301049	0.6021398	0.2203026
ENSBTAG00000008082	-0.072813	0.7690632	0.1140379
ENSBTAG00000008083	0.0390527	0.879656	0.0556871
ENSBTAG00000008084	-0.229724	0.6354764	0.1969006
ENSBTAG00000008088	NA	NA	NA
ENSBTAG00000008089	0.587484	0.1498559	0.824326
ENSBTAG00000008090	0.1905465	0.5442098	0.2642337
ENSBTAG00000008091	0.7087032	0.0176882	1.7523153
ENSBTAG00000008092	NA	NA	NA
ENSBTAG00000008093	NA	NA	NA
ENSBTAG00000008096	1.3614289	0.0053666	2.2702995
ENSBTAG00000008097	NA	NA	NA
ENSBTAG00000008098	0.5396442	0.2563604	0.5911491
ENSBTAG00000008099	NA	NA	NA
ENSBTAG00000008100	-0.247672	0.6474611	0.1887863
ENSBTAG00000008101	0.1472409	0.651238	0.1862603

ENSBTAG00000008102	NA	NA	NA
ENSBTAG00000008103	-0.454659	0.0833365	1.0791646
ENSBTAG00000008105	0.8098738	0.0154702	1.8105049
ENSBTAG00000008109	0.2829497	0.5929611	0.2269738
ENSBTAG00000008110	0.0346803	0.9175199	0.0373845
ENSBTAG00000008111	NA	NA	NA
ENSBTAG00000008112	0.132739	0.6360116	0.196535
ENSBTAG00000008113	-0.095861	0.8825385	0.0542664
ENSBTAG00000008114	0.0939259	0.7026901	0.1532362
ENSBTAG00000008115	0.363785	0.2470768	0.607168
ENSBTAG00000008116	0.0230346	0.9260986	0.0333428
ENSBTAG00000008118	-0.119001	0.6506939	0.1866233
ENSBTAG00000008120	0.5287982	0.0989839	1.0044353
ENSBTAG00000008121	NA	NA	NA
ENSBTAG00000008122	-0.000946	0.9976385	0.0010268
ENSBTAG00000008124	0.5516993	0.1493124	0.8259042
ENSBTAG00000008125	NA	NA	NA
ENSBTAG00000008126	NA	NA	NA
ENSBTAG00000008129	NA	NA	NA
ENSBTAG00000008130	-0.118601	0.7211777	0.1419577
ENSBTAG00000008131	0.913351	0.0156326	1.8059682
ENSBTAG00000008132	-0.014431	0.9773878	0.0099331
ENSBTAG00000008133	0.0189611	0.9495478	0.0224832
ENSBTAG00000008134	NA	NA	NA
ENSBTAG00000008135	-0.040411	0.8796156	0.0557071
ENSBTAG00000008136	-0.184428	0.4506053	0.3462037
ENSBTAG00000008137	NA	NA	NA
ENSBTAG00000008138	NA	NA	NA
ENSBTAG00000008139	-1.060173	0.0354704	1.4501343
ENSBTAG00000008140	-0.138512	0.7542727	0.1224716
ENSBTAG00000008142	0.6190858	0.1087934	0.9633974
ENSBTAG00000008143	0.0607059	0.8529039	0.0690999
ENSBTAG00000008144	NA	NA	NA
ENSBTAG00000008145	0.3513779	0.4693073	0.3285427
ENSBTAG00000008147	-0.039464	0.9285061	0.0322152
ENSBTAG00000008150	-0.415396	0.1153814	0.9378642
ENSBTAG00000008151	0.3741817	0.1787474	0.7477601
ENSBTAG00000008153	0.0339121	0.8973085	0.0470582
ENSBTAG00000008154	1.2743507	0.0183047	1.7374386
ENSBTAG00000008155	NA	NA	NA
ENSBTAG00000008158	0.9027121	0.0389622	1.4093567
ENSBTAG00000008159	NA	NA	NA
ENSBTAG00000008160	0.559517	0.2238928	0.6499599
ENSBTAG00000008161	NA	NA	NA
ENSBTAG00000008165	-1.01311	0.0093504	2.0291679
ENSBTAG00000008167	-0.172269	0.5079184	0.2942061
ENSBTAG00000008168	NA	NA	NA
ENSBTAG00000008169	-0.179196	0.6506003	0.1866858
ENSBTAG00000008170	0.1226945	0.6723887	0.1723796
ENSBTAG00000008172	-0.587617	0.0244263	1.612143
ENSBTAG00000008173	0.039063	0.9086454	0.0416056
ENSBTAG00000008175	-0.693168	0.105539	0.9765871
ENSBTAG00000008180	NA	NA	NA
ENSBTAG00000008181	0.0948877	0.8276394	0.0821588
ENSBTAG00000008182	-0.733035	0.0895117	1.0481201
ENSBTAG00000008183	0.1608401	0.5657227	0.2473964
ENSBTAG00000008184	-0.328686	0.185061	0.7326852
ENSBTAG00000008185	-0.12054	0.6401447	0.1937219

ENSBTAG00000008186	0.0384272	0.8938943	0.0487138
ENSBTAG00000008188	0.0817323	0.8011196	0.0963026
ENSBTAG00000008190	NA	NA	NA
ENSBTAG00000008191	0.1800063	0.5919381	0.2277237
ENSBTAG00000008192	-0.895138	0.239242	0.6211626
ENSBTAG00000008193	NA	NA	NA
ENSBTAG00000008195	-0.246235	0.356653	0.4477541
ENSBTAG00000008196	0.0128462	0.9822073	0.0077968
ENSBTAG00000008197	NA	NA	NA
ENSBTAG00000008198	NA	NA	NA
ENSBTAG00000008201	NA	NA	NA
ENSBTAG00000008202	0.4176454	0.1736848	0.7602381
ENSBTAG00000008203	-0.068041	0.8115276	0.0906967
ENSBTAG00000008204	-0.236245	0.4592262	0.3379734
ENSBTAG00000008213	-0.317562	0.3108674	0.5074248
ENSBTAG00000008216	NA	NA	NA
ENSBTAG00000008218	-0.331176	0.2926154	0.5337029
ENSBTAG00000008219	-0.158654	0.5783612	0.2378009
ENSBTAG00000008220	-0.372424	0.4239222	0.3727139
ENSBTAG00000008222	0.2964027	0.5392823	0.2681838
ENSBTAG00000008223	NA	NA	NA
ENSBTAG00000008224	-0.088847	0.7190965	0.1432128
ENSBTAG00000008227	0.0486416	0.9256448	0.0335556
ENSBTAG00000008228	NA	NA	NA
ENSBTAG00000008232	-0.306024	0.2978697	0.5259736
ENSBTAG00000008236	NA	NA	NA
ENSBTAG00000008237	0.072969	0.7809794	0.1073604
ENSBTAG00000008238	NA	NA	NA
ENSBTAG00000008240	1.158072	0.0057704	2.238796
ENSBTAG00000008241	0.3190961	0.1925691	0.7154135
ENSBTAG00000008243	-0.13256	0.6491964	0.1876239
ENSBTAG00000008248	-0.448826	0.0956937	1.0191166
ENSBTAG00000008250	0.0152542	0.9814309	0.0081403
ENSBTAG00000008251	-0.100551	0.6901544	0.1610538
ENSBTAG00000008253	NA	NA	NA
ENSBTAG00000008255	NA	NA	NA
ENSBTAG00000008259	NA	NA	NA
ENSBTAG00000008260	0.0588133	0.8203405	0.0860058
ENSBTAG00000008267	0.3778978	0.2542478	0.5947429
ENSBTAG00000008269	-0.402041	0.1529514	0.8154464
ENSBTAG00000008271	0.098421	0.7250144	0.1396534
ENSBTAG00000008272	0.4289825	0.2300388	0.6381988
ENSBTAG00000008274	NA	NA	NA
ENSBTAG00000008275	0.8858547	0.0044434	2.3522875
ENSBTAG00000008278	NA	NA	NA
ENSBTAG00000008279	-0.41602	0.1378804	0.8604975
ENSBTAG00000008280	NA	NA	NA
ENSBTAG00000008283	0.1618188	0.607792	0.216245
ENSBTAG00000008285	-0.340847	0.187536	0.7269153
ENSBTAG00000008287	0.0451685	0.8864105	0.0523651
ENSBTAG00000008288	0.3395236	0.3646146	0.438166
ENSBTAG00000008289	-0.040868	0.8909109	0.0501657
ENSBTAG00000008291	0.8341213	0.0127936	1.8930073
ENSBTAG00000008292	-0.087809	0.7643744	0.1166939
ENSBTAG00000008293	-0.543033	0.0611145	1.2138555
ENSBTAG00000008294	1.1448714	6.96E-05	4.157178
ENSBTAG00000008295	NA	NA	NA
ENSBTAG00000008296	NA	NA	NA

ENSBTAG00000008299	NA	NA	NA
ENSBTAG00000008300	0.1282597	0.6728705	0.1720685
ENSBTAG00000008301	-1.180346	0.0001218	3.9144276
ENSBTAG00000008302	-0.095242	0.7407057	0.1303543
ENSBTAG00000008303	0.0578928	0.8186254	0.0869148
ENSBTAG00000008306	-0.017875	0.9428461	0.0255592
ENSBTAG00000008307	NA	NA	NA
ENSBTAG00000008309	-0.091046	0.8152511	0.0887086
ENSBTAG00000008310	-0.222817	0.3729306	0.428372
ENSBTAG00000008312	NA	NA	NA
ENSBTAG00000008313	0.1332339	0.7302293	0.1365407
ENSBTAG00000008314	0.0030189	0.9906261	0.0040902
ENSBTAG00000008320	0.2804701	0.5526886	0.2575195
ENSBTAG00000008321	0.0177014	0.9543978	0.0202706
ENSBTAG00000008322	NA	NA	NA
ENSBTAG00000008323	NA	NA	NA
ENSBTAG00000008327	NA	NA	NA
ENSBTAG00000008329	-0.083475	0.8511715	0.0699829
ENSBTAG00000008330	-0.326293	0.2836078	0.5472819
ENSBTAG00000008331	NA	NA	NA
ENSBTAG00000008332	-0.971394	0.0134615	1.870906
ENSBTAG00000008333	NA	NA	NA
ENSBTAG00000008338	-0.346491	0.3705491	0.4311542
ENSBTAG00000008339	-0.01127	0.9656321	0.0151883
ENSBTAG00000008340	0.1085928	0.7348994	0.1337721
ENSBTAG00000008341	0.2437433	0.5606458	0.2513115
ENSBTAG00000008343	NA	NA	NA
ENSBTAG00000008346	0.0757166	0.8025849	0.095509
ENSBTAG00000008347	-0.094754	0.7354485	0.1334477
ENSBTAG00000008348	NA	NA	NA
ENSBTAG00000008349	-0.091202	0.840276	0.0755781
ENSBTAG00000008350	-0.052811	0.8716164	0.0596746
ENSBTAG00000008351	NA	NA	NA
ENSBTAG00000008352	NA	NA	NA
ENSBTAG00000008353	0.691725	0.0381489	1.4185182
ENSBTAG00000008355	0.4362338	0.1682857	0.7739529
ENSBTAG00000008359	NA	NA	NA
ENSBTAG00000008361	0.0300011	0.9254434	0.0336501
ENSBTAG00000008362	0.1269448	0.6684424	0.174936
ENSBTAG00000008363	NA	NA	NA
ENSBTAG00000008365	NA	NA	NA
ENSBTAG00000008366	NA	NA	NA
ENSBTAG00000008367	-0.933112	0.0835146	1.0782378
ENSBTAG00000008369	2.5237164	0.0225345	1.6471529
ENSBTAG00000008370	NA	NA	NA
ENSBTAG00000008371	0.127851	0.6345017	0.1975672
ENSBTAG00000008372	0.101468	0.7690674	0.1140356
ENSBTAG00000008373	NA	NA	NA
ENSBTAG00000008376	0.5969407	0.2477118	0.6060534
ENSBTAG00000008378	0.0098827	0.9686702	0.0138241
ENSBTAG00000008380	-0.400715	0.3748827	0.4261046
ENSBTAG00000008382	0.3295511	0.503491	0.2980083
ENSBTAG00000008384	NA	NA	NA
ENSBTAG00000008385	0.0882672	0.774301	0.1110902
ENSBTAG00000008386	-0.227541	0.3920102	0.4067027
ENSBTAG00000008388	0.2943065	0.3357292	0.4740108
ENSBTAG00000008389	-0.394173	0.1195222	0.9225514
ENSBTAG00000008390	-0.027169	0.9131105	0.0394767

ENSBTAG00000008394	0.6166134	0.3198922	0.4949964
ENSBTAG00000008395	0.217806	0.4735796	0.3246071
ENSBTAG00000008396	0.0406315	0.8709099	0.0600268
ENSBTAG00000008397	0.5520362	0.3560142	0.4485327
ENSBTAG00000008399	0.5154383	0.2551622	0.5931837
ENSBTAG00000008401	-0.611568	0.2361171	0.6268726
ENSBTAG00000008403	-0.13702	0.5828639	0.2344328
ENSBTAG00000008406	-0.395225	0.4382899	0.3582385
ENSBTAG00000008409	-0.255917	0.5528517	0.2573914
ENSBTAG00000008410	NA	NA	NA
ENSBTAG00000008411	-0.217119	0.4898509	0.3099361
ENSBTAG00000008412	NA	NA	NA
ENSBTAG00000008414	-0.181737	0.4784988	0.3201191
ENSBTAG00000008416	-0.048578	0.8966573	0.0473735
ENSBTAG00000008417	-0.111715	0.6888677	0.1618642
ENSBTAG00000008419	0.4935624	0.1087838	0.9634359
ENSBTAG00000008420	NA	NA	NA
ENSBTAG00000008421	-0.072796	0.7747963	0.1108125
ENSBTAG00000008423	0.1251089	0.7082023	0.1498427
ENSBTAG00000008424	0.0770017	0.8084214	0.0923622
ENSBTAG00000008428	NA	NA	NA
ENSBTAG00000008429	-0.237028	0.3648529	0.4378822
ENSBTAG00000008430	NA	NA	NA
ENSBTAG00000008431	-0.930859	0.1783845	0.7486429
ENSBTAG00000008432	-0.202213	0.4237324	0.3729083
ENSBTAG00000008433	-0.074063	0.8310711	0.0803618
ENSBTAG00000008434	-0.144129	0.642697	0.1919937
ENSBTAG00000008435	0.1531867	0.5345616	0.2720023
ENSBTAG00000008436	NA	NA	NA
ENSBTAG00000008437	0.0484065	0.9087121	0.0415737
ENSBTAG00000008438	0.1202642	0.6640323	0.1778108
ENSBTAG00000008439	-0.131393	0.7304215	0.1364264
ENSBTAG00000008441	-1.073845	0.0246904	1.6074725
ENSBTAG00000008442	0.054128	0.8627391	0.0641205
ENSBTAG00000008443	-0.186206	0.4654409	0.3321355
ENSBTAG00000008446	0.4577476	0.0804581	1.0944305
ENSBTAG00000008448	-0.303865	0.3281593	0.4839153
ENSBTAG00000008449	NA	NA	NA
ENSBTAG00000008452	NA	NA	NA
ENSBTAG00000008453	0.3506682	0.2045297	0.6892436
ENSBTAG00000008457	0.0570219	0.825674	0.0831914
ENSBTAG00000008461	0.0384777	0.8788581	0.0560813
ENSBTAG00000008462	-0.347641	0.3813009	0.4187322
ENSBTAG00000008463	NA	NA	NA
ENSBTAG00000008464	NA	NA	NA
ENSBTAG00000008465	0.3710858	0.4568227	0.3402523
ENSBTAG00000008466	0.215437	0.4158439	0.3810696
ENSBTAG00000008467	NA	NA	NA
ENSBTAG00000008468	-0.314924	0.298916	0.5244508
ENSBTAG00000008470	0.2492826	0.3539793	0.4510221
ENSBTAG00000008471	NA	NA	NA
ENSBTAG00000008472	-0.014611	0.9549512	0.0200188
ENSBTAG00000008477	NA	NA	NA
ENSBTAG00000008479	NA	NA	NA
ENSBTAG00000008480	-0.050459	0.8403502	0.0755397
ENSBTAG00000008482	-0.322931	0.1901269	0.7209565
ENSBTAG00000008483	0.3243908	0.2006609	0.6975373
ENSBTAG00000008484	0.0537611	0.8690026	0.0609789

ENSBTAG00000008485	-0.27095	0.4837969	0.315337
ENSBTAG00000008487	NA	NA	NA
ENSBTAG00000008490	-0.141419	0.5969783	0.2240415
ENSBTAG00000008492	-0.321203	0.3805569	0.4195804
ENSBTAG00000008493	0.1654414	0.7170965	0.1444224
ENSBTAG00000008494	NA	NA	NA
ENSBTAG00000008495	NA	NA	NA
ENSBTAG00000008497	-0.659304	0.0275372	1.5600798
ENSBTAG00000008498	-0.137984	0.6029198	0.2197404
ENSBTAG00000008499	NA	NA	NA
ENSBTAG00000008501	NA	NA	NA
ENSBTAG00000008504	NA	NA	NA
ENSBTAG00000008505	NA	NA	NA
ENSBTAG00000008507	NA	NA	NA
ENSBTAG00000008509	NA	NA	NA
ENSBTAG00000008510	0.2946052	0.3464542	0.4603541
ENSBTAG00000008513	NA	NA	NA
ENSBTAG00000008517	NA	NA	NA
ENSBTAG00000008518	-0.143453	0.6949127	0.1580697
ENSBTAG00000008520	0.4850386	0.0791293	1.1016627
ENSBTAG00000008523	0.4656247	0.1733649	0.7610389
ENSBTAG00000008525	-0.228308	0.3605454	0.44304
ENSBTAG00000008527	0.1128833	0.6647042	0.1773715
ENSBTAG00000008528	0.2124405	0.433566	0.3629448
ENSBTAG00000008530	0.3842493	0.4272148	0.3693537
ENSBTAG00000008533	NA	NA	NA
ENSBTAG00000008534	NA	NA	NA
ENSBTAG00000008535	-0.162328	0.5963445	0.2245028
ENSBTAG00000008537	0.0842871	0.8343529	0.0786502
ENSBTAG00000008538	NA	NA	NA
ENSBTAG00000008539	NA	NA	NA
ENSBTAG00000008540	NA	NA	NA
ENSBTAG00000008541	-0.17912	0.7026916	0.1532352
ENSBTAG00000008542	NA	NA	NA
ENSBTAG00000008543	-0.134459	0.6946394	0.1582406
ENSBTAG00000008545	-0.317333	0.476573	0.3218706
ENSBTAG00000008548	-0.056847	0.8336383	0.0790223
ENSBTAG00000008550	-0.616561	0.2539574	0.5952391
ENSBTAG00000008551	0.2041359	0.6495452	0.1873906
ENSBTAG00000008552	0.0393454	0.9416686	0.0261019
ENSBTAG00000008553	NA	NA	NA
ENSBTAG00000008554	NA	NA	NA
ENSBTAG00000008555	NA	NA	NA
ENSBTAG00000008556	NA	NA	NA
ENSBTAG00000008562	0.350642	0.2551743	0.593163
ENSBTAG00000008564	0.8664021	0.2220834	0.6534839
ENSBTAG00000008567	NA	NA	NA
ENSBTAG00000008568	NA	NA	NA
ENSBTAG00000008570	0.0816456	0.756868	0.1209799
ENSBTAG00000008571	NA	NA	NA
ENSBTAG00000008573	0.5364632	0.1571789	0.8036059
ENSBTAG00000008575	0.255266	0.5694267	0.2445621
ENSBTAG00000008576	NA	NA	NA
ENSBTAG00000008577	-0.112167	0.6623414	0.1789181
ENSBTAG00000008578	-0.171333	0.5556279	0.255216
ENSBTAG00000008579	0.5825471	0.145024	0.83856
ENSBTAG00000008583	-0.41853	0.3632489	0.4397957
ENSBTAG00000008584	0.0341098	0.9365527	0.0284678

ENSBTAG00000008585	-0.197693	0.5416547	0.2662775
ENSBTAG00000008587	NA	NA	NA
ENSBTAG00000008590	-0.325683	0.2240918	0.649574
ENSBTAG00000008591	0.2727708	0.3226055	0.4913282
ENSBTAG00000008592	NA	NA	NA
ENSBTAG00000008593	0.0871829	0.8171795	0.0876825
ENSBTAG00000008595	-0.028147	0.9242642	0.0342039
ENSBTAG00000008596	0.5214744	0.1448245	0.8391579
ENSBTAG00000008597	NA	NA	NA
ENSBTAG00000008600	0.3192568	0.4609944	0.3363044
ENSBTAG00000008603	NA	NA	NA
ENSBTAG00000008605	NA	NA	NA
ENSBTAG00000008606	-0.089582	0.7441487	0.1283403
ENSBTAG00000008607	0.1526849	0.7447161	0.1280092
ENSBTAG00000008609	0.8965526	0.0073007	2.1366357
ENSBTAG00000008611	0.6838239	0.0632117	1.1992027
ENSBTAG00000008612	0.3239383	0.217568	0.662405
ENSBTAG00000008613	-0.142388	0.6511952	0.1862888
ENSBTAG00000008614	NA	NA	NA
ENSBTAG00000008617	0.0044428	0.988983	0.0048112
ENSBTAG00000008619	-0.10162	0.6920997	0.1598314
ENSBTAG00000008621	0.220902	0.4254003	0.3712022
ENSBTAG00000008624	NA	NA	NA
ENSBTAG00000008625	0.0216574	0.9520443	0.0213428
ENSBTAG00000008626	NA	NA	NA
ENSBTAG00000008629	0.0747893	0.8200606	0.0861541
ENSBTAG00000008631	0.2889464	0.4651501	0.3324069
ENSBTAG00000008632	-0.117833	0.7098906	0.1488086
ENSBTAG00000008633	0.1801898	0.6109358	0.2140044
ENSBTAG00000008634	0.6377838	0.5099864	0.2924414
ENSBTAG00000008635	0.7698992	0.0054506	2.2635583
ENSBTAG00000008636	-0.511192	0.1795678	0.7457716
ENSBTAG00000008638	NA	NA	NA
ENSBTAG00000008639	NA	NA	NA
ENSBTAG00000008641	NA	NA	NA
ENSBTAG00000008642	0.0455589	0.8817847	0.0546374
ENSBTAG00000008644	NA	NA	NA
ENSBTAG00000008645	0.3388544	0.1999169	0.6991505
ENSBTAG00000008646	-0.064527	0.8136393	0.0895681
ENSBTAG00000008647	NA	NA	NA
ENSBTAG00000008648	-0.182751	0.4672283	0.3304708
ENSBTAG00000008649	-0.472298	0.1888036	0.7239897
ENSBTAG00000008650	-0.706229	0.1557242	0.8076438
ENSBTAG00000008652	0.124028	0.6336294	0.1981647
ENSBTAG00000008654	-0.529647	0.1179588	0.9282698
ENSBTAG00000008661	0.5298316	0.2777276	0.5563809
ENSBTAG00000008664	0.1747745	0.5249302	0.2798985
ENSBTAG00000008665	-0.062278	0.8443266	0.0734895
ENSBTAG00000008673	NA	NA	NA
ENSBTAG00000008674	0.0364182	0.896354	0.0475204
ENSBTAG00000008682	1.1701207	0.010846	1.9647296
ENSBTAG00000008683	-0.767241	0.0028248	2.5490188
ENSBTAG00000008684	NA	NA	NA
ENSBTAG00000008685	0.093951	0.7903839	0.1021619
ENSBTAG00000008686	0.2604409	0.4890007	0.3106906
ENSBTAG00000008688	-0.815032	0.0160099	1.7956109
ENSBTAG00000008690	NA	NA	NA
ENSBTAG00000008692	0.064675	0.8086003	0.0922661

ENSBTAG00000008693	NA	NA	NA
ENSBTAG00000008695	0.1874533	0.6150016	0.2111237
ENSBTAG00000008696	0.3316985	0.2484819	0.6047053
ENSBTAG00000008699	-0.493438	0.0441685	1.3548873
ENSBTAG00000008703	0.1961498	0.5091697	0.2931375
ENSBTAG00000008704	NA	NA	NA
ENSBTAG00000008705	0.8165961	0.0637788	1.1953236
ENSBTAG00000008708	NA	NA	NA
ENSBTAG00000008709	0.0712787	0.8320755	0.0798373
ENSBTAG00000008710	NA	NA	NA
ENSBTAG00000008711	0.3561637	0.4183321	0.3784788
ENSBTAG00000008713	0.4347817	0.1832867	0.736869
ENSBTAG00000008714	-0.536315	0.2200633	0.6574524
ENSBTAG00000008716	-0.434716	0.1004441	0.9980757
ENSBTAG00000008717	0.9417034	0.0217978	1.6615879
ENSBTAG00000008718	0.3484121	0.479926	0.3188257
ENSBTAG00000008719	-0.036223	0.9528205	0.0209889
ENSBTAG00000008721	0.431196	0.3934268	0.4051361
ENSBTAG00000008723	-0.371411	0.3555722	0.4490722
ENSBTAG00000008726	0.4756712	0.2893253	0.5386136
ENSBTAG00000008728	-0.264585	0.4252686	0.3713367
ENSBTAG00000008729	-0.261288	0.3964265	0.4018373
ENSBTAG00000008730	-0.380468	0.2777322	0.5563738
ENSBTAG00000008731	-0.267771	0.2955833	0.5293202
ENSBTAG00000008732	0.3670116	0.2931471	0.5329145
ENSBTAG00000008733	-0.070918	0.8708502	0.0600565
ENSBTAG00000008734	0.5560283	0.2119577	0.6737508
ENSBTAG00000008735	0.135949	0.7527393	0.1233554
ENSBTAG00000008736	-0.105199	0.6689663	0.1745958
ENSBTAG00000008737	-0.258882	0.2953326	0.5296886
ENSBTAG00000008739	-0.64762	0.1363854	0.8652322
ENSBTAG00000008743	-0.000466	0.9988623	0.0004944
ENSBTAG00000008744	0.1320768	0.6329217	0.19865
ENSBTAG00000008747	0.3375848	0.371209	0.4303815
ENSBTAG00000008749	NA	NA	NA
ENSBTAG00000008752	NA	NA	NA
ENSBTAG00000008753	0.0684173	0.7915478	0.1015228
ENSBTAG00000008755	0.3607252	0.1728258	0.7623915
ENSBTAG00000008756	NA	NA	NA
ENSBTAG00000008758	NA	NA	NA
ENSBTAG00000008759	0.0238141	0.9409107	0.0264516
ENSBTAG00000008761	0.031598	0.9210139	0.0357338
ENSBTAG00000008762	NA	NA	NA
ENSBTAG00000008763	-1.338251	0.008418	2.0747912
ENSBTAG00000008764	0.1246049	0.7499801	0.1249503
ENSBTAG00000008765	0.0607264	0.8360432	0.0777713
ENSBTAG00000008766	0.3216285	0.4666162	0.3310402
ENSBTAG00000008771	0.1456524	0.6564383	0.1828061
ENSBTAG00000008772	0.2793832	0.4555668	0.341448
ENSBTAG00000008773	NA	NA	NA
ENSBTAG00000008774	-0.07773	0.8749963	0.0579938
ENSBTAG00000008777	NA	NA	NA
ENSBTAG00000008778	NA	NA	NA
ENSBTAG00000008783	NA	NA	NA
ENSBTAG00000008787	-0.026297	0.9431361	0.0254256
ENSBTAG00000008788	NA	NA	NA
ENSBTAG00000008789	0.3067828	0.4773758	0.3211396
ENSBTAG00000008792	0.2849714	0.528649	0.2768325

ENSBTAG00000008793	NA	NA	NA
ENSBTAG00000008794	0.1583618	0.6388176	0.1946232
ENSBTAG00000008797	NA	NA	NA
ENSBTAG00000008798	0.1002528	0.8223053	0.0849669
ENSBTAG00000008800	NA	NA	NA
ENSBTAG00000008801	0.0237697	0.9423307	0.0257966
ENSBTAG00000008802	-0.225778	0.4246792	0.371939
ENSBTAG00000008804	0.1633674	0.6972133	0.1566343
ENSBTAG00000008805	0.1815809	0.4912084	0.3087342
ENSBTAG00000008807	NA	NA	NA
ENSBTAG00000008808	0.2847211	0.5810091	0.235817
ENSBTAG00000008809	-0.419137	0.1769169	0.7522308
ENSBTAG00000008810	-0.13682	0.5879525	0.2306577
ENSBTAG00000008812	-0.486272	0.0536751	1.2702269
ENSBTAG00000008814	0.2531668	0.526319	0.2787509
ENSBTAG00000008815	0.1320197	0.7023656	0.1534368
ENSBTAG00000008816	-0.929269	0.0041482	2.3821427
ENSBTAG00000008817	-0.052716	0.836456	0.0775569
ENSBTAG00000008819	NA	NA	NA
ENSBTAG00000008821	-0.072867	0.7958644	0.0991609
ENSBTAG00000008825	0.0786252	0.8066744	0.0933017
ENSBTAG00000008826	-0.137637	0.739604	0.1310008
ENSBTAG00000008827	-1.951413	1.81E-05	4.7427055
ENSBTAG00000008828	NA	NA	NA
ENSBTAG00000008832	-1.213449	0.0194343	1.7114318
ENSBTAG00000008833	NA	NA	NA
ENSBTAG00000008835	0.0919765	0.7228894	0.1409282
ENSBTAG00000008836	-0.133081	0.6925887	0.1595246
ENSBTAG00000008837	0.3018842	0.3067262	0.5132491
ENSBTAG00000008839	-0.180032	0.7599613	0.1192085
ENSBTAG00000008840	0.6307528	0.0493731	1.3065093
ENSBTAG00000008841	0.4431702	0.2716272	0.5660268
ENSBTAG00000008842	-0.150208	0.5607763	0.2512104
ENSBTAG00000008844	0.2613899	0.4515604	0.3452842
ENSBTAG00000008845	0.3095687	0.3590892	0.4447976
ENSBTAG00000008849	-0.117891	0.7157235	0.1452547
ENSBTAG00000008851	NA	NA	NA
ENSBTAG00000008852	-0.498584	0.3585027	0.4455075
ENSBTAG00000008853	0.1349378	0.6473247	0.1888778
ENSBTAG00000008854	0.1754127	0.710117	0.1486701
ENSBTAG00000008857	NA	NA	NA
ENSBTAG00000008860	0.1122459	0.7052591	0.1516513
ENSBTAG00000008862	0.1084628	0.6623408	0.1789185
ENSBTAG00000008863	0.1753411	0.4856158	0.3137072
ENSBTAG00000008864	0.5436301	0.2094089	0.6790049
ENSBTAG00000008866	-0.609903	0.0212712	1.672208
ENSBTAG00000008868	-0.163246	0.5223474	0.2820406
ENSBTAG00000008870	NA	NA	NA
ENSBTAG00000008873	0.1052249	0.6959869	0.1573989
ENSBTAG00000008877	0.0613737	0.8852663	0.0529261
ENSBTAG00000008880	0.0832861	0.847565	0.071827
ENSBTAG00000008881	0.1579998	0.7055305	0.1514842
ENSBTAG00000008883	0.2544975	0.3556878	0.4489311
ENSBTAG00000008884	0.1059045	0.6849159	0.1643628
ENSBTAG00000008886	0.6832411	0.0881812	1.0546238
ENSBTAG00000008887	0.2060955	0.5545132	0.2560881
ENSBTAG00000008888	0.2945001	0.3240217	0.489426
ENSBTAG00000008893	-0.158739	0.6003154	0.2216205

ENSBTAG00000008895	-0.249029	0.3089683	0.5100861
ENSBTAG00000008896	-0.056655	0.8194295	0.0864884
ENSBTAG00000008897	NA	NA	NA
ENSBTAG00000008898	NA	NA	NA
ENSBTAG00000008900	NA	NA	NA
ENSBTAG00000008902	-0.056018	0.9032761	0.0441795
ENSBTAG00000008905	NA	NA	NA
ENSBTAG00000008908	-0.278086	0.4464033	0.3502726
ENSBTAG00000008909	-0.156841	0.5557961	0.2550845
ENSBTAG00000008911	1.2250558	0.0587045	1.2313283
ENSBTAG00000008913	0.6906742	0.2620277	0.5816528
ENSBTAG00000008915	-0.152722	0.5818286	0.2352049
ENSBTAG00000008916	0.0785981	0.8621469	0.0644188
ENSBTAG00000008920	-0.177437	0.5484214	0.2608856
ENSBTAG00000008921	-0.180391	0.6371058	0.1957884
ENSBTAG00000008922	NA	NA	NA
ENSBTAG00000008923	NA	NA	NA
ENSBTAG00000008924	NA	NA	NA
ENSBTAG00000008925	0.17711	0.515597	0.2876896
ENSBTAG00000008926	-0.081431	0.7477494	0.1262439
ENSBTAG00000008931	0.1169538	0.6483312	0.1882031
ENSBTAG00000008932	NA	NA	NA
ENSBTAG00000008933	0.5045895	0.1138187	0.9437866
ENSBTAG00000008934	NA	NA	NA
ENSBTAG00000008935	-0.0518	0.8405888	0.0754164
ENSBTAG00000008936	NA	NA	NA
ENSBTAG00000008937	0.1352382	0.6653466	0.176952
ENSBTAG00000008938	0.4576338	0.3771095	0.4235325
ENSBTAG00000008939	0.5372072	0.1013877	0.9940148
ENSBTAG00000008940	NA	NA	NA
ENSBTAG00000008942	NA	NA	NA
ENSBTAG00000008943	0.3716787	0.3896428	0.4093333
ENSBTAG00000008944	NA	NA	NA
ENSBTAG00000008945	NA	NA	NA
ENSBTAG00000008946	NA	NA	NA
ENSBTAG00000008947	NA	NA	NA
ENSBTAG00000008948	NA	NA	NA
ENSBTAG00000008950	NA	NA	NA
ENSBTAG00000008951	1.1166605	0.0101297	1.994402
ENSBTAG00000008952	NA	NA	NA
ENSBTAG00000008953	-0.013826	0.9652436	0.0153631
ENSBTAG00000008954	-0.124602	0.7573915	0.1206796
ENSBTAG00000008956	NA	NA	NA
ENSBTAG00000008958	0.2285205	0.4424366	0.354149
ENSBTAG00000008959	0.2451655	0.5645298	0.2483132
ENSBTAG00000008962	-0.049127	0.8778375	0.0565859
ENSBTAG00000008963	NA	NA	NA
ENSBTAG00000008964	-0.179545	0.5715139	0.2429732
ENSBTAG00000008966	-0.256508	0.4083387	0.3889795
ENSBTAG00000008967	-0.12292	0.6314835	0.199638
ENSBTAG00000008969	0.3806517	0.2091664	0.679508
ENSBTAG00000008973	0.3910511	0.3884582	0.4106557
ENSBTAG00000008977	0.0163359	0.95082	0.0219017
ENSBTAG00000008978	-0.07178	0.7715736	0.1126227
ENSBTAG00000008981	0.1217408	0.7620748	0.1180024
ENSBTAG00000008985	0.0645698	0.8187462	0.0868507
ENSBTAG00000008987	0.1145228	0.6475674	0.188715
ENSBTAG00000008989	0.0276535	0.9537987	0.0205433

ENSBTAG00000008990	-0.086742	0.7560645	0.1214411
ENSBTAG00000008991	0.1950084	0.5329449	0.2733177
ENSBTAG00000008993	-0.196091	0.5613178	0.2507911
ENSBTAG00000008994	-0.288935	0.433493	0.3630179
ENSBTAG00000008996	0.4590395	0.3847415	0.414831
ENSBTAG00000008997	1.0857712	0.0113848	1.943673
ENSBTAG00000008998	-0.247484	0.4804312	0.3183688
ENSBTAG00000009002	NA	NA	NA
ENSBTAG00000009005	-0.046982	0.8785928	0.0562124
ENSBTAG00000009006	0.3103008	0.3084074	0.5108752
ENSBTAG00000009012	-0.449633	0.4770421	0.3214432
ENSBTAG00000009014	NA	NA	NA
ENSBTAG00000009019	0.2696092	0.4822187	0.316756
ENSBTAG00000009020	0.1671868	0.6470685	0.1890497
ENSBTAG00000009021	-0.139259	0.6190056	0.2083054
ENSBTAG00000009022	NA	NA	NA
ENSBTAG00000009023	-0.026639	0.9153715	0.0384026
ENSBTAG00000009024	NA	NA	NA
ENSBTAG00000009026	-0.244858	0.5401909	0.2674527
ENSBTAG00000009029	0.4506863	0.2140756	0.6694328
ENSBTAG00000009030	NA	NA	NA
ENSBTAG00000009032	-0.612209	0.1123504	0.9494252
ENSBTAG00000009033	NA	NA	NA
ENSBTAG00000009034	0.2010317	0.4342801	0.3622301
ENSBTAG00000009035	NA	NA	NA
ENSBTAG00000009036	0.2081525	0.4371244	0.3593949
ENSBTAG00000009037	0.0476531	0.9219438	0.0352955
ENSBTAG00000009039	NA	NA	NA
ENSBTAG00000009042	-0.284973	0.2574958	0.5892299
ENSBTAG00000009046	NA	NA	NA
ENSBTAG00000009047	0.2217261	0.4270406	0.3695308
ENSBTAG00000009048	0.1398856	0.6904095	0.1608932
ENSBTAG00000009049	-0.024526	0.9708855	0.012832
ENSBTAG00000009050	0.0494878	0.8686933	0.0611335
ENSBTAG00000009051	NA	NA	NA
ENSBTAG00000009055	-0.044372	0.8747807	0.0581008
ENSBTAG00000009058	-0.404741	0.4230661	0.3735917
ENSBTAG00000009059	0.0815645	0.8271123	0.0824355
ENSBTAG00000009061	-0.476781	0.0635656	1.196778
ENSBTAG00000009062	NA	NA	NA
ENSBTAG00000009064	-0.063311	0.9085635	0.0416447
ENSBTAG00000009065	-0.293339	0.3393512	0.4693506
ENSBTAG00000009067	-0.028581	0.9077527	0.0420325
ENSBTAG00000009071	0.3228639	0.3144014	0.5025155
ENSBTAG00000009072	-0.233525	0.4319903	0.364526
ENSBTAG00000009075	0.1785155	0.7275088	0.1381618
ENSBTAG00000009076	NA	NA	NA
ENSBTAG00000009077	0.4514372	0.1911097	0.7187173
ENSBTAG00000009078	NA	NA	NA
ENSBTAG00000009079	0.0700154	0.8409846	0.075212
ENSBTAG00000009080	-1.078789	0.0003029	3.5186563
ENSBTAG00000009084	-0.077523	0.7721972	0.1122718
ENSBTAG00000009085	0.0129019	0.9659928	0.0150261
ENSBTAG00000009086	0.2399913	0.5236423	0.2809653
ENSBTAG00000009087	-0.104349	0.6704294	0.1736469
ENSBTAG00000009091	0.1987611	0.624596	0.2044008
ENSBTAG00000009097	0.3652603	0.4331355	0.3633762
ENSBTAG00000009098	-0.118228	0.6409131	0.1932008

ENSBTAG00000009099	0.397784	0.3666467	0.4357523
ENSBTAG00000009101	0.1533535	0.7519586	0.123806
ENSBTAG00000009103	-0.050673	0.8637511	0.0636114
ENSBTAG00000009104	-0.178321	0.562122	0.2501694
ENSBTAG00000009105	NA	NA	NA
ENSBTAG00000009106	-0.151136	0.5422013	0.2658395
ENSBTAG00000009107	-0.607288	0.257085	0.5899232
ENSBTAG00000009109	0.056887	0.8215257	0.0853788
ENSBTAG00000009110	-0.595801	0.2615438	0.5824555
ENSBTAG00000009112	0.3228619	0.4732424	0.3249164
ENSBTAG00000009115	-1.066016	0.0111809	1.9515222
ENSBTAG00000009117	-0.307048	0.4089626	0.3883164
ENSBTAG00000009121	-0.23506	0.3642132	0.4386443
ENSBTAG00000009123	0.203281	0.6369024	0.1959271
ENSBTAG00000009124	-0.163739	0.7283819	0.1376408
ENSBTAG00000009125	-0.464262	0.2205744	0.656445
ENSBTAG00000009126	NA	NA	NA
ENSBTAG00000009127	-0.562617	0.0398512	1.3995588
ENSBTAG00000009128	NA	NA	NA
ENSBTAG00000009129	0.1947367	0.5221473	0.282207
ENSBTAG00000009131	-0.347755	0.1900574	0.7211151
ENSBTAG00000009132	NA	NA	NA
ENSBTAG00000009137	NA	NA	NA
ENSBTAG00000009138	0.019542	0.9544844	0.0202311
ENSBTAG00000009139	-0.859243	0.0026144	2.5826339
ENSBTAG00000009140	NA	NA	NA
ENSBTAG00000009141	-0.481553	0.1337843	0.8735948
ENSBTAG00000009142	0.4058254	0.2611021	0.5831897
ENSBTAG00000009143	NA	NA	NA
ENSBTAG00000009144	1.2053523	0.2827544	0.5485906
ENSBTAG00000009145	NA	NA	NA
ENSBTAG00000009148	NA	NA	NA
ENSBTAG00000009150	0.5415567	0.2528602	0.5971195
ENSBTAG00000009151	-0.194305	0.5369126	0.2700964
ENSBTAG00000009152	NA	NA	NA
ENSBTAG00000009153	0.7017665	0.1615589	0.7916691
ENSBTAG00000009154	-0.202962	0.4147319	0.3822325
ENSBTAG00000009156	0.3518522	0.4419208	0.3546556
ENSBTAG00000009157	0.3370579	0.4798939	0.3188548
ENSBTAG00000009159	0.0505681	0.8693756	0.0607926
ENSBTAG00000009161	NA	NA	NA
ENSBTAG00000009163	0.122481	0.7574764	0.1206309
ENSBTAG00000009165	0.5664408	0.1931508	0.7141035
ENSBTAG00000009167	0.4870839	0.112417	0.9491678
ENSBTAG00000009169	0.335208	0.3541187	0.4508512
ENSBTAG00000009171	NA	NA	NA
ENSBTAG00000009172	0.3780999	0.3336198	0.4767482
ENSBTAG00000009174	NA	NA	NA
ENSBTAG00000009175	0.2439491	0.3843039	0.4153253
ENSBTAG00000009176	0.3230286	0.3921885	0.4065052
ENSBTAG00000009177	0.1074031	0.7757627	0.1102711
ENSBTAG00000009178	0.3304202	0.4463985	0.3502773
ENSBTAG00000009181	-0.347247	0.1630876	0.7875792
ENSBTAG00000009182	-0.027831	0.9107206	0.0406148
ENSBTAG00000009183	0.2065144	0.6696148	0.1741749
ENSBTAG00000009186	NA	NA	NA
ENSBTAG00000009188	-0.309669	0.2219081	0.6538269
ENSBTAG00000009190	0.2782602	0.4676482	0.3300808

ENSBTAG00000009191	-0.029278	0.9431185	0.0254337
ENSBTAG00000009194	-0.669274	0.0477288	1.3212193
ENSBTAG00000009198	NA	NA	NA
ENSBTAG00000009199	0.0654658	0.9013511	0.045106
ENSBTAG00000009200	-0.019592	0.9482728	0.0230667
ENSBTAG00000009201	0.2216965	0.6483915	0.1881627
ENSBTAG00000009206	NA	NA	NA
ENSBTAG00000009207	-0.271585	0.2981028	0.525634
ENSBTAG00000009208	0.0492076	0.9092283	0.0413271
ENSBTAG00000009209	-0.268077	0.5760572	0.2395344
ENSBTAG00000009210	-0.275808	0.2976151	0.526345
ENSBTAG00000009211	-0.243667	0.4015257	0.3962866
ENSBTAG00000009212	NA	NA	NA
ENSBTAG00000009213	-0.164362	0.5925484	0.2272761
ENSBTAG00000009214	-0.393937	0.1864029	0.7295474
ENSBTAG00000009216	-0.284664	0.4930667	0.3070943
ENSBTAG00000009217	NA	NA	NA
ENSBTAG00000009218	0.1771409	0.7114193	0.1478744
ENSBTAG00000009219	NA	NA	NA
ENSBTAG00000009223	NA	NA	NA
ENSBTAG00000009226	NA	NA	NA
ENSBTAG00000009228	0.1677519	0.5698568	0.2442342
ENSBTAG00000009230	NA	NA	NA
ENSBTAG00000009231	0.0879304	0.8391246	0.0761736
ENSBTAG00000009232	-0.23794	0.3370025	0.4723668
ENSBTAG00000009233	-0.030266	0.9056066	0.0430604
ENSBTAG00000009235	NA	NA	NA
ENSBTAG00000009236	0.2648727	0.3855421	0.4139282
ENSBTAG00000009237	0.1098146	0.6911843	0.1604061
ENSBTAG00000009238	-0.399956	0.2754456	0.5599642
ENSBTAG00000009239	0.7359428	0.0129313	1.8883569
ENSBTAG00000009242	0.0459592	0.8607428	0.0651266
ENSBTAG00000009243	-0.161092	0.6555034	0.183425
ENSBTAG00000009245	-0.053421	0.8459462	0.0726573
ENSBTAG00000009246	-0.143947	0.5968379	0.2241436
ENSBTAG00000009249	NA	NA	NA
ENSBTAG00000009251	0.0750208	0.7660148	0.1157628
ENSBTAG00000009252	NA	NA	NA
ENSBTAG00000009255	NA	NA	NA
ENSBTAG00000009256	-0.189984	0.6164701	0.210088
ENSBTAG00000009258	NA	NA	NA
ENSBTAG00000009260	0.3664873	0.2792711	0.5539741
ENSBTAG00000009263	-0.16324	0.5867164	0.2315718
ENSBTAG00000009265	NA	NA	NA
ENSBTAG00000009266	NA	NA	NA
ENSBTAG00000009267	0.1515453	0.5492096	0.2602619
ENSBTAG00000009269	NA	NA	NA
ENSBTAG00000009271	0.333082	0.4505594	0.3462479
ENSBTAG00000009272	0.4362483	0.1394392	0.8556152
ENSBTAG00000009273	NA	NA	NA
ENSBTAG00000009274	NA	NA	NA
ENSBTAG00000009275	-0.210687	0.4776648	0.3208768
ENSBTAG00000009277	NA	NA	NA
ENSBTAG00000009278	-0.476977	0.0845227	1.0730264
ENSBTAG00000009279	0.0746103	0.7650295	0.1163218
ENSBTAG00000009281	-0.031112	0.9201272	0.0361521
ENSBTAG00000009282	NA	NA	NA
ENSBTAG00000009284	NA	NA	NA

ENSBTAG00000009286	0.2637432	0.4052086	0.3923213
ENSBTAG00000009287	-0.780274	0.0447023	1.3496698
ENSBTAG00000009288	0.613929	0.1620261	0.790415
ENSBTAG00000009289	NA	NA	NA
ENSBTAG00000009290	0.1097182	0.8229887	0.0846061
ENSBTAG00000009291	NA	NA	NA
ENSBTAG00000009292	-1.148161	0.0028587	2.5438358
ENSBTAG00000009293	0.4134508	0.1800591	0.7445849
ENSBTAG00000009294	NA	NA	NA
ENSBTAG00000009297	0.5608079	0.2195191	0.6585277
ENSBTAG00000009298	0.0532629	0.8789112	0.056055
ENSBTAG00000009299	-0.247956	0.3136655	0.5035332
ENSBTAG00000009302	0.5841073	0.2771432	0.5572957
ENSBTAG00000009304	-0.211042	0.4627549	0.3346489
ENSBTAG00000009305	-0.12024	0.6764659	0.1697541
ENSBTAG00000009306	NA	NA	NA
ENSBTAG00000009307	0.0439918	0.8633436	0.0638164
ENSBTAG00000009308	0.0193412	0.9489503	0.0227565
ENSBTAG00000009309	0.0378142	0.9158042	0.0381974
ENSBTAG00000009310	0.3991248	0.2847838	0.5454848
ENSBTAG00000009314	NA	NA	NA
ENSBTAG00000009315	-0.085806	0.7821999	0.1066822
ENSBTAG00000009327	NA	NA	NA
ENSBTAG00000009328	NA	NA	NA
ENSBTAG00000009330	0.3198473	0.2614025	0.5826902
ENSBTAG00000009331	NA	NA	NA
ENSBTAG00000009332	NA	NA	NA
ENSBTAG00000009334	0.4143642	0.6374905	0.1955263
ENSBTAG00000009336	NA	NA	NA
ENSBTAG00000009337	NA	NA	NA
ENSBTAG00000009338	NA	NA	NA
ENSBTAG00000009340	-0.025106	0.9409058	0.0264538
ENSBTAG00000009341	NA	NA	NA
ENSBTAG00000009343	0.0048502	0.990072	0.0043332
ENSBTAG00000009345	-0.137185	0.6004576	0.2215177
ENSBTAG00000009346	-0.0389	0.8962314	0.0475799
ENSBTAG00000009348	NA	NA	NA
ENSBTAG00000009349	NA	NA	NA
ENSBTAG00000009350	NA	NA	NA
ENSBTAG00000009351	-0.183675	0.4646753	0.3328504
ENSBTAG00000009353	NA	NA	NA
ENSBTAG00000009354	-0.164382	0.7257483	0.139214
ENSBTAG00000009355	NA	NA	NA
ENSBTAG00000009357	0.1204373	0.7602377	0.1190506
ENSBTAG00000009358	-0.009839	0.9862922	0.0059944
ENSBTAG00000009362	-0.618591	0.0155591	1.8080166
ENSBTAG00000009363	1.6274507	0.0017	2.7695518
ENSBTAG00000009366	-0.231841	0.5113121	0.2913139
ENSBTAG00000009368	0.0321839	0.9145927	0.0387723
ENSBTAG00000009371	NA	NA	NA
ENSBTAG00000009372	-0.093882	0.7437644	0.1285646
ENSBTAG00000009374	0.0950533	0.8011176	0.0963037
ENSBTAG00000009376	0.2611562	0.4736649	0.3245288
ENSBTAG00000009378	0.0091513	0.9927481	0.003161
ENSBTAG00000009381	0.287603	0.5634195	0.2491681
ENSBTAG00000009382	NA	NA	NA
ENSBTAG00000009383	NA	NA	NA
ENSBTAG00000009384	0.1949673	0.5746487	0.2405976

ENSBTAG00000009386	NA	NA	NA
ENSBTAG00000009387	0.0325901	0.9105947	0.0406749
ENSBTAG00000009389	-0.188998	0.4811248	0.3177422
ENSBTAG00000009390	-0.273065	0.5586104	0.2528909
ENSBTAG00000009391	-0.029652	0.9335016	0.029885
ENSBTAG00000009392	NA	NA	NA
ENSBTAG00000009393	NA	NA	NA
ENSBTAG00000009394	-0.213195	0.5150887	0.288118
ENSBTAG00000009396	NA	NA	NA
ENSBTAG00000009400	0.3726877	0.3727939	0.4285312
ENSBTAG00000009401	0.1823451	0.5797328	0.2367721
ENSBTAG00000009405	0.0007798	1	0
ENSBTAG00000009406	NA	NA	NA
ENSBTAG00000009407	-0.472172	0.1799271	0.7449035
ENSBTAG00000009408	NA	NA	NA
ENSBTAG00000009410	-0.094765	0.7531341	0.1231277
ENSBTAG00000009412	-0.22336	0.5618423	0.2503855
ENSBTAG00000009414	0.0314098	0.8992958	0.0460975
ENSBTAG00000009415	0.449908	0.1534091	0.814149
ENSBTAG00000009416	0.1488366	0.609798	0.214814
ENSBTAG00000009417	-0.020912	0.9387337	0.0274576
ENSBTAG00000009418	NA	NA	NA
ENSBTAG00000009420	-0.274203	0.5692104	0.2447271
ENSBTAG00000009421	NA	NA	NA
ENSBTAG00000009423	NA	NA	NA
ENSBTAG00000009426	-0.049109	0.9082956	0.0417728
ENSBTAG00000009427	0.1152523	0.7336523	0.1345097
ENSBTAG00000009428	-0.084606	0.7617752	0.1181731
ENSBTAG00000009430	-0.384724	0.4636911	0.3337712
ENSBTAG00000009431	0.2112718	0.5014447	0.299777
ENSBTAG00000009432	-0.184964	0.6464673	0.1894534
ENSBTAG00000009433	NA	NA	NA
ENSBTAG00000009434	0.6860126	0.0552049	1.2580225
ENSBTAG00000009435	-0.066228	0.7888938	0.1029815
ENSBTAG00000009436	NA	NA	NA
ENSBTAG00000009438	NA	NA	NA
ENSBTAG00000009439	-0.090693	0.7672603	0.1150573
ENSBTAG00000009441	-0.212585	0.4714779	0.3265386
ENSBTAG00000009443	NA	NA	NA
ENSBTAG00000009444	NA	NA	NA
ENSBTAG00000009446	-0.104366	0.69364	0.1588659
ENSBTAG00000009449	-0.171577	0.4885001	0.3111354
ENSBTAG00000009451	-0.480785	0.1749402	0.7571104
ENSBTAG00000009453	0.1156834	0.7172123	0.1443523
ENSBTAG00000009455	NA	NA	NA
ENSBTAG00000009458	0.0871633	0.7968201	0.0986397
ENSBTAG00000009459	0.0037591	0.9964898	0.0015272
ENSBTAG00000009460	0.4798159	0.2136252	0.6703476
ENSBTAG00000009461	0.1076577	0.766408	0.11554
ENSBTAG00000009462	0.1817673	0.4957631	0.3047258
ENSBTAG00000009468	NA	NA	NA
ENSBTAG00000009469	-0.098347	0.7113618	0.1479095
ENSBTAG00000009470	0.0456316	0.8668055	0.0620783
ENSBTAG00000009471	-0.312926	0.4817199	0.3172054
ENSBTAG00000009472	-0.361295	0.2310175	0.6363552
ENSBTAG00000009475	-0.069768	0.8174091	0.0875605
ENSBTAG00000009476	0.2333547	0.5921749	0.22755
ENSBTAG00000009477	0.0442924	0.8929992	0.0491489

ENSBTAG00000009478	NA	NA	NA
ENSBTAG00000009479	-0.025583	0.9295532	0.0317257
ENSBTAG00000009481	NA	NA	NA
ENSBTAG00000009482	-0.503335	0.0925358	1.0336902
ENSBTAG00000009483	-0.093582	0.738019	0.1319324
ENSBTAG00000009484	0.3531814	0.3408914	0.4673839
ENSBTAG00000009486	-0.404217	0.6777875	0.1689064
ENSBTAG00000009487	-0.002331	0.9952574	0.0020646
ENSBTAG00000009488	0.5093306	0.158171	0.8008731
ENSBTAG00000009489	-0.918914	0.0317758	1.497904
ENSBTAG00000009490	-0.173602	0.704706	0.151992
ENSBTAG00000009491	-0.219033	0.4632561	0.3341789
ENSBTAG00000009493	-0.572305	0.2657715	0.5754917
ENSBTAG00000009494	NA	NA	NA
ENSBTAG00000009495	0.3856799	0.1502034	0.8233202
ENSBTAG00000009496	0.2902841	0.3984579	0.3996176
ENSBTAG00000009498	-0.054015	0.8456155	0.0728271
ENSBTAG00000009500	0.0064054	0.9802042	0.0086835
ENSBTAG00000009501	NA	NA	NA
ENSBTAG00000009502	-0.174035	0.8484565	0.0713704
ENSBTAG00000009504	0.8796745	0.0626927	1.2027829
ENSBTAG00000009506	0.034281	0.9154457	0.0383674
ENSBTAG00000009507	NA	NA	NA
ENSBTAG00000009508	-0.188816	0.4517923	0.3450612
ENSBTAG00000009509	-0.226186	0.3939411	0.4045687
ENSBTAG00000009510	0.3140521	0.5338246	0.2726014
ENSBTAG00000009512	NA	NA	NA
ENSBTAG00000009513	0.0616975	0.8395636	0.0759464
ENSBTAG00000009514	-0.087986	0.7423786	0.1293746
ENSBTAG00000009516	0.1820761	0.4894876	0.3102583
ENSBTAG00000009517	-0.022543	0.9355515	0.0289323
ENSBTAG00000009518	-0.22449	0.3998082	0.3981483
ENSBTAG00000009519	-0.173445	0.5294623	0.2761649
ENSBTAG00000009520	NA	NA	NA
ENSBTAG00000009521	-0.371373	0.2273215	0.6433595
ENSBTAG00000009522	-0.080416	0.7725045	0.112099
ENSBTAG00000009523	-0.437391	0.1072984	0.9694067
ENSBTAG00000009526	0.0320874	0.8984667	0.046498
ENSBTAG00000009527	0.0146516	0.976567	0.010298
ENSBTAG00000009533	0.0302424	0.9093858	0.0412518
ENSBTAG00000009534	0.0091137	0.9727345	0.0120057
ENSBTAG00000009535	-0.058276	0.8237086	0.0842264
ENSBTAG00000009537	NA	NA	NA
ENSBTAG00000009541	-0.316662	0.2155528	0.6664462
ENSBTAG00000009542	0.3190276	0.3494001	0.456677
ENSBTAG00000009543	0.0443466	0.8706137	0.0601745
ENSBTAG00000009545	0.2385278	0.544989	0.2636122
ENSBTAG00000009547	-0.312421	0.2119263	0.6738152
ENSBTAG00000009548	NA	NA	NA
ENSBTAG00000009549	NA	NA	NA
ENSBTAG00000009552	-0.246175	0.3550146	0.4497538
ENSBTAG00000009554	-0.102198	0.6977147	0.1563221
ENSBTAG00000009558	NA	NA	NA
ENSBTAG00000009560	0.0036847	0.9934396	0.0028585
ENSBTAG00000009562	0.019232	0.9713473	0.0126255
ENSBTAG00000009565	-0.092095	0.7176596	0.1440815
ENSBTAG00000009566	-0.174754	0.5964665	0.2244139
ENSBTAG00000009568	0.0680035	0.8429659	0.07419

ENSBTAG00000009569	0.0513212	0.8708637	0.0600498
ENSBTAG00000009570	-0.720614	0.1975609	0.704299
ENSBTAG00000009573	NA	NA	NA
ENSBTAG00000009574	0.1719413	0.5594516	0.2522375
ENSBTAG00000009575	-0.115326	0.7697581	0.1136457
ENSBTAG00000009576	-4.075095	3.54E-05	4.45051
ENSBTAG00000009578	0.0342141	0.8893221	0.0509409
ENSBTAG00000009579	0.1901764	0.5001883	0.3008665
ENSBTAG00000009580	0.372761	0.3285895	0.4833464
ENSBTAG00000009581	NA	NA	NA
ENSBTAG00000009584	-0.344401	0.1800271	0.744662
ENSBTAG00000009585	NA	NA	NA
ENSBTAG00000009586	NA	NA	NA
ENSBTAG00000009595	0.1288303	0.6587709	0.1812656
ENSBTAG00000009596	NA	NA	NA
ENSBTAG00000009598	-0.141505	0.5746531	0.2405943
ENSBTAG00000009599	NA	NA	NA
ENSBTAG00000009600	-0.159153	0.6114422	0.2136446
ENSBTAG00000009602	-0.051925	0.842362	0.0745012
ENSBTAG00000009603	-0.029239	0.9190417	0.0366648
ENSBTAG00000009611	0.0041317	0.9933218	0.00291
ENSBTAG00000009614	0.5908242	0.1527285	0.8160799
ENSBTAG00000009615	0.0647631	0.8128962	0.0899649
ENSBTAG00000009617	-0.523906	0.2632499	0.5796318
ENSBTAG00000009618	NA	NA	NA
ENSBTAG00000009620	-0.458358	0.3405939	0.4677631
ENSBTAG00000009622	NA	NA	NA
ENSBTAG00000009624	-0.138635	0.5737894	0.2412475
ENSBTAG00000009625	NA	NA	NA
ENSBTAG00000009631	0.0370207	0.9590123	0.0181758
ENSBTAG00000009632	0.0583391	0.8454635	0.0729051
ENSBTAG00000009634	NA	NA	NA
ENSBTAG00000009635	0.0934326	0.7263135	0.1388759
ENSBTAG00000009636	0.2241238	0.4670056	0.3306779
ENSBTAG00000009637	-0.979236	0.0014193	2.8479172
ENSBTAG00000009638	0.0166957	0.9606348	0.0174417
ENSBTAG00000009639	NA	NA	NA
ENSBTAG00000009641	-0.040692	0.8783257	0.0563444
ENSBTAG00000009642	NA	NA	NA
ENSBTAG00000009643	-0.227013	0.4829373	0.3161092
ENSBTAG00000009646	-0.19111	0.4592409	0.3379594
ENSBTAG00000009647	-0.092009	0.710429	0.1484793
ENSBTAG00000009649	NA	NA	NA
ENSBTAG00000009654	-0.271623	0.4096563	0.3875804
ENSBTAG00000009655	0.2223905	0.5025734	0.2988005
ENSBTAG00000009656	-1.928232	0.2506209	0.6009828
ENSBTAG00000009657	-0.128573	0.6289824	0.2013615
ENSBTAG00000009658	NA	NA	NA
ENSBTAG00000009661	-0.513775	0.1579139	0.8015797
ENSBTAG00000009663	0.2404751	0.3614336	0.4419715
ENSBTAG00000009664	0.1868767	0.5477822	0.2613921
ENSBTAG00000009665	0.0645667	0.8042896	0.0945875
ENSBTAG00000009667	-0.107016	0.6652484	0.1770162
ENSBTAG00000009668	0.0926531	0.7877095	0.1036339
ENSBTAG00000009673	NA	NA	NA
ENSBTAG00000009674	NA	NA	NA
ENSBTAG00000009676	-0.044838	0.8685518	0.0612043
ENSBTAG00000009677	0.6268693	0.1332269	0.875408

ENSBTAG00000009679	-0.358028	0.2772028	0.5572024
ENSBTAG00000009680	-0.21394	0.526052	0.2789714
ENSBTAG00000009681	-0.073527	0.8263395	0.0828415
ENSBTAG00000009682	0.031305	0.9284788	0.032228
ENSBTAG00000009683	-0.056131	0.8247132	0.0836971
ENSBTAG00000009685	0.5130054	0.1650284	0.7824414
ENSBTAG00000009686	0.1185193	0.8120992	0.0903909
ENSBTAG00000009687	0.0381804	0.8764538	0.057271
ENSBTAG00000009689	-0.1761	0.4823692	0.3166204
ENSBTAG00000009691	NA	NA	NA
ENSBTAG00000009693	0.2625746	0.3492965	0.4568058
ENSBTAG00000009694	0.1225887	0.6801155	0.1674173
ENSBTAG00000009696	-0.159309	0.5748943	0.240412
ENSBTAG00000009698	-0.106699	0.6727134	0.1721699
ENSBTAG00000009701	NA	NA	NA
ENSBTAG00000009702	-0.925784	0.000448	3.3486958
ENSBTAG00000009703	-0.116712	0.6641713	0.1777199
ENSBTAG00000009704	0.3201347	0.2924935	0.5338837
ENSBTAG00000009705	0.2162903	0.5114693	0.2911805
ENSBTAG00000009707	-0.320613	0.2145095	0.6685534
ENSBTAG00000009708	-0.233626	0.5137178	0.2892754
ENSBTAG00000009709	-0.204962	0.5523945	0.2577507
ENSBTAG00000009711	-0.073459	0.773845	0.111346
ENSBTAG00000009713	-0.029667	0.9215881	0.0354631
ENSBTAG00000009714	NA	NA	NA
ENSBTAG00000009715	0.3325414	0.2327976	0.6330215
ENSBTAG00000009717	-0.061498	0.8128085	0.0900118
ENSBTAG00000009719	0.3367824	0.4753544	0.3229824
ENSBTAG00000009725	1.4485193	2.50E-06	5.6026354
ENSBTAG00000009726	0.0228475	0.9395572	0.0270768
ENSBTAG00000009727	-0.070831	0.7931742	0.1006314
ENSBTAG00000009732	0.118542	0.7063197	0.1509987
ENSBTAG00000009733	-0.623026	0.2252789	0.6472796
ENSBTAG00000009734	-0.042514	0.8833022	0.0538907
ENSBTAG00000009735	NA	NA	NA
ENSBTAG00000009736	NA	NA	NA
ENSBTAG00000009737	NA	NA	NA
ENSBTAG00000009738	0.1375831	0.644902	0.1905063
ENSBTAG00000009739	NA	NA	NA
ENSBTAG00000009742	0.2081743	0.5500459	0.2596011
ENSBTAG00000009743	-0.070664	0.7886586	0.103111
ENSBTAG00000009744	0.2405473	0.4972754	0.303403
ENSBTAG00000009746	-0.309969	0.3456177	0.4614041
ENSBTAG00000009747	-0.215011	0.431532	0.364987
ENSBTAG00000009748	NA	NA	NA
ENSBTAG00000009749	-0.309795	0.3835965	0.4161253
ENSBTAG00000009755	-0.059414	0.8208747	0.0857231
ENSBTAG00000009757	0.0451484	0.8624199	0.0642812
ENSBTAG00000009760	NA	NA	NA
ENSBTAG00000009761	0.0700064	0.7823998	0.1065713
ENSBTAG00000009764	NA	NA	NA
ENSBTAG00000009765	0.0451283	0.8751752	0.057905
ENSBTAG00000009768	0.5406963	0.226168	0.6455687
ENSBTAG00000009770	-0.036808	0.8833194	0.0538822
ENSBTAG00000009772	0.1806624	0.6912073	0.1603917
ENSBTAG00000009773	-0.824522	0.0208998	1.6798583
ENSBTAG00000009774	NA	NA	NA
ENSBTAG00000009775	NA	NA	NA

ENSBTAG00000009777	-0.159184	0.5941259	0.2261215
ENSBTAG00000009778	-0.375751	0.3050919	0.5155693
ENSBTAG00000009780	0.1432889	0.5606681	0.2512942
ENSBTAG00000009782	NA	NA	NA
ENSBTAG00000009783	NA	NA	NA
ENSBTAG00000009784	-0.043609	0.9060143	0.042865
ENSBTAG00000009785	-0.060503	0.8899462	0.0506363
ENSBTAG00000009786	-0.451509	0.2065685	0.684936
ENSBTAG00000009788	NA	NA	NA
ENSBTAG00000009789	0.0538217	0.8432324	0.0740527
ENSBTAG00000009795	0.1811987	0.5552467	0.255514
ENSBTAG00000009796	0.255804	0.602377	0.2201316
ENSBTAG00000009797	NA	NA	NA
ENSBTAG00000009798	NA	NA	NA
ENSBTAG00000009800	NA	NA	NA
ENSBTAG00000009803	NA	NA	NA
ENSBTAG00000009804	0.1524103	0.560523	0.2514065
ENSBTAG00000009805	-0.07221	0.7725755	0.112059
ENSBTAG00000009806	-0.115741	0.6605929	0.1800661
ENSBTAG00000009811	0.0064935	0.9863865	0.0059529
ENSBTAG00000009812	NA	NA	NA
ENSBTAG00000009813	0.3485377	0.3869066	0.4123939
ENSBTAG00000009816	NA	NA	NA
ENSBTAG00000009819	NA	NA	NA
ENSBTAG00000009822	0.0274036	0.9264749	0.0331663
ENSBTAG00000009824	0.5662949	0.2175007	0.6625394
ENSBTAG00000009826	-0.446399	0.1455728	0.8369197
ENSBTAG00000009828	NA	NA	NA
ENSBTAG00000009830	-0.657998	0.1306272	0.8839664
ENSBTAG00000009831	NA	NA	NA
ENSBTAG00000009832	NA	NA	NA
ENSBTAG00000009833	NA	NA	NA
ENSBTAG00000009834	-0.797601	0.1298046	0.8867098
ENSBTAG00000009835	NA	NA	NA
ENSBTAG00000009836	NA	NA	NA
ENSBTAG00000009837	NA	NA	NA
ENSBTAG00000009838	-0.353207	0.2186915	0.6601681
ENSBTAG00000009839	0.3806772	0.2833426	0.5476882
ENSBTAG00000009841	0.2953427	0.4495559	0.3472163
ENSBTAG00000009842	NA	NA	NA
ENSBTAG00000009844	0.6602149	0.0634189	1.1977812
ENSBTAG00000009845	-0.141031	0.716068	0.1450457
ENSBTAG00000009846	-0.00273	1	0
ENSBTAG00000009848	NA	NA	NA
ENSBTAG00000009849	NA	NA	NA
ENSBTAG00000009850	-0.59376	0.2208214	0.6559587
ENSBTAG00000009851	0.2464503	0.4836779	0.3154438
ENSBTAG00000009852	0.2296212	0.5419867	0.2660114
ENSBTAG00000009854	0.0141361	0.9670422	0.0145546
ENSBTAG00000009855	-0.039638	0.8865787	0.0522827
ENSBTAG00000009856	0.1919501	0.6184758	0.2086773
ENSBTAG00000009859	-0.273842	0.2766174	0.5581205
ENSBTAG00000009861	0.2232923	0.60215	0.2202953
ENSBTAG00000009863	0.0920833	0.7582331	0.1201972
ENSBTAG00000009867	NA	NA	NA
ENSBTAG00000009868	NA	NA	NA
ENSBTAG00000009870	NA	NA	NA
ENSBTAG00000009871	-0.146035	0.5565462	0.2544988

ENSBTAG00000009872	0.6447761	0.143835	0.8421355
ENSBTAG00000009873	NA	NA	NA
ENSBTAG00000009874	0.2654936	0.4608241	0.3364649
ENSBTAG00000009876	NA	NA	NA
ENSBTAG00000009877	-0.158387	0.5334864	0.2728766
ENSBTAG00000009879	0.213446	0.6162073	0.2102732
ENSBTAG00000009881	-0.161095	0.5874226	0.2310494
ENSBTAG00000009885	0.0127662	0.9651584	0.0154014
ENSBTAG00000009886	-0.088237	0.818737	0.0868556
ENSBTAG00000009887	0.0885759	0.7311528	0.1359919
ENSBTAG00000009888	-0.159297	0.5699993	0.2441257
ENSBTAG00000009889	0.3686236	0.2031674	0.692146
ENSBTAG00000009891	NA	NA	NA
ENSBTAG00000009894	NA	NA	NA
ENSBTAG00000009895	0.5559627	0.1085438	0.9643951
ENSBTAG00000009897	-0.32252	0.2090706	0.679707
ENSBTAG00000009899	-0.116356	0.6737035	0.1715312
ENSBTAG00000009902	-0.136366	0.5795845	0.2368832
ENSBTAG00000009903	0.3187911	0.395801	0.4025231
ENSBTAG00000009905	0.1178379	0.7680266	0.1146238
ENSBTAG00000009906	-0.213644	0.3993983	0.3985938
ENSBTAG00000009907	-0.467814	0.382264	0.4176366
ENSBTAG00000009908	-0.091135	0.7211428	0.1419787
ENSBTAG00000009911	NA	NA	NA
ENSBTAG00000009912	-0.102672	0.692501	0.1595796
ENSBTAG00000009914	-0.365316	0.1904198	0.7202879
ENSBTAG00000009915	0.2142741	0.412972	0.3840794
ENSBTAG00000009916	0.5014517	0.2228129	0.6520597
ENSBTAG00000009917	-0.417968	0.3597599	0.4439873
ENSBTAG00000009918	0.2865145	0.4076842	0.3896761
ENSBTAG00000009923	0.8045638	0.0086434	2.0633134
ENSBTAG00000009924	-0.14043	0.7877352	0.1036198
ENSBTAG00000009928	-0.102971	0.7453936	0.1276143
ENSBTAG00000009933	0.3712836	0.278568	0.5550688
ENSBTAG00000009937	NA	NA	NA
ENSBTAG00000009938	NA	NA	NA
ENSBTAG00000009939	0.0167121	0.9467003	0.0237875
ENSBTAG00000009941	-0.18733	0.5243561	0.2803737
ENSBTAG00000009942	0.0280427	0.9197694	0.036321
ENSBTAG00000009943	NA	NA	NA
ENSBTAG00000009945	NA	NA	NA
ENSBTAG00000009948	0.5996875	0.1439711	0.8417246
ENSBTAG00000009949	-0.299293	0.2594355	0.5859705
ENSBTAG00000009950	NA	NA	NA
ENSBTAG00000009951	-0.458771	0.0873604	1.0586852
ENSBTAG00000009952	-0.766812	0.0124288	1.9055701
ENSBTAG00000009956	NA	NA	NA
ENSBTAG00000009958	0.3014998	0.5178582	0.2857891
ENSBTAG00000009959	0.440844	0.0939989	1.0268771
ENSBTAG00000009960	-0.007405	0.9785993	0.0093951
ENSBTAG00000009961	0.2612284	0.4474365	0.3492686
ENSBTAG00000009962	-0.314114	0.239713	0.6203084
ENSBTAG00000009963	0.2240208	0.4575645	0.3395477
ENSBTAG00000009964	NA	NA	NA
ENSBTAG00000009965	0.0858898	0.7695509	0.1137627
ENSBTAG00000009966	0.5474757	0.2127173	0.6721973
ENSBTAG00000009969	0.1693407	0.5241753	0.2805235
ENSBTAG00000009972	NA	NA	NA

ENSBTAG00000009974	-0.148132	0.7504854	0.1246577
ENSBTAG00000009975	NA	NA	NA
ENSBTAG00000009978	NA	NA	NA
ENSBTAG00000009979	0.1306254	0.8056855	0.0938345
ENSBTAG00000009982	-0.109125	0.7834623	0.1059819
ENSBTAG00000009983	NA	NA	NA
ENSBTAG00000009984	-0.36823	0.2501181	0.6018549
ENSBTAG00000009985	0.1674303	0.5555855	0.2552491
ENSBTAG00000009987	0.5994207	0.182538	0.7386466
ENSBTAG00000009988	-0.315823	0.3654091	0.4372206
ENSBTAG00000009990	0.1587069	0.5844046	0.2332864
ENSBTAG00000009991	0.8667075	0.0080341	2.0950627
ENSBTAG00000009994	0.4799651	0.2010009	0.6968021
ENSBTAG00000009995	-0.097996	0.694316	0.1584428
ENSBTAG00000009996	0.6346086	0.0834144	1.0787588
ENSBTAG00000009997	0.5884735	0.155795	0.8074464
ENSBTAG00000009998	0.0518489	0.8790355	0.0559936
ENSBTAG00000010001	0.3949961	0.1566204	0.8051518
ENSBTAG00000010002	0.3159981	0.4136747	0.3833411
ENSBTAG00000010006	-0.146936	0.5647301	0.2481591
ENSBTAG00000010007	NA	NA	NA
ENSBTAG00000010008	0.90826	0.10164	0.9929355
ENSBTAG00000010009	0.0117281	0.9661357	0.0149619
ENSBTAG00000010012	0.0458722	0.8542287	0.0684259
ENSBTAG00000010013	0.0874417	0.7230295	0.140844
ENSBTAG00000010015	0.2911188	0.5928589	0.2270486
ENSBTAG00000010016	NA	NA	NA
ENSBTAG00000010018	-0.209254	0.4434638	0.3531418
ENSBTAG00000010023	-0.231589	0.5291729	0.2764024
ENSBTAG00000010024	NA	NA	NA
ENSBTAG00000010026	0.0804484	0.8528822	0.0691109
ENSBTAG00000010027	NA	NA	NA
ENSBTAG00000010029	NA	NA	NA
ENSBTAG00000010030	1.1153874	0.0178988	1.7471759
ENSBTAG00000010032	NA	NA	NA
ENSBTAG00000010033	NA	NA	NA
ENSBTAG00000010036	0.0487577	0.8545192	0.0682782
ENSBTAG00000010037	-0.018766	0.9673313	0.0144248
ENSBTAG00000010042	0.196645	0.4543325	0.3426262
ENSBTAG00000010046	0.271774	0.4290171	0.3675254
ENSBTAG00000010047	0.217845	0.512292	0.2904824
ENSBTAG00000010048	NA	NA	NA
ENSBTAG00000010050	0.1532619	0.6555333	0.1834052
ENSBTAG00000010052	0.0350981	0.9288509	0.032054
ENSBTAG00000010057	NA	NA	NA
ENSBTAG00000010059	0.3988228	0.1978708	0.7036183
ENSBTAG00000010060	NA	NA	NA
ENSBTAG00000010062	NA	NA	NA
ENSBTAG00000010063	-0.113385	0.6661913	0.1764011
ENSBTAG00000010065	NA	NA	NA
ENSBTAG00000010068	NA	NA	NA
ENSBTAG00000010069	0.4212321	0.4406882	0.3558686
ENSBTAG00000010070	NA	NA	NA
ENSBTAG00000010071	-0.182809	0.4703251	0.3276019
ENSBTAG00000010073	-0.139911	0.5688419	0.2450084
ENSBTAG00000010077	0.9456182	0.0599851	1.2219564
ENSBTAG00000010082	-0.265487	0.2935095	0.5323779
ENSBTAG00000010083	-0.184472	0.4840178	0.3151387

ENSBTAG00000010085	-0.315901	0.2647543	0.577157
ENSBTAG00000010089	NA	NA	NA
ENSBTAG00000010091	-0.040014	0.9117661	0.0401165
ENSBTAG00000010096	NA	NA	NA
ENSBTAG00000010100	-0.567426	0.1075865	0.9682424
ENSBTAG00000010101	0.1954678	0.6278373	0.2021529
ENSBTAG00000010103	NA	NA	NA
ENSBTAG00000010105	0.4981465	0.1571795	0.803604
ENSBTAG00000010106	0.4039278	0.2302559	0.6377893
ENSBTAG00000010107	NA	NA	NA
ENSBTAG00000010109	NA	NA	NA
ENSBTAG00000010111	-0.970399	0.03924	1.4062714
ENSBTAG00000010112	NA	NA	NA
ENSBTAG00000010113	0.2637498	0.4584367	0.3387206
ENSBTAG00000010116	NA	NA	NA
ENSBTAG00000010118	0.0197822	0.9462702	0.0239848
ENSBTAG00000010119	0.5470866	0.3058902	0.5144344
ENSBTAG00000010120	-0.011218	0.9683208	0.0139807
ENSBTAG00000010123	0.5059665	0.0971666	1.0124832
ENSBTAG00000010124	0.2232493	0.5146957	0.2884494
ENSBTAG00000010125	-0.06253	0.8188213	0.0868109
ENSBTAG00000010126	0.4757135	0.140405	0.8526174
ENSBTAG00000010127	NA	NA	NA
ENSBTAG00000010128	NA	NA	NA
ENSBTAG00000010129	NA	NA	NA
ENSBTAG00000010130	0.276201	0.372389	0.4290032
ENSBTAG00000010131	0.0993792	0.7787544	0.1085995
ENSBTAG00000010132	-0.364736	0.1788248	0.7475722
ENSBTAG00000010134	0.1445405	0.5913018	0.2281908
ENSBTAG00000010135	-0.127825	0.7159381	0.1451245
ENSBTAG00000010136	-0.108693	0.7033356	0.1528374
ENSBTAG00000010138	-0.001422	0.9982176	0.0007748
ENSBTAG00000010144	-0.146575	0.5756346	0.2398531
ENSBTAG00000010145	NA	NA	NA
ENSBTAG00000010146	NA	NA	NA
ENSBTAG00000010149	0.0997558	0.7354256	0.1334612
ENSBTAG00000010151	NA	NA	NA
ENSBTAG00000010152	0.7667305	0.0761905	1.118099
ENSBTAG00000010153	-0.177277	0.488436	0.3111924
ENSBTAG00000010155	0.4900457	0.3264783	0.4861457
ENSBTAG00000010156	-0.130671	0.6098224	0.2147966
ENSBTAG00000010157	0.0614121	0.812011	0.0904381
ENSBTAG00000010158	0.7233889	0.1177768	0.9289404
ENSBTAG00000010161	0.6311849	0.2499198	0.6021993
ENSBTAG00000010164	NA	NA	NA
ENSBTAG00000010165	-1.002725	0.0213961	1.6696654
ENSBTAG00000010166	-0.394613	0.5937612	0.2263882
ENSBTAG00000010167	NA	NA	NA
ENSBTAG00000010169	-0.050467	0.8487595	0.0712154
ENSBTAG00000010170	0.254441	0.3344777	0.4756329
ENSBTAG00000010171	-0.795921	0.0342265	1.4656379
ENSBTAG00000010174	NA	NA	NA
ENSBTAG00000010175	NA	NA	NA
ENSBTAG00000010176	-0.168057	0.5198957	0.2840838
ENSBTAG00000010177	NA	NA	NA
ENSBTAG00000010178	-0.126855	0.744401	0.128193
ENSBTAG00000010179	0.245406	0.3360625	0.4735799
ENSBTAG00000010180	0.1707075	0.5929039	0.2270157

ENSBTAG00000010181	0.0903758	0.7137257	0.1464687
ENSBTAG00000010182	NA	NA	NA
ENSBTAG00000010184	NA	NA	NA
ENSBTAG00000010185	-0.628991	0.1673922	0.7762649
ENSBTAG00000010188	NA	NA	NA
ENSBTAG00000010191	-0.763321	0.0052784	2.2774978
ENSBTAG00000010192	NA	NA	NA
ENSBTAG00000010193	0.09809	0.7169787	0.1444938
ENSBTAG00000010195	-0.245108	0.5196496	0.2842894
ENSBTAG00000010196	-0.881891	0.0014836	2.8286917
ENSBTAG00000010198	NA	NA	NA
ENSBTAG00000010204	-0.446801	0.0815962	1.08833
ENSBTAG00000010206	-0.115699	0.6495047	0.1874177
ENSBTAG00000010207	0.1648955	0.5299907	0.2757318
ENSBTAG00000010208	0.3874836	0.203778	0.6908428
ENSBTAG00000010210	-0.438301	0.1678869	0.7749833
ENSBTAG00000010215	NA	NA	NA
ENSBTAG00000010217	-0.413124	0.1427165	0.8455258
ENSBTAG00000010219	0.3162322	0.5268446	0.2783175
ENSBTAG00000010220	0.7018725	0.0374352	1.4267197
ENSBTAG00000010221	0.5539137	0.0336381	1.4731679
ENSBTAG00000010223	NA	NA	NA
ENSBTAG00000010225	-0.029771	0.9225712	0.0350001
ENSBTAG00000010227	-0.075178	0.7738783	0.1113273
ENSBTAG00000010228	NA	NA	NA
ENSBTAG00000010229	0.6507857	0.0119108	1.9240577
ENSBTAG00000010230	-0.266979	0.3036984	0.5175574
ENSBTAG00000010232	-0.076217	0.7704853	0.1132356
ENSBTAG00000010234	-0.249645	0.5060427	0.2958129
ENSBTAG00000010235	0.0492818	0.8731527	0.0589098
ENSBTAG00000010236	NA	NA	NA
ENSBTAG00000010238	NA	NA	NA
ENSBTAG00000010239	NA	NA	NA
ENSBTAG00000010241	NA	NA	NA
ENSBTAG00000010242	0.2280169	0.3640019	0.4388964
ENSBTAG00000010243	0.1819769	0.4795153	0.3191976
ENSBTAG00000010244	-0.938474	0.1071957	0.9698225
ENSBTAG00000010245	NA	NA	NA
ENSBTAG00000010246	-0.365438	0.2854513	0.544468
ENSBTAG00000010247	-0.104103	0.7783462	0.1088272
ENSBTAG00000010249	0.2225299	0.5328077	0.2734295
ENSBTAG00000010251	NA	NA	NA
ENSBTAG00000010252	0.393676	0.2801671	0.5525829
ENSBTAG00000010253	NA	NA	NA
ENSBTAG00000010254	0.6815309	0.1744969	0.7582123
ENSBTAG00000010255	0.3456194	0.1983148	0.7026449
ENSBTAG00000010256	-0.161888	0.5148895	0.288286
ENSBTAG00000010264	0.3609381	0.2920646	0.5345211
ENSBTAG00000010265	-0.007779	0.9752231	0.010896
ENSBTAG00000010270	NA	NA	NA
ENSBTAG00000010271	-0.028778	0.9121185	0.0399487
ENSBTAG00000010273	NA	NA	NA
ENSBTAG00000010275	-0.002327	0.9943973	0.0024401
ENSBTAG00000010276	NA	NA	NA
ENSBTAG00000010277	NA	NA	NA
ENSBTAG00000010278	NA	NA	NA
ENSBTAG00000010279	0.410017	0.3585181	0.4454889
ENSBTAG00000010283	NA	NA	NA

ENSBTAG00000010284	0.1461721	0.55675	0.2543398
ENSBTAG00000010285	0.4886032	0.2109985	0.6757206
ENSBTAG00000010286	0.1108645	0.6749173	0.1707494
ENSBTAG00000010289	-0.042386	0.9056304	0.043049
ENSBTAG00000010290	0.2812275	0.27693	0.55763
ENSBTAG00000010291	-0.098027	0.7445418	0.1281109
ENSBTAG00000010292	0.2985866	0.2627184	0.5805095
ENSBTAG00000010293	-0.192923	0.5423711	0.2657034
ENSBTAG00000010297	0.2240143	0.4057488	0.3917427
ENSBTAG00000010298	-0.069301	0.7891766	0.1028258
ENSBTAG00000010300	NA	NA	NA
ENSBTAG00000010303	-0.302124	0.4401132	0.3564356
ENSBTAG00000010304	NA	NA	NA
ENSBTAG00000010306	NA	NA	NA
ENSBTAG00000010309	0.2039008	0.5058098	0.2960127
ENSBTAG00000010311	0.1458259	0.6776409	0.1690004
ENSBTAG00000010312	0.0919219	0.7694663	0.1138104
ENSBTAG00000010313	-0.096318	0.7182227	0.1437409
ENSBTAG00000010316	NA	NA	NA
ENSBTAG00000010318	-0.268106	0.4321959	0.3643194
ENSBTAG00000010321	-0.240829	0.3311479	0.4799779
ENSBTAG00000010322	0.2224659	0.4681373	0.3296268
ENSBTAG00000010324	-0.109699	0.713547	0.1465774
ENSBTAG00000010326	-0.361747	0.3444494	0.4628746
ENSBTAG00000010328	NA	NA	NA
ENSBTAG00000010334	0.3649595	0.4087656	0.3885256
ENSBTAG00000010336	0.1444015	0.5720851	0.2425393
ENSBTAG00000010337	0.5269438	0.1450205	0.8385706
ENSBTAG00000010338	NA	NA	NA
ENSBTAG00000010339	-0.19836	0.6078665	0.2161918
ENSBTAG00000010341	-0.437757	0.1213029	0.9161288
ENSBTAG00000010343	0.6321831	0.1571895	0.8035765
ENSBTAG00000010344	NA	NA	NA
ENSBTAG00000010345	NA	NA	NA
ENSBTAG00000010346	NA	NA	NA
ENSBTAG00000010347	-0.212229	0.4275751	0.3689876
ENSBTAG00000010348	-0.302404	0.316608	0.4994781
ENSBTAG00000010349	NA	NA	NA
ENSBTAG00000010350	-0.239804	0.4459143	0.3507486
ENSBTAG00000010351	0.0749414	0.8209528	0.0856818
ENSBTAG00000010352	NA	NA	NA
ENSBTAG00000010353	1.2606715	0.0073433	2.1341075
ENSBTAG00000010355	-0.104642	0.6853279	0.1641016
ENSBTAG00000010356	0.0991057	0.6994181	0.1552631
ENSBTAG00000010357	0.2387135	0.5882791	0.2304166
ENSBTAG00000010359	0.2715105	0.4249648	0.3716471
ENSBTAG00000010360	-0.264209	0.290832	0.5363577
ENSBTAG00000010361	-0.293994	0.4192436	0.3775336
ENSBTAG00000010362	0.7859217	0.0342838	1.4649105
ENSBTAG00000010363	0.3412419	0.3252509	0.4877815
ENSBTAG00000010365	0.3191415	0.3274165	0.4848994
ENSBTAG00000010366	1.1256757	0.0320474	1.4942067
ENSBTAG00000010367	0.0014227	0.9994122	0.0002554
ENSBTAG00000010368	0.54536	0.1427523	0.8454167
ENSBTAG00000010370	NA	NA	NA
ENSBTAG00000010371	NA	NA	NA
ENSBTAG00000010372	0.1080016	0.8226019	0.0848103
ENSBTAG00000010373	-0.048995	0.875576	0.0577062

ENSBTAG00000010375	0.129946	0.6127961	0.212684
ENSBTAG00000010376	0.0834536	0.7514384	0.1241066
ENSBTAG00000010378	-0.010431	0.9735851	0.0116261
ENSBTAG00000010379	-0.061006	0.8769825	0.0570091
ENSBTAG00000010380	-0.076044	0.766532	0.1154697
ENSBTAG00000010381	-0.02405	0.9323537	0.0304193
ENSBTAG00000010382	NA	NA	NA
ENSBTAG00000010383	0.1168644	0.6705206	0.1735879
ENSBTAG00000010384	NA	NA	NA
ENSBTAG00000010386	-0.31937	0.210609	0.6765231
ENSBTAG00000010387	0.170475	0.5779852	0.2380833
ENSBTAG00000010388	0.033632	0.9166892	0.0377779
ENSBTAG00000010389	-0.615247	0.2210671	0.6554759
ENSBTAG00000010390	0.289983	0.3657931	0.4367645
ENSBTAG00000010392	0.8159238	0.0581985	1.2350878
ENSBTAG00000010393	0.0523276	0.8895271	0.0508408
ENSBTAG00000010394	NA	NA	NA
ENSBTAG00000010395	0.1422708	0.5739647	0.2411148
ENSBTAG00000010397	-0.145534	0.7676075	0.1148608
ENSBTAG00000010399	-0.14549	0.7466194	0.1269007
ENSBTAG00000010401	0.0322091	0.9035876	0.0440298
ENSBTAG00000010402	0.2497878	0.3388529	0.4699888
ENSBTAG00000010403	0.1050886	0.8203389	0.0860067
ENSBTAG00000010407	NA	NA	NA
ENSBTAG00000010408	NA	NA	NA
ENSBTAG00000010411	NA	NA	NA
ENSBTAG00000010413	NA	NA	NA
ENSBTAG00000010414	NA	NA	NA
ENSBTAG00000010416	-0.365264	0.3782614	0.422208
ENSBTAG00000010419	0.4058971	0.4017809	0.3960107
ENSBTAG00000010420	0.7745668	0.0746127	1.1271872
ENSBTAG00000010422	-0.094604	0.7289514	0.1373014
ENSBTAG00000010423	0.0546236	0.8348683	0.078382
ENSBTAG00000010426	0.2619256	0.4099213	0.3872995
ENSBTAG00000010427	-0.203748	0.6633478	0.1782587
ENSBTAG00000010428	-0.417868	0.1020412	0.9912244
ENSBTAG00000010431	1.383696	0.0080464	2.0943983
ENSBTAG00000010432	0.0757703	0.8117042	0.0906022
ENSBTAG00000010433	NA	NA	NA
ENSBTAG00000010437	0.0476373	0.8514055	0.0698635
ENSBTAG00000010439	0.8022428	0.00477	2.3214822
ENSBTAG00000010442	1.0457264	0.0001626	3.7889382
ENSBTAG00000010444	0.7125281	0.1640164	0.7851128
ENSBTAG00000010447	0.1623039	0.5323579	0.2737963
ENSBTAG00000010448	NA	NA	NA
ENSBTAG00000010449	0.1083658	0.6771826	0.1692942
ENSBTAG00000010450	0.3954363	0.3051831	0.5154395
ENSBTAG00000010451	0.0350479	0.8891187	0.0510403
ENSBTAG00000010452	-0.147087	0.7247054	0.1398385
ENSBTAG00000010455	0.6183918	0.0930881	1.031106
ENSBTAG00000010456	-0.025788	0.9311103	0.0309989
ENSBTAG00000010457	-0.373442	0.177244	0.7514285
ENSBTAG00000010458	NA	NA	NA
ENSBTAG00000010460	-0.330002	0.3763182	0.4244448
ENSBTAG00000010462	NA	NA	NA
ENSBTAG00000010463	NA	NA	NA
ENSBTAG00000010464	0.2992716	0.3911286	0.4076804
ENSBTAG00000010465	-0.245231	0.4593753	0.3378323

ENSBTAG00000010467	0.2142166	0.4260134	0.3705768
ENSBTAG00000010470	-0.37967	0.2127083	0.6722156
ENSBTAG00000010472	NA	NA	NA
ENSBTAG00000010475	NA	NA	NA
ENSBTAG00000010477	NA	NA	NA
ENSBTAG00000010478	0.1898036	0.5037317	0.2978007
ENSBTAG00000010480	0.1520329	0.5911443	0.2283065
ENSBTAG00000010481	-0.143674	0.5792285	0.2371501
ENSBTAG00000010482	0.2787177	0.5101388	0.2923116
ENSBTAG00000010484	0.0557691	0.8603483	0.0653257
ENSBTAG00000010485	-0.05273	0.8500061	0.0705779
ENSBTAG00000010487	1.0953884	0.0033657	2.4729298
ENSBTAG00000010490	0.1627325	0.5153327	0.2879123
ENSBTAG00000010492	-0.135654	0.6006921	0.2213481
ENSBTAG00000010493	-0.08999	0.7716026	0.1126063
ENSBTAG00000010496	NA	NA	NA
ENSBTAG00000010497	-0.096244	0.7845786	0.1053636
ENSBTAG00000010498	0.002133	0.9953329	0.0020316
ENSBTAG00000010500	-0.299929	0.2209552	0.6556957
ENSBTAG00000010501	NA	NA	NA
ENSBTAG00000010502	0.2817068	0.3507015	0.4550623
ENSBTAG00000010503	0.2706505	0.4677233	0.330011
ENSBTAG00000010504	0.2207096	0.3823776	0.4175075
ENSBTAG00000010505	-0.021071	0.9451326	0.0245073
ENSBTAG00000010506	0.1403713	0.6201835	0.2074798
ENSBTAG00000010507	-1.20397	0.0044925	2.347511
ENSBTAG00000010508	-0.107088	0.7163452	0.1448777
ENSBTAG00000010509	0.4529006	0.2152954	0.6669653
ENSBTAG00000010510	-0.271294	0.3076357	0.5119633
ENSBTAG00000010511	0.0458495	0.8626101	0.0641855
ENSBTAG00000010513	0.2209675	0.4530399	0.3438635
ENSBTAG00000010514	0.0489316	0.8820663	0.0544988
ENSBTAG00000010515	0.1797663	0.5111668	0.2914374
ENSBTAG00000010517	NA	NA	NA
ENSBTAG00000010518	-0.016563	0.9487259	0.0228592
ENSBTAG00000010519	0.261229	0.369719	0.4321282
ENSBTAG00000010520	0.2523049	0.5062294	0.2956526
ENSBTAG00000010521	NA	NA	NA
ENSBTAG00000010522	NA	NA	NA
ENSBTAG00000010524	0.3734942	0.2531091	0.5966923
ENSBTAG00000010526	-0.212321	0.4089133	0.3883688
ENSBTAG00000010527	-0.415147	0.3850076	0.4145307
ENSBTAG00000010529	0.1707251	0.5592101	0.252425
ENSBTAG00000010531	0.1335418	0.8042775	0.0945941
ENSBTAG00000010532	-0.005831	0.9896763	0.0045068
ENSBTAG00000010533	0.2985943	0.4159958	0.3809111
ENSBTAG00000010534	0.1104714	0.6699624	0.1739496
ENSBTAG00000010535	NA	NA	NA
ENSBTAG00000010536	NA	NA	NA
ENSBTAG00000010541	0.0787438	0.8711178	0.0599231
ENSBTAG00000010542	-0.294639	0.4473834	0.3493201
ENSBTAG00000010543	-0.493407	0.2026151	0.6933282
ENSBTAG00000010545	0.1280629	0.6232615	0.2053297
ENSBTAG00000010546	-0.110552	0.6863515	0.1634534
ENSBTAG00000010547	-0.08281	0.7953381	0.0994482
ENSBTAG00000010548	NA	NA	NA
ENSBTAG00000010549	-0.428115	0.1323418	0.8783029
ENSBTAG00000010550	NA	NA	NA

ENSBTAG00000010551	-0.180937	0.4931143	0.3070524
ENSBTAG00000010552	0.2552214	0.4169051	0.3799628
ENSBTAG00000010555	NA	NA	NA
ENSBTAG00000010559	0.1069432	0.6933794	0.1590291
ENSBTAG00000010562	-0.032262	0.8988815	0.0462976
ENSBTAG00000010563	-0.020033	0.9463113	0.023966
ENSBTAG00000010564	-1.161936	0.0710296	1.1485604
ENSBTAG00000010566	-0.020109	0.9427598	0.0255989
ENSBTAG00000010568	-0.000933	0.9980608	0.000843
ENSBTAG00000010571	-0.820391	0.0014061	2.8519736
ENSBTAG00000010573	NA	NA	NA
ENSBTAG00000010576	0.4154585	0.2569222	0.5901983
ENSBTAG00000010577	-0.620842	0.019047	1.7201739
ENSBTAG00000010578	NA	NA	NA
ENSBTAG00000010579	-0.273514	0.5052478	0.2964956
ENSBTAG00000010581	-0.483095	0.0599458	1.2222413
ENSBTAG00000010582	0.0172769	0.9638566	0.0159876
ENSBTAG00000010584	-0.038167	0.8857673	0.0526804
ENSBTAG00000010587	-0.261953	0.3840511	0.415611
ENSBTAG00000010590	-0.603083	0.1838173	0.7356137
ENSBTAG00000010591	0.2885087	0.4953468	0.3050906
ENSBTAG00000010593	-0.117976	0.6330448	0.1985655
ENSBTAG00000010595	-0.250172	0.4181309	0.3786877
ENSBTAG00000010597	0.0373252	0.949191	0.0226464
ENSBTAG00000010598	-0.02736	0.9164991	0.0378679
ENSBTAG00000010599	-0.142977	0.7036753	0.1526277
ENSBTAG00000010601	0.3577388	0.2876732	0.5411006
ENSBTAG00000010602	0.1472654	0.6195661	0.2079123
ENSBTAG00000010604	-0.173707	0.5462075	0.2626423
ENSBTAG00000010605	NA	NA	NA
ENSBTAG00000010606	-0.357296	0.3579209	0.446213
ENSBTAG00000010609	NA	NA	NA
ENSBTAG00000010610	-0.455693	0.1298578	0.886532
ENSBTAG00000010611	0.0533239	0.8305327	0.0806432
ENSBTAG00000010612	0.1961909	0.4390276	0.3575082
ENSBTAG00000010613	0.2907432	0.3729729	0.4283227
ENSBTAG00000010615	NA	NA	NA
ENSBTAG00000010616	0.0176091	0.9807483	0.0084424
ENSBTAG00000010617	0.0807561	0.7970248	0.0985282
ENSBTAG00000010619	-0.482062	0.0525403	1.2795072
ENSBTAG00000010620	NA	NA	NA
ENSBTAG00000010624	-0.040178	0.8735821	0.0586963
ENSBTAG00000010626	-0.206959	0.4978519	0.3028998
ENSBTAG00000010627	0.1098539	0.6576718	0.1819908
ENSBTAG00000010630	0.1302985	0.6007573	0.221301
ENSBTAG00000010632	NA	NA	NA
ENSBTAG00000010634	NA	NA	NA
ENSBTAG00000010635	-0.227172	0.5930444	0.2269128
ENSBTAG00000010637	1.9924886	3.82E-06	5.4175139
ENSBTAG00000010638	NA	NA	NA
ENSBTAG00000010639	0.1023266	0.7562329	0.1213445
ENSBTAG00000010641	-0.586356	0.0917454	1.0374158
ENSBTAG00000010642	-0.089097	0.7502993	0.1247654
ENSBTAG00000010644	NA	NA	NA
ENSBTAG00000010645	-0.094538	0.706775	0.1507188
ENSBTAG00000010647	-0.035178	0.9226233	0.0349756
ENSBTAG00000010649	-0.643967	0.0465771	1.3318278
ENSBTAG00000010652	0.0570103	0.8523106	0.0694021

ENSBTAG00000010653	0.4615759	0.1323034	0.8784291
ENSBTAG00000010655	NA	NA	NA
ENSBTAG00000010657	NA	NA	NA
ENSBTAG00000010658	0.1906592	0.5536499	0.2567647
ENSBTAG00000010659	-0.011365	0.9659523	0.0150443
ENSBTAG00000010660	-0.049435	0.9213723	0.0355648
ENSBTAG00000010661	0.2346043	0.4086925	0.3886034
ENSBTAG00000010662	-0.3186	0.3542281	0.450717
ENSBTAG00000010663	0.5286401	0.0663055	1.1784502
ENSBTAG00000010664	-0.042774	0.8689671	0.0609966
ENSBTAG00000010665	NA	NA	NA
ENSBTAG00000010666	0.2751348	0.3888907	0.4101725
ENSBTAG00000010667	-0.20184	0.5832808	0.2341223
ENSBTAG00000010668	0.2591079	0.5011777	0.3000083
ENSBTAG00000010670	NA	NA	NA
ENSBTAG00000010671	NA	NA	NA
ENSBTAG00000010672	-0.272753	0.4163808	0.3805093
ENSBTAG00000010673	NA	NA	NA
ENSBTAG00000010676	NA	NA	NA
ENSBTAG00000010677	-0.469436	0.1024032	0.9896864
ENSBTAG00000010679	NA	NA	NA
ENSBTAG00000010681	-0.12229	0.6721719	0.1725197
ENSBTAG00000010682	-0.6547	0.0591561	1.2280001
ENSBTAG00000010686	NA	NA	NA
ENSBTAG00000010689	NA	NA	NA
ENSBTAG00000010691	-0.091284	0.7425658	0.129265
ENSBTAG00000010692	0.213268	0.4025085	0.395225
ENSBTAG00000010693	0.2498522	0.3987455	0.3993042
ENSBTAG00000010694	-0.524517	0.1983436	0.7025819
ENSBTAG00000010696	-0.029724	0.9070403	0.0423734
ENSBTAG00000010698	-0.038841	0.9113833	0.0402989
ENSBTAG00000010701	0.0273949	0.9138156	0.0391414
ENSBTAG00000010704	NA	NA	NA
ENSBTAG00000010709	-0.013944	0.9580277	0.0186219
ENSBTAG00000010711	NA	NA	NA
ENSBTAG00000010715	0.0310081	0.9062869	0.0427343
ENSBTAG00000010716	0.6437273	0.0746969	1.1266975
ENSBTAG00000010717	0.0148255	0.9669303	0.0146048
ENSBTAG00000010718	NA	NA	NA
ENSBTAG00000010719	-0.443269	0.165912	0.7801223
ENSBTAG00000010720	-0.006749	0.9800837	0.0087368
ENSBTAG00000010721	0.2235353	0.58106	0.235779
ENSBTAG00000010723	-0.219941	0.4106392	0.3865396
ENSBTAG00000010726	0.4980565	0.0622655	1.2057522
ENSBTAG00000010727	-0.184126	0.4951842	0.3052332
ENSBTAG00000010728	NA	NA	NA
ENSBTAG00000010729	0.2166219	0.5141272	0.2889294
ENSBTAG00000010730	NA	NA	NA
ENSBTAG00000010731	-0.369608	0.2947855	0.5304939
ENSBTAG00000010732	NA	NA	NA
ENSBTAG00000010734	0.0693045	0.8209279	0.085695
ENSBTAG00000010735	0.2775988	0.3328364	0.4777692
ENSBTAG00000010736	-0.218863	0.5225427	0.2818782
ENSBTAG00000010737	0.3734884	0.2445006	0.6117201
ENSBTAG00000010738	0.4527416	0.239723	0.6202903
ENSBTAG00000010739	-0.148219	0.6263779	0.2031636
ENSBTAG00000010740	0.0720981	0.7749882	0.1107049
ENSBTAG00000010741	-0.11026	0.6650347	0.1771557

ENSBTAG00000010742	NA	NA	NA
ENSBTAG00000010743	0.0051702	0.9858833	0.0061745
ENSBTAG00000010745	-0.107191	0.6692666	0.1744009
ENSBTAG00000010756	-0.287841	0.4294515	0.3670859
ENSBTAG00000010758	NA	NA	NA
ENSBTAG00000010760	0.1110389	0.6971582	0.1566687
ENSBTAG00000010763	-0.105876	0.7445599	0.1281004
ENSBTAG00000010765	0.2371048	0.5575549	0.2537123
ENSBTAG00000010766	NA	NA	NA
ENSBTAG00000010772	-0.050893	0.8905802	0.0503269
ENSBTAG00000010773	-0.187882	0.4430488	0.3535484
ENSBTAG00000010774	NA	NA	NA
ENSBTAG00000010775	-0.179137	0.5395402	0.2679762
ENSBTAG00000010777	-0.129762	0.6406543	0.1933762
ENSBTAG00000010778	-0.066468	0.8050377	0.0941838
ENSBTAG00000010784	-0.000594	0.9989866	0.0004403
ENSBTAG00000010785	0.4847187	0.1927672	0.7149668
ENSBTAG00000010786	0.2074641	0.4308602	0.3656636
ENSBTAG00000010787	0.0092778	0.9836101	0.007177
ENSBTAG00000010788	NA	NA	NA
ENSBTAG00000010789	0.0200845	0.9527966	0.0209998
ENSBTAG00000010790	0.0403687	0.8752037	0.0578909
ENSBTAG00000010792	0.0115317	0.9688407	0.0137476
ENSBTAG00000010793	-0.065616	0.7969925	0.0985458
ENSBTAG00000010795	-0.006673	0.9810719	0.0082992
ENSBTAG00000010798	NA	NA	NA
ENSBTAG00000010799	-0.139704	0.5858018	0.2322493
ENSBTAG00000010801	-0.296286	0.2605531	0.5841038
ENSBTAG00000010802	NA	NA	NA
ENSBTAG00000010803	NA	NA	NA
ENSBTAG00000010805	0.199165	0.5495294	0.2600091
ENSBTAG00000010809	0.7510097	0.0463867	1.3336067
ENSBTAG00000010810	-0.414052	0.3740688	0.4270485
ENSBTAG00000010812	0.23692	0.4957016	0.3047796
ENSBTAG00000010813	NA	NA	NA
ENSBTAG00000010815	0.6014301	0.0835654	1.0779735
ENSBTAG00000010818	-0.392506	0.1356216	0.8676713
ENSBTAG00000010819	-0.669651	0.0481962	1.3169874
ENSBTAG00000010820	0.7163836	0.0390676	1.4081838
ENSBTAG00000010821	NA	NA	NA
ENSBTAG00000010822	NA	NA	NA
ENSBTAG00000010826	-0.352008	0.1672732	0.7765735
ENSBTAG00000010828	NA	NA	NA
ENSBTAG00000010829	NA	NA	NA
ENSBTAG00000010830	0.3022265	0.2458704	0.6092938
ENSBTAG00000010832	0.2656181	0.4183937	0.3784148
ENSBTAG00000010833	NA	NA	NA
ENSBTAG00000010835	0.4302198	0.1812629	0.7416911
ENSBTAG00000010836	NA	NA	NA
ENSBTAG00000010837	NA	NA	NA
ENSBTAG00000010838	0.1746653	0.6073456	0.2165641
ENSBTAG00000010841	0.3183803	0.491105	0.3088256
ENSBTAG00000010843	-0.302891	0.2638418	0.5786563
ENSBTAG00000010846	0.1604899	0.6133611	0.2122838
ENSBTAG00000010847	NA	NA	NA
ENSBTAG00000010849	-0.53685	0.0415508	1.3814208
ENSBTAG00000010850	NA	NA	NA
ENSBTAG00000010852	NA	NA	NA

ENSBTAG00000010856	NA	NA	NA
ENSBTAG00000010858	NA	NA	NA
ENSBTAG00000010859	0.1618228	0.7231156	0.1407923
ENSBTAG00000010863	-0.043464	0.8791677	0.0559283
ENSBTAG00000010866	-0.139804	0.7326824	0.1350842
ENSBTAG00000010867	-0.065865	0.7932709	0.1005785
ENSBTAG00000010868	0.3116918	0.3239718	0.4894928
ENSBTAG00000010871	-0.145048	0.5669878	0.2464263
ENSBTAG00000010874	NA	NA	NA
ENSBTAG00000010875	NA	NA	NA
ENSBTAG00000010877	0.5006526	0.5148201	0.2883445
ENSBTAG00000010878	NA	NA	NA
ENSBTAG00000010880	-0.322347	0.2526676	0.5974504
ENSBTAG00000010881	NA	NA	NA
ENSBTAG00000010882	-0.617621	0.0376581	1.4241413
ENSBTAG00000010884	0.3006525	0.337726	0.4714355
ENSBTAG00000010885	0.005681	0.9829967	0.0074479
ENSBTAG00000010888	0.5081278	0.0632466	1.1989629
ENSBTAG00000010890	0.0073839	0.9779612	0.0096784
ENSBTAG00000010897	NA	NA	NA
ENSBTAG00000010898	0.014693	0.9623089	0.0166855
ENSBTAG00000010899	-0.18645	0.4635338	0.3339186
ENSBTAG00000010904	NA	NA	NA
ENSBTAG00000010906	-0.278699	0.4438466	0.3527671
ENSBTAG00000010907	-0.227762	0.4149552	0.3819988
ENSBTAG00000010909	0.003894	0.9902636	0.0042492
ENSBTAG00000010910	-0.097216	0.7183618	0.1436568
ENSBTAG00000010913	0.6923147	0.1750213	0.7569091
ENSBTAG00000010915	0.7623524	0.0595951	1.2247894
ENSBTAG00000010916	NA	NA	NA
ENSBTAG00000010919	-0.548373	0.0314942	1.5017701
ENSBTAG00000010922	-0.294935	0.4148897	0.3820673
ENSBTAG00000010923	0.3018603	0.3170051	0.4989337
ENSBTAG00000010924	0.318564	0.336734	0.472713
ENSBTAG00000010927	0.2015325	0.6398188	0.193943
ENSBTAG00000010928	-0.04718	0.91311	0.0394769
ENSBTAG00000010931	-0.144866	0.5560577	0.2548801
ENSBTAG00000010932	-0.23536	0.4398358	0.3567094
ENSBTAG00000010934	0.1903335	0.5269717	0.2782127
ENSBTAG00000010935	-0.242016	0.3835847	0.4161388
ENSBTAG00000010937	-0.066322	0.7924222	0.1010434
ENSBTAG00000010939	0.7602514	0.1680853	0.7744702
ENSBTAG00000010940	0.9651051	0.0024948	2.6029669
ENSBTAG00000010943	0.4085266	0.2355079	0.6279944
ENSBTAG00000010944	NA	NA	NA
ENSBTAG00000010945	-0.20619	0.4283503	0.3682009
ENSBTAG00000010947	NA	NA	NA
ENSBTAG00000010948	0.7692489	0.024094	1.6180905
ENSBTAG00000010949	-0.129581	0.7966708	0.0987211
ENSBTAG00000010951	-0.222624	0.5061455	0.2957246
ENSBTAG00000010952	NA	NA	NA
ENSBTAG00000010954	0.3306358	0.2576151	0.5890287
ENSBTAG00000010955	-0.294795	0.3315653	0.4794309
ENSBTAG00000010956	-0.342332	0.1748295	0.7573853
ENSBTAG00000010957	-0.214604	0.5482844	0.2609941
ENSBTAG00000010958	-2.117495	0.0008987	3.0463867
ENSBTAG00000010959	-0.047848	0.8533451	0.0688753
ENSBTAG00000010961	0.0732391	0.8038196	0.0948414

ENSBTAG00000010963	0.292779	0.5350583	0.2715989
ENSBTAG00000010964	NA	NA	NA
ENSBTAG00000010967	-0.829535	0.1135133	0.9449533
ENSBTAG00000010968	-0.039158	0.8827111	0.0541814
ENSBTAG00000010971	-0.102299	0.7156148	0.1453207
ENSBTAG00000010976	-0.393512	0.1830405	0.7374528
ENSBTAG00000010977	-0.117655	0.7138939	0.1463663
ENSBTAG00000010978	0.6058083	0.3167253	0.4993172
ENSBTAG00000010979	NA	NA	NA
ENSBTAG00000010980	0.1256654	0.631077	0.1999177
ENSBTAG00000010981	-0.661773	0.1928796	0.7147138
ENSBTAG00000010982	0.4035598	0.1854982	0.7316604
ENSBTAG00000010984	-0.159939	0.597624	0.223572
ENSBTAG00000010986	NA	NA	NA
ENSBTAG00000010987	-0.173517	0.7059649	0.1512169
ENSBTAG00000010988	-0.067219	0.7882443	0.1033392
ENSBTAG00000010989	0.1462316	0.655759	0.1832557
ENSBTAG00000010990	NA	NA	NA
ENSBTAG00000010991	NA	NA	NA
ENSBTAG00000010992	0.573674	0.0651117	1.186341
ENSBTAG00000010993	-0.021715	0.9551365	0.0199346
ENSBTAG00000010994	0.1249952	0.6437013	0.1913156
ENSBTAG00000010995	0.2200544	0.4527763	0.3441163
ENSBTAG00000010998	0.5454967	0.0560861	1.251145
ENSBTAG00000010999	-0.564808	0.1543681	0.8114424
ENSBTAG00000011000	0.6259393	0.1443331	0.8406339
ENSBTAG00000011001	0.3487042	0.2893825	0.5385277
ENSBTAG00000011002	NA	NA	NA
ENSBTAG00000011003	NA	NA	NA
ENSBTAG00000011007	0.0264287	0.9649241	0.0155069
ENSBTAG00000011010	NA	NA	NA
ENSBTAG00000011011	0.6417266	0.0274272	1.5618179
ENSBTAG00000011012	NA	NA	NA
ENSBTAG00000011014	-0.203848	0.589031	0.2298618
ENSBTAG00000011017	0.2560811	0.5743478	0.240825
ENSBTAG00000011019	NA	NA	NA
ENSBTAG00000011021	NA	NA	NA
ENSBTAG00000011022	-0.308726	0.2217479	0.6541405
ENSBTAG00000011024	0.3072505	0.2747863	0.5610049
ENSBTAG00000011025	-0.05935	0.8194696	0.0864671
ENSBTAG00000011027	0.1856729	0.7154375	0.1454283
ENSBTAG00000011028	0.0866089	0.7524539	0.1235201
ENSBTAG00000011032	0.049429	0.8670177	0.061972
ENSBTAG00000011034	0.0455969	0.9045144	0.0435845
ENSBTAG00000011036	NA	NA	NA
ENSBTAG00000011037	NA	NA	NA
ENSBTAG00000011038	NA	NA	NA
ENSBTAG00000011041	-0.031992	0.9053894	0.0431646
ENSBTAG00000011042	0.8236402	0.0292092	1.5344802
ENSBTAG00000011043	0.2779077	0.5309649	0.2749342
ENSBTAG00000011044	0.1317019	0.7726228	0.1120325
ENSBTAG00000011045	-0.156617	0.5714173	0.2430466
ENSBTAG00000011046	0.0496882	0.8767071	0.0571455
ENSBTAG00000011048	NA	NA	NA
ENSBTAG00000011050	NA	NA	NA
ENSBTAG00000011052	-0.651364	0.0952568	1.0211041
ENSBTAG00000011056	0.3282243	0.4212242	0.3754867
ENSBTAG00000011057	NA	NA	NA

ENSBTAG00000011059	-0.31167	0.2495736	0.6028014
ENSBTAG00000011062	NA	NA	NA
ENSBTAG00000011063	0.666345	0.2418692	0.6164194
ENSBTAG00000011064	0.3608738	0.2716105	0.5660535
ENSBTAG00000011067	0.554284	0.0805123	1.0941378
ENSBTAG00000011068	-0.208547	0.4201528	0.3765927
ENSBTAG00000011070	0.1624562	0.5168766	0.2866131
ENSBTAG00000011071	NA	NA	NA
ENSBTAG00000011072	-0.384189	0.1668029	0.7777964
ENSBTAG00000011074	-0.136144	0.6120478	0.2132146
ENSBTAG00000011075	-0.046297	0.8727416	0.0591143
ENSBTAG00000011076	NA	NA	NA
ENSBTAG00000011079	0.5657278	0.2054098	0.6873789
ENSBTAG00000011080	NA	NA	NA
ENSBTAG00000011081	0.0816754	0.7852729	0.1049794
ENSBTAG00000011082	0.3719058	0.433949	0.3625613
ENSBTAG00000011083	0.4342566	0.2576364	0.5889927
ENSBTAG00000011087	-0.140798	0.5981976	0.2231554
ENSBTAG00000011088	NA	NA	NA
ENSBTAG00000011091	NA	NA	NA
ENSBTAG00000011095	0.2827021	0.4998627	0.3011493
ENSBTAG00000011096	-0.315255	0.2118874	0.6738949
ENSBTAG00000011097	NA	NA	NA
ENSBTAG00000011098	NA	NA	NA
ENSBTAG00000011100	0.0786674	0.8442882	0.0735093
ENSBTAG00000011101	0.4116665	0.3317208	0.4792273
ENSBTAG00000011102	0.02286	0.9472149	0.0235515
ENSBTAG00000011103	0.0809141	0.8443726	0.0734659
ENSBTAG00000011104	-0.586612	0.0347358	1.4592228
ENSBTAG00000011105	-0.142978	0.5934197	0.2266381
ENSBTAG00000011106	NA	NA	NA
ENSBTAG00000011108	-0.043811	0.8733518	0.0588108
ENSBTAG00000011110	NA	NA	NA
ENSBTAG00000011111	-0.039689	0.8932869	0.049009
ENSBTAG00000011112	NA	NA	NA
ENSBTAG00000011115	-0.340372	0.7998106	0.0970128
ENSBTAG00000011116	NA	NA	NA
ENSBTAG00000011120	NA	NA	NA
ENSBTAG00000011121	0.134602	0.5939093	0.2262799
ENSBTAG00000011124	NA	NA	NA
ENSBTAG00000011125	0.1273759	0.7020592	0.1536263
ENSBTAG00000011126	0.0701101	0.8089295	0.0920894
ENSBTAG00000011127	-0.460345	0.0809338	1.0918703
ENSBTAG00000011129	-0.096712	0.7801073	0.1078457
ENSBTAG00000011131	NA	NA	NA
ENSBTAG00000011132	NA	NA	NA
ENSBTAG00000011133	NA	NA	NA
ENSBTAG00000011134	0.1510121	0.6409121	0.1932016
ENSBTAG00000011135	NA	NA	NA
ENSBTAG00000011136	-0.124106	0.6735281	0.1716443
ENSBTAG00000011137	0.112881	0.8317199	0.0800229
ENSBTAG00000011138	-0.04522	0.8799365	0.0555487
ENSBTAG00000011139	-0.116757	0.79767	0.0981767
ENSBTAG00000011140	-0.098616	0.6947154	0.1581931
ENSBTAG00000011143	-0.002901	0.9909964	0.0039279
ENSBTAG00000011145	0.025501	0.9278773	0.0325095
ENSBTAG00000011146	-0.312859	0.2421549	0.6159067
ENSBTAG00000011147	-0.268748	0.294277	0.5312437

ENSBTAG00000011149	0.401466	0.4911331	0.3088008
ENSBTAG00000011150	-0.03492	0.8879915	0.0515912
ENSBTAG00000011153	NA	NA	NA
ENSBTAG00000011154	NA	NA	NA
ENSBTAG00000011155	-0.029697	0.9153938	0.0383921
ENSBTAG00000011156	-0.133221	0.6125526	0.2128566
ENSBTAG00000011162	NA	NA	NA
ENSBTAG00000011170	NA	NA	NA
ENSBTAG00000011171	-0.185426	0.6980489	0.1561142
ENSBTAG00000011172	0.1392006	0.5889817	0.2298982
ENSBTAG00000011173	-0.618852	0.0208837	1.6801922
ENSBTAG00000011176	-0.10322	0.7235839	0.1405111
ENSBTAG00000011178	0.1398934	0.7415419	0.1298643
ENSBTAG00000011179	0.2070515	0.5165007	0.2869291
ENSBTAG00000011180	0.192167	0.5307882	0.2750787
ENSBTAG00000011182	0.1374472	0.596376	0.2244798
ENSBTAG00000011184	0.3653801	0.2101501	0.6774704
ENSBTAG00000011185	-0.456624	0.1558895	0.8071831
ENSBTAG00000011186	NA	NA	NA
ENSBTAG00000011187	-0.347332	0.3785579	0.4218676
ENSBTAG00000011189	0.8013001	0.0191231	1.7184422
ENSBTAG00000011190	0.3157407	0.2157618	0.6660255
ENSBTAG00000011192	NA	NA	NA
ENSBTAG00000011193	0.1152558	0.7933862	0.1005154
ENSBTAG00000011195	NA	NA	NA
ENSBTAG00000011196	0.0533985	0.8928688	0.0492123
ENSBTAG00000011197	0.4223369	0.2517662	0.5990026
ENSBTAG00000011198	-0.231837	0.3582497	0.4458141
ENSBTAG00000011200	0.0729806	0.7965043	0.0988119
ENSBTAG00000011202	0.0855963	0.8040085	0.0947394
ENSBTAG00000011203	NA	NA	NA
ENSBTAG00000011204	-0.023339	0.9650825	0.0154355
ENSBTAG00000011205	NA	NA	NA
ENSBTAG00000011206	-0.450297	0.0841574	1.0749076
ENSBTAG00000011207	-0.104851	0.7216077	0.1416988
ENSBTAG00000011209	0.1292623	0.7681734	0.1145407
ENSBTAG00000011212	-0.107166	0.6730442	0.1719564
ENSBTAG00000011214	-0.162698	0.5964682	0.2244127
ENSBTAG00000011215	0.1170406	0.6432402	0.1916268
ENSBTAG00000011217	0.1854678	0.4882103	0.311393
ENSBTAG00000011224	0.3095561	0.2986161	0.5248868
ENSBTAG00000011225	0.2399277	0.4263366	0.3702474
ENSBTAG00000011226	-0.059922	0.8601089	0.0654466
ENSBTAG00000011227	-0.103663	0.8452386	0.0730207
ENSBTAG00000011228	-0.026089	0.9213267	0.0355863
ENSBTAG00000011229	0.2562677	0.5192151	0.2846527
ENSBTAG00000011234	0.2852124	0.4453185	0.3513292
ENSBTAG00000011236	NA	NA	NA
ENSBTAG00000011237	NA	NA	NA
ENSBTAG00000011238	-0.524565	0.2524281	0.5978622
ENSBTAG00000011239	0.1173608	0.7007944	0.1544094
ENSBTAG00000011241	NA	NA	NA
ENSBTAG00000011242	-0.156055	0.7217819	0.141594
ENSBTAG00000011243	0.4102057	0.230918	0.6365423
ENSBTAG00000011244	0.4335252	0.3944655	0.403991
ENSBTAG00000011245	-0.296673	0.5438916	0.2644876
ENSBTAG00000011246	-1.85468	2.72E-09	8.5649827
ENSBTAG00000011247	0.2986648	0.2745014	0.5614555

ENSBTAG00000011248	-0.094921	0.7685245	0.1143423
ENSBTAG00000011249	0.0022103	0.9938925	0.0026606
ENSBTAG00000011250	-0.121231	0.6286001	0.2016256
ENSBTAG00000011252	-0.441599	0.1426509	0.8457255
ENSBTAG00000011256	0.2816012	0.2898514	0.5378246
ENSBTAG00000011257	-0.492085	0.1096737	0.9598974
ENSBTAG00000011258	-0.061617	0.8398361	0.0758055
ENSBTAG00000011262	0.0871329	0.8403004	0.0755655
ENSBTAG00000011263	0.0800879	0.7867924	0.1041398
ENSBTAG00000011266	-0.065994	0.8442388	0.0735347
ENSBTAG00000011268	-0.254491	0.5207217	0.2833943
ENSBTAG00000011271	NA	NA	NA
ENSBTAG00000011274	NA	NA	NA
ENSBTAG00000011275	NA	NA	NA
ENSBTAG00000011277	NA	NA	NA
ENSBTAG00000011278	0.2394438	0.6104083	0.2143796
ENSBTAG00000011279	NA	NA	NA
ENSBTAG00000011280	-0.106348	0.729965	0.1366979
ENSBTAG00000011283	NA	NA	NA
ENSBTAG00000011284	-0.113696	0.7004906	0.1545977
ENSBTAG00000011285	-0.158646	0.5413937	0.2664868
ENSBTAG00000011287	-0.497096	0.1318398	0.8799534
ENSBTAG00000011291	0.1138478	0.7915179	0.1015393
ENSBTAG00000011292	0.2206812	0.4769127	0.3215611
ENSBTAG00000011298	-0.740411	0.0234364	1.6301086
ENSBTAG00000011304	0.1482552	0.5918792	0.2277669
ENSBTAG00000011305	NA	NA	NA
ENSBTAG00000011307	0.2787883	0.5193846	0.2845109
ENSBTAG00000011310	NA	NA	NA
ENSBTAG00000011311	0.2468586	0.3990326	0.3989917
ENSBTAG00000011312	NA	NA	NA
ENSBTAG00000011313	-0.139521	0.6305467	0.2002828
ENSBTAG00000011315	NA	NA	NA
ENSBTAG00000011316	-0.863123	0.0504398	1.2972269
ENSBTAG00000011317	-0.00114	0.9966743	0.0014468
ENSBTAG00000011319	-0.418539	0.0980323	1.008631
ENSBTAG00000011321	NA	NA	NA
ENSBTAG00000011322	-0.073468	0.7623531	0.1178438
ENSBTAG00000011324	0.1616215	0.5452	0.2634442
ENSBTAG00000011325	0.0592672	0.8951298	0.048114
ENSBTAG00000011327	-0.131515	0.6783369	0.1685545
ENSBTAG00000011328	0.0183017	0.9781063	0.009614
ENSBTAG00000011330	NA	NA	NA
ENSBTAG00000011332	0.6826369	0.0653973	1.1844399
ENSBTAG00000011333	-0.183871	0.4640637	0.3334224
ENSBTAG00000011334	-0.318084	0.2443715	0.6119495
ENSBTAG00000011336	-0.116539	0.7872748	0.1038736
ENSBTAG00000011337	0.0137061	0.9572738	0.0189638
ENSBTAG00000011338	-0.81328	0.0059592	2.2248157
ENSBTAG00000011339	0.5979262	0.0444734	1.3518998
ENSBTAG00000011340	-0.117958	0.744399	0.1281942
ENSBTAG00000011343	NA	NA	NA
ENSBTAG00000011344	0.1369954	0.6768261	0.1695229
ENSBTAG00000011345	-0.247796	0.446941	0.3497498
ENSBTAG00000011349	NA	NA	NA
ENSBTAG00000011350	0.0333957	0.9148713	0.03864
ENSBTAG00000011351	0.0977313	0.6950319	0.1579953
ENSBTAG00000011352	NA	NA	NA

ENSBTAG00000011354	0.032594	0.8987079	0.0463814
ENSBTAG00000011356	NA	NA	NA
ENSBTAG00000011358	-0.847868	0.0077616	2.1100482
ENSBTAG00000011359	NA	NA	NA
ENSBTAG00000011360	0.258518	0.3509818	0.4547153
ENSBTAG00000011363	-0.384658	0.1198945	0.9212006
ENSBTAG00000011366	NA	NA	NA
ENSBTAG00000011367	NA	NA	NA
ENSBTAG00000011368	-0.201088	0.491218	0.3087258
ENSBTAG00000011373	NA	NA	NA
ENSBTAG00000011374	0.1662415	0.7299562	0.1367032
ENSBTAG00000011375	NA	NA	NA
ENSBTAG00000011377	NA	NA	NA
ENSBTAG00000011379	NA	NA	NA
ENSBTAG00000011381	NA	NA	NA
ENSBTAG00000011382	0.0054722	1	0
ENSBTAG00000011383	-0.37516	0.136601	0.8645462
ENSBTAG00000011384	NA	NA	NA
ENSBTAG00000011387	0.1270888	0.6981376	0.156059
ENSBTAG00000011388	-0.092474	0.7119308	0.1475622
ENSBTAG00000011389	0.8141914	0.0773194	1.1117115
ENSBTAG00000011390	-2.142527	0.0084823	2.0714847
ENSBTAG00000011392	-0.436612	0.0867471	1.0617453
ENSBTAG00000011394	-0.423009	0.3408591	0.4674251
ENSBTAG00000011395	-0.320783	0.2249423	0.6479289
ENSBTAG00000011396	-0.363077	0.1605535	0.7943803
ENSBTAG00000011397	0.2681362	0.3269766	0.4854834
ENSBTAG00000011398	NA	NA	NA
ENSBTAG00000011399	-0.187601	0.5894428	0.2295584
ENSBTAG00000011400	-0.272885	0.3656983	0.4368771
ENSBTAG00000011401	-0.099831	0.7249721	0.1396787
ENSBTAG00000011402	NA	NA	NA
ENSBTAG00000011403	-0.440274	0.1239777	0.9066566
ENSBTAG00000011405	NA	NA	NA
ENSBTAG00000011406	0.1529003	0.6677227	0.1754039
ENSBTAG00000011409	NA	NA	NA
ENSBTAG00000011411	NA	NA	NA
ENSBTAG00000011412	0.3087926	0.2366062	0.6259738
ENSBTAG00000011413	-0.366581	0.3689107	0.4330787
ENSBTAG00000011414	0.0407905	0.8728593	0.0590557
ENSBTAG00000011415	-0.146368	0.5793836	0.2370338
ENSBTAG00000011416	NA	NA	NA
ENSBTAG00000011417	0.1154387	0.7236582	0.1404665
ENSBTAG00000011419	-0.044833	0.867444	0.0617586
ENSBTAG00000011420	NA	NA	NA
ENSBTAG00000011421	NA	NA	NA
ENSBTAG00000011423	NA	NA	NA
ENSBTAG00000011424	-0.296036	0.2497174	0.6025512
ENSBTAG00000011425	0.0422803	0.8707068	0.0601281
ENSBTAG00000011427	NA	NA	NA
ENSBTAG00000011429	-0.134091	0.7173334	0.144279
ENSBTAG00000011431	0.1572666	0.5295555	0.2760885
ENSBTAG00000011433	-0.265146	0.41305	0.3839974
ENSBTAG00000011434	0.2306681	0.5912545	0.2282256
ENSBTAG00000011435	-0.334764	0.1726959	0.762718
ENSBTAG00000011437	-0.740539	0.0087432	2.0583288
ENSBTAG00000011442	0.0496061	0.9238341	0.034406
ENSBTAG00000011444	0.0808635	0.7747659	0.1108295

ENSBTAG00000011445	0.3324231	0.4474276	0.3492772
ENSBTAG00000011446	-0.619036	0.0958813	1.0182663
ENSBTAG00000011447	-0.118623	0.7826147	0.106452
ENSBTAG00000011454	0.1587291	0.7312963	0.1359066
ENSBTAG00000011455	0.0450575	0.9284447	0.032244
ENSBTAG00000011456	-0.008834	0.9787566	0.0093253
ENSBTAG00000011458	0.6079258	0.165845	0.7802975
ENSBTAG00000011460	NA	NA	NA
ENSBTAG00000011461	NA	NA	NA
ENSBTAG00000011463	0.3335479	0.3905911	0.4082776
ENSBTAG00000011465	-1.057259	0.0157488	1.8027539
ENSBTAG00000011466	0.1990916	0.5189499	0.2848745
ENSBTAG00000011467	NA	NA	NA
ENSBTAG00000011470	NA	NA	NA
ENSBTAG00000011471	-0.193426	0.4723011	0.3257811
ENSBTAG00000011473	0.3591259	0.3016212	0.5205381
ENSBTAG00000011476	-3.000657	2.62E-05	4.5817435
ENSBTAG00000011478	0.494164	0.2799475	0.5529233
ENSBTAG00000011479	0.2273617	0.615396	0.2108454
ENSBTAG00000011480	-0.342489	0.4288446	0.3677
ENSBTAG00000011481	NA	NA	NA
ENSBTAG00000011482	1.1907209	0.0008366	3.0774925
ENSBTAG00000011483	0.8910086	0.0015641	2.8057458
ENSBTAG00000011484	-0.028758	0.9171774	0.0375467
ENSBTAG00000011487	NA	NA	NA
ENSBTAG00000011488	0.1129887	0.6498704	0.1871733
ENSBTAG00000011489	NA	NA	NA
ENSBTAG00000011490	-1.144621	0.0784766	1.10526
ENSBTAG00000011491	0.6094382	0.0763685	1.1170857
ENSBTAG00000011494	0.3333768	0.2865771	0.5427585
ENSBTAG00000011495	0.2225781	0.4571034	0.3399856
ENSBTAG00000011498	0.1641803	0.5937445	0.2264004
ENSBTAG00000011500	0.1837551	0.6644094	0.1775643
ENSBTAG00000011504	NA	NA	NA
ENSBTAG00000011505	-0.353468	0.1549433	0.8098272
ENSBTAG00000011507	-0.06247	0.83003	0.0809062
ENSBTAG00000011509	0.0940799	0.7575615	0.1205821
ENSBTAG00000011511	0.3518022	0.2703416	0.5680872
ENSBTAG00000011512	-0.074183	0.8306768	0.0805679
ENSBTAG00000011514	-0.055797	0.8331513	0.0792761
ENSBTAG00000011515	0.4561156	0.3325049	0.4782019
ENSBTAG00000011516	-0.506806	0.0822367	1.0849342
ENSBTAG00000011517	-0.010837	0.973574	0.011631
ENSBTAG00000011518	0.7141473	0.0486411	1.312997
ENSBTAG00000011524	-0.285967	0.2876913	0.5410733
ENSBTAG00000011525	0.3778442	0.445234	0.3514117
ENSBTAG00000011527	0.2337787	0.5710484	0.2433271
ENSBTAG00000011528	-0.374001	0.1653922	0.781485
ENSBTAG00000011529	-0.17571	0.5690858	0.2448222
ENSBTAG00000011530	0.1184957	0.7456964	0.127438
ENSBTAG00000011531	0.0274321	0.9200041	0.0362102
ENSBTAG00000011532	0.0420997	0.8677913	0.0615847
ENSBTAG00000011533	NA	NA	NA
ENSBTAG00000011534	-0.538318	0.035798	1.446141
ENSBTAG00000011538	NA	NA	NA
ENSBTAG00000011539	NA	NA	NA
ENSBTAG00000011540	0.3325903	0.3319146	0.4789737
ENSBTAG00000011541	0.6664333	0.0754648	1.1222556

ENSBTAG00000011543	NA	NA	NA
ENSBTAG00000011544	0.0151127	0.9537874	0.0205484
ENSBTAG00000011545	-0.158855	0.6700981	0.1738616
ENSBTAG00000011547	-0.441146	0.1229085	0.9104183
ENSBTAG00000011548	-0.785014	0.0026203	2.5816515
ENSBTAG00000011549	NA	NA	NA
ENSBTAG00000011551	NA	NA	NA
ENSBTAG00000011553	-0.30741	0.250893	0.6005114
ENSBTAG00000011554	-0.000287	1	0
ENSBTAG00000011556	0.1741508	0.6327365	0.1987771
ENSBTAG00000011558	NA	NA	NA
ENSBTAG00000011559	0.0578121	0.8210059	0.0856537
ENSBTAG00000011562	NA	NA	NA
ENSBTAG00000011563	NA	NA	NA
ENSBTAG00000011567	0.5569128	0.0440861	1.355698
ENSBTAG00000011569	NA	NA	NA
ENSBTAG00000011571	0.2908598	0.2399499	0.6198794
ENSBTAG00000011572	NA	NA	NA
ENSBTAG00000011573	NA	NA	NA
ENSBTAG00000011575	0.0036138	0.9912241	0.0038282
ENSBTAG00000011578	-0.474702	0.1437653	0.8423459
ENSBTAG00000011579	NA	NA	NA
ENSBTAG00000011580	-0.200253	0.5004735	0.3006189
ENSBTAG00000011581	NA	NA	NA
ENSBTAG00000011582	-0.12315	0.6900943	0.1610915
ENSBTAG00000011583	0.1535118	0.5621206	0.2501705
ENSBTAG00000011584	0.1874372	0.8301256	0.0808562
ENSBTAG00000011585	0.4948059	0.3361651	0.4734473
ENSBTAG00000011586	0.3121602	0.3959105	0.402403
ENSBTAG00000011587	NA	NA	NA
ENSBTAG00000011588	0.5826715	0.0252147	1.5983463
ENSBTAG00000011589	0.4218212	0.1699114	0.7697775
ENSBTAG00000011590	NA	NA	NA
ENSBTAG00000011591	-0.191159	0.4557582	0.3412655
ENSBTAG00000011593	0.1049161	0.6987528	0.1556764
ENSBTAG00000011595	NA	NA	NA
ENSBTAG00000011596	0.0597306	0.8247669	0.0836688
ENSBTAG00000011597	-0.19256	0.4985571	0.3022851
ENSBTAG00000011598	NA	NA	NA
ENSBTAG00000011600	-0.386306	0.3746437	0.4263815
ENSBTAG00000011601	0.3493058	0.3698459	0.4319792
ENSBTAG00000011602	NA	NA	NA
ENSBTAG00000011608	0.0767028	0.8038518	0.094824
ENSBTAG00000011611	0.0201327	0.9454745	0.0243502
ENSBTAG00000011613	0.3296267	0.2360405	0.6270135
ENSBTAG00000011614	-0.376513	0.2066432	0.6847789
ENSBTAG00000011617	0.1435634	0.7028996	0.1531067
ENSBTAG00000011619	-0.205387	0.4604612	0.3368069
ENSBTAG00000011620	0.2633059	0.5078678	0.2942493
ENSBTAG00000011621	NA	NA	NA
ENSBTAG00000011622	-0.136874	0.6928126	0.1593842
ENSBTAG00000011623	0.1295629	0.6475017	0.1887591
ENSBTAG00000011625	0.3049974	0.4418049	0.3547695
ENSBTAG00000011626	-0.574104	0.0315518	1.5009762
ENSBTAG00000011628	0.3331934	0.2359038	0.6272651
ENSBTAG00000011632	0.3867951	0.1170948	0.9314624
ENSBTAG00000011633	NA	NA	NA
ENSBTAG00000011634	NA	NA	NA

ENSBTAG00000011635	NA	NA	NA
ENSBTAG00000011636	NA	NA	NA
ENSBTAG00000011638	NA	NA	NA
ENSBTAG00000011639	-0.304924	0.2431209	0.6141777
ENSBTAG00000011640	0.0089094	0.9738232	0.0115199
ENSBTAG00000011642	-0.160152	0.5369227	0.2700882
ENSBTAG00000011643	0.3772148	0.1447134	0.8394912
ENSBTAG00000011644	-0.146991	0.773956	0.1112837
ENSBTAG00000011645	-0.119706	0.6471436	0.1889994
ENSBTAG00000011646	-0.061126	0.8703165	0.0603228
ENSBTAG00000011647	0.2800648	0.4066082	0.3908238
ENSBTAG00000011648	-0.03582	0.9079163	0.0419542
ENSBTAG00000011649	-0.120094	0.6276306	0.2022959
ENSBTAG00000011650	NA	NA	NA
ENSBTAG00000011651	-0.584226	0.186404	0.7295449
ENSBTAG00000011654	NA	NA	NA
ENSBTAG00000011655	NA	NA	NA
ENSBTAG00000011656	0.2285601	0.3659522	0.4365757
ENSBTAG00000011657	NA	NA	NA
ENSBTAG00000011658	0.202349	0.6840044	0.1649411
ENSBTAG00000011659	-0.246185	0.5016641	0.299587
ENSBTAG00000011661	0.7351567	0.1040937	0.9825754
ENSBTAG00000011662	-0.832884	0.113538	0.9448589
ENSBTAG00000011664	NA	NA	NA
ENSBTAG00000011666	-1.064163	0.1317015	0.8804094
ENSBTAG00000011667	-0.366306	0.3731824	0.4280789
ENSBTAG00000011668	NA	NA	NA
ENSBTAG00000011672	NA	NA	NA
ENSBTAG00000011677	-0.137166	0.6818181	0.1663315
ENSBTAG00000011680	0.2585985	0.5977791	0.2234592
ENSBTAG00000011682	NA	NA	NA
ENSBTAG00000011683	-0.068739	0.8186381	0.086908
ENSBTAG00000011684	0.517633	0.2766149	0.5581244
ENSBTAG00000011689	0.4913258	0.0868424	1.0612683
ENSBTAG00000011692	NA	NA	NA
ENSBTAG00000011694	-0.022398	0.9361961	0.0286332
ENSBTAG00000011698	0.0726213	0.8251173	0.0834843
ENSBTAG00000011699	-0.143	0.6016732	0.2206393
ENSBTAG00000011700	NA	NA	NA
ENSBTAG00000011702	NA	NA	NA
ENSBTAG00000011704	NA	NA	NA
ENSBTAG00000011706	NA	NA	NA
ENSBTAG00000011709	-0.210755	0.5123837	0.2904047
ENSBTAG00000011713	NA	NA	NA
ENSBTAG00000011715	0.3373877	0.4131863	0.3838541
ENSBTAG00000011717	0.1047126	0.688765	0.1619289
ENSBTAG00000011720	NA	NA	NA
ENSBTAG00000011721	-0.094532	0.7129957	0.1469131
ENSBTAG00000011723	NA	NA	NA
ENSBTAG00000011726	0.4949598	0.0826078	1.0829791
ENSBTAG00000011727	-0.317653	0.2110985	0.6755149
ENSBTAG00000011729	0.1479771	0.6651171	0.1771019
ENSBTAG00000011730	0.4599184	0.1443402	0.8406127
ENSBTAG00000011731	1.7625087	0.0001533	3.8143587
ENSBTAG00000011732	0.1857438	0.5436504	0.2646803
ENSBTAG00000011733	0.0168452	0.9600986	0.0176841
ENSBTAG00000011734	0.3136121	0.4463199	0.3503538
ENSBTAG00000011736	NA	NA	NA

ENSBTAG00000011738	NA	NA	NA
ENSBTAG00000011740	NA	NA	NA
ENSBTAG00000011741	NA	NA	NA
ENSBTAG00000011742	-0.937646	0.0130422	1.8846499
ENSBTAG00000011743	NA	NA	NA
ENSBTAG00000011744	-0.160311	0.6933752	0.1590317
ENSBTAG00000011748	-0.517673	0.0918932	1.0367168
ENSBTAG00000011752	-0.367833	0.1605934	0.7942722
ENSBTAG00000011753	0.1682247	0.5502642	0.2594287
ENSBTAG00000011754	-0.036494	0.8810362	0.0550062
ENSBTAG00000011756	NA	NA	NA
ENSBTAG00000011757	0.1115818	0.6728328	0.1720929
ENSBTAG00000011758	-1.213166	0.0114805	1.9400374
ENSBTAG00000011759	NA	NA	NA
ENSBTAG00000011760	-0.127076	0.6193287	0.2080788
ENSBTAG00000011761	-0.303376	0.300997	0.5214378
ENSBTAG00000011762	0.5548711	0.0716065	1.1450473
ENSBTAG00000011763	0.1113241	0.6789209	0.1681808
ENSBTAG00000011765	0.8485542	0.0055267	2.2575329
ENSBTAG00000011766	0.9523104	0.0008682	3.0613792
ENSBTAG00000011767	-0.241191	0.4016049	0.396201
ENSBTAG00000011770	0.4579318	0.1446983	0.8395365
ENSBTAG00000011771	0.019305	0.9619131	0.0168642
ENSBTAG00000011772	0.3875538	0.2067907	0.684469
ENSBTAG00000011779	NA	NA	NA
ENSBTAG00000011780	NA	NA	NA
ENSBTAG00000011782	NA	NA	NA
ENSBTAG00000011784	-0.672729	0.1614082	0.7920745
ENSBTAG00000011785	0.0016173	0.9954696	0.001972
ENSBTAG00000011786	0.4092088	0.1108049	0.955441
ENSBTAG00000011787	0.2533144	0.311638	0.5063496
ENSBTAG00000011788	-0.397099	0.2466669	0.6078891
ENSBTAG00000011789	-0.271586	0.3528856	0.4523661
ENSBTAG00000011790	-0.351942	0.216508	0.664526
ENSBTAG00000011793	-0.167683	0.5702734	0.2439169
ENSBTAG00000011795	0.4138996	0.3492683	0.4568408
ENSBTAG00000011796	0.0516385	0.8641717	0.0634
ENSBTAG00000011798	-0.011981	0.96691	0.014614
ENSBTAG00000011800	NA	NA	NA
ENSBTAG00000011802	0.2405476	0.3811785	0.4188715
ENSBTAG00000011803	-3.20879	4.42E-06	5.3541829
ENSBTAG00000011804	NA	NA	NA
ENSBTAG00000011805	0.3048667	0.3154259	0.5011027
ENSBTAG00000011808	-2.216671	0.000842	3.0747106
ENSBTAG00000011809	0.2933162	0.3893963	0.4096082
ENSBTAG00000011810	-1.19746	0.000233	3.6326031
ENSBTAG00000011811	0.1956106	0.4657613	0.3318366
ENSBTAG00000011812	-0.171287	0.5204944	0.2835839
ENSBTAG00000011814	NA	NA	NA
ENSBTAG00000011815	0.736229	0.0593527	1.2265595
ENSBTAG00000011818	NA	NA	NA
ENSBTAG00000011819	-0.048333	0.8608128	0.0650913
ENSBTAG00000011820	0.0301954	0.9376682	0.0279508
ENSBTAG00000011822	NA	NA	NA
ENSBTAG00000011823	0.3248034	0.2023033	0.6939971
ENSBTAG00000011824	0.147345	0.5814691	0.2354733
ENSBTAG00000011825	0.0327936	0.9034548	0.0440936
ENSBTAG00000011826	0.3865646	0.2678468	0.5721136

ENSBTAG00000011828	0.0421944	0.9369868	0.0282665
ENSBTAG00000011829	0.0207216	0.9347587	0.0293005
ENSBTAG00000011831	-0.135632	0.6051035	0.2181703
ENSBTAG00000011832	-0.205287	0.4565938	0.34047
ENSBTAG00000011833	0.5180977	0.1626606	0.7887176
ENSBTAG00000011834	-0.106743	0.674586	0.1709627
ENSBTAG00000011836	NA	NA	NA
ENSBTAG00000011837	0.0085783	0.9749697	0.0110089
ENSBTAG00000011838	NA	NA	NA
ENSBTAG00000011839	-0.096192	0.7165836	0.1447331
ENSBTAG00000011841	-0.411758	0.389119	0.4099175
ENSBTAG00000011842	0.4134813	0.2006382	0.6975863
ENSBTAG00000011843	0.1551681	0.5410944	0.2667269
ENSBTAG00000011844	NA	NA	NA
ENSBTAG00000011846	0.3399592	0.2820892	0.5496135
ENSBTAG00000011847	0.1213799	0.7184684	0.1435923
ENSBTAG00000011849	-0.43119	0.1347533	0.8704606
ENSBTAG00000011850	-0.036648	0.9174445	0.0374202
ENSBTAG00000011851	0.1400543	0.6384355	0.194883
ENSBTAG00000011854	NA	NA	NA
ENSBTAG00000011855	0.1158557	0.7309962	0.1360849
ENSBTAG00000011857	NA	NA	NA
ENSBTAG00000011859	0.7636411	0.0360435	1.4431733
ENSBTAG00000011860	-0.236687	0.4154399	0.3814918
ENSBTAG00000011861	NA	NA	NA
ENSBTAG00000011862	NA	NA	NA
ENSBTAG00000011864	-0.631702	0.0324331	1.489011
ENSBTAG00000011865	NA	NA	NA
ENSBTAG00000011866	-0.250397	0.748107	0.1260363
ENSBTAG00000011869	1.1647077	0.0111706	1.9519238
ENSBTAG00000011872	0.3342001	0.2159814	0.6655837
ENSBTAG00000011873	NA	NA	NA
ENSBTAG00000011875	0.6163198	0.0984478	1.0067938
ENSBTAG00000011876	0.1906456	0.465198	0.3323622
ENSBTAG00000011879	-0.078939	0.7968934	0.0985998
ENSBTAG00000011880	NA	NA	NA
ENSBTAG00000011881	NA	NA	NA
ENSBTAG00000011882	NA	NA	NA
ENSBTAG00000011883	-0.046985	0.8592047	0.0659034
ENSBTAG00000011885	-0.006971	0.9794139	0.0090337
ENSBTAG00000011887	NA	NA	NA
ENSBTAG00000011888	NA	NA	NA
ENSBTAG00000011889	0.0021137	0.9987433	0.0005461
ENSBTAG00000011892	0.507081	0.2643127	0.5778821
ENSBTAG00000011893	-0.064332	0.8076194	0.0927933
ENSBTAG00000011894	NA	NA	NA
ENSBTAG00000011895	-0.109652	0.6864257	0.1634064
ENSBTAG00000011896	-0.817792	0.006638	2.1779612
ENSBTAG00000011898	0.0164342	0.9656293	0.0151896
ENSBTAG00000011899	0.1385549	0.6067549	0.2169867
ENSBTAG00000011902	-0.073986	0.8148574	0.0889184
ENSBTAG00000011904	0.4399485	0.1206073	0.9186264
ENSBTAG00000011905	0.3155245	0.4402459	0.3563047
ENSBTAG00000011908	-0.280772	0.580653	0.2360833
ENSBTAG00000011909	-0.197076	0.54758	0.2615524
ENSBTAG00000011910	NA	NA	NA
ENSBTAG00000011911	NA	NA	NA
ENSBTAG00000011912	-0.073413	0.8512738	0.0699308

ENSBTAG00000011913	0.499944	0.1982552	0.7027754
ENSBTAG00000011916	0.1982504	0.4532215	0.3436895
ENSBTAG00000011917	-0.244756	0.3616445	0.4417182
ENSBTAG00000011918	0.153919	0.5440694	0.2643457
ENSBTAG00000011921	NA	NA	NA
ENSBTAG00000011922	0.2496272	0.3949223	0.4034883
ENSBTAG00000011926	-0.154607	0.5939431	0.2262552
ENSBTAG00000011927	NA	NA	NA
ENSBTAG00000011928	-0.451727	0.1671979	0.7767692
ENSBTAG00000011929	NA	NA	NA
ENSBTAG00000011930	-0.245846	0.3502125	0.4556683
ENSBTAG00000011931	0.197708	0.4445253	0.3521035
ENSBTAG00000011932	NA	NA	NA
ENSBTAG00000011933	-1.061431	0.0353239	1.451931
ENSBTAG00000011934	1.0366583	0.1299319	0.8862843
ENSBTAG00000011936	0.1519218	0.6805195	0.1671594
ENSBTAG00000011937	0.2910256	0.4454262	0.3512242
ENSBTAG00000011940	NA	NA	NA
ENSBTAG00000011943	-0.113248	0.6553969	0.1834956
ENSBTAG00000011944	NA	NA	NA
ENSBTAG00000011945	-0.053961	0.8347238	0.0784572
ENSBTAG00000011946	-0.140843	0.633935	0.1979552
ENSBTAG00000011950	0.1169148	0.6640702	0.177786
ENSBTAG00000011951	0.1456173	0.6310205	0.1999565
ENSBTAG00000011953	-0.551743	0.0361138	1.4423269
ENSBTAG00000011954	0.1156972	0.7230284	0.1408446
ENSBTAG00000011957	0.0966565	0.7332653	0.1347389
ENSBTAG00000011959	-0.692948	0.1714078	0.7659695
ENSBTAG00000011960	0.4234432	0.1901502	0.7209033
ENSBTAG00000011961	NA	NA	NA
ENSBTAG00000011963	0.1730818	0.4914	0.3085648
ENSBTAG00000011964	0.0583916	0.8554014	0.06783
ENSBTAG00000011966	0.4481153	0.0712445	1.1472486
ENSBTAG00000011969	1.0361449	0.007242	2.1401432
ENSBTAG00000011970	-0.177815	0.6260611	0.2033833
ENSBTAG00000011971	0.4597469	0.1501034	0.8236094
ENSBTAG00000011973	NA	NA	NA
ENSBTAG00000011975	-0.34904	0.3707512	0.4309174
ENSBTAG00000011976	1.4589225	0.0163763	1.7857836
ENSBTAG00000011981	NA	NA	NA
ENSBTAG00000011982	NA	NA	NA
ENSBTAG00000011984	NA	NA	NA
ENSBTAG00000011986	0.0275544	0.9366336	0.0284303
ENSBTAG00000011987	-0.233822	0.4422671	0.3543154
ENSBTAG00000011988	-0.024165	0.9300647	0.0314868
ENSBTAG00000011990	NA	NA	NA
ENSBTAG00000011991	NA	NA	NA
ENSBTAG00000011992	NA	NA	NA
ENSBTAG00000011994	0.0743318	0.8004925	0.0966427
ENSBTAG00000011997	0.2725843	0.4254868	0.3711139
ENSBTAG00000011998	-0.317872	0.2487363	0.6042608
ENSBTAG00000012002	0.3293698	0.3967038	0.4015337
ENSBTAG00000012003	-0.010629	0.969349	0.0135198
ENSBTAG00000012004	0.0270549	0.9426604	0.0256448
ENSBTAG00000012005	0.250693	0.5712618	0.2431648
ENSBTAG00000012007	-1.79999	0.000512	3.2907716
ENSBTAG00000012010	-0.100361	0.748472	0.1258245
ENSBTAG00000012012	0.4086045	0.2731339	0.5636244

ENSBTAG00000012016	NA	NA	NA
ENSBTAG00000012019	0.4167374	0.1549002	0.8099479
ENSBTAG00000012020	-0.360574	0.2964988	0.5279771
ENSBTAG00000012022	0.3569069	0.3541088	0.4508633
ENSBTAG00000012024	0.6104445	0.1253568	0.9018522
ENSBTAG00000012025	NA	NA	NA
ENSBTAG00000012026	0.4641077	0.3293881	0.482292
ENSBTAG00000012029	NA	NA	NA
ENSBTAG00000012030	-0.54569	0.1552692	0.8089146
ENSBTAG00000012031	NA	NA	NA
ENSBTAG00000012032	0.3978178	0.2277402	0.6425604
ENSBTAG00000012034	NA	NA	NA
ENSBTAG00000012035	NA	NA	NA
ENSBTAG00000012036	0.5492014	0.233945	0.6308862
ENSBTAG00000012037	-0.354348	0.2216069	0.6544168
ENSBTAG00000012039	0.5236006	0.2177	0.6621416
ENSBTAG00000012040	0.2124448	0.5302844	0.2754912
ENSBTAG00000012041	0.1850222	0.4717016	0.3263326
ENSBTAG00000012044	0.0007331	0.9978765	0.0009232
ENSBTAG00000012046	0.0709846	0.8357825	0.0779067
ENSBTAG00000012047	-0.280267	0.2670309	0.5734384
ENSBTAG00000012048	0.0422799	0.8703352	0.0603134
ENSBTAG00000012049	-0.02052	0.9451195	0.0245133
ENSBTAG00000012050	NA	NA	NA
ENSBTAG00000012052	NA	NA	NA
ENSBTAG00000012053	NA	NA	NA
ENSBTAG00000012058	-0.239545	0.4916588	0.3083362
ENSBTAG00000012059	-0.519612	0.0648176	1.1883069
ENSBTAG00000012060	0.7565225	0.1024529	0.9894759
ENSBTAG00000012061	NA	NA	NA
ENSBTAG00000012062	0.0487132	0.8527855	0.0691602
ENSBTAG00000012063	0.1549312	0.6116371	0.2135062
ENSBTAG00000012064	-0.256541	0.5328191	0.2734202
ENSBTAG00000012065	-0.172275	0.5676473	0.2459214
ENSBTAG00000012066	0.5298878	0.039581	1.402513
ENSBTAG00000012067	0.1640244	0.5103689	0.2921158
ENSBTAG00000012068	NA	NA	NA
ENSBTAG00000012069	-0.588871	0.1570918	0.8038465
ENSBTAG00000012070	-0.214969	0.4565067	0.3405528
ENSBTAG00000012071	NA	NA	NA
ENSBTAG00000012072	-0.047994	0.8565331	0.0672558
ENSBTAG00000012073	1.0071511	0.0111261	1.9536582
ENSBTAG00000012074	NA	NA	NA
ENSBTAG00000012077	-0.078618	0.7685264	0.1143412
ENSBTAG00000012078	-0.337163	0.4993057	0.3016335
ENSBTAG00000012079	0.013171	0.98395	0.007027
ENSBTAG00000012081	-0.169007	0.5788893	0.2374044
ENSBTAG00000012082	0.3774318	0.3901125	0.4088101
ENSBTAG00000012083	NA	NA	NA
ENSBTAG00000012086	0.4691399	0.0965201	1.0153824
ENSBTAG00000012087	0.501457	0.4069815	0.3904253
ENSBTAG00000012088	0.0589011	0.8620464	0.0644693
ENSBTAG00000012090	0.0657547	0.7934312	0.1004907
ENSBTAG00000012094	-0.058803	0.8208024	0.0857614
ENSBTAG00000012095	0.1390625	0.6415867	0.1927447
ENSBTAG00000012096	-0.332077	0.2376147	0.6241267
ENSBTAG00000012097	NA	NA	NA
ENSBTAG00000012099	-0.002414	0.9936568	0.0027636

ENSBTAG00000012100	0.5012457	0.1214367	0.9156502
ENSBTAG00000012101	0.2333559	0.428547	0.3680015
ENSBTAG00000012102	0.0118994	0.9637601	0.0160311
ENSBTAG00000012103	0.0179099	0.9489062	0.0227767
ENSBTAG00000012104	NA	NA	NA
ENSBTAG00000012106	0.1861349	0.6864026	0.1634211
ENSBTAG00000012107	0.1272205	0.6660195	0.1765131
ENSBTAG00000012109	NA	NA	NA
ENSBTAG00000012111	-0.221017	0.6176476	0.2092592
ENSBTAG00000012112	0.5227504	0.1485761	0.8280511
ENSBTAG00000012113	0.1691383	0.5257431	0.2792265
ENSBTAG00000012116	NA	NA	NA
ENSBTAG00000012117	0.1247758	0.6274857	0.2023961
ENSBTAG00000012119	NA	NA	NA
ENSBTAG00000012120	-1.31688	0.0039196	2.4067569
ENSBTAG00000012121	NA	NA	NA
ENSBTAG00000012124	-0.146904	0.5581254	0.2532682
ENSBTAG00000012125	0.2467584	0.4303033	0.3662253
ENSBTAG00000012126	0.3019231	0.3873253	0.4119241
ENSBTAG00000012128	-0.637356	0.0994306	1.0024799
ENSBTAG00000012135	-0.111274	0.6987851	0.1556563
ENSBTAG00000012137	-0.011031	0.9863593	0.0059649
ENSBTAG00000012139	0.2379779	0.3981848	0.3999154
ENSBTAG00000012140	NA	NA	NA
ENSBTAG00000012141	0.1865945	0.7086271	0.1495823
ENSBTAG00000012142	0.4855605	0.2823624	0.5491931
ENSBTAG00000012143	0.3284649	0.3340691	0.4761637
ENSBTAG00000012144	-0.254033	0.4096988	0.3875353
ENSBTAG00000012146	0.0080928	0.9742131	0.011346
ENSBTAG00000012147	-0.199402	0.6021796	0.2202739
ENSBTAG00000012148	0.218526	0.4046298	0.3929422
ENSBTAG00000012149	-0.379325	0.3047785	0.5160156
ENSBTAG00000012150	NA	NA	NA
ENSBTAG00000012152	0.1219998	0.6193118	0.2080906
ENSBTAG00000012156	-0.139716	0.7250614	0.1396252
ENSBTAG00000012157	NA	NA	NA
ENSBTAG00000012159	NA	NA	NA
ENSBTAG00000012163	-0.522802	0.060464	1.2185033
ENSBTAG00000012164	NA	NA	NA
ENSBTAG00000012168	-0.060607	0.8373905	0.077072
ENSBTAG00000012169	0.2836634	0.4969551	0.3036828
ENSBTAG00000012170	-0.160102	0.5242254	0.280482
ENSBTAG00000012172	-0.058731	0.8361507	0.0777154
ENSBTAG00000012176	NA	NA	NA
ENSBTAG00000012177	0.0650332	0.8011791	0.0962704
ENSBTAG00000012178	0.4769084	0.1581502	0.8009304
ENSBTAG00000012180	1.1379876	0.0038178	2.4181822
ENSBTAG00000012181	0.1212789	0.6744333	0.171061
ENSBTAG00000012182	NA	NA	NA
ENSBTAG00000012184	NA	NA	NA
ENSBTAG00000012185	NA	NA	NA
ENSBTAG00000012186	NA	NA	NA
ENSBTAG00000012189	NA	NA	NA
ENSBTAG00000012191	-0.175946	0.6038721	0.219055
ENSBTAG00000012192	NA	NA	NA
ENSBTAG00000012193	-0.151347	0.5902517	0.2289627
ENSBTAG00000012194	0.1834655	0.6917381	0.1600583
ENSBTAG00000012197	0.5172578	0.1517733	0.8188046

ENSBTAG00000012200	0.2173583	0.6162829	0.2102199
ENSBTAG00000012201	NA	NA	NA
ENSBTAG00000012205	NA	NA	NA
ENSBTAG00000012206	0.4529886	0.2678124	0.5721694
ENSBTAG00000012208	-0.128222	0.8726667	0.0591516
ENSBTAG00000012210	NA	NA	NA
ENSBTAG00000012211	NA	NA	NA
ENSBTAG00000012212	NA	NA	NA
ENSBTAG00000012213	0.0218613	0.9291719	0.031904
ENSBTAG00000012215	NA	NA	NA
ENSBTAG00000012216	NA	NA	NA
ENSBTAG00000012219	0.4903499	0.2102337	0.6772977
ENSBTAG00000012222	NA	NA	NA
ENSBTAG00000012223	NA	NA	NA
ENSBTAG00000012225	0.0870122	0.7906318	0.1020257
ENSBTAG00000012228	NA	NA	NA
ENSBTAG00000012232	-0.000807	0.9976064	0.0010408
ENSBTAG00000012234	NA	NA	NA
ENSBTAG00000012235	0.1288103	0.6750992	0.1706324
ENSBTAG00000012237	0.3227921	0.5106493	0.2918773
ENSBTAG00000012239	-0.067316	0.858179	0.0664221
ENSBTAG00000012241	-0.153391	0.6488257	0.187872
ENSBTAG00000012242	-0.073108	0.7691631	0.1139815
ENSBTAG00000012243	0.1277382	0.7130146	0.1469016
ENSBTAG00000012244	-0.022159	0.9363857	0.0285452
ENSBTAG00000012246	NA	NA	NA
ENSBTAG00000012247	-0.374828	0.3330438	0.4774986
ENSBTAG00000012249	NA	NA	NA
ENSBTAG00000012250	-0.219661	0.4305845	0.3659417
ENSBTAG00000012251	-0.041515	0.8737209	0.0586273
ENSBTAG00000012252	-0.002956	0.9975285	0.0010747
ENSBTAG00000012253	-0.121289	0.6382298	0.1950229
ENSBTAG00000012254	NA	NA	NA
ENSBTAG00000012259	0.1117631	0.7836316	0.1058881
ENSBTAG00000012260	0.0457019	0.8711275	0.0599183
ENSBTAG00000012261	NA	NA	NA
ENSBTAG00000012262	1.9852017	1.56E-05	4.8065025
ENSBTAG00000012263	-0.564994	0.1339096	0.8731882
ENSBTAG00000012265	0.5955039	0.057371	1.2413078
ENSBTAG00000012267	-0.093566	0.7166798	0.1446748
ENSBTAG00000012271	0.0146087	0.960966	0.017292
ENSBTAG00000012272	0.1421635	0.5622604	0.2500625
ENSBTAG00000012273	-0.033565	0.9251747	0.0337763
ENSBTAG00000012274	0.1133064	0.7481983	0.1259833
ENSBTAG00000012275	NA	NA	NA
ENSBTAG00000012276	0.4672854	0.4547909	0.3421882
ENSBTAG00000012277	-0.070333	0.885384	0.0528683
ENSBTAG00000012278	-0.286374	0.3987276	0.3993237
ENSBTAG00000012279	-0.107382	0.7283052	0.1376866
ENSBTAG00000012280	NA	NA	NA
ENSBTAG00000012284	NA	NA	NA
ENSBTAG00000012285	0.7783198	0.063411	1.1978356
ENSBTAG00000012288	0.2828645	0.3125818	0.5050363
ENSBTAG00000012289	-0.171481	0.6359895	0.19655
ENSBTAG00000012290	NA	NA	NA
ENSBTAG00000012291	0.2573189	0.424241	0.3723874
ENSBTAG00000012293	-0.364846	0.1412887	0.8498924
ENSBTAG00000012295	NA	NA	NA

ENSBTAG00000012297	0.8125172	0.0397271	1.4009135
ENSBTAG00000012299	-0.201796	0.461107	0.3361983
ENSBTAG00000012302	NA	NA	NA
ENSBTAG00000012305	0.0735384	0.7758127	0.1102431
ENSBTAG00000012307	-0.215319	0.3955231	0.4028282
ENSBTAG00000012311	NA	NA	NA
ENSBTAG00000012312	NA	NA	NA
ENSBTAG00000012314	-0.274667	0.5942803	0.2260087
ENSBTAG00000012317	0.6885136	0.0054878	2.2606026
ENSBTAG00000012319	NA	NA	NA
ENSBTAG00000012321	-0.106537	0.677602	0.1690253
ENSBTAG00000012322	-0.289427	0.4677064	0.3300267
ENSBTAG00000012325	NA	NA	NA
ENSBTAG00000012326	NA	NA	NA
ENSBTAG00000012328	NA	NA	NA
ENSBTAG00000012330	0.0495991	0.8553404	0.067861
ENSBTAG00000012332	0.0021225	0.9960667	0.0017116
ENSBTAG00000012334	NA	NA	NA
ENSBTAG00000012335	0.625722	0.1113345	0.9533701
ENSBTAG00000012338	-0.201135	0.6303025	0.200451
ENSBTAG00000012341	-0.170221	0.4901492	0.3096717
ENSBTAG00000012342	-0.009961	0.9775238	0.0098727
ENSBTAG00000012343	0.9147623	0.0563138	1.2493849
ENSBTAG00000012344	0.0633767	0.8132557	0.0897729
ENSBTAG00000012347	NA	NA	NA
ENSBTAG00000012348	NA	NA	NA
ENSBTAG00000012349	NA	NA	NA
ENSBTAG00000012350	0.0889006	0.7771529	0.1094935
ENSBTAG00000012351	NA	NA	NA
ENSBTAG00000012352	0.7031085	0.1157934	0.9363161
ENSBTAG00000012353	-0.509362	0.1043306	0.9815882
ENSBTAG00000012355	-0.30336	0.2237232	0.6502889
ENSBTAG00000012357	-0.074156	0.7916329	0.1014762
ENSBTAG00000012361	0.1914815	0.4857467	0.3135901
ENSBTAG00000012365	0.0650464	0.8028072	0.0953888
ENSBTAG00000012366	-0.113896	0.6597738	0.1806049
ENSBTAG00000012370	0.800121	0.016286	1.7881856
ENSBTAG00000012371	0.0169427	0.9465516	0.0238557
ENSBTAG00000012372	NA	NA	NA
ENSBTAG00000012374	NA	NA	NA
ENSBTAG00000012375	-0.26283	0.7991896	0.0973502
ENSBTAG00000012377	0.1927505	0.4642806	0.3332195
ENSBTAG00000012380	0.0828233	0.7834505	0.1059884
ENSBTAG00000012382	-0.391549	0.2015789	0.695555
ENSBTAG00000012383	-0.024666	0.92146	0.0355235
ENSBTAG00000012384	-0.027975	0.91593	0.0381377
ENSBTAG00000012385	-0.107911	0.6763702	0.1698156
ENSBTAG00000012387	-0.020752	0.9365303	0.0284782
ENSBTAG00000012390	NA	NA	NA
ENSBTAG00000012391	0.2908686	0.2746405	0.5612355
ENSBTAG00000012393	0.9489515	0.0444708	1.3519252
ENSBTAG00000012394	-0.354483	0.3435939	0.4639546
ENSBTAG00000012397	0.067063	0.8633394	0.0638184
ENSBTAG00000012398	NA	NA	NA
ENSBTAG00000012403	NA	NA	NA
ENSBTAG00000012405	0.1884264	0.6448908	0.1905138
ENSBTAG00000012406	NA	NA	NA
ENSBTAG00000012407	NA	NA	NA

ENSBTAG00000012408	0.4915181	0.2813399	0.5507687
ENSBTAG00000012409	0.2106354	0.7391814	0.131249
ENSBTAG00000012411	NA	NA	NA
ENSBTAG00000012412	0.4797828	0.1526844	0.8162052
ENSBTAG00000012413	NA	NA	NA
ENSBTAG00000012414	NA	NA	NA
ENSBTAG00000012416	-0.50567	0.148357	0.8286918
ENSBTAG00000012417	0.0276774	0.9178905	0.0372091
ENSBTAG00000012418	-0.062618	0.8100164	0.0915062
ENSBTAG00000012419	NA	NA	NA
ENSBTAG00000012421	0.244835	0.6058324	0.2176475
ENSBTAG00000012423	0.152136	0.6044728	0.2186232
ENSBTAG00000012425	NA	NA	NA
ENSBTAG00000012426	-0.009282	0.9741076	0.011393
ENSBTAG00000012432	-0.361355	0.1993666	0.7003476
ENSBTAG00000012433	-0.97308	0.3212569	0.4931475
ENSBTAG00000012434	0.882326	0.0523078	1.2814332
ENSBTAG00000012436	NA	NA	NA
ENSBTAG00000012437	NA	NA	NA
ENSBTAG00000012439	NA	NA	NA
ENSBTAG00000012441	-1.883596	2.49E-07	6.6032795
ENSBTAG00000012442	0.1593508	0.5284665	0.2769825
ENSBTAG00000012443	NA	NA	NA
ENSBTAG00000012444	NA	NA	NA
ENSBTAG00000012446	-0.184919	0.6507801	0.1865657
ENSBTAG00000012447	0.0074524	0.9809008	0.0083749
ENSBTAG00000012448	-0.260971	0.3276825	0.4845467
ENSBTAG00000012449	NA	NA	NA
ENSBTAG00000012450	-0.516477	0.2131878	0.6712377
ENSBTAG00000012451	-0.028433	0.9291963	0.0318925
ENSBTAG00000012454	-0.058124	0.8443094	0.0734984
ENSBTAG00000012456	NA	NA	NA
ENSBTAG00000012462	NA	NA	NA
ENSBTAG00000012463	-0.500776	0.2144691	0.6686352
ENSBTAG00000012464	-0.092655	0.8431822	0.0740786
ENSBTAG00000012465	NA	NA	NA
ENSBTAG00000012467	0.1338682	0.6417787	0.1926147
ENSBTAG00000012470	-0.007162	0.9787714	0.0093187
ENSBTAG00000012471	-0.311186	0.2112436	0.6752163
ENSBTAG00000012476	0.1795976	0.5664515	0.2468373
ENSBTAG00000012480	0.2893467	0.3691992	0.4327392
ENSBTAG00000012481	0.1571646	0.5816891	0.2353091
ENSBTAG00000012482	NA	NA	NA
ENSBTAG00000012484	0.0564536	0.8461109	0.0725727
ENSBTAG00000012485	-0.326501	0.4209101	0.3758106
ENSBTAG00000012489	-0.753152	0.0148989	1.8268471
ENSBTAG00000012490	-0.087467	0.7258638	0.1391449
ENSBTAG00000012495	-0.109586	0.9027836	0.0444164
ENSBTAG00000012496	0.5119378	0.2336577	0.6314198
ENSBTAG00000012497	-0.174204	0.5237315	0.2808913
ENSBTAG00000012499	-0.28229	0.5170585	0.2864603
ENSBTAG00000012500	-0.114456	0.74222	0.1294674
ENSBTAG00000012501	-0.028594	0.9164472	0.0378925
ENSBTAG00000012503	NA	NA	NA
ENSBTAG00000012504	-0.537711	0.2387781	0.6220055
ENSBTAG00000012505	0.5576844	0.0821544	1.085369
ENSBTAG00000012507	NA	NA	NA
ENSBTAG00000012508	NA	NA	NA

ENSBTAG00000012509	-0.248436	0.3498987	0.4560577
ENSBTAG00000012510	0.0414011	0.8921743	0.0495503
ENSBTAG00000012511	0.0971053	0.8123204	0.0902726
ENSBTAG00000012512	0.2692992	0.5719436	0.2426468
ENSBTAG00000012514	0.1656432	0.5563395	0.2546601
ENSBTAG00000012516	NA	NA	NA
ENSBTAG00000012518	0.5736401	0.1410941	0.8504912
ENSBTAG00000012519	0.761877	0.0151527	1.8195106
ENSBTAG00000012522	0.1132634	0.7525148	0.1234849
ENSBTAG00000012525	NA	NA	NA
ENSBTAG00000012526	NA	NA	NA
ENSBTAG00000012529	NA	NA	NA
ENSBTAG00000012534	NA	NA	NA
ENSBTAG00000012535	NA	NA	NA
ENSBTAG00000012537	-0.118379	0.7532891	0.1230383
ENSBTAG00000012540	NA	NA	NA
ENSBTAG00000012541	NA	NA	NA
ENSBTAG00000012543	-0.461982	0.2500789	0.6019229
ENSBTAG00000012544	0.0981979	0.6986997	0.1557094
ENSBTAG00000012545	-0.154864	0.5340356	0.2724298
ENSBTAG00000012550	0.2687836	0.4235466	0.3730988
ENSBTAG00000012552	-0.241885	0.4335342	0.3629767
ENSBTAG00000012554	NA	NA	NA
ENSBTAG00000012555	NA	NA	NA
ENSBTAG00000012557	-0.16466	0.5598317	0.2519425
ENSBTAG00000012558	0.2284733	0.554007	0.2564848
ENSBTAG00000012559	NA	NA	NA
ENSBTAG00000012561	0.1884977	0.5556237	0.2552192
ENSBTAG00000012562	1.1827833	0.0204891	1.6884779
ENSBTAG00000012564	-0.009101	0.9723543	0.0121754
ENSBTAG00000012565	-0.198308	0.455035	0.3419552
ENSBTAG00000012566	NA	NA	NA
ENSBTAG00000012567	NA	NA	NA
ENSBTAG00000012575	0.1816181	0.5402891	0.2673738
ENSBTAG00000012577	0.1583601	0.5855659	0.2324242
ENSBTAG00000012581	-0.029676	0.919252	0.0365654
ENSBTAG00000012582	-0.155976	0.5322026	0.273923
ENSBTAG00000012584	NA	NA	NA
ENSBTAG00000012585	-0.23666	0.3425494	0.4652768
ENSBTAG00000012586	-0.138668	0.5899819	0.2291613
ENSBTAG00000012587	0.1025538	0.6990795	0.1554735
ENSBTAG00000012589	0.0606508	0.8219312	0.0851645
ENSBTAG00000012592	NA	NA	NA
ENSBTAG00000012594	-0.044533	0.8893972	0.0509042
ENSBTAG00000012595	-0.054212	0.8793688	0.055829
ENSBTAG00000012596	1.0449004	0.0333165	1.4773401
ENSBTAG00000012599	-0.12109	0.7182802	0.1437061
ENSBTAG00000012600	-0.092346	0.7774904	0.109305
ENSBTAG00000012604	NA	NA	NA
ENSBTAG00000012605	0.7495806	0.0349369	1.4567153
ENSBTAG00000012607	0.1357941	0.6517449	0.1859223
ENSBTAG00000012608	-0.362999	0.2533324	0.5963093
ENSBTAG00000012609	0.2024513	0.6104649	0.2143393
ENSBTAG00000012615	0.0425148	0.8652325	0.0628672
ENSBTAG00000012618	NA	NA	NA
ENSBTAG00000012619	0.1488757	0.6394558	0.1941895
ENSBTAG00000012620	0.0314316	0.9046488	0.04352
ENSBTAG00000012621	NA	NA	NA

ENSBTAG00000012622	-0.32402	0.3267837	0.4857396
ENSBTAG00000012623	NA	NA	NA
ENSBTAG00000012624	NA	NA	NA
ENSBTAG00000012626	0.3125179	0.3149765	0.5017219
ENSBTAG00000012628	NA	NA	NA
ENSBTAG00000012629	-0.038454	0.935891	0.0287747
ENSBTAG00000012630	NA	NA	NA
ENSBTAG00000012632	-0.115337	0.6798225	0.1676045
ENSBTAG00000012634	0.1396712	0.5837644	0.2337624
ENSBTAG00000012636	NA	NA	NA
ENSBTAG00000012637	NA	NA	NA
ENSBTAG00000012638	NA	NA	NA
ENSBTAG00000012640	NA	NA	NA
ENSBTAG00000012642	0.3960165	0.3889423	0.4101149
ENSBTAG00000012644	-0.05628	0.8235079	0.0843322
ENSBTAG00000012646	NA	NA	NA
ENSBTAG00000012647	-0.272094	0.4114549	0.3856778
ENSBTAG00000012648	1.1573972	0.0099614	2.0016802
ENSBTAG00000012649	-0.027091	0.9160266	0.0380919
ENSBTAG00000012652	NA	NA	NA
ENSBTAG00000012653	0.2499459	0.4689031	0.3289169
ENSBTAG00000012654	-0.182693	0.4983394	0.3024748
ENSBTAG00000012656	NA	NA	NA
ENSBTAG00000012657	0.2197581	0.4300867	0.366444
ENSBTAG00000012658	-0.330454	0.2875027	0.5413581
ENSBTAG00000012659	-0.393809	0.1868576	0.7284892
ENSBTAG00000012664	0.3356664	0.3879329	0.4112434
ENSBTAG00000012667	-0.702512	0.0108949	1.9627755
ENSBTAG00000012668	NA	NA	NA
ENSBTAG00000012671	-0.023978	0.9251526	0.0337866
ENSBTAG00000012672	-0.368258	0.2495291	0.6028787
ENSBTAG00000012673	1.3487725	0.0087116	2.0599011
ENSBTAG00000012674	-0.695555	0.0751032	1.1243413
ENSBTAG00000012675	0.869748	0.0612772	1.2127009
ENSBTAG00000012676	NA	NA	NA
ENSBTAG00000012677	-0.194629	0.4858144	0.3135296
ENSBTAG00000012678	0.6024751	0.101176	0.9949223
ENSBTAG00000012681	0.5293886	0.2492192	0.6034185
ENSBTAG00000012682	NA	NA	NA
ENSBTAG00000012683	0.4985338	0.0492552	1.3075482
ENSBTAG00000012684	NA	NA	NA
ENSBTAG00000012687	0.6656345	0.0092985	2.0315884
ENSBTAG00000012691	0.1864245	0.4989389	0.3019526
ENSBTAG00000012692	NA	NA	NA
ENSBTAG00000012693	NA	NA	NA
ENSBTAG00000012694	-0.270711	0.3156005	0.5008623
ENSBTAG00000012695	-0.04687	0.9283571	0.032285
ENSBTAG00000012697	0.8171116	0.0016499	2.7825516
ENSBTAG00000012698	0.134527	0.6234181	0.2052206
ENSBTAG00000012699	0.1970143	0.5492121	0.2602599
ENSBTAG00000012700	0.1610977	0.6702309	0.1737756
ENSBTAG00000012702	0.3432697	0.3890887	0.4099514
ENSBTAG00000012703	-0.755202	0.004683	2.3294738
ENSBTAG00000012704	0.1339272	0.6371681	0.1957459
ENSBTAG00000012705	0.2688412	0.4157233	0.3811956
ENSBTAG00000012708	NA	NA	NA
ENSBTAG00000012710	NA	NA	NA
ENSBTAG00000012712	0.0423036	0.8719733	0.0594968

ENSBTAG00000012715	NA	NA	NA
ENSBTAG00000012718	-0.023012	0.9562765	0.0194165
ENSBTAG00000012719	-0.845372	0.0095664	2.0192535
ENSBTAG00000012720	0.8884107	0.0129574	1.8874822
ENSBTAG00000012721	0.2896318	0.6111642	0.2138421
ENSBTAG00000012722	NA	NA	NA
ENSBTAG00000012723	-0.142873	0.7298888	0.1367433
ENSBTAG00000012724	0.2352743	0.5909353	0.2284601
ENSBTAG00000012725	0.1693958	0.7099536	0.14877
ENSBTAG00000012726	-0.107306	0.6712274	0.1731303
ENSBTAG00000012729	-0.007346	0.9821534	0.0078207
ENSBTAG00000012736	NA	NA	NA
ENSBTAG00000012737	NA	NA	NA
ENSBTAG00000012738	0.5027253	0.3919859	0.4067295
ENSBTAG00000012739	-0.008645	0.9726675	0.0120356
ENSBTAG00000012740	-0.633095	0.087647	1.0572628
ENSBTAG00000012741	0.0483548	0.8497921	0.0706873
ENSBTAG00000012742	NA	NA	NA
ENSBTAG00000012744	-0.16164	0.5354226	0.2713033
ENSBTAG00000012745	0.4841353	0.154692	0.8105321
ENSBTAG00000012746	0.0130174	0.960196	0.0176401
ENSBTAG00000012747	0.4330551	0.2154903	0.6665722
ENSBTAG00000012748	NA	NA	NA
ENSBTAG00000012749	-0.132355	0.6031904	0.2195455
ENSBTAG00000012750	NA	NA	NA
ENSBTAG00000012751	-0.291206	0.3880549	0.4111068
ENSBTAG00000012752	0.2479482	0.4164616	0.380425
ENSBTAG00000012753	-0.10354	0.7406134	0.1304084
ENSBTAG00000012755	-0.017368	0.9585696	0.0183764
ENSBTAG00000012756	0.0450616	0.8585692	0.0662247
ENSBTAG00000012757	1.1735359	0.0475162	1.3231585
ENSBTAG00000012758	0.1377453	0.5987585	0.2227483
ENSBTAG00000012759	NA	NA	NA
ENSBTAG00000012760	0.0115502	0.9667456	0.0146878
ENSBTAG00000012761	1.1817315	0.0225745	1.6463811
ENSBTAG00000012762	-0.088073	0.774861	0.1107762
ENSBTAG00000012763	NA	NA	NA
ENSBTAG00000012765	NA	NA	NA
ENSBTAG00000012768	0.2316292	0.3874021	0.4118381
ENSBTAG00000012771	0.0300533	0.9347665	0.0292969
ENSBTAG00000012772	NA	NA	NA
ENSBTAG00000012774	-0.118124	0.746148	0.127175
ENSBTAG00000012777	-0.297457	0.2967783	0.5275678
ENSBTAG00000012778	0.2990767	0.2427402	0.6148584
ENSBTAG00000012779	1.3796384	0.0076399	2.1169099
ENSBTAG00000012780	NA	NA	NA
ENSBTAG00000012781	0.133542	0.6216152	0.2064784
ENSBTAG00000012782	NA	NA	NA
ENSBTAG00000012784	-0.286994	0.4370342	0.3594846
ENSBTAG00000012787	-0.376062	0.2639436	0.5784889
ENSBTAG00000012788	0.1011313	0.7326145	0.1351245
ENSBTAG00000012789	0.1849454	0.6125227	0.2128778
ENSBTAG00000012790	-0.131992	0.7026326	0.1532717
ENSBTAG00000012791	-0.020283	0.9365729	0.0284584
ENSBTAG00000012792	0.135759	0.6890538	0.1617468
ENSBTAG00000012794	NA	NA	NA
ENSBTAG00000012796	-0.146981	0.7460123	0.127254
ENSBTAG00000012797	-0.240119	0.3556781	0.4489429

ENSBTAG00000012798	NA	NA	NA
ENSBTAG00000012800	-0.24772	0.4339569	0.3625534
ENSBTAG00000012801	NA	NA	NA
ENSBTAG00000012803	0.0124498	0.9670405	0.0145553
ENSBTAG00000012804	-0.468401	0.1253942	0.9017227
ENSBTAG00000012805	0.6907672	0.0075328	2.1230419
ENSBTAG00000012809	NA	NA	NA
ENSBTAG00000012815	NA	NA	NA
ENSBTAG00000012817	0.0764065	0.7780349	0.1090009
ENSBTAG00000012818	-1.067851	0.0018725	2.7275747
ENSBTAG00000012820	-0.408162	0.2772024	0.5572029
ENSBTAG00000012822	NA	NA	NA
ENSBTAG00000012823	NA	NA	NA
ENSBTAG00000012824	-0.494938	0.2730485	0.5637602
ENSBTAG00000012827	0.2489218	0.708722	0.1495241
ENSBTAG00000012828	0.2532619	0.3572272	0.4470555
ENSBTAG00000012829	NA	NA	NA
ENSBTAG00000012830	0.4517338	0.2025119	0.6935495
ENSBTAG00000012833	0.0724791	0.773955	0.1112843
ENSBTAG00000012834	NA	NA	NA
ENSBTAG00000012837	-0.494209	0.2530944	0.5967175
ENSBTAG00000012838	NA	NA	NA
ENSBTAG00000012844	0.1212415	0.6990465	0.1554939
ENSBTAG00000012845	0.1360767	0.6460262	0.1897499
ENSBTAG00000012846	NA	NA	NA
ENSBTAG00000012847	0.0959119	0.7395655	0.1310234
ENSBTAG00000012848	0.4099621	0.3415812	0.4665061
ENSBTAG00000012849	0.7449147	0.0039152	2.4072514
ENSBTAG00000012850	NA	NA	NA
ENSBTAG00000012851	NA	NA	NA
ENSBTAG00000012852	-0.164629	0.5392701	0.2681937
ENSBTAG00000012854	0.3214843	0.4938268	0.3064253
ENSBTAG00000012855	-0.160894	0.6479466	0.1884608
ENSBTAG00000012857	0.8704298	0.018827	1.7252182
ENSBTAG00000012858	-0.309492	0.2360865	0.6269289
ENSBTAG00000012860	0.0567965	0.8167891	0.0878901
ENSBTAG00000012861	NA	NA	NA
ENSBTAG00000012865	-0.324853	0.1984544	0.7023393
ENSBTAG00000012866	0.8776559	0.0089048	2.0503772
ENSBTAG00000012867	0.1461998	0.63039	0.2003907
ENSBTAG00000012868	2.2660013	8.00E-05	4.0969524
ENSBTAG00000012870	0.2941109	0.3387771	0.4700859
ENSBTAG00000012873	-0.165907	0.5454682	0.2632305
ENSBTAG00000012878	0.016508	0.9528722	0.0209654
ENSBTAG00000012880	-0.295532	0.3357918	0.4739299
ENSBTAG00000012881	0.0718963	0.776966	0.109598
ENSBTAG00000012882	-0.408894	0.1023994	0.9897026
ENSBTAG00000012885	-0.125658	0.6760852	0.1699985
ENSBTAG00000012887	NA	NA	NA
ENSBTAG00000012888	NA	NA	NA
ENSBTAG00000012890	0.0125954	0.9628905	0.0164231
ENSBTAG00000012892	NA	NA	NA
ENSBTAG00000012894	-0.303235	0.6320878	0.1992226
ENSBTAG00000012896	NA	NA	NA
ENSBTAG00000012897	0.8124376	0.0031147	2.506579
ENSBTAG00000012898	-0.37405	0.1451159	0.8382849
ENSBTAG00000012899	0.0174608	0.9466901	0.0237922
ENSBTAG00000012900	NA	NA	NA

ENSBTAG00000012902	-0.272104	0.3800467	0.4201631
ENSBTAG00000012904	-0.028544	0.9250826	0.0338195
ENSBTAG00000012907	-0.423663	0.2438181	0.612934
ENSBTAG00000012908	0.0754761	0.792839	0.100815
ENSBTAG00000012909	NA	NA	NA
ENSBTAG00000012912	NA	NA	NA
ENSBTAG00000012915	NA	NA	NA
ENSBTAG00000012919	-0.264293	0.4124236	0.3846565
ENSBTAG00000012920	0.0670062	0.7902904	0.1022133
ENSBTAG00000012921	NA	NA	NA
ENSBTAG00000012922	0.053896	0.8429614	0.0741923
ENSBTAG00000012925	NA	NA	NA
ENSBTAG00000012926	-0.31988	0.2098706	0.6780485
ENSBTAG00000012927	-0.283808	0.3364073	0.4731346
ENSBTAG00000012928	-0.141858	0.5832453	0.2341488
ENSBTAG00000012929	0.0516902	0.8351747	0.0782227
ENSBTAG00000012931	-0.400942	0.1592696	0.7978671
ENSBTAG00000012936	0.1162612	0.6487582	0.1879171
ENSBTAG00000012937	-0.059031	0.8454257	0.0729246
ENSBTAG00000012938	-0.078169	0.7841146	0.1056204
ENSBTAG00000012939	-0.552993	0.0381888	1.4180645
ENSBTAG00000012940	-0.439909	0.3665137	0.4359098
ENSBTAG00000012941	0.0061034	0.9885201	0.0050145
ENSBTAG00000012943	-0.419913	0.2314433	0.6355553
ENSBTAG00000012944	NA	NA	NA
ENSBTAG00000012945	NA	NA	NA
ENSBTAG00000012946	0.9444457	0.0003381	3.4709638
ENSBTAG00000012953	NA	NA	NA
ENSBTAG00000012954	NA	NA	NA
ENSBTAG00000012955	0.3837132	0.3738135	0.4273451
ENSBTAG00000012957	-0.204525	0.4402861	0.3562651
ENSBTAG00000012961	0.1836542	0.5858446	0.2322175
ENSBTAG00000012962	-0.161142	0.5293338	0.2762704
ENSBTAG00000012963	NA	NA	NA
ENSBTAG00000012966	-0.498764	0.0578195	1.2379256
ENSBTAG00000012968	0.0963849	0.737703	0.1321184
ENSBTAG00000012969	-1.589201	5.39E-06	5.2682219
ENSBTAG00000012970	-0.192385	0.4504877	0.3463171
ENSBTAG00000012972	0.2213115	0.5139482	0.2890807
ENSBTAG00000012975	-0.099085	0.707816	0.1500796
ENSBTAG00000012976	1.4105062	4.86E-05	4.3132485
ENSBTAG00000012977	0.2098024	0.6886934	0.1619741
ENSBTAG00000012979	NA	NA	NA
ENSBTAG00000012980	0.1202951	0.7658412	0.1158613
ENSBTAG00000012981	0.3119676	0.2673133	0.5729794
ENSBTAG00000012982	0.2790258	0.3987731	0.3992741
ENSBTAG00000012985	0.3339844	0.2931248	0.5329474
ENSBTAG00000012987	0.7860511	0.0128133	1.8923399
ENSBTAG00000012988	0.0229055	0.9366967	0.028401
ENSBTAG00000012989	-0.015095	0.9717129	0.012462
ENSBTAG00000012990	0.1997386	0.5585391	0.2529464
ENSBTAG00000012991	0.186799	0.6486415	0.1879953
ENSBTAG00000012992	0.9676225	0.0366989	1.4353464
ENSBTAG00000012994	0.9806729	0.0253284	1.5963928
ENSBTAG00000012995	NA	NA	NA
ENSBTAG00000012996	-1.037341	0.0001416	3.8490073
ENSBTAG00000012997	NA	NA	NA
ENSBTAG00000012998	-0.117768	0.6761844	0.1699348

ENSBTAG00000012999	-0.05985	0.8493344	0.0709213
ENSBTAG00000013001	-0.175489	0.6067238	0.217009
ENSBTAG00000013004	0.0840053	0.7503	0.1247651
ENSBTAG00000013007	-0.049187	0.8610973	0.0649478
ENSBTAG00000013008	NA	NA	NA
ENSBTAG00000013009	-0.112874	0.8366609	0.0774505
ENSBTAG00000013010	0.2766773	0.4122568	0.3848321
ENSBTAG00000013011	-0.035087	0.888982	0.051107
ENSBTAG00000013013	0.054486	0.8370142	0.0772672
ENSBTAG00000013015	NA	NA	NA
ENSBTAG00000013016	-0.485782	0.0982193	1.0078032
ENSBTAG00000013017	NA	NA	NA
ENSBTAG00000013018	0.6645775	0.1270583	0.895997
ENSBTAG00000013023	0.0868401	0.7625101	0.1177544
ENSBTAG00000013025	-0.129847	0.6259963	0.2034282
ENSBTAG00000013026	NA	NA	NA
ENSBTAG00000013027	NA	NA	NA
ENSBTAG00000013029	1.1613627	0.0003952	3.4032358
ENSBTAG00000013030	-0.029549	0.9398679	0.0269332
ENSBTAG00000013031	-0.029819	0.9131724	0.0394472
ENSBTAG00000013032	-0.102467	0.716518	0.1447729
ENSBTAG00000013033	-0.168623	0.5140554	0.28899
ENSBTAG00000013036	NA	NA	NA
ENSBTAG00000013038	0.0211363	0.9365757	0.0284571
ENSBTAG00000013039	NA	NA	NA
ENSBTAG00000013042	-0.208166	0.4110956	0.3860572
ENSBTAG00000013043	-0.03349	0.9046422	0.0435231
ENSBTAG00000013044	0.1484137	0.7014445	0.1540067
ENSBTAG00000013045	0.1188471	0.6568351	0.1825436
ENSBTAG00000013046	-0.111843	0.6971177	0.1566939
ENSBTAG00000013047	NA	NA	NA
ENSBTAG00000013048	0.0173346	0.9582649	0.0185144
ENSBTAG00000013050	-0.104601	0.7570937	0.1208504
ENSBTAG00000013052	NA	NA	NA
ENSBTAG00000013054	NA	NA	NA
ENSBTAG00000013057	0.0661613	0.8171528	0.0876967
ENSBTAG00000013060	0.0365246	0.8847412	0.0531838
ENSBTAG00000013063	0.112872	0.7196513	0.1428779
ENSBTAG00000013064	NA	NA	NA
ENSBTAG00000013065	NA	NA	NA
ENSBTAG00000013066	-0.372685	0.3550165	0.4497514
ENSBTAG00000013068	0.1995548	0.5801222	0.2364805
ENSBTAG00000013069	-0.181266	0.6426118	0.1920513
ENSBTAG00000013070	NA	NA	NA
ENSBTAG00000013071	-0.303899	0.4297474	0.3667867
ENSBTAG00000013072	0.0155762	0.9577269	0.0187583
ENSBTAG00000013074	0.1222608	0.6655299	0.1768324
ENSBTAG00000013076	-0.203748	0.4382266	0.3583012
ENSBTAG00000013078	0.5053184	0.2369922	0.625266
ENSBTAG00000013081	-0.257815	0.5539117	0.2565594
ENSBTAG00000013084	0.1399298	0.6111408	0.2138587
ENSBTAG00000013090	NA	NA	NA
ENSBTAG00000013093	0.4758182	0.2898977	0.5377553
ENSBTAG00000013096	0.0819841	0.8624256	0.0642784
ENSBTAG00000013098	-0.105926	0.7241665	0.1401616
ENSBTAG00000013099	NA	NA	NA
ENSBTAG00000013100	NA	NA	NA
ENSBTAG00000013103	0.1968931	0.5985783	0.2228791

ENSBTAG00000013105	NA	NA	NA
ENSBTAG00000013107	NA	NA	NA
ENSBTAG00000013108	-0.993047	0.0004351	3.3614548
ENSBTAG00000013109	0.4624317	0.0781292	1.1071867
ENSBTAG00000013110	NA	NA	NA
ENSBTAG00000013111	-0.426028	0.0905706	1.0430129
ENSBTAG00000013112	0.0298624	0.9079819	0.0419228
ENSBTAG00000013113	-0.250667	0.3542029	0.4507479
ENSBTAG00000013114	0.3357187	0.2460636	0.6089525
ENSBTAG00000013116	0.0051437	0.9852493	0.0064539
ENSBTAG00000013117	-0.534838	0.2682786	0.5714139
ENSBTAG00000013118	0.1814728	0.6871145	0.1629709
ENSBTAG00000013120	0.2212484	0.4416497	0.3549221
ENSBTAG00000013123	0.2006086	0.443663	0.3529468
ENSBTAG00000013124	NA	NA	NA
ENSBTAG00000013125	NA	NA	NA
ENSBTAG00000013126	0.0249206	0.9276848	0.0325995
ENSBTAG00000013127	0.1030518	0.6955047	0.1577
ENSBTAG00000013128	0.0647668	0.7939403	0.1002122
ENSBTAG00000013131	0.278913	0.529776	0.2759077
ENSBTAG00000013132	0.1721568	0.5831332	0.2342322
ENSBTAG00000013133	NA	NA	NA
ENSBTAG00000013134	NA	NA	NA
ENSBTAG00000013136	-0.357795	0.3553271	0.4493716
ENSBTAG00000013138	NA	NA	NA
ENSBTAG00000013141	-0.713691	0.1587878	0.7991827
ENSBTAG00000013142	-0.242875	0.3463576	0.4604753
ENSBTAG00000013143	0.4548365	0.1969559	0.7056309
ENSBTAG00000013145	0.0385957	0.883303	0.0538903
ENSBTAG00000013147	NA	NA	NA
ENSBTAG00000013148	NA	NA	NA
ENSBTAG00000013150	0.0720741	0.7959693	0.0991037
ENSBTAG00000013152	-0.576959	0.2016283	0.6954484
ENSBTAG00000013153	-0.206591	0.4218409	0.3748513
ENSBTAG00000013155	NA	NA	NA
ENSBTAG00000013157	NA	NA	NA
ENSBTAG00000013159	-0.010599	0.9815348	0.0080943
ENSBTAG00000013160	NA	NA	NA
ENSBTAG00000013162	-0.199079	0.4849322	0.3143189
ENSBTAG00000013163	0.1498703	0.6627856	0.1786269
ENSBTAG00000013165	0.593063	0.0549246	1.2602329
ENSBTAG00000013166	-0.348393	0.1635398	0.7863766
ENSBTAG00000013167	NA	NA	NA
ENSBTAG00000013168	-0.336257	0.2249447	0.6479241
ENSBTAG00000013169	-0.094152	0.7194853	0.1429781
ENSBTAG00000013170	NA	NA	NA
ENSBTAG00000013173	NA	NA	NA
ENSBTAG00000013175	0.1845845	0.4900764	0.3097362
ENSBTAG00000013176	-0.384715	0.2098767	0.6780357
ENSBTAG00000013177	0.4821337	0.2710385	0.5669691
ENSBTAG00000013180	0.1623454	0.5925672	0.2272624
ENSBTAG00000013183	NA	NA	NA
ENSBTAG00000013184	-0.142203	0.5780567	0.2380295
ENSBTAG00000013185	NA	NA	NA
ENSBTAG00000013187	0.5272678	0.0910481	1.0407293
ENSBTAG00000013191	0.6387675	0.1138302	0.9437424
ENSBTAG00000013192	-0.410118	0.2923057	0.5341627
ENSBTAG00000013196	0.4513899	0.1293519	0.8882272

ENSBTAG00000013197	0.4933925	0.2081272	0.6816711
ENSBTAG00000013198	0.2157948	0.5553297	0.2554491
ENSBTAG00000013201	NA	NA	NA
ENSBTAG00000013203	NA	NA	NA
ENSBTAG00000013204	-0.139502	0.5973764	0.2237519
ENSBTAG00000013205	1.15902	0.0172291	1.7637381
ENSBTAG00000013208	0.2424885	0.3969144	0.4013032
ENSBTAG00000013210	0.6430014	0.1351868	0.8690658
ENSBTAG00000013211	-0.465405	0.0854705	1.0681838
ENSBTAG00000013212	0.1569357	0.5666604	0.2466771
ENSBTAG00000013213	0.1601053	0.7186838	0.1434621
ENSBTAG00000013215	NA	NA	NA
ENSBTAG00000013218	0.0914988	0.7283539	0.1376576
ENSBTAG00000013219	NA	NA	NA
ENSBTAG00000013221	0.4194318	0.2016222	0.6954616
ENSBTAG00000013222	0.2919772	0.2763555	0.5585318
ENSBTAG00000013224	-0.062645	0.8796222	0.0557038
ENSBTAG00000013225	-0.124133	0.6321934	0.19915
ENSBTAG00000013226	0.0850456	0.8141778	0.0892808
ENSBTAG00000013227	-1.334579	0.0321598	1.4926869
ENSBTAG00000013231	-0.353512	0.2853755	0.5445833
ENSBTAG00000013235	0.1950479	0.5000561	0.3009812
ENSBTAG00000013236	-0.104232	0.6927236	0.15944
ENSBTAG00000013238	-0.180985	0.5049302	0.2967687
ENSBTAG00000013239	NA	NA	NA
ENSBTAG00000013240	0.4550764	0.1499118	0.8241643
ENSBTAG00000013242	NA	NA	NA
ENSBTAG00000013244	0.0199722	0.9537771	0.0205531
ENSBTAG00000013245	0.3666793	0.4218348	0.3748576
ENSBTAG00000013249	NA	NA	NA
ENSBTAG00000013250	NA	NA	NA
ENSBTAG00000013251	-0.115284	0.6558166	0.1832176
ENSBTAG00000013253	0.2252444	0.3826912	0.4171515
ENSBTAG00000013254	-0.512048	0.0447159	1.3495378
ENSBTAG00000013256	NA	NA	NA
ENSBTAG00000013259	0.3856516	0.1681629	0.7742697
ENSBTAG00000013260	NA	NA	NA
ENSBTAG00000013263	NA	NA	NA
ENSBTAG00000013264	-0.098925	0.7054986	0.1515038
ENSBTAG00000013265	0.1243729	0.7099128	0.148795
ENSBTAG00000013270	0.2186177	0.4833434	0.3157442
ENSBTAG00000013271	-0.131746	0.594823	0.2256122
ENSBTAG00000013273	0.1909003	0.6556138	0.1833519
ENSBTAG00000013274	-0.071553	0.8422216	0.0745736
ENSBTAG00000013275	0.2375579	0.627755	0.2022098
ENSBTAG00000013277	0.0814967	0.8048147	0.0943041
ENSBTAG00000013278	0.0410912	0.9364909	0.0284964
ENSBTAG00000013279	NA	NA	NA
ENSBTAG00000013281	NA	NA	NA
ENSBTAG00000013282	-0.052378	0.8712465	0.0598589
ENSBTAG00000013283	NA	NA	NA
ENSBTAG00000013284	-0.119462	0.6983257	0.155942
ENSBTAG00000013287	0.3352945	0.436697	0.3598198
ENSBTAG00000013288	-0.04018	0.8921309	0.0495714
ENSBTAG00000013289	0.0814142	0.7808335	0.1074415
ENSBTAG00000013290	0.0674507	0.8176049	0.0874565
ENSBTAG00000013291	-0.047644	0.8913915	0.0499315
ENSBTAG00000013292	NA	NA	NA

ENSBTAG00000013294	-0.268781	0.5477653	0.2614055
ENSBTAG00000013298	0.0412625	0.9277893	0.0325507
ENSBTAG00000013300	0.1484558	0.6190644	0.2082642
ENSBTAG00000013301	NA	NA	NA
ENSBTAG00000013302	-0.151898	0.6442195	0.1909662
ENSBTAG00000013303	-0.495964	0.0997162	1.0012343
ENSBTAG00000013305	NA	NA	NA
ENSBTAG00000013306	NA	NA	NA
ENSBTAG00000013308	-0.142599	0.5733252	0.241599
ENSBTAG00000013309	0.3185865	0.3519856	0.4534751
ENSBTAG00000013314	NA	NA	NA
ENSBTAG00000013315	-0.000279	0.9991717	0.0003599
ENSBTAG00000013316	0.1169939	0.7276717	0.1380645
ENSBTAG00000013317	0.3936969	0.1606437	0.7941363
ENSBTAG00000013319	-0.100592	0.7230565	0.1408278
ENSBTAG00000013320	NA	NA	NA
ENSBTAG00000013321	NA	NA	NA
ENSBTAG00000013322	0.1126703	0.6673216	0.1756648
ENSBTAG00000013326	-0.42136	0.1730691	0.7617806
ENSBTAG00000013329	0.3511054	0.2556804	0.5923025
ENSBTAG00000013330	0.0582926	0.836343	0.0776156
ENSBTAG00000013333	NA	NA	NA
ENSBTAG00000013334	NA	NA	NA
ENSBTAG00000013336	NA	NA	NA
ENSBTAG00000013337	-0.517892	0.07112	1.1480082
ENSBTAG00000013338	NA	NA	NA
ENSBTAG00000013339	0.0928632	0.8042944	0.094585
ENSBTAG00000013340	NA	NA	NA
ENSBTAG00000013341	-1.262	0.0002092	3.6794113
ENSBTAG00000013343	0.7880221	0.0043578	2.3607352
ENSBTAG00000013345	NA	NA	NA
ENSBTAG00000013346	0.4456413	0.385733	0.4137132
ENSBTAG00000013347	-0.212056	0.5213046	0.2829084
ENSBTAG00000013352	NA	NA	NA
ENSBTAG00000013353	0.0099083	0.9793683	0.009054
ENSBTAG00000013357	-0.183866	0.5369069	0.270101
ENSBTAG00000013358	0.0357295	0.8931959	0.0490533
ENSBTAG00000013362	-0.079399	0.7872275	0.1038998
ENSBTAG00000013363	-0.052325	0.8346656	0.0784875
ENSBTAG00000013366	NA	NA	NA
ENSBTAG00000013367	-0.05165	0.8708784	0.0600425
ENSBTAG00000013368	NA	NA	NA
ENSBTAG00000013369	-0.097941	0.7105235	0.1484216
ENSBTAG00000013371	0.4956023	0.3180226	0.497542
ENSBTAG00000013378	NA	NA	NA
ENSBTAG00000013380	0.1641079	0.5801037	0.2364944
ENSBTAG00000013384	NA	NA	NA
ENSBTAG00000013387	-0.102333	0.6948008	0.1581397
ENSBTAG00000013390	-0.088172	0.7268621	0.138548
ENSBTAG00000013391	-0.191044	0.5022738	0.2990594
ENSBTAG00000013392	0.3999144	0.3277176	0.4845002
ENSBTAG00000013393	NA	NA	NA
ENSBTAG00000013395	NA	NA	NA
ENSBTAG00000013401	0.5551382	0.0764905	1.1163923
ENSBTAG00000013402	0.4040571	0.4550929	0.3419
ENSBTAG00000013403	NA	NA	NA
ENSBTAG00000013405	-9.63E-05	1	0
ENSBTAG00000013406	-0.431591	0.2755234	0.5598415

ENSBTAG00000013407	0.4546676	0.36331	0.4397226
ENSBTAG00000013408	0.7069503	0.628023	0.2020245
ENSBTAG00000013410	-0.197107	0.4318123	0.3647049
ENSBTAG00000013411	0.0523339	0.8359812	0.0778035
ENSBTAG00000013412	-0.436626	0.0908645	1.0416058
ENSBTAG00000013413	0.8528102	0.0450154	1.3466384
ENSBTAG00000013414	-0.022067	0.9469325	0.023681
ENSBTAG00000013419	-0.16777	0.6762031	0.1699229
ENSBTAG00000013420	-0.165552	0.5487074	0.2606592
ENSBTAG00000013421	-0.039031	0.8882681	0.0514559
ENSBTAG00000013423	-0.595454	0.0352652	1.452653
ENSBTAG00000013425	-0.015185	0.9536968	0.0205897
ENSBTAG00000013426	-0.150019	0.7103401	0.1485337
ENSBTAG00000013429	NA	NA	NA
ENSBTAG00000013436	0.2419674	0.4643572	0.3331478
ENSBTAG00000013439	0.5701512	0.2867817	0.5424486
ENSBTAG00000013440	-0.085502	0.7639234	0.1169502
ENSBTAG00000013442	NA	NA	NA
ENSBTAG00000013444	NA	NA	NA
ENSBTAG00000013445	NA	NA	NA
ENSBTAG00000013449	-0.224149	0.3694878	0.4323999
ENSBTAG00000013451	NA	NA	NA
ENSBTAG00000013452	NA	NA	NA
ENSBTAG00000013454	-0.360653	0.1506286	0.8220924
ENSBTAG00000013455	0.0266908	0.9445683	0.0247666
ENSBTAG00000013460	-0.451285	0.0806954	1.0931513
ENSBTAG00000013461	0.0163107	0.9491533	0.0226637
ENSBTAG00000013462	NA	NA	NA
ENSBTAG00000013463	-0.552816	0.060246	1.220072
ENSBTAG00000013464	-0.218313	0.4069853	0.3904212
ENSBTAG00000013465	0.2096506	0.6321121	0.1992059
ENSBTAG00000013468	-0.239189	0.3927047	0.4059339
ENSBTAG00000013469	NA	NA	NA
ENSBTAG00000013472	0.1361118	0.7090761	0.1493072
ENSBTAG00000013473	NA	NA	NA
ENSBTAG00000013475	-0.205693	0.6285243	0.2016779
ENSBTAG00000013476	-0.535044	0.2879682	0.5406555
ENSBTAG00000013477	0.3327187	0.5244758	0.2802746
ENSBTAG00000013478	0.2029564	0.567565	0.2459844
ENSBTAG00000013479	-0.393356	0.2312263	0.6359627
ENSBTAG00000013480	0.291709	0.3636297	0.4393406
ENSBTAG00000013483	0.0426602	0.9083265	0.041758
ENSBTAG00000013485	-0.466171	0.2801266	0.5526457
ENSBTAG00000013486	-0.896183	0.0371015	1.4306081
ENSBTAG00000013488	NA	NA	NA
ENSBTAG00000013489	0.2574022	0.418578	0.3782236
ENSBTAG00000013491	-0.455484	0.082805	1.0819433
ENSBTAG00000013492	-0.553305	0.0526194	1.2788541
ENSBTAG00000013493	0.0822947	0.8250263	0.0835322
ENSBTAG00000013495	0.2936658	0.264113	0.5782103
ENSBTAG00000013496	0.5702343	0.1609341	0.793352
ENSBTAG00000013498	NA	NA	NA
ENSBTAG00000013501	NA	NA	NA
ENSBTAG00000013503	NA	NA	NA
ENSBTAG00000013505	NA	NA	NA
ENSBTAG00000013510	NA	NA	NA
ENSBTAG00000013511	NA	NA	NA
ENSBTAG00000013513	-0.04556	0.8575385	0.0667464

ENSBTAG00000013515	-0.296498	0.312129	0.5056659
ENSBTAG00000013523	0.7794161	0.0715241	1.1455475
ENSBTAG00000013524	0.4503442	0.2943555	0.5311278
ENSBTAG00000013525	NA	NA	NA
ENSBTAG00000013526	-0.057426	0.8244973	0.0838108
ENSBTAG00000013527	0.005058	0.9902355	0.0042615
ENSBTAG00000013528	-0.269057	0.2816588	0.5502767
ENSBTAG00000013529	NA	NA	NA
ENSBTAG00000013530	-0.551219	0.0892706	1.0492915
ENSBTAG00000013531	-0.22217	0.5221956	0.2821668
ENSBTAG00000013533	-0.353836	0.2705574	0.5677405
ENSBTAG00000013534	NA	NA	NA
ENSBTAG00000013535	0.2134085	0.5020831	0.2992244
ENSBTAG00000013536	NA	NA	NA
ENSBTAG00000013537	NA	NA	NA
ENSBTAG00000013538	-0.090411	0.7874382	0.1037835
ENSBTAG00000013541	NA	NA	NA
ENSBTAG00000013544	0.2995123	0.270477	0.5678697
ENSBTAG00000013545	0.4381877	0.081429	1.0892211
ENSBTAG00000013548	NA	NA	NA
ENSBTAG00000013550	NA	NA	NA
ENSBTAG00000013555	-0.093095	0.813311	0.0897434
ENSBTAG00000013556	0.131128	0.7728967	0.1118785
ENSBTAG00000013557	1.0026614	0.0040344	2.3942187
ENSBTAG00000013560	-0.137027	0.7696245	0.1137211
ENSBTAG00000013562	0.1008847	0.8469983	0.0721175
ENSBTAG00000013563	0.1094617	0.6905362	0.1608136
ENSBTAG00000013568	-0.051408	0.8658545	0.0625551
ENSBTAG00000013569	NA	NA	NA
ENSBTAG00000013573	-0.54818	0.2330978	0.6324619
ENSBTAG00000013577	0.4226109	0.2389773	0.6216434
ENSBTAG00000013579	0.0921992	0.7364767	0.132841
ENSBTAG00000013580	-0.048133	0.8534218	0.0688362
ENSBTAG00000013581	0.8130767	0.0156379	1.8058226
ENSBTAG00000013586	NA	NA	NA
ENSBTAG00000013587	-0.252388	0.4148363	0.3821233
ENSBTAG00000013588	0.3139776	0.2679329	0.5719739
ENSBTAG00000013589	-0.27553	0.2732403	0.5634552
ENSBTAG00000013591	-0.027944	0.9421052	0.0259006
ENSBTAG00000013592	0.2291537	0.4647483	0.3327822
ENSBTAG00000013593	-0.064592	0.8426124	0.0743721
ENSBTAG00000013596	NA	NA	NA
ENSBTAG00000013598	-0.272432	0.3254874	0.4874658
ENSBTAG00000013600	0.1665296	0.5763475	0.2393156
ENSBTAG00000013602	-0.054773	0.8319655	0.0798947
ENSBTAG00000013606	0.0355971	0.9103636	0.0407851
ENSBTAG00000013607	0.001431	0.9958074	0.0018247
ENSBTAG00000013611	0.1169509	0.7816834	0.1069691
ENSBTAG00000013612	-0.501567	0.0886532	1.0523055
ENSBTAG00000013613	0.2194077	0.4742969	0.3239497
ENSBTAG00000013614	0.0090812	0.9723025	0.0121986
ENSBTAG00000013615	-0.117594	0.6683813	0.1749757
ENSBTAG00000013616	-0.094668	0.7013867	0.1540425
ENSBTAG00000013620	-0.271621	0.2863041	0.5431725
ENSBTAG00000013621	-0.117578	0.6653781	0.1769315
ENSBTAG00000013623	-0.166094	0.5270886	0.2781164
ENSBTAG00000013624	-0.285079	0.3924425	0.406224
ENSBTAG00000013625	NA	NA	NA

ENSBTAG00000013627	0.2174028	0.3889464	0.4101102
ENSBTAG00000013628	-0.497886	0.204887	0.6884856
ENSBTAG00000013629	-0.007146	0.9841997	0.0069168
ENSBTAG00000013631	0.8285397	0.0086044	2.0652773
ENSBTAG00000013632	NA	NA	NA
ENSBTAG00000013636	-0.166155	0.6367948	0.1960005
ENSBTAG00000013638	NA	NA	NA
ENSBTAG00000013640	NA	NA	NA
ENSBTAG00000013641	0.10091	0.7318338	0.1355876
ENSBTAG00000013642	NA	NA	NA
ENSBTAG00000013645	0.8250705	0.0255467	1.5926644
ENSBTAG00000013648	NA	NA	NA
ENSBTAG00000013650	0.3533897	0.4539036	0.3430364
ENSBTAG00000013651	0.1321118	0.6260089	0.2034195
ENSBTAG00000013652	-0.016446	0.9469043	0.0236939
ENSBTAG00000013653	-0.029037	0.9303366	0.0313599
ENSBTAG00000013657	NA	NA	NA
ENSBTAG00000013658	0.0138616	0.9642614	0.0158052
ENSBTAG00000013662	0.1144024	0.7917974	0.1013859
ENSBTAG00000013663	-0.082443	0.8026642	0.0954661
ENSBTAG00000013664	NA	NA	NA
ENSBTAG00000013665	-2.340653	1.37E-05	4.8619469
ENSBTAG00000013666	-0.417614	0.2287807	0.6405806
ENSBTAG00000013667	NA	NA	NA
ENSBTAG00000013669	-0.44944	0.1992556	0.7005894
ENSBTAG00000013670	-0.673726	0.2319531	0.6345998
ENSBTAG00000013671	0.317649	0.2169679	0.6636046
ENSBTAG00000013674	1.1370322	0.0035514	2.4495949
ENSBTAG00000013676	-0.178528	0.6650366	0.1771545
ENSBTAG00000013677	-0.381243	0.2057666	0.6866251
ENSBTAG00000013678	-0.494999	0.0735646	1.1333312
ENSBTAG00000013680	NA	NA	NA
ENSBTAG00000013685	NA	NA	NA
ENSBTAG00000013686	NA	NA	NA
ENSBTAG00000013688	0.0346833	0.9447295	0.0246925
ENSBTAG00000013689	NA	NA	NA
ENSBTAG00000013693	NA	NA	NA
ENSBTAG00000013697	0.414242	0.3400325	0.4684796
ENSBTAG00000013698	NA	NA	NA
ENSBTAG00000013699	0.5798274	0.045889	1.3382911
ENSBTAG00000013702	NA	NA	NA
ENSBTAG00000013705	0.3306086	0.4242799	0.3723475
ENSBTAG00000013706	-0.194379	0.6226652	0.2057454
ENSBTAG00000013708	-0.135582	0.7111019	0.1480682
ENSBTAG00000013711	NA	NA	NA
ENSBTAG00000013715	NA	NA	NA
ENSBTAG00000013716	-0.128351	0.683085	0.1655253
ENSBTAG00000013717	NA	NA	NA
ENSBTAG00000013718	NA	NA	NA
ENSBTAG00000013721	-0.109357	0.6959213	0.1574399
ENSBTAG00000013722	0.3734785	0.2217834	0.6540709
ENSBTAG00000013723	0.1462775	0.5573665	0.2538592
ENSBTAG00000013724	0.018618	0.9434095	0.0252998
ENSBTAG00000013725	-0.132302	0.6681854	0.175103
ENSBTAG00000013726	0.0124352	0.9824837	0.0076746
ENSBTAG00000013727	0.0085497	0.980457	0.0085715
ENSBTAG00000013730	NA	NA	NA
ENSBTAG00000013732	NA	NA	NA

ENSBTAG00000013734	NA	NA	NA
ENSBTAG00000013735	-0.29569	0.311408	0.5066702
ENSBTAG00000013736	NA	NA	NA
ENSBTAG00000013737	NA	NA	NA
ENSBTAG00000013740	NA	NA	NA
ENSBTAG00000013744	0.3535708	0.1921113	0.716447
ENSBTAG00000013745	-0.047054	0.8724404	0.0592642
ENSBTAG00000013749	-0.473947	0.0715163	1.1455947
ENSBTAG00000013750	-1.759314	0.0023814	2.62316
ENSBTAG00000013753	0.0684858	0.8094198	0.0918262
ENSBTAG00000013755	0.4355383	0.1016525	0.9928821
ENSBTAG00000013756	NA	NA	NA
ENSBTAG00000013757	-0.288732	0.2495479	0.6028462
ENSBTAG00000013759	0.8708804	0.0050084	2.3003043
ENSBTAG00000013760	0.2307053	0.6509215	0.1864714
ENSBTAG00000013761	0.1963767	0.6165143	0.2100568
ENSBTAG00000013763	NA	NA	NA
ENSBTAG00000013764	NA	NA	NA
ENSBTAG00000013766	NA	NA	NA
ENSBTAG00000013769	-0.313428	0.2984369	0.5251475
ENSBTAG00000013772	0.0804214	0.7616045	0.1182705
ENSBTAG00000013773	-0.015179	0.9561303	0.0194829
ENSBTAG00000013774	0.3538455	0.2191722	0.6592146
ENSBTAG00000013776	0.1087086	0.7101177	0.1486697
ENSBTAG00000013778	NA	NA	NA
ENSBTAG00000013779	0.2731511	0.4047488	0.3928144
ENSBTAG00000013781	0.0666656	0.7996671	0.0970908
ENSBTAG00000013782	-0.089561	0.7242477	0.1401129
ENSBTAG00000013784	0.0669656	0.8214114	0.0854393
ENSBTAG00000013790	-0.042929	0.8799335	0.0555502
ENSBTAG00000013792	-0.426465	0.0958592	1.0183664
ENSBTAG00000013793	-0.599524	0.036038	1.4432392
ENSBTAG00000013798	NA	NA	NA
ENSBTAG00000013801	-0.16727	0.5843739	0.2333092
ENSBTAG00000013802	NA	NA	NA
ENSBTAG00000013806	-0.400189	0.3964807	0.4017779
ENSBTAG00000013810	NA	NA	NA
ENSBTAG00000013812	0.2056651	0.4629079	0.3345054
ENSBTAG00000013813	0.1195176	0.7710918	0.1128939
ENSBTAG00000013819	NA	NA	NA
ENSBTAG00000013821	0.1470937	0.6540169	0.184411
ENSBTAG00000013822	0.3876427	0.4499482	0.3468375
ENSBTAG00000013824	0.0686039	0.8395782	0.0759388
ENSBTAG00000013825	NA	NA	NA
ENSBTAG00000013830	0.1161316	0.6804331	0.1672146
ENSBTAG00000013831	NA	NA	NA
ENSBTAG00000013832	-0.227032	0.4707464	0.327213
ENSBTAG00000013834	0.1938959	0.463605	0.3338519
ENSBTAG00000013836	-0.185195	0.6777179	0.168951
ENSBTAG00000013837	-0.240611	0.5640117	0.2487119
ENSBTAG00000013841	NA	NA	NA
ENSBTAG00000013842	-0.10692	0.7455591	0.1275179
ENSBTAG00000013843	-0.272827	0.4152799	0.3816591
ENSBTAG00000013848	0.5169193	0.1922994	0.7160222
ENSBTAG00000013849	NA	NA	NA
ENSBTAG00000013851	0.0696667	0.8401927	0.0756211
ENSBTAG00000013855	-0.290265	0.2589449	0.5867926
ENSBTAG00000013856	-0.311346	0.2419222	0.6163242

ENSBTAG00000013857	NA	NA	NA
ENSBTAG00000013858	-0.288763	0.3207442	0.4938412
ENSBTAG00000013859	-0.340631	0.1711364	0.7666576
ENSBTAG00000013860	0.4757791	0.0934449	1.0294443
ENSBTAG00000013861	0.2644082	0.4152633	0.3816765
ENSBTAG00000013863	0.10296	0.7037971	0.1525525
ENSBTAG00000013866	0.3040057	0.2481924	0.6052115
ENSBTAG00000013867	NA	NA	NA
ENSBTAG00000013869	0.687466	0.0582836	1.2344538
ENSBTAG00000013871	NA	NA	NA
ENSBTAG00000013873	NA	NA	NA
ENSBTAG00000013874	0.0898716	0.7566639	0.121097
ENSBTAG00000013877	NA	NA	NA
ENSBTAG00000013879	0.1940098	0.4882819	0.3113294
ENSBTAG00000013880	NA	NA	NA
ENSBTAG00000013881	-0.430011	0.2216966	0.6542409
ENSBTAG00000013882	0.0311571	0.90182	0.0448802
ENSBTAG00000013884	0.1417469	0.6794565	0.1678383
ENSBTAG00000013885	0.0023409	0.9962988	0.0016104
ENSBTAG00000013888	NA	NA	NA
ENSBTAG00000013889	0.077786	0.8248524	0.0836238
ENSBTAG00000013895	0.2522548	0.4534015	0.3435171
ENSBTAG00000013899	-0.450798	0.0869628	1.0606664
ENSBTAG00000013900	0.7114752	0.0954767	1.0201025
ENSBTAG00000013901	-0.034239	0.897837	0.0468025
ENSBTAG00000013905	-0.596288	0.0548673	1.2606866
ENSBTAG00000013906	NA	NA	NA
ENSBTAG00000013907	NA	NA	NA
ENSBTAG00000013909	0.2770646	0.4871057	0.3123768
ENSBTAG00000013910	-0.198864	0.5184906	0.2852591
ENSBTAG00000013912	0.2917755	0.2493974	0.6031081
ENSBTAG00000013914	0.1061282	0.6733206	0.1717781
ENSBTAG00000013915	-0.108162	0.8050857	0.0941579
ENSBTAG00000013916	0.0973886	0.7169485	0.144512
ENSBTAG00000013917	0.372542	0.1797762	0.7452677
ENSBTAG00000013918	0.1273709	0.7367706	0.1326677
ENSBTAG00000013919	-0.046881	0.8675021	0.0617295
ENSBTAG00000013920	NA	NA	NA
ENSBTAG00000013921	-0.119127	0.6672892	0.1756859
ENSBTAG00000013922	0.0464809	0.8657075	0.0626288
ENSBTAG00000013923	-0.171969	0.6366762	0.1960814
ENSBTAG00000013924	0.0887573	0.7394503	0.131091
ENSBTAG00000013925	0.331822	0.2889561	0.5391682
ENSBTAG00000013926	0.6241677	0.0213528	1.6705458
ENSBTAG00000013927	0.3145924	0.4792139	0.3194706
ENSBTAG00000013929	-0.320558	0.2593147	0.5861728
ENSBTAG00000013930	-0.007916	0.9770392	0.010088
ENSBTAG00000013931	0.1707135	0.5261435	0.2788958
ENSBTAG00000013932	0.3194046	0.3848859	0.414668
ENSBTAG00000013935	-0.419794	0.0939188	1.0272475
ENSBTAG00000013937	0.1152925	0.7253591	0.1394469
ENSBTAG00000013938	-0.038915	0.9049025	0.0433982
ENSBTAG00000013941	0.0615987	0.8427627	0.0742947
ENSBTAG00000013942	-0.127922	0.8032473	0.0951507
ENSBTAG00000013943	0.7649847	0.0645749	1.1899362
ENSBTAG00000013946	0.0521471	0.8418037	0.0747892
ENSBTAG00000013947	-0.063266	0.864969	0.0629994
ENSBTAG00000013949	-0.072703	0.7744238	0.1110213

ENSBTAG00000013951	0.0452608	0.8974332	0.0469979
ENSBTAG00000013952	-0.29024	0.248038	0.6054819
ENSBTAG00000013953	0.2690797	0.2816485	0.5502925
ENSBTAG00000013955	-0.167228	0.6042786	0.2187628
ENSBTAG00000013956	0.3113119	0.2295646	0.6390951
ENSBTAG00000013957	NA	NA	NA
ENSBTAG00000013958	-0.174913	0.6267245	0.2029233
ENSBTAG00000013960	0.2589533	0.5814715	0.2354716
ENSBTAG00000013961	0.0780986	0.7687558	0.1142116
ENSBTAG00000013971	0.5070291	0.0974933	1.0110253
ENSBTAG00000013973	NA	NA	NA
ENSBTAG00000013974	NA	NA	NA
ENSBTAG00000013976	NA	NA	NA
ENSBTAG00000013977	NA	NA	NA
ENSBTAG00000013979	NA	NA	NA
ENSBTAG00000013980	-0.56069	0.2825854	0.5488503
ENSBTAG00000013981	-0.075342	0.8151791	0.088747
ENSBTAG00000013982	-0.490136	0.0699739	1.1550641
ENSBTAG00000013983	NA	NA	NA
ENSBTAG00000013984	NA	NA	NA
ENSBTAG00000013985	0.2752319	0.3488724	0.4573334
ENSBTAG00000013988	0.106005	0.8152732	0.0886968
ENSBTAG00000013989	NA	NA	NA
ENSBTAG00000013990	NA	NA	NA
ENSBTAG00000013991	NA	NA	NA
ENSBTAG00000013992	-0.065029	0.8500517	0.0705547
ENSBTAG00000013994	NA	NA	NA
ENSBTAG00000013996	0.1155764	0.8016229	0.0960299
ENSBTAG00000013997	NA	NA	NA
ENSBTAG00000013999	-0.174478	0.5650957	0.247878
ENSBTAG00000014001	0.6473577	0.0161393	1.7921155
ENSBTAG00000014002	-0.130164	0.6634179	0.1782128
ENSBTAG00000014003	0.7802325	0.0090574	2.0429978
ENSBTAG00000014005	0.0931484	0.7484922	0.1258127
ENSBTAG00000014006	-0.262002	0.4738323	0.3243753
ENSBTAG00000014007	0.2419155	0.3939911	0.4045136
ENSBTAG00000014011	0.6305408	0.0418478	1.3783273
ENSBTAG00000014014	1.2803386	0.0027988	2.5530224
ENSBTAG00000014015	0.4757962	0.2085042	0.6808852
ENSBTAG00000014016	-0.12531	0.7751188	0.1106317
ENSBTAG00000014017	0.2679947	0.4353276	0.3611838
ENSBTAG00000014020	NA	NA	NA
ENSBTAG00000014021	0.0810237	0.7704655	0.1132468
ENSBTAG00000014023	-0.208036	0.4397007	0.3568428
ENSBTAG00000014024	-0.098581	0.6986333	0.1557507
ENSBTAG00000014026	0.0090257	0.9805939	0.0085108
ENSBTAG00000014027	-0.955458	0.0446543	1.3501369
ENSBTAG00000014028	-0.34278	0.4542465	0.3427084
ENSBTAG00000014029	-0.107699	0.6776324	0.1690058
ENSBTAG00000014030	-0.302739	0.4365737	0.3599425
ENSBTAG00000014031	NA	NA	NA
ENSBTAG00000014032	0.0594266	0.8188905	0.0867742
ENSBTAG00000014034	NA	NA	NA
ENSBTAG00000014038	NA	NA	NA
ENSBTAG00000014041	-0.095998	0.7462741	0.1271016
ENSBTAG00000014042	NA	NA	NA
ENSBTAG00000014043	0.2341466	0.3467958	0.4599261
ENSBTAG00000014044	-0.069609	0.782665	0.1064241

ENSBTAG00000014046	NA	NA	NA
ENSBTAG00000014048	0.1590126	0.7151337	0.1456128
ENSBTAG00000014051	-0.384093	0.4783456	0.3202582
ENSBTAG00000014056	NA	NA	NA
ENSBTAG00000014057	0.0507405	0.8626903	0.0641451
ENSBTAG00000014058	-0.552251	0.1527986	0.8158806
ENSBTAG00000014059	-0.119237	0.687472	0.162745
ENSBTAG00000014060	0.1846419	0.5150426	0.2881569
ENSBTAG00000014062	NA	NA	NA
ENSBTAG00000014063	NA	NA	NA
ENSBTAG00000014064	NA	NA	NA
ENSBTAG00000014068	-0.190377	0.45402	0.342925
ENSBTAG00000014069	0.490119	0.1198705	0.9212877
ENSBTAG00000014072	NA	NA	NA
ENSBTAG00000014074	-0.438092	0.1188299	0.9250744
ENSBTAG00000014075	NA	NA	NA
ENSBTAG00000014076	0.5491976	0.1474655	0.8313095
ENSBTAG00000014078	NA	NA	NA
ENSBTAG00000014079	0.07389	0.76719	0.1150971
ENSBTAG00000014080	0.1248509	0.6641557	0.1777301
ENSBTAG00000014081	0.1902478	0.4753433	0.3229926
ENSBTAG00000014082	NA	NA	NA
ENSBTAG00000014083	0.732646	0.0514251	1.2888247
ENSBTAG00000014087	-0.00569	0.990889	0.003975
ENSBTAG00000014088	0.5220256	0.4778712	0.3206891
ENSBTAG00000014089	-0.4143	0.346577	0.4602003
ENSBTAG00000014090	-0.497898	0.1984989	0.7022419
ENSBTAG00000014091	0.1863362	0.5477594	0.2614102
ENSBTAG00000014092	NA	NA	NA
ENSBTAG00000014093	0.1831257	0.4798245	0.3189176
ENSBTAG00000014095	-0.190566	0.5808522	0.2359344
ENSBTAG00000014096	-0.126801	0.6915086	0.1602024
ENSBTAG00000014099	-0.314967	0.2096633	0.6784777
ENSBTAG00000014100	NA	NA	NA
ENSBTAG00000014101	-0.179871	0.5291699	0.2764049
ENSBTAG00000014102	0.2264227	0.4544499	0.342514
ENSBTAG00000014103	NA	NA	NA
ENSBTAG00000014104	NA	NA	NA
ENSBTAG00000014105	0.0959164	0.7188428	0.1433661
ENSBTAG00000014106	0.0854817	0.7484794	0.1258201
ENSBTAG00000014111	0.1061914	0.6760515	0.1700202
ENSBTAG00000014112	-0.124186	0.6177609	0.2091796
ENSBTAG00000014113	NA	NA	NA
ENSBTAG00000014114	-0.174945	0.674439	0.1710573
ENSBTAG00000014117	0.2593604	0.4140316	0.3829665
ENSBTAG00000014118	0.163406	0.6024628	0.2200698
ENSBTAG00000014119	-0.143958	0.6606356	0.180038
ENSBTAG00000014122	0.0599382	0.8409792	0.0752147
ENSBTAG00000014123	0.3570923	0.224428	0.6489229
ENSBTAG00000014124	-0.476274	0.1403048	0.8529276
ENSBTAG00000014126	0.0382011	0.9340855	0.0296134
ENSBTAG00000014127	-0.356186	0.4549392	0.3420467
ENSBTAG00000014129	-0.091451	0.7222141	0.141334
ENSBTAG00000014130	-0.029003	0.9195156	0.0364409
ENSBTAG00000014132	0.6620608	0.1718481	0.7648551
ENSBTAG00000014135	-0.286016	0.3996284	0.3983437
ENSBTAG00000014136	-0.708907	0.0649214	1.1876123
ENSBTAG00000014137	0.3845093	0.3600158	0.4436785

ENSBTAG00000014138	-0.336901	0.2357099	0.6276221
ENSBTAG00000014140	-0.227012	0.4964	0.3041682
ENSBTAG00000014141	NA	NA	NA
ENSBTAG00000014143	1.0475139	0.0002729	3.5640601
ENSBTAG00000014146	-0.21708	0.4226974	0.3739704
ENSBTAG00000014150	-0.116136	0.7560881	0.1214276
ENSBTAG00000014151	-0.783111	0.0034558	2.4614498
ENSBTAG00000014153	0.2954696	0.3587212	0.4452429
ENSBTAG00000014154	0.1376769	0.8239219	0.084114
ENSBTAG00000014156	-0.553078	0.1681781	0.7742307
ENSBTAG00000014158	0.0601759	0.8116767	0.0906169
ENSBTAG00000014159	-0.357093	0.2363457	0.6264524
ENSBTAG00000014161	0.0751227	0.7674877	0.1149286
ENSBTAG00000014162	NA	NA	NA
ENSBTAG00000014166	0.0034593	0.9901957	0.0042789
ENSBTAG00000014167	0.291191	0.3415514	0.4665439
ENSBTAG00000014169	NA	NA	NA
ENSBTAG00000014170	-0.071414	0.8089898	0.0920569
ENSBTAG00000014171	-0.162633	0.5418126	0.2661509
ENSBTAG00000014172	0.2017833	0.6797291	0.1676642
ENSBTAG00000014175	-0.144401	0.5564903	0.2545424
ENSBTAG00000014176	0.1308416	0.7266639	0.1386664
ENSBTAG00000014177	NA	NA	NA
ENSBTAG00000014178	0.3621293	0.3123193	0.5054011
ENSBTAG00000014179	NA	NA	NA
ENSBTAG00000014181	-0.205073	0.4550661	0.3419255
ENSBTAG00000014182	0.229596	0.3691896	0.4327505
ENSBTAG00000014186	-0.018307	0.9513991	0.0216372
ENSBTAG00000014187	NA	NA	NA
ENSBTAG00000014189	-0.493454	0.2922707	0.5342147
ENSBTAG00000014191	0.2120802	0.4633182	0.3341206
ENSBTAG00000014192	NA	NA	NA
ENSBTAG00000014194	NA	NA	NA
ENSBTAG00000014197	0.1512023	0.8334557	0.0791175
ENSBTAG00000014199	NA	NA	NA
ENSBTAG00000014202	0.558188	0.1028754	0.9876883
ENSBTAG00000014204	-0.248928	0.3400312	0.4684812
ENSBTAG00000014205	-0.168399	0.5056223	0.2961738
ENSBTAG00000014207	0.9561354	0.0135753	1.8672503
ENSBTAG00000014208	0.1528505	0.5522178	0.2578896
ENSBTAG00000014211	NA	NA	NA
ENSBTAG00000014215	-0.085453	0.8117119	0.0905981
ENSBTAG00000014217	0.3719797	0.4633487	0.334092
ENSBTAG00000014218	-0.588659	0.247285	0.6068022
ENSBTAG00000014220	NA	NA	NA
ENSBTAG00000014221	NA	NA	NA
ENSBTAG00000014223	NA	NA	NA
ENSBTAG00000014224	NA	NA	NA
ENSBTAG00000014225	NA	NA	NA
ENSBTAG00000014226	0.0972581	0.7160229	0.1450731
ENSBTAG00000014227	-0.309002	0.2242183	0.649329
ENSBTAG00000014228	NA	NA	NA
ENSBTAG00000014229	NA	NA	NA
ENSBTAG00000014230	-0.24115	0.5825203	0.234689
ENSBTAG00000014232	NA	NA	NA
ENSBTAG00000014233	-0.068032	0.7829767	0.1062511
ENSBTAG00000014235	-0.034216	0.9085235	0.0416639
ENSBTAG00000014237	-0.237875	0.5848102	0.2329851

ENSBTAG00000014238	-0.210123	0.4051565	0.3923772
ENSBTAG00000014239	NA	NA	NA
ENSBTAG00000014246	NA	NA	NA
ENSBTAG00000014247	NA	NA	NA
ENSBTAG00000014248	-0.02506	0.9376184	0.0279739
ENSBTAG00000014249	-0.22327	0.4165788	0.3803028
ENSBTAG00000014250	0.1378185	0.7242685	0.1401004
ENSBTAG00000014251	NA	NA	NA
ENSBTAG00000014252	0.3914412	0.2301144	0.6380562
ENSBTAG00000014253	0.3282107	0.3383257	0.470665
ENSBTAG00000014255	-0.12845	0.5981152	0.2232151
ENSBTAG00000014259	NA	NA	NA
ENSBTAG00000014261	-0.243715	0.3276367	0.4846074
ENSBTAG00000014262	-0.271192	0.2956986	0.5291507
ENSBTAG00000014265	0.0664879	0.8477949	0.0717092
ENSBTAG00000014267	-0.398859	0.1567266	0.8048572
ENSBTAG00000014269	0.2807467	0.4888058	0.3108636
ENSBTAG00000014270	0.2682157	0.570974	0.2433836
ENSBTAG00000014272	-0.345816	0.1930239	0.714389
ENSBTAG00000014273	0.1753833	0.570227	0.2439522
ENSBTAG00000014278	0.1451157	0.7026106	0.1532853
ENSBTAG00000014284	-0.728832	0.0071895	2.1433015
ENSBTAG00000014286	0.2153009	0.5707149	0.2435808
ENSBTAG00000014289	-0.322395	0.1956596	0.7084989
ENSBTAG00000014291	NA	NA	NA
ENSBTAG00000014294	-0.258314	0.2998873	0.523042
ENSBTAG00000014295	-0.331955	0.2205057	0.6565802
ENSBTAG00000014296	NA	NA	NA
ENSBTAG00000014297	0.0749357	0.8533643	0.0688655
ENSBTAG00000014299	NA	NA	NA
ENSBTAG00000014300	NA	NA	NA
ENSBTAG00000014302	-0.075443	0.7614432	0.1183625
ENSBTAG00000014304	-0.137097	0.6914486	0.1602401
ENSBTAG00000014306	0.2747571	0.3675593	0.4346726
ENSBTAG00000014310	-0.018176	0.9454113	0.0243792
ENSBTAG00000014312	NA	NA	NA
ENSBTAG00000014313	0.263283	0.5987799	0.2227328
ENSBTAG00000014314	0.516493	0.127695	0.8938262
ENSBTAG00000014315	NA	NA	NA
ENSBTAG00000014316	-0.490064	0.0891733	1.0497652
ENSBTAG00000014318	0.2295004	0.3912819	0.4075103
ENSBTAG00000014319	0.3137731	0.4612389	0.3360741
ENSBTAG00000014320	-0.111711	0.6533521	0.1848527
ENSBTAG00000014322	0.2213428	0.59343	0.2266305
ENSBTAG00000014324	0.177869	0.484498	0.3147081
ENSBTAG00000014325	0.4605703	0.2735644	0.5629404
ENSBTAG00000014326	NA	NA	NA
ENSBTAG00000014328	-0.624679	0.0661273	1.1796192
ENSBTAG00000014331	-0.017399	0.946644	0.0238133
ENSBTAG00000014332	NA	NA	NA
ENSBTAG00000014333	0.4561869	0.2920009	0.5346158
ENSBTAG00000014334	-0.083972	0.7578356	0.120425
ENSBTAG00000014335	NA	NA	NA
ENSBTAG00000014336	0.0861064	0.8086423	0.0922436
ENSBTAG00000014337	-0.015583	0.9516337	0.0215302
ENSBTAG00000014339	0.30462	0.340693	0.4676368
ENSBTAG00000014340	-0.957582	0.1656712	0.7807531
ENSBTAG00000014343	0.7291245	0.1211629	0.9166302

ENSBTAG00000014345	NA	NA	NA
ENSBTAG00000014349	0.1537247	0.6079025	0.2161661
ENSBTAG00000014351	0.3792083	0.205703	0.6867593
ENSBTAG00000014353	-0.113995	0.641166	0.1930295
ENSBTAG00000014354	0.4263586	0.2240867	0.649584
ENSBTAG00000014355	NA	NA	NA
ENSBTAG00000014357	-0.526708	0.0448882	1.3478682
ENSBTAG00000014358	-0.312744	0.4616599	0.3356778
ENSBTAG00000014361	0.2682706	0.3847871	0.4147795
ENSBTAG00000014366	0.1005246	0.7392333	0.1312185
ENSBTAG00000014367	-0.513809	0.2091315	0.6795806
ENSBTAG00000014368	0.3969898	0.4376081	0.3589146
ENSBTAG00000014370	-1.11847	0.0193909	1.7124013
ENSBTAG00000014371	0.4896503	0.2094199	0.678982
ENSBTAG00000014372	-0.101593	0.6917011	0.1600816
ENSBTAG00000014373	-0.173718	0.5763775	0.239293
ENSBTAG00000014374	0.5535225	0.2158491	0.6658497
ENSBTAG00000014375	NA	NA	NA
ENSBTAG00000014376	-0.328821	0.3514234	0.4541694
ENSBTAG00000014377	-0.131788	0.6510673	0.1863741
ENSBTAG00000014380	0.090095	0.7745762	0.1109359
ENSBTAG00000014381	NA	NA	NA
ENSBTAG00000014382	0.4101458	0.3575761	0.4466316
ENSBTAG00000014387	-0.404095	0.1042767	0.9818129
ENSBTAG00000014388	0.164511	0.518087	0.2855973
ENSBTAG00000014389	0.2869125	0.3819349	0.4180107
ENSBTAG00000014390	0.7261755	0.0928721	1.0321146
ENSBTAG00000014392	-0.263757	0.2799438	0.5529292
ENSBTAG00000014393	0.2444361	0.4627767	0.3346285
ENSBTAG00000014396	0.2682177	0.4334925	0.3630185
ENSBTAG00000014397	NA	NA	NA
ENSBTAG00000014399	-0.540929	0.0680217	1.1673524
ENSBTAG00000014400	NA	NA	NA
ENSBTAG00000014401	0.0035204	0.9903535	0.0042098
ENSBTAG00000014402	-0.025639	0.9486566	0.022891
ENSBTAG00000014405	0.1190686	0.6963798	0.1571538
ENSBTAG00000014406	0.0126674	0.9730949	0.0118448
ENSBTAG00000014407	-0.128203	0.7338544	0.1343901
ENSBTAG00000014411	-0.04641	0.8527387	0.069184
ENSBTAG00000014412	NA	NA	NA
ENSBTAG00000014415	-0.122806	0.7745224	0.110966
ENSBTAG00000014417	0.2270318	0.371367	0.4301967
ENSBTAG00000014418	NA	NA	NA
ENSBTAG00000014420	NA	NA	NA
ENSBTAG00000014421	-0.273491	0.3413499	0.4668002
ENSBTAG00000014422	-0.175785	0.4796395	0.3190851
ENSBTAG00000014423	-0.08196	0.7490192	0.125507
ENSBTAG00000014424	NA	NA	NA
ENSBTAG00000014426	0.0489765	0.8782313	0.0563911
ENSBTAG00000014429	0.139816	0.5728091	0.2419901
ENSBTAG00000014430	-0.270347	0.5112346	0.2913798
ENSBTAG00000014432	0.1716177	0.6764749	0.1697483
ENSBTAG00000014433	-0.300768	0.2491318	0.6035708
ENSBTAG00000014434	0.275348	0.5948475	0.2255944
ENSBTAG00000014435	NA	NA	NA
ENSBTAG00000014438	0.3438558	0.2670389	0.5734255
ENSBTAG00000014439	NA	NA	NA
ENSBTAG00000014440	0.1582765	0.5516066	0.2583706

ENSBTAG00000014441	0.1420846	0.8127438	0.0900463
ENSBTAG00000014447	0.0740596	0.8453549	0.0729609
ENSBTAG00000014448	-0.587605	0.219618	0.6583321
ENSBTAG00000014449	-0.329465	0.6886573	0.1619968
ENSBTAG00000014450	-0.074074	0.772501	0.1121009
ENSBTAG00000014451	0.2034866	0.4860936	0.3132801
ENSBTAG00000014453	-0.404765	0.4143137	0.3826707
ENSBTAG00000014454	0.3049874	0.4554557	0.3415538
ENSBTAG00000014455	0.1113905	0.7190949	0.1432138
ENSBTAG00000014456	NA	NA	NA
ENSBTAG00000014458	-0.074888	0.8274021	0.0822834
ENSBTAG00000014459	-0.120933	0.7559009	0.1215351
ENSBTAG00000014460	-0.020795	0.9362497	0.0286083
ENSBTAG00000014461	-0.384819	0.1578998	0.8016185
ENSBTAG00000014463	-0.435224	0.0972444	1.0121353
ENSBTAG00000014465	-0.568332	0.1364693	0.864965
ENSBTAG00000014466	-0.90974	0.0441689	1.3548836
ENSBTAG00000014467	NA	NA	NA
ENSBTAG00000014468	-0.023257	0.9576765	0.0187812
ENSBTAG00000014469	-0.169508	0.5283971	0.2770396
ENSBTAG00000014470	NA	NA	NA
ENSBTAG00000014471	0.550682	0.0784234	1.1055542
ENSBTAG00000014472	NA	NA	NA
ENSBTAG00000014474	-0.187993	0.5129295	0.2899423
ENSBTAG00000014476	0.025837	0.9546764	0.0201438
ENSBTAG00000014478	0.6676344	0.0536116	1.2707414
ENSBTAG00000014479	0.629794	0.0589911	1.2292132
ENSBTAG00000014482	-0.241175	0.3390968	0.4696763
ENSBTAG00000014484	NA	NA	NA
ENSBTAG00000014486	NA	NA	NA
ENSBTAG00000014488	NA	NA	NA
ENSBTAG00000014490	0.335135	0.1925864	0.7153743
ENSBTAG00000014491	NA	NA	NA
ENSBTAG00000014492	-0.473646	0.0717836	1.1439747
ENSBTAG00000014493	0.040527	0.9016944	0.0449406
ENSBTAG00000014494	0.3741809	0.3491318	0.4570106
ENSBTAG00000014495	-0.085429	0.8020501	0.0957985
ENSBTAG00000014496	NA	NA	NA
ENSBTAG00000014497	NA	NA	NA
ENSBTAG00000014498	0.3830553	0.297509	0.5264999
ENSBTAG00000014501	-0.360159	0.1500606	0.8237333
ENSBTAG00000014502	0.058916	0.8966054	0.0473987
ENSBTAG00000014503	0.3639174	0.3229911	0.4908095
ENSBTAG00000014505	0.1088706	0.6701465	0.1738303
ENSBTAG00000014506	0.8768436	0.027189	1.5656063
ENSBTAG00000014508	0.6341022	0.0817128	1.0877102
ENSBTAG00000014511	0.103777	0.7955444	0.0993356
ENSBTAG00000014512	0.2572642	0.4671424	0.3305507
ENSBTAG00000014514	NA	NA	NA
ENSBTAG00000014515	-0.152662	0.5945067	0.2258432
ENSBTAG00000014517	NA	NA	NA
ENSBTAG00000014518	0.014392	0.9550263	0.0199847
ENSBTAG00000014520	-0.212234	0.4047443	0.3928193
ENSBTAG00000014521	0.3218766	0.3019995	0.5199937
ENSBTAG00000014522	0.0945842	0.7543602	0.1224212
ENSBTAG00000014523	NA	NA	NA
ENSBTAG00000014525	NA	NA	NA
ENSBTAG00000014526	0.1652666	0.7040791	0.1523785

ENSBTAG00000014529	0.7151074	0.2432467	0.6139531
ENSBTAG00000014530	NA	NA	NA
ENSBTAG00000014533	NA	NA	NA
ENSBTAG00000014534	0.5796224	0.0646009	1.1897615
ENSBTAG00000014536	NA	NA	NA
ENSBTAG00000014537	NA	NA	NA
ENSBTAG00000014538	0.6331757	0.0899491	1.046003
ENSBTAG00000014540	0.5202681	0.0798615	1.0976625
ENSBTAG00000014541	-0.290848	0.351288	0.4543367
ENSBTAG00000014543	0.4807423	0.2330754	0.6325035
ENSBTAG00000014546	NA	NA	NA
ENSBTAG00000014547	-0.512625	0.0542406	1.2656753
ENSBTAG00000014548	-0.040547	0.8792146	0.0559051
ENSBTAG00000014549	-0.099505	0.7184689	0.1435921
ENSBTAG00000014550	0.1614068	0.6678372	0.1753294
ENSBTAG00000014551	0.0528004	0.9062819	0.0427367
ENSBTAG00000014553	0.4569071	0.0897709	1.0468646
ENSBTAG00000014554	NA	NA	NA
ENSBTAG00000014555	NA	NA	NA
ENSBTAG00000014558	0.0261815	0.9162186	0.0380009
ENSBTAG00000014560	1.9221697	9.85E-05	4.0067256
ENSBTAG00000014561	0.0255152	0.9225414	0.0350141
ENSBTAG00000014564	-0.118515	0.8269496	0.082521
ENSBTAG00000014567	0.2013595	0.4811856	0.3176874
ENSBTAG00000014574	NA	NA	NA
ENSBTAG00000014575	-0.120022	0.7527794	0.1233323
ENSBTAG00000014576	0.5525169	0.0394331	1.4041391
ENSBTAG00000014579	0.0577028	0.8151363	0.0887698
ENSBTAG00000014581	-0.33015	0.2825191	0.5489522
ENSBTAG00000014583	-0.163543	0.5488115	0.2605768
ENSBTAG00000014585	NA	NA	NA
ENSBTAG00000014587	NA	NA	NA
ENSBTAG00000014588	0.2376369	0.4974417	0.3032579
ENSBTAG00000014589	NA	NA	NA
ENSBTAG00000014592	0.2350757	0.4417973	0.3547769
ENSBTAG00000014593	0.4029654	0.2500649	0.6019473
ENSBTAG00000014595	-0.108093	0.8043128	0.094575
ENSBTAG00000014596	-0.122422	0.7689963	0.1140757
ENSBTAG00000014597	NA	NA	NA
ENSBTAG00000014598	-0.341575	0.1997098	0.6996005
ENSBTAG00000014599	NA	NA	NA
ENSBTAG00000014600	NA	NA	NA
ENSBTAG00000014601	-0.227697	0.4105026	0.3866841
ENSBTAG00000014602	-1.214225	0.0013824	2.8593521
ENSBTAG00000014603	-0.288544	0.5750495	0.2402948
ENSBTAG00000014605	-0.22694	0.5372826	0.2697972
ENSBTAG00000014606	NA	NA	NA
ENSBTAG00000014607	-0.069414	0.8327919	0.0794635
ENSBTAG00000014608	-0.145423	0.6355694	0.196837
ENSBTAG00000014609	-0.182517	0.4879038	0.3116658
ENSBTAG00000014610	0.0885069	0.7512175	0.1242343
ENSBTAG00000014611	0.0724041	0.8631445	0.0639165
ENSBTAG00000014612	-0.091863	0.7926469	0.1009202
ENSBTAG00000014614	0.3157483	0.2610399	0.583293
ENSBTAG00000014615	-0.077568	0.8654049	0.0627806
ENSBTAG00000014616	-0.270664	0.3608429	0.4426818
ENSBTAG00000014619	0.1010913	0.7473586	0.126471
ENSBTAG00000014623	-0.429073	0.3820411	0.4178899

ENSBTAG00000014626	0.0444449	0.8596264	0.0656902
ENSBTAG00000014628	NA	NA	NA
ENSBTAG00000014633	-0.026264	0.9512407	0.0217096
ENSBTAG00000014636	-0.031701	0.9155577	0.0383143
ENSBTAG00000014642	0.5976429	0.1029533	0.9873599
ENSBTAG00000014643	0.0029195	0.9912794	0.0038039
ENSBTAG00000014644	-0.17879	0.5863757	0.231824
ENSBTAG00000014645	NA	NA	NA
ENSBTAG00000014646	-0.428588	0.1911616	0.7185993
ENSBTAG00000014648	-0.137022	0.5919518	0.2277136
ENSBTAG00000014649	0.585214	0.0517306	1.2862526
ENSBTAG00000014650	0.1655311	0.6151673	0.2110067
ENSBTAG00000014651	NA	NA	NA
ENSBTAG00000014653	0.4698438	0.1260672	0.8993978
ENSBTAG00000014655	NA	NA	NA
ENSBTAG00000014659	0.0451496	0.9322481	0.0304685
ENSBTAG00000014660	0.1953416	0.5581882	0.2532194
ENSBTAG00000014661	0.1649207	0.6936699	0.1588471
ENSBTAG00000014665	-0.191912	0.547711	0.2614486
ENSBTAG00000014666	NA	NA	NA
ENSBTAG00000014667	-0.392978	0.161695	0.7913033
ENSBTAG00000014668	0.3984624	0.3556008	0.4490372
ENSBTAG00000014669	-0.422474	0.3299035	0.4816131
ENSBTAG00000014670	NA	NA	NA
ENSBTAG00000014674	NA	NA	NA
ENSBTAG00000014675	0.249071	0.3953799	0.4029854
ENSBTAG00000014676	NA	NA	NA
ENSBTAG00000014677	-0.136521	0.6221233	0.2061235
ENSBTAG00000014679	-0.132143	0.6027874	0.2198358
ENSBTAG00000014680	-0.147941	0.7171857	0.1443684
ENSBTAG00000014682	NA	NA	NA
ENSBTAG00000014685	-0.169755	0.5184645	0.285281
ENSBTAG00000014687	-0.620977	0.174993	0.7569793
ENSBTAG00000014689	-0.434	0.1463455	0.8346206
ENSBTAG00000014691	-0.391763	0.120919	0.9175054
ENSBTAG00000014692	NA	NA	NA
ENSBTAG00000014693	0.2886052	0.5270197	0.2781731
ENSBTAG00000014694	0.0488236	0.8609424	0.0650259
ENSBTAG00000014695	-0.104811	0.6864072	0.1634181
ENSBTAG00000014696	NA	NA	NA
ENSBTAG00000014697	0.1266089	0.6196834	0.2078301
ENSBTAG00000014698	0.556825	0.2580063	0.5883697
ENSBTAG00000014699	NA	NA	NA
ENSBTAG00000014700	-0.069775	0.7746723	0.110882
ENSBTAG00000014705	0.0930901	0.7925345	0.1009818
ENSBTAG00000014707	0.7238003	0.2230895	0.6515208
ENSBTAG00000014710	0.1219558	0.7434626	0.1287409
ENSBTAG00000014711	-0.014892	0.9816718	0.0080337
ENSBTAG00000014712	0.0236277	0.9314012	0.0308632
ENSBTAG00000014713	-0.565922	0.2742665	0.5618273
ENSBTAG00000014714	0.1854275	0.4909385	0.3089729
ENSBTAG00000014718	-0.800135	0.097242	1.012146
ENSBTAG00000014719	-0.032699	0.9024653	0.0445695
ENSBTAG00000014721	NA	NA	NA
ENSBTAG00000014722	-0.005713	0.9876354	0.0054033
ENSBTAG00000014724	0.0342914	0.8940538	0.0486363
ENSBTAG00000014725	NA	NA	NA
ENSBTAG00000014726	-0.317034	0.2102559	0.6772518

ENSBTAG00000014727	0.1956273	0.5730786	0.2417858
ENSBTAG00000014728	0.0900912	0.7907722	0.1019486
ENSBTAG00000014729	-0.155571	0.5538653	0.2565958
ENSBTAG00000014730	0.0053215	0.9850111	0.0065589
ENSBTAG00000014731	-0.313758	0.2449347	0.6109497
ENSBTAG00000014733	0.4113005	0.1775969	0.7505646
ENSBTAG00000014734	0.0707066	0.7731018	0.1117633
ENSBTAG00000014735	NA	NA	NA
ENSBTAG00000014737	0.1354074	0.7421102	0.1295316
ENSBTAG00000014738	NA	NA	NA
ENSBTAG00000014742	0.332708	0.390832	0.4080099
ENSBTAG00000014744	0.2548043	0.402176	0.3955838
ENSBTAG00000014746	NA	NA	NA
ENSBTAG00000014749	0.1248546	0.8261397	0.0829465
ENSBTAG00000014750	-0.571488	0.0813601	1.0895884
ENSBTAG00000014751	0.2121026	0.5579521	0.2534031
ENSBTAG00000014752	0.1798407	0.5236154	0.2809876
ENSBTAG00000014758	NA	NA	NA
ENSBTAG00000014759	0.1608168	0.5285693	0.2768981
ENSBTAG00000014762	NA	NA	NA
ENSBTAG00000014764	0.0807918	0.8114686	0.0907283
ENSBTAG00000014766	0.1315881	0.6040008	0.2189625
ENSBTAG00000014768	0.0148265	0.9613833	0.0171034
ENSBTAG00000014769	0.0519434	0.8668495	0.0620563
ENSBTAG00000014771	0.4954325	0.2452819	0.6103345
ENSBTAG00000014772	-0.089358	0.7626708	0.1176629
ENSBTAG00000014773	-0.180555	0.5750383	0.2403032
ENSBTAG00000014774	NA	NA	NA
ENSBTAG00000014775	0.3142952	0.3191	0.4960732
ENSBTAG00000014777	0.7194853	0.1064471	0.9728662
ENSBTAG00000014779	-0.086528	0.7416248	0.1298158
ENSBTAG00000014782	0.1521203	0.5857282	0.2323039
ENSBTAG00000014784	NA	NA	NA
ENSBTAG00000014786	0.0282345	0.9140516	0.0390293
ENSBTAG00000014788	NA	NA	NA
ENSBTAG00000014790	0.6695851	0.1206355	0.9185247
ENSBTAG00000014791	0.3825	0.4932709	0.3069145
ENSBTAG00000014792	-0.186338	0.4889581	0.3107284
ENSBTAG00000014793	0.2422988	0.6168268	0.2098368
ENSBTAG00000014794	0.3470722	0.2122874	0.6730759
ENSBTAG00000014797	0.1387936	0.6999214	0.1549507
ENSBTAG00000014798	NA	NA	NA
ENSBTAG00000014799	0.3338566	0.4355375	0.3609745
ENSBTAG00000014803	NA	NA	NA
ENSBTAG00000014804	-0.136435	0.619437	0.2080029
ENSBTAG00000014805	-0.208245	0.5207881	0.283339
ENSBTAG00000014806	0.3395212	0.4031519	0.3945313
ENSBTAG00000014807	-0.29202	0.2565937	0.590754
ENSBTAG00000014809	-0.209047	0.4096287	0.3876096
ENSBTAG00000014813	NA	NA	NA
ENSBTAG00000014814	0.1222287	0.6579363	0.1818161
ENSBTAG00000014818	0.0057638	0.9883726	0.0050793
ENSBTAG00000014820	0.1280272	0.6038305	0.2190849
ENSBTAG00000014821	NA	NA	NA
ENSBTAG00000014822	0.0133773	0.9634063	0.0161905
ENSBTAG00000014823	-0.095007	0.7224959	0.1411646
ENSBTAG00000014824	0.4696106	0.1412209	0.8501012
ENSBTAG00000014825	0.0946451	0.7617444	0.1181907

ENSBTAG00000014826	-0.250798	0.3461932	0.4606815
ENSBTAG00000014827	0.0938284	0.7011859	0.1541668
ENSBTAG00000014828	0.4019634	0.3756918	0.4251683
ENSBTAG00000014829	-0.157224	0.6891345	0.161696
ENSBTAG00000014830	NA	NA	NA
ENSBTAG00000014831	-0.685196	0.02152	1.6671582
ENSBTAG00000014832	0.2136492	0.5911171	0.2283265
ENSBTAG00000014834	NA	NA	NA
ENSBTAG00000014835	0.4049196	0.1639181	0.785373
ENSBTAG00000014838	0.1054322	0.7029788	0.1530578
ENSBTAG00000014840	0.6278181	0.0614755	1.2112981
ENSBTAG00000014841	0.5109682	0.1312745	0.8818195
ENSBTAG00000014843	-0.126753	0.6154504	0.210807
ENSBTAG00000014847	NA	NA	NA
ENSBTAG00000014848	NA	NA	NA
ENSBTAG00000014849	0.0278513	0.9428539	0.0255556
ENSBTAG00000014850	0.2134241	0.578525	0.2376778
ENSBTAG00000014855	-0.276765	0.4216771	0.37502
ENSBTAG00000014857	0.1587232	0.7389611	0.1313784
ENSBTAG00000014858	-0.179204	0.5454599	0.2632372
ENSBTAG00000014859	NA	NA	NA
ENSBTAG00000014861	0.8007635	0.0143794	1.8422584
ENSBTAG00000014862	-0.32189	0.4824614	0.3165374
ENSBTAG00000014863	-0.298011	0.2550966	0.5932954
ENSBTAG00000014871	NA	NA	NA
ENSBTAG00000014872	-0.105913	0.6859467	0.1637097
ENSBTAG00000014873	-0.14394	0.6315076	0.1996214
ENSBTAG00000014874	-0.474806	0.3291362	0.4826244
ENSBTAG00000014877	0.1693827	0.5972024	0.2238785
ENSBTAG00000014878	0.0753861	0.796436	0.0988491
ENSBTAG00000014879	0.5453067	0.1335635	0.8743123
ENSBTAG00000014880	0.1051435	0.7537456	0.1227752
ENSBTAG00000014883	0.1599535	0.5372043	0.2698605
ENSBTAG00000014884	-0.378605	0.1577049	0.8021549
ENSBTAG00000014885	0.1417027	0.6613996	0.1795361
ENSBTAG00000014886	0.1954305	0.5635193	0.2490912
ENSBTAG00000014889	0.3533531	0.269369	0.5696523
ENSBTAG00000014890	NA	NA	NA
ENSBTAG00000014891	0.2800094	0.2656388	0.5757085
ENSBTAG00000014897	NA	NA	NA
ENSBTAG00000014898	-0.573546	0.1760323	0.7544077
ENSBTAG00000014899	NA	NA	NA
ENSBTAG00000014900	0.5880071	0.1647899	0.7830694
ENSBTAG00000014903	-0.058449	0.9037859	0.0439345
ENSBTAG00000014904	-0.611898	0.0420918	1.3758028
ENSBTAG00000014906	0.7501959	0.0321035	1.4934476
ENSBTAG00000014907	-0.169927	0.5368946	0.270111
ENSBTAG00000014908	NA	NA	NA
ENSBTAG00000014910	NA	NA	NA
ENSBTAG00000014911	-0.203616	0.7631036	0.1174165
ENSBTAG00000014912	0.209587	0.6238739	0.2049032
ENSBTAG00000014913	-0.309384	0.4998284	0.3011791
ENSBTAG00000014915	0.8069422	0.0659134	1.1810265
ENSBTAG00000014916	NA	NA	NA
ENSBTAG00000014917	-0.06655	0.7955527	0.099331
ENSBTAG00000014918	-0.137038	0.6974018	0.156517
ENSBTAG00000014920	NA	NA	NA
ENSBTAG00000014921	NA	NA	NA

ENSBTAG00000014922	-0.022718	0.9654984	0.0152485
ENSBTAG00000014923	0.2852651	0.3909057	0.407928
ENSBTAG00000014926	0.1676304	0.7007402	0.154443
ENSBTAG00000014927	-0.318659	0.2216166	0.6543977
ENSBTAG00000014930	-0.40063	0.1720497	0.7643461
ENSBTAG00000014931	-0.027077	0.913808	0.039145
ENSBTAG00000014932	NA	NA	NA
ENSBTAG00000014933	0.2497712	0.3693028	0.4326174
ENSBTAG00000014934	-0.204829	0.4874622	0.3120591
ENSBTAG00000014936	0.042474	0.875455	0.0577662
ENSBTAG00000014940	NA	NA	NA
ENSBTAG00000014943	0.1112798	0.6613066	0.1795972
ENSBTAG00000014944	NA	NA	NA
ENSBTAG00000014945	NA	NA	NA
ENSBTAG00000014947	0.3115981	0.294808	0.5304607
ENSBTAG00000014948	NA	NA	NA
ENSBTAG00000014949	NA	NA	NA
ENSBTAG00000014955	NA	NA	NA
ENSBTAG00000014956	0.0012528	0.9964209	0.0015572
ENSBTAG00000014958	-0.107818	0.7342438	0.1341597
ENSBTAG00000014960	-0.099709	0.6924794	0.1595932
ENSBTAG00000014964	0.0158353	0.9717852	0.0124297
ENSBTAG00000014966	0.2718003	0.3665651	0.4358488
ENSBTAG00000014967	NA	NA	NA
ENSBTAG00000014968	0.618573	0.2560026	0.5917557
ENSBTAG00000014969	NA	NA	NA
ENSBTAG00000014970	0.0022561	0.9935008	0.0028318
ENSBTAG00000014971	-0.27233	0.3065297	0.5135275
ENSBTAG00000014972	0.5270409	0.0810883	1.0910415
ENSBTAG00000014974	0.2516418	0.4534807	0.3434412
ENSBTAG00000014975	NA	NA	NA
ENSBTAG00000014979	0.2606977	0.4619402	0.3354142
ENSBTAG00000014981	0.0991578	0.8481399	0.0715325
ENSBTAG00000014982	NA	NA	NA
ENSBTAG00000014983	NA	NA	NA
ENSBTAG00000014984	-0.222657	0.4795398	0.3191753
ENSBTAG00000014990	NA	NA	NA
ENSBTAG00000014991	-0.058753	0.8267274	0.0826377
ENSBTAG00000014992	NA	NA	NA
ENSBTAG00000014995	0.1943428	0.6862346	0.1635274
ENSBTAG00000014996	0.4737262	0.3035224	0.5178093
ENSBTAG00000015000	0.0693103	0.7878903	0.1035342
ENSBTAG00000015001	NA	NA	NA
ENSBTAG00000015002	0.2990107	0.2688713	0.5704556
ENSBTAG00000015004	0.1186672	0.6450001	0.1904402
ENSBTAG00000015005	0.00056	0.9990441	0.0004153
ENSBTAG00000015006	0.0019968	0.9943198	0.0024739
ENSBTAG00000015007	-0.075035	0.7794115	0.1082332
ENSBTAG00000015008	-0.223213	0.6441195	0.1910335
ENSBTAG00000015009	-0.043503	0.8677173	0.0616217
ENSBTAG00000015010	-0.135896	0.5810112	0.2358155
ENSBTAG00000015011	0.6777417	0.1187606	0.9253275
ENSBTAG00000015012	-0.116553	0.8165724	0.0880053
ENSBTAG00000015013	0.2403039	0.5284302	0.2770124
ENSBTAG00000015014	-0.039275	0.9512606	0.0217005
ENSBTAG00000015015	0.534176	0.1268191	0.8968155
ENSBTAG00000015016	0.3804653	0.1634314	0.7866644
ENSBTAG00000015017	NA	NA	NA

ENSBTAG00000015018	-0.290693	0.2524911	0.5977539
ENSBTAG00000015019	-0.371844	0.3245641	0.4886995
ENSBTAG00000015022	-0.328348	0.2315809	0.6352973
ENSBTAG00000015023	-0.193181	0.4652927	0.3322738
ENSBTAG00000015024	0.1930459	0.4991804	0.3017425
ENSBTAG00000015025	-0.509153	0.1441646	0.8411413
ENSBTAG00000015026	-0.011883	0.9724742	0.0121219
ENSBTAG00000015027	NA	NA	NA
ENSBTAG00000015032	0.3360358	0.3848376	0.4147225
ENSBTAG00000015034	-0.020438	0.9372622	0.0281389
ENSBTAG00000015036	-0.426107	0.2552793	0.5929845
ENSBTAG00000015037	0.3034053	0.5578252	0.2535019
ENSBTAG00000015038	0.4951385	0.0491323	1.3086331
ENSBTAG00000015040	NA	NA	NA
ENSBTAG00000015041	-0.166068	0.5676395	0.2459274
ENSBTAG00000015042	0.3400894	0.1975788	0.7042597
ENSBTAG00000015043	0.3002323	0.3748289	0.4261669
ENSBTAG00000015044	-0.034878	0.9288176	0.0320696
ENSBTAG00000015046	NA	NA	NA
ENSBTAG00000015047	NA	NA	NA
ENSBTAG00000015049	0.2172134	0.3944537	0.404004
ENSBTAG00000015050	0.2366744	0.6001895	0.2217116
ENSBTAG00000015051	0.4327113	0.1992554	0.7005898
ENSBTAG00000015052	NA	NA	NA
ENSBTAG00000015053	NA	NA	NA
ENSBTAG00000015058	0.9192087	0.0610881	1.2140437
ENSBTAG00000015060	-0.344161	0.5573904	0.2538405
ENSBTAG00000015065	NA	NA	NA
ENSBTAG00000015066	0.0165516	0.9585177	0.0183999
ENSBTAG00000015069	-0.069811	0.7878173	0.1035745
ENSBTAG00000015072	0.1869546	0.6967801	0.1569042
ENSBTAG00000015073	0.1253716	0.6885969	0.162035
ENSBTAG00000015074	0.1419782	0.8168721	0.0878459
ENSBTAG00000015075	-0.252634	0.46591	0.331698
ENSBTAG00000015076	-0.079046	0.8052393	0.094075
ENSBTAG00000015077	0.1391721	0.6680335	0.1752017
ENSBTAG00000015080	NA	NA	NA
ENSBTAG00000015081	-0.326927	0.3784229	0.4220226
ENSBTAG00000015083	-0.16261	0.5431646	0.2650685
ENSBTAG00000015085	NA	NA	NA
ENSBTAG00000015086	-0.399414	0.4405714	0.3559837
ENSBTAG00000015087	NA	NA	NA
ENSBTAG00000015089	-0.360508	0.1745525	0.7580739
ENSBTAG00000015090	-0.058495	0.8425967	0.0743802
ENSBTAG00000015092	-0.102383	0.816995	0.0877806
ENSBTAG00000015093	NA	NA	NA
ENSBTAG00000015094	-0.761572	0.0273921	1.5623754
ENSBTAG00000015097	-0.093879	0.7820209	0.1067816
ENSBTAG00000015098	0.1473615	0.6958918	0.1574583
ENSBTAG00000015099	-0.244785	0.5310576	0.2748584
ENSBTAG00000015100	0.2737206	0.3870101	0.4122777
ENSBTAG00000015101	0.2486651	0.3829545	0.4168528
ENSBTAG00000015103	0.1977366	0.6644088	0.1775646
ENSBTAG00000015104	-0.197245	0.5791739	0.237191
ENSBTAG00000015105	-0.198398	0.4649998	0.3325473
ENSBTAG00000015106	-0.172093	0.7481698	0.1259998
ENSBTAG00000015107	0.0192436	0.9570856	0.0190492
ENSBTAG00000015108	0.6740456	0.0539891	1.2676936

ENSBTAG00000015109	0.3823933	0.2058752	0.6863959
ENSBTAG00000015112	-0.064696	0.7990397	0.0974317
ENSBTAG00000015113	NA	NA	NA
ENSBTAG00000015114	0.1816079	0.4601929	0.33706
ENSBTAG00000015115	-0.242447	0.5039556	0.2976077
ENSBTAG00000015116	0.1059144	0.6959902	0.1573969
ENSBTAG00000015117	-0.050131	0.8612917	0.0648497
ENSBTAG00000015118	NA	NA	NA
ENSBTAG00000015119	-0.975868	0.0036238	2.4408379
ENSBTAG00000015124	-0.075493	0.8096387	0.0917087
ENSBTAG00000015126	-0.036971	0.9394543	0.0271243
ENSBTAG00000015127	0.3709401	0.4613364	0.3359823
ENSBTAG00000015128	NA	NA	NA
ENSBTAG00000015129	NA	NA	NA
ENSBTAG00000015131	0.5935923	0.0238536	1.6224456
ENSBTAG00000015132	-0.289949	0.57238	0.2423156
ENSBTAG00000015133	NA	NA	NA
ENSBTAG00000015136	-0.141982	0.5696733	0.2443742
ENSBTAG00000015138	0.1388745	0.6321311	0.1991929
ENSBTAG00000015140	-0.083729	0.811841	0.090529
ENSBTAG00000015142	0.9561253	0.0439914	1.3566322
ENSBTAG00000015144	0.7183405	0.1018988	0.9918308
ENSBTAG00000015145	0.2307328	0.6026328	0.2199472
ENSBTAG00000015146	-0.068454	0.7864886	0.1043076
ENSBTAG00000015147	-0.061649	0.8474759	0.0718727
ENSBTAG00000015148	0.0269778	0.9343411	0.0294945
ENSBTAG00000015150	NA	NA	NA
ENSBTAG00000015151	-0.027527	0.9315063	0.0308142
ENSBTAG00000015154	0.1981395	0.4522487	0.3446227
ENSBTAG00000015155	0.4609142	0.1514138	0.8198346
ENSBTAG00000015156	-0.097284	0.7234564	0.1405876
ENSBTAG00000015157	NA	NA	NA
ENSBTAG00000015158	-0.034729	0.9099026	0.0410051
ENSBTAG00000015160	-0.350743	0.4621415	0.3352251
ENSBTAG00000015163	0.3364888	0.2109363	0.6758486
ENSBTAG00000015164	NA	NA	NA
ENSBTAG00000015165	0.6516325	0.1611351	0.7928099
ENSBTAG00000015166	-0.042756	0.9373487	0.0280988
ENSBTAG00000015169	-0.30933	0.2152265	0.6671042
ENSBTAG00000015170	NA	NA	NA
ENSBTAG00000015171	0.1809026	0.6474015	0.1888263
ENSBTAG00000015172	0.8412434	0.0133067	1.8759303
ENSBTAG00000015176	NA	NA	NA
ENSBTAG00000015177	-0.009503	0.9731975	0.011799
ENSBTAG00000015178	0.1428106	0.5694	0.2445825
ENSBTAG00000015180	NA	NA	NA
ENSBTAG00000015181	-0.100922	0.6991264	0.1554443
ENSBTAG00000015182	0.7435522	0.1287546	0.8902374
ENSBTAG00000015183	0.2355991	0.5682099	0.2454912
ENSBTAG00000015184	0.2167474	0.5890724	0.2298313
ENSBTAG00000015185	-0.147448	0.7321678	0.1353893
ENSBTAG00000015186	0.6093196	0.1163929	0.9340737
ENSBTAG00000015187	0.2297322	0.4541161	0.3428331
ENSBTAG00000015188	-0.181322	0.4891749	0.3105359
ENSBTAG00000015190	0.1850649	0.5761699	0.2394495
ENSBTAG00000015191	NA	NA	NA
ENSBTAG00000015192	0.3676942	0.382234	0.4176707
ENSBTAG00000015193	0.0473097	0.8819162	0.0545727

ENSBTAG00000015195	1.0347677	0.0047837	2.3202386
ENSBTAG00000015196	-0.019649	0.9578692	0.0186938
ENSBTAG00000015197	NA	NA	NA
ENSBTAG00000015198	NA	NA	NA
ENSBTAG00000015200	-0.301402	0.2369867	0.625276
ENSBTAG00000015202	-0.307417	0.3066542	0.5133511
ENSBTAG00000015204	-0.312719	0.2553066	0.5929379
ENSBTAG00000015205	-0.461413	0.1570921	0.8038455
ENSBTAG00000015208	NA	NA	NA
ENSBTAG00000015209	-0.171267	0.5896476	0.2294074
ENSBTAG00000015210	NA	NA	NA
ENSBTAG00000015212	0.3033187	0.3795439	0.420738
ENSBTAG00000015214	-0.12324	0.7021173	0.1535903
ENSBTAG00000015220	-0.15659	0.6364798	0.1962153
ENSBTAG00000015221	-0.086268	0.7581293	0.1202567
ENSBTAG00000015222	0.2932016	0.2942919	0.5312217
ENSBTAG00000015224	0.2720481	0.3834557	0.4162848
ENSBTAG00000015225	-0.106812	0.6775463	0.169061
ENSBTAG00000015226	NA	NA	NA
ENSBTAG00000015228	0.0076932	0.9758067	0.0106362
ENSBTAG00000015229	NA	NA	NA
ENSBTAG00000015230	-0.141488	0.5838649	0.2336876
ENSBTAG00000015232	NA	NA	NA
ENSBTAG00000015235	-0.080174	0.8419016	0.0747386
ENSBTAG00000015238	NA	NA	NA
ENSBTAG00000015240	0.1341586	0.5881971	0.2304771
ENSBTAG00000015241	-0.345435	0.3902134	0.4086978
ENSBTAG00000015242	0.0171308	0.9682762	0.0140008
ENSBTAG00000015248	0.1982884	0.4729881	0.3251497
ENSBTAG00000015249	0.3810102	0.2299197	0.6384239
ENSBTAG00000015251	0.1158049	0.7458316	0.1273592
ENSBTAG00000015253	0.3142477	0.256964	0.5901277
ENSBTAG00000015254	0.1106763	0.7873652	0.1038238
ENSBTAG00000015255	-0.32827	0.2843387	0.546164
ENSBTAG00000015258	0.8245086	0.0029784	2.5260211
ENSBTAG00000015259	NA	NA	NA
ENSBTAG00000015261	NA	NA	NA
ENSBTAG00000015263	0.3032792	0.3353534	0.4744973
ENSBTAG00000015264	0.1211313	0.7422425	0.1294542
ENSBTAG00000015265	-0.046964	0.8769449	0.0570277
ENSBTAG00000015266	-0.064353	0.7997641	0.0970381
ENSBTAG00000015267	0.3565091	0.395965	0.4023432
ENSBTAG00000015268	0.2491706	0.5325959	0.2736022
ENSBTAG00000015271	-0.209059	0.5834846	0.2339706
ENSBTAG00000015272	0.2293794	0.4213243	0.3753835
ENSBTAG00000015273	-0.024234	0.9369295	0.0282931
ENSBTAG00000015275	NA	NA	NA
ENSBTAG00000015276	0.1575882	0.692703	0.1594529
ENSBTAG00000015277	-0.115249	0.6867743	0.163186
ENSBTAG00000015278	-0.20416	0.4237208	0.3729202
ENSBTAG00000015280	NA	NA	NA
ENSBTAG00000015283	0.0396598	0.8775567	0.0567248
ENSBTAG00000015285	0.1039595	0.6848751	0.1643886
ENSBTAG00000015286	0.2135026	0.4182736	0.3785395
ENSBTAG00000015288	NA	NA	NA
ENSBTAG00000015290	NA	NA	NA
ENSBTAG00000015291	-0.112685	0.6555035	0.183425
ENSBTAG00000015293	NA	NA	NA

ENSBTAG00000015294	-0.028488	0.9177921	0.0372557
ENSBTAG00000015296	0.1410854	0.5885751	0.2301981
ENSBTAG00000015297	-0.590248	0.2604133	0.5843368
ENSBTAG00000015299	NA	NA	NA
ENSBTAG00000015301	0.0751619	0.7836562	0.1058744
ENSBTAG00000015303	-0.604766	0.0142517	1.8461328
ENSBTAG00000015304	1.2884253	0.0053172	2.2743168
ENSBTAG00000015307	NA	NA	NA
ENSBTAG00000015308	0.0579445	0.8210166	0.0856481
ENSBTAG00000015309	NA	NA	NA
ENSBTAG00000015310	-0.383996	0.1382935	0.8591983
ENSBTAG00000015311	-0.737596	0.1125856	0.9485172
ENSBTAG00000015312	0.2330017	0.515237	0.287993
ENSBTAG00000015313	NA	NA	NA
ENSBTAG00000015314	0.2445533	0.4766759	0.3217768
ENSBTAG00000015315	NA	NA	NA
ENSBTAG00000015316	-0.035376	0.8893123	0.0509457
ENSBTAG00000015317	NA	NA	NA
ENSBTAG00000015318	0.7431922	0.371133	0.4304705
ENSBTAG00000015319	0.2052888	0.5778232	0.238205
ENSBTAG00000015321	0.3094552	0.4885984	0.311048
ENSBTAG00000015325	0.3701523	0.1387507	0.8577649
ENSBTAG00000015326	NA	NA	NA
ENSBTAG00000015327	0.0653374	0.79096	0.1018455
ENSBTAG00000015329	0.2931038	0.3311554	0.4799681
ENSBTAG00000015330	0.7260021	0.1892728	0.7229118
ENSBTAG00000015333	-0.183567	0.4541162	0.342833
ENSBTAG00000015334	-0.000481	0.9995131	0.0002115
ENSBTAG00000015335	NA	NA	NA
ENSBTAG00000015336	0.0121449	0.9695311	0.0134382
ENSBTAG00000015337	0.0803416	0.8002227	0.0967891
ENSBTAG00000015338	-0.183673	0.7196613	0.1428719
ENSBTAG00000015339	0.2866843	0.480128	0.3186429
ENSBTAG00000015340	NA	NA	NA
ENSBTAG00000015341	-0.126838	0.6978772	0.156221
ENSBTAG00000015343	-0.008813	0.9864652	0.0059182
ENSBTAG00000015345	0.6972586	0.1930343	0.7143655
ENSBTAG00000015346	0.0621458	0.8266826	0.0826612
ENSBTAG00000015347	NA	NA	NA
ENSBTAG00000015348	-0.096574	0.7490048	0.1255154
ENSBTAG00000015349	NA	NA	NA
ENSBTAG00000015350	-0.063792	0.9020224	0.0447827
ENSBTAG00000015351	NA	NA	NA
ENSBTAG00000015352	0.1009913	0.7287774	0.1374051
ENSBTAG00000015353	NA	NA	NA
ENSBTAG00000015354	0.3058007	0.4385217	0.3580089
ENSBTAG00000015355	NA	NA	NA
ENSBTAG00000015356	NA	NA	NA
ENSBTAG00000015358	0.7615085	0.1944994	0.7110818
ENSBTAG00000015361	NA	NA	NA
ENSBTAG00000015362	NA	NA	NA
ENSBTAG00000015363	0.3291501	0.2367206	0.625764
ENSBTAG00000015366	-0.02771	0.9548051	0.0200853
ENSBTAG00000015368	0.0913925	0.7639061	0.11696
ENSBTAG00000015369	2.3081657	0.0003219	3.4922507
ENSBTAG00000015371	0.3424706	0.3615835	0.4417914
ENSBTAG00000015373	NA	NA	NA
ENSBTAG00000015374	NA	NA	NA

ENSBTAG00000015375	-0.363596	0.3222066	0.4918656
ENSBTAG00000015376	0.0095656	0.9734547	0.0116843
ENSBTAG00000015377	NA	NA	NA
ENSBTAG00000015378	NA	NA	NA
ENSBTAG00000015379	-0.00601	0.9813305	0.0081847
ENSBTAG00000015380	NA	NA	NA
ENSBTAG00000015381	-0.530669	0.041074	1.3864333
ENSBTAG00000015384	0.427424	0.3346612	0.4753946
ENSBTAG00000015385	0.2198791	0.4682725	0.3295013
ENSBTAG00000015386	-0.199191	0.5714107	0.2430516
ENSBTAG00000015387	NA	NA	NA
ENSBTAG00000015388	0.1195403	0.6439602	0.191141
ENSBTAG00000015390	0.0839439	0.8128592	0.0899847
ENSBTAG00000015392	0.2946792	0.4486892	0.3480544
ENSBTAG00000015394	-0.509506	0.2598492	0.5852786
ENSBTAG00000015396	-0.28825	0.5476614	0.2614879
ENSBTAG00000015398	0.5492684	0.0296119	1.5285344
ENSBTAG00000015401	0.1143382	0.70701	0.1505744
ENSBTAG00000015402	1.4338573	0.0026914	2.5700206
ENSBTAG00000015403	0.3510506	0.2996557	0.5233774
ENSBTAG00000015405	-0.083699	0.7903822	0.1021629
ENSBTAG00000015406	0.5584677	0.1387398	0.857799
ENSBTAG00000015408	0.0444357	0.9214553	0.0355257
ENSBTAG00000015412	NA	NA	NA
ENSBTAG00000015413	-0.203079	0.5211757	0.2830159
ENSBTAG00000015414	-0.030764	0.915629	0.0382804
ENSBTAG00000015415	NA	NA	NA
ENSBTAG00000015416	-0.226075	0.4443068	0.3523171
ENSBTAG00000015419	-0.416477	0.2041908	0.6899638
ENSBTAG00000015424	0.0706567	0.8199113	0.0862331
ENSBTAG00000015426	-0.105475	0.79585	0.0991688
ENSBTAG00000015427	-0.20723	0.4476436	0.3490676
ENSBTAG00000015428	0.1180744	0.7075229	0.1502595
ENSBTAG00000015432	NA	NA	NA
ENSBTAG00000015433	NA	NA	NA
ENSBTAG00000015434	0.1517058	0.5845134	0.2332055
ENSBTAG00000015436	NA	NA	NA
ENSBTAG00000015437	-0.105811	0.7185637	0.1435347
ENSBTAG00000015438	0.1045266	0.7423382	0.1293982
ENSBTAG00000015439	NA	NA	NA
ENSBTAG00000015441	1.0852163	0.0196827	1.7059149
ENSBTAG00000015444	NA	NA	NA
ENSBTAG00000015449	NA	NA	NA
ENSBTAG00000015450	0.4823889	0.0556853	1.2542596
ENSBTAG00000015453	NA	NA	NA
ENSBTAG00000015457	0.1208438	0.6955801	0.1576528
ENSBTAG00000015459	NA	NA	NA
ENSBTAG00000015460	-0.69807	0.0305451	1.5150585
ENSBTAG00000015463	0.439507	0.2404687	0.6189415
ENSBTAG00000015466	0.0061926	0.9819684	0.0079025
ENSBTAG00000015467	-0.075309	0.8429859	0.0741797
ENSBTAG00000015473	0.0128093	0.9617687	0.0169294
ENSBTAG00000015474	-0.250343	0.3571854	0.4471063
ENSBTAG00000015475	NA	NA	NA
ENSBTAG00000015478	-0.828854	0.050301	1.2984235
ENSBTAG00000015479	-0.208752	0.5654363	0.2476163
ENSBTAG00000015481	-0.186701	0.5887608	0.2300611
ENSBTAG00000015482	0.1048548	0.7033989	0.1527983

ENSBTAG00000015483	NA	NA	NA
ENSBTAG00000015485	NA	NA	NA
ENSBTAG00000015486	-0.030917	0.9022956	0.0446511
ENSBTAG00000015487	0.0260239	0.921498	0.0355056
ENSBTAG00000015490	0.349067	0.4042803	0.3933174
ENSBTAG00000015497	0.3095383	0.3898478	0.409105
ENSBTAG00000015498	NA	NA	NA
ENSBTAG00000015499	-0.218178	0.4700834	0.3278251
ENSBTAG00000015504	-0.190384	0.5248505	0.2799644
ENSBTAG00000015505	-0.521305	0.1383457	0.8590344
ENSBTAG00000015509	1.1383281	0.000528	3.2773965
ENSBTAG00000015510	NA	NA	NA
ENSBTAG00000015511	-0.683618	0.0365438	1.437186
ENSBTAG00000015512	0.0530347	0.8935794	0.0488668
ENSBTAG00000015513	-0.19587	0.6444037	0.190842
ENSBTAG00000015516	-0.365043	0.3051497	0.5154871
ENSBTAG00000015519	-0.104195	0.6735265	0.1716453
ENSBTAG00000015520	NA	NA	NA
ENSBTAG00000015521	-0.031945	0.9248977	0.0339063
ENSBTAG00000015522	0.3987826	0.2296988	0.6388413
ENSBTAG00000015525	NA	NA	NA
ENSBTAG00000015527	0.2416812	0.4592195	0.3379797
ENSBTAG00000015529	0.3785369	0.2804674	0.5521176
ENSBTAG00000015532	NA	NA	NA
ENSBTAG00000015533	NA	NA	NA
ENSBTAG00000015534	-0.424948	0.2016799	0.6953373
ENSBTAG00000015535	NA	NA	NA
ENSBTAG00000015536	0.3975203	0.1287486	0.8902573
ENSBTAG00000015537	0.1072549	0.7137301	0.146466
ENSBTAG00000015538	0.1723319	0.5278857	0.2774601
ENSBTAG00000015539	0.4042662	0.3946654	0.403771
ENSBTAG00000015541	0.1662012	0.5433648	0.2649085
ENSBTAG00000015543	0.0893164	0.7232417	0.1407165
ENSBTAG00000015546	-0.306108	0.3378453	0.4712821
ENSBTAG00000015547	NA	NA	NA
ENSBTAG00000015549	0.2345324	0.4923862	0.3076941
ENSBTAG00000015553	-0.456817	0.1435436	0.843016
ENSBTAG00000015554	-0.07606	0.7685657	0.114319
ENSBTAG00000015556	-0.216877	0.3836235	0.4160947
ENSBTAG00000015557	NA	NA	NA
ENSBTAG00000015558	NA	NA	NA
ENSBTAG00000015559	-0.009142	0.9722358	0.0122284
ENSBTAG00000015562	-0.235276	0.3724683	0.4289107
ENSBTAG00000015563	0.111972	0.6962679	0.1572236
ENSBTAG00000015565	NA	NA	NA
ENSBTAG00000015566	0.1982854	0.4392729	0.3572656
ENSBTAG00000015567	0.0471508	0.8577061	0.0666615
ENSBTAG00000015569	NA	NA	NA
ENSBTAG00000015571	0.2129846	0.5201351	0.2838838
ENSBTAG00000015572	-0.215152	0.3887097	0.4103746
ENSBTAG00000015574	NA	NA	NA
ENSBTAG00000015575	NA	NA	NA
ENSBTAG00000015576	NA	NA	NA
ENSBTAG00000015578	0.1951599	0.6521133	0.185677
ENSBTAG00000015579	-0.0649	0.8262203	0.0829042
ENSBTAG00000015580	-0.421919	0.206304	0.6854923
ENSBTAG00000015581	NA	NA	NA
ENSBTAG00000015582	1.0833923	0.000245	3.6109208

ENSBTAG00000015584	-0.434233	0.0879338	1.0558442
ENSBTAG00000015587	0.0355166	0.9329163	0.0301573
ENSBTAG00000015588	0.1977935	0.6355065	0.19688
ENSBTAG00000015590	0.1336424	0.7090229	0.1493397
ENSBTAG00000015591	0.076116	0.765797	0.1158864
ENSBTAG00000015593	-0.338918	0.4216571	0.3750406
ENSBTAG00000015595	-0.062075	0.862594	0.0641936
ENSBTAG00000015596	-0.020864	0.9504013	0.022093
ENSBTAG00000015598	0.0074021	0.9773536	0.0099483
ENSBTAG00000015599	-0.472923	0.2159518	0.6656431
ENSBTAG00000015602	0.2721688	0.575662	0.2398325
ENSBTAG00000015604	NA	NA	NA
ENSBTAG00000015606	NA	NA	NA
ENSBTAG00000015607	0.455552	0.1729445	0.7620933
ENSBTAG00000015609	0.3138703	0.4346143	0.3618959
ENSBTAG00000015611	0.1044147	0.6862055	0.1635458
ENSBTAG00000015612	0.0650007	0.7956211	0.0992937
ENSBTAG00000015614	0.3379642	0.3107325	0.5076133
ENSBTAG00000015615	NA	NA	NA
ENSBTAG00000015618	NA	NA	NA
ENSBTAG00000015625	0.093113	0.7078534	0.1500567
ENSBTAG00000015628	0.276181	0.437748	0.3587759
ENSBTAG00000015630	-0.22649	0.3766718	0.4240369
ENSBTAG00000015632	NA	NA	NA
ENSBTAG00000015635	NA	NA	NA
ENSBTAG00000015636	0.6940832	0.1542154	0.8118723
ENSBTAG00000015637	0.0014129	0.9960758	0.0017076
ENSBTAG00000015638	NA	NA	NA
ENSBTAG00000015639	0.4227432	0.204641	0.6890074
ENSBTAG00000015642	0.2011359	0.4633903	0.3340531
ENSBTAG00000015644	1.4909433	0.0019578	2.7082359
ENSBTAG00000015646	-0.483558	0.1285119	0.8910566
ENSBTAG00000015648	-0.110654	0.6598178	0.180576
ENSBTAG00000015649	-0.324996	0.4250705	0.371539
ENSBTAG00000015650	NA	NA	NA
ENSBTAG00000015651	NA	NA	NA
ENSBTAG00000015654	NA	NA	NA
ENSBTAG00000015655	-0.032446	0.9351358	0.0291253
ENSBTAG00000015656	-0.101837	0.8101053	0.0914585
ENSBTAG00000015659	0.1369787	0.5984324	0.2229849
ENSBTAG00000015662	0.075785	0.8380086	0.0767515
ENSBTAG00000015663	-0.254433	0.3053826	0.5151558
ENSBTAG00000015666	NA	NA	NA
ENSBTAG00000015667	0.4982732	0.2109959	0.6757259
ENSBTAG00000015669	0.3935906	0.2303969	0.6375233
ENSBTAG00000015670	NA	NA	NA
ENSBTAG00000015676	NA	NA	NA
ENSBTAG00000015677	-0.004497	0.985944	0.0061478
ENSBTAG00000015678	0.6625613	0.1061079	0.9742523
ENSBTAG00000015681	NA	NA	NA
ENSBTAG00000015683	-0.221164	0.3912614	0.407533
ENSBTAG00000015685	NA	NA	NA
ENSBTAG00000015686	0.0982881	0.7044185	0.1521692
ENSBTAG00000015690	-0.035189	0.8896802	0.0507661
ENSBTAG00000015692	-0.62207	0.2313998	0.6356371
ENSBTAG00000015694	0.8152702	0.0550644	1.2591289
ENSBTAG00000015698	0.5778641	0.0395089	1.403305
ENSBTAG00000015700	NA	NA	NA

ENSBTAG00000015702	0.4494982	0.5190608	0.2847818
ENSBTAG00000015704	0.9628479	0.0027555	2.5598056
ENSBTAG00000015707	NA	NA	NA
ENSBTAG00000015708	NA	NA	NA
ENSBTAG00000015710	NA	NA	NA
ENSBTAG00000015711	-1.193087	0.0105041	1.9786412
ENSBTAG00000015712	NA	NA	NA
ENSBTAG00000015713	0.0619631	0.8072511	0.0929913
ENSBTAG00000015715	NA	NA	NA
ENSBTAG00000015716	0.2441368	0.4387685	0.3577645
ENSBTAG00000015717	-0.754219	0.0565591	1.2474975
ENSBTAG00000015718	0.4600878	0.2014881	0.6957506
ENSBTAG00000015719	-0.245329	0.5061419	0.2957277
ENSBTAG00000015720	-0.532256	0.0509085	1.2932097
ENSBTAG00000015721	0.5175592	0.2142633	0.6690523
ENSBTAG00000015723	-0.139276	0.5883868	0.2303371
ENSBTAG00000015724	NA	NA	NA
ENSBTAG00000015727	1.9754583	1.50E-06	5.8231219
ENSBTAG00000015728	-0.219153	0.4526491	0.3442384
ENSBTAG00000015730	-0.025975	0.9439728	0.0250405
ENSBTAG00000015731	-0.046502	0.8547566	0.0681576
ENSBTAG00000015732	-0.22678	0.3666234	0.4357798
ENSBTAG00000015734	-0.034714	0.898567	0.0464495
ENSBTAG00000015735	NA	NA	NA
ENSBTAG00000015738	0.3820053	0.2112192	0.6752666
ENSBTAG00000015739	0.3505972	0.1899462	0.7213693
ENSBTAG00000015742	0.0161323	0.9716842	0.0124749
ENSBTAG00000015743	-0.011488	0.9642254	0.0158214
ENSBTAG00000015744	-0.052562	0.8424079	0.0744776
ENSBTAG00000015745	0.2545198	0.4920055	0.30803
ENSBTAG00000015749	NA	NA	NA
ENSBTAG00000015750	NA	NA	NA
ENSBTAG00000015751	-0.072736	0.8916536	0.0498038
ENSBTAG00000015752	0.4723888	0.3402316	0.4682253
ENSBTAG00000015753	-0.702255	0.0807429	1.0928958
ENSBTAG00000015757	NA	NA	NA
ENSBTAG00000015758	0.4557861	0.2137741	0.6700449
ENSBTAG00000015761	0.2546449	0.3587038	0.4452641
ENSBTAG00000015764	-0.202824	0.583445	0.2340001
ENSBTAG00000015766	NA	NA	NA
ENSBTAG00000015767	-0.019242	0.9388134	0.0274207
ENSBTAG00000015769	NA	NA	NA
ENSBTAG00000015771	0.3865337	0.141754	0.8484646
ENSBTAG00000015772	-0.017796	0.9783494	0.009506
ENSBTAG00000015773	NA	NA	NA
ENSBTAG00000015775	NA	NA	NA
ENSBTAG00000015776	-0.209584	0.3983324	0.3997543
ENSBTAG00000015778	0.6212528	0.0583772	1.233757
ENSBTAG00000015779	0.7845173	0.0974912	1.0110348
ENSBTAG00000015780	-0.341921	0.2179713	0.6616008
ENSBTAG00000015781	0.125218	0.7027712	0.153186
ENSBTAG00000015782	0.2118098	0.50849	0.2937176
ENSBTAG00000015784	NA	NA	NA
ENSBTAG00000015786	-0.427726	0.1130019	0.9469143
ENSBTAG00000015788	-0.073116	0.8667121	0.0621252
ENSBTAG00000015792	NA	NA	NA
ENSBTAG00000015793	NA	NA	NA
ENSBTAG00000015794	-0.508611	0.1216633	0.9148404

ENSBTAG00000015796	0.1319611	0.6181261	0.2089229
ENSBTAG00000015798	0.0590406	0.8192608	0.0865778
ENSBTAG00000015800	NA	NA	NA
ENSBTAG00000015801	0.1764804	0.6322705	0.1990971
ENSBTAG00000015802	0.1042447	0.7139492	0.1463327
ENSBTAG00000015804	NA	NA	NA
ENSBTAG00000015805	0.1640931	0.6709581	0.1733046
ENSBTAG00000015806	-0.311222	0.2099611	0.6778611
ENSBTAG00000015807	0.2467165	0.5972535	0.2238413
ENSBTAG00000015808	0.3275914	0.3263918	0.4862608
ENSBTAG00000015815	0.800092	0.1431917	0.8440822
ENSBTAG00000015817	-0.358904	0.2823626	0.5491928
ENSBTAG00000015820	0.0228429	0.9473765	0.0234774
ENSBTAG00000015821	1.6062145	2.30E-06	5.6376156
ENSBTAG00000015824	-0.170341	0.503655	0.2978668
ENSBTAG00000015825	NA	NA	NA
ENSBTAG00000015827	NA	NA	NA
ENSBTAG00000015828	0.0724574	0.8936196	0.0488473
ENSBTAG00000015829	0.1939738	0.679109	0.1680605
ENSBTAG00000015830	NA	NA	NA
ENSBTAG00000015831	-0.025135	0.9227264	0.034927
ENSBTAG00000015833	0.3538307	0.1873009	0.7274602
ENSBTAG00000015834	NA	NA	NA
ENSBTAG00000015835	NA	NA	NA
ENSBTAG00000015836	NA	NA	NA
ENSBTAG00000015837	NA	NA	NA
ENSBTAG00000015839	-0.154888	0.5651374	0.2478459
ENSBTAG00000015840	NA	NA	NA
ENSBTAG00000015841	0.5054868	0.1598239	0.7963583
ENSBTAG00000015844	0.7733895	0.0040524	2.3922824
ENSBTAG00000015848	-0.242704	0.3920613	0.406646
ENSBTAG00000015849	0.3407414	0.4703024	0.3276228
ENSBTAG00000015853	-0.17913	0.4824294	0.3165663
ENSBTAG00000015856	NA	NA	NA
ENSBTAG00000015857	-0.034629	0.8923793	0.0494505
ENSBTAG00000015858	-0.062513	0.7962624	0.0989438
ENSBTAG00000015859	-0.232595	0.5561421	0.2548143
ENSBTAG00000015860	NA	NA	NA
ENSBTAG00000015866	0.0188245	0.9614435	0.0170762
ENSBTAG00000015868	-0.720285	0.0684786	1.1644454
ENSBTAG00000015872	NA	NA	NA
ENSBTAG00000015874	0.2028049	0.619831	0.2077267
ENSBTAG00000015875	NA	NA	NA
ENSBTAG00000015878	NA	NA	NA
ENSBTAG00000015879	0.0859792	0.8579031	0.0665618
ENSBTAG00000015880	NA	NA	NA
ENSBTAG00000015882	0.1388128	0.662652	0.1787145
ENSBTAG00000015885	0.6821939	0.1330587	0.8759568
ENSBTAG00000015887	-0.177985	0.4748945	0.3234028
ENSBTAG00000015888	-0.153054	0.6877659	0.1625594
ENSBTAG00000015889	0.2994623	0.2682	0.5715412
ENSBTAG00000015891	0.2441554	0.3473693	0.4592086
ENSBTAG00000015892	0.0631142	0.8168904	0.0878362
ENSBTAG00000015893	NA	NA	NA
ENSBTAG00000015894	0.0048846	0.9947767	0.0022744
ENSBTAG00000015895	NA	NA	NA
ENSBTAG00000015896	-0.079417	0.767923	0.1146823
ENSBTAG00000015898	-0.160422	0.5455202	0.2631892

ENSBTAG00000015899	0.6552137	0.1187078	0.9255208
ENSBTAG00000015900	-0.235617	0.5791457	0.2372122
ENSBTAG00000015901	0.0891112	0.7423	0.1294206
ENSBTAG00000015902	NA	NA	NA
ENSBTAG00000015904	-1.42636	8.57E-05	4.0671981
ENSBTAG00000015905	-0.124314	0.7704739	0.1132421
ENSBTAG00000015908	0.090305	0.8386536	0.0764174
ENSBTAG00000015909	-0.491819	0.0641756	1.1926301
ENSBTAG00000015910	0.0703105	0.7774424	0.1093318
ENSBTAG00000015911	0.1161287	0.7882806	0.1033192
ENSBTAG00000015913	NA	NA	NA
ENSBTAG00000015915	NA	NA	NA
ENSBTAG00000015917	NA	NA	NA
ENSBTAG00000015919	NA	NA	NA
ENSBTAG00000015920	-0.016859	0.9470107	0.0236451
ENSBTAG00000015925	0.3242704	0.3956434	0.402696
ENSBTAG00000015926	0.7010753	0.0307375	1.5123319
ENSBTAG00000015930	0.0234576	0.940207	0.0267765
ENSBTAG00000015931	0.0249911	0.9323517	0.0304202
ENSBTAG00000015935	-2.271488	0.0001088	3.9631835
ENSBTAG00000015936	NA	NA	NA
ENSBTAG00000015938	0.4228473	0.2239986	0.6497547
ENSBTAG00000015939	-0.428591	0.3312813	0.4798031
ENSBTAG00000015940	NA	NA	NA
ENSBTAG00000015942	-0.120695	0.6357458	0.1967165
ENSBTAG00000015943	-0.100823	0.69487	0.1580964
ENSBTAG00000015944	-0.032082	0.910209	0.0408589
ENSBTAG00000015945	NA	NA	NA
ENSBTAG00000015946	0.0893367	0.7311539	0.1359912
ENSBTAG00000015949	0.23118	0.6508123	0.1865442
ENSBTAG00000015950	0.6803344	0.0142312	1.8467589
ENSBTAG00000015951	-0.22881	0.3797948	0.420451
ENSBTAG00000015952	0.2619539	0.5838039	0.233733
ENSBTAG00000015953	NA	NA	NA
ENSBTAG00000015955	-0.39153	0.1759939	0.7545025
ENSBTAG00000015958	0.3005435	0.3279042	0.484253
ENSBTAG00000015961	-0.2121	0.6489863	0.1877645
ENSBTAG00000015962	-0.184315	0.4829672	0.3160823
ENSBTAG00000015963	0.3934866	0.3228152	0.491046
ENSBTAG00000015965	-0.511587	0.1124617	0.9489955
ENSBTAG00000015968	0.2688526	0.5429934	0.2652054
ENSBTAG00000015969	-0.146749	0.6033079	0.219461
ENSBTAG00000015972	-0.199375	0.5072062	0.2948155
ENSBTAG00000015973	-0.037518	0.9230233	0.0347874
ENSBTAG00000015974	0.5056278	0.1177459	0.9290543
ENSBTAG00000015976	0.5591963	0.2425321	0.6152308
ENSBTAG00000015977	-0.319489	0.3034979	0.5178443
ENSBTAG00000015978	0.0536183	0.8465742	0.072335
ENSBTAG00000015979	NA	NA	NA
ENSBTAG00000015980	-0.662858	0.2625392	0.5808059
ENSBTAG00000015981	0.2160765	0.6730116	0.1719774
ENSBTAG00000015982	NA	NA	NA
ENSBTAG00000015984	-0.619637	0.1321974	0.8787771
ENSBTAG00000015985	NA	NA	NA
ENSBTAG00000015986	-0.309829	0.4303775	0.3661505
ENSBTAG00000015987	NA	NA	NA
ENSBTAG00000015988	0.2990817	0.3486309	0.4576341
ENSBTAG00000015989	0.0911139	0.7231092	0.1407961

ENSBTAG00000015991	NA	NA	NA
ENSBTAG00000015994	NA	NA	NA
ENSBTAG00000015996	0.058044	0.8397535	0.0758482
ENSBTAG00000016000	0.2588583	0.3979213	0.4002029
ENSBTAG00000016002	-0.337164	0.5376018	0.2695393
ENSBTAG00000016004	-0.647727	0.0236539	1.6260972
ENSBTAG00000016005	-0.884757	0.0004672	3.3305093
ENSBTAG00000016006	0.2728729	0.3085129	0.5107266
ENSBTAG00000016007	-0.139167	0.6043018	0.2187461
ENSBTAG00000016010	0.0556772	0.872606	0.0591818
ENSBTAG00000016012	NA	NA	NA
ENSBTAG00000016013	-0.238764	0.380078	0.4201272
ENSBTAG00000016014	0.1106699	0.718948	0.1433025
ENSBTAG00000016017	NA	NA	NA
ENSBTAG00000016021	0.4596105	0.173979	0.7595033
ENSBTAG00000016023	0.2196584	0.3958228	0.4024992
ENSBTAG00000016024	-0.615081	0.5554565	0.25535
ENSBTAG00000016026	0.5726515	0.049536	1.305079
ENSBTAG00000016028	0.1536938	0.6382588	0.1950032
ENSBTAG00000016030	NA	NA	NA
ENSBTAG00000016032	0.5686329	0.2324197	0.6337271
ENSBTAG00000016033	0.0359453	0.9147862	0.0386804
ENSBTAG00000016034	-0.12128	0.7453078	0.1276643
ENSBTAG00000016037	-0.183597	0.4866639	0.3127709
ENSBTAG00000016038	-0.040481	0.8950248	0.0481649
ENSBTAG00000016041	NA	NA	NA
ENSBTAG00000016042	NA	NA	NA
ENSBTAG00000016043	NA	NA	NA
ENSBTAG00000016045	0.4111573	0.2186921	0.6601669
ENSBTAG00000016046	-0.268992	0.2876008	0.5412098
ENSBTAG00000016048	1.0396783	0.0002975	3.526532
ENSBTAG00000016050	0.0422531	0.8985828	0.0464419
ENSBTAG00000016053	0.2374375	0.5913434	0.2281603
ENSBTAG00000016057	0.4371309	0.1087309	0.9636469
ENSBTAG00000016058	NA	NA	NA
ENSBTAG00000016060	-1.209126	0.0054865	2.2607066
ENSBTAG00000016061	1.3331776	0.0105959	1.9748626
ENSBTAG00000016062	0.2984249	0.3733657	0.4278656
ENSBTAG00000016063	NA	NA	NA
ENSBTAG00000016069	0.1414638	0.569591	0.2444368
ENSBTAG00000016071	NA	NA	NA
ENSBTAG00000016072	NA	NA	NA
ENSBTAG00000016073	-0.18561	0.6245892	0.2044055
ENSBTAG00000016074	0.0582183	0.8332819	0.079208
ENSBTAG00000016075	-0.075459	0.7822702	0.1066432
ENSBTAG00000016076	-0.142324	0.5871392	0.2312589
ENSBTAG00000016077	0.267026	0.4238436	0.3727944
ENSBTAG00000016078	0.2383672	0.3971821	0.4010103
ENSBTAG00000016079	0.0239493	0.9314762	0.0308282
ENSBTAG00000016080	0.2021093	0.44837	0.3483635
ENSBTAG00000016081	NA	NA	NA
ENSBTAG00000016085	0.6038591	0.0617233	1.2095512
ENSBTAG00000016091	0.5516032	0.0895569	1.0479007
ENSBTAG00000016092	0.1159338	0.6584321	0.181489
ENSBTAG00000016093	-0.383541	0.1872739	0.7275228
ENSBTAG00000016094	0.1377992	0.7737014	0.1114266
ENSBTAG00000016095	0.8422428	0.0773139	1.1117424
ENSBTAG00000016096	NA	NA	NA

ENSBTAG00000016097	NA	NA	NA
ENSBTAG00000016098	-0.074396	0.7782916	0.1088577
ENSBTAG00000016101	0.3091638	0.4090106	0.3882654
ENSBTAG00000016103	0.0332096	0.907604	0.0421036
ENSBTAG00000016104	-0.532696	0.040137	1.3964549
ENSBTAG00000016105	-0.164565	0.5125821	0.2902365
ENSBTAG00000016107	NA	NA	NA
ENSBTAG00000016108	0.0613587	0.8380459	0.0767322
ENSBTAG00000016109	-0.047875	0.8992836	0.0461033
ENSBTAG00000016112	1.8486272	0.0051202	2.2907173
ENSBTAG00000016121	NA	NA	NA
ENSBTAG00000016122	0.2738764	0.4079273	0.3894172
ENSBTAG00000016124	NA	NA	NA
ENSBTAG00000016125	-0.548551	0.0313633	1.5035785
ENSBTAG00000016126	0.1802369	0.5531601	0.2571491
ENSBTAG00000016128	0.1490083	0.6628055	0.1786139
ENSBTAG00000016131	-0.098785	0.8340257	0.0788206
ENSBTAG00000016133	-0.1168	0.6563067	0.1828931
ENSBTAG00000016137	0.9976798	0.0106112	1.9742338
ENSBTAG00000016139	1.0602409	0.0019272	2.7150656
ENSBTAG00000016140	-0.176213	0.6219437	0.2062489
ENSBTAG00000016142	NA	NA	NA
ENSBTAG00000016144	NA	NA	NA
ENSBTAG00000016145	NA	NA	NA
ENSBTAG00000016147	NA	NA	NA
ENSBTAG00000016148	NA	NA	NA
ENSBTAG00000016149	NA	NA	NA
ENSBTAG00000016151	NA	NA	NA
ENSBTAG00000016152	0.1281352	0.700524	0.154577
ENSBTAG00000016155	NA	NA	NA
ENSBTAG00000016156	-0.034401	0.9095234	0.0411861
ENSBTAG00000016158	NA	NA	NA
ENSBTAG00000016159	NA	NA	NA
ENSBTAG00000016161	0.3295814	0.2143674	0.6688413
ENSBTAG00000016163	NA	NA	NA
ENSBTAG00000016164	-0.144076	0.5796873	0.2368062
ENSBTAG00000016165	NA	NA	NA
ENSBTAG00000016166	NA	NA	NA
ENSBTAG00000016167	-0.524797	0.0391991	1.4067242
ENSBTAG00000016168	0.2793782	0.2698007	0.568957
ENSBTAG00000016169	-0.992975	0.0006317	3.1995139
ENSBTAG00000016170	-0.190278	0.5345438	0.2720167
ENSBTAG00000016171	0.1190693	0.801261	0.096226
ENSBTAG00000016174	-0.193462	0.5006206	0.3004912
ENSBTAG00000016175	0.5528788	0.318867	0.4963904
ENSBTAG00000016177	0.2959802	0.4309805	0.3655424
ENSBTAG00000016185	0.3919871	0.166881	0.7775932
ENSBTAG00000016190	0.3233217	0.2532214	0.5964996
ENSBTAG00000016191	-0.218664	0.4326631	0.3638501
ENSBTAG00000016192	NA	NA	NA
ENSBTAG00000016193	-0.141354	0.5763782	0.2392925
ENSBTAG00000016194	0.8092047	0.0097485	2.0110622
ENSBTAG00000016197	-0.119301	0.6494214	0.1874734
ENSBTAG00000016198	0.0161971	0.9522663	0.0212416
ENSBTAG00000016199	-0.095082	0.7001325	0.1548198
ENSBTAG00000016202	NA	NA	NA
ENSBTAG00000016203	0.0772116	0.8648231	0.0630727
ENSBTAG00000016204	NA	NA	NA

ENSBTAG00000016206	0.5189597	0.0534047	1.2724206
ENSBTAG00000016208	0.083111	0.7730573	0.1117883
ENSBTAG00000016209	NA	NA	NA
ENSBTAG00000016210	NA	NA	NA
ENSBTAG00000016211	-0.092772	0.7097147	0.1489162
ENSBTAG00000016213	NA	NA	NA
ENSBTAG00000016217	0.9260475	0.0164877	1.7828407
ENSBTAG00000016218	-0.187394	0.4708811	0.3270888
ENSBTAG00000016220	0.8091566	0.0110112	1.9581641
ENSBTAG00000016221	-0.002776	0.9924629	0.0032857
ENSBTAG00000016223	-0.4152	0.3217892	0.4924285
ENSBTAG00000016224	-0.020101	0.9381105	0.027746
ENSBTAG00000016227	NA	NA	NA
ENSBTAG00000016228	0.1014397	0.6894399	0.1615036
ENSBTAG00000016229	0.3115227	0.2441176	0.612401
ENSBTAG00000016230	0.2198556	0.4235655	0.3730794
ENSBTAG00000016231	0.3630426	0.3570773	0.4472377
ENSBTAG00000016234	NA	NA	NA
ENSBTAG00000016235	-0.347687	0.2429969	0.6143993
ENSBTAG00000016236	0.5023618	0.0889577	1.0508166
ENSBTAG00000016240	-0.293456	0.3888269	0.4102437
ENSBTAG00000016243	-0.050584	0.8406867	0.0753658
ENSBTAG00000016244	NA	NA	NA
ENSBTAG00000016246	NA	NA	NA
ENSBTAG00000016247	NA	NA	NA
ENSBTAG00000016249	NA	NA	NA
ENSBTAG00000016250	-0.17631	0.491939	0.3080887
ENSBTAG00000016251	0.041007	0.8835407	0.0537735
ENSBTAG00000016252	-0.20133	0.4177344	0.3790998
ENSBTAG00000016253	-0.192571	0.6677633	0.1753775
ENSBTAG00000016254	0.1239793	0.6610233	0.1797832
ENSBTAG00000016257	-0.166275	0.636048	0.1965101
ENSBTAG00000016259	-0.319111	0.4384991	0.3580313
ENSBTAG00000016260	-0.049859	0.8533138	0.0688912
ENSBTAG00000016263	0.0531747	0.9140535	0.0390284
ENSBTAG00000016264	-0.024735	0.9249525	0.0338806
ENSBTAG00000016265	-0.191838	0.4976492	0.3030767
ENSBTAG00000016266	-0.032888	0.8954405	0.0479632
ENSBTAG00000016267	0.4729174	0.1472703	0.8318847
ENSBTAG00000016268	0.0403774	0.9123227	0.0398515
ENSBTAG00000016269	-0.025252	0.9304397	0.0313118
ENSBTAG00000016270	NA	NA	NA
ENSBTAG00000016271	0.053696	0.8580128	0.0665062
ENSBTAG00000016275	NA	NA	NA
ENSBTAG00000016276	NA	NA	NA
ENSBTAG00000016277	0.2647319	0.3557627	0.4488396
ENSBTAG00000016278	0.0116993	0.9641078	0.0158744
ENSBTAG00000016279	-0.371572	0.3652096	0.4374579
ENSBTAG00000016281	0.1404504	0.6418108	0.1925929
ENSBTAG00000016282	0.0876656	0.7550085	0.1220481
ENSBTAG00000016283	NA	NA	NA
ENSBTAG00000016284	NA	NA	NA
ENSBTAG00000016285	NA	NA	NA
ENSBTAG00000016287	0.1929158	0.6475574	0.1887217
ENSBTAG00000016290	NA	NA	NA
ENSBTAG00000016291	NA	NA	NA
ENSBTAG00000016294	0.2985142	0.4660946	0.331526
ENSBTAG00000016295	-0.034638	0.8981111	0.0466699

ENSBTAG00000016296	-0.599005	0.0206409	1.6852716
ENSBTAG00000016297	-0.185682	0.6371606	0.1957511
ENSBTAG00000016298	-0.006537	0.9791133	0.0091671
ENSBTAG00000016299	0.3829418	0.2673879	0.5728582
ENSBTAG00000016302	NA	NA	NA
ENSBTAG00000016305	NA	NA	NA
ENSBTAG00000016307	-0.012372	0.9809414	0.0083569
ENSBTAG00000016309	-0.054909	0.8293507	0.0812618
ENSBTAG00000016311	-0.377234	0.2059515	0.6862351
ENSBTAG00000016312	0.3085822	0.2249651	0.6478849
ENSBTAG00000016313	NA	NA	NA
ENSBTAG00000016315	0.1713097	0.7154074	0.1454466
ENSBTAG00000016316	0.0451702	0.8560714	0.06749
ENSBTAG00000016319	-0.046878	0.8985288	0.046468
ENSBTAG00000016320	NA	NA	NA
ENSBTAG00000016324	NA	NA	NA
ENSBTAG00000016326	-0.091484	0.7734006	0.1115955
ENSBTAG00000016327	NA	NA	NA
ENSBTAG00000016328	-0.029061	0.9168732	0.0376907
ENSBTAG00000016331	-0.086877	0.7695986	0.1137357
ENSBTAG00000016332	0.0007027	1	0
ENSBTAG00000016334	NA	NA	NA
ENSBTAG00000016335	-0.268634	0.5387465	0.2686156
ENSBTAG00000016336	0.1351309	0.5845278	0.2331948
ENSBTAG00000016337	-0.317407	0.2142079	0.6691646
ENSBTAG00000016338	0.2411168	0.4609858	0.3363124
ENSBTAG00000016341	0.2682107	0.38893	0.4101286
ENSBTAG00000016343	NA	NA	NA
ENSBTAG00000016344	NA	NA	NA
ENSBTAG00000016345	0.1847749	0.7243951	0.1400245
ENSBTAG00000016347	-0.294993	0.5542737	0.2562758
ENSBTAG00000016348	NA	NA	NA
ENSBTAG00000016349	-0.215125	0.5357022	0.2710766
ENSBTAG00000016350	-0.041172	0.8982171	0.0466187
ENSBTAG00000016352	-0.421003	0.2079776	0.6819834
ENSBTAG00000016354	0.2869841	0.3436671	0.4638621
ENSBTAG00000016355	0.1367477	0.6374902	0.1955265
ENSBTAG00000016357	0.7510359	0.0797696	1.0981625
ENSBTAG00000016359	NA	NA	NA
ENSBTAG00000016360	NA	NA	NA
ENSBTAG00000016362	-0.125754	0.705332	0.1516064
ENSBTAG00000016363	0.1125734	0.6649671	0.1771999
ENSBTAG00000016365	NA	NA	NA
ENSBTAG00000016366	NA	NA	NA
ENSBTAG00000016367	0.0407833	0.8810002	0.055024
ENSBTAG00000016368	-0.017175	0.946462	0.0238968
ENSBTAG00000016369	-0.105557	0.7219964	0.141465
ENSBTAG00000016373	NA	NA	NA
ENSBTAG00000016374	0.1577902	0.5331534	0.2731478
ENSBTAG00000016377	-0.764436	0.0128896	1.8897598
ENSBTAG00000016378	0.3823263	0.1817052	0.7406327
ENSBTAG00000016381	NA	NA	NA
ENSBTAG00000016384	NA	NA	NA
ENSBTAG00000016385	NA	NA	NA
ENSBTAG00000016387	0.3409656	0.32695	0.4855187
ENSBTAG00000016388	NA	NA	NA
ENSBTAG00000016391	NA	NA	NA
ENSBTAG00000016396	NA	NA	NA

ENSBTAG00000016397	0.0154564	0.9643943	0.0157454
ENSBTAG00000016398	NA	NA	NA
ENSBTAG00000016399	NA	NA	NA
ENSBTAG00000016401	-0.335696	0.2216517	0.654329
ENSBTAG00000016406	-0.23603	0.4433315	0.3532715
ENSBTAG00000016407	NA	NA	NA
ENSBTAG00000016411	-0.389861	0.2290096	0.6401463
ENSBTAG00000016412	NA	NA	NA
ENSBTAG00000016413	-0.00934	0.9748793	0.0110491
ENSBTAG00000016414	NA	NA	NA
ENSBTAG00000016415	0.2539273	0.3856868	0.4137652
ENSBTAG00000016420	-0.214305	0.3876427	0.4115684
ENSBTAG00000016424	NA	NA	NA
ENSBTAG00000016427	-0.018926	0.9526196	0.0210805
ENSBTAG00000016429	-0.437786	0.2001566	0.69863
ENSBTAG00000016430	-0.165104	0.6233346	0.2052788
ENSBTAG00000016432	-0.020459	0.9744518	0.0112396
ENSBTAG00000016433	NA	NA	NA
ENSBTAG00000016435	-0.132584	0.6531077	0.1850152
ENSBTAG00000016438	-0.172901	0.7213301	0.1418659
ENSBTAG00000016441	1.2628311	6.50E-05	4.1868255
ENSBTAG00000016442	-0.128863	0.6684002	0.1749635
ENSBTAG00000016444	0.7212668	0.0124634	1.9043621
ENSBTAG00000016445	-0.087336	0.7223129	0.1412746
ENSBTAG00000016448	-0.05598	0.8394446	0.076008
ENSBTAG00000016450	-0.589719	0.0504172	1.2974211
ENSBTAG00000016451	-0.288067	0.2482886	0.6050433
ENSBTAG00000016455	-0.445679	0.1466528	0.8337096
ENSBTAG00000016456	0.035711	0.8889727	0.0511116
ENSBTAG00000016457	-0.391232	0.1364574	0.8650029
ENSBTAG00000016462	0.301814	0.4501528	0.34664
ENSBTAG00000016465	-0.359904	0.3274261	0.4848867
ENSBTAG00000016467	-0.040235	0.9309301	0.0310829
ENSBTAG00000016469	0.2441986	0.3523087	0.4530766
ENSBTAG00000016470	0.3428404	0.3150539	0.5016151
ENSBTAG00000016471	0.1274311	0.6237965	0.2049571
ENSBTAG00000016472	-0.694539	0.42106	0.375656
ENSBTAG00000016473	0.3066688	0.448663	0.3480798
ENSBTAG00000016477	NA	NA	NA
ENSBTAG00000016481	0.1090822	0.6591858	0.1809921
ENSBTAG00000016484	0.1227497	0.7130858	0.1468582
ENSBTAG00000016486	1.0394889	0.0895546	1.0479122
ENSBTAG00000016494	-0.522615	0.3183865	0.4970453
ENSBTAG00000016495	0.2116506	0.5032114	0.2982496
ENSBTAG00000016496	0.2294914	0.3929797	0.4056299
ENSBTAG00000016497	0.4382392	0.1128166	0.947627
ENSBTAG00000016498	0.3783832	0.1610112	0.7931439
ENSBTAG00000016501	1.1126983	0.0046079	2.3365011
ENSBTAG00000016502	0.2974131	0.5363266	0.2705706
ENSBTAG00000016504	0.1706611	0.5435997	0.2647208
ENSBTAG00000016505	0.1832457	0.7292177	0.1371428
ENSBTAG00000016506	-0.141818	0.6875993	0.1626646
ENSBTAG00000016507	NA	NA	NA
ENSBTAG00000016508	0.3502658	0.200624	0.697617
ENSBTAG00000016510	0.0257847	0.9402129	0.0267738
ENSBTAG00000016511	0.3507471	0.1577921	0.8019148
ENSBTAG00000016512	-0.133436	0.6137609	0.2120008
ENSBTAG00000016513	0.2869571	0.5139785	0.289055

ENSBTAG00000016514	0.0460296	0.9083134	0.0417643
ENSBTAG00000016515	NA	NA	NA
ENSBTAG00000016516	0.2710999	0.2714879	0.5662495
ENSBTAG00000016519	0.5435228	0.2046641	0.6889584
ENSBTAG00000016520	-0.44593	0.3150342	0.5016422
ENSBTAG00000016522	0.0074127	0.9820606	0.0078617
ENSBTAG00000016523	NA	NA	NA
ENSBTAG00000016524	-0.100481	0.6821795	0.1661013
ENSBTAG00000016525	0.2802124	0.2648239	0.5770428
ENSBTAG00000016526	0.1824906	0.4950352	0.305364
ENSBTAG00000016527	0.2765619	0.5543626	0.2562061
ENSBTAG00000016528	0.4566707	0.1624826	0.7891932
ENSBTAG00000016529	0.355735	0.158885	0.7989171
ENSBTAG00000016532	0.6421767	0.0239192	1.6212529
ENSBTAG00000016533	-0.23307	0.4247743	0.3718418
ENSBTAG00000016534	NA	NA	NA
ENSBTAG00000016541	NA	NA	NA
ENSBTAG00000016542	0.3754508	0.5326041	0.2735955
ENSBTAG00000016544	NA	NA	NA
ENSBTAG00000016546	-0.147496	0.6824737	0.1659141
ENSBTAG00000016547	-0.147759	0.650668	0.1866406
ENSBTAG00000016548	NA	NA	NA
ENSBTAG00000016549	-0.551813	0.170457	0.7683851
ENSBTAG00000016550	NA	NA	NA
ENSBTAG00000016551	NA	NA	NA
ENSBTAG00000016552	-0.106038	0.7366515	0.1327379
ENSBTAG00000016553	0.7917938	0.0328619	1.4833074
ENSBTAG00000016555	-0.082706	0.7956903	0.0992559
ENSBTAG00000016556	NA	NA	NA
ENSBTAG00000016557	0.0186468	0.9456684	0.0242611
ENSBTAG00000016558	0.2182666	0.4420553	0.3545234
ENSBTAG00000016559	0.0086678	0.9766191	0.0102748
ENSBTAG00000016560	0.2611088	0.4677872	0.3299517
ENSBTAG00000016561	0.1740655	0.6413665	0.1928938
ENSBTAG00000016562	-0.021573	0.9409089	0.0264524
ENSBTAG00000016563	-0.436965	0.1048157	0.9795735
ENSBTAG00000016564	0.0412739	0.9341247	0.0295951
ENSBTAG00000016566	0.2279397	0.497106	0.303551
ENSBTAG00000016567	NA	NA	NA
ENSBTAG00000016568	-0.040583	0.8732057	0.0588834
ENSBTAG00000016571	0.0286459	0.9362148	0.0286245
ENSBTAG00000016572	0.0788908	0.7456759	0.1274499
ENSBTAG00000016573	NA	NA	NA
ENSBTAG00000016574	NA	NA	NA
ENSBTAG00000016575	NA	NA	NA
ENSBTAG00000016577	-0.144715	0.6003703	0.2215808
ENSBTAG00000016578	0.0743862	0.7832213	0.1061155
ENSBTAG00000016580	-0.509471	0.1509551	0.8211521
ENSBTAG00000016581	0.1566814	0.5524655	0.2576949
ENSBTAG00000016582	0.0343796	0.917238	0.037518
ENSBTAG00000016584	-0.263568	0.3936027	0.404942
ENSBTAG00000016588	NA	NA	NA
ENSBTAG00000016589	0.2733308	0.4071434	0.3902526
ENSBTAG00000016590	0.5468677	0.1651561	0.7821053
ENSBTAG00000016591	0.3089679	0.2939746	0.5316902
ENSBTAG00000016592	0.8772295	0.0320697	1.4939052
ENSBTAG00000016593	0.0205775	0.9481911	0.0231041
ENSBTAG00000016594	0.3812641	0.2688408	0.5705048

ENSBTAG00000016595	0.2159339	0.3874406	0.4117949
ENSBTAG00000016596	-0.179589	0.4908284	0.3090703
ENSBTAG00000016598	0.3175651	0.2043161	0.6896974
ENSBTAG00000016599	-0.011016	0.9651445	0.0154076
ENSBTAG00000016600	0.5072624	0.2316986	0.6350765
ENSBTAG00000016603	NA	NA	NA
ENSBTAG00000016606	0.220864	0.5270288	0.2781656
ENSBTAG00000016607	NA	NA	NA
ENSBTAG00000016609	NA	NA	NA
ENSBTAG00000016612	0.0164507	0.9475359	0.0234043
ENSBTAG00000016615	-1.180472	0.0007675	3.1149075
ENSBTAG00000016618	-0.032806	0.9013694	0.0450972
ENSBTAG00000016619	NA	NA	NA
ENSBTAG00000016620	NA	NA	NA
ENSBTAG00000016621	0.0525518	0.8632817	0.0638475
ENSBTAG00000016622	-0.232172	0.5648563	0.248062
ENSBTAG00000016625	-0.029662	0.9197188	0.0363449
ENSBTAG00000016627	NA	NA	NA
ENSBTAG00000016629	-0.021111	0.9345124	0.0294149
ENSBTAG00000016635	-0.013334	0.9600452	0.0177083
ENSBTAG00000016637	-0.209035	0.4218947	0.374796
ENSBTAG00000016640	-0.138021	0.6549645	0.1837823
ENSBTAG00000016643	NA	NA	NA
ENSBTAG00000016644	-0.075644	0.7796533	0.1080985
ENSBTAG00000016646	NA	NA	NA
ENSBTAG00000016648	0.0492119	0.8534218	0.0688363
ENSBTAG00000016649	0.1861477	0.6995466	0.1551834
ENSBTAG00000016650	-0.026581	0.94847	0.0229764
ENSBTAG00000016651	NA	NA	NA
ENSBTAG00000016656	0.5862727	0.0784301	1.1055171
ENSBTAG00000016658	0.6653422	0.0242962	1.6144612
ENSBTAG00000016661	0.7624007	0.0896247	1.0475721
ENSBTAG00000016662	NA	NA	NA
ENSBTAG00000016663	-0.030664	0.9168186	0.0377166
ENSBTAG00000016664	0.0819179	0.8525051	0.069303
ENSBTAG00000016666	-0.371501	0.1404527	0.85247
ENSBTAG00000016667	-0.447117	0.2944284	0.5310203
ENSBTAG00000016675	-0.033322	0.9043401	0.0436682
ENSBTAG00000016676	-1.030129	0.0007569	3.1209403
ENSBTAG00000016677	NA	NA	NA
ENSBTAG00000016679	-0.014833	0.9534868	0.0206853
ENSBTAG00000016680	-0.136935	0.5850173	0.2328313
ENSBTAG00000016683	0.2810337	0.5544126	0.2561669
ENSBTAG00000016684	0.7588983	0.0709087	1.1493003
ENSBTAG00000016685	NA	NA	NA
ENSBTAG00000016691	-0.140255	0.7288134	0.1373837
ENSBTAG00000016692	NA	NA	NA
ENSBTAG00000016694	0.2506216	0.3858483	0.4135834
ENSBTAG00000016696	0.0037419	0.9928721	0.0031067
ENSBTAG00000016698	NA	NA	NA
ENSBTAG00000016703	-0.386929	0.1672067	0.7767464
ENSBTAG00000016704	NA	NA	NA
ENSBTAG00000016705	0.102619	0.7389547	0.1313822
ENSBTAG00000016707	-0.051283	0.8574152	0.0668088
ENSBTAG00000016708	-0.01183	0.9623116	0.0166843
ENSBTAG00000016709	-0.16947	0.5306418	0.2751986
ENSBTAG00000016710	0.1335838	0.7115777	0.1477777
ENSBTAG00000016711	0.7298742	0.0897461	1.0469843

ENSBTAG00000016712	NA	NA	NA
ENSBTAG00000016713	NA	NA	NA
ENSBTAG00000016714	0.3925889	0.3333891	0.4770486
ENSBTAG00000016716	0.5133805	0.1555969	0.807999
ENSBTAG00000016717	0.439322	0.1443622	0.8405466
ENSBTAG00000016720	0.0375933	0.8905538	0.0503399
ENSBTAG00000016721	NA	NA	NA
ENSBTAG00000016722	0.1614764	0.6844869	0.1646349
ENSBTAG00000016723	NA	NA	NA
ENSBTAG00000016724	-0.03995	0.8848925	0.0531095
ENSBTAG00000016725	-0.065108	0.8282823	0.0818216
ENSBTAG00000016726	NA	NA	NA
ENSBTAG00000016728	-0.11784	0.776202	0.1100252
ENSBTAG00000016729	NA	NA	NA
ENSBTAG00000016730	-0.334375	0.229516	0.6391871
ENSBTAG00000016731	-0.112619	0.8348865	0.0783726
ENSBTAG00000016733	0.1334755	0.6914538	0.1602369
ENSBTAG00000016734	NA	NA	NA
ENSBTAG00000016735	0.1798608	0.6808974	0.1669183
ENSBTAG00000016736	0.2938202	0.5055304	0.2962527
ENSBTAG00000016737	-0.454847	0.1182861	0.9270661
ENSBTAG00000016738	0.6014194	0.1232238	0.9093054
ENSBTAG00000016739	0.7823174	0.0772536	1.1120811
ENSBTAG00000016740	0.0337554	0.9178166	0.0372441
ENSBTAG00000016741	NA	NA	NA
ENSBTAG00000016742	-0.237682	0.6293712	0.2010931
ENSBTAG00000016743	0.3128021	0.3814454	0.4185676
ENSBTAG00000016744	-0.257188	0.5576627	0.2536284
ENSBTAG00000016746	NA	NA	NA
ENSBTAG00000016747	1.5810268	0.0101484	1.9936013
ENSBTAG00000016748	NA	NA	NA
ENSBTAG00000016750	-0.07762	0.831142	0.0803248
ENSBTAG00000016751	0.4415373	0.1098547	0.9591813
ENSBTAG00000016752	0.1378815	0.620442	0.2072988
ENSBTAG00000016754	0.8355632	0.0042261	2.3740613
ENSBTAG00000016757	0.184221	0.487251	0.3122473
ENSBTAG00000016758	-0.05676	0.8429594	0.0741933
ENSBTAG00000016760	-0.510017	0.0389533	1.4094557
ENSBTAG00000016761	NA	NA	NA
ENSBTAG00000016762	-0.26131	0.321754	0.4924761
ENSBTAG00000016763	-0.125962	0.6655711	0.1768055
ENSBTAG00000016764	0.064113	0.795904	0.0991393
ENSBTAG00000016765	-0.373818	0.3474665	0.459087
ENSBTAG00000016766	0.6620664	0.1265956	0.8975812
ENSBTAG00000016768	0.8863971	0.1195528	0.9224404
ENSBTAG00000016769	0.0496061	0.8397067	0.0758724
ENSBTAG00000016770	NA	NA	NA
ENSBTAG00000016771	-0.001914	0.9950351	0.0021616
ENSBTAG00000016772	-0.052114	0.8533738	0.0688607
ENSBTAG00000016775	0.3354028	0.2465823	0.6080381
ENSBTAG00000016776	0.2152669	0.6589104	0.1811737
ENSBTAG00000016779	-0.006288	0.980493	0.0085555
ENSBTAG00000016782	0.0114848	0.968426	0.0139336
ENSBTAG00000016783	0.0175603	0.9865433	0.0058838
ENSBTAG00000016784	NA	NA	NA
ENSBTAG00000016791	-0.197167	0.5036878	0.2978386
ENSBTAG00000016793	NA	NA	NA
ENSBTAG00000016794	NA	NA	NA

ENSBTAG00000016795	-0.365132	0.1533151	0.8144151
ENSBTAG00000016797	-0.007098	0.9812305	0.008229
ENSBTAG00000016799	0.2431701	0.4758755	0.3225067
ENSBTAG00000016800	-0.077737	0.8239403	0.0841043
ENSBTAG00000016801	-1.027653	0.0011423	2.9422149
ENSBTAG00000016804	-0.285398	0.2935195	0.5323631
ENSBTAG00000016805	-0.316154	0.3354888	0.4743219
ENSBTAG00000016806	-0.211443	0.3916791	0.4070696
ENSBTAG00000016810	0.0112889	0.9686723	0.0138231
ENSBTAG00000016813	0.245319	0.4216415	0.3750567
ENSBTAG00000016817	-0.348252	0.1854909	0.7316774
ENSBTAG00000016818	-0.305478	0.275594	0.5597303
ENSBTAG00000016819	0.7244984	0.0307382	1.5123214
ENSBTAG00000016820	NA	NA	NA
ENSBTAG00000016821	NA	NA	NA
ENSBTAG00000016822	0.1535748	0.540602	0.2671224
ENSBTAG00000016823	0.1230428	0.7251953	0.139545
ENSBTAG00000016824	0.059209	0.8429573	0.0741944
ENSBTAG00000016826	NA	NA	NA
ENSBTAG00000016827	-0.008638	0.9735593	0.0116376
ENSBTAG00000016828	0.5303521	0.060838	1.2158251
ENSBTAG00000016829	0.128038	0.7739492	0.1112875
ENSBTAG00000016830	0.2159114	0.4471034	0.349592
ENSBTAG00000016836	-0.190569	0.43886	0.357674
ENSBTAG00000016838	0.0412248	0.8849814	0.0530659
ENSBTAG00000016839	-0.431226	0.2419855	0.6162107
ENSBTAG00000016841	NA	NA	NA
ENSBTAG00000016844	NA	NA	NA
ENSBTAG00000016845	0.2522058	0.456634	0.3404317
ENSBTAG00000016846	0.3521183	0.1529221	0.8155296
ENSBTAG00000016847	NA	NA	NA
ENSBTAG00000016848	0.5040548	0.1033363	0.9857471
ENSBTAG00000016849	NA	NA	NA
ENSBTAG00000016851	-0.120543	0.6659033	0.1765888
ENSBTAG00000016852	0.1619573	0.6577495	0.1819395
ENSBTAG00000016854	NA	NA	NA
ENSBTAG00000016855	0.4103049	0.2513779	0.5996728
ENSBTAG00000016857	1.0006994	0.0050352	2.297982
ENSBTAG00000016858	0.0278501	0.9095359	0.0411801
ENSBTAG00000016864	NA	NA	NA
ENSBTAG00000016867	NA	NA	NA
ENSBTAG00000016869	0.7248023	0.068298	1.1655923
ENSBTAG00000016873	0.4741842	0.0858287	1.0663673
ENSBTAG00000016874	0.0513017	0.8891689	0.0510157
ENSBTAG00000016878	-0.078086	0.820912	0.0857034
ENSBTAG00000016879	0.2735225	0.4821636	0.3168056
ENSBTAG00000016880	NA	NA	NA
ENSBTAG00000016881	-0.167034	0.7547027	0.1222241
ENSBTAG00000016882	0.0695811	0.7965566	0.0987834
ENSBTAG00000016883	0.230418	0.6291203	0.2012663
ENSBTAG00000016885	NA	NA	NA
ENSBTAG00000016886	NA	NA	NA
ENSBTAG00000016887	-0.188157	0.4981153	0.3026702
ENSBTAG00000016890	-0.086062	0.7457695	0.1273954
ENSBTAG00000016891	NA	NA	NA
ENSBTAG00000016894	0.1815869	0.4705336	0.3274094
ENSBTAG00000016895	0.0868371	0.7937484	0.1003171
ENSBTAG00000016896	0.5556982	0.0423635	1.3730086

ENSBTAG00000016899	-0.311431	0.4266715	0.3699064
ENSBTAG00000016900	-0.172576	0.4878007	0.3117576
ENSBTAG00000016902	NA	NA	NA
ENSBTAG00000016903	-0.062944	0.9014277	0.0450691
ENSBTAG00000016904	NA	NA	NA
ENSBTAG00000016905	NA	NA	NA
ENSBTAG00000016906	NA	NA	NA
ENSBTAG00000016908	0.6726186	0.113896	0.9434917
ENSBTAG00000016910	NA	NA	NA
ENSBTAG00000016911	-0.971608	0.039864	1.3994194
ENSBTAG00000016912	-0.067807	0.8012886	0.096211
ENSBTAG00000016913	NA	NA	NA
ENSBTAG00000016915	-0.092299	0.8125288	0.0901612
ENSBTAG00000016918	0.2738443	0.3952095	0.4031726
ENSBTAG00000016920	NA	NA	NA
ENSBTAG00000016924	-0.195986	0.4393639	0.3571756
ENSBTAG00000016928	NA	NA	NA
ENSBTAG00000016929	0.3834522	0.3574075	0.4468364
ENSBTAG00000016931	NA	NA	NA
ENSBTAG00000016932	0.038989	0.8870626	0.0520457
ENSBTAG00000016933	-0.112497	0.7463783	0.127041
ENSBTAG00000016934	NA	NA	NA
ENSBTAG00000016936	0.2090724	0.597249	0.2238446
ENSBTAG00000016939	-0.336597	0.6371151	0.1957821
ENSBTAG00000016940	NA	NA	NA
ENSBTAG00000016943	-0.466047	0.0936616	1.0284383
ENSBTAG00000016944	0.2660429	0.5997097	0.2220589
ENSBTAG00000016948	NA	NA	NA
ENSBTAG00000016950	-0.009675	0.9719053	0.0123761
ENSBTAG00000016951	0.0737626	0.7838716	0.105755
ENSBTAG00000016952	0.1477148	0.5926941	0.2271694
ENSBTAG00000016954	0.0453702	0.9093652	0.0412617
ENSBTAG00000016956	0.3656226	0.1708295	0.7674371
ENSBTAG00000016957	-0.048302	0.872747	0.0591117
ENSBTAG00000016958	0.1398127	0.7594131	0.1195219
ENSBTAG00000016959	0.0423258	0.8823988	0.0543351
ENSBTAG00000016961	0.1744957	0.6720086	0.1726252
ENSBTAG00000016963	-0.385093	0.4913899	0.3085738
ENSBTAG00000016967	-0.052634	0.8488512	0.0711685
ENSBTAG00000016968	NA	NA	NA
ENSBTAG00000016969	-0.139709	0.6515814	0.1860313
ENSBTAG00000016972	NA	NA	NA
ENSBTAG00000016975	-0.019678	0.9381427	0.0277311
ENSBTAG00000016977	-0.292834	0.2602415	0.5846234
ENSBTAG00000016979	-0.086595	0.7262136	0.1389356
ENSBTAG00000016980	-0.243259	0.4064107	0.3910348
ENSBTAG00000016981	-0.023301	0.9668817	0.0146267
ENSBTAG00000016982	NA	NA	NA
ENSBTAG00000016984	0.0596024	0.8628991	0.06404
ENSBTAG00000016987	0.5300442	0.2257904	0.6462946
ENSBTAG00000016988	0.1598158	0.5427869	0.2653707
ENSBTAG00000016990	-0.161455	0.5201709	0.2838539
ENSBTAG00000016991	0.3252799	0.2292876	0.6396195
ENSBTAG00000016995	0.127062	0.655921	0.1831484
ENSBTAG00000016997	NA	NA	NA
ENSBTAG00000016998	-0.229536	0.6121503	0.213142
ENSBTAG00000016999	-0.391877	0.1141199	0.9426385
ENSBTAG00000017001	0.2117004	0.6078325	0.2162161

ENSBTAG00000017002	0.0423411	0.871106	0.059929
ENSBTAG00000017007	NA	NA	NA
ENSBTAG00000017009	-0.117039	0.6515795	0.1860326
ENSBTAG00000017010	-0.236259	0.4516572	0.3451911
ENSBTAG00000017011	-0.173169	0.7466736	0.1268692
ENSBTAG00000017012	NA	NA	NA
ENSBTAG00000017016	NA	NA	NA
ENSBTAG00000017017	0.1008977	0.7535286	0.1229003
ENSBTAG00000017019	0.3295023	0.2702773	0.5681904
ENSBTAG00000017020	0.2401545	0.669814	0.1740458
ENSBTAG00000017021	0.6225995	0.1846719	0.7335991
ENSBTAG00000017023	NA	NA	NA
ENSBTAG00000017024	-0.354354	0.4295307	0.3670058
ENSBTAG00000017026	NA	NA	NA
ENSBTAG00000017027	0.5964932	0.0645095	1.1903765
ENSBTAG00000017028	-0.243587	0.335149	0.4747621
ENSBTAG00000017029	NA	NA	NA
ENSBTAG00000017031	NA	NA	NA
ENSBTAG00000017032	-1.264042	0.0011422	2.9422514
ENSBTAG00000017033	NA	NA	NA
ENSBTAG00000017035	0.2582366	0.3389633	0.4698474
ENSBTAG00000017037	0.0544215	0.8404029	0.0755125
ENSBTAG00000017038	0.2971681	0.4804386	0.3183621
ENSBTAG00000017039	NA	NA	NA
ENSBTAG00000017040	0.2944444	0.5310582	0.2748579
ENSBTAG00000017041	NA	NA	NA
ENSBTAG00000017042	NA	NA	NA
ENSBTAG00000017043	NA	NA	NA
ENSBTAG00000017044	NA	NA	NA
ENSBTAG00000017048	-0.24136	0.548132	0.2611148
ENSBTAG00000017051	0.013293	0.9685027	0.0138992
ENSBTAG00000017053	-0.090869	0.7396308	0.130985
ENSBTAG00000017054	NA	NA	NA
ENSBTAG00000017055	NA	NA	NA
ENSBTAG00000017056	NA	NA	NA
ENSBTAG00000017060	0.0139795	0.9766315	0.0102693
ENSBTAG00000017061	0.1761438	0.5948803	0.2255704
ENSBTAG00000017063	NA	NA	NA
ENSBTAG00000017064	0.0446339	0.8697598	0.0606007
ENSBTAG00000017067	-0.190211	0.4611968	0.3361137
ENSBTAG00000017068	-0.026285	0.9305268	0.0312711
ENSBTAG00000017069	0.909099	0.002068	2.6844484
ENSBTAG00000017071	-0.209432	0.4949193	0.3054656
ENSBTAG00000017072	0.0073678	0.9841042	0.0069589
ENSBTAG00000017073	NA	NA	NA
ENSBTAG00000017074	0.0363423	0.9116669	0.0401638
ENSBTAG00000017075	NA	NA	NA
ENSBTAG00000017077	0.6613426	0.0151134	1.820637
ENSBTAG00000017078	-0.143203	0.5606304	0.2513233
ENSBTAG00000017079	0.3156026	0.2764876	0.5583243
ENSBTAG00000017081	-0.201056	0.4180144	0.3788088
ENSBTAG00000017082	0.597775	0.0330376	1.4809914
ENSBTAG00000017083	NA	NA	NA
ENSBTAG00000017085	0.2743457	0.4532004	0.3437098
ENSBTAG00000017086	-0.267313	0.2844344	0.5460179
ENSBTAG00000017087	0.4974813	0.1016828	0.9927526
ENSBTAG00000017090	0.5402507	0.1858515	0.7308339
ENSBTAG00000017091	0.24687	0.351457	0.4541278

ENSBTAG00000017094	-0.528896	0.2528902	0.597068
ENSBTAG00000017095	0.0958548	0.6962509	0.1572342
ENSBTAG00000017096	0.2363207	0.5106119	0.291909
ENSBTAG00000017097	NA	NA	NA
ENSBTAG00000017103	0.5754664	0.2166713	0.6641987
ENSBTAG00000017104	NA	NA	NA
ENSBTAG00000017105	NA	NA	NA
ENSBTAG00000017108	-0.383391	0.1901222	0.7209671
ENSBTAG00000017112	NA	NA	NA
ENSBTAG00000017113	NA	NA	NA
ENSBTAG00000017115	0.4479771	0.1708894	0.7672849
ENSBTAG00000017116	0.1817441	0.7425526	0.1292728
ENSBTAG00000017118	0.2817775	0.3675016	0.4347407
ENSBTAG00000017120	0.1638956	0.5308276	0.2750465
ENSBTAG00000017121	NA	NA	NA
ENSBTAG00000017122	0.384841	0.1210222	0.9171349
ENSBTAG00000017123	-0.058528	0.8604545	0.0652721
ENSBTAG00000017124	-0.530741	0.1110116	0.9546316
ENSBTAG00000017125	0.034143	0.8918684	0.0496992
ENSBTAG00000017128	-0.035498	0.8896306	0.0507903
ENSBTAG00000017129	-0.242619	0.3316641	0.4793015
ENSBTAG00000017132	NA	NA	NA
ENSBTAG00000017133	NA	NA	NA
ENSBTAG00000017135	0.0386721	0.8920604	0.0496057
ENSBTAG00000017136	NA	NA	NA
ENSBTAG00000017137	-0.158331	0.5460661	0.2627548
ENSBTAG00000017138	NA	NA	NA
ENSBTAG00000017139	-0.458731	0.0635395	1.1969565
ENSBTAG00000017140	0.2081825	0.5428962	0.2652832
ENSBTAG00000017141	-0.329453	0.3111999	0.5069605
ENSBTAG00000017143	0.2233759	0.4225553	0.3741165
ENSBTAG00000017146	NA	NA	NA
ENSBTAG00000017147	-0.193283	0.44526	0.3513863
ENSBTAG00000017150	NA	NA	NA
ENSBTAG00000017151	0.2699087	0.4949599	0.30543
ENSBTAG00000017155	-0.156577	0.6689771	0.1745888
ENSBTAG00000017157	NA	NA	NA
ENSBTAG00000017158	NA	NA	NA
ENSBTAG00000017160	-0.046349	0.9032106	0.044211
ENSBTAG00000017161	0.1749834	0.5387421	0.2686191
ENSBTAG00000017162	0.492496	0.2360185	0.627054
ENSBTAG00000017164	0.0980113	0.6933136	0.1590703
ENSBTAG00000017165	0.2476531	0.5056535	0.296147
ENSBTAG00000017167	NA	NA	NA
ENSBTAG00000017170	NA	NA	NA
ENSBTAG00000017174	NA	NA	NA
ENSBTAG00000017175	-0.09133	0.7558814	0.1215463
ENSBTAG00000017177	-0.076553	0.7623699	0.1178343
ENSBTAG00000017179	-0.170726	0.5979845	0.2233101
ENSBTAG00000017180	NA	NA	NA
ENSBTAG00000017181	-0.071106	0.793173	0.1006321
ENSBTAG00000017183	0.0072091	0.9782378	0.0095556
ENSBTAG00000017184	0.3538958	0.2303579	0.637597
ENSBTAG00000017187	-1.348867	0.0056546	2.2475977
ENSBTAG00000017188	0.654701	0.1461305	0.8352591
ENSBTAG00000017189	-0.262306	0.489196	0.3105171
ENSBTAG00000017191	0.0386986	0.8847187	0.0531948
ENSBTAG00000017194	NA	NA	NA

ENSBTAG00000017195	-0.208956	0.6153785	0.2108577
ENSBTAG00000017196	0.3391232	0.2214775	0.6546705
ENSBTAG00000017200	0.8445957	0.0347166	1.4594624
ENSBTAG00000017201	-0.492618	0.1652177	0.7819435
ENSBTAG00000017205	NA	NA	NA
ENSBTAG00000017211	NA	NA	NA
ENSBTAG00000017212	0.1491472	0.5662537	0.2469889
ENSBTAG00000017213	0.0681296	0.8050641	0.0941695
ENSBTAG00000017214	NA	NA	NA
ENSBTAG00000017215	0.135349	0.7307977	0.1362028
ENSBTAG00000017216	NA	NA	NA
ENSBTAG00000017218	-0.034529	0.9293538	0.0318189
ENSBTAG00000017219	0.0725016	0.8525837	0.069263
ENSBTAG00000017221	NA	NA	NA
ENSBTAG00000017222	NA	NA	NA
ENSBTAG00000017223	0.8929025	0.0554999	1.2557077
ENSBTAG00000017225	-0.048787	0.8886965	0.0512465
ENSBTAG00000017227	NA	NA	NA
ENSBTAG00000017228	NA	NA	NA
ENSBTAG00000017232	-0.048385	0.8622272	0.0643783
ENSBTAG00000017233	0.1924744	0.5336288	0.2727607
ENSBTAG00000017239	-0.085953	0.8153464	0.0886578
ENSBTAG00000017240	0.1689817	0.629928	0.2007091
ENSBTAG00000017242	NA	NA	NA
ENSBTAG00000017243	NA	NA	NA
ENSBTAG00000017244	-0.039427	0.8732828	0.0588451
ENSBTAG00000017245	-0.458388	0.0990719	1.0040493
ENSBTAG00000017246	-0.077416	0.7745315	0.1109609
ENSBTAG00000017249	NA	NA	NA
ENSBTAG00000017251	NA	NA	NA
ENSBTAG00000017252	NA	NA	NA
ENSBTAG00000017253	-0.201026	0.4807232	0.3181049
ENSBTAG00000017255	0.0172433	0.945255	0.024451
ENSBTAG00000017256	NA	NA	NA
ENSBTAG00000017258	0.4867492	0.0942876	1.0255452
ENSBTAG00000017260	-0.971751	0.0033201	2.4788461
ENSBTAG00000017263	0.0189809	0.948674	0.022883
ENSBTAG00000017265	-0.193382	0.4362209	0.3602935
ENSBTAG00000017266	-0.267522	0.350565	0.4552314
ENSBTAG00000017267	0.1406218	0.6044608	0.2186319
ENSBTAG00000017268	-0.22219	0.6203791	0.2073429
ENSBTAG00000017271	NA	NA	NA
ENSBTAG00000017273	NA	NA	NA
ENSBTAG00000017275	0.1102252	0.7078918	0.1500331
ENSBTAG00000017276	NA	NA	NA
ENSBTAG00000017277	NA	NA	NA
ENSBTAG00000017278	0.2675152	0.3643881	0.4384358
ENSBTAG00000017279	0.0345114	0.8946861	0.0483293
ENSBTAG00000017280	0.1374426	0.7563086	0.121301
ENSBTAG00000017281	0.2079961	0.4301629	0.366367
ENSBTAG00000017282	NA	NA	NA
ENSBTAG00000017283	0.4999601	0.1119116	0.951125
ENSBTAG00000017284	NA	NA	NA
ENSBTAG00000017287	0.6509599	0.1848339	0.7332184
ENSBTAG00000017289	0.941393	0.0072698	2.1384747
ENSBTAG00000017291	0.4335072	0.3069519	0.5129297
ENSBTAG00000017294	NA	NA	NA
ENSBTAG00000017296	NA	NA	NA

ENSBTAG00000017298	-0.093276	0.7219313	0.1415042
ENSBTAG00000017299	-0.180545	0.4685004	0.3292901
ENSBTAG00000017301	NA	NA	NA
ENSBTAG00000017306	0.1999375	0.685551	0.1639602
ENSBTAG00000017310	-0.847755	0.0701558	1.1539362
ENSBTAG00000017313	NA	NA	NA
ENSBTAG00000017318	0.767509	0.111821	0.9514765
ENSBTAG00000017321	-0.269369	0.2842845	0.5462468
ENSBTAG00000017325	0.2931162	0.3384597	0.470493
ENSBTAG00000017326	0.2909879	0.3536937	0.4513727
ENSBTAG00000017328	-0.285748	0.4863554	0.3130463
ENSBTAG00000017329	0.0817021	0.8435452	0.0738916
ENSBTAG00000017330	-0.185801	0.8137544	0.0895067
ENSBTAG00000017333	-0.237752	0.6658147	0.1766467
ENSBTAG00000017335	NA	NA	NA
ENSBTAG00000017338	NA	NA	NA
ENSBTAG00000017339	0.1661026	0.6988383	0.1556233
ENSBTAG00000017343	NA	NA	NA
ENSBTAG00000017345	NA	NA	NA
ENSBTAG00000017346	-0.097541	0.7931714	0.100633
ENSBTAG00000017349	0.1887295	0.5007849	0.3003487
ENSBTAG00000017350	NA	NA	NA
ENSBTAG00000017352	-0.512577	0.0560053	1.2517707
ENSBTAG00000017354	-0.300259	0.4029369	0.394763
ENSBTAG00000017355	0.0402049	0.9012857	0.0451375
ENSBTAG00000017357	NA	NA	NA
ENSBTAG00000017360	-0.061856	0.8457782	0.0727435
ENSBTAG00000017361	-0.208919	0.4561305	0.3409109
ENSBTAG00000017362	-0.092188	0.7748217	0.1107982
ENSBTAG00000017363	0.012285	0.9699715	0.013241
ENSBTAG00000017365	-0.312385	0.2072311	0.6835452
ENSBTAG00000017366	NA	NA	NA
ENSBTAG00000017367	0.4444001	0.1211482	0.9166831
ENSBTAG00000017368	-0.020251	0.9363077	0.0285814
ENSBTAG00000017369	0.0822947	0.869375	0.0607929
ENSBTAG00000017370	-0.283388	0.5318647	0.2741988
ENSBTAG00000017371	NA	NA	NA
ENSBTAG00000017373	-0.558187	0.0717755	1.1440237
ENSBTAG00000017375	-0.053077	0.8998074	0.0458504
ENSBTAG00000017376	NA	NA	NA
ENSBTAG00000017378	NA	NA	NA
ENSBTAG00000017379	0.2235779	0.3716292	0.4298902
ENSBTAG00000017380	0.2197321	0.4681436	0.3296209
ENSBTAG00000017381	NA	NA	NA
ENSBTAG00000017382	0.6855856	0.0489119	1.3105854
ENSBTAG00000017385	NA	NA	NA
ENSBTAG00000017386	0.1425611	0.6582204	0.1816286
ENSBTAG00000017387	NA	NA	NA
ENSBTAG00000017388	-0.086828	0.7943681	0.0999782
ENSBTAG00000017389	0.0638309	0.8047854	0.0943199
ENSBTAG00000017390	NA	NA	NA
ENSBTAG00000017391	NA	NA	NA
ENSBTAG00000017393	0.4235948	0.2189984	0.6595591
ENSBTAG00000017395	-0.000182	1	0
ENSBTAG00000017397	0.1072521	0.7104173	0.1484865
ENSBTAG00000017401	-0.040718	0.8811492	0.0549506
ENSBTAG00000017402	0.1183604	0.642888	0.1918647
ENSBTAG00000017404	NA	NA	NA

ENSBTAG00000017405	-0.267551	0.5547025	0.2559399
ENSBTAG00000017407	-0.149512	0.7117761	0.1476566
ENSBTAG00000017408	NA	NA	NA
ENSBTAG00000017411	NA	NA	NA
ENSBTAG00000017412	-0.586695	0.3819979	0.4179391
ENSBTAG00000017416	-0.199659	0.4216634	0.3750341
ENSBTAG00000017418	-0.359084	0.2818908	0.549919
ENSBTAG00000017419	0.0585096	0.8220638	0.0850945
ENSBTAG00000017420	NA	NA	NA
ENSBTAG00000017422	0.3370805	0.24213	0.6159515
ENSBTAG00000017423	0.1120203	0.6930175	0.1592558
ENSBTAG00000017424	-0.148164	0.6160344	0.210395
ENSBTAG00000017425	NA	NA	NA
ENSBTAG00000017426	0.1485437	0.5561667	0.254795
ENSBTAG00000017427	NA	NA	NA
ENSBTAG00000017429	-0.545401	0.0385437	1.4140465
ENSBTAG00000017430	-0.400122	0.3471058	0.4595381
ENSBTAG00000017431	NA	NA	NA
ENSBTAG00000017434	NA	NA	NA
ENSBTAG00000017435	0.3559286	0.4034541	0.3942059
ENSBTAG00000017436	NA	NA	NA
ENSBTAG00000017437	-0.363744	0.3294745	0.4821781
ENSBTAG00000017438	NA	NA	NA
ENSBTAG00000017439	0.1058584	0.7483716	0.1258827
ENSBTAG00000017440	0.0379053	0.8926956	0.0492966
ENSBTAG00000017441	0.0526126	0.8406003	0.0754105
ENSBTAG00000017442	-0.308886	0.5289046	0.2766227
ENSBTAG00000017448	-0.36237	0.2256428	0.6465786
ENSBTAG00000017450	0.3042906	0.2697439	0.5690484
ENSBTAG00000017451	-0.07101	0.7774063	0.109352
ENSBTAG00000017452	-0.635773	0.196296	0.7070885
ENSBTAG00000017455	-0.737853	0.0082711	2.0824354
ENSBTAG00000017456	NA	NA	NA
ENSBTAG00000017457	-0.011646	0.9731191	0.011834
ENSBTAG00000017458	NA	NA	NA
ENSBTAG00000017459	0.0945927	0.8532548	0.0689213
ENSBTAG00000017460	-0.105028	0.7769961	0.1095812
ENSBTAG00000017461	-0.357858	0.2266299	0.6446827
ENSBTAG00000017462	-0.079495	0.7677541	0.1147779
ENSBTAG00000017463	-0.159378	0.6303813	0.2003967
ENSBTAG00000017465	0.0141082	0.9547582	0.0201066
ENSBTAG00000017466	NA	NA	NA
ENSBTAG00000017468	-0.333352	0.2798026	0.5531483
ENSBTAG00000017469	-0.040411	0.8921406	0.0495667
ENSBTAG00000017471	-1.345767	0.0039345	2.4051055
ENSBTAG00000017473	NA	NA	NA
ENSBTAG00000017475	-0.110201	0.6603596	0.1802195
ENSBTAG00000017480	NA	NA	NA
ENSBTAG00000017482	-0.274908	0.530678	0.2751689
ENSBTAG00000017483	NA	NA	NA
ENSBTAG00000017486	-0.088244	0.7991314	0.0973818
ENSBTAG00000017488	-0.308029	0.3532801	0.4518808
ENSBTAG00000017489	1.2594059	0.0123389	1.9087218
ENSBTAG00000017490	0.8199424	0.0078411	2.1056252
ENSBTAG00000017492	-0.028826	0.908207	0.0418151
ENSBTAG00000017493	-0.169132	0.5665341	0.246774
ENSBTAG00000017496	-0.025411	0.9261775	0.0333058
ENSBTAG00000017500	NA	NA	NA

ENSBTAG00000017502	NA	NA	NA
ENSBTAG00000017504	NA	NA	NA
ENSBTAG00000017505	0.2133177	0.617471	0.2093834
ENSBTAG00000017507	NA	NA	NA
ENSBTAG00000017508	0.1412774	0.816129	0.0882412
ENSBTAG00000017509	-0.114125	0.6515997	0.1860191
ENSBTAG00000017512	-0.139702	0.6810258	0.1668365
ENSBTAG00000017514	0.2510896	0.5698505	0.2442391
ENSBTAG00000017515	NA	NA	NA
ENSBTAG00000017517	0.1843799	0.5173409	0.2862232
ENSBTAG00000017521	NA	NA	NA
ENSBTAG00000017524	-0.792551	0.1564254	0.8056928
ENSBTAG00000017525	0.1550749	0.6291512	0.201245
ENSBTAG00000017527	0.7417083	0.0203253	1.691962
ENSBTAG00000017528	-0.828249	0.1656775	0.7807365
ENSBTAG00000017529	NA	NA	NA
ENSBTAG00000017530	-0.178851	0.5025364	0.2988325
ENSBTAG00000017533	NA	NA	NA
ENSBTAG00000017536	NA	NA	NA
ENSBTAG00000017537	0.3862167	0.1970055	0.7055217
ENSBTAG00000017540	0.3702641	0.3184469	0.496963
ENSBTAG00000017541	NA	NA	NA
ENSBTAG00000017542	-0.298033	0.5764665	0.2392259
ENSBTAG00000017543	0.2750619	0.3929297	0.4056852
ENSBTAG00000017545	0.0679835	0.7906804	0.101999
ENSBTAG00000017547	0.1336478	0.6330718	0.198547
ENSBTAG00000017549	0.5376896	0.0541598	1.2663229
ENSBTAG00000017550	0.0187918	0.9454606	0.0243566
ENSBTAG00000017551	-0.024456	0.9450775	0.0245326
ENSBTAG00000017554	0.1608095	0.5663365	0.2469254
ENSBTAG00000017557	0.0601169	0.8208077	0.0857586
ENSBTAG00000017560	-0.328687	0.2997682	0.5232144
ENSBTAG00000017561	NA	NA	NA
ENSBTAG00000017564	0.5967173	0.2658646	0.5753395
ENSBTAG00000017565	-0.082651	0.7468708	0.1267545
ENSBTAG00000017566	-0.456436	0.2371124	0.6250457
ENSBTAG00000017567	-0.254	0.5634559	0.2491401
ENSBTAG00000017569	0.1900368	0.5592623	0.2523845
ENSBTAG00000017571	-0.00029	1	0
ENSBTAG00000017573	-0.072044	0.7763707	0.1099309
ENSBTAG00000017574	-0.204163	0.4183862	0.3784226
ENSBTAG00000017580	-0.150618	0.6661191	0.1764481
ENSBTAG00000017582	NA	NA	NA
ENSBTAG00000017584	0.330116	0.2733755	0.5632405
ENSBTAG00000017592	1.6270414	8.86E-06	5.0525271
ENSBTAG00000017593	NA	NA	NA
ENSBTAG00000017594	NA	NA	NA
ENSBTAG00000017595	-0.142391	0.6920029	0.1598921
ENSBTAG00000017597	0.2279898	0.4582212	0.3389249
ENSBTAG00000017599	0.4396806	0.380353	0.4198131
ENSBTAG00000017604	-0.089516	0.7765498	0.1098307
ENSBTAG00000017605	0.0597784	0.8137513	0.0895083
ENSBTAG00000017610	0.4623783	0.1815287	0.7410546
ENSBTAG00000017611	-0.402102	0.1262505	0.898767
ENSBTAG00000017613	0.3980297	0.2657522	0.5755232
ENSBTAG00000017616	-0.758427	0.005498	2.2597969
ENSBTAG00000017617	NA	NA	NA
ENSBTAG00000017618	-0.06626	0.8520388	0.0695406

ENSBTAG00000017622	-0.193753	0.6330922	0.1985331
ENSBTAG00000017624	-1.479396	0.0785255	1.1049891
ENSBTAG00000017626	-0.317927	0.2114639	0.6747637
ENSBTAG00000017628	NA	NA	NA
ENSBTAG00000017629	NA	NA	NA
ENSBTAG00000017631	0.1355258	0.6799482	0.1675242
ENSBTAG00000017632	0.354371	0.457379	0.3397238
ENSBTAG00000017633	-0.17482	0.5039178	0.2976403
ENSBTAG00000017636	0.1309866	0.6139479	0.2118685
ENSBTAG00000017639	-0.589143	0.0888309	1.051436
ENSBTAG00000017641	NA	NA	NA
ENSBTAG00000017642	NA	NA	NA
ENSBTAG00000017644	NA	NA	NA
ENSBTAG00000017649	-0.013208	0.9829378	0.0074739
ENSBTAG00000017650	NA	NA	NA
ENSBTAG00000017651	1.6737375	0.0049084	2.3090576
ENSBTAG00000017652	0.0010279	0.998021	0.0008603
ENSBTAG00000017655	-0.318886	0.2856818	0.5441174
ENSBTAG00000017656	0.3197445	0.2404274	0.619016
ENSBTAG00000017659	NA	NA	NA
ENSBTAG00000017660	NA	NA	NA
ENSBTAG00000017661	-0.691487	0.1457961	0.836254
ENSBTAG00000017662	-0.090153	0.7176632	0.1440793
ENSBTAG00000017663	NA	NA	NA
ENSBTAG00000017664	NA	NA	NA
ENSBTAG00000017665	0.1093912	0.7094895	0.1490541
ENSBTAG00000017666	-0.659662	0.0085742	2.0668079
ENSBTAG00000017670	0.0658095	0.8821073	0.0544786
ENSBTAG00000017672	NA	NA	NA
ENSBTAG00000017674	NA	NA	NA
ENSBTAG00000017676	0.1535689	0.7510609	0.1243248
ENSBTAG00000017677	NA	NA	NA
ENSBTAG00000017678	NA	NA	NA
ENSBTAG00000017679	NA	NA	NA
ENSBTAG00000017680	-1.096456	0.0106554	1.9724305
ENSBTAG00000017681	1.5331555	0.0023299	2.6326674
ENSBTAG00000017682	-0.116575	0.6544635	0.1841146
ENSBTAG00000017683	-0.593374	0.1822656	0.7392952
ENSBTAG00000017684	NA	NA	NA
ENSBTAG00000017685	0.44996	0.1531234	0.8149586
ENSBTAG00000017689	0.3428404	0.3071495	0.5126502
ENSBTAG00000017690	-1.119426	0.0009351	3.0291573
ENSBTAG00000017694	0.0917313	0.8075962	0.0928057
ENSBTAG00000017695	0.1297154	0.7757133	0.1102988
ENSBTAG00000017697	NA	NA	NA
ENSBTAG00000017701	NA	NA	NA
ENSBTAG00000017704	0.6156967	0.0832116	1.0798163
ENSBTAG00000017706	0.4901434	0.1454058	0.8374183
ENSBTAG00000017709	-0.935923	0.0071214	2.1474343
ENSBTAG00000017710	-0.12116	0.6254381	0.2038157
ENSBTAG00000017711	0.2163641	0.4122483	0.3848411
ENSBTAG00000017713	-0.341877	0.205281	0.6876513
ENSBTAG00000017714	NA	NA	NA
ENSBTAG00000017715	-0.075438	0.776572	0.1098183
ENSBTAG00000017716	NA	NA	NA
ENSBTAG00000017717	NA	NA	NA
ENSBTAG00000017718	NA	NA	NA
ENSBTAG00000017719	-0.815914	0.0032425	2.4891234

ENSBTAG00000017721	-0.094551	0.7881405	0.1033964
ENSBTAG00000017722	NA	NA	NA
ENSBTAG00000017727	-0.313155	0.2080504	0.6818315
ENSBTAG00000017729	0.2325086	0.4583575	0.3387957
ENSBTAG00000017731	0.4484446	0.2724097	0.5647775
ENSBTAG00000017733	-0.132981	0.7022906	0.1534832
ENSBTAG00000017734	0.0045668	0.9876349	0.0054036
ENSBTAG00000017738	NA	NA	NA
ENSBTAG00000017739	NA	NA	NA
ENSBTAG00000017740	NA	NA	NA
ENSBTAG00000017741	0.0654277	0.8942646	0.0485339
ENSBTAG00000017743	0.2172969	0.5182555	0.2854561
ENSBTAG00000017744	-0.306864	0.2874297	0.5414684
ENSBTAG00000017745	-0.290271	0.25515	0.5932044
ENSBTAG00000017746	0.4975172	0.2827533	0.5485923
ENSBTAG00000017747	0.1594691	0.6437438	0.191287
ENSBTAG00000017748	0.6455001	0.1345091	0.8712484
ENSBTAG00000017750	-0.143292	0.6773748	0.1691709
ENSBTAG00000017751	NA	NA	NA
ENSBTAG00000017752	NA	NA	NA
ENSBTAG00000017753	0.2653529	0.2989606	0.5243861
ENSBTAG00000017755	-0.043064	0.8647363	0.0631163
ENSBTAG00000017759	-0.019069	0.938521	0.027556
ENSBTAG00000017761	-0.006293	0.9806334	0.0084933
ENSBTAG00000017763	-0.451336	0.1726466	0.7628419
ENSBTAG00000017764	0.3549335	0.2375298	0.624282
ENSBTAG00000017765	-0.413215	0.2457585	0.6094914
ENSBTAG00000017767	NA	NA	NA
ENSBTAG00000017769	NA	NA	NA
ENSBTAG00000017770	0.0281634	0.9097127	0.0410958
ENSBTAG00000017771	-0.429812	0.1114847	0.9527846
ENSBTAG00000017775	-0.283397	0.4586663	0.3385032
ENSBTAG00000017776	-0.018506	0.943192	0.0253999
ENSBTAG00000017779	-0.257167	0.3034926	0.5178519
ENSBTAG00000017780	NA	NA	NA
ENSBTAG00000017781	0.2320776	0.60202	0.220389
ENSBTAG00000017783	-0.200631	0.5544291	0.256154
ENSBTAG00000017785	NA	NA	NA
ENSBTAG00000017786	NA	NA	NA
ENSBTAG00000017788	0.1242481	0.6448711	0.1905271
ENSBTAG00000017790	0.7427755	0.1047702	0.9797621
ENSBTAG00000017793	NA	NA	NA
ENSBTAG00000017794	NA	NA	NA
ENSBTAG00000017797	-0.483967	0.2965556	0.5278939
ENSBTAG00000017798	-0.166707	0.5159377	0.2874028
ENSBTAG00000017799	0.0125663	0.9728155	0.0119695
ENSBTAG00000017801	0.2254203	0.4073588	0.3900229
ENSBTAG00000017802	-0.494811	0.274669	0.5611903
ENSBTAG00000017803	0.2497109	0.4026481	0.3950743
ENSBTAG00000017804	-0.290004	0.2960825	0.5285872
ENSBTAG00000017805	0.8737051	0.0255809	1.5920842
ENSBTAG00000017808	0.2599808	0.6251328	0.2040277
ENSBTAG00000017809	0.0615005	0.8075999	0.0928038
ENSBTAG00000017810	NA	NA	NA
ENSBTAG00000017811	0.1844163	0.5566396	0.2544259
ENSBTAG00000017812	0.6484769	0.0108249	1.9655755
ENSBTAG00000017814	-0.03197	0.9274869	0.0326922
ENSBTAG00000017815	0.0758118	0.7742061	0.1111434

ENSBTAG00000017816	-0.072272	0.7701448	0.1134276
ENSBTAG00000017818	NA	NA	NA
ENSBTAG00000017819	-0.227589	0.538878	0.2685095
ENSBTAG00000017823	NA	NA	NA
ENSBTAG00000017824	-0.363776	0.3321153	0.4787112
ENSBTAG00000017825	0.0828186	0.7531354	0.1231269
ENSBTAG00000017826	0.0393116	0.9124842	0.0397747
ENSBTAG00000017829	NA	NA	NA
ENSBTAG00000017830	-0.232535	0.4202202	0.3765231
ENSBTAG00000017831	0.1854838	0.5111486	0.2914528
ENSBTAG00000017832	0.0595354	0.864317	0.0633269
ENSBTAG00000017833	-0.346225	0.1714715	0.7658081
ENSBTAG00000017834	0.0724359	0.828956	0.0814685
ENSBTAG00000017835	-0.198187	0.6846408	0.1645372
ENSBTAG00000017836	0.0911061	0.7743665	0.1110535
ENSBTAG00000017837	NA	NA	NA
ENSBTAG00000017839	0.0089643	0.9880768	0.0052093
ENSBTAG00000017840	0.1051321	0.7262515	0.138913
ENSBTAG00000017843	NA	NA	NA
ENSBTAG00000017844	NA	NA	NA
ENSBTAG00000017845	0.1631029	0.5849922	0.2328499
ENSBTAG00000017846	0.666164	0.0135143	1.869207
ENSBTAG00000017847	-0.035745	0.9114244	0.0402793
ENSBTAG00000017850	-0.092376	0.7124649	0.1472365
ENSBTAG00000017851	-0.173068	0.5463587	0.2625221
ENSBTAG00000017852	0.3290601	0.445381	0.3512683
ENSBTAG00000017855	0.3944915	0.1990541	0.701029
ENSBTAG00000017856	-0.116804	0.8071863	0.0930262
ENSBTAG00000017860	-0.001844	0.9955269	0.001947
ENSBTAG00000017861	NA	NA	NA
ENSBTAG00000017863	-0.082498	0.7921638	0.101185
ENSBTAG00000017866	0.5862074	0.0225522	1.6468111
ENSBTAG00000017867	NA	NA	NA
ENSBTAG00000017868	0.046706	0.8681792	0.0613906
ENSBTAG00000017869	0.4273852	0.1072259	0.9697002
ENSBTAG00000017871	0.0170592	0.9631353	0.0163127
ENSBTAG00000017872	0.2366957	0.426432	0.3701502
ENSBTAG00000017873	0.2437928	0.382545	0.4173175
ENSBTAG00000017875	0.3678965	0.3451404	0.4620042
ENSBTAG00000017877	NA	NA	NA
ENSBTAG00000017879	-0.203917	0.6011272	0.2210336
ENSBTAG00000017882	NA	NA	NA
ENSBTAG00000017883	NA	NA	NA
ENSBTAG00000017885	-0.054236	0.8394578	0.0760011
ENSBTAG00000017886	NA	NA	NA
ENSBTAG00000017889	0.0109138	0.9656607	0.0151754
ENSBTAG00000017890	0.2876348	0.3912575	0.4075373
ENSBTAG00000017892	NA	NA	NA
ENSBTAG00000017894	0.532662	0.1080008	0.9665729
ENSBTAG00000017895	NA	NA	NA
ENSBTAG00000017896	-0.222403	0.6428624	0.191882
ENSBTAG00000017901	-0.251528	0.5496397	0.2599219
ENSBTAG00000017905	0.4991455	0.1016794	0.9927668
ENSBTAG00000017907	0.3318613	0.2706369	0.567613
ENSBTAG00000017911	NA	NA	NA
ENSBTAG00000017912	NA	NA	NA
ENSBTAG00000017913	0.132375	0.7211526	0.1419728
ENSBTAG00000017919	-0.050711	0.8685012	0.0612296

ENSBTAG00000017921	0.2592581	0.4561436	0.3408984
ENSBTAG00000017922	0.3008856	0.4914975	0.3084787
ENSBTAG00000017925	0.3614231	0.3108189	0.5074926
ENSBTAG00000017929	NA	NA	NA
ENSBTAG00000017932	0.0012965	1	0
ENSBTAG00000017936	NA	NA	NA
ENSBTAG00000017939	-0.086759	0.8480364	0.0715855
ENSBTAG00000017941	0.0983096	0.789954	0.1023982
ENSBTAG00000017942	0.142012	0.7233494	0.1406519
ENSBTAG00000017943	NA	NA	NA
ENSBTAG00000017945	0.2527797	0.4173565	0.3794928
ENSBTAG00000017946	NA	NA	NA
ENSBTAG00000017948	NA	NA	NA
ENSBTAG00000017949	NA	NA	NA
ENSBTAG00000017950	NA	NA	NA
ENSBTAG00000017951	0.0262434	0.9384351	0.0275957
ENSBTAG00000017955	NA	NA	NA
ENSBTAG00000017956	0.0163372	0.9631153	0.0163217
ENSBTAG00000017957	0.0450111	0.8581608	0.0664313
ENSBTAG00000017958	-0.014108	0.9650946	0.0154301
ENSBTAG00000017964	-0.26024	0.5086137	0.2936119
ENSBTAG00000017967	-0.139331	0.6546185	0.1840117
ENSBTAG00000017969	-0.257172	0.6647158	0.177364
ENSBTAG00000017970	0.159633	0.635436	0.1969282
ENSBTAG00000017976	0.1087561	0.7154443	0.1454241
ENSBTAG00000017981	NA	NA	NA
ENSBTAG00000017982	NA	NA	NA
ENSBTAG00000017983	0.1800883	0.6134342	0.212232
ENSBTAG00000017986	NA	NA	NA
ENSBTAG00000017992	-0.305772	0.2322807	0.6339868
ENSBTAG00000017994	0.3889875	0.1625739	0.7889491
ENSBTAG00000017996	-0.104093	0.6740197	0.1713274
ENSBTAG00000017999	0.043632	0.8731289	0.0589216
ENSBTAG00000018000	0.0856134	0.8277363	0.082108
ENSBTAG00000018002	0.5548317	0.1840238	0.7351259
ENSBTAG00000018003	0.0512957	0.8624519	0.0642651
ENSBTAG00000018004	NA	NA	NA
ENSBTAG00000018007	0.6026336	0.128432	0.8913267
ENSBTAG00000018009	NA	NA	NA
ENSBTAG00000018010	NA	NA	NA
ENSBTAG00000018011	NA	NA	NA
ENSBTAG00000018013	-0.135553	0.6932388	0.1591171
ENSBTAG00000018015	NA	NA	NA
ENSBTAG00000018016	1.1612678	0.0010344	2.9853279
ENSBTAG00000018019	NA	NA	NA
ENSBTAG00000018020	NA	NA	NA
ENSBTAG00000018022	0.4478771	0.2524183	0.5978792
ENSBTAG00000018024	-0.790551	0.0051106	2.2915318
ENSBTAG00000018025	NA	NA	NA
ENSBTAG00000018026	0.0401508	0.8778949	0.0565575
ENSBTAG00000018031	NA	NA	NA
ENSBTAG00000018033	-0.01804	0.9640585	0.0158966
ENSBTAG00000018035	-0.178673	0.5062728	0.2956154
ENSBTAG00000018036	-0.002615	0.9924419	0.0032949
ENSBTAG00000018037	0.1033341	0.8121459	0.090366
ENSBTAG00000018040	0.1102417	0.7064501	0.1509185
ENSBTAG00000018041	-0.4934	0.0595931	1.2248037
ENSBTAG00000018043	NA	NA	NA

ENSBTAG00000018044	0.2370494	0.4473847	0.3493189
ENSBTAG00000018045	0.2401192	0.4032776	0.394396
ENSBTAG00000018046	-0.04788	0.8582042	0.0664094
ENSBTAG00000018048	0.4267152	0.2614677	0.5825819
ENSBTAG00000018049	0.1458663	0.6147291	0.2113162
ENSBTAG00000018050	0.0279751	0.9463101	0.0239665
ENSBTAG00000018052	0.5870209	0.0425395	1.3712076
ENSBTAG00000018053	-0.418657	0.09368	1.0283533
ENSBTAG00000018054	NA	NA	NA
ENSBTAG00000018057	0.2793754	0.4873332	0.312174
ENSBTAG00000018058	NA	NA	NA
ENSBTAG00000018059	NA	NA	NA
ENSBTAG00000018060	-0.111623	0.6560915	0.1830356
ENSBTAG00000018061	0.26659	0.3459545	0.460981
ENSBTAG00000018062	0.2683682	0.5377505	0.2694192
ENSBTAG00000018063	0.1994709	0.5873152	0.2311288
ENSBTAG00000018064	NA	NA	NA
ENSBTAG00000018065	0.0754905	0.7712061	0.1128295
ENSBTAG00000018067	0.04582	0.8820778	0.0544931
ENSBTAG00000018069	NA	NA	NA
ENSBTAG00000018070	-0.318058	0.3149142	0.5018077
ENSBTAG00000018071	-0.079564	0.7687662	0.1142057
ENSBTAG00000018072	0.0833714	0.7928555	0.100806
ENSBTAG00000018073	0.1905622	0.5721535	0.2424874
ENSBTAG00000018074	0.4027728	0.1826348	0.7384165
ENSBTAG00000018082	NA	NA	NA
ENSBTAG00000018084	0.2107222	0.5672996	0.2461875
ENSBTAG00000018085	NA	NA	NA
ENSBTAG00000018086	-0.169423	0.6753963	0.1704413
ENSBTAG00000018088	-1.111052	0.0003222	3.4918664
ENSBTAG00000018090	0.1903503	0.6510626	0.1863772
ENSBTAG00000018093	-0.206078	0.4425951	0.3539934
ENSBTAG00000018094	1.0401034	0.0247691	1.6060901
ENSBTAG00000018097	-0.537601	0.0742613	1.1292376
ENSBTAG00000018101	-0.130821	0.6336163	0.1981736
ENSBTAG00000018103	-0.169827	0.5153805	0.287872
ENSBTAG00000018105	-0.788815	0.0259161	1.5864298
ENSBTAG00000018106	NA	NA	NA
ENSBTAG00000018108	0.1011312	0.7630842	0.1174275
ENSBTAG00000018112	-0.028753	0.9095402	0.0411781
ENSBTAG00000018114	0.3176088	0.2143255	0.6689261
ENSBTAG00000018115	-0.510674	0.0555636	1.2552095
ENSBTAG00000018116	0.0810983	0.8206699	0.0858315
ENSBTAG00000018119	NA	NA	NA
ENSBTAG00000018123	0.1843924	0.537976	0.2692371
ENSBTAG00000018125	NA	NA	NA
ENSBTAG00000018126	0.08316	0.7824633	0.106536
ENSBTAG00000018127	-0.242371	0.4905108	0.3093514
ENSBTAG00000018130	-0.273181	0.5691762	0.2447532
ENSBTAG00000018131	-0.131014	0.5989321	0.2226224
ENSBTAG00000018133	NA	NA	NA
ENSBTAG00000018137	0.155412	0.5828677	0.23443
ENSBTAG00000018138	0.5508569	0.2068002	0.6844491
ENSBTAG00000018142	NA	NA	NA
ENSBTAG00000018143	NA	NA	NA
ENSBTAG00000018146	0.9827578	0.0340857	1.4674274
ENSBTAG00000018147	NA	NA	NA
ENSBTAG00000018151	0.0708037	0.8746712	0.0581552

ENSBTAG00000018152	0.3229946	0.3399733	0.4685552
ENSBTAG00000018153	-0.239824	0.3438668	0.4636097
ENSBTAG00000018155	-0.219666	0.4040324	0.3935839
ENSBTAG00000018156	NA	NA	NA
ENSBTAG00000018157	0.2484771	0.4961375	0.304398
ENSBTAG00000018159	0.3460442	0.4429726	0.3536231
ENSBTAG00000018160	NA	NA	NA
ENSBTAG00000018161	1.1175812	0.0251913	1.5987496
ENSBTAG00000018162	NA	NA	NA
ENSBTAG00000018164	NA	NA	NA
ENSBTAG00000018165	0.0944692	0.8194992	0.0864515
ENSBTAG00000018167	-1.493796	0.0033031	2.4810826
ENSBTAG00000018169	0.2636596	0.5087542	0.293492
ENSBTAG00000018170	NA	NA	NA
ENSBTAG00000018172	NA	NA	NA
ENSBTAG00000018175	0.2998165	0.4121629	0.3849311
ENSBTAG00000018176	0.4062002	0.2186128	0.6603243
ENSBTAG00000018178	-0.033095	0.9039087	0.0438755
ENSBTAG00000018179	NA	NA	NA
ENSBTAG00000018180	NA	NA	NA
ENSBTAG00000018181	-0.411361	0.1887588	0.7240928
ENSBTAG00000018182	NA	NA	NA
ENSBTAG00000018185	-0.288585	0.3786941	0.4217114
ENSBTAG00000018186	0.0448823	0.9102655	0.0408319
ENSBTAG00000018188	-0.16181	0.5517573	0.2582519
ENSBTAG00000018189	NA	NA	NA
ENSBTAG00000018192	0.1045294	0.8375286	0.0770003
ENSBTAG00000018193	NA	NA	NA
ENSBTAG00000018196	-0.076721	0.7593763	0.1195429
ENSBTAG00000018199	-0.197069	0.6512792	0.1862328
ENSBTAG00000018200	-0.2473	0.4925628	0.3075384
ENSBTAG00000018201	-0.254094	0.5567255	0.2543589
ENSBTAG00000018202	0.5169363	0.15673	0.8048479
ENSBTAG00000018203	NA	NA	NA
ENSBTAG00000018204	-1.069039	0.0982088	1.0078497
ENSBTAG00000018205	0.1221949	0.781458	0.1070943
ENSBTAG00000018206	-0.107221	0.8487972	0.0711961
ENSBTAG00000018207	0.1794022	0.4646445	0.3328792
ENSBTAG00000018213	0.4172913	0.2634508	0.5793004
ENSBTAG00000018214	-1.319158	1.68E-06	5.7738204
ENSBTAG00000018216	NA	NA	NA
ENSBTAG00000018220	NA	NA	NA
ENSBTAG00000018223	-0.092071	0.8592607	0.065875
ENSBTAG00000018225	0.3290065	0.3534954	0.4516163
ENSBTAG00000018227	0.0115212	0.9691304	0.0136178
ENSBTAG00000018229	-0.315653	0.2137366	0.6701211
ENSBTAG00000018232	NA	NA	NA
ENSBTAG00000018235	NA	NA	NA
ENSBTAG00000018236	0.1156143	0.7135984	0.1465461
ENSBTAG00000018237	NA	NA	NA
ENSBTAG00000018238	-0.033049	0.9240988	0.0342816
ENSBTAG00000018239	-0.069108	0.8457596	0.0727531
ENSBTAG00000018240	NA	NA	NA
ENSBTAG00000018244	NA	NA	NA
ENSBTAG00000018245	0.1579199	0.6141013	0.21176
ENSBTAG00000018246	NA	NA	NA
ENSBTAG00000018247	0.0986207	0.7413343	0.1299859
ENSBTAG00000018248	0.7957607	0.0041131	2.3858351

ENSBTAG00000018249	-0.018604	0.9539986	0.0204523
ENSBTAG00000018252	-0.681889	0.0369999	1.431799
ENSBTAG00000018253	-0.118654	0.675375	0.170455
ENSBTAG00000018254	0.1991051	0.4358885	0.3606246
ENSBTAG00000018255	0.1584986	0.583783	0.2337485
ENSBTAG00000018256	-0.159326	0.5672228	0.2462463
ENSBTAG00000018257	-0.223807	0.4755627	0.3227922
ENSBTAG00000018258	0.1055568	0.6858654	0.1637611
ENSBTAG00000018260	0.3446279	0.2095439	0.6787251
ENSBTAG00000018261	-0.180692	0.4750173	0.3232906
ENSBTAG00000018263	-0.44066	0.0846577	1.0723337
ENSBTAG00000018267	0.7629191	0.008532	2.0689512
ENSBTAG00000018268	NA	NA	NA
ENSBTAG00000018269	0.1425011	0.6759076	0.1701127
ENSBTAG00000018270	-0.189246	0.6053207	0.2180145
ENSBTAG00000018271	0.4757428	0.1263444	0.898444
ENSBTAG00000018272	0.0304211	0.9234229	0.0345993
ENSBTAG00000018274	1.5661988	0.060669	1.217033
ENSBTAG00000018277	NA	NA	NA
ENSBTAG00000018278	-0.20322	0.4513745	0.3454629
ENSBTAG00000018279	0.016851	0.9597124	0.0178589
ENSBTAG00000018280	NA	NA	NA
ENSBTAG00000018281	0.4113004	0.4345271	0.3619831
ENSBTAG00000018282	-0.630211	0.0487631	1.3119086
ENSBTAG00000018283	0.0322229	0.8963832	0.0475063
ENSBTAG00000018285	-0.221263	0.4508793	0.3459397
ENSBTAG00000018286	0.0998971	0.7068151	0.1506942
ENSBTAG00000018287	NA	NA	NA
ENSBTAG00000018289	NA	NA	NA
ENSBTAG00000018291	0.4302822	0.3796788	0.4205837
ENSBTAG00000018292	0.0879977	0.7527665	0.1233397
ENSBTAG00000018294	NA	NA	NA
ENSBTAG00000018295	-0.02115	0.9349521	0.0292106
ENSBTAG00000018297	NA	NA	NA
ENSBTAG00000018299	-0.098328	0.7894379	0.102682
ENSBTAG00000018300	-0.492629	0.1041336	0.9824092
ENSBTAG00000018303	NA	NA	NA
ENSBTAG00000018307	-0.17278	0.6523405	0.1855256
ENSBTAG00000018310	-0.457821	0.0818893	1.0867729
ENSBTAG00000018311	NA	NA	NA
ENSBTAG00000018312	0.5725395	0.2158642	0.6658194
ENSBTAG00000018313	-0.122984	0.6462679	0.1895874
ENSBTAG00000018314	0.2869547	0.4326954	0.3638177
ENSBTAG00000018315	0.4052234	0.2182119	0.6611216
ENSBTAG00000018316	NA	NA	NA
ENSBTAG00000018317	-0.132618	0.6808643	0.1669394
ENSBTAG00000018318	0.0392318	0.9145592	0.0387882
ENSBTAG00000018320	0.0124955	0.9618968	0.0168715
ENSBTAG00000018321	0.0539552	0.8677642	0.0615983
ENSBTAG00000018322	0.2374693	0.36152	0.4418677
ENSBTAG00000018323	NA	NA	NA
ENSBTAG00000018324	-0.069834	0.80437	0.0945441
ENSBTAG00000018326	NA	NA	NA
ENSBTAG00000018329	-0.042924	0.8949547	0.0481989
ENSBTAG00000018330	-0.365182	0.2947337	0.5305702
ENSBTAG00000018331	0.6547494	0.0105377	1.9772528
ENSBTAG00000018332	0.281334	0.3375686	0.471638
ENSBTAG00000018334	-0.352578	0.4701874	0.327729

ENSBTAG00000018339	0.0496025	0.8630999	0.0639389
ENSBTAG00000018342	NA	NA	NA
ENSBTAG00000018343	NA	NA	NA
ENSBTAG00000018345	NA	NA	NA
ENSBTAG00000018346	NA	NA	NA
ENSBTAG00000018347	0.5922804	0.1158835	0.9359786
ENSBTAG00000018348	0.0729119	0.8110938	0.0909289
ENSBTAG00000018349	-0.132646	0.8400795	0.0756796
ENSBTAG00000018352	-0.40915	0.2327752	0.6330632
ENSBTAG00000018356	NA	NA	NA
ENSBTAG00000018358	-0.081854	0.7587813	0.1198834
ENSBTAG00000018360	-0.28646	0.3029058	0.5186925
ENSBTAG00000018361	0.0687391	0.8031863	0.0951837
ENSBTAG00000018362	0.0297806	0.906291	0.0427323
ENSBTAG00000018363	0.0977766	0.7299157	0.1367273
ENSBTAG00000018364	0.0503969	0.877167	0.0569177
ENSBTAG00000018365	NA	NA	NA
ENSBTAG00000018366	NA	NA	NA
ENSBTAG00000018367	NA	NA	NA
ENSBTAG00000018369	0.1936533	0.4576687	0.3394488
ENSBTAG00000018372	-0.188869	0.5064819	0.2954361
ENSBTAG00000018373	0.4532557	0.0671756	1.1727887
ENSBTAG00000018374	0.3364132	0.2364098	0.6263345
ENSBTAG00000018375	NA	NA	NA
ENSBTAG00000018379	0.6266875	0.1583991	0.8002472
ENSBTAG00000018381	0.506693	0.1151806	0.9386207
ENSBTAG00000018382	-0.025245	0.9345866	0.0293804
ENSBTAG00000018383	0.2456104	0.6395661	0.1941146
ENSBTAG00000018385	-0.148989	0.5529683	0.2572998
ENSBTAG00000018386	0.2835345	0.3182389	0.4972467
ENSBTAG00000018387	0.1267766	0.6789203	0.1681812
ENSBTAG00000018390	NA	NA	NA
ENSBTAG00000018394	NA	NA	NA
ENSBTAG00000018395	-0.144033	0.6129422	0.2125805
ENSBTAG00000018397	NA	NA	NA
ENSBTAG00000018398	NA	NA	NA
ENSBTAG00000018399	NA	NA	NA
ENSBTAG00000018400	-0.393974	0.1942674	0.7116
ENSBTAG00000018401	0.2736441	0.3465773	0.4601998
ENSBTAG00000018402	0.2022057	0.5287933	0.276714
ENSBTAG00000018403	NA	NA	NA
ENSBTAG00000018404	-0.078892	0.8224152	0.0849089
ENSBTAG00000018405	0.0790293	0.7605808	0.1188546
ENSBTAG00000018408	-0.089012	0.7352228	0.1335811
ENSBTAG00000018410	0.9720334	0.0141039	1.8506603
ENSBTAG00000018413	0.2020127	0.6467152	0.1892869
ENSBTAG00000018415	-0.036888	0.9269435	0.0329468
ENSBTAG00000018416	NA	NA	NA
ENSBTAG00000018417	-0.016329	0.9517418	0.0214808
ENSBTAG00000018418	NA	NA	NA
ENSBTAG00000018419	0.2861015	0.4143717	0.3826099
ENSBTAG00000018420	-0.003847	0.9944471	0.0024183
ENSBTAG00000018421	0.0607232	0.8308512	0.0804768
ENSBTAG00000018422	0.3660602	0.4344205	0.3620897
ENSBTAG00000018423	-0.148045	0.5961312	0.2246581
ENSBTAG00000018424	-0.090011	0.7812873	0.1071892
ENSBTAG00000018425	-0.026384	0.9186034	0.036872
ENSBTAG00000018426	-0.482786	0.28865	0.5396285

ENSBTAG00000018427	0.2713011	0.3127811	0.5047594
ENSBTAG00000018430	-0.268549	0.5677754	0.2458234
ENSBTAG00000018431	0.2085712	0.6586294	0.1813589
ENSBTAG00000018432	NA	NA	NA
ENSBTAG00000018433	-0.23391	0.4531112	0.3437952
ENSBTAG00000018435	NA	NA	NA
ENSBTAG00000018436	0.0015374	0.9982183	0.0007745
ENSBTAG00000018437	-0.255719	0.2938018	0.5319455
ENSBTAG00000018438	-0.367856	0.1544445	0.8112277
ENSBTAG00000018440	NA	NA	NA
ENSBTAG00000018442	NA	NA	NA
ENSBTAG00000018445	NA	NA	NA
ENSBTAG00000018446	NA	NA	NA
ENSBTAG00000018447	-0.155574	0.535464	0.2712697
ENSBTAG00000018448	NA	NA	NA
ENSBTAG00000018449	-0.050012	0.8574999	0.0667659
ENSBTAG00000018451	0.0421716	0.9004395	0.0455455
ENSBTAG00000018452	-0.274597	0.3650118	0.4376931
ENSBTAG00000018453	-1.234308	0.0063362	2.1981735
ENSBTAG00000018454	-0.276486	0.3572544	0.4470224
ENSBTAG00000018455	-0.003205	0.9935214	0.0028228
ENSBTAG00000018456	0.0400616	0.9061986	0.0427766
ENSBTAG00000018460	-0.749374	0.157329	0.8031913
ENSBTAG00000018463	0.2214468	0.3908666	0.4079714
ENSBTAG00000018464	-0.217627	0.6193117	0.2080907
ENSBTAG00000018465	0.0702566	0.8706516	0.0601556
ENSBTAG00000018467	NA	NA	NA
ENSBTAG00000018469	-0.181817	0.4985876	0.3022586
ENSBTAG00000018471	0.4258518	0.1676526	0.7755896
ENSBTAG00000018474	0.7757762	0.0069571	2.1575704
ENSBTAG00000018478	0.0991052	0.6897009	0.1613392
ENSBTAG00000018479	-0.084575	0.7934198	0.100497
ENSBTAG00000018481	NA	NA	NA
ENSBTAG00000018482	0.0524885	0.8575739	0.0667284
ENSBTAG00000018483	-0.037552	0.8834606	0.0538128
ENSBTAG00000018487	NA	NA	NA
ENSBTAG00000018488	-0.411961	0.1607711	0.7937919
ENSBTAG00000018493	NA	NA	NA
ENSBTAG00000018495	NA	NA	NA
ENSBTAG00000018496	-0.125243	0.6161889	0.2102861
ENSBTAG00000018497	0.2557584	0.308382	0.510911
ENSBTAG00000018498	0.0233212	0.9473503	0.0234894
ENSBTAG00000018499	-0.831194	0.0287108	1.5419553
ENSBTAG00000018501	0.1320793	0.6538211	0.184541
ENSBTAG00000018502	0.5419039	0.2437069	0.6131322
ENSBTAG00000018506	-0.445454	0.3325621	0.4781272
ENSBTAG00000018508	NA	NA	NA
ENSBTAG00000018509	NA	NA	NA
ENSBTAG00000018513	-0.131326	0.6189695	0.2083308
ENSBTAG00000018517	-0.148298	0.5635476	0.2490694
ENSBTAG00000018518	NA	NA	NA
ENSBTAG00000018519	NA	NA	NA
ENSBTAG00000018520	NA	NA	NA
ENSBTAG00000018522	-0.2416	0.3481272	0.458262
ENSBTAG00000018523	0.4599359	0.2327508	0.6331088
ENSBTAG00000018524	NA	NA	NA
ENSBTAG00000018527	-0.206224	0.5775587	0.2384039
ENSBTAG00000018528	0.4308987	0.1070331	0.9704818

ENSBTAG00000018530	-0.118195	0.6415706	0.1927555
ENSBTAG00000018531	-0.611784	0.0915435	1.0383726
ENSBTAG00000018534	0.141411	0.6687154	0.1747587
ENSBTAG00000018535	0.068275	0.789582	0.1026028
ENSBTAG00000018538	-0.038541	0.8866676	0.0522392
ENSBTAG00000018540	1.1049546	0.0115489	1.9374604
ENSBTAG00000018542	-0.007601	0.9778689	0.0097194
ENSBTAG00000018543	NA	NA	NA
ENSBTAG00000018546	-0.264737	0.3188659	0.4963919
ENSBTAG00000018548	0.2855013	0.3487213	0.4575215
ENSBTAG00000018549	NA	NA	NA
ENSBTAG00000018554	NA	NA	NA
ENSBTAG00000018555	0.3399102	0.4244255	0.3721985
ENSBTAG00000018557	0.1357118	0.5853235	0.232604
ENSBTAG00000018559	-0.560121	0.0726819	1.1385738
ENSBTAG00000018560	NA	NA	NA
ENSBTAG00000018562	0.2126773	0.487051	0.3124256
ENSBTAG00000018563	0.0933581	0.870351	0.0603056
ENSBTAG00000018564	0.1959803	0.5381469	0.2690992
ENSBTAG00000018566	0.2891342	0.5459053	0.2628827
ENSBTAG00000018569	-0.22486	0.4077628	0.3895924
ENSBTAG00000018571	-0.618951	0.2397086	0.6203164
ENSBTAG00000018572	NA	NA	NA
ENSBTAG00000018575	NA	NA	NA
ENSBTAG00000018576	NA	NA	NA
ENSBTAG00000018577	0.193622	0.693932	0.1586831
ENSBTAG00000018578	-0.129201	0.7814103	0.1071209
ENSBTAG00000018579	0.1794792	0.6801904	0.1673695
ENSBTAG00000018580	NA	NA	NA
ENSBTAG00000018581	-0.010779	0.9654727	0.01526
ENSBTAG00000018585	NA	NA	NA
ENSBTAG00000018588	-0.11175	0.6912347	0.1603745
ENSBTAG00000018589	-0.080242	0.8106658	0.0911582
ENSBTAG00000018590	-0.011854	0.9671189	0.0145201
ENSBTAG00000018593	-0.144842	0.5700892	0.2440572
ENSBTAG00000018594	0.4107137	0.2875129	0.5413426
ENSBTAG00000018596	-0.2743	0.2916855	0.5350851
ENSBTAG00000018598	0.2664459	0.3177102	0.4979689
ENSBTAG00000018599	NA	NA	NA
ENSBTAG00000018600	-0.087237	0.8240325	0.0840557
ENSBTAG00000018603	-0.19498	0.5962832	0.2245474
ENSBTAG00000018604	NA	NA	NA
ENSBTAG00000018607	NA	NA	NA
ENSBTAG00000018613	0.0041259	0.9881067	0.0051962
ENSBTAG00000018616	-0.102931	0.6830432	0.1655518
ENSBTAG00000018622	-0.172405	0.4935962	0.3066282
ENSBTAG00000018625	-0.260067	0.5145802	0.2885469
ENSBTAG00000018628	-0.00812	0.9748629	0.0110564
ENSBTAG00000018629	-0.153659	0.5313752	0.2745987
ENSBTAG00000018631	-0.238556	0.3406399	0.4677045
ENSBTAG00000018633	-0.059555	0.8197327	0.0863278
ENSBTAG00000018634	0.5355805	0.2213521	0.6549163
ENSBTAG00000018636	0.1452841	0.713302	0.1467266
ENSBTAG00000018637	0.0851045	0.8279382	0.0820021
ENSBTAG00000018638	0.4604669	0.3192181	0.4959126
ENSBTAG00000018639	0.162905	0.5721067	0.2425229
ENSBTAG00000018642	0.6731449	0.1503901	0.8227809
ENSBTAG00000018643	0.1940941	0.6411177	0.1930622

ENSBTAG00000018644	-0.307595	0.249281	0.6033108
ENSBTAG00000018646	-0.08048	0.8089521	0.0920772
ENSBTAG00000018647	NA	NA	NA
ENSBTAG00000018650	NA	NA	NA
ENSBTAG00000018653	-0.325555	0.4631271	0.3342998
ENSBTAG00000018655	0.5177104	0.1880012	0.7258394
ENSBTAG00000018656	0.3480876	0.2134265	0.6707517
ENSBTAG00000018657	NA	NA	NA
ENSBTAG00000018658	0.122393	0.6613791	0.1795495
ENSBTAG00000018660	0.3007678	0.2706651	0.5675677
ENSBTAG00000018661	-0.003336	0.9967283	0.0014232
ENSBTAG00000018663	-0.135757	0.6201985	0.2074693
ENSBTAG00000018667	0.3712466	0.2774277	0.5568502
ENSBTAG00000018669	NA	NA	NA
ENSBTAG00000018671	-0.274636	0.412936	0.3841172
ENSBTAG00000018673	NA	NA	NA
ENSBTAG00000018674	-0.130768	0.6115611	0.2135602
ENSBTAG00000018680	-0.019032	0.9445159	0.0247907
ENSBTAG00000018681	NA	NA	NA
ENSBTAG00000018682	0.4051341	0.3032223	0.5182389
ENSBTAG00000018688	0.0919186	0.797008	0.0985373
ENSBTAG00000018689	-0.319792	0.2629407	0.5801421
ENSBTAG00000018690	-0.562404	0.0358914	1.4450091
ENSBTAG00000018691	-0.420633	0.4254712	0.3711299
ENSBTAG00000018694	0.1517971	0.6305555	0.2002767
ENSBTAG00000018697	-0.136877	0.6450398	0.1904135
ENSBTAG00000018699	0.2752456	0.2645231	0.5775363
ENSBTAG00000018703	NA	NA	NA
ENSBTAG00000018704	0.1362623	0.7034615	0.1527597
ENSBTAG00000018705	0.3568837	0.3988948	0.3991417
ENSBTAG00000018706	0.0648647	0.8637142	0.0636299
ENSBTAG00000018707	0.1087709	0.6730209	0.1719714
ENSBTAG00000018708	0.2357536	0.5314928	0.2745026
ENSBTAG00000018709	NA	NA	NA
ENSBTAG00000018710	0.2879568	0.3870942	0.4121833
ENSBTAG00000018711	0.2912636	0.326013	0.4867651
ENSBTAG00000018713	NA	NA	NA
ENSBTAG00000018715	-0.636378	0.0515154	1.2880625
ENSBTAG00000018717	-0.034335	0.8964696	0.0474644
ENSBTAG00000018718	0.0140737	0.9635776	0.0161133
ENSBTAG00000018722	0.0646568	0.8035338	0.0949958
ENSBTAG00000018723	0.3902835	0.2132075	0.6711975
ENSBTAG00000018724	NA	NA	NA
ENSBTAG00000018725	0.2116455	0.4472402	0.3494591
ENSBTAG00000018726	NA	NA	NA
ENSBTAG00000018727	-0.2414	0.452926	0.3439727
ENSBTAG00000018729	0.3268144	0.2330117	0.6326223
ENSBTAG00000018730	0.270419	0.3096739	0.5090953
ENSBTAG00000018731	NA	NA	NA
ENSBTAG00000018732	-0.466648	0.148863	0.8272133
ENSBTAG00000018735	-0.26577	0.3390778	0.4697006
ENSBTAG00000018737	-0.273204	0.4262245	0.3703616
ENSBTAG00000018739	0.2158944	0.4922296	0.3078323
ENSBTAG00000018742	-0.368366	0.1960571	0.7076175
ENSBTAG00000018743	-0.380401	0.1350287	0.869574
ENSBTAG00000018744	0.1672266	0.5725473	0.2421886
ENSBTAG00000018745	-0.456985	0.1149583	0.9394596
ENSBTAG00000018746	-0.315787	0.4919841	0.3080489

ENSBTAG00000018747	-0.739829	0.1220826	0.9133463
ENSBTAG00000018749	0.1725712	0.6672957	0.1756817
ENSBTAG00000018752	-0.077175	0.7921092	0.101215
ENSBTAG00000018753	NA	NA	NA
ENSBTAG00000018761	0.221353	0.5245624	0.2802029
ENSBTAG00000018765	NA	NA	NA
ENSBTAG00000018767	NA	NA	NA
ENSBTAG00000018768	-0.060599	0.8108893	0.0910384
ENSBTAG00000018770	-0.07672	0.8431742	0.0740827
ENSBTAG00000018771	3.836461	1.01E-08	7.9972639
ENSBTAG00000018772	0.452907	0.2794635	0.5536749
ENSBTAG00000018773	-0.402786	0.3613304	0.4420955
ENSBTAG00000018774	NA	NA	NA
ENSBTAG00000018775	-1.21329	0.0003861	3.4133216
ENSBTAG00000018777	0.228399	0.6070159	0.2167999
ENSBTAG00000018778	NA	NA	NA
ENSBTAG00000018783	-0.139192	0.5995279	0.2221906
ENSBTAG00000018784	-0.280277	0.5204704	0.283604
ENSBTAG00000018785	NA	NA	NA
ENSBTAG00000018790	-0.371704	0.1588155	0.7991072
ENSBTAG00000018792	NA	NA	NA
ENSBTAG00000018795	-0.11788	0.6485518	0.1880553
ENSBTAG00000018796	0.0647059	0.8078745	0.0926561
ENSBTAG00000018799	NA	NA	NA
ENSBTAG00000018800	0.0273841	0.9125963	0.0397213
ENSBTAG00000018801	-0.027573	0.913391	0.0393433
ENSBTAG00000018802	0.1016097	0.6938361	0.1587431
ENSBTAG00000018803	0.0617022	0.8182032	0.0871388
ENSBTAG00000018804	0.2180511	0.6154392	0.2108149
ENSBTAG00000018805	0.168724	0.7197855	0.1427969
ENSBTAG00000018806	NA	NA	NA
ENSBTAG00000018807	0.8330588	0.0946381	1.0239338
ENSBTAG00000018808	NA	NA	NA
ENSBTAG00000018809	-0.381064	0.2599837	0.585054
ENSBTAG00000018810	0.3983599	0.3316342	0.4793406
ENSBTAG00000018811	NA	NA	NA
ENSBTAG00000018812	-0.043601	0.8611004	0.0649462
ENSBTAG00000018813	0.004502	0.9864505	0.0059247
ENSBTAG00000018819	NA	NA	NA
ENSBTAG00000018823	0.2411088	0.4449612	0.3516779
ENSBTAG00000018824	0.0057757	0.981892	0.0079363
ENSBTAG00000018825	0.3764528	0.4171611	0.3796962
ENSBTAG00000018828	0.3376756	0.3836742	0.4160374
ENSBTAG00000018829	-0.018622	0.9482214	0.0230902
ENSBTAG00000018833	NA	NA	NA
ENSBTAG00000018834	0.1475604	0.6219268	0.2062607
ENSBTAG00000018837	0.2420733	0.5219189	0.282397
ENSBTAG00000018838	NA	NA	NA
ENSBTAG00000018841	0.5488509	0.1505983	0.8221799
ENSBTAG00000018843	NA	NA	NA
ENSBTAG00000018845	NA	NA	NA
ENSBTAG00000018846	-0.245208	0.3803732	0.4197901
ENSBTAG00000018847	0.0873267	0.7510752	0.1243166
ENSBTAG00000018848	-0.011794	0.9646723	0.0156202
ENSBTAG00000018851	0.0063169	0.9809215	0.0083658
ENSBTAG00000018852	-0.472438	0.1080056	0.9665537
ENSBTAG00000018854	-0.242672	0.3322785	0.4784978
ENSBTAG00000018855	-0.041595	0.90473	0.043481

ENSBTAG00000018857	-0.128787	0.6966039	0.1570141
ENSBTAG00000018859	0.0534232	0.8877984	0.0516857
ENSBTAG00000018861	NA	NA	NA
ENSBTAG00000018862	0.596822	0.1333811	0.8749059
ENSBTAG00000018863	-0.548709	0.2526605	0.5974627
ENSBTAG00000018864	-0.195842	0.6788452	0.1682292
ENSBTAG00000018868	-0.158337	0.5621496	0.2501481
ENSBTAG00000018869	NA	NA	NA
ENSBTAG00000018870	NA	NA	NA
ENSBTAG00000018872	NA	NA	NA
ENSBTAG00000018873	0.2927549	0.4493068	0.347457
ENSBTAG00000018877	0.2705983	0.5361061	0.2707492
ENSBTAG00000018879	-0.130689	0.6018127	0.2205387
ENSBTAG00000018880	NA	NA	NA
ENSBTAG00000018881	NA	NA	NA
ENSBTAG00000018883	0.1988281	0.5480198	0.2612037
ENSBTAG00000018884	-0.033054	0.9045013	0.0435908
ENSBTAG00000018887	0.0301957	0.9024559	0.044574
ENSBTAG00000018888	0.4369122	0.2645876	0.5774305
ENSBTAG00000018889	0.0994063	0.7145178	0.145987
ENSBTAG00000018893	0.1572777	0.5562101	0.2547611
ENSBTAG00000018894	-0.426458	0.2365641	0.6260512
ENSBTAG00000018897	0.2092898	0.4291912	0.3673491
ENSBTAG00000018898	0.0939509	0.7862291	0.1044509
ENSBTAG00000018900	NA	NA	NA
ENSBTAG00000018901	0.1255106	0.6973843	0.1565278
ENSBTAG00000018902	0.0401562	0.9134179	0.0393305
ENSBTAG00000018903	NA	NA	NA
ENSBTAG00000018904	NA	NA	NA
ENSBTAG00000018905	0.0092795	0.9727251	0.0120099
ENSBTAG00000018906	NA	NA	NA
ENSBTAG00000018908	0.2797064	0.4278879	0.36867
ENSBTAG00000018909	0.1952514	0.5393315	0.2681442
ENSBTAG00000018910	0.1904472	0.5942549	0.2260272
ENSBTAG00000018912	0.1971749	0.5540722	0.2564336
ENSBTAG00000018913	0.0425394	0.9068142	0.0424817
ENSBTAG00000018914	NA	NA	NA
ENSBTAG00000018915	-0.000234	0.9997153	0.0001237
ENSBTAG00000018917	NA	NA	NA
ENSBTAG00000018918	0.0769259	0.7910053	0.1018206
ENSBTAG00000018919	NA	NA	NA
ENSBTAG00000018920	-0.425939	0.219137	0.6592843
ENSBTAG00000018921	0.5469755	0.0786973	1.10404
ENSBTAG00000018922	0.2163009	0.4905991	0.3092732
ENSBTAG00000018924	0.1890135	0.4927063	0.3074119
ENSBTAG00000018925	0.0856412	0.7766698	0.1097636
ENSBTAG00000018926	NA	NA	NA
ENSBTAG00000018928	0.3419152	0.1968284	0.7059123
ENSBTAG00000018935	-0.180988	0.61474	0.2113085
ENSBTAG00000018936	-0.191415	0.6244366	0.2045116
ENSBTAG00000018937	0.8969898	0.0099235	2.0033336
ENSBTAG00000018938	0.1793003	0.5248611	0.2799556
ENSBTAG00000018940	NA	NA	NA
ENSBTAG00000018941	0.3822365	0.534109	0.2723701
ENSBTAG00000018942	-0.31128	0.28857	0.5397489
ENSBTAG00000018945	-0.132909	0.6061251	0.2174377
ENSBTAG00000018946	NA	NA	NA
ENSBTAG00000018948	NA	NA	NA

ENSBTAG00000018951	0.1539197	0.6784997	0.1684504
ENSBTAG00000018954	-0.123844	0.630273	0.2004713
ENSBTAG00000018955	-0.049559	0.9533349	0.0207545
ENSBTAG00000018959	-0.066692	0.8206151	0.0858605
ENSBTAG00000018960	NA	NA	NA
ENSBTAG00000018965	NA	NA	NA
ENSBTAG00000018966	0.2046278	0.5995121	0.2222021
ENSBTAG00000018967	0.1596357	0.5545419	0.2560657
ENSBTAG00000018969	0.5022428	0.1383214	0.8591107
ENSBTAG00000018970	0.3637298	0.2571816	0.58976
ENSBTAG00000018971	-0.272817	0.5216724	0.2826021
ENSBTAG00000018972	-0.200549	0.4849687	0.3142863
ENSBTAG00000018973	-0.029856	0.9049593	0.043371
ENSBTAG00000018975	-0.372398	0.3436084	0.4639362
ENSBTAG00000018978	0.2587978	0.3510595	0.4546193
ENSBTAG00000018979	-0.081695	0.7435572	0.1286856
ENSBTAG00000018981	NA	NA	NA
ENSBTAG00000018984	0.5057198	0.2849097	0.5452928
ENSBTAG00000018986	-0.200234	0.490595	0.3092769
ENSBTAG00000018987	-0.196773	0.5231651	0.2813613
ENSBTAG00000018991	0.1048611	0.8075331	0.0928396
ENSBTAG00000018993	-0.273015	0.541465	0.2664296
ENSBTAG00000018994	0.9796855	0.0451957	1.3449029
ENSBTAG00000018996	-0.055968	0.865634	0.0626657
ENSBTAG00000018999	0.2567155	0.3303908	0.4809721
ENSBTAG00000019001	0.4095162	0.2813628	0.5507333
ENSBTAG00000019002	-0.560404	0.0866543	1.0622098
ENSBTAG00000019006	0.5238066	0.1341344	0.8724599
ENSBTAG00000019007	NA	NA	NA
ENSBTAG00000019010	0.1231942	0.6693656	0.1743366
ENSBTAG00000019011	-0.352507	0.1781906	0.7491153
ENSBTAG00000019012	NA	NA	NA
ENSBTAG00000019013	0.1397721	0.617346	0.2094714
ENSBTAG00000019014	NA	NA	NA
ENSBTAG00000019015	0.5375726	0.0899659	1.0459223
ENSBTAG00000019017	0.1444411	0.655919	0.1831498
ENSBTAG00000019018	0.1332815	0.7642538	0.1167624
ENSBTAG00000019020	0.0174503	0.9445325	0.0247831
ENSBTAG00000019021	NA	NA	NA
ENSBTAG00000019023	0.0440651	0.9000904	0.0457139
ENSBTAG00000019024	-0.159585	0.6651953	0.1770508
ENSBTAG00000019025	0.1220165	0.6348218	0.1973482
ENSBTAG00000019026	-0.10953	0.7976024	0.0982135
ENSBTAG00000019027	0.4026786	0.1549323	0.8098581
ENSBTAG00000019028	NA	NA	NA
ENSBTAG00000019029	0.0898212	0.7349416	0.1337472
ENSBTAG00000019031	NA	NA	NA
ENSBTAG00000019033	-0.330994	0.4854281	0.3138751
ENSBTAG00000019036	0.1308168	0.6750865	0.1706406
ENSBTAG00000019037	-1.073252	0.0088484	2.0531337
ENSBTAG00000019039	NA	NA	NA
ENSBTAG00000019040	0.4856591	0.1131275	0.9464318
ENSBTAG00000019043	-0.198523	0.4426377	0.3539516
ENSBTAG00000019044	0.8985748	0.0070166	2.1538727
ENSBTAG00000019045	-0.100651	0.7385044	0.1316469
ENSBTAG00000019049	NA	NA	NA
ENSBTAG00000019051	0.3386325	0.3198709	0.4950253
ENSBTAG00000019052	-0.6275	0.0336847	1.4725678

ENSBTAG00000019053	NA	NA	NA
ENSBTAG00000019054	0.379782	0.309047	0.5099754
ENSBTAG00000019058	NA	NA	NA
ENSBTAG00000019059	-0.779499	0.0941224	1.0263071
ENSBTAG00000019060	NA	NA	NA
ENSBTAG00000019061	0.1344455	0.7667082	0.1153699
ENSBTAG00000019062	-0.137328	0.6899802	0.1611634
ENSBTAG00000019065	-0.336138	0.298929	0.5244319
ENSBTAG00000019066	NA	NA	NA
ENSBTAG00000019067	0.0722573	0.9171642	0.0375529
ENSBTAG00000019069	0.0810693	0.7466668	0.1268732
ENSBTAG00000019070	-0.667915	0.0224807	1.6481893
ENSBTAG00000019071	-0.224239	0.3840877	0.4155696
ENSBTAG00000019072	NA	NA	NA
ENSBTAG00000019073	-0.119002	0.6682376	0.1750691
ENSBTAG00000019075	-0.297405	0.2992083	0.5240263
ENSBTAG00000019076	NA	NA	NA
ENSBTAG00000019077	-0.153235	0.5400076	0.2676001
ENSBTAG00000019079	NA	NA	NA
ENSBTAG00000019081	0.1551254	0.7560737	0.1214359
ENSBTAG00000019082	-0.153053	0.6546934	0.1839621
ENSBTAG00000019085	-0.118315	0.62991	0.2007215
ENSBTAG00000019086	0.2411132	0.555426	0.2553738
ENSBTAG00000019087	NA	NA	NA
ENSBTAG00000019090	-0.204559	0.5474478	0.2616573
ENSBTAG00000019091	-0.07271	0.7778069	0.1091282
ENSBTAG00000019092	NA	NA	NA
ENSBTAG00000019093	-0.073254	0.8143576	0.0891849
ENSBTAG00000019094	0.1371488	0.7719241	0.1124254
ENSBTAG00000019095	NA	NA	NA
ENSBTAG00000019096	0.032997	0.9012641	0.0451479
ENSBTAG00000019097	NA	NA	NA
ENSBTAG00000019099	1.0583891	0.0054757	2.2615589
ENSBTAG00000019104	0.2462759	0.5679344	0.2457019
ENSBTAG00000019105	-0.031198	0.9015348	0.0450175
ENSBTAG00000019106	0.2015724	0.5604245	0.2514829
ENSBTAG00000019107	0.0057697	0.9872587	0.005569
ENSBTAG00000019110	-0.10388	0.6876429	0.1626371
ENSBTAG00000019115	NA	NA	NA
ENSBTAG00000019116	-0.32406	0.1972445	0.704995
ENSBTAG00000019119	-0.017558	0.9485914	0.0229208
ENSBTAG00000019120	-0.581534	0.2463284	0.6084855
ENSBTAG00000019121	0.1401897	0.7410366	0.1301603
ENSBTAG00000019122	NA	NA	NA
ENSBTAG00000019123	NA	NA	NA
ENSBTAG00000019124	0.0613267	0.8261597	0.082936
ENSBTAG00000019125	NA	NA	NA
ENSBTAG00000019129	0.2217471	0.4944519	0.3058759
ENSBTAG00000019130	-0.318952	0.2814299	0.5506298
ENSBTAG00000019132	NA	NA	NA
ENSBTAG00000019133	-0.221852	0.4211666	0.3755461
ENSBTAG00000019134	NA	NA	NA
ENSBTAG00000019136	-0.157994	0.5252991	0.2795934
ENSBTAG00000019137	0.6668883	0.1455849	0.8368836
ENSBTAG00000019138	-0.50705	0.2905143	0.5368325
ENSBTAG00000019139	0.0240967	0.9235864	0.0345225
ENSBTAG00000019140	NA	NA	NA
ENSBTAG00000019143	NA	NA	NA

ENSBTAG00000019145	NA	NA	NA
ENSBTAG00000019146	1.2512752	0.0027687	2.5577244
ENSBTAG00000019147	-0.023073	0.9270386	0.0329022
ENSBTAG00000019150	0.0587257	0.8607878	0.0651039
ENSBTAG00000019152	NA	NA	NA
ENSBTAG00000019153	0.0291474	0.9249484	0.0338825
ENSBTAG00000019155	0.2211168	0.4920164	0.3080204
ENSBTAG00000019156	-0.049101	0.8468248	0.0722064
ENSBTAG00000019157	NA	NA	NA
ENSBTAG00000019158	NA	NA	NA
ENSBTAG00000019159	NA	NA	NA
ENSBTAG00000019160	-0.126619	0.6288636	0.2014435
ENSBTAG00000019161	0.1176102	0.688232	0.1622651
ENSBTAG00000019162	0.4320982	0.2074017	0.6831877
ENSBTAG00000019163	-0.345748	0.4752746	0.3230554
ENSBTAG00000019164	-0.416377	0.1435867	0.8428857
ENSBTAG00000019166	0.0527035	0.8705662	0.0601982
ENSBTAG00000019167	0.137984	0.5918871	0.2277611
ENSBTAG00000019174	NA	NA	NA
ENSBTAG00000019175	-0.53207	0.2110291	0.6756577
ENSBTAG00000019177	-0.268269	0.2945747	0.5308046
ENSBTAG00000019179	NA	NA	NA
ENSBTAG00000019180	0.3221281	0.2518645	0.598833
ENSBTAG00000019181	NA	NA	NA
ENSBTAG00000019182	0.23095	0.4722701	0.3258096
ENSBTAG00000019183	-0.221278	0.5068872	0.2950886
ENSBTAG00000019184	0.344378	0.2370385	0.625181
ENSBTAG00000019187	-0.358668	0.2338746	0.6310169
ENSBTAG00000019188	0.0783821	0.7698619	0.1135871
ENSBTAG00000019192	NA	NA	NA
ENSBTAG00000019193	-0.300262	0.2522175	0.5982248
ENSBTAG00000019194	-0.038704	0.9092903	0.0412974
ENSBTAG00000019197	0.018347	0.9427361	0.0256099
ENSBTAG00000019198	0.0151923	0.9796034	0.0089497
ENSBTAG00000019202	0.283611	0.3390452	0.4697424
ENSBTAG00000019203	-0.328588	0.3137384	0.5034323
ENSBTAG00000019204	0.0195731	0.9510729	0.0217862
ENSBTAG00000019208	-0.067411	0.8860093	0.0525617
ENSBTAG00000019209	0.5996915	0.1271116	0.895815
ENSBTAG00000019210	0.1250736	0.6251935	0.2039855
ENSBTAG00000019211	0.2902895	0.3710648	0.4305503
ENSBTAG00000019212	NA	NA	NA
ENSBTAG00000019213	-0.275101	0.4894557	0.3102867
ENSBTAG00000019214	-0.19624	0.4324823	0.3640316
ENSBTAG00000019215	-0.001863	0.9962352	0.0016381
ENSBTAG00000019216	0.2003817	0.4193105	0.3774643
ENSBTAG00000019217	-0.158515	0.5527833	0.2574451
ENSBTAG00000019218	0.0347297	0.8949314	0.0482102
ENSBTAG00000019219	0.2154318	0.466425	0.3312182
ENSBTAG00000019220	0.2233416	0.4230624	0.3735955
ENSBTAG00000019221	NA	NA	NA
ENSBTAG00000019225	0.783734	0.0047543	2.3229172
ENSBTAG00000019227	NA	NA	NA
ENSBTAG00000019229	NA	NA	NA
ENSBTAG00000019230	0.8273037	0.1576673	0.8022583
ENSBTAG00000019231	-0.045675	0.8903376	0.0504453
ENSBTAG00000019232	-0.128386	0.6238835	0.2048965
ENSBTAG00000019234	0.4306006	0.2059268	0.6862871

ENSBTAG00000019235	0.1392777	0.7518358	0.123877
ENSBTAG00000019237	0.6019913	0.1341043	0.8725573
ENSBTAG00000019238	0.2556449	0.3866201	0.4127156
ENSBTAG00000019241	NA	NA	NA
ENSBTAG00000019242	0.3886877	0.1710509	0.7668748
ENSBTAG00000019244	0.5992486	0.1210947	0.9168748
ENSBTAG00000019246	-0.197025	0.6341166	0.1978309
ENSBTAG00000019249	NA	NA	NA
ENSBTAG00000019250	NA	NA	NA
ENSBTAG00000019251	-0.306867	0.3558175	0.4487727
ENSBTAG00000019252	NA	NA	NA
ENSBTAG00000019253	0.098154	0.7137547	0.146451
ENSBTAG00000019255	0.0401239	0.8825205	0.0542752
ENSBTAG00000019256	0.4350273	0.1835293	0.7362946
ENSBTAG00000019260	NA	NA	NA
ENSBTAG00000019262	-0.411295	0.4216642	0.3750332
ENSBTAG00000019264	-0.148936	0.5526563	0.2575448
ENSBTAG00000019265	0.1106378	0.7759283	0.1101784
ENSBTAG00000019266	NA	NA	NA
ENSBTAG00000019267	0.5123038	0.0604308	1.218742
ENSBTAG00000019268	NA	NA	NA
ENSBTAG00000019269	0.3207129	0.2229677	0.6517581
ENSBTAG00000019271	0.1049493	0.6750641	0.170655
ENSBTAG00000019272	-0.066986	0.8978157	0.0468128
ENSBTAG00000019274	-0.401428	0.1419117	0.8479819
ENSBTAG00000019275	0.1479503	0.7419635	0.1296175
ENSBTAG00000019277	NA	NA	NA
ENSBTAG00000019278	NA	NA	NA
ENSBTAG00000019279	-0.115691	0.7095998	0.1489865
ENSBTAG00000019280	NA	NA	NA
ENSBTAG00000019281	0.5022564	0.1370465	0.863132
ENSBTAG00000019282	NA	NA	NA
ENSBTAG00000019283	-0.332199	0.4597293	0.3374979
ENSBTAG00000019285	-0.355339	0.1548877	0.809983
ENSBTAG00000019287	NA	NA	NA
ENSBTAG00000019289	-1.213062	0.0103502	1.9850506
ENSBTAG00000019290	0.0890511	0.7438734	0.128501
ENSBTAG00000019291	NA	NA	NA
ENSBTAG00000019293	NA	NA	NA
ENSBTAG00000019294	-0.201308	0.4437595	0.3528523
ENSBTAG00000019295	0.0781654	0.7981785	0.0979
ENSBTAG00000019297	-0.201816	0.5789265	0.2373765
ENSBTAG00000019298	0.1134829	0.6485188	0.1880774
ENSBTAG00000019299	-0.072989	0.7997572	0.0970419
ENSBTAG00000019300	-0.329359	0.2657868	0.5754667
ENSBTAG00000019302	0.8595232	0.0097413	2.0113852
ENSBTAG00000019303	0.2379873	0.4217836	0.3749103
ENSBTAG00000019304	-0.270581	0.3493545	0.4567337
ENSBTAG00000019305	0.5333505	0.2840336	0.5466303
ENSBTAG00000019309	0.1341662	0.749403	0.1252846
ENSBTAG00000019310	0.5929642	0.0556521	1.2545187
ENSBTAG00000019312	-0.536803	0.0695758	1.1575416
ENSBTAG00000019313	-0.344917	0.3410825	0.4671406
ENSBTAG00000019314	-0.212981	0.4407583	0.3557995
ENSBTAG00000019315	0.4681072	0.1920662	0.7165491
ENSBTAG00000019317	-0.257599	0.4469283	0.3497621
ENSBTAG00000019321	0.4248372	0.2787892	0.554724
ENSBTAG00000019322	NA	NA	NA

ENSBTAG00000019325	0.03395	0.9127744	0.0396366
ENSBTAG00000019327	0.2682087	0.3870905	0.4121874
ENSBTAG00000019329	0.5990288	0.2132504	0.6711101
ENSBTAG00000019330	NA	NA	NA
ENSBTAG00000019331	NA	NA	NA
ENSBTAG00000019332	NA	NA	NA
ENSBTAG00000019334	0.1765447	0.5922281	0.227511
ENSBTAG00000019336	0.0490245	0.88052	0.0552608
ENSBTAG00000019337	NA	NA	NA
ENSBTAG00000019338	-0.298237	0.3273068	0.485045
ENSBTAG00000019339	0.3813233	0.174522	0.7581498
ENSBTAG00000019340	0.717328	0.3961239	0.402169
ENSBTAG00000019341	-0.471387	0.157051	0.8039594
ENSBTAG00000019342	0.453743	0.3105631	0.5078502
ENSBTAG00000019343	NA	NA	NA
ENSBTAG00000019345	0.0724237	0.8215777	0.0853513
ENSBTAG00000019347	-0.332661	0.3546959	0.4501438
ENSBTAG00000019348	NA	NA	NA
ENSBTAG00000019349	NA	NA	NA
ENSBTAG00000019350	NA	NA	NA
ENSBTAG00000019353	-0.012442	0.9704521	0.0130259
ENSBTAG00000019354	NA	NA	NA
ENSBTAG00000019357	0.4213658	0.1996388	0.6997551
ENSBTAG00000019358	0.242113	0.4197758	0.3769826
ENSBTAG00000019359	-0.327414	0.3649367	0.4377824
ENSBTAG00000019362	0.2094315	0.5028851	0.2985313
ENSBTAG00000019365	0.2948658	0.3668267	0.435539
ENSBTAG00000019366	-0.169008	0.5125822	0.2902365
ENSBTAG00000019368	0.0776568	0.7571971	0.1207911
ENSBTAG00000019369	0.4140603	0.1946916	0.7106527
ENSBTAG00000019370	-0.3057	0.2993758	0.5237833
ENSBTAG00000019371	NA	NA	NA
ENSBTAG00000019373	0.1651996	0.6824068	0.1659567
ENSBTAG00000019375	0.6925767	0.1097937	0.9594228
ENSBTAG00000019376	-0.008301	0.9758377	0.0106224
ENSBTAG00000019378	0.7179393	0.1555254	0.8081988
ENSBTAG00000019379	0.2966568	0.5984751	0.2229539
ENSBTAG00000019382	0.3511507	0.2538492	0.5954242
ENSBTAG00000019385	0.0003866	1	0
ENSBTAG00000019386	-0.316193	0.2653767	0.5761373
ENSBTAG00000019387	-0.004137	0.9911082	0.0038789
ENSBTAG00000019388	0.0760451	0.7775041	0.1092973
ENSBTAG00000019390	-0.871577	0.2989574	0.5243907
ENSBTAG00000019394	-0.714458	0.0133177	1.8755717
ENSBTAG00000019399	0.6057678	0.1062642	0.973613
ENSBTAG00000019401	NA	NA	NA
ENSBTAG00000019402	NA	NA	NA
ENSBTAG00000019403	-0.276284	0.4851221	0.3141489
ENSBTAG00000019404	-0.156327	0.6706938	0.1734757
ENSBTAG00000019406	NA	NA	NA
ENSBTAG00000019409	1.2447885	0.0002676	3.5725772
ENSBTAG00000019410	-0.212832	0.4238052	0.3728338
ENSBTAG00000019412	-0.048365	0.8574514	0.0667905
ENSBTAG00000019414	-0.159413	0.5564728	0.2545561
ENSBTAG00000019416	NA	NA	NA
ENSBTAG00000019417	0.1679834	0.6728712	0.1720681
ENSBTAG00000019418	-0.488674	0.1971825	0.7051316
ENSBTAG00000019419	-0.095423	0.7147635	0.1458376

ENSBTAG00000019421	0.1771593	0.6061378	0.2174287
ENSBTAG00000019423	NA	NA	NA
ENSBTAG00000019427	-0.254718	0.3481656	0.4582141
ENSBTAG00000019428	0.036029	0.9376592	0.027955
ENSBTAG00000019429	NA	NA	NA
ENSBTAG00000019432	0.7623294	0.0494808	1.3055629
ENSBTAG00000019434	-0.356672	0.1914797	0.7178772
ENSBTAG00000019436	-0.231449	0.3612448	0.4421984
ENSBTAG00000019437	0.9070553	0.0475657	1.322706
ENSBTAG00000019440	NA	NA	NA
ENSBTAG00000019441	0.0224128	0.9437635	0.0251368
ENSBTAG00000019443	0.0653644	0.7879438	0.1035047
ENSBTAG00000019445	NA	NA	NA
ENSBTAG00000019446	0.0889571	0.7279992	0.1378691
ENSBTAG00000019447	NA	NA	NA
ENSBTAG00000019448	NA	NA	NA
ENSBTAG00000019451	0.0275371	0.952178	0.0212818
ENSBTAG00000019453	NA	NA	NA
ENSBTAG00000019454	0.089893	0.7979765	0.0980099
ENSBTAG00000019455	NA	NA	NA
ENSBTAG00000019456	0.5705646	0.2011154	0.6965546
ENSBTAG00000019457	-0.354962	0.2050228	0.6881978
ENSBTAG00000019458	-0.07447	0.7957277	0.0992355
ENSBTAG00000019459	-0.086357	0.8109658	0.0909974
ENSBTAG00000019460	NA	NA	NA
ENSBTAG00000019461	-0.026599	0.9510114	0.0218143
ENSBTAG00000019462	0.4051747	0.3846212	0.4149668
ENSBTAG00000019463	0.141117	0.6568644	0.1825243
ENSBTAG00000019465	0.0292327	0.9305822	0.0312453
ENSBTAG00000019467	NA	NA	NA
ENSBTAG00000019470	-0.145164	0.6360182	0.1965305
ENSBTAG00000019471	-0.015284	0.9605307	0.0174888
ENSBTAG00000019472	-0.155654	0.5927045	0.2271618
ENSBTAG00000019473	NA	NA	NA
ENSBTAG00000019474	-0.19101	0.4513054	0.3455295
ENSBTAG00000019478	-0.171449	0.7290644	0.1372341
ENSBTAG00000019479	0.4125499	0.2754431	0.5599681
ENSBTAG00000019480	0.2454958	0.4862983	0.3130972
ENSBTAG00000019486	0.4617238	0.2820461	0.54968
ENSBTAG00000019488	-0.062365	0.8932641	0.0490201
ENSBTAG00000019490	NA	NA	NA
ENSBTAG00000019492	-0.181098	0.5954828	0.2251308
ENSBTAG00000019494	0.0549927	0.8246829	0.083713
ENSBTAG00000019495	0.4426778	0.221923	0.6537978
ENSBTAG00000019496	NA	NA	NA
ENSBTAG00000019497	NA	NA	NA
ENSBTAG00000019498	-0.201951	0.5682798	0.2454378
ENSBTAG00000019500	0.0077279	0.976594	0.010286
ENSBTAG00000019501	-0.41909	0.1123879	0.9492805
ENSBTAG00000019502	-0.061656	0.8283352	0.0817939
ENSBTAG00000019504	NA	NA	NA
ENSBTAG00000019506	NA	NA	NA
ENSBTAG00000019508	-0.125234	0.6349113	0.1972869
ENSBTAG00000019509	0.2922035	0.497195	0.3034733
ENSBTAG00000019510	0.0510489	0.8397834	0.0758327
ENSBTAG00000019511	-0.095155	0.7235975	0.1405029
ENSBTAG00000019512	0.1517563	0.7811911	0.1072427
ENSBTAG00000019513	-0.151537	0.705299	0.1516267

ENSBTAG00000019514	-0.046063	0.8641934	0.0633891
ENSBTAG00000019515	0.025078	0.9256434	0.0335563
ENSBTAG00000019516	-0.17066	0.7159764	0.1451013
ENSBTAG00000019517	0.3350162	0.1865162	0.7292835
ENSBTAG00000019518	0.0426235	0.9101232	0.0408998
ENSBTAG00000019519	NA	NA	NA
ENSBTAG00000019520	0.3321194	0.311581	0.506429
ENSBTAG00000019521	0.0826952	0.7548312	0.1221502
ENSBTAG00000019522	0.0393448	0.8882848	0.0514478
ENSBTAG00000019524	NA	NA	NA
ENSBTAG00000019525	-0.125332	0.6548996	0.1838253
ENSBTAG00000019526	0.0362392	0.8840561	0.0535202
ENSBTAG00000019529	NA	NA	NA
ENSBTAG00000019530	NA	NA	NA
ENSBTAG00000019532	NA	NA	NA
ENSBTAG00000019533	NA	NA	NA
ENSBTAG00000019534	-0.052152	0.8420213	0.0746769
ENSBTAG00000019536	0.021606	0.9314432	0.0308436
ENSBTAG00000019537	-0.147958	0.7259472	0.139095
ENSBTAG00000019538	-0.045482	0.8622169	0.0643835
ENSBTAG00000019539	NA	NA	NA
ENSBTAG00000019542	-0.202507	0.4258493	0.3707441
ENSBTAG00000019543	-0.090812	0.7314877	0.1357929
ENSBTAG00000019545	0.6082547	0.2010053	0.6967925
ENSBTAG00000019547	NA	NA	NA
ENSBTAG00000019548	NA	NA	NA
ENSBTAG00000019550	0.0430941	0.9323436	0.030424
ENSBTAG00000019552	-0.182187	0.5287642	0.276738
ENSBTAG00000019553	NA	NA	NA
ENSBTAG00000019554	0.0142869	0.957909	0.0186757
ENSBTAG00000019555	0.6835037	0.0384013	1.4156535
ENSBTAG00000019556	0.2911401	0.4316206	0.3648978
ENSBTAG00000019557	NA	NA	NA
ENSBTAG00000019563	NA	NA	NA
ENSBTAG00000019564	-0.071532	0.77167	0.1125684
ENSBTAG00000019565	0.0462101	0.8535795	0.068756
ENSBTAG00000019567	NA	NA	NA
ENSBTAG00000019568	-0.332558	0.2678837	0.5720536
ENSBTAG00000019569	0.3060083	0.2279742	0.6421142
ENSBTAG00000019574	0.1336002	0.6064241	0.2172236
ENSBTAG00000019577	NA	NA	NA
ENSBTAG00000019579	-0.398399	0.3615571	0.4418231
ENSBTAG00000019581	0.1149662	0.6770118	0.1694038
ENSBTAG00000019585	-0.108619	0.6766082	0.1696628
ENSBTAG00000019587	-0.144456	0.8009481	0.0963956
ENSBTAG00000019588	0.717712	0.2504676	0.6012485
ENSBTAG00000019589	-0.076579	0.8262028	0.0829134
ENSBTAG00000019591	NA	NA	NA
ENSBTAG00000019592	-0.081986	0.791718	0.1014295
ENSBTAG00000019593	-0.018366	0.9666883	0.0147135
ENSBTAG00000019595	0.3772753	0.2725809	0.5645046
ENSBTAG00000019596	-0.068652	0.8046212	0.0944085
ENSBTAG00000019600	NA	NA	NA
ENSBTAG00000019601	0.1927295	0.429052	0.3674901
ENSBTAG00000019602	NA	NA	NA
ENSBTAG00000019603	0.0883107	0.7658886	0.1158344
ENSBTAG00000019604	-0.206187	0.5147531	0.288401
ENSBTAG00000019605	0.4896193	0.2557449	0.592193

ENSBTAG00000019606	0.2392776	0.5807625	0.2360014
ENSBTAG00000019608	0.5388672	0.0841196	1.0751027
ENSBTAG00000019611	0.1414119	0.6646101	0.1774331
ENSBTAG00000019612	0.0300727	0.9093087	0.0412886
ENSBTAG00000019614	-0.250179	0.4370227	0.359496
ENSBTAG00000019615	-0.176838	0.500963	0.3001944
ENSBTAG00000019616	NA	NA	NA
ENSBTAG00000019617	NA	NA	NA
ENSBTAG00000019621	-0.433393	0.2319557	0.634595
ENSBTAG00000019623	-0.386884	0.4383241	0.3582046
ENSBTAG00000019625	-0.337771	0.2704787	0.567867
ENSBTAG00000019627	-0.043867	0.9015599	0.0450054
ENSBTAG00000019628	NA	NA	NA
ENSBTAG00000019630	0.756399	0.0180084	1.7445251
ENSBTAG00000019633	-0.377904	0.4431217	0.353477
ENSBTAG00000019634	NA	NA	NA
ENSBTAG00000019636	0.3382111	0.1757076	0.7552093
ENSBTAG00000019639	0.3043993	0.2849785	0.5451879
ENSBTAG00000019642	NA	NA	NA
ENSBTAG00000019644	0.2622023	0.3758197	0.4250205
ENSBTAG00000019645	-0.437729	0.2018453	0.6949814
ENSBTAG00000019646	-0.043752	0.8893084	0.0509476
ENSBTAG00000019648	0.1376447	0.6008533	0.2212315
ENSBTAG00000019651	0.1992447	0.6187592	0.2084784
ENSBTAG00000019652	-0.569416	0.1373367	0.8622135
ENSBTAG00000019658	-0.26865	0.3002943	0.5224529
ENSBTAG00000019660	-0.258447	0.4678382	0.3299043
ENSBTAG00000019662	-0.293829	0.3846703	0.4149114
ENSBTAG00000019663	-0.242359	0.400829	0.3970409
ENSBTAG00000019665	NA	NA	NA
ENSBTAG00000019667	-0.312721	0.228699	0.6407358
ENSBTAG00000019669	0.8677155	0.0099853	2.0006393
ENSBTAG00000019670	NA	NA	NA
ENSBTAG00000019672	NA	NA	NA
ENSBTAG00000019674	NA	NA	NA
ENSBTAG00000019675	-0.228422	0.3947588	0.4036682
ENSBTAG00000019676	NA	NA	NA
ENSBTAG00000019678	0.3910953	0.44534	0.3513083
ENSBTAG00000019679	NA	NA	NA
ENSBTAG00000019680	-0.134778	0.585847	0.2322158
ENSBTAG00000019682	0.3748137	0.161413	0.7920616
ENSBTAG00000019684	-0.352381	0.2084834	0.6809285
ENSBTAG00000019685	0.3467591	0.1903512	0.7204444
ENSBTAG00000019686	-0.24613	0.5425922	0.2655265
ENSBTAG00000019687	-0.260891	0.5466558	0.262286
ENSBTAG00000019689	0.1226286	0.7975622	0.0982354
ENSBTAG00000019692	0.2265515	0.464204	0.3332911
ENSBTAG00000019694	NA	NA	NA
ENSBTAG00000019695	-0.1749	0.4777641	0.3207865
ENSBTAG00000019696	-0.086142	0.8806898	0.055177
ENSBTAG00000019697	NA	NA	NA
ENSBTAG00000019698	NA	NA	NA
ENSBTAG00000019700	-0.485485	0.1359476	0.8666285
ENSBTAG00000019701	0.0403945	0.8764861	0.057255
ENSBTAG00000019703	-0.108975	0.6694322	0.1742934
ENSBTAG00000019704	-0.12898	0.6504493	0.1867865
ENSBTAG00000019707	0.0101033	0.9832652	0.0073293
ENSBTAG00000019708	-0.110889	0.8127538	0.090041

ENSBTAG00000019711	NA	NA	NA
ENSBTAG00000019712	NA	NA	NA
ENSBTAG00000019714	0.1838074	0.5143081	0.2887767
ENSBTAG00000019716	NA	NA	NA
ENSBTAG00000019718	0.081038	0.7562913	0.1213109
ENSBTAG00000019721	0.1008986	0.779137	0.1083862
ENSBTAG00000019722	NA	NA	NA
ENSBTAG00000019723	1.1052942	0.0078872	2.1030796
ENSBTAG00000019725	0.1466615	0.5847616	0.2330212
ENSBTAG00000019726	NA	NA	NA
ENSBTAG00000019729	0.1283178	0.6003739	0.2215782
ENSBTAG00000019730	-0.161362	0.5645627	0.2482878
ENSBTAG00000019733	0.9532762	0.0118055	1.9279143
ENSBTAG00000019734	-0.045259	0.9046153	0.0435361
ENSBTAG00000019735	-0.035505	0.8950885	0.048134
ENSBTAG00000019738	-0.237911	0.642446	0.1921634
ENSBTAG00000019741	0.039667	0.946024	0.0240979
ENSBTAG00000019742	-0.254125	0.3056649	0.5147545
ENSBTAG00000019743	NA	NA	NA
ENSBTAG00000019745	0.301999	0.3932976	0.4052787
ENSBTAG00000019746	-0.017101	0.9458117	0.0241953
ENSBTAG00000019748	-0.153715	0.5385008	0.2688136
ENSBTAG00000019750	0.0533752	0.8301949	0.08082
ENSBTAG00000019752	1.0521079	0.370716	0.4309587
ENSBTAG00000019754	0.4347995	0.1544221	0.8112905
ENSBTAG00000019755	0.0053445	0.9853398	0.006414
ENSBTAG00000019757	0.0165005	0.9499646	0.0222926
ENSBTAG00000019759	0.1023734	0.6907093	0.1607047
ENSBTAG00000019761	-0.083895	0.8090646	0.0920168
ENSBTAG00000019764	NA	NA	NA
ENSBTAG00000019767	-0.020962	0.9717228	0.0124576
ENSBTAG00000019770	NA	NA	NA
ENSBTAG00000019771	0.4893646	0.1508236	0.8215308
ENSBTAG00000019772	NA	NA	NA
ENSBTAG00000019777	NA	NA	NA
ENSBTAG00000019779	NA	NA	NA
ENSBTAG00000019780	0.1409228	0.5719845	0.2426158
ENSBTAG00000019781	-0.135246	0.5851504	0.2327325
ENSBTAG00000019782	-0.514139	0.0479541	1.3191743
ENSBTAG00000019783	-0.105708	0.7764858	0.1098665
ENSBTAG00000019784	-0.028356	0.9091847	0.0413479
ENSBTAG00000019785	0.5339929	0.1155417	0.9372611
ENSBTAG00000019787	-0.176018	0.7181858	0.1437632
ENSBTAG00000019788	0.1067076	0.7317139	0.1356587
ENSBTAG00000019790	NA	NA	NA
ENSBTAG00000019791	NA	NA	NA
ENSBTAG00000019792	NA	NA	NA
ENSBTAG00000019793	NA	NA	NA
ENSBTAG00000019794	-0.157587	0.5464611	0.2624407
ENSBTAG00000019798	NA	NA	NA
ENSBTAG00000019800	NA	NA	NA
ENSBTAG00000019801	NA	NA	NA
ENSBTAG00000019803	-0.084204	0.7360222	0.1331091
ENSBTAG00000019804	-0.143064	0.5736317	0.2413669
ENSBTAG00000019805	-0.313164	0.3787264	0.4216745
ENSBTAG00000019806	0.0280211	0.9391811	0.0272506
ENSBTAG00000019807	0.1600663	0.7562963	0.121308
ENSBTAG00000019808	0.0766355	0.843682	0.0738212

ENSBTAG00000019810	-0.178487	0.4773782	0.3211374
ENSBTAG00000019812	-0.116748	0.7572184	0.1207788
ENSBTAG00000019813	0.2362655	0.6370656	0.1958159
ENSBTAG00000019814	NA	NA	NA
ENSBTAG00000019818	0.7745035	0.0217791	1.661961
ENSBTAG00000019819	-0.027369	0.9415495	0.0261568
ENSBTAG00000019820	NA	NA	NA
ENSBTAG00000019821	-0.200431	0.4239634	0.3726717
ENSBTAG00000019822	-0.544499	0.0466702	1.3309603
ENSBTAG00000019823	NA	NA	NA
ENSBTAG00000019828	-0.020908	0.9520864	0.0213236
ENSBTAG00000019830	-0.315631	0.2835845	0.5473175
ENSBTAG00000019831	0.061782	0.8311803	0.0803048
ENSBTAG00000019832	0.1748001	0.4895905	0.3101671
ENSBTAG00000019834	0.4066969	0.2013143	0.6961253
ENSBTAG00000019836	-0.247199	0.3612977	0.4421348
ENSBTAG00000019838	0.2419214	0.3970648	0.4011386
ENSBTAG00000019839	0.2193614	0.4278543	0.3687041
ENSBTAG00000019841	NA	NA	NA
ENSBTAG00000019843	0.0996179	0.704219	0.1522922
ENSBTAG00000019844	NA	NA	NA
ENSBTAG00000019845	0.280381	0.4944228	0.3059015
ENSBTAG00000019846	-0.081635	0.8252731	0.0834023
ENSBTAG00000019847	-0.019634	0.948927	0.0227672
ENSBTAG00000019848	-0.33875	0.2114803	0.67473
ENSBTAG00000019851	0.3122581	0.2066515	0.6847614
ENSBTAG00000019852	-0.145336	0.6018473	0.2205137
ENSBTAG00000019853	-0.316944	0.2199723	0.657632
ENSBTAG00000019854	-0.101257	0.6903394	0.1609373
ENSBTAG00000019855	NA	NA	NA
ENSBTAG00000019857	-0.252807	0.3110817	0.5071256
ENSBTAG00000019859	-0.666952	0.1460225	0.8355803
ENSBTAG00000019864	NA	NA	NA
ENSBTAG00000019866	0.2652931	0.3777117	0.4228395
ENSBTAG00000019867	-0.206414	0.4683592	0.3294209
ENSBTAG00000019868	-0.119032	0.6751289	0.1706133
ENSBTAG00000019869	-0.190002	0.459007	0.3381807
ENSBTAG00000019870	NA	NA	NA
ENSBTAG00000019871	-0.089009	0.8245094	0.0838044
ENSBTAG00000019872	-0.265497	0.3504623	0.4553587
ENSBTAG00000019876	NA	NA	NA
ENSBTAG00000019877	-0.280773	0.2522736	0.5981281
ENSBTAG00000019880	NA	NA	NA
ENSBTAG00000019881	2.6721805	6.01E-07	6.2210489
ENSBTAG00000019883	0.1151722	0.6459888	0.189775
ENSBTAG00000019884	NA	NA	NA
ENSBTAG00000019885	0.350416	0.4655254	0.3320566
ENSBTAG00000019886	0.8118362	0.0676111	1.1699819
ENSBTAG00000019887	-0.022253	0.9591546	0.0181114
ENSBTAG00000019888	0.0666029	0.828399	0.0817604
ENSBTAG00000019889	-0.211597	0.5252294	0.279651
ENSBTAG00000019891	0.1503227	0.6903545	0.1609279
ENSBTAG00000019892	0.4660422	0.2969663	0.5272929
ENSBTAG00000019894	0.1106788	0.7381683	0.1318446
ENSBTAG00000019895	-0.04271	0.8935619	0.0488753
ENSBTAG00000019900	0.0964424	0.7072413	0.1504324
ENSBTAG00000019901	-0.037119	0.9569153	0.0191265
ENSBTAG00000019903	0.5853762	0.0407143	1.3902534

ENSBTAG00000019904	0.3133853	0.2893428	0.5385873
ENSBTAG00000019906	0.0675788	0.8037311	0.0948892
ENSBTAG00000019907	NA	NA	NA
ENSBTAG00000019908	-0.074571	0.8950234	0.0481656
ENSBTAG00000019909	0.9191236	0.0394155	1.4043329
ENSBTAG00000019910	0.0921157	0.7265714	0.1387217
ENSBTAG00000019911	0.0638622	0.7987429	0.097593
ENSBTAG00000019912	NA	NA	NA
ENSBTAG00000019913	-0.101373	0.8139489	0.0894029
ENSBTAG00000019914	-0.171226	0.4940891	0.3061947
ENSBTAG00000019915	0.4648582	0.0760321	1.1190031
ENSBTAG00000019916	0.4219306	0.2908177	0.5363792
ENSBTAG00000019918	-0.045297	0.8958835	0.0477485
ENSBTAG00000019919	NA	NA	NA
ENSBTAG00000019920	NA	NA	NA
ENSBTAG00000019924	0.1571443	0.6779326	0.1688135
ENSBTAG00000019925	NA	NA	NA
ENSBTAG00000019927	-0.014066	0.9582867	0.0185045
ENSBTAG00000019928	NA	NA	NA
ENSBTAG00000019929	0.1235731	0.6802862	0.1673084
ENSBTAG00000019930	-0.216743	0.3839245	0.4157542
ENSBTAG00000019931	NA	NA	NA
ENSBTAG00000019933	0.7840029	0.0093562	2.0288988
ENSBTAG00000019937	0.4125737	0.1878266	0.726243
ENSBTAG00000019938	0.0937181	0.7173202	0.1442869
ENSBTAG00000019939	NA	NA	NA
ENSBTAG00000019940	NA	NA	NA
ENSBTAG00000019942	NA	NA	NA
ENSBTAG00000019944	-0.600937	0.0329444	1.482218
ENSBTAG00000019946	NA	NA	NA
ENSBTAG00000019947	0.5607588	0.224125	0.6495097
ENSBTAG00000019948	0.164367	0.6135613	0.212142
ENSBTAG00000019949	0.7096694	0.0399729	1.3982339
ENSBTAG00000019950	0.0989481	0.7431589	0.1289183
ENSBTAG00000019951	NA	NA	NA
ENSBTAG00000019952	-0.03755	0.8848564	0.0531272
ENSBTAG00000019953	-0.150585	0.7049937	0.1518148
ENSBTAG00000019954	1.4063972	6.95E-06	5.158284
ENSBTAG00000019956	-0.045345	0.8749978	0.057993
ENSBTAG00000019958	NA	NA	NA
ENSBTAG00000019960	0.916058	0.1199559	0.9209786
ENSBTAG00000019961	NA	NA	NA
ENSBTAG00000019964	0.5498372	0.0733655	1.1345083
ENSBTAG00000019966	NA	NA	NA
ENSBTAG00000019967	-0.177498	0.5502094	0.259472
ENSBTAG00000019971	-0.084483	0.7502641	0.1247858
ENSBTAG00000019972	0.2535036	0.4991007	0.3018118
ENSBTAG00000019975	-0.429538	0.4916897	0.3083089
ENSBTAG00000019977	NA	NA	NA
ENSBTAG00000019979	0.2445196	0.5552673	0.2554979
ENSBTAG00000019980	NA	NA	NA
ENSBTAG00000019983	-0.090949	0.7684564	0.1143808
ENSBTAG00000019984	NA	NA	NA
ENSBTAG00000019986	0.2379823	0.3670641	0.4352581
ENSBTAG00000019987	NA	NA	NA
ENSBTAG00000019988	NA	NA	NA
ENSBTAG00000019989	0.067961	0.8516276	0.0697503
ENSBTAG00000019993	-0.995945	0.0240622	1.6186641

ENSBTAG00000019995	-0.234387	0.3690412	0.4329251
ENSBTAG00000019997	NA	NA	NA
ENSBTAG00000019998	-0.004515	0.9865536	0.0058793
ENSBTAG00000020000	0.3287629	0.5004089	0.300675
ENSBTAG00000020001	NA	NA	NA
ENSBTAG00000020003	NA	NA	NA
ENSBTAG00000020004	-0.118086	0.7050322	0.151791
ENSBTAG00000020009	NA	NA	NA
ENSBTAG00000020010	NA	NA	NA
ENSBTAG00000020012	0.2511427	0.3093615	0.5095337
ENSBTAG00000020014	-0.121357	0.6739703	0.1713593
ENSBTAG00000020015	0.538354	0.2965491	0.5279034
ENSBTAG00000020017	NA	NA	NA
ENSBTAG00000020018	0.3938188	0.4034368	0.3942245
ENSBTAG00000020019	NA	NA	NA
ENSBTAG00000020022	NA	NA	NA
ENSBTAG00000020023	-0.053839	0.8931076	0.0490962
ENSBTAG00000020026	-0.219291	0.3752083	0.4257276
ENSBTAG00000020027	NA	NA	NA
ENSBTAG00000020028	-0.011192	0.9907303	0.0040446
ENSBTAG00000020030	1.3026386	4.15E-05	4.3822366
ENSBTAG00000020031	0.1488569	0.5898371	0.2292679
ENSBTAG00000020032	NA	NA	NA
ENSBTAG00000020034	0.0321175	0.9422055	0.0258544
ENSBTAG00000020035	-0.823051	0.0674377	1.1710975
ENSBTAG00000020036	NA	NA	NA
ENSBTAG00000020037	-0.425073	0.3258123	0.4870325
ENSBTAG00000020040	0.5906521	0.1845633	0.7338548
ENSBTAG00000020041	-0.294185	0.2618467	0.581953
ENSBTAG00000020042	-0.234998	0.3821199	0.4178004
ENSBTAG00000020043	0.1130705	0.7204894	0.1423724
ENSBTAG00000020046	0.4755488	0.2768831	0.5577036
ENSBTAG00000020047	-0.093372	0.7749132	0.1107469
ENSBTAG00000020048	NA	NA	NA
ENSBTAG00000020049	NA	NA	NA
ENSBTAG00000020050	-0.325433	0.2614756	0.5825688
ENSBTAG00000020053	0.031935	0.9230602	0.03477
ENSBTAG00000020054	-0.054219	0.867673	0.0616439
ENSBTAG00000020055	-0.103801	0.7283221	0.1376765
ENSBTAG00000020056	0.9079875	0.0394577	1.4038683
ENSBTAG00000020059	NA	NA	NA
ENSBTAG00000020060	0.1446017	0.5886743	0.230125
ENSBTAG00000020061	-0.557405	0.0291319	1.5356308
ENSBTAG00000020062	NA	NA	NA
ENSBTAG00000020066	0.345882	0.2056068	0.6869626
ENSBTAG00000020067	0.2203684	0.6639371	0.1778731
ENSBTAG00000020070	0.103789	0.7986536	0.0976416
ENSBTAG00000020073	0.7728076	0.0252812	1.5972024
ENSBTAG00000020074	NA	NA	NA
ENSBTAG00000020076	NA	NA	NA
ENSBTAG00000020079	0.0200536	0.9409463	0.0264351
ENSBTAG00000020080	-0.116755	0.6641439	0.1777378
ENSBTAG00000020082	NA	NA	NA
ENSBTAG00000020084	-0.590648	0.0725865	1.1391439
ENSBTAG00000020087	-0.536289	0.0452996	1.3439057
ENSBTAG00000020089	-0.249626	0.3467384	0.4599981
ENSBTAG00000020090	NA	NA	NA
ENSBTAG00000020093	0.0744294	0.7909108	0.1018725

ENSBTAG00000020096	NA	NA	NA
ENSBTAG00000020097	NA	NA	NA
ENSBTAG00000020099	0.4466869	0.3232419	0.4904724
ENSBTAG00000020100	NA	NA	NA
ENSBTAG00000020105	-0.181744	0.4953419	0.305095
ENSBTAG00000020106	0.0786464	0.7578399	0.1204225
ENSBTAG00000020107	-0.282544	0.4731643	0.324988
ENSBTAG00000020108	0.2568642	0.3430841	0.4645994
ENSBTAG00000020109	0.4579933	0.1035997	0.9846414
ENSBTAG00000020115	0.1554352	0.5929938	0.2269499
ENSBTAG00000020116	-1.360864	0.0351631	1.4539127
ENSBTAG00000020117	0.2326189	0.4960707	0.3044564
ENSBTAG00000020119	NA	NA	NA
ENSBTAG00000020121	-0.221598	0.4603348	0.3369262
ENSBTAG00000020122	-0.18377	0.4501361	0.3466561
ENSBTAG00000020123	0.0958543	0.700669	0.1544871
ENSBTAG00000020124	0.3772699	0.247339	0.6067075
ENSBTAG00000020125	-0.217686	0.4271118	0.3694584
ENSBTAG00000020126	-0.026115	0.92423	0.0342199
ENSBTAG00000020127	NA	NA	NA
ENSBTAG00000020130	0.3483487	0.2344662	0.6299198
ENSBTAG00000020132	-0.042043	0.8983112	0.0465732
ENSBTAG00000020133	-0.132132	0.6220965	0.2061423
ENSBTAG00000020136	-0.42328	0.1533405	0.814343
ENSBTAG00000020139	-0.013415	0.9589613	0.0181989
ENSBTAG00000020141	-0.154065	0.7506659	0.1245533
ENSBTAG00000020142	0.1053719	0.6613181	0.1795896
ENSBTAG00000020143	-0.062652	0.8763985	0.0572984
ENSBTAG00000020144	NA	NA	NA
ENSBTAG00000020147	0.062992	0.8882399	0.0514697
ENSBTAG00000020148	0.27772	0.308947	0.5101161
ENSBTAG00000020149	-0.198527	0.5105985	0.2919205
ENSBTAG00000020150	NA	NA	NA
ENSBTAG00000020151	NA	NA	NA
ENSBTAG00000020152	0.07014	0.8478391	0.0716865
ENSBTAG00000020153	-0.368846	0.327522	0.4847596
ENSBTAG00000020154	0.1546919	0.6191588	0.2081979
ENSBTAG00000020155	NA	NA	NA
ENSBTAG00000020159	-0.174312	0.7034256	0.1527819
ENSBTAG00000020160	-0.234138	0.5875738	0.2309376
ENSBTAG00000020161	-0.287825	0.4115177	0.3856115
ENSBTAG00000020164	0.0349131	0.9026614	0.0444751
ENSBTAG00000020166	0.2605179	0.3946513	0.4037864
ENSBTAG00000020169	-0.030201	0.9200131	0.036206
ENSBTAG00000020172	NA	NA	NA
ENSBTAG00000020173	0.1646068	0.7062563	0.1510376
ENSBTAG00000020174	-0.348995	0.1625854	0.7889184
ENSBTAG00000020175	-0.083889	0.7641018	0.1168488
ENSBTAG00000020177	0.2079712	0.5258466	0.279141
ENSBTAG00000020178	-0.120725	0.769745	0.1136531
ENSBTAG00000020179	0.3023782	0.49002	0.3097862
ENSBTAG00000020180	-0.007947	0.979299	0.0090847
ENSBTAG00000020185	NA	NA	NA
ENSBTAG00000020186	0.4151816	0.2964549	0.5280414
ENSBTAG00000020190	NA	NA	NA
ENSBTAG00000020192	-0.101114	0.6791001	0.1680662
ENSBTAG00000020193	-0.203259	0.4885759	0.3110679
ENSBTAG00000020194	-0.339321	0.1991218	0.7008812

ENSBTAG00000020196	NA	NA	NA
ENSBTAG00000020198	-1.147641	0.0274995	1.560675
ENSBTAG00000020199	0.8067069	0.0433073	1.3634392
ENSBTAG00000020202	NA	NA	NA
ENSBTAG00000020203	NA	NA	NA
ENSBTAG00000020205	-0.773958	0.0070409	2.1523694
ENSBTAG00000020212	-0.231679	0.3775987	0.4229695
ENSBTAG00000020213	NA	NA	NA
ENSBTAG00000020214	1.1030931	0.0145486	1.8371783
ENSBTAG00000020215	0.0073748	0.9769731	0.0101174
ENSBTAG00000020218	-0.501796	0.0518751	1.285041
ENSBTAG00000020219	-1.537391	8.18E-06	5.0872525
ENSBTAG00000020221	1.1262624	0.0325919	1.4868907
ENSBTAG00000020223	-0.372864	0.1401095	0.8535323
ENSBTAG00000020225	NA	NA	NA
ENSBTAG00000020226	-0.301569	0.3828117	0.4170148
ENSBTAG00000020227	NA	NA	NA
ENSBTAG00000020229	NA	NA	NA
ENSBTAG00000020232	0.3321034	0.3242689	0.4890947
ENSBTAG00000020233	-0.287715	0.4244978	0.3721246
ENSBTAG00000020236	NA	NA	NA
ENSBTAG00000020237	0.0826053	0.7785427	0.1087176
ENSBTAG00000020238	0.0669358	0.9150179	0.0385704
ENSBTAG00000020242	NA	NA	NA
ENSBTAG00000020243	0.2716631	0.3210828	0.493383
ENSBTAG00000020244	0.1215693	0.7409958	0.1301843
ENSBTAG00000020247	NA	NA	NA
ENSBTAG00000020250	1.0064714	0.433635	0.3628757
ENSBTAG00000020252	NA	NA	NA
ENSBTAG00000020253	NA	NA	NA
ENSBTAG00000020257	NA	NA	NA
ENSBTAG00000020260	NA	NA	NA
ENSBTAG00000020261	-0.178972	0.6941263	0.1585615
ENSBTAG00000020262	0.1483666	0.5721779	0.2424689
ENSBTAG00000020263	0.4978489	0.097328	1.0117624
ENSBTAG00000020264	NA	NA	NA
ENSBTAG00000020266	0.1714781	0.6420912	0.1924033
ENSBTAG00000020267	-0.471104	0.2325785	0.6334304
ENSBTAG00000020268	-0.239592	0.3332161	0.4772741
ENSBTAG00000020269	NA	NA	NA
ENSBTAG00000020270	0.1251131	0.6475076	0.1887551
ENSBTAG00000020272	-0.513842	0.0522624	1.2818102
ENSBTAG00000020273	-0.00781	0.9878243	0.0053203
ENSBTAG00000020277	-0.496223	0.1639201	0.7853678
ENSBTAG00000020279	0.0282884	0.9323255	0.0304324
ENSBTAG00000020281	-0.062396	0.8068567	0.0932036
ENSBTAG00000020282	NA	NA	NA
ENSBTAG00000020283	NA	NA	NA
ENSBTAG00000020286	0.1743182	0.6662843	0.1763404
ENSBTAG00000020289	NA	NA	NA
ENSBTAG00000020292	-0.29559	0.246742	0.6077569
ENSBTAG00000020294	-0.167077	0.7092125	0.1492236
ENSBTAG00000020296	-0.301048	0.2282701	0.641551
ENSBTAG00000020297	0.3407174	0.3753064	0.425614
ENSBTAG00000020298	NA	NA	NA
ENSBTAG00000020299	NA	NA	NA
ENSBTAG00000020300	NA	NA	NA
ENSBTAG00000020301	0.1506931	0.6835984	0.1651989

ENSBTAG00000020303	0.1160322	0.6393448	0.1942649
ENSBTAG00000020304	0.1378505	0.6260592	0.2033846
ENSBTAG00000020305	0.1464529	0.6716886	0.172832
ENSBTAG00000020307	-0.181906	0.5227353	0.2817182
ENSBTAG00000020308	0.2746785	0.3161175	0.5001515
ENSBTAG00000020309	0.0663	0.791059	0.1017911
ENSBTAG00000020311	0.4704036	0.1629025	0.7880723
ENSBTAG00000020312	-0.101463	0.7509488	0.1243897
ENSBTAG00000020313	-0.169659	0.5670815	0.2463545
ENSBTAG00000020314	0.1056763	0.7012915	0.1541014
ENSBTAG00000020315	-0.090812	0.7323327	0.1352916
ENSBTAG00000020316	0.0152425	0.9505732	0.0220144
ENSBTAG00000020318	NA	NA	NA
ENSBTAG00000020319	NA	NA	NA
ENSBTAG00000020321	0.2042598	0.5093499	0.2929838
ENSBTAG00000020327	-0.077813	0.7600289	0.1191699
ENSBTAG00000020329	0.4442499	0.2376779	0.6240112
ENSBTAG00000020330	0.7044803	0.0131754	1.8802349
ENSBTAG00000020331	0.1849258	0.5175846	0.2860186
ENSBTAG00000020332	0.0998425	0.7230758	0.1408162
ENSBTAG00000020334	NA	NA	NA
ENSBTAG00000020335	NA	NA	NA
ENSBTAG00000020338	-0.801847	0.0586701	1.2315829
ENSBTAG00000020340	0.2933058	0.3543472	0.450571
ENSBTAG00000020341	NA	NA	NA
ENSBTAG00000020342	0.139438	0.6003464	0.2215981
ENSBTAG00000020344	NA	NA	NA
ENSBTAG00000020345	-0.065669	0.7970243	0.0985285
ENSBTAG00000020346	-0.445647	0.2840157	0.5466577
ENSBTAG00000020348	NA	NA	NA
ENSBTAG00000020350	-0.364207	0.465746	0.3318509
ENSBTAG00000020351	NA	NA	NA
ENSBTAG00000020352	NA	NA	NA
ENSBTAG00000020353	NA	NA	NA
ENSBTAG00000020355	-0.549334	0.0814053	1.0893475
ENSBTAG00000020356	0.0544679	0.836152	0.0777148
ENSBTAG00000020357	0.8162349	0.0107739	1.9676261
ENSBTAG00000020359	1.1541924	0.0043416	2.3623552
ENSBTAG00000020361	NA	NA	NA
ENSBTAG00000020363	NA	NA	NA
ENSBTAG00000020366	0.2218712	0.4332513	0.3632601
ENSBTAG00000020367	0.0869527	0.7668839	0.1152704
ENSBTAG00000020369	NA	NA	NA
ENSBTAG00000020371	0.2680152	0.4680563	0.3297019
ENSBTAG00000020373	NA	NA	NA
ENSBTAG00000020374	0.286079	0.5114339	0.2912105
ENSBTAG00000020376	-0.173589	0.4873769	0.312135
ENSBTAG00000020378	-0.041544	0.8946724	0.048336
ENSBTAG00000020379	0.2250917	0.452119	0.3447472
ENSBTAG00000020381	-0.012003	0.9739529	0.0114621
ENSBTAG00000020382	0.8286016	0.0205247	1.687723
ENSBTAG00000020384	-0.063996	0.7981196	0.097932
ENSBTAG00000020385	NA	NA	NA
ENSBTAG00000020387	-0.021377	0.9323198	0.0304351
ENSBTAG00000020389	-0.139159	0.5727522	0.2420332
ENSBTAG00000020391	NA	NA	NA
ENSBTAG00000020393	-0.46461	0.1160931	0.9351937
ENSBTAG00000020394	NA	NA	NA

ENSBTAG00000020395	0.5456834	0.1015886	0.993155
ENSBTAG00000020397	NA	NA	NA
ENSBTAG00000020398	0.4552868	0.2565447	0.5908369
ENSBTAG00000020399	-0.190172	0.4787965	0.319849
ENSBTAG00000020402	NA	NA	NA
ENSBTAG00000020403	-0.290083	0.2461962	0.6087186
ENSBTAG00000020404	NA	NA	NA
ENSBTAG00000020405	0.0175444	0.9471493	0.0235815
ENSBTAG00000020406	-0.24245	0.5657623	0.247366
ENSBTAG00000020407	0.0054887	0.9875916	0.0054226
ENSBTAG00000020409	NA	NA	NA
ENSBTAG00000020410	-0.096229	0.6947995	0.1581405
ENSBTAG00000020412	0.054994	0.867285	0.0618381
ENSBTAG00000020413	NA	NA	NA
ENSBTAG00000020414	0.7168254	0.0548911	1.2604984
ENSBTAG00000020415	NA	NA	NA
ENSBTAG00000020416	-0.071969	0.8075188	0.0928473
ENSBTAG00000020417	0.1492546	0.5957421	0.2249417
ENSBTAG00000020418	0.5445382	0.2788809	0.5545812
ENSBTAG00000020419	-0.255552	0.5828657	0.2344315
ENSBTAG00000020420	-0.532879	0.0676677	1.1696184
ENSBTAG00000020421	-0.037422	0.8816703	0.0546938
ENSBTAG00000020422	0.1155797	0.6645613	0.177465
ENSBTAG00000020423	0.0988801	0.7399437	0.1308013
ENSBTAG00000020425	NA	NA	NA
ENSBTAG00000020428	0.5841808	0.140602	0.8520085
ENSBTAG00000020429	0.1061557	0.7224209	0.1412097
ENSBTAG00000020430	NA	NA	NA
ENSBTAG00000020431	-0.172795	0.5322625	0.2738741
ENSBTAG00000020432	0.1795122	0.5579132	0.2534334
ENSBTAG00000020433	NA	NA	NA
ENSBTAG00000020434	0.3069699	0.398211	0.3998868
ENSBTAG00000020436	-0.000785	0.9986335	0.0005939
ENSBTAG00000020439	0.084521	0.7496906	0.125118
ENSBTAG00000020440	0.0469781	0.8862217	0.0524576
ENSBTAG00000020441	0.0105903	0.9671048	0.0145265
ENSBTAG00000020444	-0.62381	0.0545916	1.2628744
ENSBTAG00000020445	0.2670507	0.6336552	0.198147
ENSBTAG00000020446	0.5365845	0.0471901	1.3261495
ENSBTAG00000020447	NA	NA	NA
ENSBTAG00000020448	0.1736214	0.6659876	0.1765338
ENSBTAG00000020449	-0.054179	0.8275459	0.0822079
ENSBTAG00000020450	NA	NA	NA
ENSBTAG00000020451	0.0302839	0.9108112	0.0405716
ENSBTAG00000020453	NA	NA	NA
ENSBTAG00000020454	-0.410465	0.1036693	0.9843499
ENSBTAG00000020455	0.0233288	0.9243719	0.0341533
ENSBTAG00000020456	-0.167978	0.4932387	0.3069428
ENSBTAG00000020457	NA	NA	NA
ENSBTAG00000020458	0.325871	0.377771	0.4227713
ENSBTAG00000020459	0.4990762	0.1615502	0.7916925
ENSBTAG00000020465	NA	NA	NA
ENSBTAG00000020466	NA	NA	NA
ENSBTAG00000020467	NA	NA	NA
ENSBTAG00000020468	0.3341335	0.1994615	0.700141
ENSBTAG00000020469	NA	NA	NA
ENSBTAG00000020471	NA	NA	NA
ENSBTAG00000020472	-0.036548	0.8860386	0.0525474

ENSBTAG00000020474	NA	NA	NA
ENSBTAG00000020475	0.4600976	0.1190067	0.9244287
ENSBTAG00000020477	0.0699997	0.7787633	0.1085945
ENSBTAG00000020480	-0.178405	0.5914295	0.228097
ENSBTAG00000020481	-0.169904	0.6861643	0.1635719
ENSBTAG00000020482	-0.033648	0.8981835	0.0466349
ENSBTAG00000020484	0.8868728	0.0255391	1.5927945
ENSBTAG00000020485	0.1701079	0.5803222	0.2363308
ENSBTAG00000020489	0.3293406	0.4011389	0.3967052
ENSBTAG00000020490	-0.155708	0.6804163	0.1672253
ENSBTAG00000020491	NA	NA	NA
ENSBTAG00000020494	NA	NA	NA
ENSBTAG00000020495	-0.553307	0.2084787	0.6809384
ENSBTAG00000020496	-0.025334	0.9209238	0.0357763
ENSBTAG00000020498	-0.143009	0.5899767	0.2291651
ENSBTAG00000020499	NA	NA	NA
ENSBTAG00000020503	0.3417879	0.3313322	0.4797363
ENSBTAG00000020504	-0.331375	0.2515853	0.5993147
ENSBTAG00000020505	-0.196681	0.4266948	0.3698827
ENSBTAG00000020512	NA	NA	NA
ENSBTAG00000020514	NA	NA	NA
ENSBTAG00000020515	0.0527282	0.8858446	0.0526425
ENSBTAG00000020516	NA	NA	NA
ENSBTAG00000020517	0.3381758	0.4177504	0.3790831
ENSBTAG00000020518	-0.279531	0.3061569	0.514056
ENSBTAG00000020520	-0.482646	0.2312812	0.6358597
ENSBTAG00000020523	NA	NA	NA
ENSBTAG00000020524	NA	NA	NA
ENSBTAG00000020525	-0.098075	0.7030608	0.1530071
ENSBTAG00000020527	0.3417269	0.2866629	0.5426286
ENSBTAG00000020528	0.2230729	0.443486	0.3531201
ENSBTAG00000020530	-0.13325	0.6530113	0.1850793
ENSBTAG00000020533	0.0325686	0.9160944	0.0380598
ENSBTAG00000020535	NA	NA	NA
ENSBTAG00000020536	0.6336067	0.1001063	0.9995388
ENSBTAG00000020537	NA	NA	NA
ENSBTAG00000020538	-0.110034	0.8194799	0.0864617
ENSBTAG00000020539	0.5491292	0.1212672	0.9162567
ENSBTAG00000020540	NA	NA	NA
ENSBTAG00000020541	-0.41665	0.0971067	1.0127509
ENSBTAG00000020542	-0.521241	0.0919455	1.0364694
ENSBTAG00000020543	NA	NA	NA
ENSBTAG00000020544	-0.03021	0.9263028	0.033247
ENSBTAG00000020546	0.0115877	0.9749118	0.0110347
ENSBTAG00000020547	-0.162181	0.5454806	0.2632207
ENSBTAG00000020548	NA	NA	NA
ENSBTAG00000020551	-0.059179	0.843659	0.0738331
ENSBTAG00000020552	0.0910402	0.7643636	0.1167
ENSBTAG00000020554	0.2179755	0.6579413	0.1818128
ENSBTAG00000020555	NA	NA	NA
ENSBTAG00000020559	NA	NA	NA
ENSBTAG00000020560	0.1550488	0.5908142	0.2285491
ENSBTAG00000020561	0.2499508	0.4331061	0.3634057
ENSBTAG00000020563	-0.202589	0.5271468	0.2780685
ENSBTAG00000020566	-0.206509	0.5517619	0.2582483
ENSBTAG00000020567	-0.083441	0.7414608	0.1299118
ENSBTAG00000020568	NA	NA	NA
ENSBTAG00000020569	-0.540579	0.0393521	1.405032

ENSBTAG00000020570	NA	NA	NA
ENSBTAG00000020573	0.1216772	0.6699927	0.1739299
ENSBTAG00000020575	NA	NA	NA
ENSBTAG00000020578	NA	NA	NA
ENSBTAG00000020580	NA	NA	NA
ENSBTAG00000020583	-0.307873	0.2627647	0.5804329
ENSBTAG00000020584	-0.639387	0.0265632	1.57572
ENSBTAG00000020589	0.0818352	0.848819	0.0711849
ENSBTAG00000020590	NA	NA	NA
ENSBTAG00000020591	-0.207062	0.5745573	0.2406667
ENSBTAG00000020592	NA	NA	NA
ENSBTAG00000020594	-0.370531	0.1599225	0.7960905
ENSBTAG00000020595	-0.035626	0.8848344	0.053138
ENSBTAG00000020596	-0.465993	0.1189584	0.924605
ENSBTAG00000020597	NA	NA	NA
ENSBTAG00000020598	0.4861656	0.2920496	0.5345434
ENSBTAG00000020600	NA	NA	NA
ENSBTAG00000020601	0.1107626	0.8008108	0.0964701
ENSBTAG00000020602	-1.489946	0.0001633	3.7869207
ENSBTAG00000020605	0.0676383	0.8147207	0.0889912
ENSBTAG00000020606	0.4524335	0.2220389	0.6535709
ENSBTAG00000020607	0.0189956	0.9464395	0.0239072
ENSBTAG00000020608	0.2013561	0.4357184	0.3607941
ENSBTAG00000020610	0.3130676	0.3565481	0.4478819
ENSBTAG00000020611	0.3491112	0.4366433	0.3598732
ENSBTAG00000020612	-0.074588	0.8325451	0.0795922
ENSBTAG00000020613	0.1293291	0.7021309	0.1535819
ENSBTAG00000020614	0.0524509	0.8643181	0.0633264
ENSBTAG00000020615	NA	NA	NA
ENSBTAG00000020616	0.6118209	0.0194571	1.7109219
ENSBTAG00000020617	0.3681462	0.2058012	0.6865521
ENSBTAG00000020619	0.1781763	0.532529	0.2736567
ENSBTAG00000020626	0.1058438	0.6817151	0.1663971
ENSBTAG00000020629	0.0655445	0.8126932	0.0900734
ENSBTAG00000020630	-0.44748	0.1332545	0.8753181
ENSBTAG00000020632	-0.57196	0.0433618	1.3628923
ENSBTAG00000020633	0.1825708	0.678857	0.1682217
ENSBTAG00000020634	0.1155945	0.7193778	0.1430429
ENSBTAG00000020635	0.0064196	0.9821211	0.007835
ENSBTAG00000020636	NA	NA	NA
ENSBTAG00000020638	-0.072113	0.7795015	0.1081831
ENSBTAG00000020641	0.1088342	0.6739762	0.1713554
ENSBTAG00000020642	0.4935758	0.0920734	1.0358657
ENSBTAG00000020643	NA	NA	NA
ENSBTAG00000020644	-0.251252	0.5364115	0.2705019
ENSBTAG00000020645	0.1511677	0.5647466	0.2481464
ENSBTAG00000020646	-0.197007	0.5533343	0.2570124
ENSBTAG00000020647	NA	NA	NA
ENSBTAG00000020648	-0.01	0.9726793	0.0120303
ENSBTAG00000020649	-1.224371	0.0002847	3.5455485
ENSBTAG00000020652	NA	NA	NA
ENSBTAG00000020653	-0.076138	0.7792615	0.1083168
ENSBTAG00000020654	-0.255529	0.3069465	0.5129373
ENSBTAG00000020655	0.2434285	0.3522668	0.4531283
ENSBTAG00000020657	-0.237245	0.420009	0.3767414
ENSBTAG00000020658	0.8019425	0.0404403	1.3931856
ENSBTAG00000020661	0.0134375	0.9641077	0.0158744
ENSBTAG00000020662	-0.183025	0.488292	0.3113204

ENSBTAG00000020663	0.3432694	0.3909705	0.4078561
ENSBTAG00000020664	0.0366018	0.8880771	0.0515493
ENSBTAG00000020665	NA	NA	NA
ENSBTAG00000020667	NA	NA	NA
ENSBTAG00000020669	NA	NA	NA
ENSBTAG00000020671	NA	NA	NA
ENSBTAG00000020674	NA	NA	NA
ENSBTAG00000020676	NA	NA	NA
ENSBTAG00000020679	-0.13257	0.7339092	0.1343577
ENSBTAG00000020681	0.0229601	0.9345104	0.0294158
ENSBTAG00000020685	NA	NA	NA
ENSBTAG00000020688	0.4159536	0.3354765	0.4743379
ENSBTAG00000020689	-0.09317	0.8548535	0.0681083
ENSBTAG00000020693	0.070757	0.7831437	0.1061586
ENSBTAG00000020696	0.4523847	0.3144625	0.5024312
ENSBTAG00000020698	0.5701488	0.041641	1.3804784
ENSBTAG00000020699	NA	NA	NA
ENSBTAG00000020700	0.3676373	0.3183539	0.4970899
ENSBTAG00000020701	-0.359608	0.1637048	0.7859385
ENSBTAG00000020704	0.8470572	0.0820153	1.0861052
ENSBTAG00000020705	NA	NA	NA
ENSBTAG00000020707	-0.641957	0.1010157	0.9956111
ENSBTAG00000020709	0.6443982	0.1499115	0.8241649
ENSBTAG00000020710	-0.845013	0.0405574	1.3919298
ENSBTAG00000020713	-1.932872	6.90E-06	5.1608879
ENSBTAG00000020714	NA	NA	NA
ENSBTAG00000020715	NA	NA	NA
ENSBTAG00000020717	0.1898818	0.5088475	0.2934123
ENSBTAG00000020720	0.2699621	0.4468709	0.3498179
ENSBTAG00000020721	-0.151093	0.5938444	0.2263274
ENSBTAG00000020725	NA	NA	NA
ENSBTAG00000020726	0.3114371	0.213404	0.6707975
ENSBTAG00000020729	0.2696459	0.4863911	0.3130144
ENSBTAG00000020733	0.0989537	0.7043669	0.1522011
ENSBTAG00000020734	-0.079337	0.7627895	0.1175953
ENSBTAG00000020735	-0.443683	0.0898653	1.0464081
ENSBTAG00000020736	NA	NA	NA
ENSBTAG00000020737	0.5226113	0.3152653	0.5013238
ENSBTAG00000020739	0.3222528	0.4485576	0.3481818
ENSBTAG00000020742	-0.535943	0.1895164	0.7223531
ENSBTAG00000020745	NA	NA	NA
ENSBTAG00000020747	0.1833818	0.5585781	0.2529161
ENSBTAG00000020748	0.2942063	0.4200868	0.376661
ENSBTAG00000020749	-0.101334	0.7289828	0.1372827
ENSBTAG00000020750	NA	NA	NA
ENSBTAG00000020751	0.0558322	0.8518998	0.0696115
ENSBTAG00000020752	0.1290699	0.7902255	0.1022489
ENSBTAG00000020754	0.4315541	0.2678077	0.572177
ENSBTAG00000020755	0.3093934	0.5758486	0.2396917
ENSBTAG00000020756	0.1059204	0.7551274	0.1219798
ENSBTAG00000020757	0.1005176	0.6906418	0.1607471
ENSBTAG00000020758	0.6609058	0.1346395	0.8708274
ENSBTAG00000020759	0.6226379	0.0513201	1.2897123
ENSBTAG00000020760	0.5539283	0.1546805	0.8105645
ENSBTAG00000020761	-0.083764	0.7366252	0.1327534
ENSBTAG00000020762	NA	NA	NA
ENSBTAG00000020764	-0.655042	0.0744829	1.1279432
ENSBTAG00000020766	-0.566201	0.3460665	0.4608404

ENSBTAG00000020769	NA	NA	NA
ENSBTAG00000020772	0.3067168	0.465191	0.3323687
ENSBTAG00000020773	0.0047969	0.9940584	0.0025881
ENSBTAG00000020775	-0.027956	0.9161402	0.0380381
ENSBTAG00000020776	-0.169623	0.4916611	0.3083342
ENSBTAG00000020777	-0.019517	0.9558217	0.0196231
ENSBTAG00000020780	0.4263966	0.2094834	0.6788503
ENSBTAG00000020781	-0.40105	0.201585	0.6955418
ENSBTAG00000020782	-0.10281	0.6768494	0.1695079
ENSBTAG00000020783	-0.029053	0.9087813	0.0415406
ENSBTAG00000020787	-0.107293	0.663609	0.1780878
ENSBTAG00000020788	NA	NA	NA
ENSBTAG00000020789	NA	NA	NA
ENSBTAG00000020791	0.0317153	0.9012096	0.0451742
ENSBTAG00000020792	NA	NA	NA
ENSBTAG00000020793	NA	NA	NA
ENSBTAG00000020795	0.062684	0.806215	0.0935492
ENSBTAG00000020796	0.2578769	0.472509	0.3255899
ENSBTAG00000020797	0.3888927	0.2830548	0.5481294
ENSBTAG00000020798	NA	NA	NA
ENSBTAG00000020799	0.0946314	0.7433417	0.1288115
ENSBTAG00000020800	0.0777989	0.8442381	0.0735351
ENSBTAG00000020801	-0.463101	0.0823949	1.0840994
ENSBTAG00000020802	-0.461325	0.0930953	1.0310721
ENSBTAG00000020803	NA	NA	NA
ENSBTAG00000020806	0.1942786	0.5764361	0.2392488
ENSBTAG00000020807	0.165428	0.5010766	0.3000959
ENSBTAG00000020810	NA	NA	NA
ENSBTAG00000020814	NA	NA	NA
ENSBTAG00000020815	0.1501151	0.6159745	0.2104373
ENSBTAG00000020819	-0.036406	0.8843103	0.0533953
ENSBTAG00000020824	-0.05508	0.8879533	0.0516099
ENSBTAG00000020825	1.0766272	0.0283904	1.5468291
ENSBTAG00000020826	NA	NA	NA
ENSBTAG00000020828	NA	NA	NA
ENSBTAG00000020829	0.1468042	0.664754	0.177339
ENSBTAG00000020831	0.1422767	0.609999	0.2146709
ENSBTAG00000020835	0.0848198	0.7452952	0.1276717
ENSBTAG00000020837	NA	NA	NA
ENSBTAG00000020839	-0.129218	0.7647558	0.1164772
ENSBTAG00000020843	NA	NA	NA
ENSBTAG00000020844	0.4082326	0.3085071	0.5107349
ENSBTAG00000020848	0.47416	0.267129	0.5732789
ENSBTAG00000020850	0.3478142	0.2591835	0.5863926
ENSBTAG00000020852	NA	NA	NA
ENSBTAG00000020853	NA	NA	NA
ENSBTAG00000020854	-0.34071	0.3184311	0.4969845
ENSBTAG00000020855	-0.113761	0.6680867	0.1751672
ENSBTAG00000020856	NA	NA	NA
ENSBTAG00000020858	0.1126159	0.7966998	0.0987053
ENSBTAG00000020859	NA	NA	NA
ENSBTAG00000020860	-0.28112	0.255972	0.5918075
ENSBTAG00000020861	0.4346176	0.2436335	0.613263
ENSBTAG00000020865	0.3407158	0.2622537	0.5812783
ENSBTAG00000020869	-0.173443	0.7628517	0.1175599
ENSBTAG00000020872	0.1814529	0.6772961	0.1692215
ENSBTAG00000020873	-0.031032	0.9109544	0.0405034
ENSBTAG00000020878	0.0423891	0.8842114	0.0534439

ENSBTAG00000020880	NA	NA	NA
ENSBTAG00000020883	NA	NA	NA
ENSBTAG00000020884	0.7329967	0.0398458	1.3996175
ENSBTAG00000020886	0.2919052	0.3155688	0.500906
ENSBTAG00000020887	NA	NA	NA
ENSBTAG00000020889	NA	NA	NA
ENSBTAG00000020890	NA	NA	NA
ENSBTAG00000020892	NA	NA	NA
ENSBTAG00000020893	NA	NA	NA
ENSBTAG00000020894	-0.00972	0.9686654	0.0138262
ENSBTAG00000020895	0.5271991	0.2242104	0.6493443
ENSBTAG00000020898	-0.149874	0.6984387	0.1558717
ENSBTAG00000020900	-0.014967	0.968584	0.0138627
ENSBTAG00000020904	-0.005515	0.988774	0.004903
ENSBTAG00000020905	0.0342371	0.890841	0.0501998
ENSBTAG00000020907	NA	NA	NA
ENSBTAG00000020908	-0.066681	0.792967	0.1007449
ENSBTAG00000020911	-0.084712	0.7482604	0.1259472
ENSBTAG00000020914	0.3202861	0.3364011	0.4731426
ENSBTAG00000020921	-0.224172	0.6242661	0.2046303
ENSBTAG00000020922	0.3664054	0.1746572	0.7578134
ENSBTAG00000020923	-1.318195	0.0427885	1.368673
ENSBTAG00000020924	NA	NA	NA
ENSBTAG00000020927	-0.01738	0.9444994	0.0247983
ENSBTAG00000020928	0.4532015	0.18782	0.7262582
ENSBTAG00000020929	NA	NA	NA
ENSBTAG00000020930	0.5746952	0.0742747	1.129159
ENSBTAG00000020931	NA	NA	NA
ENSBTAG00000020933	-0.092868	0.7292327	0.1371338
ENSBTAG00000020934	0.1907822	0.6615977	0.179406
ENSBTAG00000020935	0.0142426	0.9587853	0.0182786
ENSBTAG00000020936	0.0222833	0.9755287	0.01076
ENSBTAG00000020937	-0.512698	0.2083227	0.6812634
ENSBTAG00000020938	0.0232876	0.9406203	0.0265857
ENSBTAG00000020939	0.5318797	0.1829437	0.7376826
ENSBTAG00000020940	0.0549403	0.8319754	0.0798895
ENSBTAG00000020942	-0.263692	0.4558793	0.3411501
ENSBTAG00000020943	-0.178693	0.6067434	0.2169949
ENSBTAG00000020944	-0.148386	0.6310763	0.1999182
ENSBTAG00000020947	-0.416262	0.1790107	0.7471211
ENSBTAG00000020954	-0.035086	0.8927947	0.0492484
ENSBTAG00000020956	NA	NA	NA
ENSBTAG00000020957	NA	NA	NA
ENSBTAG00000020958	0.8324879	0.0670777	1.1734221
ENSBTAG00000020959	-0.260642	0.295981	0.5287362
ENSBTAG00000020961	-0.530229	0.1155655	0.9371718
ENSBTAG00000020962	-0.176391	0.7062077	0.1510676
ENSBTAG00000020963	0.0826947	0.7986024	0.0976694
ENSBTAG00000020964	-0.149593	0.5902426	0.2289694
ENSBTAG00000020965	NA	NA	NA
ENSBTAG00000020968	-0.069084	0.8259865	0.0830271
ENSBTAG00000020969	-0.173552	0.5293942	0.2762208
ENSBTAG00000020973	-0.004578	0.9959572	0.0017593
ENSBTAG00000020975	NA	NA	NA
ENSBTAG00000020979	NA	NA	NA
ENSBTAG00000020980	0.3542604	0.1800751	0.7445463
ENSBTAG00000020981	-0.121068	0.6278768	0.2021256
ENSBTAG00000020983	0.3366145	0.3111716	0.5070001

ENSBTAG00000020984	1.0951051	0.0070429	2.1522469
ENSBTAG00000020985	-0.067832	0.8974904	0.0469702
ENSBTAG00000020987	NA	NA	NA
ENSBTAG00000020988	-0.616635	0.1347083	0.8706058
ENSBTAG00000020989	NA	NA	NA
ENSBTAG00000020990	NA	NA	NA
ENSBTAG00000020991	NA	NA	NA
ENSBTAG00000020992	NA	NA	NA
ENSBTAG00000020994	0.1350169	0.6580437	0.1817453
ENSBTAG00000020996	-0.437462	0.1434185	0.8433948
ENSBTAG00000020998	-0.117026	0.650741	0.1865918
ENSBTAG00000020999	0.221922	0.6227327	0.2056984
ENSBTAG00000021000	0.2219227	0.6022375	0.2202322
ENSBTAG00000021002	0.2566355	0.4320807	0.3644351
ENSBTAG00000021003	NA	NA	NA
ENSBTAG00000021007	NA	NA	NA
ENSBTAG00000021008	0.1253668	0.6766324	0.1696472
ENSBTAG00000021009	-0.228127	0.4119971	0.3851058
ENSBTAG00000021013	NA	NA	NA
ENSBTAG00000021015	NA	NA	NA
ENSBTAG00000021016	0.2614658	0.3412764	0.4668938
ENSBTAG00000021018	0.1303277	0.6664108	0.176258
ENSBTAG00000021019	0.5273463	0.1677912	0.7752307
ENSBTAG00000021020	-0.165138	0.5071247	0.2948852
ENSBTAG00000021021	NA	NA	NA
ENSBTAG00000021023	-0.12133	0.6268518	0.2028352
ENSBTAG00000021024	0.2641231	0.3413239	0.4668333
ENSBTAG00000021025	-0.168709	0.7137533	0.1464519
ENSBTAG00000021029	NA	NA	NA
ENSBTAG00000021033	NA	NA	NA
ENSBTAG00000021035	-0.434265	0.6345669	0.1975226
ENSBTAG00000021036	0.1005105	0.7120805	0.1474709
ENSBTAG00000021037	0.1867242	0.4752215	0.3231039
ENSBTAG00000021039	-0.034785	0.8949126	0.0482194
ENSBTAG00000021041	NA	NA	NA
ENSBTAG00000021045	0.348722	0.3680848	0.4340521
ENSBTAG00000021048	0.6181127	0.0956728	1.0192115
ENSBTAG00000021050	NA	NA	NA
ENSBTAG00000021051	0.2062474	0.5772896	0.2386062
ENSBTAG00000021052	0.1649855	0.6206654	0.2071424
ENSBTAG00000021058	-0.053171	0.8391236	0.0761741
ENSBTAG00000021059	NA	NA	NA
ENSBTAG00000021060	0.5289269	0.1091457	0.9619934
ENSBTAG00000021061	0.1616395	0.5522916	0.2578316
ENSBTAG00000021062	0.1512163	0.6113156	0.2137345
ENSBTAG00000021064	NA	NA	NA
ENSBTAG00000021065	-0.131499	0.715093	0.1456375
ENSBTAG00000021066	NA	NA	NA
ENSBTAG00000021067	0.13294	0.5906176	0.2286936
ENSBTAG00000021068	-1.034649	0.0144707	1.8395115
ENSBTAG00000021069	NA	NA	NA
ENSBTAG00000021071	0.2649427	0.3907575	0.4080927
ENSBTAG00000021072	-0.092635	0.8312523	0.0802671
ENSBTAG00000021073	-0.575674	0.206005	0.6861221
ENSBTAG00000021076	0.0581363	0.8307358	0.0805371
ENSBTAG00000021077	NA	NA	NA
ENSBTAG00000021078	-0.034527	0.8976337	0.0469009
ENSBTAG00000021079	NA	NA	NA

ENSBTAG00000021082	NA	NA	NA
ENSBTAG00000021083	0.0256443	0.9450812	0.0245309
ENSBTAG00000021087	-0.049769	0.840939	0.0752355
ENSBTAG00000021090	-0.090498	0.7470433	0.1266542
ENSBTAG00000021091	0.1811505	0.5003354	0.3007388
ENSBTAG00000021092	NA	NA	NA
ENSBTAG00000021093	0.3823592	0.138885	0.8573446
ENSBTAG00000021096	0.4962612	0.2063594	0.6853758
ENSBTAG00000021097	0.6203549	0.0966777	1.0146739
ENSBTAG00000021100	-0.278097	0.3747829	0.4262202
ENSBTAG00000021101	NA	NA	NA
ENSBTAG00000021102	-0.590921	0.1160056	0.935521
ENSBTAG00000021103	-0.25209	0.3148943	0.5018352
ENSBTAG00000021105	0.020076	0.9556537	0.0196995
ENSBTAG00000021107	-0.218707	0.4740151	0.3242078
ENSBTAG00000021111	NA	NA	NA
ENSBTAG00000021113	NA	NA	NA
ENSBTAG00000021115	0.1282012	0.7391271	0.1312809
ENSBTAG00000021116	-0.227371	0.3700234	0.4317708
ENSBTAG00000021118	NA	NA	NA
ENSBTAG00000021119	NA	NA	NA
ENSBTAG00000021120	-0.3421	0.1985624	0.7021029
ENSBTAG00000021121	0.2338686	0.4728151	0.3253087
ENSBTAG00000021122	NA	NA	NA
ENSBTAG00000021125	0.1403108	0.6415172	0.1927917
ENSBTAG00000021127	NA	NA	NA
ENSBTAG00000021128	-0.21728	0.4030051	0.3946894
ENSBTAG00000021130	-0.157864	0.7196326	0.1428892
ENSBTAG00000021131	0.0049366	0.9843874	0.0068339
ENSBTAG00000021132	-0.034336	0.936085	0.0286847
ENSBTAG00000021133	0.6135978	0.1166747	0.9330232
ENSBTAG00000021134	0.3795567	0.1316445	0.8805971
ENSBTAG00000021139	NA	NA	NA
ENSBTAG00000021140	-0.270574	0.2931276	0.5329432
ENSBTAG00000021141	NA	NA	NA
ENSBTAG00000021143	-0.298486	0.2699505	0.5687158
ENSBTAG00000021144	-0.181834	0.6932301	0.1591226
ENSBTAG00000021145	NA	NA	NA
ENSBTAG00000021147	NA	NA	NA
ENSBTAG00000021151	0.3022852	0.308135	0.5112589
ENSBTAG00000021156	-0.000595	1	0
ENSBTAG00000021157	0.2366819	0.3758014	0.4250416
ENSBTAG00000021158	-0.37831	0.1724004	0.7634618
ENSBTAG00000021160	NA	NA	NA
ENSBTAG00000021161	NA	NA	NA
ENSBTAG00000021162	NA	NA	NA
ENSBTAG00000021164	-0.146954	0.5660478	0.2471469
ENSBTAG00000021165	0.2207304	0.6210167	0.2068967
ENSBTAG00000021166	NA	NA	NA
ENSBTAG00000021168	0.0534286	0.8959229	0.0477294
ENSBTAG00000021170	-0.388821	0.1359907	0.8664908
ENSBTAG00000021172	-0.003046	0.9905575	0.0041203
ENSBTAG00000021173	-0.056142	0.8951001	0.0481284
ENSBTAG00000021174	-0.524703	0.0430224	1.3663057
ENSBTAG00000021176	0.7156718	0.0043313	2.3633825
ENSBTAG00000021177	NA	NA	NA
ENSBTAG00000021181	NA	NA	NA
ENSBTAG00000021182	-0.344797	0.2444326	0.6118408

ENSBTAG00000021183	0.392955	0.3227355	0.4911532
ENSBTAG00000021187	0.1776051	0.6050347	0.2182197
ENSBTAG00000021188	-0.279592	0.2899965	0.5376072
ENSBTAG00000021189	NA	NA	NA
ENSBTAG00000021190	NA	NA	NA
ENSBTAG00000021191	0.3768196	0.1658573	0.7802655
ENSBTAG00000021192	0.0520145	0.8452084	0.0730362
ENSBTAG00000021193	NA	NA	NA
ENSBTAG00000021195	NA	NA	NA
ENSBTAG00000021196	-0.410549	0.235286	0.6284039
ENSBTAG00000021199	-0.13236	0.6158619	0.2105166
ENSBTAG00000021200	NA	NA	NA
ENSBTAG00000021201	-0.014495	0.9555974	0.0197251
ENSBTAG00000021202	-0.46021	0.2617632	0.5820914
ENSBTAG00000021204	-0.134958	0.6400001	0.19382
ENSBTAG00000021205	-0.455277	0.0777258	1.1094347
ENSBTAG00000021208	-0.210964	0.5507222	0.2590674
ENSBTAG00000021209	-0.249196	0.3385293	0.4704038
ENSBTAG00000021210	-0.604731	0.1540522	0.8123322
ENSBTAG00000021211	0.3143662	0.2980861	0.5256583
ENSBTAG00000021214	-0.119085	0.6299653	0.2006834
ENSBTAG00000021215	0.2484039	0.5360484	0.270796
ENSBTAG00000021216	NA	NA	NA
ENSBTAG00000021217	NA	NA	NA
ENSBTAG00000021218	-0.280879	0.2865898	0.5427392
ENSBTAG00000021219	NA	NA	NA
ENSBTAG00000021220	NA	NA	NA
ENSBTAG00000021222	-0.379321	0.1546284	0.8107108
ENSBTAG00000021223	-1.007661	0.0036582	2.4367299
ENSBTAG00000021224	-0.824714	0.1046808	0.9801328
ENSBTAG00000021225	-0.005622	0.982297	0.0077572
ENSBTAG00000021226	-0.015243	0.9537222	0.0205781
ENSBTAG00000021227	0.0583232	0.8205353	0.0859027
ENSBTAG00000021230	NA	NA	NA
ENSBTAG00000021231	-0.238972	0.4833378	0.3157493
ENSBTAG00000021232	0.0187793	0.9402788	0.0267433
ENSBTAG00000021235	NA	NA	NA
ENSBTAG00000021237	-0.440318	0.0871252	1.0598561
ENSBTAG00000021240	NA	NA	NA
ENSBTAG00000021242	0.119588	0.6604626	0.1801518
ENSBTAG00000021245	0.2913571	0.2904285	0.5369608
ENSBTAG00000021246	-0.020354	0.947779	0.0232929
ENSBTAG00000021248	0.1641605	0.6184849	0.2086709
ENSBTAG00000021249	NA	NA	NA
ENSBTAG00000021250	-0.292783	0.2497364	0.6025181
ENSBTAG00000021251	NA	NA	NA
ENSBTAG00000021252	0.273323	0.5920057	0.2276741
ENSBTAG00000021253	0.8277421	0.0677564	1.1690495
ENSBTAG00000021254	-0.293365	0.3327837	0.477838
ENSBTAG00000021257	NA	NA	NA
ENSBTAG00000021259	-0.632567	0.12744	0.8946942
ENSBTAG00000021260	-0.204105	0.476701	0.321754
ENSBTAG00000021262	-0.101846	0.6867028	0.1632312
ENSBTAG00000021263	0.6629829	0.0689389	1.1615357
ENSBTAG00000021270	NA	NA	NA
ENSBTAG00000021272	-0.217115	0.5942784	0.22601
ENSBTAG00000021273	0.9735209	0.1105594	0.9564045
ENSBTAG00000021275	-0.123761	0.7372372	0.1323927

ENSBTAG00000021276	NA	NA	NA
ENSBTAG00000021282	0.1861524	0.6321223	0.1991989
ENSBTAG00000021283	NA	NA	NA
ENSBTAG00000021286	1.1205011	0.0211966	1.6737346
ENSBTAG00000021287	-0.33218	0.4408652	0.3556942
ENSBTAG00000021288	-0.016679	0.9477321	0.0233144
ENSBTAG00000021289	-0.237854	0.3927297	0.4059063
ENSBTAG00000021291	0.057719	0.8543002	0.0683895
ENSBTAG00000021292	NA	NA	NA
ENSBTAG00000021293	-0.067887	0.8121815	0.0903469
ENSBTAG00000021294	-0.696136	0.0397612	1.4005409
ENSBTAG00000021298	-0.459181	0.2542381	0.5947594
ENSBTAG00000021301	0.2351444	0.4754079	0.3229336
ENSBTAG00000021302	NA	NA	NA
ENSBTAG00000021303	0.2830861	0.3842486	0.4153877
ENSBTAG00000021304	-0.03975	0.8748358	0.0580735
ENSBTAG00000021306	NA	NA	NA
ENSBTAG00000021307	0.1733865	0.5326505	0.2735577
ENSBTAG00000021308	-0.463088	0.179487	0.7459671
ENSBTAG00000021310	-0.507584	0.2034129	0.6916214
ENSBTAG00000021313	NA	NA	NA
ENSBTAG00000021316	-0.4739	0.2993231	0.5238598
ENSBTAG00000021318	NA	NA	NA
ENSBTAG00000021319	-0.085901	0.7299556	0.1367035
ENSBTAG00000021321	NA	NA	NA
ENSBTAG00000021322	NA	NA	NA
ENSBTAG00000021323	-0.347027	0.1761547	0.7541057
ENSBTAG00000021325	NA	NA	NA
ENSBTAG00000021327	NA	NA	NA
ENSBTAG00000021328	NA	NA	NA
ENSBTAG00000021329	-0.066358	0.8896454	0.0507831
ENSBTAG00000021333	NA	NA	NA
ENSBTAG00000021334	-0.196486	0.5248402	0.2799729
ENSBTAG00000021336	NA	NA	NA
ENSBTAG00000021337	0.3342967	0.221392	0.6548381
ENSBTAG00000021338	0.0943122	0.8205975	0.0858698
ENSBTAG00000021339	NA	NA	NA
ENSBTAG00000021340	NA	NA	NA
ENSBTAG00000021341	-0.173248	0.5536609	0.2567561
ENSBTAG00000021342	-0.135534	0.7482417	0.1259581
ENSBTAG00000021343	0.4242912	0.0890466	1.0503826
ENSBTAG00000021346	-0.359584	0.475362	0.3229755
ENSBTAG00000021347	NA	NA	NA
ENSBTAG00000021351	0.1769053	0.5649346	0.2480018
ENSBTAG00000021357	-1.28422	0.0725151	1.1395713
ENSBTAG00000021358	NA	NA	NA
ENSBTAG00000021359	NA	NA	NA
ENSBTAG00000021360	NA	NA	NA
ENSBTAG00000021361	NA	NA	NA
ENSBTAG00000021364	0.5479743	0.1113336	0.9533738
ENSBTAG00000021367	0.7487363	0.0998058	1.000844
ENSBTAG00000021368	NA	NA	NA
ENSBTAG00000021370	-0.214681	0.3934618	0.4050975
ENSBTAG00000021372	0.0685035	0.7911308	0.1017517
ENSBTAG00000021373	0.3193363	0.3594818	0.4443231
ENSBTAG00000021374	0.1819404	0.5138375	0.2891742
ENSBTAG00000021377	-0.019044	0.9625056	0.0165967
ENSBTAG00000021378	0.3123249	0.2985068	0.5250457

ENSBTAG00000021381	-0.047219	0.8775342	0.0567359
ENSBTAG00000021386	NA	NA	NA
ENSBTAG00000021390	NA	NA	NA
ENSBTAG00000021392	-0.084125	0.7432182	0.1288837
ENSBTAG00000021393	NA	NA	NA
ENSBTAG00000021394	-0.258187	0.3473778	0.4591979
ENSBTAG00000021395	-0.259193	0.3325298	0.4781694
ENSBTAG00000021396	-0.239836	0.4098026	0.3874253
ENSBTAG00000021397	NA	NA	NA
ENSBTAG00000021398	-0.029112	0.9127887	0.0396298
ENSBTAG00000021407	-0.328192	0.3143378	0.5026033
ENSBTAG00000021408	NA	NA	NA
ENSBTAG00000021410	-0.198722	0.5848231	0.2329755
ENSBTAG00000021413	2.0704753	4.60E-05	4.3376474
ENSBTAG00000021415	NA	NA	NA
ENSBTAG00000021416	-0.211649	0.4711743	0.3268184
ENSBTAG00000021417	-0.078912	0.8157339	0.0884515
ENSBTAG00000021420	0.4404277	0.3621631	0.4410958
ENSBTAG00000021421	0.0656378	0.7932355	0.1005979
ENSBTAG00000021422	NA	NA	NA
ENSBTAG00000021423	NA	NA	NA
ENSBTAG00000021424	-0.077521	0.8551568	0.0679542
ENSBTAG00000021425	NA	NA	NA
ENSBTAG00000021426	NA	NA	NA
ENSBTAG00000021427	NA	NA	NA
ENSBTAG00000021430	NA	NA	NA
ENSBTAG00000021433	-0.74496	0.0678411	1.1685069
ENSBTAG00000021435	0.8187439	0.0141447	1.8494064
ENSBTAG00000021437	NA	NA	NA
ENSBTAG00000021438	-0.537768	0.2115489	0.6745893
ENSBTAG00000021442	0.025978	0.9463667	0.0239405
ENSBTAG00000021444	NA	NA	NA
ENSBTAG00000021445	0.4109087	0.3103388	0.508164
ENSBTAG00000021447	0.1639059	0.8317811	0.079991
ENSBTAG00000021449	0.0339392	0.9056296	0.0430494
ENSBTAG00000021452	0.6024827	0.0758601	1.1199863
ENSBTAG00000021454	NA	NA	NA
ENSBTAG00000021455	0.2946062	0.2692741	0.5698054
ENSBTAG00000021456	0.170093	0.5737217	0.2412987
ENSBTAG00000021457	0.1770019	0.68784	0.1625126
ENSBTAG00000021458	NA	NA	NA
ENSBTAG00000021461	0.2784263	0.3084715	0.510785
ENSBTAG00000021462	NA	NA	NA
ENSBTAG00000021464	-0.17101	0.5732241	0.2416756
ENSBTAG00000021466	-0.167887	0.6408191	0.1932646
ENSBTAG00000021467	0.2551583	0.3727868	0.4285394
ENSBTAG00000021468	0.2555132	0.3642556	0.4385938
ENSBTAG00000021469	0.2490513	0.6203791	0.2073428
ENSBTAG00000021471	0.0820323	0.7992972	0.0972917
ENSBTAG00000021472	0.5067474	0.2475133	0.6064014
ENSBTAG00000021474	0.5684001	0.1399116	0.8541463
ENSBTAG00000021480	0.2916071	0.5489284	0.2604843
ENSBTAG00000021481	0.1501013	0.6515786	0.1860332
ENSBTAG00000021482	0.2306149	0.4622468	0.3351261
ENSBTAG00000021483	0.4186725	0.2563184	0.5912202
ENSBTAG00000021484	NA	NA	NA
ENSBTAG00000021485	0.0226778	0.9496287	0.0224462
ENSBTAG00000021487	-0.18323	0.656453	0.1827964

ENSBTAG00000021490	0.3370604	0.4576441	0.3394722
ENSBTAG00000021491	0.0079383	0.9747333	0.0111142
ENSBTAG00000021492	NA	NA	NA
ENSBTAG00000021494	NA	NA	NA
ENSBTAG00000021497	0.0263899	0.9469372	0.0236788
ENSBTAG00000021499	0.2113329	0.4303218	0.3662066
ENSBTAG00000021501	NA	NA	NA
ENSBTAG00000021504	NA	NA	NA
ENSBTAG00000021505	0.410329	0.2017029	0.695288
ENSBTAG00000021506	-0.105188	0.6822872	0.1660328
ENSBTAG00000021508	-0.736798	0.0193659	1.7129626
ENSBTAG00000021512	0.3258843	0.2769292	0.5576312
ENSBTAG00000021514	0.0470063	0.854224	0.0684282
ENSBTAG00000021515	NA	NA	NA
ENSBTAG00000021516	0.505832	0.7444411	0.1281696
ENSBTAG00000021517	NA	NA	NA
ENSBTAG00000021519	0.0703507	0.7959222	0.0991294
ENSBTAG00000021520	-0.163272	0.5390281	0.2683886
ENSBTAG00000021521	0.022661	0.9316369	0.0307533
ENSBTAG00000021522	NA	NA	NA
ENSBTAG00000021523	0.0158511	0.9489714	0.0227469
ENSBTAG00000021525	NA	NA	NA
ENSBTAG00000021526	NA	NA	NA
ENSBTAG00000021527	0.4815731	0.0951194	1.0217308
ENSBTAG00000021531	0.9074614	0.041542	1.3815125
ENSBTAG00000021535	0.0651112	0.8362741	0.0776513
ENSBTAG00000021537	0.0737453	0.8654518	0.0627571
ENSBTAG00000021538	0.3961782	0.2887624	0.5394594
ENSBTAG00000021540	0.0113203	0.970896	0.0128273
ENSBTAG00000021543	-0.203224	0.5935881	0.2265148
ENSBTAG00000021544	-0.007356	0.9810194	0.0083224
ENSBTAG00000021547	NA	NA	NA
ENSBTAG00000021549	-0.004977	0.9845964	0.0067418
ENSBTAG00000021550	NA	NA	NA
ENSBTAG00000021553	-0.055707	0.8398731	0.0757863
ENSBTAG00000021554	-0.094276	0.7049305	0.1518537
ENSBTAG00000021556	-0.101421	0.6950799	0.1579653
ENSBTAG00000021557	NA	NA	NA
ENSBTAG00000021558	0.1465114	0.613428	0.2122364
ENSBTAG00000021565	0.1172888	0.9149429	0.038606
ENSBTAG00000021568	NA	NA	NA
ENSBTAG00000021569	0.2648359	0.2916359	0.535159
ENSBTAG00000021570	NA	NA	NA
ENSBTAG00000021573	0.0751958	0.7719227	0.1124262
ENSBTAG00000021574	0.4337726	0.2881348	0.5404043
ENSBTAG00000021575	-0.235243	0.356805	0.4475691
ENSBTAG00000021576	0.1459609	0.5715283	0.2429622
ENSBTAG00000021577	-0.033009	0.894572	0.0483847
ENSBTAG00000021580	-1.303318	8.51E-05	4.0700342
ENSBTAG00000021581	-0.334043	0.3220434	0.4920856
ENSBTAG00000021582	0.0087375	0.9933025	0.0029185
ENSBTAG00000021583	0.1123169	0.786617	0.1042367
ENSBTAG00000021586	0.0403745	0.9229414	0.0348259
ENSBTAG00000021587	1.1974334	0.0001504	3.8226396
ENSBTAG00000021588	NA	NA	NA
ENSBTAG00000021590	NA	NA	NA
ENSBTAG00000021592	-0.027438	0.9221973	0.0351761
ENSBTAG00000021594	NA	NA	NA

ENSBTAG00000021595	0.8238537	0.0062341	2.2052283
ENSBTAG00000021596	NA	NA	NA
ENSBTAG00000021600	0.348263	0.2055231	0.6871393
ENSBTAG00000021601	NA	NA	NA
ENSBTAG00000021602	0.0095509	0.9791769	0.0091388
ENSBTAG00000021604	0.5610945	0.1531157	0.8149804
ENSBTAG00000021606	-0.206411	0.5217039	0.2825759
ENSBTAG00000021607	-0.087613	0.831225	0.0802814
ENSBTAG00000021609	NA	NA	NA
ENSBTAG00000021611	-0.146257	0.6339656	0.1979343
ENSBTAG00000021614	-0.058851	0.8728083	0.0590811
ENSBTAG00000021616	NA	NA	NA
ENSBTAG00000021617	0.3514569	0.2533762	0.5962341
ENSBTAG00000021620	0.1127786	0.7003216	0.1547025
ENSBTAG00000021626	NA	NA	NA
ENSBTAG00000021630	-0.47444	0.1806091	0.7432603
ENSBTAG00000021632	-0.145543	0.570009	0.2441183
ENSBTAG00000021633	0.0413964	0.8942276	0.0485519
ENSBTAG00000021634	-0.175595	0.6831757	0.1654676
ENSBTAG00000021635	0.52063	0.142066	0.8475097
ENSBTAG00000021636	-0.162166	0.5847415	0.233036
ENSBTAG00000021637	0.0633275	0.8425068	0.0744266
ENSBTAG00000021639	NA	NA	NA
ENSBTAG00000021643	NA	NA	NA
ENSBTAG00000021645	-0.001705	0.9993731	0.0002724
ENSBTAG00000021647	NA	NA	NA
ENSBTAG00000021649	NA	NA	NA
ENSBTAG00000021651	-0.133618	0.6243021	0.2046052
ENSBTAG00000021652	NA	NA	NA
ENSBTAG00000021653	-0.36141	0.1579456	0.8014925
ENSBTAG00000021654	NA	NA	NA
ENSBTAG00000021656	0.2842087	0.35761	0.4465904
ENSBTAG00000021657	0.1633948	0.567912	0.245719
ENSBTAG00000021658	0.2359295	0.4608294	0.3364598
ENSBTAG00000021660	-0.051743	0.8623114	0.0643359
ENSBTAG00000021661	0.527187	0.0782044	1.1067689
ENSBTAG00000021663	-0.073402	0.8157076	0.0884655
ENSBTAG00000021664	NA	NA	NA
ENSBTAG00000021667	NA	NA	NA
ENSBTAG00000021668	-0.014107	0.9729535	0.0119079
ENSBTAG00000021669	0.228648	0.5064913	0.295428
ENSBTAG00000021671	0.0315493	0.9293246	0.0318326
ENSBTAG00000021672	-0.567069	0.2094679	0.6788826
ENSBTAG00000021673	NA	NA	NA
ENSBTAG00000021675	-0.196889	0.4432642	0.3533373
ENSBTAG00000021677	0.1690843	0.605665	0.2177675
ENSBTAG00000021678	0.4062261	0.1708828	0.7673017
ENSBTAG00000021680	-0.308573	0.2960442	0.5286434
ENSBTAG00000021681	NA	NA	NA
ENSBTAG00000021684	NA	NA	NA
ENSBTAG00000021685	0.132301	0.6421055	0.1923936
ENSBTAG00000021686	NA	NA	NA
ENSBTAG00000021687	0.2815496	0.4195925	0.3771723
ENSBTAG00000021688	-0.004475	0.9859245	0.0061563
ENSBTAG00000021691	-0.09548	0.7096773	0.1489391
ENSBTAG00000021692	NA	NA	NA
ENSBTAG00000021693	NA	NA	NA
ENSBTAG00000021694	-0.095971	0.7102316	0.1486

ENSBTAG00000021695	-0.143503	0.6583537	0.1815407
ENSBTAG00000021696	NA	NA	NA
ENSBTAG00000021697	0.0676043	0.8256609	0.0831983
ENSBTAG00000021699	NA	NA	NA
ENSBTAG00000021700	-0.475139	0.1368425	0.863779
ENSBTAG00000021703	0.2430024	0.6331298	0.1985073
ENSBTAG00000021705	-0.049892	0.8729815	0.058995
ENSBTAG00000021706	-0.009467	0.9834617	0.0072426
ENSBTAG00000021707	NA	NA	NA
ENSBTAG00000021708	-0.300901	0.410665	0.3865123
ENSBTAG00000021709	NA	NA	NA
ENSBTAG00000021713	0.3678216	0.5053291	0.2964257
ENSBTAG00000021714	NA	NA	NA
ENSBTAG00000021715	-0.113847	0.7287544	0.1374188
ENSBTAG00000021717	NA	NA	NA
ENSBTAG00000021720	-0.157148	0.5330121	0.2732629
ENSBTAG00000021723	0.3908915	0.3843948	0.4152225
ENSBTAG00000021724	-0.181685	0.5262108	0.2788403
ENSBTAG00000021725	-0.165275	0.5898897	0.2292292
ENSBTAG00000021726	-0.35471	0.1734291	0.760878
ENSBTAG00000021728	NA	NA	NA
ENSBTAG00000021730	NA	NA	NA
ENSBTAG00000021731	0.1690191	0.6193559	0.2080597
ENSBTAG00000021735	0.2066707	0.6648039	0.1773064
ENSBTAG00000021739	NA	NA	NA
ENSBTAG00000021741	0.5367508	0.1027019	0.9884214
ENSBTAG00000021742	-0.079191	0.7915914	0.1014989
ENSBTAG00000021743	-1.151947	5.20E-05	4.2835918
ENSBTAG00000021744	0.0648495	0.8084058	0.0923706
ENSBTAG00000021745	0.0619581	0.8267128	0.0826453
ENSBTAG00000021746	0.3154335	0.2224569	0.6527541
ENSBTAG00000021751	0.5169422	0.4305145	0.3660123
ENSBTAG00000021752	-0.551194	0.080839	1.0923793
ENSBTAG00000021754	-0.362195	0.3928357	0.4057891
ENSBTAG00000021756	-0.104292	0.6922052	0.1597651
ENSBTAG00000021759	-0.045967	0.8605003	0.065249
ENSBTAG00000021761	-0.434056	0.0892488	1.0493975
ENSBTAG00000021762	-0.216037	0.3999297	0.3980163
ENSBTAG00000021764	NA	NA	NA
ENSBTAG00000021766	0.663506	0.0536534	1.2704028
ENSBTAG00000021767	-0.094939	0.7086828	0.1495481
ENSBTAG00000021768	-0.725057	0.0341186	1.4670086
ENSBTAG00000021769	-0.226291	0.3602947	0.4433421
ENSBTAG00000021771	0.1703762	0.5147689	0.2883877
ENSBTAG00000021772	0.1557527	0.5313554	0.2746149
ENSBTAG00000021773	0.3108116	0.3358992	0.4737911
ENSBTAG00000021774	NA	NA	NA
ENSBTAG00000021775	NA	NA	NA
ENSBTAG00000021776	-0.141076	0.5814901	0.2354577
ENSBTAG00000021777	NA	NA	NA
ENSBTAG00000021778	0.8782935	0.0214285	1.6690084
ENSBTAG00000021779	-0.058602	0.8347868	0.0784244
ENSBTAG00000021780	-0.03364	0.8967526	0.0473274
ENSBTAG00000021781	0.1523312	0.5383231	0.268957
ENSBTAG00000021785	-0.230716	0.4247488	0.3718679
ENSBTAG00000021786	NA	NA	NA
ENSBTAG00000021787	NA	NA	NA
ENSBTAG00000021789	0.2329664	0.5487961	0.260589

ENSBTAG00000021790	-0.416169	0.1276771	0.8938868
ENSBTAG00000021791	0.1778639	0.626339	0.2031905
ENSBTAG00000021796	-0.254006	0.5062095	0.2956697
ENSBTAG00000021798	-0.305116	0.2740922	0.5621034
ENSBTAG00000021799	0.0509816	0.8916695	0.0497961
ENSBTAG00000021801	-0.28074	0.2951934	0.5298934
ENSBTAG00000021802	-0.511782	0.1281092	0.8924198
ENSBTAG00000021803	0.0430277	0.9126737	0.0396844
ENSBTAG00000021805	-0.069609	0.7829841	0.1062471
ENSBTAG00000021808	-0.08695	0.8390495	0.0762124
ENSBTAG00000021809	NA	NA	NA
ENSBTAG00000021810	-0.111409	0.659977	0.1804712
ENSBTAG00000021811	-0.1208	0.7474174	0.1264368
ENSBTAG00000021813	-0.132238	0.6263331	0.2031946
ENSBTAG00000021815	0.1212803	0.6606881	0.1800035
ENSBTAG00000021816	NA	NA	NA
ENSBTAG00000021818	-0.643602	0.0200629	1.6976067
ENSBTAG00000021819	0.3157659	0.3560024	0.448547
ENSBTAG00000021820	-0.273015	0.290427	0.5369631
ENSBTAG00000021822	-0.023833	0.941453	0.0262014
ENSBTAG00000021823	-0.707672	0.0058498	2.2328559
ENSBTAG00000021827	NA	NA	NA
ENSBTAG00000021829	-0.425647	0.3383301	0.4706593
ENSBTAG00000021830	-0.099438	0.7017998	0.1537868
ENSBTAG00000021832	-0.511667	0.1203932	0.9193981
ENSBTAG00000021835	0.2475123	0.5212096	0.2829876
ENSBTAG00000021836	-0.026744	0.9547683	0.020102
ENSBTAG00000021837	NA	NA	NA
ENSBTAG00000021841	-0.368197	0.188764	0.7240809
ENSBTAG00000021842	NA	NA	NA
ENSBTAG00000021843	-0.491952	0.5385623	0.268764
ENSBTAG00000021845	-0.239505	0.3546116	0.4502471
ENSBTAG00000021846	NA	NA	NA
ENSBTAG00000021849	0.0728078	0.8678274	0.0615666
ENSBTAG00000021850	0.2805846	0.5710071	0.2433585
ENSBTAG00000021851	-0.42546	0.1377853	0.860797
ENSBTAG00000021853	-0.29215	0.3227516	0.4911317
ENSBTAG00000021855	NA	NA	NA
ENSBTAG00000021856	0.2317382	0.4852037	0.3140759
ENSBTAG00000021857	-0.077064	0.7918154	0.101376
ENSBTAG00000021859	-0.120154	0.6981994	0.1560205
ENSBTAG00000021861	0.0247745	0.9373218	0.0281113
ENSBTAG00000021862	0.61711	0.2033132	0.6918344
ENSBTAG00000021864	0.0193254	0.9583643	0.0184694
ENSBTAG00000021865	-0.06161	0.8188821	0.0867786
ENSBTAG00000021867	NA	NA	NA
ENSBTAG00000021869	-0.026858	0.93376	0.0297647
ENSBTAG00000021870	-0.090384	0.7881264	0.1034041
ENSBTAG00000021872	0.1169037	0.7951802	0.0995344
ENSBTAG00000021874	NA	NA	NA
ENSBTAG00000021875	-0.469818	0.1865555	0.7291919
ENSBTAG00000021876	NA	NA	NA
ENSBTAG00000021879	0.1926555	0.4790154	0.3196505
ENSBTAG00000021880	0.344513	0.1695807	0.7706236
ENSBTAG00000021883	0.0965155	0.7336762	0.1344955
ENSBTAG00000021884	0.3298645	0.2797101	0.5532919
ENSBTAG00000021886	NA	NA	NA
ENSBTAG00000021887	NA	NA	NA

ENSBTAG00000021893	0.5178712	0.2366985	0.6258046
ENSBTAG00000021894	-0.125152	0.7132702	0.1467459
ENSBTAG00000021896	0.1361076	0.6574447	0.1821407
ENSBTAG00000021897	0.1488177	0.7219129	0.1415152
ENSBTAG00000021899	0.7029964	0.1019283	0.9917052
ENSBTAG00000021900	-0.446684	0.1053358	0.977424
ENSBTAG00000021901	-0.406068	0.3685633	0.433488
ENSBTAG00000021902	-0.144048	0.7150038	0.1456917
ENSBTAG00000021903	NA	NA	NA
ENSBTAG00000021904	0.0551395	0.8952134	0.0480734
ENSBTAG00000021905	NA	NA	NA
ENSBTAG00000021906	NA	NA	NA
ENSBTAG00000021910	-0.159306	0.542499	0.2656011
ENSBTAG00000021911	0.7210472	0.0099809	2.0008284
ENSBTAG00000021912	0.4076867	0.244343	0.6120001
ENSBTAG00000021913	NA	NA	NA
ENSBTAG00000021916	NA	NA	NA
ENSBTAG00000021918	0.1052149	0.6905279	0.1608188
ENSBTAG00000021919	0.3689549	0.2905022	0.5368505
ENSBTAG00000021920	-0.26196	0.4023894	0.3953534
ENSBTAG00000021921	-0.176307	0.475348	0.3229884
ENSBTAG00000021922	0.0839624	0.7968435	0.0986269
ENSBTAG00000021923	NA	NA	NA
ENSBTAG00000021924	NA	NA	NA
ENSBTAG00000021927	0.1743995	0.6137127	0.2120349
ENSBTAG00000021928	NA	NA	NA
ENSBTAG00000021931	0.1909805	0.5619827	0.250277
ENSBTAG00000021932	0.1059824	0.7434589	0.128743
ENSBTAG00000021933	NA	NA	NA
ENSBTAG00000021934	-0.074277	0.8406701	0.0753744
ENSBTAG00000021935	0.0639947	0.818968	0.0867331
ENSBTAG00000021938	0.1937194	0.6999719	0.1549194
ENSBTAG00000021939	0.2121911	0.4428326	0.3537604
ENSBTAG00000021940	-0.019135	0.9499643	0.0222927
ENSBTAG00000021941	NA	NA	NA
ENSBTAG00000021942	0.8745194	0.052417	1.2805277
ENSBTAG00000021943	-0.051998	0.8652689	0.0628489
ENSBTAG00000021944	-0.182519	0.4745194	0.323746
ENSBTAG00000021945	0.5007355	0.0672189	1.1725089
ENSBTAG00000021948	0.2629132	0.3752401	0.4256907
ENSBTAG00000021949	NA	NA	NA
ENSBTAG00000021951	-0.166831	0.5059519	0.2958908
ENSBTAG00000021953	0.4174187	0.290414	0.5369825
ENSBTAG00000021955	0.0289239	0.9132961	0.0393884
ENSBTAG00000021956	0.5025978	0.1051539	0.9781747
ENSBTAG00000021957	0.6527518	0.0742953	1.1290386
ENSBTAG00000021958	NA	NA	NA
ENSBTAG00000021959	NA	NA	NA
ENSBTAG00000021960	0.0794367	0.8017152	0.0959799
ENSBTAG00000021961	0.0468773	0.8857831	0.0526726
ENSBTAG00000021962	-0.308777	0.2393505	0.6209658
ENSBTAG00000021963	-0.046315	0.8707459	0.0601086
ENSBTAG00000021964	NA	NA	NA
ENSBTAG00000021965	-0.34313	0.1758032	0.7549731
ENSBTAG00000021967	0.4387094	0.1070123	0.9705662
ENSBTAG00000021968	0.1924161	0.6231098	0.2054355
ENSBTAG00000021969	-0.457991	0.1750559	0.7568232
ENSBTAG00000021970	-1.348971	0.0014873	2.8275932

ENSBTAG00000021971	NA	NA	NA
ENSBTAG00000021972	NA	NA	NA
ENSBTAG00000021974	0.163971	0.5811139	0.2357387
ENSBTAG00000021975	-0.206968	0.4051987	0.3923319
ENSBTAG00000021976	-0.3281	0.2252014	0.647429
ENSBTAG00000021977	0.246347	0.4833388	0.3157484
ENSBTAG00000021978	-0.391551	0.2887503	0.5394775
ENSBTAG00000021979	0.0546237	0.830575	0.0806211
ENSBTAG00000021980	0.4438453	0.3982452	0.3998495
ENSBTAG00000021981	0.5769566	0.1792065	0.7466462
ENSBTAG00000021984	NA	NA	NA
ENSBTAG00000021986	-1.061568	0.0031683	2.49917
ENSBTAG00000021988	0.0130865	0.9673783	0.0144037
ENSBTAG00000021991	-0.143699	0.7921632	0.1011854
ENSBTAG00000021992	-0.438	0.1105458	0.9564578
ENSBTAG00000021993	NA	NA	NA
ENSBTAG00000021994	NA	NA	NA
ENSBTAG00000021995	NA	NA	NA
ENSBTAG00000021996	0.0240927	0.9470055	0.0236475
ENSBTAG00000021997	0.0391209	0.8835054	0.0537908
ENSBTAG00000021999	0.8347914	0.0046778	2.3299615
ENSBTAG00000022000	-0.298368	0.5938347	0.2263344
ENSBTAG00000022003	-0.161668	0.6013106	0.2209012
ENSBTAG00000022004	-0.181056	0.4619857	0.3353715
ENSBTAG00000022006	0.7122956	0.1081627	0.9659226
ENSBTAG00000022007	-0.087599	0.8124845	0.0901849
ENSBTAG00000022009	0.1825341	0.488344	0.3112742
ENSBTAG00000022013	0.1271725	0.6772019	0.1692818
ENSBTAG00000022020	-0.135634	0.6164211	0.2101225
ENSBTAG00000022022	0.0844392	0.744088	0.1283757
ENSBTAG00000022027	0.2841533	0.4564671	0.3405905
ENSBTAG00000022028	NA	NA	NA
ENSBTAG00000022032	0.1305764	0.6351221	0.1971428
ENSBTAG00000022036	0.1393105	0.7632176	0.1173517
ENSBTAG00000022039	NA	NA	NA
ENSBTAG00000022044	0.1008357	0.7550596	0.1220188
ENSBTAG00000022058	1.1994935	5.99E-06	5.2226856
ENSBTAG00000022067	NA	NA	NA
ENSBTAG00000022069	0.0003207	0.9993364	0.0002883
ENSBTAG00000022083	NA	NA	NA
ENSBTAG00000022109	-0.332447	0.1712716	0.7663145
ENSBTAG00000022114	-0.651209	0.0151634	1.8192042
ENSBTAG00000022120	-0.411384	0.4531636	0.343745
ENSBTAG00000022142	NA	NA	NA
ENSBTAG00000022147	-0.132669	0.7783116	0.1088465
ENSBTAG00000022150	-0.123632	0.7728271	0.1119176
ENSBTAG00000022155	0.1254785	0.6233311	0.2052812
ENSBTAG00000022158	0.0366463	0.8871179	0.0520186
ENSBTAG00000022160	-0.247081	0.3455785	0.4614533
ENSBTAG00000022161	NA	NA	NA
ENSBTAG00000022167	-0.324253	0.3137327	0.5034402
ENSBTAG00000022169	-0.026273	0.9188198	0.0367697
ENSBTAG00000022185	0.5472631	0.1252565	0.9021996
ENSBTAG00000022188	0.1689103	0.5335353	0.2728368
ENSBTAG00000022204	-0.266403	0.4964143	0.3041557
ENSBTAG00000022209	NA	NA	NA
ENSBTAG00000022227	0.5111361	0.1274646	0.8946105
ENSBTAG00000022238	0.2645249	0.4457784	0.350881

ENSBTAG00000022242	0.3938024	0.1620308	0.7904025
ENSBTAG00000022244	-0.222999	0.4183697	0.3784398
ENSBTAG00000022255	-0.334836	0.2670975	0.5733301
ENSBTAG00000022275	NA	NA	NA
ENSBTAG00000022278	0.7047368	0.0098888	2.004855
ENSBTAG00000022288	NA	NA	NA
ENSBTAG00000022292	-0.055361	0.8755286	0.0577296
ENSBTAG00000022293	-0.171191	0.6728789	0.1720631
ENSBTAG00000022294	NA	NA	NA
ENSBTAG00000022314	-0.296521	0.3637964	0.4391416
ENSBTAG00000022329	NA	NA	NA
ENSBTAG00000022360	0.9846232	0.0126116	1.8992302
ENSBTAG00000022373	NA	NA	NA
ENSBTAG00000022379	NA	NA	NA
ENSBTAG00000022381	NA	NA	NA
ENSBTAG00000022382	-0.081587	0.845561	0.0728551
ENSBTAG00000022394	NA	NA	NA
ENSBTAG00000022395	NA	NA	NA
ENSBTAG00000022396	NA	NA	NA
ENSBTAG00000022427	NA	NA	NA
ENSBTAG00000022449	-0.114036	0.8485058	0.0713452
ENSBTAG00000022450	-0.430241	0.1603	0.7950664
ENSBTAG00000022461	0.111335	0.681244	0.1666973
ENSBTAG00000022471	NA	NA	NA
ENSBTAG00000022489	0.2766473	0.4137131	0.3833007
ENSBTAG00000022498	NA	NA	NA
ENSBTAG00000022501	NA	NA	NA
ENSBTAG00000022504	NA	NA	NA
ENSBTAG00000022509	NA	NA	NA
ENSBTAG00000022514	1.0774616	0.0179233	1.7465826
ENSBTAG00000022520	0.0618983	0.8649908	0.0629885
ENSBTAG00000022530	0.2675882	0.4906113	0.3092624
ENSBTAG00000022535	NA	NA	NA
ENSBTAG00000022564	-0.747763	0.0138476	1.8586247
ENSBTAG00000022570	-0.372751	0.5395047	0.2680048
ENSBTAG00000022571	NA	NA	NA
ENSBTAG00000022575	0.2081122	0.4916524	0.3083418
ENSBTAG00000022580	-0.134648	0.830822	0.080492
ENSBTAG00000022583	NA	NA	NA
ENSBTAG00000022588	NA	NA	NA
ENSBTAG00000022590	-1.19984	0.0151058	1.8208563
ENSBTAG00000022598	-0.410625	0.3961385	0.4021529
ENSBTAG00000022613	-0.007143	0.982719	0.0075707
ENSBTAG00000022622	-1.334806	0.0221234	1.6551474
ENSBTAG00000022632	0.0424374	0.8985468	0.0464593
ENSBTAG00000022635	0.1869277	0.4759068	0.3224781
ENSBTAG00000022656	0.1153111	0.7585348	0.1200245
ENSBTAG00000022671	NA	NA	NA
ENSBTAG00000022681	0.8820858	0.1926508	0.7152291
ENSBTAG00000022684	NA	NA	NA
ENSBTAG00000022689	-0.484588	0.2910387	0.5360492
ENSBTAG00000022690	NA	NA	NA
ENSBTAG00000022699	-0.509395	0.0727904	1.1379258
ENSBTAG00000022714	-8.006781	1.21E-66	65.918152
ENSBTAG00000022715	NA	NA	NA
ENSBTAG00000022721	0.4512152	0.2170345	0.6634712
ENSBTAG00000022731	0.0611591	0.8139625	0.0893956
ENSBTAG00000022733	0.2799131	0.7921935	0.1011687

ENSBTAG00000022741	NA	NA	NA
ENSBTAG00000022751	-0.464101	0.2875703	0.541256
ENSBTAG00000022767	NA	NA	NA
ENSBTAG00000022775	NA	NA	NA
ENSBTAG00000022777	-0.01614	0.9495803	0.0224683
ENSBTAG00000022779	NA	NA	NA
ENSBTAG00000022783	NA	NA	NA
ENSBTAG00000022799	-0.284758	0.3614954	0.4418973
ENSBTAG00000022801	0.2596058	0.4479668	0.3487542
ENSBTAG00000022807	NA	NA	NA
ENSBTAG00000022808	-0.46972	0.1089081	0.9629399
ENSBTAG00000022813	NA	NA	NA
ENSBTAG00000022819	NA	NA	NA
ENSBTAG00000022825	-0.122446	0.6650957	0.1771159
ENSBTAG00000022829	NA	NA	NA
ENSBTAG00000022837	0.6553238	0.2719254	0.5655502
ENSBTAG00000022847	0.0429867	0.8632801	0.0638483
ENSBTAG00000022886	0.5554781	0.1745772	0.7580125
ENSBTAG00000022887	0.0341953	0.8978663	0.0467883
ENSBTAG00000022890	-0.388486	0.3379384	0.4711625
ENSBTAG00000022893	NA	NA	NA
ENSBTAG00000022895	-0.560911	0.224019	0.6497152
ENSBTAG00000022902	0.2879314	0.2960838	0.5285854
ENSBTAG00000022915	NA	NA	NA
ENSBTAG00000022917	NA	NA	NA
ENSBTAG00000022920	-0.394124	0.1518147	0.8186862
ENSBTAG00000022922	-0.130476	0.801143	0.09629
ENSBTAG00000022927	NA	NA	NA
ENSBTAG00000022931	NA	NA	NA
ENSBTAG00000022937	NA	NA	NA
ENSBTAG00000022942	NA	NA	NA
ENSBTAG00000022954	NA	NA	NA
ENSBTAG00000022960	NA	NA	NA
ENSBTAG00000022962	NA	NA	NA
ENSBTAG00000022971	NA	NA	NA
ENSBTAG00000022986	NA	NA	NA
ENSBTAG00000022989	-0.896246	0.1103076	0.9573945
ENSBTAG00000022991	-0.410574	0.1705814	0.7680684
ENSBTAG00000023002	-0.40582	0.1062784	0.9735551
ENSBTAG00000023007	NA	NA	NA
ENSBTAG00000023018	-0.045179	0.8615787	0.064705
ENSBTAG00000023023	NA	NA	NA
ENSBTAG00000023026	NA	NA	NA
ENSBTAG00000023028	0.2762787	0.4230406	0.373618
ENSBTAG00000023032	NA	NA	NA
ENSBTAG00000023039	-0.185491	0.5096773	0.2927047
ENSBTAG00000023054	0.0191465	0.9588821	0.0182348
ENSBTAG00000023064	NA	NA	NA
ENSBTAG00000023073	0.5699665	0.1935243	0.7132644
ENSBTAG00000023074	-0.095337	0.7937045	0.1003411
ENSBTAG00000023106	-0.032272	0.9395506	0.0270798
ENSBTAG00000023144	-0.137519	0.6816455	0.1664414
ENSBTAG00000023146	0.1642111	0.7324446	0.1352252
ENSBTAG00000023147	-0.176345	0.5149237	0.2882571
ENSBTAG00000023157	NA	NA	NA
ENSBTAG00000023169	0.2050011	0.6382404	0.1950157
ENSBTAG00000023172	-0.008194	0.9752338	0.0108912
ENSBTAG00000023177	1.4089017	0.1178046	0.9288377

ENSBTAG00000023179	-0.253645	0.410472	0.3867165
ENSBTAG00000023182	NA	NA	NA
ENSBTAG00000023186	NA	NA	NA
ENSBTAG00000023216	NA	NA	NA
ENSBTAG00000023218	-0.20994	0.4467089	0.3499754
ENSBTAG00000023259	-0.238882	0.3539718	0.4510313
ENSBTAG00000023270	-0.508787	0.3365377	0.4729663
ENSBTAG00000023274	0.0285939	0.9107389	0.0406061
ENSBTAG00000023279	0.435105	0.6693568	0.1743423
ENSBTAG00000023283	0.1167278	0.8213738	0.0854592
ENSBTAG00000023289	0.5446106	0.2126277	0.6723801
ENSBTAG00000023309	NA	NA	NA
ENSBTAG00000023333	0.0851669	0.8053621	0.0940088
ENSBTAG00000023338	0.6476278	0.2057134	0.6867375
ENSBTAG00000023343	0.4477374	0.1253019	0.9020423
ENSBTAG00000023365	NA	NA	NA
ENSBTAG00000023372	NA	NA	NA
ENSBTAG00000023374	NA	NA	NA
ENSBTAG00000023375	-0.096588	0.7411636	0.1300859
ENSBTAG00000023377	-0.488572	0.0566766	1.246596
ENSBTAG00000023384	-0.108844	0.7721192	0.1123157
ENSBTAG00000023398	NA	NA	NA
ENSBTAG00000023411	NA	NA	NA
ENSBTAG00000023415	0.2818514	0.536047	0.2707971
ENSBTAG00000023416	-0.643293	0.0195377	1.709126
ENSBTAG00000023417	0.3421296	0.2740849	0.5621148
ENSBTAG00000023419	NA	NA	NA
ENSBTAG00000023426	0.0598927	0.9160266	0.0380919
ENSBTAG00000023429	-0.736475	0.0740922	1.1302274
ENSBTAG00000023431	NA	NA	NA
ENSBTAG00000023445	-0.700831	0.0892136	1.0495691
ENSBTAG00000023452	-0.26318	0.4837976	0.3153363
ENSBTAG00000023453	NA	NA	NA
ENSBTAG00000023462	0.0971797	0.6949077	0.1580729
ENSBTAG00000023464	-0.285113	0.3315552	0.4794441
ENSBTAG00000023471	NA	NA	NA
ENSBTAG00000023472	0.2769061	0.4083264	0.3889926
ENSBTAG00000023487	0.0619888	0.82013	0.0861173
ENSBTAG00000023511	NA	NA	NA
ENSBTAG00000023513	-0.011739	0.9649489	0.0154957
ENSBTAG00000023523	-0.375701	0.162096	0.7902277
ENSBTAG00000023529	NA	NA	NA
ENSBTAG00000023541	NA	NA	NA
ENSBTAG00000023549	NA	NA	NA
ENSBTAG00000023551	-0.404269	0.3305306	0.4807884
ENSBTAG00000023562	NA	NA	NA
ENSBTAG00000023563	NA	NA	NA
ENSBTAG00000023593	NA	NA	NA
ENSBTAG00000023600	0.0696866	0.8724544	0.0592573
ENSBTAG00000023601	-0.048831	0.8950997	0.0481286
ENSBTAG00000023607	0.0743994	0.8053366	0.0940226
ENSBTAG00000023610	0.1902005	0.5359579	0.2708693
ENSBTAG00000023611	NA	NA	NA
ENSBTAG00000023614	0.259815	0.4729411	0.3251929
ENSBTAG00000023617	NA	NA	NA
ENSBTAG00000023628	0.2711353	0.4588919	0.3382896
ENSBTAG00000023629	NA	NA	NA
ENSBTAG00000023632	NA	NA	NA

ENSBTAG00000023635	NA	NA	NA
ENSBTAG00000023648	-1.233004	0.0262494	1.5808806
ENSBTAG00000023652	0.0837818	0.8115305	0.0906952
ENSBTAG00000023659	3.5698277	3.85E-07	6.4145697
ENSBTAG00000023675	-0.33749	0.1847097	0.7335103
ENSBTAG00000023697	NA	NA	NA
ENSBTAG00000023726	NA	NA	NA
ENSBTAG00000023730	NA	NA	NA
ENSBTAG00000023731	NA	NA	NA
ENSBTAG00000023734	NA	NA	NA
ENSBTAG00000023736	-0.239686	0.6503432	0.1868574
ENSBTAG00000023744	0.1232661	0.6290926	0.2012854
ENSBTAG00000023745	-0.077658	0.8391763	0.0761468
ENSBTAG00000023752	NA	NA	NA
ENSBTAG00000023765	NA	NA	NA
ENSBTAG00000023776	-0.395216	0.3402444	0.468209
ENSBTAG00000023780	-0.235612	0.649275	0.1875713
ENSBTAG00000023784	NA	NA	NA
ENSBTAG00000023787	-0.282322	0.7515419	0.1240468
ENSBTAG00000023788	0.2011528	0.6511965	0.186288
ENSBTAG00000023792	-0.980338	0.041542	1.3815127
ENSBTAG00000023795	0.484443	0.1599046	0.796139
ENSBTAG00000023797	NA	NA	NA
ENSBTAG00000023806	-0.491698	0.0584062	1.2335409
ENSBTAG00000023814	-1.428121	0.0011251	2.9488212
ENSBTAG00000023823	0.5308435	0.303338	0.5180732
ENSBTAG00000023831	0.0831175	0.788977	0.1029357
ENSBTAG00000023832	0.1491414	0.7708073	0.1130542
ENSBTAG00000023840	0.3727641	0.2807496	0.5516808
ENSBTAG00000023843	-0.348816	0.4425433	0.3540443
ENSBTAG00000023845	0.1878823	0.5971436	0.2239212
ENSBTAG00000023846	0.3634733	0.3018041	0.5202749
ENSBTAG00000023847	-0.200351	0.546465	0.2624376
ENSBTAG00000023851	NA	NA	NA
ENSBTAG00000023867	0.231776	0.4239321	0.3727037
ENSBTAG00000023885	-0.374821	0.462044	0.3353167
ENSBTAG00000023891	-1.054581	0.0003169	3.4990517
ENSBTAG00000023907	0.5003463	0.0746769	1.1268139
ENSBTAG00000023912	NA	NA	NA
ENSBTAG00000023918	NA	NA	NA
ENSBTAG00000023920	NA	NA	NA
ENSBTAG00000023928	0.2965337	0.305732	0.5146591
ENSBTAG00000023929	0.0701356	0.8270732	0.0824561
ENSBTAG00000023933	0.191145	0.5022001	0.2991232
ENSBTAG00000023938	NA	NA	NA
ENSBTAG00000023939	NA	NA	NA
ENSBTAG00000023941	NA	NA	NA
ENSBTAG00000023947	0.2534093	0.4139029	0.3831015
ENSBTAG00000023954	NA	NA	NA
ENSBTAG00000023955	NA	NA	NA
ENSBTAG00000023963	0.25565	0.5129992	0.2898833
ENSBTAG00000023970	-0.647548	0.3456828	0.4613222
ENSBTAG00000023976	0.0498119	0.909907	0.041003
ENSBTAG00000023978	NA	NA	NA
ENSBTAG00000023986	NA	NA	NA
ENSBTAG00000023989	0.1060972	0.7420468	0.1295687
ENSBTAG00000023997	-0.03759	0.9259019	0.033435
ENSBTAG00000023999	NA	NA	NA

ENSBTAG00000024000	-0.035973	0.9415702	0.0261473
ENSBTAG00000024015	0.0127805	0.9604808	0.0175113
ENSBTAG00000024021	0.4230396	0.4208548	0.3758677
ENSBTAG00000024027	-0.369263	0.1943995	0.711305
ENSBTAG00000024042	-0.111694	0.7546471	0.1222561
ENSBTAG00000024044	NA	NA	NA
ENSBTAG00000024058	NA	NA	NA
ENSBTAG00000024061	NA	NA	NA
ENSBTAG00000024081	0.0106032	0.9675797	0.0143132
ENSBTAG00000024086	NA	NA	NA
ENSBTAG00000024091	-0.489437	0.2835381	0.5473886
ENSBTAG00000024095	-0.144922	0.5700488	0.244088
ENSBTAG00000024096	-0.04908	0.8992009	0.0461433
ENSBTAG00000024097	-0.132824	0.6160158	0.2104081
ENSBTAG00000024105	0.2195904	0.5794372	0.2369936
ENSBTAG00000024107	0.1058715	0.7730671	0.1117828
ENSBTAG00000024115	-1.245422	0.0167793	1.7752254
ENSBTAG00000024125	NA	NA	NA
ENSBTAG00000024132	NA	NA	NA
ENSBTAG00000024137	0.2898076	0.5377879	0.269389
ENSBTAG00000024144	NA	NA	NA
ENSBTAG00000024153	NA	NA	NA
ENSBTAG00000024157	0.2276164	0.6562981	0.1828989
ENSBTAG00000024162	NA	NA	NA
ENSBTAG00000024169	NA	NA	NA
ENSBTAG00000024175	NA	NA	NA
ENSBTAG00000024176	0.245503	0.6421169	0.1923859
ENSBTAG00000024177	NA	NA	NA
ENSBTAG00000024178	NA	NA	NA
ENSBTAG00000024179	-0.77138	0.1226394	0.91137
ENSBTAG00000024180	NA	NA	NA
ENSBTAG00000024182	0.0228742	0.9789929	0.0092205
ENSBTAG00000024183	NA	NA	NA
ENSBTAG00000024184	0.3273741	0.5054399	0.2963305
ENSBTAG00000024185	0.4226482	0.1955993	0.7086327
ENSBTAG00000024186	0.2895517	0.5257127	0.2792516
ENSBTAG00000024187	-0.399678	0.4112116	0.3859346
ENSBTAG00000024188	-0.424073	0.3813307	0.4186982
ENSBTAG00000024199	-0.38748	0.1341524	0.8724015
ENSBTAG00000024204	NA	NA	NA
ENSBTAG00000024210	NA	NA	NA
ENSBTAG00000024231	NA	NA	NA
ENSBTAG00000024233	NA	NA	NA
ENSBTAG00000024240	0.0553739	0.8374694	0.077031
ENSBTAG00000024269	0.2855374	0.3142895	0.5026701
ENSBTAG00000024272	-1.299543	0.264777	0.5771198
ENSBTAG00000024275	-0.146399	0.6330584	0.1985562
ENSBTAG00000024288	NA	NA	NA
ENSBTAG00000024291	NA	NA	NA
ENSBTAG00000024311	-0.670287	0.157185	0.8035889
ENSBTAG00000024318	NA	NA	NA
ENSBTAG00000024340	1.3712353	0.0028177	2.550102
ENSBTAG00000024341	2.4974873	0.057312	1.2417543
ENSBTAG00000024361	NA	NA	NA
ENSBTAG00000024378	-0.139513	0.5907607	0.2285884
ENSBTAG00000024379	-0.421461	0.3747767	0.4262275
ENSBTAG00000024381	0.0527188	0.8719874	0.0594898
ENSBTAG00000024387	-0.327397	0.1963664	0.7069328

ENSBTAG00000024394	NA	NA	NA
ENSBTAG00000024406	NA	NA	NA
ENSBTAG00000024420	NA	NA	NA
ENSBTAG00000024426	-0.2494	0.4138278	0.3831804
ENSBTAG00000024431	-0.12105	0.6885759	0.1620482
ENSBTAG00000024443	0.0456934	0.8867585	0.0521947
ENSBTAG00000024449	-0.238139	0.6716123	0.1728814
ENSBTAG00000024450	0.0132843	0.961681	0.016969
ENSBTAG00000024453	NA	NA	NA
ENSBTAG00000024470	NA	NA	NA
ENSBTAG00000024476	-0.250114	0.5212643	0.2829421
ENSBTAG00000024482	-0.368872	0.1870576	0.7280246
ENSBTAG00000024485	-0.044159	0.8737282	0.0586236
ENSBTAG00000024490	NA	NA	NA
ENSBTAG00000024492	0.7327608	0.138123	0.8597339
ENSBTAG00000024493	-0.75233	0.0075088	2.1244295
ENSBTAG00000024496	NA	NA	NA
ENSBTAG00000024503	NA	NA	NA
ENSBTAG00000024507	NA	NA	NA
ENSBTAG00000024509	-0.236415	0.5749391	0.2403781
ENSBTAG00000024520	-0.136562	0.7015057	0.1539688
ENSBTAG00000024526	-0.002366	0.9956628	0.0018877
ENSBTAG00000024534	-0.025769	0.950139	0.0222129
ENSBTAG00000024539	-0.695152	0.0648044	1.1883956
ENSBTAG00000024542	-0.166956	0.533656	0.2727386
ENSBTAG00000024545	NA	NA	NA
ENSBTAG00000024549	0.030011	0.9033822	0.0441285
ENSBTAG00000024555	-0.514645	0.0788798	1.1030344
ENSBTAG00000024560	NA	NA	NA
ENSBTAG00000024561	-0.016226	0.9477761	0.0232943
ENSBTAG00000024582	NA	NA	NA
ENSBTAG00000024585	-0.75007	0.0635576	1.1968323
ENSBTAG00000024595	-0.159268	0.5353838	0.2713348
ENSBTAG00000024603	1.236179	0.0053066	2.275185
ENSBTAG00000024604	NA	NA	NA
ENSBTAG00000024605	-0.322653	0.285422	0.5445126
ENSBTAG00000024608	-0.215583	0.6409143	0.1932
ENSBTAG00000024623	NA	NA	NA
ENSBTAG00000024632	NA	NA	NA
ENSBTAG00000024633	-0.018712	0.9579955	0.0186365
ENSBTAG00000024641	-0.105131	0.7536384	0.122837
ENSBTAG00000024643	NA	NA	NA
ENSBTAG00000024647	NA	NA	NA
ENSBTAG00000024648	0.1047092	0.8431627	0.0740886
ENSBTAG00000024657	0.1764107	0.5700948	0.2440529
ENSBTAG00000024662	NA	NA	NA
ENSBTAG00000024663	-0.871886	0.2058058	0.6865424
ENSBTAG00000024675	NA	NA	NA
ENSBTAG00000024688	-0.032424	0.8998364	0.0458364
ENSBTAG00000024701	0.1608504	0.5712401	0.2431813
ENSBTAG00000024708	0.3188299	0.2463134	0.608512
ENSBTAG00000024715	-0.428705	0.3479166	0.4585248
ENSBTAG00000024723	0.042632	0.8661901	0.0623868
ENSBTAG00000024726	NA	NA	NA
ENSBTAG00000024751	NA	NA	NA
ENSBTAG00000024756	0.1252985	0.7142567	0.1461457
ENSBTAG00000024771	NA	NA	NA
ENSBTAG00000024772	-0.489767	0.2830139	0.5481922

ENSBTAG00000024781	-0.1255	0.6433833	0.1915302
ENSBTAG00000024787	-0.218322	0.5139458	0.2890827
ENSBTAG00000024788	NA	NA	NA
ENSBTAG00000024798	NA	NA	NA
ENSBTAG00000024801	0.0336261	0.9262992	0.0332487
ENSBTAG00000024803	-0.37221	0.1610308	0.7930909
ENSBTAG00000024814	NA	NA	NA
ENSBTAG00000024815	-0.411464	0.1066463	0.9720543
ENSBTAG00000024822	-0.117671	0.6378305	0.1952947
ENSBTAG00000024826	-1.06663	0.000374	3.4271179
ENSBTAG00000024839	NA	NA	NA
ENSBTAG00000024851	NA	NA	NA
ENSBTAG00000024852	NA	NA	NA
ENSBTAG00000024869	NA	NA	NA
ENSBTAG00000024874	NA	NA	NA
ENSBTAG00000024878	NA	NA	NA
ENSBTAG00000024884	0.131815	0.6902412	0.1609991
ENSBTAG00000024888	-0.113389	0.6869665	0.1630644
ENSBTAG00000024889	-0.089695	0.716121	0.1450136
ENSBTAG00000024901	NA	NA	NA
ENSBTAG00000024903	NA	NA	NA
ENSBTAG00000024904	NA	NA	NA
ENSBTAG00000024909	-0.273892	0.2812398	0.5509232
ENSBTAG00000024918	0.0443959	0.8659082	0.0625282
ENSBTAG00000024928	-0.014823	0.9663127	0.0148823
ENSBTAG00000024929	0.7905008	0.0072217	2.1413576
ENSBTAG00000024932	NA	NA	NA
ENSBTAG00000024934	NA	NA	NA
ENSBTAG00000024942	NA	NA	NA
ENSBTAG00000024947	NA	NA	NA
ENSBTAG00000024950	0.7733579	0.0867216	1.0618728
ENSBTAG00000024957	NA	NA	NA
ENSBTAG00000024958	-0.4743	0.1541188	0.8121444
ENSBTAG00000024960	-1.314204	0.0025146	2.5995231
ENSBTAG00000024974	-0.762904	0.1153689	0.9379112
ENSBTAG00000024975	NA	NA	NA
ENSBTAG00000024980	0.0799094	0.8074625	0.0928776
ENSBTAG00000024983	-0.320214	0.4890231	0.3106706
ENSBTAG00000024984	-0.869041	0.220021	0.6575359
ENSBTAG00000024991	-0.688447	0.1827152	0.7382254
ENSBTAG00000025001	-0.096037	0.7089657	0.1493748
ENSBTAG00000025003	0.0519156	0.8507618	0.070192
ENSBTAG00000025005	-0.869774	0.0008282	3.0818564
ENSBTAG00000025021	NA	NA	NA
ENSBTAG00000025023	0.2211273	0.6570054	0.1824311
ENSBTAG00000025028	-0.306418	0.2251038	0.6476171
ENSBTAG00000025029	0.0231078	0.938074	0.0277629
ENSBTAG00000025035	NA	NA	NA
ENSBTAG00000025046	-0.181942	0.4682309	0.3295399
ENSBTAG00000025062	NA	NA	NA
ENSBTAG00000025071	NA	NA	NA
ENSBTAG00000025078	NA	NA	NA
ENSBTAG00000025088	NA	NA	NA
ENSBTAG00000025099	0.0186758	0.9480182	0.0231833
ENSBTAG00000025101	NA	NA	NA
ENSBTAG00000025121	-0.091703	0.7264136	0.138816
ENSBTAG00000025124	NA	NA	NA
ENSBTAG00000025126	NA	NA	NA

ENSBTAG00000025129	0.4268126	0.3144744	0.5024147
ENSBTAG00000025130	-0.162219	0.6474897	0.1887671
ENSBTAG00000025136	-0.486813	0.0529277	1.2763168
ENSBTAG00000025140	NA	NA	NA
ENSBTAG00000025146	-0.080027	0.8182694	0.0871037
ENSBTAG00000025148	0.5688686	0.2027578	0.6930224
ENSBTAG00000025149	0.2287732	0.5223832	0.2820108
ENSBTAG00000025161	0.2468488	0.3503761	0.4554655
ENSBTAG00000025167	NA	NA	NA
ENSBTAG00000025181	-0.075166	0.8433516	0.0739913
ENSBTAG00000025182	NA	NA	NA
ENSBTAG00000025191	-0.197545	0.4312629	0.3652579
ENSBTAG00000025192	NA	NA	NA
ENSBTAG00000025200	NA	NA	NA
ENSBTAG00000025210	0.6109297	0.0213616	1.6703667
ENSBTAG00000025211	-0.230697	0.7316478	0.1356979
ENSBTAG00000025212	-0.009267	0.9748407	0.0110664
ENSBTAG00000025213	NA	NA	NA
ENSBTAG00000025219	NA	NA	NA
ENSBTAG00000025221	0.0165802	0.9747128	0.0111233
ENSBTAG00000025226	NA	NA	NA
ENSBTAG00000025233	NA	NA	NA
ENSBTAG00000025242	NA	NA	NA
ENSBTAG00000025246	NA	NA	NA
ENSBTAG00000025250	NA	NA	NA
ENSBTAG00000025257	NA	NA	NA
ENSBTAG00000025258	0.2441516	0.5719798	0.2426193
ENSBTAG00000025260	NA	NA	NA
ENSBTAG00000025263	-0.778288	0.005358	2.2709941
ENSBTAG00000025274	-0.287596	0.2841668	0.5464266
ENSBTAG00000025277	-0.183218	0.4743185	0.32393
ENSBTAG00000025280	-0.19004	0.5287481	0.2767512
ENSBTAG00000025288	NA	NA	NA
ENSBTAG00000025297	0.0257732	0.9300332	0.0315015
ENSBTAG00000025308	-0.593419	0.3386714	0.4702214
ENSBTAG00000025310	NA	NA	NA
ENSBTAG00000025311	-0.02333	0.9627692	0.0164778
ENSBTAG00000025313	-0.115438	0.6496985	0.1872882
ENSBTAG00000025317	NA	NA	NA
ENSBTAG00000025320	0.5889277	0.0337891	1.4712232
ENSBTAG00000025324	NA	NA	NA
ENSBTAG00000025329	0.8498164	0.2435205	0.6134645
ENSBTAG00000025337	NA	NA	NA
ENSBTAG00000025340	NA	NA	NA
ENSBTAG00000025345	-0.21573	0.6824594	0.1659232
ENSBTAG00000025358	0.1789903	0.5001395	0.3009089
ENSBTAG00000025372	0.9048741	0.0052109	2.2830848
ENSBTAG00000025385	-0.228995	0.4929285	0.307216
ENSBTAG00000025391	0.4695135	0.0743793	1.1285482
ENSBTAG00000025398	NA	NA	NA
ENSBTAG00000025400	0.0548104	0.857701	0.0666641
ENSBTAG00000025401	NA	NA	NA
ENSBTAG00000025402	0.4044122	0.3024478	0.5193496
ENSBTAG00000025403	0.3324806	0.3869693	0.4123235
ENSBTAG00000025405	NA	NA	NA
ENSBTAG00000025410	-0.319155	0.5338533	0.272578
ENSBTAG00000025413	0.1606723	0.5480619	0.2611704
ENSBTAG00000025424	-0.081809	0.843188	0.0740756

ENSBTAG00000025425	0.2343657	0.4806747	0.3181487
ENSBTAG00000025426	0.0278255	0.9115501	0.0402195
ENSBTAG00000025428	NA	NA	NA
ENSBTAG00000025434	0.7343861	0.0361203	1.4422486
ENSBTAG00000025441	0.2567259	0.4719098	0.326141
ENSBTAG00000025442	0.0322072	0.9720629	0.0123056
ENSBTAG00000025443	-0.172247	0.5132402	0.2896794
ENSBTAG00000025448	NA	NA	NA
ENSBTAG00000025450	-0.548476	0.0352443	1.4529112
ENSBTAG00000025452	0.5048971	0.1824146	0.7389404
ENSBTAG00000025458	0.4735218	0.0832031	1.0798604
ENSBTAG00000025462	1.2137327	0.0019456	2.7109409
ENSBTAG00000025471	NA	NA	NA
ENSBTAG00000025477	-0.218166	0.4298054	0.3667282
ENSBTAG00000025485	NA	NA	NA
ENSBTAG00000025494	1.0173436	0.028362	1.5472639
ENSBTAG00000025496	-0.654696	0.0802061	1.0957928
ENSBTAG00000025502	NA	NA	NA
ENSBTAG00000025516	-0.15822	0.6498683	0.1871746
ENSBTAG00000025522	NA	NA	NA
ENSBTAG00000025526	0.2051034	0.6071688	0.2166906
ENSBTAG00000025535	NA	NA	NA
ENSBTAG00000025540	-0.113886	0.6587173	0.1813009
ENSBTAG00000025550	NA	NA	NA
ENSBTAG00000025564	-0.083568	0.7387143	0.1315235
ENSBTAG00000025571	-0.072134	0.7673048	0.1150321
ENSBTAG00000025589	-0.375933	0.1765484	0.7531361
ENSBTAG00000025593	-0.279478	0.3151254	0.5015166
ENSBTAG00000025595	NA	NA	NA
ENSBTAG00000025597	NA	NA	NA
ENSBTAG00000025606	0.1953338	0.5362274	0.270651
ENSBTAG00000025612	0.1630599	0.5438253	0.2645406
ENSBTAG00000025617	-0.199594	0.4852351	0.3140478
ENSBTAG00000025621	NA	NA	NA
ENSBTAG00000025622	NA	NA	NA
ENSBTAG00000025632	0.0611008	0.8266375	0.0826849
ENSBTAG00000025634	NA	NA	NA
ENSBTAG00000025642	-0.088379	0.8450897	0.0730972
ENSBTAG00000025644	-0.094944	0.7709114	0.1129956
ENSBTAG00000025652	NA	NA	NA
ENSBTAG00000025659	NA	NA	NA
ENSBTAG00000025664	NA	NA	NA
ENSBTAG00000025666	NA	NA	NA
ENSBTAG00000025667	NA	NA	NA
ENSBTAG00000025669	-0.348988	0.2670258	0.5734467
ENSBTAG00000025718	0.3334211	0.4401122	0.3564366
ENSBTAG00000025720	NA	NA	NA
ENSBTAG00000025752	-0.246895	0.5398203	0.2677508
ENSBTAG00000025755	NA	NA	NA
ENSBTAG00000025756	-0.116991	0.7675284	0.1149055
ENSBTAG00000025760	NA	NA	NA
ENSBTAG00000025762	-0.439141	0.2078816	0.6821839
ENSBTAG00000025767	NA	NA	NA
ENSBTAG00000025775	NA	NA	NA
ENSBTAG00000025778	-0.431558	0.1297208	0.8869904
ENSBTAG00000025782	NA	NA	NA
ENSBTAG00000025788	-0.353664	0.303635	0.5176482
ENSBTAG00000025792	NA	NA	NA

ENSBTAG00000025803	-0.354959	0.1407517	0.8515463
ENSBTAG00000025809	0.0311448	0.9233795	0.0346198
ENSBTAG00000025814	NA	NA	NA
ENSBTAG00000025817	0.0883023	0.7431229	0.1289394
ENSBTAG00000025822	-0.426123	0.3319985	0.4788639
ENSBTAG00000025826	NA	NA	NA
ENSBTAG00000025830	-0.314153	0.3385304	0.4704023
ENSBTAG00000025837	NA	NA	NA
ENSBTAG00000025847	NA	NA	NA
ENSBTAG00000025848	-0.872351	0.0583475	1.233978
ENSBTAG00000025853	-0.307223	0.2529447	0.5969744
ENSBTAG00000025856	0.5513656	0.0484799	1.3144379
ENSBTAG00000025859	0.4468995	0.1863374	0.7297
ENSBTAG00000025868	0.2697552	0.2768601	0.5577397
ENSBTAG00000025893	NA	NA	NA
ENSBTAG00000025898	0.6382727	0.0406251	1.3912059
ENSBTAG00000025903	NA	NA	NA
ENSBTAG00000025929	0.5925682	0.0933062	1.0300894
ENSBTAG00000025931	0.1749578	0.5963756	0.2244802
ENSBTAG00000025942	-0.169566	0.6259335	0.2034718
ENSBTAG00000025964	-0.043741	0.8677844	0.0615882
ENSBTAG00000026003	NA	NA	NA
ENSBTAG00000026004	NA	NA	NA
ENSBTAG00000026008	-0.231787	0.4638592	0.3336139
ENSBTAG00000026016	NA	NA	NA
ENSBTAG00000026025	NA	NA	NA
ENSBTAG00000026032	NA	NA	NA
ENSBTAG00000026067	NA	NA	NA
ENSBTAG00000026070	NA	NA	NA
ENSBTAG00000026080	NA	NA	NA
ENSBTAG00000026088	NA	NA	NA
ENSBTAG00000026097	0.0918643	0.7800102	0.1078997
ENSBTAG00000026111	-0.190159	0.5883712	0.2303486
ENSBTAG00000026114	0.0491123	0.8635475	0.0637138
ENSBTAG00000026119	NA	NA	NA
ENSBTAG00000026122	NA	NA	NA
ENSBTAG00000026139	NA	NA	NA
ENSBTAG00000026141	NA	NA	NA
ENSBTAG00000026156	0.4376724	0.2163632	0.6648166
ENSBTAG00000026172	0.9063596	0.050742	1.2946325
ENSBTAG00000026181	NA	NA	NA
ENSBTAG00000026191	-0.015008	0.9536755	0.0205994
ENSBTAG00000026192	-0.046782	0.8648948	0.0630367
ENSBTAG00000026194	NA	NA	NA
ENSBTAG00000026199	0.1121882	0.6858502	0.1637707
ENSBTAG00000026232	-1.127185	0.0104565	1.9806138
ENSBTAG00000026233	NA	NA	NA
ENSBTAG00000026234	-0.438889	0.1308842	0.8831129
ENSBTAG00000026236	NA	NA	NA
ENSBTAG00000026242	NA	NA	NA
ENSBTAG00000026243	0.283685	0.4292191	0.367321
ENSBTAG00000026246	0.0482048	0.9233269	0.0346445
ENSBTAG00000026247	0.4705568	0.2690316	0.5701967
ENSBTAG00000026248	0.1406023	0.6615383	0.179445
ENSBTAG00000026249	NA	NA	NA
ENSBTAG00000026254	0.3123294	0.326509	0.4861048
ENSBTAG00000026263	0.1220657	0.7072509	0.1504265
ENSBTAG00000026266	0.5415474	0.1135744	0.9447196

ENSBTAG00000026275	NA	NA	NA
ENSBTAG00000026278	NA	NA	NA
ENSBTAG00000026283	-0.310913	0.2789359	0.5544956
ENSBTAG00000026286	0.2381484	0.4872845	0.3122174
ENSBTAG00000026290	0.0013802	0.9961554	0.0016729
ENSBTAG00000026306	NA	NA	NA
ENSBTAG00000026307	0.5371317	0.1353477	0.8685491
ENSBTAG00000026309	-0.059445	0.8880755	0.0515501
ENSBTAG00000026320	0.044039	0.8799925	0.055521
ENSBTAG00000026322	NA	NA	NA
ENSBTAG00000026323	NA	NA	NA
ENSBTAG00000026326	0.0473634	0.92177	0.0353774
ENSBTAG00000026327	0.0084496	0.9731484	0.0118209
ENSBTAG00000026344	NA	NA	NA
ENSBTAG00000026356	0.121048	0.7016847	0.153858
ENSBTAG00000026369	-0.230897	0.61351	0.2121783
ENSBTAG00000026371	0.3416547	0.3162177	0.5000139
ENSBTAG00000026375	-0.620364	0.2149831	0.6675957
ENSBTAG00000026376	0.372953	0.4097256	0.3875069
ENSBTAG00000026381	NA	NA	NA
ENSBTAG00000026384	NA	NA	NA
ENSBTAG00000026394	1.1911336	0.0308085	1.5113289
ENSBTAG00000026403	0.2083084	0.3997056	0.3982598
ENSBTAG00000026408	-0.699789	0.0612601	1.2128221
ENSBTAG00000026415	-0.092555	0.721927	0.1415067
ENSBTAG00000026418	NA	NA	NA
ENSBTAG00000026422	0.0012942	0.997919	0.0009047
ENSBTAG00000026428	0.0554208	0.8827218	0.0541761
ENSBTAG00000026429	0.0142046	0.9707933	0.0128732
ENSBTAG00000026437	1.1986269	0.1343975	0.8716087
ENSBTAG00000026461	0.5442424	0.16266	0.7887191
ENSBTAG00000026467	NA	NA	NA
ENSBTAG00000026481	NA	NA	NA
ENSBTAG00000026495	NA	NA	NA
ENSBTAG00000026497	NA	NA	NA
ENSBTAG00000026501	-0.523633	0.1967092	0.7061752
ENSBTAG00000026502	NA	NA	NA
ENSBTAG00000026519	0.2199512	0.5936587	0.2264631
ENSBTAG00000026523	0.0728583	0.8797852	0.0556234
ENSBTAG00000026531	NA	NA	NA
ENSBTAG00000026580	NA	NA	NA
ENSBTAG00000026585	-0.107083	0.6913704	0.1602892
ENSBTAG00000026586	-0.813002	0.0022044	2.6567055
ENSBTAG00000026604	-0.341049	0.3749011	0.4260832
ENSBTAG00000026610	NA	NA	NA
ENSBTAG00000026611	NA	NA	NA
ENSBTAG00000026613	-0.144537	0.6937929	0.1587701
ENSBTAG00000026624	-0.13791	0.5928395	0.2270628
ENSBTAG00000026626	NA	NA	NA
ENSBTAG00000026637	0.6159088	0.3555674	0.449078
ENSBTAG00000026638	0.2483232	0.6126944	0.2127561
ENSBTAG00000026645	NA	NA	NA
ENSBTAG00000026650	NA	NA	NA
ENSBTAG00000026657	0.3116536	0.4411739	0.3553902
ENSBTAG00000026660	0.2886898	0.2930651	0.5330358
ENSBTAG00000026666	NA	NA	NA
ENSBTAG00000026672	NA	NA	NA
ENSBTAG00000026673	-0.37085	0.3087965	0.5103276

ENSBTAG00000026676	NA	NA	NA
ENSBTAG00000026684	-0.296582	0.2816248	0.5503291
ENSBTAG00000026696	NA	NA	NA
ENSBTAG00000026704	NA	NA	NA
ENSBTAG00000026708	NA	NA	NA
ENSBTAG00000026710	NA	NA	NA
ENSBTAG00000026716	-0.151848	0.5567891	0.2543093
ENSBTAG00000026748	NA	NA	NA
ENSBTAG00000026751	NA	NA	NA
ENSBTAG00000026753	NA	NA	NA
ENSBTAG00000026754	0.2721278	0.4142968	0.3826884
ENSBTAG00000026758	NA	NA	NA
ENSBTAG00000026768	NA	NA	NA
ENSBTAG00000026769	NA	NA	NA
ENSBTAG00000026772	0.7274143	0.028703	1.5420724
ENSBTAG00000026779	0.9195317	0.0546304	1.2625659
ENSBTAG00000026792	NA	NA	NA
ENSBTAG00000026796	0.9051613	0.0249727	1.602535
ENSBTAG00000026812	NA	NA	NA
ENSBTAG00000026813	NA	NA	NA
ENSBTAG00000026819	-0.017815	0.9471337	0.0235887
ENSBTAG00000026825	NA	NA	NA
ENSBTAG00000026829	-0.277229	0.3713618	0.4302028
ENSBTAG00000026836	0.1582494	0.7201547	0.1425742
ENSBTAG00000026842	-0.19874	0.505724	0.2960865
ENSBTAG00000026851	0.38558	0.2481221	0.6053345
ENSBTAG00000026885	NA	NA	NA
ENSBTAG00000026886	0.3654967	0.2101296	0.6775127
ENSBTAG00000026893	NA	NA	NA
ENSBTAG00000026896	NA	NA	NA
ENSBTAG00000026913	-0.300666	0.4570872	0.3400009
ENSBTAG00000026915	-0.337954	0.2231445	0.6514137
ENSBTAG00000026916	0.2509729	0.5717242	0.2428134
ENSBTAG00000026917	NA	NA	NA
ENSBTAG00000026919	-0.69274	0.1003901	0.998309
ENSBTAG00000026929	NA	NA	NA
ENSBTAG00000026936	NA	NA	NA
ENSBTAG00000026944	NA	NA	NA
ENSBTAG00000026953	-0.223098	0.3806017	0.4195292
ENSBTAG00000026962	-0.021632	0.932608	0.0303009
ENSBTAG00000026963	-0.072623	0.8311256	0.0803334
ENSBTAG00000026966	NA	NA	NA
ENSBTAG00000026971	NA	NA	NA
ENSBTAG00000026972	0.384561	0.3963969	0.4018698
ENSBTAG00000026977	NA	NA	NA
ENSBTAG00000026986	-0.446359	0.1507137	0.8218474
ENSBTAG00000026993	-0.109445	0.6761266	0.169972
ENSBTAG00000026994	-0.242403	0.6938669	0.1587238
ENSBTAG00000026995	0.0297731	0.905122	0.0432929
ENSBTAG00000027015	NA	NA	NA
ENSBTAG00000027017	NA	NA	NA
ENSBTAG00000027020	0.0453119	0.8722198	0.059374
ENSBTAG00000027024	-0.333863	0.2708468	0.5672762
ENSBTAG00000027033	NA	NA	NA
ENSBTAG00000027049	-0.292162	0.3456626	0.4613476
ENSBTAG00000027051	-0.582159	0.2175587	0.6624235
ENSBTAG00000027058	NA	NA	NA
ENSBTAG00000027059	0.1393267	0.7511547	0.1242706

ENSBTAG00000027064	2.2114229	4.54E-08	7.3425913
ENSBTAG00000027069	0.2822875	0.3571059	0.447203
ENSBTAG00000027074	0.5283693	0.2433348	0.6137958
ENSBTAG00000027075	0.6752791	0.1935251	0.7132626
ENSBTAG00000027080	-0.698888	0.0675947	1.1700876
ENSBTAG00000027081	0.0230177	0.9675283	0.0143363
ENSBTAG00000027113	NA	NA	NA
ENSBTAG00000027118	NA	NA	NA
ENSBTAG00000027126	NA	NA	NA
ENSBTAG00000027134	-0.235155	0.6866414	0.16327
ENSBTAG00000027151	-0.673953	0.0616069	1.2103708
ENSBTAG00000027159	-0.183824	0.4834129	0.3156818
ENSBTAG00000027162	NA	NA	NA
ENSBTAG00000027170	NA	NA	NA
ENSBTAG00000027172	NA	NA	NA
ENSBTAG00000027173	0.1519084	0.6481803	0.1883042
ENSBTAG00000027181	-0.277021	0.5880758	0.2305667
ENSBTAG00000027182	-0.917262	0.0014622	2.8349929
ENSBTAG00000027197	NA	NA	NA
ENSBTAG00000027201	-0.54441	0.0649894	1.1871572
ENSBTAG00000027204	0.0543763	0.9204569	0.0359965
ENSBTAG00000027205	NA	NA	NA
ENSBTAG00000027213	NA	NA	NA
ENSBTAG00000027221	NA	NA	NA
ENSBTAG00000027225	NA	NA	NA
ENSBTAG00000027245	NA	NA	NA
ENSBTAG00000027246	-0.539957	0.5125321	0.2902789
ENSBTAG00000027251	NA	NA	NA
ENSBTAG00000027274	NA	NA	NA
ENSBTAG00000027312	NA	NA	NA
ENSBTAG00000027316	-0.311583	0.2566169	0.5907147
ENSBTAG00000027317	0.2208237	0.4108842	0.3862806
ENSBTAG00000027320	0.830014	0.0167989	1.7747201
ENSBTAG00000027321	-0.603361	0.1088567	0.9631449
ENSBTAG00000027326	0.2185732	0.5191337	0.2847208
ENSBTAG00000027328	NA	NA	NA
ENSBTAG00000027337	0.64286	0.0310787	1.5075367
ENSBTAG00000027341	NA	NA	NA
ENSBTAG00000027348	NA	NA	NA
ENSBTAG00000027361	-0.014622	0.9564951	0.0193172
ENSBTAG00000027375	-0.388245	0.1826983	0.7382654
ENSBTAG00000027387	NA	NA	NA
ENSBTAG00000027390	0.0263513	0.9140842	0.0390138
ENSBTAG00000027397	NA	NA	NA
ENSBTAG00000027405	NA	NA	NA
ENSBTAG00000027407	NA	NA	NA
ENSBTAG00000027409	NA	NA	NA
ENSBTAG00000027412	NA	NA	NA
ENSBTAG00000027419	NA	NA	NA
ENSBTAG00000027425	NA	NA	NA
ENSBTAG00000027431	0.2265332	0.4611987	0.3361119
ENSBTAG00000027434	NA	NA	NA
ENSBTAG00000027438	NA	NA	NA
ENSBTAG00000027442	0.4498838	0.1959249	0.7079105
ENSBTAG00000027444	0.0427765	0.8737967	0.0585896
ENSBTAG00000027446	-0.182252	0.5755328	0.2399299
ENSBTAG00000027453	-0.424394	0.3274501	0.4848549
ENSBTAG00000027464	-0.012689	0.97818	0.0095812

ENSBTAG00000027477	0.3884747	0.3449176	0.4622846
ENSBTAG00000027490	NA	NA	NA
ENSBTAG00000027506	-0.139487	0.5988576	0.2226764
ENSBTAG00000027516	0.4703481	0.2325173	0.6335447
ENSBTAG00000027524	0.3466678	0.1817848	0.7404424
ENSBTAG00000027557	0.1216681	0.6855838	0.1639395
ENSBTAG00000027563	NA	NA	NA
ENSBTAG00000027569	0.6712948	0.0152355	1.8171441
ENSBTAG00000027610	0.0392986	0.903104	0.0442622
ENSBTAG00000027612	0.0887512	0.7555307	0.1217479
ENSBTAG00000027625	-0.462941	0.2731878	0.5635386
ENSBTAG00000027626	2.94E-05	1	0
ENSBTAG00000027629	0.0278542	0.9110218	0.0404712
ENSBTAG00000027630	-0.020234	0.9533212	0.0207607
ENSBTAG00000027635	NA	NA	NA
ENSBTAG00000027637	NA	NA	NA
ENSBTAG00000027654	0.0136281	0.9625601	0.0165722
ENSBTAG00000027655	NA	NA	NA
ENSBTAG00000027665	0.3491285	0.3587998	0.4451478
ENSBTAG00000027676	NA	NA	NA
ENSBTAG00000027684	NA	NA	NA
ENSBTAG00000027694	0.1930675	0.6697447	0.1740907
ENSBTAG00000027696	NA	NA	NA
ENSBTAG00000027711	0.0445835	0.8737428	0.0586164
ENSBTAG00000027713	-0.599194	0.0867042	1.0619597
ENSBTAG00000027716	NA	NA	NA
ENSBTAG00000027722	-0.00886	0.9720086	0.0123299
ENSBTAG00000027727	NA	NA	NA
ENSBTAG00000027728	-0.096126	0.7215595	0.1417279
ENSBTAG00000027764	-0.450062	0.0835541	1.0780324
ENSBTAG00000027766	0.2649072	0.4928287	0.307304
ENSBTAG00000027770	0.1510588	0.7346789	0.1339024
ENSBTAG00000027772	-0.032438	0.9033038	0.0441662
ENSBTAG00000027787	-0.257045	0.4772261	0.3212758
ENSBTAG00000027789	-0.036802	0.8913672	0.0499434
ENSBTAG00000027795	-0.641967	0.0226898	1.6441692
ENSBTAG00000027809	0.1046007	0.8727615	0.0591044
ENSBTAG00000027825	NA	NA	NA
ENSBTAG00000027832	NA	NA	NA
ENSBTAG00000027841	NA	NA	NA
ENSBTAG00000027843	-0.261373	0.4089643	0.3883146
ENSBTAG00000027854	NA	NA	NA
ENSBTAG00000027867	NA	NA	NA
ENSBTAG00000027868	-0.286979	0.4426683	0.3539215
ENSBTAG00000027875	0.1993878	0.625575	0.2037206
ENSBTAG00000027878	NA	NA	NA
ENSBTAG00000027879	-0.112282	0.7002583	0.1547417
ENSBTAG00000027899	0.9821466	0.0296885	1.5274111
ENSBTAG00000027900	NA	NA	NA
ENSBTAG00000027916	-0.013074	0.9663402	0.01487
ENSBTAG00000027924	-0.220062	0.399442	0.3985463
ENSBTAG00000027930	-0.115069	0.7511927	0.1242486
ENSBTAG00000027932	-0.133209	0.6068703	0.2169041
ENSBTAG00000027936	NA	NA	NA
ENSBTAG00000027937	0.0670557	0.7879712	0.1034897
ENSBTAG00000027974	NA	NA	NA
ENSBTAG00000027980	NA	NA	NA
ENSBTAG00000027983	0.0568462	0.8458281	0.0727179

ENSBTAG00000027991	0.0033234	0.9931163	0.0029999
ENSBTAG00000030162	NA	NA	NA
ENSBTAG00000030164	0.1906517	0.4575723	0.3395403
ENSBTAG00000030166	NA	NA	NA
ENSBTAG00000030168	0.2409688	0.4518272	0.3450276
ENSBTAG00000030169	-0.085571	0.7383328	0.1317478
ENSBTAG00000030170	0.2083866	0.4686581	0.3291439
ENSBTAG00000030172	0.1298589	0.6762383	0.1699002
ENSBTAG00000030173	NA	NA	NA
ENSBTAG00000030174	0.0394451	0.8828406	0.0541177
ENSBTAG00000030175	0.0565877	0.8830361	0.0540215
ENSBTAG00000030179	NA	NA	NA
ENSBTAG00000030180	0.3003421	0.3611023	0.4423698
ENSBTAG00000030182	NA	NA	NA
ENSBTAG00000030186	NA	NA	NA
ENSBTAG00000030189	NA	NA	NA
ENSBTAG00000030190	0.1539691	0.5668759	0.246512
ENSBTAG00000030193	NA	NA	NA
ENSBTAG00000030198	NA	NA	NA
ENSBTAG00000030200	0.1533426	0.6821812	0.1661003
ENSBTAG00000030208	-0.653677	0.1366107	0.8645152
ENSBTAG00000030209	0.3074593	0.2150306	0.6674998
ENSBTAG00000030210	NA	NA	NA
ENSBTAG00000030222	0.3546941	0.3232039	0.4905234
ENSBTAG00000030225	-0.109687	0.6592429	0.1809545
ENSBTAG00000030227	0.9642314	0.0243058	1.614291
ENSBTAG00000030246	NA	NA	NA
ENSBTAG00000030247	-0.456598	0.3171733	0.4987034
ENSBTAG00000030255	0.1651591	0.698009	0.156139
ENSBTAG00000030257	NA	NA	NA
ENSBTAG00000030258	0.2848538	0.3441584	0.4632416
ENSBTAG00000030259	0.1726564	0.6093515	0.2151321
ENSBTAG00000030269	NA	NA	NA
ENSBTAG00000030274	NA	NA	NA
ENSBTAG00000030278	0.0274594	0.9340858	0.0296132
ENSBTAG00000030282	0.3760265	0.385442	0.414041
ENSBTAG00000030285	0.280212	0.4536064	0.3433208
ENSBTAG00000030286	NA	NA	NA
ENSBTAG00000030296	NA	NA	NA
ENSBTAG00000030297	NA	NA	NA
ENSBTAG00000030301	-0.185293	0.4613748	0.3359461
ENSBTAG00000030302	-0.035707	0.8921329	0.0495704
ENSBTAG00000030305	NA	NA	NA
ENSBTAG00000030317	0.0392085	0.9228683	0.0348603
ENSBTAG00000030319	NA	NA	NA
ENSBTAG00000030322	NA	NA	NA
ENSBTAG00000030333	0.4139537	0.3512921	0.4543317
ENSBTAG00000030334	NA	NA	NA
ENSBTAG00000030335	-0.119497	0.6471749	0.1889784
ENSBTAG00000030337	-0.134652	0.6835165	0.165251
ENSBTAG00000030340	-1.02946	0.0125724	1.9005808
ENSBTAG00000030347	-0.644447	0.2607178	0.5838292
ENSBTAG00000030348	0.4779665	0.1815101	0.7410992
ENSBTAG00000030366	0.4488097	0.1568595	0.8044893
ENSBTAG00000030369	0.3191918	0.4288622	0.3676823
ENSBTAG00000030371	NA	NA	NA
ENSBTAG00000030384	0.098323	0.6955876	0.1576481
ENSBTAG00000030393	1.1905202	0.0041466	2.382306

ENSBTAG00000030403	0.265548	0.5367695	0.2702122
ENSBTAG00000030423	NA	NA	NA
ENSBTAG00000030424	0.7239713	0.0395747	1.4025829
ENSBTAG00000030425	-0.187108	0.5319868	0.2740991
ENSBTAG00000030426	-0.081675	0.8351507	0.0782352
ENSBTAG00000030432	NA	NA	NA
ENSBTAG00000030434	0.6048678	0.0788183	1.1033727
ENSBTAG00000030435	0.0547193	0.8373424	0.0770969
ENSBTAG00000030453	-0.293094	0.245185	0.6105061
ENSBTAG00000030456	-0.212334	0.4849428	0.3143095
ENSBTAG00000030461	NA	NA	NA
ENSBTAG00000030468	NA	NA	NA
ENSBTAG00000030470	0.2225824	0.4696888	0.3281898
ENSBTAG00000030471	NA	NA	NA
ENSBTAG00000030472	NA	NA	NA
ENSBTAG00000030474	NA	NA	NA
ENSBTAG00000030482	NA	NA	NA
ENSBTAG00000030483	NA	NA	NA
ENSBTAG00000030484	NA	NA	NA
ENSBTAG00000030490	NA	NA	NA
ENSBTAG00000030493	0.730059	0.1819803	0.7399757
ENSBTAG00000030499	0.0982745	0.8146132	0.0890486
ENSBTAG00000030502	NA	NA	NA
ENSBTAG00000030503	-0.049523	0.8813569	0.0548482
ENSBTAG00000030504	0.0344887	0.9407149	0.026542
ENSBTAG00000030518	0.2523762	0.4467164	0.3499681
ENSBTAG00000030520	0.7676326	0.2959621	0.5287639
ENSBTAG00000030521	-0.032062	0.9734734	0.0116759
ENSBTAG00000030523	NA	NA	NA
ENSBTAG00000030529	0.0701899	0.789555	0.1026176
ENSBTAG00000030533	NA	NA	NA
ENSBTAG00000030539	NA	NA	NA
ENSBTAG00000030540	NA	NA	NA
ENSBTAG00000030543	NA	NA	NA
ENSBTAG00000030546	NA	NA	NA
ENSBTAG00000030556	0.4460432	0.2688091	0.5705561
ENSBTAG00000030557	0.5333506	0.1090056	0.9625511
ENSBTAG00000030563	NA	NA	NA
ENSBTAG00000030566	0.0425585	0.8911788	0.0500352
ENSBTAG00000030567	0.169806	0.57029	0.2439042
ENSBTAG00000030575	-0.047641	0.8873163	0.0519215
ENSBTAG00000030578	-0.052055	0.8617671	0.0646101
ENSBTAG00000030581	-0.096428	0.7512625	0.1242083
ENSBTAG00000030584	0.2131174	0.5342462	0.2722585
ENSBTAG00000030587	0.1000322	0.6979826	0.1561554
ENSBTAG00000030589	-0.006597	0.9863331	0.0059764
ENSBTAG00000030591	0.4808702	0.1585052	0.7999565
ENSBTAG00000030592	-0.015255	0.9706571	0.0129342
ENSBTAG00000030593	0.6582098	0.1174316	0.930215
ENSBTAG00000030595	-0.063068	0.8342974	0.0786791
ENSBTAG00000030599	-0.297067	0.4034135	0.3942496
ENSBTAG00000030600	-0.043139	0.8720831	0.0594421
ENSBTAG00000030608	-0.101971	0.7737348	0.1114079
ENSBTAG00000030616	-0.432014	0.1901649	0.7208697
ENSBTAG00000030622	NA	NA	NA
ENSBTAG00000030623	NA	NA	NA
ENSBTAG00000030632	0.0577013	0.8308644	0.0804699
ENSBTAG00000030646	NA	NA	NA

ENSBTAG00000030648	0.0944208	0.7676442	0.11484
ENSBTAG00000030650	-0.037826	0.9305211	0.0312738
ENSBTAG00000030667	NA	NA	NA
ENSBTAG00000030669	0.0870183	0.7521999	0.1236667
ENSBTAG00000030670	0.1664141	0.5249503	0.2798818
ENSBTAG00000030671	NA	NA	NA
ENSBTAG00000030674	0.361289	0.1931526	0.7140994
ENSBTAG00000030675	-0.191323	0.5760714	0.2395237
ENSBTAG00000030676	NA	NA	NA
ENSBTAG00000030683	NA	NA	NA
ENSBTAG00000030686	-0.310865	0.386415	0.412946
ENSBTAG00000030690	-0.132808	0.795389	0.0994204
ENSBTAG00000030705	NA	NA	NA
ENSBTAG00000030706	-0.202254	0.5584401	0.2530234
ENSBTAG00000030710	0.257193	0.4306467	0.3658788
ENSBTAG00000030711	NA	NA	NA
ENSBTAG00000030735	NA	NA	NA
ENSBTAG00000030744	-0.136092	0.6043456	0.2187146
ENSBTAG00000030749	NA	NA	NA
ENSBTAG00000030757	NA	NA	NA
ENSBTAG00000030769	0.1839827	0.5804287	0.2362511
ENSBTAG00000030775	NA	NA	NA
ENSBTAG00000030784	-0.197866	0.4381487	0.3583785
ENSBTAG00000030792	NA	NA	NA
ENSBTAG00000030801	-0.100924	0.6954451	0.1577371
ENSBTAG00000030805	-0.2104	0.4259814	0.3706093
ENSBTAG00000030814	NA	NA	NA
ENSBTAG00000030817	-0.13137	0.6875739	0.1626806
ENSBTAG00000030820	-0.032797	0.9211477	0.0356707
ENSBTAG00000030824	NA	NA	NA
ENSBTAG00000030834	NA	NA	NA
ENSBTAG00000030836	-0.578642	0.113944	0.9433086
ENSBTAG00000030839	0.1046819	0.7361093	0.1330577
ENSBTAG00000030841	NA	NA	NA
ENSBTAG00000030852	NA	NA	NA
ENSBTAG00000030855	NA	NA	NA
ENSBTAG00000030864	NA	NA	NA
ENSBTAG00000030881	0.4523707	0.1947097	0.7106123
ENSBTAG00000030882	NA	NA	NA
ENSBTAG00000030885	-0.090109	0.8425511	0.0744037
ENSBTAG00000030890	NA	NA	NA
ENSBTAG00000030897	-0.040753	0.9483795	0.0230179
ENSBTAG00000030898	NA	NA	NA
ENSBTAG00000030910	NA	NA	NA
ENSBTAG00000030913	1.4214199	0.0257276	1.5896012
ENSBTAG00000030915	-0.228744	0.3807199	0.4193944
ENSBTAG00000030920	NA	NA	NA
ENSBTAG00000030921	NA	NA	NA
ENSBTAG00000030922	NA	NA	NA
ENSBTAG00000030929	0.1254055	0.7250344	0.1396414
ENSBTAG00000030930	-0.328664	0.400628	0.3972586
ENSBTAG00000030932	0.6695748	0.1313416	0.8815976
ENSBTAG00000030933	0.3619286	0.3286529	0.4832625
ENSBTAG00000030939	NA	NA	NA
ENSBTAG00000030940	-0.439291	0.3308238	0.4804032
ENSBTAG00000030941	NA	NA	NA
ENSBTAG00000030942	-0.101482	0.6839834	0.1649544
ENSBTAG00000030951	0.8615922	0.0819276	1.0865697

ENSBTAG00000030956	-0.079675	0.7615306	0.1183126
ENSBTAG00000030960	0.2418518	0.5774414	0.2384921
ENSBTAG00000030962	0.0800606	0.8277443	0.0821038
ENSBTAG00000030965	0.0743272	0.7723473	0.1121874
ENSBTAG00000030966	0.7414509	0.0443571	1.3530369
ENSBTAG00000030973	NA	NA	NA
ENSBTAG00000030974	-0.094147	0.742802	0.129127
ENSBTAG00000030977	-0.283341	0.2885497	0.5397793
ENSBTAG00000030979	NA	NA	NA
ENSBTAG00000030990	-0.057218	0.8431943	0.0740723
ENSBTAG00000030994	NA	NA	NA
ENSBTAG00000030999	-0.469828	0.296696	0.5276884
ENSBTAG00000031001	0.0711581	0.7815885	0.1070218
ENSBTAG00000031010	0.0986628	0.7415781	0.1298431
ENSBTAG00000031012	0.1008125	0.7119268	0.1475647
ENSBTAG00000031014	0.7131763	0.0911254	1.0403607
ENSBTAG00000031017	NA	NA	NA
ENSBTAG00000031018	NA	NA	NA
ENSBTAG00000031041	0.0499672	0.9088713	0.0414976
ENSBTAG00000031044	-0.182642	0.5534699	0.256906
ENSBTAG00000031052	NA	NA	NA
ENSBTAG00000031059	NA	NA	NA
ENSBTAG00000031061	0.0970538	0.7042846	0.1522518
ENSBTAG00000031065	NA	NA	NA
ENSBTAG00000031069	-0.033756	0.9239464	0.0343532
ENSBTAG00000031071	-0.125946	0.7537022	0.1228002
ENSBTAG00000031082	-0.033187	0.9526361	0.021073
ENSBTAG00000031084	NA	NA	NA
ENSBTAG00000031088	-0.4492	0.2402446	0.6193464
ENSBTAG00000031106	0.0135076	0.9773921	0.0099312
ENSBTAG00000031107	NA	NA	NA
ENSBTAG00000031115	NA	NA	NA
ENSBTAG00000031134	0.1377496	0.89567	0.047852
ENSBTAG00000031135	-0.887761	0.1100419	0.958442
ENSBTAG00000031146	-0.16724	0.6477081	0.1886206
ENSBTAG00000031160	NA	NA	NA
ENSBTAG00000031165	-0.743004	0.0105056	1.9785793
ENSBTAG00000031166	NA	NA	NA
ENSBTAG00000031171	-0.162426	0.5159145	0.2874223
ENSBTAG00000031178	-0.201471	0.554533	0.2560726
ENSBTAG00000031184	0.0226636	0.9501488	0.0222084
ENSBTAG00000031185	-0.132111	0.6786861	0.168331
ENSBTAG00000031186	NA	NA	NA
ENSBTAG00000031188	NA	NA	NA
ENSBTAG00000031194	NA	NA	NA
ENSBTAG00000031197	NA	NA	NA
ENSBTAG00000031205	-0.617223	0.2457961	0.609425
ENSBTAG00000031209	-0.30844	0.4076155	0.3897493
ENSBTAG00000031210	NA	NA	NA
ENSBTAG00000031214	1.0610065	0.0210903	1.6759179
ENSBTAG00000031216	NA	NA	NA
ENSBTAG00000031217	0.9773256	0.0867949	1.0615058
ENSBTAG00000031231	-0.563077	0.0838727	1.0763796
ENSBTAG00000031234	NA	NA	NA
ENSBTAG00000031236	NA	NA	NA
ENSBTAG00000031238	NA	NA	NA
ENSBTAG00000031242	NA	NA	NA
ENSBTAG00000031246	NA	NA	NA

ENSBTAG00000031249	-0.007662	0.9789623	0.009234
ENSBTAG00000031252	-0.074978	0.7738968	0.111317
ENSBTAG00000031261	NA	NA	NA
ENSBTAG00000031265	NA	NA	NA
ENSBTAG00000031267	-0.293168	0.4792879	0.3194035
ENSBTAG00000031278	NA	NA	NA
ENSBTAG00000031279	0.3673451	0.3005845	0.5220334
ENSBTAG00000031287	NA	NA	NA
ENSBTAG00000031295	0.5824374	0.0360932	1.4425751
ENSBTAG00000031299	-0.014466	0.9584549	0.0184283
ENSBTAG00000031306	NA	NA	NA
ENSBTAG00000031309	NA	NA	NA
ENSBTAG00000031327	0.0058722	0.9877542	0.0053511
ENSBTAG00000031330	NA	NA	NA
ENSBTAG00000031332	-0.181761	0.6918836	0.159967
ENSBTAG00000031335	0.1621851	0.6003967	0.2215617
ENSBTAG00000031340	NA	NA	NA
ENSBTAG00000031346	NA	NA	NA
ENSBTAG00000031347	NA	NA	NA
ENSBTAG00000031348	NA	NA	NA
ENSBTAG00000031351	NA	NA	NA
ENSBTAG00000031352	0.4357283	0.3439631	0.4634881
ENSBTAG00000031355	NA	NA	NA
ENSBTAG00000031358	-0.074777	0.7601942	0.1190755
ENSBTAG00000031359	NA	NA	NA
ENSBTAG00000031362	NA	NA	NA
ENSBTAG00000031363	-0.025911	0.9205573	0.0359492
ENSBTAG00000031364	NA	NA	NA
ENSBTAG00000031376	NA	NA	NA
ENSBTAG00000031377	NA	NA	NA
ENSBTAG00000031383	NA	NA	NA
ENSBTAG00000031385	-0.122396	0.6215251	0.2065413
ENSBTAG00000031387	-0.134999	0.6616285	0.1793858
ENSBTAG00000031388	NA	NA	NA
ENSBTAG00000031395	NA	NA	NA
ENSBTAG00000031396	NA	NA	NA
ENSBTAG00000031397	NA	NA	NA
ENSBTAG00000031402	0.4894402	0.1146148	0.9407594
ENSBTAG00000031430	0.4421715	0.3421559	0.4657759
ENSBTAG00000031432	0.2763198	0.4250942	0.3715148
ENSBTAG00000031433	-0.181449	0.6612398	0.179641
ENSBTAG00000031435	-0.19178	0.4927683	0.3073572
ENSBTAG00000031439	-0.236142	0.3761299	0.4246622
ENSBTAG00000031441	-0.242489	0.5511997	0.258691
ENSBTAG00000031444	NA	NA	NA
ENSBTAG00000031447	NA	NA	NA
ENSBTAG00000031453	0.3245346	0.5058237	0.2960008
ENSBTAG00000031454	NA	NA	NA
ENSBTAG00000031461	-0.06876	0.8507186	0.0702141
ENSBTAG00000031462	NA	NA	NA
ENSBTAG00000031468	NA	NA	NA
ENSBTAG00000031473	-0.269273	0.3346199	0.4754482
ENSBTAG00000031476	-0.533161	0.1040832	0.9826195
ENSBTAG00000031481	NA	NA	NA
ENSBTAG00000031500	-0.014775	0.9611811	0.0171948
ENSBTAG00000031503	NA	NA	NA
ENSBTAG00000031507	NA	NA	NA
ENSBTAG00000031509	0.4569093	0.2331653	0.632336

ENSBTAG00000031517	NA	NA	NA
ENSBTAG00000031519	NA	NA	NA
ENSBTAG00000031523	NA	NA	NA
ENSBTAG00000031524	-0.319371	0.3795635	0.4207155
ENSBTAG00000031532	0.9704791	0.0369108	1.4328468
ENSBTAG00000031544	0.1519281	0.6645217	0.1774908
ENSBTAG00000031548	0.5170804	0.0943561	1.0252299
ENSBTAG00000031551	NA	NA	NA
ENSBTAG00000031553	0.5964859	0.1202026	0.9200861
ENSBTAG00000031558	NA	NA	NA
ENSBTAG00000031561	-0.171318	0.5471661	0.2618809
ENSBTAG00000031564	-0.180071	0.506514	0.2954086
ENSBTAG00000031567	-0.210474	0.4270504	0.3695208
ENSBTAG00000031569	0.227172	0.3908763	0.4079607
ENSBTAG00000031572	-0.308551	0.3371731	0.4721471
ENSBTAG00000031573	-0.282794	0.2945891	0.5307834
ENSBTAG00000031575	0.3185937	0.4122231	0.3848676
ENSBTAG00000031579	-0.03823	0.9143722	0.038877
ENSBTAG00000031583	0.3467042	0.4443446	0.3522801
ENSBTAG00000031590	0.4991383	0.1832776	0.7368906
ENSBTAG00000031595	NA	NA	NA
ENSBTAG00000031598	-0.092297	0.7456824	0.1274461
ENSBTAG00000031609	-0.206461	0.4300495	0.3664815
ENSBTAG00000031614	-0.605086	0.0662106	1.1790727
ENSBTAG00000031618	NA	NA	NA
ENSBTAG00000031625	NA	NA	NA
ENSBTAG00000031631	NA	NA	NA
ENSBTAG00000031632	NA	NA	NA
ENSBTAG00000031641	0.2449414	0.3242676	0.4890965
ENSBTAG00000031647	NA	NA	NA
ENSBTAG00000031648	NA	NA	NA
ENSBTAG00000031654	NA	NA	NA
ENSBTAG00000031656	-0.298542	0.2631659	0.5797704
ENSBTAG00000031658	0.8990027	0.0546301	1.2625679
ENSBTAG00000031669	NA	NA	NA
ENSBTAG00000031673	0.8243763	0.0958732	1.0183027
ENSBTAG00000031679	NA	NA	NA
ENSBTAG00000031682	-0.433527	0.3472914	0.459306
ENSBTAG00000031686	0.0594017	0.880671	0.0551863
ENSBTAG00000031687	0.1295086	0.6791988	0.1680031
ENSBTAG00000031688	0.2029359	0.4445676	0.3520622
ENSBTAG00000031693	NA	NA	NA
ENSBTAG00000031696	0.2968097	0.3325408	0.478155
ENSBTAG00000031697	-0.101481	0.7716844	0.1125603
ENSBTAG00000031701	-0.504726	0.2730122	0.5638179
ENSBTAG00000031704	NA	NA	NA
ENSBTAG00000031707	-0.309673	0.2463834	0.6083886
ENSBTAG00000031709	0.0274513	0.9280617	0.0324231
ENSBTAG00000031711	NA	NA	NA
ENSBTAG00000031715	0.6732176	0.0099192	2.0035232
ENSBTAG00000031716	-0.268484	0.5709535	0.2433993
ENSBTAG00000031717	0.0729421	0.8717811	0.0595925
ENSBTAG00000031718	0.5192572	0.1792474	0.7465471
ENSBTAG00000031719	0.5774909	0.0741299	1.1300065
ENSBTAG00000031721	NA	NA	NA
ENSBTAG00000031723	0.0639991	0.8010283	0.0963521
ENSBTAG00000031724	NA	NA	NA
ENSBTAG00000031725	0.4938803	0.25407	0.5950466

ENSBTAG00000031731	NA	NA	NA
ENSBTAG00000031737	NA	NA	NA
ENSBTAG00000031738	0.6583392	0.0659273	1.1809345
ENSBTAG00000031741	0.3577273	0.3139021	0.5032057
ENSBTAG00000031747	-0.139623	0.594876	0.2255735
ENSBTAG00000031749	-0.238013	0.5743156	0.2408494
ENSBTAG00000031750	0.8618981	0.1402367	0.8531385
ENSBTAG00000031752	0.1175841	0.6891337	0.1616965
ENSBTAG00000031756	0.2317577	0.391119	0.4076911
ENSBTAG00000031757	0.3178488	0.3583496	0.4456931
ENSBTAG00000031758	0.2714563	0.3938774	0.4046389
ENSBTAG00000031759	NA	NA	NA
ENSBTAG00000031760	0.0337676	0.941727	0.026075
ENSBTAG00000031761	-0.231944	0.6037098	0.2191718
ENSBTAG00000031762	0.2665432	0.5873027	0.231138
ENSBTAG00000031763	0.3716976	0.204536	0.6892302
ENSBTAG00000031765	NA	NA	NA
ENSBTAG00000031766	0.2910495	0.52601	0.279006
ENSBTAG00000031768	-0.106424	0.8458645	0.0726992
ENSBTAG00000031769	NA	NA	NA
ENSBTAG00000031770	0.2588701	0.5666795	0.2466625
ENSBTAG00000031771	0.7320215	0.0954752	1.0201094
ENSBTAG00000031772	NA	NA	NA
ENSBTAG00000031773	-0.081902	0.8595443	0.0657317
ENSBTAG00000031774	-0.617236	0.1900399	0.7211551
ENSBTAG00000031776	NA	NA	NA
ENSBTAG00000031777	-0.478655	0.3536575	0.4514171
ENSBTAG00000031778	-0.374969	0.1790712	0.7469741
ENSBTAG00000031780	NA	NA	NA
ENSBTAG00000031785	0.2523835	0.3850384	0.414496
ENSBTAG00000031786	-0.107196	0.6949742	0.1580313
ENSBTAG00000031788	-0.43008	0.1636759	0.7860154
ENSBTAG00000031789	0.5917788	0.134091	0.8726004
ENSBTAG00000031792	0.1099252	0.7795651	0.1081476
ENSBTAG00000031793	-0.162263	0.6091423	0.2152812
ENSBTAG00000031794	NA	NA	NA
ENSBTAG00000031795	NA	NA	NA
ENSBTAG00000031797	-0.023591	0.9374209	0.0280654
ENSBTAG00000031800	0.2223801	0.4158534	0.3810597
ENSBTAG00000031802	NA	NA	NA
ENSBTAG00000031806	1.3552178	0.001027	2.9884401
ENSBTAG00000031809	NA	NA	NA
ENSBTAG00000031810	NA	NA	NA
ENSBTAG00000031814	NA	NA	NA
ENSBTAG00000031824	0.4807023	0.1369282	0.863507
ENSBTAG00000031825	NA	NA	NA
ENSBTAG00000031828	NA	NA	NA
ENSBTAG00000031829	0.273986	0.4816372	0.31728
ENSBTAG00000031834	-0.750431	0.051661	1.2868375
ENSBTAG00000031837	0.2251199	0.3833117	0.4164479
ENSBTAG00000031846	0.3718437	0.2297264	0.638789
ENSBTAG00000031849	NA	NA	NA
ENSBTAG00000031851	NA	NA	NA
ENSBTAG00000031852	-0.054775	0.8523642	0.0693748
ENSBTAG00000031861	0.0143262	0.9553194	0.0198514
ENSBTAG00000031863	0.4097358	0.3195892	0.4954079
ENSBTAG00000031866	NA	NA	NA
ENSBTAG00000031869	0.1046948	0.785797	0.1046896

ENSBTAG00000031871	0.0586432	0.8981238	0.0466638
ENSBTAG00000031873	0.0840962	0.8408561	0.0752783
ENSBTAG00000031874	NA	NA	NA
ENSBTAG00000031875	0.0191795	0.9399335	0.0269029
ENSBTAG00000031885	0.7554321	0.0274677	1.5611769
ENSBTAG00000031886	0.2852014	0.4322157	0.3642995
ENSBTAG00000031888	NA	NA	NA
ENSBTAG00000031889	0.2845566	0.4871219	0.3123624
ENSBTAG00000031890	0.0751111	0.7705883	0.1131776
ENSBTAG00000031891	NA	NA	NA
ENSBTAG00000031895	-0.042988	0.8823094	0.0543791
ENSBTAG00000031898	NA	NA	NA
ENSBTAG00000031906	NA	NA	NA
ENSBTAG00000031916	0.1246292	0.8590389	0.0659872
ENSBTAG00000031917	-0.369154	0.2260748	0.6457478
ENSBTAG00000031919	-0.113933	0.729644	0.136889
ENSBTAG00000031933	NA	NA	NA
ENSBTAG00000031937	NA	NA	NA
ENSBTAG00000031940	0.3273692	0.2378347	0.6237249
ENSBTAG00000031941	0.3605279	0.2083791	0.6811458
ENSBTAG00000031943	NA	NA	NA
ENSBTAG00000031950	0.05535	0.8883538	0.0514141
ENSBTAG00000031962	NA	NA	NA
ENSBTAG00000031965	NA	NA	NA
ENSBTAG00000031967	NA	NA	NA
ENSBTAG00000031981	0.2702358	0.399092	0.398927
ENSBTAG00000031993	NA	NA	NA
ENSBTAG00000031998	0.0495492	0.9113223	0.040328
ENSBTAG00000032003	NA	NA	NA
ENSBTAG00000032007	0.2132758	0.5612312	0.2508582
ENSBTAG00000032017	-1.238986	0.0246277	1.6085757
ENSBTAG00000032018	-0.343059	0.4405773	0.3559778
ENSBTAG00000032021	0.0066621	0.9795209	0.0089863
ENSBTAG00000032022	0.1537134	0.6511198	0.1863391
ENSBTAG00000032024	-0.429652	0.0828268	1.0818292
ENSBTAG00000032026	0.9210818	0.0044709	2.3496007
ENSBTAG00000032031	NA	NA	NA
ENSBTAG00000032034	NA	NA	NA
ENSBTAG00000032047	0.3479406	0.4602318	0.3370234
ENSBTAG00000032051	NA	NA	NA
ENSBTAG00000032055	0.5896999	0.0511829	1.2908751
ENSBTAG00000032057	-0.088638	0.8869665	0.0520928
ENSBTAG00000032059	NA	NA	NA
ENSBTAG00000032068	NA	NA	NA
ENSBTAG00000032071	NA	NA	NA
ENSBTAG00000032079	-0.319524	0.508356	0.293832
ENSBTAG00000032083	0.0748934	0.8442562	0.0735257
ENSBTAG00000032084	NA	NA	NA
ENSBTAG00000032087	0.1977039	0.5533783	0.2569779
ENSBTAG00000032089	0.4036968	0.3350175	0.4749326
ENSBTAG00000032092	0.255123	0.4207633	0.3759622
ENSBTAG00000032097	0.0563687	0.8282122	0.0818584
ENSBTAG00000032106	NA	NA	NA
ENSBTAG00000032121	-0.501492	0.1582034	0.8007843
ENSBTAG00000032122	NA	NA	NA
ENSBTAG00000032125	NA	NA	NA
ENSBTAG00000032130	NA	NA	NA
ENSBTAG00000032132	NA	NA	NA

ENSBTAG00000032137	0.2501108	0.4359219	0.3605913
ENSBTAG00000032140	NA	NA	NA
ENSBTAG00000032148	0.9782626	0.0215584	1.6663841
ENSBTAG00000032151	NA	NA	NA
ENSBTAG00000032152	NA	NA	NA
ENSBTAG00000032156	NA	NA	NA
ENSBTAG00000032163	0.0170087	0.9513886	0.021642
ENSBTAG00000032166	-0.385363	0.1920725	0.7165349
ENSBTAG00000032178	NA	NA	NA
ENSBTAG00000032183	NA	NA	NA
ENSBTAG00000032187	0.6137711	0.2076988	0.6825659
ENSBTAG00000032198	NA	NA	NA
ENSBTAG00000032200	0.1739409	0.5223998	0.281997
ENSBTAG00000032206	NA	NA	NA
ENSBTAG00000032209	NA	NA	NA
ENSBTAG00000032223	0.2169613	0.5221817	0.2821783
ENSBTAG00000032227	0.0176984	0.9477624	0.0233005
ENSBTAG00000032233	NA	NA	NA
ENSBTAG00000032234	0.0056493	0.9876478	0.0053979
ENSBTAG00000032236	NA	NA	NA
ENSBTAG00000032247	-0.071069	0.8224078	0.0849128
ENSBTAG00000032253	0.116274	0.6837947	0.1650743
ENSBTAG00000032259	NA	NA	NA
ENSBTAG00000032260	-0.424146	0.1788443	0.7475248
ENSBTAG00000032265	1.0755758	0.0527941	1.2774146
ENSBTAG00000032277	NA	NA	NA
ENSBTAG00000032288	NA	NA	NA
ENSBTAG00000032289	-0.178492	0.716688	0.1446699
ENSBTAG00000032292	-0.45903	0.2201467	0.6572879
ENSBTAG00000032293	NA	NA	NA
ENSBTAG00000032299	NA	NA	NA
ENSBTAG00000032301	0.224944	0.6651046	0.1771101
ENSBTAG00000032304	-0.128738	0.6921096	0.1598251
ENSBTAG00000032331	0.1389102	0.6366712	0.1960848
ENSBTAG00000032340	-0.188312	0.6340464	0.1978789
ENSBTAG00000032350	NA	NA	NA
ENSBTAG00000032366	0.3069299	0.3178938	0.497718
ENSBTAG00000032369	0.4520725	0.3091539	0.5098253
ENSBTAG00000032372	NA	NA	NA
ENSBTAG00000032373	NA	NA	NA
ENSBTAG00000032374	-0.257931	0.3525006	0.4528401
ENSBTAG00000032396	NA	NA	NA
ENSBTAG00000032405	NA	NA	NA
ENSBTAG00000032427	0.1701105	0.5082268	0.2939424
ENSBTAG00000032432	-1.647464	0.0204515	1.6892744
ENSBTAG00000032433	-0.376392	0.1849328	0.7329861
ENSBTAG00000032436	0.1299435	0.9077395	0.0420388
ENSBTAG00000032446	-0.170917	0.5744542	0.2407446
ENSBTAG00000032450	NA	NA	NA
ENSBTAG00000032451	0.8470397	0.0836436	1.0775672
ENSBTAG00000032452	NA	NA	NA
ENSBTAG00000032453	0.0967633	0.7991804	0.0973552
ENSBTAG00000032455	0.3572349	0.4923366	0.3077379
ENSBTAG00000032456	1.2681587	0.0209495	1.6788265
ENSBTAG00000032458	-0.318945	0.5220466	0.2822908
ENSBTAG00000032477	-0.153695	0.5462676	0.2625946
ENSBTAG00000032481	NA	NA	NA
ENSBTAG00000032485	NA	NA	NA

ENSBTAG00000032493	0.0353023	0.9267692	0.0330284
ENSBTAG00000032503	NA	NA	NA
ENSBTAG00000032508	NA	NA	NA
ENSBTAG00000032509	NA	NA	NA
ENSBTAG00000032515	-0.639446	0.0627429	1.2024352
ENSBTAG00000032517	0.6063283	0.0631054	1.1999337
ENSBTAG00000032518	0.5129895	0.0672831	1.1720939
ENSBTAG00000032519	-0.420777	0.1669378	0.7774454
ENSBTAG00000032521	1.0716845	0.0022455	2.6486867
ENSBTAG00000032527	-0.502565	0.0619071	1.2082599
ENSBTAG00000032531	0.2395298	0.390001	0.4089342
ENSBTAG00000032534	-0.002565	0.9943713	0.0024514
ENSBTAG00000032538	NA	NA	NA
ENSBTAG00000032544	-0.556751	0.2666648	0.5740342
ENSBTAG00000032548	0.7836389	0.1016613	0.9928445
ENSBTAG00000032557	0.1866717	0.4972582	0.3034181
ENSBTAG00000032558	0.5541121	0.2168465	0.6638476
ENSBTAG00000032588	0.8099123	0.1120285	0.9506716
ENSBTAG00000032591	NA	NA	NA
ENSBTAG00000032598	0.1466226	0.7071799	0.1504701
ENSBTAG00000032603	-0.06795	0.8270505	0.082468
ENSBTAG00000032613	NA	NA	NA
ENSBTAG00000032617	-0.85496	0.0984405	1.0068261
ENSBTAG00000032623	NA	NA	NA
ENSBTAG00000032631	NA	NA	NA
ENSBTAG00000032637	-0.463575	0.0742306	1.1294169
ENSBTAG00000032640	-0.36728	0.262906	0.5801996
ENSBTAG00000032642	NA	NA	NA
ENSBTAG00000032643	NA	NA	NA
ENSBTAG00000032650	NA	NA	NA
ENSBTAG00000032656	NA	NA	NA
ENSBTAG00000032657	-0.558245	0.0551348	1.2585739
ENSBTAG00000032660	0.2424525	0.4111441	0.386006
ENSBTAG00000032674	0.026296	0.9231633	0.0347215
ENSBTAG00000032680	0.4024836	0.2141028	0.6693777
ENSBTAG00000032684	-0.325749	0.5102149	0.2922468
ENSBTAG00000032686	NA	NA	NA
ENSBTAG00000032704	-0.450333	0.2212394	0.6551376
ENSBTAG00000032705	-0.387329	0.1177219	0.9291428
ENSBTAG00000032709	NA	NA	NA
ENSBTAG00000032719	0.2040841	0.5386961	0.2686562
ENSBTAG00000032733	NA	NA	NA
ENSBTAG00000032738	NA	NA	NA
ENSBTAG00000032761	NA	NA	NA
ENSBTAG00000032763	0.0733918	0.8134466	0.089671
ENSBTAG00000032774	0.0406037	0.8938194	0.0487502
ENSBTAG00000032775	NA	NA	NA
ENSBTAG00000032777	-0.043212	0.8661262	0.0624188
ENSBTAG00000032782	-0.846069	0.0792912	1.1007748
ENSBTAG00000032790	0.6082465	0.2579251	0.5885064
ENSBTAG00000032806	NA	NA	NA
ENSBTAG00000032808	NA	NA	NA
ENSBTAG00000032812	NA	NA	NA
ENSBTAG00000032821	NA	NA	NA
ENSBTAG00000032829	-0.200503	0.4462432	0.3504284
ENSBTAG00000032831	0.1842575	0.5584464	0.2530185
ENSBTAG00000032839	-0.395923	0.1748763	0.7572691
ENSBTAG00000032844	NA	NA	NA

ENSBTAG00000032846	NA	NA	NA
ENSBTAG00000032848	-0.047091	0.854379	0.0683494
ENSBTAG00000032852	NA	NA	NA
ENSBTAG00000032858	NA	NA	NA
ENSBTAG00000032859	0.41881	0.3814731	0.418536
ENSBTAG00000032862	NA	NA	NA
ENSBTAG00000032869	NA	NA	NA
ENSBTAG00000032872	0.0432473	0.9009331	0.0453075
ENSBTAG00000032873	NA	NA	NA
ENSBTAG00000032875	NA	NA	NA
ENSBTAG00000032880	NA	NA	NA
ENSBTAG00000032881	0.2796427	0.3602074	0.4434474
ENSBTAG00000032887	0.202041	0.6805015	0.1671709
ENSBTAG00000032893	NA	NA	NA
ENSBTAG00000032894	NA	NA	NA
ENSBTAG00000032899	NA	NA	NA
ENSBTAG00000032902	0.0779531	0.8187911	0.0868269
ENSBTAG00000032905	0.3287768	0.3332766	0.4771951
ENSBTAG00000032908	NA	NA	NA
ENSBTAG00000032914	0.0219249	0.9382762	0.0276693
ENSBTAG00000032933	-0.244694	0.6839328	0.1649866
ENSBTAG00000032947	NA	NA	NA
ENSBTAG00000032951	NA	NA	NA
ENSBTAG00000032954	0.9507236	0.0482135	1.3168311
ENSBTAG00000032961	-0.161251	0.5728935	0.2419261
ENSBTAG00000032962	NA	NA	NA
ENSBTAG00000032964	-0.180774	0.5151959	0.2880276
ENSBTAG00000032979	NA	NA	NA
ENSBTAG00000032982	-0.050878	0.8584292	0.0662955
ENSBTAG00000032996	-0.274878	0.2859701	0.5436794
ENSBTAG00000033008	-0.160588	0.5291327	0.2764354
ENSBTAG00000033010	-0.114943	0.6815444	0.1665058
ENSBTAG00000033015	-0.116169	0.6640485	0.1778002
ENSBTAG00000033030	NA	NA	NA
ENSBTAG00000033032	0.0175857	0.95539	0.0198193
ENSBTAG00000033041	-0.148567	0.61375	0.2120085
ENSBTAG00000033056	NA	NA	NA
ENSBTAG00000033076	0.6292386	0.2197083	0.6581535
ENSBTAG00000033077	0.0393388	0.9168142	0.0377187
ENSBTAG00000033078	-0.148498	0.6133319	0.2123045
ENSBTAG00000033080	0.0101351	0.9680069	0.0141216
ENSBTAG00000033089	-0.971058	0.0005054	3.2963493
ENSBTAG00000033095	-0.069507	0.8003832	0.0967021
ENSBTAG00000033096	NA	NA	NA
ENSBTAG00000033107	0.5695176	0.0466131	1.331492
ENSBTAG00000033117	NA	NA	NA
ENSBTAG00000033122	NA	NA	NA
ENSBTAG00000033136	-0.200625	0.5117138	0.2909729
ENSBTAG00000033137	0.0258258	0.9296196	0.0316948
ENSBTAG00000033140	NA	NA	NA
ENSBTAG00000033153	NA	NA	NA
ENSBTAG00000033160	0.0221824	0.9397485	0.0269884
ENSBTAG00000033166	NA	NA	NA
ENSBTAG00000033167	NA	NA	NA
ENSBTAG00000033169	NA	NA	NA
ENSBTAG00000033170	0.5410777	0.0382565	1.4172949
ENSBTAG00000033173	NA	NA	NA
ENSBTAG00000033174	0.1773002	0.7412172	0.1300545

ENSBTAG00000033180	0.2345667	0.4853198	0.313972
ENSBTAG00000033182	-0.014993	0.96368	0.0160672
ENSBTAG00000033186	-0.277238	0.2776663	0.5564768
ENSBTAG00000033190	0.2612424	0.2944831	0.5309397
ENSBTAG00000033195	NA	NA	NA
ENSBTAG00000033197	-0.10809	0.7502093	0.1248176
ENSBTAG00000033214	-0.033412	0.9214228	0.035541
ENSBTAG00000033217	-0.049218	0.8528299	0.0691376
ENSBTAG00000033218	NA	NA	NA
ENSBTAG00000033220	NA	NA	NA
ENSBTAG00000033221	NA	NA	NA
ENSBTAG00000033222	NA	NA	NA
ENSBTAG00000033225	NA	NA	NA
ENSBTAG00000033248	0.48929	0.3258876	0.4869322
ENSBTAG00000033252	NA	NA	NA
ENSBTAG00000033254	NA	NA	NA
ENSBTAG00000033255	0.1153758	0.6963209	0.1571905
ENSBTAG00000033267	-0.773011	0.049347	1.3067395
ENSBTAG00000033268	0.0301801	0.91248	0.0397767
ENSBTAG00000033284	-0.171299	0.5564866	0.2545453
ENSBTAG00000033290	-0.127639	0.7144457	0.1460308
ENSBTAG00000033291	NA	NA	NA
ENSBTAG00000033292	NA	NA	NA
ENSBTAG00000033298	0.2756955	0.4216435	0.3750546
ENSBTAG00000033299	NA	NA	NA
ENSBTAG00000033304	-0.294258	0.387752	0.411446
ENSBTAG00000033313	0.280814	0.3747153	0.4262986
ENSBTAG00000033315	-0.100053	0.7454031	0.1276088
ENSBTAG00000033316	NA	NA	NA
ENSBTAG00000033319	0.3925651	0.2833753	0.547638
ENSBTAG00000033322	-0.293213	0.2481844	0.6052256
ENSBTAG00000033326	NA	NA	NA
ENSBTAG00000033327	NA	NA	NA
ENSBTAG00000033331	-0.064736	0.816342	0.0881279
ENSBTAG00000033333	0.238996	0.5173106	0.2862486
ENSBTAG00000033334	0.9277546	0.0666337	1.1763064
ENSBTAG00000033335	NA	NA	NA
ENSBTAG00000033339	-0.100878	0.6790729	0.1680836
ENSBTAG00000033344	-0.068068	0.8452768	0.0730011
ENSBTAG00000033345	NA	NA	NA
ENSBTAG00000033346	NA	NA	NA
ENSBTAG00000033351	NA	NA	NA
ENSBTAG00000033352	NA	NA	NA
ENSBTAG00000033365	0.04734	0.92008	0.0361744
ENSBTAG00000033367	-0.199571	0.4630318	0.3343892
ENSBTAG00000033395	-0.272812	0.3024759	0.5193092
ENSBTAG00000033396	NA	NA	NA
ENSBTAG00000033397	NA	NA	NA
ENSBTAG00000033398	NA	NA	NA
ENSBTAG00000033412	-0.108457	0.7525059	0.1234901
ENSBTAG00000033413	-0.173294	0.5846656	0.2330925
ENSBTAG00000033422	-0.369383	0.1589142	0.7988372
ENSBTAG00000033423	NA	NA	NA
ENSBTAG00000033429	0.0683931	0.8373002	0.0771188
ENSBTAG00000033437	NA	NA	NA
ENSBTAG00000033441	0.9372941	0.1202451	0.9199325
ENSBTAG00000033445	NA	NA	NA
ENSBTAG00000033446	-0.351086	0.2308149	0.6367362

ENSBTAG00000033449	0.385911	0.3276864	0.4845416
ENSBTAG00000033453	0.0946094	0.7087142	0.1495289
ENSBTAG00000033457	-0.087627	0.7502576	0.1247896
ENSBTAG00000033460	0.7310219	0.0587793	1.2307757
ENSBTAG00000033464	-0.59736	0.2124458	0.6727519
ENSBTAG00000033476	NA	NA	NA
ENSBTAG00000033481	NA	NA	NA
ENSBTAG00000033486	-0.356057	0.245103	0.6106513
ENSBTAG00000033504	-0.240338	0.4066899	0.3907366
ENSBTAG00000033510	NA	NA	NA
ENSBTAG00000033515	-0.638689	0.1715906	0.7655064
ENSBTAG00000033529	-0.173466	0.76042	0.1189465
ENSBTAG00000033531	NA	NA	NA
ENSBTAG00000033535	NA	NA	NA
ENSBTAG00000033543	0.1637428	0.5851187	0.2327561
ENSBTAG00000033563	NA	NA	NA
ENSBTAG00000033603	-0.035398	0.9013063	0.0451276
ENSBTAG00000033604	NA	NA	NA
ENSBTAG00000033621	-0.608133	0.2565457	0.5908352
ENSBTAG00000033642	0.5037191	0.1572638	0.8033712
ENSBTAG00000033643	NA	NA	NA
ENSBTAG00000033648	-0.015949	0.958868	0.0182412
ENSBTAG00000033657	NA	NA	NA
ENSBTAG00000033662	-0.308494	0.2247754	0.6482511
ENSBTAG00000033669	0.8581468	0.0294332	1.5311627
ENSBTAG00000033672	0.1712456	0.6704399	0.1736401
ENSBTAG00000033677	0.7993522	0.1032456	0.9861284
ENSBTAG00000033679	-0.584198	0.0421529	1.3751722
ENSBTAG00000033680	0.1935402	0.5741352	0.2409858
ENSBTAG00000033685	0.7246859	0.1029783	0.9872545
ENSBTAG00000033690	-0.223603	0.666235	0.1763725
ENSBTAG00000033699	NA	NA	NA
ENSBTAG00000033702	0.0430398	0.8977374	0.0468507
ENSBTAG00000033721	NA	NA	NA
ENSBTAG00000033726	-0.558311	0.2386136	0.6223048
ENSBTAG00000033727	0.1455761	0.7014136	0.1540258
ENSBTAG00000033731	0.1491106	0.6702459	0.1737658
ENSBTAG00000033735	0.1871034	0.4785995	0.3200278
ENSBTAG00000033739	NA	NA	NA
ENSBTAG00000033747	-0.313017	0.2979788	0.5258146
ENSBTAG00000033748	NA	NA	NA
ENSBTAG00000033759	NA	NA	NA
ENSBTAG00000033801	-0.204143	0.5089032	0.2933648
ENSBTAG00000033803	NA	NA	NA
ENSBTAG00000033806	NA	NA	NA
ENSBTAG00000033835	-0.025177	0.9617335	0.0169453
ENSBTAG00000033841	NA	NA	NA
ENSBTAG00000033884	NA	NA	NA
ENSBTAG00000033887	NA	NA	NA
ENSBTAG00000033891	0.4369949	0.2647281	0.5772
ENSBTAG00000033902	-0.295838	0.3541921	0.4507612
ENSBTAG00000033961	-0.728925	0.0803804	1.0948498
ENSBTAG00000033983	-0.09756	0.7060048	0.1511924
ENSBTAG00000033998	NA	NA	NA
ENSBTAG00000034005	0.0802226	0.7866257	0.1042319
ENSBTAG00000034033	0.1673751	0.5663648	0.2469037
ENSBTAG00000034045	NA	NA	NA
ENSBTAG00000034069	0.2806235	0.5053234	0.2964306

ENSBTAG00000034075	NA	NA	NA
ENSBTAG00000034077	NA	NA	NA
ENSBTAG00000034089	NA	NA	NA
ENSBTAG00000034090	NA	NA	NA
ENSBTAG00000034091	-1.094273	0.026656	1.5742053
ENSBTAG00000034106	NA	NA	NA
ENSBTAG00000034113	0.4284834	0.2043106	0.6897092
ENSBTAG00000034138	NA	NA	NA
ENSBTAG00000034139	NA	NA	NA
ENSBTAG00000034140	NA	NA	NA
ENSBTAG00000034145	NA	NA	NA
ENSBTAG00000034147	-0.083723	0.8532259	0.0689359
ENSBTAG00000034154	0.4263892	0.4069557	0.3904528
ENSBTAG00000034159	NA	NA	NA
ENSBTAG00000034170	NA	NA	NA
ENSBTAG00000034174	NA	NA	NA
ENSBTAG00000034185	0.2592125	0.3401215	0.4683659
ENSBTAG00000034189	NA	NA	NA
ENSBTAG00000034192	NA	NA	NA
ENSBTAG00000034193	-1.16398	0.007192	2.1431531
ENSBTAG00000034196	NA	NA	NA
ENSBTAG00000034206	0.0160227	0.955788	0.0196384
ENSBTAG00000034220	NA	NA	NA
ENSBTAG00000034222	-0.118639	0.7692411	0.1139375
ENSBTAG00000034225	0.333914	0.3177927	0.4978561
ENSBTAG00000034238	NA	NA	NA
ENSBTAG00000034246	NA	NA	NA
ENSBTAG00000034255	-0.273744	0.4044189	0.3931685
ENSBTAG00000034261	NA	NA	NA
ENSBTAG00000034269	0.2071679	0.5818811	0.2351657
ENSBTAG00000034281	NA	NA	NA
ENSBTAG00000034283	NA	NA	NA
ENSBTAG00000034295	NA	NA	NA
ENSBTAG00000034302	NA	NA	NA
ENSBTAG00000034323	NA	NA	NA
ENSBTAG00000034332	NA	NA	NA
ENSBTAG00000034347	NA	NA	NA
ENSBTAG00000034349	1.0633573	0.0757155	1.1208154
ENSBTAG00000034360	-0.162766	0.7366104	0.1327622
ENSBTAG00000034366	-1.433931	0.0010937	2.9610998
ENSBTAG00000034368	NA	NA	NA
ENSBTAG00000034373	-0.057247	0.837245	0.0771475
ENSBTAG00000034384	-0.22727	0.6835314	0.1652415
ENSBTAG00000034385	NA	NA	NA
ENSBTAG00000034391	NA	NA	NA
ENSBTAG00000034393	NA	NA	NA
ENSBTAG00000034396	0.0998027	0.7937008	0.1003432
ENSBTAG00000034402	NA	NA	NA
ENSBTAG00000034411	NA	NA	NA
ENSBTAG00000034430	0.6491797	0.1741126	0.7591698
ENSBTAG00000034433	NA	NA	NA
ENSBTAG00000034435	-0.109088	0.7931359	0.1006524
ENSBTAG00000034436	0.2950132	0.2757969	0.5594106
ENSBTAG00000034437	NA	NA	NA
ENSBTAG00000034441	NA	NA	NA
ENSBTAG00000034442	NA	NA	NA
ENSBTAG00000034449	0.1887944	0.5362386	0.2706419
ENSBTAG00000034493	-0.73169	0.0186885	1.7284248

ENSBTAG00000034495	-0.813951	0.0277145	1.5572923
ENSBTAG00000034496	0.0315331	0.9285894	0.0321763
ENSBTAG00000034498	NA	NA	NA
ENSBTAG00000034501	NA	NA	NA
ENSBTAG00000034506	-0.235731	0.5304592	0.275348
ENSBTAG00000034519	NA	NA	NA
ENSBTAG00000034522	0.075976	0.8404686	0.0754785
ENSBTAG00000034529	0.0879626	0.8301224	0.0808579
ENSBTAG00000034531	0.0749965	0.7701403	0.1134301
ENSBTAG00000034580	-0.13992	0.7537426	0.1227769
ENSBTAG00000034586	0.7533833	0.0934874	1.029247
ENSBTAG00000034598	0.2455695	0.3915916	0.4071666
ENSBTAG00000034609	NA	NA	NA
ENSBTAG00000034611	NA	NA	NA
ENSBTAG00000034613	NA	NA	NA
ENSBTAG00000034626	NA	NA	NA
ENSBTAG00000034632	NA	NA	NA
ENSBTAG00000034633	-0.010063	0.9826782	0.0075887
ENSBTAG00000034643	NA	NA	NA
ENSBTAG00000034645	0.2979369	0.3515774	0.4539791
ENSBTAG00000034656	NA	NA	NA
ENSBTAG00000034659	-0.372743	0.4160348	0.3808704
ENSBTAG00000034674	NA	NA	NA
ENSBTAG00000034676	NA	NA	NA
ENSBTAG00000034678	NA	NA	NA
ENSBTAG00000034680	NA	NA	NA
ENSBTAG00000034681	NA	NA	NA
ENSBTAG00000034689	-0.342623	0.1743305	0.7586267
ENSBTAG00000034691	0.1460511	0.6316834	0.1995006
ENSBTAG00000034693	NA	NA	NA
ENSBTAG00000034700	-0.040868	0.9041674	0.0437512
ENSBTAG00000034711	NA	NA	NA
ENSBTAG00000034712	NA	NA	NA
ENSBTAG00000034753	NA	NA	NA
ENSBTAG00000034761	NA	NA	NA
ENSBTAG00000034776	0.1360991	0.7951584	0.0995464
ENSBTAG00000034785	0.1394485	0.643807	0.1912443
ENSBTAG00000034796	-0.0754	0.8397271	0.0758618
ENSBTAG00000034823	-0.461075	0.0608432	1.2157883
ENSBTAG00000034827	-0.460743	0.2825721	0.5488707
ENSBTAG00000034841	NA	NA	NA
ENSBTAG00000034844	NA	NA	NA
ENSBTAG00000034848	NA	NA	NA
ENSBTAG00000034850	-0.416534	0.1864752	0.7293789
ENSBTAG00000034854	NA	NA	NA
ENSBTAG00000034867	0.4399665	0.16606	0.7797349
ENSBTAG00000034871	NA	NA	NA
ENSBTAG00000034875	0.4851296	0.2843045	0.5462162
ENSBTAG00000034883	NA	NA	NA
ENSBTAG00000034885	0.2281557	0.4000766	0.3978568
ENSBTAG00000034905	NA	NA	NA
ENSBTAG00000034918	NA	NA	NA
ENSBTAG00000034925	NA	NA	NA
ENSBTAG00000034936	-0.422884	0.1488485	0.8272555
ENSBTAG00000034940	NA	NA	NA
ENSBTAG00000034949	NA	NA	NA
ENSBTAG00000034952	NA	NA	NA
ENSBTAG00000034963	NA	NA	NA

ENSBTAG00000034973	NA	NA	NA
ENSBTAG00000034978	0.1547913	0.5258427	0.2791442
ENSBTAG00000034985	NA	NA	NA
ENSBTAG00000034987	-0.049502	0.8948042	0.048272
ENSBTAG00000034991	0.0985503	0.7957328	0.0992327
ENSBTAG00000034992	-0.00352	0.990338	0.0042166
ENSBTAG00000034995	NA	NA	NA
ENSBTAG00000034998	0.1936472	0.6791448	0.1680376
ENSBTAG00000035007	NA	NA	NA
ENSBTAG00000035012	NA	NA	NA
ENSBTAG00000035018	-0.454493	0.3975527	0.4006053
ENSBTAG00000035030	0.1534621	0.5328518	0.2733936
ENSBTAG00000035054	NA	NA	NA
ENSBTAG00000035064	-0.172685	0.7275503	0.138137
ENSBTAG00000035072	NA	NA	NA
ENSBTAG00000035081	-0.041719	0.8906835	0.0502766
ENSBTAG00000035083	0.1433734	0.557441	0.2538011
ENSBTAG00000035084	0.43261	0.2369795	0.6252892
ENSBTAG00000035110	NA	NA	NA
ENSBTAG00000035122	NA	NA	NA
ENSBTAG00000035129	NA	NA	NA
ENSBTAG00000035144	NA	NA	NA
ENSBTAG00000035147	NA	NA	NA
ENSBTAG00000035158	NA	NA	NA
ENSBTAG00000035174	0.0192281	0.9612488	0.0171642
ENSBTAG00000035175	-0.248221	0.4444957	0.3521325
ENSBTAG00000035182	NA	NA	NA
ENSBTAG00000035183	NA	NA	NA
ENSBTAG00000035199	0.0480177	0.9056118	0.0430579
ENSBTAG00000035226	-0.060475	0.8106444	0.0911696
ENSBTAG00000035230	-0.091202	0.7239015	0.1403205
ENSBTAG00000035244	-0.289942	0.3928645	0.4057572
ENSBTAG00000035247	-0.074054	0.814853	0.0889207
ENSBTAG00000035254	-0.135828	0.6271262	0.2026451
ENSBTAG00000035265	-0.073356	0.8343473	0.0786531
ENSBTAG00000035286	-0.096784	0.7397034	0.1309424
ENSBTAG00000035293	NA	NA	NA
ENSBTAG00000035319	-0.765145	0.1047983	0.9796458
ENSBTAG00000035323	0.1366505	0.5862234	0.2319368
ENSBTAG00000035370	-0.20506	0.5640328	0.2486957
ENSBTAG00000035373	NA	NA	NA
ENSBTAG00000035399	NA	NA	NA
ENSBTAG00000035437	-0.144571	0.5648673	0.2480536
ENSBTAG00000035438	-0.229678	0.6310045	0.1999675
ENSBTAG00000035442	NA	NA	NA
ENSBTAG00000035544	NA	NA	NA
ENSBTAG00000035556	-0.273152	0.3580843	0.4460147
ENSBTAG00000035584	-0.753352	0.123415	0.9086321
ENSBTAG00000035587	0.7680956	0.1134117	0.9453423
ENSBTAG00000035615	-0.381866	0.3575336	0.4466831
ENSBTAG00000035631	NA	NA	NA
ENSBTAG00000035643	NA	NA	NA
ENSBTAG00000035654	NA	NA	NA
ENSBTAG00000035660	NA	NA	NA
ENSBTAG00000035662	-0.436507	0.2896934	0.5380614
ENSBTAG00000035675	NA	NA	NA
ENSBTAG00000035697	0.0357887	0.935378	0.0290129
ENSBTAG00000035705	-3.575458	0.000339	3.4698387

ENSBTAG00000035706	NA	NA	NA
ENSBTAG00000035709	NA	NA	NA
ENSBTAG00000035710	NA	NA	NA
ENSBTAG00000035735	-0.431762	0.0857335	1.0668493
ENSBTAG00000035744	0.5899374	0.0867923	1.0615187
ENSBTAG00000035764	NA	NA	NA
ENSBTAG00000035776	NA	NA	NA
ENSBTAG00000035782	-0.14833	0.5842691	0.2333871
ENSBTAG00000035827	0.0788275	0.807481	0.0928677
ENSBTAG00000035836	-0.010651	0.9724691	0.0121242
ENSBTAG00000035844	-0.348912	0.172169	0.7640452
ENSBTAG00000035858	0.2515025	0.49577	0.3047198
ENSBTAG00000035868	NA	NA	NA
ENSBTAG00000035907	0.0770472	0.7737806	0.1113822
ENSBTAG00000035915	NA	NA	NA
ENSBTAG00000035926	0.3312592	0.5519945	0.2580653
ENSBTAG00000035945	NA	NA	NA
ENSBTAG00000035958	0.1168958	0.71502	0.1456818
ENSBTAG00000035975	NA	NA	NA
ENSBTAG00000035988	NA	NA	NA
ENSBTAG00000035995	-0.063576	0.8670544	0.0619537
ENSBTAG00000035998	-0.196343	0.7588721	0.1198314
ENSBTAG00000036009	0.085651	0.7463512	0.1270568
ENSBTAG00000036016	0.8933119	0.072479	1.1397879
ENSBTAG00000036019	NA	NA	NA
ENSBTAG00000036028	-0.755285	0.1131563	0.9463214
ENSBTAG00000036060	NA	NA	NA
ENSBTAG00000036061	-0.525019	0.4246686	0.3719498
ENSBTAG00000036078	-1.005869	0.0071377	2.1464437
ENSBTAG00000036087	0.7098545	0.0959787	1.0178253
ENSBTAG00000036099	NA	NA	NA
ENSBTAG00000036101	0.3411245	0.3021549	0.5197704
ENSBTAG00000036102	NA	NA	NA
ENSBTAG00000036113	NA	NA	NA
ENSBTAG00000036115	NA	NA	NA
ENSBTAG00000036127	-0.571286	0.1204669	0.9191321
ENSBTAG00000036154	NA	NA	NA
ENSBTAG00000036183	0.1787205	0.5222861	0.2820915
ENSBTAG00000036222	NA	NA	NA
ENSBTAG00000036224	NA	NA	NA
ENSBTAG00000036242	NA	NA	NA
ENSBTAG00000036257	NA	NA	NA
ENSBTAG00000036258	NA	NA	NA
ENSBTAG00000036260	-0.373197	0.4232728	0.3733796
ENSBTAG00000036262	0.0212407	0.9360634	0.0286947
ENSBTAG00000036277	NA	NA	NA
ENSBTAG00000036282	-0.131187	0.6639102	0.1778907
ENSBTAG00000036287	NA	NA	NA
ENSBTAG00000036297	-0.531959	0.1360056	0.8664433
ENSBTAG00000036298	NA	NA	NA
ENSBTAG00000036310	0.5420472	0.2817545	0.5501291
ENSBTAG00000036343	NA	NA	NA
ENSBTAG00000036349	NA	NA	NA
ENSBTAG00000037375	0.0096442	0.9771202	0.010052
ENSBTAG00000037377	0.2408223	0.543542	0.2647669
ENSBTAG00000037383	-0.176617	0.5010434	0.3001247
ENSBTAG00000037389	-0.180058	0.6344724	0.1975873
ENSBTAG00000037393	0.1337268	0.6441022	0.1910452

ENSBTAG00000037397	NA	NA	NA
ENSBTAG00000037399	NA	NA	NA
ENSBTAG00000037400	0.2768393	0.3326515	0.4780105
ENSBTAG00000037404	NA	NA	NA
ENSBTAG00000037413	-0.084938	0.8258461	0.0831009
ENSBTAG00000037415	-0.192041	0.5319433	0.2741347
ENSBTAG00000037418	NA	NA	NA
ENSBTAG00000037429	NA	NA	NA
ENSBTAG00000037440	-0.118312	0.6904573	0.1608632
ENSBTAG00000037449	-0.409093	0.2689894	0.5702648
ENSBTAG00000037452	-0.394469	0.6101117	0.2145907
ENSBTAG00000037453	NA	NA	NA
ENSBTAG00000037456	0.1845083	0.6016003	0.2206919
ENSBTAG00000037457	-0.131813	0.6548276	0.183873
ENSBTAG00000037461	0.209565	0.5504928	0.2592484
ENSBTAG00000037465	1.0753974	0.0493633	1.306596
ENSBTAG00000037470	NA	NA	NA
ENSBTAG00000037489	0.5427053	0.10388	0.983468
ENSBTAG00000037490	NA	NA	NA
ENSBTAG00000037493	0.1856615	0.5497405	0.2598423
ENSBTAG00000037508	0.6185621	0.0174096	1.7592113
ENSBTAG00000037509	NA	NA	NA
ENSBTAG00000037510	0.0984512	0.8381844	0.0766604
ENSBTAG00000037523	NA	NA	NA
ENSBTAG00000037526	0.2389869	0.6476221	0.1886784
ENSBTAG00000037527	1.2114387	0.0854617	1.0682287
ENSBTAG00000037533	0.9694786	0.0164926	1.7827112
ENSBTAG00000037539	-0.529688	0.153585	0.8136512
ENSBTAG00000037549	0.0767483	0.8439319	0.0736926
ENSBTAG00000037552	NA	NA	NA
ENSBTAG00000037558	NA	NA	NA
ENSBTAG00000037565	NA	NA	NA
ENSBTAG00000037566	0.0651839	0.8845162	0.0532942
ENSBTAG00000037571	-0.029869	0.9072928	0.0422525
ENSBTAG00000037578	NA	NA	NA
ENSBTAG00000037580	-0.511877	0.2700045	0.5686291
ENSBTAG00000037581	0.2732866	0.4300432	0.3664879
ENSBTAG00000037595	0.4102422	0.2248311	0.6481435
ENSBTAG00000037600	0.2687338	0.5307788	0.2750864
ENSBTAG00000037603	NA	NA	NA
ENSBTAG00000037604	NA	NA	NA
ENSBTAG00000037605	0.0582655	0.9531107	0.0208567
ENSBTAG00000037608	NA	NA	NA
ENSBTAG00000037613	NA	NA	NA
ENSBTAG00000037616	NA	NA	NA
ENSBTAG00000037625	NA	NA	NA
ENSBTAG00000037632	NA	NA	NA
ENSBTAG00000037634	-1.259664	0.0983774	1.0071048
ENSBTAG00000037640	0.6412772	0.0563355	1.2492182
ENSBTAG00000037649	0.0782225	0.8791381	0.0559429
ENSBTAG00000037650	NA	NA	NA
ENSBTAG00000037651	0.0109601	0.9721644	0.0122603
ENSBTAG00000037661	0.1520321	0.5888194	0.2300179
ENSBTAG00000037673	-0.364756	0.331941	0.4789392
ENSBTAG00000037679	1.1792803	0.012743	1.8947294
ENSBTAG00000037686	0.6471236	0.1579261	0.801546
ENSBTAG00000037687	NA	NA	NA
ENSBTAG00000037699	NA	NA	NA

ENSBTAG00000037702	0.0864739	0.8416689	0.0748587
ENSBTAG00000037703	0.5629887	0.1321588	0.8789039
ENSBTAG00000037710	NA	NA	NA
ENSBTAG00000037717	-0.078813	0.885608	0.0527585
ENSBTAG00000037718	NA	NA	NA
ENSBTAG00000037726	0.4042394	0.241682	0.6167557
ENSBTAG00000037729	-0.189293	0.6537423	0.1845934
ENSBTAG00000037735	2.3285197	1.42E-07	6.8484708
ENSBTAG00000037743	NA	NA	NA
ENSBTAG00000037746	-0.09592	0.7108303	0.1482341
ENSBTAG00000037756	-0.767936	0.0496802	1.3038165
ENSBTAG00000037757	0.752252	0.2522761	0.598124
ENSBTAG00000037764	NA	NA	NA
ENSBTAG00000037765	-0.129502	0.7698431	0.1135978
ENSBTAG00000037768	NA	NA	NA
ENSBTAG00000037775	1.3006963	0.0209574	1.6786625
ENSBTAG00000037778	-0.929859	0.0822651	1.0847843
ENSBTAG00000037781	0.3978528	0.3841227	0.41553
ENSBTAG00000037783	NA	NA	NA
ENSBTAG00000037786	NA	NA	NA
ENSBTAG00000037794	-0.340742	0.3190615	0.4961256
ENSBTAG00000037795	NA	NA	NA
ENSBTAG00000037799	0.4539013	0.130926	0.8829742
ENSBTAG00000037803	-0.099452	0.7737954	0.1113739
ENSBTAG00000037804	-0.264189	0.3786008	0.4218184
ENSBTAG00000037811	0.0211851	0.9641943	0.0158354
ENSBTAG00000037812	NA	NA	NA
ENSBTAG00000037813	0.1496285	0.5699739	0.244145
ENSBTAG00000037819	0.415739	0.1243066	0.9055059
ENSBTAG00000037821	NA	NA	NA
ENSBTAG00000037826	NA	NA	NA
ENSBTAG00000037830	NA	NA	NA
ENSBTAG00000037832	NA	NA	NA
ENSBTAG00000037842	NA	NA	NA
ENSBTAG00000037844	NA	NA	NA
ENSBTAG00000037858	-0.506638	0.3670314	0.4352968
ENSBTAG00000037881	NA	NA	NA
ENSBTAG00000037882	0.1107535	0.8097027	0.0916744
ENSBTAG00000037885	NA	NA	NA
ENSBTAG00000037890	NA	NA	NA
ENSBTAG00000037896	NA	NA	NA
ENSBTAG00000037899	1.2366344	0.0043615	2.3603599
ENSBTAG00000037902	NA	NA	NA
ENSBTAG00000037906	NA	NA	NA
ENSBTAG00000037907	0.688091	0.130946	0.8829078
ENSBTAG00000037908	NA	NA	NA
ENSBTAG00000037917	NA	NA	NA
ENSBTAG00000037924	NA	NA	NA
ENSBTAG00000037927	NA	NA	NA
ENSBTAG00000037931	NA	NA	NA
ENSBTAG00000037932	NA	NA	NA
ENSBTAG00000037935	0.4770046	0.2186904	0.6601704
ENSBTAG00000037937	NA	NA	NA
ENSBTAG00000037938	-0.175956	0.6283068	0.2018282
ENSBTAG00000037941	0.3117946	0.382348	0.4175412
ENSBTAG00000037942	NA	NA	NA
ENSBTAG00000037945	NA	NA	NA
ENSBTAG00000037951	-0.289894	0.5479006	0.2612982

ENSBTAG00000037959	NA	NA	NA
ENSBTAG00000037962	1.1809842	0.0039689	2.4013271
ENSBTAG00000037964	1.2171191	0.024797	1.6056011
ENSBTAG00000037965	NA	NA	NA
ENSBTAG00000037968	NA	NA	NA
ENSBTAG00000037972	0.1004155	0.7006123	0.1545222
ENSBTAG00000037977	NA	NA	NA
ENSBTAG00000037980	-0.091092	0.8009963	0.0963695
ENSBTAG00000037981	0.3837628	0.4634958	0.3339542
ENSBTAG00000037983	NA	NA	NA
ENSBTAG00000037986	NA	NA	NA
ENSBTAG00000037988	NA	NA	NA
ENSBTAG00000037989	NA	NA	NA
ENSBTAG00000037991	0.2807598	0.4550424	0.3419482
ENSBTAG00000037996	-0.103989	0.7725385	0.1120799
ENSBTAG00000038011	-0.520495	0.1572022	0.8035414
ENSBTAG00000038020	NA	NA	NA
ENSBTAG00000038025	0.2162696	0.3967112	0.4015255
ENSBTAG00000038030	NA	NA	NA
ENSBTAG00000038031	NA	NA	NA
ENSBTAG00000038034	0.2501944	0.4708741	0.3270952
ENSBTAG00000038042	NA	NA	NA
ENSBTAG00000038043	NA	NA	NA
ENSBTAG00000038045	NA	NA	NA
ENSBTAG00000038047	0.2734765	0.5501648	0.2595072
ENSBTAG00000038048	-1.499665	0.0011179	2.9515807
ENSBTAG00000038050	-0.009365	0.9827179	0.0075711
ENSBTAG00000038051	0.0632283	0.8058646	0.0937379
ENSBTAG00000038055	-0.407015	0.3371242	0.47221
ENSBTAG00000038058	NA	NA	NA
ENSBTAG00000038062	-0.142424	0.7825581	0.1064834
ENSBTAG00000038063	NA	NA	NA
ENSBTAG00000038064	NA	NA	NA
ENSBTAG00000038067	4.0584832	1.13E-05	4.9468716
ENSBTAG00000038069	NA	NA	NA
ENSBTAG00000038074	NA	NA	NA
ENSBTAG00000038079	-0.286691	0.3647826	0.4379659
ENSBTAG00000038080	NA	NA	NA
ENSBTAG00000038084	NA	NA	NA
ENSBTAG00000038085	0.4649822	0.2859679	0.5436827
ENSBTAG00000038088	-0.019441	0.9493341	0.0225809
ENSBTAG00000038093	0.758639	0.0951261	1.0217003
ENSBTAG00000038098	NA	NA	NA
ENSBTAG00000038104	0.2867417	0.2598461	0.5852837
ENSBTAG00000038107	0.1201952	0.6648926	0.1772485
ENSBTAG00000038112	1.0562599	0.0929703	1.031656
ENSBTAG00000038115	0.1221439	0.7911415	0.1017458
ENSBTAG00000038116	0.2071035	0.4996834	0.3013051
ENSBTAG00000038117	-0.191101	0.4972495	0.3034257
ENSBTAG00000038124	NA	NA	NA
ENSBTAG00000038126	0.2010851	0.5543819	0.2561909
ENSBTAG00000038128	-0.901797	0.4212849	0.3754242
ENSBTAG00000038131	NA	NA	NA
ENSBTAG00000038132	NA	NA	NA
ENSBTAG00000038134	0.8786543	0.0273529	1.5629965
ENSBTAG00000038138	NA	NA	NA
ENSBTAG00000038139	0.1165983	0.7946487	0.0998248
ENSBTAG00000038141	0.0148713	0.9682872	0.0139958

ENSBTAG00000038148	NA	NA	NA
ENSBTAG00000038149	NA	NA	NA
ENSBTAG00000038151	-0.020653	0.94582	0.0241915
ENSBTAG00000038154	NA	NA	NA
ENSBTAG00000038156	-0.188559	0.493218	0.3069611
ENSBTAG00000038168	NA	NA	NA
ENSBTAG00000038171	NA	NA	NA
ENSBTAG00000038173	0.4174677	0.2713729	0.5664335
ENSBTAG00000038178	-0.008127	0.9802	0.0086853
ENSBTAG00000038180	NA	NA	NA
ENSBTAG00000038181	-0.170512	0.4997772	0.3012236
ENSBTAG00000038186	-0.020289	0.9450944	0.0245248
ENSBTAG00000038190	-0.048249	0.8669636	0.0619991
ENSBTAG00000038195	0.2697668	0.3683544	0.4337341
ENSBTAG00000038209	NA	NA	NA
ENSBTAG00000038215	NA	NA	NA
ENSBTAG00000038221	NA	NA	NA
ENSBTAG00000038228	-0.029657	0.9322193	0.0304819
ENSBTAG00000038232	NA	NA	NA
ENSBTAG00000038233	NA	NA	NA
ENSBTAG00000038234	NA	NA	NA
ENSBTAG00000038235	-0.329981	0.5001436	0.3009053
ENSBTAG00000038238	NA	NA	NA
ENSBTAG00000038240	-0.313922	0.3339424	0.4763284
ENSBTAG00000038241	0.3941781	0.1620282	0.7904093
ENSBTAG00000038251	0.0026496	0.9926322	0.0032116
ENSBTAG00000038257	NA	NA	NA
ENSBTAG00000038258	NA	NA	NA
ENSBTAG00000038261	NA	NA	NA
ENSBTAG00000038263	NA	NA	NA
ENSBTAG00000038277	NA	NA	NA
ENSBTAG00000038278	NA	NA	NA
ENSBTAG00000038281	NA	NA	NA
ENSBTAG00000038283	-0.197074	0.5188429	0.2849641
ENSBTAG00000038284	0.2214369	0.491381	0.3085816
ENSBTAG00000038286	0.9316137	0.0141579	1.8490005
ENSBTAG00000038288	NA	NA	NA
ENSBTAG00000038292	NA	NA	NA
ENSBTAG00000038298	0.1210138	0.7276297	0.1380896
ENSBTAG00000038306	NA	NA	NA
ENSBTAG00000038316	-0.140353	0.5897846	0.2293066
ENSBTAG00000038317	NA	NA	NA
ENSBTAG00000038318	NA	NA	NA
ENSBTAG00000038321	1.2277756	0.0002579	3.5885756
ENSBTAG00000038322	NA	NA	NA
ENSBTAG00000038323	NA	NA	NA
ENSBTAG00000038325	NA	NA	NA
ENSBTAG00000038327	NA	NA	NA
ENSBTAG00000038330	-0.303973	0.287806	0.5409001
ENSBTAG00000038333	-0.036214	0.9057599	0.0429869
ENSBTAG00000038335	-0.222649	0.5822725	0.2348737
ENSBTAG00000038340	NA	NA	NA
ENSBTAG00000038345	NA	NA	NA
ENSBTAG00000038351	NA	NA	NA
ENSBTAG00000038361	NA	NA	NA
ENSBTAG00000038364	NA	NA	NA
ENSBTAG00000038366	NA	NA	NA
ENSBTAG00000038368	NA	NA	NA

ENSBTAG00000038375	0.3521774	0.1841378	0.7348572
ENSBTAG00000038379	0.1755458	0.6901065	0.1610839
ENSBTAG00000038381	0.3348836	0.3520482	0.4533978
ENSBTAG00000038386	NA	NA	NA
ENSBTAG00000038387	NA	NA	NA
ENSBTAG00000038409	-0.27152	0.3873394	0.4119084
ENSBTAG00000038412	NA	NA	NA
ENSBTAG00000038416	NA	NA	NA
ENSBTAG00000038426	NA	NA	NA
ENSBTAG00000038428	-0.201316	0.6642117	0.1776935
ENSBTAG00000038433	NA	NA	NA
ENSBTAG00000038434	-0.276196	0.2672602	0.5730657
ENSBTAG00000038438	NA	NA	NA
ENSBTAG00000038439	-0.117915	0.6679856	0.1752329
ENSBTAG00000038442	NA	NA	NA
ENSBTAG00000038448	NA	NA	NA
ENSBTAG00000038461	NA	NA	NA
ENSBTAG00000038462	0.2586568	0.4228937	0.3737688
ENSBTAG00000038464	1.0389955	0.0009335	3.0299001
ENSBTAG00000038476	NA	NA	NA
ENSBTAG00000038477	0.168992	0.5609275	0.2510933
ENSBTAG00000038480	-0.142342	0.6996547	0.1551162
ENSBTAG00000038486	NA	NA	NA
ENSBTAG00000038487	0.1868322	0.5707663	0.2435417
ENSBTAG00000038488	0.2641866	0.3017779	0.5203126
ENSBTAG00000038490	NA	NA	NA
ENSBTAG00000038494	NA	NA	NA
ENSBTAG00000038495	0.6319592	0.0852929	1.0690872
ENSBTAG00000038496	0.1968566	0.6108894	0.2140374
ENSBTAG00000038497	NA	NA	NA
ENSBTAG00000038498	0.3039879	0.4864806	0.3129344
ENSBTAG00000038500	-0.199792	0.6847863	0.1644449
ENSBTAG00000038502	NA	NA	NA
ENSBTAG00000038504	NA	NA	NA
ENSBTAG00000038520	NA	NA	NA
ENSBTAG00000038523	NA	NA	NA
ENSBTAG00000038527	-0.331134	0.42246	0.3742144
ENSBTAG00000038531	NA	NA	NA
ENSBTAG00000038532	NA	NA	NA
ENSBTAG00000038534	NA	NA	NA
ENSBTAG00000038536	0.4922287	0.1213404	0.9159945
ENSBTAG00000038540	-0.573407	0.1199683	0.9209334
ENSBTAG00000038541	-0.751653	0.0998224	1.0007719
ENSBTAG00000038548	NA	NA	NA
ENSBTAG00000038549	NA	NA	NA
ENSBTAG00000038565	NA	NA	NA
ENSBTAG00000038576	NA	NA	NA
ENSBTAG00000038577	NA	NA	NA
ENSBTAG00000038584	-0.544592	0.2445153	0.611694
ENSBTAG00000038598	NA	NA	NA
ENSBTAG00000038600	NA	NA	NA
ENSBTAG00000038604	0.0829881	0.844195	0.0735572
ENSBTAG00000038606	NA	NA	NA
ENSBTAG00000038610	NA	NA	NA
ENSBTAG00000038613	NA	NA	NA
ENSBTAG00000038617	0.6975153	0.3019599	0.5200507
ENSBTAG00000038619	0.31027	0.276503	0.5583002
ENSBTAG00000038620	-0.192861	0.4962312	0.304316

ENSBTAG00000038625	-0.172239	0.7322563	0.1353369
ENSBTAG00000038627	NA	NA	NA
ENSBTAG00000038629	NA	NA	NA
ENSBTAG00000038630	NA	NA	NA
ENSBTAG00000038635	0.1153989	0.7101999	0.1486194
ENSBTAG00000038636	NA	NA	NA
ENSBTAG00000038639	NA	NA	NA
ENSBTAG00000038640	-0.340357	0.3368883	0.4725141
ENSBTAG00000038650	NA	NA	NA
ENSBTAG00000038652	0.9515542	0.0003452	3.4619531
ENSBTAG00000038659	NA	NA	NA
ENSBTAG00000038662	NA	NA	NA
ENSBTAG00000038665	NA	NA	NA
ENSBTAG00000038670	NA	NA	NA
ENSBTAG00000038674	0.4030041	0.3822407	0.4176631
ENSBTAG00000038682	NA	NA	NA
ENSBTAG00000038691	-0.082027	0.7996249	0.0971137
ENSBTAG00000038696	-0.026505	0.9541485	0.020384
ENSBTAG00000038698	NA	NA	NA
ENSBTAG00000038700	0.4060995	0.279722	0.5532733
ENSBTAG00000038702	NA	NA	NA
ENSBTAG00000038706	NA	NA	NA
ENSBTAG00000038710	-0.049298	0.865259	0.0628539
ENSBTAG00000038711	NA	NA	NA
ENSBTAG00000038715	NA	NA	NA
ENSBTAG00000038716	-0.057583	0.8550268	0.0680203
ENSBTAG00000038732	NA	NA	NA
ENSBTAG00000038733	NA	NA	NA
ENSBTAG00000038735	-0.174395	0.7336776	0.1344948
ENSBTAG00000038737	NA	NA	NA
ENSBTAG00000038738	0.3666238	0.3154939	0.501009
ENSBTAG00000038739	NA	NA	NA
ENSBTAG00000038745	-0.234281	0.4141045	0.38289
ENSBTAG00000038748	NA	NA	NA
ENSBTAG00000038756	NA	NA	NA
ENSBTAG00000038759	NA	NA	NA
ENSBTAG00000038770	0.1902799	0.5712549	0.24317
ENSBTAG00000038777	NA	NA	NA
ENSBTAG00000038782	NA	NA	NA
ENSBTAG00000038783	NA	NA	NA
ENSBTAG00000038794	-0.552124	0.0335027	1.47492
ENSBTAG00000038795	0.148734	0.6704775	0.1736158
ENSBTAG00000038797	NA	NA	NA
ENSBTAG00000038806	NA	NA	NA
ENSBTAG00000038815	0.3585941	0.277882	0.5561395
ENSBTAG00000038831	-0.031353	0.9193531	0.0365176
ENSBTAG00000038835	NA	NA	NA
ENSBTAG00000038842	-0.043313	0.8743308	0.0583242
ENSBTAG00000038844	0.2514559	0.5549122	0.2557757
ENSBTAG00000038845	0.8855314	0.0323252	1.4904593
ENSBTAG00000038849	-0.744813	0.0157073	1.8038987
ENSBTAG00000038854	NA	NA	NA
ENSBTAG00000038865	-0.132088	0.5964919	0.2243954
ENSBTAG00000038866	0.0859528	0.7772799	0.1094226
ENSBTAG00000038869	0.1413361	0.6640854	0.1777761
ENSBTAG00000038878	NA	NA	NA
ENSBTAG00000038891	NA	NA	NA
ENSBTAG00000038893	NA	NA	NA

ENSBTAG00000038896	0.0546452	0.8284237	0.0817475
ENSBTAG00000038900	NA	NA	NA
ENSBTAG00000038903	NA	NA	NA
ENSBTAG00000038904	NA	NA	NA
ENSBTAG00000038910	NA	NA	NA
ENSBTAG00000038916	0.2194993	0.6635774	0.1781084
ENSBTAG00000038920	-0.371696	0.1353475	0.8685499
ENSBTAG00000038926	-0.188475	0.6792496	0.1679706
ENSBTAG00000038928	NA	NA	NA
ENSBTAG00000038929	-0.311289	0.5137453	0.2892521
ENSBTAG00000038930	NA	NA	NA
ENSBTAG00000038931	-0.041569	0.9244436	0.0341196
ENSBTAG00000038933	NA	NA	NA
ENSBTAG00000038938	2.7822804	0.0009226	3.0349936
ENSBTAG00000038943	NA	NA	NA
ENSBTAG00000038949	-0.08919	0.7574009	0.1206742
ENSBTAG00000038951	0.3320573	0.3163003	0.4999004
ENSBTAG00000038955	NA	NA	NA
ENSBTAG00000038960	NA	NA	NA
ENSBTAG00000038970	NA	NA	NA
ENSBTAG00000038974	NA	NA	NA
ENSBTAG00000038978	NA	NA	NA
ENSBTAG00000038979	0.2842675	0.3339787	0.4762812
ENSBTAG00000038981	NA	NA	NA
ENSBTAG00000038982	NA	NA	NA
ENSBTAG00000038985	NA	NA	NA
ENSBTAG00000038990	0.2797053	0.5160123	0.28734
ENSBTAG00000038992	0.6154017	0.1018048	0.9922318
ENSBTAG00000038993	NA	NA	NA
ENSBTAG00000039012	NA	NA	NA
ENSBTAG00000039014	0.2935205	0.4356165	0.3608956
ENSBTAG00000039015	0.0067411	1	0
ENSBTAG00000039016	NA	NA	NA
ENSBTAG00000039023	NA	NA	NA
ENSBTAG00000039028	0.8614331	0.4606536	0.3366255
ENSBTAG00000039035	-0.311494	0.6316486	0.1995245
ENSBTAG00000039041	NA	NA	NA
ENSBTAG00000039046	NA	NA	NA
ENSBTAG00000039049	NA	NA	NA
ENSBTAG00000039050	-0.82712	0.0061364	2.2120877
ENSBTAG00000039055	NA	NA	NA
ENSBTAG00000039059	NA	NA	NA
ENSBTAG00000039065	NA	NA	NA
ENSBTAG00000039067	NA	NA	NA
ENSBTAG00000039068	NA	NA	NA
ENSBTAG00000039071	-0.377408	0.2363114	0.6265153
ENSBTAG00000039075	-0.024237	0.9466985	0.0237883
ENSBTAG00000039077	NA	NA	NA
ENSBTAG00000039079	NA	NA	NA
ENSBTAG00000039080	NA	NA	NA
ENSBTAG00000039086	NA	NA	NA
ENSBTAG00000039090	-0.24131	0.4980339	0.3027411
ENSBTAG00000039091	0.4386917	0.5030039	0.2984286
ENSBTAG00000039105	NA	NA	NA
ENSBTAG00000039117	0.204587	0.436935	0.3595831
ENSBTAG00000039121	-0.028653	0.9162585	0.037982
ENSBTAG00000039129	-0.180912	0.7031215	0.1529696
ENSBTAG00000039130	NA	NA	NA

ENSBTAG00000039132	-0.21642	0.6608245	0.1799138
ENSBTAG00000039133	NA	NA	NA
ENSBTAG00000039138	0.0655884	0.8093564	0.0918602
ENSBTAG00000039141	NA	NA	NA
ENSBTAG00000039145	NA	NA	NA
ENSBTAG00000039151	NA	NA	NA
ENSBTAG00000039153	-0.190562	0.5890428	0.2298532
ENSBTAG00000039157	NA	NA	NA
ENSBTAG00000039160	-0.103	0.8333254	0.0791854
ENSBTAG00000039161	0.1164507	0.6570654	0.1823914
ENSBTAG00000039162	0.6932809	0.1524783	0.8167918
ENSBTAG00000039163	-0.762358	0.0033801	2.4710721
ENSBTAG00000039172	-0.106071	0.6711954	0.173151
ENSBTAG00000039178	NA	NA	NA
ENSBTAG00000039182	NA	NA	NA
ENSBTAG00000039190	-0.941434	0.0027081	2.5673297
ENSBTAG00000039194	NA	NA	NA
ENSBTAG00000039195	NA	NA	NA
ENSBTAG00000039196	NA	NA	NA
ENSBTAG00000039197	0.3871319	0.4009867	0.39687
ENSBTAG00000039208	-0.183377	0.5209946	0.2831668
ENSBTAG00000039212	NA	NA	NA
ENSBTAG00000039213	NA	NA	NA
ENSBTAG00000039215	NA	NA	NA
ENSBTAG00000039223	NA	NA	NA
ENSBTAG00000039231	0.2137166	0.5148959	0.2882806
ENSBTAG00000039237	NA	NA	NA
ENSBTAG00000039242	0.2694123	0.4869363	0.3125278
ENSBTAG00000039245	NA	NA	NA
ENSBTAG00000039246	0.3997012	0.2530277	0.596832
ENSBTAG00000039256	NA	NA	NA
ENSBTAG00000039257	NA	NA	NA
ENSBTAG00000039263	NA	NA	NA
ENSBTAG00000039274	NA	NA	NA
ENSBTAG00000039275	0.3424966	0.41672	0.3801556
ENSBTAG00000039276	NA	NA	NA
ENSBTAG00000039287	-0.371466	0.2218481	0.6539443
ENSBTAG00000039289	NA	NA	NA
ENSBTAG00000039299	NA	NA	NA
ENSBTAG00000039302	NA	NA	NA
ENSBTAG00000039307	-0.291454	0.3250393	0.4880641
ENSBTAG00000039310	NA	NA	NA
ENSBTAG00000039313	0.0361055	0.947874	0.0232494
ENSBTAG00000039319	NA	NA	NA
ENSBTAG00000039321	NA	NA	NA
ENSBTAG00000039326	-0.169848	0.7222431	0.1413166
ENSBTAG00000039328	NA	NA	NA
ENSBTAG00000039329	1.5885431	0.0248754	1.6042298
ENSBTAG00000039334	NA	NA	NA
ENSBTAG00000039335	0.3386257	0.3098841	0.5088007
ENSBTAG00000039337	NA	NA	NA
ENSBTAG00000039340	-0.065603	0.822748	0.0847331
ENSBTAG00000039341	-0.78618	0.2911183	0.5359305
ENSBTAG00000039343	NA	NA	NA
ENSBTAG00000039346	NA	NA	NA
ENSBTAG00000039347	NA	NA	NA
ENSBTAG00000039353	NA	NA	NA
ENSBTAG00000039354	NA	NA	NA

ENSBTAG00000039356	-0.532791	0.2198214	0.65793
ENSBTAG00000039362	NA	NA	NA
ENSBTAG00000039366	-0.748798	0.0631252	1.1997974
ENSBTAG00000039370	NA	NA	NA
ENSBTAG00000039374	-0.22558	0.36894	0.4330443
ENSBTAG00000039377	NA	NA	NA
ENSBTAG00000039380	NA	NA	NA
ENSBTAG00000039384	NA	NA	NA
ENSBTAG00000039391	NA	NA	NA
ENSBTAG00000039401	NA	NA	NA
ENSBTAG00000039402	NA	NA	NA
ENSBTAG00000039413	NA	NA	NA
ENSBTAG00000039415	0.3307149	0.378368	0.4220856
ENSBTAG00000039425	NA	NA	NA
ENSBTAG00000039426	NA	NA	NA
ENSBTAG00000039433	NA	NA	NA
ENSBTAG00000039434	NA	NA	NA
ENSBTAG00000039435	-0.392503	0.186695	0.7288674
ENSBTAG00000039440	-0.611649	0.542076	0.2659398
ENSBTAG00000039441	NA	NA	NA
ENSBTAG00000039442	-0.077046	0.7570352	0.1208839
ENSBTAG00000039444	NA	NA	NA
ENSBTAG00000039446	NA	NA	NA
ENSBTAG00000039448	NA	NA	NA
ENSBTAG00000039453	0.782129	0.1014392	0.993794
ENSBTAG00000039456	0.1045484	0.7111373	0.1480465
ENSBTAG00000039462	NA	NA	NA
ENSBTAG00000039466	0.3553952	0.4968819	0.3037468
ENSBTAG00000039467	NA	NA	NA
ENSBTAG00000039470	NA	NA	NA
ENSBTAG00000039477	NA	NA	NA
ENSBTAG00000039486	-0.278816	0.5774618	0.2384768
ENSBTAG00000039491	NA	NA	NA
ENSBTAG00000039492	NA	NA	NA
ENSBTAG00000039493	-0.140516	0.6864901	0.1633657
ENSBTAG00000039499	NA	NA	NA
ENSBTAG00000039504	NA	NA	NA
ENSBTAG00000039509	NA	NA	NA
ENSBTAG00000039512	0.6587397	0.1413243	0.8497831
ENSBTAG00000039513	NA	NA	NA
ENSBTAG00000039520	NA	NA	NA
ENSBTAG00000039523	-0.895897	0.2035452	0.6913391
ENSBTAG00000039524	NA	NA	NA
ENSBTAG00000039529	NA	NA	NA
ENSBTAG00000039530	NA	NA	NA
ENSBTAG00000039531	NA	NA	NA
ENSBTAG00000039534	NA	NA	NA
ENSBTAG00000039540	NA	NA	NA
ENSBTAG00000039552	0.0169146	0.9750463	0.0109747
ENSBTAG00000039555	-0.132126	0.7718534	0.1124652
ENSBTAG00000039556	0.3048666	0.2723346	0.5648973
ENSBTAG00000039559	NA	NA	NA
ENSBTAG00000039563	0.4208692	0.3706809	0.4309998
ENSBTAG00000039571	0.7629374	0.0996481	1.001531
ENSBTAG00000039573	-0.000481	0.9989907	0.0004385
ENSBTAG00000039574	-1.136995	0.0340917	1.4673517
ENSBTAG00000039581	NA	NA	NA
ENSBTAG00000039582	NA	NA	NA

ENSBTAG00000039587	NA	NA	NA
ENSBTAG00000039588	NA	NA	NA
ENSBTAG00000039591	-0.421426	0.1331452	0.8756745
ENSBTAG00000039593	-0.689006	0.0621906	1.2062755
ENSBTAG00000039594	0.7003706	0.0485857	1.3134918
ENSBTAG00000039597	NA	NA	NA
ENSBTAG00000039599	0.7526642	0.1828382	0.737933
ENSBTAG00000039601	NA	NA	NA
ENSBTAG00000039618	-0.282297	0.7863271	0.1043968
ENSBTAG00000039620	NA	NA	NA
ENSBTAG00000039630	-0.135108	0.6132005	0.2123975
ENSBTAG00000039634	0.3250972	0.2313399	0.6357494
ENSBTAG00000039635	0.0910423	0.8047946	0.0943149
ENSBTAG00000039643	-3.226554	2.47E-09	8.6079682
ENSBTAG00000039644	NA	NA	NA
ENSBTAG00000039647	NA	NA	NA
ENSBTAG00000039652	NA	NA	NA
ENSBTAG00000039657	-0.239805	0.38922	0.4098049
ENSBTAG00000039658	-0.007554	0.9837222	0.0071275
ENSBTAG00000039662	NA	NA	NA
ENSBTAG00000039682	-0.239419	0.3548778	0.4499212
ENSBTAG00000039684	-0.056101	0.8382715	0.0766153
ENSBTAG00000039686	0.5339415	0.1777931	0.7500851
ENSBTAG00000039688	1.4798298	0.00214	2.6695899
ENSBTAG00000039691	NA	NA	NA
ENSBTAG00000039695	0.050457	0.9105469	0.0406977
ENSBTAG00000039696	NA	NA	NA
ENSBTAG00000039702	NA	NA	NA
ENSBTAG00000039705	0.4067204	0.3127736	0.5047699
ENSBTAG00000039708	0.8489351	0.162562	0.7889809
ENSBTAG00000039709	NA	NA	NA
ENSBTAG00000039711	1.6780453	0.0165249	1.7818609
ENSBTAG00000039714	NA	NA	NA
ENSBTAG00000039719	0.478514	0.3170688	0.4988466
ENSBTAG00000039720	-0.361541	0.3382621	0.4707466
ENSBTAG00000039722	NA	NA	NA
ENSBTAG00000039727	-0.321926	0.5670646	0.2463675
ENSBTAG00000039728	-0.292374	0.3331373	0.4773767
ENSBTAG00000039731	-0.074585	0.813531	0.0896259
ENSBTAG00000039732	0.0524505	0.8632826	0.063847
ENSBTAG00000039738	NA	NA	NA
ENSBTAG00000039740	NA	NA	NA
ENSBTAG00000039757	NA	NA	NA
ENSBTAG00000039764	0.6582816	0.6244944	0.2044715
ENSBTAG00000039766	0.4085669	0.2311074	0.6361861
ENSBTAG00000039770	-0.142646	0.5639453	0.248763
ENSBTAG00000039772	NA	NA	NA
ENSBTAG00000039782	0.5653197	0.1556818	0.8077623
ENSBTAG00000039784	-0.310433	0.3039101	0.5172549
ENSBTAG00000039789	NA	NA	NA
ENSBTAG00000039793	-0.248356	0.3317238	0.4792234
ENSBTAG00000039794	0.3694509	0.3790304	0.421326
ENSBTAG00000039803	0.1508488	0.7749616	0.1107198
ENSBTAG00000039807	NA	NA	NA
ENSBTAG00000039812	0.49838	0.1191069	0.9240633
ENSBTAG00000039813	NA	NA	NA
ENSBTAG00000039815	NA	NA	NA
ENSBTAG00000039817	NA	NA	NA

ENSBTAG00000039819	-0.026547	0.9508047	0.0219087
ENSBTAG00000039823	1.3847488	0.0332336	1.4784231
ENSBTAG00000039825	NA	NA	NA
ENSBTAG00000039834	NA	NA	NA
ENSBTAG00000039839	NA	NA	NA
ENSBTAG00000039845	NA	NA	NA
ENSBTAG00000039847	NA	NA	NA
ENSBTAG00000039849	NA	NA	NA
ENSBTAG00000039850	NA	NA	NA
ENSBTAG00000039851	0.0782514	0.7598921	0.1192481
ENSBTAG00000039855	0.2867032	0.3103468	0.5081527
ENSBTAG00000039861	0.9533623	0.0931498	1.0308179
ENSBTAG00000039868	NA	NA	NA
ENSBTAG00000039870	NA	NA	NA
ENSBTAG00000039875	1.4300605	0.3185693	0.496796
ENSBTAG00000039886	-0.152182	0.6861102	0.1636061
ENSBTAG00000039888	-0.285163	0.239422	0.620836
ENSBTAG00000039890	NA	NA	NA
ENSBTAG00000039892	NA	NA	NA
ENSBTAG00000039893	NA	NA	NA
ENSBTAG00000039904	NA	NA	NA
ENSBTAG00000039914	NA	NA	NA
ENSBTAG00000039916	-0.397462	0.1140805	0.9427884
ENSBTAG00000039922	-0.632593	0.0877472	1.0567667
ENSBTAG00000039928	NA	NA	NA
ENSBTAG00000039929	NA	NA	NA
ENSBTAG00000039932	NA	NA	NA
ENSBTAG00000039933	NA	NA	NA
ENSBTAG00000039935	NA	NA	NA
ENSBTAG00000039937	NA	NA	NA
ENSBTAG00000039943	0.1577628	0.6276699	0.2022687
ENSBTAG00000039946	NA	NA	NA
ENSBTAG00000039950	NA	NA	NA
ENSBTAG00000039951	0.3350632	0.3389177	0.4699058
ENSBTAG00000039954	0.0042979	0.9958438	0.0018088
ENSBTAG00000039958	0.1658197	0.5165694	0.2868713
ENSBTAG00000039962	-0.105852	0.8149368	0.0888761
ENSBTAG00000039967	NA	NA	NA
ENSBTAG00000039968	-0.121171	0.7053567	0.1515912
ENSBTAG00000039973	NA	NA	NA
ENSBTAG00000039980	NA	NA	NA
ENSBTAG00000039992	-0.51933	0.1913055	0.7182725
ENSBTAG00000039993	-0.236729	0.4662897	0.3313441
ENSBTAG00000039994	NA	NA	NA
ENSBTAG00000039995	0.0894539	0.7225238	0.1411478
ENSBTAG00000040000	NA	NA	NA
ENSBTAG00000040001	-0.067777	0.9036976	0.0439769
ENSBTAG00000040005	NA	NA	NA
ENSBTAG00000040006	-0.181806	0.4934525	0.3067547
ENSBTAG00000040023	NA	NA	NA
ENSBTAG00000040026	NA	NA	NA
ENSBTAG00000040028	0.1555546	0.6153723	0.2108621
ENSBTAG00000040031	-0.056406	0.8222088	0.0850179
ENSBTAG00000040034	NA	NA	NA
ENSBTAG00000040038	NA	NA	NA
ENSBTAG00000040042	NA	NA	NA
ENSBTAG00000040043	NA	NA	NA
ENSBTAG00000040046	0.0183757	0.9665703	0.0147665

ENSBTAG00000040051	NA	NA	NA
ENSBTAG00000040053	0.7033519	0.1523235	0.8172331
ENSBTAG00000040055	0.0590881	0.8116501	0.0906311
ENSBTAG00000040056	NA	NA	NA
ENSBTAG00000040058	0.4730092	0.2625632	0.5807661
ENSBTAG00000040061	0.2633372	0.4037497	0.3938878
ENSBTAG00000040063	0.1981496	0.6015473	0.2207302
ENSBTAG00000040064	NA	NA	NA
ENSBTAG00000040065	NA	NA	NA
ENSBTAG00000040067	NA	NA	NA
ENSBTAG00000040072	0.0361162	0.9097233	0.0410907
ENSBTAG00000040076	0.0244316	0.9236539	0.0344907
ENSBTAG00000040078	NA	NA	NA
ENSBTAG00000040082	-0.207091	0.6413039	0.1929361
ENSBTAG00000040083	NA	NA	NA
ENSBTAG00000040086	NA	NA	NA
ENSBTAG00000040088	NA	NA	NA
ENSBTAG00000040098	0.1538974	0.9181182	0.0371014
ENSBTAG00000040103	NA	NA	NA
ENSBTAG00000040106	NA	NA	NA
ENSBTAG00000040108	0.2558031	0.5286568	0.2768262
ENSBTAG00000040109	NA	NA	NA
ENSBTAG00000040111	NA	NA	NA
ENSBTAG00000040116	0.0514198	0.8369864	0.0772816
ENSBTAG00000040126	NA	NA	NA
ENSBTAG00000040128	0.049936	0.8743695	0.058305
ENSBTAG00000040131	0.3667805	0.248652	0.6044081
ENSBTAG00000040132	-0.778889	0.1037695	0.9839304
ENSBTAG00000040133	NA	NA	NA
ENSBTAG00000040144	NA	NA	NA
ENSBTAG00000040147	NA	NA	NA
ENSBTAG00000040151	-0.635869	0.0400529	1.397366
ENSBTAG00000040155	NA	NA	NA
ENSBTAG00000040167	NA	NA	NA
ENSBTAG00000040168	NA	NA	NA
ENSBTAG00000040169	0.2371659	0.5462851	0.2625806
ENSBTAG00000040171	NA	NA	NA
ENSBTAG00000040187	NA	NA	NA
ENSBTAG00000040190	0.2101516	0.6796299	0.1677275
ENSBTAG00000040193	NA	NA	NA
ENSBTAG00000040199	-0.287494	0.3217185	0.492524
ENSBTAG00000040206	0.4906019	0.6511905	0.1862919
ENSBTAG00000040208	NA	NA	NA
ENSBTAG00000040209	0.8027324	0.0851696	1.0697155
ENSBTAG00000040215	-0.057763	0.8191603	0.0866311
ENSBTAG00000040237	NA	NA	NA
ENSBTAG00000040244	0.0785194	0.8839049	0.0535945
ENSBTAG00000040250	NA	NA	NA
ENSBTAG00000040253	NA	NA	NA
ENSBTAG00000040255	NA	NA	NA
ENSBTAG00000040261	NA	NA	NA
ENSBTAG00000040268	NA	NA	NA
ENSBTAG00000040277	0.2317879	0.384416	0.4151985
ENSBTAG00000040279	NA	NA	NA
ENSBTAG00000040282	NA	NA	NA
ENSBTAG00000040290	NA	NA	NA
ENSBTAG00000040295	0.0029542	0.9910292	0.0039135
ENSBTAG00000040296	NA	NA	NA

ENSBTAG00000040298	0.3568138	0.2855836	0.5442667
ENSBTAG00000040304	NA	NA	NA
ENSBTAG00000040308	0.0882787	0.7329762	0.1349102
ENSBTAG00000040313	NA	NA	NA
ENSBTAG00000040316	NA	NA	NA
ENSBTAG00000040323	-0.600106	0.1289341	0.8896323
ENSBTAG00000040330	NA	NA	NA
ENSBTAG00000040331	NA	NA	NA
ENSBTAG00000040333	-0.129371	0.6266597	0.2029682
ENSBTAG00000040336	-0.362902	0.2949681	0.5302249
ENSBTAG00000040337	NA	NA	NA
ENSBTAG00000040338	-0.104266	0.7258282	0.1391662
ENSBTAG00000040347	0.0622696	0.8903222	0.0504528
ENSBTAG00000040350	-0.135949	0.6803378	0.1672754
ENSBTAG00000040351	NA	NA	NA
ENSBTAG00000040356	NA	NA	NA
ENSBTAG00000040358	0.2348388	0.6921445	0.1598032
ENSBTAG00000040360	NA	NA	NA
ENSBTAG00000040361	-0.556577	0.1199293	0.9210749
ENSBTAG00000040368	-0.147943	0.5613787	0.2507441
ENSBTAG00000040378	NA	NA	NA
ENSBTAG00000040381	-0.223286	0.464501	0.3330133
ENSBTAG00000040384	-0.141783	0.585392	0.2325532
ENSBTAG00000040386	NA	NA	NA
ENSBTAG00000040390	0.5284851	0.1331318	0.8757184
ENSBTAG00000040392	0.6273007	0.0865947	1.0625086
ENSBTAG00000040393	NA	NA	NA
ENSBTAG00000040394	NA	NA	NA
ENSBTAG00000040398	-0.567318	0.1161852	0.9348491
ENSBTAG00000040399	NA	NA	NA
ENSBTAG00000040409	NA	NA	NA
ENSBTAG00000040411	0.2137031	0.6001522	0.2217386
ENSBTAG00000040413	NA	NA	NA
ENSBTAG00000040418	NA	NA	NA
ENSBTAG00000040419	NA	NA	NA
ENSBTAG00000040422	-0.177955	0.595937	0.2247996
ENSBTAG00000040427	-0.110602	0.7325609	0.1351563
ENSBTAG00000040432	NA	NA	NA
ENSBTAG00000040435	-0.204191	0.6342236	0.1977576
ENSBTAG00000040442	0.3039359	0.5399623	0.2676365
ENSBTAG00000040445	NA	NA	NA
ENSBTAG00000040460	NA	NA	NA
ENSBTAG00000040461	NA	NA	NA
ENSBTAG00000040473	0.2870535	0.5441686	0.2642665
ENSBTAG00000040477	NA	NA	NA
ENSBTAG00000040482	NA	NA	NA
ENSBTAG00000040490	0.0060233	0.9899405	0.0043909
ENSBTAG00000040494	0.0636409	0.8559534	0.0675499
ENSBTAG00000040496	NA	NA	NA
ENSBTAG00000040504	NA	NA	NA
ENSBTAG00000040507	0.3518907	0.3300092	0.481474
ENSBTAG00000040509	NA	NA	NA
ENSBTAG00000040512	-0.295403	0.6207769	0.2070644
ENSBTAG00000040521	NA	NA	NA
ENSBTAG00000040541	NA	NA	NA
ENSBTAG00000040543	NA	NA	NA
ENSBTAG00000040547	NA	NA	NA
ENSBTAG00000040550	NA	NA	NA

ENSBTAG00000040551	-0.136685	0.7123911	0.1472815
ENSBTAG00000040555	0.5495393	0.2112544	0.6751943
ENSBTAG00000040559	NA	NA	NA
ENSBTAG00000040564	-0.02911	0.9494918	0.0225088
ENSBTAG00000040567	NA	NA	NA
ENSBTAG00000040568	0.6010686	0.0971794	1.0124257
ENSBTAG00000040569	0.3241319	0.4180869	0.3787335
ENSBTAG00000040570	NA	NA	NA
ENSBTAG00000040574	NA	NA	NA
ENSBTAG00000040575	1.3334813	0.0001841	3.73505
ENSBTAG00000040580	NA	NA	NA
ENSBTAG00000040584	NA	NA	NA
ENSBTAG00000040585	NA	NA	NA
ENSBTAG00000040596	NA	NA	NA
ENSBTAG00000040597	-0.126262	0.7456546	0.1274623
ENSBTAG00000040602	-0.27312	0.2808884	0.5514662
ENSBTAG00000040603	0.0381742	0.91611	0.0380524
ENSBTAG00000040605	NA	NA	NA
ENSBTAG00000040607	-0.05555	0.9148433	0.0386533
ENSBTAG00000043546	NA	NA	NA
ENSBTAG00000043550	0.2206279	0.4594255	0.3377849
ENSBTAG00000043553	0.9379011	0.0046203	2.3353332
ENSBTAG00000043556	0.229275	0.3868534	0.4124536
ENSBTAG00000043558	0.1938779	0.5202326	0.2838024
ENSBTAG00000043559	-0.070726	0.8045225	0.0944618
ENSBTAG00000043560	0.2771562	0.3002984	0.5224469
ENSBTAG00000043561	0.2198105	0.5248251	0.2799854
ENSBTAG00000043563	-0.010688	0.9725298	0.0120971
ENSBTAG00000043564	-0.143118	0.6082948	0.2158859
ENSBTAG00000043568	0.3643511	0.1897209	0.7218848
ENSBTAG00000043571	-0.084664	0.7918169	0.1013752
ENSBTAG00000043577	0.0601149	0.8365388	0.0775139
ENSBTAG00000043578	NA	NA	NA
ENSBTAG00000043581	0.2616212	0.4489773	0.3477756
ENSBTAG00000043584	-2.915445	4.12E-06	5.3851945
ENSBTAG00000043948	NA	NA	NA
ENSBTAG00000043949	-0.059768	0.85575	0.0676531
ENSBTAG00000043950	NA	NA	NA
ENSBTAG00000043951	-0.494182	0.2209785	0.65565
ENSBTAG00000043953	NA	NA	NA
ENSBTAG00000043956	0.3179076	0.3602806	0.4433591
ENSBTAG00000043957	NA	NA	NA
ENSBTAG00000043958	0.6247343	0.1439762	0.8417094
ENSBTAG00000043959	1.1867142	0.0056758	2.2459729
ENSBTAG00000043960	-0.061241	0.857174	0.066931
ENSBTAG00000043961	-0.418816	0.1297739	0.8868128
ENSBTAG00000043962	0.3775737	0.4146673	0.3823002
ENSBTAG00000043963	NA	NA	NA
ENSBTAG00000043964	0.1857647	0.4560781	0.3409608
ENSBTAG00000043969	0.4879802	0.3050828	0.5155823
ENSBTAG00000043970	-0.203144	0.4862126	0.3131738
ENSBTAG00000043971	0.2045978	0.5245247	0.2802341
ENSBTAG00000043972	NA	NA	NA
ENSBTAG00000043974	0.2844429	0.2776408	0.5565168
ENSBTAG00000043975	0.7794903	0.0081518	2.088747
ENSBTAG00000043976	-0.244369	0.5727064	0.242068
ENSBTAG00000043979	NA	NA	NA
ENSBTAG00000043981	-0.112607	0.6616092	0.1793985

ENSBTAG00000043985	-0.338663	0.4024405	0.3952983
ENSBTAG00000043987	-0.18244	0.5406283	0.2671012
ENSBTAG00000043989	0.2038357	0.5178253	0.2858167
ENSBTAG00000043990	NA	NA	NA
ENSBTAG00000043991	NA	NA	NA
ENSBTAG00000043992	NA	NA	NA
ENSBTAG00000043993	NA	NA	NA
ENSBTAG00000043994	0.0104113	0.9854919	0.006347
ENSBTAG00000043996	-0.493386	0.3688395	0.4331625
ENSBTAG00000043999	-0.331421	0.1949594	0.7100558
ENSBTAG00000044000	0.0576509	0.8686133	0.0611735
ENSBTAG00000044001	0.1299799	0.6507529	0.1865839
ENSBTAG00000044003	0.0871014	0.7768752	0.1096488
ENSBTAG00000044004	NA	NA	NA
ENSBTAG00000044006	NA	NA	NA
ENSBTAG00000044007	-0.239181	0.6493196	0.1875415
ENSBTAG00000044009	NA	NA	NA
ENSBTAG00000044010	NA	NA	NA
ENSBTAG00000044011	NA	NA	NA
ENSBTAG00000044012	NA	NA	NA
ENSBTAG00000044015	NA	NA	NA
ENSBTAG00000044017	-0.188493	0.4507309	0.3460827
ENSBTAG00000044018	NA	NA	NA
ENSBTAG00000044019	0.7721578	0.0544944	1.2636482
ENSBTAG00000044021	NA	NA	NA
ENSBTAG00000044022	0.5083544	0.2673594	0.5729045
ENSBTAG00000044023	0.1661495	0.5687117	0.2451079
ENSBTAG00000044025	NA	NA	NA
ENSBTAG00000044027	0.6832736	0.1472715	0.8318814
ENSBTAG00000044029	-0.177982	0.5285109	0.2769461
ENSBTAG00000044032	0.3013843	0.2413684	0.6173196
ENSBTAG00000044033	NA	NA	NA
ENSBTAG00000044035	NA	NA	NA
ENSBTAG00000044038	-0.010433	0.9691974	0.0135878
ENSBTAG00000044040	0.1113122	0.6640576	0.1777943
ENSBTAG00000044044	0.3868943	0.1497378	0.8246686
ENSBTAG00000044046	-1.386749	6.17E-08	7.2099239
ENSBTAG00000044047	NA	NA	NA
ENSBTAG00000044048	0.4856804	0.1565496	0.805348
ENSBTAG00000044050	NA	NA	NA
ENSBTAG00000044053	-0.317606	0.3616401	0.4417235
ENSBTAG00000044055	NA	NA	NA
ENSBTAG00000044056	NA	NA	NA
ENSBTAG00000044058	0.0776124	0.7958322	0.0991785
ENSBTAG00000044059	NA	NA	NA
ENSBTAG00000044061	0.328994	0.3049902	0.5157141
ENSBTAG00000044062	0.4840092	0.1739945	0.7594645
ENSBTAG00000044063	0.452843	0.3575502	0.4466629
ENSBTAG00000044064	-0.109095	0.7392053	0.1312349
ENSBTAG00000044065	0.5680051	0.2034716	0.6914963
ENSBTAG00000044066	NA	NA	NA
ENSBTAG00000044067	0.4474899	0.4311237	0.3653981
ENSBTAG00000044068	NA	NA	NA
ENSBTAG00000044070	0.749774	0.1252122	0.9023534
ENSBTAG00000044071	NA	NA	NA
ENSBTAG00000044074	-0.007789	0.9791965	0.0091301
ENSBTAG00000044075	0.2236712	0.4031492	0.3945342
ENSBTAG00000044077	-0.175946	0.7637655	0.11704

ENSBTAG00000044078	NA	NA	NA
ENSBTAG00000044079	-0.271014	0.3805938	0.4195383
ENSBTAG00000044080	NA	NA	NA
ENSBTAG00000044081	-0.216555	0.6049967	0.218247
ENSBTAG00000044082	NA	NA	NA
ENSBTAG00000044083	0.0388905	0.9211393	0.0356747
ENSBTAG00000044087	0.0421025	0.905034	0.0433351
ENSBTAG00000044091	NA	NA	NA
ENSBTAG00000044092	NA	NA	NA
ENSBTAG00000044093	NA	NA	NA
ENSBTAG00000044097	0.034009	0.903616	0.0440161
ENSBTAG00000044099	NA	NA	NA
ENSBTAG00000044100	0.0933145	0.746934	0.1267178
ENSBTAG00000044101	NA	NA	NA
ENSBTAG00000044105	-0.892254	0.0268632	1.570843
ENSBTAG00000044106	0.419778	0.2548084	0.5937862
ENSBTAG00000044111	NA	NA	NA
ENSBTAG00000044112	0.1096786	0.758762	0.1198944
ENSBTAG00000044113	0.0411059	0.9331705	0.030039
ENSBTAG00000044117	NA	NA	NA
ENSBTAG00000044119	NA	NA	NA
ENSBTAG00000044121	0.0944745	0.8045172	0.0944646
ENSBTAG00000044125	-0.599369	0.0425977	1.3706137
ENSBTAG00000044126	-0.093172	0.731951	0.135518
ENSBTAG00000044129	0.1591715	0.6676183	0.1754717
ENSBTAG00000044132	NA	NA	NA
ENSBTAG00000044137	NA	NA	NA
ENSBTAG00000044138	-0.035162	0.9383507	0.0276348
ENSBTAG00000044139	NA	NA	NA
ENSBTAG00000044141	NA	NA	NA
ENSBTAG00000044144	NA	NA	NA
ENSBTAG00000044150	0.7364399	0.0749992	1.1249435
ENSBTAG00000044151	NA	NA	NA
ENSBTAG00000044153	0.0369924	0.8823642	0.0543521
ENSBTAG00000044155	NA	NA	NA
ENSBTAG00000044158	-0.609762	0.1642026	0.78462
ENSBTAG00000044159	-0.03401	0.929935	0.0315474
ENSBTAG00000044160	0.1950771	0.5609687	0.2510614
ENSBTAG00000044161	NA	NA	NA
ENSBTAG00000044167	NA	NA	NA
ENSBTAG00000044169	-0.267824	0.5256698	0.279287
ENSBTAG00000044171	NA	NA	NA
ENSBTAG00000044172	0.2673456	0.5574284	0.2538109
ENSBTAG00000044173	-0.572626	0.024207	1.6160589
ENSBTAG00000044175	NA	NA	NA
ENSBTAG00000044176	NA	NA	NA
ENSBTAG00000044178	-0.204595	0.4922966	0.3077732
ENSBTAG00000044179	NA	NA	NA
ENSBTAG00000044181	NA	NA	NA
ENSBTAG00000044184	0.0822352	0.770255	0.1133655
ENSBTAG00000044185	-0.512919	0.0577543	1.238416
ENSBTAG00000044190	-0.041039	0.9331903	0.0300298
ENSBTAG00000044191	NA	NA	NA
ENSBTAG00000044192	-1.03406	0.3563811	0.4480854
ENSBTAG00000044194	0.3995654	0.2817868	0.5500794
ENSBTAG00000044195	0.509934	0.3321517	0.4786635
ENSBTAG00000044197	NA	NA	NA
ENSBTAG00000044198	0.0030438	0.9950525	0.002154

ENSBTAG00000044202	0.1728688	0.5829463	0.2343715
ENSBTAG00000044204	NA	NA	NA
ENSBTAG00000044207	0.1319032	0.689927	0.1611968
ENSBTAG00000044208	NA	NA	NA
ENSBTAG00000044210	NA	NA	NA
ENSBTAG00000044212	NA	NA	NA
ENSBTAG00000044492	NA	NA	NA
ENSBTAG00000045488	NA	NA	NA
ENSBTAG00000045492	-0.566327	0.3248816	0.488275
ENSBTAG00000045497	0.0250471	0.9477395	0.023311
ENSBTAG00000045498	NA	NA	NA
ENSBTAG00000045500	-0.152741	0.7316154	0.1357172
ENSBTAG00000045504	0.1230364	0.6448976	0.1905093
ENSBTAG00000045507	NA	NA	NA
ENSBTAG00000045509	NA	NA	NA
ENSBTAG00000045510	-0.432391	0.313287	0.5040576
ENSBTAG00000045513	-0.604203	0.1582512	0.8006529
ENSBTAG00000045514	NA	NA	NA
ENSBTAG00000045520	NA	NA	NA
ENSBTAG00000045528	NA	NA	NA
ENSBTAG00000045529	NA	NA	NA
ENSBTAG00000045531	NA	NA	NA
ENSBTAG00000045538	-0.009961	0.9727167	0.0120136
ENSBTAG00000045539	NA	NA	NA
ENSBTAG00000045544	-0.254442	0.3136638	0.5035356
ENSBTAG00000045547	NA	NA	NA
ENSBTAG00000045548	0.2113997	0.5377588	0.2694125
ENSBTAG00000045550	-0.237811	0.4604113	0.336854
ENSBTAG00000045551	NA	NA	NA
ENSBTAG00000045556	NA	NA	NA
ENSBTAG00000045558	NA	NA	NA
ENSBTAG00000045565	0.4739448	0.2353507	0.6282845
ENSBTAG00000045566	NA	NA	NA
ENSBTAG00000045567	NA	NA	NA
ENSBTAG00000045569	NA	NA	NA
ENSBTAG00000045572	NA	NA	NA
ENSBTAG00000045579	NA	NA	NA
ENSBTAG00000045580	NA	NA	NA
ENSBTAG00000045581	-0.611896	0.0751736	1.1239345
ENSBTAG00000045582	0.0970113	0.7202759	0.1425011
ENSBTAG00000045583	NA	NA	NA
ENSBTAG00000045584	0.4419559	0.1257612	0.9004534
ENSBTAG00000045585	NA	NA	NA
ENSBTAG00000045588	2.2251078	4.57E-05	4.3404308
ENSBTAG00000045590	NA	NA	NA
ENSBTAG00000045591	NA	NA	NA
ENSBTAG00000045593	-0.094833	0.9202018	0.0361169
ENSBTAG00000045594	NA	NA	NA
ENSBTAG00000045595	NA	NA	NA
ENSBTAG00000045596	-0.354373	0.3817686	0.4181998
ENSBTAG00000045601	NA	NA	NA
ENSBTAG00000045602	NA	NA	NA
ENSBTAG00000045603	NA	NA	NA
ENSBTAG00000045604	1.192348	0.0001057	3.9757361
ENSBTAG00000045609	NA	NA	NA
ENSBTAG00000045610	NA	NA	NA
ENSBTAG00000045612	NA	NA	NA
ENSBTAG00000045617	NA	NA	NA

ENSBTAG00000045619	NA	NA	NA
ENSBTAG00000045621	NA	NA	NA
ENSBTAG00000045622	0.3310697	0.2426328	0.6150504
ENSBTAG00000045623	NA	NA	NA
ENSBTAG00000045625	NA	NA	NA
ENSBTAG00000045626	NA	NA	NA
ENSBTAG00000045628	-0.382846	0.3504753	0.4553426
ENSBTAG00000045631	NA	NA	NA
ENSBTAG00000045633	NA	NA	NA
ENSBTAG00000045639	NA	NA	NA
ENSBTAG00000045643	NA	NA	NA
ENSBTAG00000045645	NA	NA	NA
ENSBTAG00000045647	NA	NA	NA
ENSBTAG00000045648	-1.416358	0.0032963	2.4819749
ENSBTAG00000045653	NA	NA	NA
ENSBTAG00000045658	NA	NA	NA
ENSBTAG00000045660	NA	NA	NA
ENSBTAG00000045662	NA	NA	NA
ENSBTAG00000045664	0.0149014	0.9617652	0.0169309
ENSBTAG00000045665	NA	NA	NA
ENSBTAG00000045672	NA	NA	NA
ENSBTAG00000045673	NA	NA	NA
ENSBTAG00000045678	NA	NA	NA
ENSBTAG00000045679	NA	NA	NA
ENSBTAG00000045683	NA	NA	NA
ENSBTAG00000045685	-0.600023	0.1547553	0.8103544
ENSBTAG00000045690	NA	NA	NA
ENSBTAG00000045692	0.3132302	0.3993776	0.3986163
ENSBTAG00000045694	NA	NA	NA
ENSBTAG00000045695	NA	NA	NA
ENSBTAG00000045697	NA	NA	NA
ENSBTAG00000045699	-1.008486	9.43E-05	4.0252899
ENSBTAG00000045702	0.2647188	0.7324856	0.1352009
ENSBTAG00000045703	0.2089474	0.4156365	0.3812863
ENSBTAG00000045704	NA	NA	NA
ENSBTAG00000045708	0.3693636	0.4897113	0.3100599
ENSBTAG00000045711	NA	NA	NA
ENSBTAG00000045714	NA	NA	NA
ENSBTAG00000045717	NA	NA	NA
ENSBTAG00000045728	-0.839782	0.0942443	1.025745
ENSBTAG00000045729	-0.059615	0.8467842	0.0722272
ENSBTAG00000045734	NA	NA	NA
ENSBTAG00000045742	NA	NA	NA
ENSBTAG00000045743	NA	NA	NA
ENSBTAG00000045744	0.0137506	0.9781296	0.0096036
ENSBTAG00000045746	-0.552216	0.516251	0.2871391
ENSBTAG00000045748	-0.202454	0.4036466	0.3939987
ENSBTAG00000045750	-0.156138	0.577777	0.2382398
ENSBTAG00000045751	-1.623506	0.0151643	1.8191771
ENSBTAG00000045754	NA	NA	NA
ENSBTAG00000045756	NA	NA	NA
ENSBTAG00000045757	-0.075336	0.7656693	0.1159588
ENSBTAG00000045762	0.5975133	0.1003562	0.9984558
ENSBTAG00000045767	NA	NA	NA
ENSBTAG00000045771	NA	NA	NA
ENSBTAG00000045772	NA	NA	NA
ENSBTAG00000045776	-0.196105	0.5779883	0.238081
ENSBTAG00000045779	-0.251709	0.3184865	0.496909

ENSBTAG00000045784	NA	NA	NA
ENSBTAG00000045785	0.051627	0.8451054	0.0730891
ENSBTAG00000045786	NA	NA	NA
ENSBTAG00000045787	0.2416118	0.5803493	0.2363106
ENSBTAG00000045788	NA	NA	NA
ENSBTAG00000045789	NA	NA	NA
ENSBTAG00000045794	-0.342073	0.1805282	0.743455
ENSBTAG00000045795	NA	NA	NA
ENSBTAG00000045808	NA	NA	NA
ENSBTAG00000045812	NA	NA	NA
ENSBTAG00000045813	NA	NA	NA
ENSBTAG00000045817	0.2308773	0.4087881	0.3885017
ENSBTAG00000045824	NA	NA	NA
ENSBTAG00000045826	NA	NA	NA
ENSBTAG00000045828	-0.071725	0.8172415	0.0876496
ENSBTAG00000045832	-0.086353	0.7494236	0.1252727
ENSBTAG00000045834	NA	NA	NA
ENSBTAG00000045849	NA	NA	NA
ENSBTAG00000045850	NA	NA	NA
ENSBTAG00000045852	NA	NA	NA
ENSBTAG00000045854	NA	NA	NA
ENSBTAG00000045857	-0.150956	0.6033811	0.2194083
ENSBTAG00000045859	NA	NA	NA
ENSBTAG00000045861	NA	NA	NA
ENSBTAG00000045862	0.1483417	0.7508195	0.1244645
ENSBTAG00000045864	NA	NA	NA
ENSBTAG00000045867	NA	NA	NA
ENSBTAG00000045868	0.1121687	0.8091418	0.0919754
ENSBTAG00000045876	0.2449089	0.5325043	0.2736769
ENSBTAG00000045877	0.1614844	0.532139	0.2739749
ENSBTAG00000045879	0.8850398	0.0175482	1.7557686
ENSBTAG00000045880	NA	NA	NA
ENSBTAG00000045884	NA	NA	NA
ENSBTAG00000045886	0.3061151	0.3971136	0.4010852
ENSBTAG00000045887	NA	NA	NA
ENSBTAG00000045888	-0.016425	0.9707396	0.0128973
ENSBTAG00000045889	-0.587704	0.0202109	1.6944148
ENSBTAG00000045892	NA	NA	NA
ENSBTAG00000045893	0.223089	0.5342043	0.2722926
ENSBTAG00000045895	-0.467317	0.3937028	0.4048315
ENSBTAG00000045896	NA	NA	NA
ENSBTAG00000045902	NA	NA	NA
ENSBTAG00000045904	0.4226301	0.1698745	0.7698719
ENSBTAG00000045905	NA	NA	NA
ENSBTAG00000045907	NA	NA	NA
ENSBTAG00000045909	-0.497786	0.0634546	1.197537
ENSBTAG00000045910	0.4882676	0.2438072	0.6129534
ENSBTAG00000045914	-0.056184	0.9116348	0.0401791
ENSBTAG00000045915	NA	NA	NA
ENSBTAG00000045925	NA	NA	NA
ENSBTAG00000045928	NA	NA	NA
ENSBTAG00000045929	NA	NA	NA
ENSBTAG00000045931	-0.097858	0.6882443	0.1622573
ENSBTAG00000045934	NA	NA	NA
ENSBTAG00000045938	NA	NA	NA
ENSBTAG00000045939	NA	NA	NA
ENSBTAG00000045941	NA	NA	NA
ENSBTAG00000045943	NA	NA	NA

ENSBTAG00000045946	NA	NA	NA
ENSBTAG00000045947	0.2655847	0.5102006	0.292259
ENSBTAG00000045948	0.304126	0.4795814	0.3191377
ENSBTAG00000045951	NA	NA	NA
ENSBTAG00000045954	-0.068426	0.8392763	0.076095
ENSBTAG00000045955	NA	NA	NA
ENSBTAG00000045957	-0.053993	0.8384976	0.0764982
ENSBTAG00000045964	NA	NA	NA
ENSBTAG00000045966	0.4795079	0.3125557	0.5050726
ENSBTAG00000045967	NA	NA	NA
ENSBTAG00000045969	NA	NA	NA
ENSBTAG00000045971	-0.26251	0.4193864	0.3773857
ENSBTAG00000045976	NA	NA	NA
ENSBTAG00000045980	0.7680609	0.3698368	0.4319898
ENSBTAG00000045981	NA	NA	NA
ENSBTAG00000045985	0.1590127	0.6297756	0.2008142
ENSBTAG00000045987	NA	NA	NA
ENSBTAG00000045989	-0.110967	0.8087839	0.0921675
ENSBTAG00000045990	NA	NA	NA
ENSBTAG00000045991	NA	NA	NA
ENSBTAG00000045998	NA	NA	NA
ENSBTAG00000046003	-0.349629	0.2702864	0.5681758
ENSBTAG00000046005	0.1293283	0.7168085	0.1445969
ENSBTAG00000046006	NA	NA	NA
ENSBTAG00000046008	NA	NA	NA
ENSBTAG00000046010	NA	NA	NA
ENSBTAG00000046012	NA	NA	NA
ENSBTAG00000046014	0.0837747	0.8204184	0.0859646
ENSBTAG00000046016	NA	NA	NA
ENSBTAG00000046017	0.0935731	0.7310713	0.1360403
ENSBTAG00000046019	0.0063226	0.9802948	0.0086433
ENSBTAG00000046022	NA	NA	NA
ENSBTAG00000046024	0.2206139	0.4695331	0.3283338
ENSBTAG00000046029	NA	NA	NA
ENSBTAG00000046031	-0.189547	0.5875168	0.2309797
ENSBTAG00000046033	0.0101833	0.9719157	0.0123714
ENSBTAG00000046034	0.3290628	0.4867014	0.3127374
ENSBTAG00000046037	0.5386921	0.2224694	0.6527298
ENSBTAG00000046041	-0.456077	0.6445218	0.1907624
ENSBTAG00000046044	NA	NA	NA
ENSBTAG00000046046	0.8747881	0.0064474	2.1906188
ENSBTAG00000046047	-1.367708	0.077564	1.1103396
ENSBTAG00000046048	NA	NA	NA
ENSBTAG00000046051	NA	NA	NA
ENSBTAG00000046052	NA	NA	NA
ENSBTAG00000046054	-0.026199	0.9216527	0.0354327
ENSBTAG00000046057	NA	NA	NA
ENSBTAG00000046062	NA	NA	NA
ENSBTAG00000046073	NA	NA	NA
ENSBTAG00000046076	NA	NA	NA
ENSBTAG00000046080	NA	NA	NA
ENSBTAG00000046086	NA	NA	NA
ENSBTAG00000046087	NA	NA	NA
ENSBTAG00000046090	NA	NA	NA
ENSBTAG00000046092	0.3240881	0.3787995	0.4215906
ENSBTAG00000046095	-0.893763	0.0642752	1.1919563
ENSBTAG00000046096	NA	NA	NA
ENSBTAG00000046103	NA	NA	NA

ENSBTAG00000046104	-0.3115	0.4390689	0.3574673
ENSBTAG00000046108	NA	NA	NA
ENSBTAG00000046110	NA	NA	NA
ENSBTAG00000046111	-0.274477	0.5208416	0.2832944
ENSBTAG00000046115	NA	NA	NA
ENSBTAG00000046117	0.1376745	0.7134467	0.1466385
ENSBTAG00000046121	-0.188205	0.7333893	0.1346654
ENSBTAG00000046122	NA	NA	NA
ENSBTAG00000046123	-0.462976	0.1007824	0.9966152
ENSBTAG00000046129	0.2940866	0.4995141	0.3014523
ENSBTAG00000046131	NA	NA	NA
ENSBTAG00000046132	NA	NA	NA
ENSBTAG00000046135	NA	NA	NA
ENSBTAG00000046140	0.618052	0.1090489	0.9623788
ENSBTAG00000046142	-0.035879	0.9432668	0.0253655
ENSBTAG00000046150	NA	NA	NA
ENSBTAG00000046152	NA	NA	NA
ENSBTAG00000046155	-0.16732	0.7215992	0.141704
ENSBTAG00000046156	0.8030321	0.0322155	1.4919352
ENSBTAG00000046158	0.891237	0.0406153	1.3913105
ENSBTAG00000046159	NA	NA	NA
ENSBTAG00000046160	-0.270399	0.3490913	0.457061
ENSBTAG00000046161	NA	NA	NA
ENSBTAG00000046162	NA	NA	NA
ENSBTAG00000046166	NA	NA	NA
ENSBTAG00000046172	0.2673309	0.3616011	0.4417702
ENSBTAG00000046173	0.3768797	0.3510821	0.4545913
ENSBTAG00000046174	0.1412995	0.5749092	0.2404007
ENSBTAG00000046175	NA	NA	NA
ENSBTAG00000046176	0.1206313	0.6563367	0.1828733
ENSBTAG00000046177	-0.099334	0.7190163	0.1432613
ENSBTAG00000046181	NA	NA	NA
ENSBTAG00000046185	-0.191505	0.5260031	0.2790117
ENSBTAG00000046188	NA	NA	NA
ENSBTAG00000046189	NA	NA	NA
ENSBTAG00000046190	NA	NA	NA
ENSBTAG00000046191	NA	NA	NA
ENSBTAG00000046192	-0.185136	0.4753348	0.3230004
ENSBTAG00000046196	NA	NA	NA
ENSBTAG00000046199	NA	NA	NA
ENSBTAG00000046202	NA	NA	NA
ENSBTAG00000046204	-0.450314	0.2637545	0.5788002
ENSBTAG00000046208	NA	NA	NA
ENSBTAG00000046210	NA	NA	NA
ENSBTAG00000046211	NA	NA	NA
ENSBTAG00000046215	NA	NA	NA
ENSBTAG00000046218	0.700891	0.0133804	1.8735297
ENSBTAG00000046220	NA	NA	NA
ENSBTAG00000046223	0.2515322	0.3641405	0.438731
ENSBTAG00000046227	NA	NA	NA
ENSBTAG00000046229	NA	NA	NA
ENSBTAG00000046232	-0.4642	0.6347949	0.1973665
ENSBTAG00000046235	NA	NA	NA
ENSBTAG00000046239	NA	NA	NA
ENSBTAG00000046243	NA	NA	NA
ENSBTAG00000046245	NA	NA	NA
ENSBTAG00000046248	0.4094326	0.1359398	0.8666535
ENSBTAG00000046250	NA	NA	NA

ENSBTAG00000046255	NA	NA	NA
ENSBTAG00000046256	NA	NA	NA
ENSBTAG00000046257	-0.214714	0.6297522	0.2008303
ENSBTAG00000046258	NA	NA	NA
ENSBTAG00000046262	0.085347	0.9081678	0.0418339
ENSBTAG00000046263	0.4530359	0.16789	0.7749752
ENSBTAG00000046264	-0.222219	0.6631659	0.1783778
ENSBTAG00000046265	NA	NA	NA
ENSBTAG00000046266	NA	NA	NA
ENSBTAG00000046268	NA	NA	NA
ENSBTAG00000046271	NA	NA	NA
ENSBTAG00000046273	-0.110308	0.8129301	0.0899468
ENSBTAG00000046277	NA	NA	NA
ENSBTAG00000046281	NA	NA	NA
ENSBTAG00000046282	0.314123	0.2741316	0.5620409
ENSBTAG00000046286	0.3150951	0.3670931	0.4352238
ENSBTAG00000046288	NA	NA	NA
ENSBTAG00000046293	NA	NA	NA
ENSBTAG00000046295	NA	NA	NA
ENSBTAG00000046297	NA	NA	NA
ENSBTAG00000046298	NA	NA	NA
ENSBTAG00000046301	0.4601819	0.2399056	0.6199597
ENSBTAG00000046303	0.3889929	0.3686751	0.4333562
ENSBTAG00000046308	NA	NA	NA
ENSBTAG00000046309	0.3321031	0.2076172	0.6827367
ENSBTAG00000046314	NA	NA	NA
ENSBTAG00000046315	NA	NA	NA
ENSBTAG00000046319	0.0241017	0.9272432	0.0328063
ENSBTAG00000046321	NA	NA	NA
ENSBTAG00000046323	NA	NA	NA
ENSBTAG00000046324	0.2294982	0.4607998	0.3364877
ENSBTAG00000046325	-0.047454	0.9199208	0.0362496
ENSBTAG00000046328	0.2021686	0.5440669	0.2643477
ENSBTAG00000046332	-0.081301	0.7720991	0.112327
ENSBTAG00000046333	0.8102911	0.0427981	1.3685751
ENSBTAG00000046337	-0.605671	0.0777845	1.109107
ENSBTAG00000046338	-0.373803	0.260824	0.5836525
ENSBTAG00000046339	0.1879721	0.6522648	0.1855761
ENSBTAG00000046343	NA	NA	NA
ENSBTAG00000046345	NA	NA	NA
ENSBTAG00000046346	-0.077741	0.7687385	0.1142214
ENSBTAG00000046348	NA	NA	NA
ENSBTAG00000046350	NA	NA	NA
ENSBTAG00000046357	0.1676823	0.7084359	0.1496994
ENSBTAG00000046358	0.3683772	0.1568196	0.8045996
ENSBTAG00000046359	0.2528099	0.3824562	0.4174182
ENSBTAG00000046362	-0.330696	0.3889605	0.4100945
ENSBTAG00000046364	0.4973538	0.1254011	0.9016988
ENSBTAG00000046365	NA	NA	NA
ENSBTAG00000046367	0.8200379	0.0439405	1.3571354
ENSBTAG00000046368	-0.237496	0.6710775	0.1732273
ENSBTAG00000046376	NA	NA	NA
ENSBTAG00000046379	NA	NA	NA
ENSBTAG00000046383	NA	NA	NA
ENSBTAG00000046385	0.152296	0.6435955	0.191387
ENSBTAG00000046386	NA	NA	NA
ENSBTAG00000046389	NA	NA	NA
ENSBTAG00000046391	NA	NA	NA

ENSBTAG00000046394	0.0245269	0.9322386	0.0304729
ENSBTAG00000046396	NA	NA	NA
ENSBTAG00000046399	NA	NA	NA
ENSBTAG00000046402	NA	NA	NA
ENSBTAG00000046406	0.0707213	0.825162	0.0834608
ENSBTAG00000046407	NA	NA	NA
ENSBTAG00000046408	NA	NA	NA
ENSBTAG00000046409	NA	NA	NA
ENSBTAG00000046410	NA	NA	NA
ENSBTAG00000046415	0.1917441	0.5250106	0.2798319
ENSBTAG00000046416	0.5207156	0.1117757	0.9516526
ENSBTAG00000046418	NA	NA	NA
ENSBTAG00000046419	NA	NA	NA
ENSBTAG00000046430	NA	NA	NA
ENSBTAG00000046431	NA	NA	NA
ENSBTAG00000046432	NA	NA	NA
ENSBTAG00000046435	NA	NA	NA
ENSBTAG00000046439	NA	NA	NA
ENSBTAG00000046440	NA	NA	NA
ENSBTAG00000046449	NA	NA	NA
ENSBTAG00000046450	0.2102344	0.4430878	0.3535102
ENSBTAG00000046451	NA	NA	NA
ENSBTAG00000046452	NA	NA	NA
ENSBTAG00000046454	NA	NA	NA
ENSBTAG00000046457	NA	NA	NA
ENSBTAG00000046458	NA	NA	NA
ENSBTAG00000046467	0.4761555	0.0983151	1.0073798
ENSBTAG00000046470	NA	NA	NA
ENSBTAG00000046472	NA	NA	NA
ENSBTAG00000046476	NA	NA	NA
ENSBTAG00000046478	NA	NA	NA
ENSBTAG00000046481	0.2075865	0.5668995	0.2464939
ENSBTAG00000046482	NA	NA	NA
ENSBTAG00000046484	0.5755437	0.1454483	0.8372913
ENSBTAG00000046485	0.1733821	0.5168976	0.2865955
ENSBTAG00000046486	NA	NA	NA
ENSBTAG00000046491	NA	NA	NA
ENSBTAG00000046493	-0.207736	0.6102447	0.214496
ENSBTAG00000046498	-0.244041	0.3567691	0.4476128
ENSBTAG00000046500	NA	NA	NA
ENSBTAG00000046502	-0.261746	0.4401071	0.3564417
ENSBTAG00000046503	0.5031756	0.1152143	0.9384936
ENSBTAG00000046509	-0.333116	0.3759943	0.4248187
ENSBTAG00000046510	NA	NA	NA
ENSBTAG00000046512	1.491456	0.0002884	3.5399746
ENSBTAG00000046514	-0.204855	0.4493074	0.3474564
ENSBTAG00000046516	NA	NA	NA
ENSBTAG00000046518	NA	NA	NA
ENSBTAG00000046519	0.2470894	0.5748494	0.2404459
ENSBTAG00000046520	0.1578367	0.7188118	0.1433848
ENSBTAG00000046521	NA	NA	NA
ENSBTAG00000046526	0.0019027	0.9953718	0.0020147
ENSBTAG00000046531	0.3527443	0.5647586	0.2481372
ENSBTAG00000046533	-0.384954	0.1788962	0.7473989
ENSBTAG00000046536	NA	NA	NA
ENSBTAG00000046540	NA	NA	NA
ENSBTAG00000046542	0.447837	0.3062155	0.5139728
ENSBTAG00000046544	-0.220169	0.6581192	0.1816954

ENSBTAG00000046545	0.1529681	0.577928	0.2381262
ENSBTAG00000046547	NA	NA	NA
ENSBTAG00000046548	-0.194887	0.5413091	0.2665547
ENSBTAG00000046549	-0.046587	0.8625702	0.0642056
ENSBTAG00000046552	NA	NA	NA
ENSBTAG00000046553	-1.877232	2.19E-05	4.6591377
ENSBTAG00000046555	NA	NA	NA
ENSBTAG00000046556	NA	NA	NA
ENSBTAG00000046559	NA	NA	NA
ENSBTAG00000046561	0.1980205	0.4850358	0.3142262
ENSBTAG00000046569	NA	NA	NA
ENSBTAG00000046573	-0.304302	0.5153729	0.2878784
ENSBTAG00000046574	NA	NA	NA
ENSBTAG00000046575	NA	NA	NA
ENSBTAG00000046580	NA	NA	NA
ENSBTAG00000046583	NA	NA	NA
ENSBTAG00000046586	NA	NA	NA
ENSBTAG00000046587	-0.512571	0.2483585	0.6049209
ENSBTAG00000046588	0.3932196	0.1560959	0.8066086
ENSBTAG00000046590	NA	NA	NA
ENSBTAG00000046593	NA	NA	NA
ENSBTAG00000046594	NA	NA	NA
ENSBTAG00000046595	NA	NA	NA
ENSBTAG00000046597	NA	NA	NA
ENSBTAG00000046602	-0.961531	0.0552212	1.2578938
ENSBTAG00000046604	NA	NA	NA
ENSBTAG00000046607	-0.262726	0.5153473	0.2879
ENSBTAG00000046611	NA	NA	NA
ENSBTAG00000046612	0.2094614	0.4283213	0.3682304
ENSBTAG00000046614	NA	NA	NA
ENSBTAG00000046620	NA	NA	NA
ENSBTAG00000046621	NA	NA	NA
ENSBTAG00000046623	-0.216637	0.4630677	0.3343555
ENSBTAG00000046625	0.0563597	0.8993465	0.046073
ENSBTAG00000046626	NA	NA	NA
ENSBTAG00000046627	NA	NA	NA
ENSBTAG00000046628	NA	NA	NA
ENSBTAG00000046638	NA	NA	NA
ENSBTAG00000046644	-0.527602	0.0407316	1.390069
ENSBTAG00000046648	NA	NA	NA
ENSBTAG00000046649	NA	NA	NA
ENSBTAG00000046651	NA	NA	NA
ENSBTAG00000046654	NA	NA	NA
ENSBTAG00000046655	NA	NA	NA
ENSBTAG00000046657	-0.107955	0.7751058	0.110639
ENSBTAG00000046659	NA	NA	NA
ENSBTAG00000046662	NA	NA	NA
ENSBTAG00000046666	NA	NA	NA
ENSBTAG00000046668	NA	NA	NA
ENSBTAG00000046670	-0.129075	0.5943309	0.2259717
ENSBTAG00000046671	0.4655377	0.3425674	0.465254
ENSBTAG00000046672	-0.388664	0.3661232	0.4363728
ENSBTAG00000046677	NA	NA	NA
ENSBTAG00000046678	NA	NA	NA
ENSBTAG00000046684	0.0044615	0.987595	0.0054211
ENSBTAG00000046688	0.1810864	0.6994763	0.155227
ENSBTAG00000046690	NA	NA	NA
ENSBTAG00000046694	NA	NA	NA

ENSBTAG00000046699	NA	NA	NA
ENSBTAG00000046701	NA	NA	NA
ENSBTAG00000046708	NA	NA	NA
ENSBTAG00000046710	NA	NA	NA
ENSBTAG00000046712	-0.040886	0.8738521	0.0585621
ENSBTAG00000046717	NA	NA	NA
ENSBTAG00000046718	NA	NA	NA
ENSBTAG00000046721	NA	NA	NA
ENSBTAG00000046723	NA	NA	NA
ENSBTAG00000046724	0.0795984	0.821709	0.085282
ENSBTAG00000046725	-0.312709	0.2340712	0.630652
ENSBTAG00000046726	NA	NA	NA
ENSBTAG00000046727	NA	NA	NA
ENSBTAG00000046730	-0.145489	0.5664982	0.2468015
ENSBTAG00000046733	NA	NA	NA
ENSBTAG00000046738	NA	NA	NA
ENSBTAG00000046739	NA	NA	NA
ENSBTAG00000046744	NA	NA	NA
ENSBTAG00000046746	0.2770161	0.435873	0.36064
ENSBTAG00000046747	NA	NA	NA
ENSBTAG00000046749	NA	NA	NA
ENSBTAG00000046750	0.0057486	0.990252	0.0042543
ENSBTAG00000046752	NA	NA	NA
ENSBTAG00000046753	NA	NA	NA
ENSBTAG00000046755	NA	NA	NA
ENSBTAG00000046757	0.0149579	0.9687619	0.013783
ENSBTAG00000046758	NA	NA	NA
ENSBTAG00000046760	-0.079883	0.7950114	0.0996266
ENSBTAG00000046762	NA	NA	NA
ENSBTAG00000046763	0.6070552	0.0453091	1.3438143
ENSBTAG00000046765	0.2516735	0.3618295	0.4414961
ENSBTAG00000046768	NA	NA	NA
ENSBTAG00000046769	NA	NA	NA
ENSBTAG00000046771	NA	NA	NA
ENSBTAG00000046773	NA	NA	NA
ENSBTAG00000046774	0.0311866	0.9504825	0.0220559
ENSBTAG00000046775	0.3820877	0.2847653	0.5455129
ENSBTAG00000046783	NA	NA	NA
ENSBTAG00000046786	-0.272202	0.3761482	0.424641
ENSBTAG00000046792	NA	NA	NA
ENSBTAG00000046796	NA	NA	NA
ENSBTAG00000046797	-0.149284	0.5711494	0.2432503
ENSBTAG00000046800	NA	NA	NA
ENSBTAG00000046802	NA	NA	NA
ENSBTAG00000046803	0.5742211	0.2240385	0.6496774
ENSBTAG00000046808	-0.044041	0.8971476	0.0471361
ENSBTAG00000046809	NA	NA	NA
ENSBTAG00000046813	NA	NA	NA
ENSBTAG00000046814	1.1629194	0.0004875	3.3120592
ENSBTAG00000046817	NA	NA	NA
ENSBTAG00000046823	-0.048865	0.8932127	0.0490451
ENSBTAG00000046828	0.7462625	0.0946362	1.0239426
ENSBTAG00000046829	NA	NA	NA
ENSBTAG00000046833	NA	NA	NA
ENSBTAG00000046835	NA	NA	NA
ENSBTAG00000046837	0.072007	0.8154855	0.0885838
ENSBTAG00000046838	-0.4032	0.1712439	0.7663849
ENSBTAG00000046839	-0.063108	0.9129648	0.039546

ENSBTAG00000046840	0.0329944	0.9140284	0.0390403
ENSBTAG00000046841	-0.093236	0.7738422	0.1113476
ENSBTAG00000046846	0.2183525	0.4192847	0.377491
ENSBTAG00000046848	NA	NA	NA
ENSBTAG00000046851	NA	NA	NA
ENSBTAG00000046854	NA	NA	NA
ENSBTAG00000046857	NA	NA	NA
ENSBTAG00000046862	NA	NA	NA
ENSBTAG00000046865	NA	NA	NA
ENSBTAG00000046866	0.391147	0.4019653	0.3958114
ENSBTAG00000046867	NA	NA	NA
ENSBTAG00000046869	0.0509674	0.8566585	0.0671923
ENSBTAG00000046875	-0.243404	0.473488	0.324691
ENSBTAG00000046878	NA	NA	NA
ENSBTAG00000046886	-0.613201	0.1514835	0.8196347
ENSBTAG00000046900	0.3637123	0.4414999	0.3550694
ENSBTAG00000046901	NA	NA	NA
ENSBTAG00000046905	NA	NA	NA
ENSBTAG00000046908	NA	NA	NA
ENSBTAG00000046911	NA	NA	NA
ENSBTAG00000046919	0.1245663	0.7801977	0.1077953
ENSBTAG00000046922	NA	NA	NA
ENSBTAG00000046924	0.6007498	0.0433694	1.3628161
ENSBTAG00000046927	NA	NA	NA
ENSBTAG00000046932	NA	NA	NA
ENSBTAG00000046935	NA	NA	NA
ENSBTAG00000046936	0.0146695	0.9541994	0.0203609
ENSBTAG00000046938	NA	NA	NA
ENSBTAG00000046939	-0.095762	0.719099	0.1432113
ENSBTAG00000046942	NA	NA	NA
ENSBTAG00000046952	NA	NA	NA
ENSBTAG00000046953	NA	NA	NA
ENSBTAG00000046956	NA	NA	NA
ENSBTAG00000046957	NA	NA	NA
ENSBTAG00000046958	NA	NA	NA
ENSBTAG00000046959	0.2052846	0.4893627	0.3103691
ENSBTAG00000046966	0.2830052	0.4286894	0.3678573
ENSBTAG00000046971	0.432631	0.3007303	0.5218229
ENSBTAG00000046972	NA	NA	NA
ENSBTAG00000046977	NA	NA	NA
ENSBTAG00000046979	-0.331961	0.2447887	0.6112086
ENSBTAG00000046980	NA	NA	NA
ENSBTAG00000046981	-2.788873	0.0327296	1.4850598
ENSBTAG00000046984	-0.486846	0.4957834	0.304708
ENSBTAG00000046987	NA	NA	NA
ENSBTAG00000046988	NA	NA	NA
ENSBTAG00000046989	NA	NA	NA
ENSBTAG00000046990	-0.229069	0.4535035	0.3434193
ENSBTAG00000046994	NA	NA	NA
ENSBTAG00000046996	0.1098796	0.6711449	0.1731837
ENSBTAG00000047001	NA	NA	NA
ENSBTAG00000047002	-0.023663	0.9423509	0.0257874
ENSBTAG00000047003	NA	NA	NA
ENSBTAG00000047007	NA	NA	NA
ENSBTAG00000047009	NA	NA	NA
ENSBTAG00000047024	NA	NA	NA
ENSBTAG00000047025	NA	NA	NA
ENSBTAG00000047027	-0.04095	0.9074133	0.0421949

ENSBTAG00000047029	-0.901445	0.1207189	0.9182249
ENSBTAG00000047030	NA	NA	NA
ENSBTAG00000047031	0.1122703	0.7110618	0.1480927
ENSBTAG00000047035	0.1021048	0.7247127	0.1398341
ENSBTAG00000047036	NA	NA	NA
ENSBTAG00000047038	NA	NA	NA
ENSBTAG00000047039	NA	NA	NA
ENSBTAG00000047040	NA	NA	NA
ENSBTAG00000047048	NA	NA	NA
ENSBTAG00000047053	NA	NA	NA
ENSBTAG00000047059	-0.03689	0.9039143	0.0438728
ENSBTAG00000047061	0.6195678	0.0846986	1.0721235
ENSBTAG00000047062	-0.278094	0.3930635	0.4055372
ENSBTAG00000047073	NA	NA	NA
ENSBTAG00000047076	NA	NA	NA
ENSBTAG00000047077	0.3621606	0.3876037	0.4116121
ENSBTAG00000047078	-0.178104	0.7084687	0.1496793
ENSBTAG00000047080	NA	NA	NA
ENSBTAG00000047083	NA	NA	NA
ENSBTAG00000047085	NA	NA	NA
ENSBTAG00000047088	1.7715681	9.35E-09	8.0291317
ENSBTAG00000047092	NA	NA	NA
ENSBTAG00000047096	NA	NA	NA
ENSBTAG00000047101	NA	NA	NA
ENSBTAG00000047103	NA	NA	NA
ENSBTAG00000047107	NA	NA	NA
ENSBTAG00000047111	NA	NA	NA
ENSBTAG00000047113	NA	NA	NA
ENSBTAG00000047116	-0.748504	0.1112605	0.953659
ENSBTAG00000047118	NA	NA	NA
ENSBTAG00000047119	NA	NA	NA
ENSBTAG00000047121	NA	NA	NA
ENSBTAG00000047124	NA	NA	NA
ENSBTAG00000047125	NA	NA	NA
ENSBTAG00000047127	0.3036915	0.4674127	0.3302995
ENSBTAG00000047129	NA	NA	NA
ENSBTAG00000047133	NA	NA	NA
ENSBTAG00000047135	0.264067	0.4442582	0.3523645
ENSBTAG00000047136	-0.052684	0.8757659	0.057612
ENSBTAG00000047138	NA	NA	NA
ENSBTAG00000047139	-0.37595	0.1893221	0.7227988
ENSBTAG00000047144	NA	NA	NA
ENSBTAG00000047147	-1.212067	0.1748225	0.7574028
ENSBTAG00000047148	0.318784	0.3776599	0.4228991
ENSBTAG00000047150	0.5220878	0.1972373	0.7050109
ENSBTAG00000047155	-0.243318	0.7481591	0.1260061
ENSBTAG00000047156	NA	NA	NA
ENSBTAG00000047158	NA	NA	NA
ENSBTAG00000047161	NA	NA	NA
ENSBTAG00000047162	NA	NA	NA
ENSBTAG00000047164	0.0272357	0.9575227	0.0188509
ENSBTAG00000047165	NA	NA	NA
ENSBTAG00000047166	NA	NA	NA
ENSBTAG00000047169	-0.222914	0.501495	0.2997334
ENSBTAG00000047170	NA	NA	NA
ENSBTAG00000047177	NA	NA	NA
ENSBTAG00000047178	NA	NA	NA
ENSBTAG00000047181	NA	NA	NA

ENSBTAG00000047183	-0.789057	0.0542207	1.2658347
ENSBTAG00000047186	-0.1668	0.5858573	0.2322082
ENSBTAG00000047190	NA	NA	NA
ENSBTAG00000047191	NA	NA	NA
ENSBTAG00000047196	0.1387514	0.7076152	0.1502028
ENSBTAG00000047200	0.2093542	0.5819608	0.2351063
ENSBTAG00000047202	NA	NA	NA
ENSBTAG00000047203	NA	NA	NA
ENSBTAG00000047205	NA	NA	NA
ENSBTAG00000047206	0.3022305	0.3388977	0.4699314
ENSBTAG00000047214	-0.062339	0.8373337	0.0771014
ENSBTAG00000047216	-0.659598	0.0337702	1.471467
ENSBTAG00000047217	0.3243632	0.3788744	0.4215047
ENSBTAG00000047218	NA	NA	NA
ENSBTAG00000047219	-0.170294	0.6380432	0.1951499
ENSBTAG00000047223	NA	NA	NA
ENSBTAG00000047225	NA	NA	NA
ENSBTAG00000047226	-0.261443	0.4954248	0.3050222
ENSBTAG00000047227	NA	NA	NA
ENSBTAG00000047229	0.027429	0.9521741	0.0212836
ENSBTAG00000047231	0.591474	0.0210344	1.6770709
ENSBTAG00000047238	0.6905346	0.1744432	0.758346
ENSBTAG00000047240	NA	NA	NA
ENSBTAG00000047248	NA	NA	NA
ENSBTAG00000047249	0.119463	0.6609384	0.179839
ENSBTAG00000047250	NA	NA	NA
ENSBTAG00000047254	0.5828105	0.1898863	0.7215064
ENSBTAG00000047255	NA	NA	NA
ENSBTAG00000047258	NA	NA	NA
ENSBTAG00000047260	NA	NA	NA
ENSBTAG00000047264	NA	NA	NA
ENSBTAG00000047265	0.0434996	0.8913598	0.0499469
ENSBTAG00000047266	NA	NA	NA
ENSBTAG00000047268	NA	NA	NA
ENSBTAG00000047270	NA	NA	NA
ENSBTAG00000047274	NA	NA	NA
ENSBTAG00000047277	-0.304973	0.4806173	0.3182006
ENSBTAG00000047278	0.106484	0.680191	0.1673691
ENSBTAG00000047280	NA	NA	NA
ENSBTAG00000047281	NA	NA	NA
ENSBTAG00000047287	NA	NA	NA
ENSBTAG00000047288	NA	NA	NA
ENSBTAG00000047293	7.088318	2.96E-35	34.528268
ENSBTAG00000047294	NA	NA	NA
ENSBTAG00000047299	-0.024426	0.9253764	0.0336816
ENSBTAG00000047302	NA	NA	NA
ENSBTAG00000047303	NA	NA	NA
ENSBTAG00000047304	NA	NA	NA
ENSBTAG00000047313	NA	NA	NA
ENSBTAG00000047314	0.0480613	0.8716509	0.0596574
ENSBTAG00000047316	NA	NA	NA
ENSBTAG00000047319	0.1272519	0.6711301	0.1731933
ENSBTAG00000047322	NA	NA	NA
ENSBTAG00000047323	NA	NA	NA
ENSBTAG00000047325	NA	NA	NA
ENSBTAG00000047326	0.0520084	0.9215246	0.0354931
ENSBTAG00000047327	NA	NA	NA
ENSBTAG00000047330	1.3708265	1.70E-07	6.7685151

ENSBTAG00000047332	NA	NA	NA
ENSBTAG00000047336	NA	NA	NA
ENSBTAG00000047338	0.5072333	0.2393496	0.6209674
ENSBTAG00000047339	0.819874	0.0212982	1.6716564
ENSBTAG00000047340	NA	NA	NA
ENSBTAG00000047341	0.0255599	0.973237	0.0117814
ENSBTAG00000047342	NA	NA	NA
ENSBTAG00000047344	NA	NA	NA
ENSBTAG00000047345	-0.081916	0.7693053	0.1139013
ENSBTAG00000047347	NA	NA	NA
ENSBTAG00000047349	NA	NA	NA
ENSBTAG00000047357	NA	NA	NA
ENSBTAG00000047362	0.1631036	0.5840104	0.2335794
ENSBTAG00000047363	NA	NA	NA
ENSBTAG00000047367	0.0427488	0.9103367	0.040798
ENSBTAG00000047374	NA	NA	NA
ENSBTAG00000047375	NA	NA	NA
ENSBTAG00000047376	0.0851332	0.7627843	0.1175982
ENSBTAG00000047379	NA	NA	NA
ENSBTAG00000047383	NA	NA	NA
ENSBTAG00000047389	0.9323577	0.0395791	1.402534
ENSBTAG00000047395	NA	NA	NA
ENSBTAG00000047402	NA	NA	NA
ENSBTAG00000047405	NA	NA	NA
ENSBTAG00000047410	NA	NA	NA
ENSBTAG00000047412	-0.315551	0.5021532	0.2991638
ENSBTAG00000047415	NA	NA	NA
ENSBTAG00000047416	-0.002198	1	0
ENSBTAG00000047417	NA	NA	NA
ENSBTAG00000047418	0.2065285	0.4081347	0.3891965
ENSBTAG00000047420	0.9665708	0.0264135	1.5781744
ENSBTAG00000047424	0.2834091	0.2755884	0.559739
ENSBTAG00000047425	0.151488	0.7442335	0.1282908
ENSBTAG00000047426	-1.861959	3.25E-05	4.4876412
ENSBTAG00000047428	-0.077349	0.846535	0.0723551
ENSBTAG00000047433	NA	NA	NA
ENSBTAG00000047434	-0.011612	0.9685407	0.0138821
ENSBTAG00000047436	NA	NA	NA
ENSBTAG00000047442	NA	NA	NA
ENSBTAG00000047444	NA	NA	NA
ENSBTAG00000047448	NA	NA	NA
ENSBTAG00000047450	-0.505679	0.1130934	0.9465629
ENSBTAG00000047458	NA	NA	NA
ENSBTAG00000047461	-0.022227	0.9601324	0.0176689
ENSBTAG00000047462	NA	NA	NA
ENSBTAG00000047466	NA	NA	NA
ENSBTAG00000047467	0.2763402	0.5247781	0.2800243
ENSBTAG00000047468	NA	NA	NA
ENSBTAG00000047471	NA	NA	NA
ENSBTAG00000047472	NA	NA	NA
ENSBTAG00000047474	NA	NA	NA
ENSBTAG00000047475	NA	NA	NA
ENSBTAG00000047478	NA	NA	NA
ENSBTAG00000047482	NA	NA	NA
ENSBTAG00000047483	NA	NA	NA
ENSBTAG00000047484	NA	NA	NA
ENSBTAG00000047488	NA	NA	NA
ENSBTAG00000047490	0.2071111	0.6729931	0.1719894

ENSBTAG00000047491	-0.061952	0.8272103	0.082384
ENSBTAG00000047495	-0.352076	0.2325723	0.6334419
ENSBTAG00000047496	NA	NA	NA
ENSBTAG00000047498	NA	NA	NA
ENSBTAG00000047499	NA	NA	NA
ENSBTAG00000047502	-0.115569	0.8413064	0.0750458
ENSBTAG00000047503	NA	NA	NA
ENSBTAG00000047508	NA	NA	NA
ENSBTAG00000047509	NA	NA	NA
ENSBTAG00000047511	NA	NA	NA
ENSBTAG00000047514	NA	NA	NA
ENSBTAG00000047518	NA	NA	NA
ENSBTAG00000047520	NA	NA	NA
ENSBTAG00000047524	-0.657864	0.2592646	0.5862568
ENSBTAG00000047528	NA	NA	NA
ENSBTAG00000047529	NA	NA	NA
ENSBTAG00000047531	0.0764044	0.8742613	0.0583588
ENSBTAG00000047532	-0.180506	0.694658	0.158229
ENSBTAG00000047534	NA	NA	NA
ENSBTAG00000047536	NA	NA	NA
ENSBTAG00000047537	-0.371297	0.164136	0.7847963
ENSBTAG00000047538	NA	NA	NA
ENSBTAG00000047539	NA	NA	NA
ENSBTAG00000047543	1.7900048	5.62E-05	4.2504175
ENSBTAG00000047545	-0.504279	0.3047999	0.5159851
ENSBTAG00000047546	NA	NA	NA
ENSBTAG00000047547	-0.201757	0.8642969	0.063337
ENSBTAG00000047550	NA	NA	NA
ENSBTAG00000047551	NA	NA	NA
ENSBTAG00000047555	NA	NA	NA
ENSBTAG00000047559	NA	NA	NA
ENSBTAG00000047560	NA	NA	NA
ENSBTAG00000047561	0.2757037	0.3861629	0.4132295
ENSBTAG00000047567	0.0576691	0.8636084	0.0636831
ENSBTAG00000047568	NA	NA	NA
ENSBTAG00000047569	NA	NA	NA
ENSBTAG00000047570	NA	NA	NA
ENSBTAG00000047572	0.1405798	0.7653404	0.1161454
ENSBTAG00000047575	NA	NA	NA
ENSBTAG00000047577	NA	NA	NA
ENSBTAG00000047582	0.1106771	0.8080406	0.0925668
ENSBTAG00000047586	-0.216082	0.6978484	0.1562389
ENSBTAG00000047591	NA	NA	NA
ENSBTAG00000047594	NA	NA	NA
ENSBTAG00000047597	NA	NA	NA
ENSBTAG00000047598	0.3213294	0.398678	0.3993777
ENSBTAG00000047599	NA	NA	NA
ENSBTAG00000047600	NA	NA	NA
ENSBTAG00000047601	NA	NA	NA
ENSBTAG00000047602	NA	NA	NA
ENSBTAG00000047605	1.6000727	3.36E-05	4.4740318
ENSBTAG00000047606	0.5586322	0.2821483	0.5495225
ENSBTAG00000047608	-0.056976	0.8295605	0.0811519
ENSBTAG00000047609	-0.626427	0.2662556	0.5747013
ENSBTAG00000047611	NA	NA	NA
ENSBTAG00000047612	NA	NA	NA
ENSBTAG00000047613	NA	NA	NA
ENSBTAG00000047616	NA	NA	NA

ENSBTAG00000047621	0.8098589	0.0663595	1.1780968
ENSBTAG00000047624	NA	NA	NA
ENSBTAG00000047626	0.9417921	0.0635104	1.1971553
ENSBTAG00000047628	0.0035001	0.9893292	0.0046592
ENSBTAG00000047633	0.3380697	0.3223019	0.4917371
ENSBTAG00000047635	0.6967288	0.1139486	0.9432909
ENSBTAG00000047639	NA	NA	NA
ENSBTAG00000047642	NA	NA	NA
ENSBTAG00000047643	NA	NA	NA
ENSBTAG00000047645	NA	NA	NA
ENSBTAG00000047647	NA	NA	NA
ENSBTAG00000047648	NA	NA	NA
ENSBTAG00000047649	-0.201949	0.4488263	0.3479217
ENSBTAG00000047650	NA	NA	NA
ENSBTAG00000047654	NA	NA	NA
ENSBTAG00000047655	NA	NA	NA
ENSBTAG00000047658	-0.283841	0.5361626	0.2707035
ENSBTAG00000047664	NA	NA	NA
ENSBTAG00000047668	0.3157159	0.5106254	0.2918976
ENSBTAG00000047670	NA	NA	NA
ENSBTAG00000047673	-0.137244	0.5781052	0.2379931
ENSBTAG00000047675	NA	NA	NA
ENSBTAG00000047676	0.8008094	0.1122716	0.9497303
ENSBTAG00000047679	-0.117791	0.6298882	0.2007365
ENSBTAG00000047680	0.1096281	0.8276658	0.082145
ENSBTAG00000047684	NA	NA	NA
ENSBTAG00000047685	NA	NA	NA
ENSBTAG00000047690	NA	NA	NA
ENSBTAG00000047694	0.0943071	0.7217859	0.1415916
ENSBTAG00000047695	NA	NA	NA
ENSBTAG00000047696	NA	NA	NA
ENSBTAG00000047697	NA	NA	NA
ENSBTAG00000047698	NA	NA	NA
ENSBTAG00000047699	NA	NA	NA
ENSBTAG00000047700	NA	NA	NA
ENSBTAG00000047702	0.1417966	0.6983043	0.1559553
ENSBTAG00000047706	-0.012159	0.9797048	0.0089048
ENSBTAG00000047707	NA	NA	NA
ENSBTAG00000047708	-0.013012	0.966336	0.0148718
ENSBTAG00000047713	NA	NA	NA
ENSBTAG00000047715	0.1489837	0.6392095	0.1943568
ENSBTAG00000047716	NA	NA	NA
ENSBTAG00000047717	0.5260011	0.2538787	0.5953737
ENSBTAG00000047718	-0.371322	0.3266518	0.485915
ENSBTAG00000047719	NA	NA	NA
ENSBTAG00000047727	NA	NA	NA
ENSBTAG00000047729	NA	NA	NA
ENSBTAG00000047731	0.2083995	0.6563821	0.1828433
ENSBTAG00000047734	0.4208765	0.3060703	0.5141788
ENSBTAG00000047739	0.36361	0.1466681	0.8336643
ENSBTAG00000047743	NA	NA	NA
ENSBTAG00000047747	0.0241139	0.9249239	0.033894
ENSBTAG00000047749	NA	NA	NA
ENSBTAG00000047755	NA	NA	NA
ENSBTAG00000047756	-0.107211	0.7746594	0.1108892
ENSBTAG00000047760	0.4034908	0.2565135	0.5908898
ENSBTAG00000047761	0.6544747	0.1675417	0.7758771
ENSBTAG00000047764	-0.629594	0.6459986	0.1897684

ENSBTAG00000047766	-0.49411	0.3938549	0.4046637
ENSBTAG00000047767	NA	NA	NA
ENSBTAG00000047768	-0.149372	0.6039306	0.2190129
ENSBTAG00000047772	NA	NA	NA
ENSBTAG00000047776	NA	NA	NA
ENSBTAG00000047777	NA	NA	NA
ENSBTAG00000047779	NA	NA	NA
ENSBTAG00000047780	-0.297919	0.2350021	0.6289283
ENSBTAG00000047781	NA	NA	NA
ENSBTAG00000047783	NA	NA	NA
ENSBTAG00000047784	NA	NA	NA
ENSBTAG00000047786	NA	NA	NA
ENSBTAG00000047787	NA	NA	NA
ENSBTAG00000047788	-0.096776	0.7284774	0.1375839
ENSBTAG00000047791	NA	NA	NA
ENSBTAG00000047793	-0.288227	0.4526972	0.3441922
ENSBTAG00000047794	NA	NA	NA
ENSBTAG00000047799	NA	NA	NA
ENSBTAG00000047801	0.2699393	0.5056191	0.2961765
ENSBTAG00000047802	NA	NA	NA
ENSBTAG00000047804	NA	NA	NA
ENSBTAG00000047806	0.0711914	0.8268487	0.082574
ENSBTAG00000047807	NA	NA	NA
ENSBTAG00000047809	0.0410528	0.8985858	0.0464405
ENSBTAG00000047810	NA	NA	NA
ENSBTAG00000047815	NA	NA	NA
ENSBTAG00000047816	NA	NA	NA
ENSBTAG00000047818	-0.519788	0.3003543	0.5223662
ENSBTAG00000047821	NA	NA	NA
ENSBTAG00000047822	-0.0791	0.9243196	0.0341778
ENSBTAG00000047827	NA	NA	NA
ENSBTAG00000047828	0.1162856	0.8040048	0.0947414
ENSBTAG00000047829	NA	NA	NA
ENSBTAG00000047830	NA	NA	NA
ENSBTAG00000047833	NA	NA	NA
ENSBTAG00000047834	-0.356209	0.1671771	0.7768231
ENSBTAG00000047836	NA	NA	NA
ENSBTAG00000047837	NA	NA	NA
ENSBTAG00000047839	NA	NA	NA
ENSBTAG00000047845	-0.156606	0.7257319	0.1392238
ENSBTAG00000047850	NA	NA	NA
ENSBTAG00000047855	-0.067658	0.8032959	0.0951244
ENSBTAG00000047856	-0.146841	0.5970897	0.2239604
ENSBTAG00000047857	0.4494465	0.3354858	0.4743259
ENSBTAG00000047858	NA	NA	NA
ENSBTAG00000047860	NA	NA	NA
ENSBTAG00000047866	NA	NA	NA
ENSBTAG00000047868	0.5597647	0.1389634	0.8570996
ENSBTAG00000047869	NA	NA	NA
ENSBTAG00000047870	NA	NA	NA
ENSBTAG00000047874	-0.039883	0.8923279	0.0494755
ENSBTAG00000047876	0.2624973	0.576029	0.2395556
ENSBTAG00000047877	NA	NA	NA
ENSBTAG00000047879	NA	NA	NA
ENSBTAG00000047880	NA	NA	NA
ENSBTAG00000047881	NA	NA	NA
ENSBTAG00000047883	-0.667958	0.0591444	1.2280862
ENSBTAG00000047885	NA	NA	NA

ENSBTAG00000047892	NA	NA	NA
ENSBTAG00000047894	NA	NA	NA
ENSBTAG00000047896	NA	NA	NA
ENSBTAG00000047900	NA	NA	NA
ENSBTAG00000047902	-0.486501	0.2761508	0.5588538
ENSBTAG00000047904	NA	NA	NA
ENSBTAG00000047906	0.0792684	0.7929115	0.1007753
ENSBTAG00000047907	NA	NA	NA
ENSBTAG00000047909	NA	NA	NA
ENSBTAG00000047919	NA	NA	NA
ENSBTAG00000047924	NA	NA	NA
ENSBTAG00000047926	0.4394861	0.3170318	0.4988971
ENSBTAG00000047930	-0.123234	0.7340678	0.1342638
ENSBTAG00000047931	NA	NA	NA
ENSBTAG00000047932	NA	NA	NA
ENSBTAG00000047933	NA	NA	NA
ENSBTAG00000047937	NA	NA	NA
ENSBTAG00000047939	0.0230499	0.9663575	0.0148622
ENSBTAG00000047942	0.3611318	0.3426343	0.4651691
ENSBTAG00000047943	NA	NA	NA
ENSBTAG00000047944	NA	NA	NA
ENSBTAG00000047946	-0.088698	0.8279945	0.0819726
ENSBTAG00000047952	NA	NA	NA
ENSBTAG00000047954	0.093412	0.7924912	0.1010055
ENSBTAG00000047955	1.2442497	0.0012961	2.8873598
ENSBTAG00000047956	0.1877838	0.5985545	0.2228963
ENSBTAG00000047957	-0.858882	0.0561692	1.2505015
ENSBTAG00000047958	0.1411508	0.7352974	0.133537
ENSBTAG00000047964	NA	NA	NA
ENSBTAG00000047965	NA	NA	NA
ENSBTAG00000047969	NA	NA	NA
ENSBTAG00000047971	NA	NA	NA
ENSBTAG00000047973	0.1337788	0.6990139	0.1555142
ENSBTAG00000047975	-0.008967	0.9825609	0.0076405
ENSBTAG00000047979	NA	NA	NA
ENSBTAG00000047982	NA	NA	NA
ENSBTAG00000047986	NA	NA	NA
ENSBTAG00000047988	NA	NA	NA
ENSBTAG00000047990	NA	NA	NA
ENSBTAG00000047991	0.1131912	0.7656907	0.1159466
ENSBTAG00000047996	0.1707637	0.7516783	0.123968
ENSBTAG00000047997	NA	NA	NA
ENSBTAG00000047998	0.0651577	0.8032059	0.0951731
ENSBTAG00000048005	NA	NA	NA
ENSBTAG00000048007	-0.669993	0.5489239	0.2604879
ENSBTAG00000048010	-0.085452	0.8406401	0.0753899
ENSBTAG00000048011	NA	NA	NA
ENSBTAG00000048017	NA	NA	NA
ENSBTAG00000048020	NA	NA	NA
ENSBTAG00000048021	NA	NA	NA
ENSBTAG00000048022	NA	NA	NA
ENSBTAG00000048023	0.1223153	0.7481626	0.126004
ENSBTAG00000048033	NA	NA	NA
ENSBTAG00000048041	0.3102681	0.2766339	0.5580946
ENSBTAG00000048042	NA	NA	NA
ENSBTAG00000048049	NA	NA	NA
ENSBTAG00000048050	NA	NA	NA
ENSBTAG00000048051	NA	NA	NA

ENSBTAG00000048054	-0.37296	0.4078096	0.3895425
ENSBTAG00000048057	-0.227302	0.4032872	0.3943856
ENSBTAG00000048058	NA	NA	NA
ENSBTAG00000048059	NA	NA	NA
ENSBTAG00000048062	0.5400708	0.1400608	0.8536834
ENSBTAG00000048063	NA	NA	NA
ENSBTAG00000048065	NA	NA	NA
ENSBTAG00000048067	NA	NA	NA
ENSBTAG00000048071	-0.065773	0.844171	0.0735696
ENSBTAG00000048073	NA	NA	NA
ENSBTAG00000048075	NA	NA	NA
ENSBTAG00000048077	-0.285471	0.4930766	0.3070856
ENSBTAG00000048080	NA	NA	NA
ENSBTAG00000048081	NA	NA	NA
ENSBTAG00000048085	NA	NA	NA
ENSBTAG00000048090	0.026729	0.9275259	0.032674
ENSBTAG00000048094	NA	NA	NA
ENSBTAG00000048097	NA	NA	NA
ENSBTAG00000048102	-0.207912	0.4703599	0.3275697
ENSBTAG00000048106	NA	NA	NA
ENSBTAG00000048107	0.0609055	0.8071473	0.0930472
ENSBTAG00000048112	NA	NA	NA
ENSBTAG00000048114	0.4914104	0.3228928	0.4909416
ENSBTAG00000048118	NA	NA	NA
ENSBTAG00000048119	NA	NA	NA
ENSBTAG00000048122	-0.165006	0.6502216	0.1869386
ENSBTAG00000048123	NA	NA	NA
ENSBTAG00000048125	NA	NA	NA
ENSBTAG00000048135	NA	NA	NA
ENSBTAG00000048137	0.2332349	0.5966409	0.224287
ENSBTAG00000048140	0.1112462	0.7072789	0.1504093
ENSBTAG00000048145	NA	NA	NA
ENSBTAG00000048151	-0.300194	0.2238815	0.6499817
ENSBTAG00000048152	0.0554034	0.8504174	0.0703678
ENSBTAG00000048154	NA	NA	NA
ENSBTAG00000048155	NA	NA	NA
ENSBTAG00000048157	0.3098812	0.3477134	0.4587786
ENSBTAG00000048159	-0.227433	0.4561565	0.3408861
ENSBTAG00000048160	-0.24676	0.3614926	0.4419005
ENSBTAG00000048162	-0.020977	0.9625207	0.0165899
ENSBTAG00000048169	NA	NA	NA
ENSBTAG00000048170	NA	NA	NA
ENSBTAG00000048172	-0.190412	0.5542497	0.2562945
ENSBTAG00000048173	0.5531608	0.2294915	0.6392334
ENSBTAG00000048175	NA	NA	NA
ENSBTAG00000048184	NA	NA	NA
ENSBTAG00000048187	NA	NA	NA
ENSBTAG00000048195	0.5624183	0.2411674	0.6176814
ENSBTAG00000048199	NA	NA	NA
ENSBTAG00000048203	-0.877402	0.1123625	0.9493786
ENSBTAG00000048204	NA	NA	NA
ENSBTAG00000048206	NA	NA	NA
ENSBTAG00000048212	NA	NA	NA
ENSBTAG00000048213	3.801636	4.66E-11	10.331732
ENSBTAG00000048216	NA	NA	NA
ENSBTAG00000048225	NA	NA	NA
ENSBTAG00000048226	NA	NA	NA
ENSBTAG00000048228	-0.812501	0.0308579	1.5106334

ENSBTAG00000048229	-0.213777	0.4065386	0.3908982
ENSBTAG00000048230	NA	NA	NA
ENSBTAG00000048234	0.103137	0.7999607	0.0969313
ENSBTAG00000048237	NA	NA	NA
ENSBTAG00000048246	1.0433557	0.1255516	0.9011779
ENSBTAG00000048249	NA	NA	NA
ENSBTAG00000048253	NA	NA	NA
ENSBTAG00000048257	NA	NA	NA
ENSBTAG00000048262	0.0605603	0.873975	0.058501
ENSBTAG00000048263	NA	NA	NA
ENSBTAG00000048264	NA	NA	NA
ENSBTAG00000048265	NA	NA	NA
ENSBTAG00000048267	NA	NA	NA
ENSBTAG00000048268	NA	NA	NA
ENSBTAG00000048269	NA	NA	NA
ENSBTAG00000048271	0.0826977	0.7731627	0.1117291
ENSBTAG00000048273	-0.196537	0.502606	0.2987723
ENSBTAG00000048278	NA	NA	NA
ENSBTAG00000048280	-0.228466	0.5273605	0.2778924
ENSBTAG00000048286	0.3244292	0.2054148	0.6873683
ENSBTAG00000048287	NA	NA	NA
ENSBTAG00000048296	0.3486742	0.4854546	0.3138513
ENSBTAG00000048304	NA	NA	NA
ENSBTAG00000048305	NA	NA	NA
ENSBTAG00000048306	0.1039935	0.8199938	0.0861894
ENSBTAG00000048307	NA	NA	NA
ENSBTAG00000048308	0.2761412	0.4742643	0.3239796
ENSBTAG00000048309	NA	NA	NA
ENSBTAG00000048312	NA	NA	NA
ENSBTAG00000048314	-0.072675	0.7773456	0.1093859
Bos_taurus_newGene_38	NA	NA	NA
Bos_taurus_newGene_43	NA	NA	NA
Bos_taurus_newGene_45	NA	NA	NA
Bos_taurus_newGene_130	NA	NA	NA
Bos_taurus_newGene_166	NA	NA	NA
Bos_taurus_newGene_188	NA	NA	NA
Bos_taurus_newGene_260	NA	NA	NA
Bos_taurus_newGene_274	NA	NA	NA
Bos_taurus_newGene_276	NA	NA	NA
Bos_taurus_newGene_302	NA	NA	NA
Bos_taurus_newGene_324	NA	NA	NA
Bos_taurus_newGene_401	0.2110104	0.6994704	0.1552307
Bos_taurus_newGene_505	NA	NA	NA
Bos_taurus_newGene_506	NA	NA	NA
Bos_taurus_newGene_508	NA	NA	NA
Bos_taurus_newGene_510	NA	NA	NA
Bos_taurus_newGene_516	NA	NA	NA
Bos_taurus_newGene_525	NA	NA	NA
Bos_taurus_newGene_529	NA	NA	NA
Bos_taurus_newGene_570	-0.178075	0.5260542	0.2789695
Bos_taurus_newGene_572	NA	NA	NA
Bos_taurus_newGene_603	0.3523303	0.5211857	0.2830075
Bos_taurus_newGene_612	NA	NA	NA
Bos_taurus_newGene_615	NA	NA	NA
Bos_taurus_newGene_648	NA	NA	NA
Bos_taurus_newGene_659	0.2512837	0.5096925	0.2926917
Bos_taurus_newGene_661	0.2656876	0.3122429	0.5055074
Bos_taurus_newGene_662	NA	NA	NA

Bos_taurus_newGene_684	-0.220231	0.6697268	0.1741023
Bos_taurus_newGene_690	0.0012572	0.9976356	0.001028
Bos_taurus_newGene_736	NA	NA	NA
Bos_taurus_newGene_760	NA	NA	NA
Bos_taurus_newGene_789	NA	NA	NA
Bos_taurus_newGene_795	NA	NA	NA
Bos_taurus_newGene_882	NA	NA	NA
Bos_taurus_newGene_900	NA	NA	NA
Bos_taurus_newGene_902	0.6320565	0.2159961	0.6655542
Bos_taurus_newGene_924	NA	NA	NA
Bos_taurus_newGene_938	NA	NA	NA
Bos_taurus_newGene_945	NA	NA	NA
Bos_taurus_newGene_981	-0.062032	0.828573	0.0816692
Bos_taurus_newGene_1017	NA	NA	NA
Bos_taurus_newGene_1028	0.0445599	0.9083991	0.0417233
Bos_taurus_newGene_1032	NA	NA	NA
Bos_taurus_newGene_1038	-0.794127	0.0847612	1.0718029
Bos_taurus_newGene_1069	NA	NA	NA
Bos_taurus_newGene_1072	NA	NA	NA
Bos_taurus_newGene_1077	NA	NA	NA
Bos_taurus_newGene_1088	NA	NA	NA
Bos_taurus_newGene_1115	NA	NA	NA
Bos_taurus_newGene_1173	-0.058523	0.9222359	0.035158
Bos_taurus_newGene_1174	NA	NA	NA
Bos_taurus_newGene_1208	0.3367118	0.1889344	0.7236889
Bos_taurus_newGene_1209	NA	NA	NA
Bos_taurus_newGene_1218	NA	NA	NA
Bos_taurus_newGene_1223	NA	NA	NA
Bos_taurus_newGene_1233	NA	NA	NA
Bos_taurus_newGene_1317	NA	NA	NA
Bos_taurus_newGene_1355	NA	NA	NA
Bos_taurus_newGene_1369	NA	NA	NA
Bos_taurus_newGene_1398	NA	NA	NA
Bos_taurus_newGene_1417	NA	NA	NA
Bos_taurus_newGene_1441	0.6471525	0.1737135	0.7601664
Bos_taurus_newGene_1453	NA	NA	NA
Bos_taurus_newGene_1472	NA	NA	NA
Bos_taurus_newGene_1476	NA	NA	NA
Bos_taurus_newGene_1477	NA	NA	NA
Bos_taurus_newGene_1515	NA	NA	NA
Bos_taurus_newGene_1538	NA	NA	NA
Bos_taurus_newGene_1549	NA	NA	NA
Bos_taurus_newGene_1603	NA	NA	NA
Bos_taurus_newGene_1606	NA	NA	NA
Bos_taurus_newGene_1635	NA	NA	NA
Bos_taurus_newGene_1662	NA	NA	NA
Bos_taurus_newGene_1754	NA	NA	NA
Bos_taurus_newGene_1763	NA	NA	NA
Bos_taurus_newGene_1778	NA	NA	NA
Bos_taurus_newGene_1792	NA	NA	NA
Bos_taurus_newGene_1902	-1.384712	0.0002269	3.6441521
Bos_taurus_newGene_1917	NA	NA	NA
Bos_taurus_newGene_1950	-0.081717	0.863166	0.0639057
Bos_taurus_newGene_1954	NA	NA	NA
Bos_taurus_newGene_1994	-0.882651	0.0017062	2.7679752
Bos_taurus_newGene_2080	-0.512632	0.2656641	0.5756672
Bos_taurus_newGene_2119	-1.253163	0.0022459	2.6486111
Bos_taurus_newGene_2125	NA	NA	NA

Bos_taurus_newGene_2136	NA	NA	NA
Bos_taurus_newGene_2143	NA	NA	NA
Bos_taurus_newGene_2144	NA	NA	NA
Bos_taurus_newGene_2146	NA	NA	NA
Bos_taurus_newGene_2165	-1.754417	0.0006745	3.1709981
Bos_taurus_newGene_2234	NA	NA	NA
Bos_taurus_newGene_2261	NA	NA	NA
Bos_taurus_newGene_2275	NA	NA	NA
Bos_taurus_newGene_2277	-1.220028	0.0350017	1.4559108
Bos_taurus_newGene_2279	NA	NA	NA
Bos_taurus_newGene_2290	-0.410175	0.3400497	0.4684576
Bos_taurus_newGene_2302	-0.072713	0.891315	0.0499688
Bos_taurus_newGene_2306	NA	NA	NA
Bos_taurus_newGene_2307	NA	NA	NA
Bos_taurus_newGene_2308	NA	NA	NA
Bos_taurus_newGene_2309	-0.188136	0.7675219	0.1149092
Bos_taurus_newGene_2312	NA	NA	NA
Bos_taurus_newGene_2350	0.481068	0.5066513	0.2952908
Bos_taurus_newGene_2364	NA	NA	NA
Bos_taurus_newGene_2411	NA	NA	NA
Bos_taurus_newGene_2518	NA	NA	NA
Bos_taurus_newGene_2591	NA	NA	NA
Bos_taurus_newGene_2607	-1.371029	0.1999033	0.6991799
Bos_taurus_newGene_2627	-0.273889	0.5585402	0.2529455
Bos_taurus_newGene_2632	-0.021358	0.9577309	0.0187565
Bos_taurus_newGene_2806	-2.61995	2.33E-11	10.632849
Bos_taurus_newGene_2821	NA	NA	NA
Bos_taurus_newGene_2829	-3.355423	2.28E-14	13.642673
Bos_taurus_newGene_2830	-3.747625	3.67E-22	21.435835
Bos_taurus_newGene_2844	-2.787069	5.19E-05	4.2850184
Bos_taurus_newGene_2847	-1.000085	0.037184	1.4296434
Bos_taurus_newGene_2850	-1.221843	0.0244784	1.6112175
Bos_taurus_newGene_2852	NA	NA	NA
Bos_taurus_newGene_2881	NA	NA	NA
Bos_taurus_newGene_3084	NA	NA	NA
Bos_taurus_newGene_3162	NA	NA	NA
Bos_taurus_newGene_3164	0.0581061	0.9141031	0.0390048
Bos_taurus_newGene_3189	NA	NA	NA
Bos_taurus_newGene_3409	NA	NA	NA
Bos_taurus_newGene_3481	NA	NA	NA
Bos_taurus_newGene_3487	NA	NA	NA
Bos_taurus_newGene_3518	NA	NA	NA
Bos_taurus_newGene_3587	0.1410113	0.741734	0.1297518
Bos_taurus_newGene_3821	NA	NA	NA
Bos_taurus_newGene_3926	-0.182919	0.6429156	0.191846
Bos_taurus_newGene_3946	NA	NA	NA
Bos_taurus_newGene_3956	NA	NA	NA
Bos_taurus_newGene_3959	NA	NA	NA
Bos_taurus_newGene_3961	NA	NA	NA
Bos_taurus_newGene_3970	NA	NA	NA
Bos_taurus_newGene_3989	0.0495004	0.9175519	0.0373694
Bos_taurus_newGene_3990	NA	NA	NA
Bos_taurus_newGene_4053	NA	NA	NA
Bos_taurus_newGene_4063	NA	NA	NA
Bos_taurus_newGene_4069	-0.47295	0.1268189	0.8968161
Bos_taurus_newGene_4073	-0.672072	0.0429186	1.3673548
Bos_taurus_newGene_4074	NA	NA	NA
Bos_taurus_newGene_4175	NA	NA	NA

Bos_taurus_newGene_4177	NA	NA	NA
Bos_taurus_newGene_4179	NA	NA	NA
Bos_taurus_newGene_4182	0.3843131	0.4640409	0.3334437
Bos_taurus_newGene_4183	0.3730762	0.4415721	0.3549984
Bos_taurus_newGene_4246	NA	NA	NA
Bos_taurus_newGene_4247	NA	NA	NA
Bos_taurus_newGene_4248	NA	NA	NA
Bos_taurus_newGene_4276	NA	NA	NA
Bos_taurus_newGene_4313	NA	NA	NA
Bos_taurus_newGene_4315	NA	NA	NA
Bos_taurus_newGene_4316	NA	NA	NA
Bos_taurus_newGene_4317	NA	NA	NA
Bos_taurus_newGene_4319	NA	NA	NA
Bos_taurus_newGene_4378	NA	NA	NA
Bos_taurus_newGene_4565	NA	NA	NA
Bos_taurus_newGene_4568	NA	NA	NA
Bos_taurus_newGene_4569	NA	NA	NA
Bos_taurus_newGene_4570	NA	NA	NA
Bos_taurus_newGene_4571	NA	NA	NA
Bos_taurus_newGene_4572	NA	NA	NA
Bos_taurus_newGene_4802	NA	NA	NA
Bos_taurus_newGene_4859	NA	NA	NA
Bos_taurus_newGene_4911	0.038263	0.9299256	0.0315518
Bos_taurus_newGene_4916	-0.26	0.6805573	0.1671353
Bos_taurus_newGene_4921	-0.011876	0.9843235	0.0068622
Bos_taurus_newGene_4923	NA	NA	NA
Bos_taurus_newGene_4926	NA	NA	NA
Bos_taurus_newGene_4927	NA	NA	NA
Bos_taurus_newGene_4928	1.0248243	0.0956384	1.0193675
Bos_taurus_newGene_4929	0.9545677	0.1285508	0.8909253
Bos_taurus_newGene_4977	NA	NA	NA
Bos_taurus_newGene_4984	-0.466542	0.4087179	0.3885763
Bos_taurus_newGene_4986	NA	NA	NA
Bos_taurus_newGene_5044	NA	NA	NA
Bos_taurus_newGene_5141	NA	NA	NA
Bos_taurus_newGene_5161	-0.182352	0.7186635	0.1434744
Bos_taurus_newGene_5165	NA	NA	NA
Bos_taurus_newGene_5237	NA	NA	NA
Bos_taurus_newGene_5238	NA	NA	NA
Bos_taurus_newGene_5270	NA	NA	NA
Bos_taurus_newGene_5342	NA	NA	NA
Bos_taurus_newGene_5348	-1.256906	0.008551	2.0679823
Bos_taurus_newGene_5355	NA	NA	NA
Bos_taurus_newGene_5356	NA	NA	NA
Bos_taurus_newGene_5391	NA	NA	NA
Bos_taurus_newGene_5394	NA	NA	NA
Bos_taurus_newGene_5420	NA	NA	NA
Bos_taurus_newGene_5465	NA	NA	NA
Bos_taurus_newGene_5473	NA	NA	NA
Bos_taurus_newGene_5641	NA	NA	NA
Bos_taurus_newGene_5648	NA	NA	NA
Bos_taurus_newGene_5730	NA	NA	NA
Bos_taurus_newGene_5745	NA	NA	NA
Bos_taurus_newGene_5758	NA	NA	NA
Bos_taurus_newGene_5939	-0.058296	0.8134651	0.0896611
Bos_taurus_newGene_5940	NA	NA	NA
Bos_taurus_newGene_5941	NA	NA	NA
Bos_taurus_newGene_5942	NA	NA	NA

Bos_taurus_newGene_5943	NA	NA	NA
Bos_taurus_newGene_5944	NA	NA	NA
Bos_taurus_newGene_5950	-0.407099	0.1955898	0.7086537
Bos_taurus_newGene_5953	NA	NA	NA
Bos_taurus_newGene_5970	-0.31319	0.2671797	0.5731966
Bos_taurus_newGene_6082	0.7775797	0.1050782	0.9784872
Bos_taurus_newGene_6116	NA	NA	NA
Bos_taurus_newGene_6120	NA	NA	NA
Bos_taurus_newGene_6146	NA	NA	NA
Bos_taurus_newGene_6196	NA	NA	NA
Bos_taurus_newGene_6199	NA	NA	NA
Bos_taurus_newGene_6201	0.6138211	0.1753242	0.7561583
Bos_taurus_newGene_6203	0.8598762	0.0928666	1.0321406
Bos_taurus_newGene_6208	NA	NA	NA
Bos_taurus_newGene_6213	0.3669877	0.4653186	0.3322495
Bos_taurus_newGene_6223	0.7404382	0.1863649	0.7296358
Bos_taurus_newGene_6227	NA	NA	NA
Bos_taurus_newGene_6228	NA	NA	NA
Bos_taurus_newGene_6229	NA	NA	NA
Bos_taurus_newGene_6280	NA	NA	NA
Bos_taurus_newGene_6290	NA	NA	NA
Bos_taurus_newGene_6367	NA	NA	NA
Bos_taurus_newGene_6377	NA	NA	NA
Bos_taurus_newGene_6408	NA	NA	NA
Bos_taurus_newGene_6440	0.8931673	0.2777744	0.5563078
Bos_taurus_newGene_6442	NA	NA	NA
Bos_taurus_newGene_6485	NA	NA	NA
Bos_taurus_newGene_6519	NA	NA	NA
Bos_taurus_newGene_6558	NA	NA	NA
Bos_taurus_newGene_6579	NA	NA	NA
Bos_taurus_newGene_6629	NA	NA	NA
Bos_taurus_newGene_6661	-0.638855	0.1761925	0.7540126
Bos_taurus_newGene_6670	0.1096752	0.7034024	0.1527961
Bos_taurus_newGene_6676	NA	NA	NA
Bos_taurus_newGene_6677	NA	NA	NA
Bos_taurus_newGene_6682	NA	NA	NA
Bos_taurus_newGene_6686	NA	NA	NA
Bos_taurus_newGene_6687	NA	NA	NA
Bos_taurus_newGene_6722	-0.173141	0.7121526	0.1474269
Bos_taurus_newGene_6776	NA	NA	NA
Bos_taurus_newGene_6806	NA	NA	NA
Bos_taurus_newGene_6826	NA	NA	NA
Bos_taurus_newGene_6839	0.1994492	0.5025195	0.2988471
Bos_taurus_newGene_6843	NA	NA	NA
Bos_taurus_newGene_6845	1.8150413	0.027364	1.5628197
Bos_taurus_newGene_6849	-1.320968	0.0229625	1.6389803
Bos_taurus_newGene_6853	0.01905	0.9674348	0.0143783
Bos_taurus_newGene_6855	-1.980118	0.0001601	3.7956982
Bos_taurus_newGene_6860	-1.894415	0.0004863	3.3130957
Bos_taurus_newGene_6862	-2.736063	4.30E-11	10.366154
Bos_taurus_newGene_6871	-1.733561	0.0023507	2.6288098
Bos_taurus_newGene_6872	NA	NA	NA
Bos_taurus_newGene_6882	0.3721068	0.5482754	0.2610012
Bos_taurus_newGene_6885	NA	NA	NA
Bos_taurus_newGene_6947	NA	NA	NA
Bos_taurus_newGene_6972	NA	NA	NA
Bos_taurus_newGene_7015	NA	NA	NA
Bos_taurus_newGene_7093	-0.005082	0.9974596	0.0011047

Bos_taurus_newGene_7097	0.4440331	0.3024047	0.5194114
Bos_taurus_newGene_7255	-0.237868	0.6111667	0.2138403
Bos_taurus_newGene_7256	NA	NA	NA
Bos_taurus_newGene_7296	NA	NA	NA
Bos_taurus_newGene_7311	NA	NA	NA
Bos_taurus_newGene_7316	NA	NA	NA
Bos_taurus_newGene_7320	-0.08086	0.8506388	0.0702548
Bos_taurus_newGene_7349	NA	NA	NA
Bos_taurus_newGene_7376	NA	NA	NA
Bos_taurus_newGene_7396	NA	NA	NA
Bos_taurus_newGene_7399	NA	NA	NA
Bos_taurus_newGene_7461	-0.322563	0.5492322	0.260244
Bos_taurus_newGene_7490	NA	NA	NA
Bos_taurus_newGene_7512	NA	NA	NA
Bos_taurus_newGene_7513	NA	NA	NA
Bos_taurus_newGene_7514	NA	NA	NA
Bos_taurus_newGene_7517	NA	NA	NA
Bos_taurus_newGene_7560	0.224788	0.6092289	0.2152195
Bos_taurus_newGene_7593	NA	NA	NA
Bos_taurus_newGene_7634	NA	NA	NA
Bos_taurus_newGene_7670	-1.376762	0.0192004	1.7166906
Bos_taurus_newGene_7674	NA	NA	NA
Bos_taurus_newGene_7715	NA	NA	NA
Bos_taurus_newGene_7727	NA	NA	NA
Bos_taurus_newGene_7819	NA	NA	NA
Bos_taurus_newGene_7885	1.6357198	0.0207729	1.6825036
Bos_taurus_newGene_7957	NA	NA	NA
Bos_taurus_newGene_7962	NA	NA	NA
Bos_taurus_newGene_7988	NA	NA	NA
Bos_taurus_newGene_8001	NA	NA	NA
Bos_taurus_newGene_8041	-0.140119	0.7378443	0.1320353
Bos_taurus_newGene_8046	NA	NA	NA
Bos_taurus_newGene_8049	NA	NA	NA
Bos_taurus_newGene_8069	NA	NA	NA
Bos_taurus_newGene_8075	NA	NA	NA
Bos_taurus_newGene_8078	NA	NA	NA
Bos_taurus_newGene_8081	NA	NA	NA
Bos_taurus_newGene_8122	NA	NA	NA
Bos_taurus_newGene_8156	NA	NA	NA
Bos_taurus_newGene_8474	0.0248765	0.9568928	0.0191367
Bos_taurus_newGene_8477	0.1158822	0.8010826	0.0963227
Bos_taurus_newGene_8527	NA	NA	NA
Bos_taurus_newGene_8548	NA	NA	NA
Bos_taurus_newGene_8603	NA	NA	NA
Bos_taurus_newGene_8663	NA	NA	NA
Bos_taurus_newGene_8677	-0.096069	0.8289604	0.0814662
Bos_taurus_newGene_8680	-0.387194	0.3975493	0.400609
Bos_taurus_newGene_8684	NA	NA	NA
Bos_taurus_newGene_8694	NA	NA	NA
Bos_taurus_newGene_8796	-0.648419	0.0614397	1.2115507
Bos_taurus_newGene_8803	NA	NA	NA
Bos_taurus_newGene_8809	NA	NA	NA
Bos_taurus_newGene_8811	NA	NA	NA
Bos_taurus_newGene_8824	-0.575391	0.2490049	0.6037921
Bos_taurus_newGene_8828	NA	NA	NA
Bos_taurus_newGene_8834	-0.446701	0.3691852	0.4327557
Bos_taurus_newGene_8836	NA	NA	NA
Bos_taurus_newGene_8839	NA	NA	NA

Bos_taurus_newGene_8844	NA	NA	NA
Bos_taurus_newGene_8859	NA	NA	NA
Bos_taurus_newGene_8874	0.1084817	0.8074213	0.0928998
Bos_taurus_newGene_8945	-1.009921	0.0039031	2.4085865
Bos_taurus_newGene_8953	NA	NA	NA
Bos_taurus_newGene_9001	NA	NA	NA
Bos_taurus_newGene_9002	NA	NA	NA
Bos_taurus_newGene_9008	NA	NA	NA
Bos_taurus_newGene_9022	NA	NA	NA
Bos_taurus_newGene_9042	NA	NA	NA
Bos_taurus_newGene_9080	NA	NA	NA
Bos_taurus_newGene_9091	NA	NA	NA
Bos_taurus_newGene_9100	NA	NA	NA
Bos_taurus_newGene_9113	NA	NA	NA
Bos_taurus_newGene_9358	NA	NA	NA
Bos_taurus_newGene_9366	NA	NA	NA
Bos_taurus_newGene_9374	NA	NA	NA
Bos_taurus_newGene_9392	NA	NA	NA
Bos_taurus_newGene_9397	NA	NA	NA
Bos_taurus_newGene_9398	-0.729691	0.2684875	0.571076
Bos_taurus_newGene_9399	NA	NA	NA
Bos_taurus_newGene_9401	NA	NA	NA
Bos_taurus_newGene_9451	NA	NA	NA
Bos_taurus_newGene_9477	-0.334834	0.2671998	0.573164
Bos_taurus_newGene_9479	NA	NA	NA
Bos_taurus_newGene_9506	NA	NA	NA
Bos_taurus_newGene_9542	1.3396762	0.3241702	0.4892269
Bos_taurus_newGene_9546	0.0563951	0.8591338	0.0659392
Bos_taurus_newGene_9549	0.337146	0.3517467	0.45377
Bos_taurus_newGene_9614	NA	NA	NA
Bos_taurus_newGene_9645	NA	NA	NA
Bos_taurus_newGene_9665	0.3618024	0.4714206	0.3265914
Bos_taurus_newGene_9689	NA	NA	NA
Bos_taurus_newGene_9705	NA	NA	NA
Bos_taurus_newGene_9775	NA	NA	NA
Bos_taurus_newGene_9781	-0.059926	0.8462895	0.0724811
Bos_taurus_newGene_9816	NA	NA	NA
Bos_taurus_newGene_9833	0.4028151	0.3244799	0.4888122
Bos_taurus_newGene_9884	NA	NA	NA
Bos_taurus_newGene_9922	0.1621235	0.6928096	0.1593861
Bos_taurus_newGene_1002	NA	NA	NA
Bos_taurus_newGene_1002	NA	NA	NA
Bos_taurus_newGene_1005	NA	NA	NA
Bos_taurus_newGene_1008	0.2138618	0.4669075	0.3307691
Bos_taurus_newGene_1009	-0.591922	0.2474678	0.6064813
Bos_taurus_newGene_1010	NA	NA	NA
Bos_taurus_newGene_1011	NA	NA	NA
Bos_taurus_newGene_1011	NA	NA	NA
Bos_taurus_newGene_1011	NA	NA	NA
Bos_taurus_newGene_1012	NA	NA	NA
Bos_taurus_newGene_1023	NA	NA	NA
Bos_taurus_newGene_1023	NA	NA	NA
Bos_taurus_newGene_1024	NA	NA	NA
Bos_taurus_newGene_1025	NA	NA	NA
Bos_taurus_newGene_1027	-0.408309	0.3870682	0.4122125
Bos_taurus_newGene_1029	NA	NA	NA
Bos_taurus_newGene_1029	NA	NA	NA
Bos_taurus_newGene_1030	NA	NA	NA

Bos_taurus_newGene_10316	NA	NA	NA
Bos_taurus_newGene_10391	NA	NA	NA
Bos_taurus_newGene_10394	NA	NA	NA
Bos_taurus_newGene_10397	NA	NA	NA
Bos_taurus_newGene_10404	NA	NA	NA
Bos_taurus_newGene_10411	NA	NA	NA
Bos_taurus_newGene_10489	NA	NA	NA
Bos_taurus_newGene_10538	NA	NA	NA
Bos_taurus_newGene_10675	-0.368755	0.3694589	0.4324339
Bos_taurus_newGene_10759	NA	NA	NA
Bos_taurus_newGene_10876	NA	NA	NA
Bos_taurus_newGene_10947	0.4702544	0.4035723	0.3940787
Bos_taurus_newGene_10978	NA	NA	NA
Bos_taurus_newGene_10980	1.0159711	0.0307883	1.5116143
Bos_taurus_newGene_11059	NA	NA	NA
Bos_taurus_newGene_11097	NA	NA	NA
Bos_taurus_newGene_11109	NA	NA	NA
Bos_taurus_newGene_11116	NA	NA	NA
Bos_taurus_newGene_11243	0.6444757	0.559294	0.2523599
Bos_taurus_newGene_11247	NA	NA	NA
Bos_taurus_newGene_11269	-4.966713	2.65E-12	11.576227
Bos_taurus_newGene_11271	NA	NA	NA
Bos_taurus_newGene_11341	NA	NA	NA
Bos_taurus_newGene_11349	NA	NA	NA
Bos_taurus_newGene_11443	NA	NA	NA
Bos_taurus_newGene_11476	NA	NA	NA
Bos_taurus_newGene_11538	NA	NA	NA
Bos_taurus_newGene_11552	NA	NA	NA
Bos_taurus_newGene_11569	NA	NA	NA
Bos_taurus_newGene_11584	NA	NA	NA
Bos_taurus_newGene_11589	NA	NA	NA
Bos_taurus_newGene_11659	NA	NA	NA
Bos_taurus_newGene_11671	NA	NA	NA
Bos_taurus_newGene_11679	-0.045966	0.8617127	0.0646375
Bos_taurus_newGene_11714	NA	NA	NA
Bos_taurus_newGene_11742	NA	NA	NA
Bos_taurus_newGene_11787	0.6823239	0.483407	0.3156871
Bos_taurus_newGene_11788	NA	NA	NA
Bos_taurus_newGene_11789	-1.264275	0.5003042	0.3007658
Bos_taurus_newGene_11791	NA	NA	NA
Bos_taurus_newGene_11794	0.6074874	0.1397079	0.8547789
Bos_taurus_newGene_11814	NA	NA	NA
Bos_taurus_newGene_11819	NA	NA	NA
Bos_taurus_newGene_11820	NA	NA	NA
Bos_taurus_newGene_11956	NA	NA	NA
Bos_taurus_newGene_12082	0.3044253	0.6711446	0.1731839
Bos_taurus_newGene_12233	NA	NA	NA
Bos_taurus_newGene_12254	NA	NA	NA
Bos_taurus_newGene_12290	NA	NA	NA
Bos_taurus_newGene_12401	NA	NA	NA
Bos_taurus_newGene_12495	NA	NA	NA
Bos_taurus_newGene_12517	NA	NA	NA
Bos_taurus_newGene_12662	NA	NA	NA
Bos_taurus_newGene_12678	NA	NA	NA
Bos_taurus_newGene_12757	NA	NA	NA
Bos_taurus_newGene_12758	0.1942963	0.6314258	0.1996777
Bos_taurus_newGene_12761	NA	NA	NA
Bos_taurus_newGene_12762	NA	NA	NA

Bos_taurus_newGene_12766	0.2993821	0.4871066	0.312376
Bos_taurus_newGene_12768	0.2720326	0.5591262	0.2524902
Bos_taurus_newGene_12769	NA	NA	NA
Bos_taurus_newGene_12858	0.1360579	0.7286505	0.1374807
Bos_taurus_newGene_12862	NA	NA	NA
Bos_taurus_newGene_12872	NA	NA	NA
Bos_taurus_newGene_12910	NA	NA	NA
Bos_taurus_newGene_13087	NA	NA	NA
Bos_taurus_newGene_13132	NA	NA	NA
Bos_taurus_newGene_13164	0.0157354	0.9773709	0.0099406
Bos_taurus_newGene_13166	-0.511561	0.3190884	0.496089
Bos_taurus_newGene_13175	-0.62935	0.2341852	0.6304406
Bos_taurus_newGene_13177	-0.37688	0.443877	0.3527374
Bos_taurus_newGene_13179	NA	NA	NA
Bos_taurus_newGene_13181	-0.673294	0.255784	0.5921266
Bos_taurus_newGene_13182	NA	NA	NA
Bos_taurus_newGene_13189	NA	NA	NA
Bos_taurus_newGene_13191	NA	NA	NA
Bos_taurus_newGene_13277	NA	NA	NA
Bos_taurus_newGene_13281	NA	NA	NA
Bos_taurus_newGene_13292	-0.410424	0.2663666	0.5745203
Bos_taurus_newGene_13333	NA	NA	NA
Bos_taurus_newGene_13367	NA	NA	NA
Bos_taurus_newGene_13488	NA	NA	NA
Bos_taurus_newGene_13492	NA	NA	NA
Bos_taurus_newGene_13498	NA	NA	NA
Bos_taurus_newGene_13502	NA	NA	NA
Bos_taurus_newGene_13520	NA	NA	NA
Bos_taurus_newGene_13540	NA	NA	NA
Bos_taurus_newGene_13541	NA	NA	NA
Bos_taurus_newGene_13542	NA	NA	NA
Bos_taurus_newGene_13543	NA	NA	NA
Bos_taurus_newGene_13545	NA	NA	NA
Bos_taurus_newGene_13546	NA	NA	NA
Bos_taurus_newGene_13549	NA	NA	NA
Bos_taurus_newGene_13553	NA	NA	NA
Bos_taurus_newGene_13554	NA	NA	NA
Bos_taurus_newGene_13597	NA	NA	NA
Bos_taurus_newGene_13606	NA	NA	NA
Bos_taurus_newGene_13612	NA	NA	NA
Bos_taurus_newGene_13658	NA	NA	NA
Bos_taurus_newGene_13660	NA	NA	NA
Bos_taurus_newGene_13662	NA	NA	NA
Bos_taurus_newGene_13665	NA	NA	NA
Bos_taurus_newGene_13670	NA	NA	NA
Bos_taurus_newGene_13679	NA	NA	NA
Bos_taurus_newGene_13687	0.1896709	0.7765979	0.1098038
Bos_taurus_newGene_13688	NA	NA	NA
Bos_taurus_newGene_13698	0.0076784	0.9944573	0.0024139
Bos_taurus_newGene_13739	NA	NA	NA
Bos_taurus_newGene_13850	NA	NA	NA
Bos_taurus_newGene_13892	NA	NA	NA
Bos_taurus_newGene_13903	NA	NA	NA
Bos_taurus_newGene_13905	NA	NA	NA
Bos_taurus_newGene_13918	0.4181699	0.2065782	0.6849154
Bos_taurus_newGene_13940	NA	NA	NA
Bos_taurus_newGene_13960	NA	NA	NA
Bos_taurus_newGene_14035	NA	NA	NA

Bos_taurus_newGene_14041	NA	NA	NA
Bos_taurus_newGene_14049	NA	NA	NA
Bos_taurus_newGene_14069	-0.980732	0.1641671	0.7847139
Bos_taurus_newGene_14070	NA	NA	NA
Bos_taurus_newGene_14083	NA	NA	NA
Bos_taurus_newGene_14090	NA	NA	NA
Bos_taurus_newGene_14159	-0.510129	0.061191	1.2133123
Bos_taurus_newGene_14160	NA	NA	NA
Bos_taurus_newGene_14170	NA	NA	NA
Bos_taurus_newGene_14175	NA	NA	NA
Bos_taurus_newGene_14206	NA	NA	NA
Bos_taurus_newGene_14208	NA	NA	NA
Bos_taurus_newGene_14212	0.0803119	0.7521308	0.1237066
Bos_taurus_newGene_14263	NA	NA	NA
Bos_taurus_newGene_14307	NA	NA	NA
Bos_taurus_newGene_14320	NA	NA	NA
Bos_taurus_newGene_14325	0.2955104	0.5029565	0.2984695
Bos_taurus_newGene_14326	0.2596407	0.4680108	0.3297442
Bos_taurus_newGene_14329	NA	NA	NA
Bos_taurus_newGene_14336	NA	NA	NA
Bos_taurus_newGene_14358	NA	NA	NA
Bos_taurus_newGene_14393	NA	NA	NA
Bos_taurus_newGene_14396	NA	NA	NA
Bos_taurus_newGene_14404	NA	NA	NA
Bos_taurus_newGene_14405	NA	NA	NA
Bos_taurus_newGene_14407	NA	NA	NA
Bos_taurus_newGene_14408	NA	NA	NA
Bos_taurus_newGene_14409	NA	NA	NA
Bos_taurus_newGene_14410	NA	NA	NA
Bos_taurus_newGene_14414	NA	NA	NA
Bos_taurus_newGene_14425	NA	NA	NA
Bos_taurus_newGene_14505	NA	NA	NA
Bos_taurus_newGene_14525	NA	NA	NA
Bos_taurus_newGene_14545	NA	NA	NA
Bos_taurus_newGene_14568	NA	NA	NA
Bos_taurus_newGene_14573	NA	NA	NA
Bos_taurus_newGene_14584	-0.599729	0.1602124	0.7953039
Bos_taurus_newGene_14639	0.1582516	0.6806485	0.1670771
Bos_taurus_newGene_14640	-0.203755	0.7135402	0.1465816
Bos_taurus_newGene_14672	0.0323958	0.904582	0.0435521
Bos_taurus_newGene_14688	NA	NA	NA
Bos_taurus_newGene_14726	-1.195567	0.0882312	1.0543776
Bos_taurus_newGene_14735	NA	NA	NA
Bos_taurus_newGene_14765	NA	NA	NA
Bos_taurus_newGene_14793	NA	NA	NA
Bos_taurus_newGene_14886	NA	NA	NA
Bos_taurus_newGene_14898	-0.004463	1	0
Bos_taurus_newGene_14902	0.128686	0.8473911	0.0719161
Bos_taurus_newGene_14903	0.1320316	0.8378592	0.0768289
Bos_taurus_newGene_14908	-0.080247	0.8647835	0.0630926
Bos_taurus_newGene_14921	NA	NA	NA
Bos_taurus_newGene_14927	NA	NA	NA
Bos_taurus_newGene_14928	-0.443033	0.2505327	0.6011356
Bos_taurus_newGene_14952	NA	NA	NA
Bos_taurus_newGene_14979	NA	NA	NA
Bos_taurus_newGene_14980	NA	NA	NA
Bos_taurus_newGene_14989	-0.384141	0.3233676	0.4903035
Bos_taurus_newGene_15075	NA	NA	NA

Bos_taurus_newGene_15084	NA	NA	NA
Bos_taurus_newGene_15086	NA	NA	NA
Bos_taurus_newGene_15089	NA	NA	NA
Bos_taurus_newGene_15198	-1.640934	0.0012274	2.9110146
Bos_taurus_newGene_15218	NA	NA	NA
Bos_taurus_newGene_15219	NA	NA	NA
Bos_taurus_newGene_15251	NA	NA	NA
Bos_taurus_newGene_15260	NA	NA	NA
Bos_taurus_newGene_15273	NA	NA	NA
Bos_taurus_newGene_15278	NA	NA	NA
Bos_taurus_newGene_15368	NA	NA	NA
Bos_taurus_newGene_15426	NA	NA	NA
Bos_taurus_newGene_15445	-0.843973	0.1333202	0.8751042
Bos_taurus_newGene_15448	-0.506922	0.4774376	0.3210834
Bos_taurus_newGene_15483	NA	NA	NA
Bos_taurus_newGene_15509	NA	NA	NA
Bos_taurus_newGene_15514	-0.573852	0.0713196	1.146791
Bos_taurus_newGene_15518	NA	NA	NA
Bos_taurus_newGene_15572	NA	NA	NA
Bos_taurus_newGene_15589	NA	NA	NA
Bos_taurus_newGene_15597	NA	NA	NA
Bos_taurus_newGene_15603	NA	NA	NA
Bos_taurus_newGene_15629	NA	NA	NA
Bos_taurus_newGene_15631	NA	NA	NA
Bos_taurus_newGene_15662	1.2327614	0.0543571	1.2647436
Bos_taurus_newGene_15664	NA	NA	NA
Bos_taurus_newGene_15751	NA	NA	NA
Bos_taurus_newGene_15756	NA	NA	NA
Bos_taurus_newGene_15821	NA	NA	NA
Bos_taurus_newGene_15823	NA	NA	NA
Bos_taurus_newGene_15830	NA	NA	NA
Bos_taurus_newGene_15837	-1.350161	0.0014521	2.8380008
Bos_taurus_newGene_15839	-1.450608	0.00048	3.3187217
Bos_taurus_newGene_15842	NA	NA	NA
Bos_taurus_newGene_15882	NA	NA	NA
Bos_taurus_newGene_15889	0.7789842	0.2929216	0.5332486
Bos_taurus_newGene_15908	NA	NA	NA
Bos_taurus_newGene_15929	NA	NA	NA
Bos_taurus_newGene_15949	NA	NA	NA
Bos_taurus_newGene_15960	NA	NA	NA
Bos_taurus_newGene_15962	NA	NA	NA
Bos_taurus_newGene_15963	NA	NA	NA
Bos_taurus_newGene_15975	NA	NA	NA
Bos_taurus_newGene_15979	NA	NA	NA
Bos_taurus_newGene_15982	NA	NA	NA
Bos_taurus_newGene_15998	-11.88699	2.20E-38	37.65723
Bos_taurus_newGene_15999	-0.656354	0.2196595	0.6582501
Bos_taurus_newGene_16004	NA	NA	NA
Bos_taurus_newGene_16056	NA	NA	NA
Bos_taurus_newGene_16099	-0.16086	0.7324074	0.1352473
Bos_taurus_newGene_16105	NA	NA	NA
Bos_taurus_newGene_16109	-1.371564	0.0859255	1.065878
Bos_taurus_newGene_16163	NA	NA	NA
Bos_taurus_newGene_16187	NA	NA	NA
Bos_taurus_newGene_16245	NA	NA	NA
Bos_taurus_newGene_16251	NA	NA	NA
Bos_taurus_newGene_16306	NA	NA	NA
Bos_taurus_newGene_16327	NA	NA	NA

Bos_taurus_newGene_16341	NA	NA	NA
Bos_taurus_newGene_16376	NA	NA	NA
Bos_taurus_newGene_16383	-0.016368	0.986372	0.0059592
Bos_taurus_newGene_16400	NA	NA	NA
Bos_taurus_newGene_16402	NA	NA	NA
Bos_taurus_newGene_16416	NA	NA	NA
Bos_taurus_newGene_16418	0.3725141	0.4508092	0.3460072
Bos_taurus_newGene_16419	-0.325056	0.581434	0.2354996
Bos_taurus_newGene_16429	NA	NA	NA
Bos_taurus_newGene_16501	NA	NA	NA
Bos_taurus_newGene_16541	NA	NA	NA
Bos_taurus_newGene_16552	NA	NA	NA
Bos_taurus_newGene_16568	-0.240897	0.4624539	0.3349315
Bos_taurus_newGene_16580	0.3220895	0.4977005	0.3030319
Bos_taurus_newGene_16605	NA	NA	NA
Bos_taurus_newGene_16613	NA	NA	NA
Bos_taurus_newGene_16644	NA	NA	NA
Bos_taurus_newGene_16678	NA	NA	NA
Bos_taurus_newGene_16680	NA	NA	NA
Bos_taurus_newGene_16685	0.3601376	0.4351961	0.3613151
Bos_taurus_newGene_16687	NA	NA	NA
Bos_taurus_newGene_16697	-0.297476	0.3394796	0.4691864
Bos_taurus_newGene_16700	0.0910573	0.7900598	0.1023401
Bos_taurus_newGene_16704	NA	NA	NA
Bos_taurus_newGene_16714	NA	NA	NA
Bos_taurus_newGene_16726	NA	NA	NA
Bos_taurus_newGene_16730	NA	NA	NA
Bos_taurus_newGene_16731	NA	NA	NA
Bos_taurus_newGene_16741	NA	NA	NA
Bos_taurus_newGene_16747	NA	NA	NA
Bos_taurus_newGene_16749	0.3505333	0.5013002	0.2999021
Bos_taurus_newGene_16759	0.0839989	0.9195345	0.036432
Bos_taurus_newGene_16771	NA	NA	NA
Bos_taurus_newGene_16773	-0.192014	0.6933491	0.1590481
Bos_taurus_newGene_16776	NA	NA	NA
Bos_taurus_newGene_16800	NA	NA	NA
Bos_taurus_newGene_16822	-0.153789	0.5337387	0.2726713
Bos_taurus_newGene_16877	NA	NA	NA
Bos_taurus_newGene_16878	NA	NA	NA
Bos_taurus_newGene_16883	NA	NA	NA
Bos_taurus_newGene_16891	NA	NA	NA
Bos_taurus_newGene_16897	NA	NA	NA
Bos_taurus_newGene_16905	NA	NA	NA
Bos_taurus_newGene_17027	-1.192283	0.0142369	1.8465832
Bos_taurus_newGene_17074	NA	NA	NA
Bos_taurus_newGene_17143	-0.624719	0.1854575	0.7317556
Bos_taurus_newGene_17185	NA	NA	NA
Bos_taurus_newGene_17186	NA	NA	NA
Bos_taurus_newGene_17188	-0.752322	0.1091692	0.9619
Bos_taurus_newGene_17242	NA	NA	NA
Bos_taurus_newGene_17335	-0.23147	0.5441296	0.2642976
Bos_taurus_newGene_17400	NA	NA	NA
Bos_taurus_newGene_17401	NA	NA	NA
Bos_taurus_newGene_17404	NA	NA	NA
Bos_taurus_newGene_17413	NA	NA	NA
Bos_taurus_newGene_17429	-0.102537	0.8068909	0.0931852
Bos_taurus_newGene_17431	-2.174991	0.0577157	1.2387062
Bos_taurus_newGene_17464	NA	NA	NA

Bos_taurus_newGene_17494	-1.010833	0.0255437	1.5927161
Bos_taurus_newGene_17495	-0.850158	0.0106715	1.9717748
Bos_taurus_newGene_17500	NA	NA	NA
Bos_taurus_newGene_17508	-1.265835	0.0001646	3.7835754
Bos_taurus_newGene_17513	-1.996525	6.62E-07	6.1788246
Bos_taurus_newGene_17516	NA	NA	NA
Bos_taurus_newGene_17578	-0.39306	0.5309699	0.2749301
Bos_taurus_newGene_17579	-0.17864	0.7381552	0.1318523
Bos_taurus_newGene_17580	NA	NA	NA
Bos_taurus_newGene_17679	-0.186855	0.6006261	0.2213958
Bos_taurus_newGene_17688	NA	NA	NA
Bos_taurus_newGene_17700	NA	NA	NA
Bos_taurus_newGene_17701	NA	NA	NA
Bos_taurus_newGene_17726	0.2447963	0.6828241	0.1656912
Bos_taurus_newGene_17740	NA	NA	NA
Bos_taurus_newGene_17821	NA	NA	NA
Bos_taurus_newGene_17861	NA	NA	NA
Bos_taurus_newGene_17864	-0.568434	0.2364729	0.6262187
Bos_taurus_newGene_17891	-1.222935	0.0138402	1.8588563
Bos_taurus_newGene_17930	NA	NA	NA
Bos_taurus_newGene_17962	NA	NA	NA
Bos_taurus_newGene_17982	NA	NA	NA
Bos_taurus_newGene_17994	NA	NA	NA
Bos_taurus_newGene_17999	NA	NA	NA
Bos_taurus_newGene_18042	NA	NA	NA
Bos_taurus_newGene_18062	NA	NA	NA
Bos_taurus_newGene_18095	0.3858319	0.5231401	0.281382
Bos_taurus_newGene_18099	0.0628635	0.898191	0.0466313
Bos_taurus_newGene_18107	NA	NA	NA
Bos_taurus_newGene_18129	NA	NA	NA
Bos_taurus_newGene_18144	-0.679428	0.1812727	0.7416677
Bos_taurus_newGene_18150	NA	NA	NA
Bos_taurus_newGene_18152	NA	NA	NA
Bos_taurus_newGene_18156	NA	NA	NA
Bos_taurus_newGene_18157	-1.61216	0.0441471	1.3550983
Bos_taurus_newGene_18191	NA	NA	NA
Bos_taurus_newGene_18210	NA	NA	NA
Bos_taurus_newGene_18235	NA	NA	NA
Bos_taurus_newGene_18252	NA	NA	NA
Bos_taurus_newGene_18270	NA	NA	NA
Bos_taurus_newGene_18282	NA	NA	NA
Bos_taurus_newGene_18301	NA	NA	NA
Bos_taurus_newGene_18308	NA	NA	NA
Bos_taurus_newGene_18313	NA	NA	NA
Bos_taurus_newGene_18318	NA	NA	NA
Bos_taurus_newGene_18332	-0.128291	0.7970414	0.0985191
Bos_taurus_newGene_18338	0.2628864	0.5738358	0.2412124
Bos_taurus_newGene_18350	NA	NA	NA
Bos_taurus_newGene_18353	NA	NA	NA
Bos_taurus_newGene_18366	NA	NA	NA
Bos_taurus_newGene_18370	-0.588966	0.210096	0.6775821
Bos_taurus_newGene_18378	0.2614243	0.65865	0.1813453
Bos_taurus_newGene_18390	NA	NA	NA
Bos_taurus_newGene_18394	-0.53851	0.2107017	0.676332
Bos_taurus_newGene_18508	NA	NA	NA
Bos_taurus_newGene_18516	NA	NA	NA
Bos_taurus_newGene_18518	0.0290343	0.9535379	0.020662
Bos_taurus_newGene_18543	NA	NA	NA

Bos_taurus_newGene_18585	NA	NA	NA
Bos_taurus_newGene_18611	NA	NA	NA
Bos_taurus_newGene_18620	0.172653	0.5717449	0.2427977
Bos_taurus_newGene_18638	NA	NA	NA
Bos_taurus_newGene_18641	NA	NA	NA
Bos_taurus_newGene_18741	-0.428438	0.2804763	0.5521038
Bos_taurus_newGene_18743	NA	NA	NA
Bos_taurus_newGene_18745	NA	NA	NA
Bos_taurus_newGene_18750	-0.254945	0.4485616	0.3481779
Bos_taurus_newGene_18761	NA	NA	NA
Bos_taurus_newGene_18766	NA	NA	NA
Bos_taurus_newGene_18768	NA	NA	NA
Bos_taurus_newGene_18870	NA	NA	NA
Bos_taurus_newGene_18884	NA	NA	NA
Bos_taurus_newGene_18983	NA	NA	NA
Bos_taurus_newGene_19034	NA	NA	NA
Bos_taurus_newGene_19035	NA	NA	NA
Bos_taurus_newGene_19093	NA	NA	NA
Bos_taurus_newGene_19094	NA	NA	NA
Bos_taurus_newGene_19095	NA	NA	NA
Bos_taurus_newGene_19222	NA	NA	NA
Bos_taurus_newGene_19270	NA	NA	NA
Bos_taurus_newGene_19283	NA	NA	NA
Bos_taurus_newGene_19313	NA	NA	NA
Bos_taurus_newGene_19630	NA	NA	NA
Bos_taurus_newGene_19646	NA	NA	NA
Bos_taurus_newGene_19651	NA	NA	NA
Bos_taurus_newGene_19653	NA	NA	NA
Bos_taurus_newGene_19665	NA	NA	NA
Bos_taurus_newGene_19704	NA	NA	NA
Bos_taurus_newGene_19707	NA	NA	NA
Bos_taurus_newGene_19754	0.6169072	0.1395301	0.8553321
Bos_taurus_newGene_19760	-0.883329	0.0014545	2.8372976
Bos_taurus_newGene_19784	NA	NA	NA
Bos_taurus_newGene_19930	-0.15755	0.727243	0.1383204
Bos_taurus_newGene_19933	NA	NA	NA
Bos_taurus_newGene_20069	NA	NA	NA
Bos_taurus_newGene_20190	0.1847471	0.6935159	0.1589436
Bos_taurus_newGene_20225	NA	NA	NA
Bos_taurus_newGene_20356	-0.041087	0.895991	0.0476964
Bos_taurus_newGene_20363	NA	NA	NA
Bos_taurus_newGene_20374	NA	NA	NA
Bos_taurus_newGene_20436	NA	NA	NA
Bos_taurus_newGene_20438	NA	NA	NA
Bos_taurus_newGene_20451	NA	NA	NA
Bos_taurus_newGene_20485	NA	NA	NA
Bos_taurus_newGene_20595	NA	NA	NA
Bos_taurus_newGene_20596	NA	NA	NA
Bos_taurus_newGene_20599	NA	NA	NA
Bos_taurus_newGene_20625	0.3299429	0.617187	0.2095832
Bos_taurus_newGene_20651	NA	NA	NA
Bos_taurus_newGene_20681	NA	NA	NA
Bos_taurus_newGene_20731	NA	NA	NA
Bos_taurus_newGene_20762	NA	NA	NA
Bos_taurus_newGene_20802	NA	NA	NA
Bos_taurus_newGene_20865	NA	NA	NA
Bos_taurus_newGene_20874	NA	NA	NA
Bos_taurus_newGene_20876	NA	NA	NA

Bos_taurus_newGene_20880	NA	NA	NA
Bos_taurus_newGene_20908	NA	NA	NA
Bos_taurus_newGene_20934	NA	NA	NA
Bos_taurus_newGene_20975	NA	NA	NA
Bos_taurus_newGene_21003	-0.689456	0.0558119	1.2532734
Bos_taurus_newGene_21025	NA	NA	NA
Bos_taurus_newGene_21028	NA	NA	NA
Bos_taurus_newGene_21032	NA	NA	NA
Bos_taurus_newGene_21054	0.9435525	0.0707182	1.150469
Bos_taurus_newGene_21068	-1.516525	0.0080607	2.0936275
Bos_taurus_newGene_21070	NA	NA	NA
Bos_taurus_newGene_21074	-0.468586	0.4273661	0.3691999
Bos_taurus_newGene_21089	-1.726348	0.0004321	3.3643785
Bos_taurus_newGene_21105	NA	NA	NA
Bos_taurus_newGene_21116	-1.60744	0.0409519	1.3877257
Bos_taurus_newGene_21142	NA	NA	NA
Bos_taurus_newGene_21145	-0.163548	0.6791603	0.1680277
Bos_taurus_newGene_21151	NA	NA	NA
Bos_taurus_newGene_21152	-0.053043	0.8795876	0.0557209
Bos_taurus_newGene_21194	NA	NA	NA
Bos_taurus_newGene_21275	-0.72283	0.0805647	1.0938552
Bos_taurus_newGene_21278	NA	NA	NA
Bos_taurus_newGene_21291	NA	NA	NA
Bos_taurus_newGene_21324	NA	NA	NA
Bos_taurus_newGene_21362	0.4492739	0.4243201	0.3723064
Bos_taurus_newGene_21365	0.9738998	0.0375951	1.4248688
Bos_taurus_newGene_21369	NA	NA	NA
Bos_taurus_newGene_21383	NA	NA	NA
Bos_taurus_newGene_21388	NA	NA	NA
Bos_taurus_newGene_21750	NA	NA	NA
Bos_taurus_newGene_21783	NA	NA	NA
Bos_taurus_newGene_21804	NA	NA	NA
Bos_taurus_newGene_21866	NA	NA	NA
Bos_taurus_newGene_21946	NA	NA	NA
Bos_taurus_newGene_22008	0.4413774	0.3882681	0.4108683
Bos_taurus_newGene_22010	0.6221028	0.3066491	0.5133583
Bos_taurus_newGene_22016	NA	NA	NA
Bos_taurus_newGene_22035	NA	NA	NA
Bos_taurus_newGene_22038	NA	NA	NA
Bos_taurus_newGene_22040	NA	NA	NA
Bos_taurus_newGene_22052	-0.096065	0.8575632	0.0667339
Bos_taurus_newGene_22056	NA	NA	NA
Bos_taurus_newGene_22071	NA	NA	NA
Bos_taurus_newGene_22078	0.1489028	0.7756888	0.1103125
Bos_taurus_newGene_22079	NA	NA	NA
Bos_taurus_newGene_22198	-0.031872	0.9339815	0.0296617
Bos_taurus_newGene_22206	NA	NA	NA
Bos_taurus_newGene_22207	0.7737771	0.1149307	0.9395638
Bos_taurus_newGene_22217	NA	NA	NA
Bos_taurus_newGene_22224	-1.171522	0.0842246	1.0745612
Bos_taurus_newGene_22226	NA	NA	NA
Bos_taurus_newGene_22228	NA	NA	NA
Bos_taurus_newGene_22235	NA	NA	NA
Bos_taurus_newGene_22236	NA	NA	NA
Bos_taurus_newGene_22320	NA	NA	NA
Bos_taurus_newGene_22329	NA	NA	NA
Bos_taurus_newGene_22335	NA	NA	NA
Bos_taurus_newGene_22347	NA	NA	NA

Bos_taurus_newGene_22352	NA	NA	NA
Bos_taurus_newGene_22403	NA	NA	NA
Bos_taurus_newGene_22410	NA	NA	NA
Bos_taurus_newGene_22418	NA	NA	NA
Bos_taurus_newGene_22503	NA	NA	NA
Bos_taurus_newGene_22515	NA	NA	NA
Bos_taurus_newGene_22517	NA	NA	NA
Bos_taurus_newGene_22561	-0.364064	0.2625781	0.5807414
Bos_taurus_newGene_22562	NA	NA	NA
Bos_taurus_newGene_22570	NA	NA	NA
Bos_taurus_newGene_22580	-0.274114	0.4891524	0.3105558
Bos_taurus_newGene_22581	NA	NA	NA
Bos_taurus_newGene_22589	NA	NA	NA
Bos_taurus_newGene_22604	NA	NA	NA
Bos_taurus_newGene_22616	0.1022653	0.7630325	0.117457
Bos_taurus_newGene_22631	NA	NA	NA
Bos_taurus_newGene_22640	NA	NA	NA
Bos_taurus_newGene_22677	NA	NA	NA
Bos_taurus_newGene_22683	NA	NA	NA
Bos_taurus_newGene_22695	NA	NA	NA
Bos_taurus_newGene_22704	NA	NA	NA
Bos_taurus_newGene_22755	NA	NA	NA
Bos_taurus_newGene_22758	NA	NA	NA
Bos_taurus_newGene_22795	NA	NA	NA
Bos_taurus_newGene_22839	0.026398	0.9743749	0.0112739
Bos_taurus_newGene_22850	0.0327169	0.9574124	0.018901
Bos_taurus_newGene_22854	0.092442	0.8865252	0.0523089
Bos_taurus_newGene_22887	NA	NA	NA
Bos_taurus_newGene_22902	NA	NA	NA
Bos_taurus_newGene_22908	NA	NA	NA
Bos_taurus_newGene_22918	NA	NA	NA
Bos_taurus_newGene_23001	NA	NA	NA
Bos_taurus_newGene_23050	NA	NA	NA
Bos_taurus_newGene_23051	NA	NA	NA
Bos_taurus_newGene_23059	NA	NA	NA
Bos_taurus_newGene_23230	NA	NA	NA
Bos_taurus_newGene_23236	NA	NA	NA
Bos_taurus_newGene_23250	NA	NA	NA
Bos_taurus_newGene_23272	1.2083769	0.0026078	2.5837286
Bos_taurus_newGene_23278	NA	NA	NA
Bos_taurus_newGene_23329	NA	NA	NA
Bos_taurus_newGene_23356	NA	NA	NA
Bos_taurus_newGene_23443	NA	NA	NA
Bos_taurus_newGene_23454	NA	NA	NA
Bos_taurus_newGene_23494	NA	NA	NA
Bos_taurus_newGene_23535	NA	NA	NA
Bos_taurus_newGene_23563	NA	NA	NA
Bos_taurus_newGene_23674	NA	NA	NA
Bos_taurus_newGene_23703	NA	NA	NA
Bos_taurus_newGene_23706	NA	NA	NA
Bos_taurus_newGene_23794	-0.048628	0.9221728	0.0351877
Bos_taurus_newGene_23817	NA	NA	NA
Bos_taurus_newGene_23915	0.0449634	0.9009285	0.0453097
Bos_taurus_newGene_23932	NA	NA	NA
Bos_taurus_newGene_23977	NA	NA	NA
Bos_taurus_newGene_23993	NA	NA	NA
Bos_taurus_newGene_23995	NA	NA	NA
Bos_taurus_newGene_24002	NA	NA	NA

Bos_taurus_newGene_24006	NA	NA	NA
Bos_taurus_newGene_24008	0.1531721	0.5955531	0.2250795
Bos_taurus_newGene_24029	-0.048924	0.9159696	0.0381189
Bos_taurus_newGene_24031	NA	NA	NA
Bos_taurus_newGene_24042	-0.124259	0.7669933	0.1152084
Bos_taurus_newGene_24055	NA	NA	NA
Bos_taurus_newGene_24064	NA	NA	NA
Bos_taurus_newGene_24065	NA	NA	NA
Bos_taurus_newGene_24069	NA	NA	NA
Bos_taurus_newGene_24070	NA	NA	NA
Bos_taurus_newGene_24072	NA	NA	NA
Bos_taurus_newGene_24084	NA	NA	NA
Bos_taurus_newGene_24113	NA	NA	NA
Bos_taurus_newGene_24114	NA	NA	NA
Bos_taurus_newGene_24116	NA	NA	NA
Bos_taurus_newGene_24124	NA	NA	NA
Bos_taurus_newGene_24127	NA	NA	NA
Bos_taurus_newGene_24154	-0.628856	0.2641927	0.5780791
Bos_taurus_newGene_24155	NA	NA	NA
Bos_taurus_newGene_24200	NA	NA	NA
Bos_taurus_newGene_24254	-0.090287	0.8490317	0.0710761
Bos_taurus_newGene_24256	-0.257067	0.6550849	0.1837024
Bos_taurus_newGene_24258	-0.024169	0.9702159	0.0131316
Bos_taurus_newGene_24259	0.0783313	0.882217	0.0544246
Bos_taurus_newGene_24276	NA	NA	NA
Bos_taurus_newGene_24281	NA	NA	NA
Bos_taurus_newGene_24283	NA	NA	NA
Bos_taurus_newGene_24284	NA	NA	NA
Bos_taurus_newGene_24285	NA	NA	NA
Bos_taurus_newGene_24286	NA	NA	NA
Bos_taurus_newGene_24287	NA	NA	NA
Bos_taurus_newGene_24288	NA	NA	NA
Bos_taurus_newGene_24289	NA	NA	NA
Bos_taurus_newGene_24290	NA	NA	NA
Bos_taurus_newGene_24291	NA	NA	NA
Bos_taurus_newGene_24292	NA	NA	NA
Bos_taurus_newGene_24295	NA	NA	NA
Bos_taurus_newGene_24296	NA	NA	NA
Bos_taurus_newGene_24299	NA	NA	NA
Bos_taurus_newGene_24308	NA	NA	NA
Bos_taurus_newGene_24320	-1.443294	0.0003014	3.5208913
Bos_taurus_newGene_24325	-0.308823	0.5699443	0.2441676
Bos_taurus_newGene_24328	-0.305533	0.5867003	0.2315837
Bos_taurus_newGene_24333	NA	NA	NA
Bos_taurus_newGene_24338	0.0304756	0.9511703	0.0217417
Bos_taurus_newGene_24339	-0.169651	0.7451389	0.1277627
Bos_taurus_newGene_24382	NA	NA	NA
Bos_taurus_newGene_24383	-0.126112	0.7720906	0.1123317
Bos_taurus_newGene_24386	NA	NA	NA
Bos_taurus_newGene_24387	0.3307192	0.3677569	0.4344391
Bos_taurus_newGene_24388	NA	NA	NA
Bos_taurus_newGene_24389	NA	NA	NA
Bos_taurus_newGene_24421	NA	NA	NA
Bos_taurus_newGene_24488	NA	NA	NA
Bos_taurus_newGene_24528	NA	NA	NA
Bos_taurus_newGene_24544	-0.438188	0.3714148	0.4301408
Bos_taurus_newGene_24557	NA	NA	NA
Bos_taurus_newGene_24572	NA	NA	NA

Bos_taurus_newGene_24598	NA	NA	NA
Bos_taurus_newGene_24601	NA	NA	NA
Bos_taurus_newGene_24629	NA	NA	NA
Bos_taurus_newGene_24632	NA	NA	NA
Bos_taurus_newGene_24666	NA	NA	NA
Bos_taurus_newGene_24688	NA	NA	NA
Bos_taurus_newGene_24710	NA	NA	NA
Bos_taurus_newGene_24712	NA	NA	NA
Bos_taurus_newGene_24722	NA	NA	NA
Bos_taurus_newGene_24726	NA	NA	NA
Bos_taurus_newGene_24737	NA	NA	NA
Bos_taurus_newGene_24739	0.1422105	0.7538629	0.1227076
Bos_taurus_newGene_24756	NA	NA	NA
Bos_taurus_newGene_24758	NA	NA	NA
Bos_taurus_newGene_24767	-1.131377	0.0272241	1.565047
Bos_taurus_newGene_24770	-1.525823	0.0007225	3.14117
Bos_taurus_newGene_24777	NA	NA	NA
Bos_taurus_newGene_24779	NA	NA	NA
Bos_taurus_newGene_24783	NA	NA	NA
Bos_taurus_newGene_24792	-1.432191	0.0006892	3.1616383
Bos_taurus_newGene_24801	NA	NA	NA
Bos_taurus_newGene_24802	NA	NA	NA
Bos_taurus_newGene_24804	NA	NA	NA
Bos_taurus_newGene_24830	NA	NA	NA
Bos_taurus_newGene_24834	0.5795816	0.1845439	0.7339004
Bos_taurus_newGene_24837	NA	NA	NA
Bos_taurus_newGene_24846	NA	NA	NA
Bos_taurus_newGene_24855	NA	NA	NA
Bos_taurus_newGene_24862	NA	NA	NA
Bos_taurus_newGene_24863	NA	NA	NA
Bos_taurus_newGene_24866	-0.017214	0.9854084	0.0063837
Bos_taurus_newGene_24898	NA	NA	NA
Bos_taurus_newGene_24920	0.102624	0.8174064	0.087562
Bos_taurus_newGene_24924	NA	NA	NA
Bos_taurus_newGene_24930	NA	NA	NA
Bos_taurus_newGene_24931	0.2736779	0.4846618	0.3145613
Bos_taurus_newGene_24943	NA	NA	NA
Bos_taurus_newGene_24946	NA	NA	NA
Bos_taurus_newGene_24956	-0.939876	0.4120283	0.3850729
Bos_taurus_newGene_24975	0.45187	0.3096313	0.5091551
Bos_taurus_newGene_25001	-0.061296	0.8226385	0.084791
Bos_taurus_newGene_25002	NA	NA	NA
Bos_taurus_newGene_25034	NA	NA	NA
Bos_taurus_newGene_25039	NA	NA	NA
Bos_taurus_newGene_25053	NA	NA	NA
Bos_taurus_newGene_25143	NA	NA	NA
Bos_taurus_newGene_25145	NA	NA	NA
Bos_taurus_newGene_25151	NA	NA	NA
Bos_taurus_newGene_25157	NA	NA	NA
Bos_taurus_newGene_25158	0.3525988	0.4914836	0.3084909
Bos_taurus_newGene_25188	NA	NA	NA
Bos_taurus_newGene_25195	NA	NA	NA
Bos_taurus_newGene_25272	NA	NA	NA
Bos_taurus_newGene_25277	0.0313993	0.9204462	0.0360016
Bos_taurus_newGene_25280	-0.918257	0.0509414	1.292929
Bos_taurus_newGene_25285	0.2114321	0.6663914	0.1762706
Bos_taurus_newGene_25291	NA	NA	NA
Bos_taurus_newGene_25322	NA	NA	NA

Bos_taurus_newGene_25340	NA	NA	NA
Bos_taurus_newGene_25345	NA	NA	NA
Bos_taurus_newGene_25347	-0.214959	0.7684929	0.1143602
Bos_taurus_newGene_25357	NA	NA	NA
Bos_taurus_newGene_25423	NA	NA	NA
Bos_taurus_newGene_25427	0.5107812	0.4203033	0.3764372
Bos_taurus_newGene_25429	NA	NA	NA
Bos_taurus_newGene_25440	2.146716	0.0941756	1.0260615
Bos_taurus_newGene_25451	NA	NA	NA
Bos_taurus_newGene_25536	NA	NA	NA
Bos_taurus_newGene_25612	NA	NA	NA
Bos_taurus_newGene_25631	NA	NA	NA
Bos_taurus_newGene_25658	NA	NA	NA
Bos_taurus_newGene_25717	-0.438119	0.2035068	0.691421
Bos_taurus_newGene_25722	-0.621008	0.270652	0.5675887
Bos_taurus_newGene_25728	-0.29979	0.5733741	0.2415619
Bos_taurus_newGene_25796	NA	NA	NA
Bos_taurus_newGene_25840	-0.158602	0.7929771	0.1007394
Bos_taurus_newGene_25843	NA	NA	NA
Bos_taurus_newGene_25849	NA	NA	NA
Bos_taurus_newGene_25861	NA	NA	NA
Bos_taurus_newGene_25866	0.3341543	0.5723227	0.242359
Bos_taurus_newGene_25869	NA	NA	NA
Bos_taurus_newGene_25905	NA	NA	NA
Bos_taurus_newGene_25976	NA	NA	NA
Bos_taurus_newGene_25994	NA	NA	NA
Bos_taurus_newGene_26027	1.9131019	0.0052295	2.2815372
Bos_taurus_newGene_26042	0.698947	0.1273625	0.8949584
Bos_taurus_newGene_26044	NA	NA	NA
Bos_taurus_newGene_26062	NA	NA	NA
Bos_taurus_newGene_26122	NA	NA	NA
Bos_taurus_newGene_26126	NA	NA	NA
Bos_taurus_newGene_26151	NA	NA	NA
Bos_taurus_newGene_26163	-0.282294	0.5578629	0.2534725
Bos_taurus_newGene_26199	NA	NA	NA
Bos_taurus_newGene_26219	NA	NA	NA
Bos_taurus_newGene_26220	0.5611955	0.0913984	1.0390615
Bos_taurus_newGene_26241	NA	NA	NA
Bos_taurus_newGene_26250	NA	NA	NA
Bos_taurus_newGene_26284	-0.928265	0.0173875	1.7597638
Bos_taurus_newGene_26293	-0.924125	0.3786381	0.4217757
Bos_taurus_newGene_26339	NA	NA	NA
Bos_taurus_newGene_26382	NA	NA	NA
Bos_taurus_newGene_26494	NA	NA	NA
Bos_taurus_newGene_26618	NA	NA	NA
Bos_taurus_newGene_26675	NA	NA	NA
Bos_taurus_newGene_26692	NA	NA	NA
Bos_taurus_newGene_26717	NA	NA	NA
Bos_taurus_newGene_26721	NA	NA	NA
Bos_taurus_newGene_26723	NA	NA	NA
Bos_taurus_newGene_26763	-0.407509	0.4136499	0.383367
Bos_taurus_newGene_26773	NA	NA	NA
Bos_taurus_newGene_26868	NA	NA	NA
Bos_taurus_newGene_26884	NA	NA	NA
Bos_taurus_newGene_26890	NA	NA	NA
Bos_taurus_newGene_26990	NA	NA	NA
Bos_taurus_newGene_26991	NA	NA	NA
Bos_taurus_newGene_27018	NA	NA	NA

Bos_taurus_newGene_27155	NA	NA	NA
Bos_taurus_newGene_27236	NA	NA	NA
Bos_taurus_newGene_27245	NA	NA	NA
Bos_taurus_newGene_27251	0.5354212	0.2706627	0.5675715
Bos_taurus_newGene_27253	NA	NA	NA
Bos_taurus_newGene_27256	NA	NA	NA
Bos_taurus_newGene_27370	NA	NA	NA
Bos_taurus_newGene_27372	NA	NA	NA
Bos_taurus_newGene_27376	NA	NA	NA
Bos_taurus_newGene_27378	0.497672	0.5094594	0.2928904
Bos_taurus_newGene_27407	NA	NA	NA
Bos_taurus_newGene_27439	NA	NA	NA
Bos_taurus_newGene_27500	NA	NA	NA
Bos_taurus_newGene_27584	NA	NA	NA
Bos_taurus_newGene_27586	NA	NA	NA
Bos_taurus_newGene_27587	NA	NA	NA
Bos_taurus_newGene_27588	NA	NA	NA
Bos_taurus_newGene_27617	NA	NA	NA
Bos_taurus_newGene_27620	NA	NA	NA
Bos_taurus_newGene_27627	2.1604421	0.2012044	0.6963626
Bos_taurus_newGene_27643	NA	NA	NA
Bos_taurus_newGene_27666	NA	NA	NA
Bos_taurus_newGene_27672	-0.161346	0.7424764	0.1293173
Bos_taurus_newGene_27680	NA	NA	NA
Bos_taurus_newGene_27683	0.1873207	0.6982822	0.155969
Bos_taurus_newGene_27684	-0.028426	0.9450531	0.0245438
Bos_taurus_newGene_27720	NA	NA	NA
Bos_taurus_newGene_27722	NA	NA	NA
Bos_taurus_newGene_27726	NA	NA	NA
Bos_taurus_newGene_27727	NA	NA	NA
Bos_taurus_newGene_27728	NA	NA	NA
Bos_taurus_newGene_27729	NA	NA	NA
Bos_taurus_newGene_27764	NA	NA	NA
Bos_taurus_newGene_27815	-0.538798	0.2688084	0.5705571
Bos_taurus_newGene_27925	NA	NA	NA
Bos_taurus_newGene_27944	NA	NA	NA
Bos_taurus_newGene_27962	NA	NA	NA
Bos_taurus_newGene_27966	0.1324661	0.7403012	0.1305916
Bos_taurus_newGene_27969	NA	NA	NA
Bos_taurus_newGene_27971	0.4553361	0.3858298	0.4136042
Bos_taurus_newGene_28067	NA	NA	NA
Bos_taurus_newGene_28135	NA	NA	NA
Bos_taurus_newGene_28140	-0.190526	0.6635364	0.1781352
Bos_taurus_newGene_28202	-0.128373	0.7222056	0.1413392
Bos_taurus_newGene_28266	NA	NA	NA
Bos_taurus_newGene_28296	NA	NA	NA
Bos_taurus_newGene_28310	0.8792471	0.0751038	1.1243378
Bos_taurus_newGene_28317	0.8828131	0.0695434	1.1577443
Bos_taurus_newGene_28323	NA	NA	NA
Bos_taurus_newGene_28334	NA	NA	NA
Bos_taurus_newGene_28347	NA	NA	NA
Bos_taurus_newGene_28348	NA	NA	NA
Bos_taurus_newGene_28372	NA	NA	NA
Bos_taurus_newGene_28388	NA	NA	NA
Bos_taurus_newGene_28395	0.1399347	0.7059776	0.1512091
Bos_taurus_newGene_28422	-0.208777	0.540252	0.2674036
Bos_taurus_newGene_28438	NA	NA	NA
Bos_taurus_newGene_28465	NA	NA	NA

Bos_taurus_newGene_28595	NA	NA	NA
Bos_taurus_newGene_28626	-0.33165	0.2024294	0.6937264
Bos_taurus_newGene_28629	NA	NA	NA
Bos_taurus_newGene_28638	NA	NA	NA
Bos_taurus_newGene_28645	NA	NA	NA
Bos_taurus_newGene_28828	NA	NA	NA
Bos_taurus_newGene_28829	NA	NA	NA
Bos_taurus_newGene_28830	NA	NA	NA
Bos_taurus_newGene_28834	NA	NA	NA
Bos_taurus_newGene_28835	NA	NA	NA
Bos_taurus_newGene_28836	NA	NA	NA
Bos_taurus_newGene_28869	NA	NA	NA
Bos_taurus_newGene_28917	NA	NA	NA
Bos_taurus_newGene_28925	1.3868519	0.0433911	1.3625991
Bos_taurus_newGene_28930	NA	NA	NA
Bos_taurus_newGene_29019	0.1732124	0.7225242	0.1411476
Bos_taurus_newGene_29073	NA	NA	NA
Bos_taurus_newGene_29089	NA	NA	NA
Bos_taurus_newGene_29092	NA	NA	NA
Bos_taurus_newGene_29121	NA	NA	NA
Bos_taurus_newGene_29136	0.2281457	0.5286744	0.2768117
Bos_taurus_newGene_29140	0.4888192	0.3002683	0.5224906
Bos_taurus_newGene_29141	NA	NA	NA
Bos_taurus_newGene_29143	NA	NA	NA
Bos_taurus_newGene_29159	NA	NA	NA
Bos_taurus_newGene_29200	NA	NA	NA
Bos_taurus_newGene_29389	NA	NA	NA
Bos_taurus_newGene_29530	NA	NA	NA
Bos_taurus_newGene_29531	NA	NA	NA
Bos_taurus_newGene_29535	NA	NA	NA
Bos_taurus_newGene_29545	NA	NA	NA
Bos_taurus_newGene_29551	NA	NA	NA
Bos_taurus_newGene_29555	NA	NA	NA
Bos_taurus_newGene_29593	NA	NA	NA
Bos_taurus_newGene_29645	NA	NA	NA
Bos_taurus_newGene_29728	NA	NA	NA
Bos_taurus_newGene_29807	0.6791957	0.0817895	1.0873022
Bos_taurus_newGene_29829	NA	NA	NA
Bos_taurus_newGene_29914	NA	NA	NA
Bos_taurus_newGene_29967	NA	NA	NA
Bos_taurus_newGene_29979	-0.232713	0.646451	0.1894644
Bos_taurus_newGene_29981	-1.562409	0.334431	0.4756934
Bos_taurus_newGene_29982	NA	NA	NA
Bos_taurus_newGene_30029	NA	NA	NA
Bos_taurus_newGene_30030	NA	NA	NA
Bos_taurus_newGene_30031	-0.151163	0.7736268	0.1114685
Bos_taurus_newGene_30032	0.1153403	0.8224787	0.0848753
Bos_taurus_newGene_30033	0.2964602	0.5171824	0.2863563
Bos_taurus_newGene_30034	NA	NA	NA
Bos_taurus_newGene_30036	NA	NA	NA
Bos_taurus_newGene_30039	NA	NA	NA
Bos_taurus_newGene_30040	NA	NA	NA
Bos_taurus_newGene_30043	NA	NA	NA
Bos_taurus_newGene_30044	NA	NA	NA
Bos_taurus_newGene_30045	NA	NA	NA
Bos_taurus_newGene_30046	NA	NA	NA
Bos_taurus_newGene_30048	NA	NA	NA
Bos_taurus_newGene_30052	NA	NA	NA

Bos_taurus_newGene_30069	NA	NA	NA
Bos_taurus_newGene_30095	NA	NA	NA
Bos_taurus_newGene_30144	-0.593981	0.0597535	1.2236365
Bos_taurus_newGene_30147	NA	NA	NA
Bos_taurus_newGene_30173	NA	NA	NA
Bos_taurus_newGene_30174	NA	NA	NA
Bos_taurus_newGene_30228	NA	NA	NA
Bos_taurus_newGene_30517	NA	NA	NA
Bos_taurus_newGene_30538	NA	NA	NA
Bos_taurus_newGene_30541	NA	NA	NA
Bos_taurus_newGene_30545	NA	NA	NA
Bos_taurus_newGene_30552	NA	NA	NA
Bos_taurus_newGene_30554	NA	NA	NA
Bos_taurus_newGene_30560	0.8730313	0.13928	0.8561112
Bos_taurus_newGene_30614	-0.520705	0.2037026	0.6910035
Bos_taurus_newGene_30703	NA	NA	NA
Bos_taurus_newGene_30722	0.0371689	0.9419475	0.0259733
Bos_taurus_newGene_30759	-0.359648	0.3905907	0.4082781
Bos_taurus_newGene_30762	NA	NA	NA
Bos_taurus_newGene_30763	NA	NA	NA
Bos_taurus_newGene_30764	-0.641283	0.2132055	0.6712016
Bos_taurus_newGene_30821	NA	NA	NA
Bos_taurus_newGene_30829	NA	NA	NA
Bos_taurus_newGene_30876	-0.399701	0.2118027	0.6740684
Bos_taurus_newGene_30883	NA	NA	NA
Bos_taurus_newGene_30885	NA	NA	NA
Bos_taurus_newGene_30940	-0.162095	0.6148655	0.2112198
Bos_taurus_newGene_30964	NA	NA	NA
Bos_taurus_newGene_30976	NA	NA	NA
Bos_taurus_newGene_30990	-0.424127	0.4805659	0.3182471
Bos_taurus_newGene_30993	NA	NA	NA
Bos_taurus_newGene_30994	NA	NA	NA
Bos_taurus_newGene_30995	NA	NA	NA
Bos_taurus_newGene_30998	NA	NA	NA
Bos_taurus_newGene_30999	NA	NA	NA
Bos_taurus_newGene_31000	NA	NA	NA
Bos_taurus_newGene_31001	NA	NA	NA
Bos_taurus_newGene_31004	NA	NA	NA
Bos_taurus_newGene_31005	NA	NA	NA
Bos_taurus_newGene_31006	NA	NA	NA
Bos_taurus_newGene_31008	NA	NA	NA
Bos_taurus_newGene_31010	NA	NA	NA
Bos_taurus_newGene_31012	NA	NA	NA
Bos_taurus_newGene_31013	NA	NA	NA
Bos_taurus_newGene_31015	NA	NA	NA
Bos_taurus_newGene_31027	NA	NA	NA
Bos_taurus_newGene_31040	-0.11768	0.7975261	0.0982551
Bos_taurus_newGene_31046	NA	NA	NA
Bos_taurus_newGene_31057	2.8807134	0.0133568	1.8742967
Bos_taurus_newGene_31062	0.4472363	0.3224694	0.4915115
Bos_taurus_newGene_31068	NA	NA	NA
Bos_taurus_newGene_31073	NA	NA	NA
Bos_taurus_newGene_31106	NA	NA	NA
Bos_taurus_newGene_31128	NA	NA	NA
Bos_taurus_newGene_31130	NA	NA	NA
Bos_taurus_newGene_31143	NA	NA	NA
Bos_taurus_newGene_31156	NA	NA	NA
Bos_taurus_newGene_31162	NA	NA	NA

Bos_taurus_newGene_31191	NA	NA	NA
Bos_taurus_newGene_31280	-0.484297	0.2255428	0.6467711
Bos_taurus_newGene_31322	-1.311975	0.0138486	1.8585949
Bos_taurus_newGene_31336	-0.993726	0.0004426	3.3539415
Bos_taurus_newGene_31343	-0.517123	0.2789715	0.5544402
Bos_taurus_newGene_31345	-0.823321	0.0222589	1.6524969
Bos_taurus_newGene_31366	NA	NA	NA
Bos_taurus_newGene_31377	-0.300296	0.4713621	0.3266453
Bos_taurus_newGene_31378	NA	NA	NA
Bos_taurus_newGene_31379	-0.21226	0.4193781	0.3773943
Bos_taurus_newGene_31380	NA	NA	NA
Bos_taurus_newGene_31381	NA	NA	NA
Bos_taurus_newGene_31385	NA	NA	NA
Bos_taurus_newGene_31404	NA	NA	NA
Bos_taurus_newGene_31425	NA	NA	NA
Bos_taurus_newGene_31429	NA	NA	NA
Bos_taurus_newGene_31437	NA	NA	NA
Bos_taurus_newGene_31440	NA	NA	NA
Bos_taurus_newGene_31456	0.1640509	0.7053429	0.1515997
Bos_taurus_newGene_31464	NA	NA	NA
Bos_taurus_newGene_31502	NA	NA	NA
Bos_taurus_newGene_31503	NA	NA	NA
Bos_taurus_newGene_31508	NA	NA	NA
Bos_taurus_newGene_31510	NA	NA	NA
Bos_taurus_newGene_31512	NA	NA	NA
Bos_taurus_newGene_31513	NA	NA	NA
Bos_taurus_newGene_31514	-0.015155	0.989849	0.004431
Bos_taurus_newGene_31515	NA	NA	NA
Bos_taurus_newGene_31540	0.5636126	0.1170133	0.9317649
Bos_taurus_newGene_31566	NA	NA	NA
Bos_taurus_newGene_31590	NA	NA	NA
Bos_taurus_newGene_31650	NA	NA	NA
Bos_taurus_newGene_31659	NA	NA	NA
Bos_taurus_newGene_31660	NA	NA	NA
Bos_taurus_newGene_31684	NA	NA	NA
Bos_taurus_newGene_31764	NA	NA	NA
Bos_taurus_newGene_31811	0.1534329	0.7465474	0.1269426
Bos_taurus_newGene_31834	NA	NA	NA
Bos_taurus_newGene_31845	NA	NA	NA
Bos_taurus_newGene_31854	NA	NA	NA
Bos_taurus_newGene_31963	NA	NA	NA
Bos_taurus_newGene_31966	0.2318236	0.4996263	0.3013547
Bos_taurus_newGene_31972	NA	NA	NA
Bos_taurus_newGene_32000	NA	NA	NA
Bos_taurus_newGene_32002	NA	NA	NA
Bos_taurus_newGene_32057	-0.287049	0.4217915	0.3749022
Bos_taurus_newGene_32058	NA	NA	NA
Bos_taurus_newGene_32062	NA	NA	NA
Bos_taurus_newGene_32078	NA	NA	NA
Bos_taurus_newGene_32102	NA	NA	NA
Bos_taurus_newGene_32216	NA	NA	NA
Bos_taurus_newGene_32274	NA	NA	NA
Bos_taurus_newGene_32275	NA	NA	NA
Bos_taurus_newGene_32386	NA	NA	NA
Bos_taurus_newGene_32403	NA	NA	NA
Bos_taurus_newGene_32406	NA	NA	NA
Bos_taurus_newGene_32408	NA	NA	NA
Bos_taurus_newGene_32409	NA	NA	NA

Bos_taurus_newGene_32570	NA	NA	NA
Bos_taurus_newGene_32582	-0.857654	0.0663286	1.1782993
Bos_taurus_newGene_32601	NA	NA	NA
Bos_taurus_newGene_32640	NA	NA	NA
Bos_taurus_newGene_32709	NA	NA	NA
Bos_taurus_newGene_32711	NA	NA	NA
Bos_taurus_newGene_32712	NA	NA	NA
Bos_taurus_newGene_32713	-0.083405	0.8982866	0.0465851
Bos_taurus_newGene_32718	NA	NA	NA
Bos_taurus_newGene_32754	NA	NA	NA
Bos_taurus_newGene_32809	0.1885064	0.4746229	0.3236513
Bos_taurus_newGene_32810	NA	NA	NA
Bos_taurus_newGene_32811	NA	NA	NA
Bos_taurus_newGene_32827	-0.387095	0.3729018	0.4284056
Bos_taurus_newGene_32838	-1.582129	0.0005751	3.2402619
Bos_taurus_newGene_32880	NA	NA	NA
Bos_taurus_newGene_32970	NA	NA	NA
Bos_taurus_newGene_33023	NA	NA	NA
Bos_taurus_newGene_33042	-0.438453	0.3493935	0.4566852
Bos_taurus_newGene_33070	0.5761073	0.2877196	0.5410305
Bos_taurus_newGene_33170	NA	NA	NA
Bos_taurus_newGene_33233	NA	NA	NA
Bos_taurus_newGene_33238	NA	NA	NA
Bos_taurus_newGene_33240	1.1572804	0.0163412	1.7867173
Bos_taurus_newGene_33241	NA	NA	NA
Bos_taurus_newGene_33242	NA	NA	NA
Bos_taurus_newGene_33243	NA	NA	NA
Bos_taurus_newGene_33244	-0.345548	0.5341994	0.2722966
Bos_taurus_newGene_33285	NA	NA	NA
Bos_taurus_newGene_33366	NA	NA	NA
Bos_taurus_newGene_33470	NA	NA	NA
Bos_taurus_newGene_33487	NA	NA	NA
Bos_taurus_newGene_33530	NA	NA	NA
Bos_taurus_newGene_33588	0.2180007	0.6516361	0.1859949
Bos_taurus_newGene_33604	NA	NA	NA
Bos_taurus_newGene_33617	-0.458002	0.2874356	0.5414595
Bos_taurus_newGene_33627	NA	NA	NA
Bos_taurus_newGene_33634	NA	NA	NA
Bos_taurus_newGene_33658	NA	NA	NA
Bos_taurus_newGene_33677	NA	NA	NA
Bos_taurus_newGene_33683	NA	NA	NA
Bos_taurus_newGene_33707	NA	NA	NA
Bos_taurus_newGene_33729	NA	NA	NA
Bos_taurus_newGene_33737	NA	NA	NA
Bos_taurus_newGene_33738	NA	NA	NA
Bos_taurus_newGene_33739	NA	NA	NA
Bos_taurus_newGene_33741	NA	NA	NA
Bos_taurus_newGene_33767	NA	NA	NA
Bos_taurus_newGene_33818	NA	NA	NA
Bos_taurus_newGene_33875	NA	NA	NA
Bos_taurus_newGene_33890	NA	NA	NA
Bos_taurus_newGene_33914	NA	NA	NA
Bos_taurus_newGene_33925	NA	NA	NA
Bos_taurus_newGene_33938	0.3445912	0.4690762	0.3287566
Bos_taurus_newGene_33996	NA	NA	NA
Bos_taurus_newGene_34000	NA	NA	NA
Bos_taurus_newGene_34044	NA	NA	NA
Bos_taurus_newGene_34068	NA	NA	NA

Bos_taurus_newGene_34106	NA	NA	NA
Bos_taurus_newGene_34108	0.2396127	0.694584	0.1582752
Bos_taurus_newGene_34114	NA	NA	NA
Bos_taurus_newGene_34140	NA	NA	NA
Bos_taurus_newGene_34250	NA	NA	NA
Bos_taurus_newGene_34324	NA	NA	NA
Bos_taurus_newGene_34325	NA	NA	NA
Bos_taurus_newGene_34327	NA	NA	NA
Bos_taurus_newGene_34328	NA	NA	NA
Bos_taurus_newGene_34336	NA	NA	NA
Bos_taurus_newGene_34339	NA	NA	NA
Bos_taurus_newGene_34341	NA	NA	NA
Bos_taurus_newGene_34345	NA	NA	NA
Bos_taurus_newGene_34346	NA	NA	NA
Bos_taurus_newGene_34347	NA	NA	NA
Bos_taurus_newGene_34350	NA	NA	NA
Bos_taurus_newGene_34370	NA	NA	NA
Bos_taurus_newGene_34396	-0.539104	0.3604488	0.4431564
Bos_taurus_newGene_34397	NA	NA	NA
Bos_taurus_newGene_34398	NA	NA	NA
Bos_taurus_newGene_34415	NA	NA	NA
Bos_taurus_newGene_34431	NA	NA	NA
Bos_taurus_newGene_34437	NA	NA	NA
Bos_taurus_newGene_34438	-0.841937	0.2358715	0.6273246
Bos_taurus_newGene_34440	NA	NA	NA
Bos_taurus_newGene_34441	NA	NA	NA
Bos_taurus_newGene_34445	NA	NA	NA
Bos_taurus_newGene_34475	NA	NA	NA
Bos_taurus_newGene_34476	-0.032285	0.9517014	0.0214993
Bos_taurus_newGene_34477	-0.165135	0.6767965	0.1695419
Bos_taurus_newGene_34556	NA	NA	NA
Bos_taurus_newGene_34563	NA	NA	NA
Bos_taurus_newGene_34565	NA	NA	NA
Bos_taurus_newGene_34566	NA	NA	NA
Bos_taurus_newGene_34568	NA	NA	NA
Bos_taurus_newGene_34578	-0.432778	0.2468163	0.6076262
Bos_taurus_newGene_34579	NA	NA	NA
Bos_taurus_newGene_34580	NA	NA	NA
Bos_taurus_newGene_34616	NA	NA	NA
Bos_taurus_newGene_34631	0.3431031	0.4777746	0.320777
Bos_taurus_newGene_34640	0.336593	0.4286383	0.367909
Bos_taurus_newGene_34683	-0.136281	0.6575677	0.1820595
Bos_taurus_newGene_34726	NA	NA	NA
Bos_taurus_newGene_34754	NA	NA	NA
Bos_taurus_newGene_34761	NA	NA	NA
Bos_taurus_newGene_34766	NA	NA	NA
Bos_taurus_newGene_34774	NA	NA	NA
Bos_taurus_newGene_34874	NA	NA	NA
Bos_taurus_newGene_34875	NA	NA	NA
Bos_taurus_newGene_35002	NA	NA	NA
Bos_taurus_newGene_35014	NA	NA	NA
Bos_taurus_newGene_35040	NA	NA	NA
Bos_taurus_newGene_35049	NA	NA	NA
Bos_taurus_newGene_35092	NA	NA	NA
Bos_taurus_newGene_35120	NA	NA	NA
Bos_taurus_newGene_35188	0.2842981	0.6761607	0.1699501
Bos_taurus_newGene_35189	0.1772386	0.7721639	0.1122905
Bos_taurus_newGene_35204	NA	NA	NA

Bos_taurus_newGene_35237	NA	NA	NA
Bos_taurus_newGene_35329	-1.049779	0.0595787	1.2249093
Bos_taurus_newGene_35358	NA	NA	NA
Bos_taurus_newGene_35403	0.0739726	0.8748931	0.058045
Bos_taurus_newGene_35416	0.4972884	0.329559	0.4820668
Bos_taurus_newGene_35432	0.2371574	0.7199824	0.1426781
Bos_taurus_newGene_35436	NA	NA	NA
Bos_taurus_newGene_35439	0.3855231	0.5834132	0.2340237
Bos_taurus_newGene_35441	NA	NA	NA
Bos_taurus_newGene_35448	NA	NA	NA
Bos_taurus_newGene_35449	NA	NA	NA
Bos_taurus_newGene_35451	0.4641759	0.4321114	0.3644043
Bos_taurus_newGene_35453	0.3796233	0.4873113	0.3121935
Bos_taurus_newGene_35457	0.4428711	0.4491986	0.3475616
Bos_taurus_newGene_35459	0.4515811	0.3736023	0.4275905
Bos_taurus_newGene_35460	0.6127542	0.5987035	0.2227882
Bos_taurus_newGene_35485	0.6634402	0.1010022	0.9956691
Bos_taurus_newGene_35664	NA	NA	NA
Bos_taurus_newGene_35720	NA	NA	NA
Bos_taurus_newGene_35808	NA	NA	NA
Bos_taurus_newGene_35809	NA	NA	NA
Bos_taurus_newGene_35814	NA	NA	NA
Bos_taurus_newGene_35879	NA	NA	NA
Bos_taurus_newGene_35880	NA	NA	NA
Bos_taurus_newGene_35881	0.1104302	0.8282999	0.0818124
Bos_taurus_newGene_35882	0.1965286	0.6337491	0.1980827
Bos_taurus_newGene_35883	NA	NA	NA
Bos_taurus_newGene_35884	NA	NA	NA
Bos_taurus_newGene_35885	-0.555498	0.2341624	0.6304828
Bos_taurus_newGene_35886	NA	NA	NA
Bos_taurus_newGene_35888	NA	NA	NA
Bos_taurus_newGene_35889	NA	NA	NA
Bos_taurus_newGene_35890	NA	NA	NA
Bos_taurus_newGene_35891	NA	NA	NA
Bos_taurus_newGene_35892	NA	NA	NA
Bos_taurus_newGene_35902	NA	NA	NA
Bos_taurus_newGene_35903	NA	NA	NA
Bos_taurus_newGene_35904	NA	NA	NA
Bos_taurus_newGene_35982	0.1174254	0.7183378	0.1436713
Bos_taurus_newGene_35996	NA	NA	NA
Bos_taurus_newGene_36010	-0.265124	0.5774627	0.2384761
Bos_taurus_newGene_36034	NA	NA	NA
Bos_taurus_newGene_36120	NA	NA	NA
Bos_taurus_newGene_36141	NA	NA	NA
Bos_taurus_newGene_36160	NA	NA	NA
Bos_taurus_newGene_36269	NA	NA	NA
Bos_taurus_newGene_36270	NA	NA	NA
Bos_taurus_newGene_36283	NA	NA	NA
Bos_taurus_newGene_36292	NA	NA	NA
Bos_taurus_newGene_36293	NA	NA	NA
Bos_taurus_newGene_36297	NA	NA	NA
Bos_taurus_newGene_36330	NA	NA	NA
Bos_taurus_newGene_36367	NA	NA	NA
Bos_taurus_newGene_36373	NA	NA	NA
Bos_taurus_newGene_36414	NA	NA	NA
Bos_taurus_newGene_36459	NA	NA	NA
Bos_taurus_newGene_36468	NA	NA	NA
Bos_taurus_newGene_36483	NA	NA	NA

Bos_taurus_newGene_36484	NA	NA	NA
Bos_taurus_newGene_36588	NA	NA	NA
Bos_taurus_newGene_36589	NA	NA	NA
Bos_taurus_newGene_36591	NA	NA	NA
Bos_taurus_newGene_36600	NA	NA	NA
Bos_taurus_newGene_36639	NA	NA	NA
Bos_taurus_newGene_36649	NA	NA	NA
Bos_taurus_newGene_36656	NA	NA	NA
Bos_taurus_newGene_36658	NA	NA	NA
Bos_taurus_newGene_36674	NA	NA	NA
Bos_taurus_newGene_36676	NA	NA	NA
Bos_taurus_newGene_36690	NA	NA	NA
Bos_taurus_newGene_36725	NA	NA	NA
Bos_taurus_newGene_36823	0.8505385	0.0342917	1.4648105
Bos_taurus_newGene_36871	0.167518	0.7909342	0.1018597
Bos_taurus_newGene_36894	NA	NA	NA
Bos_taurus_newGene_36911	NA	NA	NA
Bos_taurus_newGene_36936	-0.462619	0.5201188	0.2838974
Bos_taurus_newGene_37111	NA	NA	NA
Bos_taurus_newGene_37126	0.0413066	0.9169169	0.03767
Bos_taurus_newGene_37128	NA	NA	NA
Bos_taurus_newGene_37151	NA	NA	NA
Bos_taurus_newGene_37154	NA	NA	NA
Bos_taurus_newGene_37156	NA	NA	NA
Bos_taurus_newGene_37165	-0.227914	0.5156701	0.2876281
Bos_taurus_newGene_37169	0.2032441	0.7132159	0.146779
Bos_taurus_newGene_37222	NA	NA	NA
Bos_taurus_newGene_37231	NA	NA	NA
Bos_taurus_newGene_37232	0.4614884	0.2123794	0.6728877
Bos_taurus_newGene_37235	-0.607893	0.0906328	1.0427148
Bos_taurus_newGene_37243	-0.767913	0.2021991	0.6942207
Bos_taurus_newGene_37271	-0.241205	0.3499254	0.4560246
Bos_taurus_newGene_37306	-0.138881	0.6046273	0.2185122
Bos_taurus_newGene_37352	NA	NA	NA
Bos_taurus_newGene_37354	NA	NA	NA
Bos_taurus_newGene_37363	NA	NA	NA
Bos_taurus_newGene_37384	NA	NA	NA
Bos_taurus_newGene_37394	NA	NA	NA
Bos_taurus_newGene_37398	NA	NA	NA
Bos_taurus_newGene_37421	NA	NA	NA
Bos_taurus_newGene_37422	NA	NA	NA
Bos_taurus_newGene_37430	NA	NA	NA
Bos_taurus_newGene_37431	NA	NA	NA
Bos_taurus_newGene_37470	NA	NA	NA
Bos_taurus_newGene_37478	NA	NA	NA
Bos_taurus_newGene_37492	-0.53731	0.3481393	0.458247
Bos_taurus_newGene_37515	NA	NA	NA
Bos_taurus_newGene_37524	NA	NA	NA
Bos_taurus_newGene_37563	NA	NA	NA
Bos_taurus_newGene_37564	0.7830776	0.6821124	0.166144
Bos_taurus_newGene_37573	NA	NA	NA
Bos_taurus_newGene_37590	NA	NA	NA
Bos_taurus_newGene_37658	NA	NA	NA
Bos_taurus_newGene_37678	NA	NA	NA
Bos_taurus_newGene_37688	NA	NA	NA
Bos_taurus_newGene_37757	NA	NA	NA
Bos_taurus_newGene_37761	NA	NA	NA
Bos_taurus_newGene_37783	NA	NA	NA

Bos_taurus_newGene_37800	NA	NA	NA
Bos_taurus_newGene_37801	NA	NA	NA
Bos_taurus_newGene_37808	NA	NA	NA
Bos_taurus_newGene_37811	NA	NA	NA
Bos_taurus_newGene_37832	NA	NA	NA
Bos_taurus_newGene_37839	NA	NA	NA
Bos_taurus_newGene_37855	NA	NA	NA
Bos_taurus_newGene_37860	NA	NA	NA
Bos_taurus_newGene_37861	NA	NA	NA
Bos_taurus_newGene_37865	NA	NA	NA
Bos_taurus_newGene_37870	-0.451393	0.1331267	0.8757348
Bos_taurus_newGene_37871	NA	NA	NA
Bos_taurus_newGene_37877	-0.669887	0.1479129	0.8299938
Bos_taurus_newGene_37891	NA	NA	NA
Bos_taurus_newGene_37910	0.2829801	0.5491789	0.2602862
Bos_taurus_newGene_37944	NA	NA	NA
Bos_taurus_newGene_37964	NA	NA	NA
Bos_taurus_newGene_37966	NA	NA	NA
Bos_taurus_newGene_37973	NA	NA	NA
Bos_taurus_newGene_37974	NA	NA	NA
Bos_taurus_newGene_37986	0.3036005	0.5522381	0.2578737
Bos_taurus_newGene_37987	1.0870591	0.0274147	1.5620172
Bos_taurus_newGene_37992	NA	NA	NA
Bos_taurus_newGene_38052	NA	NA	NA
Bos_taurus_newGene_38083	0.780578	0.1088602	0.963131
Bos_taurus_newGene_38084	0.2317905	0.647613	0.1886844
Bos_taurus_newGene_38086	NA	NA	NA
Bos_taurus_newGene_38091	-0.125064	0.7869952	0.1040279
Bos_taurus_newGene_38092	-0.391483	0.3749322	0.4260473
Bos_taurus_newGene_38103	NA	NA	NA
Bos_taurus_newGene_38110	NA	NA	NA
Bos_taurus_newGene_38111	-0.235197	0.6078474	0.2162054
Bos_taurus_newGene_38112	-0.364306	0.2921463	0.5343996
Bos_taurus_newGene_38212	NA	NA	NA
Bos_taurus_newGene_38280	-0.445173	0.5147206	0.2884285
Bos_taurus_newGene_38290	NA	NA	NA
Bos_taurus_newGene_38396	-0.004533	1	0
Bos_taurus_newGene_38441	NA	NA	NA
Bos_taurus_newGene_38452	NA	NA	NA
Bos_taurus_newGene_38477	NA	NA	NA
Bos_taurus_newGene_38518	NA	NA	NA
Bos_taurus_newGene_38565	NA	NA	NA
Bos_taurus_newGene_38568	NA	NA	NA
Bos_taurus_newGene_38576	NA	NA	NA
Bos_taurus_newGene_38589	NA	NA	NA
Bos_taurus_newGene_38622	-0.44212	0.4194615	0.3773079
Bos_taurus_newGene_38682	NA	NA	NA
Bos_taurus_newGene_38698	NA	NA	NA
Bos_taurus_newGene_38751	NA	NA	NA
Bos_taurus_newGene_38797	NA	NA	NA
Bos_taurus_newGene_38802	-0.882991	0.0606461	1.2171968
Bos_taurus_newGene_38819	-1.889347	0.0005116	3.2911119
Bos_taurus_newGene_38820	NA	NA	NA
Bos_taurus_newGene_38821	NA	NA	NA
Bos_taurus_newGene_38839	NA	NA	NA
Bos_taurus_newGene_38853	NA	NA	NA
Bos_taurus_newGene_38858	NA	NA	NA
Bos_taurus_newGene_38859	NA	NA	NA

Bos_taurus_newGene_38887	0.2705953	0.3428108	0.4649455
Bos_taurus_newGene_38890	NA	NA	NA
Bos_taurus_newGene_38898	NA	NA	NA
Bos_taurus_newGene_38918	0.6319624	0.1736484	0.7603292
Bos_taurus_newGene_38927	NA	NA	NA
Bos_taurus_newGene_38943	NA	NA	NA
Bos_taurus_newGene_38948	NA	NA	NA
Bos_taurus_newGene_38949	NA	NA	NA
Bos_taurus_newGene_38956	NA	NA	NA
Bos_taurus_newGene_38959	NA	NA	NA
Bos_taurus_newGene_38986	NA	NA	NA
Bos_taurus_newGene_38998	-0.535026	0.2637763	0.5787642
Bos_taurus_newGene_38999	0.0166369	0.9847672	0.0066664
Bos_taurus_newGene_39025	NA	NA	NA
Bos_taurus_newGene_39038	NA	NA	NA
Bos_taurus_newGene_39043	NA	NA	NA
Bos_taurus_newGene_39047	-0.223524	0.6201069	0.2075334
Bos_taurus_newGene_39056	0.3054171	0.4750845	0.3232291
Bos_taurus_newGene_39059	NA	NA	NA
Bos_taurus_newGene_39116	NA	NA	NA
Bos_taurus_newGene_39117	NA	NA	NA
Bos_taurus_newGene_39120	NA	NA	NA
Bos_taurus_newGene_39130	NA	NA	NA
Bos_taurus_newGene_39145	NA	NA	NA
Bos_taurus_newGene_39152	NA	NA	NA
Bos_taurus_newGene_39157	0.1938829	0.6461398	0.1896735
Bos_taurus_newGene_39167	NA	NA	NA
Bos_taurus_newGene_39169	NA	NA	NA
Bos_taurus_newGene_39273	NA	NA	NA
Bos_taurus_newGene_39354	NA	NA	NA
Bos_taurus_newGene_39387	NA	NA	NA
Bos_taurus_newGene_39394	-0.517787	0.3065349	0.5135201
Bos_taurus_newGene_39443	NA	NA	NA
Bos_taurus_newGene_39462	NA	NA	NA
Bos_taurus_newGene_39475	NA	NA	NA
Bos_taurus_newGene_39504	NA	NA	NA
Bos_taurus_newGene_39528	1.034373	0.0199919	1.6991464
Bos_taurus_newGene_39545	NA	NA	NA
Bos_taurus_newGene_39557	NA	NA	NA
Bos_taurus_newGene_39618	NA	NA	NA
Bos_taurus_newGene_39623	NA	NA	NA
Bos_taurus_newGene_39648	NA	NA	NA
Bos_taurus_newGene_39789	NA	NA	NA
Bos_taurus_newGene_39806	NA	NA	NA
Bos_taurus_newGene_39822	NA	NA	NA
Bos_taurus_newGene_39873	NA	NA	NA
Bos_taurus_newGene_39896	NA	NA	NA
Bos_taurus_newGene_39903	NA	NA	NA
Bos_taurus_newGene_40040	NA	NA	NA
Bos_taurus_newGene_40074	-0.204766	0.6657726	0.1766741
Bos_taurus_newGene_40108	0.4857319	0.2810619	0.5511981
Bos_taurus_newGene_40153	NA	NA	NA
Bos_taurus_newGene_40154	NA	NA	NA
Bos_taurus_newGene_40155	-0.468391	0.3199674	0.4948943
Bos_taurus_newGene_40181	NA	NA	NA
Bos_taurus_newGene_40185	NA	NA	NA
Bos_taurus_newGene_40190	NA	NA	NA
Bos_taurus_newGene_40255	-0.644204	0.1043976	0.9813094

Bos_taurus_newGene_40266	NA	NA	NA
Bos_taurus_newGene_40276	NA	NA	NA
Bos_taurus_newGene_40404	NA	NA	NA
Bos_taurus_newGene_40442	NA	NA	NA
Bos_taurus_newGene_40621	NA	NA	NA
Bos_taurus_newGene_40672	NA	NA	NA
Bos_taurus_newGene_40775	NA	NA	NA
Bos_taurus_newGene_40782	0.6688889	0.1366182	0.8644913
Bos_taurus_newGene_40793	-0.625875	0.0848869	1.0711592
Bos_taurus_newGene_40817	-0.332403	0.539342	0.2681357
Bos_taurus_newGene_40855	1.264316	0.0221332	1.654956
Bos_taurus_newGene_40862	-1.615528	0.0007154	3.1454547
Bos_taurus_newGene_40865	NA	NA	NA
Bos_taurus_newGene_40867	NA	NA	NA
Bos_taurus_newGene_40869	-1.333617	0.0067767	2.1689789
Bos_taurus_newGene_40871	NA	NA	NA
Bos_taurus_newGene_40879	NA	NA	NA
Bos_taurus_newGene_40888	NA	NA	NA
Bos_taurus_newGene_40975	NA	NA	NA
Bos_taurus_newGene_41011	-0.592168	0.044766	1.3490512
Bos_taurus_newGene_41099	NA	NA	NA
Bos_taurus_newGene_41136	NA	NA	NA
Bos_taurus_newGene_41169	NA	NA	NA
Bos_taurus_newGene_41215	0.129668	0.8291701	0.0813563
Bos_taurus_newGene_41234	-1.068772	0.0539626	1.2679072
Bos_taurus_newGene_41251	NA	NA	NA
Bos_taurus_newGene_41426	NA	NA	NA
Bos_taurus_newGene_41434	NA	NA	NA
Bos_taurus_newGene_41439	NA	NA	NA
Bos_taurus_newGene_41452	NA	NA	NA
Bos_taurus_newGene_41464	NA	NA	NA
Bos_taurus_newGene_41610	NA	NA	NA
Bos_taurus_newGene_41611	-0.422367	0.2847324	0.5455631
Bos_taurus_newGene_41662	NA	NA	NA
Bos_taurus_newGene_41669	-0.067603	0.7898905	0.1024331
Bos_taurus_newGene_41684	NA	NA	NA
Bos_taurus_newGene_41686	NA	NA	NA
Bos_taurus_newGene_41694	0.311748	0.5417918	0.2661676
Bos_taurus_newGene_41701	NA	NA	NA
Bos_taurus_newGene_41770	NA	NA	NA
Bos_taurus_newGene_41781	NA	NA	NA
Bos_taurus_newGene_41793	NA	NA	NA
Bos_taurus_newGene_41893	NA	NA	NA
Bos_taurus_newGene_41899	NA	NA	NA
Bos_taurus_newGene_41989	NA	NA	NA
Bos_taurus_newGene_42069	NA	NA	NA
Bos_taurus_newGene_42128	NA	NA	NA
Bos_taurus_newGene_42129	NA	NA	NA
Bos_taurus_newGene_42130	NA	NA	NA
Bos_taurus_newGene_42250	NA	NA	NA
Bos_taurus_newGene_42260	0.4878652	0.26707	0.5733748
Bos_taurus_newGene_42378	NA	NA	NA
Bos_taurus_newGene_42457	NA	NA	NA
Bos_taurus_newGene_42537	NA	NA	NA
Bos_taurus_newGene_42613	0.2429226	0.704759	0.1519594
Bos_taurus_newGene_42639	NA	NA	NA
Bos_taurus_newGene_42646	NA	NA	NA
Bos_taurus_newGene_42759	NA	NA	NA

Bos_taurus_newGene_42785	NA	NA	NA
Bos_taurus_newGene_42910	NA	NA	NA
Bos_taurus_newGene_42953	0.5360249	0.1619548	0.7906062
Bos_taurus_newGene_42962	NA	NA	NA
Bos_taurus_newGene_42995	NA	NA	NA
Bos_taurus_newGene_43067	NA	NA	NA
Bos_taurus_newGene_43080	NA	NA	NA
Bos_taurus_newGene_43084	NA	NA	NA
Bos_taurus_newGene_43143	NA	NA	NA
Bos_taurus_newGene_43155	NA	NA	NA
Bos_taurus_newGene_43156	-0.159671	0.7489237	0.1255624
Bos_taurus_newGene_43161	NA	NA	NA
Bos_taurus_newGene_43162	NA	NA	NA
Bos_taurus_newGene_43163	NA	NA	NA
Bos_taurus_newGene_43164	NA	NA	NA
Bos_taurus_newGene_43165	NA	NA	NA
Bos_taurus_newGene_43166	NA	NA	NA
Bos_taurus_newGene_43169	NA	NA	NA
Bos_taurus_newGene_43174	NA	NA	NA
Bos_taurus_newGene_43183	NA	NA	NA
Bos_taurus_newGene_43184	NA	NA	NA
Bos_taurus_newGene_43186	NA	NA	NA
Bos_taurus_newGene_43188	NA	NA	NA
Bos_taurus_newGene_43192	NA	NA	NA
Bos_taurus_newGene_43193	NA	NA	NA
Bos_taurus_newGene_43194	NA	NA	NA
Bos_taurus_newGene_43195	NA	NA	NA
Bos_taurus_newGene_43196	NA	NA	NA
Bos_taurus_newGene_43197	NA	NA	NA
Bos_taurus_newGene_43198	NA	NA	NA
Bos_taurus_newGene_43199	NA	NA	NA
Bos_taurus_newGene_43200	NA	NA	NA
Bos_taurus_newGene_43202	NA	NA	NA
Bos_taurus_newGene_43203	NA	NA	NA
Bos_taurus_newGene_43207	NA	NA	NA
Bos_taurus_newGene_43209	NA	NA	NA
Bos_taurus_newGene_43211	NA	NA	NA
Bos_taurus_newGene_43213	NA	NA	NA
Bos_taurus_newGene_43214	NA	NA	NA
Bos_taurus_newGene_43217	NA	NA	NA
Bos_taurus_newGene_43218	NA	NA	NA
Bos_taurus_newGene_43219	NA	NA	NA
Bos_taurus_newGene_43220	NA	NA	NA
Bos_taurus_newGene_43221	NA	NA	NA
Bos_taurus_newGene_43222	NA	NA	NA
Bos_taurus_newGene_43224	NA	NA	NA
Bos_taurus_newGene_43225	NA	NA	NA
Bos_taurus_newGene_43268	NA	NA	NA
Bos_taurus_newGene_43317	NA	NA	NA
Bos_taurus_newGene_43398	NA	NA	NA
Bos_taurus_newGene_43416	NA	NA	NA
Bos_taurus_newGene_43420	NA	NA	NA
Bos_taurus_newGene_43430	NA	NA	NA
Bos_taurus_newGene_43431	NA	NA	NA
Bos_taurus_newGene_43432	NA	NA	NA
Bos_taurus_newGene_43433	NA	NA	NA
Bos_taurus_newGene_43434	NA	NA	NA
Bos_taurus_newGene_43435	NA	NA	NA

Bos_taurus_newGene_43436	NA	NA	NA
Bos_taurus_newGene_43437	NA	NA	NA
Bos_taurus_newGene_43451	NA	NA	NA
Bos_taurus_newGene_43454	NA	NA	NA
Bos_taurus_newGene_43484	0.1973868	0.6075418	0.2164238
Bos_taurus_newGene_43485	0.6064565	0.291781	0.534943
Bos_taurus_newGene_43507	-0.466517	0.2909074	0.5362453
Bos_taurus_newGene_43540	NA	NA	NA
Bos_taurus_newGene_43963	NA	NA	NA
Bos_taurus_newGene_44002	NA	NA	NA
Bos_taurus_newGene_44047	NA	NA	NA
Bos_taurus_newGene_44147	NA	NA	NA
Bos_taurus_newGene_44178	-0.12741	0.7604441	0.1189327
Bos_taurus_newGene_44181	NA	NA	NA
Bos_taurus_newGene_44182	0.4056292	0.3396307	0.468993
Bos_taurus_newGene_44183	NA	NA	NA
Bos_taurus_newGene_44185	NA	NA	NA
Bos_taurus_newGene_44243	-0.156669	0.6752018	0.1705664
Bos_taurus_newGene_44268	NA	NA	NA
Bos_taurus_newGene_44319	0.0737169	0.8921889	0.0495432
Bos_taurus_newGene_44320	-0.308236	0.5668118	0.2465611
Bos_taurus_newGene_44357	NA	NA	NA
Bos_taurus_newGene_44448	NA	NA	NA
Bos_taurus_newGene_44533	1.0621802	0.060295	1.219719
Bos_taurus_newGene_44574	NA	NA	NA
Bos_taurus_newGene_44577	NA	NA	NA
Bos_taurus_newGene_44628	NA	NA	NA
Bos_taurus_newGene_44639	NA	NA	NA
Bos_taurus_newGene_44643	NA	NA	NA
Bos_taurus_newGene_44688	NA	NA	NA
Bos_taurus_newGene_44695	NA	NA	NA
Bos_taurus_newGene_44753	NA	NA	NA
Bos_taurus_newGene_44754	NA	NA	NA
Bos_taurus_newGene_44764	NA	NA	NA
Bos_taurus_newGene_44813	NA	NA	NA
Bos_taurus_newGene_44820	-0.46745	0.2998461	0.5231015
Bos_taurus_newGene_44832	NA	NA	NA
Bos_taurus_newGene_44844	-0.438586	0.3443964	0.4629413
Bos_taurus_newGene_44889	NA	NA	NA
Bos_taurus_newGene_44893	NA	NA	NA
Bos_taurus_newGene_44954	NA	NA	NA
Bos_taurus_newGene_44982	NA	NA	NA
Bos_taurus_newGene_45058	NA	NA	NA
Bos_taurus_newGene_45072	0.0824458	0.8253346	0.08337
Bos_taurus_newGene_45105	NA	NA	NA
Bos_taurus_newGene_45134	NA	NA	NA
Bos_taurus_newGene_45172	NA	NA	NA
Bos_taurus_newGene_45326	NA	NA	NA
Bos_taurus_newGene_45379	NA	NA	NA
Bos_taurus_newGene_45385	NA	NA	NA
Bos_taurus_newGene_45456	NA	NA	NA
Bos_taurus_newGene_45473	NA	NA	NA
Bos_taurus_newGene_45502	NA	NA	NA
Bos_taurus_newGene_45591	NA	NA	NA
Bos_taurus_newGene_45592	NA	NA	NA
Bos_taurus_newGene_45593	NA	NA	NA
Bos_taurus_newGene_45625	NA	NA	NA
Bos_taurus_newGene_45630	NA	NA	NA

Bos_taurus_newGene_45650	NA	NA	NA
Bos_taurus_newGene_45657	NA	NA	NA
Bos_taurus_newGene_45661	NA	NA	NA
Bos_taurus_newGene_45684	NA	NA	NA
Bos_taurus_newGene_45696	NA	NA	NA
Bos_taurus_newGene_45741	NA	NA	NA
Bos_taurus_newGene_45745	NA	NA	NA
Bos_taurus_newGene_45747	-0.419573	0.1226168	0.9114501
Bos_taurus_newGene_45748	NA	NA	NA
Bos_taurus_newGene_45749	0.23138	0.5098474	0.2925598
Bos_taurus_newGene_45752	NA	NA	NA
Bos_taurus_newGene_45760	-0.416733	0.2508945	0.6005088
Bos_taurus_newGene_45775	-0.365976	0.44056	0.355995
Bos_taurus_newGene_45787	-0.087282	0.824466	0.0838273
Bos_taurus_newGene_45795	0.3363486	0.5060823	0.2957789
Bos_taurus_newGene_45809	NA	NA	NA
Bos_taurus_newGene_45810	NA	NA	NA
Bos_taurus_newGene_45843	-0.236528	0.632172	0.1991648
Bos_taurus_newGene_45847	0.1508113	0.7924241	0.1010423
Bos_taurus_newGene_45859	NA	NA	NA
Bos_taurus_newGene_45874	NA	NA	NA
Bos_taurus_newGene_45875	-0.865974	0.2763948	0.5584702
Bos_taurus_newGene_45940	0.0421781	0.9186053	0.0368711
Bos_taurus_newGene_45942	NA	NA	NA
Bos_taurus_newGene_45945	NA	NA	NA
Bos_taurus_newGene_45986	0.8140185	0.117363	0.9304687
Bos_taurus_newGene_45988	NA	NA	NA
Bos_taurus_newGene_45991	-0.43208	0.3117593	0.5061806
Bos_taurus_newGene_46003	-0.15158	0.7472274	0.1265472
Bos_taurus_newGene_46009	NA	NA	NA
Bos_taurus_newGene_46016	NA	NA	NA
Bos_taurus_newGene_46027	NA	NA	NA
Bos_taurus_newGene_46036	NA	NA	NA
Bos_taurus_newGene_46045	-0.16245	0.6263407	0.2031893
Bos_taurus_newGene_46078	NA	NA	NA
Bos_taurus_newGene_46107	NA	NA	NA
Bos_taurus_newGene_46289	NA	NA	NA
Bos_taurus_newGene_46290	NA	NA	NA
Bos_taurus_newGene_46291	NA	NA	NA
Bos_taurus_newGene_46292	NA	NA	NA
Bos_taurus_newGene_46297	-0.846578	0.1737904	0.7599742
Bos_taurus_newGene_46302	-0.152689	0.7892708	0.102774
Bos_taurus_newGene_46386	NA	NA	NA
Bos_taurus_newGene_46396	-0.124347	0.7770216	0.1095669
Bos_taurus_newGene_46400	NA	NA	NA
Bos_taurus_newGene_46419	-0.226883	0.4084218	0.388891
Bos_taurus_newGene_46430	NA	NA	NA
Bos_taurus_newGene_46437	-0.073229	0.8812235	0.0549139
Bos_taurus_newGene_46492	-0.363246	0.4991844	0.301739
Bos_taurus_newGene_46501	NA	NA	NA
Bos_taurus_newGene_46518	NA	NA	NA
Bos_taurus_newGene_46535	NA	NA	NA
Bos_taurus_newGene_46536	NA	NA	NA
Bos_taurus_newGene_46581	NA	NA	NA
Bos_taurus_newGene_46618	NA	NA	NA
Bos_taurus_newGene_46620	NA	NA	NA
Bos_taurus_newGene_46622	NA	NA	NA
Bos_taurus_newGene_46702	NA	NA	NA

Bos_taurus_newGene_46707	NA	NA	NA
Bos_taurus_newGene_46709	NA	NA	NA
Bos_taurus_newGene_46797	NA	NA	NA
Bos_taurus_newGene_46823	0.0116259	0.9664927	0.0148014
Bos_taurus_newGene_46824	NA	NA	NA
Bos_taurus_newGene_46825	NA	NA	NA
Bos_taurus_newGene_46852	0.4301508	0.3791208	0.4212224
Bos_taurus_newGene_46907	-0.183639	0.5912571	0.2282236
Bos_taurus_newGene_46910	0.2655019	0.6868322	0.1631494
Bos_taurus_newGene_46940	NA	NA	NA
Bos_taurus_newGene_47022	NA	NA	NA
Bos_taurus_newGene_47051	NA	NA	NA
Bos_taurus_newGene_47063	NA	NA	NA
Bos_taurus_newGene_47065	NA	NA	NA
Bos_taurus_newGene_47066	0.536477	0.2650843	0.576616
Bos_taurus_newGene_47111	NA	NA	NA
Bos_taurus_newGene_47270	0.6773771	0.1804444	0.7436566
Bos_taurus_newGene_47275	0.1358125	0.8066502	0.0933147
Bos_taurus_newGene_47281	NA	NA	NA
Bos_taurus_newGene_47284	0.259791	0.4726321	0.3254768
Bos_taurus_newGene_47294	NA	NA	NA
Bos_taurus_newGene_47308	0.8107904	0.1306355	0.8839387
Bos_taurus_newGene_47311	0.6159569	0.277845	0.5561974
Bos_taurus_newGene_47432	-0.396504	0.3232981	0.4903968
Bos_taurus_newGene_47433	NA	NA	NA
Bos_taurus_newGene_47434	NA	NA	NA
Bos_taurus_newGene_47443	-0.250987	0.6826632	0.1657935
Bos_taurus_newGene_47446	-0.352712	0.4388473	0.3576865
Bos_taurus_newGene_47448	-0.767864	0.1875121	0.7269708
Bos_taurus_newGene_47452	-0.201531	0.6483067	0.1882195
Bos_taurus_newGene_47457	NA	NA	NA
Bos_taurus_newGene_47458	-0.274843	0.6038175	0.2190943
Bos_taurus_newGene_47465	0.509869	0.312757	0.504793
Bos_taurus_newGene_47502	NA	NA	NA
Bos_taurus_newGene_47503	NA	NA	NA
Bos_taurus_newGene_47504	NA	NA	NA
Bos_taurus_newGene_47505	NA	NA	NA
Bos_taurus_newGene_47506	NA	NA	NA
Bos_taurus_newGene_47509	NA	NA	NA
Bos_taurus_newGene_47513	NA	NA	NA
Bos_taurus_newGene_47517	NA	NA	NA
Bos_taurus_newGene_47519	NA	NA	NA
Bos_taurus_newGene_47520	NA	NA	NA
Bos_taurus_newGene_47554	NA	NA	NA
Bos_taurus_newGene_47568	NA	NA	NA
Bos_taurus_newGene_47583	NA	NA	NA
Bos_taurus_newGene_47584	NA	NA	NA
Bos_taurus_newGene_47585	NA	NA	NA
Bos_taurus_newGene_47588	-0.251014	0.6921059	0.1598274
Bos_taurus_newGene_47591	NA	NA	NA
Bos_taurus_newGene_47592	NA	NA	NA
Bos_taurus_newGene_47593	NA	NA	NA
Bos_taurus_newGene_47594	NA	NA	NA
Bos_taurus_newGene_47598	NA	NA	NA
Bos_taurus_newGene_47613	NA	NA	NA
Bos_taurus_newGene_47699	NA	NA	NA
Bos_taurus_newGene_47748	NA	NA	NA
Bos_taurus_newGene_47749	NA	NA	NA

Bos_taurus_newGene_47776	0.2647283	0.5825135	0.234694
Bos_taurus_newGene_47780	-0.274642	0.2859475	0.5437137
Bos_taurus_newGene_47781	NA	NA	NA
Bos_taurus_newGene_47786	NA	NA	NA
Bos_taurus_newGene_47787	0.1360757	0.684885	0.1643823
Bos_taurus_newGene_47793	NA	NA	NA
Bos_taurus_newGene_47834	NA	NA	NA
Bos_taurus_newGene_47853	-0.025556	0.9720202	0.0123247
Bos_taurus_newGene_47854	0.3211737	0.4704533	0.3274834
Bos_taurus_newGene_47867	NA	NA	NA
Bos_taurus_newGene_47933	NA	NA	NA
Bos_taurus_newGene_47937	NA	NA	NA
Bos_taurus_newGene_47961	NA	NA	NA
Bos_taurus_newGene_47962	NA	NA	NA
Bos_taurus_newGene_47963	NA	NA	NA
Bos_taurus_newGene_47964	NA	NA	NA
Bos_taurus_newGene_47965	NA	NA	NA
Bos_taurus_newGene_47997	NA	NA	NA
Bos_taurus_newGene_48091	NA	NA	NA
Bos_taurus_newGene_48320	0.0736781	0.8810112	0.0550186
Bos_taurus_newGene_48322	0.0263965	0.954198	0.0203615
Bos_taurus_newGene_48323	NA	NA	NA
Bos_taurus_newGene_48324	0.0937992	0.8660473	0.0624584
Bos_taurus_newGene_48329	NA	NA	NA
Bos_taurus_newGene_48358	NA	NA	NA
Bos_taurus_newGene_48420	NA	NA	NA
Bos_taurus_newGene_48421	NA	NA	NA
Bos_taurus_newGene_48462	NA	NA	NA
Bos_taurus_newGene_48473	NA	NA	NA
Bos_taurus_newGene_48482	NA	NA	NA
Bos_taurus_newGene_48517	NA	NA	NA
Bos_taurus_newGene_48549	NA	NA	NA
Bos_taurus_newGene_48555	-0.410472	0.1591002	0.7983293
Bos_taurus_newGene_48563	-0.505519	0.157903	0.8016095
Bos_taurus_newGene_48579	NA	NA	NA
Bos_taurus_newGene_48583	-0.126895	0.719054	0.1432385
Bos_taurus_newGene_48587	-0.427487	0.4085353	0.3887704
Bos_taurus_newGene_48595	NA	NA	NA
Bos_taurus_newGene_48606	NA	NA	NA
Bos_taurus_newGene_48671	NA	NA	NA
Bos_taurus_newGene_48744	-0.086647	0.8616012	0.0646937
Bos_taurus_newGene_48748	NA	NA	NA
Bos_taurus_newGene_48749	-0.007675	0.9771548	0.0100366
Bos_taurus_newGene_48750	NA	NA	NA
Bos_taurus_newGene_48751	NA	NA	NA
Bos_taurus_newGene_48766	NA	NA	NA
Bos_taurus_newGene_48767	0.214047	0.6239659	0.2048391
Bos_taurus_newGene_48886	-0.693858	0.2671214	0.5732914
Bos_taurus_newGene_48901	NA	NA	NA
Bos_taurus_newGene_48964	0.0365597	0.9308712	0.0311104
Bos_taurus_newGene_48996	NA	NA	NA
Bos_taurus_newGene_49048	NA	NA	NA
Bos_taurus_newGene_49065	NA	NA	NA
Bos_taurus_newGene_49076	-0.533806	0.3375501	0.4716618
Bos_taurus_newGene_49096	NA	NA	NA
Bos_taurus_newGene_49153	NA	NA	NA
Bos_taurus_newGene_49154	NA	NA	NA
Bos_taurus_newGene_49184	-0.752671	0.0055148	2.2584694

Bos_taurus_newGene_49187	-1.612485	3.11E-05	4.5072368
Bos_taurus_newGene_49190	-1.441675	0.0001518	3.8187282
Bos_taurus_newGene_49198	-0.266579	0.4267186	0.3698584
Bos_taurus_newGene_49204	NA	NA	NA
Bos_taurus_newGene_49213	1.0294654	0.1419537	0.8478532
Bos_taurus_newGene_49221	NA	NA	NA
Bos_taurus_newGene_49223	-0.806405	0.0518251	1.2854599
Bos_taurus_newGene_49224	NA	NA	NA
Bos_taurus_newGene_49236	-0.710059	0.2637941	0.578735
Bos_taurus_newGene_49379	-0.241859	0.5719758	0.2426224
Bos_taurus_newGene_49406	0.8134646	0.0984444	1.0068088
Bos_taurus_newGene_49419	NA	NA	NA
Bos_taurus_newGene_49431	-0.675357	0.1682262	0.7741063
Bos_taurus_newGene_49432	-0.28862	0.6238752	0.2049023
Bos_taurus_newGene_49434	-0.443096	0.4463921	0.3502835
Bos_taurus_newGene_49437	-0.631143	0.3320379	0.4788124
Bos_taurus_newGene_49454	NA	NA	NA
Bos_taurus_newGene_49459	NA	NA	NA
Bos_taurus_newGene_49469	-0.906207	0.252367	0.5979675
Bos_taurus_newGene_49482	NA	NA	NA
Bos_taurus_newGene_49488	-2.349161	0.0047129	2.3267075
Bos_taurus_newGene_49516	NA	NA	NA
Bos_taurus_newGene_49524	-2.46189	0.0011829	2.9270689
Bos_taurus_newGene_49568	NA	NA	NA
Bos_taurus_newGene_49622	NA	NA	NA
Bos_taurus_newGene_49623	NA	NA	NA
Bos_taurus_newGene_49651	0.1877171	0.7754356	0.1104543
Bos_taurus_newGene_49774	NA	NA	NA
Bos_taurus_newGene_49779	NA	NA	NA
Bos_taurus_newGene_49784	-0.588346	0.1490516	0.8266633
Bos_taurus_newGene_49804	NA	NA	NA
Bos_taurus_newGene_49824	-0.036405	0.9285605	0.0321898
Bos_taurus_newGene_49839	NA	NA	NA
Bos_taurus_newGene_49841	-0.186713	0.7123326	0.1473172
Bos_taurus_newGene_49845	0.5729366	0.2871423	0.5419028
Bos_taurus_newGene_49846	NA	NA	NA
Bos_taurus_newGene_49855	NA	NA	NA
Bos_taurus_newGene_49894	NA	NA	NA
Bos_taurus_newGene_49939	NA	NA	NA
Bos_taurus_newGene_49940	NA	NA	NA
Bos_taurus_newGene_49955	NA	NA	NA
Bos_taurus_newGene_49957	NA	NA	NA
Bos_taurus_newGene_49961	NA	NA	NA
Bos_taurus_newGene_49966	NA	NA	NA
Bos_taurus_newGene_49971	NA	NA	NA
Bos_taurus_newGene_50004	0.2201371	0.6728358	0.1720909
Bos_taurus_newGene_50006	0.2511672	0.6441715	0.1909985
Bos_taurus_newGene_50057	NA	NA	NA
Bos_taurus_newGene_50141	NA	NA	NA
Bos_taurus_newGene_50145	NA	NA	NA
Bos_taurus_newGene_50205	-0.416131	0.1773121	0.7512615
Bos_taurus_newGene_50289	-0.921709	0.1331082	0.8757951
Bos_taurus_newGene_50309	NA	NA	NA
Bos_taurus_newGene_50311	NA	NA	NA
Bos_taurus_newGene_50358	NA	NA	NA
Bos_taurus_newGene_50476	NA	NA	NA
Bos_taurus_newGene_50569	-0.013724	0.9738291	0.0115172
Bos_taurus_newGene_50572	-0.178283	0.6557523	0.1832602

Bos_taurus_newGene_50580	NA	NA	NA
Bos_taurus_newGene_50607	NA	NA	NA
Bos_taurus_newGene_50664	0.4727133	0.2616215	0.5823266
Bos_taurus_newGene_50703	NA	NA	NA
Bos_taurus_newGene_50725	NA	NA	NA
Bos_taurus_newGene_50786	NA	NA	NA
Bos_taurus_newGene_50851	NA	NA	NA
Bos_taurus_newGene_51043	NA	NA	NA
Bos_taurus_newGene_51044	NA	NA	NA
Bos_taurus_newGene_51081	-0.217489	0.7521144	0.1237161
Bos_taurus_newGene_51096	NA	NA	NA
Bos_taurus_newGene_51236	NA	NA	NA
Bos_taurus_newGene_51282	NA	NA	NA
Bos_taurus_newGene_51306	NA	NA	NA
Bos_taurus_newGene_51349	NA	NA	NA
Bos_taurus_newGene_51519	NA	NA	NA
Bos_taurus_newGene_51587	-0.230408	0.4834842	0.3156177
Bos_taurus_newGene_51591	NA	NA	NA
Bos_taurus_newGene_51595	NA	NA	NA
Bos_taurus_newGene_51606	NA	NA	NA
Bos_taurus_newGene_51608	-1.079213	0.000165	3.7825266
Bos_taurus_newGene_51614	-0.978774	0.5173673	0.286201
Bos_taurus_newGene_51618	-0.753418	0.1746354	0.7578678
Bos_taurus_newGene_51628	NA	NA	NA
Bos_taurus_newGene_51630	NA	NA	NA
Bos_taurus_newGene_51631	NA	NA	NA
Bos_taurus_newGene_51633	NA	NA	NA
Bos_taurus_newGene_51637	-0.845731	0.0556621	1.2544402
Bos_taurus_newGene_51665	NA	NA	NA
Bos_taurus_newGene_51737	NA	NA	NA
Bos_taurus_newGene_51750	NA	NA	NA
Bos_taurus_newGene_51754	NA	NA	NA
Bos_taurus_newGene_51828	NA	NA	NA
Bos_taurus_newGene_52007	NA	NA	NA
Bos_taurus_newGene_52137	NA	NA	NA
Bos_taurus_newGene_52142	NA	NA	NA
Bos_taurus_newGene_52152	NA	NA	NA
Bos_taurus_newGene_52153	NA	NA	NA
Bos_taurus_newGene_52176	NA	NA	NA
Bos_taurus_newGene_52183	NA	NA	NA
Bos_taurus_newGene_52513	NA	NA	NA
Bos_taurus_newGene_52543	NA	NA	NA
Bos_taurus_newGene_52560	NA	NA	NA
Bos_taurus_newGene_52623	NA	NA	NA
Bos_taurus_newGene_52633	NA	NA	NA
Bos_taurus_newGene_52645	-0.1911	0.6790476	0.1680998
Bos_taurus_newGene_52654	-0.036001	0.9285312	0.0322035
Bos_taurus_newGene_52701	-1.034839	0.1423318	0.846698
Bos_taurus_newGene_52709	-0.055233	0.8843148	0.0533931
Bos_taurus_newGene_52718	-0.641958	0.0552688	1.2575199
Bos_taurus_newGene_52733	NA	NA	NA
Bos_taurus_newGene_52745	0.0701162	0.8858538	0.0526379
Bos_taurus_newGene_52768	-0.660881	0.2029602	0.6925891
Bos_taurus_newGene_52772	0.5958307	0.3248539	0.4883119
Bos_taurus_newGene_52781	NA	NA	NA
Bos_taurus_newGene_52790	NA	NA	NA
Bos_taurus_newGene_52808	0.1481143	0.7668198	0.1153067
Bos_taurus_newGene_52814	-0.232087	0.50141	0.299807

Bos_taurus_newGene_52817	-0.276709	0.4924821	0.3076096
Bos_taurus_newGene_52842	NA	NA	NA
Bos_taurus_newGene_52855	NA	NA	NA
Bos_taurus_newGene_52967	NA	NA	NA
Bos_taurus_newGene_52981	NA	NA	NA
Bos_taurus_newGene_52983	NA	NA	NA
Bos_taurus_newGene_52997	NA	NA	NA
Bos_taurus_newGene_53028	NA	NA	NA
Bos_taurus_newGene_53053	NA	NA	NA
Bos_taurus_newGene_53079	NA	NA	NA
Bos_taurus_newGene_53106	NA	NA	NA
Bos_taurus_newGene_53111	NA	NA	NA
Bos_taurus_newGene_53112	NA	NA	NA
Bos_taurus_newGene_53128	NA	NA	NA
Bos_taurus_newGene_53152	NA	NA	NA
Bos_taurus_newGene_53153	NA	NA	NA
Bos_taurus_newGene_53188	NA	NA	NA
Bos_taurus_newGene_53221	-0.265436	0.2954571	0.5295056
Bos_taurus_newGene_53222	NA	NA	NA
Bos_taurus_newGene_53225	-0.148683	0.8213436	0.0854751
Bos_taurus_newGene_53262	NA	NA	NA
Bos_taurus_newGene_53311	NA	NA	NA
Bos_taurus_newGene_53462	NA	NA	NA
Bos_taurus_newGene_53473	NA	NA	NA
Bos_taurus_newGene_53498	NA	NA	NA
Bos_taurus_newGene_53852	NA	NA	NA
Bos_taurus_newGene_53871	NA	NA	NA
Bos_taurus_newGene_53891	NA	NA	NA
Bos_taurus_newGene_53929	NA	NA	NA
Bos_taurus_newGene_54042	NA	NA	NA
Bos_taurus_newGene_54050	0.5080092	0.3974905	0.4006733
Bos_taurus_newGene_54056	-0.079848	0.9140822	0.0390148
Bos_taurus_newGene_54140	NA	NA	NA
Bos_taurus_newGene_54343	NA	NA	NA
Bos_taurus_newGene_54380	NA	NA	NA
Bos_taurus_newGene_54389	NA	NA	NA
Bos_taurus_newGene_54400	NA	NA	NA
Bos_taurus_newGene_54558	NA	NA	NA
Bos_taurus_newGene_54595	0.6115359	0.2893318	0.5386039
Bos_taurus_newGene_54597	0.0098377	0.9791086	0.0091692
Bos_taurus_newGene_54601	NA	NA	NA
Bos_taurus_newGene_54606	NA	NA	NA
Bos_taurus_newGene_54680	-0.222345	0.6310536	0.1999337
Bos_taurus_newGene_54752	-1.396038	0.0279962	1.5529009
Bos_taurus_newGene_54761	-1.933187	0.0004294	3.3671299
Bos_taurus_newGene_54770	NA	NA	NA
Bos_taurus_newGene_54788	NA	NA	NA
Bos_taurus_newGene_54813	NA	NA	NA
Bos_taurus_newGene_54832	-0.604736	0.1599111	0.7961214
Bos_taurus_newGene_54888	NA	NA	NA
Bos_taurus_newGene_54900	NA	NA	NA
Bos_taurus_newGene_54959	NA	NA	NA
Bos_taurus_newGene_54971	NA	NA	NA
Bos_taurus_newGene_54992	NA	NA	NA
Bos_taurus_newGene_55040	0.3695015	0.4746927	0.3235874
Bos_taurus_newGene_55058	NA	NA	NA
Bos_taurus_newGene_55063	NA	NA	NA
Bos_taurus_newGene_55080	NA	NA	NA

Bos_taurus_newGene_55087	NA	NA	NA
Bos_taurus_newGene_55108	NA	NA	NA
Bos_taurus_newGene_55125	NA	NA	NA
Bos_taurus_newGene_55145	NA	NA	NA
Bos_taurus_newGene_55178	NA	NA	NA
Bos_taurus_newGene_55183	NA	NA	NA
Bos_taurus_newGene_55256	-0.637607	0.153442	0.8140557
Bos_taurus_newGene_55261	NA	NA	NA
Bos_taurus_newGene_55274	NA	NA	NA
Bos_taurus_newGene_55294	NA	NA	NA
Bos_taurus_newGene_55313	NA	NA	NA
Bos_taurus_newGene_55401	NA	NA	NA
Bos_taurus_newGene_55402	NA	NA	NA
Bos_taurus_newGene_55404	0.0567207	0.8943615	0.0484869
Bos_taurus_newGene_55405	0.0988078	0.7965232	0.0988016
Bos_taurus_newGene_55435	NA	NA	NA
Bos_taurus_newGene_55501	-0.468505	0.4616838	0.3356553
Bos_taurus_newGene_55523	NA	NA	NA
Bos_taurus_newGene_55606	-0.397088	0.7179637	0.1438975
Bos_taurus_newGene_55613	NA	NA	NA
Bos_taurus_newGene_55645	-0.190077	0.6652319	0.1770269
Bos_taurus_newGene_55647	-0.336957	0.3219732	0.4921802
Bos_taurus_newGene_55649	-0.371461	0.4333557	0.3631555
Bos_taurus_newGene_55651	-0.304221	0.4675283	0.3301921
Bos_taurus_newGene_55658	-0.659902	0.0159305	1.7977714
Bos_taurus_newGene_55711	NA	NA	NA
Bos_taurus_newGene_55716	NA	NA	NA
Bos_taurus_newGene_55770	NA	NA	NA
Bos_taurus_newGene_55901	NA	NA	NA
Bos_taurus_newGene_55906	NA	NA	NA
Bos_taurus_newGene_55913	NA	NA	NA
Bos_taurus_newGene_55945	NA	NA	NA
Bos_taurus_newGene_55971	0.0634421	0.9041253	0.0437714
Bos_taurus_newGene_56062	NA	NA	NA
Bos_taurus_newGene_56065	0.0590773	0.9355962	0.0289115
Bos_taurus_newGene_56068	NA	NA	NA
Bos_taurus_newGene_56079	NA	NA	NA
Bos_taurus_newGene_56081	NA	NA	NA
Bos_taurus_newGene_56133	-0.342713	0.3415091	0.4665977
Bos_taurus_newGene_56135	NA	NA	NA
Bos_taurus_newGene_56136	NA	NA	NA
Bos_taurus_newGene_56144	NA	NA	NA
Bos_taurus_newGene_56146	NA	NA	NA
Bos_taurus_newGene_56259	NA	NA	NA
Bos_taurus_newGene_56265	NA	NA	NA
Bos_taurus_newGene_56300	NA	NA	NA
Bos_taurus_newGene_56358	NA	NA	NA
Bos_taurus_newGene_56410	NA	NA	NA
Bos_taurus_newGene_56549	-0.017497	0.9827199	0.0075702
Bos_taurus_newGene_56635	NA	NA	NA
Bos_taurus_newGene_56663	NA	NA	NA
Bos_taurus_newGene_56724	NA	NA	NA
Bos_taurus_newGene_56728	-0.439097	0.1299287	0.8862948
Bos_taurus_newGene_56729	-0.420566	0.4196563	0.3771063
Bos_taurus_newGene_56775	NA	NA	NA
Bos_taurus_newGene_56831	NA	NA	NA
Bos_taurus_newGene_56843	NA	NA	NA
Bos_taurus_newGene_56844	NA	NA	NA

Bos_taurus_newGene_56853	NA	NA	NA
Bos_taurus_newGene_56928	NA	NA	NA
Bos_taurus_newGene_56942	NA	NA	NA
Bos_taurus_newGene_56985	NA	NA	NA
Bos_taurus_newGene_57075	NA	NA	NA
Bos_taurus_newGene_57103	NA	NA	NA
Bos_taurus_newGene_57114	0.1994114	0.7041301	0.1523471
Bos_taurus_newGene_57123	NA	NA	NA
Bos_taurus_newGene_57124	-0.938636	0.0859062	1.0659753
Bos_taurus_newGene_57127	NA	NA	NA
Bos_taurus_newGene_57134	-0.464642	0.3266211	0.4859558
Bos_taurus_newGene_57136	NA	NA	NA
Bos_taurus_newGene_57210	0.0558039	0.8336861	0.0789974
Bos_taurus_newGene_57211	NA	NA	NA
Bos_taurus_newGene_57230	-0.217842	0.6635712	0.1781124
Bos_taurus_newGene_57373	NA	NA	NA
Bos_taurus_newGene_57401	NA	NA	NA
Bos_taurus_newGene_57412	NA	NA	NA
Bos_taurus_newGene_57470	NA	NA	NA
Bos_taurus_newGene_57495	-0.009601	0.9791513	0.0091502
Bos_taurus_newGene_57570	NA	NA	NA
Bos_taurus_newGene_57589	NA	NA	NA
Bos_taurus_newGene_57618	NA	NA	NA
Bos_taurus_newGene_57651	NA	NA	NA
Bos_taurus_newGene_57678	NA	NA	NA
Bos_taurus_newGene_57697	NA	NA	NA
Bos_taurus_newGene_57721	NA	NA	NA
Bos_taurus_newGene_57755	NA	NA	NA
Bos_taurus_newGene_57824	NA	NA	NA
Bos_taurus_newGene_57832	NA	NA	NA
Bos_taurus_newGene_57923	NA	NA	NA
Bos_taurus_newGene_57924	-0.031794	0.9655123	0.0152422
Bos_taurus_newGene_57979	NA	NA	NA
Bos_taurus_newGene_57989	NA	NA	NA
Bos_taurus_newGene_58004	NA	NA	NA
Bos_taurus_newGene_58010	-0.045932	0.9414394	0.0262076
Bos_taurus_newGene_58017	-0.068729	0.9202863	0.036077
Bos_taurus_newGene_58032	NA	NA	NA
Bos_taurus_newGene_58072	NA	NA	NA
Bos_taurus_newGene_58087	-1.763437	0.2335559	0.6316091
Bos_taurus_newGene_58097	NA	NA	NA
Bos_taurus_newGene_58112	NA	NA	NA
Bos_taurus_newGene_58114	0.0425664	0.9541233	0.0203955
Bos_taurus_newGene_58122	0.2002621	0.5657385	0.2473842
Bos_taurus_newGene_58135	0.0251129	0.9481066	0.0231428
Bos_taurus_newGene_58141	1.7220539	0.0148903	1.8270976
Bos_taurus_newGene_58149	NA	NA	NA
Bos_taurus_newGene_58187	NA	NA	NA
Bos_taurus_newGene_58207	NA	NA	NA
Bos_taurus_newGene_58226	NA	NA	NA
Bos_taurus_newGene_58235	NA	NA	NA
Bos_taurus_newGene_58242	NA	NA	NA
Bos_taurus_newGene_58335	0.5041558	0.3341671	0.4760363
Bos_taurus_newGene_58489	NA	NA	NA
Bos_taurus_newGene_58501	NA	NA	NA
Bos_taurus_newGene_58643	NA	NA	NA
Bos_taurus_newGene_58663	0.4691698	0.3747951	0.4262061
Bos_taurus_newGene_58665	NA	NA	NA

Bos_taurus_newGene_58670	-0.494335	0.3530617	0.4521494
Bos_taurus_newGene_58672	NA	NA	NA
Bos_taurus_newGene_58738	NA	NA	NA
Bos_taurus_newGene_58778	NA	NA	NA
Bos_taurus_newGene_58785	0.1898586	0.680623	0.1670934
Bos_taurus_newGene_58788	0.4783065	0.330116	0.4813334
Bos_taurus_newGene_58883	NA	NA	NA
Bos_taurus_newGene_58892	-0.375829	0.4169669	0.3798984
Bos_taurus_newGene_58918	0.5610591	0.4618891	0.3354623
Bos_taurus_newGene_58922	NA	NA	NA
Bos_taurus_newGene_58924	NA	NA	NA
Bos_taurus_newGene_58925	NA	NA	NA
Bos_taurus_newGene_58928	NA	NA	NA
Bos_taurus_newGene_58932	NA	NA	NA
Bos_taurus_newGene_58936	0.0589421	0.888642	0.0512732
Bos_taurus_newGene_58944	NA	NA	NA
Bos_taurus_newGene_58949	-0.253701	0.5553876	0.2554038
Bos_taurus_newGene_59005	NA	NA	NA
Bos_taurus_newGene_59033	NA	NA	NA
Bos_taurus_newGene_59052	NA	NA	NA
Bos_taurus_newGene_59076	0.8270467	0.0957161	1.0190152
Bos_taurus_newGene_59077	-0.123429	0.793251	0.1005894
Bos_taurus_newGene_59080	-0.176792	0.7078177	0.1500786
Bos_taurus_newGene_59083	-0.364463	0.5088678	0.293395
Bos_taurus_newGene_59084	-0.025672	0.9592716	0.0180584
Bos_taurus_newGene_59087	NA	NA	NA
Bos_taurus_newGene_59153	NA	NA	NA
Bos_taurus_newGene_59216	NA	NA	NA
Bos_taurus_newGene_59340	-0.423322	0.2736764	0.5627627
Bos_taurus_newGene_59343	-0.375312	0.423158	0.3734974
Bos_taurus_newGene_59346	NA	NA	NA
Bos_taurus_newGene_59436	NA	NA	NA
Bos_taurus_newGene_59443	NA	NA	NA
Bos_taurus_newGene_59516	NA	NA	NA
Bos_taurus_newGene_59543	NA	NA	NA
Bos_taurus_newGene_59558	NA	NA	NA
Bos_taurus_newGene_59561	NA	NA	NA
Bos_taurus_newGene_59562	NA	NA	NA
Bos_taurus_newGene_59565	NA	NA	NA
Bos_taurus_newGene_59566	NA	NA	NA
Bos_taurus_newGene_59571	NA	NA	NA
Bos_taurus_newGene_59578	NA	NA	NA
Bos_taurus_newGene_59580	NA	NA	NA
Bos_taurus_newGene_59586	NA	NA	NA
Bos_taurus_newGene_59594	NA	NA	NA
Bos_taurus_newGene_59605	-0.414709	0.4160347	0.3808705
Bos_taurus_newGene_59630	NA	NA	NA
Bos_taurus_newGene_59640	0.8001996	0.1800875	0.7445163
Bos_taurus_newGene_59641	0.4950028	0.280446	0.5521507
Bos_taurus_newGene_59654	NA	NA	NA
Bos_taurus_newGene_59734	NA	NA	NA
Bos_taurus_newGene_59736	NA	NA	NA
Bos_taurus_newGene_59755	NA	NA	NA
Bos_taurus_newGene_59768	NA	NA	NA
Bos_taurus_newGene_59775	NA	NA	NA
Bos_taurus_newGene_59799	NA	NA	NA
Bos_taurus_newGene_59802	NA	NA	NA
Bos_taurus_newGene_59811	NA	NA	NA

Bos_taurus_newGene_59824	NA	NA	NA
Bos_taurus_newGene_59830	-2.227781	0.0002859	3.5437509
Bos_taurus_newGene_59832	NA	NA	NA
Bos_taurus_newGene_59846	NA	NA	NA
Bos_taurus_newGene_59887	NA	NA	NA
Bos_taurus_newGene_59924	NA	NA	NA
Bos_taurus_newGene_59972	NA	NA	NA
Bos_taurus_newGene_59976	NA	NA	NA
Bos_taurus_newGene_59994	NA	NA	NA
Bos_taurus_newGene_60006	NA	NA	NA
Bos_taurus_newGene_60068	NA	NA	NA
Bos_taurus_newGene_60114	0.2314848	0.6858577	0.163766
Bos_taurus_newGene_60119	NA	NA	NA
Bos_taurus_newGene_60133	0.2428695	0.7920517	0.1012465
Bos_taurus_newGene_60142	NA	NA	NA
Bos_taurus_newGene_60160	NA	NA	NA
Bos_taurus_newGene_60218	NA	NA	NA
Bos_taurus_newGene_60236	NA	NA	NA
Bos_taurus_newGene_60241	NA	NA	NA
Bos_taurus_newGene_60302	NA	NA	NA
Bos_taurus_newGene_60311	NA	NA	NA
Bos_taurus_newGene_60349	-0.077	0.9573923	0.0189101
Bos_taurus_newGene_60350	0.2029569	0.694004	0.158638
Bos_taurus_newGene_60359	-0.147007	0.7497169	0.1251027
Bos_taurus_newGene_60360	NA	NA	NA
Bos_taurus_newGene_60363	-0.326416	0.5052778	0.2964698
Bos_taurus_newGene_60390	NA	NA	NA
Bos_taurus_newGene_60471	NA	NA	NA
Bos_taurus_newGene_60549	NA	NA	NA
Bos_taurus_newGene_60555	NA	NA	NA
Bos_taurus_newGene_60560	NA	NA	NA
Bos_taurus_newGene_60567	NA	NA	NA
Bos_taurus_newGene_60569	NA	NA	NA
Bos_taurus_newGene_60573	NA	NA	NA
Bos_taurus_newGene_60574	NA	NA	NA
Bos_taurus_newGene_60575	NA	NA	NA
Bos_taurus_newGene_60576	NA	NA	NA
Bos_taurus_newGene_60578	NA	NA	NA
Bos_taurus_newGene_60580	NA	NA	NA
Bos_taurus_newGene_60583	NA	NA	NA
Bos_taurus_newGene_60586	NA	NA	NA
Bos_taurus_newGene_60591	NA	NA	NA
Bos_taurus_newGene_60639	NA	NA	NA
Bos_taurus_newGene_60659	-0.192123	0.7646761	0.1165225
Bos_taurus_newGene_60663	NA	NA	NA
Bos_taurus_newGene_60751	NA	NA	NA
Bos_taurus_newGene_60790	NA	NA	NA
Bos_taurus_newGene_60829	0.2548695	0.5011337	0.3000464
Bos_taurus_newGene_60833	0.2799178	0.5536783	0.2567425
Bos_taurus_newGene_60847	0.5779302	0.1310821	0.8824566
Bos_taurus_newGene_60911	-0.189285	0.7109323	0.1481718
Bos_taurus_newGene_60967	NA	NA	NA
Bos_taurus_newGene_60991	-1.042826	0.0349128	1.4570155
Bos_taurus_newGene_60996	NA	NA	NA
Bos_taurus_newGene_61016	NA	NA	NA
Bos_taurus_newGene_61017	NA	NA	NA
Bos_taurus_newGene_61032	NA	NA	NA
Bos_taurus_newGene_61086	0.8698354	0.1045303	0.9807578

Bos_taurus_newGene_61105	NA	NA	NA
Bos_taurus_newGene_61107	NA	NA	NA
Bos_taurus_newGene_61155	NA	NA	NA
Bos_taurus_newGene_61275	NA	NA	NA
Bos_taurus_newGene_61335	NA	NA	NA
Bos_taurus_newGene_61352	NA	NA	NA
Bos_taurus_newGene_61399	NA	NA	NA
Bos_taurus_newGene_61421	NA	NA	NA
Bos_taurus_newGene_61517	NA	NA	NA
Bos_taurus_newGene_61548	NA	NA	NA
Bos_taurus_newGene_61584	NA	NA	NA
Bos_taurus_newGene_61626	NA	NA	NA
Bos_taurus_newGene_61627	NA	NA	NA
Bos_taurus_newGene_61648	0.2214554	0.4247476	0.3718691
Bos_taurus_newGene_61649	NA	NA	NA
Bos_taurus_newGene_61681	NA	NA	NA
Bos_taurus_newGene_61705	NA	NA	NA
Bos_taurus_newGene_61706	NA	NA	NA
Bos_taurus_newGene_61707	NA	NA	NA
Bos_taurus_newGene_61708	NA	NA	NA
Bos_taurus_newGene_61710	NA	NA	NA
Bos_taurus_newGene_61711	NA	NA	NA
Bos_taurus_newGene_61712	NA	NA	NA
Bos_taurus_newGene_61723	NA	NA	NA
Bos_taurus_newGene_61790	NA	NA	NA
Bos_taurus_newGene_61792	NA	NA	NA
Bos_taurus_newGene_61839	0.5966969	0.2597415	0.5854587
Bos_taurus_newGene_61849	NA	NA	NA
Bos_taurus_newGene_61866	0.1698513	0.6501164	0.1870089
Bos_taurus_newGene_61885	NA	NA	NA
Bos_taurus_newGene_61895	-0.870068	0.0690945	1.1605566
Bos_taurus_newGene_61901	-0.889459	0.0038451	2.415098
Bos_taurus_newGene_61903	-2.109243	3.26E-05	4.486841
Bos_taurus_newGene_61906	NA	NA	NA
Bos_taurus_newGene_61909	-1.944569	8.39E-08	7.076163
Bos_taurus_newGene_61911	-1.520062	0.0007067	3.1507846
Bos_taurus_newGene_61918	-1.126916	0.0145761	1.8363574
Bos_taurus_newGene_61920	0.683772	0.4412458	0.3553194
Bos_taurus_newGene_61922	NA	NA	NA
Bos_taurus_newGene_61985	NA	NA	NA
Bos_taurus_newGene_61989	NA	NA	NA
Bos_taurus_newGene_62134	NA	NA	NA
Bos_taurus_newGene_62136	NA	NA	NA
Bos_taurus_newGene_62138	NA	NA	NA
Bos_taurus_newGene_62139	NA	NA	NA
Bos_taurus_newGene_62141	NA	NA	NA
Bos_taurus_newGene_62142	NA	NA	NA
Bos_taurus_newGene_62144	NA	NA	NA
Bos_taurus_newGene_62145	NA	NA	NA
Bos_taurus_newGene_62146	NA	NA	NA
Bos_taurus_newGene_62147	NA	NA	NA
Bos_taurus_newGene_62148	NA	NA	NA
Bos_taurus_newGene_62149	NA	NA	NA
Bos_taurus_newGene_62150	NA	NA	NA
Bos_taurus_newGene_62155	-0.170733	0.7385278	0.1316331
Bos_taurus_newGene_62156	NA	NA	NA
Bos_taurus_newGene_62177	NA	NA	NA
Bos_taurus_newGene_62189	NA	NA	NA

Bos_taurus_newGene_62236	NA	NA	NA
Bos_taurus_newGene_62237	NA	NA	NA
Bos_taurus_newGene_62238	NA	NA	NA
Bos_taurus_newGene_62239	NA	NA	NA
Bos_taurus_newGene_62240	NA	NA	NA
Bos_taurus_newGene_62303	NA	NA	NA
Bos_taurus_newGene_62332	NA	NA	NA
Bos_taurus_newGene_62334	0.2458148	0.57549	0.2399622
Bos_taurus_newGene_62343	-0.321385	0.3675795	0.4346487
Bos_taurus_newGene_62474	-0.230557	0.4045503	0.3930274
Bos_taurus_newGene_62475	-0.340315	0.4379665	0.3585591
Bos_taurus_newGene_62478	-0.172959	0.4960532	0.3044717
Bos_taurus_newGene_62484	NA	NA	NA
Bos_taurus_newGene_62563	NA	NA	NA
Bos_taurus_newGene_62601	-0.251456	0.670064	0.1738837
Bos_taurus_newGene_62603	0.1851261	0.7816102	0.1070098
Bos_taurus_newGene_62604	-0.014972	0.9810884	0.0082919
Bos_taurus_newGene_62612	-0.889024	0.0563333	1.2492351
Bos_taurus_newGene_62631	-0.044989	0.9322022	0.0304899
Bos_taurus_newGene_62633	NA	NA	NA
Bos_taurus_newGene_62668	NA	NA	NA
Bos_taurus_newGene_62731	NA	NA	NA
Bos_taurus_newGene_62762	NA	NA	NA
Bos_taurus_newGene_62769	NA	NA	NA
Bos_taurus_newGene_62772	NA	NA	NA
Bos_taurus_newGene_62773	NA	NA	NA
Bos_taurus_newGene_62776	NA	NA	NA
Bos_taurus_newGene_62777	NA	NA	NA
Bos_taurus_newGene_62779	NA	NA	NA
Bos_taurus_newGene_62781	NA	NA	NA
Bos_taurus_newGene_62785	NA	NA	NA
Bos_taurus_newGene_62789	NA	NA	NA
Bos_taurus_newGene_62790	NA	NA	NA
Bos_taurus_newGene_62791	NA	NA	NA
Bos_taurus_newGene_62792	NA	NA	NA
Bos_taurus_newGene_62794	NA	NA	NA
Bos_taurus_newGene_62795	NA	NA	NA
Bos_taurus_newGene_62825	NA	NA	NA
Bos_taurus_newGene_62868	NA	NA	NA
Bos_taurus_newGene_62943	NA	NA	NA
Bos_taurus_newGene_62948	NA	NA	NA
Bos_taurus_newGene_62993	NA	NA	NA
Bos_taurus_newGene_62998	NA	NA	NA
Bos_taurus_newGene_63016	NA	NA	NA
Bos_taurus_newGene_63025	-0.002361	1	0
Bos_taurus_newGene_63036	-0.005278	0.991491	0.0037112
Bos_taurus_newGene_63045	NA	NA	NA
Bos_taurus_newGene_63407	NA	NA	NA
Bos_taurus_newGene_63418	NA	NA	NA
Bos_taurus_newGene_63476	NA	NA	NA
Bos_taurus_newGene_63557	NA	NA	NA
Bos_taurus_newGene_63565	NA	NA	NA
Bos_taurus_newGene_63713	0.7414881	0.0854089	1.0684967
Bos_taurus_newGene_63714	NA	NA	NA
Bos_taurus_newGene_63715	NA	NA	NA
Bos_taurus_newGene_63716	0.2527927	0.4837274	0.3153993
Bos_taurus_newGene_63727	NA	NA	NA
Bos_taurus_newGene_63762	NA	NA	NA

Bos_taurus_newGene_63763	NA	NA	NA
Bos_taurus_newGene_63764	-0.201363	0.5727371	0.2420447
Bos_taurus_newGene_63765	NA	NA	NA
Bos_taurus_newGene_63766	-0.077285	0.8846839	0.0532119
Bos_taurus_newGene_63767	1.3817303	0.0149554	1.8252016
Bos_taurus_newGene_63769	0.9418498	0.4098132	0.387414
Bos_taurus_newGene_63878	NA	NA	NA
Bos_taurus_newGene_63906	NA	NA	NA
Bos_taurus_newGene_64110	NA	NA	NA
Bos_taurus_newGene_64111	NA	NA	NA
Bos_taurus_newGene_64116	NA	NA	NA
Bos_taurus_newGene_64120	NA	NA	NA
Bos_taurus_newGene_64126	NA	NA	NA
Bos_taurus_newGene_64128	NA	NA	NA
Bos_taurus_newGene_64139	NA	NA	NA
Bos_taurus_newGene_64235	NA	NA	NA
Bos_taurus_newGene_64276	NA	NA	NA
Bos_taurus_newGene_64289	NA	NA	NA
Bos_taurus_newGene_64313	NA	NA	NA
Bos_taurus_newGene_64314	NA	NA	NA
Bos_taurus_newGene_64317	NA	NA	NA
Bos_taurus_newGene_64484	NA	NA	NA
Bos_taurus_newGene_64622	NA	NA	NA
Bos_taurus_newGene_64718	NA	NA	NA
Bos_taurus_newGene_64811	0.1770734	0.7270696	0.138424
Bos_taurus_newGene_64824	NA	NA	NA
Bos_taurus_newGene_64838	NA	NA	NA
Bos_taurus_newGene_64889	NA	NA	NA
Bos_taurus_newGene_64892	NA	NA	NA
Bos_taurus_newGene_64916	NA	NA	NA
Bos_taurus_newGene_64953	0.1162449	0.697733	0.1563108
Bos_taurus_newGene_64989	NA	NA	NA
Bos_taurus_newGene_65007	NA	NA	NA
Bos_taurus_newGene_65049	NA	NA	NA
Bos_taurus_newGene_65054	NA	NA	NA
Bos_taurus_newGene_65062	NA	NA	NA
Bos_taurus_newGene_65134	NA	NA	NA
Bos_taurus_newGene_65247	NA	NA	NA
Bos_taurus_newGene_65332	NA	NA	NA
Bos_taurus_newGene_65340	-0.001786	0.9979653	0.0008846
Bos_taurus_newGene_65464	NA	NA	NA
Bos_taurus_newGene_65500	NA	NA	NA
Bos_taurus_newGene_65529	NA	NA	NA
Bos_taurus_newGene_65533	NA	NA	NA
Bos_taurus_newGene_65549	NA	NA	NA
Bos_taurus_newGene_65554	NA	NA	NA
Bos_taurus_newGene_65618	0.5125257	0.2993386	0.5238373
Bos_taurus_newGene_65653	NA	NA	NA
Bos_taurus_newGene_65725	NA	NA	NA
Bos_taurus_newGene_65838	NA	NA	NA
Bos_taurus_newGene_65880	NA	NA	NA
Bos_taurus_newGene_65951	NA	NA	NA
Bos_taurus_newGene_66033	0.5047976	0.2406588	0.6185983
Bos_taurus_newGene_66034	NA	NA	NA
Bos_taurus_newGene_66049	NA	NA	NA
Bos_taurus_newGene_66051	-0.110209	0.8675283	0.0617164
Bos_taurus_newGene_66068	NA	NA	NA
Bos_taurus_newGene_66128	NA	NA	NA

Bos_taurus_newGene_66328	NA	NA	NA
Bos_taurus_newGene_66381	NA	NA	NA
Bos_taurus_newGene_66455	NA	NA	NA
Bos_taurus_newGene_66457	NA	NA	NA
Bos_taurus_newGene_66460	NA	NA	NA
Bos_taurus_newGene_66474	NA	NA	NA
Bos_taurus_newGene_66514	NA	NA	NA
Bos_taurus_newGene_66569	-0.427076	0.2752324	0.5603004
Bos_taurus_newGene_66660	NA	NA	NA
Bos_taurus_newGene_66663	NA	NA	NA
Bos_taurus_newGene_66665	NA	NA	NA
Bos_taurus_newGene_66666	0.3052244	0.4950164	0.3053805
Bos_taurus_newGene_66668	NA	NA	NA
Bos_taurus_newGene_66669	-0.040686	0.93593	0.0287566
Bos_taurus_newGene_66680	-0.52891	0.2429029	0.6145674
Bos_taurus_newGene_66684	-0.346804	0.5028689	0.2985453
Bos_taurus_newGene_66710	NA	NA	NA
Bos_taurus_newGene_66711	NA	NA	NA
Bos_taurus_newGene_66746	NA	NA	NA
Bos_taurus_newGene_66771	NA	NA	NA
Bos_taurus_newGene_66819	0.2364682	0.5538372	0.2566179
Bos_taurus_newGene_66824	-0.35796	0.4768219	0.3216438
Bos_taurus_newGene_66887	NA	NA	NA
Bos_taurus_newGene_66893	NA	NA	NA
Bos_taurus_newGene_66951	NA	NA	NA
Bos_taurus_newGene_66960	-0.422498	0.2638461	0.5786493
Bos_taurus_newGene_66967	NA	NA	NA
Bos_taurus_newGene_66982	NA	NA	NA
Bos_taurus_newGene_67041	NA	NA	NA
Bos_taurus_newGene_67060	-0.462818	0.2519556	0.5986761
Bos_taurus_newGene_67204	NA	NA	NA
Bos_taurus_newGene_67223	NA	NA	NA
Bos_taurus_newGene_67235	NA	NA	NA
Bos_taurus_newGene_67311	NA	NA	NA
Bos_taurus_newGene_67345	NA	NA	NA
Bos_taurus_newGene_67350	0.0255369	0.9654411	0.0152742
Bos_taurus_newGene_67351	NA	NA	NA
Bos_taurus_newGene_67400	NA	NA	NA
Bos_taurus_newGene_67449	NA	NA	NA
Bos_taurus_newGene_67486	NA	NA	NA
Bos_taurus_newGene_67568	-1.301573	0.4512501	0.3455827
Bos_taurus_newGene_67578	NA	NA	NA
Bos_taurus_newGene_67635	NA	NA	NA
Bos_taurus_newGene_67702	NA	NA	NA
Bos_taurus_newGene_67713	-0.762502	0.1230252	0.910006
Bos_taurus_newGene_67736	NA	NA	NA
Bos_taurus_newGene_67823	NA	NA	NA
Bos_taurus_newGene_67838	NA	NA	NA
Bos_taurus_newGene_67862	NA	NA	NA
Bos_taurus_newGene_67864	NA	NA	NA
Bos_taurus_newGene_67867	NA	NA	NA
Bos_taurus_newGene_67895	NA	NA	NA
Bos_taurus_newGene_67929	0.2804921	0.596429	0.2244413
Bos_taurus_newGene_68004	NA	NA	NA
Bos_taurus_newGene_68040	NA	NA	NA
Bos_taurus_newGene_68201	NA	NA	NA
Bos_taurus_newGene_68203	NA	NA	NA
Bos_taurus_newGene_68226	NA	NA	NA

Bos_taurus_newGene_68255	0.4931523	0.2751367	0.5604515
Bos_taurus_newGene_68276	NA	NA	NA
Bos_taurus_newGene_68282	NA	NA	NA
Bos_taurus_newGene_68285	NA	NA	NA
Bos_taurus_newGene_68300	NA	NA	NA
Bos_taurus_newGene_68318	NA	NA	NA
Bos_taurus_newGene_68370	NA	NA	NA
Bos_taurus_newGene_68381	NA	NA	NA
Bos_taurus_newGene_68383	-0.229594	0.6779968	0.1687724
Bos_taurus_newGene_68389	NA	NA	NA
Bos_taurus_newGene_68391	NA	NA	NA
Bos_taurus_newGene_68518	NA	NA	NA
Bos_taurus_newGene_68528	1.1219947	0.1072554	0.9695808
Bos_taurus_newGene_68530	NA	NA	NA
Bos_taurus_newGene_68531	1.0288648	0.0124403	1.905169
Bos_taurus_newGene_68549	NA	NA	NA
Bos_taurus_newGene_68550	NA	NA	NA
Bos_taurus_newGene_68553	NA	NA	NA
Bos_taurus_newGene_68649	-0.579067	0.4657439	0.3318528
Bos_taurus_newGene_68691	0.5796196	0.202232	0.6941502
Bos_taurus_newGene_68733	NA	NA	NA
Bos_taurus_newGene_68735	NA	NA	NA
Bos_taurus_newGene_68747	NA	NA	NA
Bos_taurus_newGene_68784	NA	NA	NA
Bos_taurus_newGene_68788	1.2236785	0.0001502	3.8233561
Bos_taurus_newGene_68790	NA	NA	NA
Bos_taurus_newGene_68792	-0.372233	0.4308248	0.3656993
Bos_taurus_newGene_68794	NA	NA	NA
Bos_taurus_newGene_68795	NA	NA	NA
Bos_taurus_newGene_68796	NA	NA	NA
Bos_taurus_newGene_68798	NA	NA	NA
Bos_taurus_newGene_68803	NA	NA	NA
Bos_taurus_newGene_68804	NA	NA	NA
Bos_taurus_newGene_68805	NA	NA	NA
Bos_taurus_newGene_68806	NA	NA	NA
Bos_taurus_newGene_68807	NA	NA	NA
Bos_taurus_newGene_68810	NA	NA	NA
Bos_taurus_newGene_68811	NA	NA	NA
Bos_taurus_newGene_68819	NA	NA	NA
Bos_taurus_newGene_68829	NA	NA	NA
Bos_taurus_newGene_68872	NA	NA	NA
Bos_taurus_newGene_68983	-0.877075	0.2186138	0.6603225
Bos_taurus_newGene_69002	NA	NA	NA
Bos_taurus_newGene_69021	NA	NA	NA
Bos_taurus_newGene_69035	NA	NA	NA
Bos_taurus_newGene_69069	NA	NA	NA
Bos_taurus_newGene_69091	NA	NA	NA
Bos_taurus_newGene_69158	-0.1323	0.7087417	0.149512
Bos_taurus_newGene_69215	NA	NA	NA
Bos_taurus_newGene_69223	-0.197868	0.5823808	0.234793
Bos_taurus_newGene_69285	NA	NA	NA
Bos_taurus_newGene_69301	NA	NA	NA
Bos_taurus_newGene_69306	NA	NA	NA
Bos_taurus_newGene_69307	NA	NA	NA
Bos_taurus_newGene_69308	NA	NA	NA
Bos_taurus_newGene_69311	NA	NA	NA
Bos_taurus_newGene_69312	NA	NA	NA
Bos_taurus_newGene_69313	NA	NA	NA

Bos_taurus_newGene_69314	NA	NA	NA
Bos_taurus_newGene_69315	NA	NA	NA
Bos_taurus_newGene_69316	NA	NA	NA
Bos_taurus_newGene_69317	NA	NA	NA
Bos_taurus_newGene_69318	NA	NA	NA
Bos_taurus_newGene_69319	NA	NA	NA
Bos_taurus_newGene_69320	NA	NA	NA
Bos_taurus_newGene_69321	NA	NA	NA
Bos_taurus_newGene_69322	NA	NA	NA
Bos_taurus_newGene_69323	NA	NA	NA
Bos_taurus_newGene_69324	NA	NA	NA
Bos_taurus_newGene_69325	NA	NA	NA
Bos_taurus_newGene_69344	NA	NA	NA
Bos_taurus_newGene_69345	NA	NA	NA
Bos_taurus_newGene_69367	NA	NA	NA
Bos_taurus_newGene_69369	-0.007454	0.984806	0.0066493
Bos_taurus_newGene_69370	NA	NA	NA
Bos_taurus_newGene_69387	NA	NA	NA
Bos_taurus_newGene_69393	-0.262083	0.3769967	0.4236624
Bos_taurus_newGene_69402	NA	NA	NA
Bos_taurus_newGene_69407	NA	NA	NA
Bos_taurus_newGene_69417	0.6699407	0.2063126	0.6854743
Bos_taurus_newGene_69442	NA	NA	NA
Bos_taurus_newGene_69445	NA	NA	NA
Bos_taurus_newGene_69508	NA	NA	NA
Bos_taurus_newGene_69521	NA	NA	NA
Bos_taurus_newGene_69523	NA	NA	NA
Bos_taurus_newGene_69535	0.7736946	0.0665502	1.1768508
Bos_taurus_newGene_69568	NA	NA	NA
Bos_taurus_newGene_69619	0.9007905	0.0917169	1.0375506
Bos_taurus_newGene_69631	-0.058086	0.8169636	0.0877973
Bos_taurus_newGene_69665	NA	NA	NA
Bos_taurus_newGene_69689	NA	NA	NA
Bos_taurus_newGene_69696	NA	NA	NA
Bos_taurus_newGene_69698	NA	NA	NA
Bos_taurus_newGene_69700	-1.738625	0.0008386	3.0764198
Bos_taurus_newGene_69778	0.5021658	0.3588742	0.4450577
Bos_taurus_newGene_69815	NA	NA	NA
Bos_taurus_newGene_69816	-0.091265	0.7994753	0.0971949
Bos_taurus_newGene_69854	NA	NA	NA
Bos_taurus_newGene_69856	NA	NA	NA
Bos_taurus_newGene_69872	NA	NA	NA
Bos_taurus_newGene_69873	NA	NA	NA
Bos_taurus_newGene_69894	NA	NA	NA
Bos_taurus_newGene_69954	NA	NA	NA
Bos_taurus_newGene_69978	NA	NA	NA
Bos_taurus_newGene_70030	0.6246578	0.2151627	0.667233
Bos_taurus_newGene_70041	NA	NA	NA
Bos_taurus_newGene_70073	NA	NA	NA
Bos_taurus_newGene_70076	NA	NA	NA
Bos_taurus_newGene_70095	NA	NA	NA
Bos_taurus_newGene_70101	NA	NA	NA
Bos_taurus_newGene_70103	0.4790944	0.2981713	0.5255341
Bos_taurus_newGene_70124	NA	NA	NA
Bos_taurus_newGene_70141	NA	NA	NA
Bos_taurus_newGene_70199	NA	NA	NA
Bos_taurus_newGene_70208	0.0885133	0.7643721	0.1166952
Bos_taurus_newGene_70311	NA	NA	NA

Bos_taurus_newGene_70319	-0.369237	0.3489331	0.4572578
Bos_taurus_newGene_70366	NA	NA	NA
Bos_taurus_newGene_70370	NA	NA	NA
Bos_taurus_newGene_70419	NA	NA	NA
Bos_taurus_newGene_70560	NA	NA	NA
Bos_taurus_newGene_70568	-1.547156	0.0371404	1.4301538
Bos_taurus_newGene_70572	NA	NA	NA
Bos_taurus_newGene_70647	-1.244634	0.0022239	2.6528781
Bos_taurus_newGene_70658	NA	NA	NA
Bos_taurus_newGene_70871	NA	NA	NA
Bos_taurus_newGene_70972	NA	NA	NA
Bos_taurus_newGene_71247	NA	NA	NA
Bos_taurus_newGene_71262	0.2845998	0.5555426	0.2552826
Bos_taurus_newGene_71265	NA	NA	NA
Bos_taurus_newGene_71434	NA	NA	NA
Bos_taurus_newGene_71448	NA	NA	NA
Bos_taurus_newGene_71451	NA	NA	NA
Bos_taurus_newGene_71452	NA	NA	NA
Bos_taurus_newGene_71459	NA	NA	NA
Bos_taurus_newGene_71466	1.035171	0.3652544	0.4374046
Bos_taurus_newGene_71490	NA	NA	NA
Bos_taurus_newGene_71496	NA	NA	NA
Bos_taurus_newGene_71512	NA	NA	NA
Bos_taurus_newGene_71593	NA	NA	NA
Bos_taurus_newGene_71746	NA	NA	NA
Bos_taurus_newGene_71776	NA	NA	NA
Bos_taurus_newGene_71783	NA	NA	NA
Bos_taurus_newGene_71853	-1.40925	0.0073592	2.1331674
Bos_taurus_newGene_71868	-0.97356	0.2489821	0.6038319
Bos_taurus_newGene_71873	NA	NA	NA
Bos_taurus_newGene_71921	NA	NA	NA
Bos_taurus_newGene_71935	NA	NA	NA
Bos_taurus_newGene_71970	0.4357322	0.4839266	0.3152205
Bos_taurus_newGene_72015	1.060925	0.0067961	2.1677386
Bos_taurus_newGene_72019	NA	NA	NA
Bos_taurus_newGene_72020	NA	NA	NA
Bos_taurus_newGene_72035	NA	NA	NA
Bos_taurus_newGene_72039	-0.036724	0.9479411	0.0232187
Bos_taurus_newGene_72040	0.167777	0.7041264	0.1523494
Bos_taurus_newGene_72044	NA	NA	NA
Bos_taurus_newGene_72068	NA	NA	NA
Bos_taurus_newGene_72261	NA	NA	NA
Bos_taurus_newGene_72278	-0.334432	0.470548	0.3273961
Bos_taurus_newGene_72292	NA	NA	NA
Bos_taurus_newGene_72296	NA	NA	NA
Bos_taurus_newGene_72301	NA	NA	NA
Bos_taurus_newGene_72316	NA	NA	NA
Bos_taurus_newGene_72319	NA	NA	NA
Bos_taurus_newGene_72320	-0.009672	0.9742308	0.0113382
Bos_taurus_newGene_72324	-0.266618	0.3139617	0.5031234
Bos_taurus_newGene_72325	NA	NA	NA
Bos_taurus_newGene_72385	NA	NA	NA
Bos_taurus_newGene_72404	NA	NA	NA
Bos_taurus_newGene_72424	NA	NA	NA
Bos_taurus_newGene_72426	NA	NA	NA
Bos_taurus_newGene_72482	NA	NA	NA
Bos_taurus_newGene_72534	NA	NA	NA
Bos_taurus_newGene_72538	NA	NA	NA

Bos_taurus_newGene_72556	NA	NA	NA
Bos_taurus_newGene_72618	NA	NA	NA
Bos_taurus_newGene_72643	-0.076773	0.7976673	0.0981782
Bos_taurus_newGene_72644	NA	NA	NA
Bos_taurus_newGene_72764	NA	NA	NA
Bos_taurus_newGene_72797	NA	NA	NA
Bos_taurus_newGene_72808	NA	NA	NA
Bos_taurus_newGene_72812	NA	NA	NA
Bos_taurus_newGene_72815	NA	NA	NA
Bos_taurus_newGene_72848	NA	NA	NA
Bos_taurus_newGene_72849	NA	NA	NA
Bos_taurus_newGene_72858	NA	NA	NA
Bos_taurus_newGene_72862	NA	NA	NA
Bos_taurus_newGene_72877	NA	NA	NA
Bos_taurus_newGene_72906	0.9452344	0.1303946	0.8847403
Bos_taurus_newGene_72907	0.8396723	0.1375967	0.8613921
Bos_taurus_newGene_72908	0.627805	0.2203268	0.6569326
Bos_taurus_newGene_72918	NA	NA	NA
Bos_taurus_newGene_73033	NA	NA	NA
Bos_taurus_newGene_73051	NA	NA	NA
Bos_taurus_newGene_73056	NA	NA	NA
Bos_taurus_newGene_73083	NA	NA	NA
Bos_taurus_newGene_73100	0.1525929	0.7288761	0.1373463
Bos_taurus_newGene_73102	NA	NA	NA
Bos_taurus_newGene_73138	-0.457627	0.3051097	0.515544
Bos_taurus_newGene_73152	-0.629359	0.1644753	0.7838993
Bos_taurus_newGene_73169	NA	NA	NA
Bos_taurus_newGene_73190	NA	NA	NA
Bos_taurus_newGene_73291	0.09633	0.8449178	0.0731855
Bos_taurus_newGene_73329	NA	NA	NA
Bos_taurus_newGene_73440	NA	NA	NA
Bos_taurus_newGene_73454	NA	NA	NA
Bos_taurus_newGene_73665	NA	NA	NA
Bos_taurus_newGene_73706	NA	NA	NA
Bos_taurus_newGene_73753	0.4110552	0.3399847	0.4685406
Bos_taurus_newGene_73754	NA	NA	NA
Bos_taurus_newGene_73773	NA	NA	NA
Bos_taurus_newGene_73826	NA	NA	NA
Bos_taurus_newGene_73831	NA	NA	NA
Bos_taurus_newGene_73837	0.6464497	0.0218914	1.6597259
Bos_taurus_newGene_73882	0.2902151	0.5895511	0.2294785
Bos_taurus_newGene_73883	0.0957503	0.9339174	0.0296915
Bos_taurus_newGene_73962	-0.978157	0.0160775	1.7937806
Bos_taurus_newGene_74024	NA	NA	NA
Bos_taurus_newGene_74216	NA	NA	NA
Bos_taurus_newGene_74219	1.2125957	0.1517746	0.818801
Bos_taurus_newGene_74221	NA	NA	NA
Bos_taurus_newGene_74236	NA	NA	NA
Bos_taurus_newGene_74316	NA	NA	NA
Bos_taurus_newGene_74543	NA	NA	NA
Bos_taurus_newGene_74621	NA	NA	NA
Bos_taurus_newGene_74644	NA	NA	NA
Bos_taurus_newGene_74646	NA	NA	NA
Bos_taurus_newGene_74647	NA	NA	NA
Bos_taurus_newGene_74648	NA	NA	NA
Bos_taurus_newGene_74649	NA	NA	NA
Bos_taurus_newGene_74650	NA	NA	NA
Bos_taurus_newGene_74653	NA	NA	NA

Bos_taurus_newGene_74659	NA	NA	NA
Bos_taurus_newGene_74661	NA	NA	NA
Bos_taurus_newGene_74662	NA	NA	NA
Bos_taurus_newGene_74663	NA	NA	NA
Bos_taurus_newGene_74688	NA	NA	NA
Bos_taurus_newGene_74828	NA	NA	NA
Bos_taurus_newGene_74882	-0.158101	0.7056882	0.1513872
Bos_taurus_newGene_74950	NA	NA	NA
Bos_taurus_newGene_74985	NA	NA	NA
Bos_taurus_newGene_74987	NA	NA	NA
Bos_taurus_newGene_75073	NA	NA	NA
Bos_taurus_newGene_75349	NA	NA	NA
Bos_taurus_newGene_75376	NA	NA	NA
Bos_taurus_newGene_75439	-0.42152	0.2890677	0.5390005
Bos_taurus_newGene_75525	NA	NA	NA
Bos_taurus_newGene_75542	NA	NA	NA
Bos_taurus_newGene_75620	NA	NA	NA
Bos_taurus_newGene_75662	NA	NA	NA
Bos_taurus_newGene_75663	NA	NA	NA
Bos_taurus_newGene_75676	NA	NA	NA
Bos_taurus_newGene_75732	NA	NA	NA
Bos_taurus_newGene_75758	NA	NA	NA
Bos_taurus_newGene_75808	NA	NA	NA
Bos_taurus_newGene_75810	-0.447737	0.2303636	0.6375861
Bos_taurus_newGene_75944	NA	NA	NA
Bos_taurus_newGene_75981	NA	NA	NA
Bos_taurus_newGene_76036	NA	NA	NA
Bos_taurus_newGene_76039	NA	NA	NA
Bos_taurus_newGene_76048	-0.694889	0.2591459	0.5864557
Bos_taurus_newGene_76080	NA	NA	NA
Bos_taurus_newGene_76086	NA	NA	NA
Bos_taurus_newGene_76168	NA	NA	NA
Bos_taurus_newGene_76207	-0.186486	0.5668792	0.2465095
Bos_taurus_newGene_76209	NA	NA	NA
Bos_taurus_newGene_76214	-0.226356	0.683413	0.1653168
Bos_taurus_newGene_76218	NA	NA	NA
Bos_taurus_newGene_76219	NA	NA	NA
Bos_taurus_newGene_76221	NA	NA	NA
Bos_taurus_newGene_76230	0.8439095	0.2401626	0.6194946
Bos_taurus_newGene_76232	-0.629271	0.2002232	0.6984857
Bos_taurus_newGene_76334	1.5473421	0.0614	1.2118318
Bos_taurus_newGene_76364	-3.393428	9.16E-07	6.037987
Bos_taurus_newGene_76399	NA	NA	NA
Bos_taurus_newGene_76526	NA	NA	NA
Bos_taurus_newGene_76601	NA	NA	NA
Bos_taurus_newGene_76616	NA	NA	NA
Bos_taurus_newGene_76675	NA	NA	NA
Bos_taurus_newGene_76697	-1.031959	0.0401199	1.3966407
Bos_taurus_newGene_76771	NA	NA	NA
Bos_taurus_newGene_76905	NA	NA	NA
Bos_taurus_newGene_76938	NA	NA	NA
Bos_taurus_newGene_77031	NA	NA	NA
Bos_taurus_newGene_77069	NA	NA	NA
Bos_taurus_newGene_77072	NA	NA	NA
Bos_taurus_newGene_77166	NA	NA	NA
Bos_taurus_newGene_77175	NA	NA	NA
Bos_taurus_newGene_77209	NA	NA	NA
Bos_taurus_newGene_77247	NA	NA	NA

Bos_taurus_newGene_77254	NA	NA	NA
Bos_taurus_newGene_77322	NA	NA	NA
Bos_taurus_newGene_77352	NA	NA	NA
Bos_taurus_newGene_77358	NA	NA	NA
Bos_taurus_newGene_77363	NA	NA	NA
Bos_taurus_newGene_77367	NA	NA	NA
Bos_taurus_newGene_77371	NA	NA	NA
Bos_taurus_newGene_77373	NA	NA	NA
Bos_taurus_newGene_77378	NA	NA	NA
Bos_taurus_newGene_77434	NA	NA	NA
Bos_taurus_newGene_77477	NA	NA	NA
Bos_taurus_newGene_77585	NA	NA	NA
Bos_taurus_newGene_77623	NA	NA	NA
Bos_taurus_newGene_77664	NA	NA	NA
Bos_taurus_newGene_77679	NA	NA	NA
Bos_taurus_newGene_77763	NA	NA	NA
Bos_taurus_newGene_77834	NA	NA	NA
Bos_taurus_newGene_77843	NA	NA	NA
Bos_taurus_newGene_77852	NA	NA	NA
Bos_taurus_newGene_77854	NA	NA	NA
Bos_taurus_newGene_77859	-0.085662	0.8677346	0.0616131
Bos_taurus_newGene_77862	NA	NA	NA
Bos_taurus_newGene_77911	NA	NA	NA
Bos_taurus_newGene_77949	NA	NA	NA
Bos_taurus_newGene_78044	NA	NA	NA
Bos_taurus_newGene_78073	NA	NA	NA
Bos_taurus_newGene_78080	NA	NA	NA
Bos_taurus_newGene_78098	NA	NA	NA
Bos_taurus_newGene_78159	NA	NA	NA
Bos_taurus_newGene_78160	NA	NA	NA
Bos_taurus_newGene_78189	NA	NA	NA
Bos_taurus_newGene_78214	0.2996375	0.5066406	0.2953
Bos_taurus_newGene_78275	-0.39761	0.5611924	0.2508882
Bos_taurus_newGene_78279	NA	NA	NA
Bos_taurus_newGene_78281	NA	NA	NA
Bos_taurus_newGene_78287	NA	NA	NA
Bos_taurus_newGene_78337	NA	NA	NA
Bos_taurus_newGene_78348	0.5130483	0.2304723	0.6373812
Bos_taurus_newGene_78376	NA	NA	NA
Bos_taurus_newGene_78403	NA	NA	NA
Bos_taurus_newGene_78410	NA	NA	NA
Bos_taurus_newGene_78444	NA	NA	NA
Bos_taurus_newGene_78468	-0.623186	0.1191427	0.9239326
Bos_taurus_newGene_78470	NA	NA	NA
Bos_taurus_newGene_78517	NA	NA	NA
Bos_taurus_newGene_78543	NA	NA	NA
Bos_taurus_newGene_78556	NA	NA	NA
Bos_taurus_newGene_78557	NA	NA	NA
Bos_taurus_newGene_78558	NA	NA	NA
Bos_taurus_newGene_78559	NA	NA	NA
Bos_taurus_newGene_78560	NA	NA	NA
Bos_taurus_newGene_78561	NA	NA	NA
Bos_taurus_newGene_78565	NA	NA	NA
Bos_taurus_newGene_78566	NA	NA	NA
Bos_taurus_newGene_78567	NA	NA	NA
Bos_taurus_newGene_78568	NA	NA	NA
Bos_taurus_newGene_78736	NA	NA	NA
Bos_taurus_newGene_78739	NA	NA	NA

Bos_taurus_newGene_78743	NA	NA	NA
Bos_taurus_newGene_78770	NA	NA	NA
Bos_taurus_newGene_78796	NA	NA	NA
Bos_taurus_newGene_78807	NA	NA	NA
Bos_taurus_newGene_78818	NA	NA	NA
Bos_taurus_newGene_78961	NA	NA	NA
Bos_taurus_newGene_78989	NA	NA	NA
Bos_taurus_newGene_78991	NA	NA	NA
Bos_taurus_newGene_79019	NA	NA	NA
Bos_taurus_newGene_79041	NA	NA	NA
Bos_taurus_newGene_79054	NA	NA	NA
Bos_taurus_newGene_79055	NA	NA	NA
Bos_taurus_newGene_79081	NA	NA	NA
Bos_taurus_newGene_79157	NA	NA	NA
Bos_taurus_newGene_79159	NA	NA	NA
Bos_taurus_newGene_79163	NA	NA	NA
Bos_taurus_newGene_79165	NA	NA	NA
Bos_taurus_newGene_79166	0.0499147	0.9333502	0.0299554
Bos_taurus_newGene_79167	NA	NA	NA
Bos_taurus_newGene_79168	NA	NA	NA
Bos_taurus_newGene_79169	NA	NA	NA
Bos_taurus_newGene_79171	NA	NA	NA
Bos_taurus_newGene_79172	NA	NA	NA
Bos_taurus_newGene_79173	NA	NA	NA
Bos_taurus_newGene_79179	NA	NA	NA
Bos_taurus_newGene_79374	NA	NA	NA
Bos_taurus_newGene_79383	NA	NA	NA
Bos_taurus_newGene_79393	NA	NA	NA
Bos_taurus_newGene_79394	-0.819816	0.0775408	1.1104699
Bos_taurus_newGene_79395	-1.222809	0.0329303	1.4824041
Bos_taurus_newGene_79398	NA	NA	NA
Bos_taurus_newGene_79507	NA	NA	NA
Bos_taurus_newGene_79514	0.6204761	0.106466	0.9727892
Bos_taurus_newGene_79519	1.3640917	0.0097947	2.0090076
Bos_taurus_newGene_79520	1.1682156	0.0245493	1.609961
Bos_taurus_newGene_79565	NA	NA	NA
Bos_taurus_newGene_79597	NA	NA	NA
Bos_taurus_newGene_79615	NA	NA	NA
Bos_taurus_newGene_79750	NA	NA	NA
Bos_taurus_newGene_79778	NA	NA	NA
Bos_taurus_newGene_79802	NA	NA	NA
Bos_taurus_newGene_79804	NA	NA	NA
Bos_taurus_newGene_79808	NA	NA	NA
Bos_taurus_newGene_79809	NA	NA	NA
Bos_taurus_newGene_79813	NA	NA	NA
Bos_taurus_newGene_79814	NA	NA	NA
Bos_taurus_newGene_79817	NA	NA	NA
Bos_taurus_newGene_79834	NA	NA	NA
Bos_taurus_newGene_79897	-0.163296	0.6557665	0.1832508
Bos_taurus_newGene_79902	-0.669744	0.3304834	0.4808504
Bos_taurus_newGene_79906	-0.142871	0.7192881	0.1430971
Bos_taurus_newGene_79909	-0.214547	0.5461298	0.2627041
Bos_taurus_newGene_79910	-0.345126	0.4157895	0.3811265
Bos_taurus_newGene_79941	-0.606736	0.0948509	1.0229588
Bos_taurus_newGene_79943	NA	NA	NA
Bos_taurus_newGene_79944	NA	NA	NA
Bos_taurus_newGene_79946	NA	NA	NA
Bos_taurus_newGene_79966	NA	NA	NA

Bos_taurus_newGene_79974	NA	NA	NA
Bos_taurus_newGene_79979	-0.068318	0.9087077	0.0415758
Bos_taurus_newGene_79989	NA	NA	NA
Bos_taurus_newGene_80029	NA	NA	NA
Bos_taurus_newGene_80040	0.6569781	0.2195573	0.6584522
Bos_taurus_newGene_80060	NA	NA	NA
Bos_taurus_newGene_80061	NA	NA	NA
Bos_taurus_newGene_80065	NA	NA	NA
Bos_taurus_newGene_80073	NA	NA	NA
Bos_taurus_newGene_80106	NA	NA	NA
Bos_taurus_newGene_80134	NA	NA	NA
Bos_taurus_newGene_80156	-0.382769	0.3618879	0.4414259
Bos_taurus_newGene_80158	-0.533734	0.3091824	0.5097852
Bos_taurus_newGene_80162	0.0590363	0.9410162	0.0264029
Bos_taurus_newGene_80163	0.937563	0.1624636	0.789244
Bos_taurus_newGene_80173	0.5908926	0.3194967	0.4955336
Bos_taurus_newGene_80239	NA	NA	NA
Bos_taurus_newGene_80272	NA	NA	NA
Bos_taurus_newGene_80417	NA	NA	NA
Bos_taurus_newGene_80421	NA	NA	NA
Bos_taurus_newGene_80481	-0.488794	0.269072	0.5701314
Bos_taurus_newGene_80497	-0.735431	0.191525	0.7177745
Bos_taurus_newGene_80507	-0.686237	0.1860449	0.7303821
Bos_taurus_newGene_80515	-0.934758	0.1707709	0.7675861
Bos_taurus_newGene_80535	NA	NA	NA
Bos_taurus_newGene_80684	NA	NA	NA
Bos_taurus_newGene_80743	NA	NA	NA
Bos_taurus_newGene_80800	0.6604275	0.5352384	0.2714527
Bos_taurus_newGene_80823	NA	NA	NA
Bos_taurus_newGene_80826	NA	NA	NA
Bos_taurus_newGene_80882	NA	NA	NA
Bos_taurus_newGene_80976	NA	NA	NA
Bos_taurus_newGene_81030	NA	NA	NA
Bos_taurus_newGene_81032	0.2400806	0.6643303	0.1776159
Bos_taurus_newGene_81058	NA	NA	NA
Bos_taurus_newGene_81078	NA	NA	NA
Bos_taurus_newGene_81099	-0.086312	0.8631317	0.0639229
Bos_taurus_newGene_81106	NA	NA	NA
Bos_taurus_newGene_81107	-0.128817	0.6322325	0.1991232
Bos_taurus_newGene_81108	NA	NA	NA
Bos_taurus_newGene_81115	-0.196828	0.5860905	0.2320353
Bos_taurus_newGene_81116	NA	NA	NA
Bos_taurus_newGene_81119	-0.589759	0.1769653	0.7521119
Bos_taurus_newGene_81123	NA	NA	NA
Bos_taurus_newGene_81191	NA	NA	NA
Bos_taurus_newGene_81201	-0.970129	0.2294495	0.6393128
Bos_taurus_newGene_81266	0.6287686	0.1882984	0.7251534
Bos_taurus_newGene_81279	NA	NA	NA
Bos_taurus_newGene_81280	NA	NA	NA
Bos_taurus_newGene_81281	NA	NA	NA
Bos_taurus_newGene_81282	NA	NA	NA
Bos_taurus_newGene_81283	NA	NA	NA
Bos_taurus_newGene_81284	NA	NA	NA
Bos_taurus_newGene_81285	NA	NA	NA
Bos_taurus_newGene_81286	NA	NA	NA
Bos_taurus_newGene_81287	-0.264367	0.6570821	0.1823804
Bos_taurus_newGene_81289	NA	NA	NA
Bos_taurus_newGene_81290	NA	NA	NA

Bos_taurus_newGene_81293	NA	NA	NA
Bos_taurus_newGene_81294	NA	NA	NA
Bos_taurus_newGene_81295	NA	NA	NA
Bos_taurus_newGene_81296	NA	NA	NA
Bos_taurus_newGene_81301	NA	NA	NA
Bos_taurus_newGene_81303	NA	NA	NA
Bos_taurus_newGene_81304	NA	NA	NA
Bos_taurus_newGene_81305	NA	NA	NA
Bos_taurus_newGene_81307	NA	NA	NA
Bos_taurus_newGene_81309	NA	NA	NA
Bos_taurus_newGene_81310	NA	NA	NA
Bos_taurus_newGene_81312	NA	NA	NA
Bos_taurus_newGene_81325	0.0935574	0.8366726	0.0774445
Bos_taurus_newGene_81326	NA	NA	NA
Bos_taurus_newGene_81358	NA	NA	NA
Bos_taurus_newGene_81381	NA	NA	NA
Bos_taurus_newGene_81387	NA	NA	NA
Bos_taurus_newGene_81388	NA	NA	NA
Bos_taurus_newGene_81410	-2.094314	0.0019631	2.7070558
Bos_taurus_newGene_81427	NA	NA	NA
Bos_taurus_newGene_81430	NA	NA	NA
Bos_taurus_newGene_81460	NA	NA	NA
Bos_taurus_newGene_81488	NA	NA	NA
Bos_taurus_newGene_81537	NA	NA	NA
Bos_taurus_newGene_81584	0.5157636	0.4161298	0.3807712
Bos_taurus_newGene_81608	NA	NA	NA
Bos_taurus_newGene_81653	0.2712661	0.4422288	0.3543529
Bos_taurus_newGene_81689	NA	NA	NA
Bos_taurus_newGene_81698	0.4696032	0.3997351	0.3982278
Bos_taurus_newGene_81700	NA	NA	NA
Bos_taurus_newGene_81704	NA	NA	NA
Bos_taurus_newGene_81705	NA	NA	NA
Bos_taurus_newGene_81730	NA	NA	NA
Bos_taurus_newGene_81759	NA	NA	NA
Bos_taurus_newGene_81768	NA	NA	NA
Bos_taurus_newGene_81818	NA	NA	NA
Bos_taurus_newGene_81822	NA	NA	NA
Bos_taurus_newGene_81843	NA	NA	NA
Bos_taurus_newGene_81988	NA	NA	NA
Bos_taurus_newGene_82096	NA	NA	NA
Bos_taurus_newGene_82116	NA	NA	NA
Bos_taurus_newGene_82188	-0.160397	0.6072188	0.2166548
Bos_taurus_newGene_82191	-0.12533	0.7249151	0.1397129
Bos_taurus_newGene_82333	NA	NA	NA
Bos_taurus_newGene_82367	NA	NA	NA
Bos_taurus_newGene_82375	NA	NA	NA
Bos_taurus_newGene_82382	0.1998622	0.6708669	0.1733636
Bos_taurus_newGene_82383	0.283326	0.6122836	0.2130474
Bos_taurus_newGene_82412	NA	NA	NA
Bos_taurus_newGene_82525	NA	NA	NA
Bos_taurus_newGene_82541	NA	NA	NA
Bos_taurus_newGene_82557	NA	NA	NA
Bos_taurus_newGene_82561	NA	NA	NA
Bos_taurus_newGene_82562	NA	NA	NA
Bos_taurus_newGene_82563	NA	NA	NA
Bos_taurus_newGene_82564	0.4347094	0.4242473	0.3723809
Bos_taurus_newGene_82566	NA	NA	NA
Bos_taurus_newGene_82567	0.150984	0.7955975	0.0993066

Bos_taurus_newGene_82571	NA	NA	NA
Bos_taurus_newGene_82573	NA	NA	NA
Bos_taurus_newGene_82576	NA	NA	NA
Bos_taurus_newGene_82582	NA	NA	NA
Bos_taurus_newGene_82583	NA	NA	NA
Bos_taurus_newGene_82586	NA	NA	NA
Bos_taurus_newGene_82595	NA	NA	NA
Bos_taurus_newGene_82596	NA	NA	NA
Bos_taurus_newGene_82600	NA	NA	NA
Bos_taurus_newGene_82601	NA	NA	NA
Bos_taurus_newGene_82602	NA	NA	NA
Bos_taurus_newGene_82603	NA	NA	NA
Bos_taurus_newGene_82604	NA	NA	NA
Bos_taurus_newGene_82605	NA	NA	NA
Bos_taurus_newGene_82607	NA	NA	NA
Bos_taurus_newGene_82609	NA	NA	NA
Bos_taurus_newGene_82610	NA	NA	NA
Bos_taurus_newGene_82611	NA	NA	NA
Bos_taurus_newGene_82612	NA	NA	NA
Bos_taurus_newGene_82613	NA	NA	NA
Bos_taurus_newGene_82616	NA	NA	NA
Bos_taurus_newGene_82621	NA	NA	NA
Bos_taurus_newGene_82622	NA	NA	NA
Bos_taurus_newGene_82625	NA	NA	NA
Bos_taurus_newGene_82629	NA	NA	NA
Bos_taurus_newGene_82630	NA	NA	NA
Bos_taurus_newGene_82656	NA	NA	NA
Bos_taurus_newGene_82745	NA	NA	NA
Bos_taurus_newGene_82766	NA	NA	NA
Bos_taurus_newGene_82795	NA	NA	NA
Bos_taurus_newGene_82798	NA	NA	NA
Bos_taurus_newGene_82818	NA	NA	NA
Bos_taurus_newGene_82820	NA	NA	NA
Bos_taurus_newGene_82850	NA	NA	NA
Bos_taurus_newGene_82855	NA	NA	NA
Bos_taurus_newGene_82887	NA	NA	NA
Bos_taurus_newGene_82896	-0.093713	0.7658816	0.1158383
Bos_taurus_newGene_82922	NA	NA	NA
Bos_taurus_newGene_82928	-0.571367	0.1071097	0.9701711
Bos_taurus_newGene_82960	NA	NA	NA
Bos_taurus_newGene_82973	NA	NA	NA
Bos_taurus_newGene_83056	NA	NA	NA
Bos_taurus_newGene_83065	NA	NA	NA
Bos_taurus_newGene_83086	NA	NA	NA
Bos_taurus_newGene_83099	NA	NA	NA
Bos_taurus_newGene_83104	NA	NA	NA
Bos_taurus_newGene_83123	-0.141104	0.8343722	0.0786402
Bos_taurus_newGene_83133	0.0248629	0.9579481	0.018658
Bos_taurus_newGene_83161	NA	NA	NA
Bos_taurus_newGene_83163	NA	NA	NA
Bos_taurus_newGene_83178	NA	NA	NA
Bos_taurus_newGene_83287	NA	NA	NA
Bos_taurus_newGene_83323	NA	NA	NA
Bos_taurus_newGene_83352	NA	NA	NA
Bos_taurus_newGene_83363	NA	NA	NA
Bos_taurus_newGene_83477	NA	NA	NA
Bos_taurus_newGene_83522	NA	NA	NA
Bos_taurus_newGene_83530	NA	NA	NA

Bos_taurus_newGene_83548	NA	NA	NA
Bos_taurus_newGene_83549	-0.122483	0.8047363	0.0943464
Bos_taurus_newGene_83560	0.3684662	0.3867442	0.4125762
Bos_taurus_newGene_83629	NA	NA	NA
Bos_taurus_newGene_83669	NA	NA	NA
Bos_taurus_newGene_83670	NA	NA	NA
Bos_taurus_newGene_83688	-1.426074	0.0021885	2.6598586
Bos_taurus_newGene_83690	NA	NA	NA
Bos_taurus_newGene_83824	NA	NA	NA
Bos_taurus_newGene_83826	NA	NA	NA
Bos_taurus_newGene_83874	NA	NA	NA
Bos_taurus_newGene_83879	NA	NA	NA
Bos_taurus_newGene_83893	-0.35989	0.2628456	0.5802993
Bos_taurus_newGene_83906	NA	NA	NA
Bos_taurus_newGene_83907	NA	NA	NA
Bos_taurus_newGene_83958	-0.248348	0.6899586	0.161177
Bos_taurus_newGene_84100	NA	NA	NA
Bos_taurus_newGene_84136	NA	NA	NA
Bos_taurus_newGene_84146	1.8249041	0.0158443	1.8001265
Bos_taurus_newGene_84166	NA	NA	NA
Bos_taurus_newGene_84172	NA	NA	NA
Bos_taurus_newGene_84173	0.5761405	0.2977167	0.5261968
Bos_taurus_newGene_84207	NA	NA	NA
Bos_taurus_newGene_84292	-0.323216	0.5167676	0.2867047
Bos_taurus_newGene_84303	NA	NA	NA
Bos_taurus_newGene_84324	NA	NA	NA
Bos_taurus_newGene_84340	NA	NA	NA
Bos_taurus_newGene_84357	NA	NA	NA
Bos_taurus_newGene_84390	NA	NA	NA
Bos_taurus_newGene_84408	-0.695211	0.0257888	1.5885695
Bos_taurus_newGene_84420	NA	NA	NA
Bos_taurus_newGene_84421	NA	NA	NA
Bos_taurus_newGene_84477	-0.50175	0.0840884	1.0752641
Bos_taurus_newGene_84541	0.4642544	0.4823991	0.3165935
Bos_taurus_newGene_84542	NA	NA	NA
Bos_taurus_newGene_84545	-0.637278	0.3385515	0.4703752
Bos_taurus_newGene_84557	0.8622082	0.0600497	1.2214892
Bos_taurus_newGene_84559	NA	NA	NA
Bos_taurus_newGene_84578	NA	NA	NA
Bos_taurus_newGene_84643	NA	NA	NA
Bos_taurus_newGene_84644	NA	NA	NA
Bos_taurus_newGene_84655	NA	NA	NA
Bos_taurus_newGene_84701	NA	NA	NA
Bos_taurus_newGene_84726	NA	NA	NA
Bos_taurus_newGene_84827	NA	NA	NA
Bos_taurus_newGene_84837	NA	NA	NA
Bos_taurus_newGene_84942	NA	NA	NA
Bos_taurus_newGene_84958	NA	NA	NA
Bos_taurus_newGene_84960	NA	NA	NA
Bos_taurus_newGene_84961	NA	NA	NA
Bos_taurus_newGene_84962	NA	NA	NA
Bos_taurus_newGene_84964	NA	NA	NA
Bos_taurus_newGene_84968	NA	NA	NA
Bos_taurus_newGene_84969	NA	NA	NA
Bos_taurus_newGene_84971	NA	NA	NA
Bos_taurus_newGene_84972	NA	NA	NA
Bos_taurus_newGene_84976	NA	NA	NA
Bos_taurus_newGene_84977	NA	NA	NA

Bos_taurus_newGene_84980	NA	NA	NA
Bos_taurus_newGene_84983	NA	NA	NA
Bos_taurus_newGene_85011	0.0585435	0.9642141	0.0158265
Bos_taurus_newGene_85078	NA	NA	NA
Bos_taurus_newGene_85161	NA	NA	NA
Bos_taurus_newGene_85162	NA	NA	NA
Bos_taurus_newGene_85163	NA	NA	NA
Bos_taurus_newGene_85256	NA	NA	NA
Bos_taurus_newGene_85273	0.8473669	0.0370158	1.4316127
Bos_taurus_newGene_85274	NA	NA	NA
Bos_taurus_newGene_85390	NA	NA	NA
Bos_taurus_newGene_85516	-0.114467	0.7331528	0.1348055
Bos_taurus_newGene_85578	1.3068829	0.4773302	0.3211811
Bos_taurus_newGene_85588	NA	NA	NA
Bos_taurus_newGene_85648	NA	NA	NA
Bos_taurus_newGene_85670	0.4212067	0.8381353	0.0766859
Bos_taurus_newGene_85733	-0.914511	0.015812	1.8010143
Bos_taurus_newGene_85746	-0.897545	0.092141	1.0355472
Bos_taurus_newGene_85765	NA	NA	NA
Bos_taurus_newGene_85774	NA	NA	NA
Bos_taurus_newGene_85776	NA	NA	NA
Bos_taurus_newGene_85927	-0.445839	0.3112414	0.5069026
Bos_taurus_newGene_85953	1.2654067	0.211803	0.6740678
Bos_taurus_newGene_86122	NA	NA	NA
Bos_taurus_newGene_86129	NA	NA	NA
Bos_taurus_newGene_86156	NA	NA	NA
Bos_taurus_newGene_86201	NA	NA	NA
Bos_taurus_newGene_86276	NA	NA	NA
Bos_taurus_newGene_86277	NA	NA	NA
Bos_taurus_newGene_86428	NA	NA	NA
Bos_taurus_newGene_86475	NA	NA	NA
Bos_taurus_newGene_86500	NA	NA	NA
Bos_taurus_newGene_86598	NA	NA	NA
Bos_taurus_newGene_86765	NA	NA	NA
Bos_taurus_newGene_86827	-0.678603	0.1778234	0.7500111
Bos_taurus_newGene_86829	-1.273222	0.0482521	1.3164837
Bos_taurus_newGene_86831	-0.510055	0.2010602	0.6966738
Bos_taurus_newGene_86835	NA	NA	NA
Bos_taurus_newGene_86845	NA	NA	NA
Bos_taurus_newGene_86994	NA	NA	NA
Bos_taurus_newGene_87187	NA	NA	NA
Bos_taurus_newGene_87213	NA	NA	NA
Bos_taurus_newGene_87234	NA	NA	NA
Bos_taurus_newGene_87246	1.8735519	0.0072236	2.1412484
Bos_taurus_newGene_87251	NA	NA	NA
Bos_taurus_newGene_87255	NA	NA	NA
Bos_taurus_newGene_87263	NA	NA	NA
Bos_taurus_newGene_87296	NA	NA	NA
Bos_taurus_newGene_87341	NA	NA	NA
Bos_taurus_newGene_87444	NA	NA	NA
Bos_taurus_newGene_87468	NA	NA	NA
Bos_taurus_newGene_87490	-0.989483	0.0752318	1.1235984
Bos_taurus_newGene_87516	NA	NA	NA
Bos_taurus_newGene_87519	NA	NA	NA
Bos_taurus_newGene_87522	NA	NA	NA
Bos_taurus_newGene_87549	NA	NA	NA
Bos_taurus_newGene_87579	NA	NA	NA
Bos_taurus_newGene_87599	NA	NA	NA

Bos_taurus_newGene_87680	NA	NA	NA
Bos_taurus_newGene_87771	NA	NA	NA
Bos_taurus_newGene_87817	NA	NA	NA
Bos_taurus_newGene_87895	NA	NA	NA
Bos_taurus_newGene_87951	-1.642394	0.0038792	2.4112578
Bos_taurus_newGene_87971	NA	NA	NA
Bos_taurus_newGene_88018	NA	NA	NA
Bos_taurus_newGene_88042	NA	NA	NA
Bos_taurus_newGene_88063	0.0241103	0.9465468	0.0238579
Bos_taurus_newGene_88077	0.1151988	0.7045153	0.1521096
Bos_taurus_newGene_88193	NA	NA	NA
Bos_taurus_newGene_88227	NA	NA	NA
Bos_taurus_newGene_88228	NA	NA	NA
Bos_taurus_newGene_88241	0.5913356	0.5605289	0.251402
Bos_taurus_newGene_88316	NA	NA	NA
Bos_taurus_newGene_88347	NA	NA	NA
Bos_taurus_newGene_88385	NA	NA	NA
Bos_taurus_newGene_88405	0.0387921	0.9640791	0.0158874
Bos_taurus_newGene_88406	NA	NA	NA
Bos_taurus_newGene_88433	-0.160806	0.580846	0.235939
Bos_taurus_newGene_88488	NA	NA	NA
Bos_taurus_newGene_88535	NA	NA	NA
Bos_taurus_newGene_88539	NA	NA	NA
Bos_taurus_newGene_88547	NA	NA	NA
Bos_taurus_newGene_88548	NA	NA	NA
Bos_taurus_newGene_88549	NA	NA	NA
Bos_taurus_newGene_88550	NA	NA	NA
Bos_taurus_newGene_88552	NA	NA	NA
Bos_taurus_newGene_88553	NA	NA	NA
Bos_taurus_newGene_88555	NA	NA	NA
Bos_taurus_newGene_88556	NA	NA	NA
Bos_taurus_newGene_88559	NA	NA	NA
Bos_taurus_newGene_88688	NA	NA	NA
Bos_taurus_newGene_88785	NA	NA	NA
Bos_taurus_newGene_88792	NA	NA	NA
Bos_taurus_newGene_88812	NA	NA	NA
Bos_taurus_newGene_88829	NA	NA	NA
Bos_taurus_newGene_88868	NA	NA	NA
Bos_taurus_newGene_88872	NA	NA	NA
Bos_taurus_newGene_88943	NA	NA	NA
Bos_taurus_newGene_88951	NA	NA	NA
Bos_taurus_newGene_88959	NA	NA	NA
Bos_taurus_newGene_88962	NA	NA	NA
Bos_taurus_newGene_88963	NA	NA	NA
Bos_taurus_newGene_88964	NA	NA	NA
Bos_taurus_newGene_88967	NA	NA	NA
Bos_taurus_newGene_88977	NA	NA	NA
Bos_taurus_newGene_89079	NA	NA	NA
Bos_taurus_newGene_89132	NA	NA	NA
Bos_taurus_newGene_89155	-0.327205	0.6378776	0.1952627
Bos_taurus_newGene_89304	-2.594525	6.22E-08	7.2061761
Bos_taurus_newGene_89323	-2.148179	2.30E-06	5.6383949
Bos_taurus_newGene_89361	4.3378135	0.0262744	1.5804673
Bos_taurus_newGene_89458	1.5277997	0.0144742	1.8394059
Bos_taurus_newGene_89471	NA	NA	NA
Bos_taurus_newGene_89511	1.4703688	0.0016088	2.7935023
Bos_taurus_newGene_89602	NA	NA	NA
Bos_taurus_newGene_89635	NA	NA	NA

Bos_taurus_newGene_89880	-1.119652	0.0119418	1.9229297
Bos_taurus_newGene_89926	NA	NA	NA
Bos_taurus_newGene_89930	NA	NA	NA
Bos_taurus_newGene_89932	-0.145551	0.7907385	0.1019671
Bos_taurus_newGene_89954	NA	NA	NA
Bos_taurus_newGene_89964	NA	NA	NA
Bos_taurus_newGene_89974	NA	NA	NA
Bos_taurus_newGene_89979	NA	NA	NA
Bos_taurus_newGene_90006	-0.778919	0.0974533	1.0112034
Bos_taurus_newGene_90050	-1.504194	0.0005611	3.250935
Bos_taurus_newGene_90100	NA	NA	NA
Bos_taurus_newGene_90106	NA	NA	NA
Bos_taurus_newGene_90107	NA	NA	NA
Bos_taurus_newGene_90152	NA	NA	NA
Bos_taurus_newGene_90163	-2.784458	0.0001503	3.8231451
Bos_taurus_newGene_90179	NA	NA	NA
Bos_taurus_newGene_90294	NA	NA	NA
Bos_taurus_newGene_90419	NA	NA	NA
Bos_taurus_newGene_90563	NA	NA	NA
Bos_taurus_newGene_90717	-1.909143	3.26E-06	5.4868317
Bos_taurus_newGene_90758	NA	NA	NA
Bos_taurus_newGene_90776	NA	NA	NA
Bos_taurus_newGene_90850	NA	NA	NA
Bos_taurus_newGene_90855	NA	NA	NA
Bos_taurus_newGene_90904	NA	NA	NA
Bos_taurus_newGene_90980	0.9182021	0.0784055	1.1056535
Bos_taurus_newGene_90982	NA	NA	NA
Bos_taurus_newGene_90987	NA	NA	NA
Bos_taurus_newGene_90991	NA	NA	NA
Bos_taurus_newGene_91005	0.5123885	0.3393618	0.469337
Bos_taurus_newGene_91017	NA	NA	NA
Bos_taurus_newGene_91043	NA	NA	NA
Bos_taurus_newGene_91053	NA	NA	NA
Bos_taurus_newGene_91058	2.6499048	8.88E-08	7.0517993
Bos_taurus_newGene_91100	NA	NA	NA
Bos_taurus_newGene_91102	NA	NA	NA
Bos_taurus_newGene_91123	NA	NA	NA
Bos_taurus_newGene_91186	NA	NA	NA
Bos_taurus_newGene_91482	NA	NA	NA
Bos_taurus_newGene_91489	-0.015528	0.9762348	0.0104457
Bos_taurus_newGene_91494	NA	NA	NA
Bos_taurus_newGene_91496	-0.239026	0.6416666	0.1926906
Bos_taurus_newGene_91538	NA	NA	NA
Bos_taurus_newGene_91539	NA	NA	NA
Bos_taurus_newGene_91545	NA	NA	NA
Bos_taurus_newGene_91548	NA	NA	NA
Bos_taurus_newGene_91588	NA	NA	NA
Bos_taurus_newGene_91593	NA	NA	NA
Bos_taurus_newGene_91595	NA	NA	NA
Bos_taurus_newGene_91612	-0.902848	0.1480939	0.8294628
Bos_taurus_newGene_91615	NA	NA	NA
Bos_taurus_newGene_91618	-0.314157	0.5626027	0.2497982
Bos_taurus_newGene_91633	NA	NA	NA
Bos_taurus_newGene_91747	NA	NA	NA
Bos_taurus_newGene_91771	NA	NA	NA
Bos_taurus_newGene_91796	NA	NA	NA
Bos_taurus_newGene_91804	-0.507366	0.2707813	0.5673813
Bos_taurus_newGene_91809	0.139403	0.8036607	0.0949273

Bos_taurus_newGene_91812	NA	NA	NA
Bos_taurus_newGene_91823	NA	NA	NA
Bos_taurus_newGene_91922	NA	NA	NA
Bos_taurus_newGene_91926	NA	NA	NA
Bos_taurus_newGene_91957	NA	NA	NA
Bos_taurus_newGene_91960	NA	NA	NA
Bos_taurus_newGene_92008	NA	NA	NA
Bos_taurus_newGene_92042	NA	NA	NA
Bos_taurus_newGene_92047	NA	NA	NA
Bos_taurus_newGene_92050	NA	NA	NA
Bos_taurus_newGene_92091	NA	NA	NA
Bos_taurus_newGene_92093	NA	NA	NA
Bos_taurus_newGene_92107	NA	NA	NA
Bos_taurus_newGene_92126	NA	NA	NA
Bos_taurus_newGene_92137	NA	NA	NA
Bos_taurus_newGene_92155	NA	NA	NA
Bos_taurus_newGene_92162	NA	NA	NA
Bos_taurus_newGene_92171	0.2126237	0.5922894	0.227466
Bos_taurus_newGene_92192	NA	NA	NA
Bos_taurus_newGene_92193	NA	NA	NA
Bos_taurus_newGene_92206	NA	NA	NA
Bos_taurus_newGene_92221	NA	NA	NA
Bos_taurus_newGene_92244	NA	NA	NA
Bos_taurus_newGene_92350	NA	NA	NA
Bos_taurus_newGene_92372	NA	NA	NA
Bos_taurus_newGene_92386	NA	NA	NA
Bos_taurus_newGene_92391	NA	NA	NA
Bos_taurus_newGene_92392	-0.069729	0.9048336	0.0434313
Bos_taurus_newGene_92507	NA	NA	NA
Bos_taurus_newGene_92567	NA	NA	NA
Bos_taurus_newGene_92635	NA	NA	NA
Bos_taurus_newGene_92659	NA	NA	NA
Bos_taurus_newGene_92661	NA	NA	NA
Bos_taurus_newGene_92672	0.0570571	0.9020422	0.0447731
Bos_taurus_newGene_92688	NA	NA	NA
Bos_taurus_newGene_92744	NA	NA	NA
Bos_taurus_newGene_92786	NA	NA	NA
Bos_taurus_newGene_92845	NA	NA	NA
Bos_taurus_newGene_92939	NA	NA	NA
Bos_taurus_newGene_92969	1.5901958	0.0004818	3.3171467
Bos_taurus_newGene_92983	-0.768845	0.1991802	0.7007539
Bos_taurus_newGene_92988	NA	NA	NA
Bos_taurus_newGene_93005	NA	NA	NA
Bos_taurus_newGene_93016	0.0708719	0.791155	0.1017384
Bos_taurus_newGene_93027	NA	NA	NA
Bos_taurus_newGene_93031	NA	NA	NA
Bos_taurus_newGene_93037	0.3895543	0.232984	0.6326739
Bos_taurus_newGene_93045	NA	NA	NA
Bos_taurus_newGene_93048	NA	NA	NA
Bos_taurus_newGene_93062	-1.636375	0.0324041	1.4894001
Bos_taurus_newGene_93071	NA	NA	NA
Bos_taurus_newGene_93112	NA	NA	NA
Bos_taurus_newGene_93218	NA	NA	NA
Bos_taurus_newGene_93332	NA	NA	NA
Bos_taurus_newGene_93334	NA	NA	NA
Bos_taurus_newGene_93339	NA	NA	NA
Bos_taurus_newGene_93344	NA	NA	NA
Bos_taurus_newGene_93352	NA	NA	NA

Bos_taurus_newGene_93353	NA	NA	NA
Bos_taurus_newGene_93354	NA	NA	NA
Bos_taurus_newGene_93356	NA	NA	NA
Bos_taurus_newGene_93357	NA	NA	NA
Bos_taurus_newGene_93358	NA	NA	NA
Bos_taurus_newGene_93360	NA	NA	NA
Bos_taurus_newGene_93361	NA	NA	NA
Bos_taurus_newGene_93365	NA	NA	NA
Bos_taurus_newGene_93366	NA	NA	NA
Bos_taurus_newGene_93372	-0.636283	0.2530881	0.5967282
Bos_taurus_newGene_93379	NA	NA	NA
Bos_taurus_newGene_93381	NA	NA	NA
Bos_taurus_newGene_93400	0.7099285	0.6477363	0.1886017
Bos_taurus_newGene_93401	NA	NA	NA
Bos_taurus_newGene_93415	-0.110274	0.8129464	0.0899381
Bos_taurus_newGene_93444	NA	NA	NA
Bos_taurus_newGene_93447	NA	NA	NA
Bos_taurus_newGene_93493	0.2058108	0.5679958	0.2456548
Bos_taurus_newGene_93508	NA	NA	NA
Bos_taurus_newGene_93529	0.1980509	0.8527639	0.0691712
Bos_taurus_newGene_93559	NA	NA	NA
Bos_taurus_newGene_93561	NA	NA	NA
Bos_taurus_newGene_93572	NA	NA	NA
Bos_taurus_newGene_93623	0.9665986	0.1107305	0.9557328
Bos_taurus_newGene_93641	NA	NA	NA
Bos_taurus_newGene_93672	NA	NA	NA
Bos_taurus_newGene_93685	-0.110813	0.7910574	0.101792
Bos_taurus_newGene_93686	NA	NA	NA
Bos_taurus_newGene_93716	NA	NA	NA
Bos_taurus_newGene_93731	NA	NA	NA
Bos_taurus_newGene_93739	NA	NA	NA
Bos_taurus_newGene_93740	NA	NA	NA
Bos_taurus_newGene_93745	NA	NA	NA
Bos_taurus_newGene_93757	NA	NA	NA
Bos_taurus_newGene_93759	NA	NA	NA
Bos_taurus_newGene_93760	NA	NA	NA
Bos_taurus_newGene_93779	NA	NA	NA
Bos_taurus_newGene_93806	NA	NA	NA
Bos_taurus_newGene_93817	NA	NA	NA
Bos_taurus_newGene_93821	NA	NA	NA
Bos_taurus_newGene_93827	NA	NA	NA
Bos_taurus_newGene_93834	NA	NA	NA
Bos_taurus_newGene_93859	-0.155539	0.6343046	0.1977021
Bos_taurus_newGene_93865	NA	NA	NA
Bos_taurus_newGene_93869	-1.91334	6.81E-05	4.1667572
Bos_taurus_newGene_93882	NA	NA	NA
Bos_taurus_newGene_93914	NA	NA	NA
Bos_taurus_newGene_93967	NA	NA	NA
Bos_taurus_newGene_93968	0.229061	0.6673416	0.1756518
Bos_taurus_newGene_93977	NA	NA	NA
Bos_taurus_newGene_94015	0.0874167	0.7756137	0.1103545
Bos_taurus_newGene_94017	NA	NA	NA
Bos_taurus_newGene_94030	-0.193995	0.6269385	0.202775
Bos_taurus_newGene_94040	-0.548224	0.2642582	0.5779715
Bos_taurus_newGene_94044	0.1729107	0.5484332	0.2608762
Bos_taurus_newGene_94046	NA	NA	NA
Bos_taurus_newGene_94052	-0.870237	0.0412897	1.3841585
Bos_taurus_newGene_94066	0.1174803	0.758728	0.1199139

Bos_taurus_newGene_94075	-0.353748	0.3134549	0.503825
Bos_taurus_newGene_94087	0.7578566	0.1015635	0.9932622
Bos_taurus_newGene_94089	0.703307	0.0459168	1.3380284
Bos_taurus_newGene_94112	0.5004023	0.3341243	0.476092
Bos_taurus_newGene_94121	NA	NA	NA
Bos_taurus_newGene_94249	NA	NA	NA
Bos_taurus_newGene_94269	NA	NA	NA
Bos_taurus_newGene_94314	-0.417462	0.1234857	0.9083835
Bos_taurus_newGene_94345	-0.129775	0.7191254	0.1431954
Bos_taurus_newGene_94391	0.1615376	0.5537454	0.2566899
Bos_taurus_newGene_94392	NA	NA	NA
Bos_taurus_newGene_94397	NA	NA	NA
Bos_taurus_newGene_94398	-0.419576	0.3700432	0.4317476
Bos_taurus_newGene_94401	NA	NA	NA
Bos_taurus_newGene_94403	NA	NA	NA
Bos_taurus_newGene_94406	NA	NA	NA
Bos_taurus_newGene_94426	NA	NA	NA
Bos_taurus_newGene_94433	NA	NA	NA
Bos_taurus_newGene_94501	NA	NA	NA
Bos_taurus_newGene_94528	NA	NA	NA
Bos_taurus_newGene_94533	NA	NA	NA
Bos_taurus_newGene_94538	NA	NA	NA
Bos_taurus_newGene_94748	0.1984341	0.6430922	0.1917267
Bos_taurus_newGene_94779	NA	NA	NA
Bos_taurus_newGene_94855	NA	NA	NA
Bos_taurus_newGene_94903	NA	NA	NA
Bos_taurus_newGene_95005	NA	NA	NA
Bos_taurus_newGene_95011	NA	NA	NA
Bos_taurus_newGene_95012	NA	NA	NA
Bos_taurus_newGene_95014	NA	NA	NA
Bos_taurus_newGene_95036	-0.401224	0.2741441	0.5620211
Bos_taurus_newGene_95039	-0.857577	0.0380499	1.419646
Bos_taurus_newGene_95044	-0.553196	0.1329608	0.8762764
Bos_taurus_newGene_95046	-0.293344	0.5870477	0.2313266
Bos_taurus_newGene_95047	-0.24924	0.6113264	0.2137268
Bos_taurus_newGene_95048	NA	NA	NA
Bos_taurus_newGene_95051	NA	NA	NA
Bos_taurus_newGene_95091	NA	NA	NA
Bos_taurus_newGene_95122	NA	NA	NA
Bos_taurus_newGene_95143	NA	NA	NA
Bos_taurus_newGene_95222	NA	NA	NA
Bos_taurus_newGene_95230	NA	NA	NA
Bos_taurus_newGene_95236	0.0037675	1	0
Bos_taurus_newGene_95237	NA	NA	NA
Bos_taurus_newGene_95238	NA	NA	NA
Bos_taurus_newGene_95239	NA	NA	NA
Bos_taurus_newGene_95240	NA	NA	NA
Bos_taurus_newGene_95247	NA	NA	NA
Bos_taurus_newGene_95252	NA	NA	NA
Bos_taurus_newGene_95253	NA	NA	NA
Bos_taurus_newGene_95254	NA	NA	NA
Bos_taurus_newGene_95255	NA	NA	NA
Bos_taurus_newGene_95256	NA	NA	NA
Bos_taurus_newGene_95257	NA	NA	NA
Bos_taurus_newGene_95325	NA	NA	NA
Bos_taurus_newGene_95333	-0.318318	0.2082197	0.6814781
Bos_taurus_newGene_95375	NA	NA	NA
Bos_taurus_newGene_95378	NA	NA	NA

Bos_taurus_newGene_95387	NA	NA	NA
Bos_taurus_newGene_95432	-1.697682	0.1492735	0.8260173
Bos_taurus_newGene_95436	-1.324033	0.0048903	2.3106608
Bos_taurus_newGene_95441	-1.028362	0.1103131	0.9573728
Bos_taurus_newGene_95443	-1.192987	0.0590534	1.2287549
Bos_taurus_newGene_95450	-0.724059	0.0584672	1.2330875
Bos_taurus_newGene_95622	NA	NA	NA
Bos_taurus_newGene_95647	NA	NA	NA
Bos_taurus_newGene_95692	NA	NA	NA
Bos_taurus_newGene_95693	NA	NA	NA
Bos_taurus_newGene_95696	0.1619732	0.7572203	0.1207778
Bos_taurus_newGene_95732	NA	NA	NA
Bos_taurus_newGene_95735	NA	NA	NA
Bos_taurus_newGene_95742	NA	NA	NA
Bos_taurus_newGene_95792	NA	NA	NA
Bos_taurus_newGene_95842	NA	NA	NA
Bos_taurus_newGene_95844	NA	NA	NA
Bos_taurus_newGene_95847	NA	NA	NA
Bos_taurus_newGene_95850	NA	NA	NA
Bos_taurus_newGene_95866	NA	NA	NA
Bos_taurus_newGene_95895	NA	NA	NA
Bos_taurus_newGene_95898	NA	NA	NA
Bos_taurus_newGene_95900	NA	NA	NA
Bos_taurus_newGene_95901	NA	NA	NA
Bos_taurus_newGene_95918	NA	NA	NA
Bos_taurus_newGene_95932	NA	NA	NA
Bos_taurus_newGene_95957	NA	NA	NA
Bos_taurus_newGene_95959	NA	NA	NA
Bos_taurus_newGene_95965	NA	NA	NA
Bos_taurus_newGene_96018	NA	NA	NA
Bos_taurus_newGene_96035	NA	NA	NA
Bos_taurus_newGene_96046	-0.008816	0.9908007	0.0040137
Bos_taurus_newGene_96050	NA	NA	NA
Bos_taurus_newGene_96052	NA	NA	NA
Bos_taurus_newGene_96055	NA	NA	NA
Bos_taurus_newGene_96056	-0.050969	0.8700561	0.0604527
Bos_taurus_newGene_96058	NA	NA	NA
Bos_taurus_newGene_96064	-0.123501	0.948259	0.023073
Bos_taurus_newGene_96069	0.579811	0.3011849	0.5211667
Bos_taurus_newGene_96090	NA	NA	NA
Bos_taurus_newGene_96099	NA	NA	NA
Bos_taurus_newGene_96251	NA	NA	NA
Bos_taurus_newGene_96256	NA	NA	NA
Bos_taurus_newGene_96262	0.7261061	0.1297454	0.886908
Bos_taurus_newGene_96269	0.4834384	0.3017253	0.5203883
Bos_taurus_newGene_96271	0.5171147	0.2307235	0.6369082
Bos_taurus_newGene_96273	NA	NA	NA
Bos_taurus_newGene_96284	NA	NA	NA
Bos_taurus_newGene_96288	0.1825281	0.5907005	0.2286327
Bos_taurus_newGene_96317	NA	NA	NA
Bos_taurus_newGene_96337	NA	NA	NA
Bos_taurus_newGene_96348	0.0339266	0.922454	0.0350553
Bos_taurus_newGene_96434	NA	NA	NA
Bos_taurus_newGene_96437	NA	NA	NA
Bos_taurus_newGene_96518	0.1956877	0.6781522	0.1686728
Bos_taurus_newGene_96535	NA	NA	NA
Bos_taurus_newGene_96539	NA	NA	NA
Bos_taurus_newGene_96586	NA	NA	NA

Bos_taurus_newGene_96602	NA	NA	NA
Bos_taurus_newGene_96665	NA	NA	NA
Bos_taurus_newGene_96666	NA	NA	NA
Bos_taurus_newGene_96698	NA	NA	NA
Bos_taurus_newGene_96777	NA	NA	NA
Bos_taurus_newGene_96825	NA	NA	NA
Bos_taurus_newGene_96826	-0.206891	0.6347482	0.1973985
Bos_taurus_newGene_96842	-0.07009	0.8353479	0.0781326
Bos_taurus_newGene_96844	-0.43522	0.1280604	0.8925852
Bos_taurus_newGene_96850	NA	NA	NA
Bos_taurus_newGene_96863	NA	NA	NA
Bos_taurus_newGene_96908	NA	NA	NA
Bos_taurus_newGene_96909	NA	NA	NA
Bos_taurus_newGene_96912	NA	NA	NA
Bos_taurus_newGene_96913	NA	NA	NA
Bos_taurus_newGene_96914	NA	NA	NA
Bos_taurus_newGene_96915	NA	NA	NA
Bos_taurus_newGene_96917	NA	NA	NA
Bos_taurus_newGene_96918	NA	NA	NA
Bos_taurus_newGene_96919	0.3322036	0.5308292	0.2750452
Bos_taurus_newGene_96921	NA	NA	NA
Bos_taurus_newGene_96934	NA	NA	NA
Bos_taurus_newGene_96936	NA	NA	NA
Bos_taurus_newGene_96956	NA	NA	NA
Bos_taurus_newGene_96967	-0.146413	0.8162822	0.0881597
Bos_taurus_newGene_97008	NA	NA	NA
Bos_taurus_newGene_97013	NA	NA	NA
Bos_taurus_newGene_97048	-0.595928	0.0242087	1.6160287
Bos_taurus_newGene_97129	NA	NA	NA
Bos_taurus_newGene_97197	NA	NA	NA
Bos_taurus_newGene_97216	NA	NA	NA
Bos_taurus_newGene_97334	NA	NA	NA
Bos_taurus_newGene_97406	NA	NA	NA
Bos_taurus_newGene_97422	NA	NA	NA
Bos_taurus_newGene_97499	NA	NA	NA
Bos_taurus_newGene_97552	NA	NA	NA
Bos_taurus_newGene_97674	NA	NA	NA
Bos_taurus_newGene_97770	-0.149541	0.6679951	0.1752267
Bos_taurus_newGene_97787	0.1632483	0.6031572	0.2195695
Bos_taurus_newGene_97861	NA	NA	NA
Bos_taurus_newGene_97895	-0.087285	0.7988119	0.0975555
Bos_taurus_newGene_97915	NA	NA	NA
Bos_taurus_newGene_97924	-0.000342	1	0
Bos_taurus_newGene_97957	NA	NA	NA
Bos_taurus_newGene_98007	NA	NA	NA
Bos_taurus_newGene_98008	-0.171046	0.6605965	0.1800637
Bos_taurus_newGene_98086	0.1076095	0.7843538	0.105488
Bos_taurus_newGene_98093	-0.127768	0.7188949	0.1433346
Bos_taurus_newGene_98099	NA	NA	NA
Bos_taurus_newGene_98100	NA	NA	NA
Bos_taurus_newGene_98101	NA	NA	NA
Bos_taurus_newGene_98102	NA	NA	NA
Bos_taurus_newGene_98242	NA	NA	NA
Bos_taurus_newGene_98269	NA	NA	NA
Bos_taurus_newGene_98277	NA	NA	NA
Bos_taurus_newGene_98301	NA	NA	NA
Bos_taurus_newGene_98369	NA	NA	NA
Bos_taurus_newGene_98370	NA	NA	NA

Bos_taurus_newGene_98374	NA	NA	NA
Bos_taurus_newGene_98409	NA	NA	NA
Bos_taurus_newGene_98410	0.5213049	0.2974107	0.5266434
Bos_taurus_newGene_98411	NA	NA	NA
Bos_taurus_newGene_98419	0.1881399	0.7014367	0.1540115
Bos_taurus_newGene_98424	NA	NA	NA
Bos_taurus_newGene_98441	NA	NA	NA
Bos_taurus_newGene_98464	NA	NA	NA
Bos_taurus_newGene_98488	-0.584413	0.1696536	0.7704369
Bos_taurus_newGene_98491	NA	NA	NA
Bos_taurus_newGene_98497	NA	NA	NA
Bos_taurus_newGene_98514	NA	NA	NA
Bos_taurus_newGene_98515	NA	NA	NA
Bos_taurus_newGene_98516	0.9819823	0.1748181	0.7574136
Bos_taurus_newGene_98545	NA	NA	NA
Bos_taurus_newGene_98557	0.0884831	0.8406524	0.0753836
Bos_taurus_newGene_98609	NA	NA	NA
Bos_taurus_newGene_98612	NA	NA	NA
Bos_taurus_newGene_98649	NA	NA	NA
Bos_taurus_newGene_98746	NA	NA	NA
Bos_taurus_newGene_98752	1.288783	0.0310588	1.5078153
Bos_taurus_newGene_98756	NA	NA	NA
Bos_taurus_newGene_98789	NA	NA	NA
Bos_taurus_newGene_98831	NA	NA	NA
Bos_taurus_newGene_98860	NA	NA	NA
Bos_taurus_newGene_98912	NA	NA	NA
Bos_taurus_newGene_98917	NA	NA	NA
Bos_taurus_newGene_98931	NA	NA	NA
Bos_taurus_newGene_98940	-0.176952	0.7219258	0.1415075
Bos_taurus_newGene_99099	-1.045853	0.0213553	1.6704945
Bos_taurus_newGene_99173	0.1098414	0.8530053	0.0690483
Bos_taurus_newGene_99187	NA	NA	NA
Bos_taurus_newGene_99192	0.2005604	0.7928205	0.1008251
Bos_taurus_newGene_99209	NA	NA	NA
Bos_taurus_newGene_99244	NA	NA	NA
Bos_taurus_newGene_99256	NA	NA	NA
Bos_taurus_newGene_99258	NA	NA	NA
Bos_taurus_newGene_99263	-0.665595	0.2076659	0.6826347
Bos_taurus_newGene_99269	NA	NA	NA
Bos_taurus_newGene_99275	NA	NA	NA
Bos_taurus_newGene_99385	NA	NA	NA
Bos_taurus_newGene_99392	NA	NA	NA
Bos_taurus_newGene_99393	NA	NA	NA
Bos_taurus_newGene_99401	NA	NA	NA
Bos_taurus_newGene_99404	NA	NA	NA
Bos_taurus_newGene_99405	NA	NA	NA
Bos_taurus_newGene_99406	NA	NA	NA
Bos_taurus_newGene_99418	NA	NA	NA
Bos_taurus_newGene_99421	-0.297641	0.5493801	0.2601271
Bos_taurus_newGene_99476	NA	NA	NA
Bos_taurus_newGene_99493	NA	NA	NA
Bos_taurus_newGene_99562	NA	NA	NA
Bos_taurus_newGene_99600	0.0540289	0.9089345	0.0414674
Bos_taurus_newGene_99656	-0.492504	0.362819	0.44031
Bos_taurus_newGene_99671	-0.80349	0.0282801	1.5485187
Bos_taurus_newGene_99681	-0.985036	0.0282304	1.5492836
Bos_taurus_newGene_99689	-1.169032	0.0031984	2.4950693
Bos_taurus_newGene_99697	-0.877846	0.0163642	1.7861059

Bos_taurus_newGene_99714	-3.147872	0.0674418	1.171071
Bos_taurus_newGene_99765	-0.756547	0.0358334	1.4457125
Bos_taurus_newGene_99779	-0.784273	0.0438695	1.3578378
Bos_taurus_newGene_99813	-1.119329	0.0035397	2.4510294
Bos_taurus_newGene_99843	NA	NA	NA
Bos_taurus_newGene_99845	NA	NA	NA
Bos_taurus_newGene_99876	NA	NA	NA
Bos_taurus_newGene_99899	NA	NA	NA
Bos_taurus_newGene_10032	NA	NA	NA
Bos_taurus_newGene_10033	0.6486689	0.1830117	0.7375211
Bos_taurus_newGene_10035	NA	NA	NA
Bos_taurus_newGene_10038	-0.371442	0.4886066	0.3110407
Bos_taurus_newGene_10043	0.4403772	0.3356247	0.474146
Bos_taurus_newGene_10044	NA	NA	NA
Bos_taurus_newGene_10048	NA	NA	NA
Bos_taurus_newGene_10049	NA	NA	NA
Bos_taurus_newGene_10050	NA	NA	NA
Bos_taurus_newGene_10050	0.5421411	0.241179	0.6176606
Bos_taurus_newGene_10051	NA	NA	NA
Bos_taurus_newGene_10055	NA	NA	NA
Bos_taurus_newGene_10057	0.5194466	0.1958174	0.7081486
Bos_taurus_newGene_10064	NA	NA	NA
Bos_taurus_newGene_10075	NA	NA	NA
Bos_taurus_newGene_10081	NA	NA	NA
Bos_taurus_newGene_10081	-2.601486	4.36E-08	7.360945
Bos_taurus_newGene_10106	NA	NA	NA
Bos_taurus_newGene_10112	0.267472	0.4055004	0.3920087
Bos_taurus_newGene_10113	NA	NA	NA
Bos_taurus_newGene_10113	NA	NA	NA
Bos_taurus_newGene_10124	NA	NA	NA
Bos_taurus_newGene_10125	NA	NA	NA
Bos_taurus_newGene_10128	NA	NA	NA
Bos_taurus_newGene_10131	NA	NA	NA
Bos_taurus_newGene_10132	NA	NA	NA
Bos_taurus_newGene_10132	NA	NA	NA
Bos_taurus_newGene_10135	NA	NA	NA
Bos_taurus_newGene_10136	NA	NA	NA
Bos_taurus_newGene_10137	NA	NA	NA
Bos_taurus_newGene_10137	NA	NA	NA
Bos_taurus_newGene_10138	NA	NA	NA
Bos_taurus_newGene_10155	NA	NA	NA
Bos_taurus_newGene_10155	NA	NA	NA
Bos_taurus_newGene_10155	NA	NA	NA
Bos_taurus_newGene_10160	NA	NA	NA
Bos_taurus_newGene_10162	NA	NA	NA
Bos_taurus_newGene_10168	-1.255487	0.0037614	2.4246467
Bos_taurus_newGene_10168	-1.773795	0.000297	3.5272669
Bos_taurus_newGene_10169	-1.948613	3.15E-05	4.5023655
Bos_taurus_newGene_10170	NA	NA	NA
Bos_taurus_newGene_10170	-0.942933	0.0565169	1.2478219
Bos_taurus_newGene_10170	-1.509725	0.0003765	3.4241889
Bos_taurus_newGene_10171	-0.270436	0.2875697	0.5412569
Bos_taurus_newGene_10171	NA	NA	NA
Bos_taurus_newGene_10171	-0.491196	0.1082184	0.9656987
Bos_taurus_newGene_10174	NA	NA	NA
Bos_taurus_newGene_10179	NA	NA	NA
Bos_taurus_newGene_10180	-0.341849	0.3387865	0.4700739
Bos_taurus_newGene_10183	NA	NA	NA

Bos_taurus_newGene_10189	NA	NA	NA
Bos_taurus_newGene_10193	NA	NA	NA
Bos_taurus_newGene_10195	NA	NA	NA
Bos_taurus_newGene_10196	NA	NA	NA
Bos_taurus_newGene_10196	0.5331093	0.1760682	0.7543191
Bos_taurus_newGene_10196	NA	NA	NA
Bos_taurus_newGene_10197	NA	NA	NA
Bos_taurus_newGene_10198	NA	NA	NA
Bos_taurus_newGene_10199	NA	NA	NA
Bos_taurus_newGene_10204	NA	NA	NA
Bos_taurus_newGene_10205	NA	NA	NA
Bos_taurus_newGene_10208	NA	NA	NA
Bos_taurus_newGene_10210	NA	NA	NA
Bos_taurus_newGene_10213	0.0581446	0.8924511	0.0494156
Bos_taurus_newGene_10214	0.9389179	0.0281425	1.550637
Bos_taurus_newGene_10215	NA	NA	NA
Bos_taurus_newGene_10217	NA	NA	NA
Bos_taurus_newGene_10222	0.348485	0.4158074	0.3811078
Bos_taurus_newGene_10222	NA	NA	NA
Bos_taurus_newGene_10223	-0.036739	0.9537672	0.0205576
Bos_taurus_newGene_10224	0.6355563	0.2749958	0.560674
Bos_taurus_newGene_10224	NA	NA	NA
Bos_taurus_newGene_10238	NA	NA	NA
Bos_taurus_newGene_10239	NA	NA	NA
Bos_taurus_newGene_10240	0.5750064	0.2630247	0.5800035
Bos_taurus_newGene_10240	NA	NA	NA
Bos_taurus_newGene_10247	1.747464	0.0087842	2.0562973
Bos_taurus_newGene_10248	0.2166407	0.5491236	0.2603299
Bos_taurus_newGene_10253	NA	NA	NA
Bos_taurus_newGene_10254	NA	NA	NA
Bos_taurus_newGene_10254	NA	NA	NA
Bos_taurus_newGene_10255	NA	NA	NA
Bos_taurus_newGene_10267	NA	NA	NA
Bos_taurus_newGene_10267	NA	NA	NA
Bos_taurus_newGene_10267	NA	NA	NA
Bos_taurus_newGene_10270	NA	NA	NA
Bos_taurus_newGene_10271	NA	NA	NA
Bos_taurus_newGene_10276	NA	NA	NA
Bos_taurus_newGene_10280	NA	NA	NA
Bos_taurus_newGene_10283	-0.012818	0.9714335	0.0125869
Bos_taurus_newGene_10283	NA	NA	NA
Bos_taurus_newGene_10285	NA	NA	NA
Bos_taurus_newGene_10286	NA	NA	NA
Bos_taurus_newGene_10291	NA	NA	NA
Bos_taurus_newGene_10291	NA	NA	NA
Bos_taurus_newGene_10291	NA	NA	NA
Bos_taurus_newGene_10296	NA	NA	NA
Bos_taurus_newGene_10303	NA	NA	NA
Bos_taurus_newGene_10310	NA	NA	NA
Bos_taurus_newGene_10316	NA	NA	NA
Bos_taurus_newGene_10324	NA	NA	NA
Bos_taurus_newGene_10324	-0.016397	0.9943124	0.0024771
Bos_taurus_newGene_10326	NA	NA	NA
Bos_taurus_newGene_10326	NA	NA	NA
Bos_taurus_newGene_10327	NA	NA	NA
Bos_taurus_newGene_10329	NA	NA	NA
Bos_taurus_newGene_10341	NA	NA	NA
Bos_taurus_newGene_10341	NA	NA	NA

Bos_taurus_newGene_103608	NA	NA	NA
Bos_taurus_newGene_103655	NA	NA	NA
Bos_taurus_newGene_103681	NA	NA	NA
Bos_taurus_newGene_103760	NA	NA	NA
Bos_taurus_newGene_103771	-0.186265	0.7247776	0.1397953
Bos_taurus_newGene_103785	NA	NA	NA
Bos_taurus_newGene_103858	NA	NA	NA
Bos_taurus_newGene_103974	NA	NA	NA
Bos_taurus_newGene_104005	NA	NA	NA
Bos_taurus_newGene_104116	NA	NA	NA
Bos_taurus_newGene_104394	-0.030869	0.9612546	0.0171616
Bos_taurus_newGene_104403	NA	NA	NA
Bos_taurus_newGene_104411	NA	NA	NA
Bos_taurus_newGene_104416	NA	NA	NA
Bos_taurus_newGene_104439	NA	NA	NA
Bos_taurus_newGene_104545	NA	NA	NA
Bos_taurus_newGene_104598	-0.093018	0.7628628	0.1175536
Bos_taurus_newGene_104702	NA	NA	NA
Bos_taurus_newGene_104706	NA	NA	NA
Bos_taurus_newGene_104758	NA	NA	NA
Bos_taurus_newGene_104789	NA	NA	NA
Bos_taurus_newGene_104914	0.0733377	0.8920084	0.0496311
Bos_taurus_newGene_104946	NA	NA	NA
Bos_taurus_newGene_104967	-0.6055	0.1265531	0.8977271
Bos_taurus_newGene_104978	-0.53298	0.1554665	0.8083633
Bos_taurus_newGene_104986	NA	NA	NA
Bos_taurus_newGene_105013	NA	NA	NA
Bos_taurus_newGene_105014	-0.335881	0.6149422	0.2111657
Bos_taurus_newGene_105015	NA	NA	NA
Bos_taurus_newGene_105016	NA	NA	NA
Bos_taurus_newGene_105017	NA	NA	NA
Bos_taurus_newGene_105076	NA	NA	NA
Bos_taurus_newGene_105209	NA	NA	NA
Bos_taurus_newGene_105277	NA	NA	NA
Bos_taurus_newGene_105343	NA	NA	NA
Bos_taurus_newGene_105344	0.0120419	0.9800334	0.0087591
Bos_taurus_newGene_105355	NA	NA	NA
Bos_taurus_newGene_105366	0.789712	0.1717326	0.7651473
Bos_taurus_newGene_105370	NA	NA	NA
Bos_taurus_newGene_105425	NA	NA	NA
Bos_taurus_newGene_105441	NA	NA	NA
Bos_taurus_newGene_105471	NA	NA	NA
Bos_taurus_newGene_105484	NA	NA	NA
Bos_taurus_newGene_105494	NA	NA	NA
Bos_taurus_newGene_105497	NA	NA	NA
Bos_taurus_newGene_105504	NA	NA	NA
Bos_taurus_newGene_105532	NA	NA	NA
Bos_taurus_newGene_105675	-0.016665	0.9815764	0.0080759
Bos_taurus_newGene_105676	NA	NA	NA
Bos_taurus_newGene_105690	NA	NA	NA
Bos_taurus_newGene_105707	NA	NA	NA
Bos_taurus_newGene_105720	NA	NA	NA
Bos_taurus_newGene_105723	NA	NA	NA
Bos_taurus_newGene_105783	NA	NA	NA
Bos_taurus_newGene_105814	0.0020042	1	0
Bos_taurus_newGene_105822	-0.470937	0.3369378	0.4724502
Bos_taurus_newGene_105841	NA	NA	NA
Bos_taurus_newGene_105863	-0.250642	0.4179228	0.378904

Bos_taurus_newGene_105918	NA	NA	NA
Bos_taurus_newGene_105919	-0.036498	0.9227838	0.0349
Bos_taurus_newGene_105942	NA	NA	NA
Bos_taurus_newGene_105997	NA	NA	NA
Bos_taurus_newGene_105999	NA	NA	NA
Bos_taurus_newGene_106055	0.3311514	0.2578185	0.5886859
Bos_taurus_newGene_106069	NA	NA	NA
Bos_taurus_newGene_106077	0.0514752	0.8886283	0.0512799
Bos_taurus_newGene_106090	NA	NA	NA
Bos_taurus_newGene_106094	NA	NA	NA
Bos_taurus_newGene_106097	NA	NA	NA
Bos_taurus_newGene_106132	0.0326172	0.8980558	0.0466967
Bos_taurus_newGene_106171	NA	NA	NA
Bos_taurus_newGene_106178	-0.21918	0.6103713	0.2144059
Bos_taurus_newGene_106182	NA	NA	NA
Bos_taurus_newGene_106184	NA	NA	NA
Bos_taurus_newGene_106198	NA	NA	NA
Bos_taurus_newGene_106203	NA	NA	NA
Bos_taurus_newGene_106218	NA	NA	NA
Bos_taurus_newGene_106220	-0.450575	0.3296662	0.4819256
Bos_taurus_newGene_106243	NA	NA	NA
Bos_taurus_newGene_106255	-0.376285	0.4370191	0.3594996
Bos_taurus_newGene_106393	NA	NA	NA
Bos_taurus_newGene_106461	NA	NA	NA
Bos_taurus_newGene_106462	0.1803648	0.6646731	0.1773919
Bos_taurus_newGene_106514	NA	NA	NA
Bos_taurus_newGene_106527	NA	NA	NA
Bos_taurus_newGene_106528	NA	NA	NA
Bos_taurus_newGene_106536	NA	NA	NA
Bos_taurus_newGene_106537	NA	NA	NA
Bos_taurus_newGene_106539	NA	NA	NA
Bos_taurus_newGene_106570	NA	NA	NA
Bos_taurus_newGene_106583	NA	NA	NA
Bos_taurus_newGene_106584	NA	NA	NA
Bos_taurus_newGene_106603	NA	NA	NA
Bos_taurus_newGene_106605	NA	NA	NA
Bos_taurus_newGene_106608	NA	NA	NA
Bos_taurus_newGene_106636	NA	NA	NA
Bos_taurus_newGene_106776	-0.332778	0.3680356	0.4341101
Bos_taurus_newGene_106787	NA	NA	NA
Bos_taurus_newGene_106821	NA	NA	NA
Bos_taurus_newGene_106878	NA	NA	NA
Bos_taurus_newGene_106920	2.2715414	5.18E-05	4.2860317
Bos_taurus_newGene_106927	-0.932088	0.0416292	1.380602
Bos_taurus_newGene_106928	NA	NA	NA
Bos_taurus_newGene_106952	NA	NA	NA
Bos_taurus_newGene_106997	0.0896053	0.8090531	0.092023
Bos_taurus_newGene_107042	-0.760681	0.1642725	0.7844352
Bos_taurus_newGene_107044	NA	NA	NA
Bos_taurus_newGene_107069	NA	NA	NA
Bos_taurus_newGene_107075	NA	NA	NA
Bos_taurus_newGene_107085	NA	NA	NA
Bos_taurus_newGene_107120	NA	NA	NA
Bos_taurus_newGene_107230	NA	NA	NA
Bos_taurus_newGene_107231	-0.065654	0.869734	0.0606136
Bos_taurus_newGene_107235	0.3370418	0.5028099	0.2985961
Bos_taurus_newGene_107259	NA	NA	NA
Bos_taurus_newGene_107261	NA	NA	NA

Bos_taurus_newGene_107264	NA	NA	NA
Bos_taurus_newGene_107269	NA	NA	NA
Bos_taurus_newGene_107352	NA	NA	NA
Bos_taurus_newGene_107363	NA	NA	NA
Bos_taurus_newGene_107425	NA	NA	NA
Bos_taurus_newGene_107445	NA	NA	NA
Bos_taurus_newGene_107465	NA	NA	NA
Bos_taurus_newGene_107547	0.1726758	0.6401181	0.1937399
Bos_taurus_newGene_107549	NA	NA	NA
Bos_taurus_newGene_107620	NA	NA	NA
Bos_taurus_newGene_107650	NA	NA	NA
Bos_taurus_newGene_107655	NA	NA	NA
Bos_taurus_newGene_107671	NA	NA	NA
Bos_taurus_newGene_107676	NA	NA	NA
Bos_taurus_newGene_107690	NA	NA	NA
Bos_taurus_newGene_107713	NA	NA	NA
Bos_taurus_newGene_107750	NA	NA	NA
Bos_taurus_newGene_107858	NA	NA	NA
Bos_taurus_newGene_107859	NA	NA	NA
Bos_taurus_newGene_107909	NA	NA	NA
Bos_taurus_newGene_107929	NA	NA	NA
Bos_taurus_newGene_107944	NA	NA	NA
Bos_taurus_newGene_107968	NA	NA	NA
Bos_taurus_newGene_107969	NA	NA	NA
Bos_taurus_newGene_107983	NA	NA	NA
Bos_taurus_newGene_107992	NA	NA	NA
Bos_taurus_newGene_108049	-0.082339	0.7911163	0.1017597
Bos_taurus_newGene_108076	NA	NA	NA
Bos_taurus_newGene_108089	NA	NA	NA
Bos_taurus_newGene_108135	NA	NA	NA
Bos_taurus_newGene_108138	NA	NA	NA
Bos_taurus_newGene_108154	NA	NA	NA
Bos_taurus_newGene_108181	0.0784529	0.8593683	0.0658207
Bos_taurus_newGene_108182	NA	NA	NA
Bos_taurus_newGene_108184	NA	NA	NA
Bos_taurus_newGene_108197	-0.103278	0.7872284	0.1038993
Bos_taurus_newGene_108198	NA	NA	NA
Bos_taurus_newGene_108199	9.8494486	4.59E-20	19.338443
Bos_taurus_newGene_108201	NA	NA	NA
Bos_taurus_newGene_108203	0.9066401	0.0331589	1.4794002
Bos_taurus_newGene_108217	NA	NA	NA
Bos_taurus_newGene_108230	NA	NA	NA
Bos_taurus_newGene_108256	NA	NA	NA
Bos_taurus_newGene_108258	NA	NA	NA
Bos_taurus_newGene_108272	NA	NA	NA
Bos_taurus_newGene_108294	-0.338995	0.439811	0.3567339
Bos_taurus_newGene_108340	0.2694076	0.480522	0.3182867
Bos_taurus_newGene_108347	NA	NA	NA
Bos_taurus_newGene_108359	0.4458437	0.3440548	0.4633724
Bos_taurus_newGene_108386	NA	NA	NA
Bos_taurus_newGene_108395	NA	NA	NA
Bos_taurus_newGene_108397	NA	NA	NA
Bos_taurus_newGene_108402	0.4391241	0.282929	0.5483226
Bos_taurus_newGene_108407	NA	NA	NA
Bos_taurus_newGene_108413	NA	NA	NA
Bos_taurus_newGene_108431	-0.115288	0.8339621	0.0788537
Bos_taurus_newGene_108433	NA	NA	NA
Bos_taurus_newGene_108475	0.2322621	0.5166153	0.2868327

Bos_taurus_newGene_10849(	NA	NA	NA
Bos_taurus_newGene_10851(	NA	NA	NA
Bos_taurus_newGene_10851(	NA	NA	NA
Bos_taurus_newGene_10852(	NA	NA	NA
Bos_taurus_newGene_10856(	NA	NA	NA
Bos_taurus_newGene_10860(	NA	NA	NA
Bos_taurus_newGene_10860(	0.8301552	0.3439998	0.4634419
Bos_taurus_newGene_10868(	NA	NA	NA
Bos_taurus_newGene_10868(	NA	NA	NA
Bos_taurus_newGene_10880(	NA	NA	NA
Bos_taurus_newGene_10888(	NA	NA	NA
Bos_taurus_newGene_10900(	NA	NA	NA
Bos_taurus_newGene_10900(	NA	NA	NA
Bos_taurus_newGene_10905(	NA	NA	NA
Bos_taurus_newGene_10906(	NA	NA	NA
Bos_taurus_newGene_10906(	0.2530787	0.6177889	0.2091599
Bos_taurus_newGene_10906(	NA	NA	NA
Bos_taurus_newGene_10910(	NA	NA	NA
Bos_taurus_newGene_10921(	NA	NA	NA
Bos_taurus_newGene_10922(	NA	NA	NA
Bos_taurus_newGene_10922(	NA	NA	NA
Bos_taurus_newGene_10924(	NA	NA	NA
Bos_taurus_newGene_10926(	NA	NA	NA
Bos_taurus_newGene_10928(	-0.160355	0.74179	0.1297191
Bos_taurus_newGene_10928(	-0.188782	0.8507189	0.0702139
Bos_taurus_newGene_10929(	NA	NA	NA
Bos_taurus_newGene_10935(	NA	NA	NA
Bos_taurus_newGene_10942(	NA	NA	NA
Bos_taurus_newGene_10954(	NA	NA	NA
Bos_taurus_newGene_10955(	NA	NA	NA
Bos_taurus_newGene_10956(	0.2311965	0.5325953	0.2736027
Bos_taurus_newGene_10957(	NA	NA	NA
Bos_taurus_newGene_10958(	NA	NA	NA
Bos_taurus_newGene_10960(	NA	NA	NA
Bos_taurus_newGene_10962(	NA	NA	NA
Bos_taurus_newGene_10963(	NA	NA	NA
Bos_taurus_newGene_10965(	0.4798501	0.2999797	0.5229082
Bos_taurus_newGene_10967(	NA	NA	NA
Bos_taurus_newGene_10967(	0.222516	0.7688527	0.1141568
Bos_taurus_newGene_10968(	0.11495	0.7821628	0.1067029
Bos_taurus_newGene_10968(	-0.253586	0.624922	0.2041742
Bos_taurus_newGene_10969(	0.2509229	0.4925356	0.3075624
Bos_taurus_newGene_10974(	NA	NA	NA
Bos_taurus_newGene_10976(	NA	NA	NA
Bos_taurus_newGene_10978(	NA	NA	NA
Bos_taurus_newGene_10985(	NA	NA	NA
Bos_taurus_newGene_10988(	-0.301808	0.69365	0.1588596
Bos_taurus_newGene_10999(	0.0202881	0.9543707	0.0202829
Bos_taurus_newGene_11001(	NA	NA	NA
Bos_taurus_newGene_11001(	NA	NA	NA
Bos_taurus_newGene_11004(	NA	NA	NA
Bos_taurus_newGene_11004(	NA	NA	NA
Bos_taurus_newGene_11010(	NA	NA	NA
Bos_taurus_newGene_11011(	NA	NA	NA
Bos_taurus_newGene_11016(	NA	NA	NA
Bos_taurus_newGene_11032(	0.8742681	0.1489332	0.8270085
Bos_taurus_newGene_11035(	NA	NA	NA
Bos_taurus_newGene_11035(	NA	NA	NA

3os_taurus_newGene_11035	NA	NA	NA
3os_taurus_newGene_11035	NA	NA	NA
3os_taurus_newGene_11039	NA	NA	NA
3os_taurus_newGene_11040	0.0151108	0.9848874	0.0066134
3os_taurus_newGene_11044	NA	NA	NA
3os_taurus_newGene_11046	NA	NA	NA
3os_taurus_newGene_11047	NA	NA	NA
3os_taurus_newGene_11048	NA	NA	NA
3os_taurus_newGene_11048	NA	NA	NA
3os_taurus_newGene_11048	-1.072282	0.0450941	1.3458801
3os_taurus_newGene_11050	NA	NA	NA
3os_taurus_newGene_11051	NA	NA	NA
3os_taurus_newGene_11055	0.1001807	0.8305826	0.0806172
3os_taurus_newGene_11060	NA	NA	NA
3os_taurus_newGene_11066	NA	NA	NA
3os_taurus_newGene_11068	0.176063	0.7708095	0.113053
3os_taurus_newGene_11069	-0.269155	0.4628758	0.3345355
3os_taurus_newGene_11079	NA	NA	NA
3os_taurus_newGene_11081	NA	NA	NA
3os_taurus_newGene_11084	NA	NA	NA
3os_taurus_newGene_11086	NA	NA	NA
3os_taurus_newGene_11090	NA	NA	NA
3os_taurus_newGene_11092	0.8398326	0.1589652	0.7986981
3os_taurus_newGene_11093	NA	NA	NA
3os_taurus_newGene_11093	NA	NA	NA
3os_taurus_newGene_11102	NA	NA	NA
3os_taurus_newGene_11105	-0.31629	0.3672948	0.4349852
3os_taurus_newGene_11107	NA	NA	NA
3os_taurus_newGene_11109	NA	NA	NA
3os_taurus_newGene_11115	NA	NA	NA
3os_taurus_newGene_11116	NA	NA	NA
3os_taurus_newGene_11116	NA	NA	NA
3os_taurus_newGene_11128	0.0602912	0.8453014	0.0729884
3os_taurus_newGene_11137	NA	NA	NA
3os_taurus_newGene_11144	NA	NA	NA
3os_taurus_newGene_11148	1.8874209	0.0028909	2.5389674
3os_taurus_newGene_11148	0.9838391	0.0712919	1.1469595
3os_taurus_newGene_11149	NA	NA	NA
3os_taurus_newGene_11150	NA	NA	NA
3os_taurus_newGene_11161	-0.162116	0.6514489	0.1861197
3os_taurus_newGene_11161	-0.058896	0.8954658	0.047951
3os_taurus_newGene_11161	NA	NA	NA
3os_taurus_newGene_11161	NA	NA	NA
3os_taurus_newGene_11162	NA	NA	NA
3os_taurus_newGene_11163	NA	NA	NA
3os_taurus_newGene_11165	-0.337984	0.3345437	0.4755471
3os_taurus_newGene_11187	NA	NA	NA
3os_taurus_newGene_11194	0.6362881	0.3472029	0.4594166
3os_taurus_newGene_11195	1.3204251	0.0034407	2.4633543
3os_taurus_newGene_11196	NA	NA	NA
3os_taurus_newGene_11196	NA	NA	NA
3os_taurus_newGene_11196	NA	NA	NA
3os_taurus_newGene_11199	NA	NA	NA
3os_taurus_newGene_11201	NA	NA	NA
3os_taurus_newGene_11216	NA	NA	NA
3os_taurus_newGene_11229	NA	NA	NA
3os_taurus_newGene_11237	NA	NA	NA
3os_taurus_newGene_11237	NA	NA	NA

Bos_taurus_newGene_11238	NA	NA	NA
Bos_taurus_newGene_11242	NA	NA	NA
Bos_taurus_newGene_11256	-1.194654	0.3232152	0.4905082
Bos_taurus_newGene_11256	NA	NA	NA
Bos_taurus_newGene_11259	NA	NA	NA
Bos_taurus_newGene_11260	NA	NA	NA
Bos_taurus_newGene_11260	-1.204373	0.1808165	0.7427619
Bos_taurus_newGene_11261	NA	NA	NA
Bos_taurus_newGene_11261	NA	NA	NA
Bos_taurus_newGene_11261	NA	NA	NA
Bos_taurus_newGene_11261	NA	NA	NA
Bos_taurus_newGene_11263	NA	NA	NA
Bos_taurus_newGene_11263	NA	NA	NA
Bos_taurus_newGene_11263	NA	NA	NA
Bos_taurus_newGene_11264	NA	NA	NA
Bos_taurus_newGene_11264	NA	NA	NA
Bos_taurus_newGene_11264	NA	NA	NA
Bos_taurus_newGene_11264	NA	NA	NA
Bos_taurus_newGene_11264	NA	NA	NA
Bos_taurus_newGene_11264	NA	NA	NA
Bos_taurus_newGene_11265	NA	NA	NA
Bos_taurus_newGene_11265	NA	NA	NA
Bos_taurus_newGene_11265	-0.68772	0.4248807	0.371733
Bos_taurus_newGene_11294	NA	NA	NA
Bos_taurus_newGene_11296	NA	NA	NA
Bos_taurus_newGene_11302	NA	NA	NA
Bos_taurus_newGene_11308	NA	NA	NA
Bos_taurus_newGene_11310	NA	NA	NA
Bos_taurus_newGene_11318	NA	NA	NA
Bos_taurus_newGene_11326	NA	NA	NA
Bos_taurus_newGene_11332	NA	NA	NA
Bos_taurus_newGene_11333	NA	NA	NA
Bos_taurus_newGene_11338	NA	NA	NA
Bos_taurus_newGene_11352	NA	NA	NA
Bos_taurus_newGene_11355	NA	NA	NA
Bos_taurus_newGene_11355	NA	NA	NA
Bos_taurus_newGene_11356	NA	NA	NA
Bos_taurus_newGene_11364	NA	NA	NA
Bos_taurus_newGene_11366	NA	NA	NA
Bos_taurus_newGene_11369	-0.473596	0.2264176	0.6450897
Bos_taurus_newGene_11372	NA	NA	NA
Bos_taurus_newGene_11372	NA	NA	NA
Bos_taurus_newGene_11372	NA	NA	NA
Bos_taurus_newGene_11374	NA	NA	NA
Bos_taurus_newGene_11374	NA	NA	NA
Bos_taurus_newGene_11375	-0.08641	0.8348154	0.0784095
Bos_taurus_newGene_11383	NA	NA	NA
Bos_taurus_newGene_11383	0.1272835	0.7829296	0.1062773
Bos_taurus_newGene_11386	0.615991	0.057589	1.2396607
Bos_taurus_newGene_11391	NA	NA	NA
Bos_taurus_newGene_11392	NA	NA	NA
Bos_taurus_newGene_11392	NA	NA	NA
Bos_taurus_newGene_11399	-0.27699	0.5513279	0.25859
Bos_taurus_newGene_11399	-0.241687	0.6792667	0.1679597
Bos_taurus_newGene_11403	NA	NA	NA
Bos_taurus_newGene_11403	NA	NA	NA
Bos_taurus_newGene_11412	NA	NA	NA
Bos_taurus_newGene_11412	NA	NA	NA

3os_taurus_newGene_11420(	NA	NA	NA
3os_taurus_newGene_11422	NA	NA	NA
3os_taurus_newGene_11423(	NA	NA	NA
3os_taurus_newGene_11423.	NA	NA	NA
3os_taurus_newGene_11433!	NA	NA	NA
3os_taurus_newGene_11438	0.6721063	0.1606301	0.7941732
3os_taurus_newGene_11438(	0.4046925	0.360968	0.4425313
3os_taurus_newGene_11438	NA	NA	NA
3os_taurus_newGene_11439(	-0.057705	0.9101515	0.0408863
3os_taurus_newGene_11439	0.2283009	0.6475166	0.1887491
3os_taurus_newGene_11440	NA	NA	NA
3os_taurus_newGene_11441	NA	NA	NA
3os_taurus_newGene_11442!	NA	NA	NA
3os_taurus_newGene_11442	NA	NA	NA
3os_taurus_newGene_11452:	NA	NA	NA
3os_taurus_newGene_11466.	NA	NA	NA
3os_taurus_newGene_11466!	NA	NA	NA
3os_taurus_newGene_11471	NA	NA	NA
3os_taurus_newGene_11471!	NA	NA	NA
3os_taurus_newGene_11472	NA	NA	NA
3os_taurus_newGene_11473!	NA	NA	NA
3os_taurus_newGene_11474	NA	NA	NA
3os_taurus_newGene_11474!	-0.147171	0.6483285	0.1882049
3os_taurus_newGene_11474	NA	NA	NA
3os_taurus_newGene_11474!	NA	NA	NA
3os_taurus_newGene_11475:	NA	NA	NA
3os_taurus_newGene_11475!	NA	NA	NA
3os_taurus_newGene_11479	NA	NA	NA
3os_taurus_newGene_11480	NA	NA	NA
3os_taurus_newGene_11487(	NA	NA	NA
3os_taurus_newGene_11487	NA	NA	NA
3os_taurus_newGene_11488	NA	NA	NA
3os_taurus_newGene_11488!	-0.648333	0.0297607	1.5263569
3os_taurus_newGene_11490!	0.9845731	0.0169131	1.7717767
3os_taurus_newGene_11493	-0.339874	0.2338635	0.6310376
3os_taurus_newGene_11493!	NA	NA	NA
3os_taurus_newGene_11494.	-0.243475	0.6377883	0.1953234
3os_taurus_newGene_11494	-0.27875	0.474473	0.3237885
3os_taurus_newGene_11494!	NA	NA	NA
3os_taurus_newGene_11495(	NA	NA	NA
3os_taurus_newGene_11495	NA	NA	NA
3os_taurus_newGene_11495!	-0.005246	1	0
3os_taurus_newGene_11505(	NA	NA	NA
3os_taurus_newGene_11505	NA	NA	NA
3os_taurus_newGene_11507	NA	NA	NA
3os_taurus_newGene_11510	NA	NA	NA
3os_taurus_newGene_11521!	-1.140106	0.0355052	1.4497086
3os_taurus_newGene_11521!	NA	NA	NA
3os_taurus_newGene_11522.	-1.064134	0.0007032	3.1529508
3os_taurus_newGene_11522!	NA	NA	NA
3os_taurus_newGene_11523(	-0.258451	0.5888641	0.2299849
3os_taurus_newGene_11527	NA	NA	NA
3os_taurus_newGene_11528:	NA	NA	NA
3os_taurus_newGene_11528	0.3281247	0.3853553	0.4141387
3os_taurus_newGene_11528(	-0.112844	0.8032246	0.095163
3os_taurus_newGene_11529!	0.3462466	0.286735	0.5425192
3os_taurus_newGene_11530!	-0.027952	0.9366926	0.0284029
3os_taurus_newGene_11531.	NA	NA	NA

Bos_taurus_newGene_11531	NA	NA	NA
Bos_taurus_newGene_11531	-0.059798	0.9142751	0.0389231
Bos_taurus_newGene_11533	-0.207162	0.5652951	0.2477248
Bos_taurus_newGene_11533	-0.551639	0.1950677	0.7098147
Bos_taurus_newGene_11538	NA	NA	NA
Bos_taurus_newGene_11542	NA	NA	NA
Bos_taurus_newGene_11543	NA	NA	NA
Bos_taurus_newGene_11546	0.310153	0.4857078	0.3136249
Bos_taurus_newGene_11553	NA	NA	NA
Bos_taurus_newGene_11560	-0.105656	0.8253022	0.083387
Bos_taurus_newGene_11560	NA	NA	NA
Bos_taurus_newGene_11570	NA	NA	NA
Bos_taurus_newGene_11577	NA	NA	NA
Bos_taurus_newGene_11591	NA	NA	NA
Bos_taurus_newGene_11603	NA	NA	NA
Bos_taurus_newGene_11615	NA	NA	NA
Bos_taurus_newGene_11616	NA	NA	NA
Bos_taurus_newGene_11618	NA	NA	NA
Bos_taurus_newGene_11618	NA	NA	NA
Bos_taurus_newGene_11618	NA	NA	NA
Bos_taurus_newGene_11621	NA	NA	NA
Bos_taurus_newGene_11636	-0.215849	0.7718084	0.1124905
Bos_taurus_newGene_11639	NA	NA	NA
Bos_taurus_newGene_11640	NA	NA	NA
Bos_taurus_newGene_11649	NA	NA	NA
Bos_taurus_newGene_11650	NA	NA	NA
Bos_taurus_newGene_11653	NA	NA	NA
Bos_taurus_newGene_11654	0.1533037	0.6789362	0.1681711
Bos_taurus_newGene_11654	NA	NA	NA
Bos_taurus_newGene_11656	NA	NA	NA
Bos_taurus_newGene_11658	NA	NA	NA
Bos_taurus_newGene_11660	NA	NA	NA
Bos_taurus_newGene_11662	NA	NA	NA
Bos_taurus_newGene_11664	-0.850134	0.0166547	1.7784632
Bos_taurus_newGene_11665	NA	NA	NA
Bos_taurus_newGene_11666	NA	NA	NA
Bos_taurus_newGene_11667	NA	NA	NA
Bos_taurus_newGene_11673	NA	NA	NA
Bos_taurus_newGene_11685	NA	NA	NA
Bos_taurus_newGene_11692	NA	NA	NA
Bos_taurus_newGene_11694	NA	NA	NA
Bos_taurus_newGene_11695	NA	NA	NA
Bos_taurus_newGene_11700	0.5550341	0.3638951	0.4390238
Bos_taurus_newGene_11716	-0.230483	0.5267768	0.2783734
Bos_taurus_newGene_11716	NA	NA	NA
Bos_taurus_newGene_11716	-0.320644	0.3771824	0.4234485
Bos_taurus_newGene_11717	NA	NA	NA
Bos_taurus_newGene_11717	NA	NA	NA
Bos_taurus_newGene_11717	NA	NA	NA
Bos_taurus_newGene_11718	NA	NA	NA
Bos_taurus_newGene_11719	NA	NA	NA
Bos_taurus_newGene_11720	NA	NA	NA
Bos_taurus_newGene_11720	-0.316501	0.6093715	0.2151179
Bos_taurus_newGene_11720	NA	NA	NA
Bos_taurus_newGene_11721	NA	NA	NA
Bos_taurus_newGene_11721	NA	NA	NA
Bos_taurus_newGene_11723	NA	NA	NA
Bos_taurus_newGene_11725	NA	NA	NA

Bos_taurus_newGene_11730	NA	NA	NA
Bos_taurus_newGene_11732	NA	NA	NA
Bos_taurus_newGene_11734	NA	NA	NA
Bos_taurus_newGene_11736	-0.002843	0.9942176	0.0025185
Bos_taurus_newGene_11736	-0.166854	0.6423866	0.1922035
Bos_taurus_newGene_11741	NA	NA	NA
Bos_taurus_newGene_11743	-0.141294	0.7379372	0.1319806
Bos_taurus_newGene_11743	-0.527289	0.2527267	0.5973489
Bos_taurus_newGene_11743	-0.216666	0.7031819	0.1529323
Bos_taurus_newGene_11743	0.2458144	0.6891316	0.1616979
Bos_taurus_newGene_11750	0.2221099	0.6176536	0.209255
Bos_taurus_newGene_11750	0.1340393	0.763997	0.1169084
Bos_taurus_newGene_11750	NA	NA	NA
Bos_taurus_newGene_11750	NA	NA	NA
Bos_taurus_newGene_11751	NA	NA	NA
Bos_taurus_newGene_11752	NA	NA	NA
Bos_taurus_newGene_11755	NA	NA	NA
Bos_taurus_newGene_11759	NA	NA	NA
Bos_taurus_newGene_11769	NA	NA	NA
Bos_taurus_newGene_11771	NA	NA	NA
Bos_taurus_newGene_11777	NA	NA	NA
Bos_taurus_newGene_11781	0.9040906	0.0378043	1.4224588
Bos_taurus_newGene_11783	NA	NA	NA
Bos_taurus_newGene_11784	NA	NA	NA
Bos_taurus_newGene_11785	0.4728729	0.3943053	0.4041674
Bos_taurus_newGene_11787	NA	NA	NA
Bos_taurus_newGene_11787	NA	NA	NA
Bos_taurus_newGene_11790	NA	NA	NA
Bos_taurus_newGene_11790	NA	NA	NA
Bos_taurus_newGene_11790	NA	NA	NA
Bos_taurus_newGene_11791	0.5998639	0.0710564	1.148397
Bos_taurus_newGene_11792	0.4973509	0.2676998	0.572352
Bos_taurus_newGene_11792	NA	NA	NA
Bos_taurus_newGene_11792	NA	NA	NA
Bos_taurus_newGene_11793	NA	NA	NA
Bos_taurus_newGene_11795	NA	NA	NA
Bos_taurus_newGene_11796	NA	NA	NA
Bos_taurus_newGene_11797	NA	NA	NA
Bos_taurus_newGene_11797	NA	NA	NA
Bos_taurus_newGene_11798	NA	NA	NA
Bos_taurus_newGene_11798	NA	NA	NA
Bos_taurus_newGene_11800	NA	NA	NA
Bos_taurus_newGene_11803	NA	NA	NA
Bos_taurus_newGene_11809	NA	NA	NA
Bos_taurus_newGene_11811	NA	NA	NA
Bos_taurus_newGene_11815	NA	NA	NA
Bos_taurus_newGene_11817	NA	NA	NA
Bos_taurus_newGene_11821	NA	NA	NA
Bos_taurus_newGene_11824	NA	NA	NA
Bos_taurus_newGene_11825	0.7281694	0.1848122	0.7332694
Bos_taurus_newGene_11825	NA	NA	NA
Bos_taurus_newGene_11830	NA	NA	NA
Bos_taurus_newGene_11840	NA	NA	NA
Bos_taurus_newGene_11844	NA	NA	NA
Bos_taurus_newGene_11848	NA	NA	NA
Bos_taurus_newGene_11854	0.2962851	0.5239886	0.2806781
Bos_taurus_newGene_11854	NA	NA	NA
Bos_taurus_newGene_11855	NA	NA	NA

Bos_taurus_newGene_11857	NA	NA	NA
Bos_taurus_newGene_11862	NA	NA	NA
Bos_taurus_newGene_11867	-0.402374	0.226678	0.6445906
Bos_taurus_newGene_11867	NA	NA	NA
Bos_taurus_newGene_11870	0.7417671	0.6897382	0.1613157
Bos_taurus_newGene_11870	NA	NA	NA
Bos_taurus_newGene_11880	-0.414711	0.3536467	0.4514304
Bos_taurus_newGene_11880	0.0735297	0.8954693	0.0479493
Bos_taurus_newGene_11881	NA	NA	NA
Bos_taurus_newGene_11892	NA	NA	NA
Bos_taurus_newGene_11895	NA	NA	NA
Bos_taurus_newGene_11895	-0.133742	0.8168037	0.0878823
Bos_taurus_newGene_11896	0.4077753	0.4205219	0.3762114
Bos_taurus_newGene_11896	0.0250253	0.9720174	0.012326
Bos_taurus_newGene_11896	-0.013637	0.9797298	0.0088937
Bos_taurus_newGene_11897	NA	NA	NA
Bos_taurus_newGene_11899	NA	NA	NA
Bos_taurus_newGene_11903	NA	NA	NA
Bos_taurus_newGene_11904	NA	NA	NA
Bos_taurus_newGene_11907	NA	NA	NA
Bos_taurus_newGene_11911	NA	NA	NA
Bos_taurus_newGene_11914	NA	NA	NA
Bos_taurus_newGene_11914	NA	NA	NA
Bos_taurus_newGene_11915	NA	NA	NA
Bos_taurus_newGene_11919	NA	NA	NA
Bos_taurus_newGene_11928	NA	NA	NA
Bos_taurus_newGene_11930	-0.296442	0.4004459	0.3974561
Bos_taurus_newGene_11941	-0.150527	0.7225096	0.1411564
Bos_taurus_newGene_11949	-0.100213	0.7125761	0.1471687
Bos_taurus_newGene_11950	NA	NA	NA
Bos_taurus_newGene_11951	-0.274253	0.5181229	0.2855672
Bos_taurus_newGene_11951	NA	NA	NA
Bos_taurus_newGene_11951	NA	NA	NA
Bos_taurus_newGene_11952	NA	NA	NA
Bos_taurus_newGene_11952	NA	NA	NA
Bos_taurus_newGene_11956	NA	NA	NA
Bos_taurus_newGene_11956	NA	NA	NA
Bos_taurus_newGene_11958	NA	NA	NA
Bos_taurus_newGene_11959	0.3562117	0.4543646	0.3425955
Bos_taurus_newGene_11963	NA	NA	NA
Bos_taurus_newGene_11963	NA	NA	NA
Bos_taurus_newGene_11964	NA	NA	NA
Bos_taurus_newGene_11964	NA	NA	NA
Bos_taurus_newGene_11965	-0.97858	0.1104885	0.9566827
Bos_taurus_newGene_11967	NA	NA	NA
Bos_taurus_newGene_11974	NA	NA	NA
Bos_taurus_newGene_11982	NA	NA	NA
Bos_taurus_newGene_11983	0.7645299	0.1712639	0.7663342
Bos_taurus_newGene_11983	NA	NA	NA
Bos_taurus_newGene_11983	0.4043462	0.3828642	0.4169553
Bos_taurus_newGene_11983	NA	NA	NA
Bos_taurus_newGene_11986	NA	NA	NA
Bos_taurus_newGene_11986	NA	NA	NA
Bos_taurus_newGene_11987	NA	NA	NA
Bos_taurus_newGene_11989	NA	NA	NA
Bos_taurus_newGene_12004	NA	NA	NA
Bos_taurus_newGene_12005	NA	NA	NA
Bos_taurus_newGene_12005	NA	NA	NA

3os_taurus_newGene_12005[illegible]	NA	NA	NA
3os_taurus_newGene_12006[illegible]	NA	NA	NA
3os_taurus_newGene_12006[illegible]	0.2620177	0.6489091	0.1878161
3os_taurus_newGene_12006[illegible]	NA	NA	NA
3os_taurus_newGene_12006[illegible]	NA	NA	NA
3os_taurus_newGene_12006[illegible]	NA	NA	NA
3os_taurus_newGene_12006[illegible]	NA	NA	NA
3os_taurus_newGene_12006[illegible]	NA	NA	NA
3os_taurus_newGene_12007[illegible]	NA	NA	NA
3os_taurus_newGene_12007[illegible]	NA	NA	NA
3os_taurus_newGene_12007[illegible]	NA	NA	NA
3os_taurus_newGene_12007[illegible]	0.0131753	0.9935172	0.0028246
3os_taurus_newGene_12007[illegible]	NA	NA	NA
3os_taurus_newGene_12007[illegible]	NA	NA	NA
3os_taurus_newGene_12007[illegible]	NA	NA	NA
3os_taurus_newGene_12008[illegible]	NA	NA	NA
3os_taurus_newGene_12008[illegible]	NA	NA	NA
3os_taurus_newGene_12008[illegible]	NA	NA	NA
3os_taurus_newGene_12008[illegible]	NA	NA	NA
3os_taurus_newGene_12009[illegible]	NA	NA	NA
3os_taurus_newGene_12010[illegible]	NA	NA	NA
3os_taurus_newGene_12011[illegible]	NA	NA	NA
3os_taurus_newGene_12012[illegible]	0.1549354	0.6388788	0.1945815
3os_taurus_newGene_12012[illegible]	NA	NA	NA
3os_taurus_newGene_12012[illegible]	NA	NA	NA
3os_taurus_newGene_12015[illegible]	NA	NA	NA
3os_taurus_newGene_12016[illegible]	-0.712633	0.1963959	0.7068675
3os_taurus_newGene_12017[illegible]	NA	NA	NA
3os_taurus_newGene_12017[illegible]	-0.118452	0.8507373	0.0702045
3os_taurus_newGene_12017[illegible]	NA	NA	NA
3os_taurus_newGene_12018[illegible]	NA	NA	NA
3os_taurus_newGene_12019[illegible]	-0.625722	0.2625629	0.5807667
3os_taurus_newGene_12019[illegible]	-1.076514	0.0030055	2.5220877
3os_taurus_newGene_12022[illegible]	NA	NA	NA
3os_taurus_newGene_12022[illegible]	NA	NA	NA
3os_taurus_newGene_12024[illegible]	-0.137758	0.730882	0.1361527
3os_taurus_newGene_12024[illegible]	NA	NA	NA
3os_taurus_newGene_12026[illegible]	NA	NA	NA
3os_taurus_newGene_12028[illegible]	0.1575576	0.638727	0.1946847
3os_taurus_newGene_12031[illegible]	NA	NA	NA
3os_taurus_newGene_12031[illegible]	NA	NA	NA
3os_taurus_newGene_12031[illegible]	NA	NA	NA
3os_taurus_newGene_12033[illegible]	NA	NA	NA
3os_taurus_newGene_12038[illegible]	NA	NA	NA
3os_taurus_newGene_12039[illegible]	0.1279778	0.8140625	0.0893423
3os_taurus_newGene_12041[illegible]	NA	NA	NA
3os_taurus_newGene_12041[illegible]	NA	NA	NA
3os_taurus_newGene_12052[illegible]	-1.390653	0.1336848	0.8739179
3os_taurus_newGene_12053[illegible]	NA	NA	NA
3os_taurus_newGene_12057[illegible]	NA	NA	NA
3os_taurus_newGene_12059[illegible]	NA	NA	NA
3os_taurus_newGene_12061[illegible]	NA	NA	NA
3os_taurus_newGene_12070[illegible]	NA	NA	NA
3os_taurus_newGene_12076[illegible]	NA	NA	NA
3os_taurus_newGene_12079[illegible]	NA	NA	NA
3os_taurus_newGene_12083[illegible]	NA	NA	NA
3os_taurus_newGene_12083[illegible]	NA	NA	NA
3os_taurus_newGene_12083[illegible]	NA	NA	NA

3os_taurus_newGene_12084(	NA	NA	NA
3os_taurus_newGene_12084:	NA	NA	NA
3os_taurus_newGene_12087!	NA	NA	NA
3os_taurus_newGene_12090:	NA	NA	NA
3os_taurus_newGene_12090	NA	NA	NA
3os_taurus_newGene_12090!	NA	NA	NA
3os_taurus_newGene_12091:	NA	NA	NA
3os_taurus_newGene_12093(	NA	NA	NA
3os_taurus_newGene_12094!	NA	NA	NA
3os_taurus_newGene_12096	NA	NA	NA
3os_taurus_newGene_12100(	NA	NA	NA
3os_taurus_newGene_12100	NA	NA	NA
3os_taurus_newGene_12100(	NA	NA	NA
3os_taurus_newGene_12101!	NA	NA	NA
3os_taurus_newGene_12102!	NA	NA	NA
3os_taurus_newGene_12103	NA	NA	NA
3os_taurus_newGene_12103	NA	NA	NA
3os_taurus_newGene_12103!	-0.903391	0.0419145	1.3776353
3os_taurus_newGene_12104	NA	NA	NA
3os_taurus_newGene_12105:	NA	NA	NA
3os_taurus_newGene_12106:	NA	NA	NA
3os_taurus_newGene_12109:	-0.353991	0.5096214	0.2927524
3os_taurus_newGene_12110:	NA	NA	NA
3os_taurus_newGene_12110(	NA	NA	NA
3os_taurus_newGene_12117:	NA	NA	NA
3os_taurus_newGene_12120	-0.519456	0.1608216	0.7936556
3os_taurus_newGene_12120!	NA	NA	NA
3os_taurus_newGene_12122:	-0.527395	0.1004032	0.9982526
3os_taurus_newGene_12122:	NA	NA	NA
3os_taurus_newGene_12132(	NA	NA	NA
3os_taurus_newGene_12134:	NA	NA	NA
3os_taurus_newGene_12136:	NA	NA	NA
3os_taurus_newGene_12140:	NA	NA	NA
3os_taurus_newGene_12140:	NA	NA	NA
3os_taurus_newGene_12145	NA	NA	NA
3os_taurus_newGene_12150	NA	NA	NA
3os_taurus_newGene_12150!	-0.296553	0.5119947	0.2907345
3os_taurus_newGene_12150(	NA	NA	NA
3os_taurus_newGene_12153(	NA	NA	NA
3os_taurus_newGene_12154:	NA	NA	NA
3os_taurus_newGene_12163!	NA	NA	NA
3os_taurus_newGene_12179!	NA	NA	NA
3os_taurus_newGene_12180:	NA	NA	NA
3os_taurus_newGene_12183!	NA	NA	NA
3os_taurus_newGene_12208:	NA	NA	NA
3os_taurus_newGene_12210:	NA	NA	NA
3os_taurus_newGene_12213:	NA	NA	NA
3os_taurus_newGene_12213	0.0506306	0.8793915	0.0558177
3os_taurus_newGene_12219!	NA	NA	NA
3os_taurus_newGene_12220!	NA	NA	NA
3os_taurus_newGene_12221!	NA	NA	NA
3os_taurus_newGene_12228!	NA	NA	NA
3os_taurus_newGene_12233:	NA	NA	NA
3os_taurus_newGene_12233:	NA	NA	NA
3os_taurus_newGene_12241!	-0.272046	0.5533531	0.2569976
3os_taurus_newGene_12242!	NA	NA	NA
3os_taurus_newGene_12243!	0.3539754	0.414149	0.3828434
3os_taurus_newGene_12243!	-0.223997	0.7170898	0.1444264

3os_taurus_newGene_12245!	NA	NA	NA
3os_taurus_newGene_12247!	NA	NA	NA
3os_taurus_newGene_12250	NA	NA	NA
3os_taurus_newGene_12250(	0.0548643	0.8726987	0.0591357
3os_taurus_newGene_12250	NA	NA	NA
3os_taurus_newGene_12252	NA	NA	NA
3os_taurus_newGene_12256	0.3030014	0.6010446	0.2210933
3os_taurus_newGene_12256!	NA	NA	NA
3os_taurus_newGene_12264!	NA	NA	NA
3os_taurus_newGene_12270	-0.318814	0.6285751	0.2016428
3os_taurus_newGene_12272!	NA	NA	NA
3os_taurus_newGene_12279	NA	NA	NA
3os_taurus_newGene_12280!	NA	NA	NA
3os_taurus_newGene_12287	0.623401	0.1507059	0.8218699
3os_taurus_newGene_12288	-0.098479	0.8378719	0.0768224
3os_taurus_newGene_12288(	1.7942169	0.2828767	0.5484029
3os_taurus_newGene_12288	-0.319206	0.8372245	0.0771581
3os_taurus_newGene_12289	NA	NA	NA
3os_taurus_newGene_12291	NA	NA	NA
3os_taurus_newGene_12292	NA	NA	NA
3os_taurus_newGene_12292(	NA	NA	NA
3os_taurus_newGene_12298(	NA	NA	NA
3os_taurus_newGene_12300	NA	NA	NA
3os_taurus_newGene_12300	NA	NA	NA
3os_taurus_newGene_12320(	0.4046706	0.7761265	0.1100675
3os_taurus_newGene_12325	NA	NA	NA
3os_taurus_newGene_12327	NA	NA	NA
3os_taurus_newGene_12328	NA	NA	NA
3os_taurus_newGene_12351!	0.2792108	0.4831942	0.3158783
3os_taurus_newGene_12355(	NA	NA	NA
3os_taurus_newGene_12366	-0.197085	0.657855	0.1818698
3os_taurus_newGene_12381	NA	NA	NA
3os_taurus_newGene_12382	0.7337281	0.115468	0.9375385
3os_taurus_newGene_12383(	NA	NA	NA
3os_taurus_newGene_12387	NA	NA	NA
3os_taurus_newGene_12391	NA	NA	NA
3os_taurus_newGene_12397	NA	NA	NA
3os_taurus_newGene_12398(	NA	NA	NA
3os_taurus_newGene_12398	0.1300912	0.8033894	0.0950739
3os_taurus_newGene_12400(	NA	NA	NA
3os_taurus_newGene_12404!	-0.418414	0.2923654	0.534074
3os_taurus_newGene_12405	NA	NA	NA
3os_taurus_newGene_12406(	NA	NA	NA
3os_taurus_newGene_12406(	NA	NA	NA
3os_taurus_newGene_12406	-0.548743	0.2161832	0.6651781
3os_taurus_newGene_12406!	-0.522911	0.3007655	0.521772
3os_taurus_newGene_12411(	NA	NA	NA
3os_taurus_newGene_12411	NA	NA	NA
3os_taurus_newGene_12418(	NA	NA	NA
3os_taurus_newGene_12421	-0.313747	0.2089286	0.6800021
3os_taurus_newGene_12421!	NA	NA	NA
3os_taurus_newGene_12422	-0.203306	0.7407865	0.130307
3os_taurus_newGene_12423	NA	NA	NA
3os_taurus_newGene_12423	NA	NA	NA
3os_taurus_newGene_12423	NA	NA	NA
3os_taurus_newGene_12423!	NA	NA	NA
3os_taurus_newGene_12424(	NA	NA	NA
3os_taurus_newGene_12424	NA	NA	NA

Bos_taurus_newGene_124250	-0.387388	0.4194453	0.3773247
Bos_taurus_newGene_124254	NA	NA	NA
Bos_taurus_newGene_124255	NA	NA	NA
Bos_taurus_newGene_124284	-0.326564	0.2239772	0.6497962
Bos_taurus_newGene_124291	NA	NA	NA
Bos_taurus_newGene_124340	NA	NA	NA
Bos_taurus_newGene_124362	0.2518815	0.6940193	0.1586284
Bos_taurus_newGene_124381	-0.361549	0.4065301	0.3909073
Bos_taurus_newGene_124492	NA	NA	NA
Bos_taurus_newGene_124554	-1.125698	0.0095147	2.0216071
Bos_taurus_newGene_124557	NA	NA	NA
Bos_taurus_newGene_124565	NA	NA	NA
Bos_taurus_newGene_124632	NA	NA	NA
Bos_taurus_newGene_124834	NA	NA	NA
Bos_taurus_newGene_124859	NA	NA	NA
Bos_taurus_newGene_124874	NA	NA	NA
Bos_taurus_newGene_124876	NA	NA	NA
Bos_taurus_newGene_124988	NA	NA	NA
Bos_taurus_newGene_124991	-1.644216	0.0057963	2.2368498
Bos_taurus_newGene_124994	NA	NA	NA
Bos_taurus_newGene_125019	NA	NA	NA
Bos_taurus_newGene_125029	NA	NA	NA
Bos_taurus_newGene_125031	NA	NA	NA
Bos_taurus_newGene_125037	NA	NA	NA
Bos_taurus_newGene_125041	NA	NA	NA
Bos_taurus_newGene_125113	NA	NA	NA
Bos_taurus_newGene_125129	0.0582636	0.9728076	0.011973
Bos_taurus_newGene_125145	NA	NA	NA
Bos_taurus_newGene_125158	NA	NA	NA
Bos_taurus_newGene_125162	NA	NA	NA
Bos_taurus_newGene_125163	NA	NA	NA
Bos_taurus_newGene_125314	NA	NA	NA
Bos_taurus_newGene_125337	NA	NA	NA
Bos_taurus_newGene_125460	NA	NA	NA
Bos_taurus_newGene_125472	NA	NA	NA
Bos_taurus_newGene_125516	NA	NA	NA
Bos_taurus_newGene_125553	NA	NA	NA
Bos_taurus_newGene_125554	NA	NA	NA
Bos_taurus_newGene_125559	NA	NA	NA
Bos_taurus_newGene_125698	0.1807408	0.5673836	0.2461232
Bos_taurus_newGene_125710	-0.027991	0.9601842	0.0176455
Bos_taurus_newGene_125711	-0.165076	0.7228273	0.1409655
Bos_taurus_newGene_125731	-0.646282	0.0337504	1.4717209
Bos_taurus_newGene_125753	NA	NA	NA
Bos_taurus_newGene_125754	NA	NA	NA
Bos_taurus_newGene_125807	NA	NA	NA
Bos_taurus_newGene_125927	NA	NA	NA
Bos_taurus_newGene_125959	NA	NA	NA
Bos_taurus_newGene_125983	NA	NA	NA
Bos_taurus_newGene_125993	NA	NA	NA
Bos_taurus_newGene_126004	-0.044847	0.9281241	0.032394
Bos_taurus_newGene_126118	1.4608434	0.0330658	1.480621
Bos_taurus_newGene_126176	NA	NA	NA
Bos_taurus_newGene_126199	NA	NA	NA
Bos_taurus_newGene_126224	NA	NA	NA
Bos_taurus_newGene_126226	-0.035908	0.9774359	0.0099117
Bos_taurus_newGene_126242	-0.622678	0.2384457	0.6226105
Bos_taurus_newGene_126244	NA	NA	NA

Bos_taurus_newGene_126247	0.0323245	0.956977	0.0190985
Bos_taurus_newGene_126253	NA	NA	NA
Bos_taurus_newGene_126256	NA	NA	NA
Bos_taurus_newGene_126269	NA	NA	NA
Bos_taurus_newGene_126281	NA	NA	NA
Bos_taurus_newGene_126317	NA	NA	NA
Bos_taurus_newGene_126319	NA	NA	NA
Bos_taurus_newGene_126334	NA	NA	NA
Bos_taurus_newGene_126337	NA	NA	NA
Bos_taurus_newGene_126339	-0.568826	0.281656	0.550281
Bos_taurus_newGene_126341	NA	NA	NA
Bos_taurus_newGene_126367	0.1483747	0.7531502	0.1231184
Bos_taurus_newGene_126402	-0.805589	0.0020227	2.6940675
Bos_taurus_newGene_126403	-1.690187	0.001177	2.9292387
Bos_taurus_newGene_126430	NA	NA	NA
Bos_taurus_newGene_126437	NA	NA	NA
Bos_taurus_newGene_126440	NA	NA	NA
Bos_taurus_newGene_126523	NA	NA	NA
Bos_taurus_newGene_126548	NA	NA	NA
Bos_taurus_newGene_126651	NA	NA	NA
Bos_taurus_newGene_126664	NA	NA	NA
Bos_taurus_newGene_126666	NA	NA	NA
Bos_taurus_newGene_126686	-0.066918	0.8748348	0.0580739
Bos_taurus_newGene_126742	NA	NA	NA
Bos_taurus_newGene_126799	NA	NA	NA
Bos_taurus_newGene_126801	NA	NA	NA
Bos_taurus_newGene_126804	NA	NA	NA
Bos_taurus_newGene_126837	NA	NA	NA
Bos_taurus_newGene_126991	NA	NA	NA
Bos_taurus_newGene_126992	NA	NA	NA
Bos_taurus_newGene_126993	NA	NA	NA
Bos_taurus_newGene_126994	NA	NA	NA
Bos_taurus_newGene_126996	NA	NA	NA
Bos_taurus_newGene_126999	NA	NA	NA
Bos_taurus_newGene_127001	NA	NA	NA
Bos_taurus_newGene_127138	NA	NA	NA
Bos_taurus_newGene_127140	-1.211214	0.0094706	2.0236222
Bos_taurus_newGene_127200	NA	NA	NA
Bos_taurus_newGene_127205	-0.606673	0.4065	0.3909394
Bos_taurus_newGene_127216	NA	NA	NA
Bos_taurus_newGene_127232	NA	NA	NA
Bos_taurus_newGene_127278	NA	NA	NA
Bos_taurus_newGene_127293	NA	NA	NA
Bos_taurus_newGene_127388	NA	NA	NA
Bos_taurus_newGene_127521	0.1080805	0.7972022	0.0984315
Bos_taurus_newGene_127531	NA	NA	NA
Bos_taurus_newGene_127532	-0.368511	0.31956	0.4954476
Bos_taurus_newGene_127534	NA	NA	NA
Bos_taurus_newGene_127571	NA	NA	NA
Bos_taurus_newGene_127606	NA	NA	NA
Bos_taurus_newGene_127616	-0.855391	0.1092165	0.9617117
Bos_taurus_newGene_127617	NA	NA	NA
Bos_taurus_newGene_127621	-0.596309	0.0255522	1.5925724
Bos_taurus_newGene_127626	NA	NA	NA
Bos_taurus_newGene_127655	NA	NA	NA
Bos_taurus_newGene_127664	NA	NA	NA
Bos_taurus_newGene_127665	0.0772642	0.8809143	0.0550663
Bos_taurus_newGene_127667	0.5741508	0.1995695	0.6999059

Bos_taurus_newGene_12767	0.6054966	0.1892574	0.7229472
Bos_taurus_newGene_12774	NA	NA	NA
Bos_taurus_newGene_12775	0.288339	0.587815	0.2307593
Bos_taurus_newGene_12775	NA	NA	NA
Bos_taurus_newGene_12782	NA	NA	NA
Bos_taurus_newGene_12785	0.2625798	0.3537566	0.4512954
Bos_taurus_newGene_12789	NA	NA	NA
Bos_taurus_newGene_12792	NA	NA	NA
Bos_taurus_newGene_12794	0.0560196	0.8948788	0.0482358
Bos_taurus_newGene_12795	-0.038899	0.9391792	0.0272515
Bos_taurus_newGene_12795	NA	NA	NA
Bos_taurus_newGene_12800	NA	NA	NA
Bos_taurus_newGene_12807	NA	NA	NA
Bos_taurus_newGene_12815	-0.47422	0.2683126	0.5713589
Bos_taurus_newGene_12816	NA	NA	NA
Bos_taurus_newGene_12819	NA	NA	NA
Bos_taurus_newGene_12822	1.2217531	0.2075274	0.6829245
Bos_taurus_newGene_12824	NA	NA	NA
Bos_taurus_newGene_12824	NA	NA	NA
Bos_taurus_newGene_12829	NA	NA	NA
Bos_taurus_newGene_12836	0.8974814	0.0166249	1.7792406
Bos_taurus_newGene_12838	NA	NA	NA
Bos_taurus_newGene_12839	-1.468863	0.0420795	1.375929
Bos_taurus_newGene_12844	NA	NA	NA
Bos_taurus_newGene_12845	NA	NA	NA
Bos_taurus_newGene_12846	-0.756134	0.5546238	0.2560015
Bos_taurus_newGene_12847	NA	NA	NA
Bos_taurus_newGene_12847	NA	NA	NA
Bos_taurus_newGene_12856	NA	NA	NA
Bos_taurus_newGene_12859	0.2703014	0.5526939	0.2575153
Bos_taurus_newGene_12863	NA	NA	NA
Bos_taurus_newGene_12867	0.4618996	0.3042506	0.5167685
Bos_taurus_newGene_12868	NA	NA	NA
Bos_taurus_newGene_12869	NA	NA	NA
Bos_taurus_newGene_12871	NA	NA	NA
Bos_taurus_newGene_12873	-0.541355	0.2930452	0.5330654
Bos_taurus_newGene_12873	NA	NA	NA
Bos_taurus_newGene_12873	0.0064966	0.9915315	0.0036935
Bos_taurus_newGene_12876	-0.376541	0.2678529	0.5721037
Bos_taurus_newGene_12886	NA	NA	NA
Bos_taurus_newGene_12892	-1.51355	0.1104826	0.956706
Bos_taurus_newGene_12893	NA	NA	NA
Bos_taurus_newGene_12893	NA	NA	NA
Bos_taurus_newGene_12894	NA	NA	NA
Bos_taurus_newGene_12894	0.117014	0.8216191	0.0853295
Bos_taurus_newGene_12900	NA	NA	NA
Bos_taurus_newGene_12901	NA	NA	NA
Bos_taurus_newGene_12901	NA	NA	NA
Bos_taurus_newGene_12907	NA	NA	NA
Bos_taurus_newGene_12907	NA	NA	NA
Bos_taurus_newGene_12931	0.0635672	0.9131824	0.0394425
Bos_taurus_newGene_12933	-0.821544	0.1089185	0.9628984
Bos_taurus_newGene_12934	NA	NA	NA
Bos_taurus_newGene_12938	0.3041373	0.7551069	0.1219916
Bos_taurus_newGene_12938	NA	NA	NA
Bos_taurus_newGene_12942	NA	NA	NA
Bos_taurus_newGene_12944	0.0228346	0.969421	0.0134876
Bos_taurus_newGene_12965	-0.001166	1	0

Bos_taurus_newGene_12980	0.3736731	0.387084	0.4121948
Bos_taurus_newGene_12987	NA	NA	NA
Bos_taurus_newGene_12990	NA	NA	NA
Bos_taurus_newGene_12990	0.9481965	0.0370854	1.4307976
Bos_taurus_newGene_12994	NA	NA	NA
Bos_taurus_newGene_12999	NA	NA	NA
Bos_taurus_newGene_13001	-0.091333	0.7975744	0.0982288
Bos_taurus_newGene_13003	NA	NA	NA
Bos_taurus_newGene_13004	NA	NA	NA
Bos_taurus_newGene_13004	NA	NA	NA
Bos_taurus_newGene_13004	NA	NA	NA
Bos_taurus_newGene_13009	-0.41409	0.4214786	0.3752245
Bos_taurus_newGene_13012	NA	NA	NA
Bos_taurus_newGene_13018	NA	NA	NA
Bos_taurus_newGene_13020	NA	NA	NA
Bos_taurus_newGene_13021	NA	NA	NA
Bos_taurus_newGene_13026	NA	NA	NA
Bos_taurus_newGene_13030	NA	NA	NA
Bos_taurus_newGene_13031	NA	NA	NA
Bos_taurus_newGene_13031	-0.064813	0.8921101	0.0495815
Bos_taurus_newGene_13031	0.3891352	0.3137742	0.5033828
Bos_taurus_newGene_13033	NA	NA	NA
Bos_taurus_newGene_13033	NA	NA	NA
Bos_taurus_newGene_13035	NA	NA	NA
Bos_taurus_newGene_13035	-0.583423	0.2857148	0.5440673
Bos_taurus_newGene_13036	-0.148579	0.5563152	0.2546791
Bos_taurus_newGene_13036	NA	NA	NA
Bos_taurus_newGene_13038	NA	NA	NA
Bos_taurus_newGene_13038	NA	NA	NA
Bos_taurus_newGene_13039	NA	NA	NA
Bos_taurus_newGene_13043	NA	NA	NA
Bos_taurus_newGene_13047	NA	NA	NA
Bos_taurus_newGene_13051	NA	NA	NA
Bos_taurus_newGene_13051	NA	NA	NA
Bos_taurus_newGene_13056	NA	NA	NA
Bos_taurus_newGene_13057	NA	NA	NA
Bos_taurus_newGene_13062	0.1565151	0.619328	0.2080793
Bos_taurus_newGene_13064	NA	NA	NA
Bos_taurus_newGene_13066	NA	NA	NA
Bos_taurus_newGene_13068	-0.187449	0.4775171	0.3210111
Bos_taurus_newGene_13071	NA	NA	NA
Bos_taurus_newGene_13073	1.3670579	0.0271913	1.5655692
Bos_taurus_newGene_13075	NA	NA	NA
Bos_taurus_newGene_13078	NA	NA	NA
Bos_taurus_newGene_13080	NA	NA	NA
Bos_taurus_newGene_13083	NA	NA	NA
Bos_taurus_newGene_13085	NA	NA	NA
Bos_taurus_newGene_13085	0.5941102	0.1866576	0.7289544
Bos_taurus_newGene_13086	NA	NA	NA
Bos_taurus_newGene_13093	0.8339369	0.070169	1.1538546
Bos_taurus_newGene_13095	-0.003692	1	0
Bos_taurus_newGene_13099	NA	NA	NA
Bos_taurus_newGene_13104	NA	NA	NA
Bos_taurus_newGene_13105	NA	NA	NA
Bos_taurus_newGene_13112	0.3331525	0.3729597	0.4283381
Bos_taurus_newGene_13113	NA	NA	NA
Bos_taurus_newGene_13114	NA	NA	NA
Bos_taurus_newGene_13116	NA	NA	NA

3os_taurus_newGene_13116[illegible]	NA	NA	NA
3os_taurus_newGene_13135[illegible]	-0.082625	0.8427719	0.07429
3os_taurus_newGene_13135[illegible]	NA	NA	NA
3os_taurus_newGene_13135[illegible]	0.3994299	0.4972293	0.3034433
3os_taurus_newGene_13137[illegible]	NA	NA	NA
3os_taurus_newGene_13138[illegible]	-0.67671	0.0558129	1.2532654
3os_taurus_newGene_13138[illegible]	NA	NA	NA
3os_taurus_newGene_13138[illegible]	-1.012969	0.0548938	1.2604763
3os_taurus_newGene_13139[illegible]	NA	NA	NA
3os_taurus_newGene_13142[illegible]	1.8782532	0.0002173	3.6629463
3os_taurus_newGene_13142[illegible]	0.6805295	0.2022053	0.6942074
3os_taurus_newGene_13164[illegible]	NA	NA	NA
3os_taurus_newGene_13164[illegible]	NA	NA	NA
3os_taurus_newGene_13164[illegible]	NA	NA	NA
3os_taurus_newGene_13165[illegible]	NA	NA	NA
3os_taurus_newGene_13165[illegible]	NA	NA	NA
3os_taurus_newGene_13165[illegible]	NA	NA	NA
3os_taurus_newGene_13170[illegible]	NA	NA	NA
3os_taurus_newGene_13171[illegible]	-0.060471	0.8847056	0.0532012
3os_taurus_newGene_13173[illegible]	1.1568373	0.0962319	1.0166809
3os_taurus_newGene_13173[illegible]	-0.242674	0.683643	0.1651707
3os_taurus_newGene_13176[illegible]	NA	NA	NA
3os_taurus_newGene_13180[illegible]	NA	NA	NA
3os_taurus_newGene_13184[illegible]	NA	NA	NA
3os_taurus_newGene_13194[illegible]	NA	NA	NA
3os_taurus_newGene_13196[illegible]	NA	NA	NA
3os_taurus_newGene_13198[illegible]	NA	NA	NA
3os_taurus_newGene_13202[illegible]	NA	NA	NA
3os_taurus_newGene_13207[illegible]	-0.047107	0.9237297	0.0344551
3os_taurus_newGene_13207[illegible]	-0.210028	0.6623917	0.1788851
3os_taurus_newGene_13208[illegible]	NA	NA	NA
3os_taurus_newGene_13210[illegible]	NA	NA	NA
3os_taurus_newGene_13210[illegible]	NA	NA	NA
3os_taurus_newGene_13211[illegible]	NA	NA	NA
3os_taurus_newGene_13213[illegible]	NA	NA	NA
3os_taurus_newGene_13221[illegible]	NA	NA	NA
3os_taurus_newGene_13225[illegible]	NA	NA	NA
3os_taurus_newGene_13226[illegible]	NA	NA	NA
3os_taurus_newGene_13234[illegible]	NA	NA	NA
3os_taurus_newGene_13234[illegible]	NA	NA	NA
3os_taurus_newGene_13239[illegible]	NA	NA	NA
3os_taurus_newGene_13241[illegible]	NA	NA	NA
3os_taurus_newGene_13244[illegible]	NA	NA	NA
3os_taurus_newGene_13248[illegible]	NA	NA	NA
3os_taurus_newGene_13260[illegible]	NA	NA	NA
3os_taurus_newGene_13263[illegible]	NA	NA	NA
3os_taurus_newGene_13265[illegible]	NA	NA	NA
3os_taurus_newGene_13270[illegible]	NA	NA	NA
3os_taurus_newGene_13271[illegible]	NA	NA	NA
3os_taurus_newGene_13280[illegible]	NA	NA	NA
3os_taurus_newGene_13282[illegible]	NA	NA	NA
3os_taurus_newGene_13287[illegible]	NA	NA	NA
3os_taurus_newGene_13290[illegible]	NA	NA	NA
3os_taurus_newGene_13300[illegible]	NA	NA	NA
3os_taurus_newGene_13301[illegible]	-0.218877	0.5798944	0.2366511
3os_taurus_newGene_13304[illegible]	NA	NA	NA
3os_taurus_newGene_13308[illegible]	NA	NA	NA
3os_taurus_newGene_13309[illegible]	0.4075758	0.3515901	0.4539633

Bos_taurus_newGene_13319	NA	NA	NA
Bos_taurus_newGene_13326	NA	NA	NA
Bos_taurus_newGene_13326	NA	NA	NA
Bos_taurus_newGene_13329	NA	NA	NA
Bos_taurus_newGene_13333	NA	NA	NA
Bos_taurus_newGene_13333	NA	NA	NA
Bos_taurus_newGene_13333	-0.402575	0.2250214	0.6477761
Bos_taurus_newGene_13334	-0.407426	0.3386996	0.4701853
Bos_taurus_newGene_13334	-0.294812	0.3426834	0.4651069
Bos_taurus_newGene_13335	NA	NA	NA
Bos_taurus_newGene_13340	0.3185636	0.5101374	0.2923128
Bos_taurus_newGene_13341	NA	NA	NA
Bos_taurus_newGene_13345	NA	NA	NA
Bos_taurus_newGene_13346	NA	NA	NA
Bos_taurus_newGene_13350	NA	NA	NA
Bos_taurus_newGene_13351	-0.338441	0.3931828	0.4054055
Bos_taurus_newGene_13351	NA	NA	NA
Bos_taurus_newGene_13352	NA	NA	NA
Bos_taurus_newGene_13353	NA	NA	NA
Bos_taurus_newGene_13354	NA	NA	NA
Bos_taurus_newGene_13356	-1.429163	3.49E-05	4.4577013
Bos_taurus_newGene_13356	NA	NA	NA
Bos_taurus_newGene_13359	NA	NA	NA
Bos_taurus_newGene_13364	NA	NA	NA
Bos_taurus_newGene_13367	-0.233865	0.505106	0.2966175
Bos_taurus_newGene_13368	NA	NA	NA
Bos_taurus_newGene_13368	NA	NA	NA
Bos_taurus_newGene_13368	NA	NA	NA
Bos_taurus_newGene_13369	NA	NA	NA
Bos_taurus_newGene_13370	-0.439902	0.2033141	0.6918325
Bos_taurus_newGene_13372	NA	NA	NA
Bos_taurus_newGene_13375	NA	NA	NA
Bos_taurus_newGene_13376	NA	NA	NA
Bos_taurus_newGene_13379	NA	NA	NA
Bos_taurus_newGene_13379	NA	NA	NA
Bos_taurus_newGene_13387	NA	NA	NA
Bos_taurus_newGene_13387	NA	NA	NA
Bos_taurus_newGene_13389	NA	NA	NA
Bos_taurus_newGene_13389	0.7636895	0.1240508	0.9064005
Bos_taurus_newGene_13392	NA	NA	NA
Bos_taurus_newGene_13393	-0.195206	0.6642355	0.1776779
Bos_taurus_newGene_13399	-0.035369	0.9415157	0.0261724
Bos_taurus_newGene_13399	NA	NA	NA
Bos_taurus_newGene_13400	0.5376212	0.35695	0.4473927
Bos_taurus_newGene_13403	-0.071505	0.8020507	0.0957982
Bos_taurus_newGene_13404	NA	NA	NA
Bos_taurus_newGene_13404	NA	NA	NA
Bos_taurus_newGene_13405	NA	NA	NA
Bos_taurus_newGene_13425	NA	NA	NA
Bos_taurus_newGene_13428	0.3990156	0.3785701	0.4218537
Bos_taurus_newGene_13428	NA	NA	NA
Bos_taurus_newGene_13429	-0.218925	0.7081169	0.149895
Bos_taurus_newGene_13429	-0.29388	0.6425892	0.1920666
Bos_taurus_newGene_13429	-0.346398	0.5763704	0.2392983
Bos_taurus_newGene_13430	0.1518732	0.7888976	0.1029794
Bos_taurus_newGene_13431	NA	NA	NA
Bos_taurus_newGene_13435	NA	NA	NA
Bos_taurus_newGene_13435	NA	NA	NA

3os_taurus_newGene_13436:	NA	NA	NA
3os_taurus_newGene_13438:	NA	NA	NA
3os_taurus_newGene_13439(	NA	NA	NA
3os_taurus_newGene_13440	-0.269664	0.4542025	0.3427505
3os_taurus_newGene_13440!	NA	NA	NA
3os_taurus_newGene_13441	NA	NA	NA
3os_taurus_newGene_13442:	NA	NA	NA
3os_taurus_newGene_13444:	NA	NA	NA
3os_taurus_newGene_13447	0.1892155	0.628593	0.2016304
3os_taurus_newGene_13447!	0.1399841	0.5923453	0.2274251
3os_taurus_newGene_13448(	NA	NA	NA
3os_taurus_newGene_13448:	0.007008	0.978722	0.0093406
3os_taurus_newGene_13448!	-1.732971	9.38E-05	4.0278166
3os_taurus_newGene_13448!	0.167716	0.814127	0.0893079
3os_taurus_newGene_13449(	NA	NA	NA
3os_taurus_newGene_13449!	NA	NA	NA
3os_taurus_newGene_13449!	NA	NA	NA
3os_taurus_newGene_13450:	NA	NA	NA
3os_taurus_newGene_13452:	0.0105936	0.9757757	0.01065
3os_taurus_newGene_13456!	NA	NA	NA
3os_taurus_newGene_13467:	-0.336333	0.5566101	0.2544489
3os_taurus_newGene_13467!	NA	NA	NA
3os_taurus_newGene_13470(	NA	NA	NA
3os_taurus_newGene_13472(	NA	NA	NA
3os_taurus_newGene_13475!	NA	NA	NA
3os_taurus_newGene_13476(	NA	NA	NA
3os_taurus_newGene_13476(	NA	NA	NA
3os_taurus_newGene_13476	1.1766285	0.2075348	0.682909
3os_taurus_newGene_13477	NA	NA	NA
3os_taurus_newGene_13477!	NA	NA	NA
3os_taurus_newGene_13477(	NA	NA	NA
3os_taurus_newGene_13489!	NA	NA	NA
3os_taurus_newGene_13490:	NA	NA	NA
3os_taurus_newGene_13490	-0.559779	0.2557166	0.592241
3os_taurus_newGene_13496(	NA	NA	NA
3os_taurus_newGene_13499(	0.285265	0.5365328	0.2704037
3os_taurus_newGene_13501:	0.0097763	0.9924109	0.0033085
3os_taurus_newGene_13501:	0.2251152	0.6461777	0.189648
3os_taurus_newGene_13501	NA	NA	NA
3os_taurus_newGene_13505:	NA	NA	NA
3os_taurus_newGene_13505	NA	NA	NA
3os_taurus_newGene_13509!	-0.27796	0.4489625	0.3477899
3os_taurus_newGene_13510:	NA	NA	NA
3os_taurus_newGene_13510!	0.6685014	0.4430997	0.3534986
3os_taurus_newGene_13511:	-0.088084	0.8181707	0.0871561
3os_taurus_newGene_13512!	NA	NA	NA
3os_taurus_newGene_13513(	NA	NA	NA
3os_taurus_newGene_13514:	-0.361285	0.4415048	0.3550646
3os_taurus_newGene_13514!	NA	NA	NA
3os_taurus_newGene_13515(	NA	NA	NA
3os_taurus_newGene_13515!	NA	NA	NA
3os_taurus_newGene_13519(	NA	NA	NA
3os_taurus_newGene_13522	NA	NA	NA
3os_taurus_newGene_13522!	NA	NA	NA
3os_taurus_newGene_13533:	NA	NA	NA
3os_taurus_newGene_13533:	NA	NA	NA
3os_taurus_newGene_13534(	-0.062557	0.8719657	0.0595006
3os_taurus_newGene_13535(	-0.454955	0.3285225	0.4834349

Bos_taurus_newGene_135359	NA	NA	NA
Bos_taurus_newGene_135361	NA	NA	NA
Bos_taurus_newGene_135368	NA	NA	NA
Bos_taurus_newGene_135399	NA	NA	NA
Bos_taurus_newGene_135440	NA	NA	NA
Bos_taurus_newGene_135447	NA	NA	NA
Bos_taurus_newGene_135473	NA	NA	NA
Bos_taurus_newGene_135482	NA	NA	NA
Bos_taurus_newGene_135499	NA	NA	NA
Bos_taurus_newGene_135506	NA	NA	NA
Bos_taurus_newGene_135510	NA	NA	NA
Bos_taurus_newGene_135549	0.3124687	0.4737109	0.3244867
Bos_taurus_newGene_135552	NA	NA	NA
Bos_taurus_newGene_135558	NA	NA	NA
Bos_taurus_newGene_135585	NA	NA	NA
Bos_taurus_newGene_135587	-0.04819	0.9093039	0.041291
Bos_taurus_newGene_135651	NA	NA	NA
Bos_taurus_newGene_135659	0.1633621	0.6781733	0.1686593
Bos_taurus_newGene_135674	NA	NA	NA
Bos_taurus_newGene_135694	-0.581004	0.1047486	0.9798518
Bos_taurus_newGene_135718	-0.623857	0.0529456	1.2761699
Bos_taurus_newGene_135743	NA	NA	NA
Bos_taurus_newGene_135764	0.0409032	0.9327462	0.0302365
Bos_taurus_newGene_135786	-0.329806	0.520677	0.2834316
Bos_taurus_newGene_135793	NA	NA	NA
Bos_taurus_newGene_135808	NA	NA	NA
Bos_taurus_newGene_135831	NA	NA	NA
Bos_taurus_newGene_135841	0.3557354	0.4350218	0.361489
Bos_taurus_newGene_135843	NA	NA	NA
Bos_taurus_newGene_135864	0.0685735	0.9134989	0.039292
Bos_taurus_newGene_135892	NA	NA	NA
Bos_taurus_newGene_135953	0.4412	0.3505197	0.4552875
Bos_taurus_newGene_135954	NA	NA	NA
Bos_taurus_newGene_136022	NA	NA	NA
Bos_taurus_newGene_136052	NA	NA	NA
Bos_taurus_newGene_136054	NA	NA	NA
Bos_taurus_newGene_136076	NA	NA	NA
Bos_taurus_newGene_136077	-0.586001	0.1383648	0.8589744
Bos_taurus_newGene_136081	NA	NA	NA
Bos_taurus_newGene_136087	NA	NA	NA
Bos_taurus_newGene_136094	NA	NA	NA
Bos_taurus_newGene_136095	NA	NA	NA
Bos_taurus_newGene_136097	NA	NA	NA
Bos_taurus_newGene_136125	NA	NA	NA
Bos_taurus_newGene_136162	-0.170496	0.7154463	0.1454229
Bos_taurus_newGene_136164	NA	NA	NA
Bos_taurus_newGene_136211	-0.476902	0.3139002	0.5032085
Bos_taurus_newGene_136237	0.399259	0.3710659	0.4305489
Bos_taurus_newGene_136242	NA	NA	NA
Bos_taurus_newGene_136244	1.0950259	0.0484889	1.3143577
Bos_taurus_newGene_136246	NA	NA	NA
Bos_taurus_newGene_136255	-0.397122	0.3837988	0.4158964
Bos_taurus_newGene_136297	NA	NA	NA
Bos_taurus_newGene_136340	NA	NA	NA
Bos_taurus_newGene_136400	NA	NA	NA
Bos_taurus_newGene_136435	NA	NA	NA
Bos_taurus_newGene_136465	NA	NA	NA
Bos_taurus_newGene_136472	-0.225129	0.6200703	0.2075591

Bos_taurus_newGene_136474	-1.371682	0.0087551	2.0577386
Bos_taurus_newGene_136476	-0.241162	0.6917414	0.1600562
Bos_taurus_newGene_136525	NA	NA	NA
Bos_taurus_newGene_136532	NA	NA	NA
Bos_taurus_newGene_136537	-0.69997	0.0214264	1.6690503
Bos_taurus_newGene_136538	-0.141312	0.7397774	0.1308989
Bos_taurus_newGene_136573	-0.546257	0.4660947	0.3315259
Bos_taurus_newGene_136575	-1.342258	0.0237961	1.623495
Bos_taurus_newGene_136578	-1.173509	0.0215989	1.6655681
Bos_taurus_newGene_136580	-0.359713	0.5508704	0.2589505
Bos_taurus_newGene_136584	-0.239727	0.64186	0.1925597
Bos_taurus_newGene_136606	NA	NA	NA
Bos_taurus_newGene_136686	-0.003527	0.9946031	0.0023502
Bos_taurus_newGene_136690	0.0794471	0.9162737	0.0379748
Bos_taurus_newGene_136696	NA	NA	NA
Bos_taurus_newGene_136707	NA	NA	NA
Bos_taurus_newGene_136715	NA	NA	NA
Bos_taurus_newGene_136717	NA	NA	NA
Bos_taurus_newGene_136718	NA	NA	NA
Bos_taurus_newGene_136731	NA	NA	NA
Bos_taurus_newGene_136745	NA	NA	NA
Bos_taurus_newGene_136753	NA	NA	NA
Bos_taurus_newGene_136774	NA	NA	NA
Bos_taurus_newGene_136778	NA	NA	NA
Bos_taurus_newGene_136790	NA	NA	NA
Bos_taurus_newGene_136801	NA	NA	NA
Bos_taurus_newGene_136802	0.264251	0.5615631	0.2506014
Bos_taurus_newGene_136847	NA	NA	NA
Bos_taurus_newGene_136852	NA	NA	NA
Bos_taurus_newGene_136854	0.1664191	0.615723	0.2106146
Bos_taurus_newGene_136857	NA	NA	NA
Bos_taurus_newGene_136877	NA	NA	NA
Bos_taurus_newGene_136972	NA	NA	NA
Bos_taurus_newGene_136996	-0.474516	0.3230131	0.4907799
Bos_taurus_newGene_137000	-1.954452	0.0048862	2.3110265
Bos_taurus_newGene_137015	NA	NA	NA
Bos_taurus_newGene_137019	NA	NA	NA
Bos_taurus_newGene_137026	NA	NA	NA
Bos_taurus_newGene_137030	0.1967082	0.7269643	0.1384869
Bos_taurus_newGene_137113	NA	NA	NA
Bos_taurus_newGene_137122	NA	NA	NA
Bos_taurus_newGene_137150	-0.785312	0.0271127	1.566827
Bos_taurus_newGene_137201	NA	NA	NA
Bos_taurus_newGene_137206	NA	NA	NA
Bos_taurus_newGene_137245	NA	NA	NA
Bos_taurus_newGene_137246	NA	NA	NA
Bos_taurus_newGene_137247	NA	NA	NA
Bos_taurus_newGene_137277	-0.016393	0.9638684	0.0159823
Bos_taurus_newGene_137311	NA	NA	NA
Bos_taurus_newGene_137453	NA	NA	NA
Bos_taurus_newGene_137616	NA	NA	NA
Bos_taurus_newGene_137730	NA	NA	NA
Bos_taurus_newGene_137822	NA	NA	NA
Bos_taurus_newGene_137858	NA	NA	NA
Bos_taurus_newGene_138049	NA	NA	NA
Bos_taurus_newGene_138176	NA	NA	NA
Bos_taurus_newGene_138193	NA	NA	NA
Bos_taurus_newGene_138212	-0.060423	0.9121277	0.0399443

Bos_taurus_newGene_13821	NA	NA	NA
Bos_taurus_newGene_13821	NA	NA	NA
Bos_taurus_newGene_13822	NA	NA	NA
Bos_taurus_newGene_13822	NA	NA	NA
Bos_taurus_newGene_13830	NA	NA	NA
Bos_taurus_newGene_13834	NA	NA	NA
Bos_taurus_newGene_13834	NA	NA	NA
Bos_taurus_newGene_13836	-0.444076	0.0790948	1.101852
Bos_taurus_newGene_13836	NA	NA	NA
Bos_taurus_newGene_13837	NA	NA	NA
Bos_taurus_newGene_13837	NA	NA	NA
Bos_taurus_newGene_13837	-0.444506	0.0728709	1.1374459
Bos_taurus_newGene_13837	NA	NA	NA
Bos_taurus_newGene_13848	NA	NA	NA
Bos_taurus_newGene_13848	NA	NA	NA
Bos_taurus_newGene_13851	NA	NA	NA
Bos_taurus_newGene_13851	-1.192496	0.0581778	1.2352424
Bos_taurus_newGene_13851	NA	NA	NA
Bos_taurus_newGene_13852	-0.571191	0.2012672	0.696227
Bos_taurus_newGene_13854	-0.127178	0.7372182	0.1324039
Bos_taurus_newGene_13868	NA	NA	NA
Bos_taurus_newGene_13869	NA	NA	NA
Bos_taurus_newGene_13869	NA	NA	NA
Bos_taurus_newGene_13874	-0.014172	0.9693691	0.0135108
Bos_taurus_newGene_13874	-0.227852	0.7726269	0.1120302
Bos_taurus_newGene_13876	NA	NA	NA
Bos_taurus_newGene_13881	NA	NA	NA
Bos_taurus_newGene_13882	NA	NA	NA
Bos_taurus_newGene_13883	NA	NA	NA
Bos_taurus_newGene_13889	-0.148396	0.5556153	0.2552258
Bos_taurus_newGene_13889	NA	NA	NA
Bos_taurus_newGene_13891	NA	NA	NA
Bos_taurus_newGene_13900	-1.934032	0.233223	0.6322285
Bos_taurus_newGene_13904	NA	NA	NA
Bos_taurus_newGene_13914	NA	NA	NA
Bos_taurus_newGene_13919	NA	NA	NA
Bos_taurus_newGene_13922	NA	NA	NA
Bos_taurus_newGene_13923	NA	NA	NA
Bos_taurus_newGene_13923	NA	NA	NA
Bos_taurus_newGene_13924	NA	NA	NA
Bos_taurus_newGene_13924	0.5168225	0.307035	0.5128121
Bos_taurus_newGene_13926	0.2399357	0.6158614	0.210517
Bos_taurus_newGene_13926	NA	NA	NA
Bos_taurus_newGene_13931	NA	NA	NA
Bos_taurus_newGene_13932	0.8983314	0.3372984	0.4719857
Bos_taurus_newGene_13932	NA	NA	NA
Bos_taurus_newGene_13932	0.5263818	0.2825275	0.5489392
Bos_taurus_newGene_13933	0.6553953	0.1830285	0.7374813
Bos_taurus_newGene_13933	0.2668866	0.7032885	0.1528665
Bos_taurus_newGene_13934	-0.770365	0.0980983	1.0083386
Bos_taurus_newGene_13942	-0.278733	0.4824586	0.3165399
Bos_taurus_newGene_13942	-0.055985	0.9592803	0.0180545
Bos_taurus_newGene_13943	0.0444997	0.8868297	0.0521598
Bos_taurus_newGene_13944	NA	NA	NA
Bos_taurus_newGene_13945	-2.221341	0.0059183	2.2278047
Bos_taurus_newGene_13946	-0.15232	0.7266742	0.1386603
Bos_taurus_newGene_13947	NA	NA	NA
Bos_taurus_newGene_13948	NA	NA	NA

3os_taurus_newGene_13948(	NA	NA	NA
3os_taurus_newGene_13951⁄	0.465667	0.1646448	0.783452
3os_taurus_newGene_13951{	NA	NA	NA
3os_taurus_newGene_13953!	NA	NA	NA
3os_taurus_newGene_13953{	NA	NA	NA
3os_taurus_newGene_13954:	0.6443669	0.0300099	1.5227348
3os_taurus_newGene_13957!	0.8111641	0.3372872	0.4720002
3os_taurus_newGene_13958(	2.4138433	0.0075755	2.1205874
3os_taurus_newGene_13959⁄	NA	NA	NA
3os_taurus_newGene_13965:	NA	NA	NA
3os_taurus_newGene_13965(	NA	NA	NA
3os_taurus_newGene_13971(	NA	NA	NA
3os_taurus_newGene_13972!	NA	NA	NA
3os_taurus_newGene_13974!	0.1503303	0.6021983	0.2202605
3os_taurus_newGene_13975!	NA	NA	NA
3os_taurus_newGene_13975!	NA	NA	NA
3os_taurus_newGene_13976(	0.1663908	0.7280877	0.1378163
3os_taurus_newGene_13977!	-0.564442	0.1719408	0.7646211
3os_taurus_newGene_13977(	NA	NA	NA
3os_taurus_newGene_13982{	NA	NA	NA
3os_taurus_newGene_13983:	NA	NA	NA
3os_taurus_newGene_13995:	NA	NA	NA
3os_taurus_newGene_13998:	-0.251301	0.4908591	0.3090431
3os_taurus_newGene_13998:	NA	NA	NA
3os_taurus_newGene_13998!	NA	NA	NA
3os_taurus_newGene_14001!	NA	NA	NA
3os_taurus_newGene_14001{	NA	NA	NA
3os_taurus_newGene_14007	-0.054991	0.9316768	0.0307347
3os_taurus_newGene_14007{	1.191179	0.001409	2.8510828
3os_taurus_newGene_14010!	NA	NA	NA
3os_taurus_newGene_14013:	-0.37284	0.4799156	0.3188351
3os_taurus_newGene_14014:	NA	NA	NA
3os_taurus_newGene_14016(	0.4310189	0.195096	0.7097516
3os_taurus_newGene_14017	NA	NA	NA
3os_taurus_newGene_14018:	NA	NA	NA
3os_taurus_newGene_14022:	NA	NA	NA
3os_taurus_newGene_14038⁄	NA	NA	NA
3os_taurus_newGene_14039!	NA	NA	NA
3os_taurus_newGene_14039	NA	NA	NA
3os_taurus_newGene_14041(	NA	NA	NA
3os_taurus_newGene_14042:	0.4490551	0.3862666	0.4131128
3os_taurus_newGene_14042⁄	-0.021655	0.9911771	0.0038487
3os_taurus_newGene_14042!	-0.187991	0.6269093	0.2027953
3os_taurus_newGene_14043:	NA	NA	NA
3os_taurus_newGene_14049!	NA	NA	NA
3os_taurus_newGene_14053⁄	NA	NA	NA
3os_taurus_newGene_14053!	NA	NA	NA
3os_taurus_newGene_14061{	NA	NA	NA
3os_taurus_newGene_14062!	-0.247632	0.3747219	0.426291
3os_taurus_newGene_14069:	-1.005087	0.0455935	1.3410974
3os_taurus_newGene_14075{	0.3294051	0.3520588	0.4533848
3os_taurus_newGene_14076:	NA	NA	NA
3os_taurus_newGene_14083(	0.4045104	0.4206156	0.3761146
3os_taurus_newGene_14085	NA	NA	NA
3os_taurus_newGene_14086⁄	NA	NA	NA
3os_taurus_newGene_14088:	-0.315539	0.6101656	0.2145523
3os_taurus_newGene_14088(	0.4031772	0.6271008	0.2026626
3os_taurus_newGene_14089:	0.7444929	0.0739973	1.130784

Bos_taurus_newGene_14089	NA	NA	NA
Bos_taurus_newGene_14091	NA	NA	NA
Bos_taurus_newGene_14093	NA	NA	NA
Bos_taurus_newGene_14095	NA	NA	NA
Bos_taurus_newGene_14096	NA	NA	NA
Bos_taurus_newGene_14098	NA	NA	NA
Bos_taurus_newGene_14107	0.9540424	0.0085206	2.0695318
Bos_taurus_newGene_14108	NA	NA	NA
Bos_taurus_newGene_14109	NA	NA	NA
Bos_taurus_newGene_14110	0.6438549	0.2152707	0.667015
Bos_taurus_newGene_14111	NA	NA	NA
Bos_taurus_newGene_14111	NA	NA	NA
Bos_taurus_newGene_14112	NA	NA	NA
Bos_taurus_newGene_14120	NA	NA	NA
Bos_taurus_newGene_14128	NA	NA	NA
Bos_taurus_newGene_14130	0.8720311	0.0011986	2.9213312
Bos_taurus_newGene_14143	NA	NA	NA
Bos_taurus_newGene_14144	NA	NA	NA
Bos_taurus_newGene_14164	-1.856199	0.0672966	1.1720068
Bos_taurus_newGene_14164	-0.468862	0.2689978	0.5702512
Bos_taurus_newGene_14164	0.1934874	0.7355746	0.1333733
Bos_taurus_newGene_14167	NA	NA	NA
Bos_taurus_newGene_14168	0.0276244	0.9338561	0.0297201
Bos_taurus_newGene_14169	0.5536664	0.283934	0.5467827
Bos_taurus_newGene_14169	NA	NA	NA
Bos_taurus_newGene_14170	NA	NA	NA
Bos_taurus_newGene_14172	-0.106267	0.7552846	0.1218894
Bos_taurus_newGene_14174	NA	NA	NA
Bos_taurus_newGene_14174	NA	NA	NA
Bos_taurus_newGene_14174	-0.087134	0.892996	0.0491505
Bos_taurus_newGene_14174	0.1161023	0.8185375	0.0869614
Bos_taurus_newGene_14175	NA	NA	NA
Bos_taurus_newGene_14176	NA	NA	NA
Bos_taurus_newGene_14176	NA	NA	NA
Bos_taurus_newGene_14176	NA	NA	NA
Bos_taurus_newGene_14176	NA	NA	NA
Bos_taurus_newGene_14177	NA	NA	NA
Bos_taurus_newGene_14177	NA	NA	NA
Bos_taurus_newGene_14177	NA	NA	NA
Bos_taurus_newGene_14177	NA	NA	NA
Bos_taurus_newGene_14177	NA	NA	NA
Bos_taurus_newGene_14178	-1.051776	0.0664095	1.1777696
Bos_taurus_newGene_14179	-0.059829	0.9014419	0.0450623
Bos_taurus_newGene_14183	NA	NA	NA
Bos_taurus_newGene_14187	-0.49369	0.323106	0.4906549
Bos_taurus_newGene_14188	0.3263108	0.3612959	0.4421369
Bos_taurus_newGene_14190	NA	NA	NA
Bos_taurus_newGene_14198	NA	NA	NA
Bos_taurus_newGene_14198	NA	NA	NA
Bos_taurus_newGene_14198	NA	NA	NA
Bos_taurus_newGene_14204	NA	NA	NA
Bos_taurus_newGene_14205	0.1084025	0.7666561	0.1153994
Bos_taurus_newGene_14205	NA	NA	NA
Bos_taurus_newGene_14207	NA	NA	NA
Bos_taurus_newGene_14210	NA	NA	NA
Bos_taurus_newGene_14216	NA	NA	NA
Bos_taurus_newGene_14218	0.135398	0.7662733	0.1156163
Bos_taurus_newGene_14218	NA	NA	NA

Bos_taurus_newGene_14222	0.218687	0.6486274	0.1880047
Bos_taurus_newGene_14233	NA	NA	NA
Bos_taurus_newGene_14234	NA	NA	NA
Bos_taurus_newGene_14235	-0.041394	0.9473554	0.0234871
Bos_taurus_newGene_14238	NA	NA	NA
Bos_taurus_newGene_14247	0.6920773	0.0547697	1.2614594
Bos_taurus_newGene_14256	0.0331685	0.9367112	0.0283943
Bos_taurus_newGene_14258	NA	NA	NA
Bos_taurus_newGene_14263	NA	NA	NA
Bos_taurus_newGene_14267	NA	NA	NA
Bos_taurus_newGene_14271	NA	NA	NA
Bos_taurus_newGene_14274	NA	NA	NA
Bos_taurus_newGene_14278	NA	NA	NA
Bos_taurus_newGene_14278	NA	NA	NA
Bos_taurus_newGene_14282	-0.179758	0.701467	0.1539928
Bos_taurus_newGene_14287	NA	NA	NA
Bos_taurus_newGene_14287	NA	NA	NA
Bos_taurus_newGene_14287	NA	NA	NA
Bos_taurus_newGene_14294	NA	NA	NA
Bos_taurus_newGene_14298	NA	NA	NA
Bos_taurus_newGene_14298	0.2791509	0.5934764	0.2265965
Bos_taurus_newGene_14299	NA	NA	NA
Bos_taurus_newGene_14300	NA	NA	NA
Bos_taurus_newGene_14302	NA	NA	NA
Bos_taurus_newGene_14303	NA	NA	NA
Bos_taurus_newGene_14306	-0.675701	0.0652419	1.1854736
Bos_taurus_newGene_14310	NA	NA	NA
Bos_taurus_newGene_14312	-0.711913	0.025748	1.5892572
Bos_taurus_newGene_14313	-0.742322	0.0930838	1.0311261
Bos_taurus_newGene_14321	NA	NA	NA
Bos_taurus_newGene_14321	NA	NA	NA
Bos_taurus_newGene_14325	NA	NA	NA
Bos_taurus_newGene_14326	NA	NA	NA
Bos_taurus_newGene_14337	NA	NA	NA
Bos_taurus_newGene_14359	NA	NA	NA
Bos_taurus_newGene_14359	NA	NA	NA
Bos_taurus_newGene_14359	NA	NA	NA
Bos_taurus_newGene_14362	1.6274842	0.0358436	1.4455886
Bos_taurus_newGene_14364	NA	NA	NA
Bos_taurus_newGene_14367	NA	NA	NA
Bos_taurus_newGene_14372	NA	NA	NA
Bos_taurus_newGene_14375	NA	NA	NA
Bos_taurus_newGene_14378	0.0722468	0.7952346	0.0995047
Bos_taurus_newGene_14380	NA	NA	NA
Bos_taurus_newGene_14383	0.2949043	0.48534	0.3139539
Bos_taurus_newGene_14384	-0.226377	0.6957135	0.1575696
Bos_taurus_newGene_14384	NA	NA	NA
Bos_taurus_newGene_14385	NA	NA	NA
Bos_taurus_newGene_14386	0.1156399	0.781065	0.1073128
Bos_taurus_newGene_14388	NA	NA	NA
Bos_taurus_newGene_14389	NA	NA	NA
Bos_taurus_newGene_14390	NA	NA	NA
Bos_taurus_newGene_14398	0.5067009	0.2383396	0.6228038
Bos_taurus_newGene_14398	NA	NA	NA
Bos_taurus_newGene_14402	NA	NA	NA
Bos_taurus_newGene_14404	NA	NA	NA
Bos_taurus_newGene_14409	NA	NA	NA
Bos_taurus_newGene_14410	-0.572516	0.2928229	0.533395

3os_taurus_newGene_14410[illegible]	-0.554733	0.4629174	0.3344965
3os_taurus_newGene_14410[illegible]	NA	NA	NA
3os_taurus_newGene_14411[illegible]	NA	NA	NA
3os_taurus_newGene_14411[illegible]	NA	NA	NA
3os_taurus_newGene_14413[illegible]	NA	NA	NA
3os_taurus_newGene_14425[illegible]	2.3797854	0.0003289	3.4829718
3os_taurus_newGene_14425[illegible]	0.2427476	0.5943119	0.2259856
3os_taurus_newGene_14427[illegible]	NA	NA	NA
3os_taurus_newGene_14427[illegible]	NA	NA	NA
3os_taurus_newGene_14429[illegible]	-0.459686	0.2536799	0.595714
3os_taurus_newGene_14429[illegible]	NA	NA	NA
3os_taurus_newGene_14431[illegible]	NA	NA	NA
3os_taurus_newGene_14433[illegible]	NA	NA	NA
3os_taurus_newGene_14434[illegible]	-0.361777	0.1745952	0.7579677
3os_taurus_newGene_14434[illegible]	NA	NA	NA
3os_taurus_newGene_14440[illegible]	NA	NA	NA
3os_taurus_newGene_14443[illegible]	NA	NA	NA
3os_taurus_newGene_14444[illegible]	NA	NA	NA
3os_taurus_newGene_14446[illegible]	0.7606238	0.1782905	0.7488718
3os_taurus_newGene_14448[illegible]	0.154348	0.7405467	0.1304476
3os_taurus_newGene_14449[illegible]	NA	NA	NA
3os_taurus_newGene_14459[illegible]	NA	NA	NA
3os_taurus_newGene_14461[illegible]	NA	NA	NA
3os_taurus_newGene_14461[illegible]	-0.229547	0.5946001	0.225775
3os_taurus_newGene_14463[illegible]	NA	NA	NA
3os_taurus_newGene_14463[illegible]	NA	NA	NA
3os_taurus_newGene_14463[illegible]	0.3908607	0.4180511	0.3787706
3os_taurus_newGene_14464[illegible]	NA	NA	NA
3os_taurus_newGene_14464[illegible]	NA	NA	NA
3os_taurus_newGene_14467[illegible]	NA	NA	NA
3os_taurus_newGene_14468[illegible]	NA	NA	NA
3os_taurus_newGene_14468[illegible]	NA	NA	NA
3os_taurus_newGene_14470[illegible]	-0.693582	0.0225448	1.6469531
3os_taurus_newGene_14470[illegible]	0.0238808	0.9307047	0.0311881
3os_taurus_newGene_14470[illegible]	NA	NA	NA
3os_taurus_newGene_14472[illegible]	NA	NA	NA
3os_taurus_newGene_14478[illegible]	-0.062653	0.8328894	0.0794127
3os_taurus_newGene_14479[illegible]	NA	NA	NA
3os_taurus_newGene_14479[illegible]	NA	NA	NA
3os_taurus_newGene_14479[illegible]	0.3287211	0.3501399	0.4557584
3os_taurus_newGene_14479[illegible]	0.8460409	0.0502498	1.2988657
3os_taurus_newGene_14479[illegible]	NA	NA	NA
3os_taurus_newGene_14479[illegible]	NA	NA	NA
3os_taurus_newGene_14480[illegible]	0.2623318	0.4117925	0.3853216
3os_taurus_newGene_14480[illegible]	NA	NA	NA
3os_taurus_newGene_14480[illegible]	-0.214112	0.5118193	0.2908833
3os_taurus_newGene_14480[illegible]	NA	NA	NA
3os_taurus_newGene_14480[illegible]	NA	NA	NA
3os_taurus_newGene_14481[illegible]	NA	NA	NA
3os_taurus_newGene_14482[illegible]	NA	NA	NA
3os_taurus_newGene_14483[illegible]	-0.102698	0.8168245	0.0878713
3os_taurus_newGene_14485[illegible]	NA	NA	NA
3os_taurus_newGene_14486[illegible]	NA	NA	NA
3os_taurus_newGene_14486[illegible]	NA	NA	NA
3os_taurus_newGene_14487[illegible]	NA	NA	NA
3os_taurus_newGene_14488[illegible]	NA	NA	NA
3os_taurus_newGene_14488[illegible]	NA	NA	NA
3os_taurus_newGene_14488[illegible]	NA	NA	NA

Bos_taurus_newGene_14489	NA	NA	NA
Bos_taurus_newGene_14489	NA	NA	NA
Bos_taurus_newGene_14490	NA	NA	NA
Bos_taurus_newGene_14491	1.3506081	0.0263987	1.5784182
Bos_taurus_newGene_14494	NA	NA	NA
Bos_taurus_newGene_14494	0.1120861	0.7502823	0.1247753
Bos_taurus_newGene_14494	NA	NA	NA
Bos_taurus_newGene_14495	NA	NA	NA
Bos_taurus_newGene_14498	NA	NA	NA
Bos_taurus_newGene_14500	NA	NA	NA
Bos_taurus_newGene_14501	NA	NA	NA
Bos_taurus_newGene_14503	NA	NA	NA
Bos_taurus_newGene_14503	NA	NA	NA
Bos_taurus_newGene_14507	NA	NA	NA
Bos_taurus_newGene_14508	NA	NA	NA
Bos_taurus_newGene_14511	0.2082705	0.4699369	0.3279604
Bos_taurus_newGene_14512	NA	NA	NA
Bos_taurus_newGene_14513	0.3778433	0.5161676	0.2872092
Bos_taurus_newGene_14515	NA	NA	NA
Bos_taurus_newGene_14515	NA	NA	NA
Bos_taurus_newGene_14515	NA	NA	NA
Bos_taurus_newGene_14516	-0.458291	0.2820563	0.5496642
Bos_taurus_newGene_14517	NA	NA	NA
Bos_taurus_newGene_14517	NA	NA	NA
Bos_taurus_newGene_14517	0.5527453	0.1641197	0.7848393
Bos_taurus_newGene_14520	0.2133364	0.5899718	0.2291688
Bos_taurus_newGene_14521	0.3251715	0.3157347	0.5006777
Bos_taurus_newGene_14521	NA	NA	NA
Bos_taurus_newGene_14521	NA	NA	NA
Bos_taurus_newGene_14521	NA	NA	NA
Bos_taurus_newGene_14522	-0.685028	0.5771756	0.238692
Bos_taurus_newGene_14523	NA	NA	NA
Bos_taurus_newGene_14524	0.1802755	0.66314	0.1783948
Bos_taurus_newGene_14525	NA	NA	NA
Bos_taurus_newGene_14525	-0.476972	0.1058041	0.9754976
Bos_taurus_newGene_14526	NA	NA	NA
Bos_taurus_newGene_14527	NA	NA	NA
Bos_taurus_newGene_14527	NA	NA	NA
Bos_taurus_newGene_14527	1.0625666	0.0532482	1.2736955
Bos_taurus_newGene_14530	NA	NA	NA
Bos_taurus_newGene_14530	-0.492913	0.2636328	0.5790005
Bos_taurus_newGene_14533	NA	NA	NA
Bos_taurus_newGene_14535	NA	NA	NA
Bos_taurus_newGene_14536	NA	NA	NA
Bos_taurus_newGene_14537	NA	NA	NA
Bos_taurus_newGene_14537	NA	NA	NA
Bos_taurus_newGene_14538	NA	NA	NA
Bos_taurus_newGene_14538	NA	NA	NA
Bos_taurus_newGene_14539	NA	NA	NA
Bos_taurus_newGene_14539	NA	NA	NA
Bos_taurus_newGene_14542	NA	NA	NA
Bos_taurus_newGene_14550	NA	NA	NA
Bos_taurus_newGene_14550	NA	NA	NA
Bos_taurus_newGene_14560	NA	NA	NA
Bos_taurus_newGene_14564	NA	NA	NA
Bos_taurus_newGene_14565	NA	NA	NA
Bos_taurus_newGene_14576	NA	NA	NA
Bos_taurus_newGene_14578	-0.503408	0.3220109	0.4921294

3os_taurus_newGene_14578[illegible]	NA	NA	NA
3os_taurus_newGene_14578[illegible]	-0.85266	0.0151253	1.8202958
3os_taurus_newGene_14579[illegible]	-1.06827	0.0764685	1.1165175
3os_taurus_newGene_14579[illegible]	-1.379312	0.0004706	3.3273462
3os_taurus_newGene_14582[illegible]	NA	NA	NA
3os_taurus_newGene_14584[illegible]	-0.088459	0.8553759	0.067843
3os_taurus_newGene_14584[illegible]	NA	NA	NA
3os_taurus_newGene_14607[illegible]	NA	NA	NA
3os_taurus_newGene_14612[illegible]	NA	NA	NA
3os_taurus_newGene_14613[illegible]	NA	NA	NA
3os_taurus_newGene_14614[illegible]	-0.325744	0.3675222	0.4347164
3os_taurus_newGene_14616[illegible]	0.1221932	0.8446845	0.0733055
3os_taurus_newGene_14617[illegible]	NA	NA	NA
3os_taurus_newGene_14618[illegible]	NA	NA	NA
3os_taurus_newGene_14619[illegible]	NA	NA	NA
3os_taurus_newGene_14624[illegible]	NA	NA	NA
3os_taurus_newGene_14625[illegible]	1.9308313	0.1525405	0.8166147
3os_taurus_newGene_14626[illegible]	0.72109	0.0583057	1.2342892
3os_taurus_newGene_14628[illegible]	-0.219732	0.3916741	0.4070751
3os_taurus_newGene_14629[illegible]	-0.931704	0.0006486	3.1880217
3os_taurus_newGene_14630[illegible]	0.4673652	0.4022015	0.3955563
3os_taurus_newGene_14630[illegible]	-1.038447	0.0453837	1.3431
3os_taurus_newGene_14635[illegible]	NA	NA	NA
3os_taurus_newGene_14638[illegible]	0.5405128	0.2888991	0.5392539
3os_taurus_newGene_14639[illegible]	NA	NA	NA
3os_taurus_newGene_14639[illegible]	10.936228	3.00E-27	26.522241
3os_taurus_newGene_14644[illegible]	NA	NA	NA
3os_taurus_newGene_14645[illegible]	NA	NA	NA
3os_taurus_newGene_14645[illegible]	NA	NA	NA
3os_taurus_newGene_14646[illegible]	NA	NA	NA
3os_taurus_newGene_14646[illegible]	0.7287122	0.1886315	0.7243859
3os_taurus_newGene_14648[illegible]	NA	NA	NA
3os_taurus_newGene_14654[illegible]	NA	NA	NA
3os_taurus_newGene_14657[illegible]	NA	NA	NA
3os_taurus_newGene_14662[illegible]	-0.261774	0.5340557	0.2724135
3os_taurus_newGene_14664[illegible]	NA	NA	NA
3os_taurus_newGene_14676[illegible]	NA	NA	NA
3os_taurus_newGene_14678[illegible]	-0.0865	0.7311198	0.1360115
3os_taurus_newGene_14679[illegible]	NA	NA	NA
3os_taurus_newGene_14680[illegible]	NA	NA	NA
3os_taurus_newGene_14685[illegible]	-0.933094	0.5933752	0.2266706
3os_taurus_newGene_14685[illegible]	NA	NA	NA
3os_taurus_newGene_14686[illegible]	NA	NA	NA
3os_taurus_newGene_14697[illegible]	NA	NA	NA
3os_taurus_newGene_14699[illegible]	NA	NA	NA
3os_taurus_newGene_14702[illegible]	NA	NA	NA
3os_taurus_newGene_14703[illegible]	-0.770436	0.0595486	1.2251286
3os_taurus_newGene_14703[illegible]	NA	NA	NA
3os_taurus_newGene_14704[illegible]	NA	NA	NA
3os_taurus_newGene_14707[illegible]	NA	NA	NA
3os_taurus_newGene_14708[illegible]	NA	NA	NA
3os_taurus_newGene_14709[illegible]	NA	NA	NA
3os_taurus_newGene_14713[illegible]	0.7818408	0.1405168	0.8522716
3os_taurus_newGene_14720[illegible]	NA	NA	NA
3os_taurus_newGene_14721[illegible]	NA	NA	NA
3os_taurus_newGene_14722[illegible]	NA	NA	NA
3os_taurus_newGene_14722[illegible]	NA	NA	NA
3os_taurus_newGene_14724[illegible]	NA	NA	NA

Bos_taurus_newGene_14727	0.1239911	0.6879623	0.1624353
Bos_taurus_newGene_14727	NA	NA	NA
Bos_taurus_newGene_14727	NA	NA	NA
Bos_taurus_newGene_14728	NA	NA	NA
Bos_taurus_newGene_14728	NA	NA	NA
Bos_taurus_newGene_14729	-0.032551	0.9341579	0.0295797
Bos_taurus_newGene_14729	NA	NA	NA
Bos_taurus_newGene_14734	NA	NA	NA
Bos_taurus_newGene_14735	NA	NA	NA
Bos_taurus_newGene_14736	NA	NA	NA
Bos_taurus_newGene_14736	NA	NA	NA
Bos_taurus_newGene_14737	-1.129573	0.0004465	3.3501737
Bos_taurus_newGene_14743	NA	NA	NA
Bos_taurus_newGene_14743	NA	NA	NA
Bos_taurus_newGene_14748	NA	NA	NA
Bos_taurus_newGene_14752	NA	NA	NA
Bos_taurus_newGene_14753	NA	NA	NA
Bos_taurus_newGene_14757	NA	NA	NA
Bos_taurus_newGene_14759	NA	NA	NA
Bos_taurus_newGene_14760	NA	NA	NA
Bos_taurus_newGene_14761	NA	NA	NA
Bos_taurus_newGene_14762	NA	NA	NA
Bos_taurus_newGene_14765	0.4977316	0.4190069	0.3777788
Bos_taurus_newGene_14767	0.2607296	0.6029967	0.219685
Bos_taurus_newGene_14771	NA	NA	NA
Bos_taurus_newGene_14771	NA	NA	NA
Bos_taurus_newGene_14773	NA	NA	NA
Bos_taurus_newGene_14775	NA	NA	NA
Bos_taurus_newGene_14777	NA	NA	NA
Bos_taurus_newGene_14781	NA	NA	NA
Bos_taurus_newGene_14782	-0.210595	0.8796517	0.0556892
Bos_taurus_newGene_14785	NA	NA	NA
Bos_taurus_newGene_14786	0.0985089	0.8574487	0.0667919
Bos_taurus_newGene_14786	NA	NA	NA
Bos_taurus_newGene_14787	NA	NA	NA
Bos_taurus_newGene_14790	NA	NA	NA
Bos_taurus_newGene_14792	0.4319958	0.14427	0.8408239
Bos_taurus_newGene_14792	NA	NA	NA
Bos_taurus_newGene_14794	NA	NA	NA
Bos_taurus_newGene_14794	-0.224405	0.6917773	0.1600337
Bos_taurus_newGene_14809	-0.295908	0.6046451	0.2184995
Bos_taurus_newGene_14817	NA	NA	NA
Bos_taurus_newGene_14817	NA	NA	NA
Bos_taurus_newGene_14817	NA	NA	NA
Bos_taurus_newGene_14817	NA	NA	NA
Bos_taurus_newGene_14818	-0.03997	0.9100731	0.0409237
Bos_taurus_newGene_14818	NA	NA	NA
Bos_taurus_newGene_14821	NA	NA	NA
Bos_taurus_newGene_14823	-0.097248	0.8679336	0.0615135
Bos_taurus_newGene_14824	NA	NA	NA
Bos_taurus_newGene_14833	NA	NA	NA
Bos_taurus_newGene_14833	NA	NA	NA
Bos_taurus_newGene_14836	NA	NA	NA
Bos_taurus_newGene_14840	NA	NA	NA
Bos_taurus_newGene_14844	0.3404492	0.6478917	0.1884976
Bos_taurus_newGene_14844	-0.245683	0.6181179	0.2089287
Bos_taurus_newGene_14850	NA	NA	NA
Bos_taurus_newGene_14851	0.1760169	0.6385073	0.1948341

3os_taurus_newGene_14853!	1.2685529	0.3881021	0.411054
3os_taurus_newGene_14860	NA	NA	NA
3os_taurus_newGene_14867	NA	NA	NA
3os_taurus_newGene_14868	NA	NA	NA
3os_taurus_newGene_14874	0.0871221	0.8182828	0.0870966
3os_taurus_newGene_14875	NA	NA	NA
3os_taurus_newGene_14876	NA	NA	NA
3os_taurus_newGene_14878	NA	NA	NA
3os_taurus_newGene_14879	-0.316753	0.5050202	0.2966912
3os_taurus_newGene_14880	0.8111088	0.0678812	1.1682508
3os_taurus_newGene_14883	-0.020045	0.9588666	0.0182418
3os_taurus_newGene_14883	-0.332164	0.5288085	0.2767016
3os_taurus_newGene_14884	NA	NA	NA
3os_taurus_newGene_14884	0.1087501	0.8151502	0.0887623
3os_taurus_newGene_14885	-0.914019	0.0345469	1.4615909
3os_taurus_newGene_14888	NA	NA	NA
3os_taurus_newGene_14888	NA	NA	NA
3os_taurus_newGene_14889	NA	NA	NA
3os_taurus_newGene_14891	NA	NA	NA
3os_taurus_newGene_14891	NA	NA	NA
3os_taurus_newGene_14892	NA	NA	NA
3os_taurus_newGene_14893	NA	NA	NA
3os_taurus_newGene_14894	NA	NA	NA
3os_taurus_newGene_14898	NA	NA	NA
3os_taurus_newGene_14898	NA	NA	NA
3os_taurus_newGene_14900	NA	NA	NA
3os_taurus_newGene_14908	-0.191031	0.4447528	0.3518813
3os_taurus_newGene_14908	NA	NA	NA
3os_taurus_newGene_14912	0.2383926	0.5546023	0.2560183
3os_taurus_newGene_14913	NA	NA	NA
3os_taurus_newGene_14913	-0.151699	0.8705837	0.0601895
3os_taurus_newGene_14921	NA	NA	NA
3os_taurus_newGene_14921	NA	NA	NA
3os_taurus_newGene_14924	NA	NA	NA
3os_taurus_newGene_14925	-0.334268	0.3151573	0.5014727
3os_taurus_newGene_14925	-0.001216	0.9984676	0.000666
3os_taurus_newGene_14926	NA	NA	NA
3os_taurus_newGene_14930	NA	NA	NA
3os_taurus_newGene_14930	NA	NA	NA
3os_taurus_newGene_14930	NA	NA	NA
3os_taurus_newGene_14931	NA	NA	NA
3os_taurus_newGene_14936	0.1715372	0.7358928	0.1331855
3os_taurus_newGene_14937	NA	NA	NA
3os_taurus_newGene_14938	-0.142124	0.5927414	0.2271347
3os_taurus_newGene_14940	NA	NA	NA
3os_taurus_newGene_14949	NA	NA	NA
3os_taurus_newGene_14952	NA	NA	NA
3os_taurus_newGene_14953	NA	NA	NA
3os_taurus_newGene_14954	NA	NA	NA
3os_taurus_newGene_14956	-1.972166	5.63E-05	4.2497655
3os_taurus_newGene_14956	-0.450392	0.4220607	0.3746251
3os_taurus_newGene_14957	NA	NA	NA
3os_taurus_newGene_14959	NA	NA	NA
3os_taurus_newGene_14960	NA	NA	NA
3os_taurus_newGene_14961	NA	NA	NA
3os_taurus_newGene_14961	NA	NA	NA
3os_taurus_newGene_14961	NA	NA	NA
3os_taurus_newGene_14963	NA	NA	NA

3os_taurus_newGene_14968(	NA	NA	NA
3os_taurus_newGene_14969(	NA	NA	NA
3os_taurus_newGene_14971(	NA	NA	NA
3os_taurus_newGene_14972:	NA	NA	NA
3os_taurus_newGene_14973!	NA	NA	NA
3os_taurus_newGene_14976·	NA	NA	NA
3os_taurus_newGene_14984:	NA	NA	NA
3os_taurus_newGene_14984	NA	NA	NA
3os_taurus_newGene_14985:	0.0337031	0.9570944	0.0190452
3os_taurus_newGene_14985:	NA	NA	NA
3os_taurus_newGene_14985	NA	NA	NA
3os_taurus_newGene_14987:	NA	NA	NA
3os_taurus_newGene_14988!	NA	NA	NA
3os_taurus_newGene_14989{	0.4621513	0.4005251	0.3973703
3os_taurus_newGene_14990(	NA	NA	NA
3os_taurus_newGene_14990!	2.6718374	1.20E-06	5.9198752
3os_taurus_newGene_14991:	-2.6038	0.0231813	1.6348631
3os_taurus_newGene_14992(	0.5711225	0.2866342	0.542672
3os_taurus_newGene_14994(	NA	NA	NA
3os_taurus_newGene_14995	NA	NA	NA
3os_taurus_newGene_15000(	NA	NA	NA
3os_taurus_newGene_15007:	NA	NA	NA
3os_taurus_newGene_15007:	NA	NA	NA
3os_taurus_newGene_15009(	NA	NA	NA
3os_taurus_newGene_15011!	NA	NA	NA
3os_taurus_newGene_15017(	NA	NA	NA
3os_taurus_newGene_15018:	NA	NA	NA
3os_taurus_newGene_15021!	-0.425363	0.3562086	0.4482955
3os_taurus_newGene_15024(	NA	NA	NA
3os_taurus_newGene_15036{	-0.070521	0.8721207	0.0594234
3os_taurus_newGene_15038(	NA	NA	NA
3os_taurus_newGene_15039(	NA	NA	NA
3os_taurus_newGene_15040:	NA	NA	NA
3os_taurus_newGene_15049·	NA	NA	NA
3os_taurus_newGene_15052:	NA	NA	NA
3os_taurus_newGene_15052·	0.7069013	0.1416586	0.848757
3os_taurus_newGene_15053(	NA	NA	NA
3os_taurus_newGene_15054(	NA	NA	NA
3os_taurus_newGene_15055!	NA	NA	NA
3os_taurus_newGene_15071:	NA	NA	NA
3os_taurus_newGene_15076!	NA	NA	NA
3os_taurus_newGene_15078!	NA	NA	NA
3os_taurus_newGene_15083:	-0.632954	0.3126822	0.5048968
3os_taurus_newGene_15093	NA	NA	NA
3os_taurus_newGene_15097:	NA	NA	NA
3os_taurus_newGene_15099·	-0.760797	0.0573391	1.2415493
3os_taurus_newGene_15101·	NA	NA	NA
3os_taurus_newGene_15108·	NA	NA	NA
3os_taurus_newGene_15114!	NA	NA	NA
3os_taurus_newGene_15119(	NA	NA	NA
3os_taurus_newGene_15124(	NA	NA	NA
3os_taurus_newGene_15125	NA	NA	NA
3os_taurus_newGene_15127{	0.1499696	0.722339	0.141259
3os_taurus_newGene_15128:	-0.007384	0.9959432	0.0017654
3os_taurus_newGene_15129·	-0.011047	0.9804574	0.0085713
3os_taurus_newGene_15130:	NA	NA	NA
3os_taurus_newGene_15131(	NA	NA	NA
3os_taurus_newGene_15133:	NA	NA	NA

3os_taurus_newGene_15134	NA	NA	NA
3os_taurus_newGene_15137	-0.460403	0.1791966	0.7466702
3os_taurus_newGene_15139	NA	NA	NA
3os_taurus_newGene_15140	NA	NA	NA
3os_taurus_newGene_15144	NA	NA	NA
3os_taurus_newGene_15147	NA	NA	NA
3os_taurus_newGene_15147	-0.246359	0.5110827	0.2915089
3os_taurus_newGene_15147	-0.501954	0.1136581	0.9443995
3os_taurus_newGene_15147	NA	NA	NA
3os_taurus_newGene_15149	0.7239996	0.0292562	1.5337826
3os_taurus_newGene_15151	NA	NA	NA
3os_taurus_newGene_15154	0.9080627	0.1259113	0.8999354
3os_taurus_newGene_15155	0.8579462	0.1132893	0.945811
3os_taurus_newGene_15155	NA	NA	NA
3os_taurus_newGene_15158	NA	NA	NA
3os_taurus_newGene_15159	NA	NA	NA
3os_taurus_newGene_15185	NA	NA	NA
3os_taurus_newGene_15187	-1.67364	0.0176071	1.7543121
3os_taurus_newGene_15188	NA	NA	NA
3os_taurus_newGene_15192	NA	NA	NA
3os_taurus_newGene_15201	NA	NA	NA
3os_taurus_newGene_15208	NA	NA	NA
3os_taurus_newGene_15210	NA	NA	NA
3os_taurus_newGene_15213	-0.013512	0.983143	0.0073833
3os_taurus_newGene_15225	NA	NA	NA
3os_taurus_newGene_15225	NA	NA	NA
3os_taurus_newGene_15229	NA	NA	NA
3os_taurus_newGene_15230	NA	NA	NA
3os_taurus_newGene_15232	NA	NA	NA
3os_taurus_newGene_15239	NA	NA	NA
3os_taurus_newGene_15239	NA	NA	NA
3os_taurus_newGene_15242	NA	NA	NA
3os_taurus_newGene_15242	-0.627513	0.1645056	0.7838193
3os_taurus_newGene_15277	0.2231937	0.6312953	0.1997674
3os_taurus_newGene_15277	NA	NA	NA
3os_taurus_newGene_15277	NA	NA	NA
3os_taurus_newGene_15280	NA	NA	NA
3os_taurus_newGene_15285	NA	NA	NA
3os_taurus_newGene_15291	NA	NA	NA
3os_taurus_newGene_15295	-0.381833	0.4624085	0.3349742
3os_taurus_newGene_15296	-0.288623	0.5556586	0.255192
3os_taurus_newGene_15296	NA	NA	NA
3os_taurus_newGene_15296	-0.484364	0.3770576	0.4235923
3os_taurus_newGene_15296	NA	NA	NA
3os_taurus_newGene_15296	-0.510732	0.2302565	0.6377881
3os_taurus_newGene_15297	NA	NA	NA
3os_taurus_newGene_15297	NA	NA	NA
3os_taurus_newGene_15297	-0.399061	0.3042496	0.51677
3os_taurus_newGene_15308	NA	NA	NA
3os_taurus_newGene_15315	0.0164219	0.9662592	0.0149064
3os_taurus_newGene_15316	NA	NA	NA
3os_taurus_newGene_15319	NA	NA	NA
3os_taurus_newGene_15321	-0.479217	0.1318138	0.8800391
3os_taurus_newGene_15321	-0.776352	0.1207357	0.9181644
3os_taurus_newGene_15324	NA	NA	NA
3os_taurus_newGene_15326	NA	NA	NA
3os_taurus_newGene_15326	NA	NA	NA
3os_taurus_newGene_15326	NA	NA	NA

Bos_taurus_newGene_15339	NA	NA	NA
Bos_taurus_newGene_15341	NA	NA	NA
Bos_taurus_newGene_15342	NA	NA	NA
Bos_taurus_newGene_15342	NA	NA	NA
Bos_taurus_newGene_15343	NA	NA	NA
Bos_taurus_newGene_15344	NA	NA	NA
Bos_taurus_newGene_15347	NA	NA	NA
Bos_taurus_newGene_15362	NA	NA	NA
Bos_taurus_newGene_15366	NA	NA	NA
Bos_taurus_newGene_15369	-0.530753	0.314916	0.5018053
Bos_taurus_newGene_15373	NA	NA	NA
Bos_taurus_newGene_15373	NA	NA	NA
Bos_taurus_newGene_15390	NA	NA	NA
Bos_taurus_newGene_15406	-1.340334	0.1885642	0.7245408
Bos_taurus_newGene_15407	NA	NA	NA
Bos_taurus_newGene_15407	NA	NA	NA
Bos_taurus_newGene_15409	NA	NA	NA
Bos_taurus_newGene_15411	-0.228037	0.3817386	0.4182339
Bos_taurus_newGene_15411	-0.044277	0.8887391	0.0512257
Bos_taurus_newGene_15411	0.642813	0.2259061	0.646072
Bos_taurus_newGene_15425	NA	NA	NA
Bos_taurus_newGene_15427	NA	NA	NA
Bos_taurus_newGene_15432	NA	NA	NA
Bos_taurus_newGene_15444	NA	NA	NA
Bos_taurus_newGene_15445	NA	NA	NA
Bos_taurus_newGene_15445	0.8660071	0.0810648	1.0911676
Bos_taurus_newGene_15445	NA	NA	NA
Bos_taurus_newGene_15445	0.0285506	0.9354038	0.0290009
Bos_taurus_newGene_15448	NA	NA	NA
Bos_taurus_newGene_15449	NA	NA	NA
Bos_taurus_newGene_15453	NA	NA	NA
Bos_taurus_newGene_15453	NA	NA	NA
Bos_taurus_newGene_15469	NA	NA	NA
Bos_taurus_newGene_15473	NA	NA	NA
Bos_taurus_newGene_15483	NA	NA	NA
Bos_taurus_newGene_15489	NA	NA	NA
Bos_taurus_newGene_15490	-0.966262	0.0139966	1.8539777
Bos_taurus_newGene_15495	-1.004827	0.0175023	1.7569052
Bos_taurus_newGene_15497	NA	NA	NA
Bos_taurus_newGene_15520	NA	NA	NA
Bos_taurus_newGene_15529	NA	NA	NA
Bos_taurus_newGene_15529	-1.876548	0.0004762	3.3221988
Bos_taurus_newGene_15533	NA	NA	NA
Bos_taurus_newGene_15534	-0.009304	0.9844086	0.0068246
Bos_taurus_newGene_15537	NA	NA	NA
Bos_taurus_newGene_15538	NA	NA	NA
Bos_taurus_newGene_15540	NA	NA	NA
Bos_taurus_newGene_15541	NA	NA	NA
Bos_taurus_newGene_15550	NA	NA	NA
Bos_taurus_newGene_15550	NA	NA	NA
Bos_taurus_newGene_15551	NA	NA	NA
Bos_taurus_newGene_15551	NA	NA	NA
Bos_taurus_newGene_15552	NA	NA	NA
Bos_taurus_newGene_15554	NA	NA	NA
Bos_taurus_newGene_15555	NA	NA	NA
Bos_taurus_newGene_15558	0.9725034	0.0667817	1.1753428
Bos_taurus_newGene_15558	-0.493284	0.148995	0.8268283
Bos_taurus_newGene_15560	NA	NA	NA

Bos_taurus_newGene_155672	NA	NA	NA
Bos_taurus_newGene_155675	NA	NA	NA
Bos_taurus_newGene_155677	0.0304604	0.9706879	0.0129204
Bos_taurus_newGene_155678	-0.269579	0.5119861	0.2907419
Bos_taurus_newGene_155683	NA	NA	NA
Bos_taurus_newGene_155729	0.1604972	0.67702	0.1693985
Bos_taurus_newGene_155730	NA	NA	NA
Bos_taurus_newGene_155731	NA	NA	NA
Bos_taurus_newGene_155747	NA	NA	NA
Bos_taurus_newGene_155814	NA	NA	NA
Bos_taurus_newGene_155840	NA	NA	NA
Bos_taurus_newGene_155842	NA	NA	NA
Bos_taurus_newGene_155871	NA	NA	NA
Bos_taurus_newGene_155907	NA	NA	NA
Bos_taurus_newGene_155963	-1.047409	0.1122999	0.9496206
Bos_taurus_newGene_155968	-0.395313	0.5776987	0.2382986
Bos_taurus_newGene_155975	NA	NA	NA
Bos_taurus_newGene_155992	NA	NA	NA
Bos_taurus_newGene_156014	NA	NA	NA
Bos_taurus_newGene_156015	NA	NA	NA
Bos_taurus_newGene_156034	-0.109172	0.8103852	0.0913085
Bos_taurus_newGene_156048	-0.912354	0.1116588	0.9521069
Bos_taurus_newGene_156050	NA	NA	NA
Bos_taurus_newGene_156051	-0.735428	0.1251931	0.9024196
Bos_taurus_newGene_156052	-0.511685	0.4048608	0.3926942
Bos_taurus_newGene_156056	-1.00329	0.0233029	1.6325902
Bos_taurus_newGene_156061	-2.154068	3.48E-12	11.457872
Bos_taurus_newGene_156068	NA	NA	NA
Bos_taurus_newGene_156070	-1.496519	0.0060235	2.2201532
Bos_taurus_newGene_156080	NA	NA	NA
Bos_taurus_newGene_156083	NA	NA	NA
Bos_taurus_newGene_156084	NA	NA	NA
Bos_taurus_newGene_156087	NA	NA	NA
Bos_taurus_newGene_156088	NA	NA	NA
Bos_taurus_newGene_156089	NA	NA	NA
Bos_taurus_newGene_156090	NA	NA	NA
Bos_taurus_newGene_156094	-0.352954	0.3080969	0.5113127
Bos_taurus_newGene_156224	NA	NA	NA
Bos_taurus_newGene_156325	-0.638267	0.1292227	0.8886613
Bos_taurus_newGene_156349	0.8322534	0.0532363	1.2737923
Bos_taurus_newGene_156412	NA	NA	NA
Bos_taurus_newGene_156413	NA	NA	NA
Bos_taurus_newGene_156427	-1.535859	0.0034141	2.4667199
Bos_taurus_newGene_156473	NA	NA	NA
Bos_taurus_newGene_156476	NA	NA	NA
Bos_taurus_newGene_156494	NA	NA	NA
Bos_taurus_newGene_156513	NA	NA	NA
Bos_taurus_newGene_156515	-0.316397	0.5232699	0.2812742
Bos_taurus_newGene_156545	NA	NA	NA
Bos_taurus_newGene_156581	NA	NA	NA
Bos_taurus_newGene_156642	NA	NA	NA
Bos_taurus_newGene_156712	-0.149394	0.8069204	0.0931693
Bos_taurus_newGene_156810	NA	NA	NA
Bos_taurus_newGene_156847	-1.212942	0.0034468	2.4625887
Bos_taurus_newGene_156850	-0.770828	0.056804	1.2456211
Bos_taurus_newGene_156851	-1.016813	0.0265439	1.5760354
Bos_taurus_newGene_156852	NA	NA	NA
Bos_taurus_newGene_156893	NA	NA	NA

Bos_taurus_newGene_15689[illegible]	NA	NA	NA
